# Causal Mediation Role of Immune Cells in Gut Microbiota–Pneumonia Associations: A Mendelian Randomisation Study

**DOI:** 10.1111/jcmm.70839

**Published:** 2025-09-11

**Authors:** Nianzong Hou, Zhenhong Zhang, Weiwei Song, Lin Wang, Guoxiang Xu, Rumin Zhang, Yulong Yang, Kai Wang

**Affiliations:** ^1^ Center of Gallbladder Disease, Shanghai East Hospital, Institute of Gallstone Disease, School of Medicine Tongji University Shanghai China; ^2^ Center of Translational Medicine, Zibo Central Hospital Binzhou Medical University Zibo Shandong China; ^3^ School of Clinical Medicine Shandong Second Medical University Weifang Shandong China; ^4^ Department of Critical Care Medicine, Zibo Central Hospital Binzhou Medical University Zibo Shandong China

**Keywords:** immune cell, mediating factor, mendelian randomization, pneumoniagut microbiota

## Abstract

The gut–lung axis plays a pivotal role in pneumonia pathogenesis, with immune regulation serving as a key mechanistic link between gut microbiota and disease progression. Despite established associations among gut microbiota, immune cell traits and pneumonia, their causal interplay and underlying mechanisms remain poorly elucidated. To investigate the causal relationships between gut microbiota and pneumonia and quantify the mediating effects of immune cell traits using Mendelian randomisation (MR), we performed a two‐sample MR and multivariable MR (MVMR) analysis employing inverse‐variance weighted (IVW) as the primary method. Genetic instruments for 211 gut microbiota taxa and 731 immune cell traits were derived from genome‐wide association studies (GWAS). Mediation analysis was conducted to estimate immune cell‐mediated effects on microbiota‐pneumonia associations. Genetically predicted abundance of the *Oxalobacteraceae* family was positively associated with pneumonia risk (OR: 1.090; 95% CI: 1.010–1.175; *p* = 0.026). Mediation analysis revealed that CD16^+^ monocytes significantly mediated this relationship (Mediated Effect: 0.025, proportion mediated: 29.1%). This study provides genetic evidence supporting *Oxalobacteraceae* as a causal risk factor for pneumonia, partially mediated through CD16^+^ monocyte regulation. These findings offer novel insights into microbiome‐directed immunomodulatory strategies for pneumonia prevention.

**Abbreviations**
CI: Confidence interval
GWAS: Genome‐wide association studies
IVs: Instrumental variables
IVW: Inverse‐variance weighted
LD: Linkage disequilibrium
MASH: Metabolic dysfunction‐associated steatohepatitis
MLR: Monocyte‐to‐lymphocyte ratio
MR: Mendelian randomisation
MVMR: Multivariable MR
OR: Odd ratio
PAGE: Population Architecture Using Genomics and Epidemiology
ROS: Reactive oxygen species
SCFA: Short‐chain fatty acid
SNPs: Single‐nucleotide polymorphisms
TAMs: Tumour‐associated macrophages

## 1 | Introduction

Pneumonia represents a major global health burden as a leading cause of acute respiratory infection‐related morbidity and mortality, particularly among aging populations [1]. This complex multisystem disorder not only affects pulmonary parenchyma but also induces systemic inflammatory responses, with pathogenesis influenced by bacterial, viral, and fungal pathogens [2]. Despite advances in antimicrobial therapy and supportive care, pneumonia remains responsible for nearly one million annual hospitalisations among elderly individuals in the United States alone, with persistently elevated mortality rates [3, 4]. These clinical challenges underscore the need to elucidate novel pathogenic mechanisms and therapeutic targets.

Emerging evidence highlights the gut–lung axis—a bidirectional communication network involving microbial metabolites, immune mediators and cellular trafficking—as a critical modulator of respiratory health [5]. The intestinal microbiota exerts remote immunomodulatory effects through multiple mechanisms, including short‐chain fatty acid (SCFA)‐dependent macrophage activation and mast cell‐mediated cross‐organ signalling [6, 7, 8]. Notably, preclinical studies demonstrate that gut microbiome depletion impairs alveolar macrophage function during *Streptococcus pneumoniae* infection, while SCFA supplementation enhances Klebsiella clearance via histone deacetylase inhibition [9, 10]. However, existing observational studies cannot establish causal relationships due to inherent confounding and reverse causation biases.

Mendelian randomisation (MR) has emerged as a powerful tool for causal inference in epidemiological research by leveraging genetic variants as instrumental variables [11]. This approach mimics randomised controlled trial design through its reliance on Mendel's second law of independent assortment, effectively minimising residual confounding [12]. Two key advantages distinguish MR from conventional methods: (1) genetic variants are fixed at conception, preventing reverse causation, and (2) the use of genome‐wide association study (GWAS) data enables high‐throughput analysis of multiple exposure–outcome relationships [13]. While MR has been applied to investigate microbiome–disease associations, no study has systematically evaluated immune cell traits as potential mediators in the gut microbiota–pneumonia relationship.

To address this knowledge gap, we conducted a comprehensive two‐sample MR analysis integrating GWAS data from 211 gut microbiota taxa, 731 immune cell traits and pneumonia outcomes. Our study has three primary objectives: 1) to identify causal associations between specific gut microbial taxa and pneumonia risk; 2) to quantify the mediating effects of immunophenotypic traits using multivariable MR; 3) to provide mechanistic insights for microbiome‐targeted preventive strategies. By employing robust genetic instrumental variable analysis, this research advances beyond correlation‐based observations to establish causal pathways within the gut–lung–immune axis.

## 2 | Methods

### 2.1 | Study Design

**FIGURE 1:**
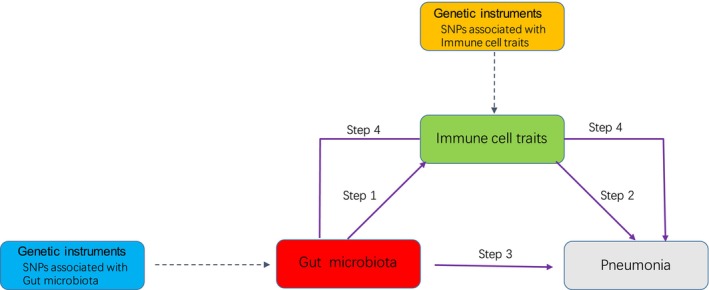
Study overview. Step 1 represents the causal effects of gut microbiota on Immune cell traits. Step 2 represents the causal effects of Immune cell traits on Pneumonia. Step 3 represents the causal effects of gut microbiota on Pneumonia. Step 4 represents a mediating analysis of Immune cell traits in the pathway from the gut microbiota to Pneumonia.

Our analytical framework comprised four key components (Figure 1). Step 1: analysis of causal effects of gut microbiota on immune cell traits; Step 2: analysis of causal effects of immune cell traits on pneumonia; Step 3: analysis of causal effects of gut microbiota on pneumonia; Step 4: mediation analysis of immune cell traits in the pathway from gut microbiota to pneumonia. We employed single‐nucleotide polymorphisms (SNPs) as instrumental variables (IVs), adhering to three fundamental Mendelian randomisation principles [14, 15]: 1) Relevance assumption: Strong association between genetic instruments and exposure variables (*p* < 5 × 10^−6^); 2) Exchangeability assumption: Genetic variants independent of potential confounders; 3) Exclusion restriction: Instruments influence outcomes exclusively through specified exposures.

### 2.2 | Data Source

The gut microbiome genetic data were obtained from the MiBioGen consortium [16], comprising 211 microbial taxa derived from 18,340 individuals of European ancestry across 24 cohorts in 11 countries. This comprehensive dataset enables taxonomic classification at five levels: 131 genera, 35 families, 20 orders, 16 classes and 9 phyla.

For immune cell characteristics, we utilised GWAS data (accession numbers GCST0001391‐GCST0002121) from 3757 Italian participants [17]. The dataset quantifies 731 immunophenotypic features, including Absolute cellular abundance (*n* = 118); Surface antigen expression levels (median fluorescence intensity, *n* = 389); Cellular morphology metrics (*n* = 32); Relative subpopulation frequencies (*n* = 192). The analysis incorporated approximately 22 million high‐density genetic markers, providing detailed characterisation of multiple immune lineages (B lymphocytes, T lymphocytes including regulatory subsets, dendritic cells, monocytes, granulocytes, myeloid cells, natural killer cells, etc.).

Pneumonia case–control data were sourced from the UK Biobank 2021 release, featuring 1) Cases: 22,567 European‐ancestry individuals; 2) Controls: 463,917 matched individuals; 3) Genomic coverage: 12,243,546 quality‐controlled SNPs.

This investigation constitutes a secondary analysis utilising exclusively aggregated GWAS summary statistics. All primary studies from which data were derived obtained appropriate institutional ethics committee approvals prior to data collection. As our research involved only de‐identified, population‐level genetic association data without access to individual participant information, additional ethical review was not mandated by our institution's research governance policies.

### 2.3 | Instrumental Variable Selection

The genetic instruments were selected from the MiBioGen consortium GWAS (18,340 individuals of European ancestry), employing a genome‐wide significance threshold (*p* < 5 × 10^−6^) to identify SNPs robustly associated with exposure. To ensure genetic independence and minimise linkage disequilibrium (LD)‐induced bias, we performed LD clumping using the 1000 Genomes European reference panel with stringent parameters (*r* ^*2*^ < 0.001 within a 10,000 kb window), retaining the most significant SNP per locus. Instrument strength was validated via F‐statistics >10, confirming avoidance of weak instrument bias. Potential pleiotropy was assessed through MR‐Egger regression, Cochran's Q test for heterogeneity and leave‐one‐out sensitivity analysis. This methodology specifically targeted variants demonstrating autonomous effects within the European population while substantially reducing potential pleiotropic confounding. The selection process prioritised instrument validity by maintaining appropriate physical distances between loci and verifying minimal correlation across genomic regions.

### 2.4 | Statistical Analysis

All analyses were conducted in R 4.3.3 using the Two Sample MR and MVMR packages, with the inverse‐variance weighted (IVW) method serving as the primary estimator for univariable Mendelian randomisation under fixed‐effect models, and statistical significance defined at *p* < 0.05 to evaluate causal effects of gut microbiota and immune traits on pneumonia risk. Sensitivity analyses were employed to address methodological assumptions: MR‐Egger regression tested directional pleiotropy; weighted median methods provided robustness against invalid instruments, while leave‐one‐out analysis was performed to assess whether any single SNP had a disproportionate impact on the overall causal estimate. For mediation analysis, multivariable MR (MVMR) jointly modelled the abundance of gut microbiome and immune cell traits as exposures, estimating the immune‐mediated proportion via the formula:(Direct effect—Total effect)/Total effect, with 95% confidence intervals derived from 1000 bootstrap iterations to account for uncertainty in effect estimates. Heterogeneity and pleiotropy diagnostics, including Cochran's Q test and MR‐Egger intercept tests, consistently supported the absence of significant bias, ensuring the reliability of causal inferences.

## 3 | Results

### 3.1 | Instrumental Variable Selection

Through our rigorous selection process, we identified 1086, 11,185 and 2494 SNPs significantly associated with gut microbiota composition, immune cell phenotypes and gut microbiota–immune cell interactions, respectively (Tables S1–S, 3). These genetic instruments met our predefined criteria for strength (*p* < 5 × 10^−6^) and independence (*r* ^*2*^ < 0.001), ensuring their validity for subsequent Mendelian randomisation analyses. The substantial number of identified variants, particularly for immune cell traits, reflects the polygenic nature of these biological characteristics and provides instruments for our causal inference framework.

### 3.2 | Causal Associations Between Gut Microbiota and Immune Cell Traits

Genetic instruments and their corresponding statistics are presented in Table S4 and Figures S1, S2. Employing a two‐sample Mendelian randomisation framework, we systematically evaluated the causal relationships between gut microbiota taxa demonstrating significant associations and immune cell traits. Our analysis identified 15 distinct microbiota‐immune cell trait pairs exhibiting causal relationships. Sensitivity analyses confirmed the robustness of these associations, revealing no evidence of horizontal pleiotropy (MR‐Egger intercept test, all *p* > 0.05) or substantial heterogeneity (Cochran's Q test, all *p* > 0.05).

### 3.3 | Causal Associations of Immune Cell Traits on Pneumonia Risk

**FIGURE 2:**
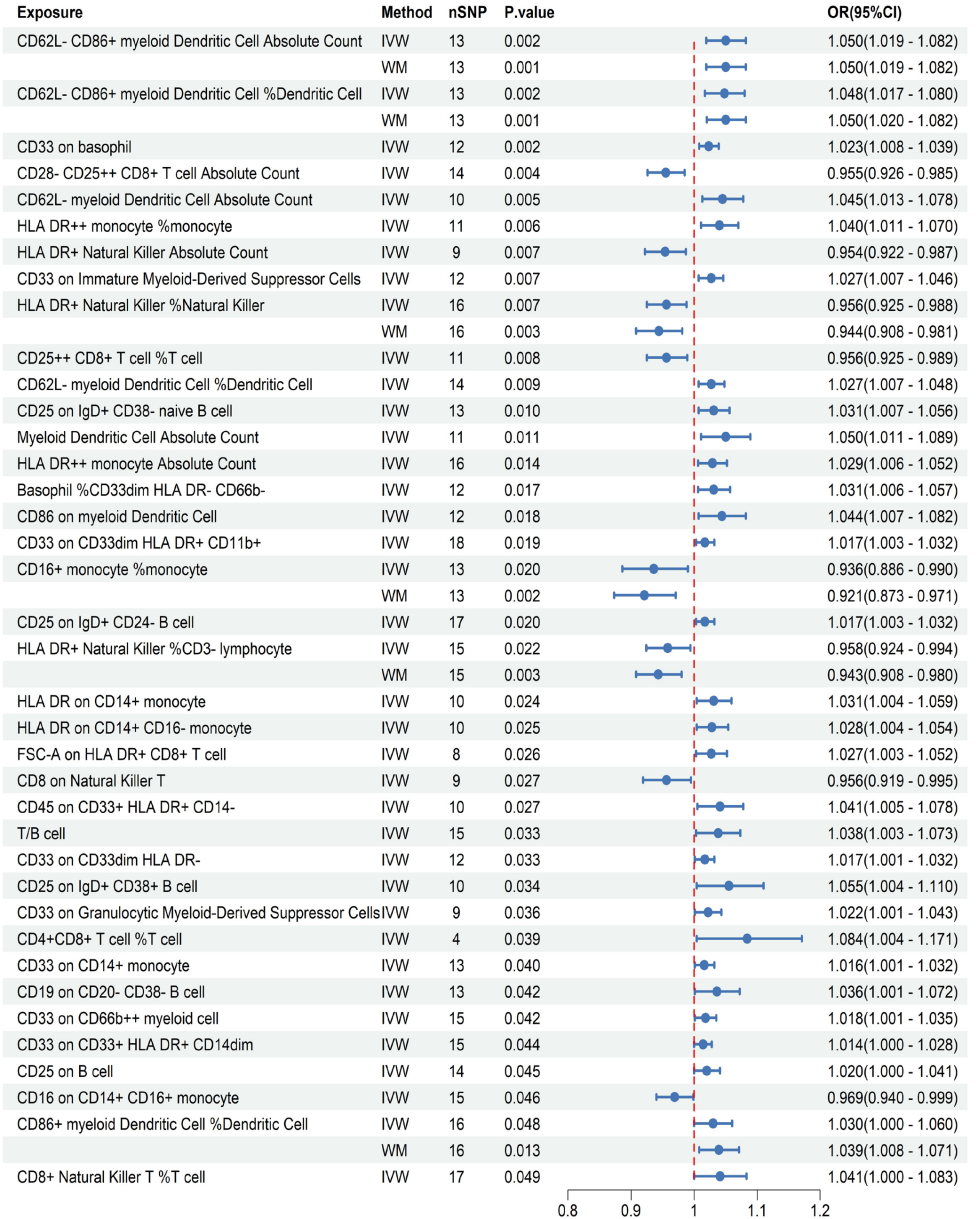
Causal associations of immune cell traits with pneumonia. CI, confidence interval; OR, odds ratio; SNP, single nucleotide polymorphism.

Using MR analysis across 731 immune cell traits, we identified 39 traits significantly associated with pneumonia susceptibility after applying stringent quality control measures. Of these, 30 immune cell traits conferred an elevated risk of pneumonia (OR > 1), whereas 9 exhibited protective effects (OR < 1). Sensitivity analyses, however, revealed inconsistencies in certain associations (Table S5, Figures S3, S, 4). Specifically, CD4^−^CD8^−^ Natural Killer T %lymphocyte demonstrated significant pleiotropy (< 0.05) and was subsequently excluded. Six additional traits, including CD62L^−^ CD86^+^ myeloid Dendritic Cell Absolute Count, displayed marked heterogeneity (*I* ^*2*^ > 50%), necessitating validation via weighted median regression (Figure 2).

### 3.4 | Causal Associations Between Gut Microbiota and Pneumonia

**FIGURE 3:**
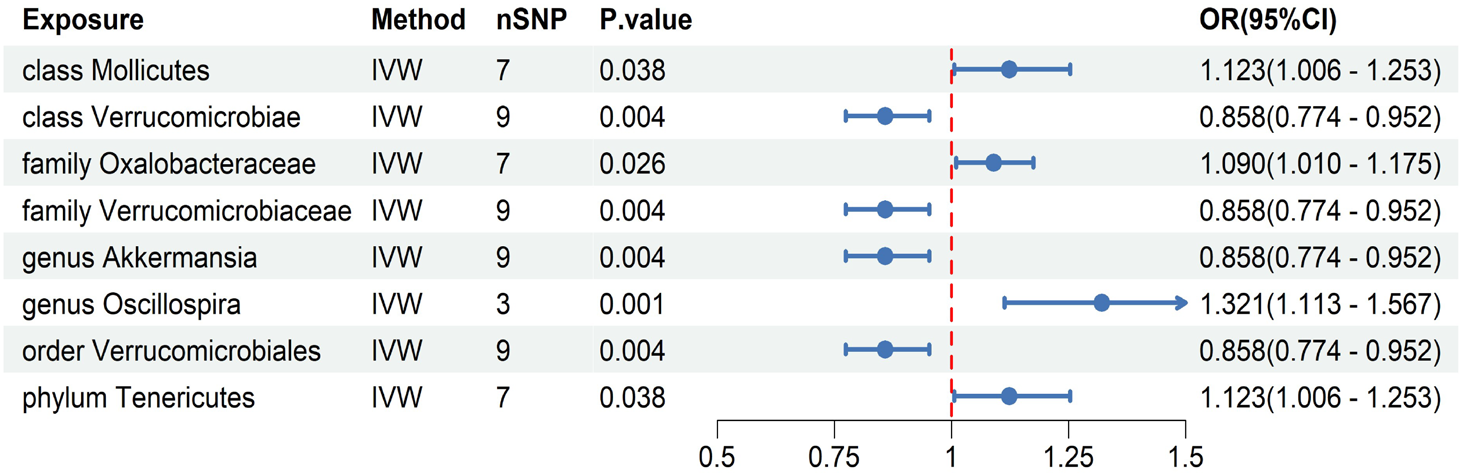
Causal associations of gut microbiota with pneumonia. CI, confidence interval; OR, odds ratio; SNP, single nucleotide polymorphism.

In our primary MR analysis of gut microbiota and pneumonia, we excluded taxa with fewer than three instrumental variables (family Clostridiaceae, genus Senegalimassilia) to mitigate weak instrument bias. Further exclusions were applied to the Lachnospiraceae NC2004 group genus due to inconsistent effect directions across MR methods. Final IVW‐derived associations (*p* < 0.05) are depicted in Figure 3. Four taxa were positively associated with pneumonia risk: genus Oscillospira (OR = 1.321, 95% CI: 1.113–1.567, *p* = 0.001), family Oxalobacteraceae (OR = 1.090, 95% CI: 1.010–1.175, *p* = 0.026), class Mollicutes (OR = 1.123, 95% CI: 1.006–1.253, *p* = 0.038) and phylum Tenericutes (OR = 1.123, 95% CI: 1.006–1.253, *p* = 0.038). Conversely, four taxa demonstrated protective effects: family Verrucomicrobiaceae (OR = 0.858, 95% CI: 0.774–0.952, *p* = 0.004), class Verrucomicrobiae (OR = 0.858, 95% CI: 0.774–0.952, *p* = 0.004), order Verrucomicrobiales (OR = 0.858, 95% CI: 0.774–0.952, *p* = 0.004) and genus Akkermansia (OR = 0.858, 95% CI: 0.774–0.952, *p* = 0.004). Sensitivity analyses confirmed the absence of pleiotropy (MR‐Egger intercept, all *p* > 0.05) and heterogeneity (IVW Q test, all *p* > 0.05; Table S6, Figures S5, S6).

### 3.5 | Mediation Analysis of Immune Cell Traits in Microbiota–Pneumonia Associations

Multivariable MR was employed to quantify mediation effects, excluding mediators with fewer than three SNPs or nonsignificant mediation proportions (95% CI crossing zero). There are 4 associations included: family Oxalobacteraceae impacting CD16^+^ monocyte % monocyte, genus Oscillospira impacting Basophil %CD33dim HLA DR^−^ CD66b^−^, class Mollicutes impacting CD8 on Natural Killer T and phylum Tenericutes impacting CD8 on Natural Killer T. While current literature has not yet established an association between Basophil %CD33dim HLA DR^−^ CD66b^−^ cells/CD8 on Natural Killer T cells and pneumonia, extensive studies have conclusively demonstrated the critical role of CD16^**+**^ monocytes in the pathogenesis and progression of pneumonia [18, 19, 20, 21, 22]. Consequently, we identified CD16^**+**^ monocytes as a key regulatory factor in the Oxalobacteraceae–pneumonia relationship (proportion mediated: 29.1%; mediated effect: 0.025; total effect OR: 1.090, 95% CI: 1.010–1.175, *p* = 0.026).

## 4 | Discussion

Our Mendelian randomisation study provides novel insights into the causal pathways linking gut microbiota, immune cell traits and pneumonia susceptibility. The findings advance our understanding of the gut–lung–immune axis through three pivotal findings that merit further exploration. Comprehensive two‐sample Mendelian randomisation revealed genetically validated causal relationships between specific gut microbial taxa and pneumonia risk. Mediation analysis demonstrated that approximately 29.0% of this effect was mediated through CD16^+^ monocyte populations, indicating their critical mediating role in translating gut microbial signals to pulmonary immune responses. This investigation represents the first MR‐based evidence of immune cell‐mediated mechanisms in microbiome–pneumonia associations, highlighting novel therapeutic targets for pneumonia prevention through gut microbiota modulation.

Monocytes represent a functionally heterogeneous population of circulating immune cells that play critical roles in both innate and adaptive immunity through multiple mechanisms, including phagocytosis, antigen presentation and cytokine production [23, 24]. These versatile cells participate in the complete spectrum of inflammatory responses, from initial activation to resolution and tissue repair [25]. While macrophages share similar functional properties, monocytes are distinguished by their circulatory nature and developmental plasticity. Current classification systems categorise human peripheral blood monocytes into three distinct subsets based on differential expression of CD14 and CD16 surface markers: classical (CD14^++^CD16^−^), intermediate (CD14^++^CD16^+^) and non‐classical (CD14^+^CD16^++^)monocytes [26]. Each subset exhibits unique functional specialisations, with CD16^+^ populations (intermediate and non‐classical) demonstrating particularly robust proinflammatory cytokine secretion capabilities [27]. The expansion of these CD16^+^ subsets has been consistently associated with various inflammatory and infectious disease states, highlighting their pathogenic significance. Recent studies have further elucidated the functional heterogeneity within monocyte subsets. For instance, CD14^++^CD16^+^ demonstrate enhanced antigen presentation capacity through upregulated MHC‐II and co‐stimulatory molecules (CD40/CD86), while CD14^+^CD16^++^ exhibit immunosuppressive properties through PD‐L1 expression and reactive oxygen species (ROS) production [28, 29]. This functional divergence is critically regulated by transcriptional networks involving CEBPB and CORO1A, which modulate differentiation trajectories and metabolic reprogramming [30]. In the context of SARS‐CoV‐2 infection, a virus‐specific monocyte inflammatory phenotype characterised by upregulated TLR4 and IL‐23 expression in CD16^+^ subsets was observed, driving pulmonary cytokine storms and chemokine‐mediated immune cell recruitment. This aligns with findings in *Mycoplasma pneumoniae* infections, where CD16^+^ monocytes mediate lung inflammation via TLR4‐dependent IL‐23 secretion, correlating with disease severity markers such as reduced HLA‐DR expression [31].

As circulating precursors to tissue macrophages and dendritic cells, monocytes contribute to essential host defence mechanisms while paradoxically participating in pathological inflammatory processes when dysregulated [32, 33]. Experimental models have demonstrated that pulmonary infections induce monocyte recruitment to lung tissue, where they undergo functional differentiation into macrophage‐like cells capable of providing sustained antimicrobial protection [34]. However, chronic inflammation leads to pathological remodelling, as seen in cGVHD, where CSF1‐dependent donor‐derived macrophages drive fibrotic disease progression [28]. Similarly, in metabolic dysfunction‐associated steatohepatitis (MASH), monocyte‐derived macrophages exhibit metabolic dysregulation with enhanced glycolysis and mitochondrial ROS production, exacerbating hepatic inflammation [30]. Clinical observations further support the prognostic value of monocyte‐related parameters, with the monocyte‐to‐lymphocyte ratio (MLR) serving as a reliable predictor of pneumonia severity in stroke patients [35]. In anti‐MDA5^+^ dermatomyositis‐associated interstitial lung disease, peripheral monocyte immunosuppression contrasts with pulmonary Mo‐AM hyperactivation, driving cytokine storms and fibrosis [36]. These findings underscore the importance of compartment‐specific monocyte dynamics in disease pathogenesis. In paediatric *Mycoplasma pneumoniae* infections, characteristic alterations in monocyte subpopulations, including increased proportions of non‐classical and intermediate subsets coupled with reduced HLA‐DR expression on CD14 bright CD16^+^ monocytes, correlate with disease severity [37]. Mechanistically, CD16^+^ monocytes have been shown to mediate pulmonary inflammation through TLR4‐dependent IL‐23 secretion in Mycoplasma pneumonia [31, 38], findings that are consistent with our current results. Emerging therapeutic strategies targeting monocyte subsets show promise. CSF1R inhibition effectively depletes pathogenic macrophages in cGVHD, while STAT3/IDO modulation enhances monocyte‐derived dendritic cell activation for cancer immunotherapy [28, 39]. In sarcoma immunoradiotherapy, elevated tumour‐associated macrophages (TAMs) expressing IL4I1/HES1 predict poor response, highlighting the need for combinatorial approaches targeting monocyte–macrophage axes [40]. These advances emphasise the translational potential of understanding monocyte biology in developing precision immunotherapies.

While our study identified CD16^+^ monocytes as key mediators of immune dysregulation, we acknowledge that MVMR did not adjust for additional immune traits such as neutrophil counts or T cell subtypes. This omission may introduce residual confounding, given the functional interplay between immune cell populations. However, robust sensitivity analyses—including bootstrap resampling, MR‐Egger intercept tests—suggested that unmeasured immune traits minimally impacted our findings. Future work should integrate multi‐omics data to map monocyte–T cell/neutrophil crosstalk and employ longitudinal MR frameworks to address time‐dependent confounding.

Our investigation specifically implicates members of the *Oxalobacteraceae* family, a taxon of Gram‐negative bacteria with both environmental and symbiotic distributions, in modulating immune responses and respiratory health. This family includes metabolically versatile taxa such as *Oxalobacter formigenes* , which has been extensively studied for its role in oxalate metabolism and protection against urolithiasis [41, 42]. Emerging evidence, however, highlights broader immunomodulatory and respiratory pathophysiological contributions of Oxalobacteraceae beyond oxalate degradation. For instance, *Oxalobacter spp*. May influence bronchial hyperresponsiveness (BHR) in asthma through interactions with airway epithelial cells and immune mediators [43]. Metagenomic and mechanistic studies suggest that *Oxalobacteraceae*‐derived metabolites, such as short‐chain fatty acids (SCFAs), could regulate airway smooth muscle tone and inflammatory signalling pathways, potentially attenuating or exacerbating conditions like asthma [44, 45].

In respiratory infections, *Oxalobacteraceae* members such as *Massiliaspp*. Exhibit adhesive properties and biofilm‐forming capabilities, enabling colonisation of the nasal and bronchial mucosa [46]. This colonisation may disrupt host–microbe homeostasis by competing with pathogenic bacteria or modulating local immune responses. For example, *Massilia polaris*, isolated from Arctic soils, encodes cold‐shock proteins that enhance survival in harsh environments, potentially facilitating persistence in the respiratory tract under inflammatory conditions [47]. Furthermore, comparative genomic analyses reveal that *Oxalobacteraceae* possess unique O‐antigen synthesis clusters, which may evade host immune recognition or mediate pathogen–host interactions [48]. The family's role in autoimmune and inflammatory diseases further underscores its immunomodulatory potential. Altered *Oxalobacteraceae* abundance has been linked to Parkinson's disease, where intestinal mucosal α‐synuclein misfolding correlates with shifts in microbial composition, including increased *Oxalobacteraceae* [49]. Similarly, in autoimmune conditions like rheumatoid arthritis, *Oxalobacteraceae*‐derived metabolites may influence systemic inflammation by modulating T‐cell responses or cytokine production [50]. These findings align with recent MR studies implicating *Oxalobacteraceae* in systemic immune dysregulation, including associations with osteoporosis and autoimmune disorders. *Oxalobacteraceae*'s dual capacity for oxalate metabolism and complex interactions with host immune pathways positions it as a critical player in respiratory and systemic health. Future research should prioritise mechanistic studies to elucidate how specific taxa influence mucosal immunity, airway remodelling and cross‐kingdom signalling in health and disease.

This study represents a significant advancement in methodological approaches to causal inference. We employed a comprehensive two‐sample Mendelian randomisation framework to systematically evaluate causal relationships between diverse gut microbiota taxa and immune cell traits. This approach addresses key limitations of observational studies by minimising confounding biases and establishing temporal precedence. The integration of mediation analysis provides a robust mechanism for quantifying intermediate biological pathways, thereby elucidating potential therapeutic targets. The genetic instruments selected for analysis rigorously adhered to Mendelian randomisation assumptions. Sensitivity analyses confirmed the robustness of our primary findings. For Oxalobacteraceae abundance, genetic instrumental variables were identified through a two‐step validation process: (1) Screening SNPs significantly associated with microbial abundance using MiBioGen consortium summary statistics, prioritising variants in gut microbiome‐associated loci with plausible mechanistic relevance to microbial colonisation, such as gut microenvironment, homeostasis, immune modulation and xenobiotic metabolism; (2) Rigorous validation of instrumental strength via *F*‐statistic calculations (all *F* > 10, Table S1), exceeding conventional thresholds for weak instrument bias mitigation. This dual‐validation framework ensures both instrumental validity and specificity, establishing robust causal inference for MR analyses. The absence of significant horizontal pleiotropy or heterogeneity strengthens confidence in causal interpretations. Identification of specific microbial–immune pathways as pneumonia risk factors suggests actionable intervention strategies: (1) Dietary modifications targeting microbial abundance; (2) Pharmacological approaches modulating immune cell activation; (3) Probiotic interventions restoring microbial–immune homeostasis. These findings hold particular relevance for high‐risk populations, where microbiome‐directed strategies could synergistically complement existing prevention frameworks.

Although bidirectional MR and reverse causality assessments are theoretically compelling, their application to the gut–lung axis in pneumonia research was methodologically and biologically constrained. Methodologically, bidirectional MR requires disentangling two independent causal pathways (microbiota–pneumonia and pneumonia–microbiota), which demands substantial statistical power to address weak instrument bias and pleiotropy. However, pneumonia‐associated genetic variants explain minimal variance in pneumonia incidence, and existing GWAS aggregates bacterial or viral etiologies, diluting causal signals specific to the outcome. This heterogeneity complicates the identification of robust instruments for reverse MR, where pneumonia‐linked SNPs may reflect confounding factors like hospitalisation or antibiotic use rather than intrinsic biological mechanisms. Temporally, acute pneumonia alters microbiota composition through transient factors, including antibiotic treatment or immune dysregulation, but these shifts resolve post‐recovery, creating ambiguity in establishing causal lags—a critical requirement for bidirectional MR. Biologically, the gut–lung axis operates predominantly in a unidirectional manner: microbial metabolites modulate pulmonary immune cells via epigenetic reprogramming of myeloid progenitors, while pneumonia‐induced microbiota changes are secondary to systemic inflammation or therapeutic interventions. Preclinical and clinical evidence support the primacy of microbiota‐driven immune dysregulation in pneumonia susceptibility, with dysbiosis preceding disease onset rather than being a downstream consequence. Thus, prioritising the microbiota–pneumonia pathway aligned with mechanistic evidence and translational priorities, enabling actionable insights, such as dietary or probiotic interventions, while avoiding speculative analyses of transient, infection‐related microbiota fluctuations.

## 5 | Limitations and Future Research

Our study provides genetic evidence for the interplay between Oxalobacteraceae and pneumonia susceptibility; however, several critical limitations must be acknowledged. Although we identified an association at the family level and hypothesised Oxalobacter formigenes as a key driver due to its established *oxalate*‐metabolising role, the lack of species‐level resolution in microbial community profiling poses ambiguity. This limitation may conflate functionally distinct taxa that could exhibit divergent immunomodulatory effects. Furthermore, the predominantly European ancestry of our genomic cohort, while foundational for initial insights, limits the generalisability of findings to non‐European populations—a critical concern given documented ethnic disparities in pneumonia pathogenesis. Additionally, demographic variables such as age, sex and population‐specific modifiers were not systematically evaluated, despite evidence that these factors shape both pneumonia susceptibility and immune cell distribution.

To address these gaps, future research should prioritise three interconnected axes. First, advancing taxonomic precision through metagenomic sequencing with strain‐level resolution will enable differentiation of *Oxalobacteraceae members* in human cohorts. Culturomic isolation of candidate strains, followed by *in vitro co*‐culture experiments with CD16^+^ monocytes, will allow quantification of strain‐specific immunomodulatory effects, such as cytokine secretion and phagocytosis modulation. Second, integrating gnotobiotic mouse models colonised with defined *Oxalobacteraceae consortia* will help dissect causal pathways in pneumonia. Targeted depletion of CD16^+^ monocytes within these models could isolate immune‐mediated mechanisms, while clinical trials pairing strain‐specific interventions with longitudinal multi‐omics profiling (metagenomics, metabolomics, single‐cell transcriptomics) will map strain dynamics to immune biomarkers and clinical outcomes. Third, cross‐disciplinary strategies combining ecological modelling, machine learning, and spatial transcriptomics could predict strain‐immune interactions while adjusting for age/sex confounders. For genomic research, cross‐ancestry meta‐analyses should identify shared and population‐specific loci influencing *Oxalobacteraceae* colonisation, with functional validation via CRISPR screens and eQTL mapping. Developing ancestry‐aware polygenic risk scores and expanding GWAS representation through initiatives like Population Architecture Using Genomics and Epidemiology (PAGE) will mitigate health disparities. Finally, integrating multi‐ethnic cohorts and epigenetic editing tools will enhance mechanistic understanding of demographic impacts on pneumonia susceptibility and immune cell regulation.

## 6 | Conclusion

This Mendelian randomisation study provides novel insights into the causal relationships between gut microbiota, immune cell traits and pneumonia risk. By identifying specific microbial families as risk factors and certain immune cells as key mediators, we have an advanced understanding of the gut–lung–immune axis. These findings reveal a potential causal pathway for developing targeted interventions that modulate microbiome–immune interactions to prevent or treat pneumonia. The study also demonstrates the power of causal inference approaches in unravelling complex biological relationships, paving the way for future research into microbial influences on respiratory health. Further investigation of these relationships may yield innovative approaches for pneumonia prevention and personalised treatment strategies.

## Author Contributions

**Nianzong Hou:** conceptualization (equal), formal analysis (equal), writing – original draft (equal), writing – review and editing (equal). **Zhenhong Zhang:** conceptualization (equal), data curation (equal), methodology (equal), project administration (equal). **Weiwei Song:** methodology (equal), visualization (equal), writing – original draft (equal). **Lin Wang:** formal analysis (equal), project administration (equal). **Guoxiang Xu:** data curation (equal), investigation (equal). **Rumin Zhang:** investigation (equal), methodology (equal), project administration (equal), writing – review and editing (equal). **Yong Yu:** investigation (equal). **Kai Wang:** conceptualization (equal), validation (equal), writing – original draft (equal), writing – review and editing (equal).

## Ethics Statement

The present study is a secondary analysis of publicly available data. Ethical approval was granted for each of the original GWAS studies. In addition, no individual‐level data were used in this study. Therefore, no new ethical review board approval was required.

## Consent

The authors have nothing to report.

## Conflicts of Interest

The authors declare no conflicts of interest.

## Supporting information


**Figure S1:** Sensitivity analysis of gut microbiota and immune cell characteristics Mendelian randomization (Scatter plot).

**Figure S2:** Sensitivity analysis of gut microbiota and immune cell characteristics Mendelian randomization (Forest plot).

**S3:** Sensitivity analysis of immune cell characteristics and Pneumonia Mendelian randomization (Scatter plot).

**S4:** Sensitivity analysis of immune cell characteristics and Pneumonia Mendelian randomization (Forest plot).

**S5:** Sensitivity analysis of gut microbiota and Pneumonia Mendelian randomization (Scatter plot).

**S6:** Sensitivity analysis of gut microbiota and Pneumonia Mendelian randomization (Forest plot).

**Table S1:** Information about instrumental variables corresponding to gut microbiota.

**Table S2:** Information about instrumental variables corresponding to immune cell phenotypes.

**Table S3:** Information about instrumental variables corresponding to gut microbiota (Mendelian randomization of gut microbiota on immune cells).

**Table S4:** Causal effects of gut microbiota on immune cells.

**Table S5:** Causal effects of immune cells on Pneumonia.

**Table S6:** Causal effects of gut microbiota on Pneumonia.

## Data Availability

All data used in the present study were obtained from genome wide association study summary statistics which were publicly released by genetic consortia.
