# Supplementary material for: Causal Mediation Role of Immune Cells in Gut Microbiota–Pneumonia Associations: A Mendelian Randomisation Study
Source: J Cell Mol Med. 2025 Sep 11;29(17):e70839. doi: 10.1111/jcmm.70839 (PMC12425809; doi:10.1111/jcmm.70839)
Supplement: Supplementary file 1 — Figure S1: Sensitivity analysis of gut microbiota and immune cell characteristics Mendelian randomization (Scatter plot). [file JCMM-29-e70839-s006.pdf]

Supplementary Figure 5 Sensitivity analysis of gut microbiota and immune cell characteristics Mendelian randomization (Scatter plot)

A

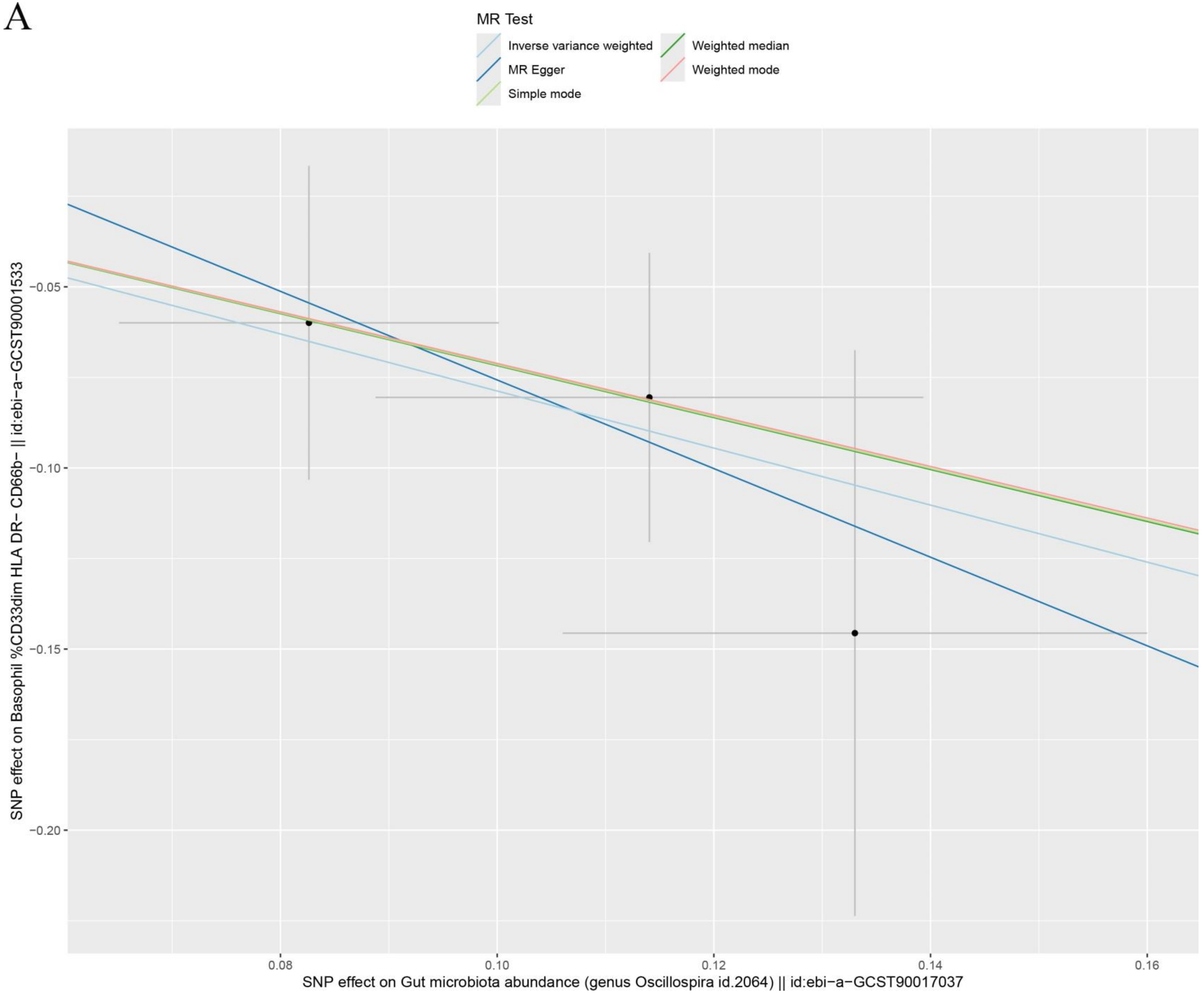

B

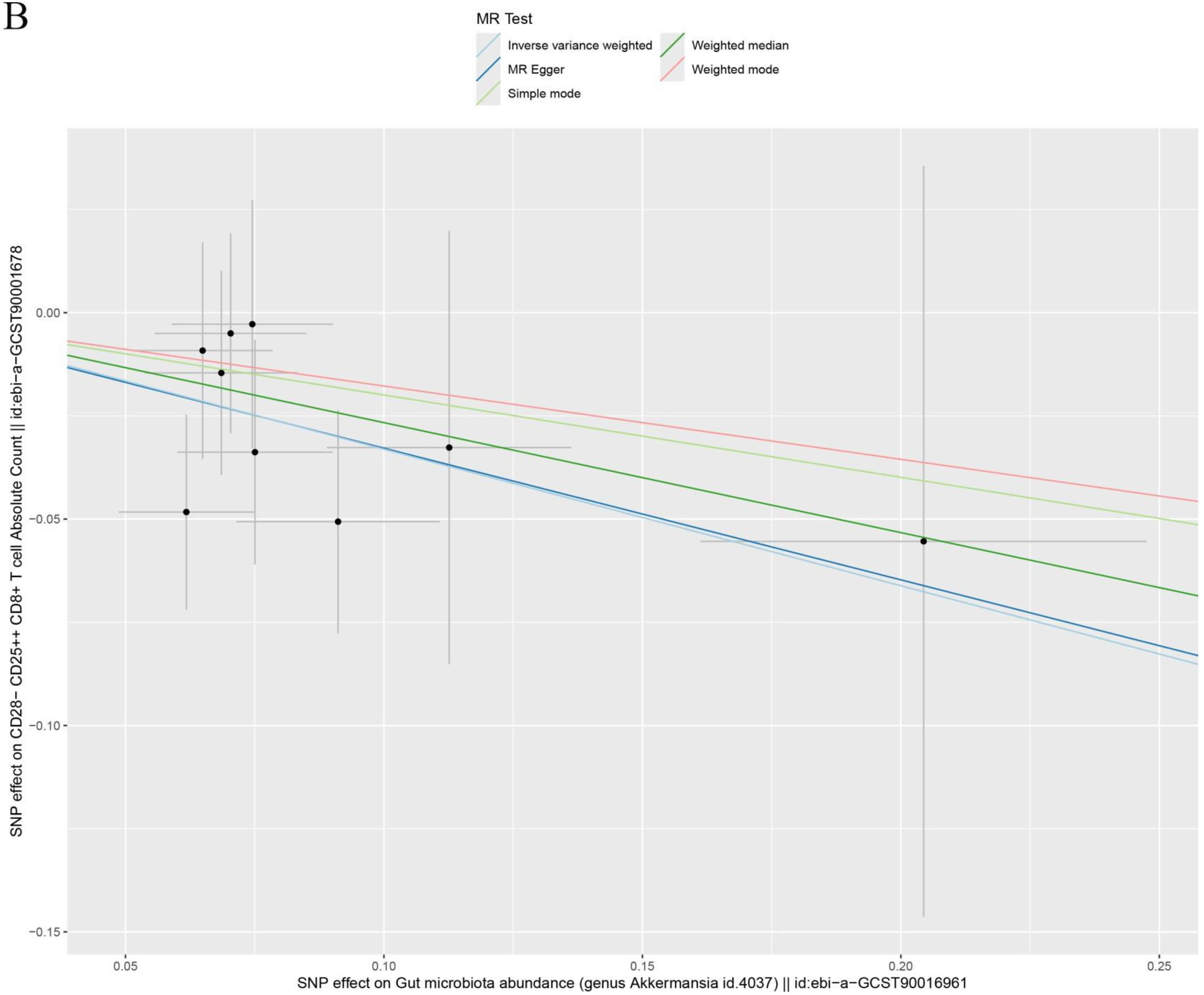
MR Test
Inverse variance weighted
MR Egger
Simple mode
Weighted median
Weighted mode
0.00
−0.05
−0.10
−0.15
0.05
0.10
0.15
0.20
0.25
SNP effect on CD28− CD25++ CD8+ T cell Absolute Count || id:ebi−a−GCST90001678
SNP effect on Gut microbiota abundance (genus Akkermansia id.4037) || id:ebi−a−GCST90016961

C

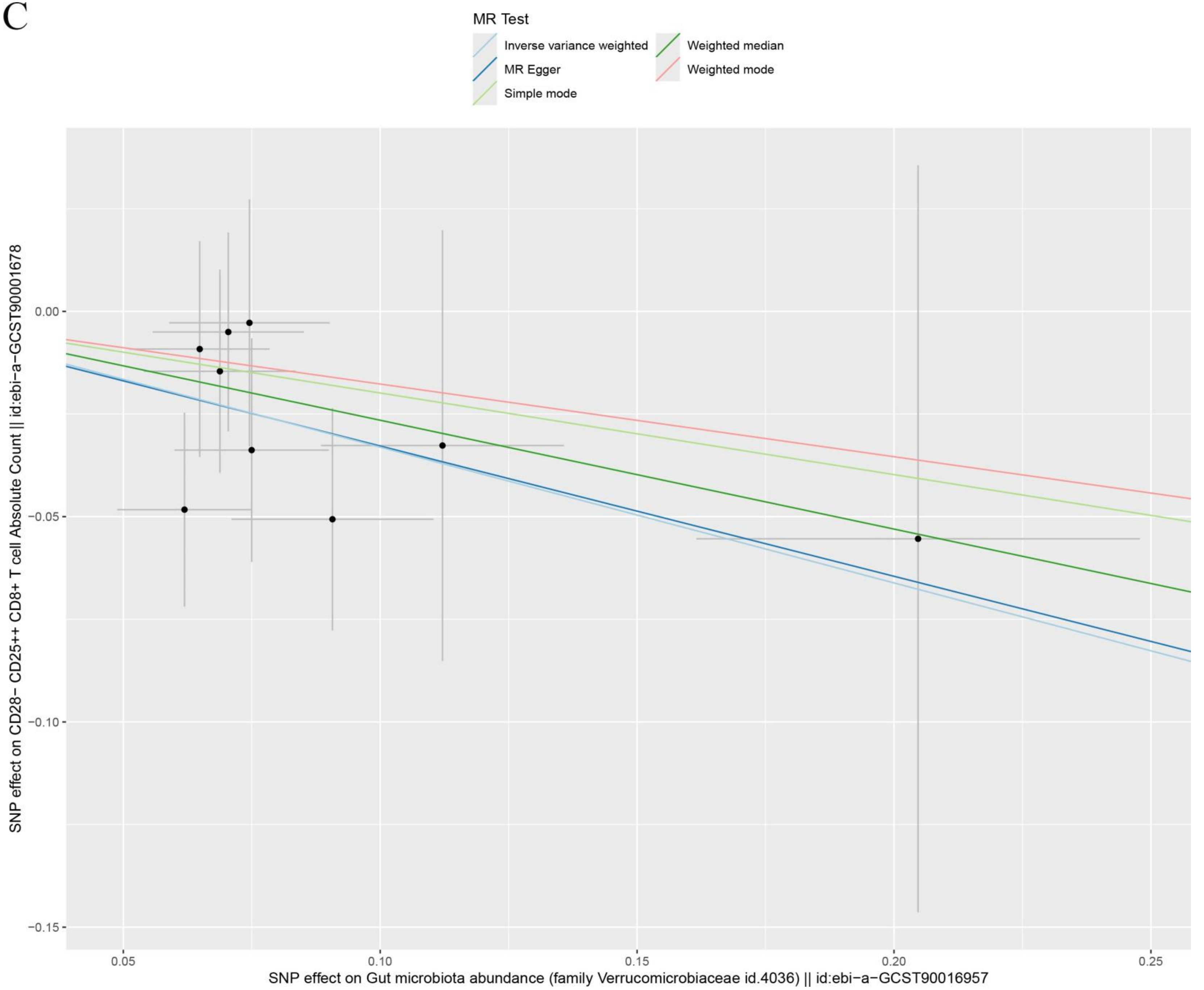
MR Test
Inverse variance weighted
MR Egger
Simple mode
Weighted median
Weighted mode
SNP effect on CD28− CD25++ CD8+ T cell Absolute Count || id:ebi−a−GCST90001678
0.00
−0.05
−0.10
−0.15
0.05
0.10
0.15
0.20
0.25
SNP effect on Gut microbiota abundance (family Verrucomicrobiaceae id.4036) || id:ebi−a−GCST90016957

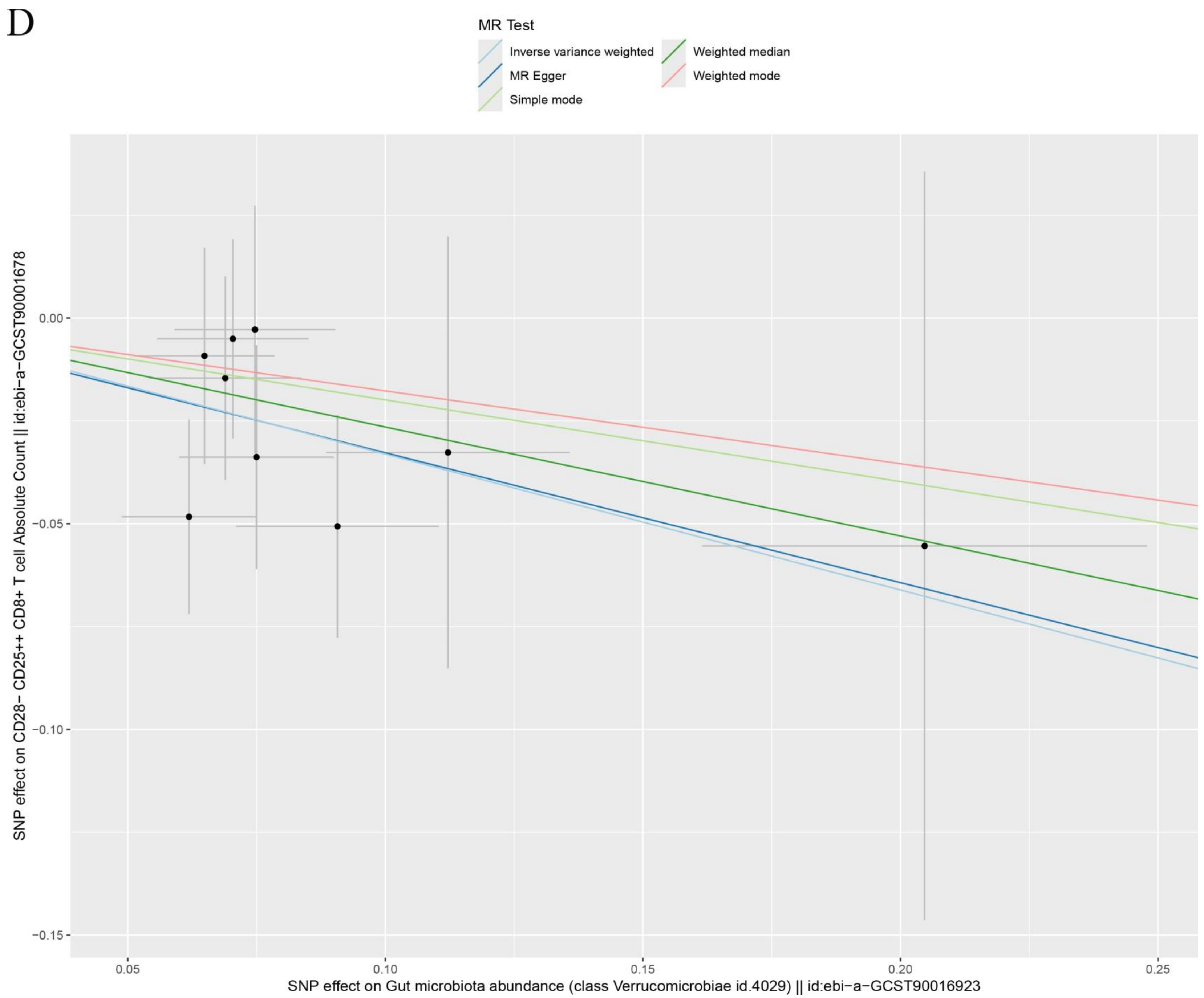
D
MR Test
Inverse variance weighted
MR Egger
Simple mode
Weighted median
Weighted mode
SNP effect on CD28− CD25++ CD8+ T cell Absolute Count || id:ebi−a−GCST90001678
0.00
−0.05
−0.10
−0.15
0.05
0.10
0.15
0.20
0.25
SNP effect on Gut microbiota abundance (class Verrucomicrobiae id.4029) || id:ebi−a−GCST90016923

E

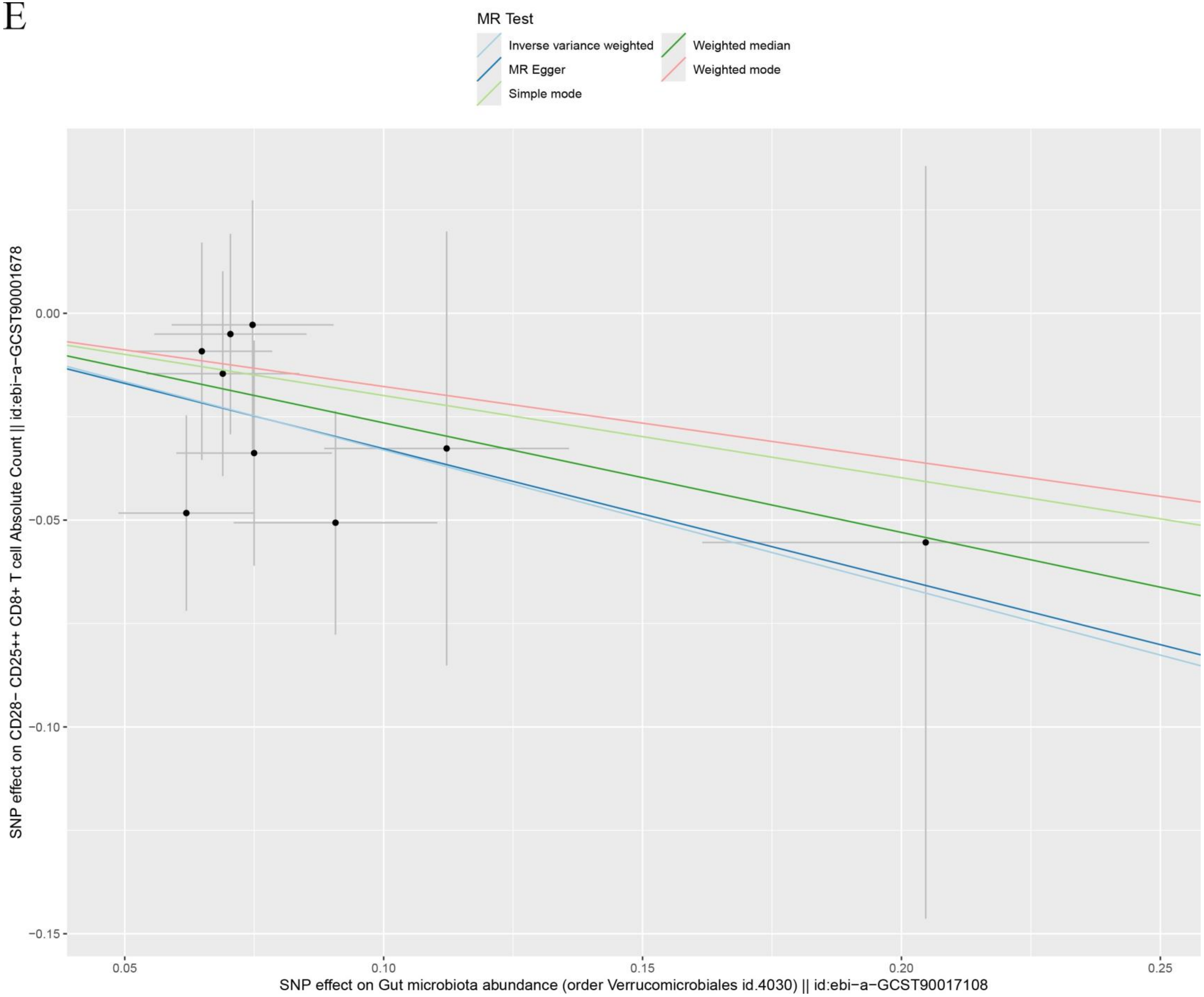

MR Test
Inverse variance weighted
MR Egger
Simple mode
Weighted median
Weighted mode
0.00
−0.05
−0.10
−0.15
0.05
0.10
0.15
0.20
0.25
SNP effect on CD28− CD25++ CD8+ T cell Absolute Count || id:ebi−a−GCST90001678
SNP effect on Gut microbiota abundance (order Verrucomicrobiales id.4030) || id:ebi−a−GCST90017108

F

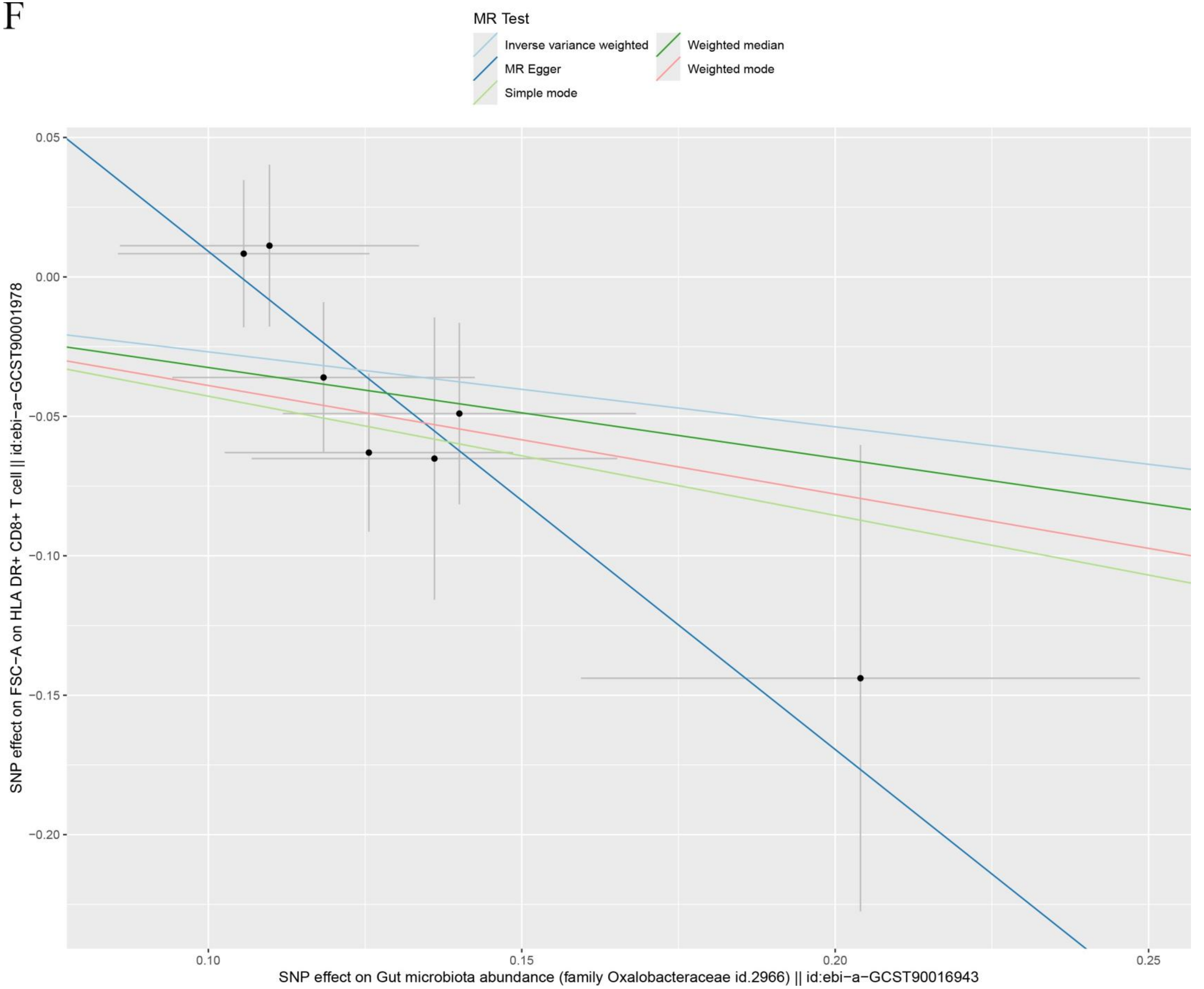
MR Test
Inverse variance weighted
MR Egger
Simple mode
Weighted median
Weighted mode
0.05
0.00
−0.05
−0.10
−0.15
−0.20
SNP effect on FSC−A on HLA DR+ CD8+ T cell || id:ebi−a−GCST90001978
0.10
0.15
0.20
0.25
SNP effect on Gut microbiota abundance (family Oxalobacteraceae id.2966) || id:ebi−a−GCST90016943

G

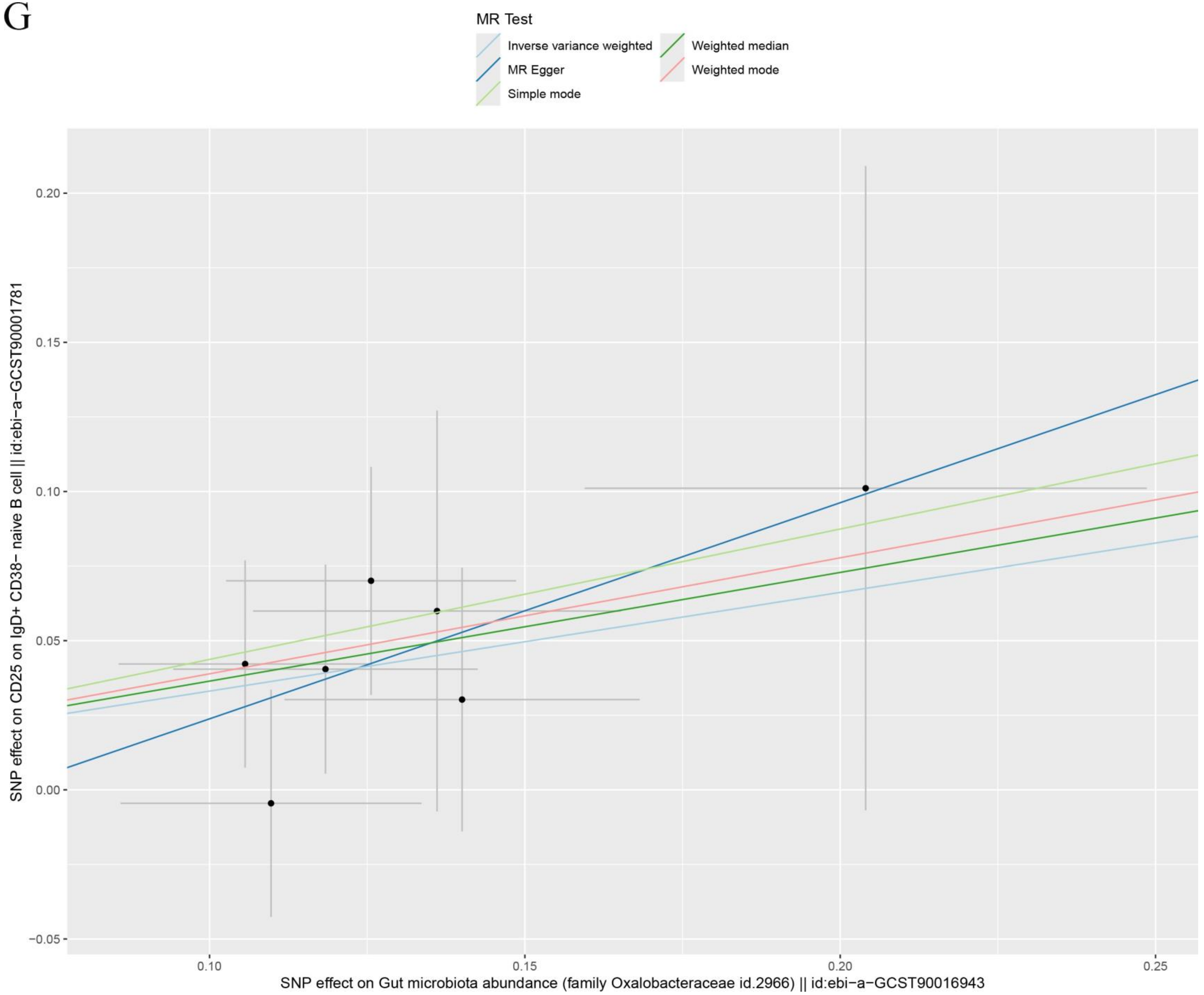
MR Test
Inverse variance weighted
MR Egger
Simple mode
Weighted median
Weighted mode
SNP effect on CD25 on IgD+ CD38− naive B cell || id:ebi−a−GCST90001781
0.20
0.15
0.10
0.05
0.00
−0.05
0.10
0.15
0.20
0.25
SNP effect on Gut microbiota abundance (family Oxalobacteraceae id.2966) || id:ebi−a−GCST90016943

H

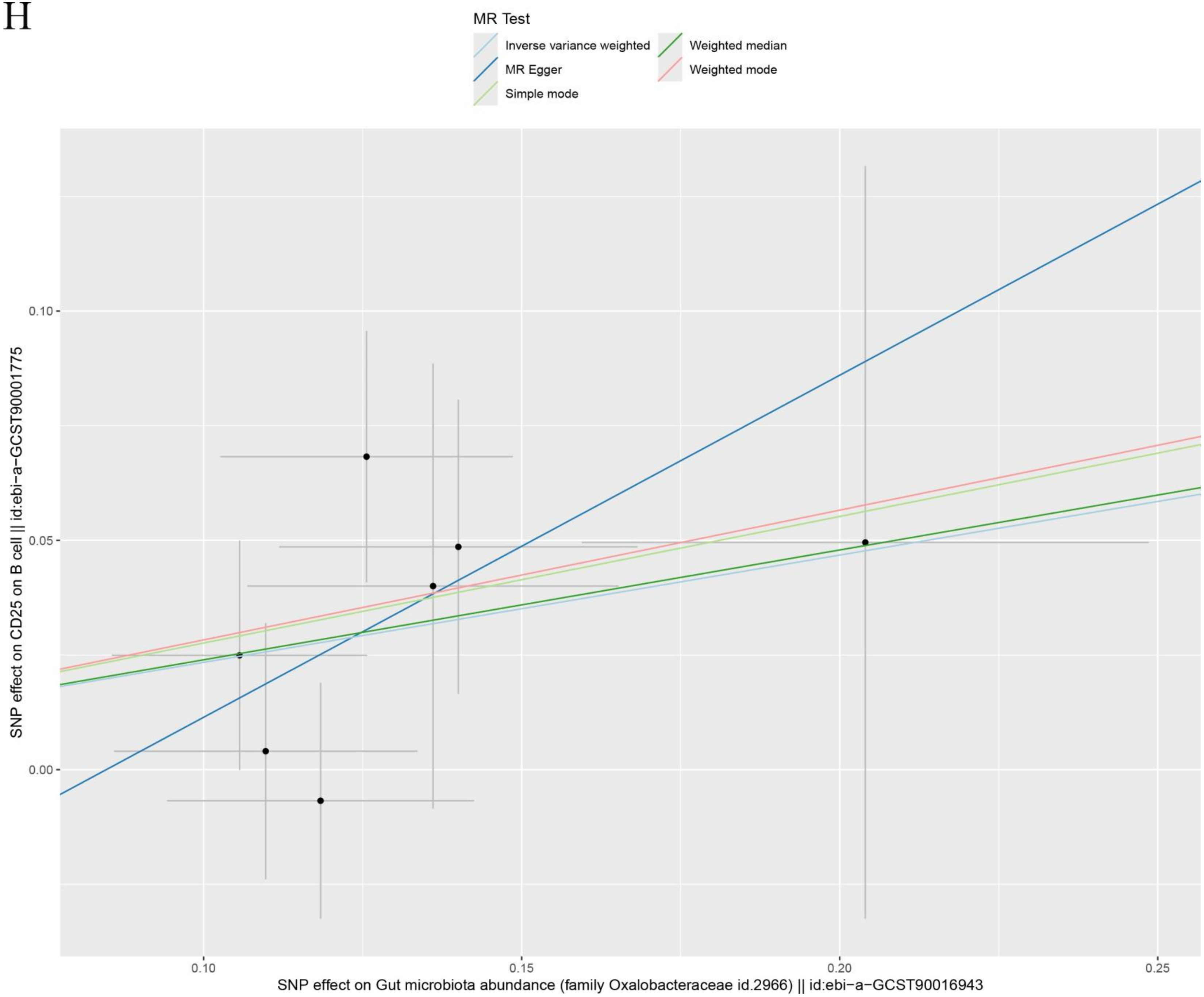
MR Test
Inverse variance weighted
MR Egger
Simple mode
Weighted median
Weighted mode
0.10
0.05
0.00
SNP effect on CD25 on B cell || id:ebi-a-GCST90001775
0.10
0.15
0.20
0.25
SNP effect on Gut microbiota abundance (family Oxalobacteraceae id.2966) || id:ebi-a-GCST90016943

I

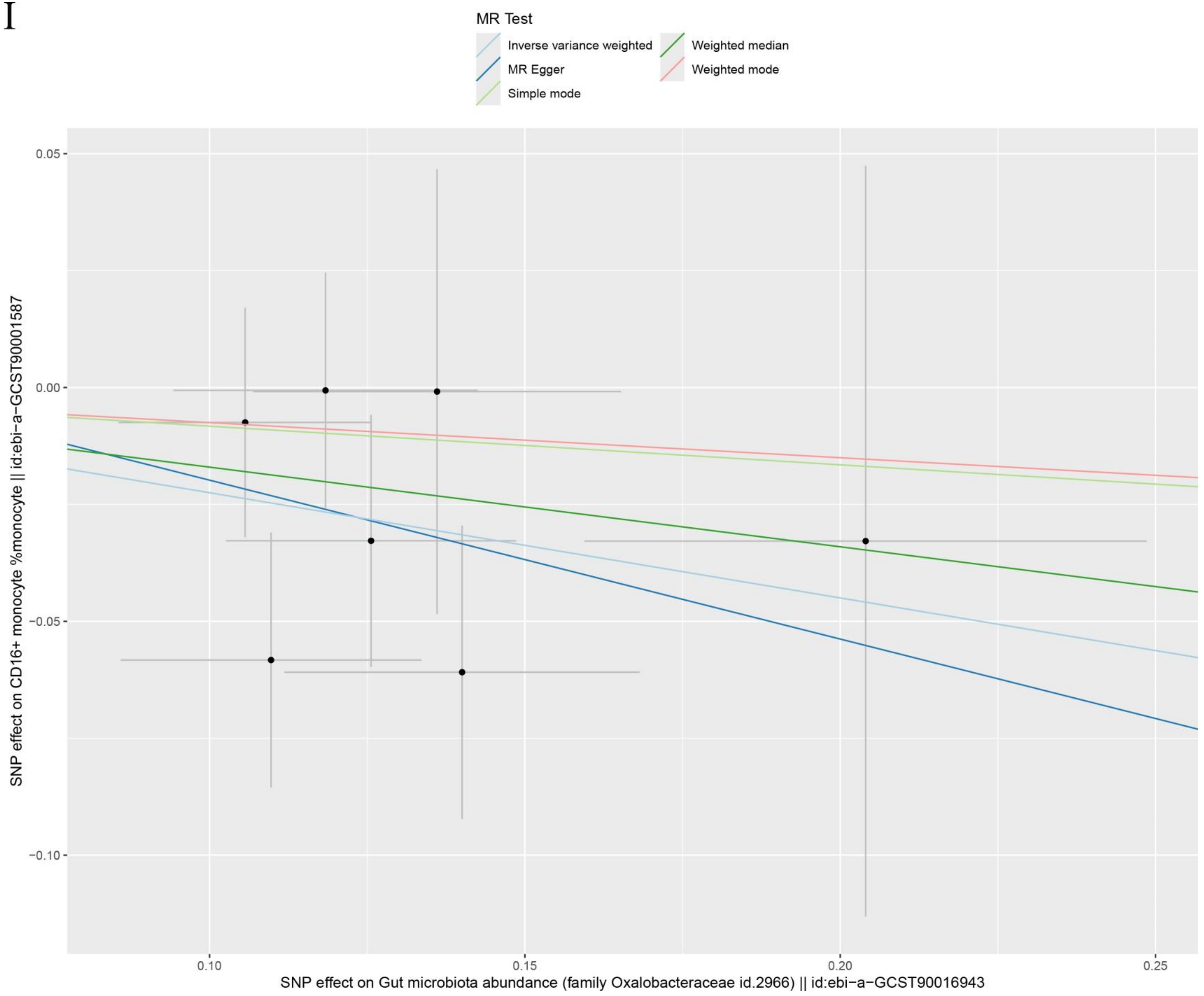

MR Test
Inverse variance weighted
MR Egger
Simple mode
Weighted median
Weighted mode
0.05
0.00
−0.05
−0.10
0.10
0.15
0.20
0.25
SNP effect on CD16+ monocyte %monocyte || id:ebi−a−GCST90001587
SNP effect on Gut microbiota abundance (family Oxalobacteraceae id.2966) || id:ebi−a−GCST90016943

J

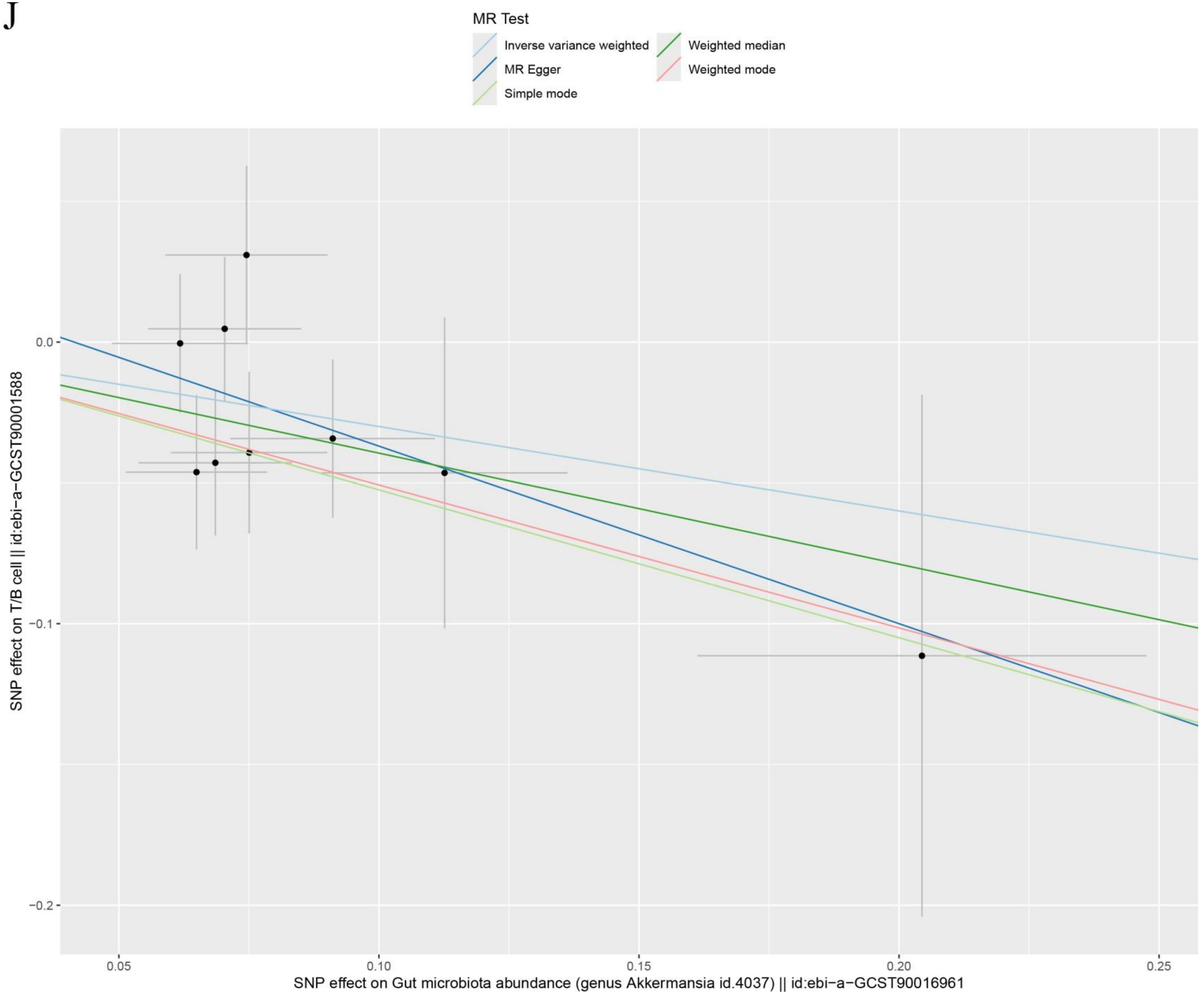
MR Test
Inverse variance weighted
MR Egger
Simple mode
Weighted median
Weighted mode
0.0
−0.1
−0.2
0.05
0.10
0.15
0.20
0.25
SNP effect on T/B cell || id:ebi−a−GCST90001588
SNP effect on Gut microbiota abundance (genus Akkermansia id.4037) || id:ebi−a−GCST90016961

K

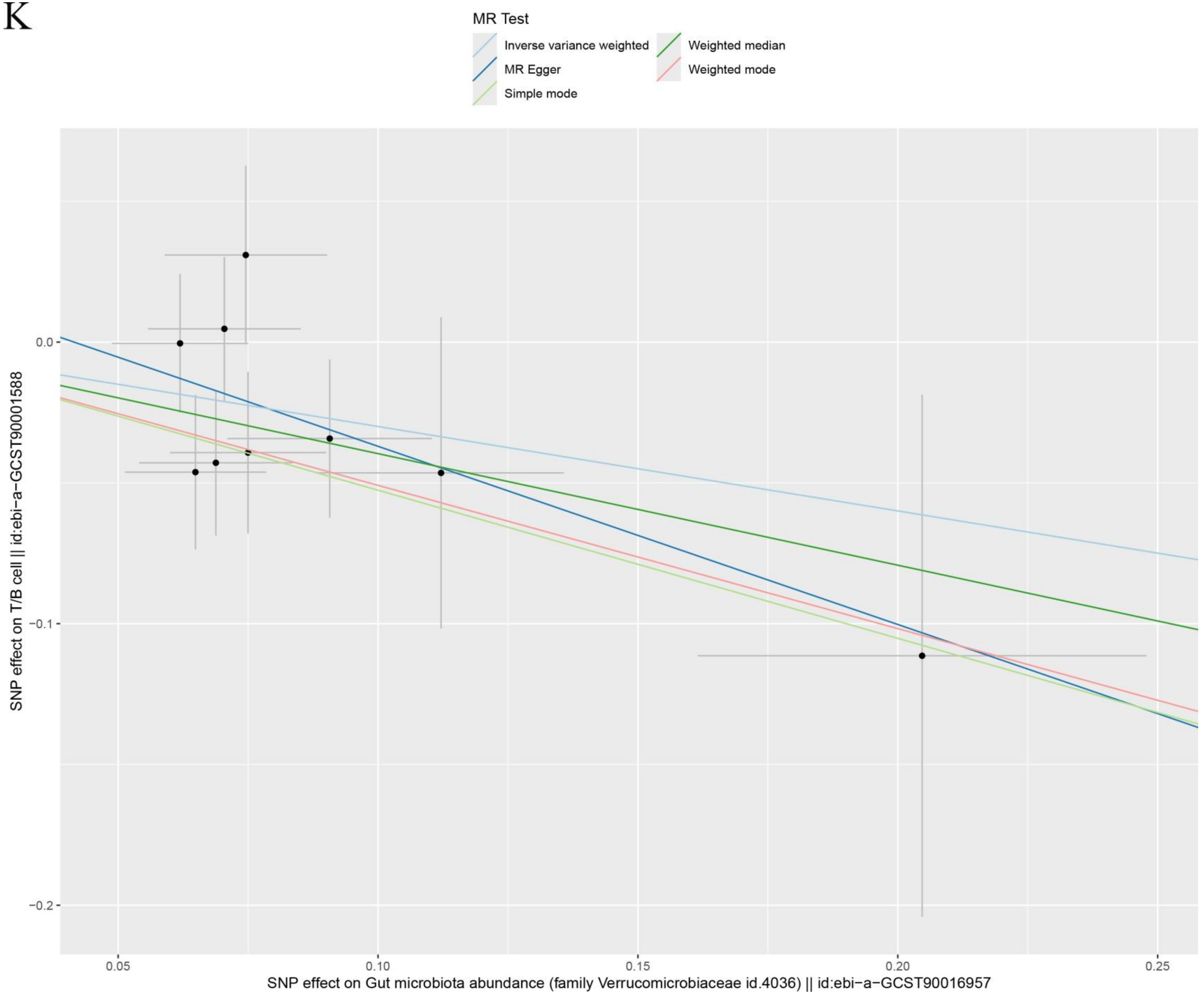
MR Test
Inverse variance weighted
MR Egger
Simple mode
Weighted median
Weighted mode
0.0
−0.1
−0.2
0.05
0.10
0.15
0.20
0.25
SNP effect on T/B cell || id:ebi−a−GCST90001588
SNP effect on Gut microbiota abundance (family Verrucomicrobiaceae id.4036) || id:ebi−a−GCST90016957

L

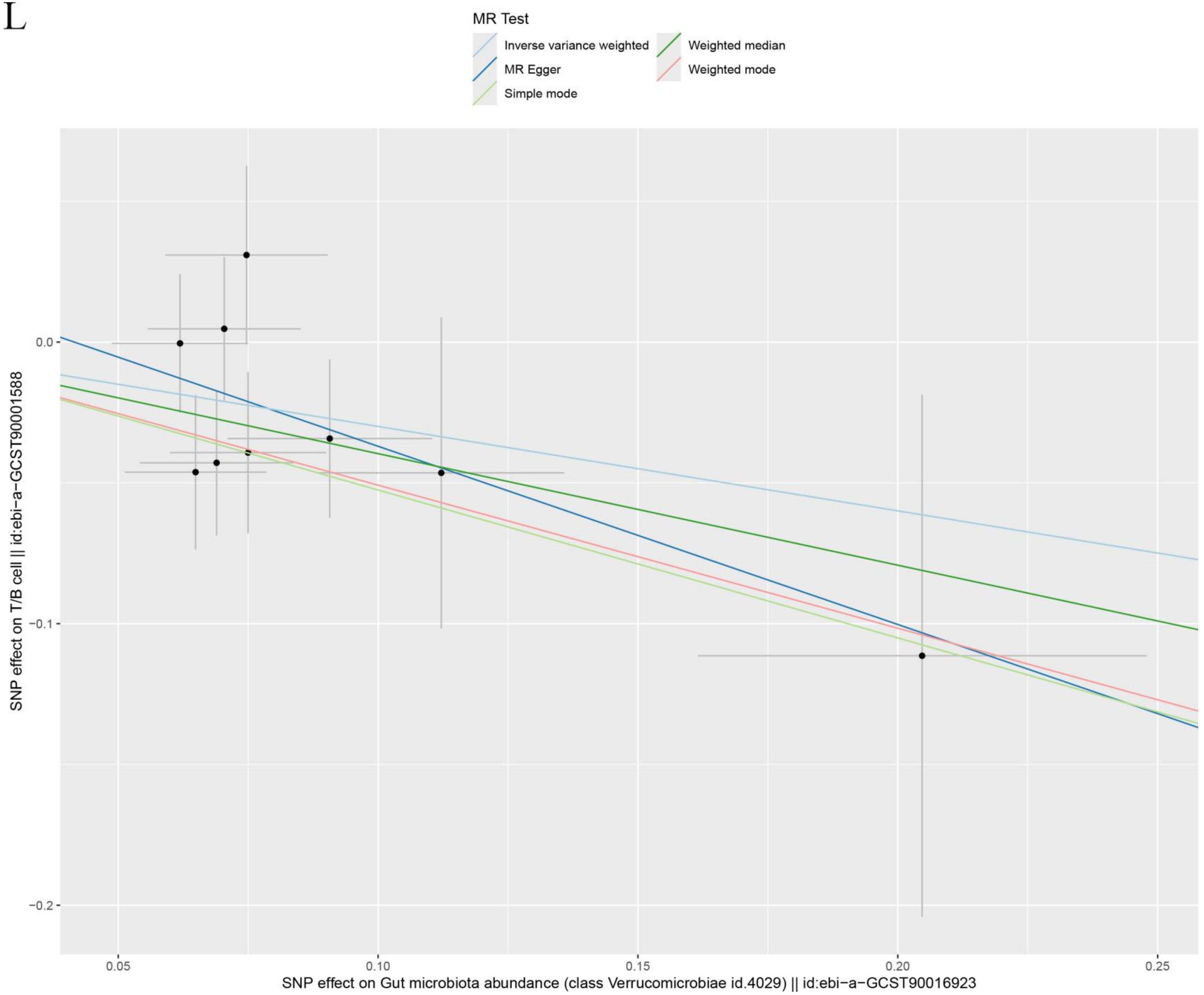

MR Test
Inverse variance weighted
MR Egger
Simple mode
Weighted median
Weighted mode
0.0
−0.1
−0.2
0.05
0.10
0.15
0.20
0.25
SNP effect on T/B cell || id:ebi−a−GCST90001588
SNP effect on Gut microbiota abundance (class Verrucomicrobiae id.4029) || id:ebi−a−GCST90016923

M

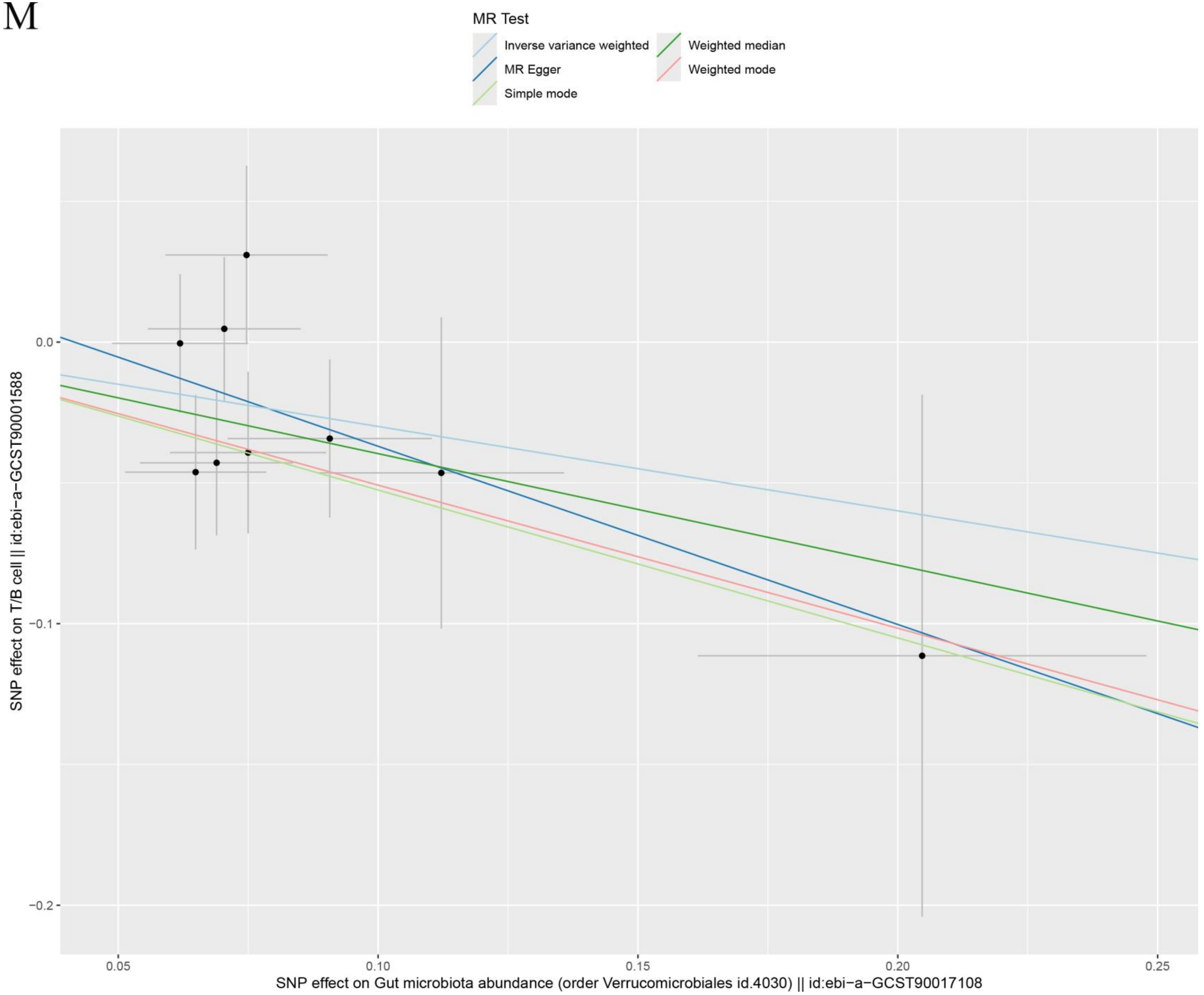
MR Test
Inverse variance weighted
MR Egger
Simple mode
Weighted median
Weighted mode
0.0
−0.1
−0.2
0.05
0.10
0.15
0.20
0.25
SNP effect on T/B cell || id:ebi−a−GCST90001588
SNP effect on Gut microbiota abundance (order Verrucomicrobiales id.4030) || id:ebi−a−GCST90017108

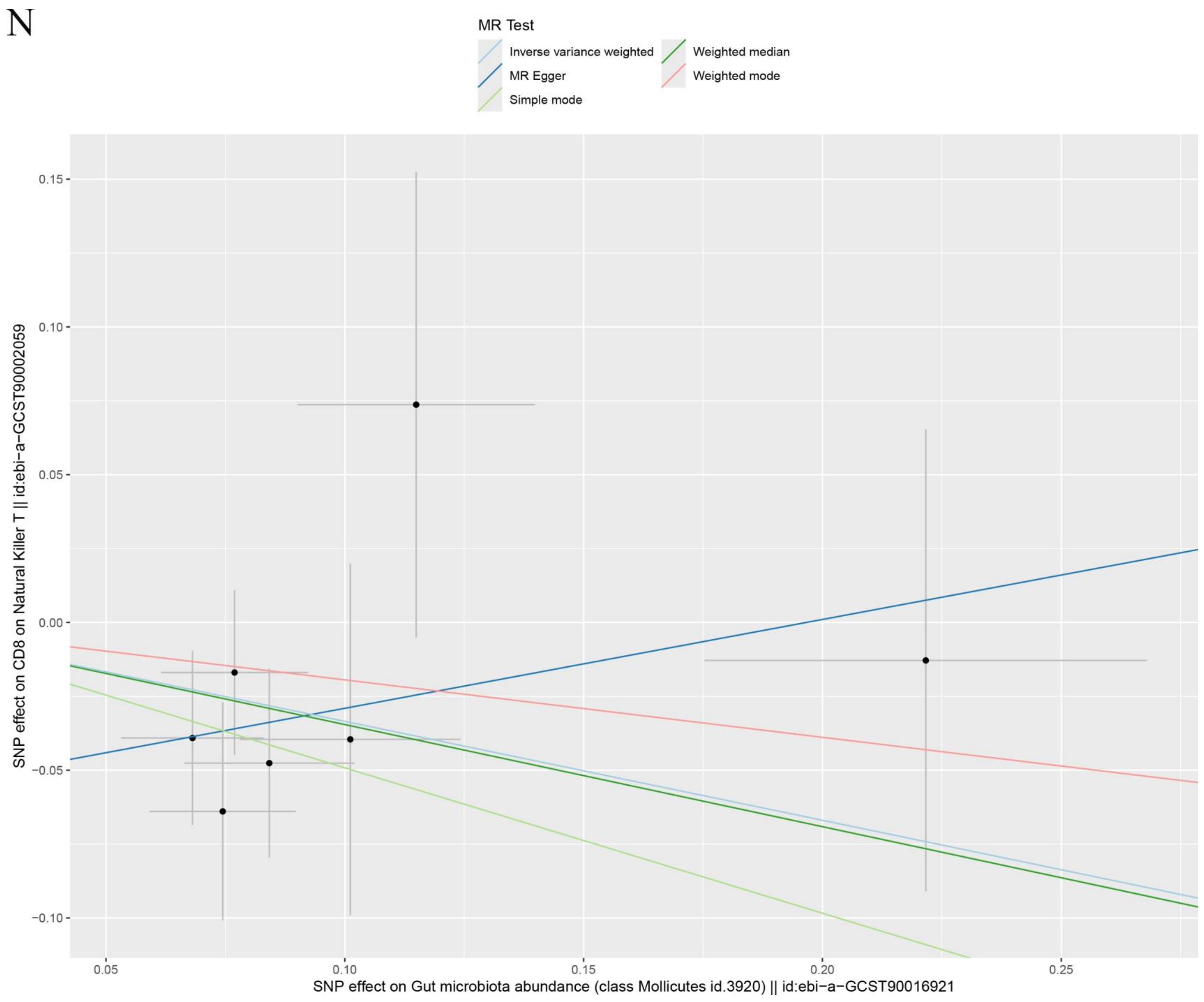
N
MR Test
Inverse variance weighted
MR Egger
Simple mode
Weighted median
Weighted mode
SNP effect on CD8 on Natural Killer T || id:ebi-a-GCST90002059
0.15
0.10
0.05
0.00
−0.05
−0.10
0.05
0.10
0.15
0.20
0.25
SNP effect on Gut microbiota abundance (class Mollicutes id.3920) || id:ebi-a-GCST90016921

O

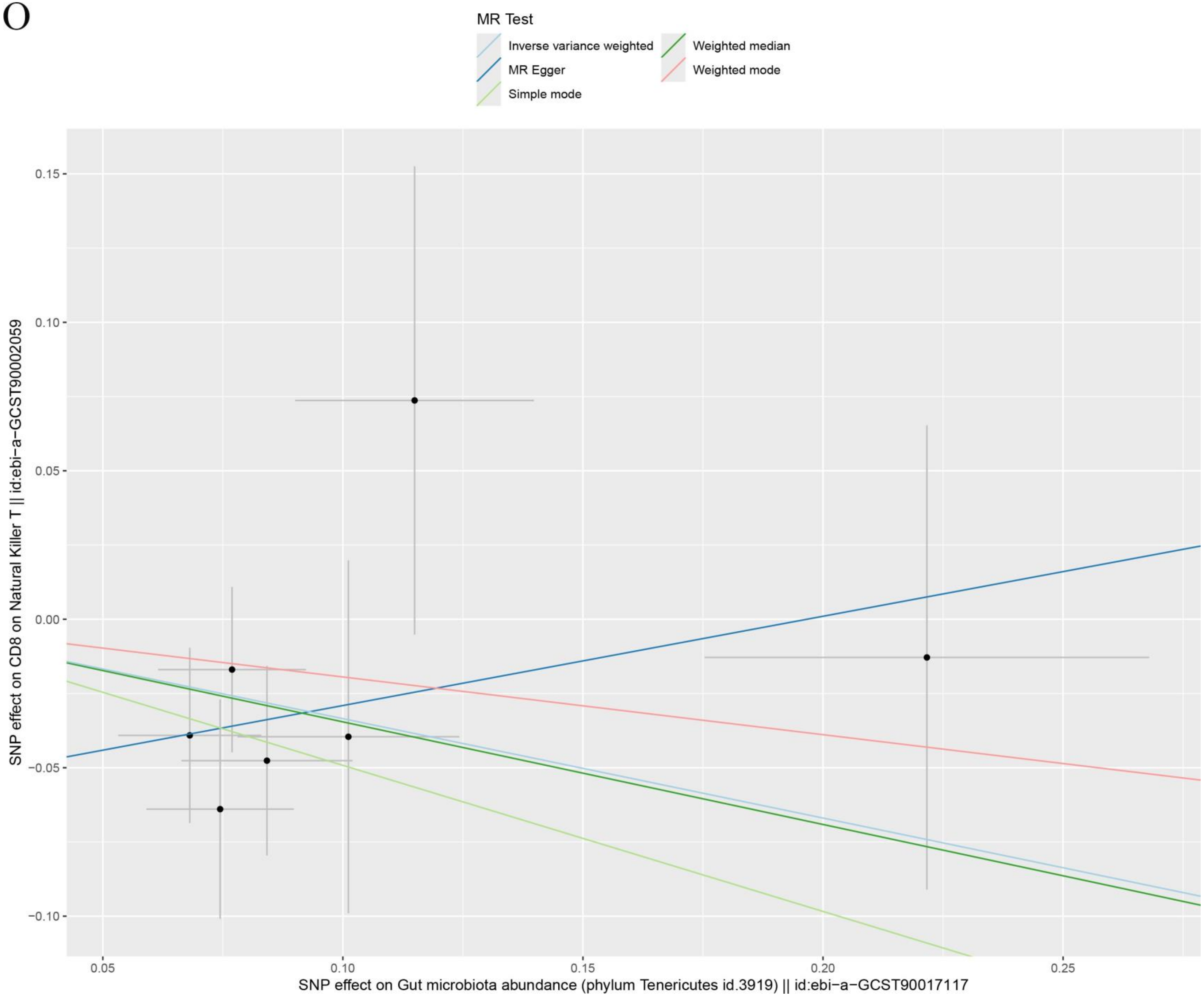
MR Test
Inverse variance weighted
MR Egger
Simple mode
Weighted median
Weighted mode
0.15
0.10
0.05
0.00
−0.05
−0.10
SNP effect on CD8 on Natural Killer T || id:ebi−a−GCST90002059
0.05
0.10
0.15
0.20
0.25
SNP effect on Gut microbiota abundance (phylum Tenericutes id.3919) || id:ebi−a−GCST90017117
