# Supplementary material for: Causal Mediation Role of Immune Cells in Gut Microbiota–Pneumonia Associations: A Mendelian Randomisation Study
Source: J Cell Mol Med. 2025 Sep 11;29(17):e70839. doi: 10.1111/jcmm.70839 (PMC12425809; doi:10.1111/jcmm.70839)
Supplement: Supplementary file 2 — Figure S2: Sensitivity analysis of gut microbiota and immune cell characteristics Mendelian randomization (Forest plot). [file JCMM-29-e70839-s009.pdf]

Supplementary Figure 6 Sensitivity analysis of gut microbiota and immune cell characteristics Mendelian randomization (Forest plot).

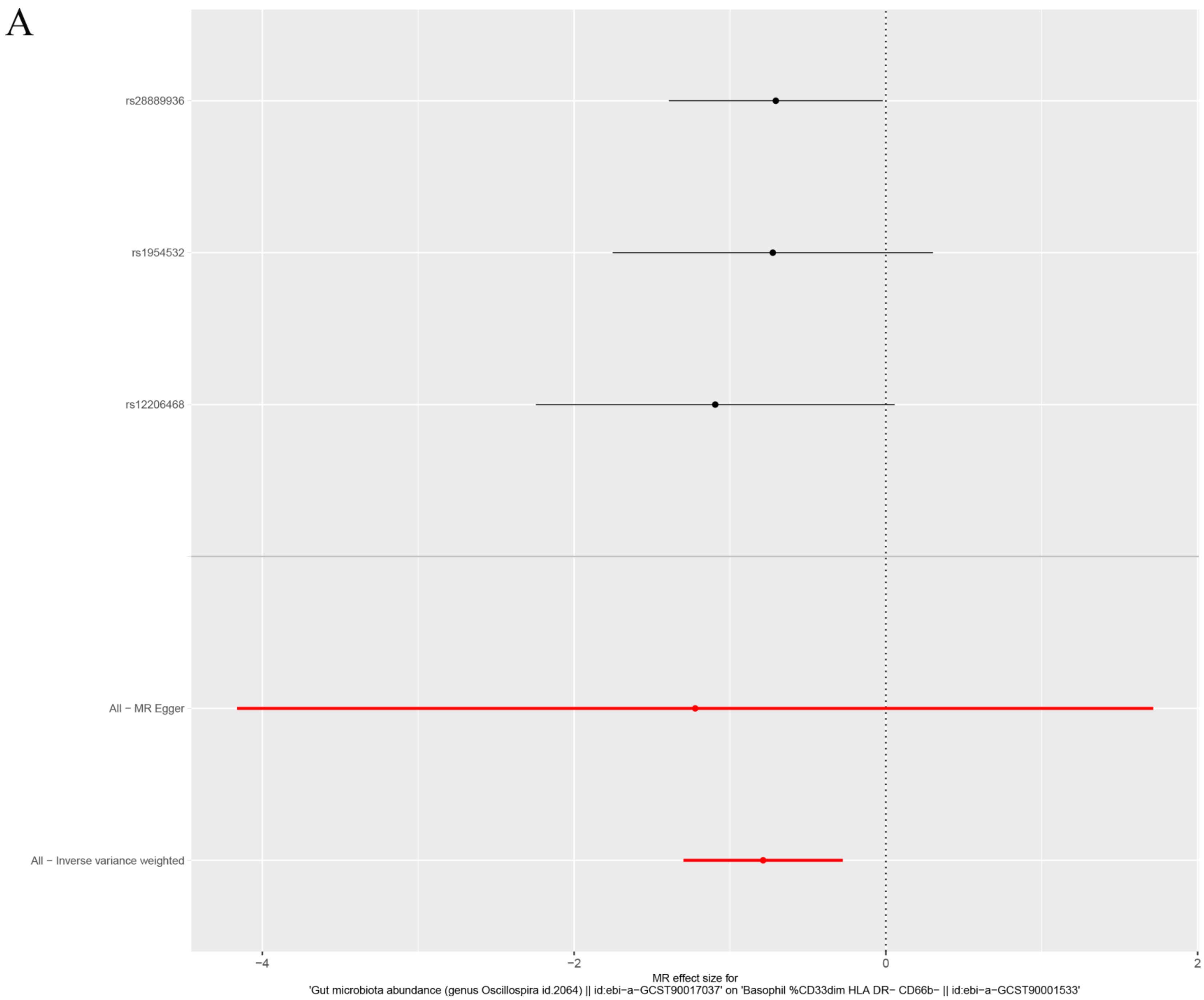

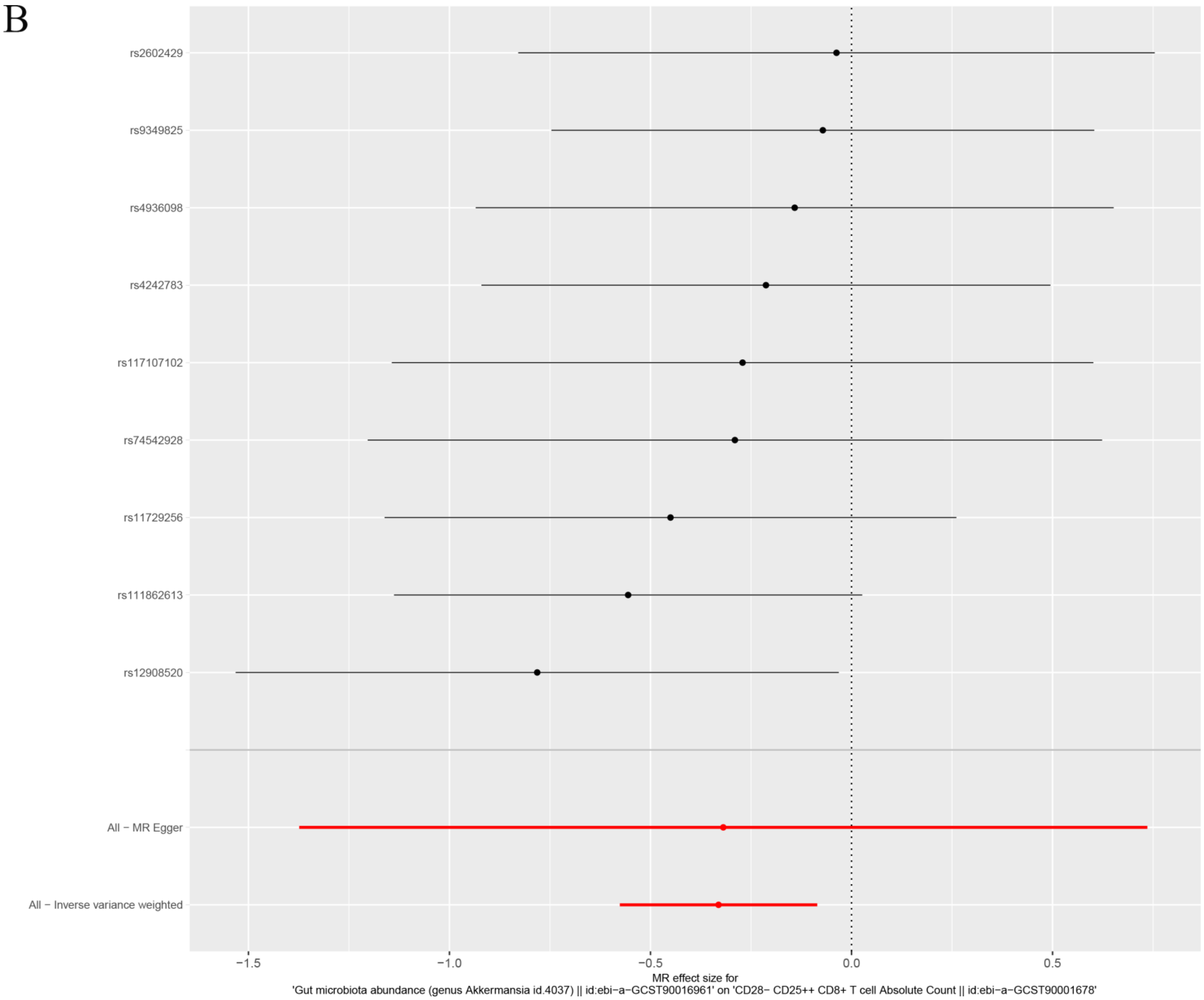
B
rs2602429
rs9349825
rs4936098
rs4242783
rs117107102
rs74542928
rs11729256
rs111862613
rs12908520
All − MR Egger
All − Inverse variance weighted
−1.5
−1.0
−0.5
0.0
0.5
MR effect size for
'Gut microbiota abundance (genus Akkermansia id.4037) || id:ebi−a−GCST90016961' on 'CD28− CD25++ CD8+ T cell Absolute Count || id:ebi−a−GCST90001678'

C

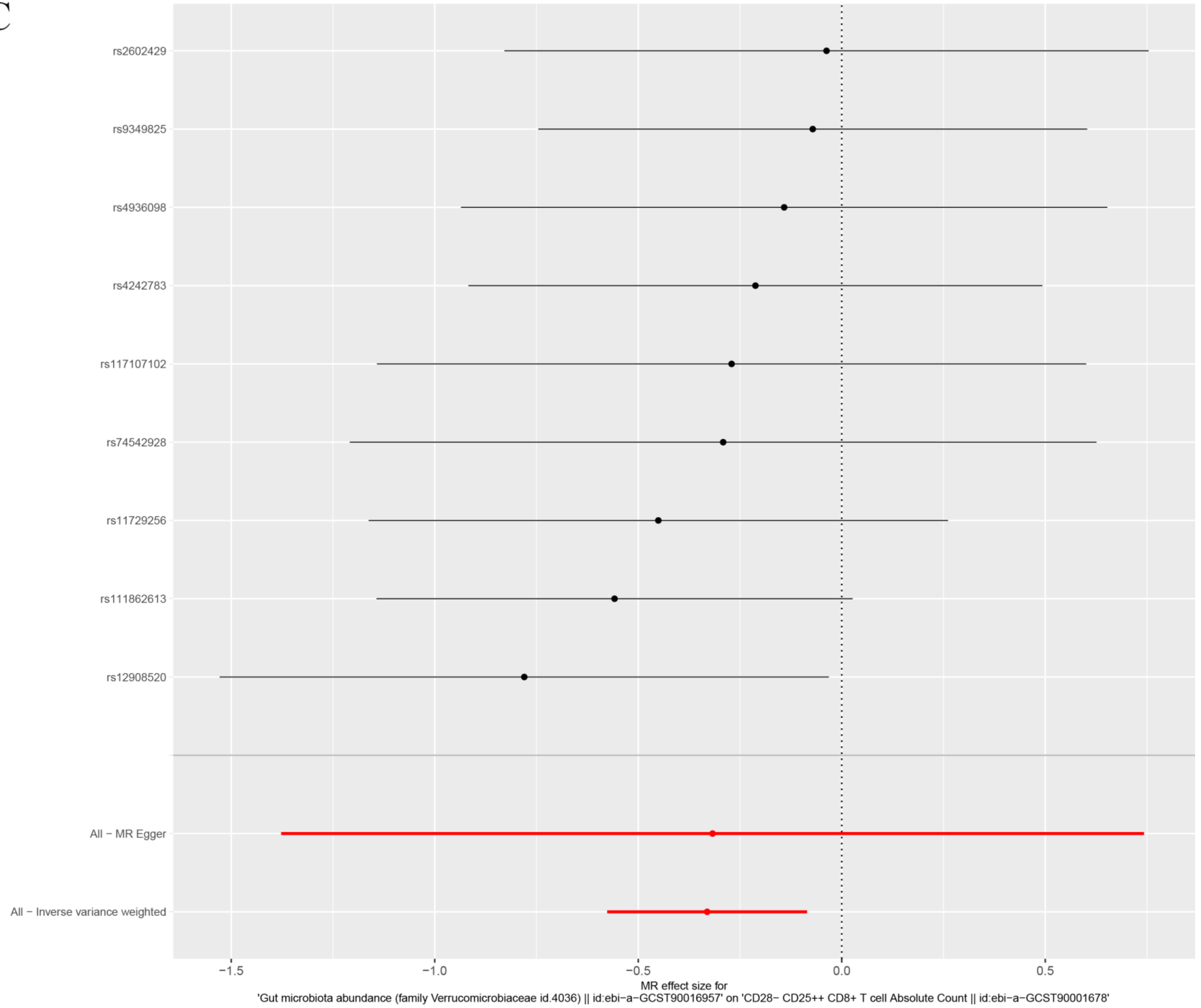
rs2602429
rs9349825
rs4936098
rs4242783
rs117107102
rs74542928
rs11729256
rs111862613
rs12908520
All − MR Egger
All − Inverse variance weighted
−1.5
−1.0
−0.5
0.0
0.5
MR effect size for
'Gut microbiota abundance (family Verrucomicrobiaceae id.4036) || id:ebi−a−GCST90016957' on 'CD28− CD25++ CD8+ T cell Absolute Count || id:ebi−a−GCST90001678'

D

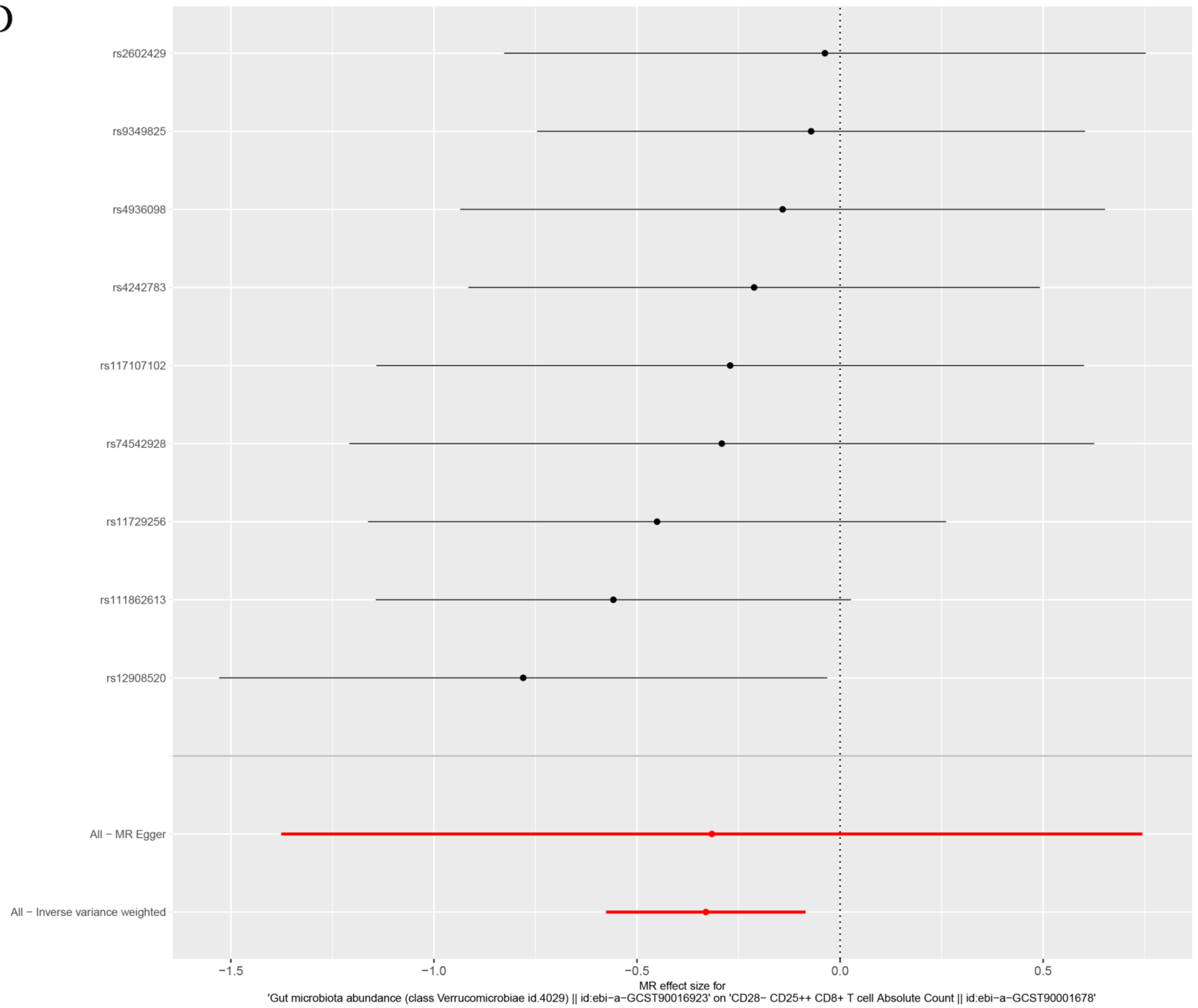

rs2602429
rs9349825
rs4936098
rs4242783
rs117107102
rs74542928
rs11729256
rs111862613
rs12908520
All − MR Egger
All − Inverse variance weighted
−1.5
−1.0
−0.5
0.0
0.5
MR effect size for
'Gut microbiota abundance (class Verrucomicrobiae id.4029) || id:ebi−a−GCST90016923' on 'CD28− CD25++ CD8+ T cell Absolute Count || id:ebi−a−GCST90001678'

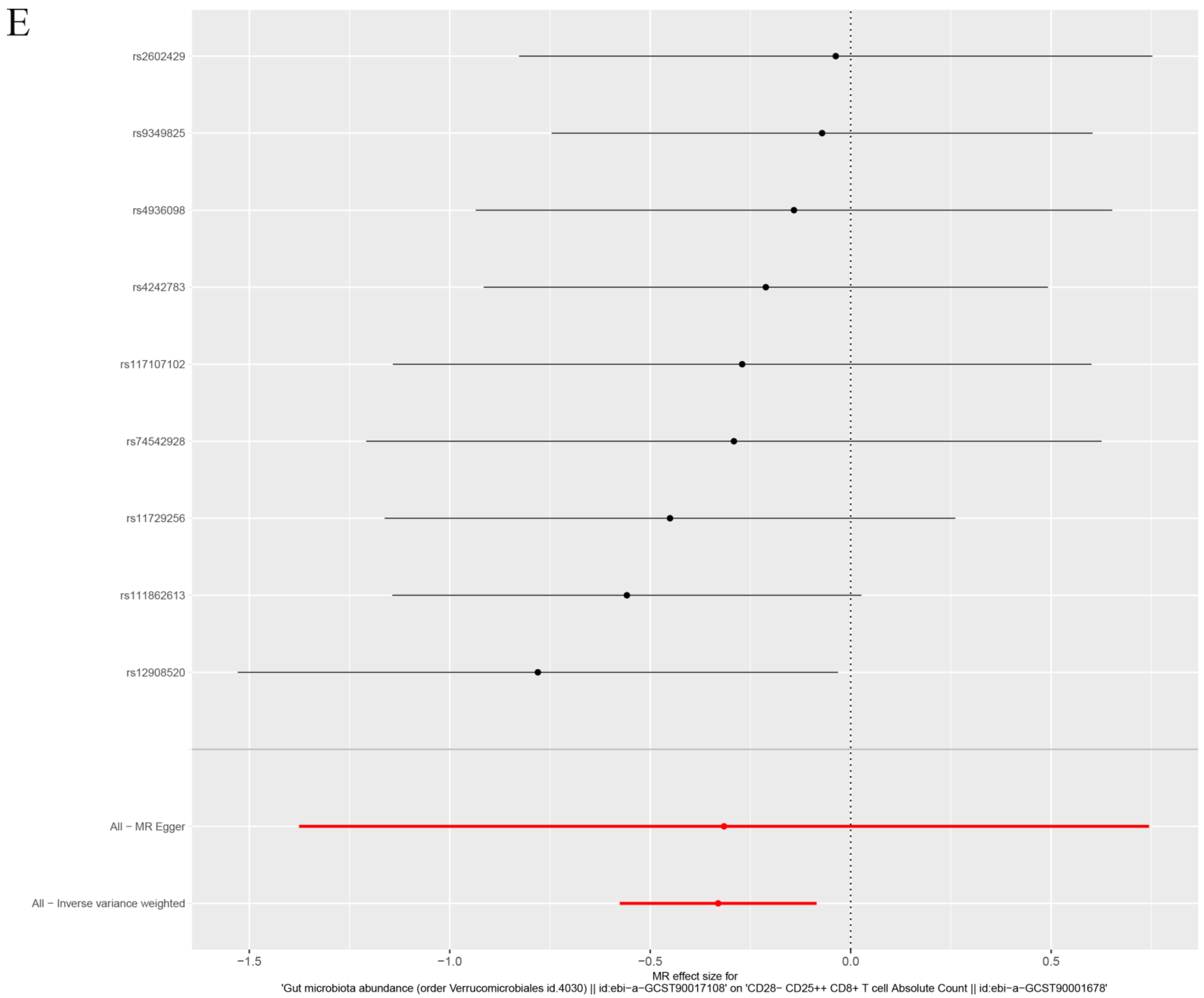
E
rs2602429
rs9349825
rs4936098
rs4242783
rs117107102
rs74542928
rs11729256
rs111862613
rs12908520
All − MR Egger
All − Inverse variance weighted
−1.5
−1.0
−0.5
0.0
0.5
MR effect size for
'Gut microbiota abundance (order Verrucomicrobiales id.4030) || id:ebi−a−GCST90017108' on 'CD28− CD25++ CD8+ T cell Absolute Count || id:ebi−a−GCST90001678'

F

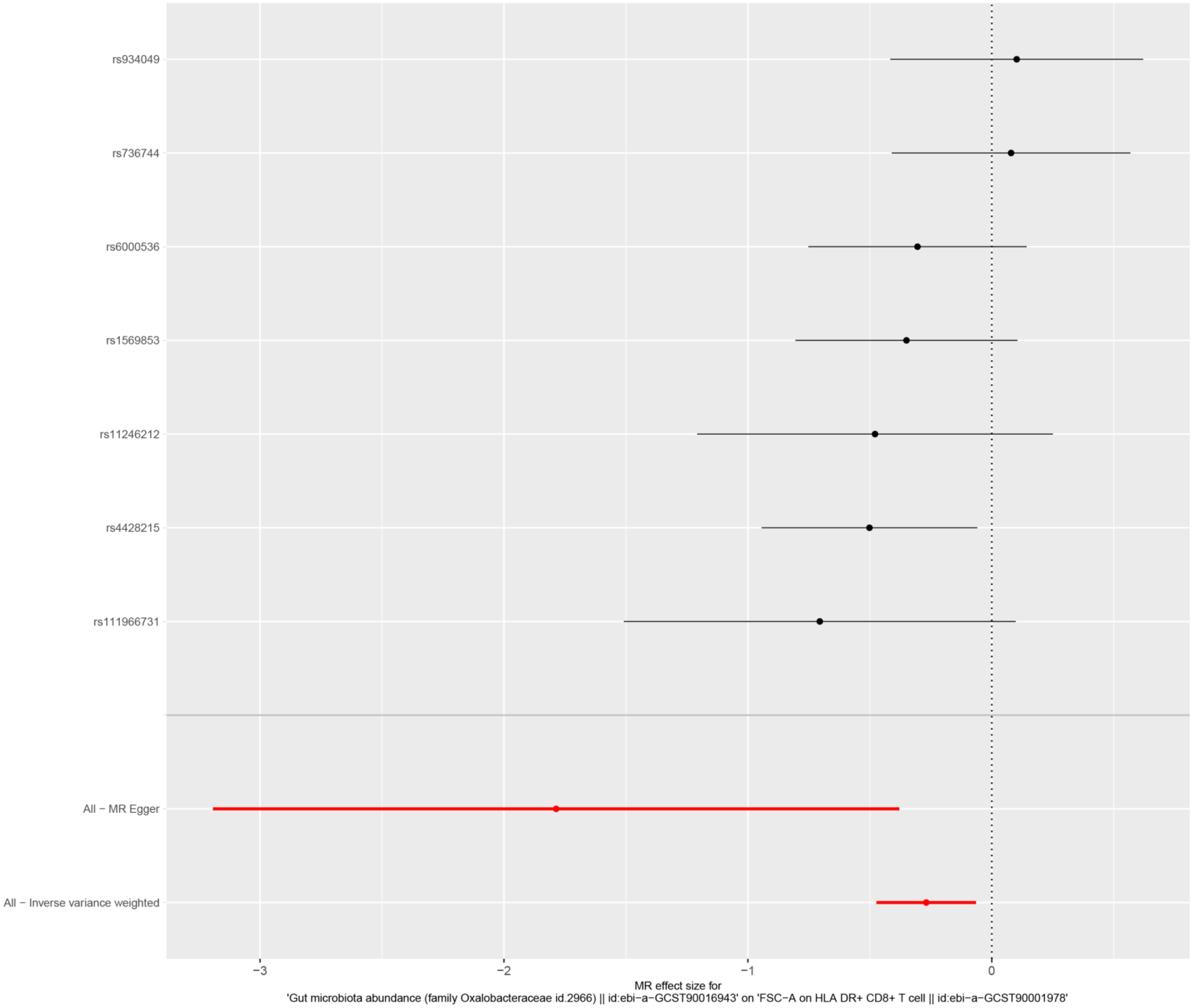

rs934049
rs736744
rs6000536
rs1569853
rs11246212
rs4428215
rs111966731
All − MR Egger
All − Inverse variance weighted
−3
−2
−1
0
MR effect size for
'Gut microbiota abundance (family Oxalobacteraceae id.2966) || id:ebi−a−GCST90016943' on 'FSC−A on HLA DR+ CD8+ T cell || id:ebi−a−GCST90001978'

G

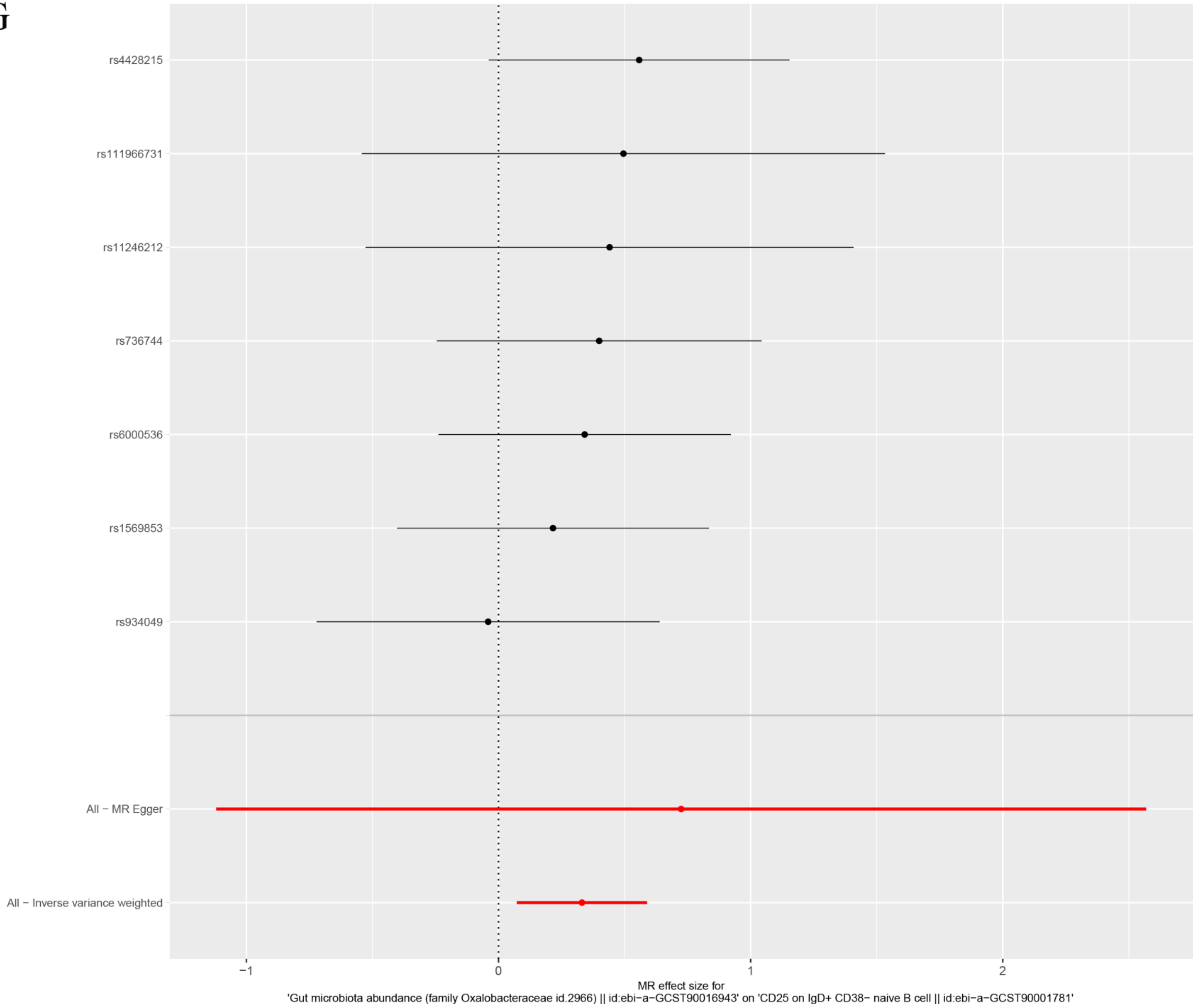

rs4428215
rs111966731
rs11246212
rs736744
rs6000536
rs1569853
rs934049
All − MR Egger
All − Inverse variance weighted
−1
0
1
2
MR effect size for
'Gut microbiota abundance (family Oxalobacteraceae id.2966) || id:ebi−a−GCST90016943' on 'CD25 on IgD+ CD38− naive B cell || id:ebi−a−GCST90001781'

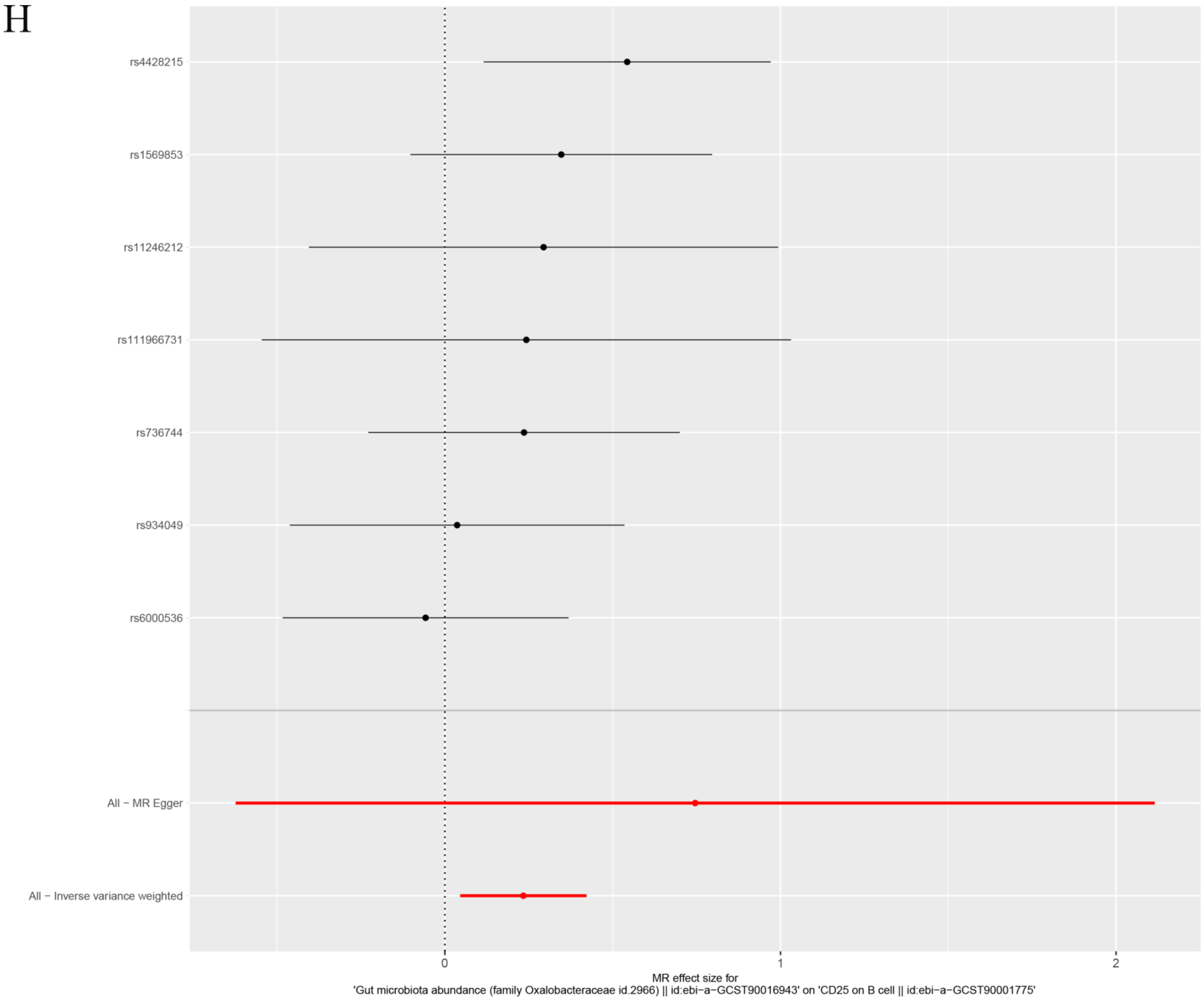
H
rs4428215
rs1569853
rs11246212
rs111966731
rs736744
rs934049
rs6000536
All − MR Egger
All − Inverse variance weighted
0
1
2
MR effect size for
'Gut microbiota abundance (family Oxalobacteraceae id.2966) || id:ebi−a−GCST90016943' on 'CD25 on B cell || id:ebi−a−GCST90001775'

I

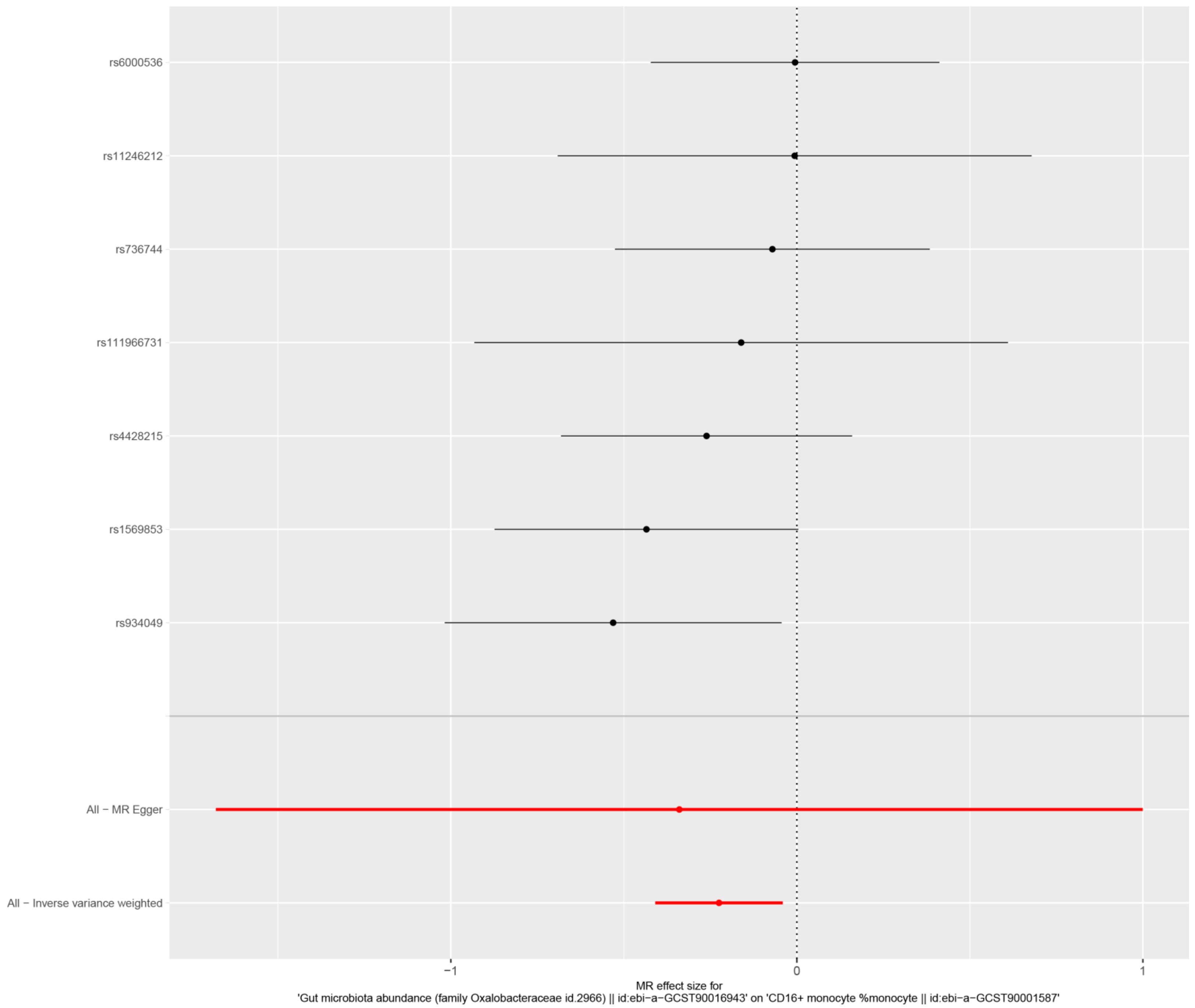

rs6000536
rs11246212
rs736744
rs111966731
rs4428215
rs1569853
rs934049
All − MR Egger
All − Inverse variance weighted
−1
0
1
MR effect size for
'Gut microbiota abundance (family Oxalobacteraceae id.2966) || id:ebi−a−GCST90016943' on 'CD16+ monocyte %monocyte || id:ebi−a−GCST90001587'

J

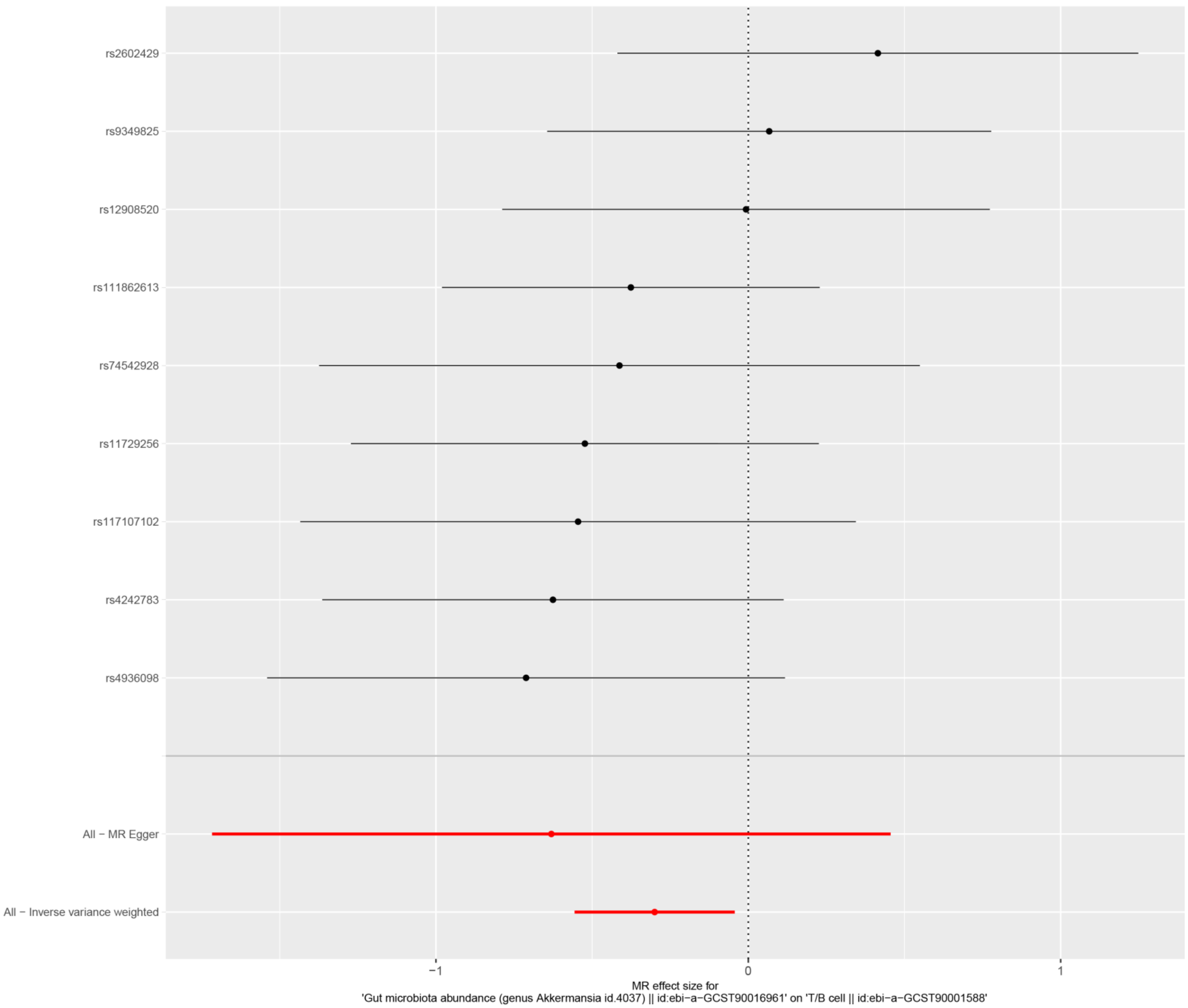
rs2602429
rs9349825
rs12908520
rs111862613
rs74542928
rs11729256
rs117107102
rs4242783
rs4936098
All − MR Egger
All − Inverse variance weighted
−1
0
1
MR effect size for
'Gut microbiota abundance (genus Akkermansia id.4037) || id:ebi−a−GCST90016961' on 'T/B cell || id:ebi−a−GCST90001588'

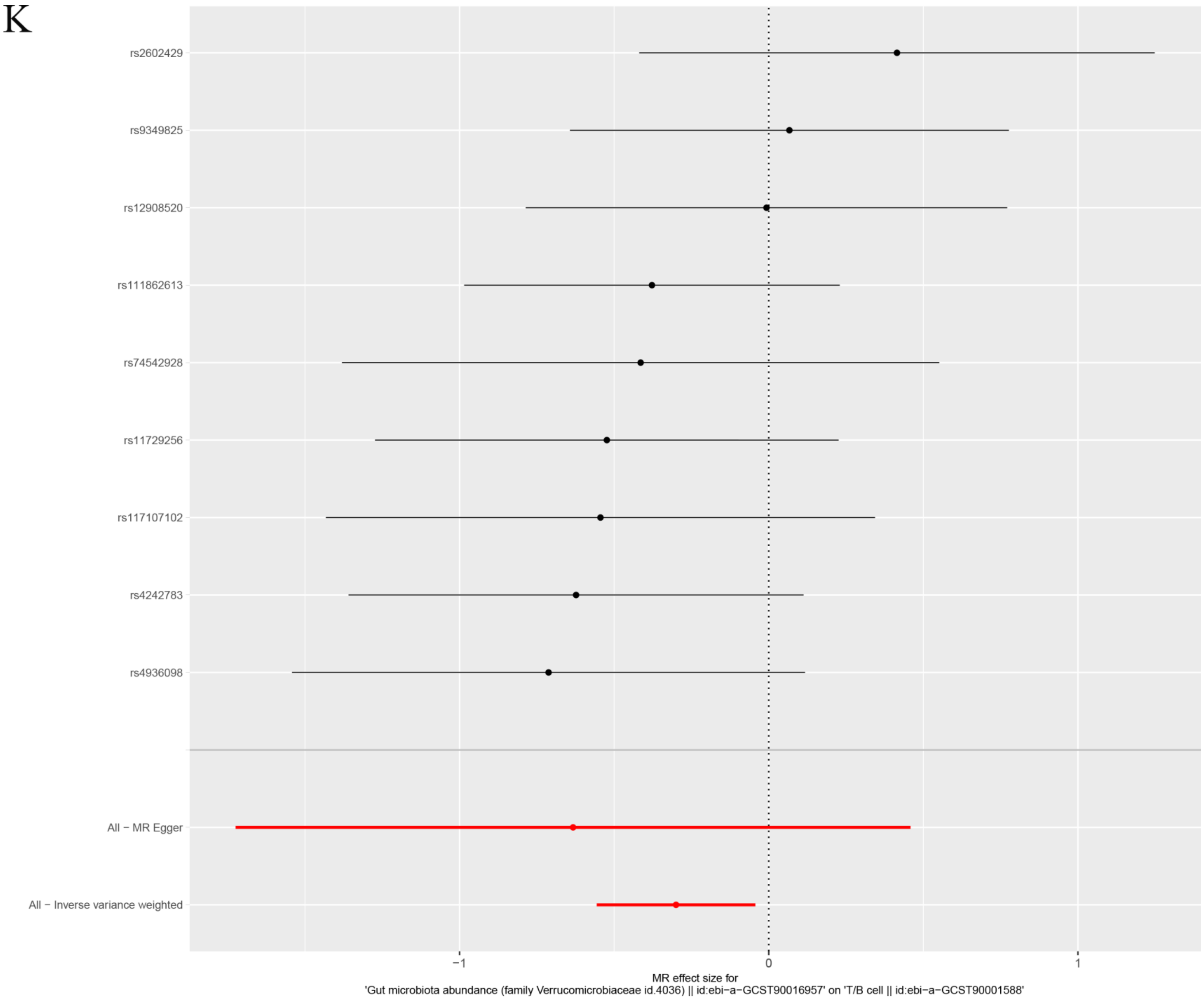

K
rs2602429
rs9349825
rs12908520
rs111862613
rs74542928
rs11729256
rs117107102
rs4242783
rs4936098
All − MR Egger
All − Inverse variance weighted
−1
0
1
MR effect size for
'Gut microbiota abundance (family Verrucomicrobiaceae id.4036) || id:ebi−a−GCST90016957' on 'T/B cell || id:ebi−a−GCST90001588'

L

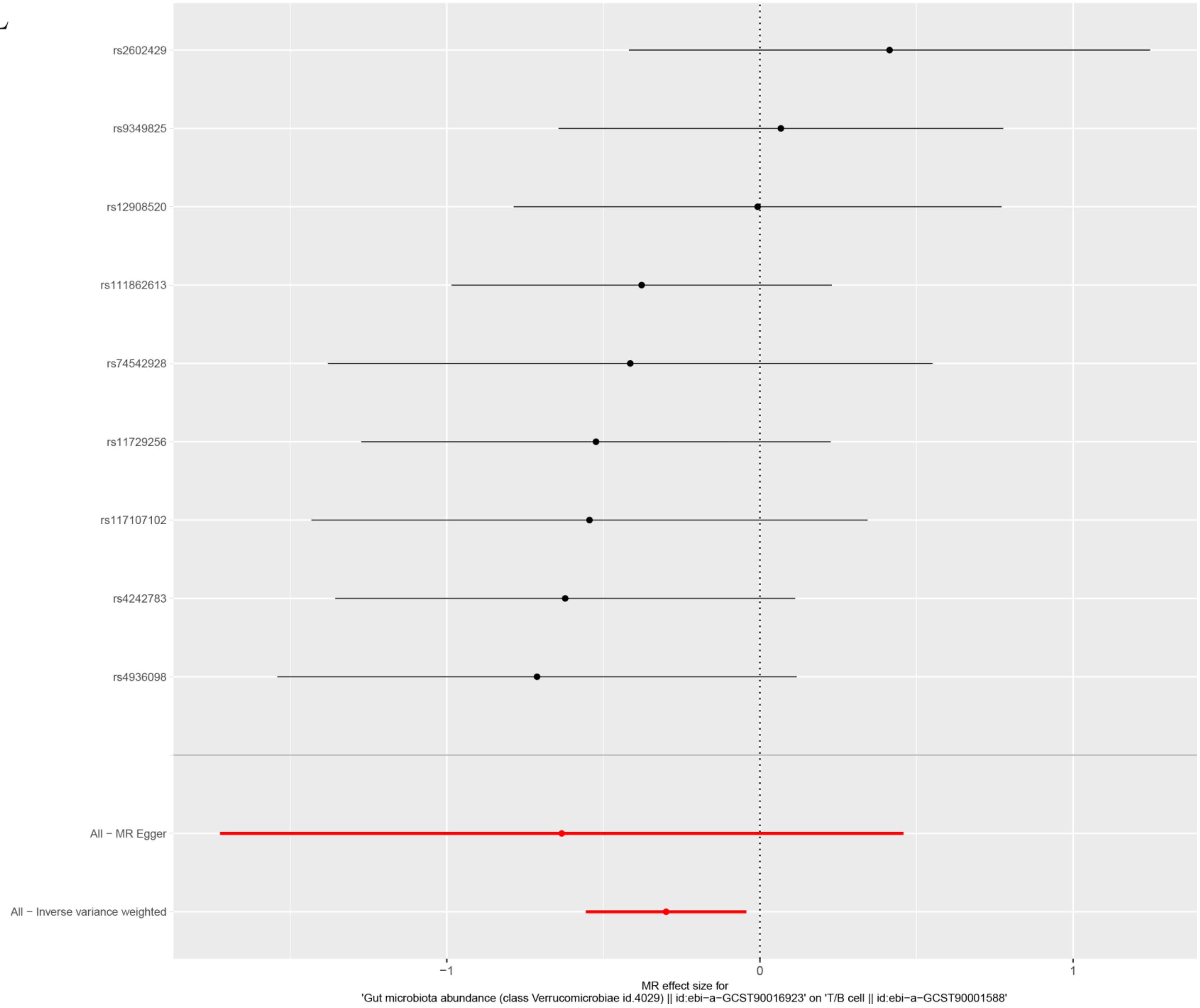
rs2602429
rs9349825
rs12908520
rs111862613
rs74542928
rs11729256
rs117107102
rs4242783
rs4936098
All − MR Egger
All − Inverse variance weighted
−1
0
1
MR effect size for
'Gut microbiota abundance (class Verrucomicrobiae id.4029) || id:ebi−a−GCST90016923' on 'T/B cell || id:ebi−a−GCST90001588'

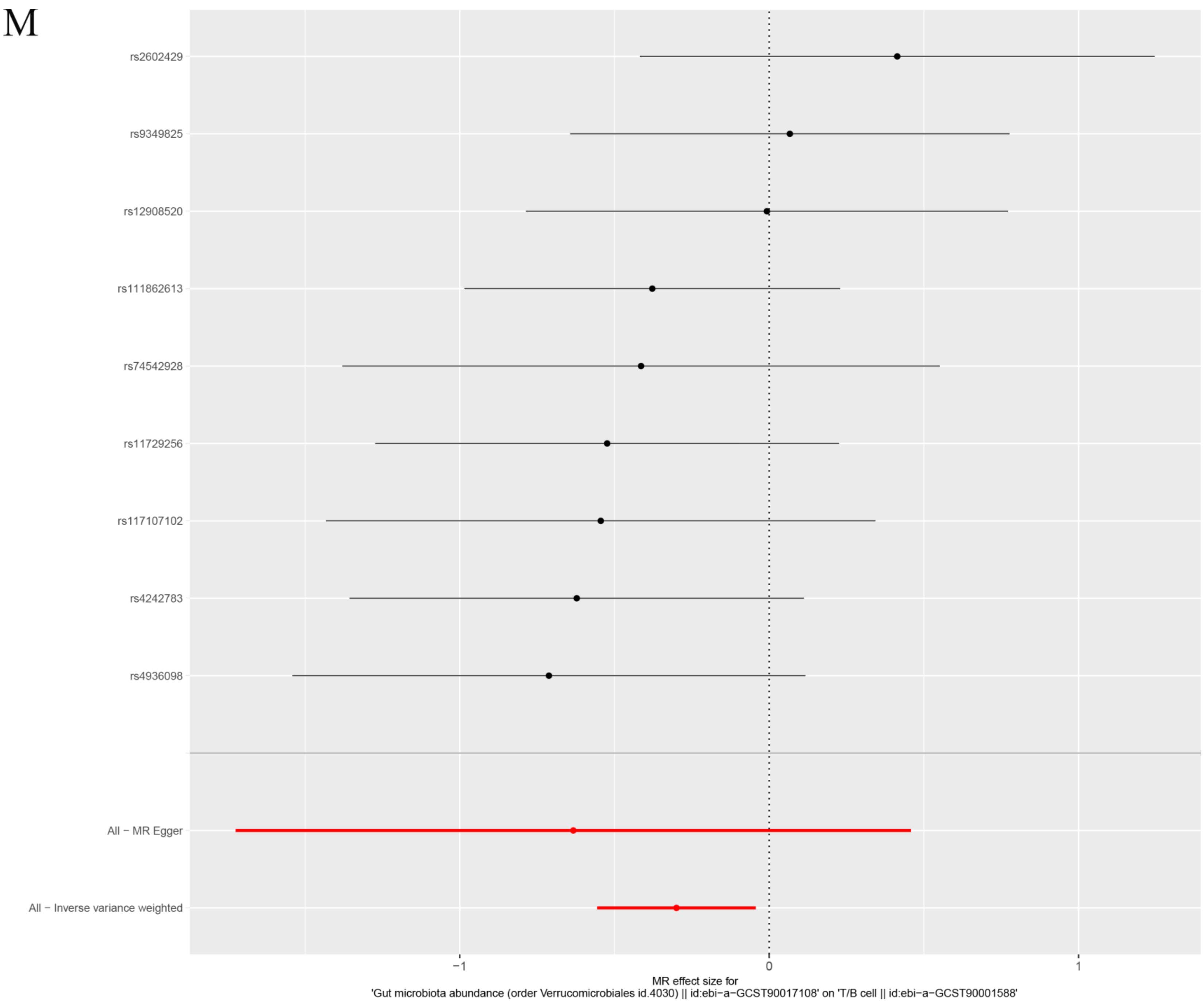
M
rs2602429
rs9349825
rs12908520
rs111862613
rs74542928
rs11729256
rs117107102
rs4242783
rs4936098
All − MR Egger
All − Inverse variance weighted
−1
0
1
MR effect size for
'Gut microbiota abundance (order Verrucomicrobiales id.4030) || id:ebi−a−GCST90017108' on 'T/B cell || id:ebi−a−GCST90001588'

N

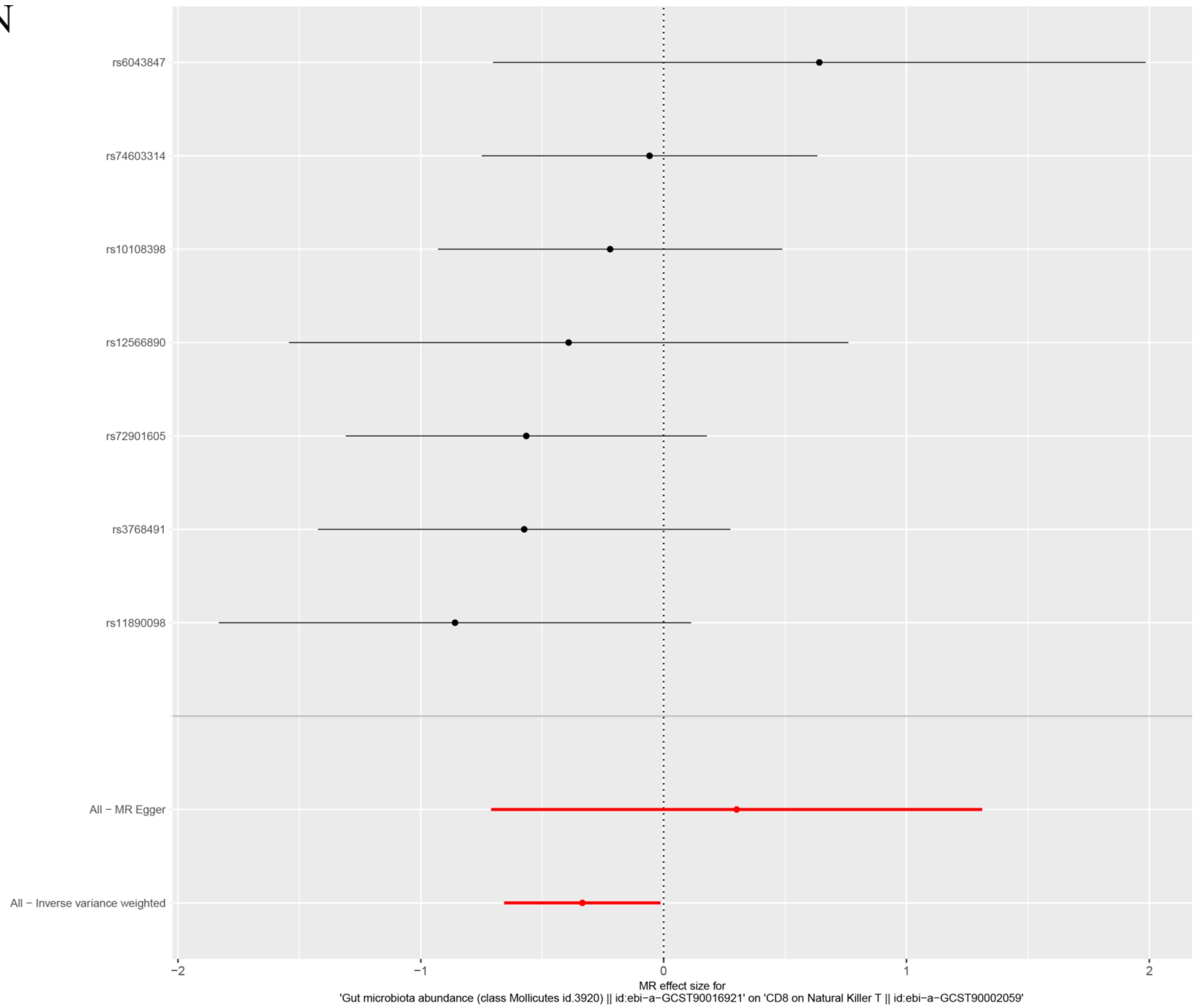

rs6043847
rs74603314
rs10108398
rs12566890
rs72901605
rs3768491
rs11890098
All − MR Egger
All − Inverse variance weighted
−2
−1
0
1
2
MR effect size for
'Gut microbiota abundance (class Mollicutes id.3920) || id:ebi−a−GCST90016921' on 'CD8 on Natural Killer T || id:ebi−a−GCST90002059'

O

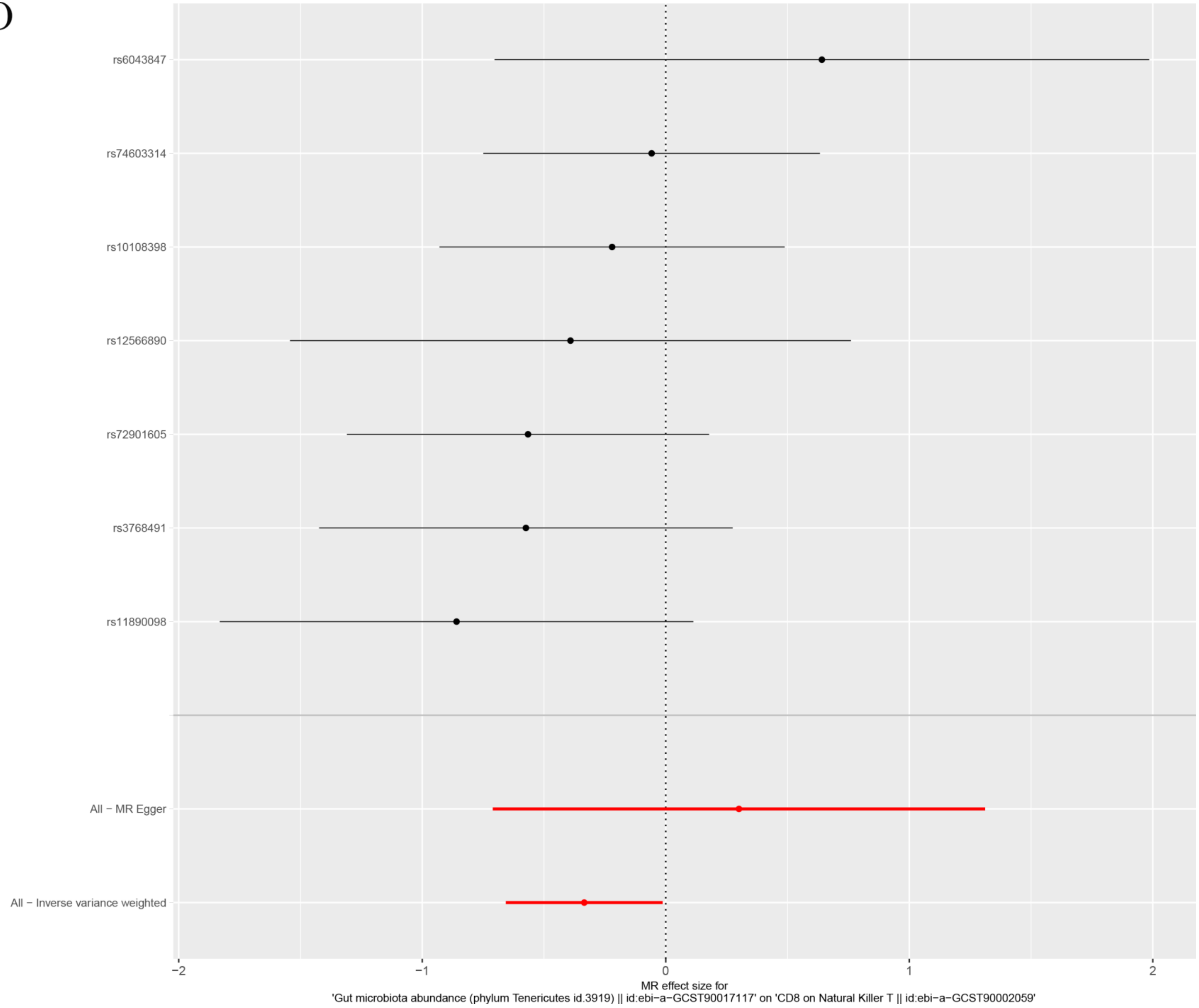

rs6043847
rs74603314
rs10108398
rs12566890
rs72901605
rs3768491
rs11890098
All − MR Egger
All − Inverse variance weighted
−2
−1
0
1
2
MR effect size for
'Gut microbiota abundance (phylum Tenericutes id.3919) || id:ebi−a−GCST90017117' on 'CD8 on Natural Killer T || id:ebi−a−GCST90002059'
