# Supplementary material for: Causal Mediation Role of Immune Cells in Gut Microbiota–Pneumonia Associations: A Mendelian Randomisation Study
Source: J Cell Mol Med. 2025 Sep 11;29(17):e70839. doi: 10.1111/jcmm.70839 (PMC12425809; doi:10.1111/jcmm.70839)
Supplement: Supplementary file 3 — S3: Sensitivity analysis of immune cell characteristics and Pneumonia Mendelian randomization (Scatter plot). [file JCMM-29-e70839-s002.pdf]

Supplementary Figure 3 Sensitivity analysis of immune cell characteristics and Pneumonia Mendelian randomization (Scatter plot)

AA

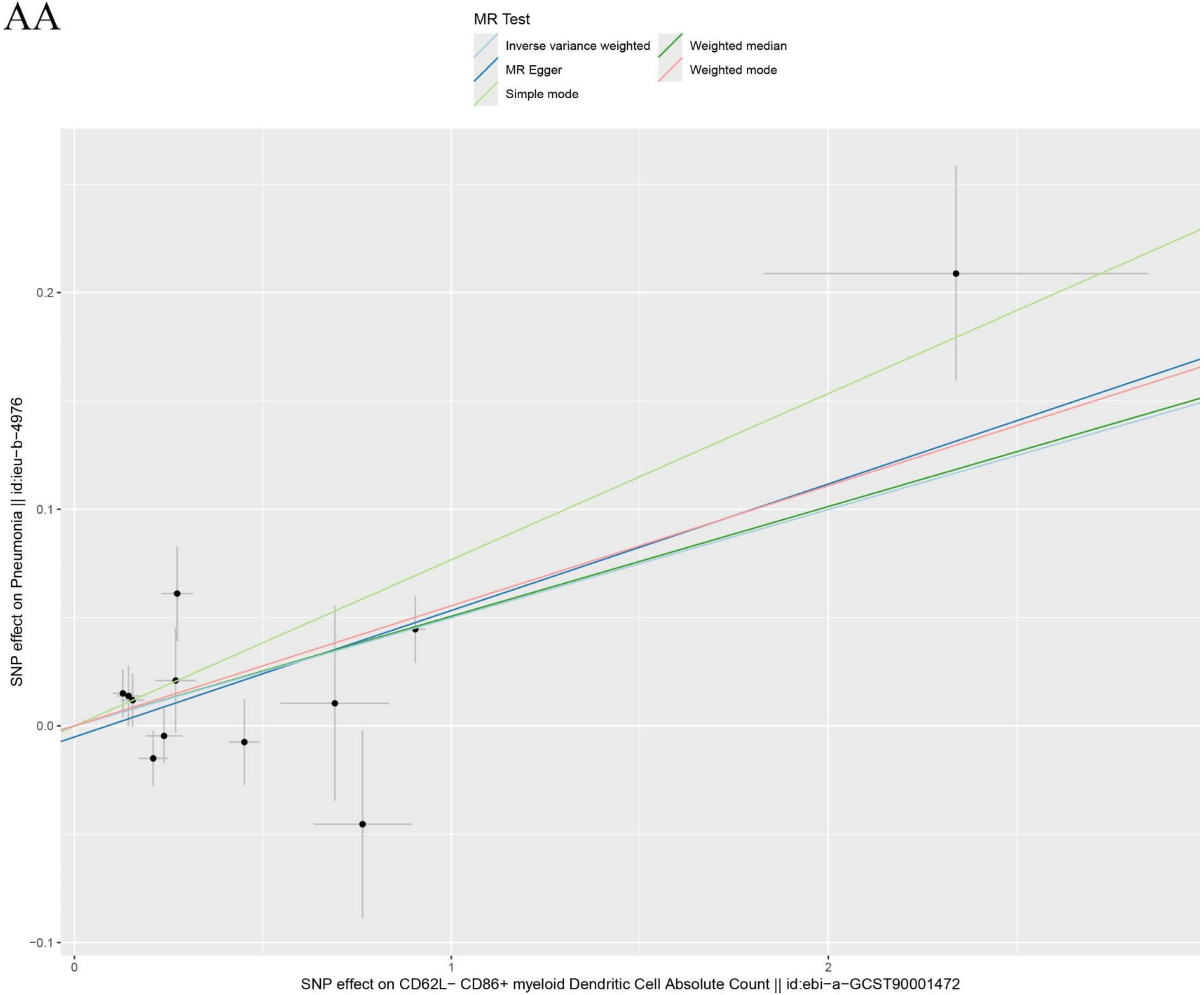

AB

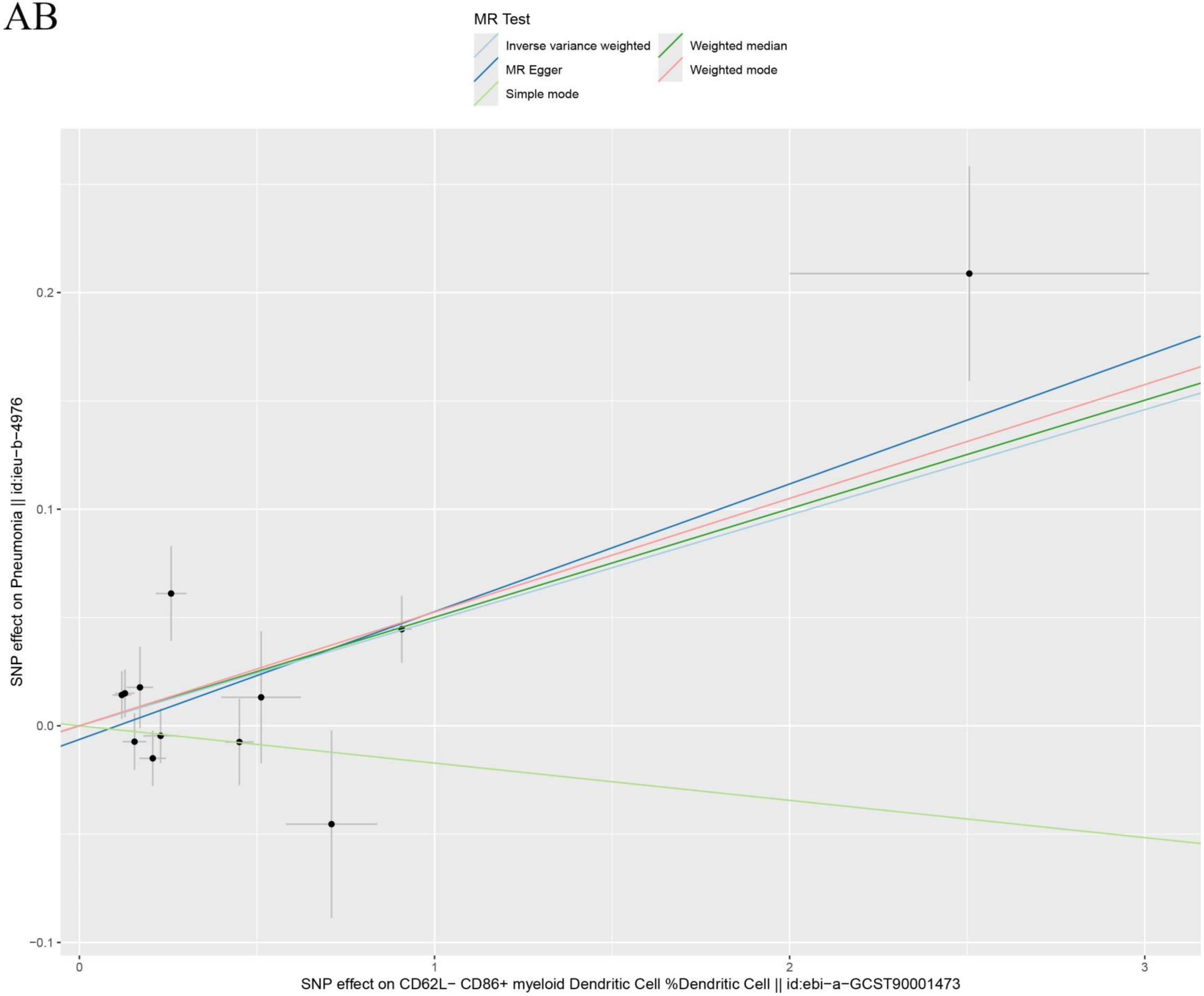
MR Test
Inverse variance weighted
MR Egger
Simple mode
Weighted median
Weighted mode
0.2
0.1
0.0
−0.1
0
1
2
3
SNP effect on Pneumonia || id:ieu-b-4976
SNP effect on CD62L− CD86+ myeloid Dendritic Cell %Dendritic Cell || id:ebi-a-GCST90001473

AC

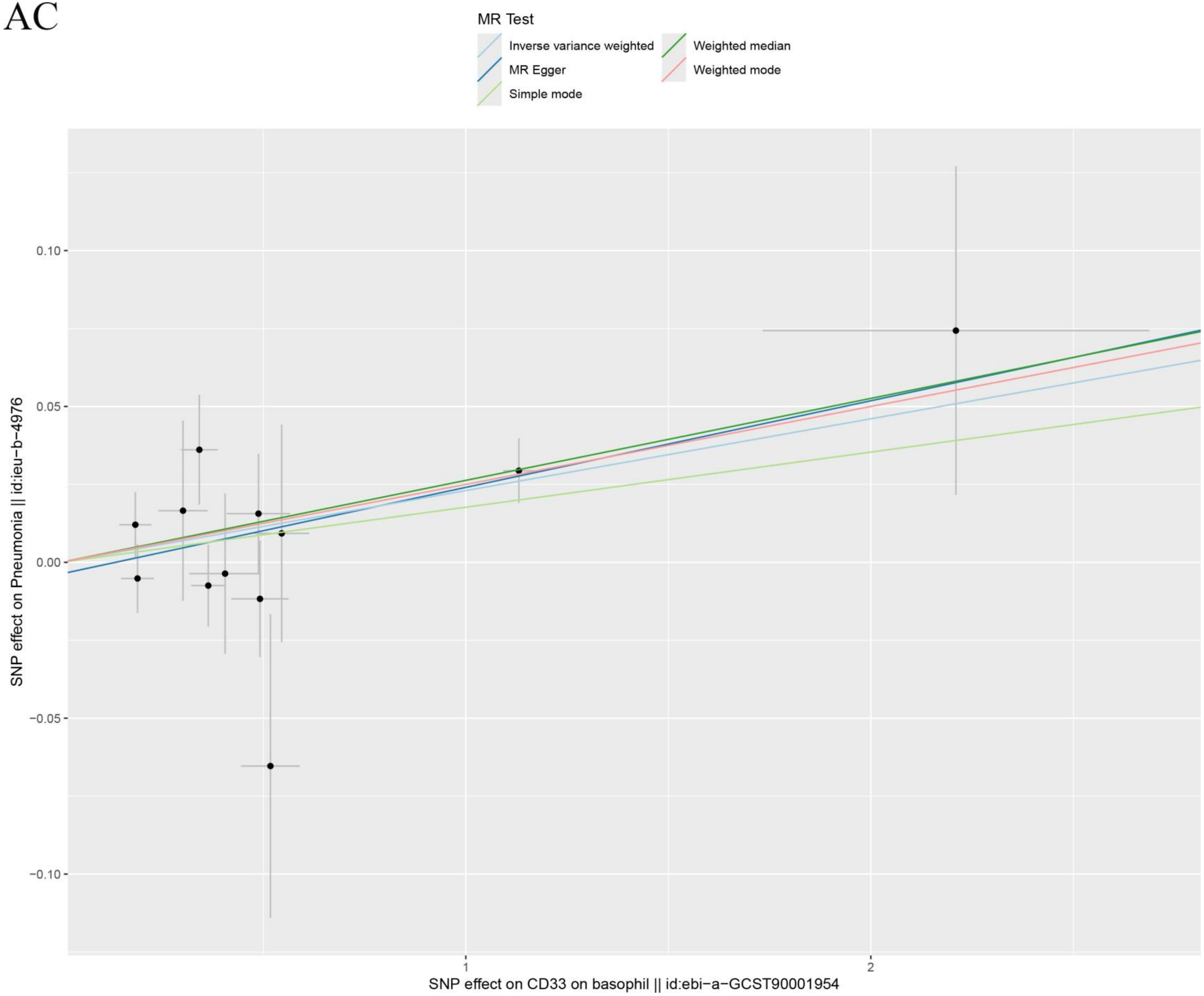
MR Test
Inverse variance weighted
MR Egger
Simple mode
Weighted median
Weighted mode
0.10
0.05
0.00
−0.05
−0.10
1
2
SNP effect on Pneumonia || id:ieu-b-4976
SNP effect on CD33 on basophil || id:ebi-a-GCST90001954

AD

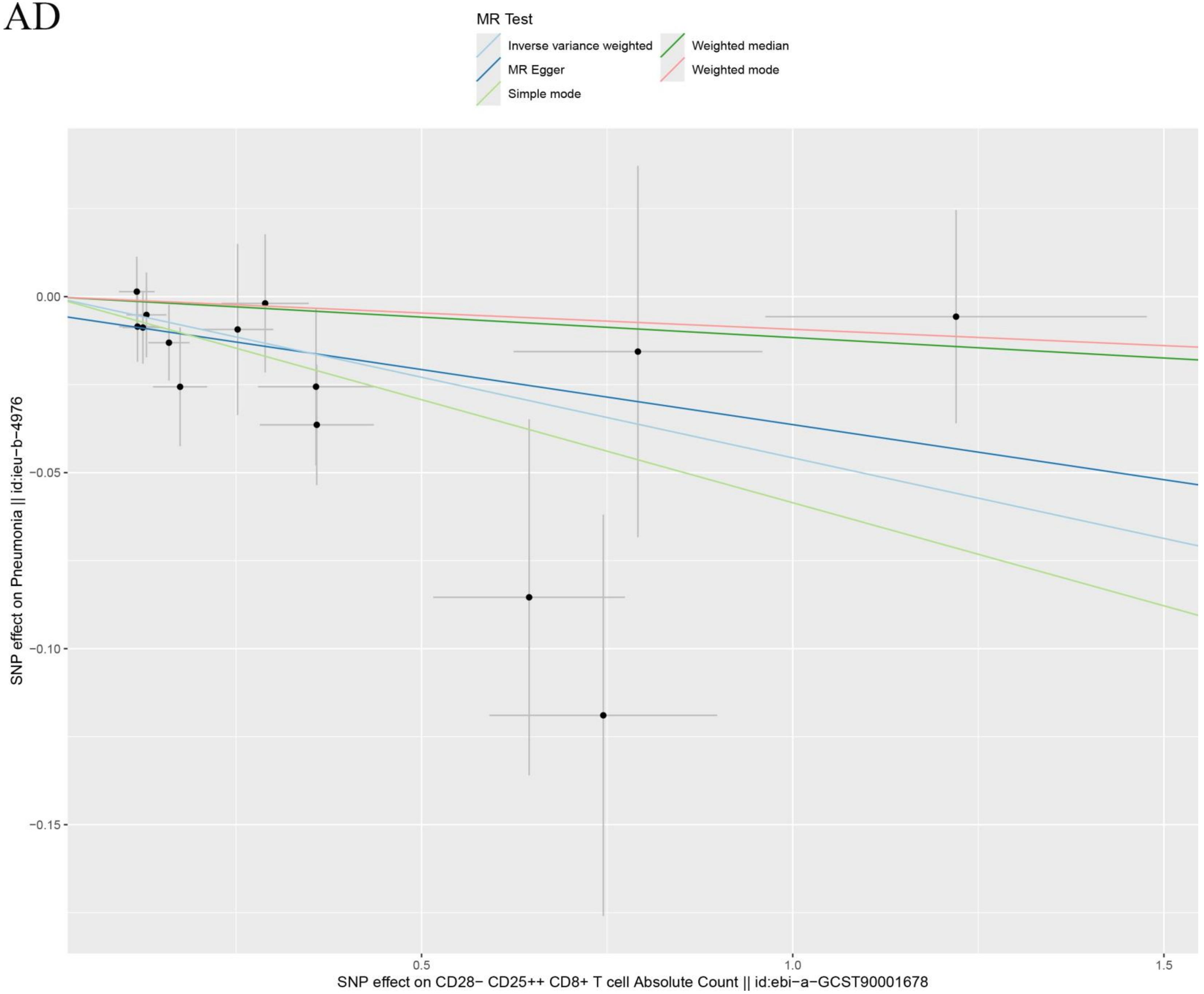

MR Test
Inverse variance weighted
MR Egger
Simple mode
Weighted median
Weighted mode
0.00
−0.05
−0.10
−0.15
SNP effect on Pneumonia || id:ieu-b-4976
0.5
1.0
1.5
SNP effect on CD28− CD25++ CD8+ T cell Absolute Count || id:ebi−a−GCST90001678

AE

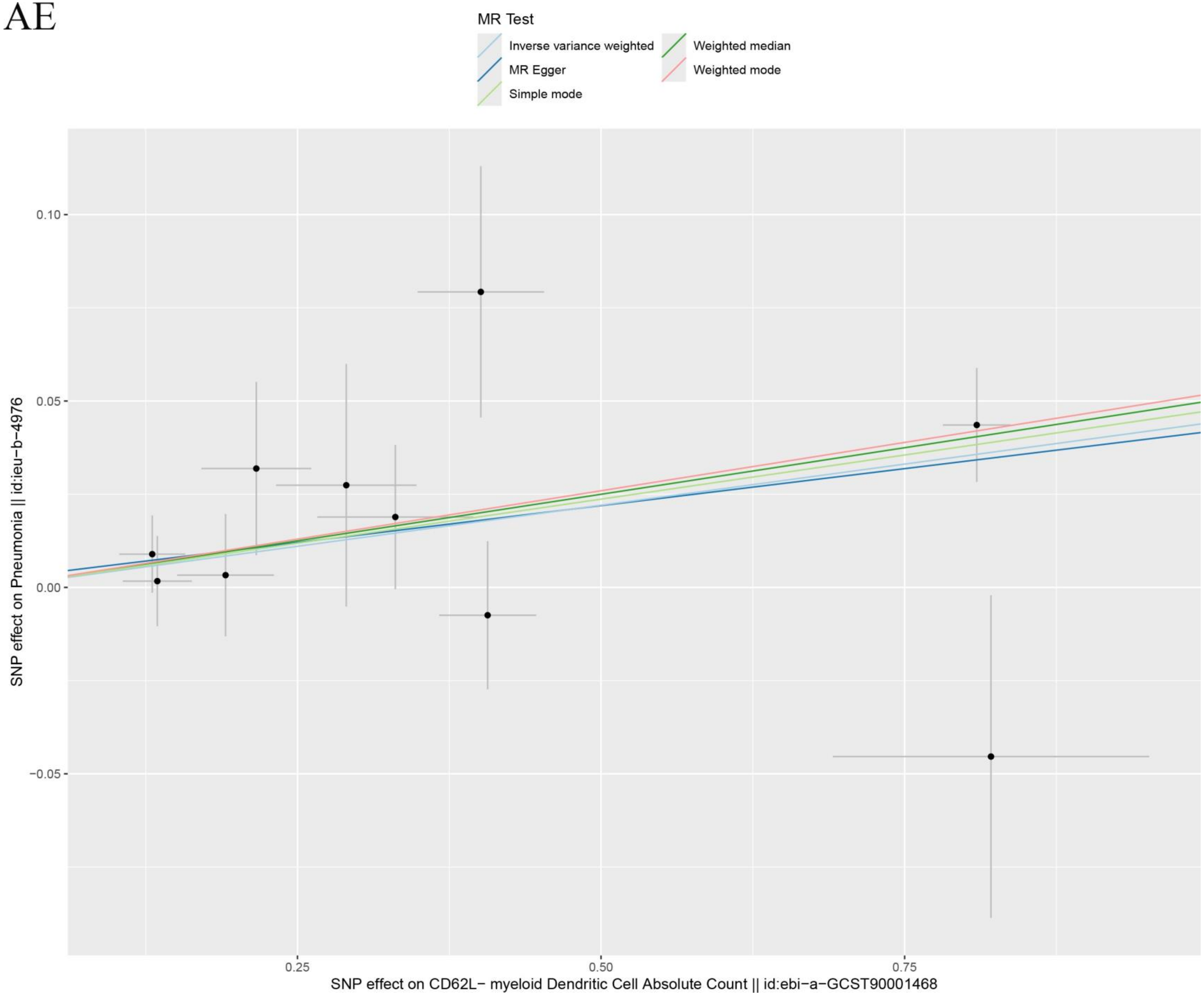
MR Test
Inverse variance weighted
MR Egger
Simple mode
Weighted median
Weighted mode
0.10
0.05
0.00
−0.05
SNP effect on Pneumonia || id:ieu-b-4976
0.25
0.50
0.75
SNP effect on CD62L− myeloid Dendritic Cell Absolute Count || id:ebi-a-GCST90001468

AF

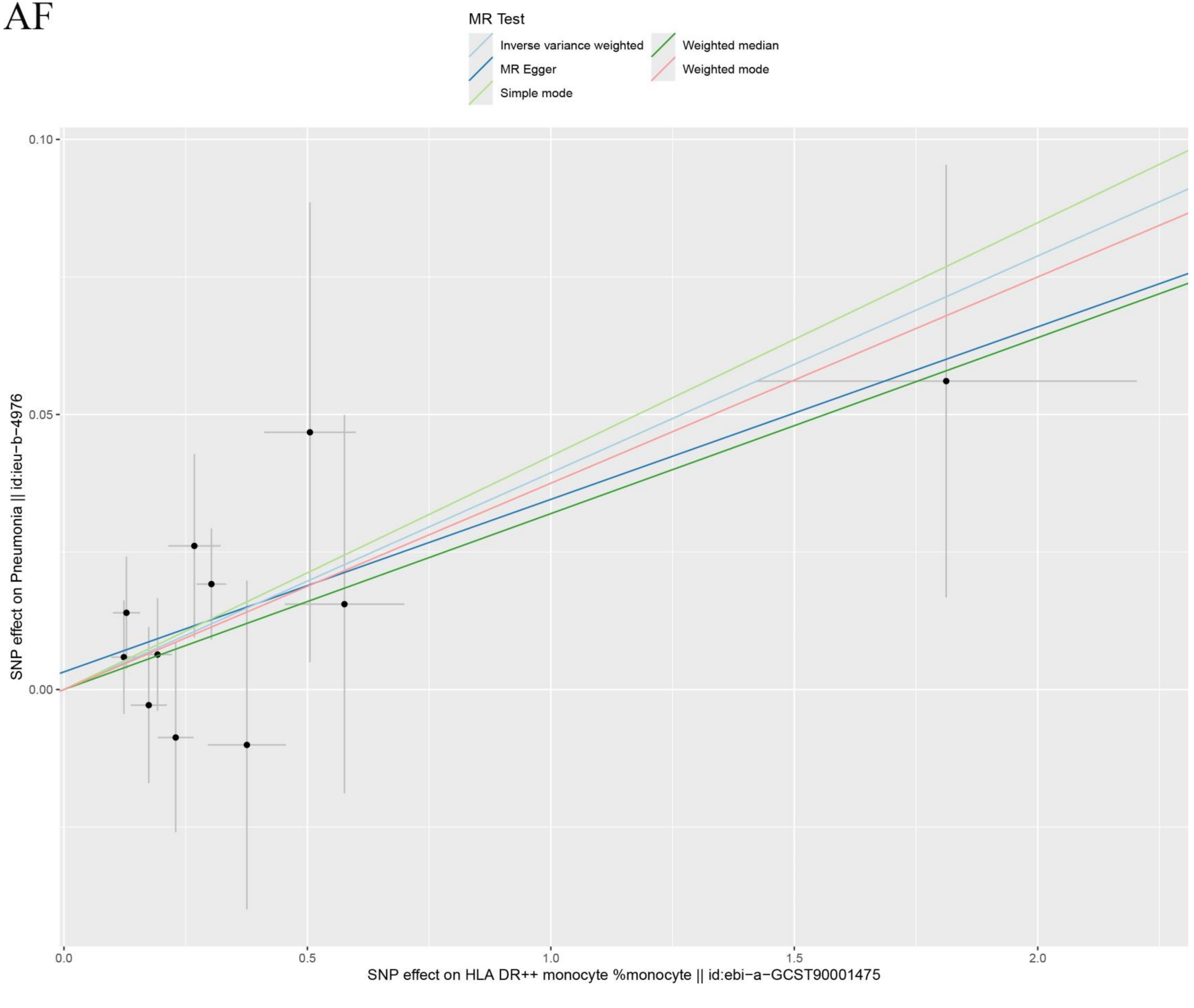
MR Test
Inverse variance weighted
MR Egger
Simple mode
Weighted median
Weighted mode
0.10
0.05
0.00
SNP effect on Pneumonia || id:ieu-b-4976
0.0
0.5
1.0
1.5
2.0
SNP effect on HLA DR++ monocyte %monocyte || id:ebi-a-GCST90001475

AG

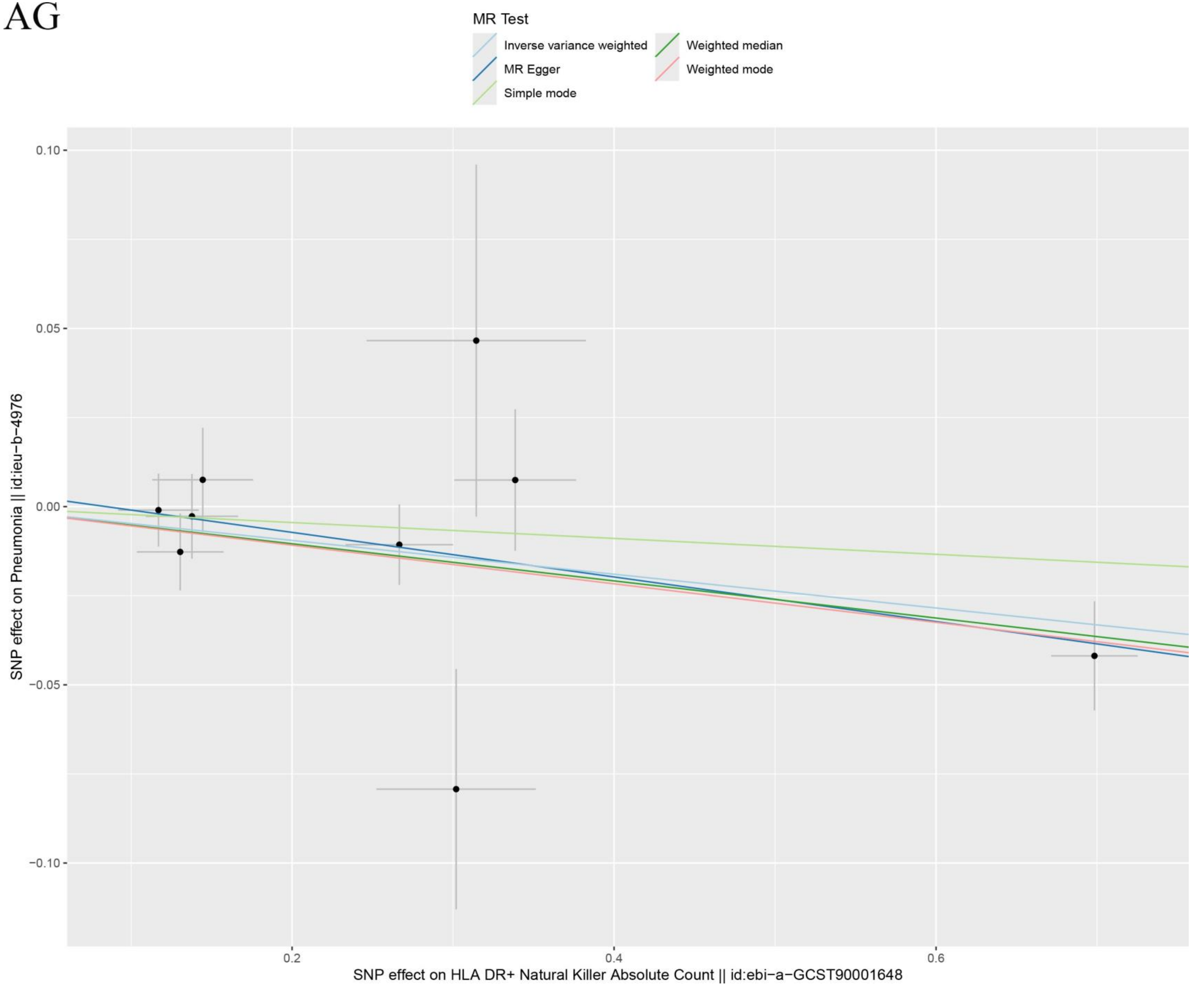

MR Test
Inverse variance weighted
MR Egger
Simple mode
Weighted median
Weighted mode
0.10
0.05
0.00
−0.05
−0.10
0.2
0.4
0.6
SNP effect on Pneumonia || id:ieu−b−4976
SNP effect on HLA DR+ Natural Killer Absolute Count || id:ebi−a−GCST90001648

AH

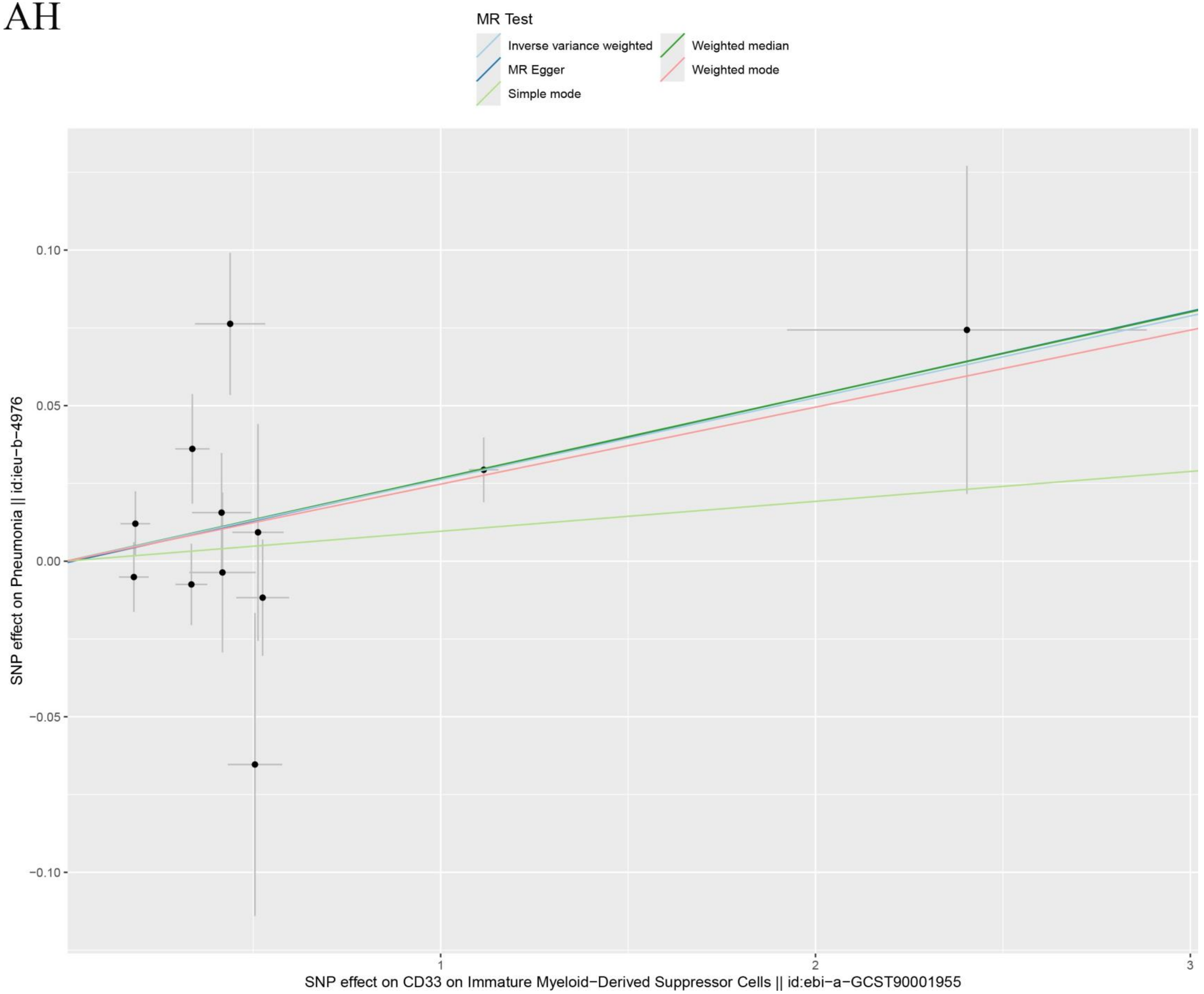

MR Test
Inverse variance weighted
MR Egger
Simple mode
Weighted median
Weighted mode
0.10
0.05
0.00
−0.05
−0.10
1
2
3
SNP effect on Pneumonia || id:ieu-b-4976
SNP effect on CD33 on Immature Myeloid−Derived Suppressor Cells || id:ebi-a-GCST90001955

AI

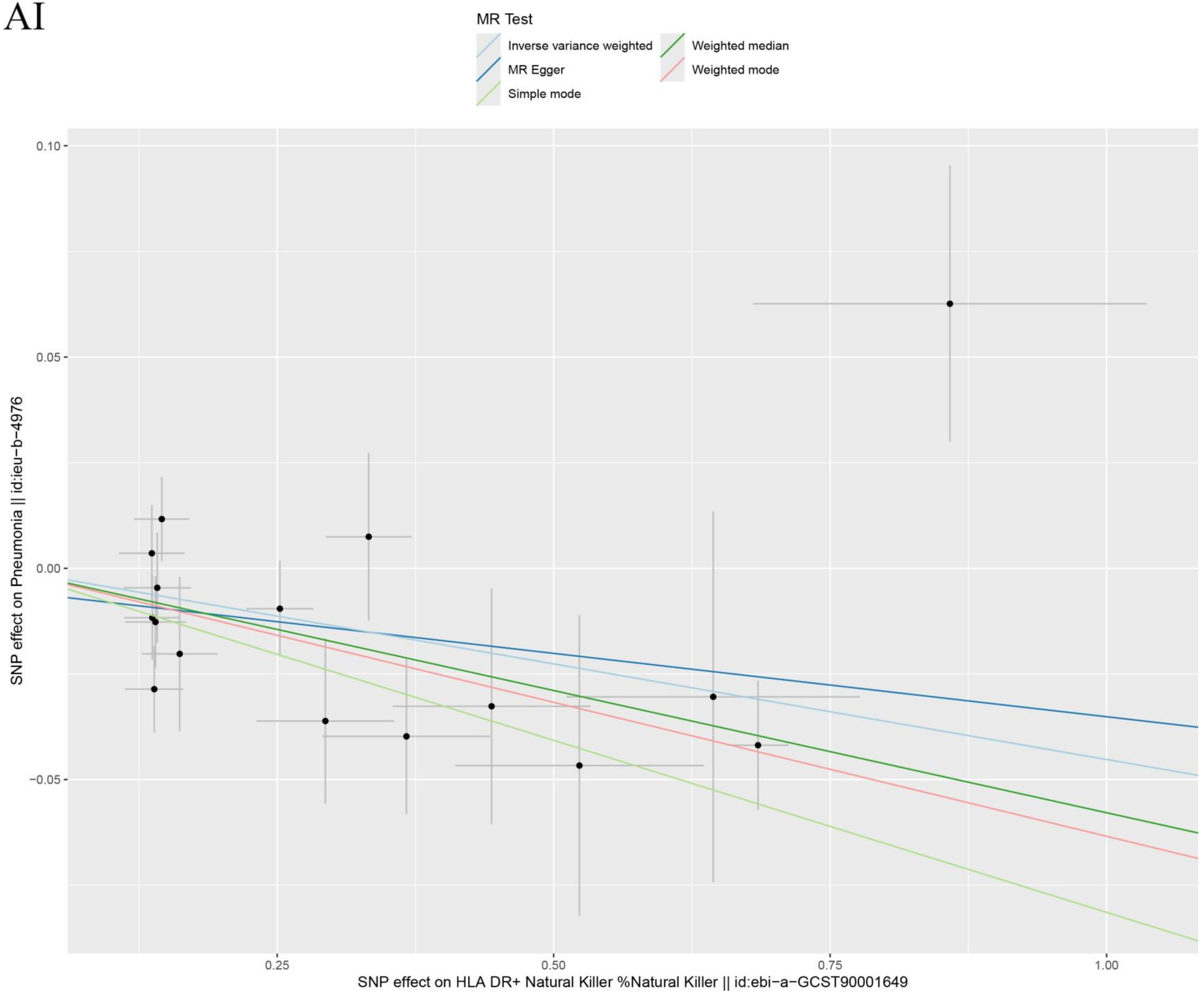
MR Test
Inverse variance weighted
MR Egger
Simple mode
Weighted median
Weighted mode
0.10
0.05
0.00
−0.05
0.25
0.50
0.75
1.00
SNP effect on Pneumonia || id:ieu−b−4976
SNP effect on HLA DR+ Natural Killer %Natural Killer || id:ebi−a−GCST90001649

AJ

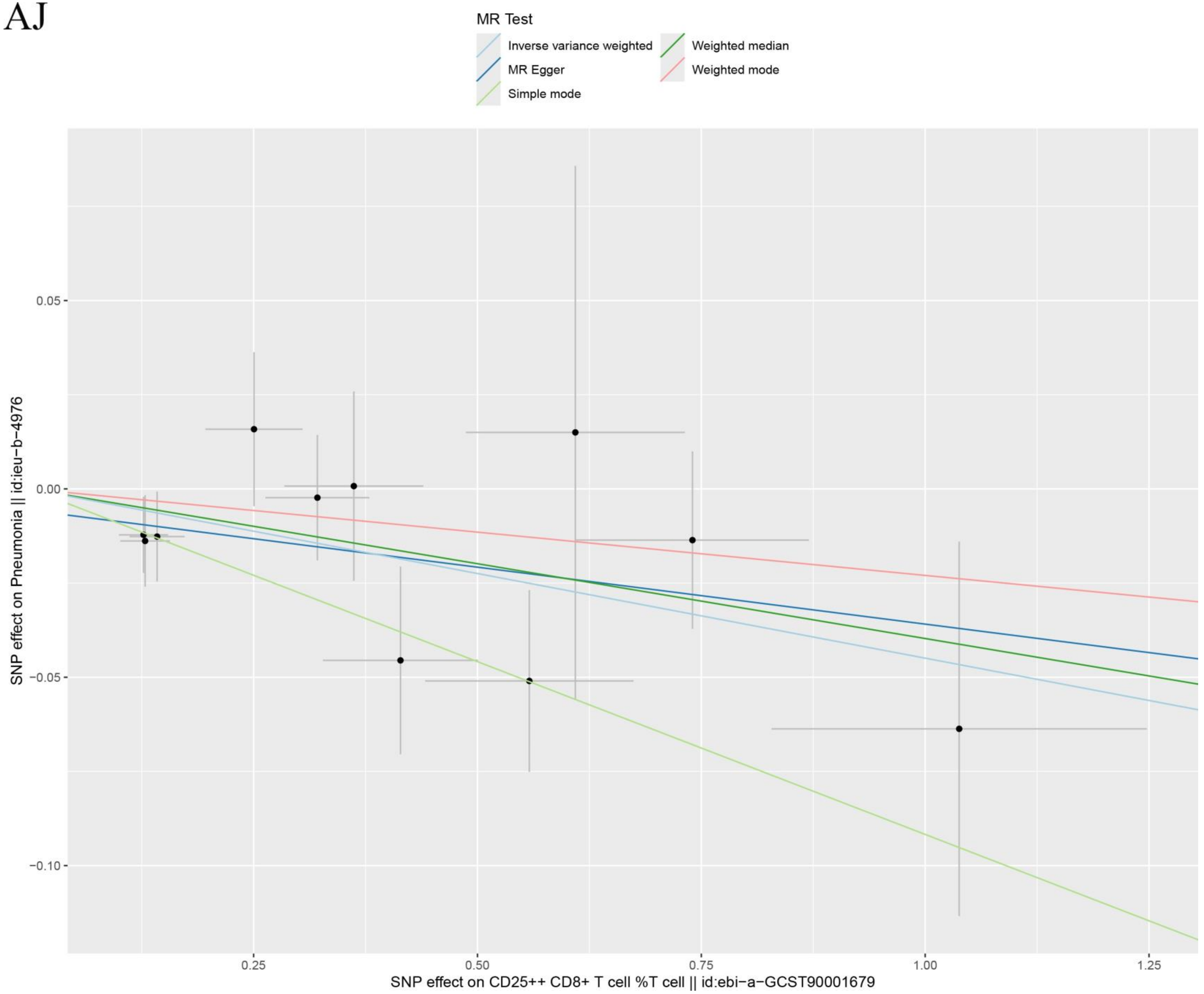
MR Test
Inverse variance weighted
MR Egger
Simple mode
Weighted median
Weighted mode
0.05
0.00
−0.05
−0.10
SNP effect on Pneumonia || id:ieu-b-4976
0.25
0.50
0.75
1.00
1.25
SNP effect on CD25++ CD8+ T cell %T cell || id:ebi-a-GCST90001679

AK

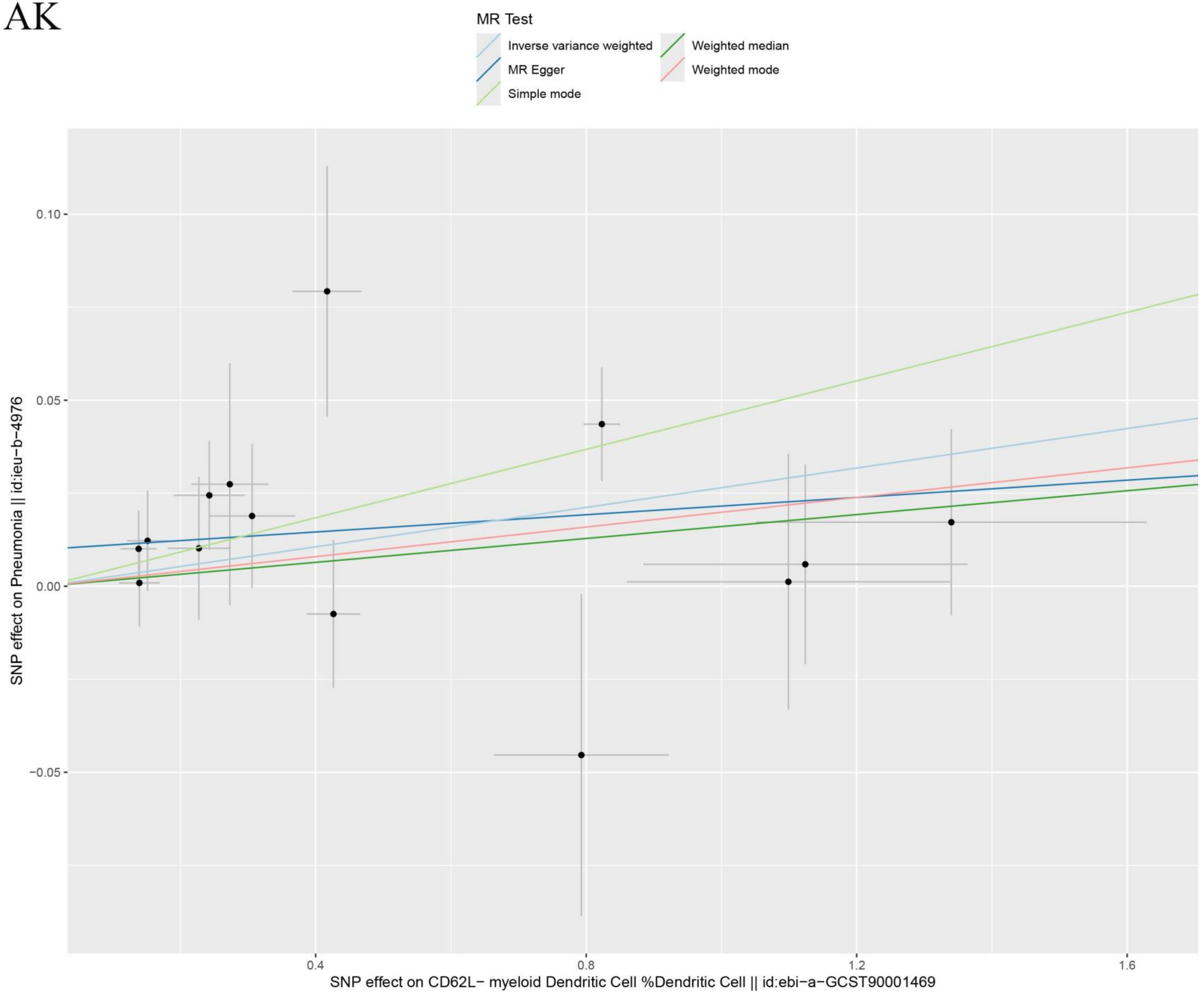

MR Test
Inverse variance weighted
MR Egger
Simple mode
Weighted median
Weighted mode
0.10
0.05
0.00
−0.05
SNP effect on Pneumonia || id:ieu-b-4976
0.4
0.8
1.2
1.6
SNP effect on CD62L− myeloid Dendritic Cell %Dendritic Cell || id:ebi-a-GCST90001469

AL

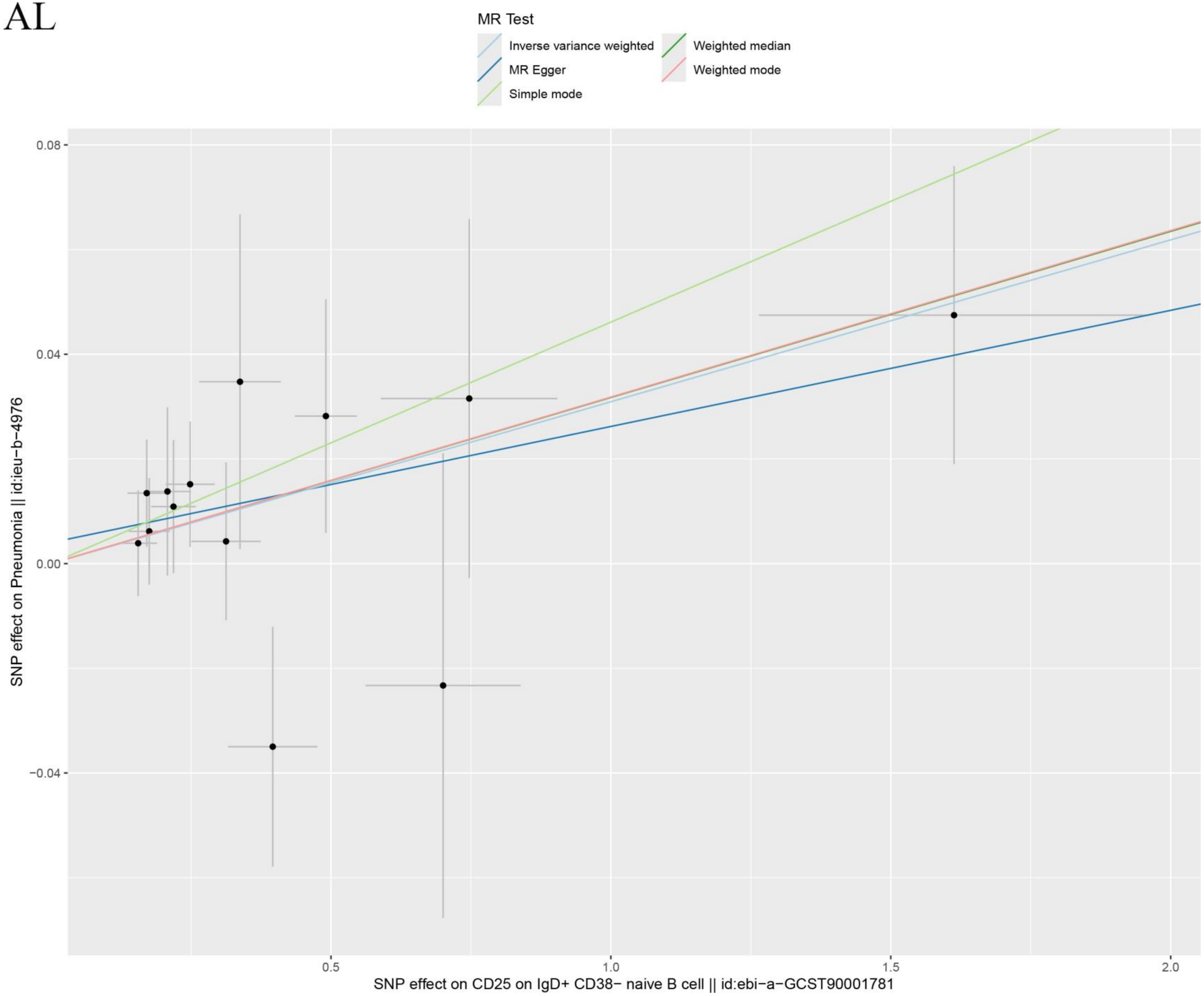

MR Test
Inverse variance weighted
MR Egger
Simple mode
Weighted median
Weighted mode
0.08
0.04
0.00
−0.04
0.5
1.0
1.5
2.0
SNP effect on Pneumonia || id:ieu−b−4976
SNP effect on CD25 on IgD+ CD38− naive B cell || id:ebi−a−GCST90001781

AM

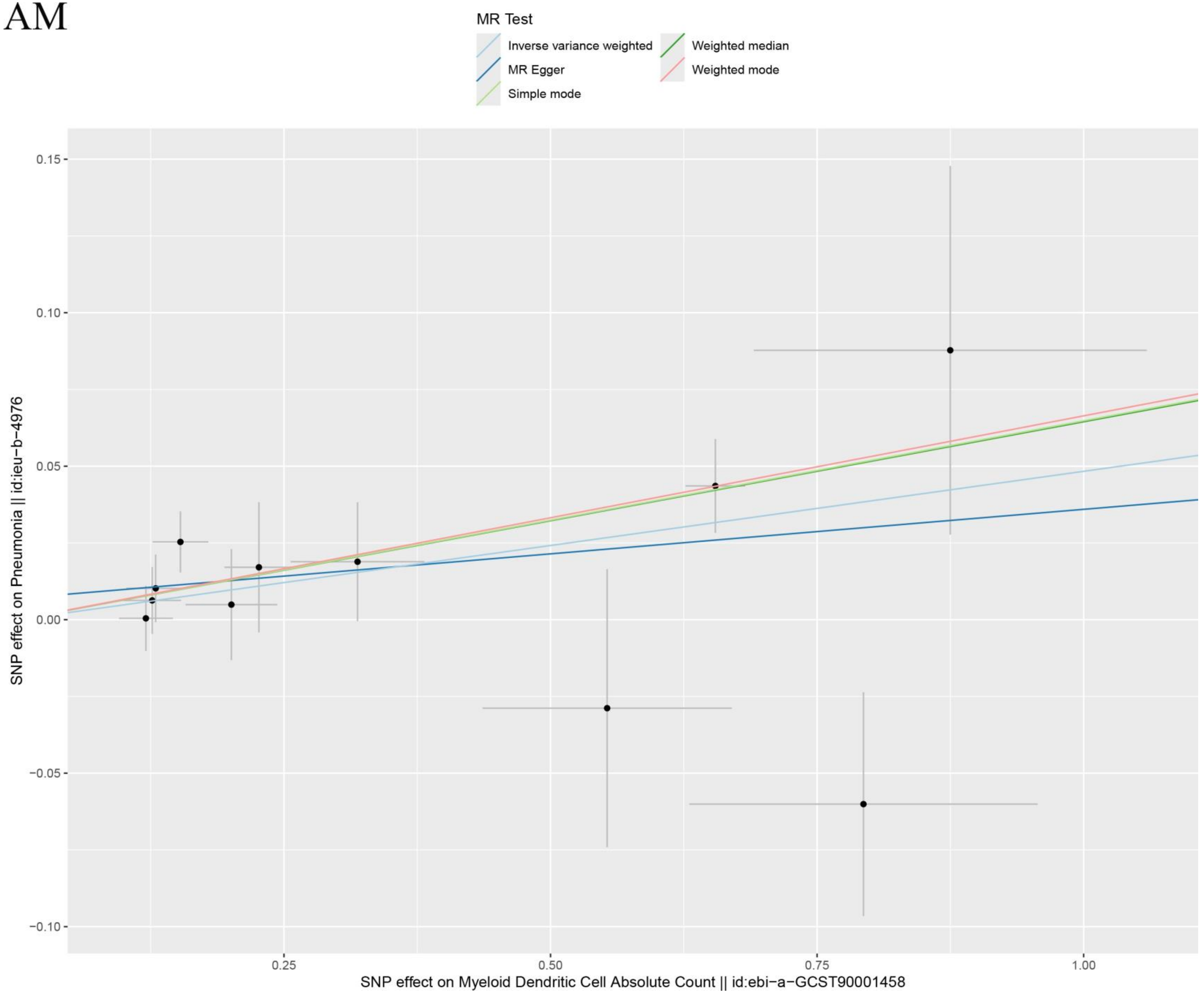

MR Test
Inverse variance weighted
MR Egger
Simple mode
Weighted median
Weighted mode
0.15
0.10
0.05
0.00
−0.05
−0.10
SNP effect on Pneumonia || id:ieu-b-4976
0.25
0.50
0.75
1.00
SNP effect on Myeloid Dendritic Cell Absolute Count || id:ebi−a−GCST90001458

AN

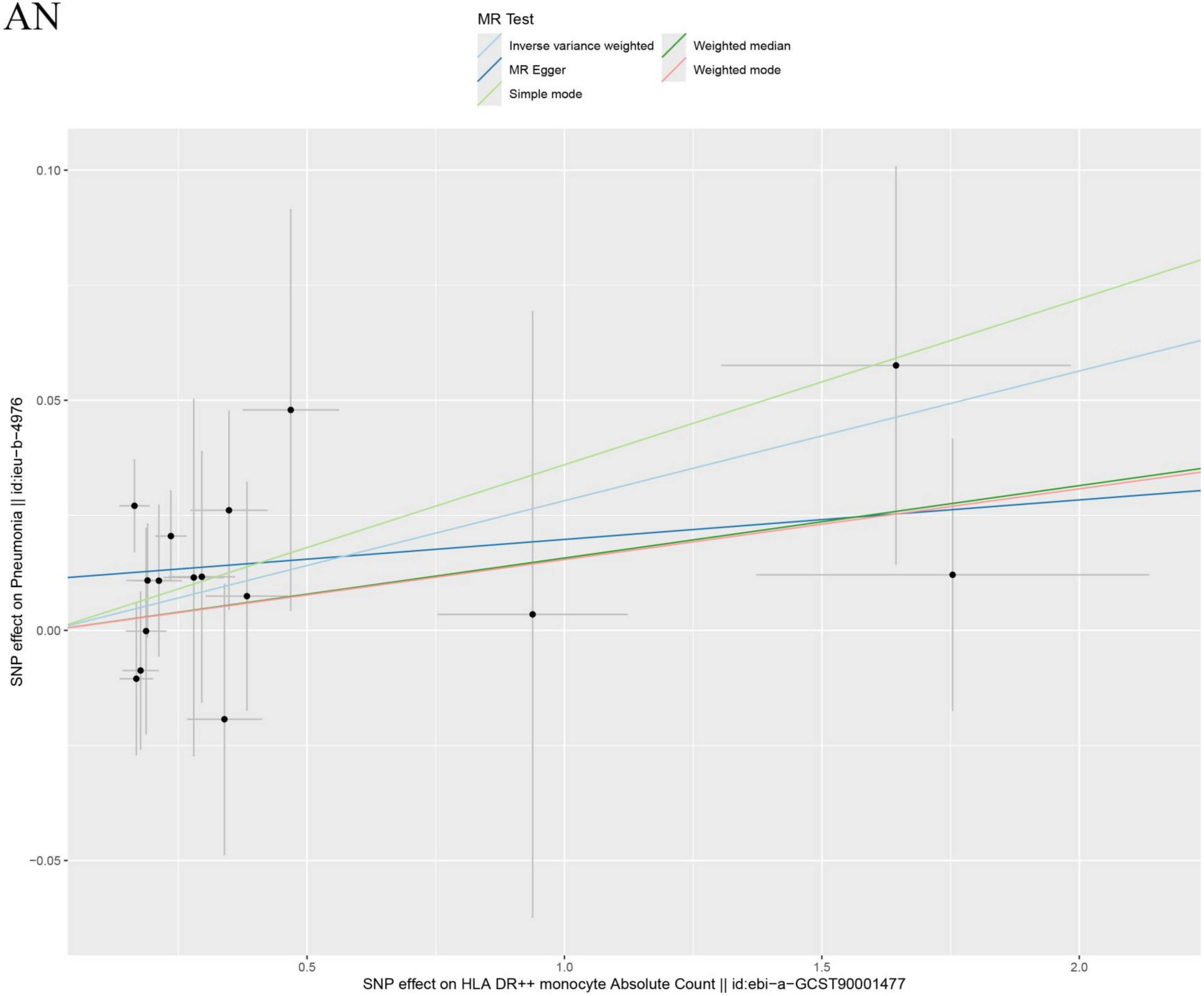
MR Test
Inverse variance weighted
MR Egger
Simple mode
Weighted median
Weighted mode
0.10
0.05
0.00
−0.05
SNP effect on Pneumonia || id:ieu-b-4976
0.5
1.0
1.5
2.0
SNP effect on HLA DR++ monocyte Absolute Count || id:ebi-a-GCST90001477

AO

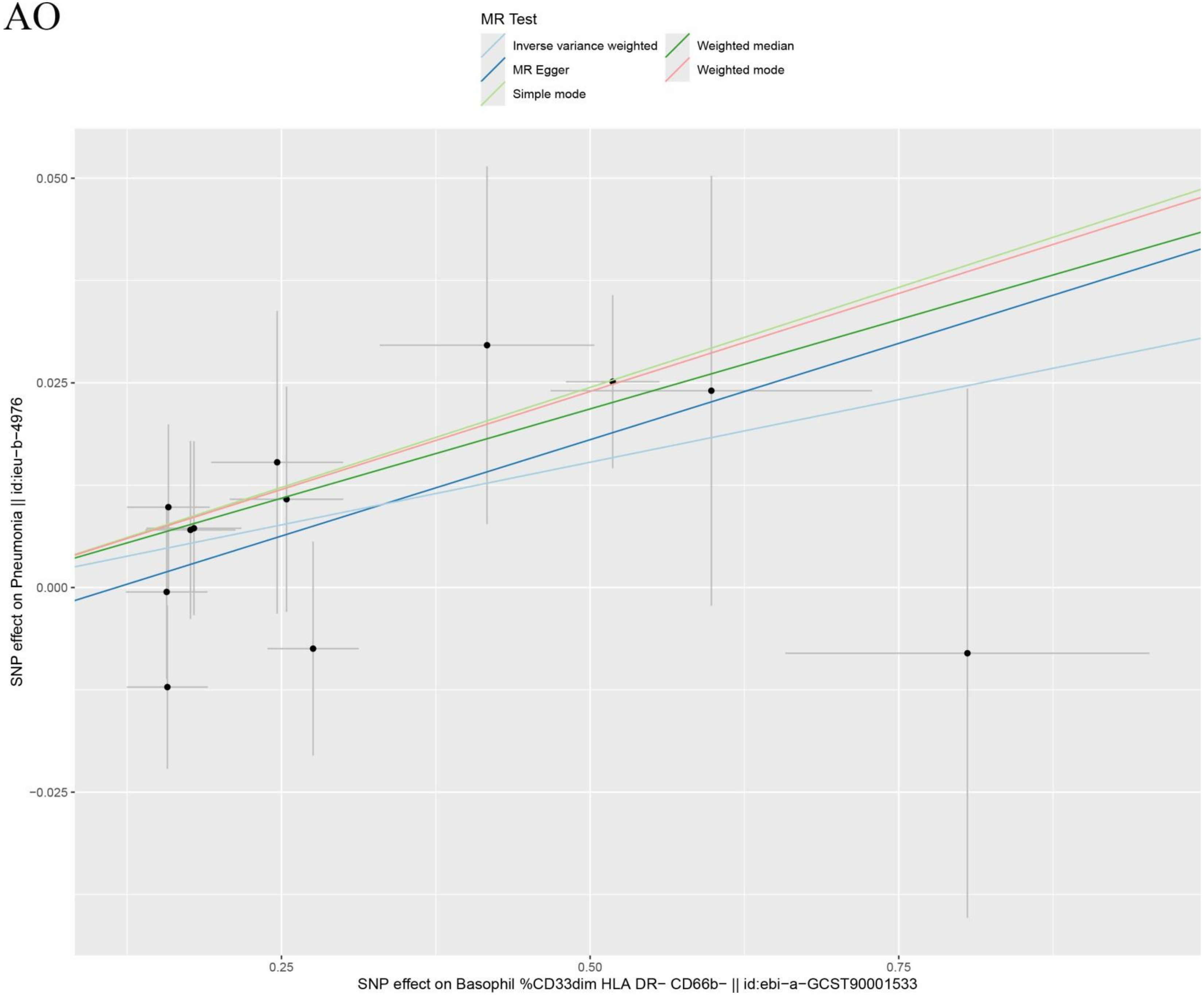
MR Test
Inverse variance weighted
MR Egger
Simple mode
Weighted median
Weighted mode
0.050
0.025
0.000
−0.025
0.25
0.50
0.75
SNP effect on Pneumonia || id:ieu-b-4976
SNP effect on Basophil %CD33dim HLA DR− CD66b− || id:ebi-a-GCST90001533

AP

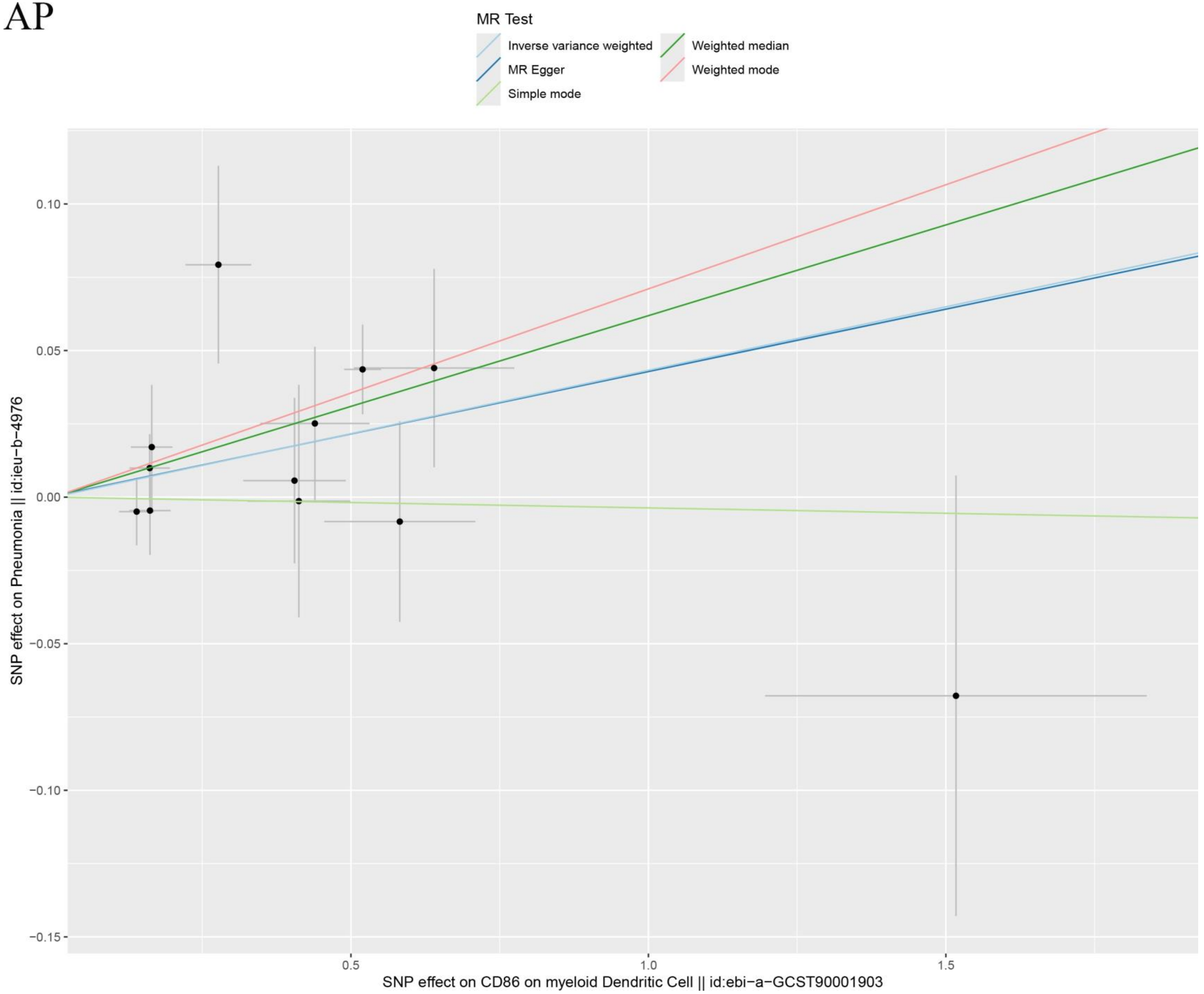
MR Test
Inverse variance weighted
MR Egger
Simple mode
Weighted median
Weighted mode
0.10
0.05
0.00
−0.05
−0.10
−0.15
0.5
1.0
1.5
SNP effect on Pneumonia || id:ieu-b-4976
SNP effect on CD86 on myeloid Dendritic Cell || id:ebi-a-GCST90001903

AQ

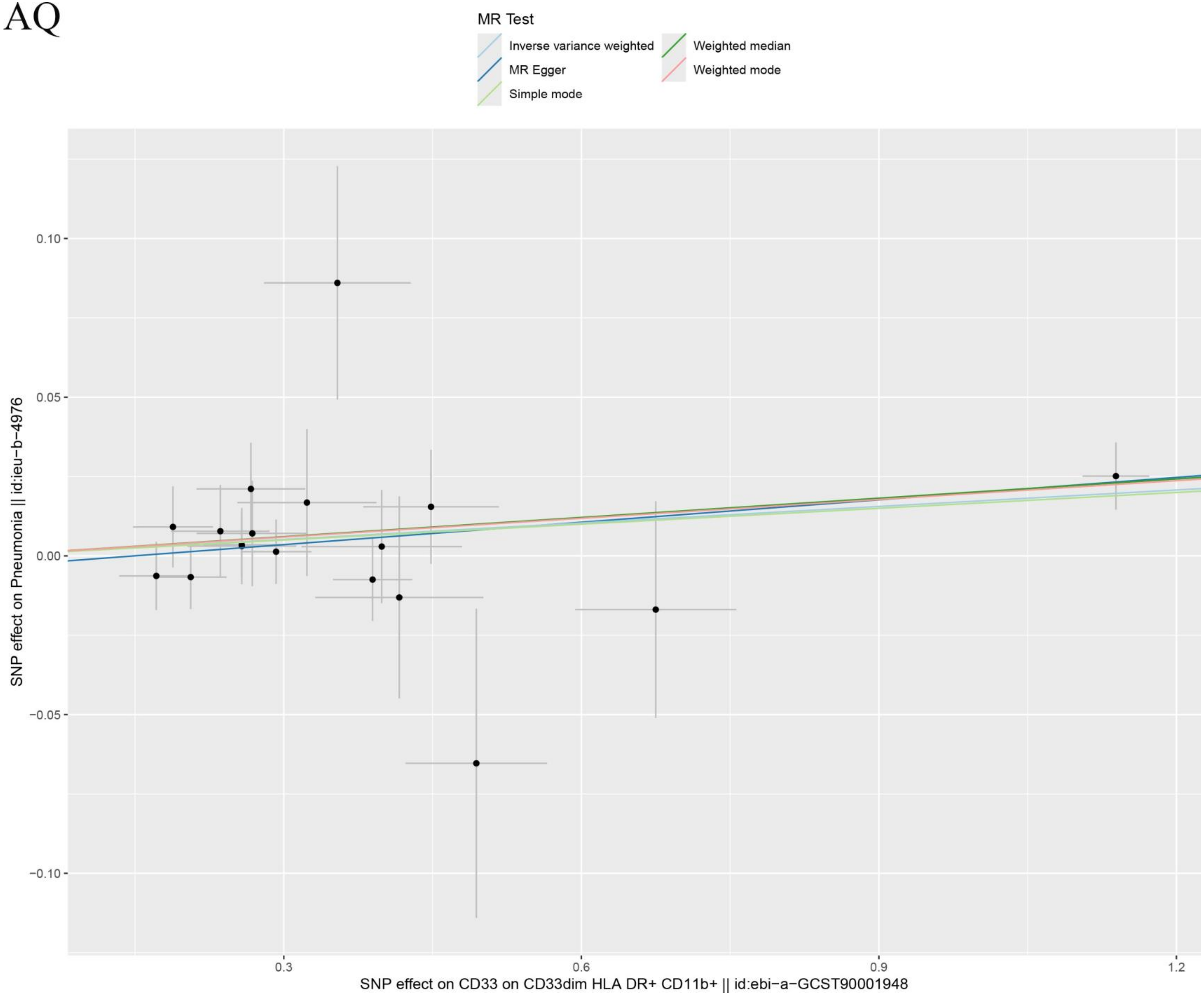

MR Test
Inverse variance weighted
MR Egger
Simple mode
Weighted median
Weighted mode
0.10
0.05
0.00
−0.05
−0.10
0.3
0.6
0.9
1.2
SNP effect on Pneumonia || id:ieu−b−4976
SNP effect on CD33 on CD33dim HLA DR+ CD11b+ || id:ebi−a−GCST90001948

AR

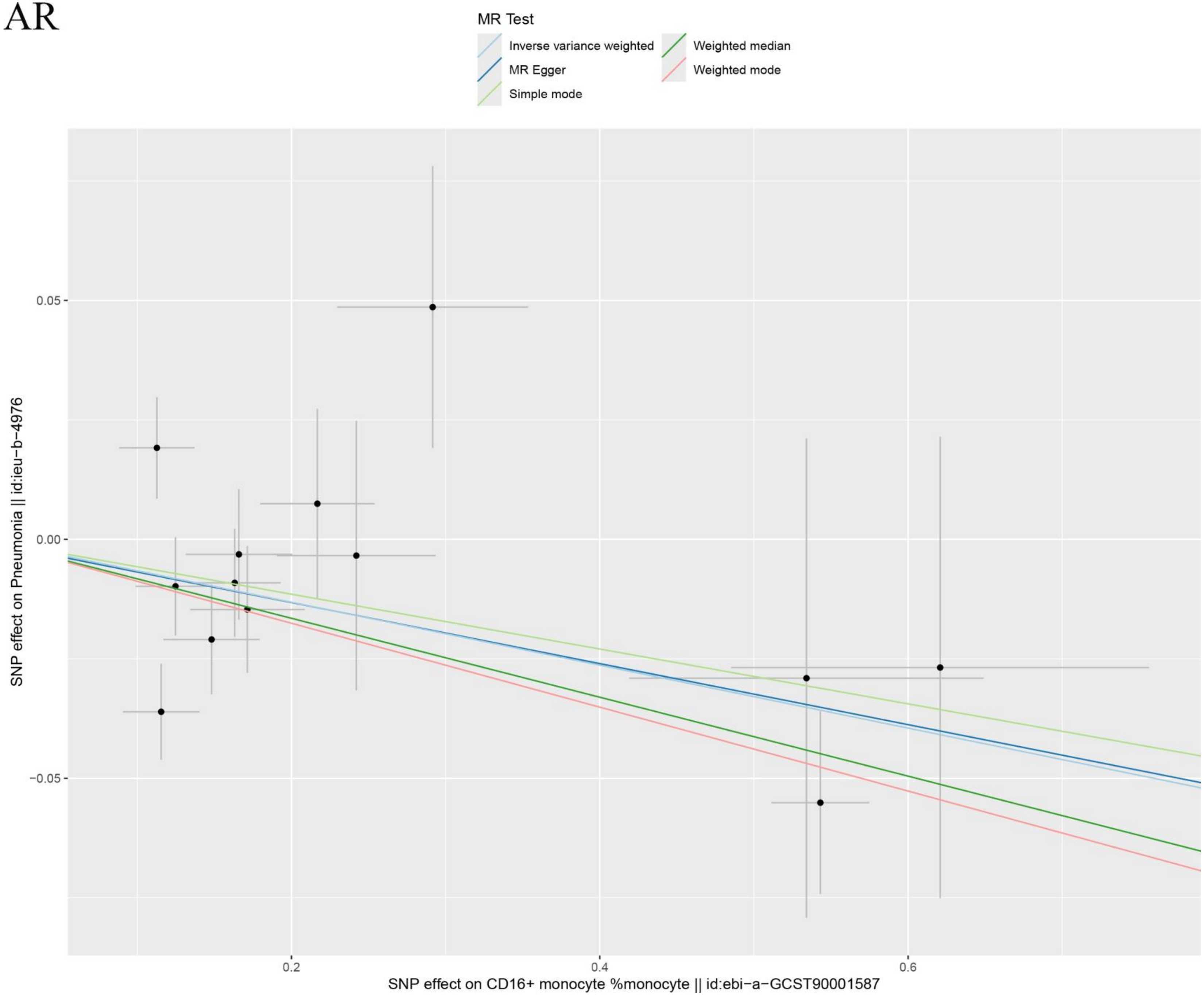

MR Test
Inverse variance weighted
MR Egger
Simple mode
Weighted median
Weighted mode
0.05
0.00
−0.05
SNP effect on Pneumonia || id:ieu-b-4976
0.2
0.4
0.6
SNP effect on CD16+ monocyte %monocyte || id:ebi-a-GCST90001587

AS

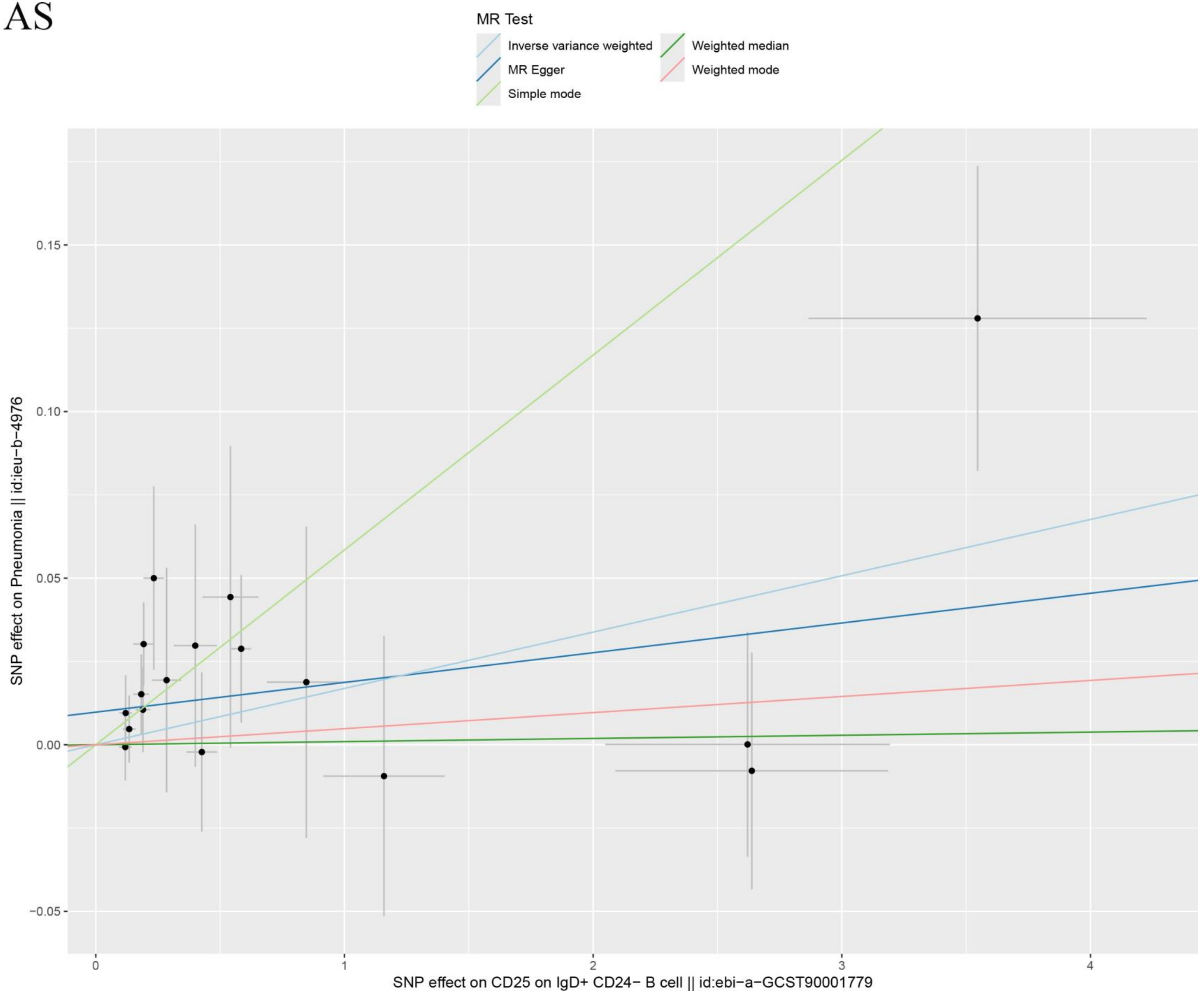
MR Test
Inverse variance weighted
MR Egger
Simple mode
Weighted median
Weighted mode
0.15
0.10
0.05
0.00
−0.05
SNP effect on Pneumonia || id:ieu-b-4976
0
1
2
3
4
SNP effect on CD25 on IgD+ CD24− B cell || id:ebi-a-GCST90001779

AT

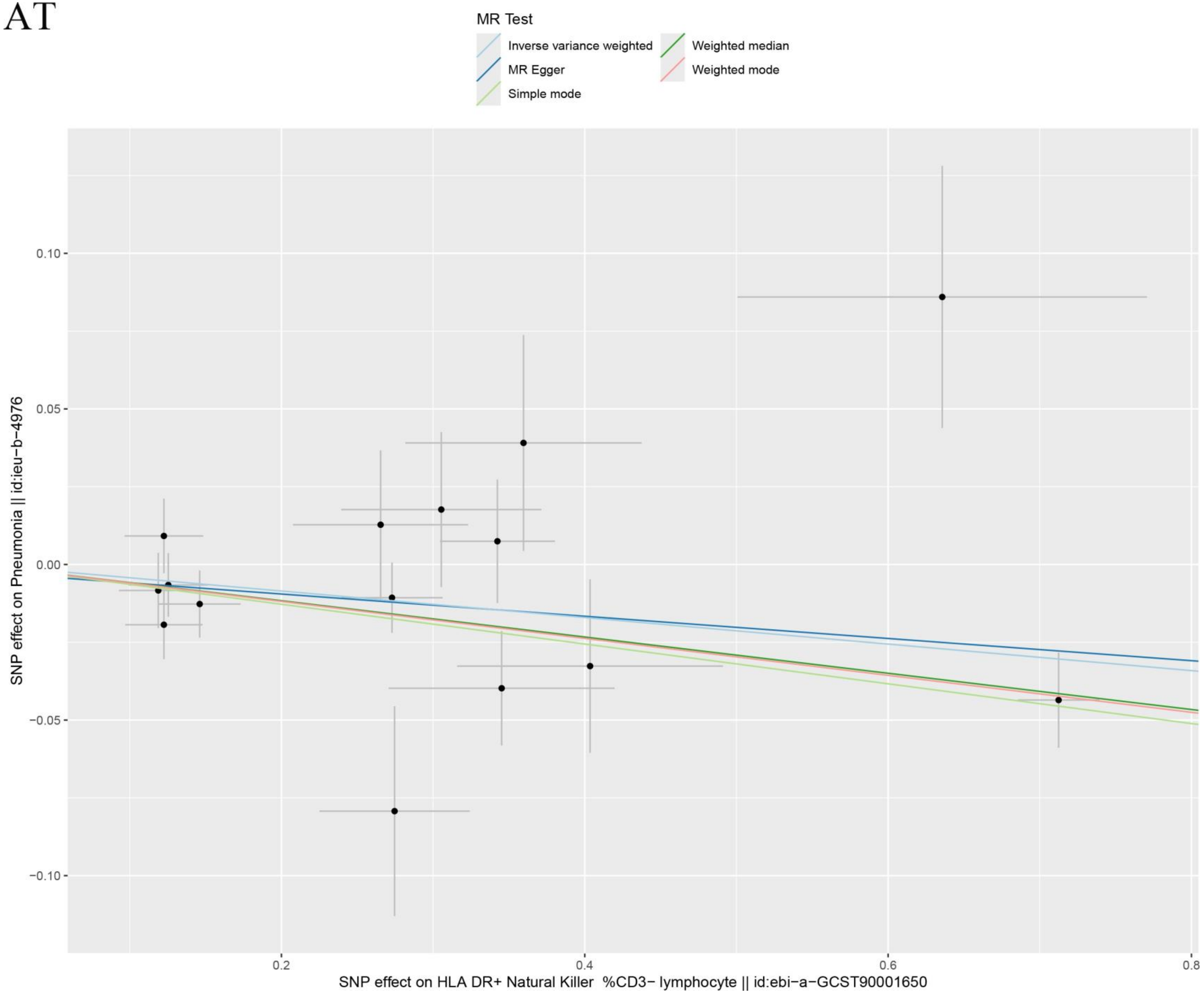
MR Test
Inverse variance weighted
MR Egger
Simple mode
Weighted median
Weighted mode
0.10
0.05
0.00
−0.05
−0.10
SNP effect on Pneumonia || id:ieu-b-4976
0.2
0.4
0.6
0.8
SNP effect on HLA DR+ Natural Killer %CD3− lymphocyte || id:ebi−a−GCST90001650

AU

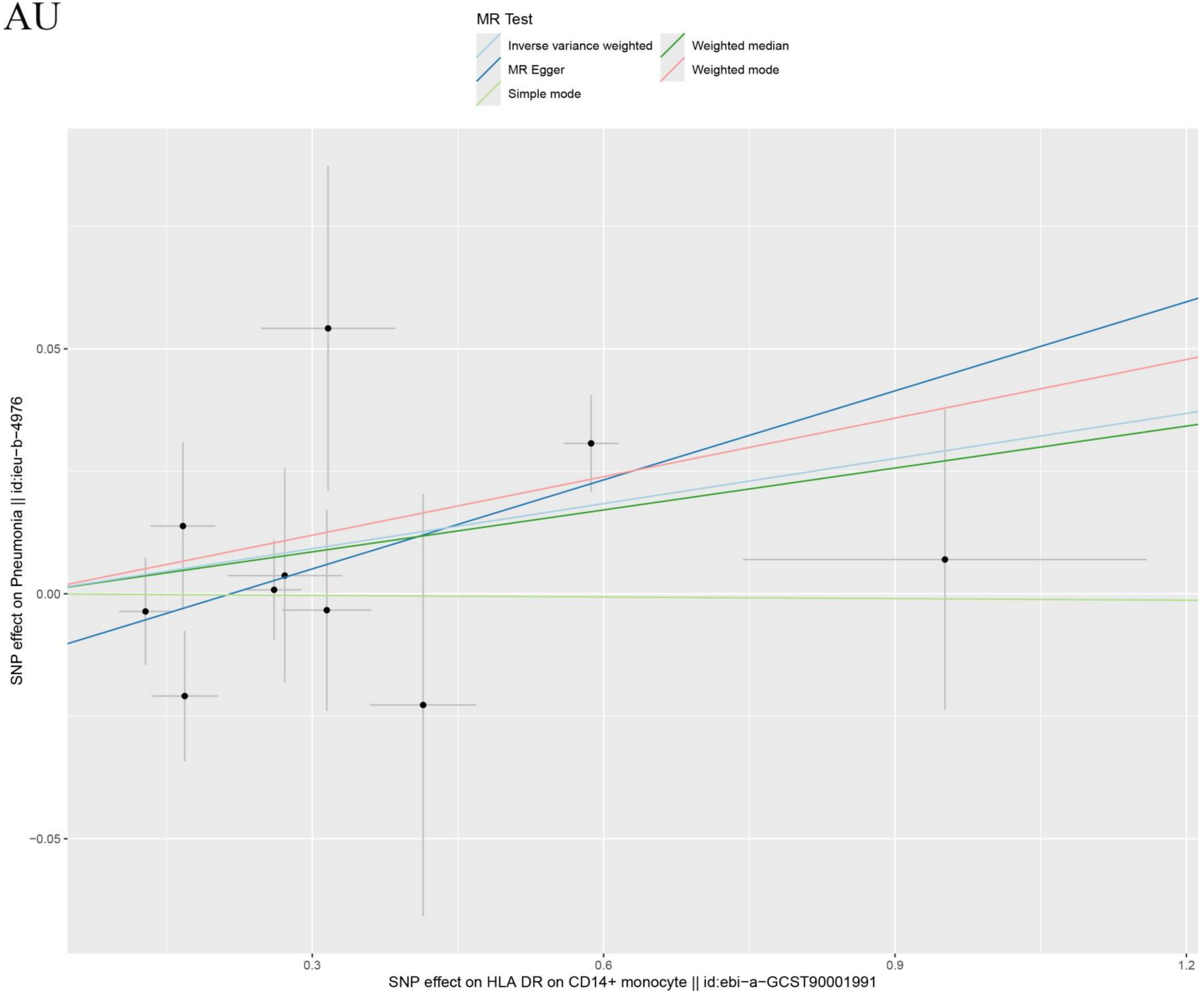
MR Test
Inverse variance weighted
MR Egger
Simple mode
Weighted median
Weighted mode
0.05
0.00
−0.05
0.3
0.6
0.9
1.2
SNP effect on Pneumonia || id:ieu−b−4976
SNP effect on HLA DR on CD14+ monocyte || id:ebi−a−GCST90001991

AV

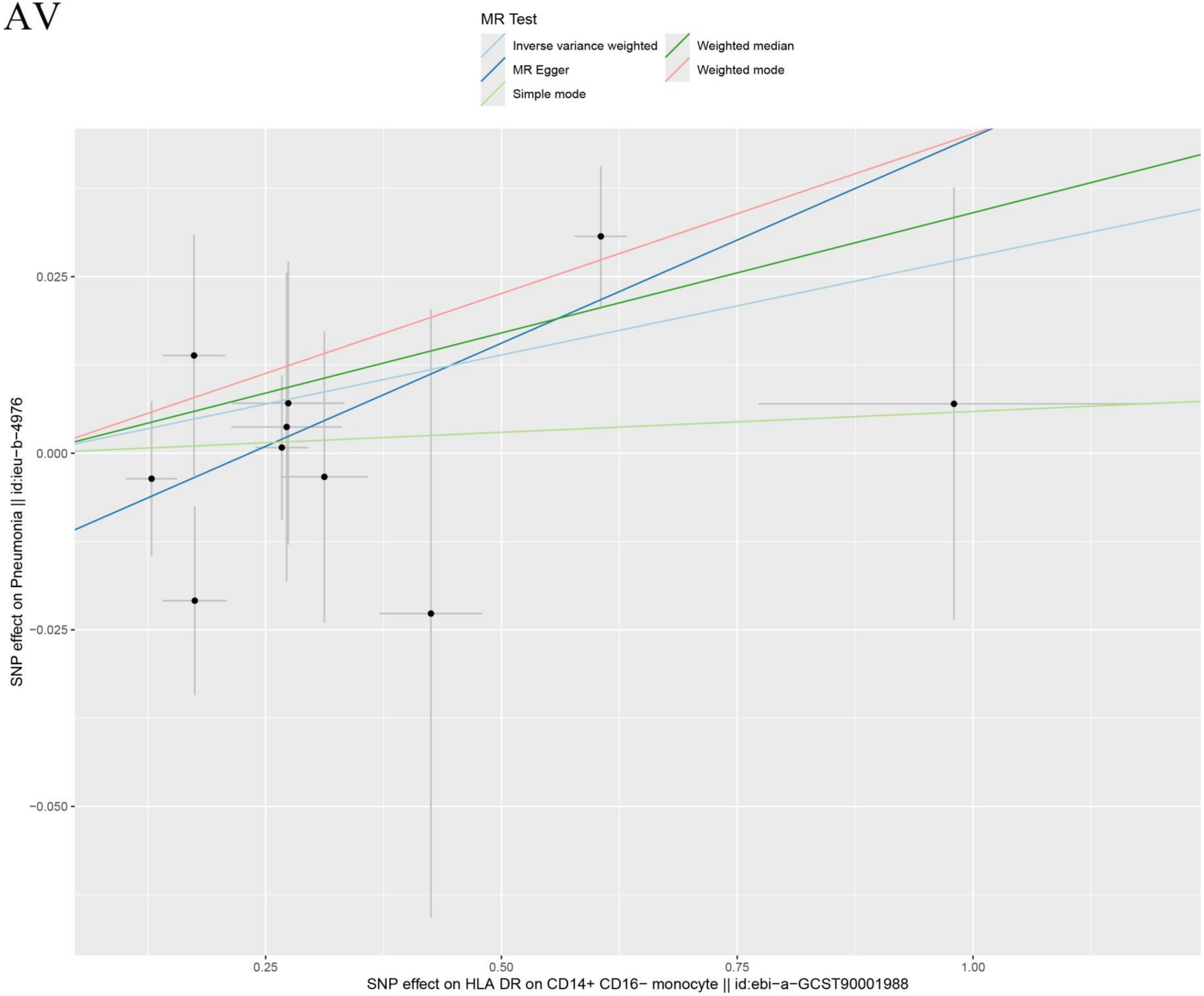
MR Test
Inverse variance weighted
MR Egger
Simple mode
Weighted median
Weighted mode
0.025
0.000
−0.025
−0.050
SNP effect on Pneumonia || id:ieu−b−4976
0.25
0.50
0.75
1.00
SNP effect on HLA DR on CD14+ CD16− monocyte || id:ebi−a−GCST90001988

AW

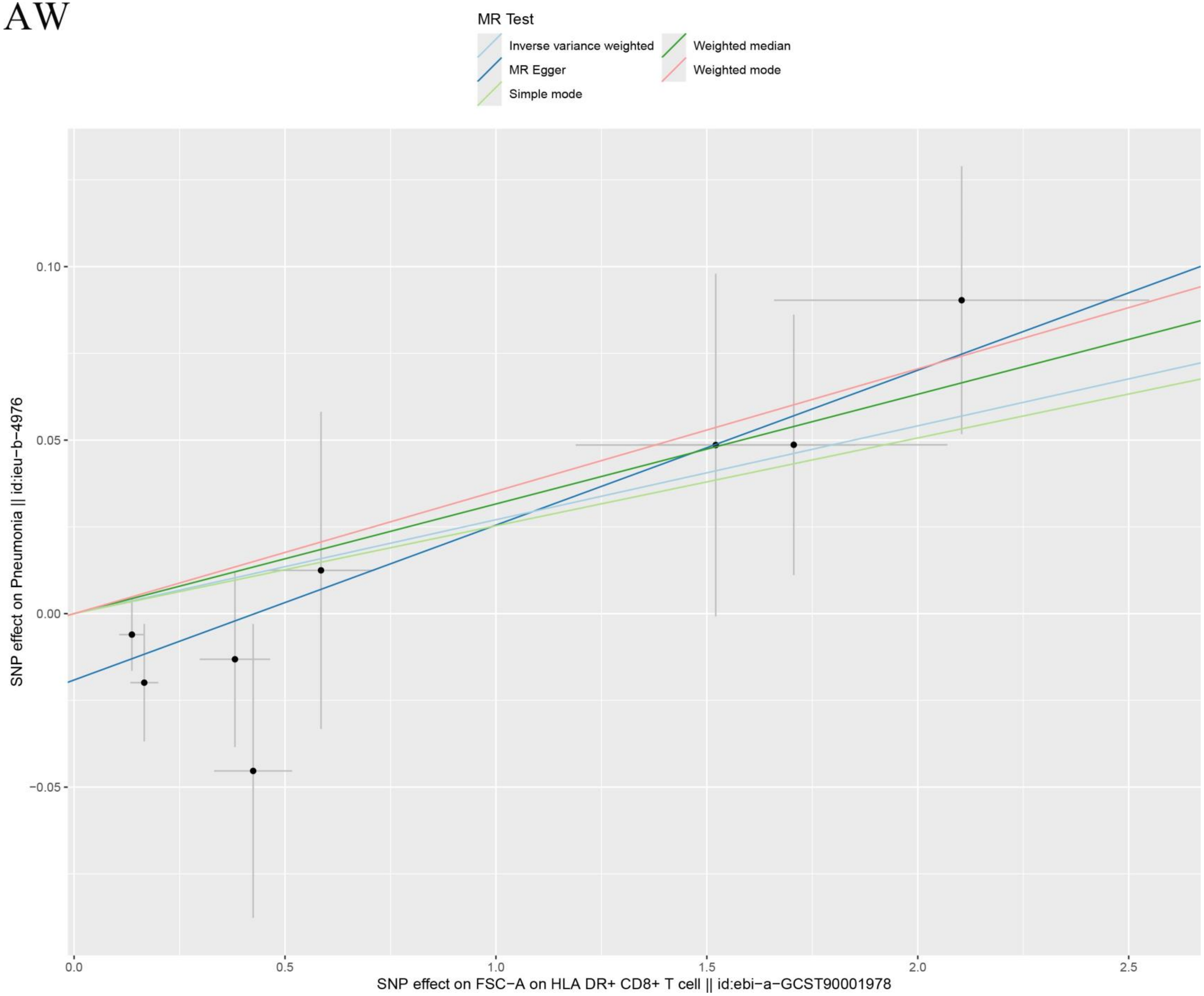
MR Test
Inverse variance weighted
MR Egger
Simple mode
Weighted median
Weighted mode
0.10
0.05
0.00
−0.05
0.0
0.5
1.0
1.5
2.0
2.5
SNP effect on Pneumonia || id:ieu−b−4976
SNP effect on FSC−A on HLA DR+ CD8+ T cell || id:ebi−a−GCST90001978

AX

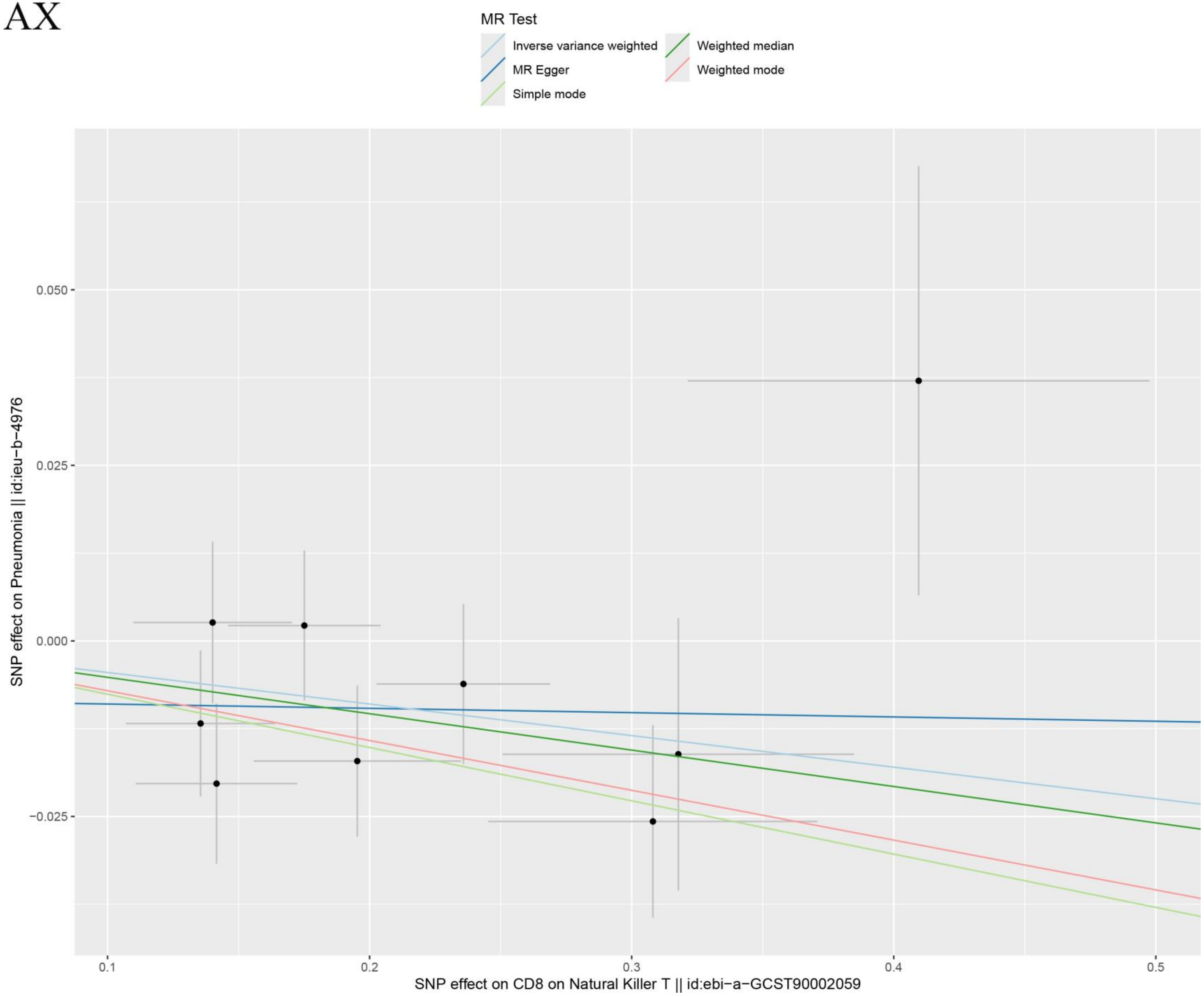
MR Test
Inverse variance weighted
MR Egger
Simple mode
Weighted median
Weighted mode
0.050
0.025
0.000
−0.025
0.1
0.2
0.3
0.4
0.5
SNP effect on Pneumonia || id:ieu−b−4976
SNP effect on CD8 on Natural Killer T || id:ebi−a−GCST90002059

AY

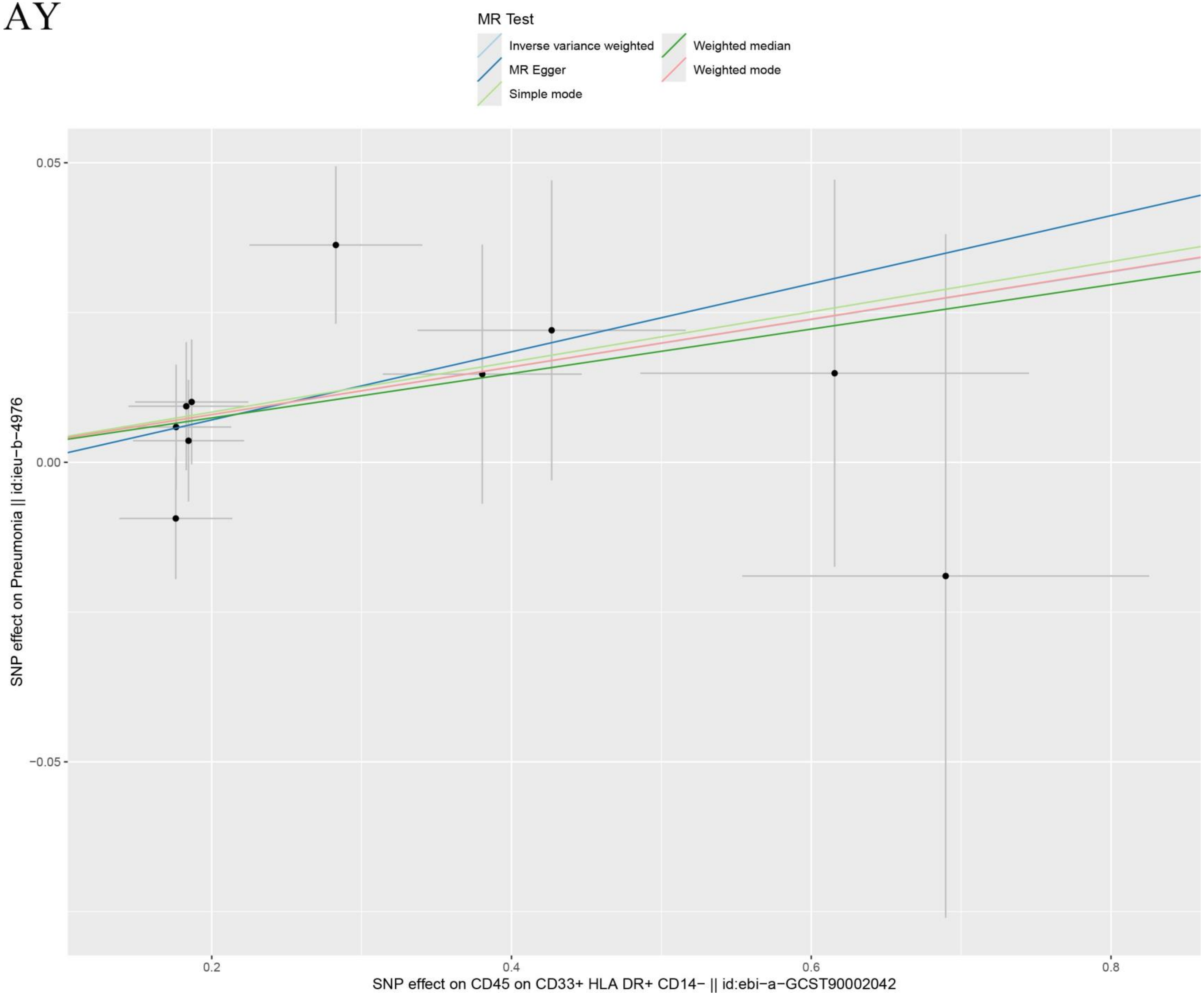

MR Test
Inverse variance weighted
MR Egger
Simple mode
Weighted median
Weighted mode
0.05
0.00
−0.05
0.2
0.4
0.6
0.8
SNP effect on Pneumonia || id:ieu−b−4976
SNP effect on CD45 on CD33+ HLA DR+ CD14− || id:ebi−a−GCST90002042

AZ

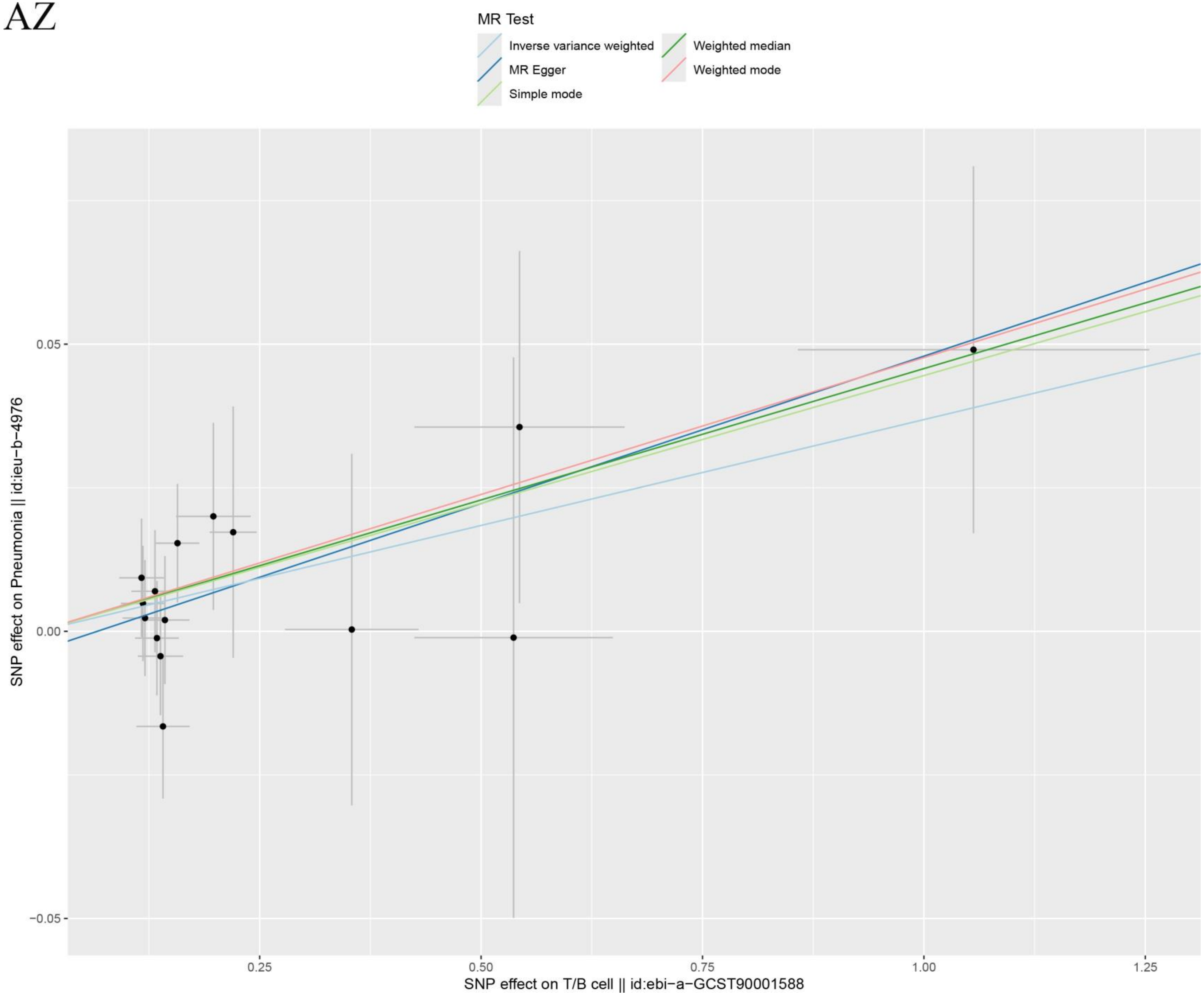

MR Test
Inverse variance weighted
MR Egger
Simple mode
Weighted median
Weighted mode
0.05
0.00
−0.05
0.25
0.50
0.75
1.00
1.25
SNP effect on Pneumonia || id:ieu−b−4976
SNP effect on T/B cell || id:ebi−a−GCST90001588

BA

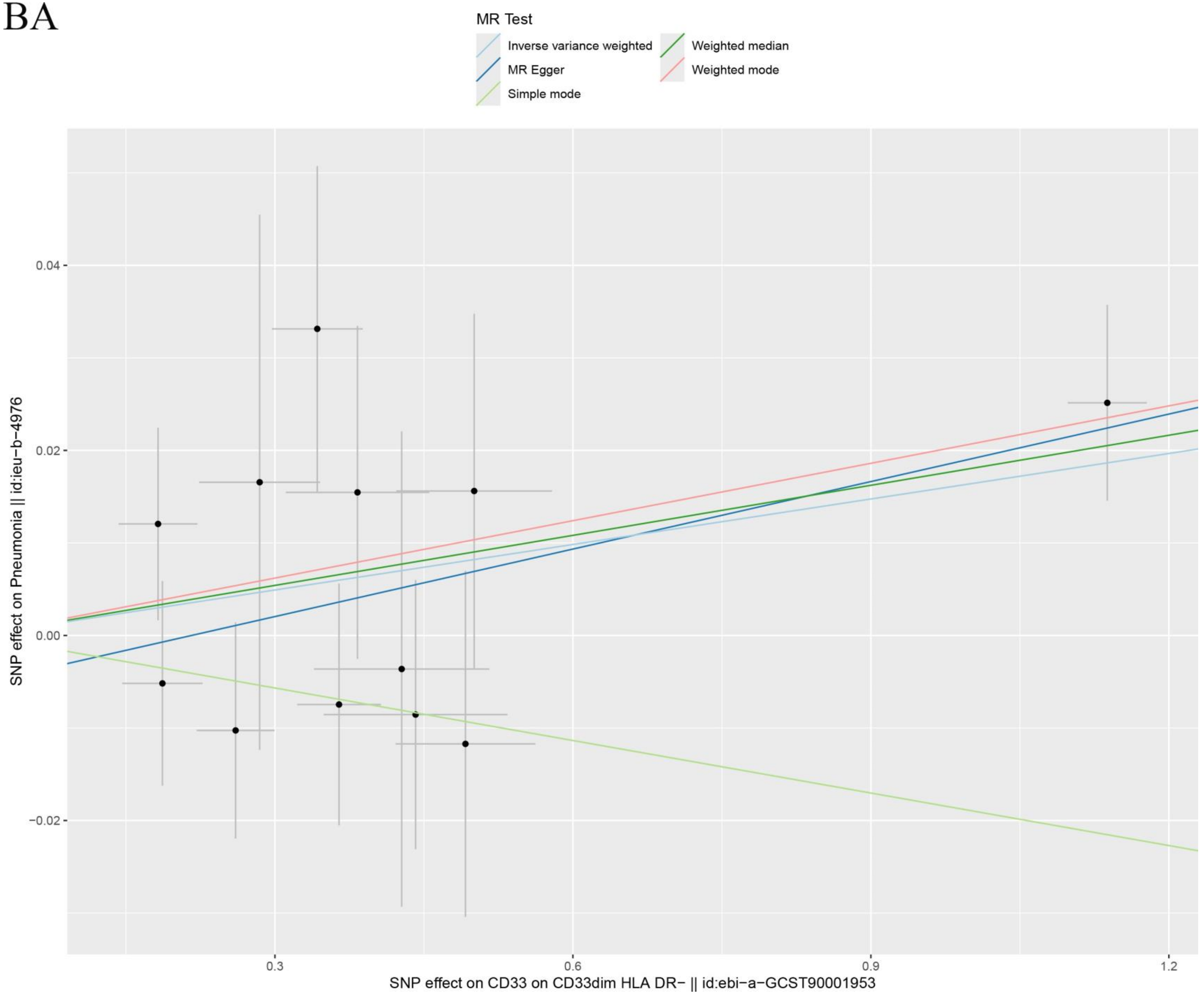

MR Test
Inverse variance weighted
MR Egger
Simple mode
Weighted median
Weighted mode
0.04
0.02
0.00
−0.02
0.3
0.6
0.9
1.2
SNP effect on Pneumonia || id:ieu−b−4976
SNP effect on CD33 on CD33dim HLA DR− || id:ebi−a−GCST90001953

BB

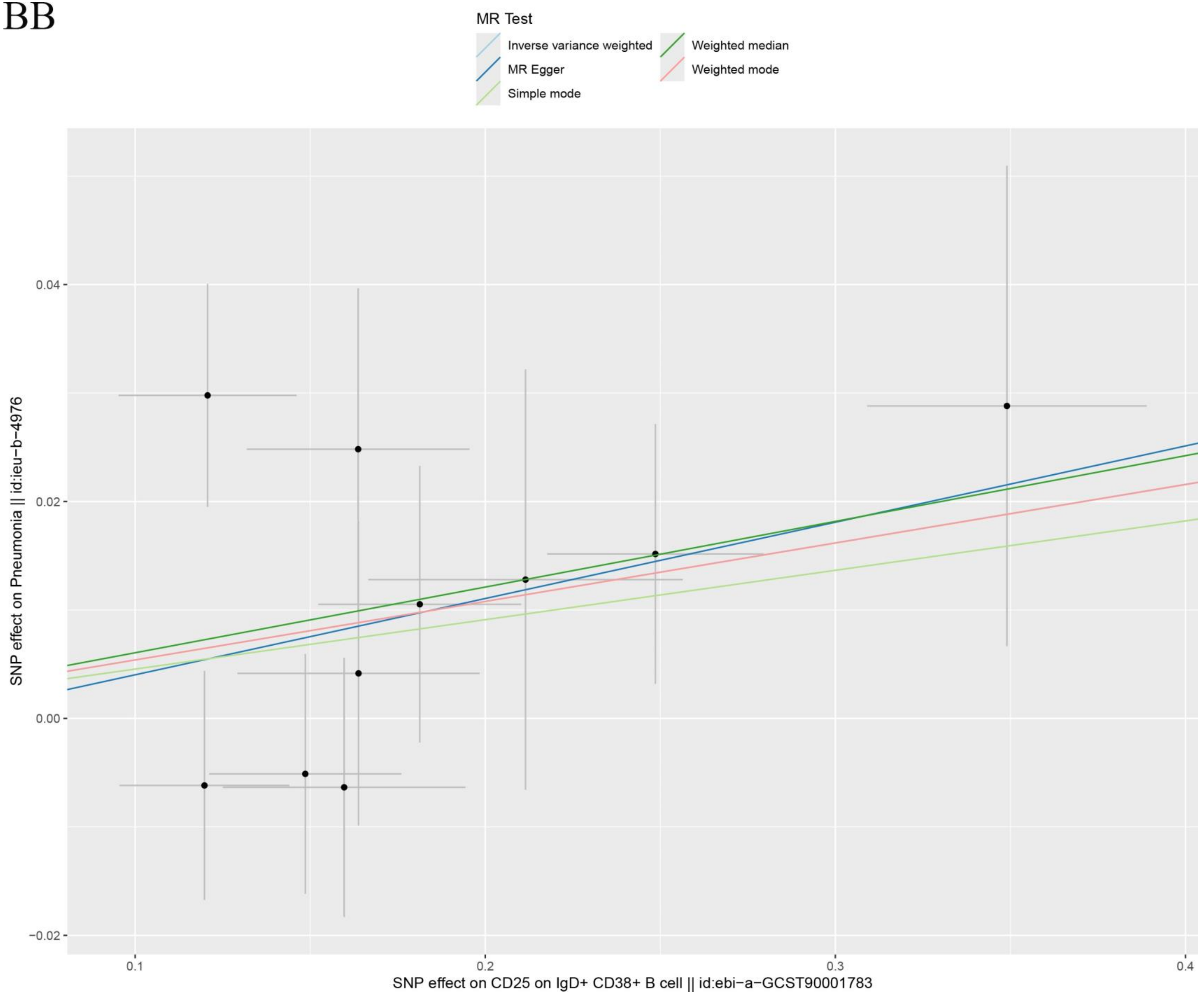
MR Test
Inverse variance weighted
MR Egger
Simple mode
Weighted median
Weighted mode
SNP effect on Pneumonia || id:ieu-b-4976
0.04
0.02
0.00
−0.02
0.1
0.2
0.3
0.4
SNP effect on CD25 on IgD+ CD38+ B cell || id:ebi-a-GCST90001783

BC

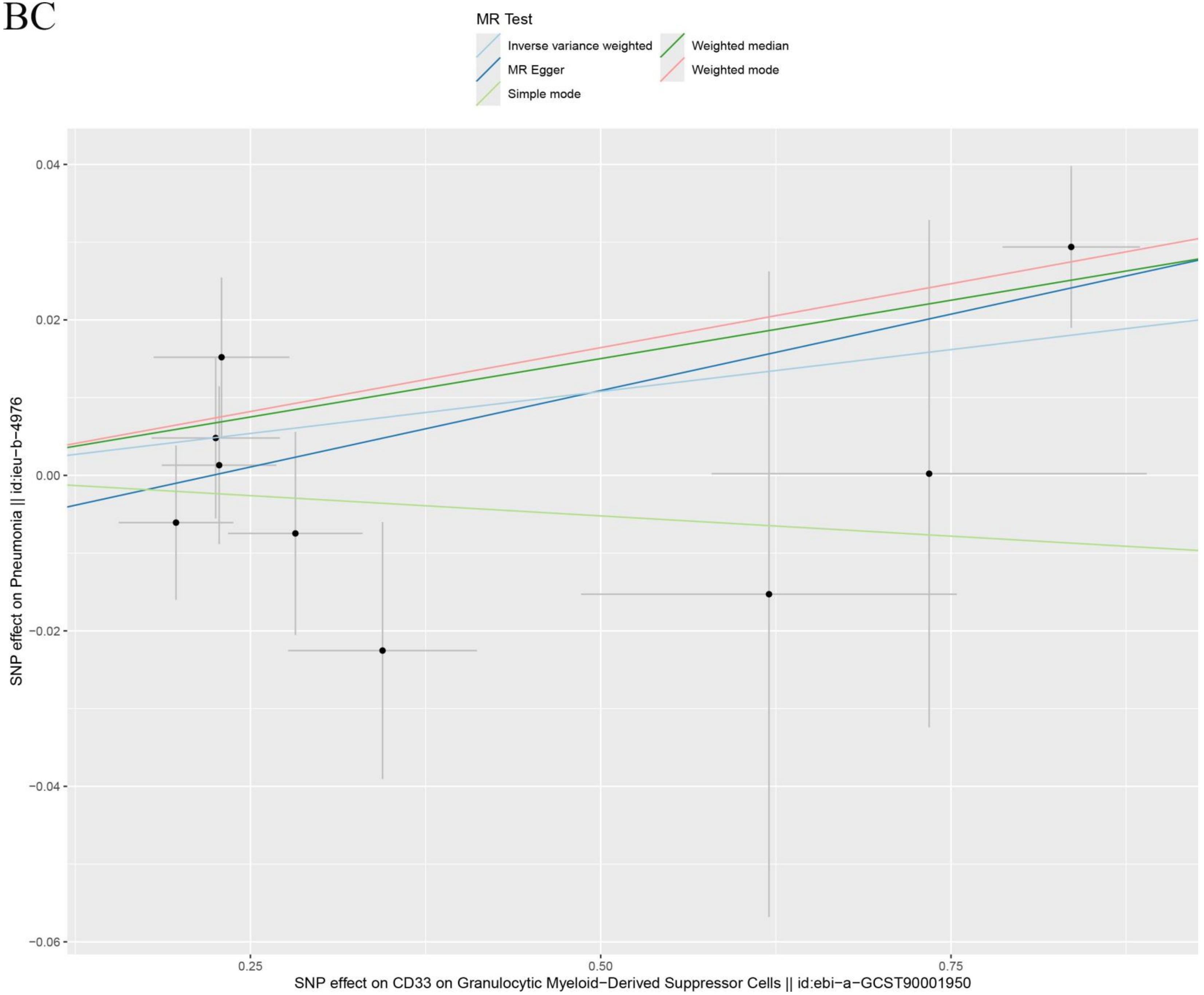
MR Test
Inverse variance weighted
MR Egger
Simple mode
Weighted median
Weighted mode
0.04
0.02
0.00
−0.02
−0.04
−0.06
0.25
0.50
0.75
SNP effect on Pneumonia || id:ieu-b-4976
SNP effect on CD33 on Granulocytic Myeloid−Derived Suppressor Cells || id:ebi−a−GCST90001950

BD

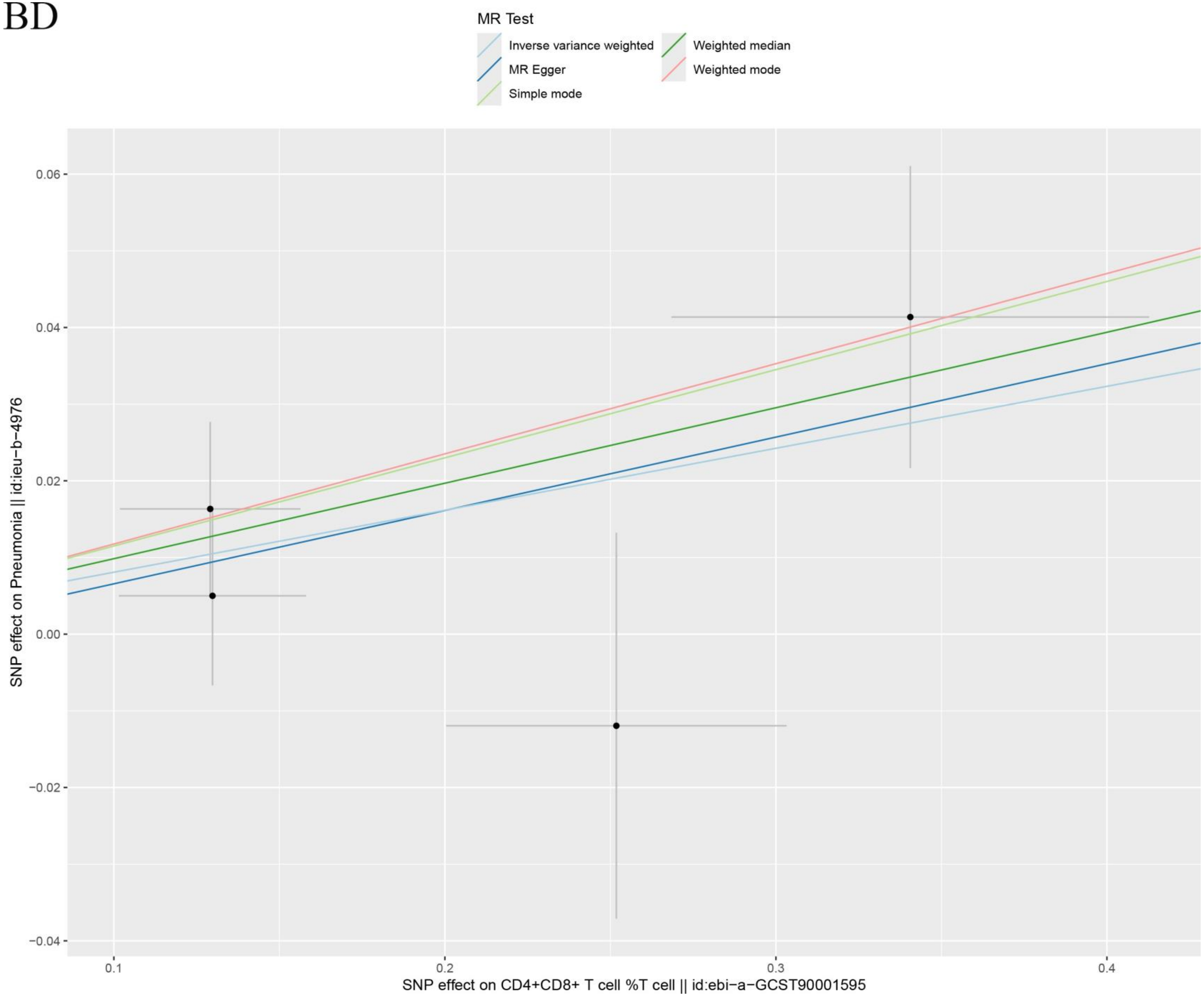
MR Test
Inverse variance weighted
MR Egger
Simple mode
Weighted median
Weighted mode
SNP effect on Pneumonia || id:ieu-b-4976
0.06
0.04
0.02
0.00
−0.02
−0.04
0.1
0.2
0.3
0.4
SNP effect on CD4+CD8+ T cell %T cell || id:ebi-a-GCST90001595

BE

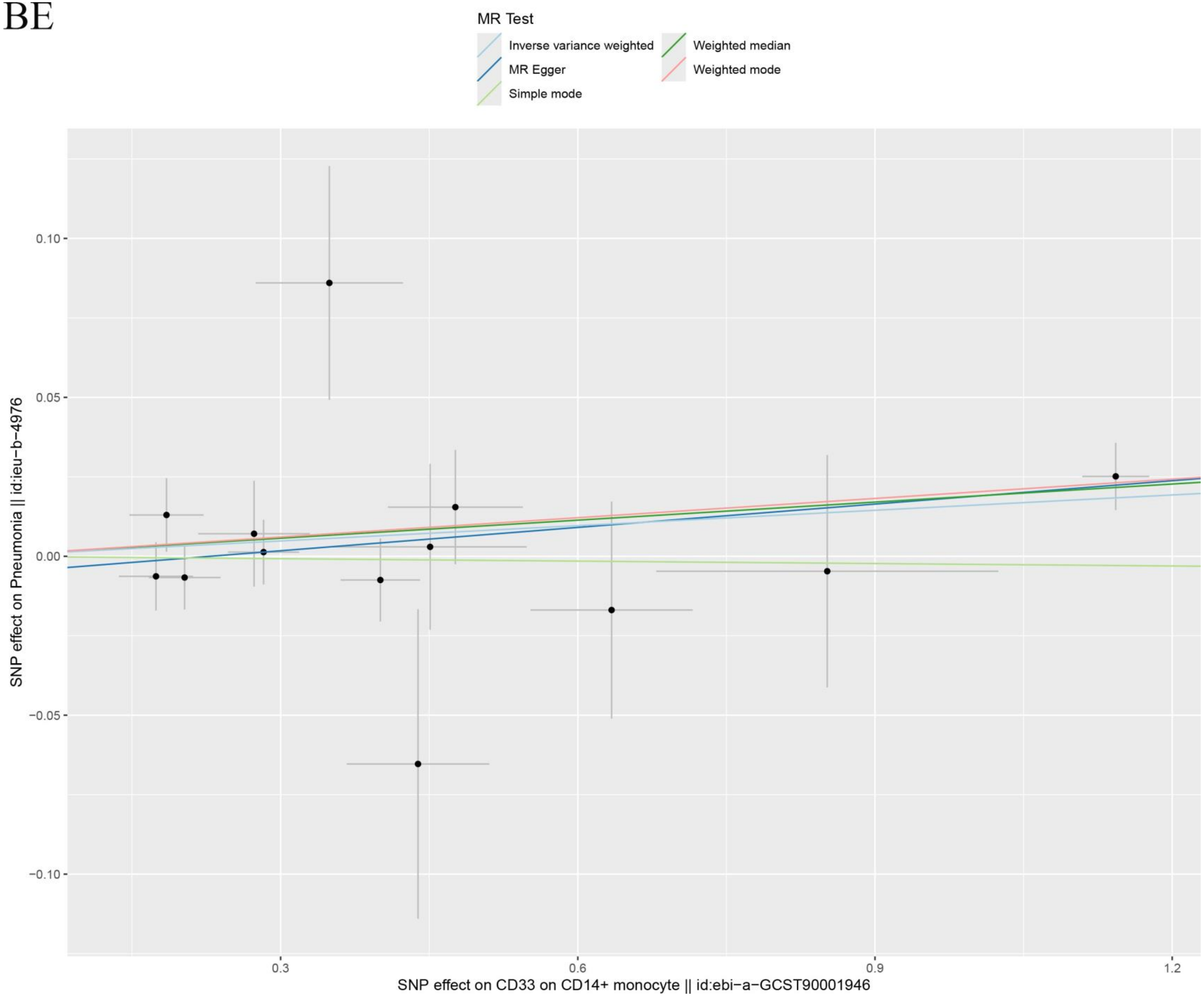
MR Test
Inverse variance weighted
MR Egger
Simple mode
Weighted median
Weighted mode
0.10
0.05
0.00
−0.05
−0.10
SNP effect on Pneumonia || id:ieu-b-4976
0.3
0.6
0.9
1.2
SNP effect on CD33 on CD14+ monocyte || id:ebi−a−GCST90001946

BF

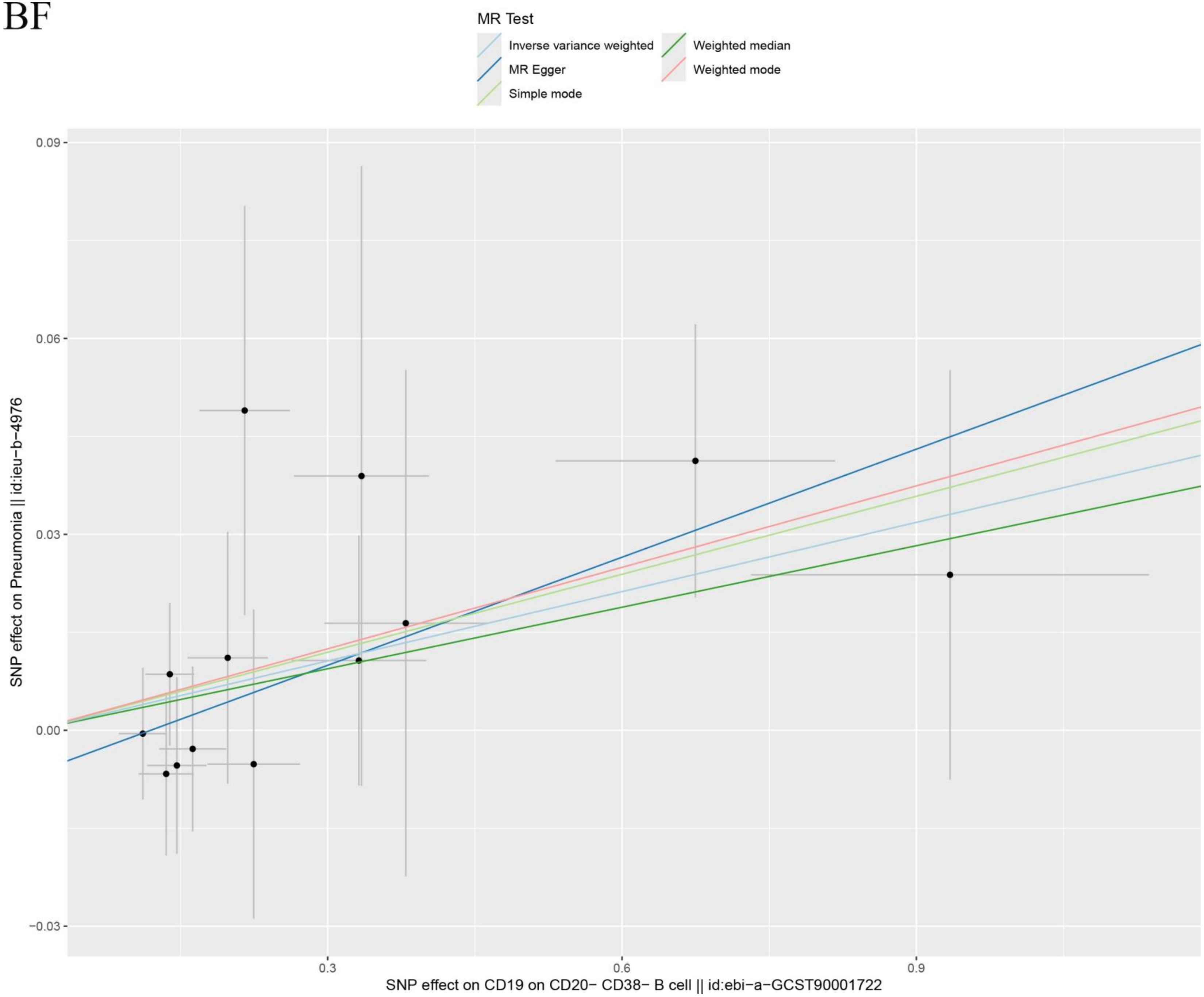
MR Test
Inverse variance weighted
MR Egger
Simple mode
Weighted median
Weighted mode
0.09
0.06
0.03
0.00
−0.03
0.3
0.6
0.9
SNP effect on Pneumonia || id:ieu−b−4976
SNP effect on CD19 on CD20− CD38− B cell || id:ebi−a−GCST90001722

BG

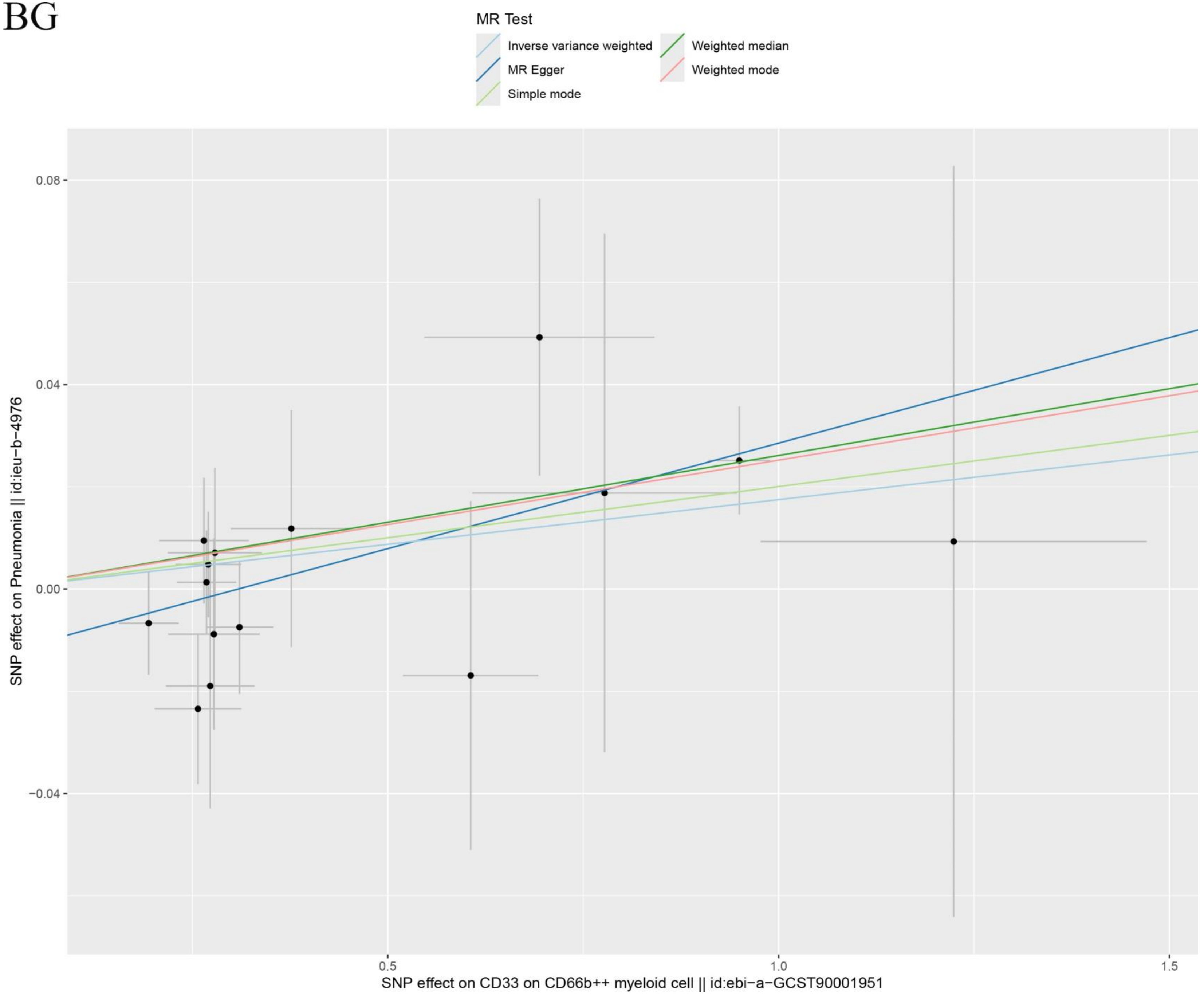
MR Test
Inverse variance weighted
MR Egger
Simple mode
Weighted median
Weighted mode
0.08
0.04
0.00
−0.04
SNP effect on Pneumonia || id:ieu−b−4976
0.5
1.0
1.5
SNP effect on CD33 on CD66b++ myeloid cell || id:ebi−a−GCST90001951

BH

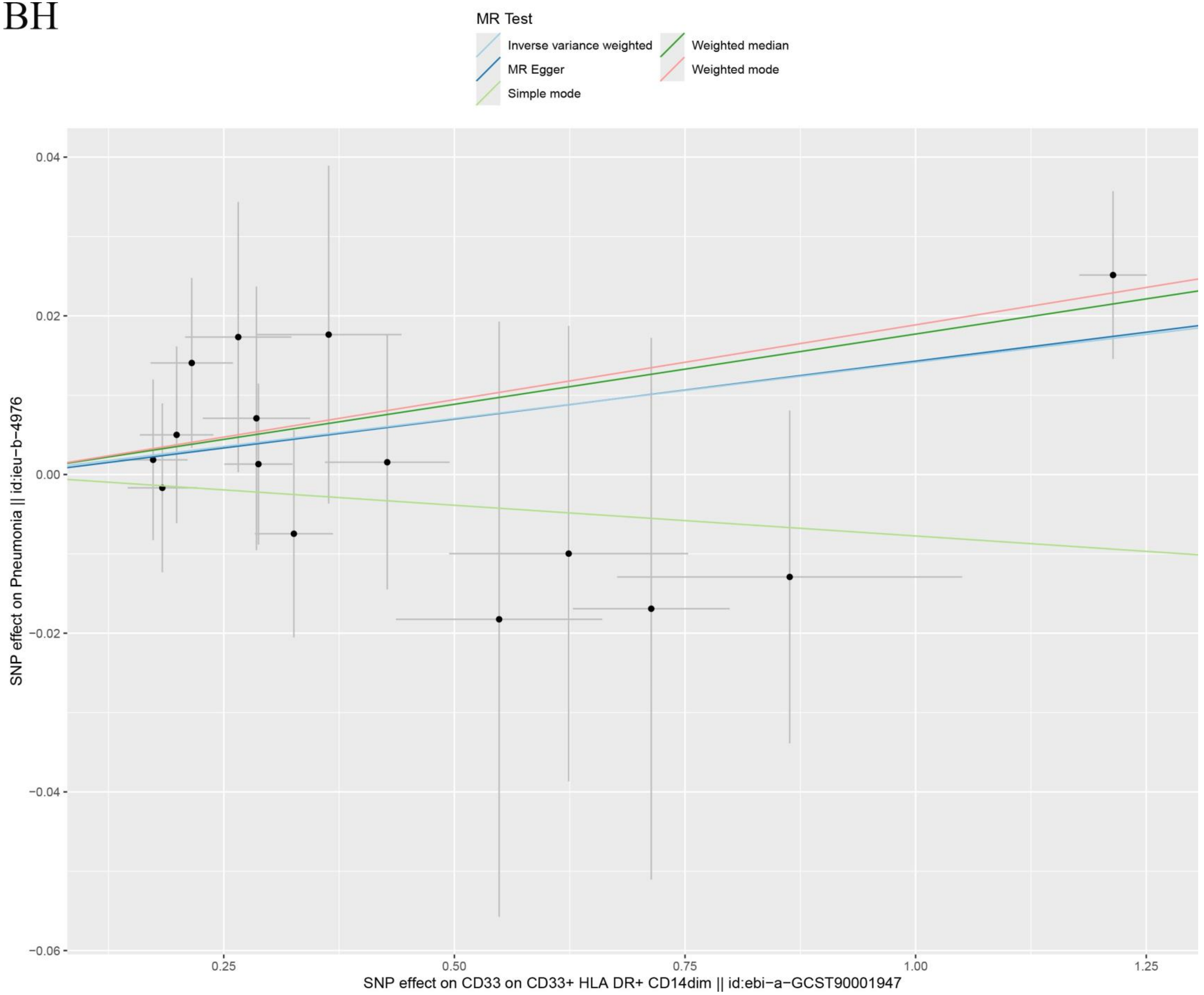
MR Test
Inverse variance weighted
MR Egger
Simple mode
Weighted median
Weighted mode
0.04
0.02
0.00
−0.02
−0.04
−0.06
0.25
0.50
0.75
1.00
1.25
SNP effect on Pneumonia || id:ieu−b−4976
SNP effect on CD33 on CD33+ HLA DR+ CD14dim || id:ebi−a−GCST90001947

BI

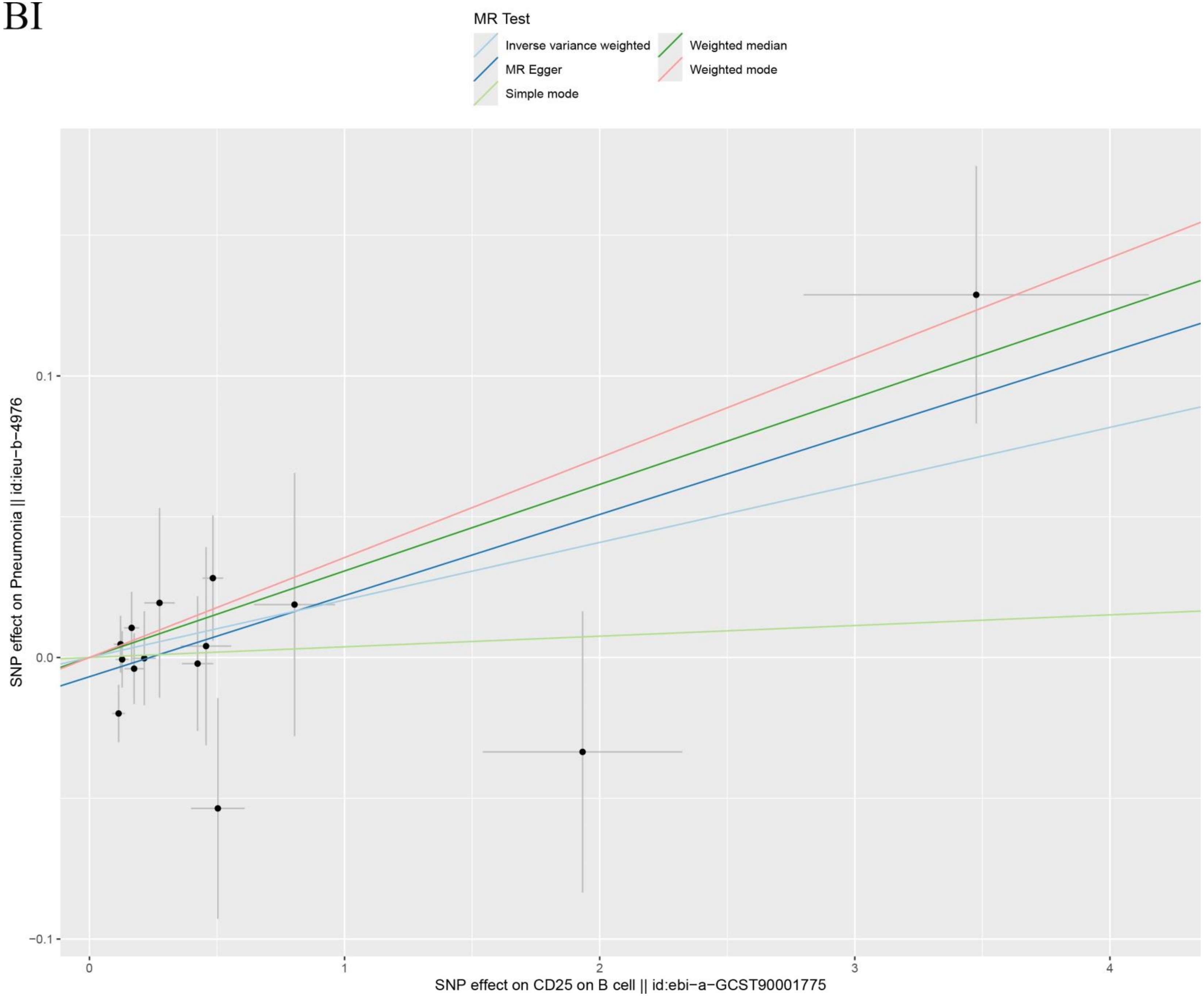
MR Test
Inverse variance weighted
MR Egger
Simple mode
Weighted median
Weighted mode
SNP effect on Pneumonia || id:ieu-b-4976
0.1
0.0
−0.1
0
1
2
3
4
SNP effect on CD25 on B cell || id:ebi-a-GCST90001775

BJ

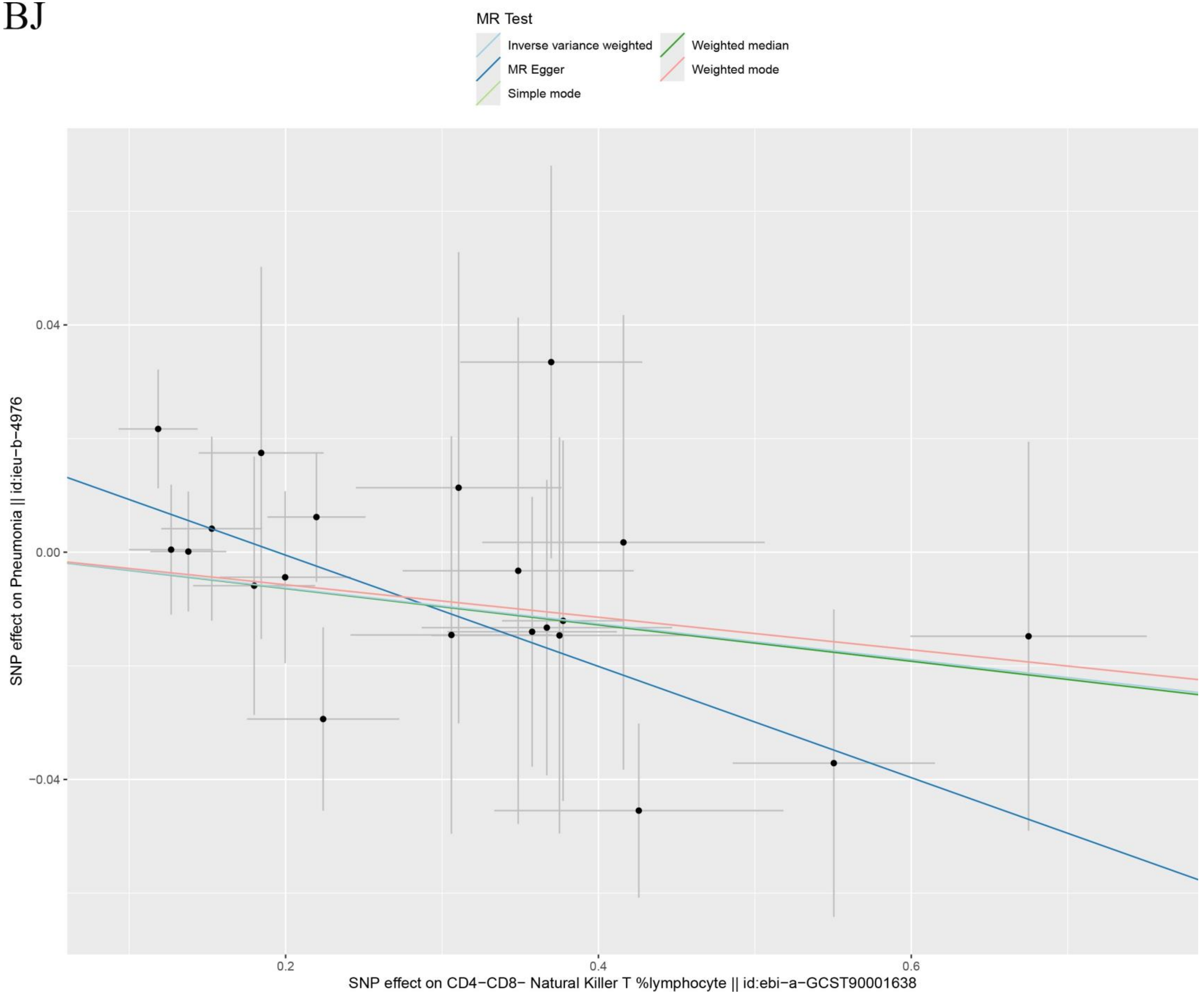
MR Test
Inverse variance weighted
MR Egger
Simple mode
Weighted median
Weighted mode
0.04
0.00
−0.04
SNP effect on Pneumonia || id:ieu−b−4976
0.2
0.4
0.6
SNP effect on CD4−CD8− Natural Killer T %lymphocyte || id:ebi−a−GCST90001638

BK

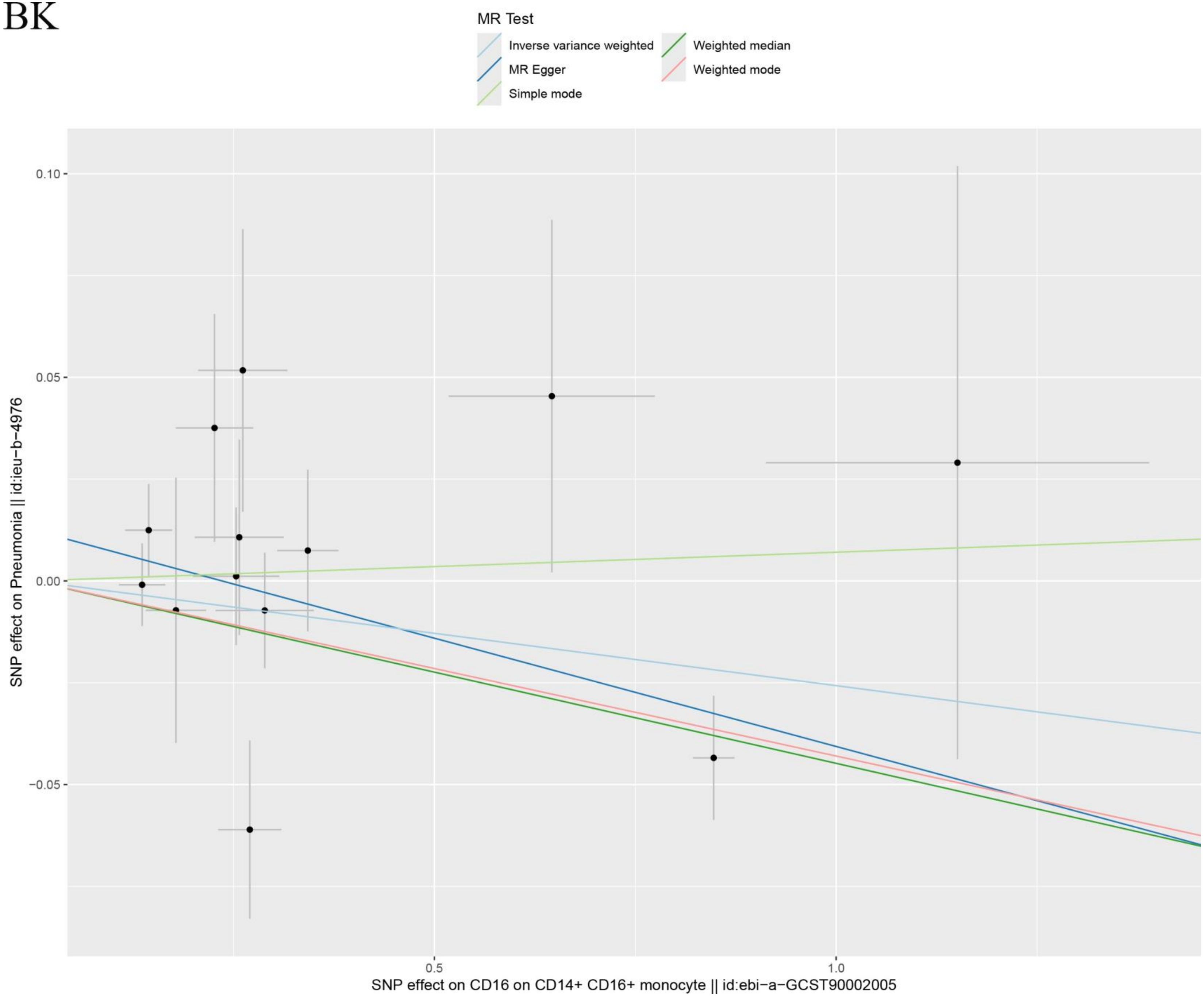
MR Test
Inverse variance weighted
MR Egger
Simple mode
Weighted median
Weighted mode
0.10
0.05
0.00
−0.05
0.5
1.0
SNP effect on Pneumonia || id:ieu−b−4976
SNP effect on CD16 on CD14+ CD16+ monocyte || id:ebi−a−GCST90002005

BL

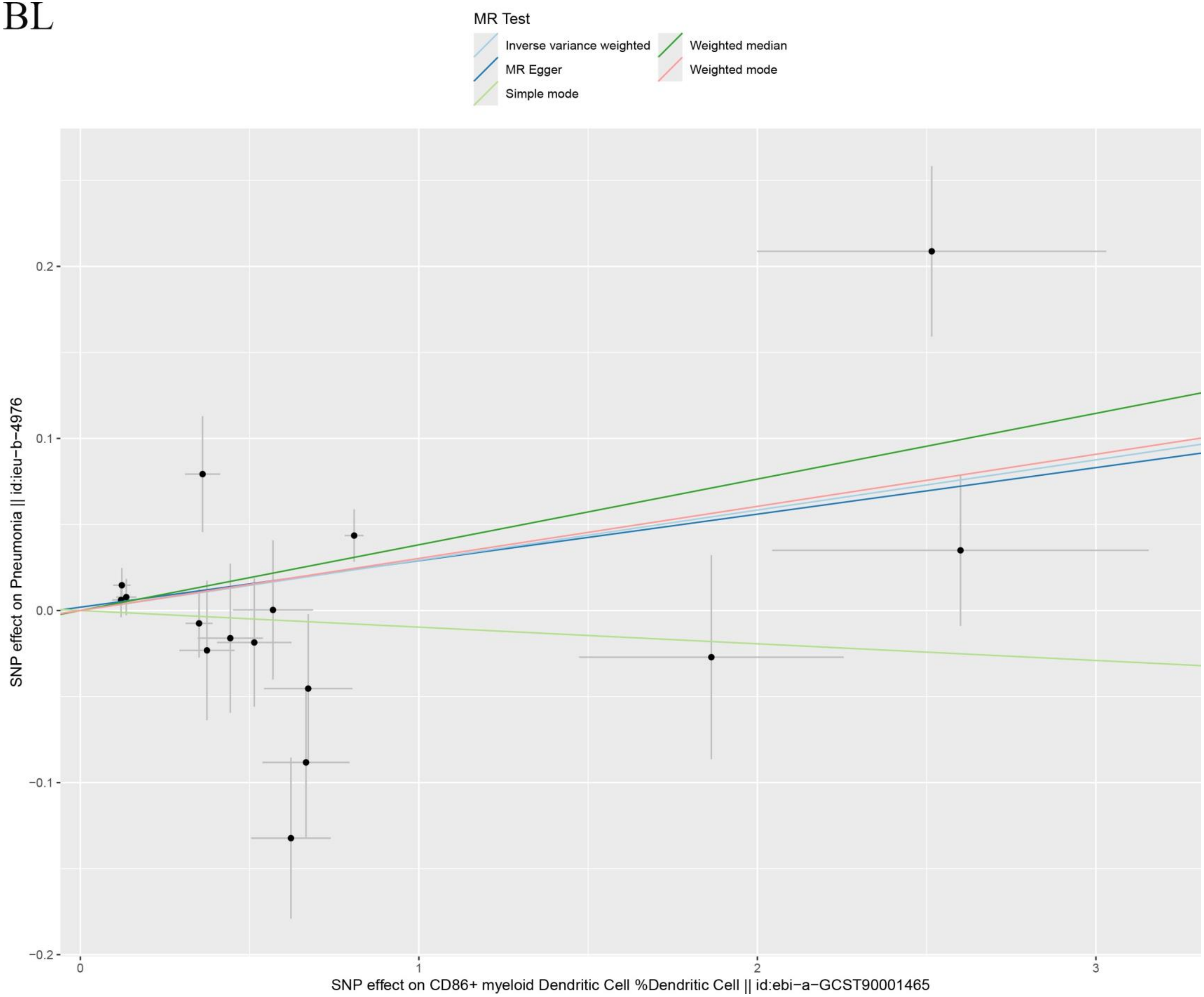
MR Test
Inverse variance weighted
MR Egger
Simple mode
Weighted median
Weighted mode
0.2
0.1
0.0
−0.1
−0.2
SNP effect on Pneumonia || id:ieu−b−4976
0
1
2
3
SNP effect on CD86+ myeloid Dendritic Cell %Dendritic Cell || id:ebi−a−GCST90001465

BM

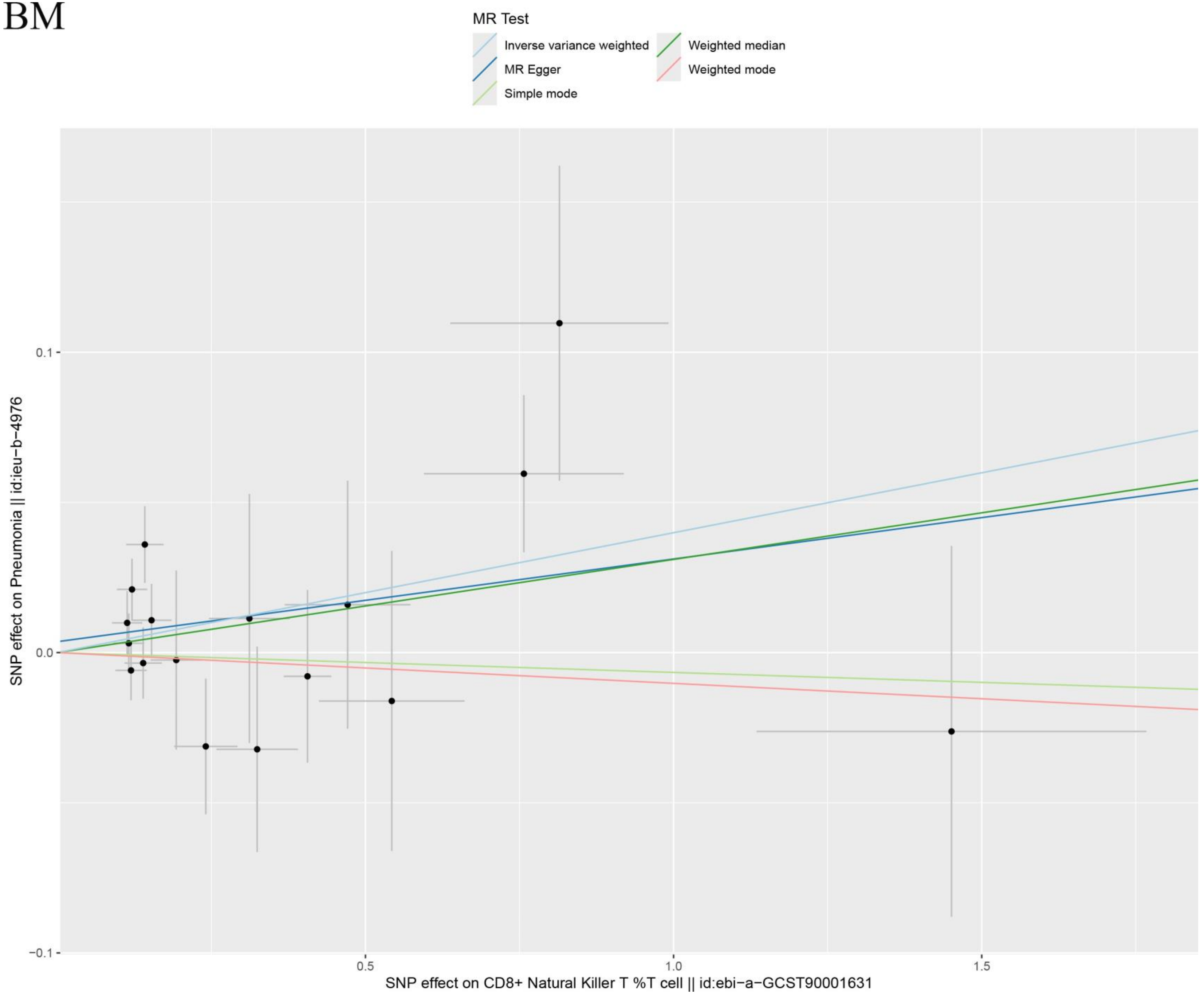
MR Test
Inverse variance weighted
MR Egger
Simple mode
Weighted median
Weighted mode
0.1
0.0
−0.1
0.5
1.0
1.5
SNP effect on Pneumonia || id:ieu−b−4976
SNP effect on CD8+ Natural Killer T %T cell || id:ebi−a−GCST90001631
