# Supplementary material for: Causal Mediation Role of Immune Cells in Gut Microbiota–Pneumonia Associations: A Mendelian Randomisation Study
Source: J Cell Mol Med. 2025 Sep 11;29(17):e70839. doi: 10.1111/jcmm.70839 (PMC12425809; doi:10.1111/jcmm.70839)
Supplement: Supplementary file 4 — S4: Sensitivity analysis of immune cell characteristics and Pneumonia Mendelian randomization (Forest plot). [file JCMM-29-e70839-s010.pdf]

Supplementary Figure 4 Sensitivity analysis of immune cell characteristics and Pneumonia Mendelian randomization (Forest plot).

AA

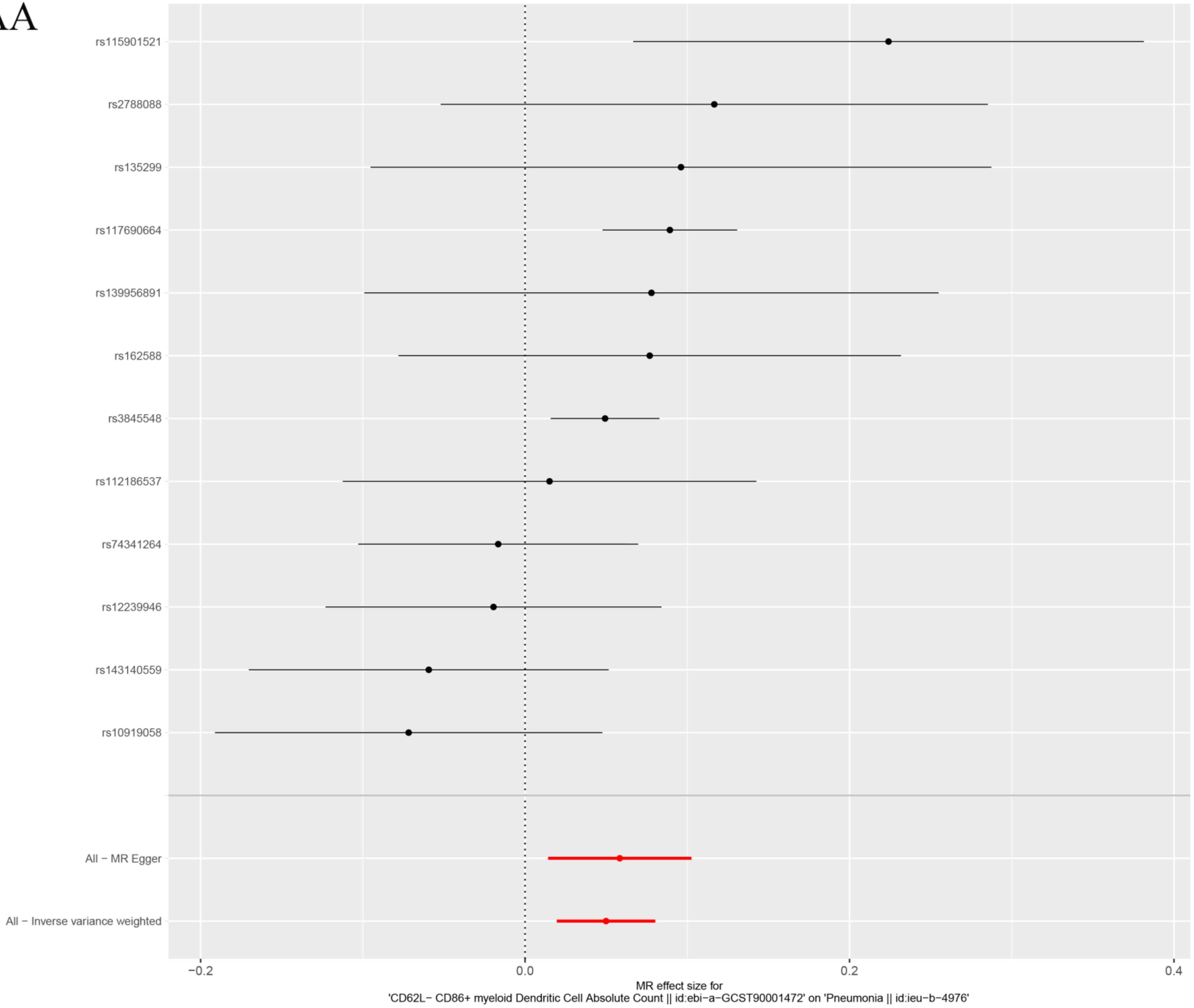

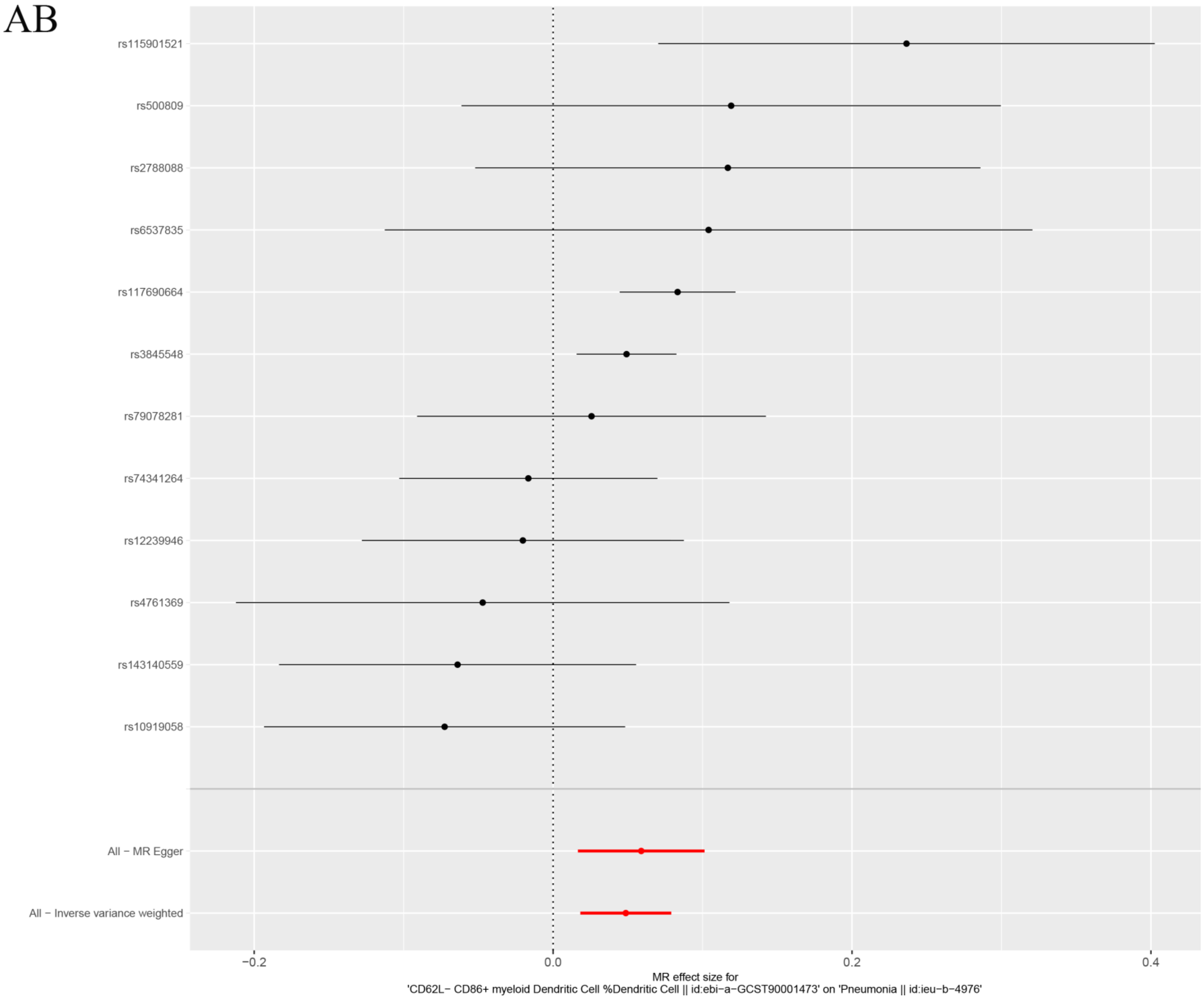
AB
rs115901521
rs500809
rs2788088
rs6537835
rs117690664
rs3845548
rs79078281
rs74341264
rs12239946
rs4761369
rs143140559
rs10919058
All − MR Egger
All − Inverse variance weighted
−0.2
0.0
0.2
0.4
MR effect size for
'CD62L− CD86+ myeloid Dendritic Cell %Dendritic Cell || id:ebi−a−GCST90001473' on 'Pneumonia || id:ieu−b−4976'

AC

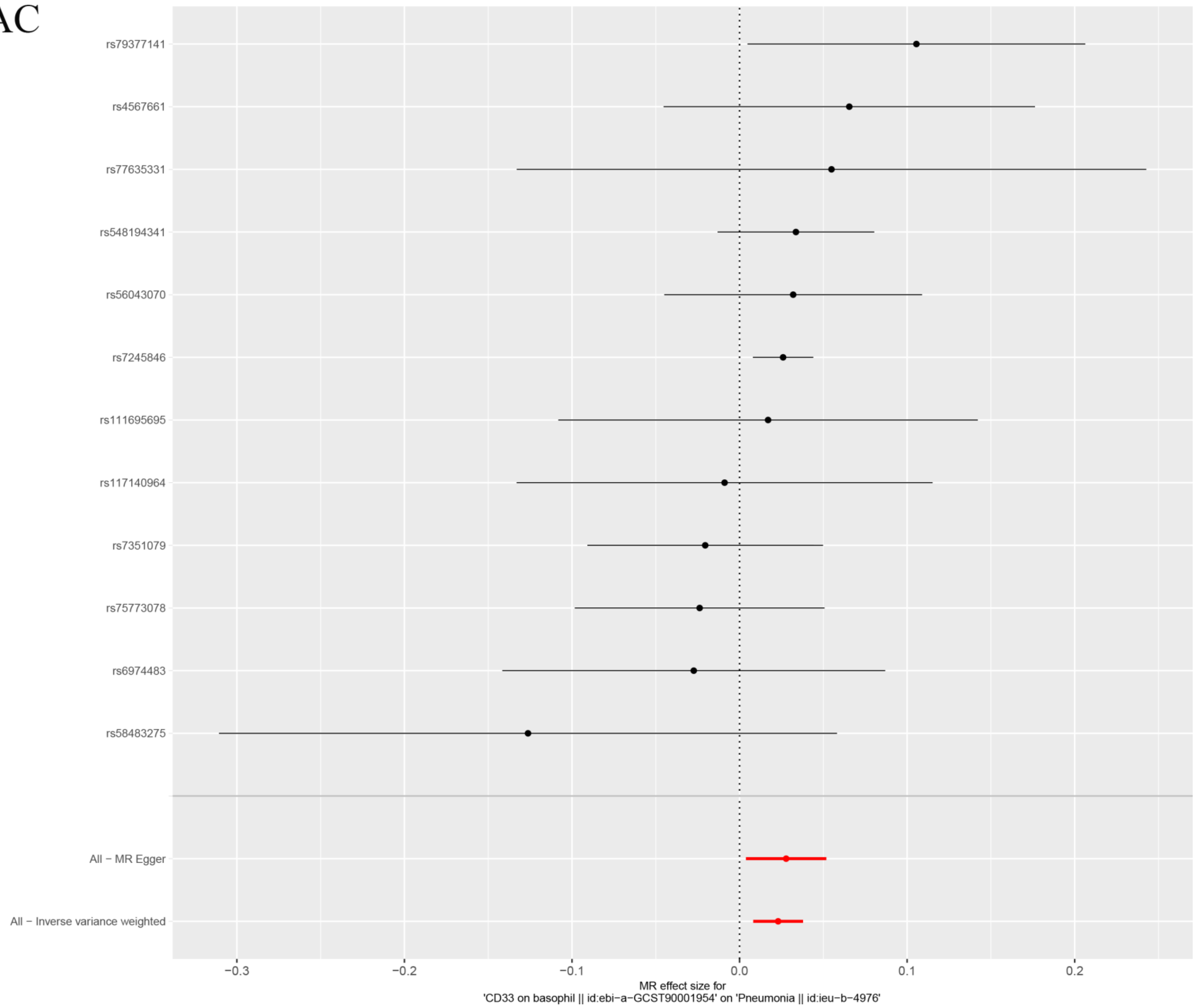
rs79377141
rs4567661
rs77635331
rs548194341
rs56043070
rs7245846
rs111695695
rs117140964
rs7351079
rs75773078
rs6974483
rs58483275
All − MR Egger
All − Inverse variance weighted
−0.3
−0.2
−0.1
0.0
0.1
0.2
MR effect size for
'CD33 on basophil || id:ebi−a−GCST90001954' on 'Pneumonia || id:ieu−b−4976'

AD

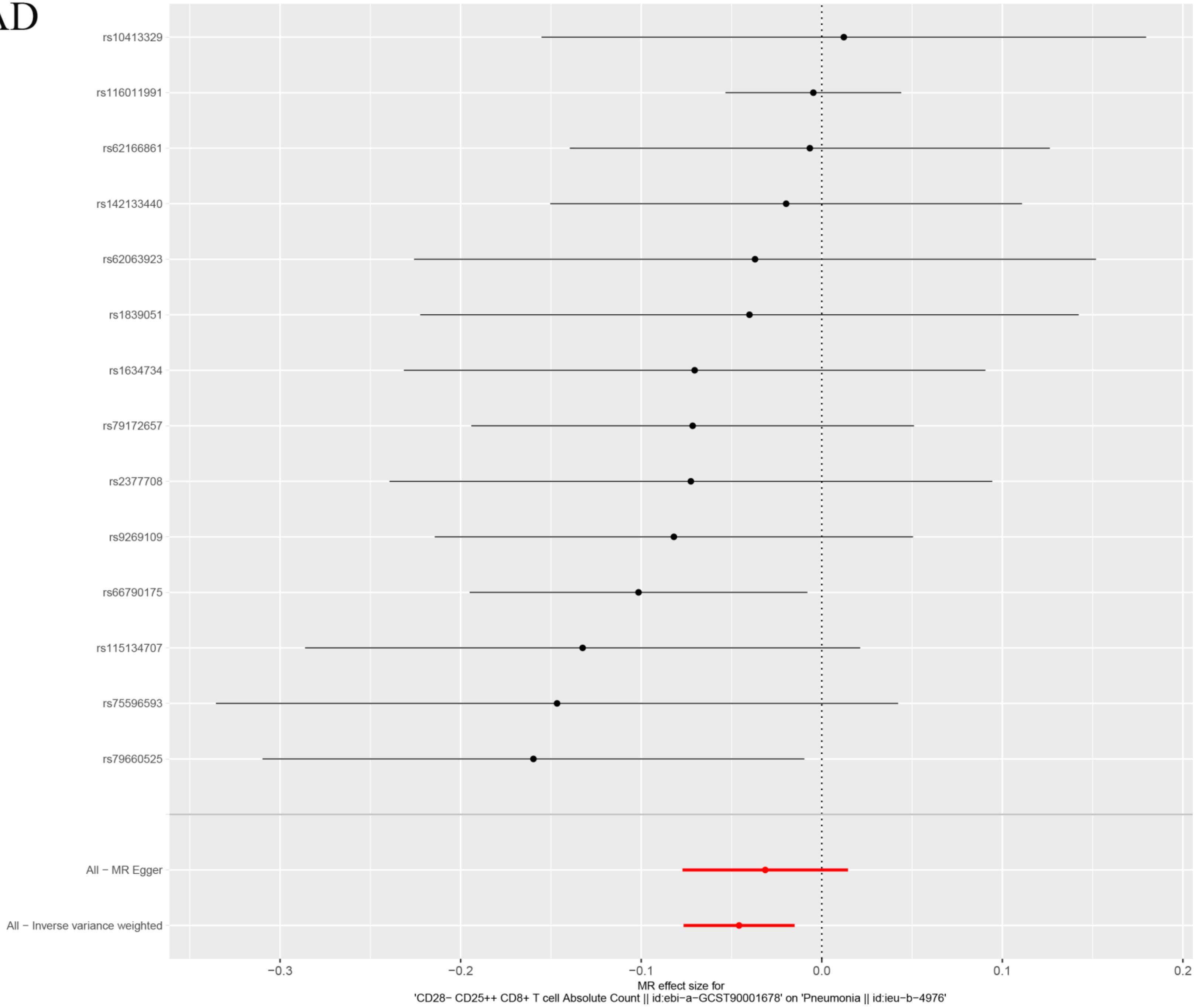
rs10413329
rs116011991
rs62166861
rs142133440
rs62063923
rs1839051
rs1634734
rs79172657
rs2377708
rs9269109
rs66790175
rs115134707
rs75596593
rs79660525
All − MR Egger
All − Inverse variance weighted
−0.3
−0.2
−0.1
0.0
0.1
0.2
MR effect size for
'CD28− CD25++ CD8+ T cell Absolute Count || id:ebi−a−GCST90001678' on 'Pneumonia || id:ieu−b−4976'

AE

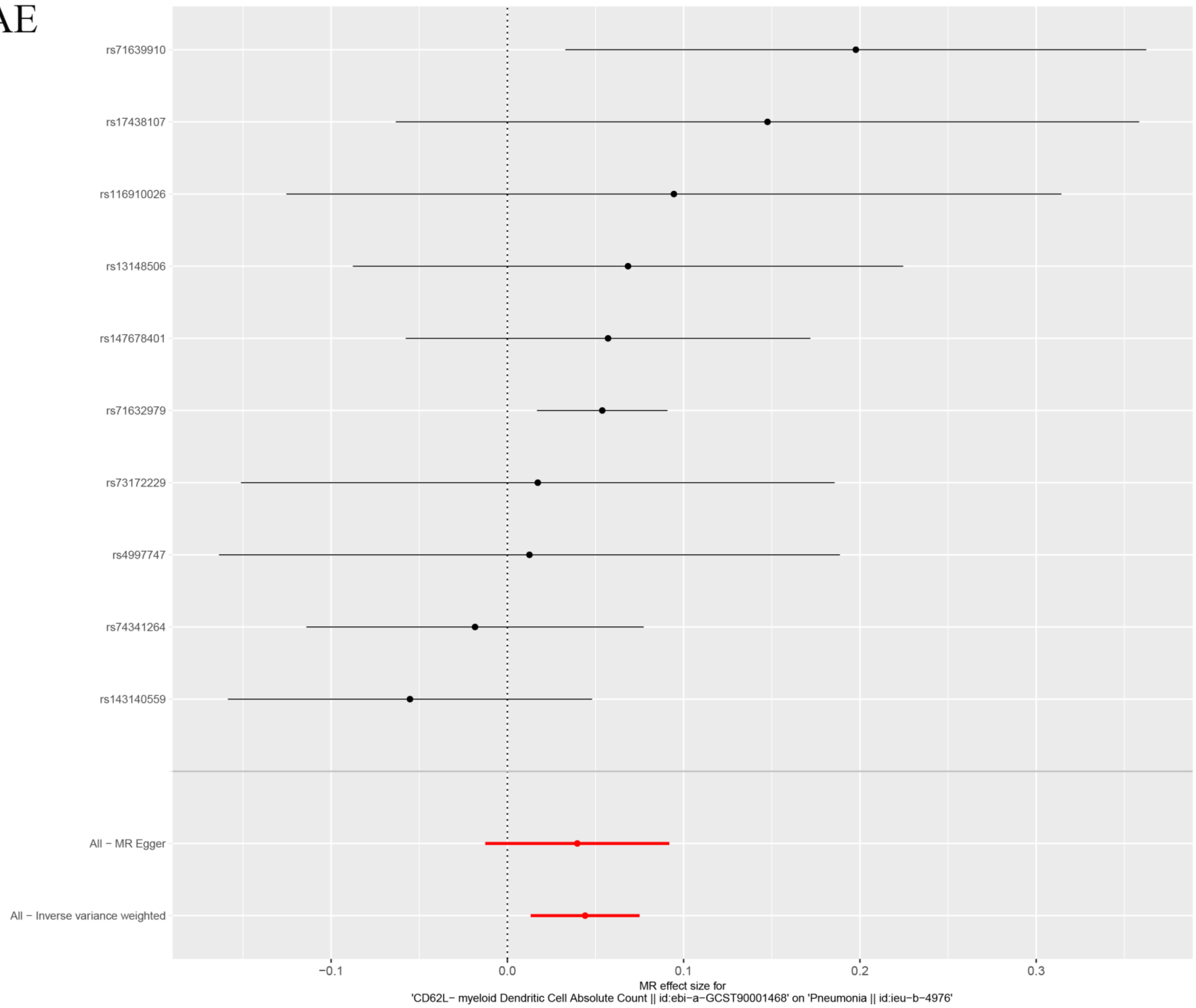

rs71639910
rs17438107
rs116910026
rs13148506
rs147678401
rs71632979
rs73172229
rs4997747
rs74341264
rs143140559
All − MR Egger
All − Inverse variance weighted
−0.1
0.0
0.1
0.2
0.3
MR effect size for
'CD62L− myeloid Dendritic Cell Absolute Count || id:ebi−a−GCST90001468' on 'Pneumonia || id:ieu−b−4976'

AF

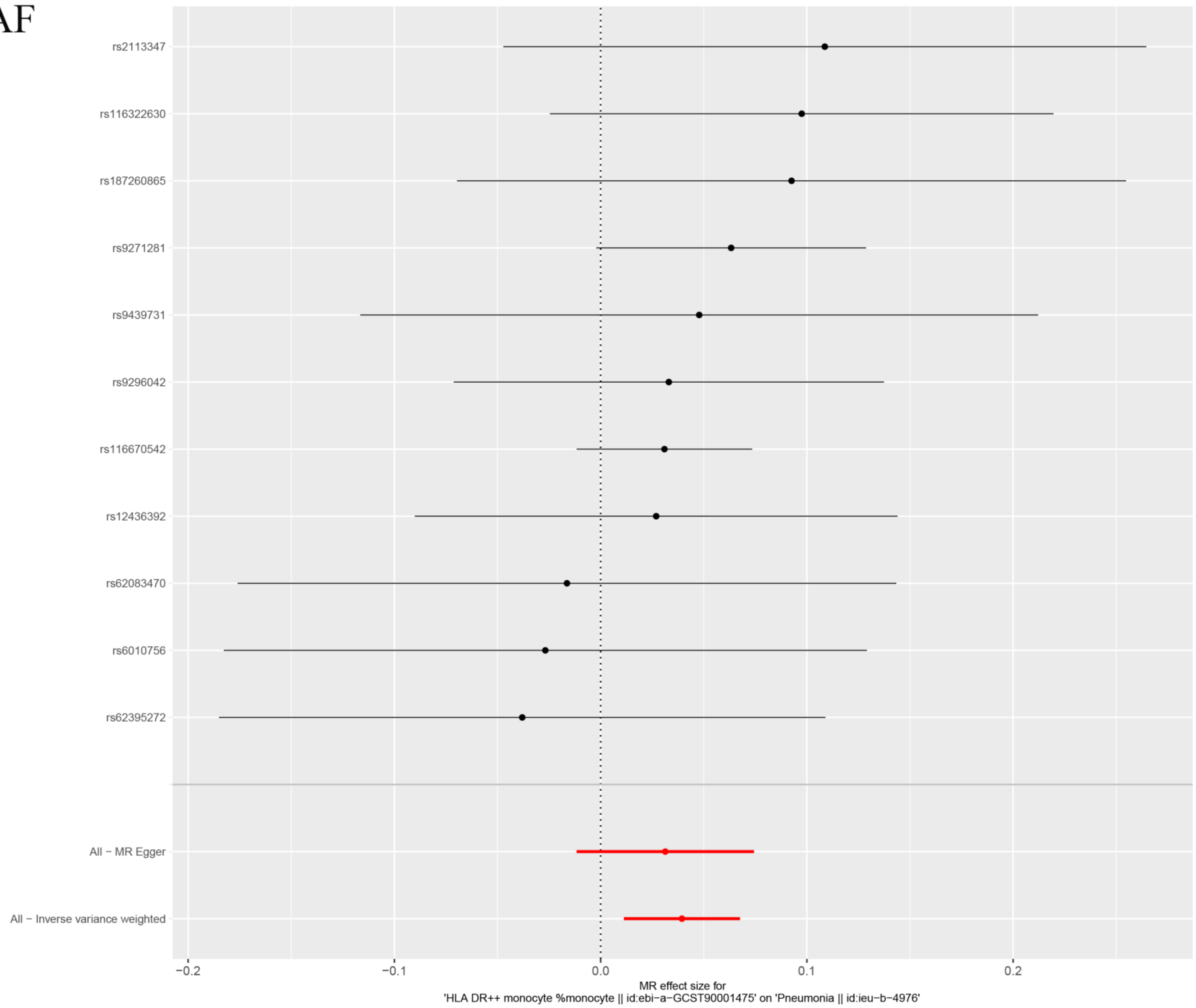
rs2113347
rs116322630
rs187260865
rs9271281
rs9439731
rs9296042
rs116670542
rs12436392
rs62083470
rs6010756
rs62395272
All − MR Egger
All − Inverse variance weighted
−0.2
−0.1
0.0
0.1
0.2
MR effect size for
'HLA DR++ monocyte %monocyte || id:ebi−a−GCST90001475' on 'Pneumonia || id:ieu−b−4976'

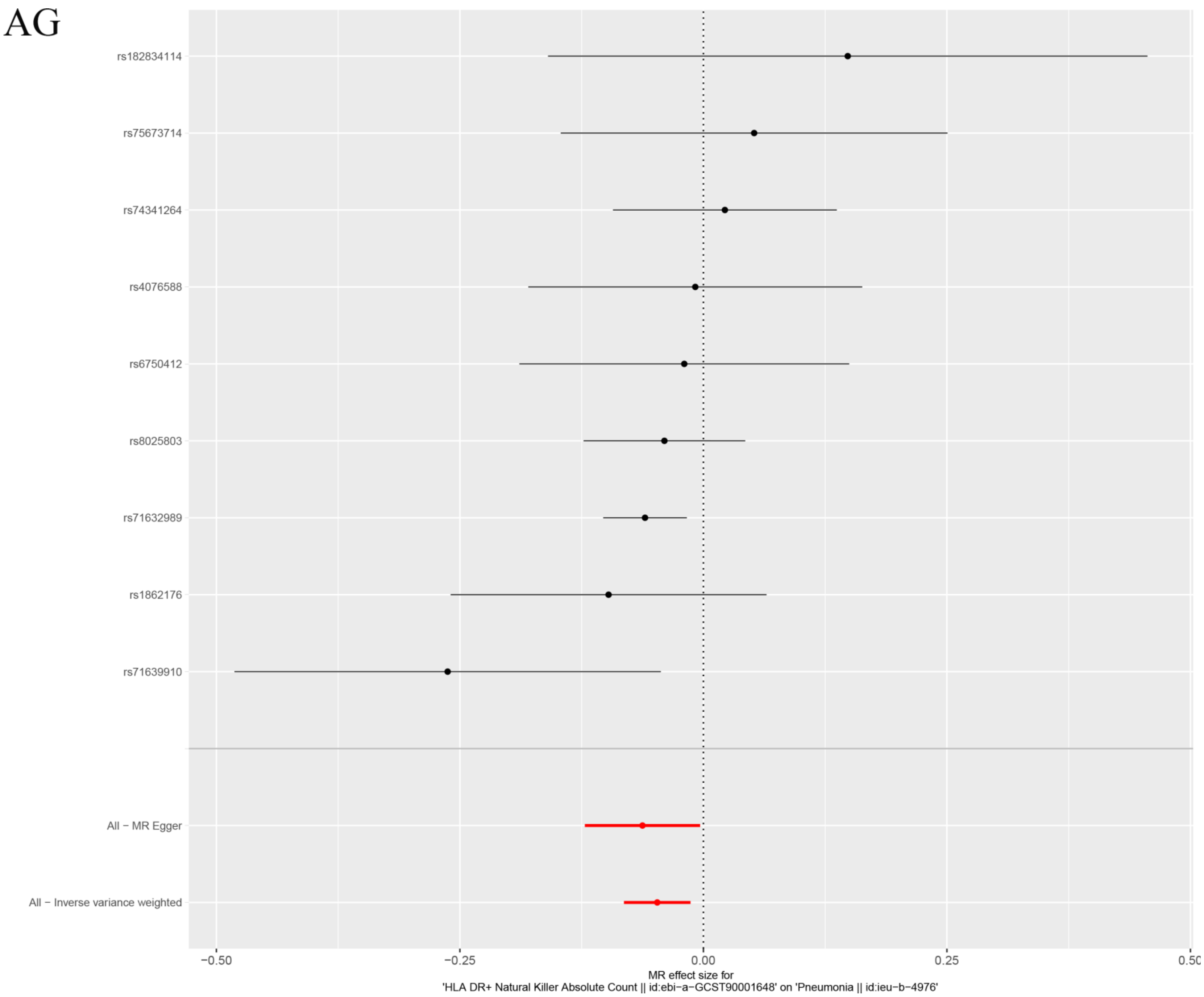
AG
rs182834114
rs75673714
rs74341264
rs4076588
rs6750412
rs8025803
rs71632989
rs1862176
rs71639910
All − MR Egger
All − Inverse variance weighted
−0.50
−0.25
0.00
0.25
MR effect size for
'HLA DR+ Natural Killer Absolute Count || id:ebi−a−GCST90001648' on 'Pneumonia || id:ieu−b−4976'

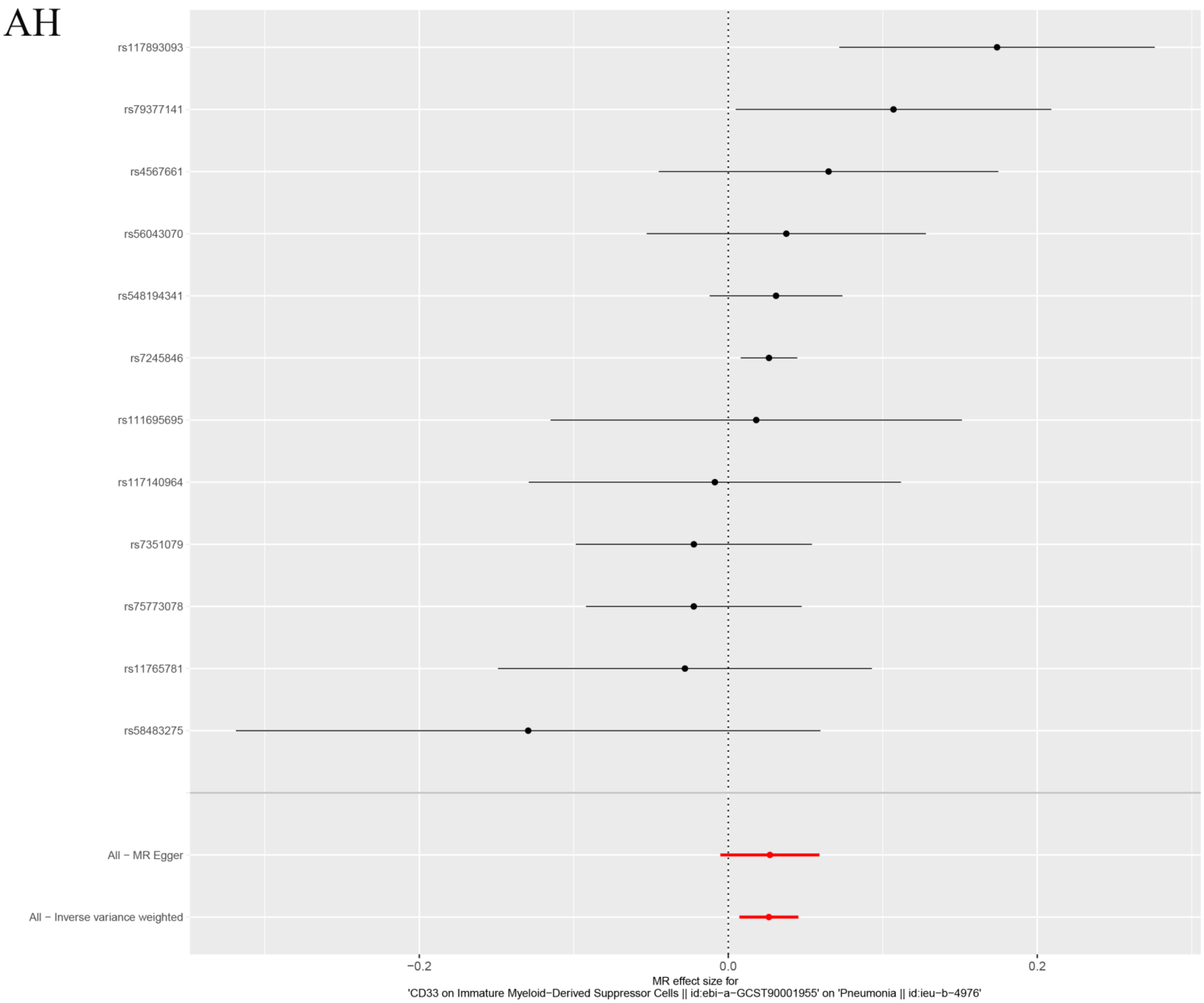
AH
rs117893093
rs79377141
rs4567661
rs56043070
rs548194341
rs7245846
rs111695695
rs117140964
rs7351079
rs75773078
rs11765781
rs58483275
All − MR Egger
All − Inverse variance weighted
−0.2
0.0
0.2
MR effect size for
'CD33 on Immature Myeloid−Derived Suppressor Cells || id:ebi−a−GCST90001955' on 'Pneumonia || id:ieu−b−4976'

AI

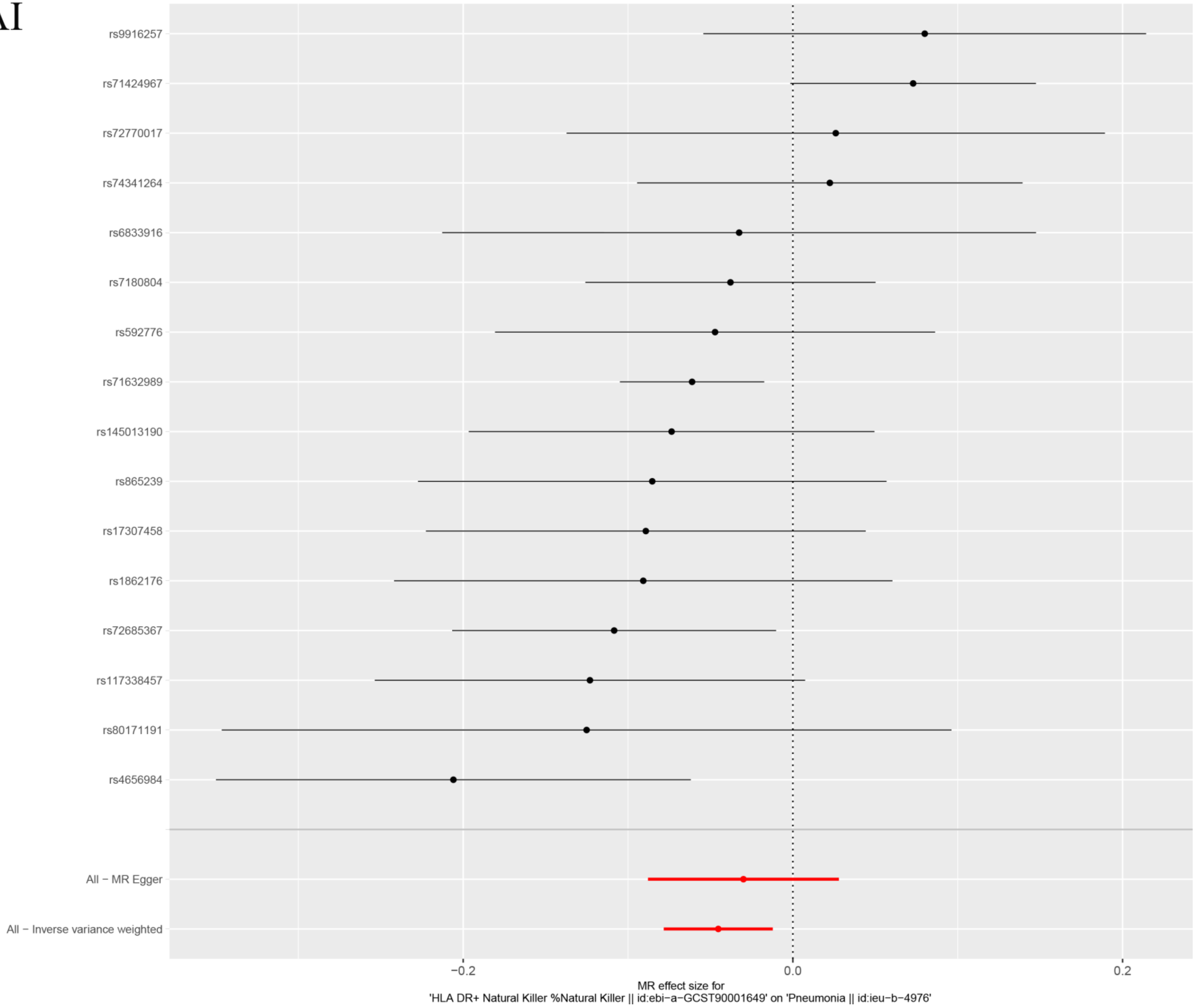

rs9916257
rs71424967
rs72770017
rs74341264
rs6833916
rs7180804
rs592776
rs71632989
rs145013190
rs865239
rs17307458
rs1862176
rs72685367
rs117338457
rs80171191
rs4656984
All − MR Egger
All − Inverse variance weighted
−0.2
0.0
0.2
MR effect size for
'HLA DR+ Natural Killer %Natural Killer || id:ebi−a−GCST90001649' on 'Pneumonia || id:ieu−b−4976'

AJ

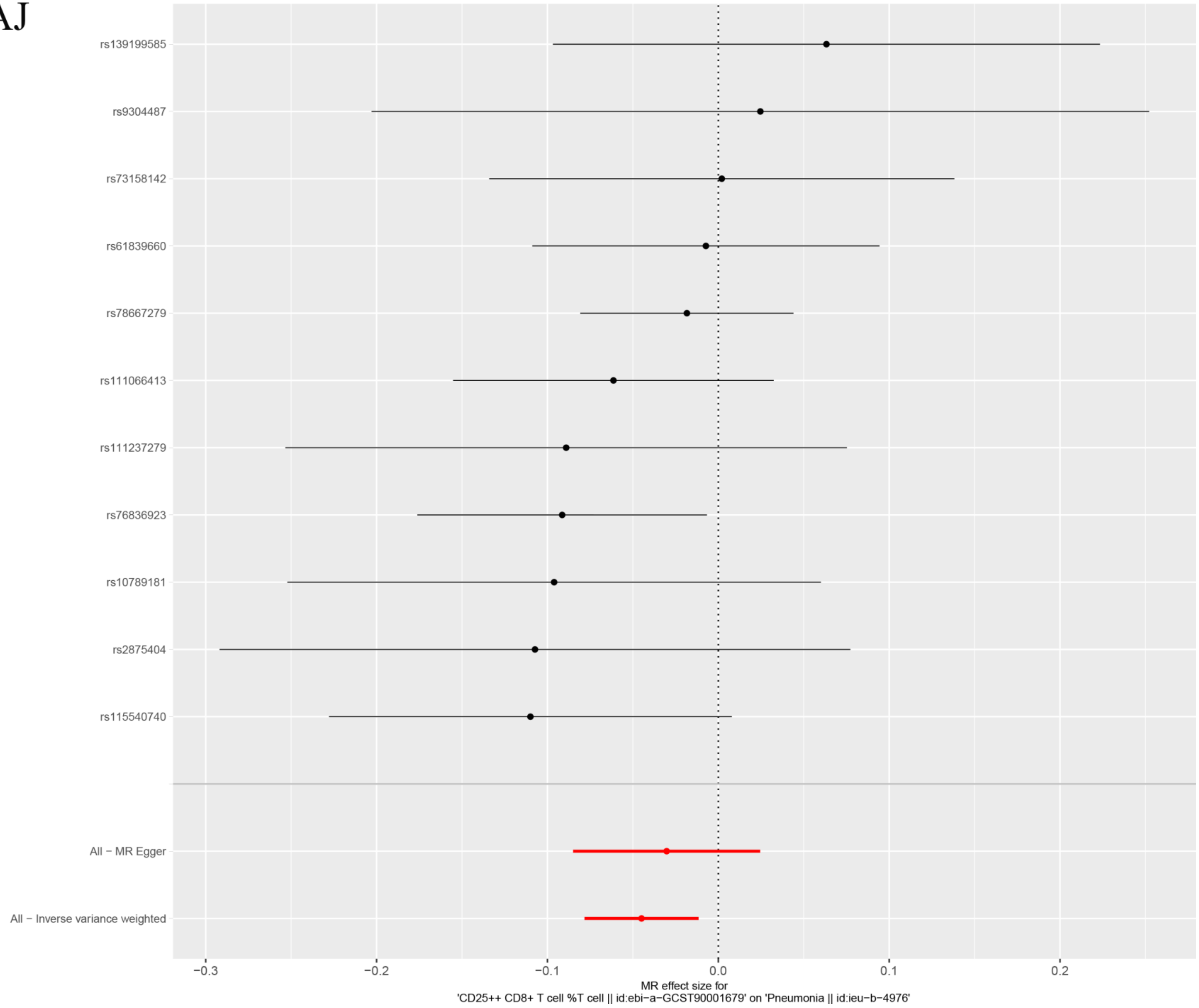

rs139199585
rs9304487
rs73158142
rs61839660
rs78667279
rs111066413
rs111237279
rs76836923
rs10789181
rs2875404
rs115540740
All − MR Egger
All − Inverse variance weighted
−0.3
−0.2
−0.1
0.0
0.1
0.2
MR effect size for
'CD25++ CD8+ T cell %T cell || id:ebi−a−GCST90001679' on 'Pneumonia || id:ieu−b−4976'

AK

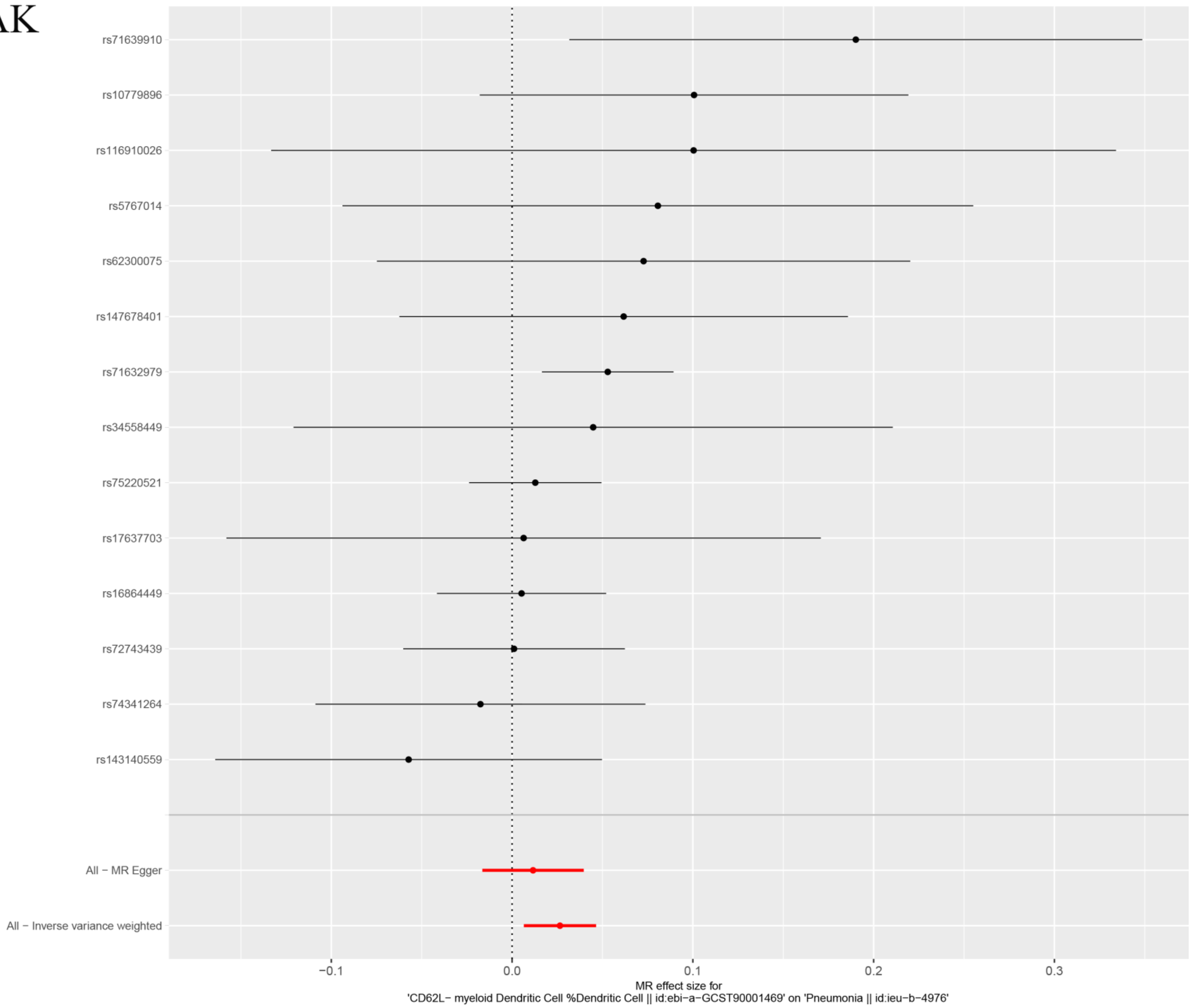
rs71639910
rs10779896
rs116910026
rs5767014
rs62300075
rs147678401
rs71632979
rs34558449
rs75220521
rs17637703
rs16864449
rs72743439
rs74341264
rs143140559
All − MR Egger
All − Inverse variance weighted
−0.1
0.0
0.1
0.2
0.3
MR effect size for
'CD62L− myeloid Dendritic Cell %Dendritic Cell || id:ebi−a−GCST90001469' on 'Pneumonia || id:ieu−b−4976'

AL

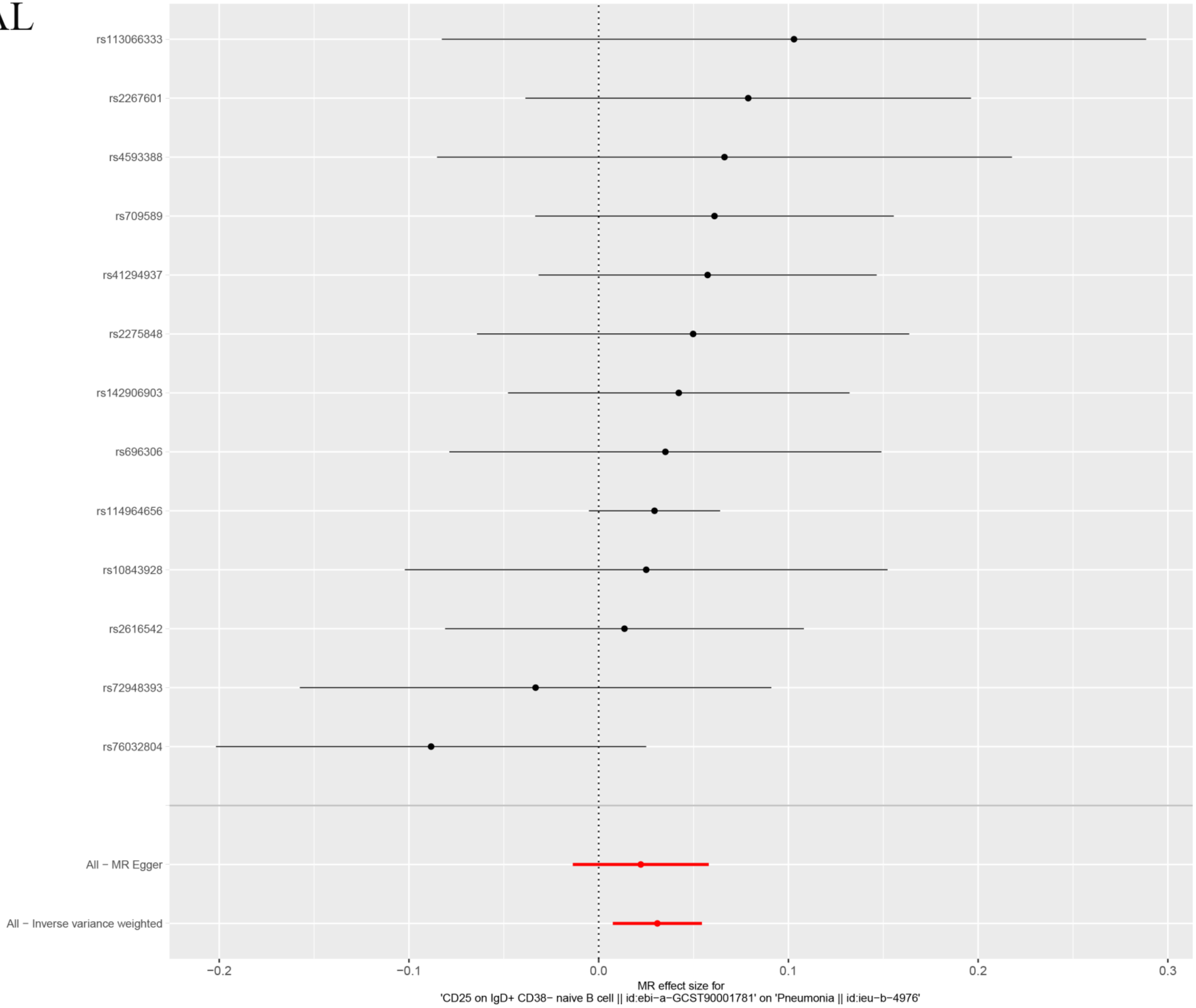
rs113066333
rs2267601
rs4593388
rs709589
rs41294937
rs2275848
rs142906903
rs696306
rs114964656
rs10843928
rs2616542
rs72948393
rs76032804
All − MR Egger
All − Inverse variance weighted
−0.2
−0.1
0.0
0.1
0.2
0.3
MR effect size for
'CD25 on IgD+ CD38− naive B cell || id:ebi−a−GCST90001781' on 'Pneumonia || id:ieu−b−4976'

AM

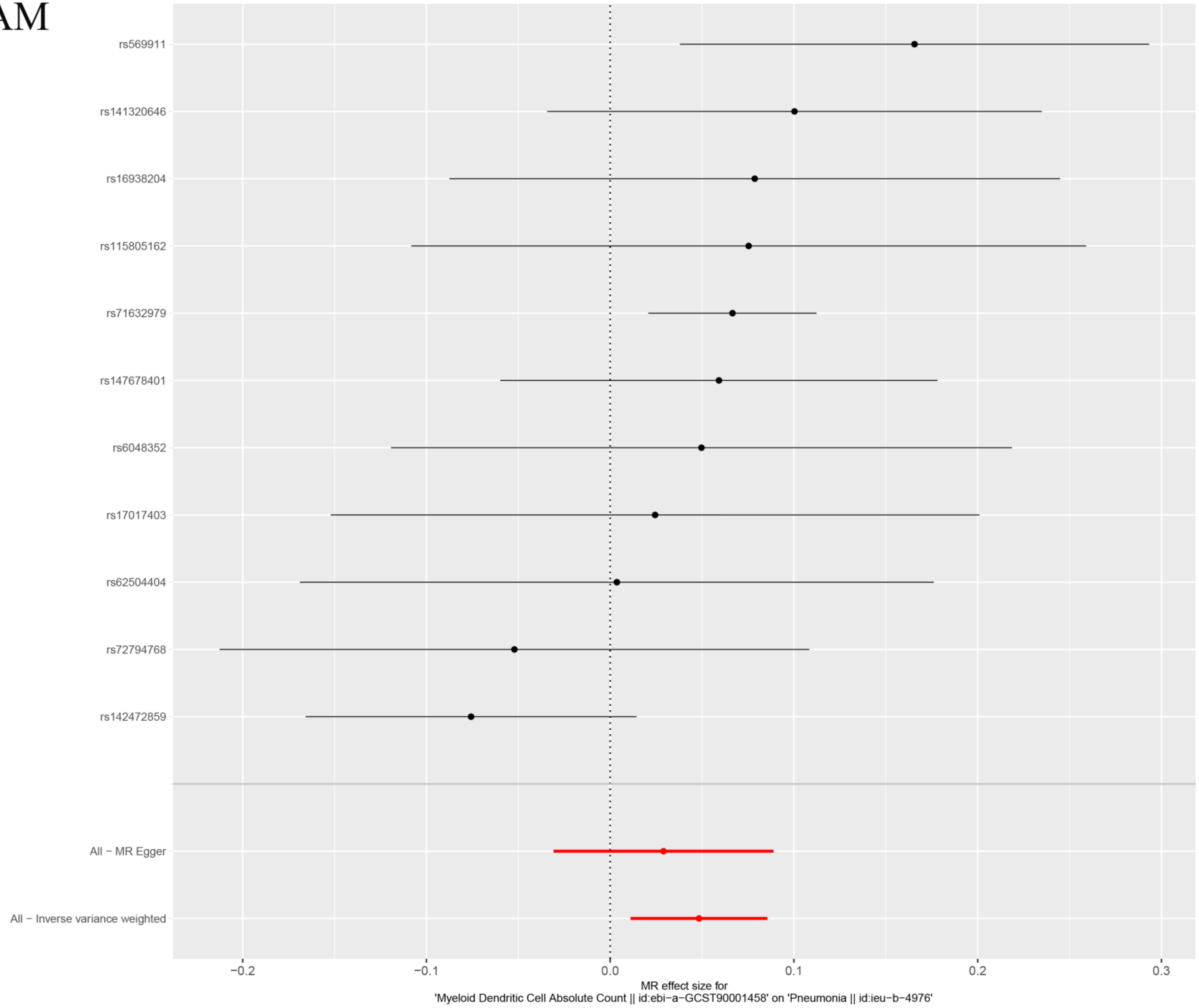

rs569911
rs141320646
rs16938204
rs115805162
rs71632979
rs147678401
rs6048352
rs17017403
rs62504404
rs72794768
rs142472859
All − MR Egger
All − Inverse variance weighted
−0.2
−0.1
0.0
0.1
0.2
0.3
MR effect size for
'Myeloid Dendritic Cell Absolute Count || id:ebi−a−GCST90001458' on 'Pneumonia || id:ieu−b−4976'

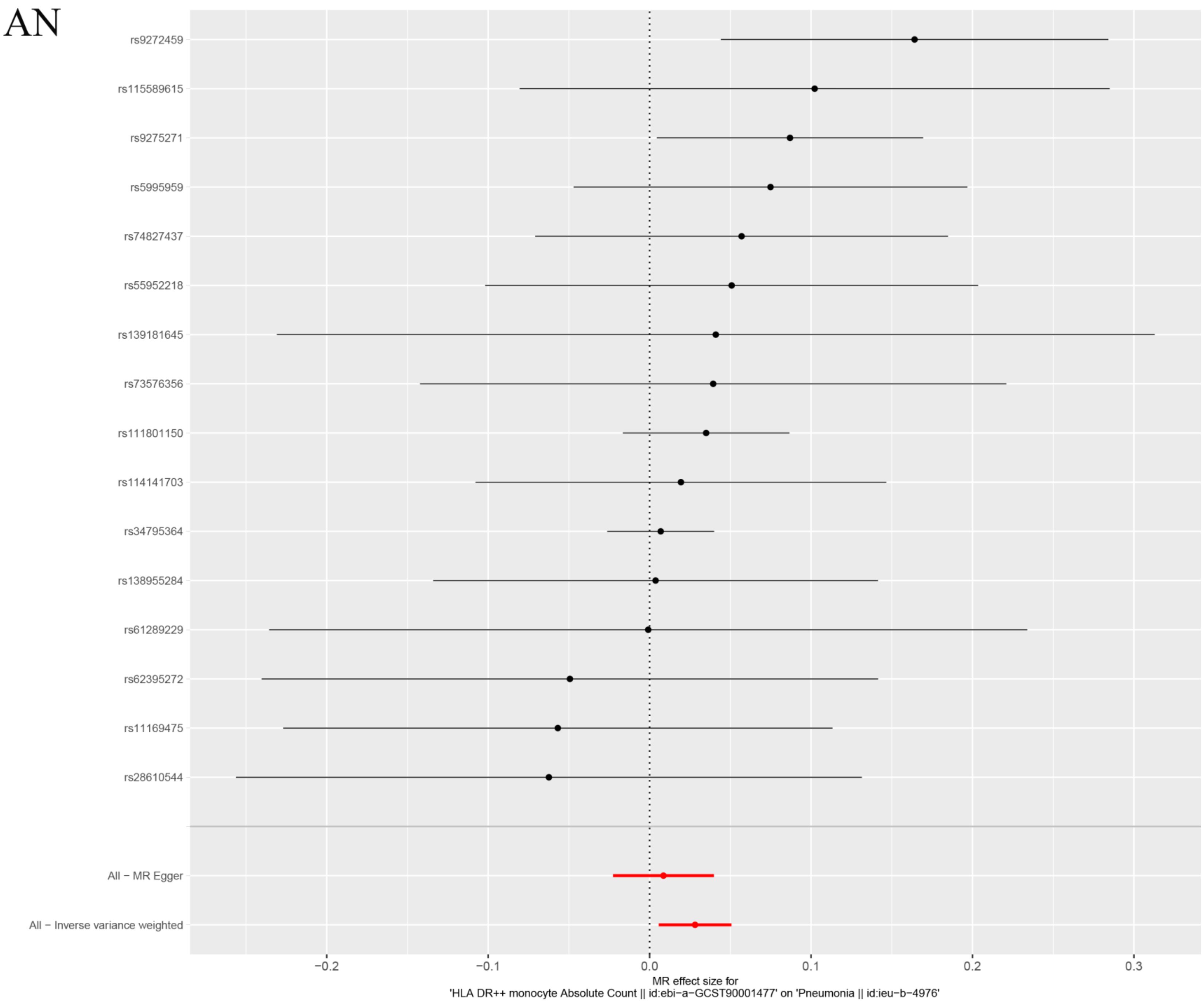
AN
rs9272459
rs115589615
rs9275271
rs5995959
rs74827437
rs55952218
rs139181645
rs73576356
rs111801150
rs114141703
rs34795364
rs138955284
rs61289229
rs62395272
rs11169475
rs28610544
All − MR Egger
All − Inverse variance weighted
−0.2
−0.1
0.0
0.1
0.2
0.3
MR effect size for
'HLA DR++ monocyte Absolute Count || id:ebi−a−GCST90001477' on 'Pneumonia || id:ieu−b−4976'

AO

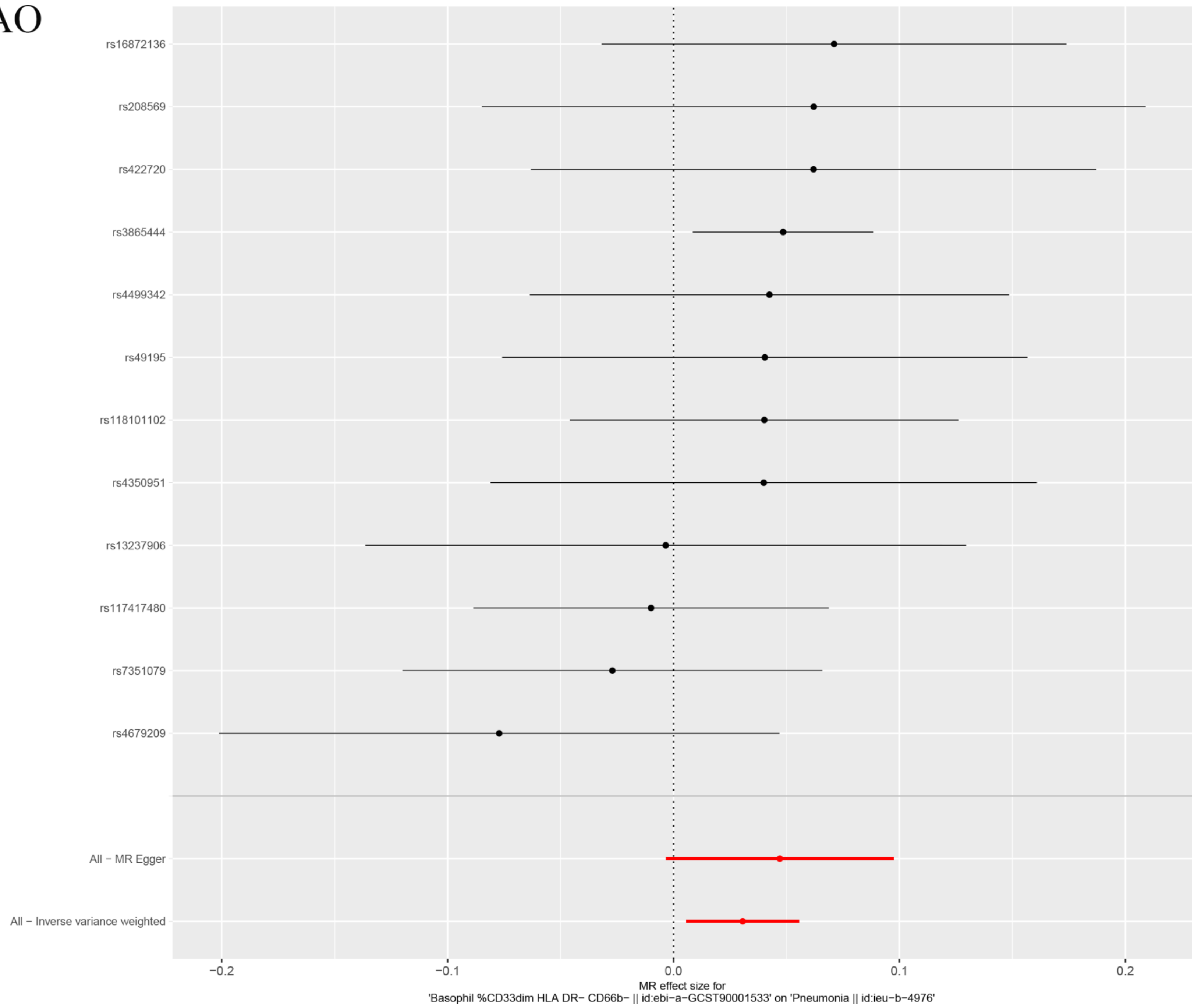
rs16872136
rs208569
rs422720
rs3865444
rs4499342
rs49195
rs118101102
rs4350951
rs13237906
rs117417480
rs7351079
rs4679209
All − MR Egger
All − Inverse variance weighted
−0.2
−0.1
0.0
0.1
0.2
MR effect size for
'Basophil %CD33dim HLA DR− CD66b− || id:ebi−a−GCST90001533' on 'Pneumonia || id:ieu−b−4976'

AP

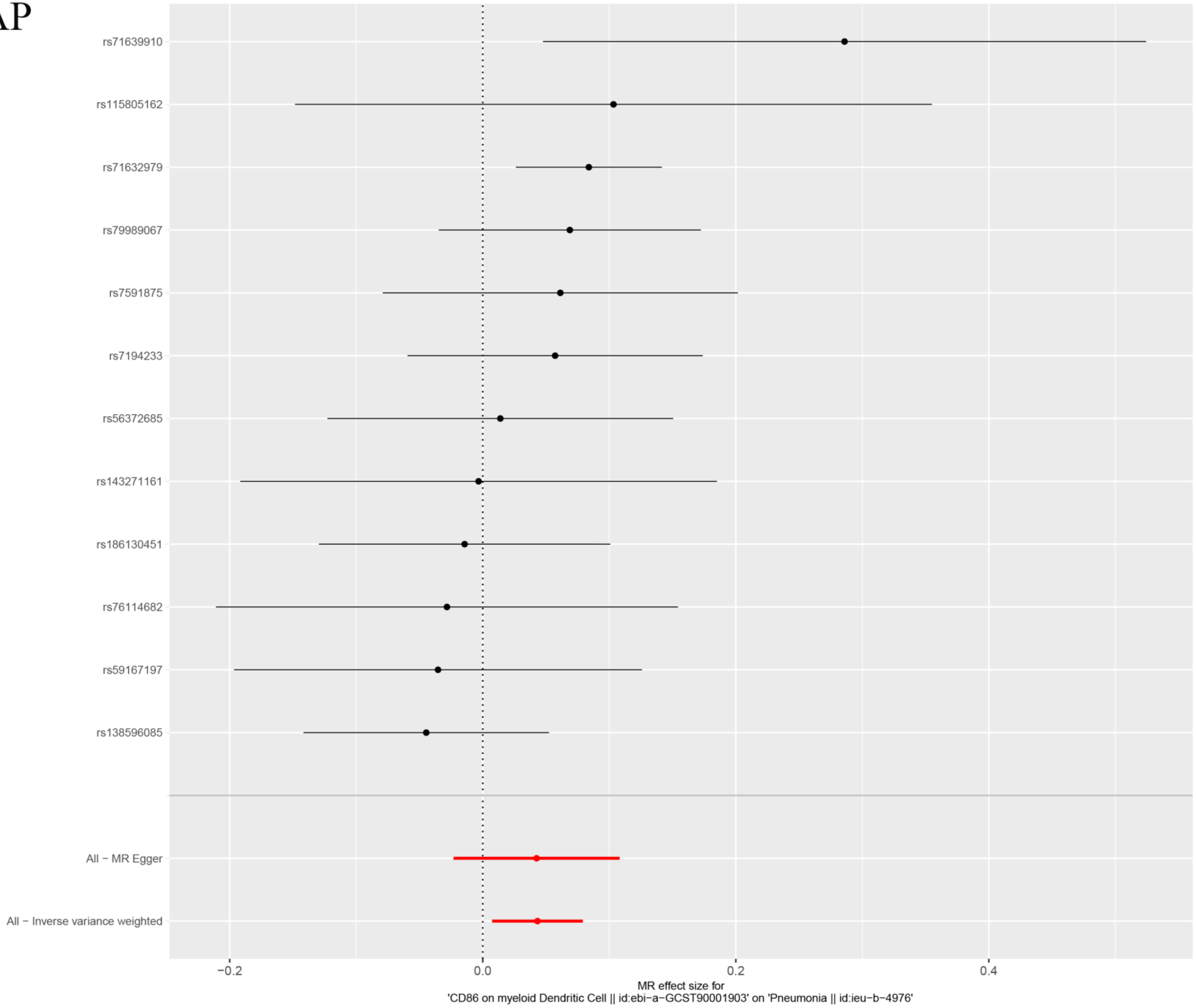

rs71639910
rs115805162
rs71632979
rs79989067
rs7591875
rs7194233
rs56372685
rs143271161
rs186130451
rs76114682
rs59167197
rs138596085
All − MR Egger
All − Inverse variance weighted
−0.2
0.0
0.2
0.4
MR effect size for
'CD86 on myeloid Dendritic Cell || id:ebi−a−GCST90001903' on 'Pneumonia || id:ieu−b−4976'

AQ

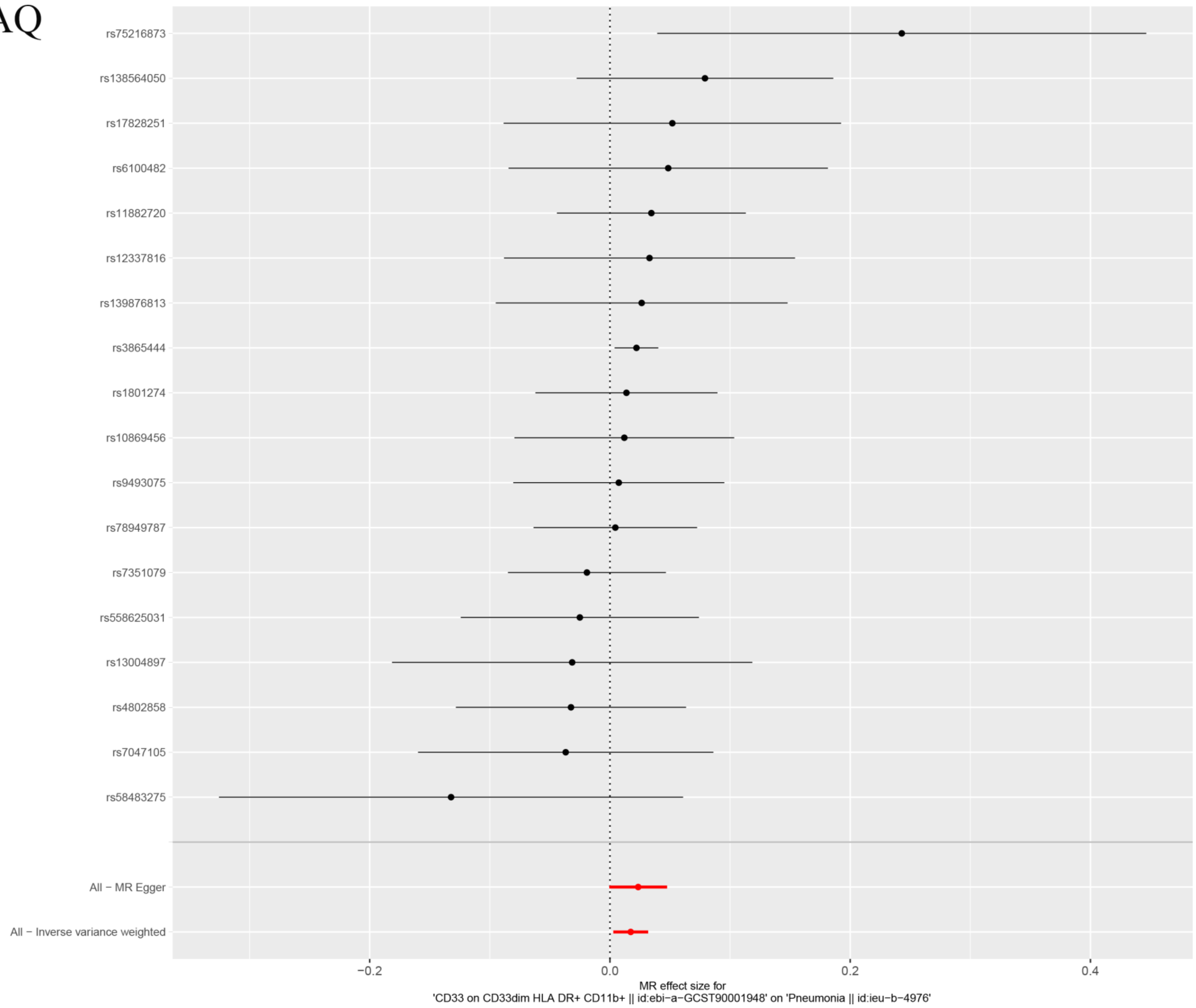
rs75216873
rs138564050
rs17828251
rs6100482
rs11882720
rs12337816
rs139876813
rs3865444
rs1801274
rs10869456
rs9493075
rs78949787
rs7351079
rs558625031
rs13004897
rs4802858
rs7047105
rs58483275
All − MR Egger
All − Inverse variance weighted
−0.2
0.0
0.2
0.4
MR effect size for
'CD33 on CD33dim HLA DR+ CD11b+ || id:ebi−a−GCST90001948' on 'Pneumonia || id:ieu−b−4976'

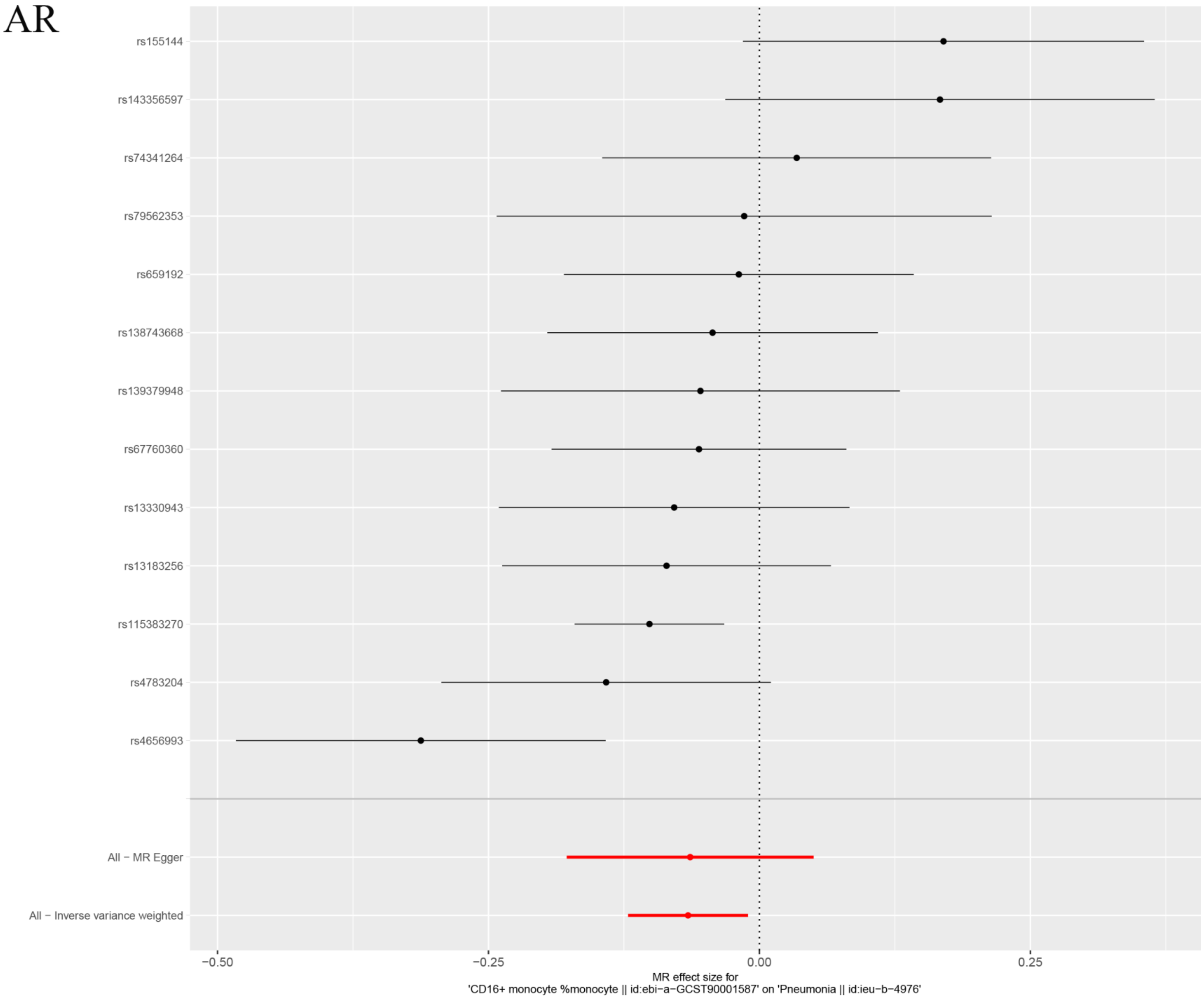

AR
rs155144
rs143356597
rs74341264
rs79562353
rs659192
rs138743668
rs139379948
rs67760360
rs13330943
rs13183256
rs115383270
rs4783204
rs4656993
All − MR Egger
All − Inverse variance weighted
−0.50
−0.25
0.00
0.25
MR effect size for
'CD16+ monocyte %monocyte || id:ebi−a−GCST90001587' on 'Pneumonia || id:ieu−b−4976'

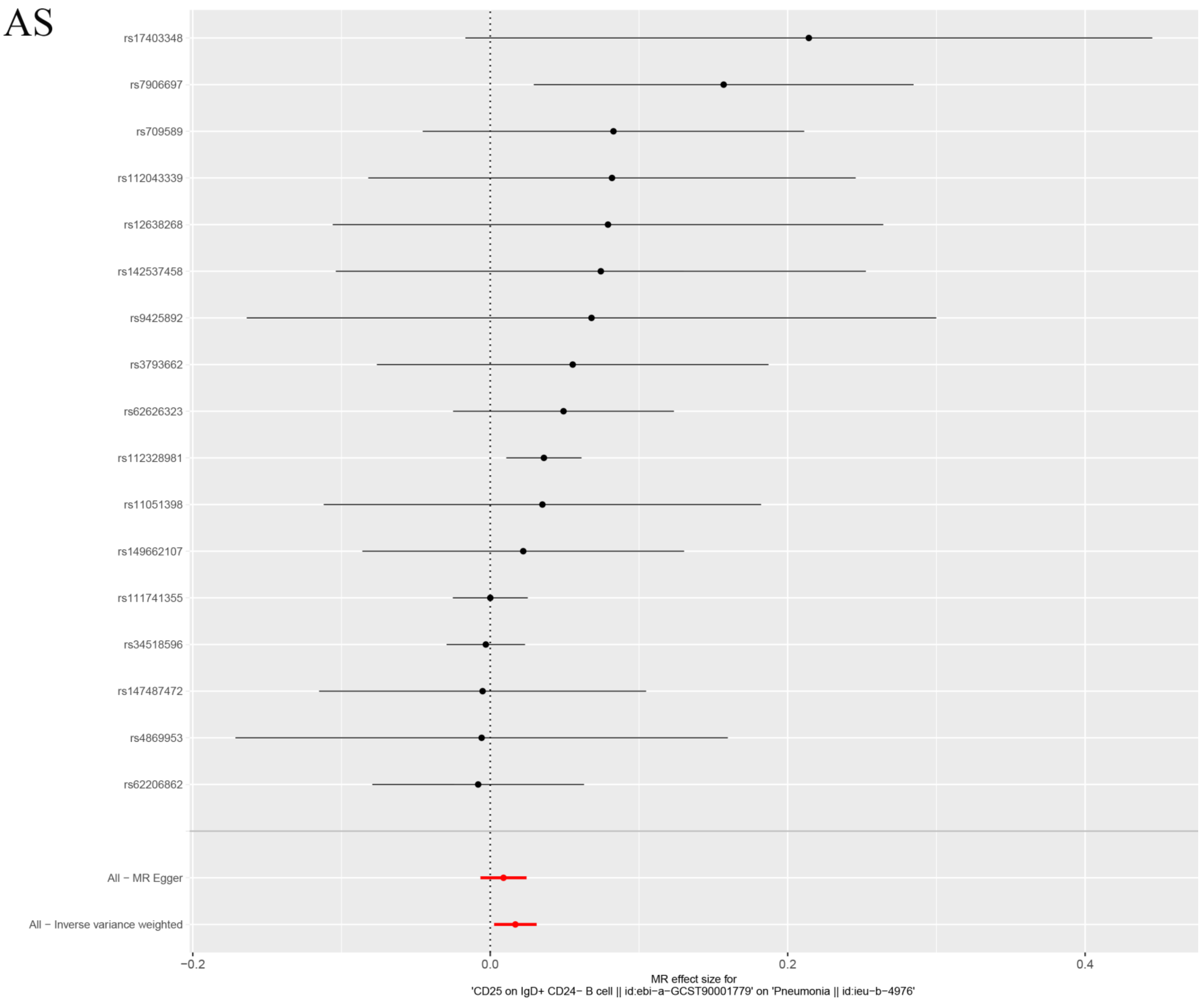
AS
rs17403348
rs7906697
rs709589
rs112043339
rs12638268
rs142537458
rs9425892
rs3793662
rs62626323
rs112328981
rs11051398
rs149662107
rs111741355
rs34518596
rs147487472
rs4869953
rs62206862
All − MR Egger
All − Inverse variance weighted
−0.2
0.0
0.2
0.4
MR effect size for
'CD25 on IgD+ CD24− B cell || id:ebi−a−GCST90001779' on 'Pneumonia || id:ieu−b−4976'

AT

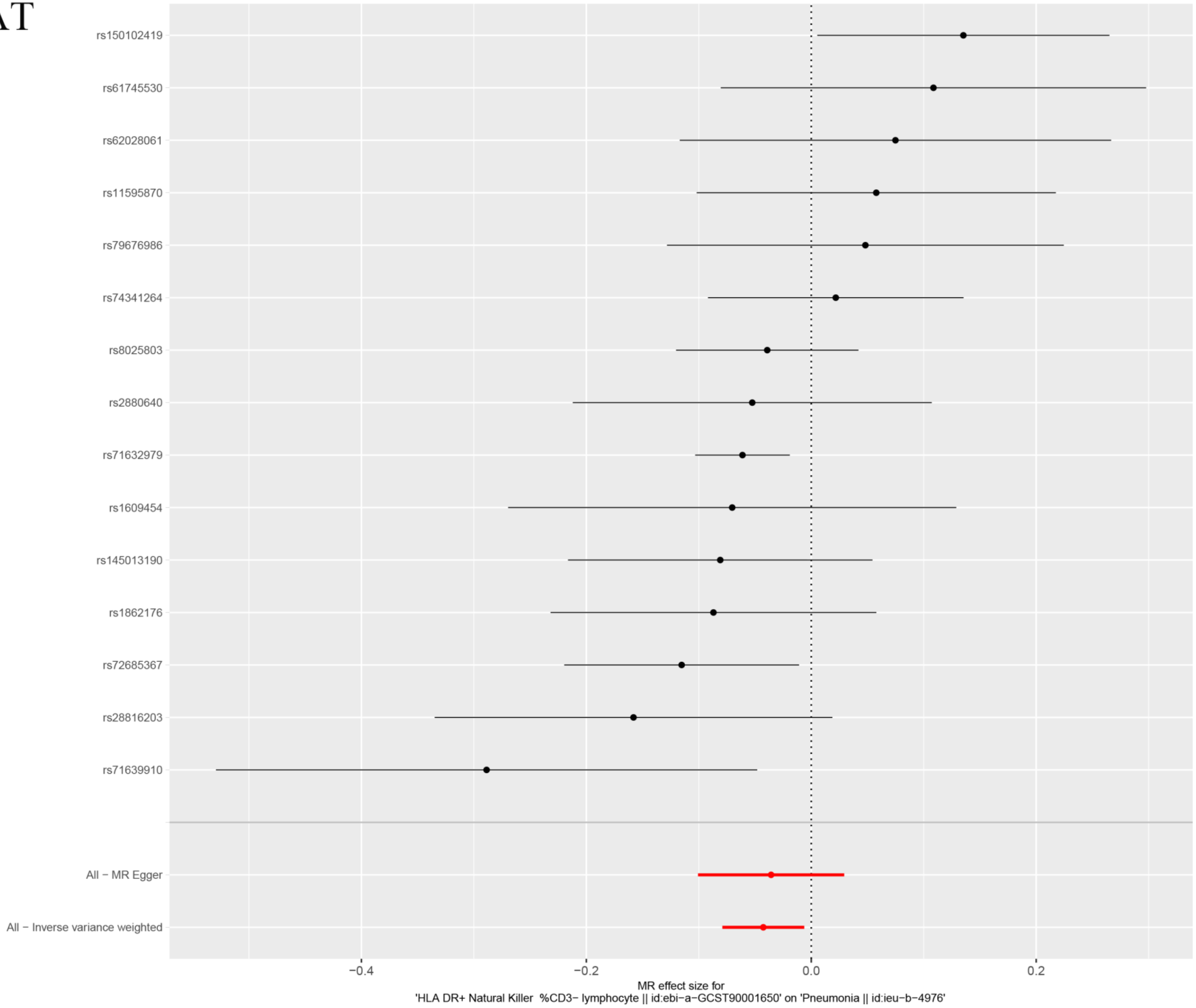

rs150102419
rs61745530
rs62028061
rs11595870
rs79676986
rs74341264
rs8025803
rs2880640
rs71632979
rs1609454
rs145013190
rs1862176
rs72685367
rs28816203
rs71639910
All − MR Egger
All − Inverse variance weighted
−0.4
−0.2
0.0
0.2
MR effect size for
'HLA DR+ Natural Killer %CD3− lymphocyte || id:ebi−a−GCST90001650' on 'Pneumonia || id:ieu−b−4976'

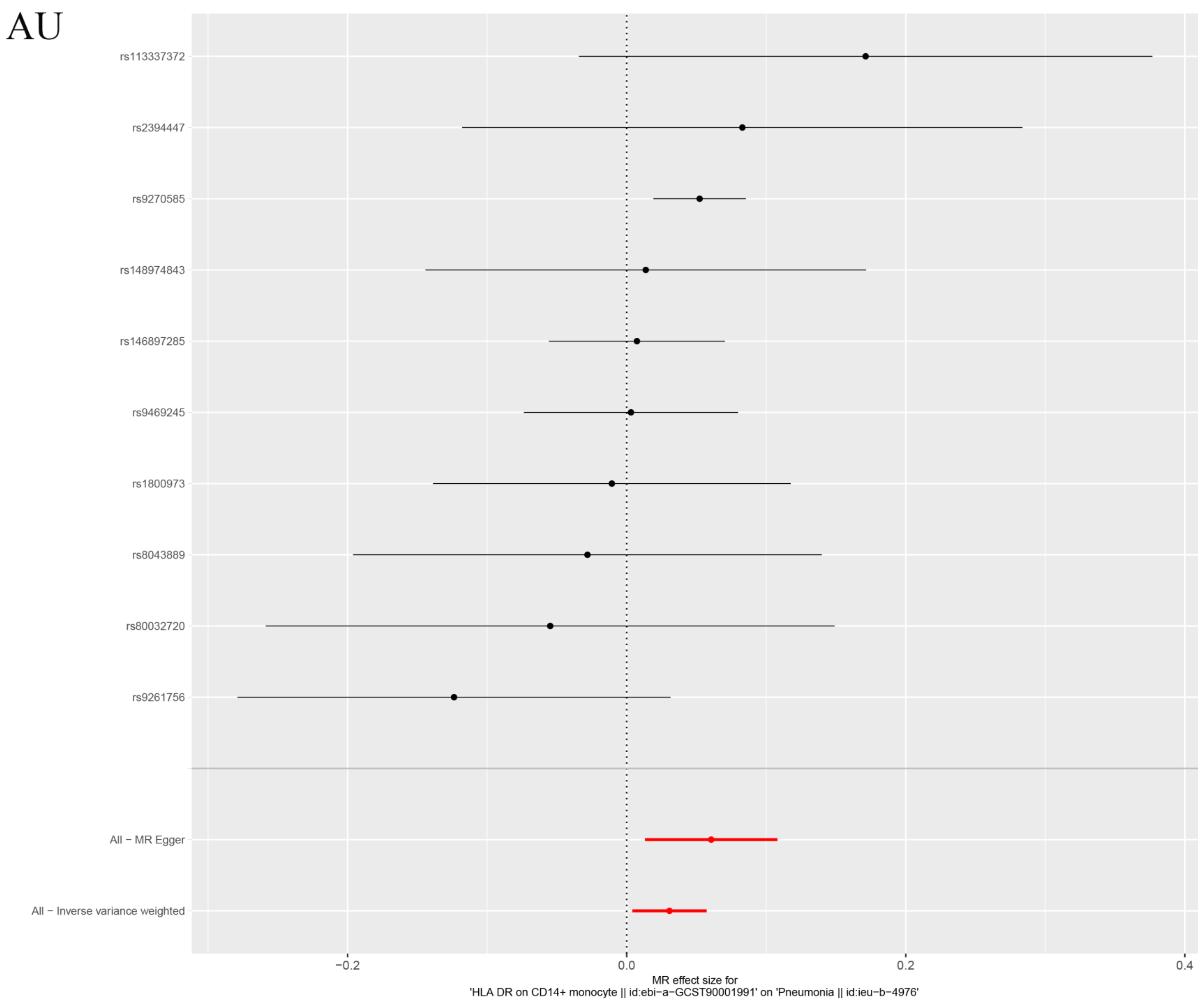

AU
rs113337372
rs2394447
rs9270585
rs148974843
rs146897285
rs9469245
rs1800973
rs8043889
rs80032720
rs9261756
All − MR Egger
All − Inverse variance weighted
−0.2
0.0
0.2
0.4
MR effect size for
'HLA DR on CD14+ monocyte || id:ebi−a−GCST90001991' on 'Pneumonia || id:ieu−b−4976'

AV

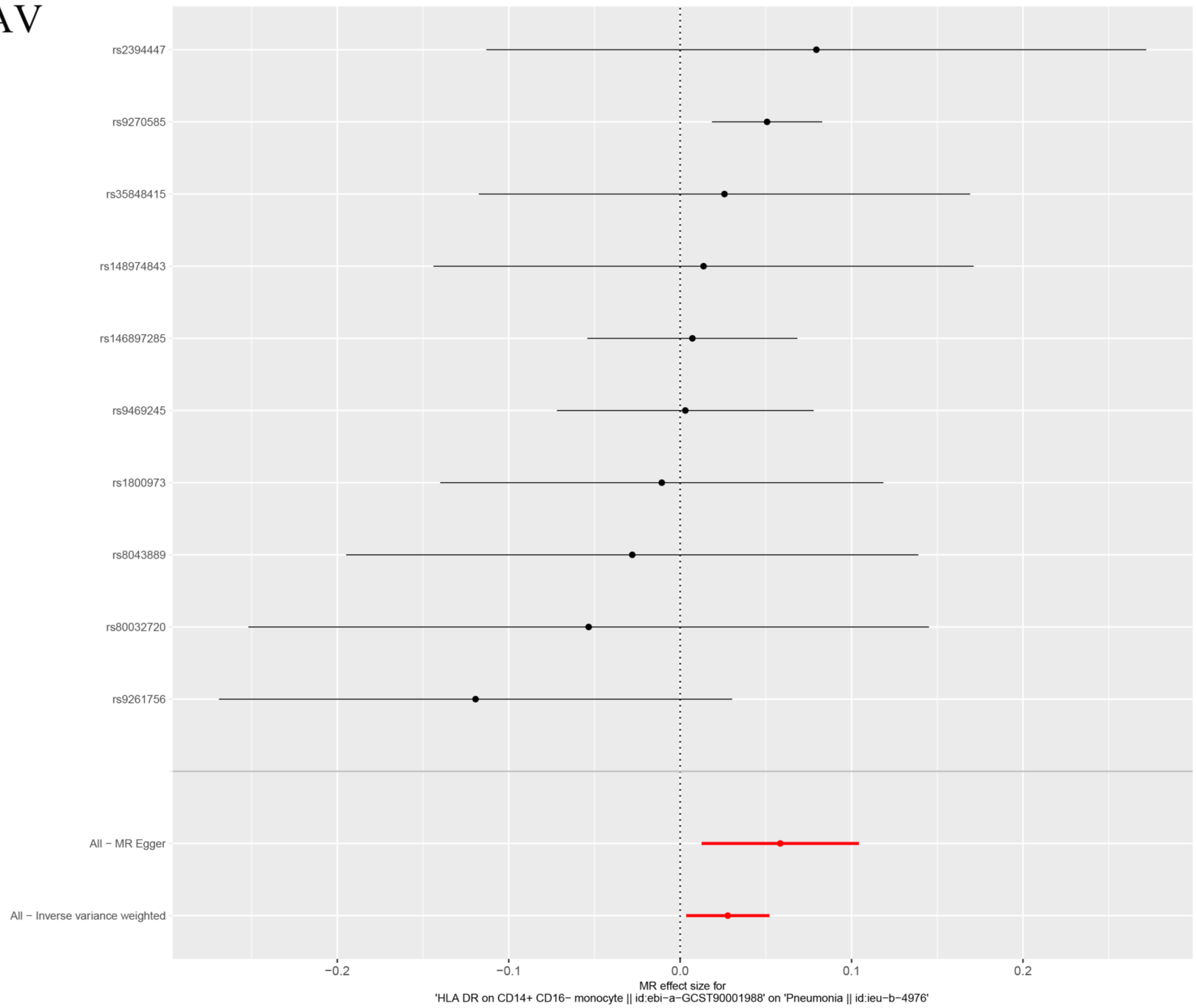
rs2394447
rs9270585
rs35848415
rs148974843
rs146897285
rs9469245
rs1800973
rs8043889
rs80032720
rs9261756
All − MR Egger
All − Inverse variance weighted
−0.2
−0.1
0.0
0.1
0.2
MR effect size for
'HLA DR on CD14+ CD16− monocyte || id:ebi−a−GCST90001988' on 'Pneumonia || id:ieu−b−4976'

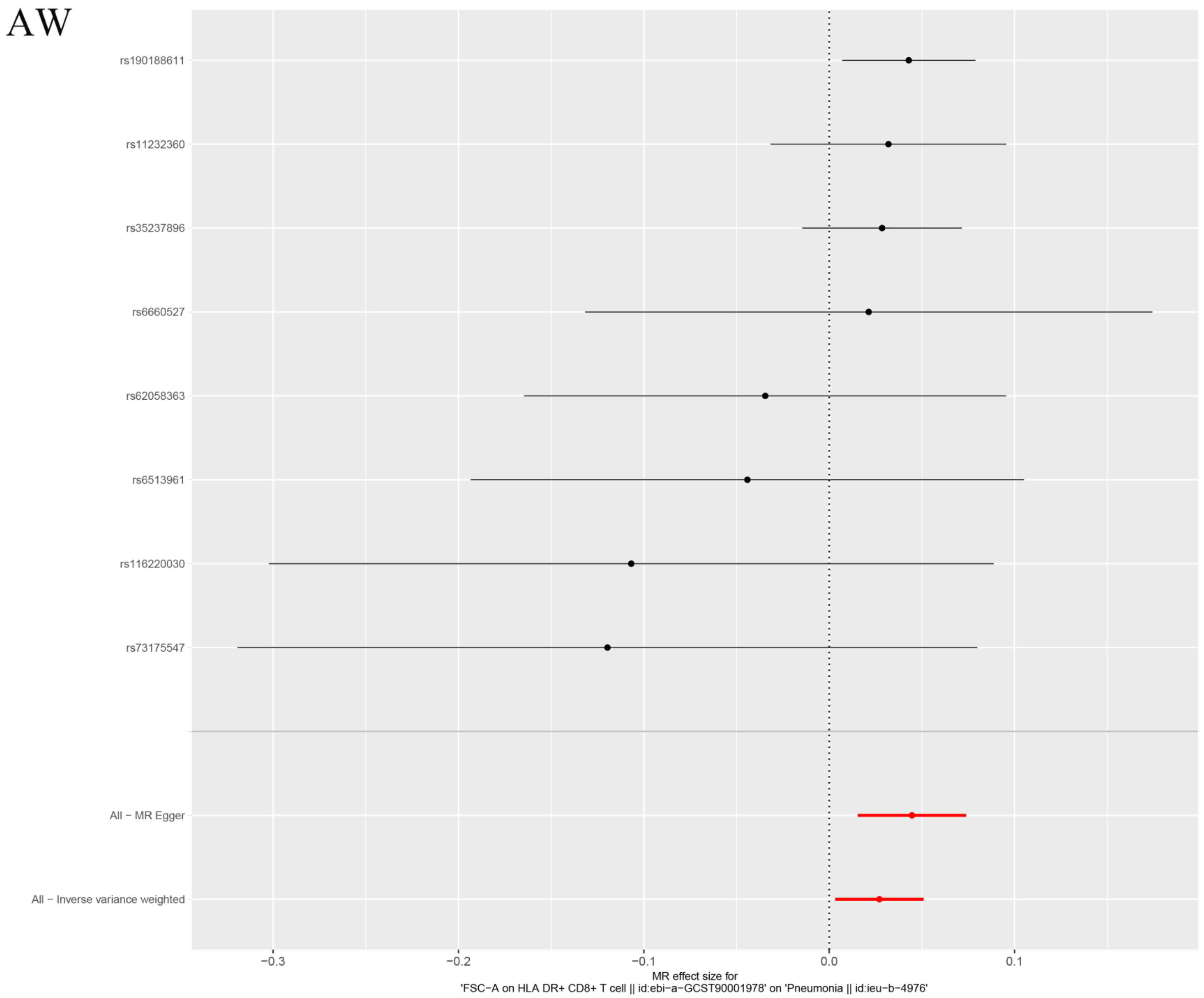
AW
rs190188611
rs11232360
rs35237896
rs6660527
rs62058363
rs6513961
rs116220030
rs73175547
All − MR Egger
All − Inverse variance weighted
−0.3
−0.2
−0.1
0.0
0.1
MR effect size for
'FSC−A on HLA DR+ CD8+ T cell || id:ebi−a−GCST90001978' on 'Pneumonia || id:ieu−b−4976'

AX

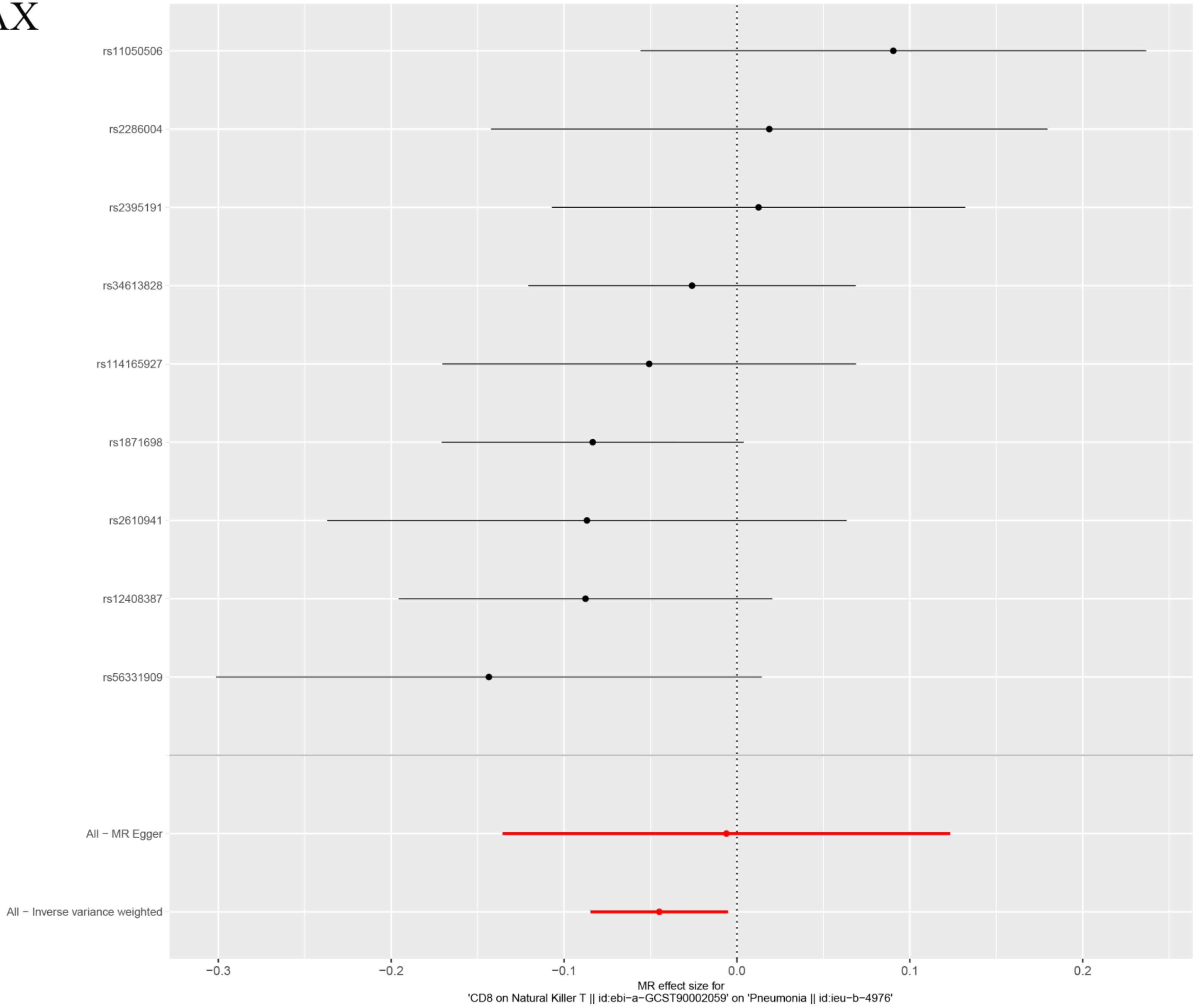

rs11050506
rs2286004
rs2395191
rs34613828
rs114165927
rs1871698
rs2610941
rs12408387
rs56331909
All − MR Egger
All − Inverse variance weighted
−0.3
−0.2
−0.1
0.0
0.1
0.2
MR effect size for
'CD8 on Natural Killer T || id:ebi−a−GCST90002059' on 'Pneumonia || id:ieu−b−4976'

AY

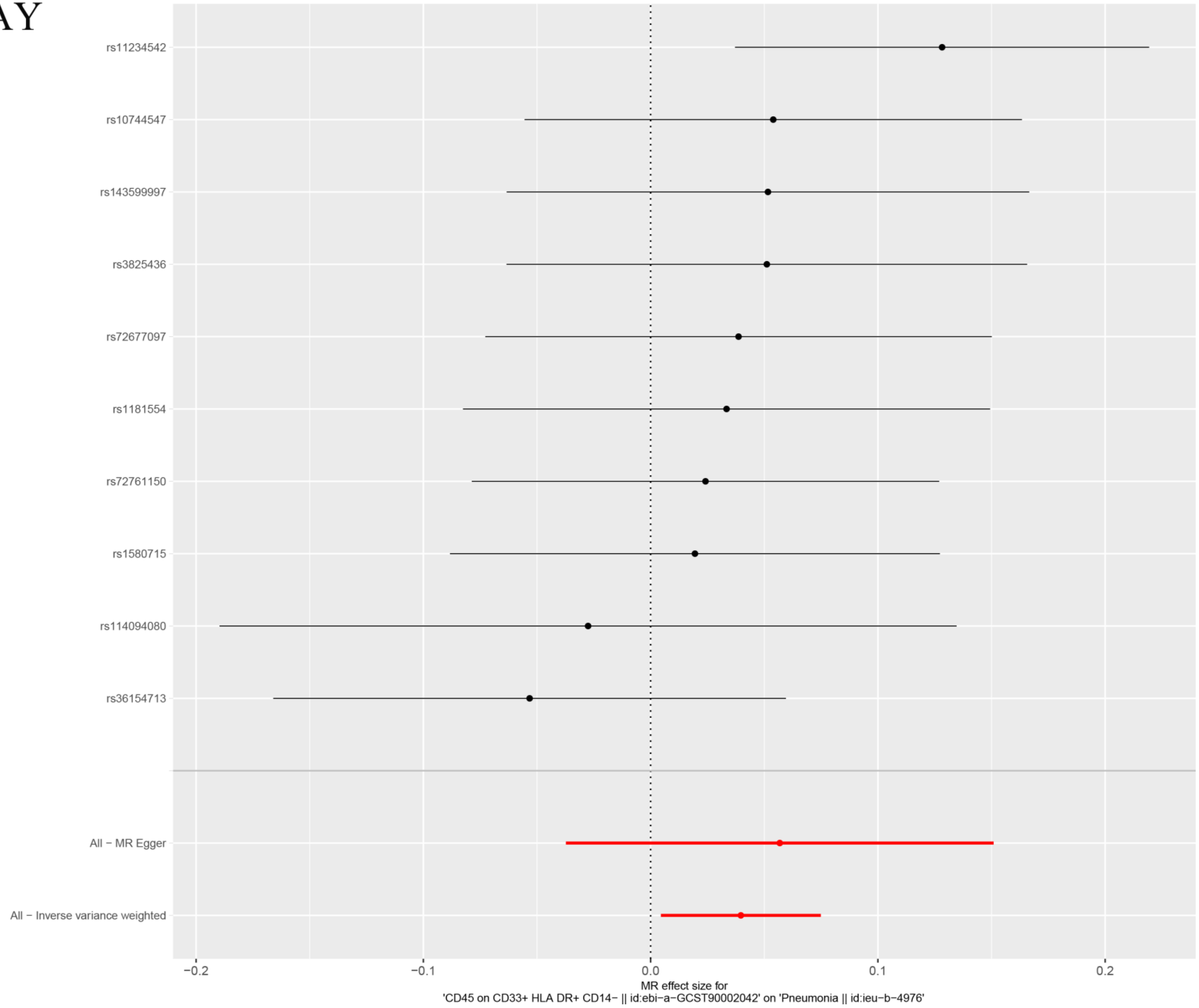

rs11234542
rs10744547
rs143599997
rs3825436
rs72677097
rs1181554
rs72761150
rs1580715
rs114094080
rs36154713
All − MR Egger
All − Inverse variance weighted
−0.2
−0.1
0.0
0.1
0.2
MR effect size for
'CD45 on CD33+ HLA DR+ CD14− || id:ebi−a−GCST90002042' on 'Pneumonia || id:ieu−b−4976'

AZ

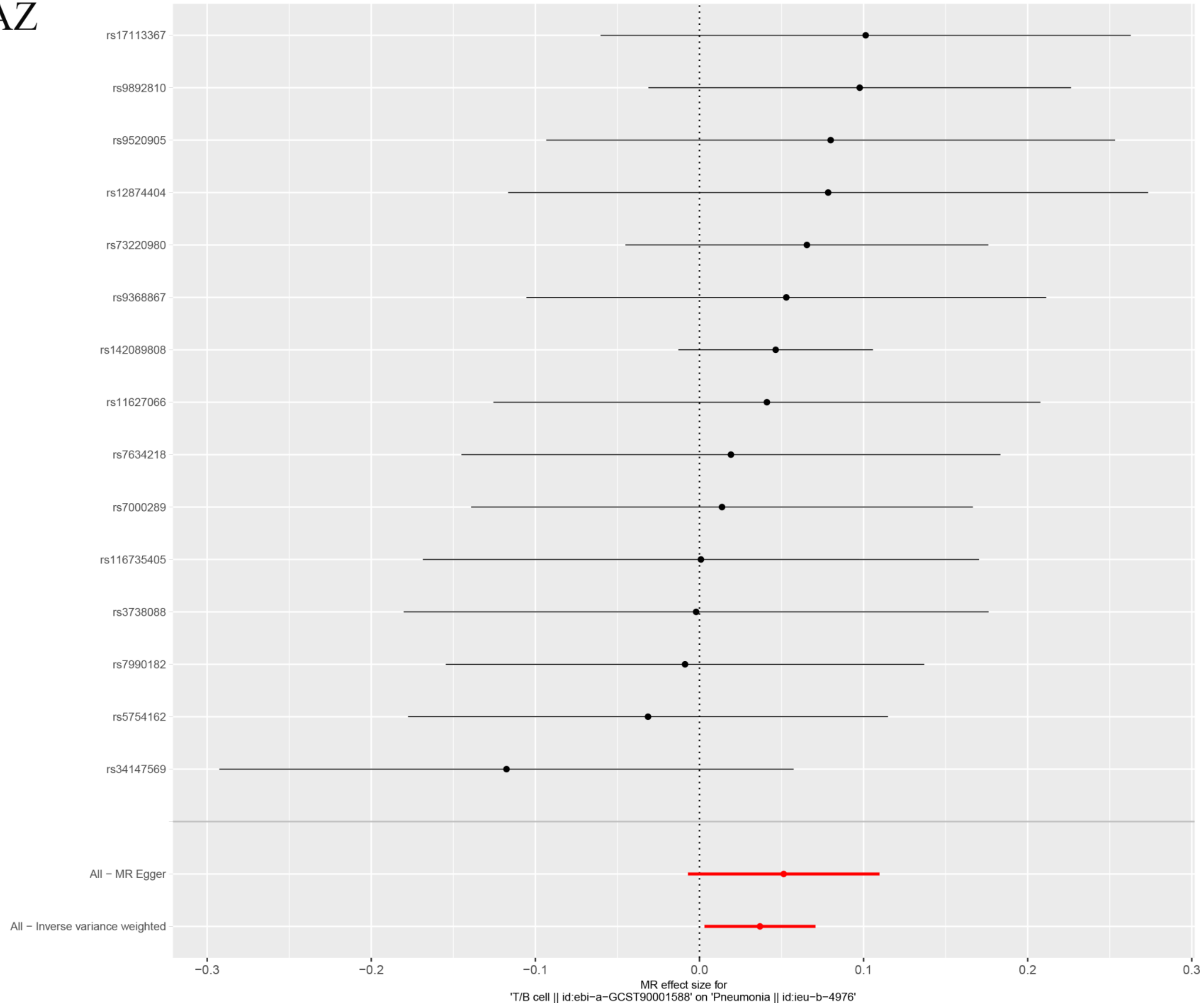

rs17113367
rs9892810
rs9520905
rs12874404
rs73220980
rs9368867
rs142089808
rs11627066
rs7634218
rs7000289
rs116735405
rs3738088
rs7990182
rs5754162
rs34147569
All − MR Egger
All − Inverse variance weighted
−0.3
−0.2
−0.1
0.0
0.1
0.2
0.3
MR effect size for
'T/B cell || id:ebi−a−GCST90001588' on 'Pneumonia || id:ieu−b−4976'

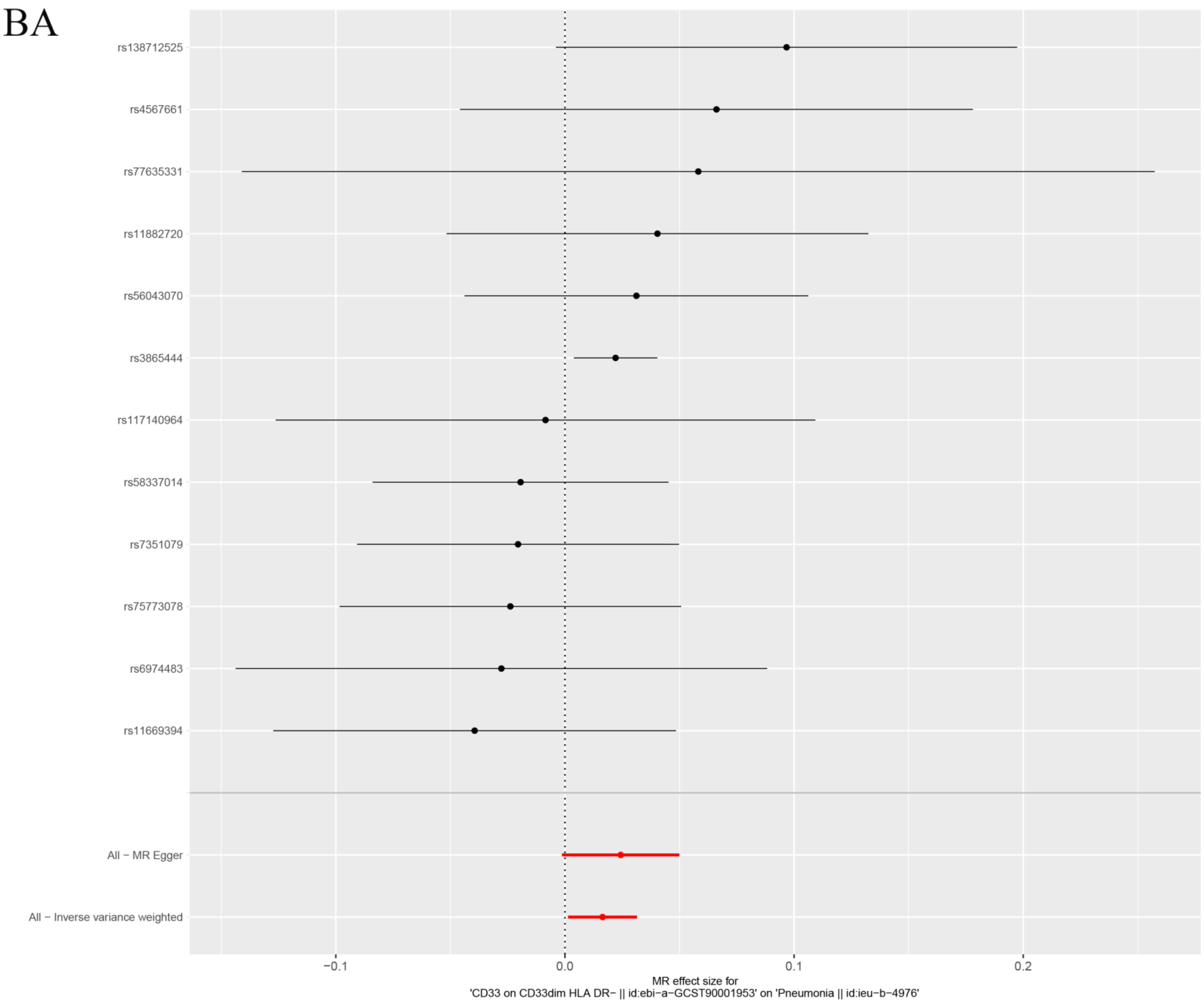

BA
rs138712525
rs4567661
rs77635331
rs11882720
rs56043070
rs3865444
rs117140964
rs58337014
rs7351079
rs75773078
rs6974483
rs11669394
All − MR Egger
All − Inverse variance weighted
−0.1
0.0
0.1
0.2
MR effect size for
'CD33 on CD33dim HLA DR− || id:ebi−a−GCST90001953' on 'Pneumonia || id:ieu−b−4976'

BB

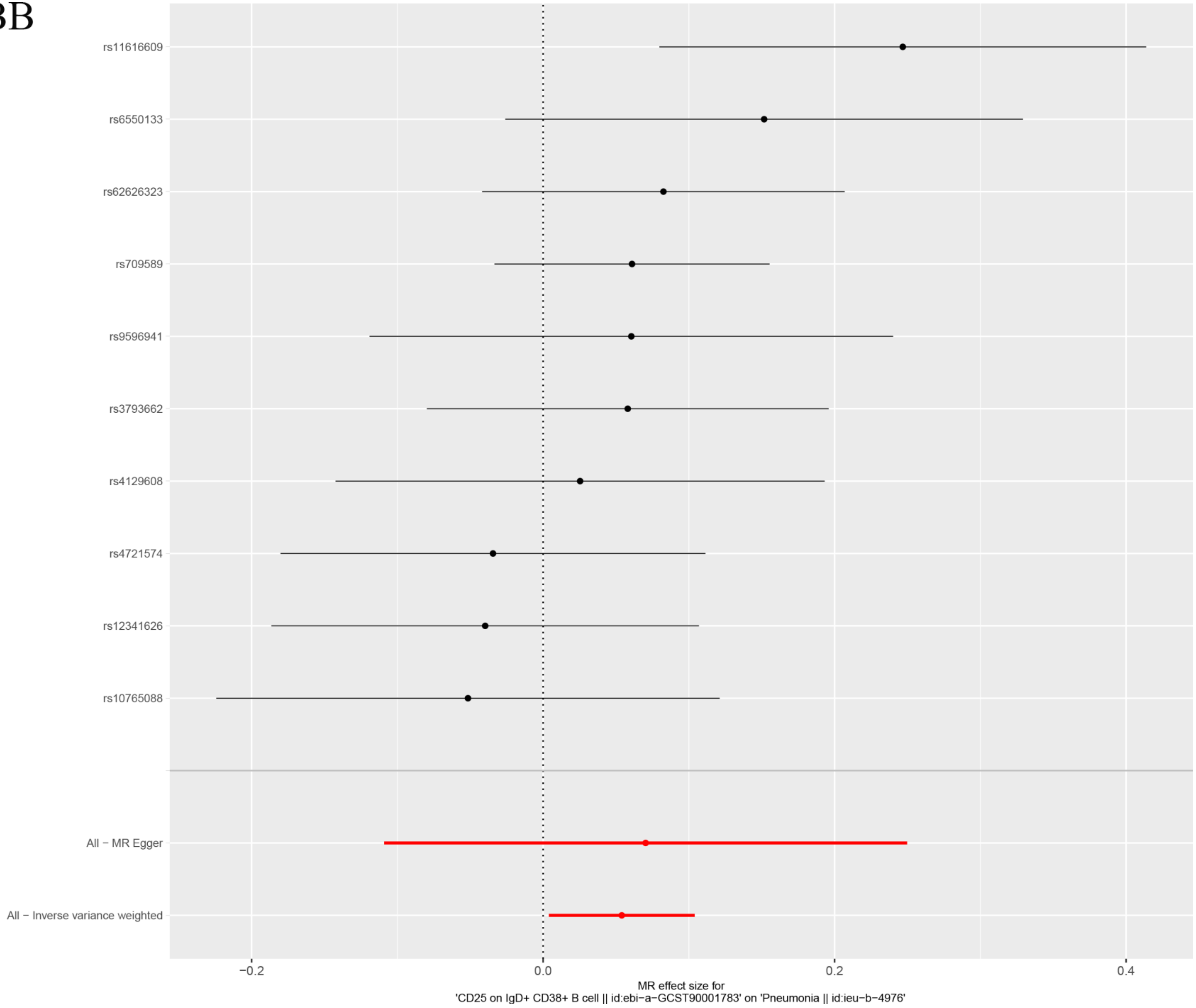
rs11616609
rs6550133
rs62626323
rs709589
rs9596941
rs3793662
rs4129608
rs4721574
rs12341626
rs10765088
All − MR Egger
All − Inverse variance weighted
−0.2
0.0
0.2
0.4
MR effect size for
'CD25 on IgD+ CD38+ B cell || id:ebi−a−GCST90001783' on 'Pneumonia || id:ieu−b−4976'

BC

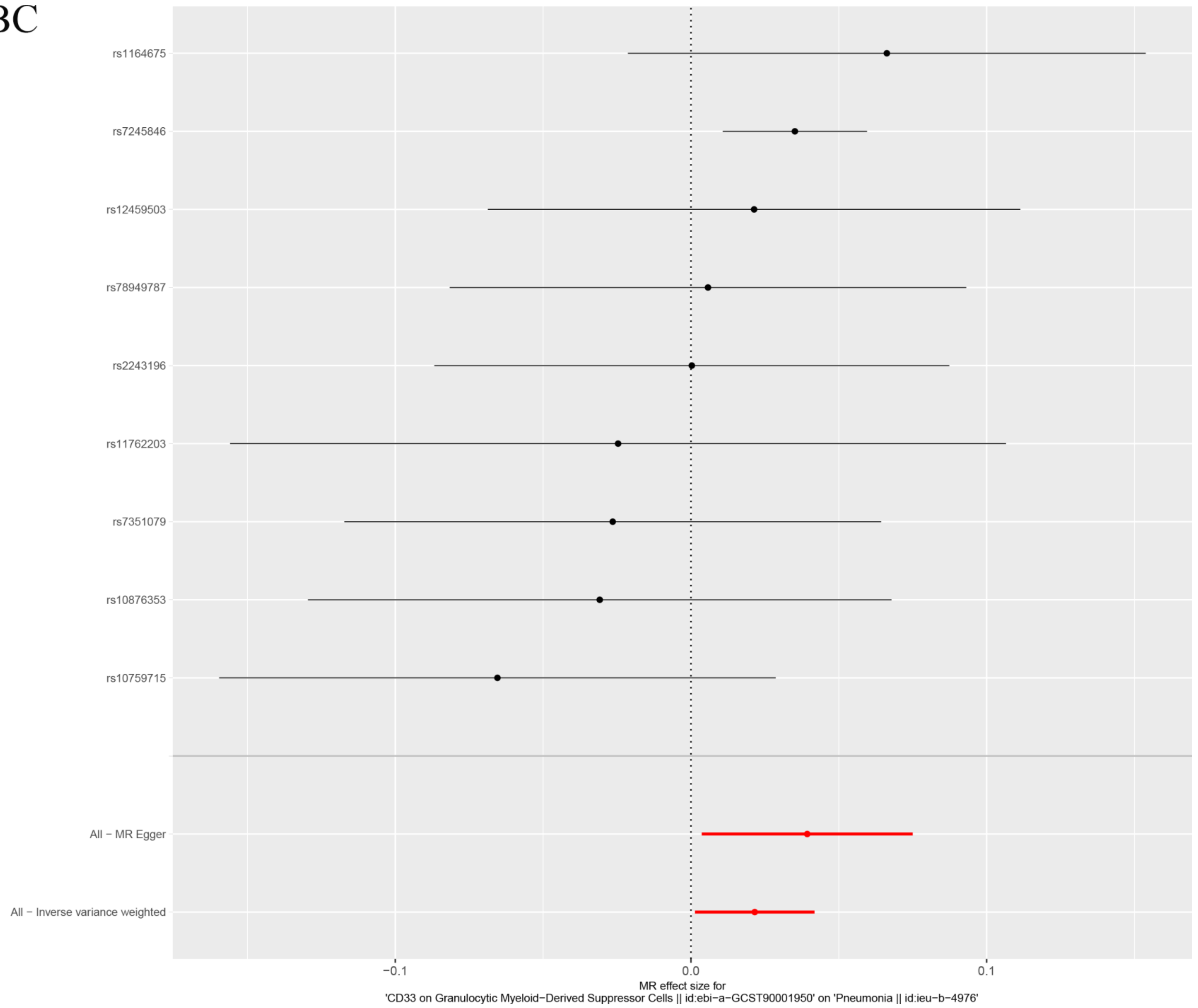

rs1164675
rs7245846
rs12459503
rs78949787
rs2243196
rs11762203
rs7351079
rs10876353
rs10759715
All − MR Egger
All − Inverse variance weighted
−0.1
0.0
0.1
MR effect size for
'CD33 on Granulocytic Myeloid−Derived Suppressor Cells || id:ebi−a−GCST90001950' on 'Pneumonia || id:ieu−b−4976'

BD

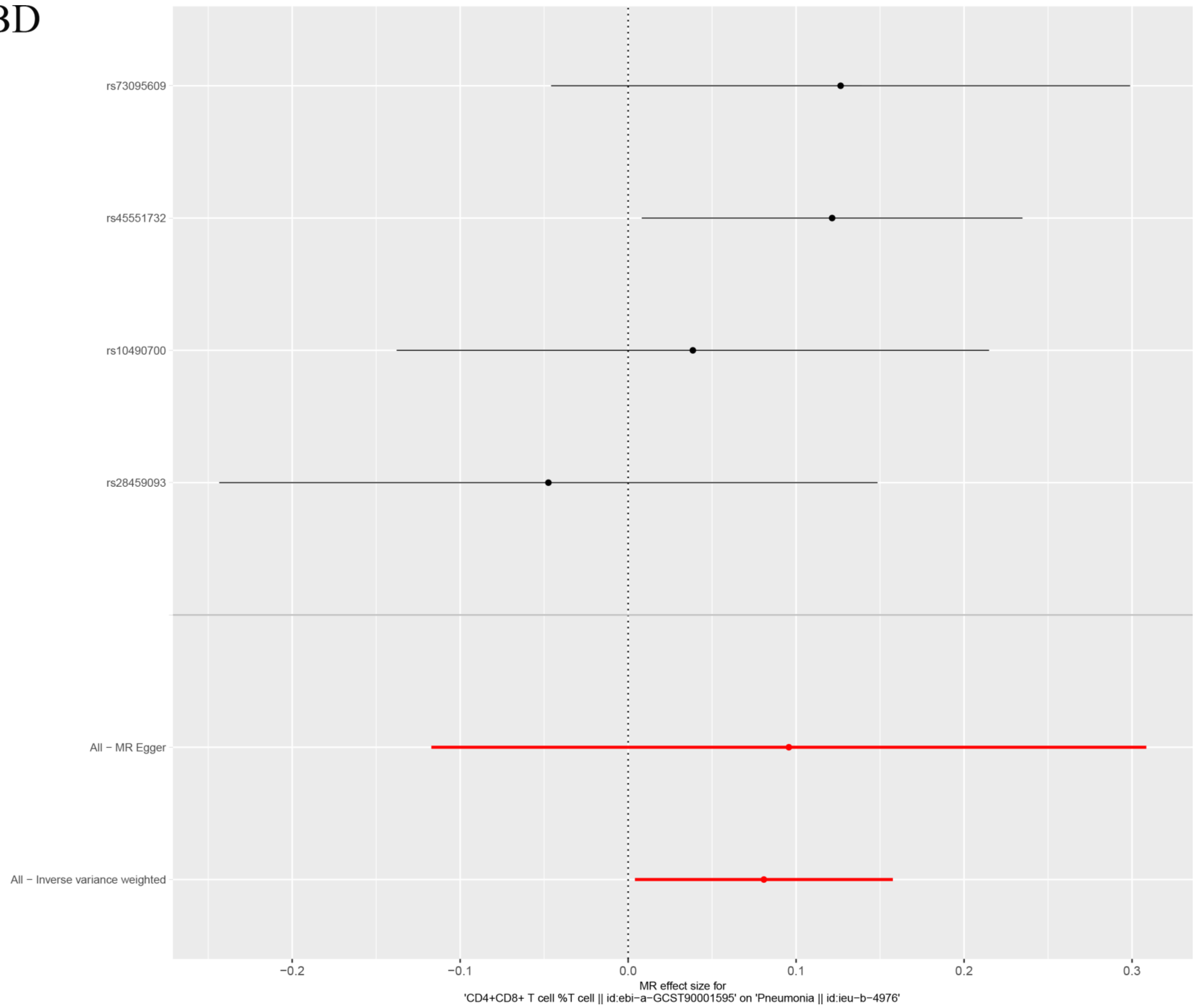
rs73095609
rs45551732
rs10490700
rs28459093
All − MR Egger
All − Inverse variance weighted
−0.2
−0.1
0.0
0.1
0.2
0.3
MR effect size for
'CD4+CD8+ T cell %T cell || id:ebi−a−GCST90001595' on 'Pneumonia || id:ieu−b−4976'

BE

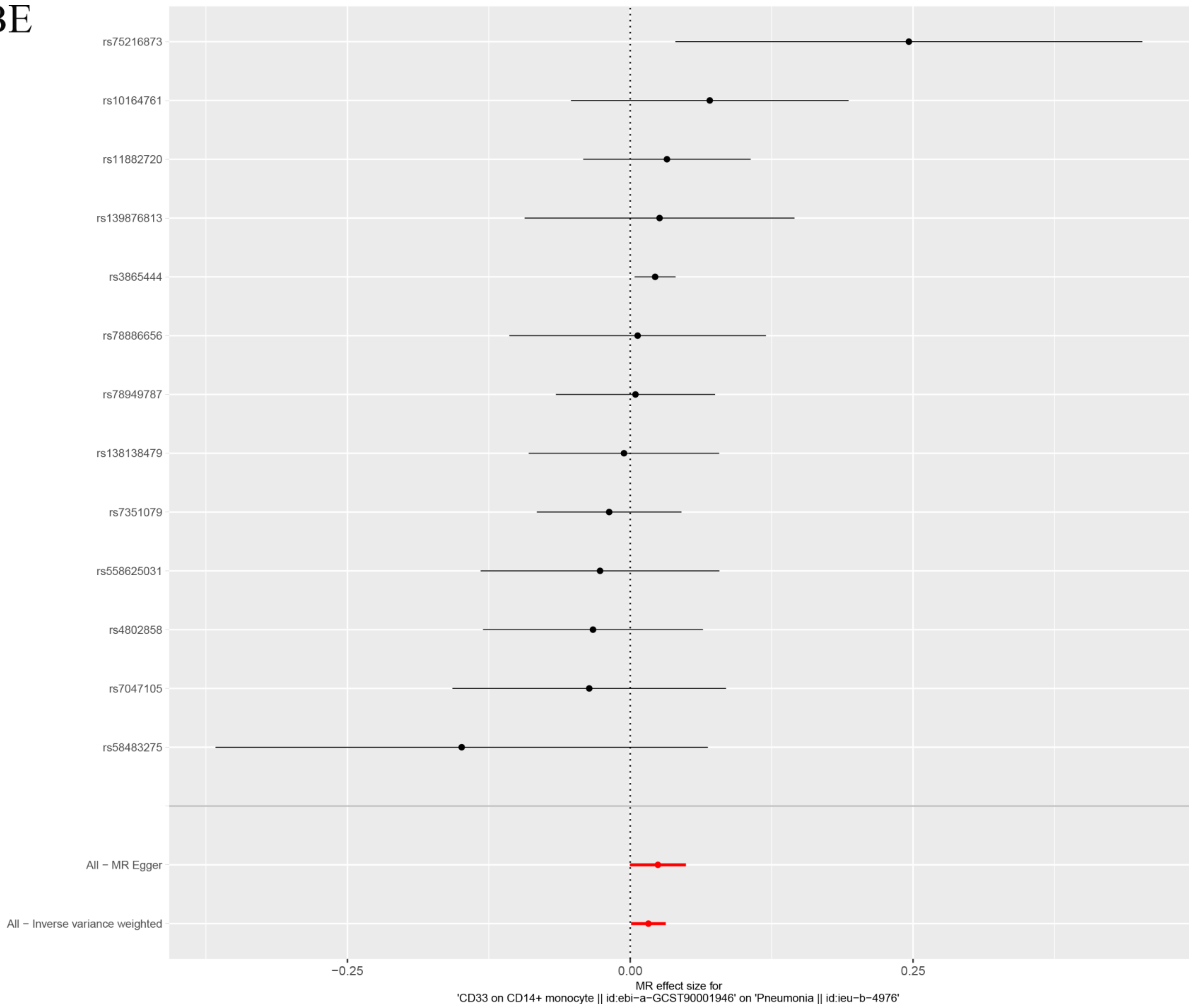
rs75216873
rs10164761
rs11882720
rs139876813
rs3865444
rs78886656
rs78949787
rs138138479
rs7351079
rs558625031
rs4802858
rs7047105
rs58483275
All − MR Egger
All − Inverse variance weighted
−0.25
0.00
0.25
MR effect size for
'CD33 on CD14+ monocyte || id:ebi−a−GCST90001946' on 'Pneumonia || id:ieu−b−4976'

BF

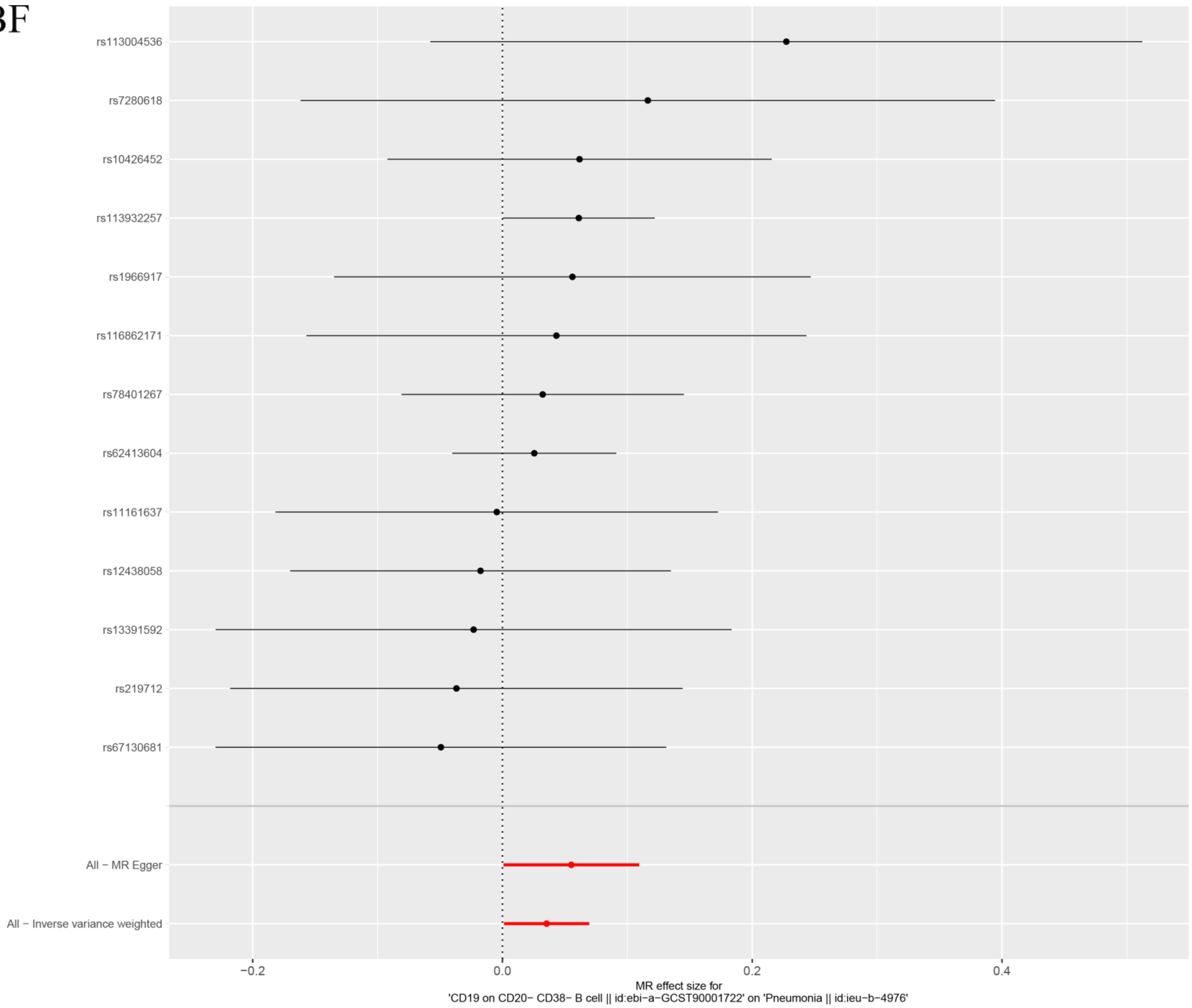
rs113004536
rs7280618
rs10426452
rs113932257
rs1966917
rs116862171
rs78401267
rs62413604
rs11161637
rs12438058
rs13391592
rs219712
rs67130681
All − MR Egger
All − Inverse variance weighted
−0.2
0.0
0.2
0.4
MR effect size for
'CD19 on CD20− CD38− B cell || id:ebi−a−GCST90001722' on 'Pneumonia || id:ieu−b−4976'

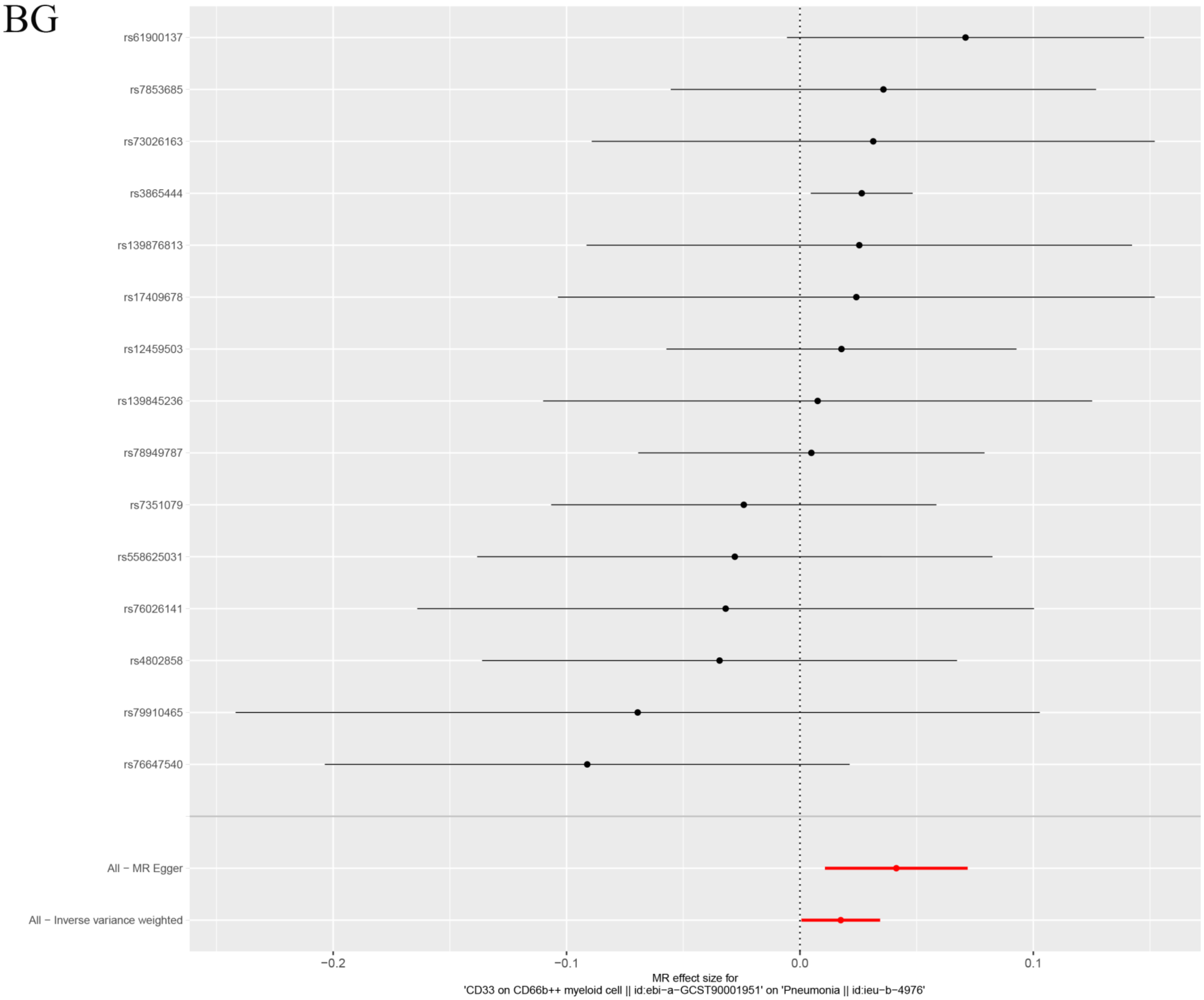

BG
rs61900137
rs7853685
rs73026163
rs3865444
rs139876813
rs17409678
rs12459503
rs139845236
rs78949787
rs7351079
rs558625031
rs76026141
rs4802858
rs79910465
rs76647540
All − MR Egger
All − Inverse variance weighted
−0.2
−0.1
0.0
0.1
MR effect size for
'CD33 on CD66b++ myeloid cell || id:ebi−a−GCST90001951' on 'Pneumonia || id:ieu−b−4976'

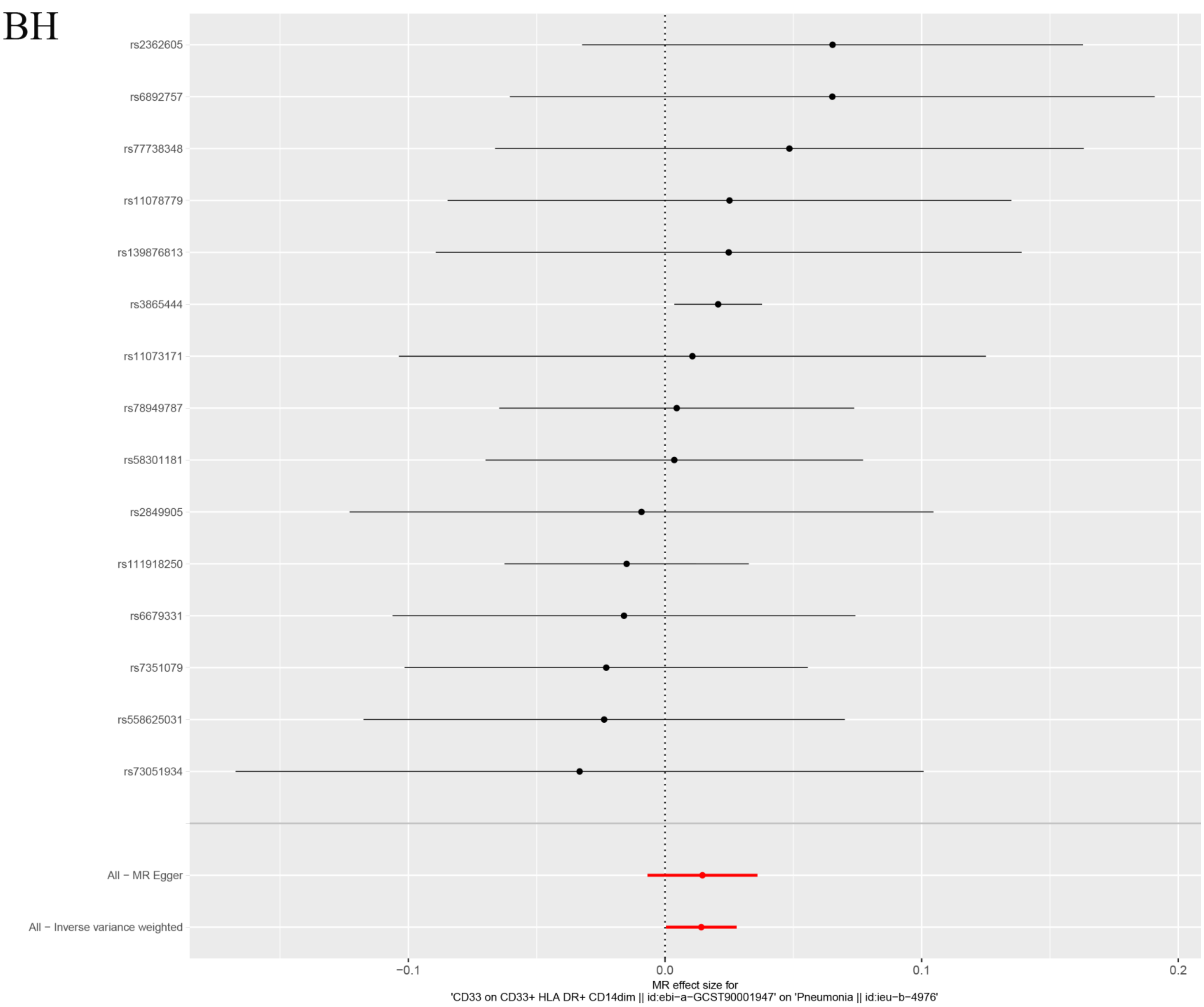

BH
rs2362605
rs6892757
rs77738348
rs11078779
rs139876813
rs3865444
rs11073171
rs78949787
rs58301181
rs2849905
rs111918250
rs6679331
rs7351079
rs558625031
rs73051934
All − MR Egger
All − Inverse variance weighted
−0.1
0.0
0.1
0.2
MR effect size for
'CD33 on CD33+ HLA DR+ CD14dim || id:ebi−a−GCST90001947' on 'Pneumonia || id:ieu−b−4976'

BI

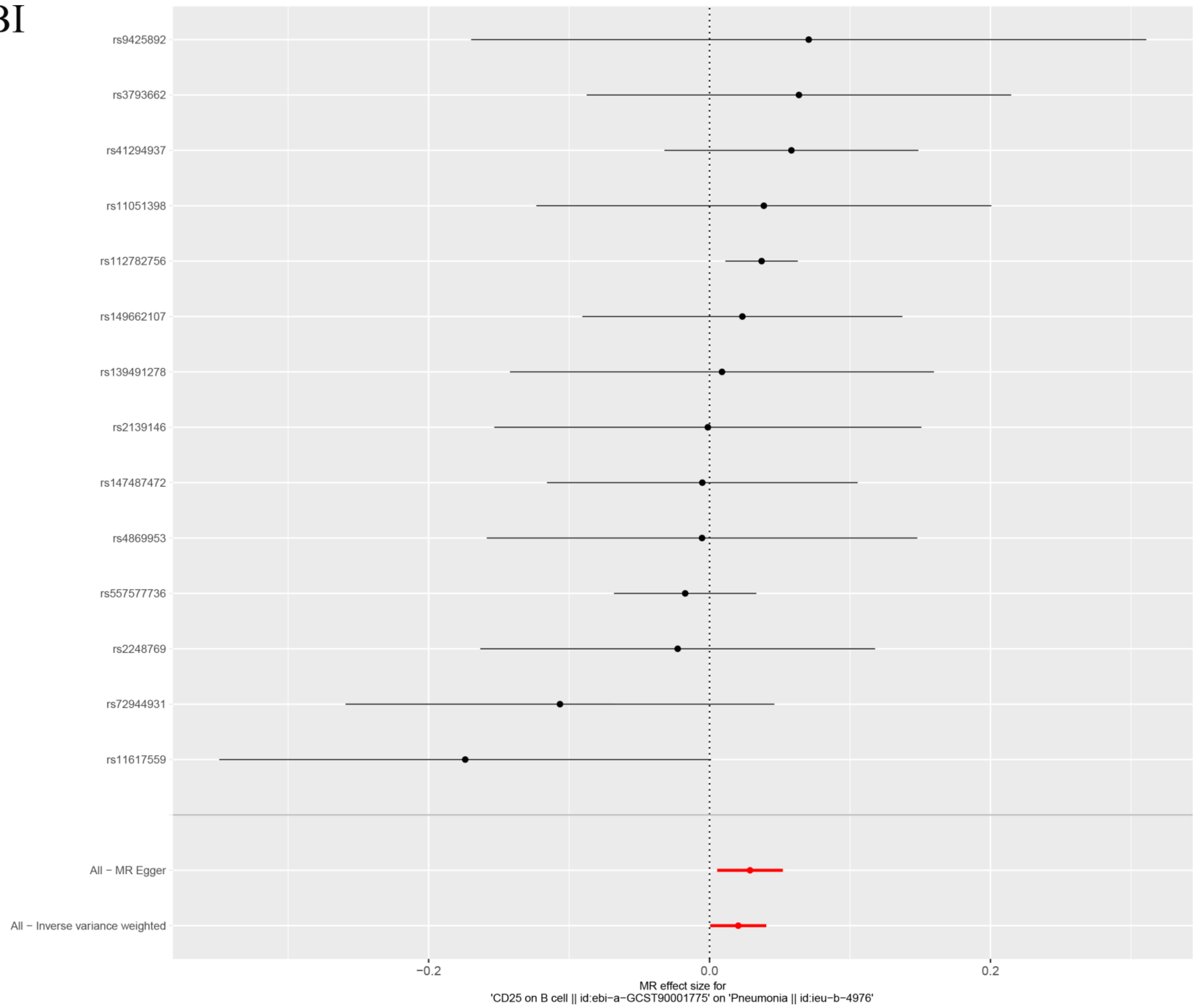

rs9425892
rs3793662
rs41294937
rs11051398
rs112782756
rs149662107
rs139491278
rs2139146
rs147487472
rs4869953
rs557577736
rs2248769
rs72944931
rs11617559
All − MR Egger
All − Inverse variance weighted
−0.2
0.0
0.2
MR effect size for
'CD25 on B cell || id:ebi−a−GCST90001775' on 'Pneumonia || id:ieu−b−4976'

BJ

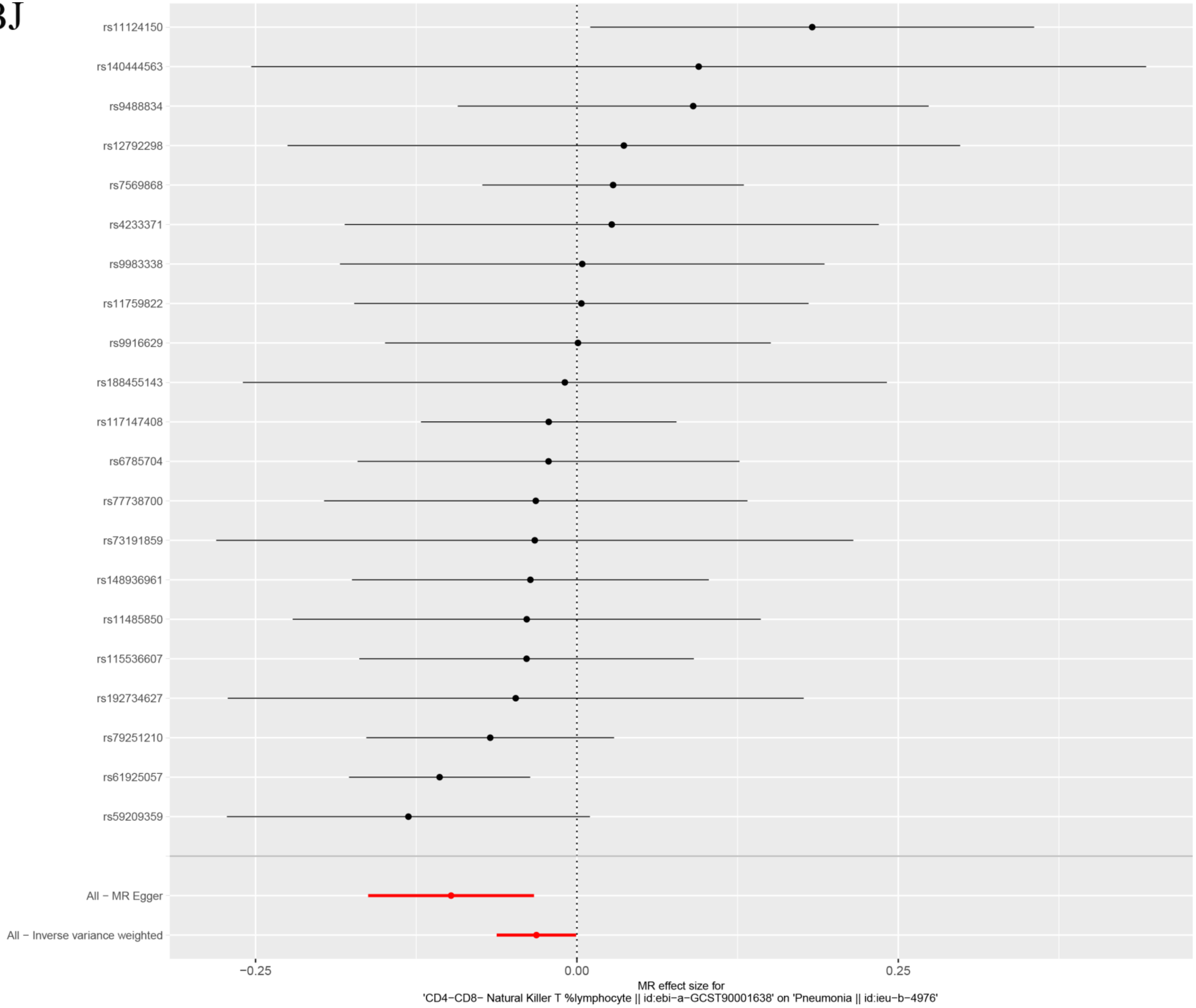
rs11124150
rs140444563
rs9488834
rs12792298
rs7569868
rs4233371
rs9983338
rs11759822
rs9916629
rs188455143
rs117147408
rs6785704
rs77738700
rs73191859
rs148936961
rs11485850
rs115536607
rs192734627
rs79251210
rs61925057
rs59209359
All − MR Egger
All − Inverse variance weighted
−0.25
0.00
0.25
MR effect size for
'CD4−CD8− Natural Killer T %lymphocyte || id:ebi−a−GCST90001638' on 'Pneumonia || id:ieu−b−4976'

BK

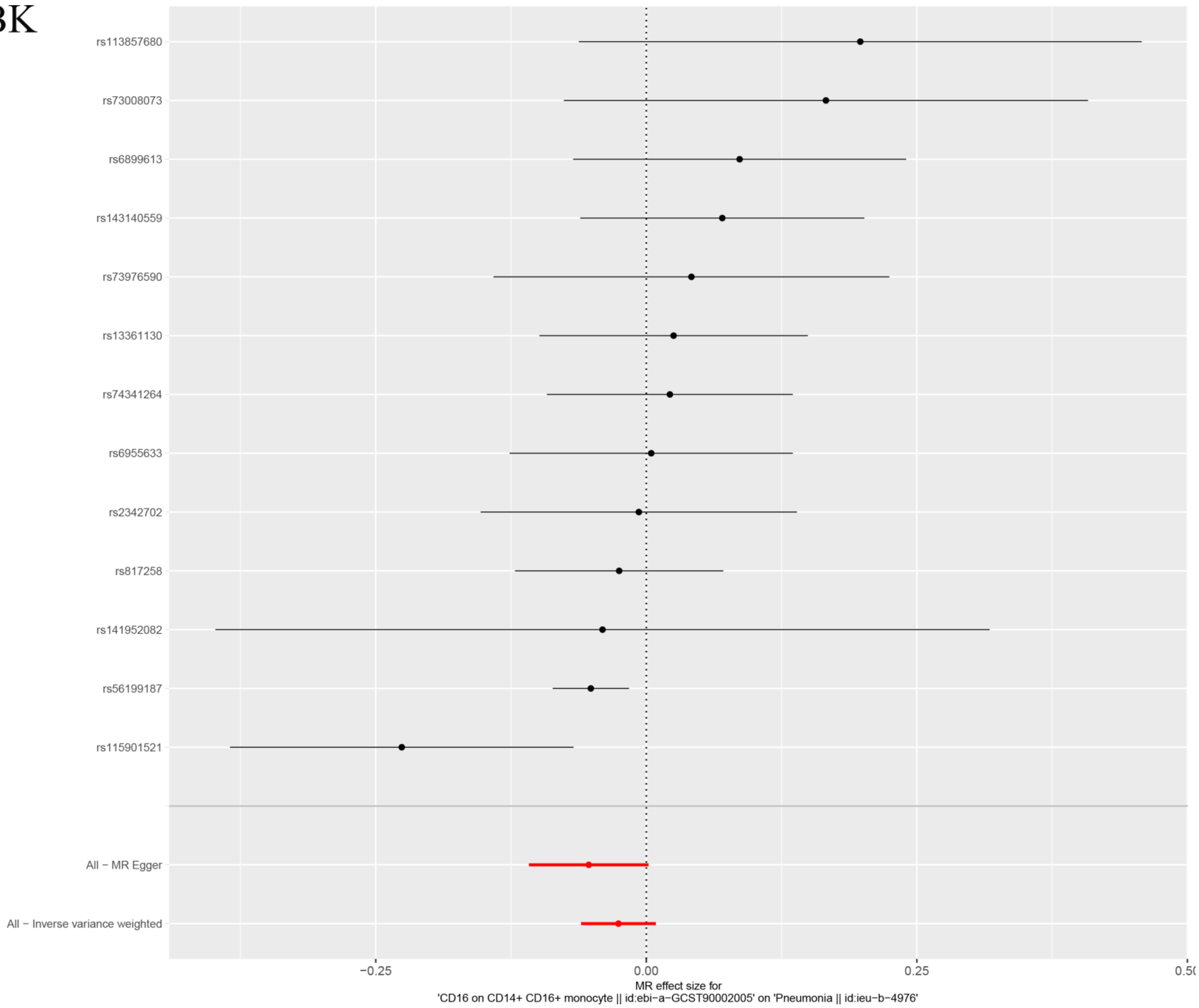

rs113857680
rs73008073
rs6899613
rs143140559
rs73976590
rs13361130
rs74341264
rs6955633
rs2342702
rs817258
rs141952082
rs56199187
rs115901521
All − MR Egger
All − Inverse variance weighted
−0.25
0.00
0.25
MR effect size for
'CD16 on CD14+ CD16+ monocyte || id:ebi−a−GCST90002005' on 'Pneumonia || id:ieu−b−4976'

BL

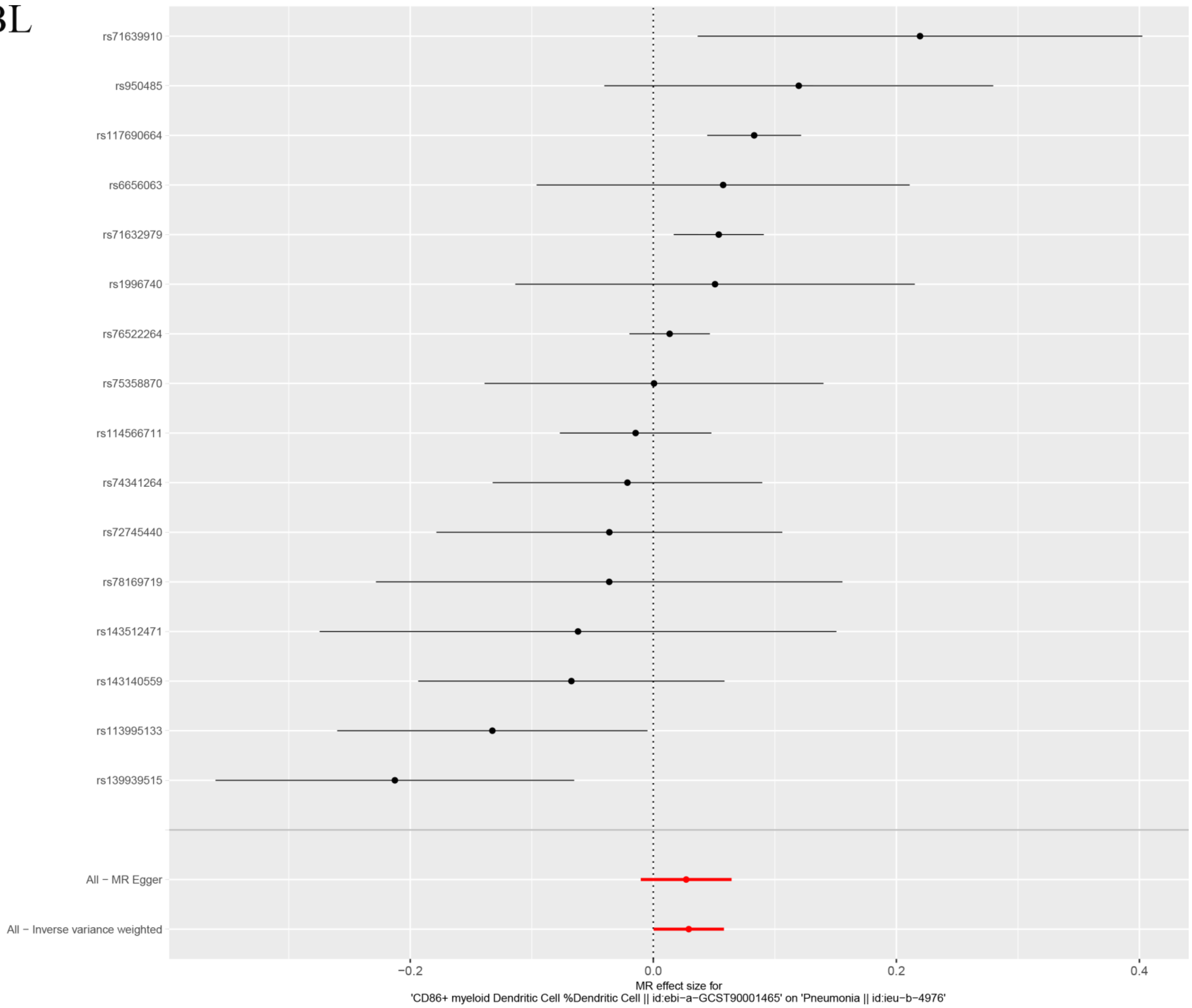

rs71639910
rs950485
rs117690664
rs6656063
rs71632979
rs1996740
rs76522264
rs75358870
rs114566711
rs74341264
rs72745440
rs78169719
rs143512471
rs143140559
rs113995133
rs139939515
All − MR Egger
All − Inverse variance weighted
−0.2
0.0
0.2
0.4
MR effect size for
'CD86+ myeloid Dendritic Cell %Dendritic Cell || id:ebi−a−GCST90001465' on 'Pneumonia || id:ieu−b−4976'

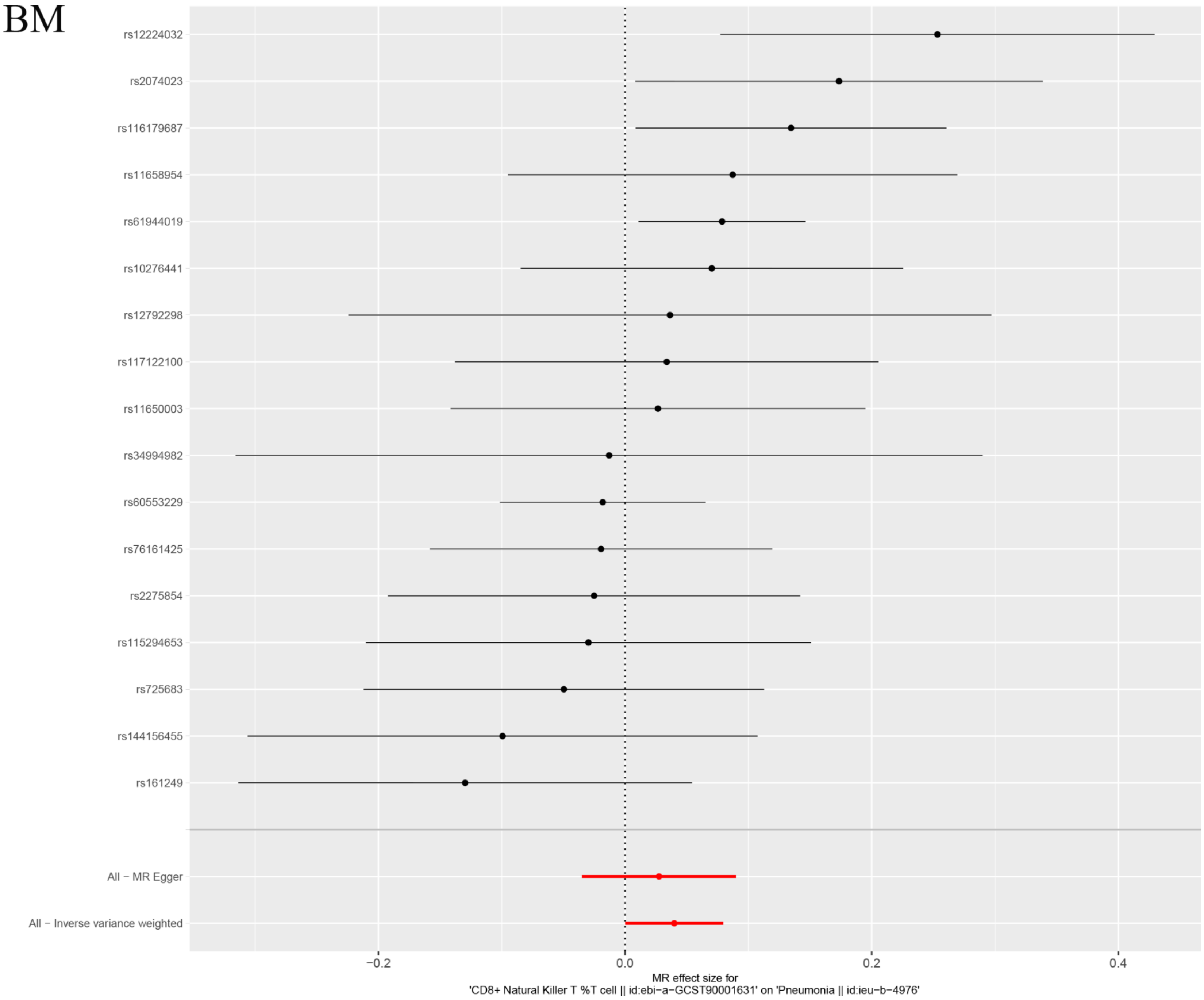

BM
rs12224032
rs2074023
rs116179687
rs11658954
rs61944019
rs10276441
rs12792298
rs117122100
rs11650003
rs34994982
rs60553229
rs76161425
rs2275854
rs115294653
rs725683
rs144156455
rs161249
All − MR Egger
All − Inverse variance weighted
−0.2
0.0
0.2
0.4
MR effect size for
'CD8+ Natural Killer T %T cell || id:ebi−a−GCST90001631' on 'Pneumonia || id:ieu−b−4976'
