# Supplementary material for: Causal Mediation Role of Immune Cells in Gut Microbiota–Pneumonia Associations: A Mendelian Randomisation Study
Source: J Cell Mol Med. 2025 Sep 11;29(17):e70839. doi: 10.1111/jcmm.70839 (PMC12425809; doi:10.1111/jcmm.70839)
Supplement: Supplementary file 5 — S5: Sensitivity analysis of gut microbiota and Pneumonia Mendelian randomization (Scatter plot). [file JCMM-29-e70839-s004.pdf]

Supplementary Figure 1 Sensitivity analysis of gut microbiota and Pneumonia Mendelian randomization (Scatter plot).

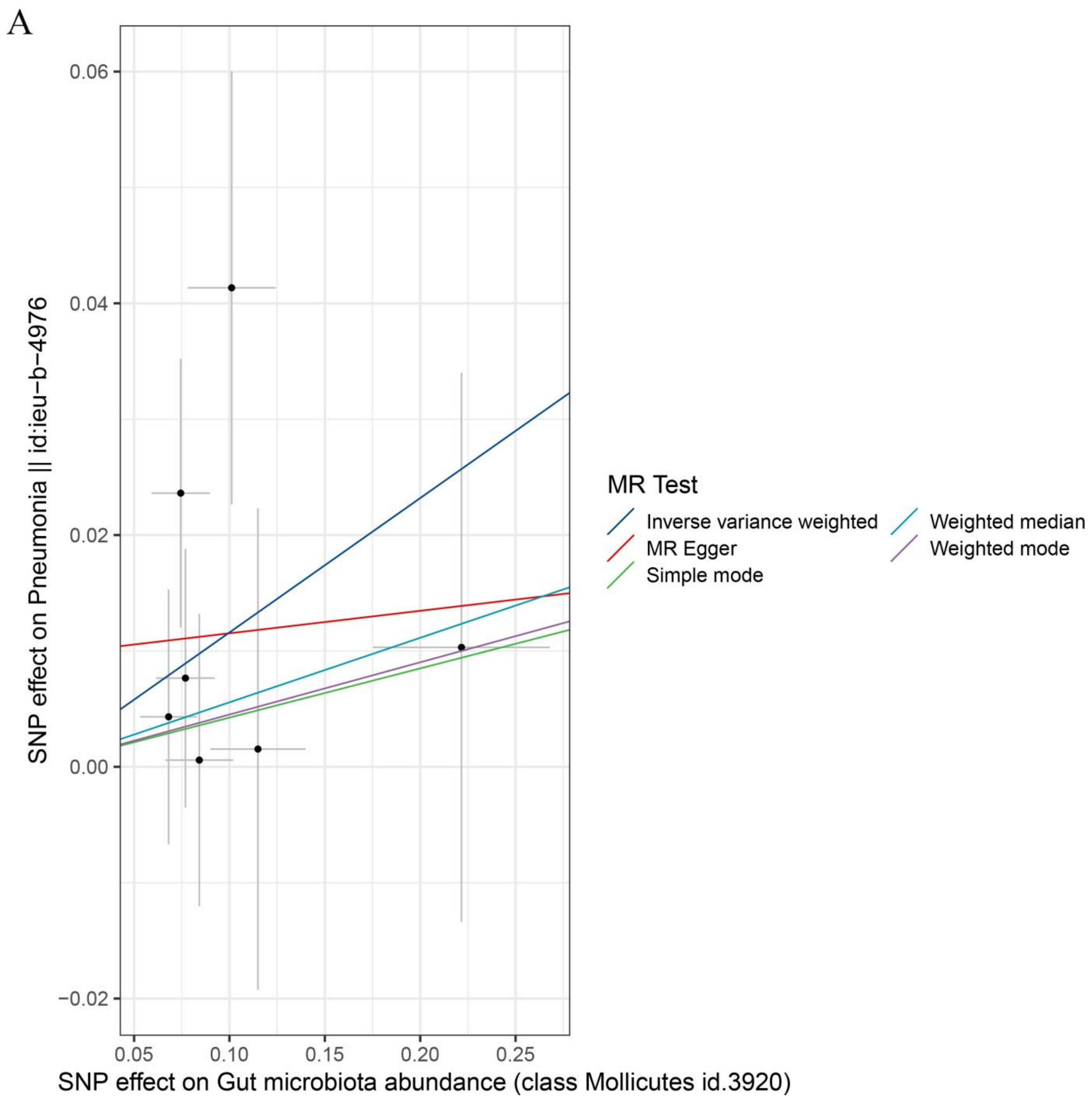

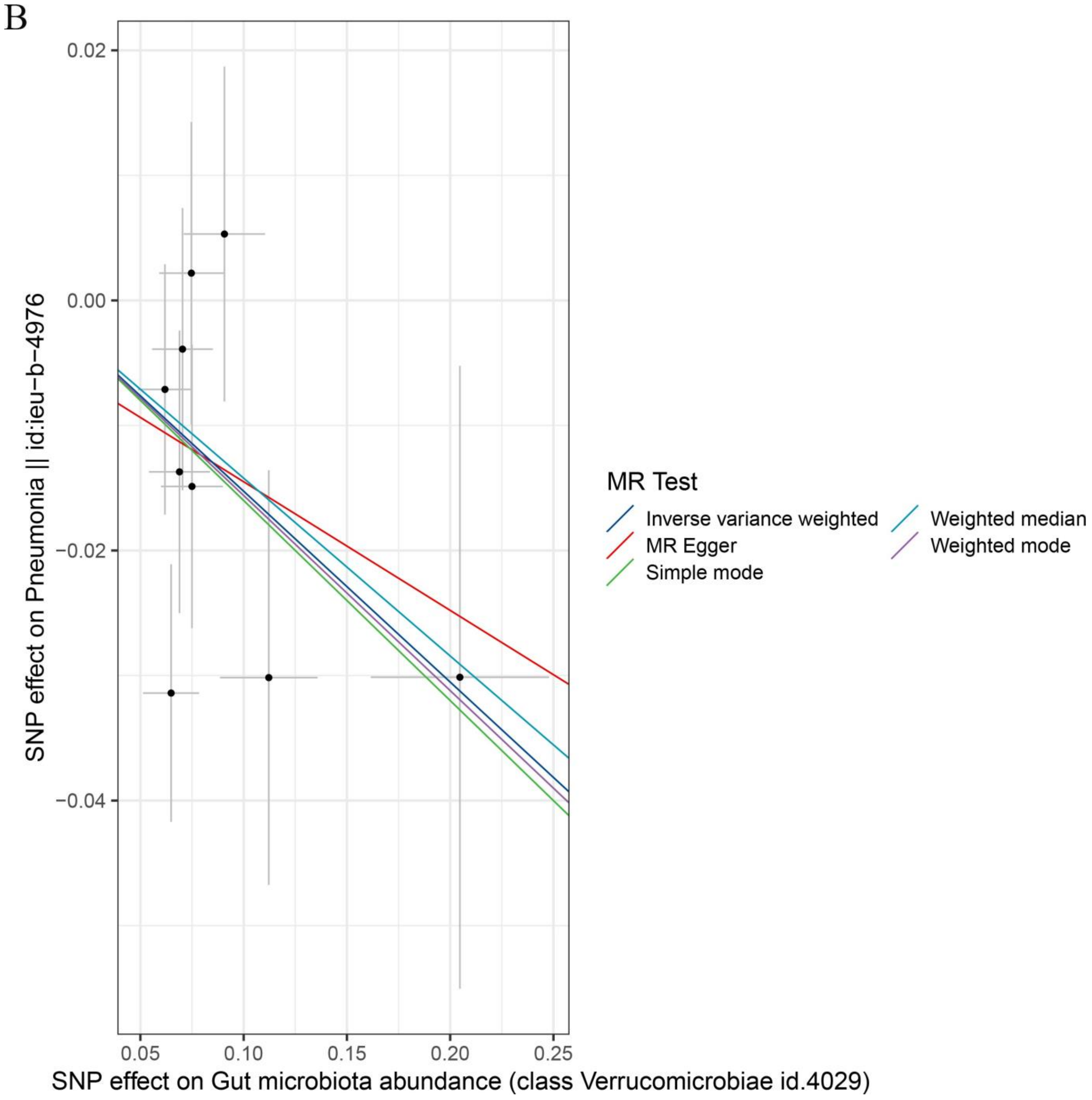
B
0.02
0.00
−0.02
−0.04
SNP effect on Pneumonia || id:ieu−b−4976
0.05
0.10
0.15
0.20
0.25
SNP effect on Gut microbiota abundance (class Verrucomicrobiae id.4029)
MR Test
Inverse variance weighted
MR Egger
Simple mode
Weighted median
Weighted mode

C

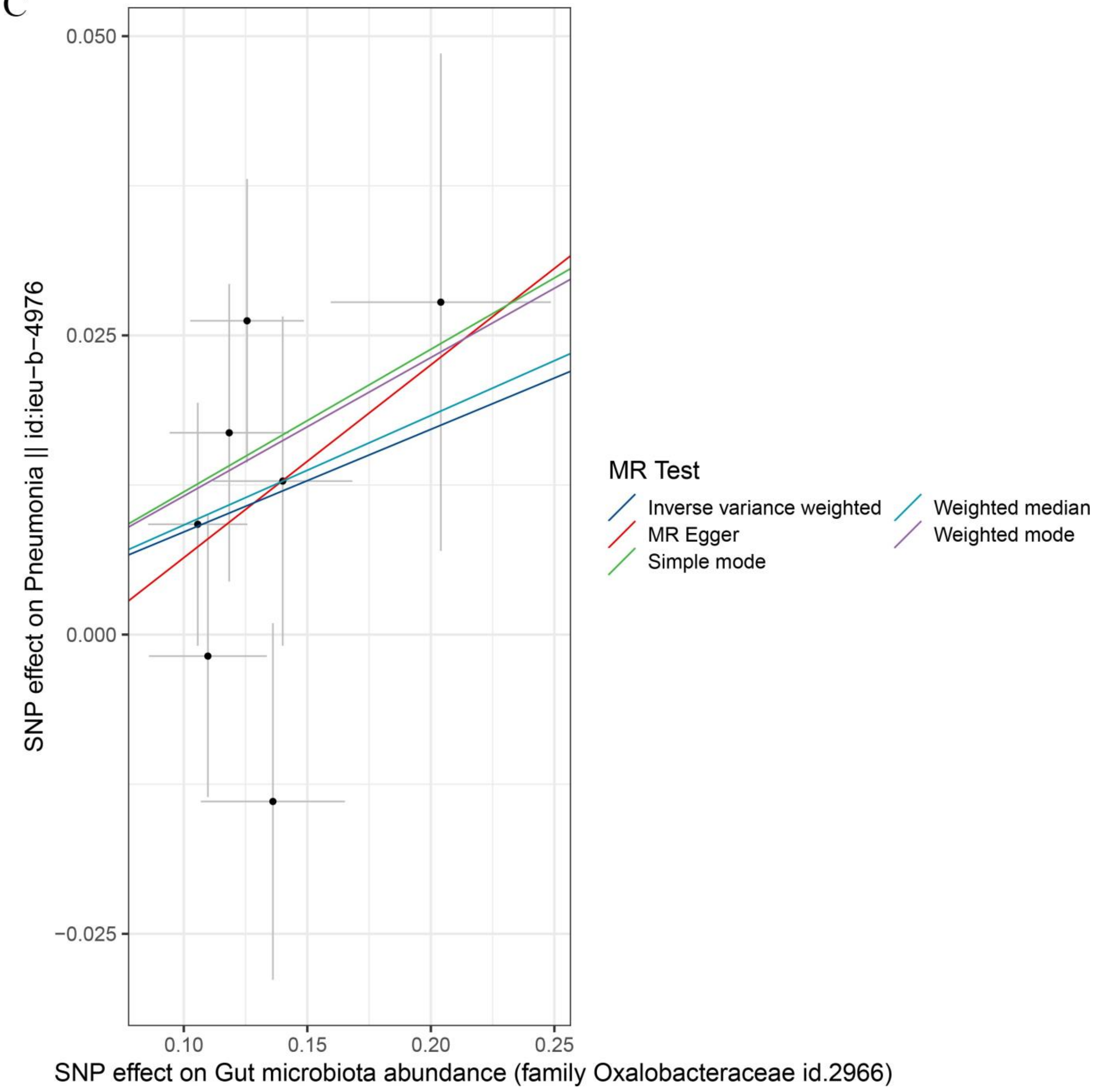

0.050
0.025
0.000
−0.025
0.10
0.15
0.20
0.25
SNP effect on Pneumonia || id:ieu−b−4976
SNP effect on Gut microbiota abundance (family Oxalobacteraceae id.2966)
MR Test
Inverse variance weighted
MR Egger
Simple mode
Weighted median
Weighted mode

D

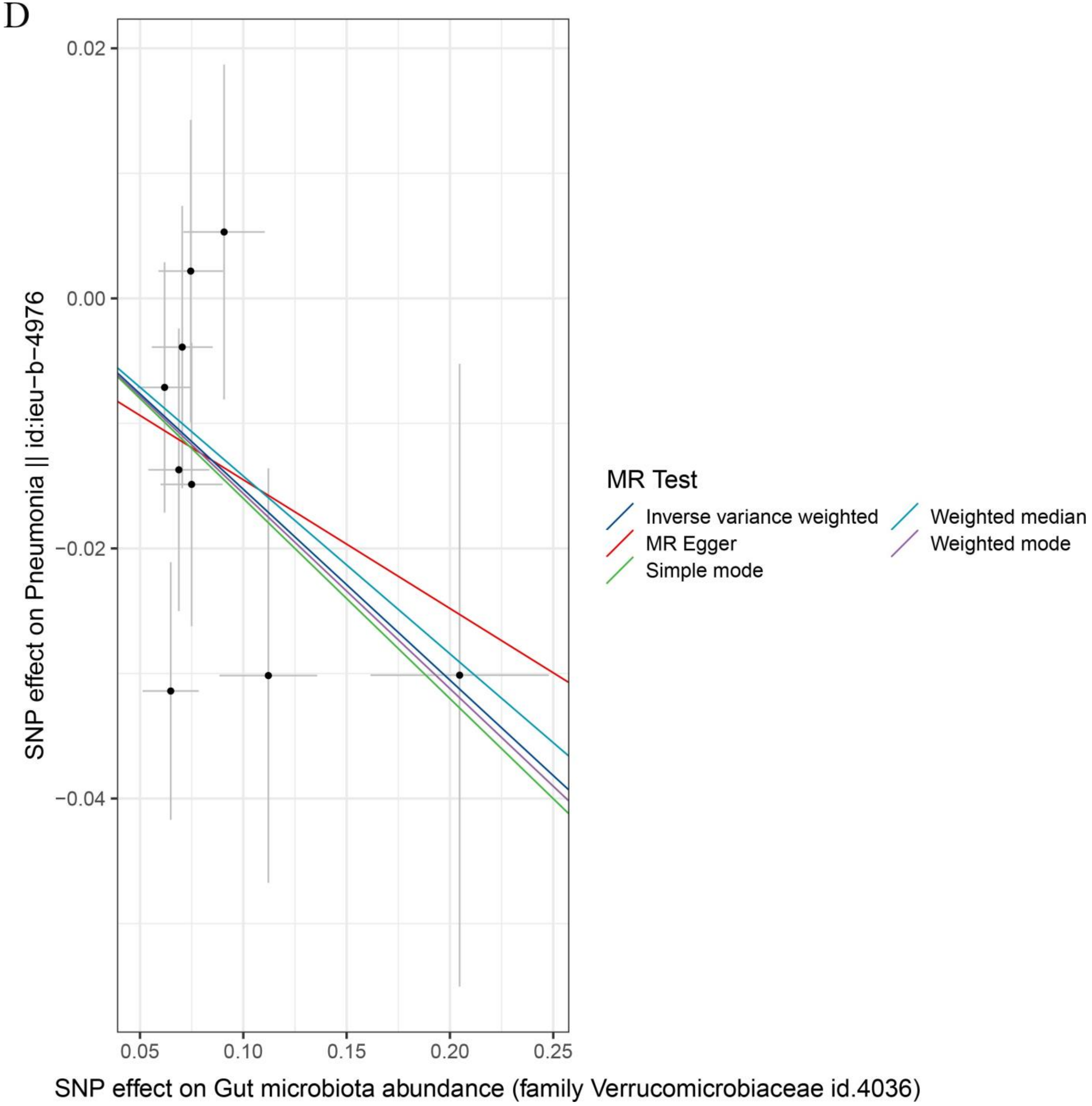

0.02
0.00
−0.02
−0.04
SNP effect on Pneumonia || id:ieu-b-4976
0.05
0.10
0.15
0.20
0.25
SNP effect on Gut microbiota abundance (family Verrucomicrobiaceae id.4036)
MR Test
Inverse variance weighted
MR Egger
Simple mode
Weighted median
Weighted mode

E

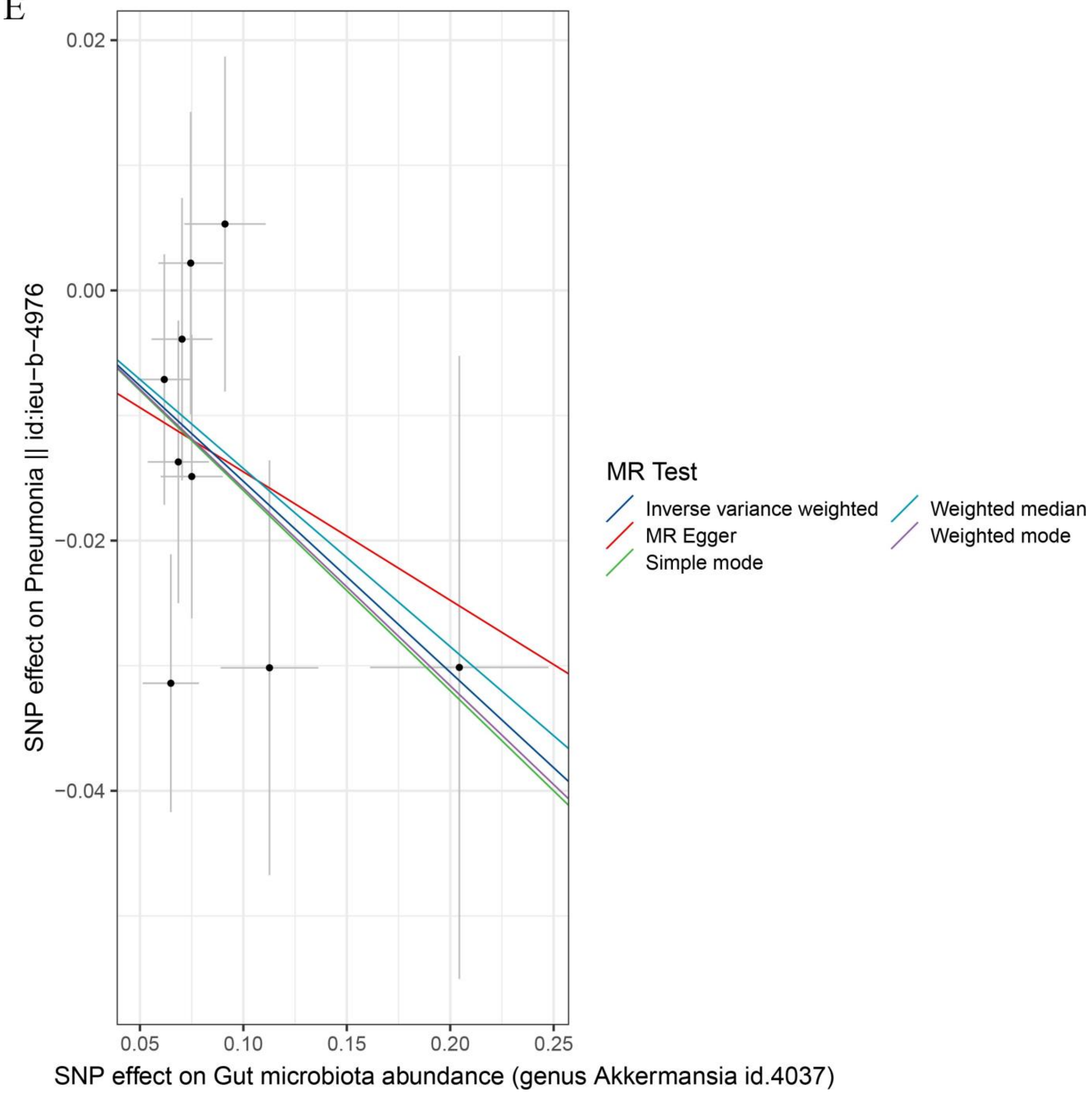

0.02
0.00
−0.02
−0.04
SNP effect on Pneumonia || id:ieu-b-4976
0.05
0.10
0.15
0.20
0.25
SNP effect on Gut microbiota abundance (genus Akkermansia id.4037)
MR Test
Inverse variance weighted
MR Egger
Simple mode
Weighted median
Weighted mode

F

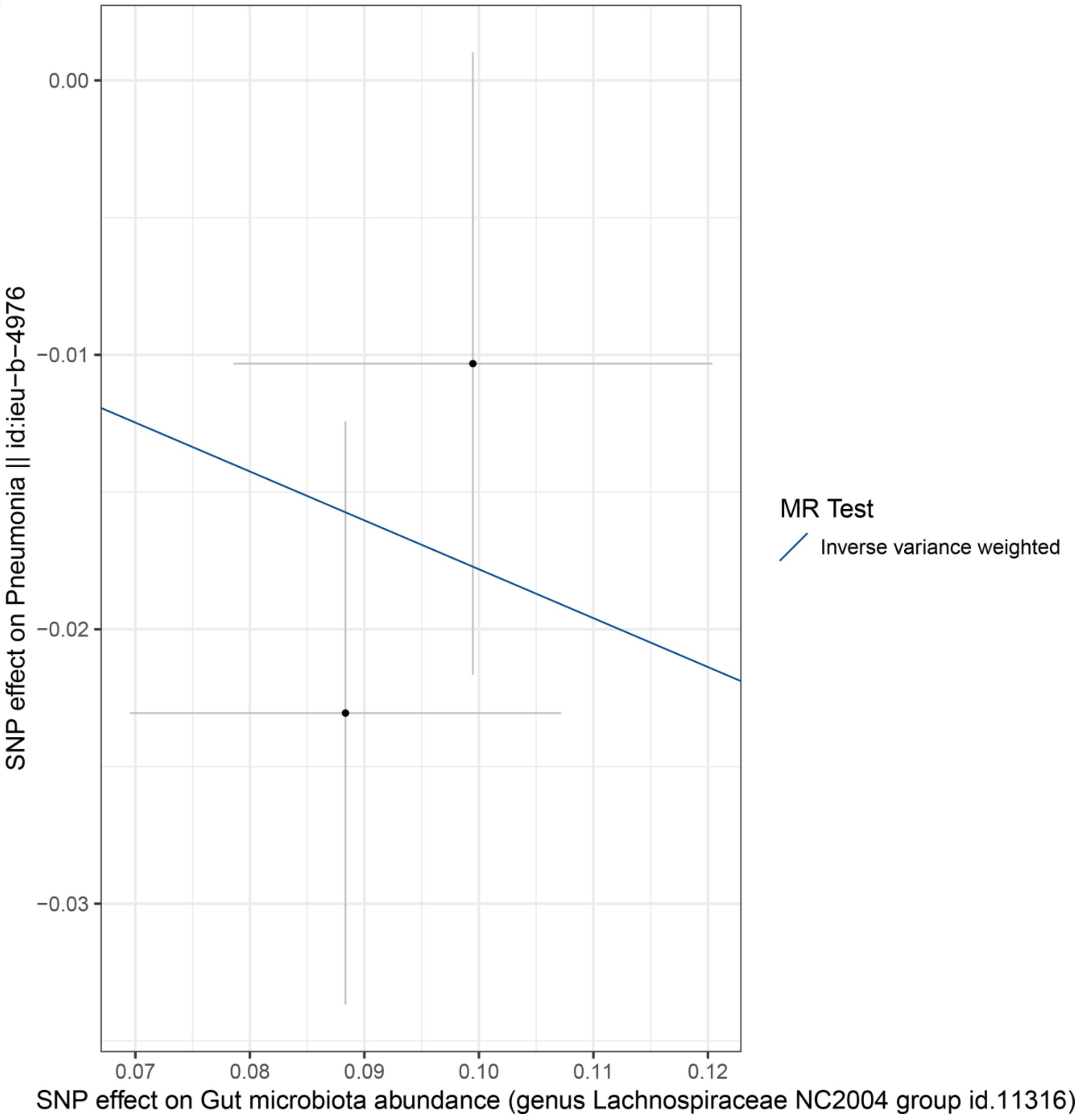
0.00
−0.01
−0.02
−0.03
SNP effect on Pneumonia || id:ieu−b−4976
0.07
0.08
0.09
0.10
0.11
0.12
SNP effect on Gut microbiota abundance (genus Lachnospiraceae NC2004 group id.11316)
MR Test
Inverse variance weighted

G

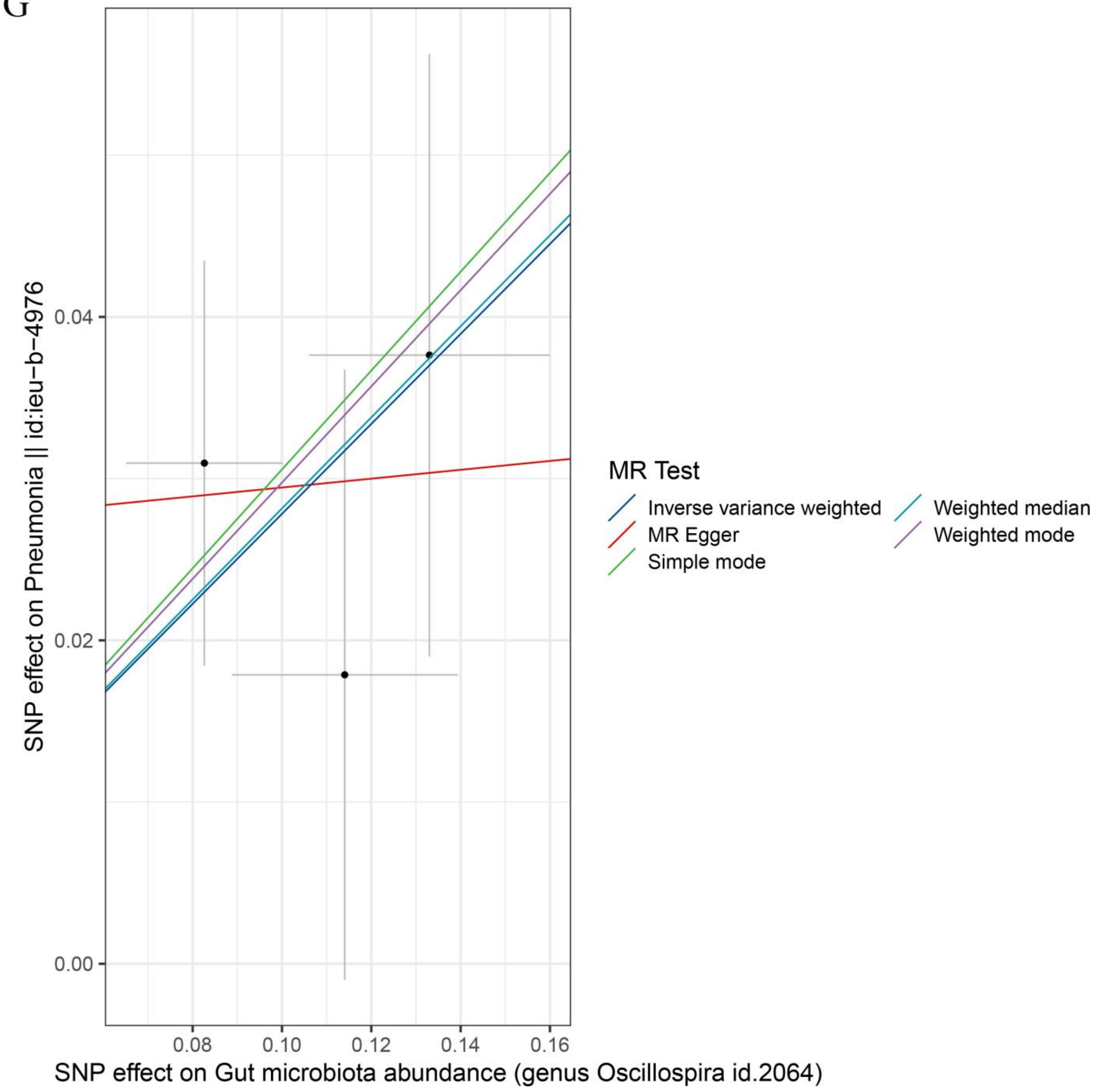

SNP effect on Pneumonia || id:ieu-b-4976
0.00
0.02
0.04
0.08
0.10
0.12
0.14
0.16
SNP effect on Gut microbiota abundance (genus Oscillospira id.2064)
MR Test
Inverse variance weighted
MR Egger
Simple mode
Weighted median
Weighted mode

H

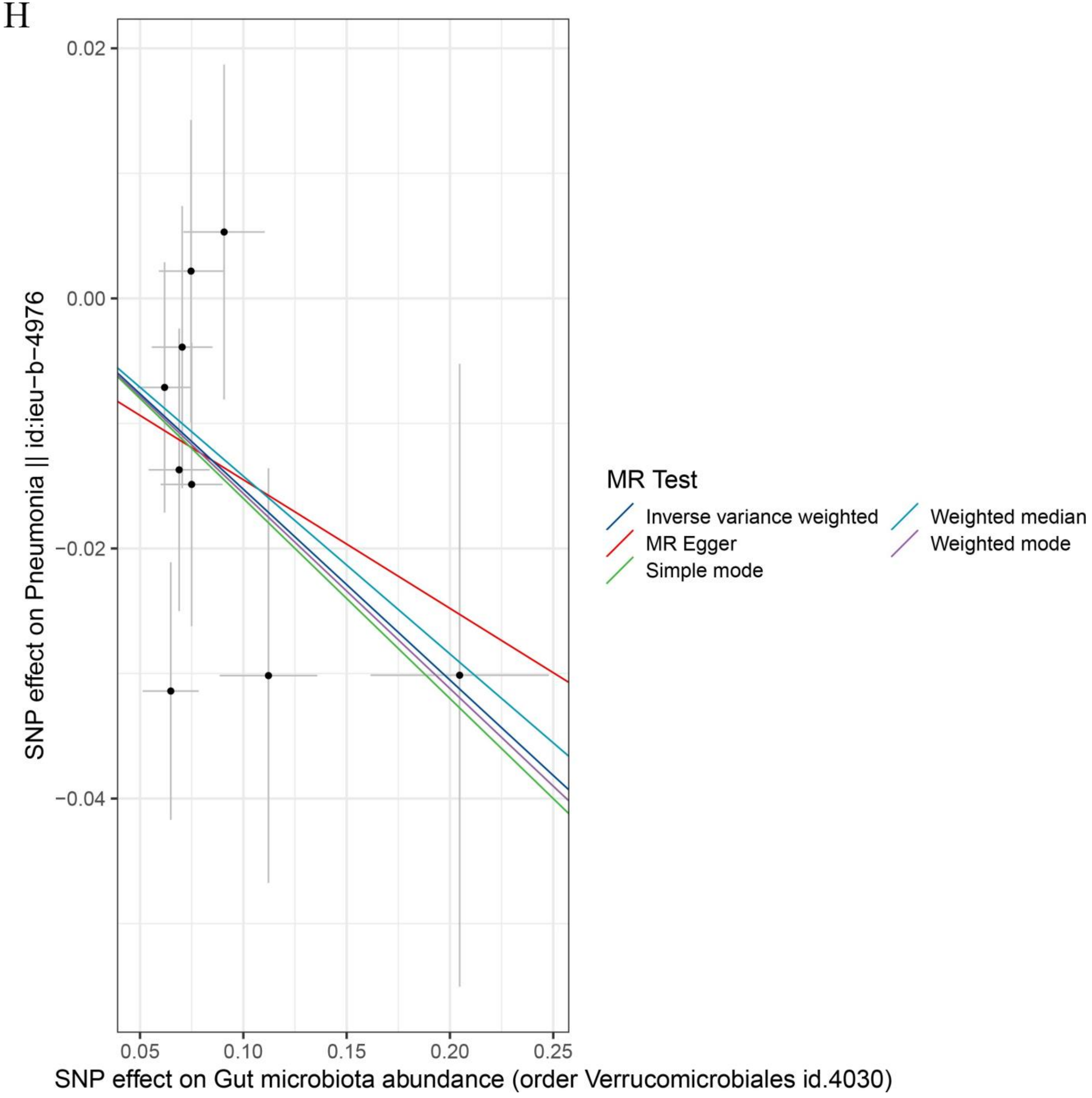

0.02
0.00
−0.02
−0.04
SNP effect on Pneumonia || id:ieu−b−4976
0.05
0.10
0.15
0.20
0.25
SNP effect on Gut microbiota abundance (order Verrucomicrobiales id.4030)
MR Test
Inverse variance weighted
MR Egger
Simple mode
Weighted median
Weighted mode

I

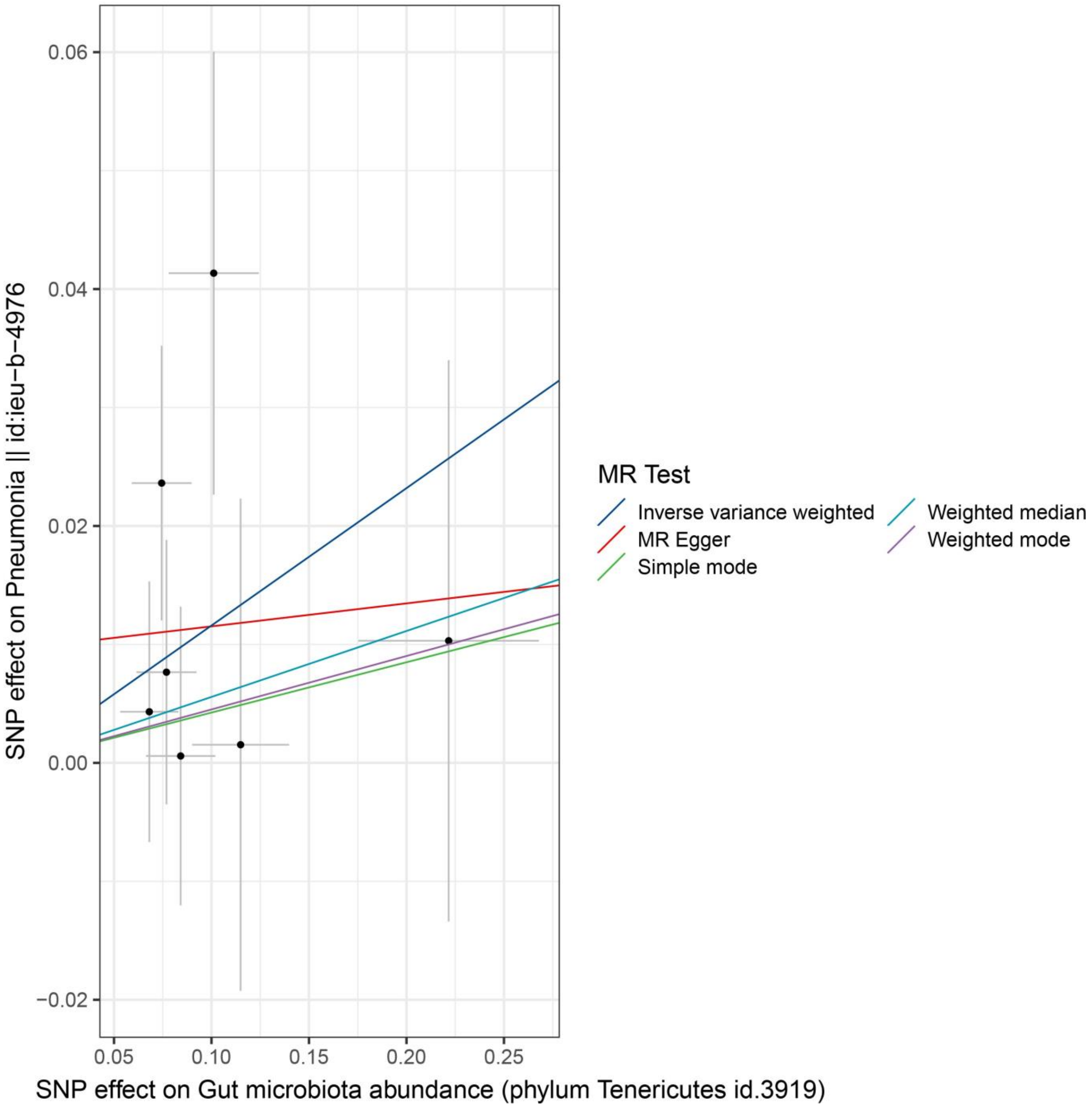

0.06
0.04
0.02
0.00
−0.02
0.05
0.10
0.15
0.20
0.25
SNP effect on Pneumonia || id:ieu-b-4976
SNP effect on Gut microbiota abundance (phylum Tenericutes id.3919)
MR Test
Inverse variance weighted
MR Egger
Simple mode
Weighted median
Weighted mode
