# Supplementary material for: Causal Mediation Role of Immune Cells in Gut Microbiota–Pneumonia Associations: A Mendelian Randomisation Study
Source: J Cell Mol Med. 2025 Sep 11;29(17):e70839. doi: 10.1111/jcmm.70839 (PMC12425809; doi:10.1111/jcmm.70839)
Supplement: Supplementary file 6 — S6: Sensitivity analysis of gut microbiota and Pneumonia Mendelian randomization (Forest plot). [file JCMM-29-e70839-s005.pdf]

Supplementary Figure 2 Sensitivity analysis of gut microbiota and Pneumonia Mendelian randomization (Forest plot).

A

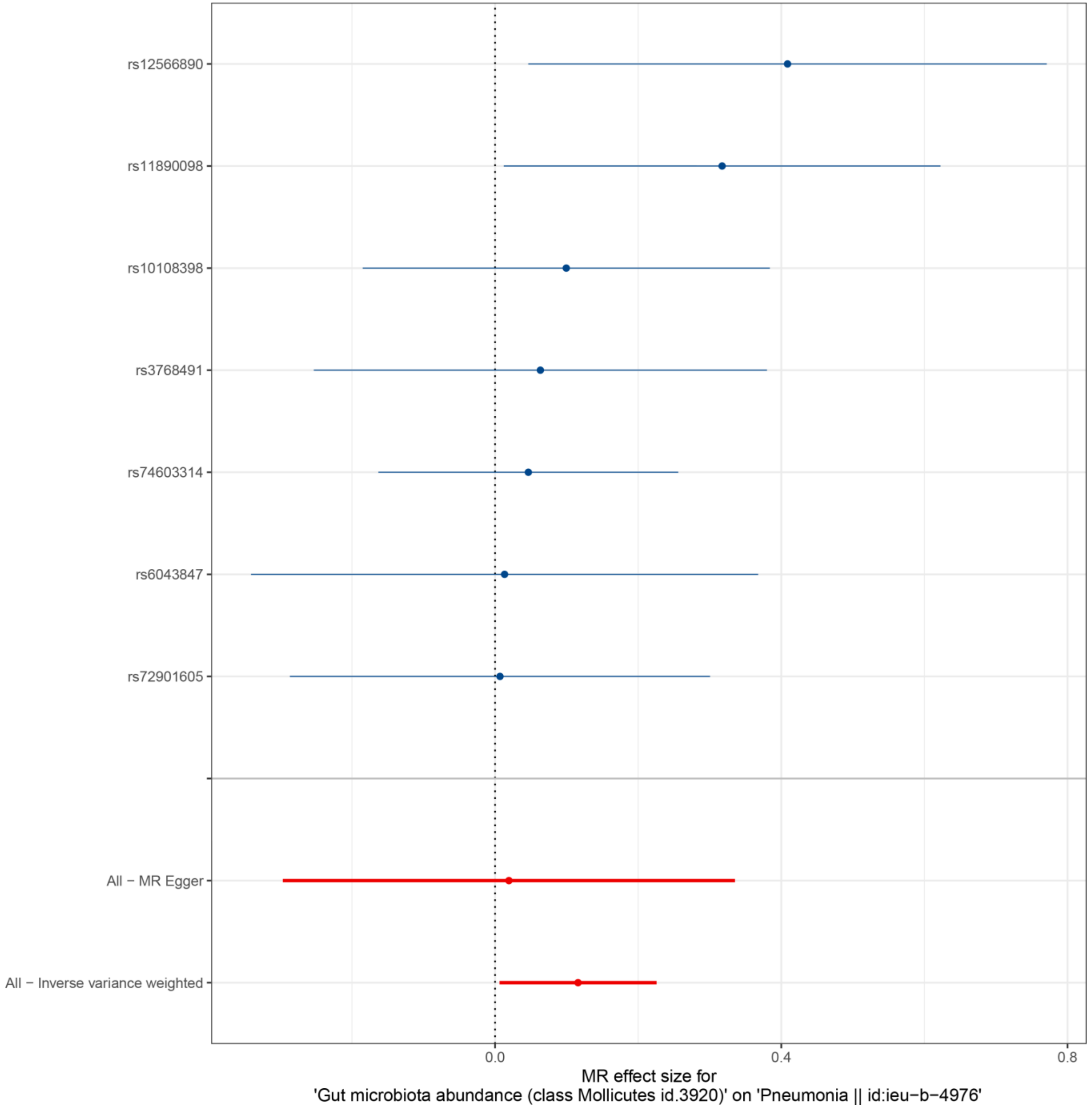

B

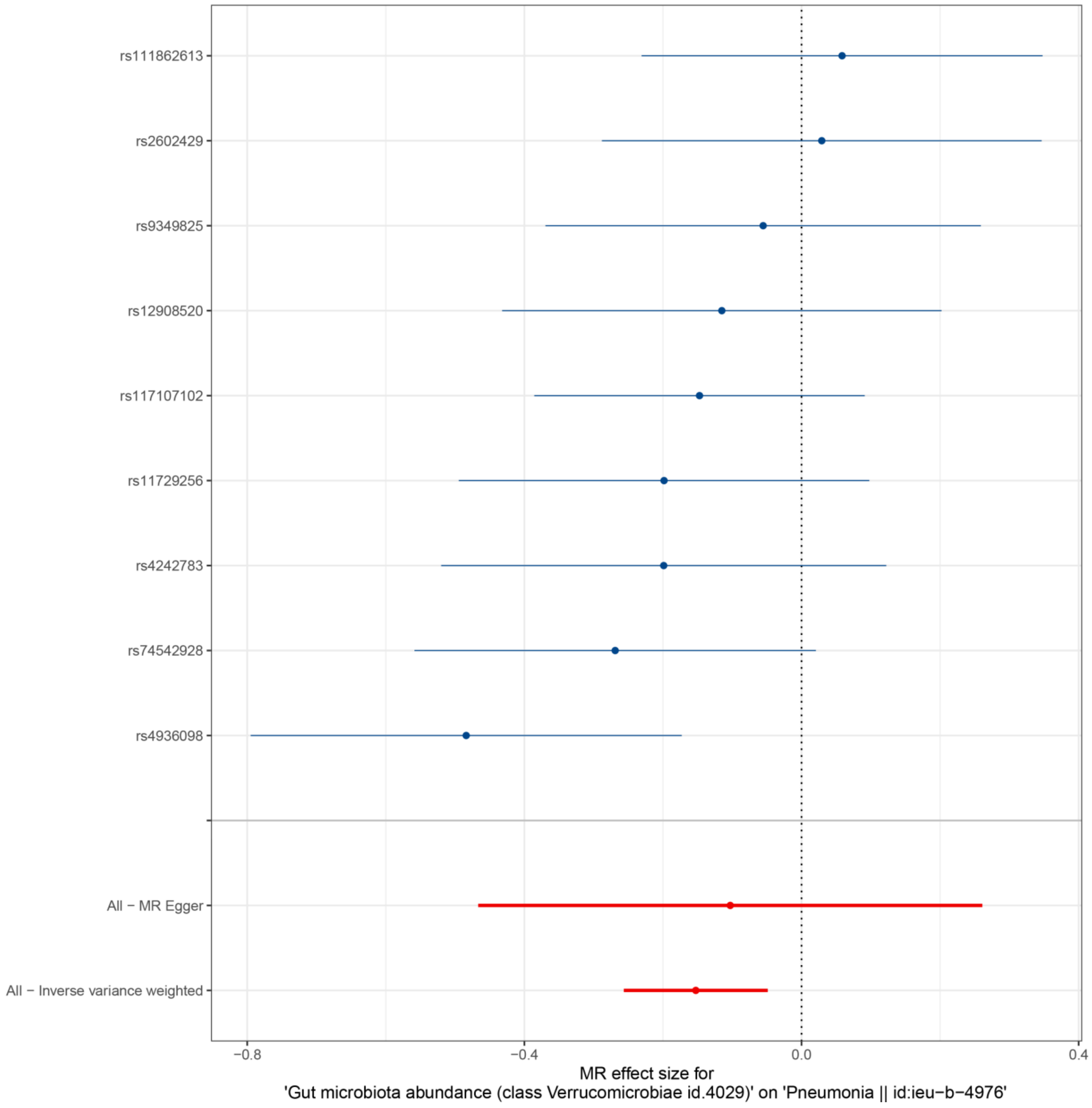
rs111862613
rs2602429
rs9349825
rs12908520
rs117107102
rs11729256
rs4242783
rs74542928
rs4936098
All − MR Egger
All − Inverse variance weighted
−0.8
−0.4
0.0
0.4
MR effect size for
'Gut microbiota abundance (class Verrucomicrobiae id.4029)' on 'Pneumonia || id:ieu−b−4976'

C

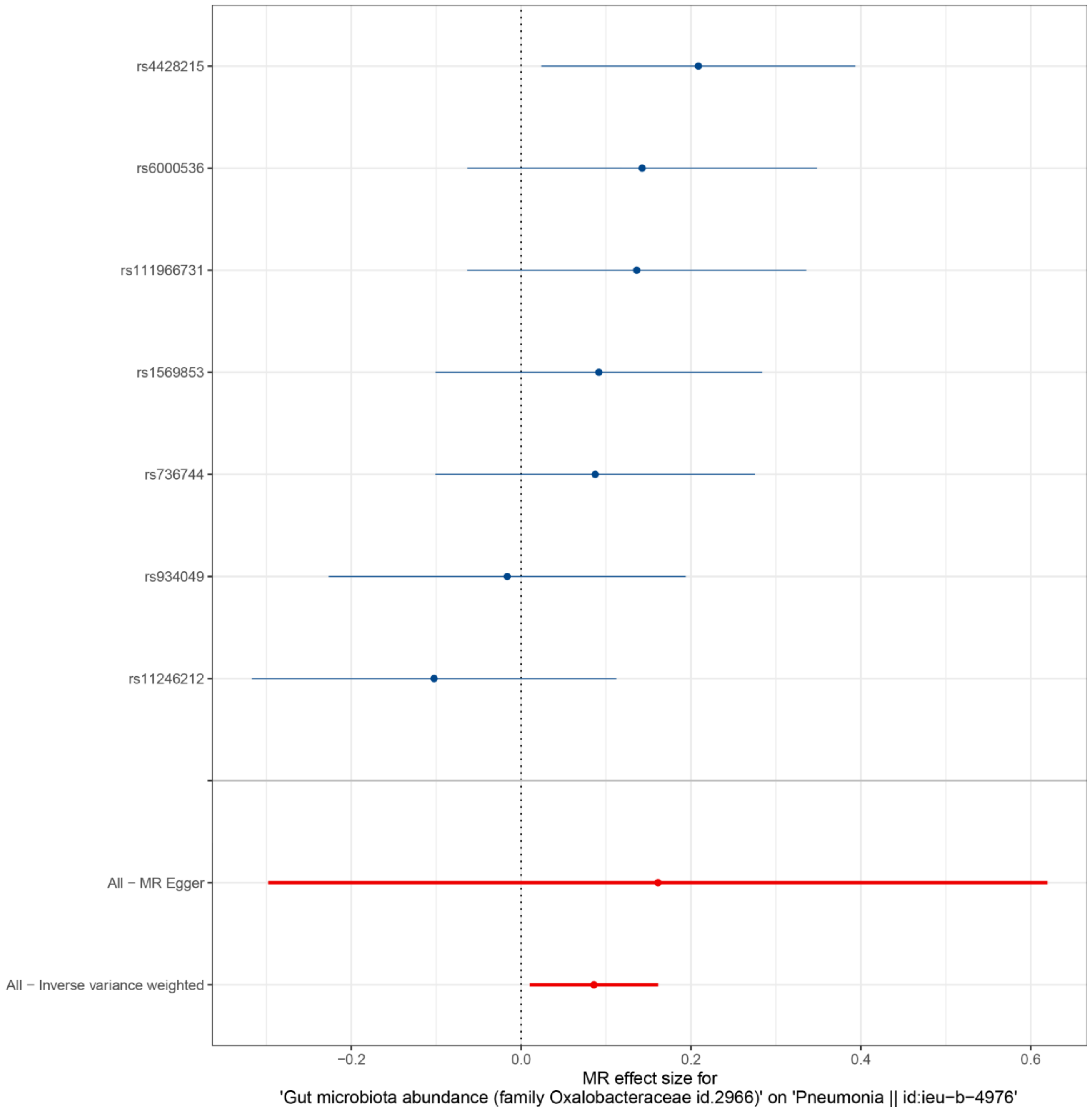
rs4428215
rs6000536
rs111966731
rs1569853
rs736744
rs934049
rs11246212
All − MR Egger
All − Inverse variance weighted
−0.2
0.0
0.2
0.4
0.6
MR effect size for
'Gut microbiota abundance (family Oxalobacteraceae id.2966)' on 'Pneumonia || id:ieu−b−4976'

D

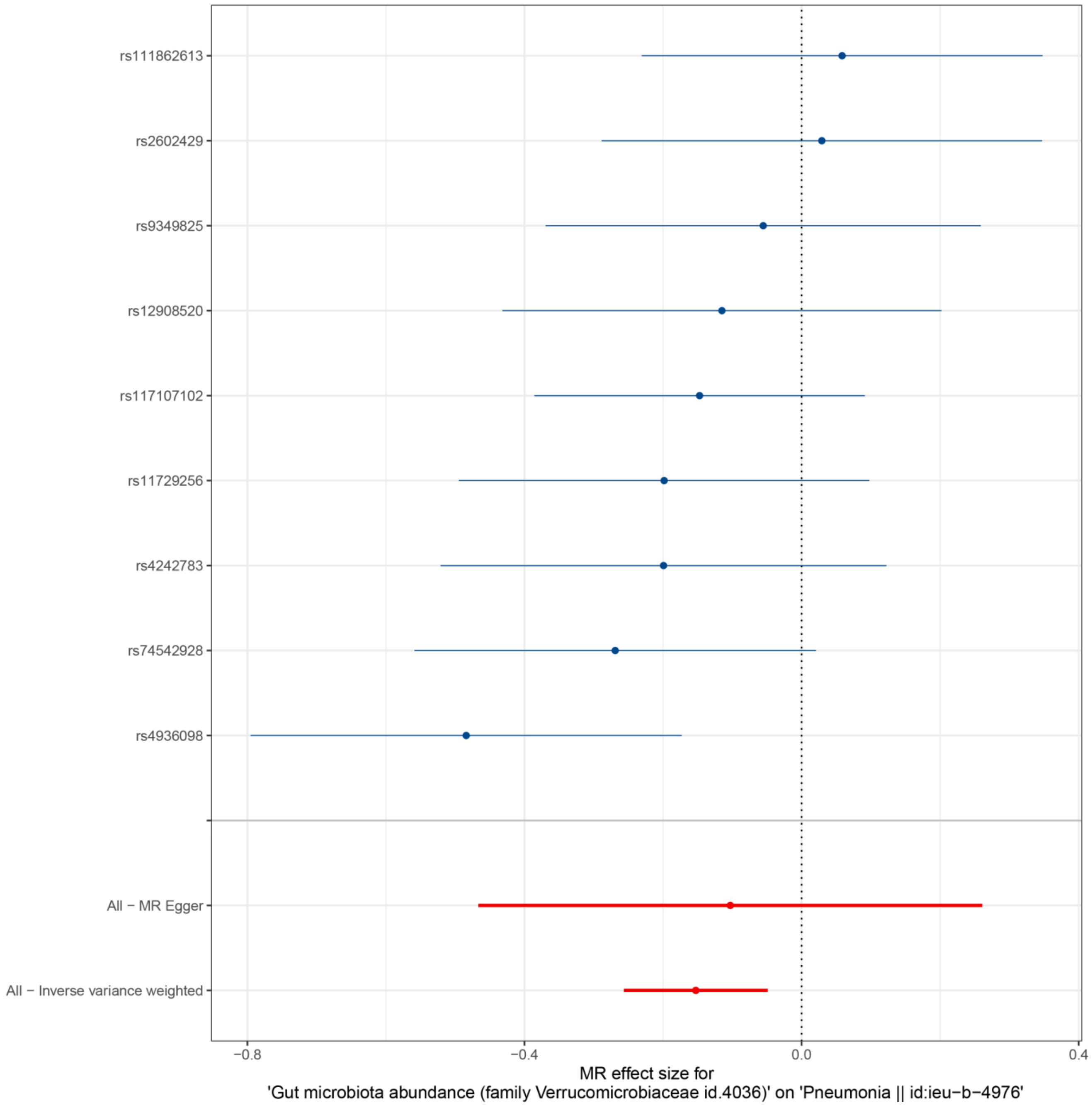
rs111862613
rs2602429
rs9349825
rs12908520
rs117107102
rs11729256
rs4242783
rs74542928
rs4936098
All − MR Egger
All − Inverse variance weighted
−0.8
−0.4
0.0
0.4
MR effect size for
'Gut microbiota abundance (family Verrucomicrobiaceae id.4036)' on 'Pneumonia || id:ieu−b−4976'

E

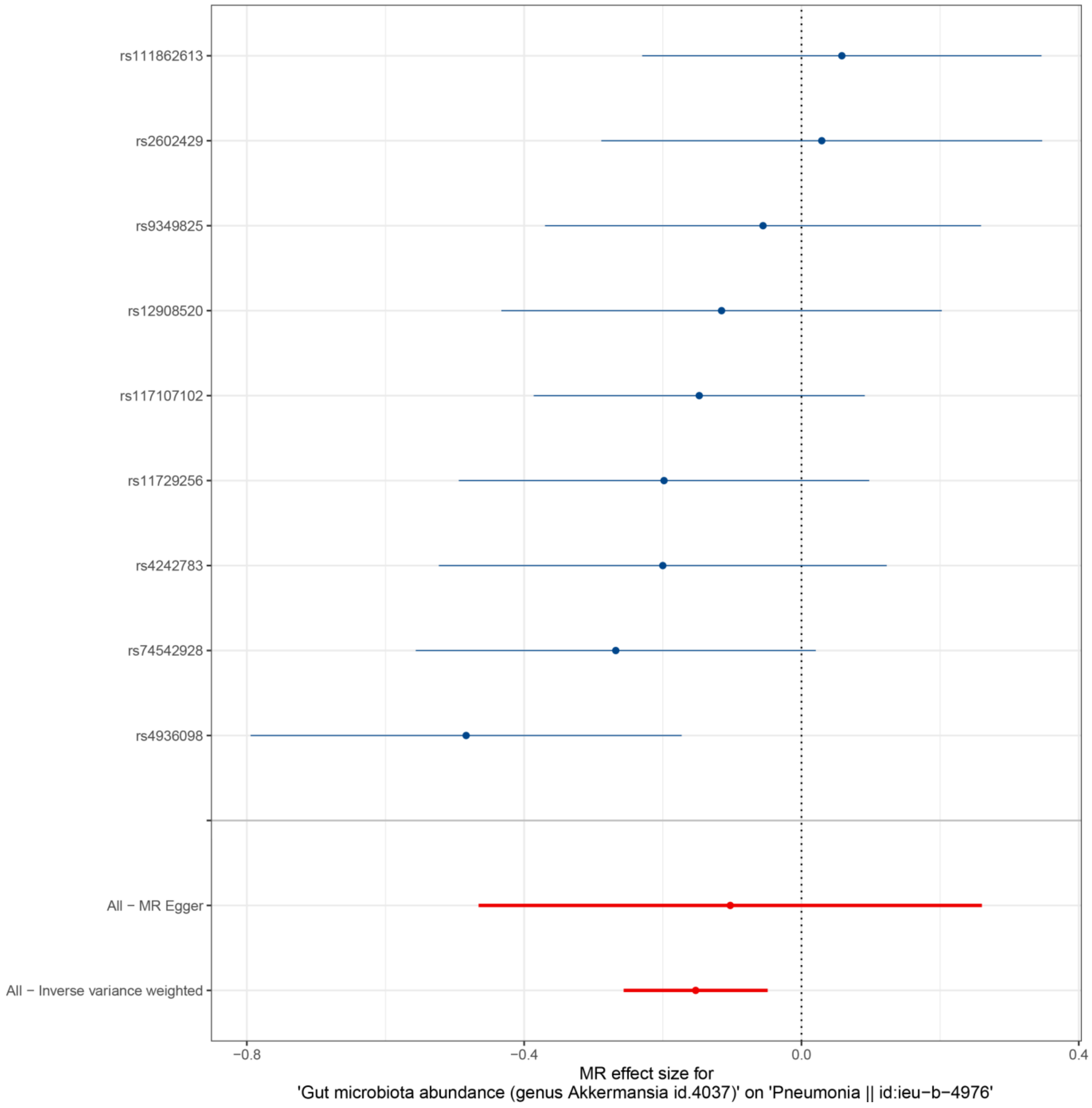
rs111862613
rs2602429
rs9349825
rs12908520
rs117107102
rs11729256
rs4242783
rs74542928
rs4936098
All − MR Egger
All − Inverse variance weighted
−0.8
−0.4
0.0
0.4
MR effect size for
'Gut microbiota abundance (genus Akkermansia id.4037)' on 'Pneumonia || id:ieu−b−4976'

F

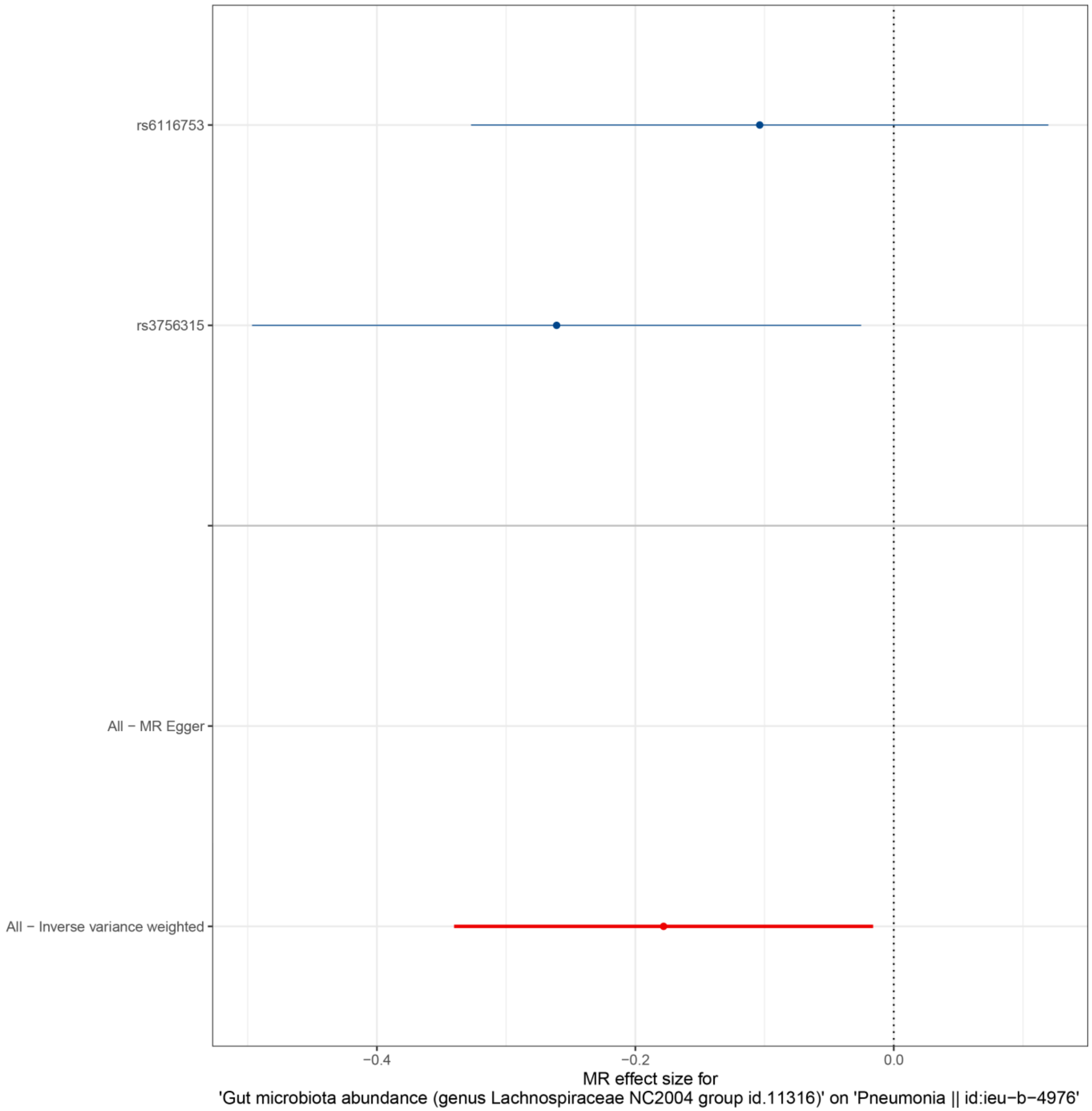

rs6116753
rs3756315
All − MR Egger
All − Inverse variance weighted
−0.4
−0.2
0.0
MR effect size for
'Gut microbiota abundance (genus Lachnospiraceae NC2004 group id.11316)' on 'Pneumonia || id:ieu−b−4976'

G

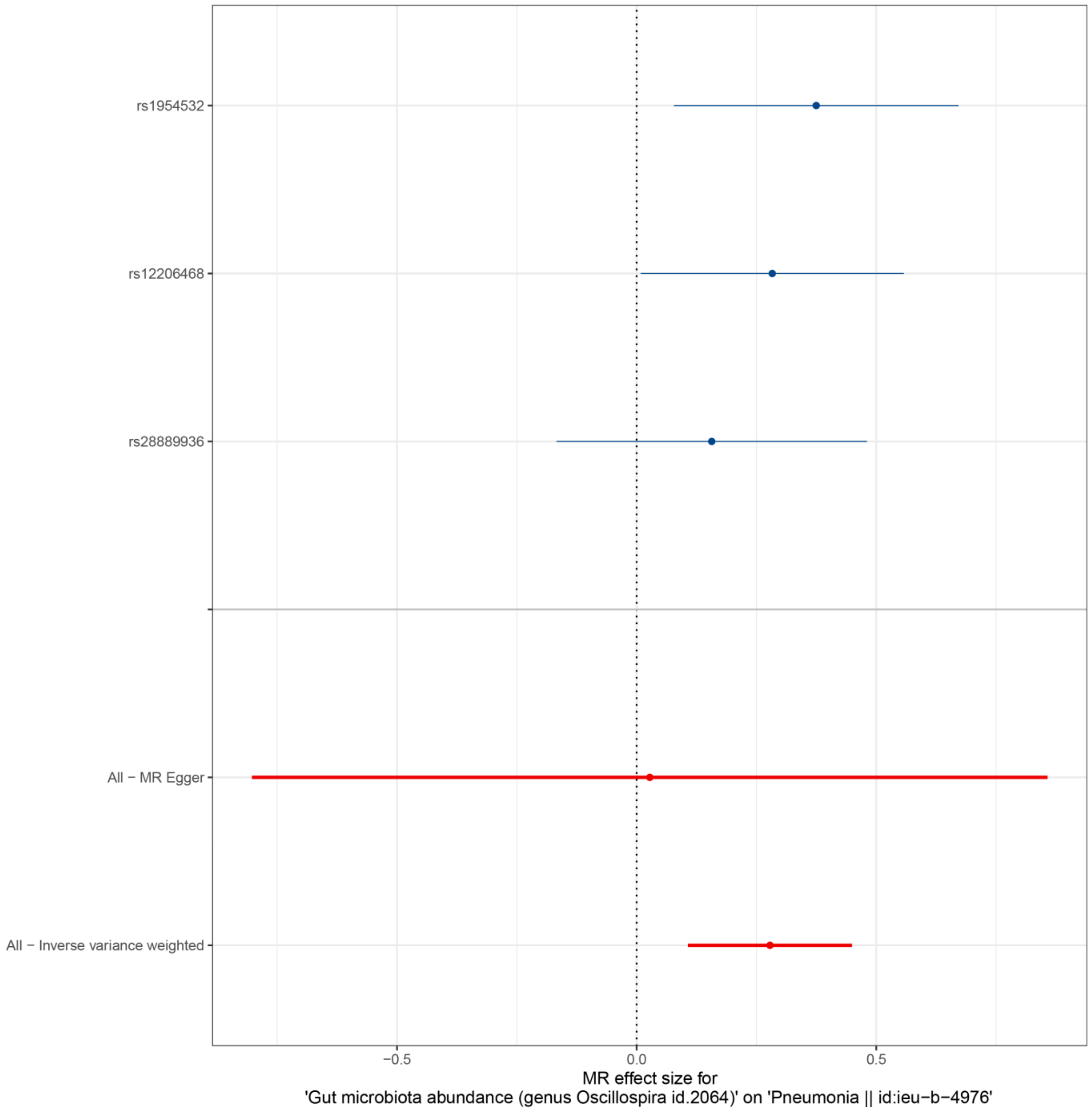

rs1954532
rs12206468
rs28889936
All − MR Egger
All − Inverse variance weighted
−0.5
0.0
0.5
MR effect size for
'Gut microbiota abundance (genus Oscillospira id.2064)' on 'Pneumonia || id:ieu−b−4976'

H

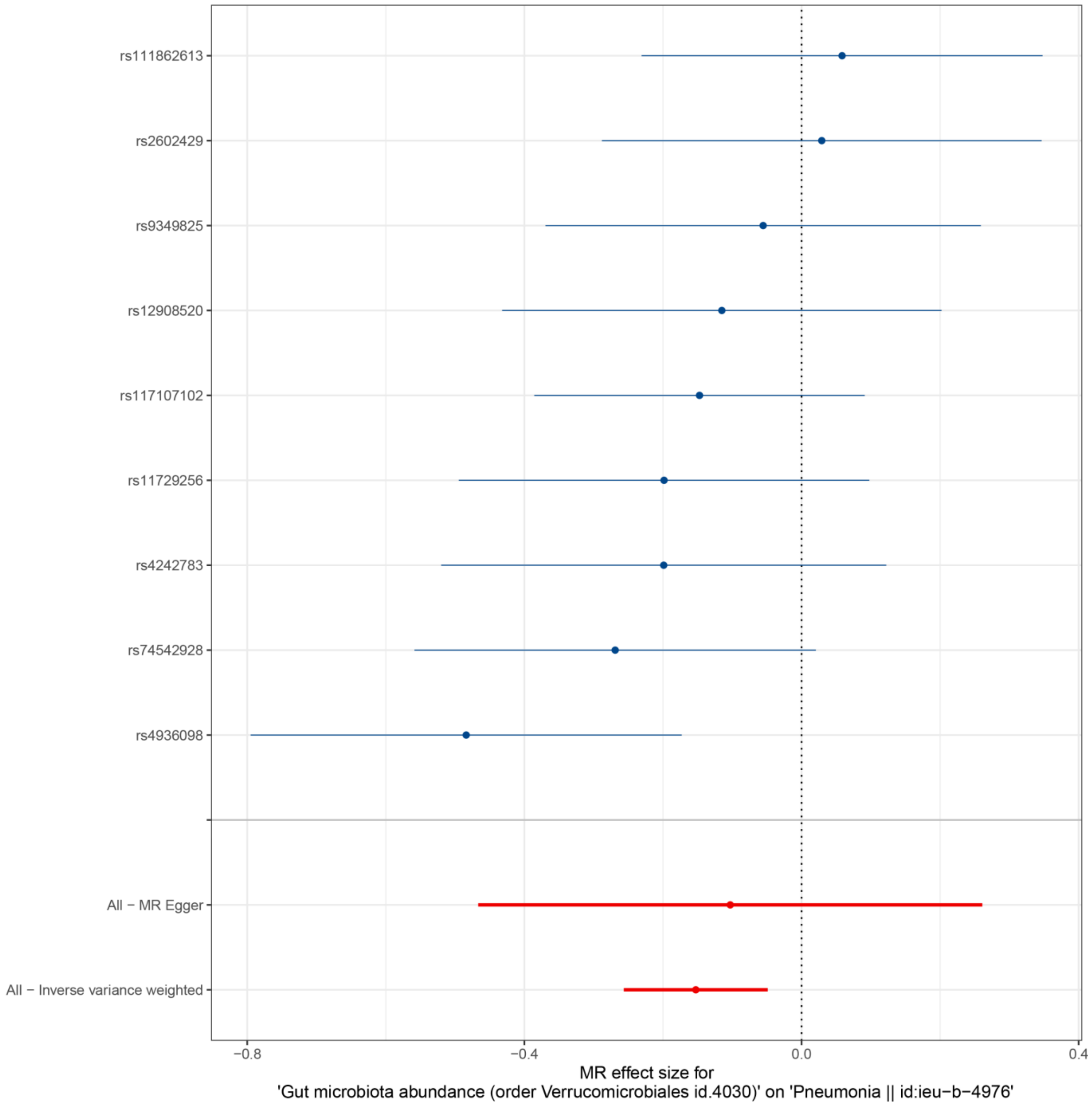

rs111862613
rs2602429
rs9349825
rs12908520
rs117107102
rs11729256
rs4242783
rs74542928
rs4936098
All − MR Egger
All − Inverse variance weighted
−0.8
−0.4
0.0
0.4
MR effect size for
'Gut microbiota abundance (order Verrucomicrobiales id.4030)' on 'Pneumonia || id:ieu−b−4976'

I

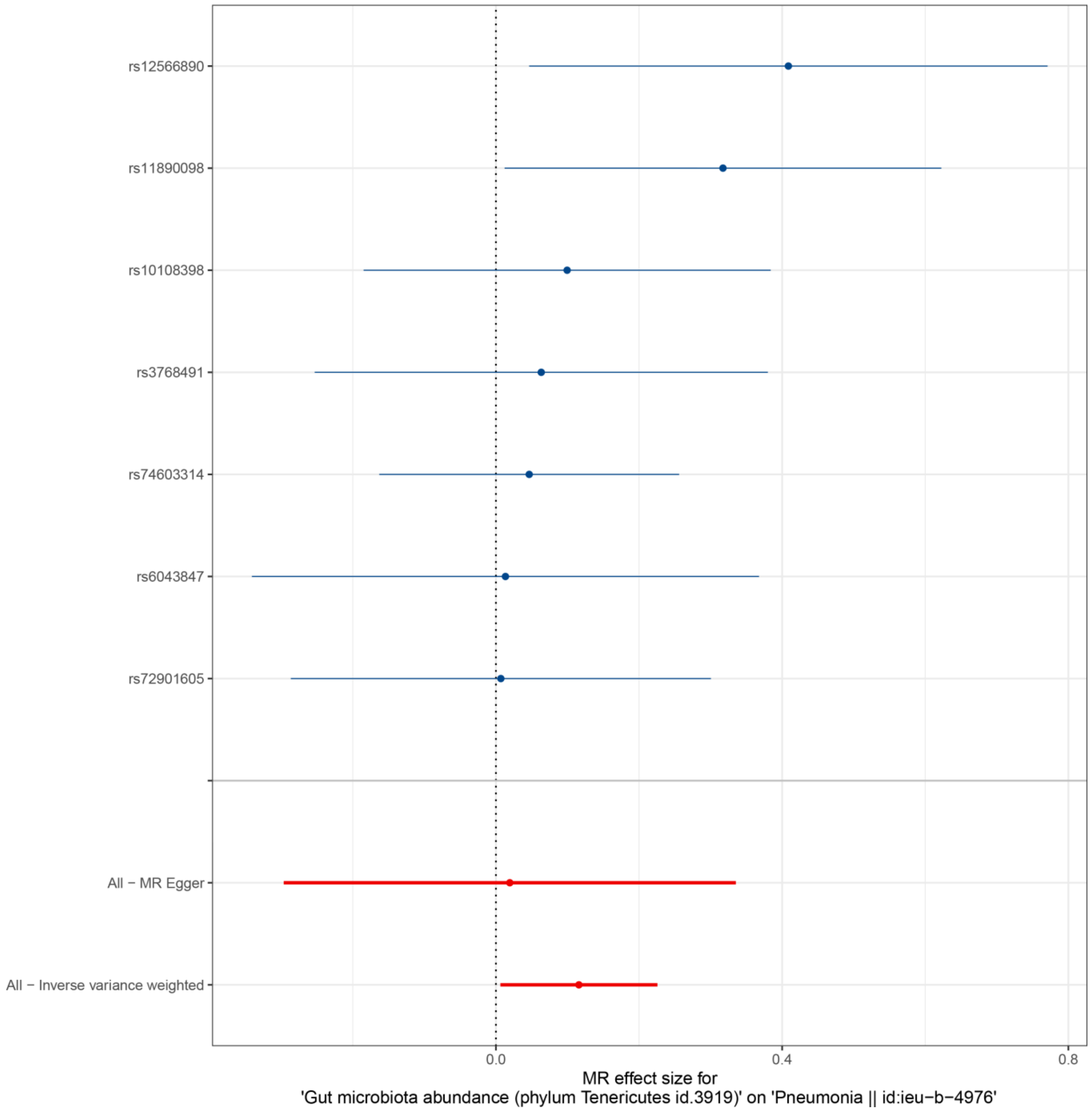

rs12566890
rs11890098
rs10108398
rs3768491
rs74603314
rs6043847
rs72901605
All − MR Egger
All − Inverse variance weighted
0.0
0.4
0.8
MR effect size for
'Gut microbiota abundance (phylum Tenericutes id.3919)' on 'Pneumonia || id:ieu−b−4976'
