# Supplementary material for: Causal Mediation Role of Immune Cells in Gut Microbiota–Pneumonia Associations: A Mendelian Randomisation Study
Source: J Cell Mol Med. 2025 Sep 11;29(17):e70839. doi: 10.1111/jcmm.70839 (PMC12425809; doi:10.1111/jcmm.70839)
Supplement: Supplementary file 7 — Table S1: Information about instrumental variables corresponding to gut microbiota. [file JCMM-29-e70839-s007.docx]

Information about instrumental variables corresponding to gut microbiota

| **Exposure** | **SNPs** | **chr** | **pos** | **EA** | **β** | **SE** | **P** | **F_stat** |
| --- | --- | --- | --- | --- | --- | --- | --- | --- |
| Gut microbiota abundance (class Actinobacteria id.419) | rs11745923 | 5 | 475408 | G | 0.0563759 | 0.0115529 | 1.58E-06 | 23.8125 |
| Gut microbiota abundance (class Actinobacteria id.419) | rs134366 | 22 | 35567355 | A | -0.111877 | 0.023501 | 1.50E-06 | 22.66256 |
| Gut microbiota abundance (class Actinobacteria id.419) | rs1515761 | 10 | 126110032 | C | -0.0762317 | 0.0169884 | 4.96E-06 | 20.13568 |
| Gut microbiota abundance (class Actinobacteria id.419) | rs182549 | 2 | 136616754 | T | -0.111489 | 0.012066 | 3.79E-20 | 85.37631 |
| Gut microbiota abundance (class Actinobacteria id.419) | rs6660520 | 1 | 207003553 | G | -0.0711483 | 0.0134495 | 1.11E-07 | 27.98445 |
| Gut microbiota abundance (class Actinobacteria id.419) | rs72767435 | 15 | 94852795 | T | -0.126352 | 0.0273626 | 2.57E-06 | 21.32306 |
| Gut microbiota abundance (class Actinobacteria id.419) | rs7322849 | 13 | 112859829 | T | 0.0944467 | 0.0192976 | 6.21E-07 | 23.95339 |
| Gut microbiota abundance (class Actinobacteria id.419) | rs8047955 | 16 | 81776768 | G | -0.0575816 | 0.0116762 | 8.34E-07 | 24.32005 |
| Gut microbiota abundance (class Alphaproteobacteria id.2379) | rs140912403 | 9 | 95517807 | C | -0.160677 | 0.0317708 | 6.20E-07 | 25.57709 |
| Gut microbiota abundance (class Alphaproteobacteria id.2379) | rs76784716 | 2 | 169033340 | A | 0.133271 | 0.0267738 | 5.09E-07 | 24.77715 |
| Gut microbiota abundance (class Alphaproteobacteria id.2379) | rs9813022 | 3 | 13726736 | A | -0.0751277 | 0.0153867 | 1.05E-06 | 23.84016 |
| Gut microbiota abundance (class Bacilli id.1673) | rs11110282 | 12 | 100585559 | A | -0.101189 | 0.0217379 | 4.85E-06 | 21.66863 |
| Gut microbiota abundance (class Bacilli id.1673) | rs11730038 | 4 | 98049499 | G | -0.0630747 | 0.0128717 | 1.96E-06 | 24.01257 |
| Gut microbiota abundance (class Bacilli id.1673) | rs4028634 | 17 | 40835649 | C | -0.0520614 | 0.0109842 | 2.21E-06 | 22.4644 |
| Gut microbiota abundance (class Bacilli id.1673) | rs4459992 | 4 | 7431487 | T | 0.0535854 | 0.0116362 | 4.30E-06 | 21.20658 |
| Gut microbiota abundance (class Bacilli id.1673) | rs57872228 | 1 | 200418805 | C | -0.0714773 | 0.0146647 | 9.22E-07 | 23.75691 |
| Gut microbiota abundance (class Bacilli id.1673) | rs77558518 | 5 | 174173171 | A | -0.107255 | 0.0222879 | 1.34E-06 | 23.15777 |
| Gut microbiota abundance (class Bacilli id.1673) | rs78938557 | 7 | 36349586 | T | 0.108007 | 0.0232858 | 1.07E-06 | 21.51402 |
| Gut microbiota abundance (class Bacilli id.1673) | rs9581006 | 13 | 24973509 | T | -0.225309 | 0.0467697 | 1.79E-06 | 23.20748 |
| Gut microbiota abundance (class Bacteroidia id.912) | rs111845179 | 14 | 59317502 | T | 0.102648 | 0.0213843 | 9.24E-07 | 23.04151 |
| Gut microbiota abundance (class Bacteroidia id.912) | rs2032750 | 2 | 53831026 | C | 0.0508393 | 0.0106807 | 1.92E-06 | 22.65686 |
| Gut microbiota abundance (class Bacteroidia id.912) | rs55773148 | 13 | 70523029 | G | -0.121514 | 0.0236759 | 3.90E-07 | 26.34145 |
| Gut microbiota abundance (class Bacteroidia id.912) | rs73975615 | 17 | 6461200 | G | -0.207018 | 0.0442633 | 1.22E-06 | 21.87402 |
| Gut microbiota abundance (class Bacteroidia id.912) | rs7631304 | 3 | 89339527 | G | -0.0645771 | 0.0132957 | 8.37E-07 | 23.59037 |
| Gut microbiota abundance (class Bacteroidia id.912) | rs929878 | 16 | 74290641 | T | 0.0548532 | 0.012153 | 4.73E-06 | 20.37215 |
| Gut microbiota abundance (class Betaproteobacteria id.2867) | rs11128180 | 3 | 70592215 | A | 0.0592944 | 0.0128769 | 3.67E-06 | 21.20336 |
| Gut microbiota abundance (class Betaproteobacteria id.2867) | rs1511453 | 4 | 23588462 | A | 0.0923492 | 0.0199158 | 4.76E-06 | 21.5016 |
| Gut microbiota abundance (class Betaproteobacteria id.2867) | rs1928341 | 1 | 153240013 | G | -0.0525581 | 0.0110408 | 2.02E-06 | 22.66096 |
| Gut microbiota abundance (class Betaproteobacteria id.2867) | rs2613606 | 7 | 111285025 | T | 0.0513106 | 0.0109091 | 2.20E-06 | 22.12261 |
| Gut microbiota abundance (class Betaproteobacteria id.2867) | rs4033856 | 4 | 45642485 | T | -0.0832423 | 0.0167237 | 5.17E-07 | 24.77556 |
| Gut microbiota abundance (class Betaproteobacteria id.2867) | rs6087811 | 20 | 30596130 | T | -0.0978177 | 0.0198522 | 7.44E-07 | 24.27826 |
| Gut microbiota abundance (class Betaproteobacteria id.2867) | rs62395635 | 5 | 173497796 | T | 0.109741 | 0.0236 | 2.94E-06 | 21.62289 |
| Gut microbiota abundance (class Betaproteobacteria id.2867) | rs9964679 | 18 | 26569901 | A | 0.052911 | 0.0114866 | 4.85E-06 | 21.21822 |
| Gut microbiota abundance (class Clostridia id.1859) | rs10774377 | 12 | 5942519 | G | -0.052659 | 0.0113858 | 3.24E-06 | 21.39036 |
| Gut microbiota abundance (class Clostridia id.1859) | rs112334273 | 21 | 40703251 | G | 0.0641008 | 0.0127404 | 3.81E-07 | 25.314 |
| Gut microbiota abundance (class Clostridia id.1859) | rs13179700 | 5 | 149077788 | C | -0.0511937 | 0.0109569 | 3.37E-06 | 21.8302 |
| Gut microbiota abundance (class Clostridia id.1859) | rs6814436 | 4 | 161507301 | C | -0.0739995 | 0.0150815 | 9.65E-07 | 24.07512 |
| Gut microbiota abundance (class Clostridia id.1859) | rs6815608 | 4 | 152131744 | C | -0.103803 | 0.0210883 | 4.02E-07 | 24.22907 |
| Gut microbiota abundance (class Clostridia id.1859) | rs72915163 | 18 | 48792829 | T | -0.0581025 | 0.0120557 | 1.34E-06 | 23.22762 |
| Gut microbiota abundance (class Clostridia id.1859) | rs992074 | 21 | 18567802 | T | -0.255639 | 0.0508787 | 8.78E-07 | 25.2454 |
| Gut microbiota abundance (class Coriobacteriia id.809) | rs11250875 | 10 | 1922731 | T | 0.06075 | 0.0130939 | 4.83E-06 | 21.52556 |
| Gut microbiota abundance (class Coriobacteriia id.809) | rs1816223 | 12 | 11494021 | G | 0.0586265 | 0.0129006 | 4.84E-06 | 20.65229 |
| Gut microbiota abundance (class Coriobacteriia id.809) | rs34739816 | 17 | 37376685 | G | 0.0965017 | 0.0207666 | 3.88E-06 | 21.5943 |
| Gut microbiota abundance (class Coriobacteriia id.809) | rs719099 | 10 | 65799217 | A | 0.0778402 | 0.0155815 | 5.43E-07 | 24.95683 |
| Gut microbiota abundance (class Deltaproteobacteria id.3087) | rs112381107 | 9 | 15269705 | C | 0.206594 | 0.0456908 | 4.63E-06 | 20.44457 |
| Gut microbiota abundance (class Deltaproteobacteria id.3087) | rs11599763 | 10 | 11855599 | C | 0.0544219 | 0.0117397 | 3.94E-06 | 21.48985 |
| Gut microbiota abundance (class Deltaproteobacteria id.3087) | rs17791387 | 9 | 81834426 | A | -0.0735881 | 0.0154246 | 1.60E-06 | 22.76079 |
| Gut microbiota abundance (class Deltaproteobacteria id.3087) | rs2692012 | 1 | 203991605 | G | -0.110335 | 0.0253336 | 3.14E-06 | 18.96849 |
| Gut microbiota abundance (class Deltaproteobacteria id.3087) | rs4506934 | 17 | 2856662 | C | -0.0936514 | 0.02012 | 3.59E-06 | 21.66569 |
| Gut microbiota abundance (class Deltaproteobacteria id.3087) | rs6058181 | 20 | 33694801 | C | 0.0825716 | 0.0165958 | 3.40E-07 | 24.75512 |
| Gut microbiota abundance (class Deltaproteobacteria id.3087) | rs62020470 | 15 | 96161065 | A | -0.0585411 | 0.0129358 | 4.85E-06 | 20.48025 |
| Gut microbiota abundance (class Erysipelotrichia id.2147) | rs10781552 | 10 | 133897233 | C | -0.0552212 | 0.0116074 | 2.33E-06 | 22.63298 |
| Gut microbiota abundance (class Erysipelotrichia id.2147) | rs17530232 | 13 | 40385457 | A | 0.10305 | 0.0224648 | 2.79E-06 | 21.04219 |
| Gut microbiota abundance (class Erysipelotrichia id.2147) | rs2300774 | 3 | 195793712 | A | -0.0524152 | 0.0106784 | 8.95E-07 | 24.09362 |
| Gut microbiota abundance (class Erysipelotrichia id.2147) | rs35161940 | 17 | 70327224 | T | -0.0806041 | 0.0167641 | 1.85E-06 | 23.11819 |
| Gut microbiota abundance (class Erysipelotrichia id.2147) | rs4078432 | 14 | 48997206 | T | 0.0608929 | 0.0133764 | 4.23E-06 | 20.7231 |
| Gut microbiota abundance (class Erysipelotrichia id.2147) | rs62504403 | 8 | 38803551 | C | 0.0681159 | 0.0127883 | 1.12E-07 | 28.37079 |
| Gut microbiota abundance (class Erysipelotrichia id.2147) | rs7234058 | 18 | 5830507 | T | -0.0945759 | 0.0194091 | 9.12E-07 | 23.7438 |
| Gut microbiota abundance (class Erysipelotrichia id.2147) | rs8003149 | 14 | 56156504 | C | 0.0538798 | 0.0116888 | 4.08E-06 | 21.24771 |
| Gut microbiota abundance (class Gammaproteobacteria id.3303) | rs11181912 | 12 | 43572952 | G | -0.0579153 | 0.0118796 | 9.95E-07 | 23.76747 |
| Gut microbiota abundance (class Gammaproteobacteria id.3303) | rs6706173 | 2 | 167986992 | A | 0.074251 | 0.0146392 | 1.99E-07 | 25.72586 |
| Gut microbiota abundance (class Gammaproteobacteria id.3303) | rs9494710 | 6 | 137612554 | C | -0.0549673 | 0.0120924 | 4.55E-06 | 20.66254 |
| Gut microbiota abundance (class Lentisphaeria id.2250) | rs11770843 | 7 | 146795379 | C | 0.109431 | 0.0234879 | 1.91E-06 | 21.70663 |
| Gut microbiota abundance (class Lentisphaeria id.2250) | rs17114848 | 15 | 25162535 | G | 0.152377 | 0.0324332 | 4.06E-06 | 22.07289 |
| Gut microbiota abundance (class Lentisphaeria id.2250) | rs2031282 | 13 | 20687179 | A | 0.122368 | 0.0270329 | 4.38E-06 | 20.4904 |
| Gut microbiota abundance (class Lentisphaeria id.2250) | rs2825714 | 21 | 21023966 | A | -0.13741 | 0.0289246 | 1.72E-06 | 22.56846 |
| Gut microbiota abundance (class Lentisphaeria id.2250) | rs62570196 | 9 | 111086170 | C | -0.21635 | 0.0439866 | 1.08E-06 | 24.19207 |
| Gut microbiota abundance (class Lentisphaeria id.2250) | rs77599476 | 20 | 61394262 | A | 0.230292 | 0.0480168 | 1.86E-06 | 23.0023 |
| Gut microbiota abundance (class Melainabacteria id.1589) | rs11150282 | 16 | 80493705 | T | 0.0989487 | 0.0196972 | 6.03E-07 | 25.23546 |
| Gut microbiota abundance (class Melainabacteria id.1589) | rs4129395 | 9 | 115975389 | G | 0.0896187 | 0.0185106 | 1.48E-06 | 23.43993 |
| Gut microbiota abundance (class Melainabacteria id.1589) | rs79790072 | 15 | 100747683 | T | 0.226722 | 0.0487841 | 3.29E-06 | 21.59886 |
| Gut microbiota abundance (class Melainabacteria id.1589) | rs9864379 | 3 | 14306949 | T | -0.159735 | 0.0292553 | 5.36E-08 | 29.812 |
| Gut microbiota abundance (class Methanobacteria id.119) | rs10202904 | 2 | 125440268 | G | 0.121754 | 0.0235357 | 3.01E-07 | 26.76161 |
| Gut microbiota abundance (class Methanobacteria id.119) | rs6776814 | 3 | 15053083 | T | -0.199566 | 0.0411825 | 1.63E-06 | 23.48268 |
| Gut microbiota abundance (class Methanobacteria id.119) | rs73457410 | 13 | 41956181 | A | 0.21534 | 0.0436695 | 1.41E-06 | 24.31605 |
| Gut microbiota abundance (class Methanobacteria id.119) | rs894996 | 4 | 104418307 | C | 0.216999 | 0.0449076 | 1.88E-06 | 23.3494 |
| Gut microbiota abundance (class Mollicutes id.3920) | rs10108398 | 8 | 59440824 | G | 0.0769142 | 0.0153953 | 1.09E-06 | 24.95955 |
| Gut microbiota abundance (class Mollicutes id.3920) | rs11890098 | 2 | 157532549 | A | 0.074438 | 0.0153389 | 9.57E-07 | 23.55054 |
| Gut microbiota abundance (class Mollicutes id.3920) | rs12566890 | 1 | 61850864 | T | -0.101147 | 0.0230978 | 3.65E-06 | 19.1763 |
| Gut microbiota abundance (class Mollicutes id.3920) | rs3768491 | 1 | 109965986 | G | 0.0681052 | 0.0149061 | 4.23E-06 | 20.87529 |
| Gut microbiota abundance (class Mollicutes id.3920) | rs6043847 | 20 | 16259524 | T | -0.114937 | 0.0248606 | 4.55E-06 | 21.37453 |
| Gut microbiota abundance (class Mollicutes id.3920) | rs72901605 | 11 | 47103877 | T | -0.0841852 | 0.0178119 | 3.26E-06 | 22.33835 |
| Gut microbiota abundance (class Mollicutes id.3920) | rs74603314 | 14 | 46519718 | T | 0.221639 | 0.0462918 | 1.56E-06 | 22.92367 |
| Gut microbiota abundance (class Negativicutes id.2164) | rs13086907 | 3 | 142135117 | G | 0.0625312 | 0.0131735 | 1.95E-06 | 22.53156 |
| Gut microbiota abundance (class Negativicutes id.2164) | rs1643968 | 5 | 165266628 | T | -0.0565284 | 0.0112298 | 4.15E-07 | 25.33899 |
| Gut microbiota abundance (class Negativicutes id.2164) | rs4722181 | 7 | 22817571 | T | 0.0501306 | 0.0105798 | 2.00E-06 | 22.45179 |
| Gut microbiota abundance (class Negativicutes id.2164) | rs60274479 | 16 | 21249925 | T | -0.0659623 | 0.0134137 | 1.16E-06 | 24.18212 |
| Gut microbiota abundance (class Negativicutes id.2164) | rs61249479 | 9 | 124912908 | A | 0.0777075 | 0.0168628 | 2.95E-06 | 21.2357 |
| Gut microbiota abundance (class Negativicutes id.2164) | rs71405394 | 15 | 101245088 | G | -0.114178 | 0.02405 | 2.17E-06 | 22.539 |
| Gut microbiota abundance (class Negativicutes id.2164) | rs73232831 | 4 | 17413426 | G | -0.151829 | 0.0314932 | 1.87E-06 | 23.24213 |
| Gut microbiota abundance (class Verrucomicrobiae id.4029) | rs111862613 | 12 | 130309670 | T | 0.090699 | 0.0196746 | 3.74E-06 | 21.25168 |
| Gut microbiota abundance (class Verrucomicrobiae id.4029) | rs117107102 | 18 | 49473635 | A | 0.204683 | 0.0431572 | 2.92E-06 | 22.4935 |
| Gut microbiota abundance (class Verrucomicrobiae id.4029) | rs11729256 | 4 | 95027272 | T | 0.0749798 | 0.0150177 | 6.73E-07 | 24.92767 |
| Gut microbiota abundance (class Verrucomicrobiae id.4029) | rs12908520 | 15 | 97570657 | G | 0.0618926 | 0.0130946 | 2.17E-06 | 22.3405 |
| Gut microbiota abundance (class Verrucomicrobiae id.4029) | rs2602429 | 16 | 81063149 | T | -0.0746844 | 0.0156194 | 2.58E-06 | 22.86289 |
| Gut microbiota abundance (class Verrucomicrobiae id.4029) | rs4242783 | 10 | 5064327 | A | -0.0689292 | 0.0147693 | 2.64E-06 | 21.78144 |
| Gut microbiota abundance (class Verrucomicrobiae id.4029) | rs4936098 | 11 | 130280667 | G | -0.0648843 | 0.0135928 | 1.12E-06 | 22.78565 |
| Gut microbiota abundance (class Verrucomicrobiae id.4029) | rs74542928 | 4 | 100544188 | T | 0.112164 | 0.0236422 | 1.63E-06 | 22.5077 |
| Gut microbiota abundance (class Verrucomicrobiae id.4029) | rs9349825 | 6 | 56341481 | A | -0.0704036 | 0.0147128 | 2.54E-06 | 22.89808 |
| Gut microbiota abundance (family Acidaminococcaceae id.2166) | rs262812 | 6 | 158647999 | T | -0.065675 | 0.0142257 | 3.25E-06 | 21.31341 |
| Gut microbiota abundance (family Acidaminococcaceae id.2166) | rs2933324 | 9 | 107117653 | G | 0.0662557 | 0.0140257 | 2.24E-06 | 22.31503 |
| Gut microbiota abundance (family Acidaminococcaceae id.2166) | rs6589457 | 11 | 114701288 | G | -0.165911 | 0.0349516 | 2.32E-06 | 22.53286 |
| Gut microbiota abundance (family Acidaminococcaceae id.2166) | rs6923842 | 6 | 5723181 | T | -0.0796106 | 0.0169192 | 2.21E-06 | 22.14023 |
| Gut microbiota abundance (family Actinomycetaceae id.421) | rs2889192 | 9 | 76394568 | T | -0.0887537 | 0.0195009 | 3.64E-06 | 20.71398 |
| Gut microbiota abundance (family Actinomycetaceae id.421) | rs35011108 | 6 | 133007480 | A | 0.241826 | 0.0503796 | 1.83E-06 | 23.04075 |
| Gut microbiota abundance (family Alcaligenaceae id.2875) | rs4033856 | 4 | 45642485 | T | -0.081834 | 0.0168931 | 1.03E-06 | 23.46653 |
| Gut microbiota abundance (family Alcaligenaceae id.2875) | rs62191117 | 2 | 239900775 | A | 0.0684302 | 0.0133636 | 2.76E-07 | 26.22097 |
| Gut microbiota abundance (family Alcaligenaceae id.2875) | rs62395635 | 5 | 173497796 | T | 0.110583 | 0.0238514 | 3.35E-06 | 21.49557 |
| Gut microbiota abundance (family Alcaligenaceae id.2875) | rs6969323 | 7 | 104214487 | A | -0.0593564 | 0.0129137 | 3.89E-06 | 21.1268 |
| Gut microbiota abundance (family Alcaligenaceae id.2875) | rs7638039 | 3 | 70588939 | T | 0.0603335 | 0.0127779 | 2.70E-06 | 22.29452 |
| Gut microbiota abundance (family Alcaligenaceae id.2875) | rs9964679 | 18 | 26569901 | A | 0.0537051 | 0.0116008 | 4.47E-06 | 21.43163 |
| Gut microbiota abundance (family Bacteroidaceae id.917) | rs11585893 | 1 | 10644351 | A | -0.0740746 | 0.0147633 | 1.80E-06 | 25.17513 |
| Gut microbiota abundance (family Bacteroidaceae id.917) | rs17619981 | 19 | 24342250 | T | 0.0880978 | 0.0187002 | 2.69E-06 | 22.1941 |
| Gut microbiota abundance (family Bacteroidaceae id.917) | rs6795673 | 3 | 10593224 | C | 0.0538565 | 0.0105251 | 3.38E-07 | 26.18326 |
| Gut microbiota abundance (family Bacteroidaceae id.917) | rs9507307 | 13 | 24910476 | C | 0.0604456 | 0.0129128 | 2.13E-06 | 21.91233 |
| Gut microbiota abundance (family Bacteroidales S24 7group id.11173) | rs17043785 | 2 | 53139362 | T | -0.176187 | 0.0347133 | 5.12E-07 | 25.7606 |
| Gut microbiota abundance (family Bacteroidales S24 7group id.11173) | rs738193 | 22 | 25943322 | T | 0.0847328 | 0.0165856 | 3.82E-07 | 26.10001 |
| Gut microbiota abundance (family Bifidobacteriaceae id.433) | rs13020688 | 2 | 192878532 | G | 0.0584045 | 0.0122081 | 1.57E-06 | 22.8874 |
| Gut microbiota abundance (family Bifidobacteriaceae id.433) | rs182549 | 2 | 136616754 | T | -0.11707 | 0.0126703 | 5.94E-20 | 85.37239 |
| Gut microbiota abundance (family Bifidobacteriaceae id.433) | rs4957061 | 5 | 521096 | T | 0.0569873 | 0.0116907 | 1.15E-06 | 23.76157 |
| Gut microbiota abundance (family Bifidobacteriaceae id.433) | rs7322849 | 13 | 112859829 | T | 0.110676 | 0.0200996 | 1.74E-08 | 30.3202 |
| Gut microbiota abundance (family Bifidobacteriaceae id.433) | rs73797465 | 5 | 142793467 | T | -0.0942612 | 0.0208421 | 4.85E-06 | 20.45422 |
| Gut microbiota abundance (family Bifidobacteriaceae id.433) | rs857444 | 6 | 14617591 | C | 0.0553965 | 0.0120669 | 3.82E-06 | 21.07527 |
| Gut microbiota abundance (family Clostridiaceae1 id.1869) | rs12341505 | 9 | 136710881 | G | 0.0814503 | 0.0179338 | 4.54E-06 | 20.62722 |
| Gut microbiota abundance (family Clostridiaceae1 id.1869) | rs2795528 | 10 | 43270264 | G | -0.180969 | 0.0390911 | 3.81E-06 | 21.4315 |
| Gut microbiota abundance (family Clostridiales vadin BB60 group id.11286) | rs118104867 | 8 | 123977538 | C | 0.214464 | 0.0455098 | 3.44E-06 | 22.20746 |
| Gut microbiota abundance (family Clostridiales vadin BB60 group id.11286) | rs13409132 | 2 | 205616346 | A | -0.165419 | 0.0352154 | 4.37E-06 | 22.06508 |
| Gut microbiota abundance (family Clostridiales vadin BB60 group id.11286) | rs2191834 | 2 | 229942951 | T | -0.0746375 | 0.0159136 | 2.50E-06 | 21.9977 |
| Gut microbiota abundance (family Clostridiales vadin BB60 group id.11286) | rs28691777 | 17 | 58149405 | C | 0.137134 | 0.0266996 | 6.96E-07 | 26.38036 |
| Gut microbiota abundance (family Clostridiales vadin BB60 group id.11286) | rs55682560 | 15 | 87392290 | C | -0.131519 | 0.0261319 | 4.97E-07 | 25.32999 |
| Gut microbiota abundance (family Clostridiales vadin BB60 group id.11286) | rs6588624 | 1 | 56849539 | A | 0.0662317 | 0.0138147 | 1.79E-06 | 22.98523 |
| Gut microbiota abundance (family Clostridiales vadin BB60 group id.11286) | rs66714985 | 8 | 3305122 | A | 0.116908 | 0.0252447 | 4.85E-06 | 21.44609 |
| Gut microbiota abundance (family Clostridiales vadin BB60 group id.11286) | rs7226487 | 18 | 74373125 | A | -0.0643682 | 0.0138701 | 3.58E-06 | 21.53692 |
| Gut microbiota abundance (family Clostridiales vadin BB60 group id.11286) | rs7538034 | 1 | 71391694 | T | -0.078598 | 0.0165982 | 2.37E-06 | 22.42338 |
| Gut microbiota abundance (family Clostridiales vadin BB60 group id.11286) | rs7725895 | 5 | 142244898 | A | -0.116224 | 0.0240367 | 3.94E-06 | 23.37986 |
| Gut microbiota abundance (family Coriobacteriaceae id.811) | rs11250875 | 10 | 1922731 | T | 0.06075 | 0.0130939 | 4.83E-06 | 21.52556 |
| Gut microbiota abundance (family Coriobacteriaceae id.811) | rs1816223 | 12 | 11494021 | G | 0.0586265 | 0.0129006 | 4.84E-06 | 20.65229 |
| Gut microbiota abundance (family Coriobacteriaceae id.811) | rs34739816 | 17 | 37376685 | G | 0.0965017 | 0.0207666 | 3.88E-06 | 21.5943 |
| Gut microbiota abundance (family Coriobacteriaceae id.811) | rs719099 | 10 | 65799217 | A | 0.0778402 | 0.0155815 | 5.43E-07 | 24.95683 |
| Gut microbiota abundance (family Defluviitaleaceae id.1924) | rs112893842 | 9 | 8786663 | T | 0.110761 | 0.0232545 | 2.75E-06 | 22.68609 |
| Gut microbiota abundance (family Defluviitaleaceae id.1924) | rs17051335 | 4 | 122202910 | C | -0.134314 | 0.0292201 | 4.58E-06 | 21.12901 |
| Gut microbiota abundance (family Defluviitaleaceae id.1924) | rs4677103 | 3 | 72207794 | A | 0.0977203 | 0.0197033 | 9.42E-07 | 24.59754 |
| Gut microbiota abundance (family Defluviitaleaceae id.1924) | rs55658617 | 21 | 41811003 | T | 0.177314 | 0.0361844 | 1.41E-06 | 24.01283 |
| Gut microbiota abundance (family Defluviitaleaceae id.1924) | rs72731813 | 4 | 147414743 | C | -0.149751 | 0.0293415 | 2.76E-07 | 26.04803 |
| Gut microbiota abundance (family Defluviitaleaceae id.1924) | rs9608282 | 22 | 24804081 | T | 0.139014 | 0.0299429 | 4.61E-06 | 21.55407 |
| Gut microbiota abundance (family Defluviitaleaceae id.1924) | rs9725395 | 1 | 85205632 | A | -0.138357 | 0.0295185 | 3.41E-06 | 21.96917 |
| Gut microbiota abundance (family Desulfovibrionaceae id.3169) | rs112381107 | 9 | 15269705 | C | 0.211435 | 0.0456962 | 2.82E-06 | 21.40886 |
| Gut microbiota abundance (family Desulfovibrionaceae id.3169) | rs11599763 | 10 | 11855599 | C | 0.0555931 | 0.0117493 | 2.50E-06 | 22.38813 |
| Gut microbiota abundance (family Desulfovibrionaceae id.3169) | rs17791387 | 9 | 81834426 | A | -0.0729251 | 0.0154381 | 2.10E-06 | 22.31343 |
| Gut microbiota abundance (family Desulfovibrionaceae id.3169) | rs2692012 | 1 | 203991605 | G | -0.114231 | 0.0253568 | 1.56E-06 | 20.29453 |
| Gut microbiota abundance (family Desulfovibrionaceae id.3169) | rs2838334 | 21 | 45064961 | G | 0.0571419 | 0.012423 | 3.82E-06 | 21.15711 |
| Gut microbiota abundance (family Desulfovibrionaceae id.3169) | rs4506934 | 17 | 2856662 | C | -0.0943381 | 0.020138 | 3.16E-06 | 21.9453 |
| Gut microbiota abundance (family Desulfovibrionaceae id.3169) | rs6058181 | 20 | 33694801 | C | 0.0834742 | 0.0166111 | 2.70E-07 | 25.2527 |
| Gut microbiota abundance (family Desulfovibrionaceae id.3169) | rs9928243 | 16 | 71541641 | C | -0.0541732 | 0.0117819 | 4.48E-06 | 21.14162 |
| Gut microbiota abundance (family Enterobacteriaceae id.3469) | rs2374342 | 2 | 42133542 | C | 0.0582927 | 0.0126195 | 4.52E-06 | 21.33753 |
| Gut microbiota abundance (family Enterobacteriaceae id.3469) | rs62210023 | 20 | 55340092 | A | 0.0606754 | 0.0130125 | 3.13E-06 | 21.74222 |
| Gut microbiota abundance (family Erysipelotrichaceae id.2149) | rs10781552 | 10 | 133897233 | C | -0.0552212 | 0.0116074 | 2.33E-06 | 22.63298 |
| Gut microbiota abundance (family Erysipelotrichaceae id.2149) | rs17530232 | 13 | 40385457 | A | 0.10305 | 0.0224648 | 2.79E-06 | 21.04219 |
| Gut microbiota abundance (family Erysipelotrichaceae id.2149) | rs2300774 | 3 | 195793712 | A | -0.0524152 | 0.0106784 | 8.95E-07 | 24.09362 |
| Gut microbiota abundance (family Erysipelotrichaceae id.2149) | rs35161940 | 17 | 70327224 | T | -0.0806041 | 0.0167641 | 1.85E-06 | 23.11819 |
| Gut microbiota abundance (family Erysipelotrichaceae id.2149) | rs4078432 | 14 | 48997206 | T | 0.0608929 | 0.0133764 | 4.23E-06 | 20.7231 |
| Gut microbiota abundance (family Erysipelotrichaceae id.2149) | rs62504403 | 8 | 38803551 | C | 0.0681159 | 0.0127883 | 1.12E-07 | 28.37079 |
| Gut microbiota abundance (family Erysipelotrichaceae id.2149) | rs7234058 | 18 | 5830507 | T | -0.0945759 | 0.0194091 | 9.12E-07 | 23.7438 |
| Gut microbiota abundance (family Erysipelotrichaceae id.2149) | rs8003149 | 14 | 56156504 | C | 0.0538798 | 0.0116888 | 4.08E-06 | 21.24771 |
| Gut microbiota abundance (family Family XI id.1936) | rs10759623 | 9 | 115821485 | C | -0.162115 | 0.032151 | 5.78E-07 | 25.42479 |
| Gut microbiota abundance (family Family XI id.1936) | rs11547158 | 7 | 148921732 | A | -0.177599 | 0.0372795 | 2.70E-06 | 22.69556 |
| Gut microbiota abundance (family Family XI id.1936) | rs17379710 | 11 | 35334668 | T | -0.116383 | 0.0252125 | 3.97E-06 | 21.30822 |
| Gut microbiota abundance (family Family XI id.1936) | rs2155352 | 11 | 95357209 | A | -0.150538 | 0.0302319 | 6.63E-07 | 24.79485 |
| Gut microbiota abundance (family Family XI id.1936) | rs3733511 | 4 | 119955787 | A | 0.128253 | 0.0274718 | 3.39E-06 | 21.7952 |
| Gut microbiota abundance (family Family XI id.1936) | rs488164 | 1 | 239995451 | G | -0.117998 | 0.025516 | 4.80E-06 | 21.38573 |
| Gut microbiota abundance (family Family XI id.1936) | rs697771 | 16 | 54115200 | A | -0.117677 | 0.0251491 | 3.19E-06 | 21.89466 |
| Gut microbiota abundance (family Family XIII id.1957) | rs118170811 | 10 | 102366896 | A | 0.151619 | 0.0317112 | 1.80E-06 | 22.8603 |
| Gut microbiota abundance (family Family XIII id.1957) | rs482905 | 1 | 166557202 | G | 0.0596528 | 0.012717 | 3.72E-06 | 22.00353 |
| Gut microbiota abundance (family Family XIII id.1957) | rs6501525 | 17 | 70218627 | A | 0.0561559 | 0.0115587 | 1.24E-06 | 23.6033 |
| Gut microbiota abundance (family Family XIII id.1957) | rs6797051 | 3 | 88995218 | C | -0.0805985 | 0.0171364 | 4.89E-06 | 22.12151 |
| Gut microbiota abundance (family Family XIII id.1957) | rs7514702 | 1 | 186914023 | T | -0.0663236 | 0.0141626 | 3.92E-06 | 21.93058 |
| Gut microbiota abundance (family Lachnospiraceae id.1987) | rs11139361 | 9 | 72179847 | C | -0.0494463 | 0.0110503 | 4.26E-06 | 20.02255 |
| Gut microbiota abundance (family Lachnospiraceae id.1987) | rs112040820 | 17 | 80469064 | A | 0.0549584 | 0.0117086 | 2.42E-06 | 22.03222 |
| Gut microbiota abundance (family Lachnospiraceae id.1987) | rs11979110 | 7 | 130436459 | T | -0.0500924 | 0.0105069 | 1.82E-06 | 22.72974 |
| Gut microbiota abundance (family Lachnospiraceae id.1987) | rs2159863 | 4 | 10245354 | A | -0.0585831 | 0.0128742 | 3.70E-06 | 20.70638 |
| Gut microbiota abundance (family Lachnospiraceae id.1987) | rs35524804 | 9 | 100113683 | T | -0.0607481 | 0.0125216 | 2.45E-06 | 23.53671 |
| Gut microbiota abundance (family Lachnospiraceae id.1987) | rs9929145 | 16 | 76559184 | G | -0.125721 | 0.0245233 | 2.84E-07 | 26.28196 |
| Gut microbiota abundance (family Lactobacillaceae id.1836) | rs11674854 | 2 | 28903275 | C | -0.0830224 | 0.0175267 | 2.60E-06 | 22.43832 |
| Gut microbiota abundance (family Lactobacillaceae id.1836) | rs16861661 | 1 | 18501459 | G | -0.193287 | 0.0379293 | 2.70E-07 | 25.96902 |
| Gut microbiota abundance (family Lactobacillaceae id.1836) | rs768253 | 8 | 69010103 | T | -0.0792344 | 0.0170604 | 3.61E-06 | 21.56995 |
| Gut microbiota abundance (family Lactobacillaceae id.1836) | rs921925 | 19 | 6928017 | A | 0.0999419 | 0.0201687 | 5.77E-07 | 24.55497 |
| Gut microbiota abundance (family Methanobacteriaceae id.121) | rs10202904 | 2 | 125440268 | G | 0.121754 | 0.0235357 | 3.01E-07 | 26.76161 |
| Gut microbiota abundance (family Methanobacteriaceae id.121) | rs6776814 | 3 | 15053083 | T | -0.199566 | 0.0411825 | 1.63E-06 | 23.48268 |
| Gut microbiota abundance (family Methanobacteriaceae id.121) | rs73457410 | 13 | 41956181 | A | 0.21534 | 0.0436695 | 1.41E-06 | 24.31605 |
| Gut microbiota abundance (family Methanobacteriaceae id.121) | rs894996 | 4 | 104418307 | C | 0.216999 | 0.0449076 | 1.88E-06 | 23.3494 |
| Gut microbiota abundance (family Oxalobacteraceae id.2966) | rs111966731 | 15 | 93941937 | T | 0.204031 | 0.0445746 | 4.56E-06 | 20.95161 |
| Gut microbiota abundance (family Oxalobacteraceae id.2966) | rs11246212 | 11 | 610277 | C | -0.13607 | 0.0291797 | 4.51E-06 | 21.74519 |
| Gut microbiota abundance (family Oxalobacteraceae id.2966) | rs1569853 | 6 | 38550301 | T | -0.140043 | 0.0281704 | 7.45E-07 | 24.71364 |
| Gut microbiota abundance (family Oxalobacteraceae id.2966) | rs4428215 | 3 | 171947435 | G | 0.12561 | 0.0230053 | 4.88E-08 | 29.8121 |
| Gut microbiota abundance (family Oxalobacteraceae id.2966) | rs6000536 | 22 | 37421469 | C | -0.118383 | 0.0241355 | 7.39E-07 | 24.05836 |
| Gut microbiota abundance (family Oxalobacteraceae id.2966) | rs736744 | 9 | 87514407 | T | -0.105642 | 0.0200648 | 1.49E-07 | 27.72066 |
| Gut microbiota abundance (family Oxalobacteraceae id.2966) | rs934049 | 2 | 16056779 | G | 0.109751 | 0.0238636 | 4.21E-06 | 21.15169 |
| Gut microbiota abundance (family Pasteurellaceae id.3689) | rs10965428 | 9 | 22718481 | C | -0.119902 | 0.0258219 | 4.29E-06 | 21.56138 |
| Gut microbiota abundance (family Pasteurellaceae id.3689) | rs35510 | 12 | 115491973 | A | 0.122729 | 0.0264958 | 4.02E-06 | 21.45558 |
| Gut microbiota abundance (family Pasteurellaceae id.3689) | rs4822728 | 22 | 26891808 | T | 0.0685479 | 0.0149032 | 4.72E-06 | 21.15579 |
| Gut microbiota abundance (family Pasteurellaceae id.3689) | rs72756943 | 5 | 26531908 | G | 0.13988 | 0.0303029 | 3.35E-06 | 21.30801 |
| Gut microbiota abundance (family Pasteurellaceae id.3689) | rs76022354 | 10 | 94306385 | C | 0.24289 | 0.0500406 | 1.83E-06 | 23.55994 |
| Gut microbiota abundance (family Pasteurellaceae id.3689) | rs78909003 | 9 | 105650242 | T | -0.241159 | 0.0498372 | 2.05E-06 | 23.4153 |
| Gut microbiota abundance (family Pasteurellaceae id.3689) | rs9382510 | 6 | 55448491 | C | -0.0881865 | 0.0169965 | 2.48E-07 | 26.92063 |
| Gut microbiota abundance (family Peptococcaceae id.2024) | rs117452796 | 9 | 9423378 | A | -0.257982 | 0.0549569 | 3.15E-06 | 22.03608 |
| Gut microbiota abundance (family Peptococcaceae id.2024) | rs12634826 | 3 | 182984048 | T | -0.0739885 | 0.0150931 | 1.01E-06 | 24.03098 |
| Gut microbiota abundance (family Peptococcaceae id.2024) | rs12992764 | 2 | 188895743 | T | 0.0684213 | 0.0141137 | 1.46E-06 | 23.50179 |
| Gut microbiota abundance (family Peptococcaceae id.2024) | rs150600492 | 10 | 129175962 | A | 0.135774 | 0.0289757 | 2.31E-06 | 21.95661 |
| Gut microbiota abundance (family Peptococcaceae id.2024) | rs35703006 | 8 | 28614217 | G | 0.0812655 | 0.0164355 | 4.95E-07 | 24.44818 |
| Gut microbiota abundance (family Peptococcaceae id.2024) | rs75430375 | 5 | 89252884 | C | -0.147941 | 0.0317498 | 3.41E-06 | 21.71176 |
| Gut microbiota abundance (family Peptococcaceae id.2024) | rs75898026 | 13 | 113561082 | A | -0.0821779 | 0.0173541 | 2.02E-06 | 22.42363 |
| Gut microbiota abundance (family Peptostreptococcaceae id.2042) | rs10805326 | 4 | 14324623 | A | -0.0566637 | 0.012276 | 4.03E-06 | 21.30571 |
| Gut microbiota abundance (family Peptostreptococcaceae id.2042) | rs117020988 | 7 | 46710977 | C | 0.182411 | 0.0372126 | 1.03E-06 | 24.02824 |
| Gut microbiota abundance (family Peptostreptococcaceae id.2042) | rs12377846 | 9 | 16786784 | C | -0.252 | 0.0511627 | 7.26E-07 | 24.26019 |
| Gut microbiota abundance (family Peptostreptococcaceae id.2042) | rs4692811 | 4 | 171180190 | C | 0.0642075 | 0.0126913 | 4.21E-07 | 25.59525 |
| Gut microbiota abundance (family Peptostreptococcaceae id.2042) | rs59987323 | 22 | 37976422 | C | -0.0537078 | 0.011443 | 2.70E-06 | 22.02901 |
| Gut microbiota abundance (family Peptostreptococcaceae id.2042) | rs61841503 | 10 | 17019559 | G | 0.0919658 | 0.0161331 | 9.80E-09 | 32.49504 |
| Gut microbiota abundance (family Peptostreptococcaceae id.2042) | rs76982728 | 7 | 44312824 | T | 0.124203 | 0.0266923 | 3.24E-06 | 21.65171 |
| Gut microbiota abundance (family Peptostreptococcaceae id.2042) | rs9573937 | 13 | 77351196 | A | -0.0693637 | 0.0142907 | 1.71E-06 | 23.55904 |
| Gut microbiota abundance (family Porphyromonadaceae id.943) | rs10858364 | 9 | 138076081 | G | 0.055316 | 0.012085 | 4.31E-06 | 20.95117 |
| Gut microbiota abundance (family Porphyromonadaceae id.943) | rs17065783 | 3 | 62035586 | A | -0.059133 | 0.0122235 | 1.79E-06 | 23.40285 |
| Gut microbiota abundance (family Porphyromonadaceae id.943) | rs6953849 | 7 | 69251692 | A | 0.0718149 | 0.0150724 | 2.44E-06 | 22.70201 |
| Gut microbiota abundance (family Prevotellaceae id.960) | rs12118202 | 1 | 210681370 | T | -0.0751986 | 0.0147265 | 5.54E-07 | 26.07477 |
| Gut microbiota abundance (family Prevotellaceae id.960) | rs148376875 | 3 | 170126707 | T | 0.084515 | 0.0179163 | 2.08E-06 | 22.2521 |
| Gut microbiota abundance (family Prevotellaceae id.960) | rs2206482 | 20 | 9771109 | T | -0.0569105 | 0.0117486 | 1.30E-06 | 23.46457 |
| Gut microbiota abundance (family Prevotellaceae id.960) | rs3860225 | 1 | 111161677 | A | 0.083936 | 0.0167936 | 5.50E-07 | 24.98095 |
| Gut microbiota abundance (family Prevotellaceae id.960) | rs4493272 | 2 | 118910912 | T | -0.0604992 | 0.0118027 | 3.02E-07 | 26.27463 |
| Gut microbiota abundance (family Prevotellaceae id.960) | rs4685827 | 3 | 4864877 | T | -0.0679529 | 0.0145407 | 2.77E-06 | 21.83963 |
| Gut microbiota abundance (family Prevotellaceae id.960) | rs912860 | 14 | 33706908 | A | 0.229046 | 0.0483124 | 9.30E-07 | 22.47647 |
| Gut microbiota abundance (family Prevotellaceae id.960) | rs9586501 | 13 | 105075823 | G | 0.0592681 | 0.0127329 | 2.59E-06 | 21.66643 |
| Gut microbiota abundance (family Prevotellaceae id.960) | rs9958960 | 18 | 63018983 | G | -0.0914807 | 0.0173611 | 1.06E-07 | 27.76543 |
| Gut microbiota abundance (family Rhodospirillaceae id.2717) | rs11591293 | 10 | 113419797 | G | 0.0743184 | 0.0158724 | 2.67E-06 | 21.92338 |
| Gut microbiota abundance (family Rhodospirillaceae id.2717) | rs1549633 | 5 | 27945645 | A | 0.0999312 | 0.0218634 | 4.70E-06 | 20.89136 |
| Gut microbiota abundance (family Rhodospirillaceae id.2717) | rs3754624 | 2 | 225633812 | C | 0.0971389 | 0.0200064 | 1.71E-06 | 23.57482 |
| Gut microbiota abundance (family Rhodospirillaceae id.2717) | rs4278423 | 10 | 2670553 | T | 0.107529 | 0.0235725 | 3.12E-06 | 20.80846 |
| Gut microbiota abundance (family Rhodospirillaceae id.2717) | rs55876211 | 3 | 84471266 | C | -0.0911851 | 0.0196395 | 2.87E-06 | 21.55693 |
| Gut microbiota abundance (family Rhodospirillaceae id.2717) | rs76784716 | 2 | 169033340 | A | 0.136049 | 0.0285767 | 1.49E-06 | 22.66557 |
| Gut microbiota abundance (family Rhodospirillaceae id.2717) | rs9813022 | 3 | 13726736 | A | -0.0842444 | 0.0163583 | 2.53E-07 | 26.52197 |
| Gut microbiota abundance (family Rikenellaceae id.967) | rs1939881 | 11 | 95304834 | G | -0.105589 | 0.0206213 | 5.64E-07 | 26.21834 |
| Gut microbiota abundance (family Rikenellaceae id.967) | rs2833282 | 21 | 32496710 | G | 0.0711137 | 0.0156966 | 4.31E-06 | 20.52558 |
| Gut microbiota abundance (family Rikenellaceae id.967) | rs4264350 | 15 | 71658642 | T | -0.0526393 | 0.0108555 | 1.35E-06 | 23.51368 |
| Gut microbiota abundance (family Rikenellaceae id.967) | rs62532512 | 9 | 14158854 | A | 0.0504257 | 0.0107399 | 2.76E-06 | 22.04466 |
| Gut microbiota abundance (family Rikenellaceae id.967) | rs67705352 | 1 | 19790706 | T | -0.0549688 | 0.0110534 | 6.58E-07 | 24.73095 |
| Gut microbiota abundance (family Rikenellaceae id.967) | rs6837275 | 4 | 187842232 | A | 0.0570355 | 0.011887 | 1.45E-06 | 23.02216 |
| Gut microbiota abundance (family Rikenellaceae id.967) | rs74474130 | 14 | 90270821 | T | 0.137747 | 0.0295688 | 3.61E-06 | 21.70186 |
| Gut microbiota abundance (family Rikenellaceae id.967) | rs77885767 | 14 | 21548634 | C | -0.156209 | 0.0336482 | 2.85E-06 | 21.55204 |
| Gut microbiota abundance (family Rikenellaceae id.967) | rs9578457 | 13 | 22867356 | G | -0.141478 | 0.0315848 | 3.99E-06 | 20.06419 |
| Gut microbiota abundance (family Rikenellaceae id.967) | rs9603208 | 13 | 38044689 | G | 0.0821173 | 0.0159596 | 1.92E-07 | 26.47435 |
| Gut microbiota abundance (family Ruminococcaceae id.2050) | rs1612733 | 1 | 107661606 | T | 0.108708 | 0.0238006 | 4.22E-06 | 20.86158 |
| Gut microbiota abundance (family Ruminococcaceae id.2050) | rs17376049 | 1 | 61425267 | T | 0.0848534 | 0.0172114 | 7.30E-07 | 24.30559 |
| Gut microbiota abundance (family Ruminococcaceae id.2050) | rs2113833 | 2 | 218216801 | C | -0.169146 | 0.0355009 | 1.14E-06 | 22.70099 |
| Gut microbiota abundance (family Ruminococcaceae id.2050) | rs55793120 | 12 | 47384118 | T | 0.13822 | 0.0266152 | 1.44E-07 | 26.97008 |
| Gut microbiota abundance (family Ruminococcaceae id.2050) | rs56199908 | 9 | 2801371 | T | -0.199327 | 0.0410444 | 1.66E-06 | 23.58438 |
| Gut microbiota abundance (family Streptococcaceae id.1850) | rs10028567 | 4 | 53657577 | C | -0.0934027 | 0.0190343 | 3.72E-06 | 24.07937 |
| Gut microbiota abundance (family Streptococcaceae id.1850) | rs11110281 | 12 | 100584014 | T | -0.130554 | 0.0225943 | 1.40E-08 | 33.3874 |
| Gut microbiota abundance (family Streptococcaceae id.1850) | rs2370083 | 14 | 97526750 | G | -0.0842751 | 0.0184509 | 4.26E-06 | 20.86236 |
| Gut microbiota abundance (family Streptococcaceae id.1850) | rs4968759 | 17 | 61298020 | A | -0.0544035 | 0.0111271 | 8.92E-07 | 23.90505 |
| Gut microbiota abundance (family Streptococcaceae id.1850) | rs9903102 | 17 | 80576624 | C | -0.0693015 | 0.0154096 | 4.92E-06 | 20.22565 |
| Gut microbiota abundance (family Veillonellaceae id.2172) | rs111810795 | 14 | 102897930 | C | -0.0867108 | 0.0180573 | 1.73E-06 | 23.05902 |
| Gut microbiota abundance (family Veillonellaceae id.2172) | rs12186441 | 5 | 132646303 | G | 0.208096 | 0.0453999 | 4.53E-06 | 21.00959 |
| Gut microbiota abundance (family Veillonellaceae id.2172) | rs12741784 | 1 | 50088819 | C | -0.0621452 | 0.0119266 | 1.28E-07 | 27.15075 |
| Gut microbiota abundance (family Veillonellaceae id.2172) | rs1442060 | 4 | 46366067 | A | 0.0514024 | 0.0112125 | 4.51E-06 | 21.01657 |
| Gut microbiota abundance (family Veillonellaceae id.2172) | rs2175069 | 4 | 23317124 | G | 0.0526752 | 0.0114886 | 4.64E-06 | 21.0222 |
| Gut microbiota abundance (family Veillonellaceae id.2172) | rs4461038 | 15 | 78546996 | G | 0.0554965 | 0.0119467 | 3.73E-06 | 21.5792 |
| Gut microbiota abundance (family Veillonellaceae id.2172) | rs4797169 | 18 | 462180 | T | 0.0587799 | 0.0128297 | 4.49E-06 | 20.99059 |
| Gut microbiota abundance (family Verrucomicrobiaceae id.4036) | rs111862613 | 12 | 130309670 | T | 0.0907065 | 0.0196746 | 3.73E-06 | 21.25519 |
| Gut microbiota abundance (family Verrucomicrobiaceae id.4036) | rs117107102 | 18 | 49473635 | A | 0.204683 | 0.0431572 | 2.92E-06 | 22.4935 |
| Gut microbiota abundance (family Verrucomicrobiaceae id.4036) | rs11729256 | 4 | 95027272 | T | 0.0749798 | 0.0150177 | 6.73E-07 | 24.92767 |
| Gut microbiota abundance (family Verrucomicrobiaceae id.4036) | rs12908520 | 15 | 97570657 | G | 0.0619104 | 0.0130946 | 2.15E-06 | 22.35336 |
| Gut microbiota abundance (family Verrucomicrobiaceae id.4036) | rs2602429 | 16 | 81063149 | T | -0.0745516 | 0.0156195 | 2.70E-06 | 22.78136 |
| Gut microbiota abundance (family Verrucomicrobiaceae id.4036) | rs4242783 | 10 | 5064327 | A | -0.0687992 | 0.0147694 | 2.75E-06 | 21.69907 |
| Gut microbiota abundance (family Verrucomicrobiaceae id.4036) | rs4936098 | 11 | 130280667 | G | -0.0648691 | 0.0135928 | 1.13E-06 | 22.77497 |
| Gut microbiota abundance (family Verrucomicrobiaceae id.4036) | rs74542928 | 4 | 100544188 | T | 0.112124 | 0.0236422 | 1.65E-06 | 22.49165 |
| Gut microbiota abundance (family Verrucomicrobiaceae id.4036) | rs9349825 | 6 | 56341481 | A | -0.0704354 | 0.0147128 | 2.51E-06 | 22.91877 |
| Gut microbiota abundance (family Victivallaceae id.2255) | rs11671100 | 19 | 711637 | A | -0.160034 | 0.0349472 | 4.08E-06 | 20.97006 |
| Gut microbiota abundance (family Victivallaceae id.2255) | rs11764871 | 7 | 146808977 | G | 0.127099 | 0.025656 | 7.49E-07 | 24.5418 |
| Gut microbiota abundance (family Victivallaceae id.2255) | rs2944282 | 7 | 57589803 | T | -0.124197 | 0.0257026 | 1.57E-06 | 23.34899 |
| Gut microbiota abundance (family Victivallaceae id.2255) | rs4396289 | 11 | 9316730 | C | -0.152918 | 0.0288525 | 1.54E-07 | 28.08991 |
| Gut microbiota abundance (family Victivallaceae id.2255) | rs61702987 | 2 | 31211657 | T | 0.145501 | 0.0300007 | 3.08E-06 | 23.52173 |
| Gut microbiota abundance (family Victivallaceae id.2255) | rs62570196 | 9 | 111086170 | C | -0.246101 | 0.0483077 | 2.70E-07 | 25.95339 |
| Gut microbiota abundance (family Victivallaceae id.2255) | rs6545794 | 2 | 60484909 | A | -0.197786 | 0.0410495 | 5.97E-07 | 23.21536 |
| Gut microbiota abundance (family Victivallaceae id.2255) | rs67832247 | 2 | 2649472 | C | -0.123489 | 0.0266695 | 3.24E-06 | 21.4401 |
| Gut microbiota abundance (family Victivallaceae id.2255) | rs7077363 | 10 | 95286954 | G | 0.148942 | 0.0318366 | 2.83E-06 | 21.88674 |
| Gut microbiota abundance (genus Actinomyces id.423) | rs34583783 | 6 | 67207371 | G | 0.126596 | 0.0268461 | 4.49E-06 | 22.23707 |
| Gut microbiota abundance (genus Adlercreutzia id.812) | rs13231526 | 7 | 48844151 | C | 0.143237 | 0.0311654 | 4.81E-06 | 21.12346 |
| Gut microbiota abundance (genus Adlercreutzia id.812) | rs2717140 | 18 | 75009770 | C | -0.119225 | 0.0251081 | 2.05E-06 | 22.54794 |
| Gut microbiota abundance (genus Adlercreutzia id.812) | rs7680684 | 4 | 171281359 | T | 0.083381 | 0.0168901 | 9.77E-07 | 24.3708 |
| Gut microbiota abundance (genus Adlercreutzia id.812) | rs9490822 | 6 | 123909083 | C | -0.0734512 | 0.0155789 | 2.54E-06 | 22.22922 |
| Gut microbiota abundance (genus Akkermansia id.4037) | rs111862613 | 12 | 130309670 | T | 0.0911199 | 0.0196748 | 3.39E-06 | 21.44894 |
| Gut microbiota abundance (genus Akkermansia id.4037) | rs117107102 | 18 | 49473635 | A | 0.204406 | 0.0431629 | 3.01E-06 | 22.42673 |
| Gut microbiota abundance (genus Akkermansia id.4037) | rs11729256 | 4 | 95027272 | T | 0.0750473 | 0.0150184 | 6.58E-07 | 24.97025 |
| Gut microbiota abundance (genus Akkermansia id.4037) | rs12908520 | 15 | 97570657 | G | 0.061772 | 0.0130954 | 2.26E-06 | 22.25081 |
| Gut microbiota abundance (genus Akkermansia id.4037) | rs2602429 | 16 | 81063149 | T | -0.0745352 | 0.0156201 | 2.72E-06 | 22.76959 |
| Gut microbiota abundance (genus Akkermansia id.4037) | rs4242783 | 10 | 5064327 | A | -0.0685454 | 0.0147701 | 3.00E-06 | 21.53723 |
| Gut microbiota abundance (genus Akkermansia id.4037) | rs4936098 | 11 | 130280667 | G | -0.0649225 | 0.0135934 | 1.10E-06 | 22.81047 |
| Gut microbiota abundance (genus Akkermansia id.4037) | rs74542928 | 4 | 100544188 | T | 0.112623 | 0.0236434 | 1.48E-06 | 22.68999 |
| Gut microbiota abundance (genus Akkermansia id.4037) | rs9349825 | 6 | 56341481 | A | -0.0703407 | 0.0147133 | 2.60E-06 | 22.85563 |
| Gut microbiota abundance (genus Alistipes id.968) | rs1107244 | 13 | 38057357 | G | 0.0758558 | 0.0171182 | 3.59E-06 | 19.63638 |
| Gut microbiota abundance (genus Alistipes id.968) | rs11769002 | 7 | 62443498 | G | -0.0528793 | 0.0109389 | 1.45E-06 | 23.36814 |
| Gut microbiota abundance (genus Alistipes id.968) | rs67705352 | 1 | 19790706 | T | -0.0532404 | 0.0110864 | 1.65E-06 | 23.06224 |
| Gut microbiota abundance (genus Alistipes id.968) | rs7129639 | 11 | 17511427 | A | 0.0524955 | 0.0109584 | 1.78E-06 | 22.94826 |
| Gut microbiota abundance (genus Alistipes id.968) | rs8130320 | 21 | 40580258 | A | -0.049002 | 0.010717 | 4.84E-06 | 20.90649 |
| Gut microbiota abundance (genus Allisonella id.2174) | rs1901739 | 5 | 114286707 | G | -0.115769 | 0.0248626 | 3.59E-06 | 21.68161 |
| Gut microbiota abundance (genus Allisonella id.2174) | rs35778461 | 9 | 100150556 | C | 0.146679 | 0.0297186 | 1.21E-06 | 24.36011 |
| Gut microbiota abundance (genus Allisonella id.2174) | rs602075 | 9 | 79110160 | G | -0.168974 | 0.0296976 | 3.57E-08 | 32.37405 |
| Gut microbiota abundance (genus Alloprevotella id.961) | rs12675596 | 8 | 69994816 | G | 0.145669 | 0.0290678 | 9.64E-07 | 25.11366 |
| Gut microbiota abundance (genus Alloprevotella id.961) | rs4680035 | 3 | 152805600 | G | 0.119599 | 0.0259446 | 4.99E-06 | 21.25011 |
| Gut microbiota abundance (genus Anaerofilum id.2053) | rs10794359 | 11 | 1051715 | C | 0.0953524 | 0.02006 | 2.23E-06 | 22.59443 |
| Gut microbiota abundance (genus Anaerofilum id.2053) | rs17096874 | 14 | 30991358 | C | -0.126337 | 0.0268919 | 2.86E-06 | 22.0708 |
| Gut microbiota abundance (genus Anaerofilum id.2053) | rs712981 | 3 | 129686434 | A | 0.10076 | 0.0202902 | 6.83E-07 | 24.6606 |
| Gut microbiota abundance (genus Anaerofilum id.2053) | rs79598899 | 2 | 191139151 | C | 0.182586 | 0.0357284 | 3.75E-07 | 26.11607 |
| Gut microbiota abundance (genus Anaerofilum id.2053) | rs816292 | 12 | 117811409 | T | -0.113003 | 0.02204 | 2.64E-07 | 26.28795 |
| Gut microbiota abundance (genus Anaerostipes id.1991) | rs2014785 | 3 | 171100102 | T | 0.0515687 | 0.0112199 | 4.68E-06 | 21.12489 |
| Gut microbiota abundance (genus Anaerostipes id.1991) | rs2396460 | 2 | 228018671 | C | 0.0512653 | 0.0109555 | 2.91E-06 | 21.8969 |
| Gut microbiota abundance (genus Anaerostipes id.1991) | rs2804244 | 10 | 117383184 | G | 0.053089 | 0.0110983 | 2.04E-06 | 22.88212 |
| Gut microbiota abundance (genus Anaerostipes id.1991) | rs3900776 | 9 | 13525082 | G | -0.110013 | 0.0236302 | 2.75E-06 | 21.67471 |
| Gut microbiota abundance (genus Anaerostipes id.1991) | rs62157625 | 2 | 142774333 | T | 0.088573 | 0.0185548 | 1.45E-06 | 22.78716 |
| Gut microbiota abundance (genus Anaerostipes id.1991) | rs6726833 | 2 | 39351569 | C | -0.0877497 | 0.0189408 | 3.32E-06 | 21.46321 |
| Gut microbiota abundance (genus Anaerostipes id.1991) | rs6854026 | 4 | 169690814 | C | 0.0508482 | 0.0109075 | 3.20E-06 | 21.73205 |
| Gut microbiota abundance (genus Anaerostipes id.1991) | rs7193624 | 16 | 77574020 | T | -0.0750642 | 0.0150723 | 5.35E-07 | 24.80314 |
| Gut microbiota abundance (genus Anaerotruncus id.2054) | rs1272208 | 9 | 78630894 | T | 0.0611743 | 0.0129831 | 4.28E-06 | 22.20144 |
| Gut microbiota abundance (genus Anaerotruncus id.2054) | rs4669806 | 2 | 12200752 | G | 0.0576389 | 0.0122994 | 2.42E-06 | 21.96158 |
| Gut microbiota abundance (genus Anaerotruncus id.2054) | rs6563550 | 13 | 38058413 | T | 0.0877135 | 0.0176745 | 2.35E-07 | 24.62854 |
| Gut microbiota abundance (genus Anaerotruncus id.2054) | rs9347879 | 6 | 165015261 | T | 0.050618 | 0.011049 | 4.22E-06 | 20.98766 |
| Gut microbiota abundance (genus Bacteroides id.918) | rs11585893 | 1 | 10644351 | A | -0.0740746 | 0.0147633 | 1.80E-06 | 25.17513 |
| Gut microbiota abundance (genus Bacteroides id.918) | rs17619981 | 19 | 24342250 | T | 0.0880978 | 0.0187002 | 2.69E-06 | 22.1941 |
| Gut microbiota abundance (genus Bacteroides id.918) | rs6795673 | 3 | 10593224 | C | 0.0538565 | 0.0105251 | 3.38E-07 | 26.18326 |
| Gut microbiota abundance (genus Bacteroides id.918) | rs9507307 | 13 | 24910476 | C | 0.0604456 | 0.0129128 | 2.13E-06 | 21.91233 |
| Gut microbiota abundance (genus Barnesiella id.944) | rs12909713 | 15 | 87145913 | C | -0.0550716 | 0.0120039 | 4.95E-06 | 21.04799 |
| Gut microbiota abundance (genus Barnesiella id.944) | rs13242616 | 7 | 122021518 | C | 0.0583747 | 0.0123176 | 2.29E-06 | 22.45935 |
| Gut microbiota abundance (genus Barnesiella id.944) | rs199035 | 6 | 23435415 | G | 0.0559495 | 0.0119716 | 3.00E-06 | 21.84178 |
| Gut microbiota abundance (genus Barnesiella id.944) | rs2276875 | 4 | 5730954 | A | -0.0697176 | 0.0139537 | 4.65E-07 | 24.96354 |
| Gut microbiota abundance (genus Barnesiella id.944) | rs2428166 | 6 | 110831148 | G | -0.165859 | 0.0337309 | 8.51E-07 | 24.1781 |
| Gut microbiota abundance (genus Barnesiella id.944) | rs35177866 | 3 | 181589155 | A | 0.0917125 | 0.0190133 | 2.95E-06 | 23.26709 |
| Gut microbiota abundance (genus Barnesiella id.944) | rs60316894 | 12 | 54872438 | C | -0.121478 | 0.025184 | 1.19E-06 | 23.26729 |
| Gut microbiota abundance (genus Barnesiella id.944) | rs77455852 | 5 | 54107036 | T | -0.0891491 | 0.0195601 | 3.16E-06 | 20.77264 |
| Gut microbiota abundance (genus Barnesiella id.944) | rs79795328 | 4 | 114546595 | A | -0.0818645 | 0.0176441 | 4.23E-06 | 21.52743 |
| Gut microbiota abundance (genus Bifidobacterium id.436) | rs182549 | 2 | 136616754 | T | -0.119703 | 0.0127294 | 1.28E-20 | 88.4289 |
| Gut microbiota abundance (genus Bifidobacterium id.436) | rs7322849 | 13 | 112859829 | T | 0.112428 | 0.0201813 | 1.08E-08 | 31.03492 |
| Gut microbiota abundance (genus Bifidobacterium id.436) | rs73797465 | 5 | 142793467 | T | -0.0953566 | 0.0209236 | 4.38E-06 | 20.76963 |
| Gut microbiota abundance (genus Bifidobacterium id.436) | rs75344046 | 21 | 31861790 | C | 0.232354 | 0.0505979 | 4.86E-06 | 21.088 |
| Gut microbiota abundance (genus Bifidobacterium id.436) | rs857444 | 6 | 14617591 | C | 0.0558234 | 0.0121219 | 3.57E-06 | 21.20758 |
| Gut microbiota abundance (genus Bilophila id.3170) | rs1241171 | 1 | 103299921 | G | -0.0692654 | 0.0150148 | 4.24E-06 | 21.28108 |
| Gut microbiota abundance (genus Bilophila id.3170) | rs1571225 | 9 | 4940871 | T | -0.0826826 | 0.0170621 | 1.12E-06 | 23.48352 |
| Gut microbiota abundance (genus Bilophila id.3170) | rs3827020 | 20 | 61980991 | C | 0.0766479 | 0.0160642 | 1.79E-06 | 22.76577 |
| Gut microbiota abundance (genus Bilophila id.3170) | rs542415 | 15 | 50011554 | T | -0.0613623 | 0.0133436 | 4.71E-06 | 21.14741 |
| Gut microbiota abundance (genus Bilophila id.3170) | rs6793291 | 3 | 194419531 | A | -0.112729 | 0.0241633 | 3.11E-06 | 21.76501 |
| Gut microbiota abundance (genus Bilophila id.3170) | rs7802841 | 7 | 138583840 | A | -0.0670097 | 0.0137701 | 1.77E-06 | 23.68106 |
| Gut microbiota abundance (genus Butyricicoccus id.2055) | rs2017189 | 4 | 7460153 | T | 0.0506956 | 0.011024 | 3.87E-06 | 21.14765 |
| Gut microbiota abundance (genus Butyricicoccus id.2055) | rs56221232 | 2 | 153453410 | T | 0.0828027 | 0.0167401 | 7.62E-07 | 24.46656 |
| Gut microbiota abundance (genus Butyricimonas id.945) | rs113054641 | 21 | 16550389 | G | -0.144881 | 0.0274574 | 1.74E-07 | 27.84223 |
| Gut microbiota abundance (genus Butyricimonas id.945) | rs1862649 | 16 | 22172866 | G | 0.113124 | 0.0248176 | 4.76E-06 | 20.77734 |
| Gut microbiota abundance (genus Butyricimonas id.945) | rs62130338 | 19 | 49162501 | A | 0.0732608 | 0.0158324 | 3.90E-06 | 21.41163 |
| Gut microbiota abundance (genus Butyricimonas id.945) | rs62390301 | 5 | 159867647 | T | -0.0872983 | 0.0174893 | 7.42E-07 | 24.91533 |
| Gut microbiota abundance (genus Butyricimonas id.945) | rs7083431 | 10 | 72711156 | A | 0.0703539 | 0.0144439 | 8.85E-07 | 23.72506 |
| Gut microbiota abundance (genus Butyricimonas id.945) | rs71428626 | 2 | 83093264 | G | -0.133188 | 0.0289905 | 4.80E-06 | 21.10662 |
| Gut microbiota abundance (genus Butyricimonas id.945) | rs78453362 | 3 | 78740009 | A | -0.14948 | 0.0327073 | 4.06E-06 | 20.88703 |
| Gut microbiota abundance (genus Butyricimonas id.945) | rs9657374 | 8 | 4834406 | C | 0.0680719 | 0.0148172 | 4.50E-06 | 21.10588 |
| Gut microbiota abundance (genus Butyrivibrio id.1993) | rs16941336 | 17 | 20575697 | C | 0.127488 | 0.0267936 | 1.53E-06 | 22.64 |
| Gut microbiota abundance (genus Butyrivibrio id.1993) | rs4537857 | 13 | 27292666 | T | -0.12459 | 0.0260992 | 1.80E-06 | 22.7883 |
| Gut microbiota abundance (genus Butyrivibrio id.1993) | rs72723662 | 14 | 76065563 | C | 0.224078 | 0.0449336 | 7.86E-07 | 24.86887 |
| Gut microbiota abundance (genus Butyrivibrio id.1993) | rs74622183 | 14 | 34150977 | A | -0.201027 | 0.0428204 | 2.46E-06 | 22.03978 |
| Gut microbiota abundance (genus Butyrivibrio id.1993) | rs7752361 | 6 | 111780705 | G | 0.11923 | 0.0239975 | 7.69E-07 | 24.68534 |
| Gut microbiota abundance (genus Candidatus Soleaferrea id.11350) | rs10090365 | 8 | 138638199 | G | 0.0834413 | 0.0180989 | 4.17E-06 | 21.25484 |
| Gut microbiota abundance (genus Candidatus Soleaferrea id.11350) | rs10108780 | 8 | 123882833 | A | -0.092834 | 0.0199807 | 3.64E-06 | 21.58702 |
| Gut microbiota abundance (genus Candidatus Soleaferrea id.11350) | rs4294381 | 1 | 224688107 | C | -0.112197 | 0.023186 | 1.37E-06 | 23.4159 |
| Gut microbiota abundance (genus Catenibacterium id.2153) | rs12404911 | 1 | 240118443 | C | 0.140717 | 0.0304142 | 2.80E-06 | 21.40624 |
| Gut microbiota abundance (genus Catenibacterium id.2153) | rs212393 | 6 | 159485742 | A | 0.135254 | 0.0286209 | 3.62E-06 | 22.33231 |
| Gut microbiota abundance (genus Catenibacterium id.2153) | rs73128290 | 7 | 57364320 | A | 0.129726 | 0.0284563 | 4.29E-06 | 20.78247 |
| Gut microbiota abundance (genus Catenibacterium id.2153) | rs77285108 | 4 | 122282768 | G | -0.161826 | 0.0353447 | 3.63E-06 | 20.96274 |
| Gut microbiota abundance (genus Christensenellaceae R 7group id.11283) | rs17081797 | 18 | 67555560 | A | -0.0904325 | 0.0204253 | 3.34E-06 | 19.60253 |
| Gut microbiota abundance (genus Christensenellaceae R 7group id.11283) | rs892686 | 9 | 83043376 | A | 0.0514099 | 0.011136 | 3.97E-06 | 21.31253 |
| Gut microbiota abundance (genus Clostridium innocuum group id.14397) | rs1942371 | 18 | 69274308 | G | -0.157938 | 0.034187 | 4.06E-06 | 21.34279 |
| Gut microbiota abundance (genus Clostridium innocuum group id.14397) | rs6890185 | 5 | 71186626 | C | -0.113424 | 0.0233137 | 1.12E-06 | 23.66941 |
| Gut microbiota abundance (genus Clostridium sensustricto1 id.1873) | rs115807074 | 5 | 84997131 | A | -0.22736 | 0.0492575 | 4.32E-06 | 21.30509 |
| Gut microbiota abundance (genus Clostridium sensustricto1 id.1873) | rs116847295 | 12 | 43485015 | C | 0.110021 | 0.0246032 | 4.58E-06 | 19.99714 |
| Gut microbiota abundance (genus Clostridium sensustricto1 id.1873) | rs12341505 | 9 | 136710881 | G | 0.0810718 | 0.0180121 | 4.82E-06 | 20.25867 |
| Gut microbiota abundance (genus Clostridium sensustricto1 id.1873) | rs2795528 | 10 | 43270264 | G | -0.184315 | 0.0392204 | 2.72E-06 | 22.085 |
| Gut microbiota abundance (genus Clostridium sensustricto1 id.1873) | rs2817172 | 1 | 3041519 | C | 0.058139 | 0.0124492 | 2.77E-06 | 21.80983 |
| Gut microbiota abundance (genus Clostridium sensustricto1 id.1873) | rs550843 | 6 | 165722832 | T | -0.0783246 | 0.0169209 | 2.05E-06 | 21.42641 |
| Gut microbiota abundance (genus Collinsella id.815) | rs2103510 | 21 | 29355467 | G | 0.0786551 | 0.0168217 | 2.42E-06 | 21.86321 |
| Gut microbiota abundance (genus Collinsella id.815) | rs9541268 | 13 | 68610061 | C | 0.09597 | 0.0197312 | 8.79E-07 | 23.65724 |
| Gut microbiota abundance (genus Coprobacter id.949) | rs143662916 | 11 | 103549974 | C | 0.253264 | 0.0540141 | 3.07E-06 | 21.98531 |
| Gut microbiota abundance (genus Coprobacter id.949) | rs213863 | 6 | 97234939 | T | 0.0887412 | 0.0188377 | 2.35E-06 | 22.19192 |
| Gut microbiota abundance (genus Coprobacter id.949) | rs305411 | 1 | 88233128 | A | 0.129218 | 0.0264518 | 1.01E-06 | 23.86358 |
| Gut microbiota abundance (genus Coprobacter id.949) | rs3828477 | 3 | 46483717 | G | -0.0912179 | 0.019569 | 2.89E-06 | 21.72816 |
| Gut microbiota abundance (genus Coprobacter id.949) | rs72821405 | 6 | 4714797 | T | -0.147365 | 0.0319978 | 4.76E-06 | 21.21038 |
| Gut microbiota abundance (genus Coprococcus1 id.11301) | rs1010560 | 1 | 30400301 | C | 0.0580232 | 0.0122722 | 1.96E-06 | 22.35417 |
| Gut microbiota abundance (genus Coprococcus1 id.11301) | rs12794898 | 11 | 124554061 | G | 0.0903299 | 0.0197212 | 4.92E-06 | 20.97956 |
| Gut microbiota abundance (genus Coprococcus1 id.11301) | rs1576241 | 6 | 72529277 | A | -0.0510343 | 0.0109535 | 3.33E-06 | 21.70793 |
| Gut microbiota abundance (genus Coprococcus1 id.11301) | rs4277593 | 20 | 4319420 | G | -0.0585649 | 0.0109914 | 1.14E-07 | 28.39022 |
| Gut microbiota abundance (genus Coprococcus1 id.11301) | rs56405618 | 4 | 174468316 | A | -0.08963 | 0.0186508 | 1.57E-06 | 23.09468 |
| Gut microbiota abundance (genus Coprococcus1 id.11301) | rs73031725 | 11 | 134622282 | T | 0.167591 | 0.0355224 | 1.98E-06 | 22.25855 |
| Gut microbiota abundance (genus Coprococcus1 id.11301) | rs74101919 | 1 | 95424321 | T | -0.0718988 | 0.0144651 | 1.03E-06 | 24.70588 |
| Gut microbiota abundance (genus Coprococcus2 id.11302) | rs2482516 | 9 | 25554068 | C | 0.0754415 | 0.0164619 | 4.72E-06 | 21.002 |
| Gut microbiota abundance (genus Coprococcus2 id.11302) | rs6677933 | 1 | 112139008 | C | -0.0804405 | 0.0164216 | 1.19E-06 | 23.99488 |
| Gut microbiota abundance (genus Coprococcus2 id.11302) | rs72680320 | 4 | 131125786 | T | -0.0649382 | 0.013919 | 2.27E-06 | 21.76629 |
| Gut microbiota abundance (genus Coprococcus3 id.11303) | rs11080344 | 17 | 26104511 | C | 0.0516921 | 0.0113054 | 4.79E-06 | 20.90627 |
| Gut microbiota abundance (genus Coprococcus3 id.11303) | rs13394391 | 2 | 240727657 | C | -0.0709302 | 0.0150918 | 2.20E-06 | 22.08922 |
| Gut microbiota abundance (genus Coprococcus3 id.11303) | rs178271 | 22 | 21331556 | C | -0.145257 | 0.0294239 | 7.81E-07 | 24.37102 |
| Gut microbiota abundance (genus Coprococcus3 id.11303) | rs7521171 | 1 | 149998497 | A | 0.0596434 | 0.0129279 | 4.32E-06 | 21.28476 |
| Gut microbiota abundance (genus Coprococcus3 id.11303) | rs8100692 | 19 | 40032132 | T | 0.0577411 | 0.0113476 | 4.16E-07 | 25.89179 |
| Gut microbiota abundance (genus Defluviitaleaceae UCG011 id.11287) | rs112893842 | 9 | 8786663 | T | 0.11381 | 0.0232806 | 1.45E-06 | 23.8986 |
| Gut microbiota abundance (genus Defluviitaleaceae UCG011 id.11287) | rs4344384 | 10 | 66407366 | T | -0.0715978 | 0.0156309 | 4.83E-06 | 20.98125 |
| Gut microbiota abundance (genus Defluviitaleaceae UCG011 id.11287) | rs4677103 | 3 | 72207794 | A | 0.0977912 | 0.0197263 | 9.60E-07 | 24.57584 |
| Gut microbiota abundance (genus Defluviitaleaceae UCG011 id.11287) | rs55658617 | 21 | 41811003 | T | 0.174369 | 0.0362226 | 2.15E-06 | 23.17284 |
| Gut microbiota abundance (genus Defluviitaleaceae UCG011 id.11287) | rs72731813 | 4 | 147414743 | C | -0.147383 | 0.0293796 | 4.33E-07 | 25.16535 |
| Gut microbiota abundance (genus Defluviitaleaceae UCG011 id.11287) | rs9608282 | 22 | 24804081 | T | 0.142939 | 0.0299793 | 2.52E-06 | 22.73309 |
| Gut microbiota abundance (genus Defluviitaleaceae UCG011 id.11287) | rs9725395 | 1 | 85205632 | A | -0.13834 | 0.029554 | 3.52E-06 | 21.91104 |
| Gut microbiota abundance (genus Desulfovibrio id.3173) | rs13066142 | 3 | 67478973 | G | 0.119141 | 0.0250891 | 3.79E-06 | 22.5503 |
| Gut microbiota abundance (genus Desulfovibrio id.3173) | rs16863365 | 2 | 198089911 | A | 0.109402 | 0.0226964 | 1.79E-06 | 23.23467 |
| Gut microbiota abundance (genus Desulfovibrio id.3173) | rs2853179 | 8 | 105462047 | T | -0.0811971 | 0.0174234 | 2.42E-06 | 21.71777 |
| Gut microbiota abundance (genus Desulfovibrio id.3173) | rs6580353 | 5 | 139410687 | T | 0.0770979 | 0.0169735 | 4.94E-06 | 20.63205 |
| Gut microbiota abundance (genus Dialister id.2183) | rs11166701 | 8 | 138295725 | G | -0.0655301 | 0.013186 | 5.51E-07 | 24.69764 |
| Gut microbiota abundance (genus Dialister id.2183) | rs4753063 | 11 | 92267129 | A | 0.0596297 | 0.0130052 | 4.86E-06 | 21.02283 |
| Gut microbiota abundance (genus Dorea id.1997) | rs13279148 | 8 | 127905865 | G | 0.0715309 | 0.0150878 | 2.25E-06 | 22.47685 |
| Gut microbiota abundance (genus Dorea id.1997) | rs1899291 | 4 | 60992340 | T | -0.0697217 | 0.0150285 | 4.57E-06 | 21.52309 |
| Gut microbiota abundance (genus Dorea id.1997) | rs4793307 | 17 | 70733523 | C | 0.0574165 | 0.0122472 | 4.01E-06 | 21.97859 |
| Gut microbiota abundance (genus Dorea id.1997) | rs62503162 | 8 | 15768657 | A | -0.0974091 | 0.0194379 | 7.47E-07 | 25.1131 |
| Gut microbiota abundance (genus Dorea id.1997) | rs73729431 | 6 | 25078050 | C | -0.13745 | 0.0299983 | 3.17E-06 | 20.99405 |
| Gut microbiota abundance (genus Eggerthella id.819) | rs112205261 | 1 | 9136139 | T | -0.188618 | 0.0403613 | 3.35E-06 | 21.83916 |
| Gut microbiota abundance (genus Eggerthella id.819) | rs2223081 | 21 | 29778032 | A | -0.102572 | 0.0221108 | 3.89E-06 | 21.52032 |
| Gut microbiota abundance (genus Eggerthella id.819) | rs2240838 | 7 | 38335954 | G | -0.0980582 | 0.0197653 | 7.36E-07 | 24.6128 |
| Gut microbiota abundance (genus Eggerthella id.819) | rs76663501 | 20 | 55647869 | C | 0.175349 | 0.0378576 | 4.83E-06 | 21.45361 |
| Gut microbiota abundance (genus Eisenbergiella id.11304) | rs11027642 | 11 | 24001840 | C | 0.129006 | 0.0284844 | 4.92E-06 | 20.51189 |
| Gut microbiota abundance (genus Eisenbergiella id.11304) | rs1508033 | 15 | 53375201 | A | 0.091546 | 0.0195787 | 3.23E-06 | 21.86306 |
| Gut microbiota abundance (genus Eisenbergiella id.11304) | rs2683098 | 15 | 36178565 | T | -0.107319 | 0.022515 | 2.24E-06 | 22.72005 |
| Gut microbiota abundance (genus Eisenbergiella id.11304) | rs3812426 | 8 | 50822676 | A | -0.106447 | 0.0224159 | 2.72E-06 | 22.55041 |
| Gut microbiota abundance (genus Eisenbergiella id.11304) | rs4462860 | 21 | 21610254 | G | 0.0939071 | 0.0201104 | 4.16E-06 | 21.80497 |
| Gut microbiota abundance (genus Enterorhabdus id.820) | rs114731706 | 2 | 19611953 | T | 0.182308 | 0.0382298 | 2.17E-06 | 22.74089 |
| Gut microbiota abundance (genus Enterorhabdus id.820) | rs3017103 | 11 | 62174193 | G | -0.0980897 | 0.0208995 | 2.94E-06 | 22.028 |
| Gut microbiota abundance (genus Enterorhabdus id.820) | rs73331712 | 12 | 69515102 | T | 0.26199 | 0.055123 | 4.85E-06 | 22.58935 |
| Gut microbiota abundance (genus Erysipelatoclostridium id.11381) | rs17804233 | 5 | 77815056 | C | 0.0662914 | 0.0144203 | 4.59E-06 | 21.13323 |
| Gut microbiota abundance (genus Erysipelatoclostridium id.11381) | rs340991 | 5 | 34892042 | A | -0.0740482 | 0.0158891 | 3.75E-06 | 21.71853 |
| Gut microbiota abundance (genus Erysipelatoclostridium id.11381) | rs4697572 | 4 | 25446787 | A | -0.0810636 | 0.0163308 | 7.59E-07 | 24.63978 |
| Gut microbiota abundance (genus Erysipelatoclostridium id.11381) | rs58236560 | 11 | 121876913 | G | -0.111157 | 0.0234426 | 2.16E-06 | 22.4834 |
| Gut microbiota abundance (genus Erysipelatoclostridium id.11381) | rs622418 | 9 | 90586043 | A | -0.0668051 | 0.0143224 | 3.68E-06 | 21.75643 |
| Gut microbiota abundance (genus Erysipelatoclostridium id.11381) | rs6474512 | 8 | 38780606 | C | -0.067015 | 0.0143201 | 3.02E-06 | 21.90039 |
| Gut microbiota abundance (genus Erysipelatoclostridium id.11381) | rs710230 | 1 | 42333631 | C | -0.143381 | 0.0281514 | 6.33E-07 | 25.94079 |
| Gut microbiota abundance (genus Erysipelatoclostridium id.11381) | rs7221249 | 17 | 10177708 | G | -0.0839861 | 0.0142741 | 4.31E-09 | 34.61923 |
| Gut microbiota abundance (genus Erysipelotrichaceae UCG003 id.11384) | rs75949021 | 2 | 175403951 | T | -0.169667 | 0.0374238 | 3.58E-06 | 20.55413 |
| Gut microbiota abundance (genus Escherichia Shigella id.3504) | rs113127095 | 13 | 23253392 | A | 0.151009 | 0.0323434 | 3.33E-06 | 21.79889 |
| Gut microbiota abundance (genus Escherichia Shigella id.3504) | rs1154904 | 11 | 134774845 | G | 0.0613376 | 0.0130644 | 3.04E-06 | 22.0432 |
| Gut microbiota abundance (genus Escherichia Shigella id.3504) | rs592299 | 9 | 136179347 | T | -0.0592004 | 0.0129486 | 4.77E-06 | 20.90276 |
| Gut microbiota abundance (genus Escherichia Shigella id.3504) | rs73208162 | 21 | 39324484 | A | -0.119296 | 0.024839 | 2.19E-06 | 23.0666 |
| Gut microbiota abundance (genus Eubacterium brachy group id.11296) | rs112617308 | 10 | 94185213 | T | -0.170873 | 0.0362844 | 2.38E-06 | 22.17721 |
| Gut microbiota abundance (genus Eubacterium brachy group id.11296) | rs2913110 | 10 | 22892667 | T | -0.105143 | 0.022942 | 4.56E-06 | 21.00381 |
| Gut microbiota abundance (genus Eubacterium brachy group id.11296) | rs4862235 | 4 | 184628931 | A | -0.104806 | 0.0225751 | 3.73E-06 | 21.55326 |
| Gut microbiota abundance (genus Eubacterium brachy group id.11296) | rs62348779 | 5 | 17460096 | T | -0.201481 | 0.043286 | 3.78E-06 | 21.66573 |
| Gut microbiota abundance (genus Eubacterium coprostanoligenes group id.11375) | rs12906958 | 15 | 36911598 | C | -0.0533159 | 0.0115935 | 4.35E-06 | 21.14873 |
| Gut microbiota abundance (genus Eubacterium coprostanoligenes group id.11375) | rs17159861 | 7 | 31085162 | C | 0.0962228 | 0.0168388 | 1.04E-08 | 32.6538 |
| Gut microbiota abundance (genus Eubacterium coprostanoligenes group id.11375) | rs4076415 | 15 | 86440997 | G | -0.0515152 | 0.0110294 | 1.99E-06 | 21.81559 |
| Gut microbiota abundance (genus Eubacterium coprostanoligenes group id.11375) | rs6762473 | 3 | 127139574 | A | -0.0521584 | 0.0112353 | 4.26E-06 | 21.55158 |
| Gut microbiota abundance (genus Eubacterium coprostanoligenes group id.11375) | rs76898927 | 3 | 81595477 | G | 0.123055 | 0.0266375 | 4.79E-06 | 21.34084 |
| Gut microbiota abundance (genus Eubacterium coprostanoligenes group id.11375) | rs9648214 | 7 | 16380806 | T | -0.0828719 | 0.016431 | 2.52E-07 | 25.43821 |
| Gut microbiota abundance (genus Eubacterium eligens group id.14372) | rs265534 | 10 | 83092007 | T | -0.0563756 | 0.0120127 | 2.27E-06 | 22.02425 |
| Gut microbiota abundance (genus Eubacterium eligens group id.14372) | rs4583233 | 16 | 81820486 | A | 0.0670205 | 0.0128122 | 2.84E-07 | 27.36326 |
| Gut microbiota abundance (genus Eubacterium eligens group id.14372) | rs6923695 | 6 | 70956815 | T | 0.103268 | 0.0229525 | 4.87E-06 | 20.24284 |
| Gut microbiota abundance (genus Eubacterium fissicatena group id.14373) | rs11876297 | 18 | 45753272 | T | 0.131469 | 0.0281712 | 2.67E-06 | 21.7789 |
| Gut microbiota abundance (genus Eubacterium fissicatena group id.14373) | rs151257695 | 7 | 73043561 | A | 0.20951 | 0.0454847 | 3.10E-06 | 21.21675 |
| Gut microbiota abundance (genus Eubacterium fissicatena group id.14373) | rs2733072 | 8 | 5433699 | G | 0.109644 | 0.0228306 | 1.49E-06 | 23.06402 |
| Gut microbiota abundance (genus Eubacterium fissicatena group id.14373) | rs3771393 | 2 | 71145246 | T | -0.130842 | 0.0266671 | 7.38E-07 | 24.0737 |
| Gut microbiota abundance (genus Eubacterium fissicatena group id.14373) | rs7104872 | 11 | 115165111 | G | 0.138612 | 0.0291906 | 2.73E-06 | 22.54839 |
| Gut microbiota abundance (genus Eubacterium hallii group id.11338) | rs10798999 | 1 | 34308917 | C | 0.0601661 | 0.0126752 | 2.61E-06 | 22.53171 |
| Gut microbiota abundance (genus Eubacterium hallii group id.11338) | rs10808115 | 7 | 100635375 | C | 0.0504747 | 0.0109922 | 4.42E-06 | 21.08523 |
| Gut microbiota abundance (genus Eubacterium hallii group id.11338) | rs13116360 | 4 | 111885431 | T | 0.154124 | 0.0297185 | 2.94E-07 | 26.89594 |
| Gut microbiota abundance (genus Eubacterium hallii group id.11338) | rs28584818 | 3 | 64664456 | A | 0.126115 | 0.0268627 | 4.43E-06 | 22.04115 |
| Gut microbiota abundance (genus Eubacterium hallii group id.11338) | rs60254196 | 7 | 148856720 | G | 0.0522832 | 0.0111865 | 2.70E-06 | 21.84419 |
| Gut microbiota abundance (genus Eubacterium hallii group id.11338) | rs74018587 | 15 | 62014160 | C | 0.208943 | 0.043822 | 3.70E-06 | 22.73376 |
| Gut microbiota abundance (genus Eubacterium hallii group id.11338) | rs949971 | 3 | 110283389 | T | -0.0540164 | 0.0116101 | 3.29E-06 | 21.64608 |
| Gut microbiota abundance (genus Eubacterium nodatum group id.11297) | rs34297067 | 14 | 52185888 | A | -0.186907 | 0.0341475 | 6.60E-08 | 29.95941 |
| Gut microbiota abundance (genus Eubacterium nodatum group id.11297) | rs61841040 | 10 | 9659749 | G | 0.160621 | 0.0341609 | 3.56E-06 | 22.10783 |
| Gut microbiota abundance (genus Eubacterium nodatum group id.11297) | rs77910827 | 9 | 96597985 | C | 0.201788 | 0.0413568 | 9.05E-07 | 23.80657 |
| Gut microbiota abundance (genus Eubacterium oxidoreducens group id.11339) | rs12423772 | 12 | 94515340 | G | 0.14098 | 0.0295029 | 2.63E-06 | 22.83419 |
| Gut microbiota abundance (genus Eubacterium oxidoreducens group id.11339) | rs2973294 | 4 | 37526679 | G | 0.0923559 | 0.0195439 | 2.39E-06 | 22.33093 |
| Gut microbiota abundance (genus Eubacterium oxidoreducens group id.11339) | rs34561138 | 16 | 86480355 | G | 0.216145 | 0.045992 | 2.51E-06 | 22.08644 |
| Gut microbiota abundance (genus Eubacterium rectale group id.14374) | rs10248854 | 7 | 121281238 | C | -0.0527827 | 0.0113464 | 4.21E-06 | 21.64049 |
| Gut microbiota abundance (genus Eubacterium rectale group id.14374) | rs10797540 | 1 | 234530370 | A | 0.0503179 | 0.0108389 | 3.53E-06 | 21.55136 |
| Gut microbiota abundance (genus Eubacterium rectale group id.14374) | rs314726 | 2 | 125553155 | T | 0.0528824 | 0.0109486 | 1.38E-06 | 23.32948 |
| Gut microbiota abundance (genus Eubacterium rectale group id.14374) | rs35398954 | 15 | 93006765 | A | -0.0901402 | 0.0174602 | 5.40E-07 | 26.65254 |
| Gut microbiota abundance (genus Eubacterium ruminantium group id.11340) | rs10131724 | 14 | 52219066 | C | 0.199832 | 0.0414577 | 2.39E-06 | 23.23377 |
| Gut microbiota abundance (genus Eubacterium ruminantium group id.11340) | rs139749 | 22 | 25303055 | C | -0.0845439 | 0.0171791 | 8.59E-07 | 24.21942 |
| Gut microbiota abundance (genus Eubacterium ruminantium group id.11340) | rs16891896 | 5 | 33897589 | G | -0.174787 | 0.0390573 | 2.38E-06 | 20.0269 |
| Gut microbiota abundance (genus Eubacterium ruminantium group id.11340) | rs17519472 | 12 | 30360954 | C | 0.107804 | 0.0233984 | 4.70E-06 | 21.22743 |
| Gut microbiota abundance (genus Eubacterium ruminantium group id.11340) | rs2116427 | 5 | 121182996 | A | 0.0911461 | 0.0182353 | 4.67E-07 | 24.98333 |
| Gut microbiota abundance (genus Eubacterium ruminantium group id.11340) | rs2229917 | 9 | 130980937 | A | 0.153538 | 0.0323922 | 2.16E-06 | 22.4673 |
| Gut microbiota abundance (genus Eubacterium ruminantium group id.11340) | rs57340348 | 6 | 130030114 | T | -0.0979429 | 0.0212166 | 4.93E-06 | 21.31053 |
| Gut microbiota abundance (genus Eubacterium ruminantium group id.11340) | rs606117 | 9 | 2539358 | G | -0.0833243 | 0.018056 | 4.82E-06 | 21.29611 |
| Gut microbiota abundance (genus Eubacterium ruminantium group id.11340) | rs7000472 | 8 | 138626268 | G | 0.0762282 | 0.016523 | 4.07E-06 | 21.28401 |
| Gut microbiota abundance (genus Eubacterium ruminantium group id.11340) | rs72836424 | 10 | 129037453 | C | -0.139825 | 0.030069 | 2.62E-06 | 21.62378 |
| Gut microbiota abundance (genus Eubacterium ventriosum group id.11341) | rs11617697 | 13 | 98785900 | A | -0.143349 | 0.0286241 | 7.22E-07 | 25.07989 |
| Gut microbiota abundance (genus Eubacterium ventriosum group id.11341) | rs12964517 | 18 | 22460785 | G | 0.0587198 | 0.0123453 | 2.08E-06 | 22.62381 |
| Gut microbiota abundance (genus Eubacterium ventriosum group id.11341) | rs16884680 | 8 | 114445055 | G | -0.0906428 | 0.0191909 | 1.74E-06 | 22.30879 |
| Gut microbiota abundance (genus Eubacterium ventriosum group id.11341) | rs3809430 | 14 | 44975510 | T | -0.0548171 | 0.0118426 | 3.55E-06 | 21.42585 |
| Gut microbiota abundance (genus Eubacterium ventriosum group id.11341) | rs73615400 | 20 | 50140919 | T | -0.0956303 | 0.0193428 | 9.54E-07 | 24.44288 |
| Gut microbiota abundance (genus Eubacterium ventriosum group id.11341) | rs876734 | 12 | 52423996 | T | 0.0618696 | 0.0132532 | 2.89E-06 | 21.7928 |
| Gut microbiota abundance (genus Eubacterium xylanophilum group id.14375) | rs10140184 | 14 | 73181783 | A | 0.0576795 | 0.012599 | 4.96E-06 | 20.95901 |
| Gut microbiota abundance (genus Eubacterium xylanophilum group id.14375) | rs112176119 | 16 | 73696771 | C | -0.113455 | 0.0245762 | 3.33E-06 | 21.31169 |
| Gut microbiota abundance (genus Eubacterium xylanophilum group id.14375) | rs13239072 | 7 | 43566344 | G | 0.0687427 | 0.0142728 | 1.82E-06 | 23.19716 |
| Gut microbiota abundance (genus Eubacterium xylanophilum group id.14375) | rs17830032 | 20 | 57995506 | G | -0.160597 | 0.0310515 | 2.39E-07 | 26.74913 |
| Gut microbiota abundance (genus Eubacterium xylanophilum group id.14375) | rs1999224 | 9 | 130498120 | G | -0.0949051 | 0.0203503 | 3.75E-06 | 21.74891 |
| Gut microbiota abundance (genus Eubacterium xylanophilum group id.14375) | rs2213117 | 11 | 131009660 | T | 0.0877797 | 0.0189012 | 4.21E-06 | 21.56798 |
| Gut microbiota abundance (genus Faecalibacterium id.2057) | rs1271565 | 14 | 64044097 | C | -0.0576251 | 0.0119649 | 1.30E-06 | 23.19558 |
| Gut microbiota abundance (genus Faecalibacterium id.2057) | rs6910935 | 6 | 131587157 | G | -0.134864 | 0.0277032 | 1.38E-06 | 23.69912 |
| Gut microbiota abundance (genus Faecalibacterium id.2057) | rs75499067 | 16 | 6099863 | C | 0.227585 | 0.0465522 | 1.76E-06 | 23.90049 |
| Gut microbiota abundance (genus Family XIII AD3011 group id.11293) | rs12812672 | 12 | 18127597 | T | -0.096065 | 0.020828 | 2.56E-06 | 21.27332 |
| Gut microbiota abundance (genus Family XIII AD3011 group id.11293) | rs16840310 | 1 | 240694163 | G | 0.0608052 | 0.0122149 | 6.75E-07 | 24.78002 |
| Gut microbiota abundance (genus Family XIII AD3011 group id.11293) | rs16940167 | 15 | 58683697 | C | 0.0732552 | 0.0159898 | 3.91E-06 | 20.98896 |
| Gut microbiota abundance (genus Family XIII AD3011 group id.11293) | rs17156849 | 7 | 28603985 | G | -0.112891 | 0.0245292 | 4.19E-06 | 21.18126 |
| Gut microbiota abundance (genus Family XIII AD3011 group id.11293) | rs62029761 | 16 | 16006242 | A | 0.128753 | 0.0276002 | 3.89E-06 | 21.76157 |
| Gut microbiota abundance (genus Family XIII AD3011 group id.11293) | rs62200412 | 20 | 4618639 | C | -0.0800851 | 0.0163834 | 5.80E-07 | 23.89436 |
| Gut microbiota abundance (genus Family XIII AD3011 group id.11293) | rs72730932 | 1 | 192019835 | C | -0.0899559 | 0.0177107 | 6.89E-07 | 25.79811 |
| Gut microbiota abundance (genus Family XIII UCG001 id.11294) | rs12049454 | 1 | 85228325 | T | -0.0647298 | 0.0134065 | 1.17E-06 | 23.3119 |
| Gut microbiota abundance (genus Family XIII UCG001 id.11294) | rs1426266 | 3 | 188224096 | C | 0.0665521 | 0.0137151 | 1.25E-06 | 23.54646 |
| Gut microbiota abundance (genus Family XIII UCG001 id.11294) | rs62414802 | 6 | 76598635 | C | -0.0611904 | 0.013457 | 4.29E-06 | 20.67617 |
| Gut microbiota abundance (genus Family XIII UCG001 id.11294) | rs7119679 | 11 | 94681786 | G | -0.0809085 | 0.0174782 | 3.52E-06 | 21.42865 |
| Gut microbiota abundance (genus Family XIII UCG001 id.11294) | rs76463770 | 3 | 45479090 | A | 0.19313 | 0.0419866 | 3.77E-06 | 21.15817 |
| Gut microbiota abundance (genus Flavonifractor id.2059) | rs11811696 | 1 | 237351354 | T | -0.116063 | 0.0241012 | 2.07E-06 | 23.19051 |
| Gut microbiota abundance (genus Flavonifractor id.2059) | rs12030302 | 1 | 77887835 | G | 0.0692276 | 0.0137467 | 5.61E-07 | 25.36072 |
| Gut microbiota abundance (genus Flavonifractor id.2059) | rs34066017 | 11 | 44849689 | A | 0.0764294 | 0.0159789 | 1.52E-06 | 22.87848 |
| Gut microbiota abundance (genus Flavonifractor id.2059) | rs806808 | 10 | 32381186 | C | -0.0667272 | 0.0136557 | 1.18E-06 | 23.8769 |
| Gut microbiota abundance (genus Fusicatenibacter id.11305) | rs1864685 | 17 | 70721782 | A | -0.0494799 | 0.0108105 | 4.96E-06 | 20.94913 |
| Gut microbiota abundance (genus Fusicatenibacter id.11305) | rs2025938 | 10 | 112177119 | G | -0.0967298 | 0.0205426 | 2.99E-06 | 22.17225 |
| Gut microbiota abundance (genus Fusicatenibacter id.11305) | rs2132128 | 8 | 15360718 | G | -0.0771914 | 0.0160344 | 1.08E-06 | 23.17568 |
| Gut microbiota abundance (genus Fusicatenibacter id.11305) | rs3303 | 10 | 120447429 | T | -0.0953657 | 0.020407 | 3.94E-06 | 21.83866 |
| Gut microbiota abundance (genus Fusicatenibacter id.11305) | rs4378146 | 1 | 24927625 | A | -0.0616691 | 0.0125259 | 7.20E-07 | 24.23915 |
| Gut microbiota abundance (genus Fusicatenibacter id.11305) | rs62187631 | 2 | 226649570 | T | -0.0710571 | 0.015924 | 4.55E-06 | 19.9118 |
| Gut microbiota abundance (genus Fusicatenibacter id.11305) | rs62353480 | 5 | 29595000 | A | -0.0701397 | 0.0145589 | 1.57E-06 | 23.20976 |
| Gut microbiota abundance (genus Fusicatenibacter id.11305) | rs704418 | 3 | 64252803 | C | -0.0739124 | 0.0151073 | 7.77E-07 | 23.93651 |
| Gut microbiota abundance (genus Fusicatenibacter id.11305) | rs8063430 | 16 | 73821805 | T | -0.104026 | 0.022217 | 4.93E-06 | 21.92366 |
| Gut microbiota abundance (genus Gordonibacter id.821) | rs322296 | 7 | 136937189 | G | 0.17869 | 0.037722 | 4.02E-06 | 22.43939 |
| Gut microbiota abundance (genus Gordonibacter id.821) | rs72714787 | 4 | 136768501 | C | 0.181405 | 0.0377102 | 1.43E-06 | 23.14093 |
| Gut microbiota abundance (genus Gordonibacter id.821) | rs7294633 | 12 | 29024534 | T | -0.12867 | 0.0249946 | 3.44E-07 | 26.501 |
| Gut microbiota abundance (genus Haemophilus id.3698) | rs10781340 | 9 | 78752170 | A | -0.0948915 | 0.0203221 | 4.32E-06 | 21.80306 |
| Gut microbiota abundance (genus Haemophilus id.3698) | rs111582866 | 16 | 48742489 | G | -0.124265 | 0.026016 | 1.27E-06 | 22.8148 |
| Gut microbiota abundance (genus Haemophilus id.3698) | rs35509 | 12 | 115493337 | G | 0.128249 | 0.0268779 | 2.01E-06 | 22.7676 |
| Gut microbiota abundance (genus Haemophilus id.3698) | rs4822728 | 22 | 26891808 | T | 0.0705861 | 0.0151388 | 3.48E-06 | 21.7398 |
| Gut microbiota abundance (genus Haemophilus id.3698) | rs76022354 | 10 | 94306385 | C | 0.244638 | 0.0505504 | 1.83E-06 | 23.42063 |
| Gut microbiota abundance (genus Haemophilus id.3698) | rs78909003 | 9 | 105650242 | T | -0.246256 | 0.0503924 | 1.67E-06 | 23.88051 |
| Gut microbiota abundance (genus Haemophilus id.3698) | rs9328464 | 6 | 8350917 | T | 0.0723095 | 0.0148799 | 1.42E-06 | 23.61515 |
| Gut microbiota abundance (genus Haemophilus id.3698) | rs9382510 | 6 | 55448491 | C | -0.093521 | 0.0172649 | 7.12E-08 | 29.34203 |
| Gut microbiota abundance (genus Haemophilus id.3698) | rs9895850 | 17 | 64535013 | T | -0.192957 | 0.0416755 | 2.14E-06 | 21.43677 |
| Gut microbiota abundance (genus Holdemanella id.11393) | rs12513188 | 4 | 71000791 | G | 0.0903906 | 0.0195276 | 4.65E-06 | 21.42638 |
| Gut microbiota abundance (genus Holdemanella id.11393) | rs607782 | 6 | 4148609 | C | 0.0854214 | 0.0172515 | 7.19E-07 | 24.5177 |
| Gut microbiota abundance (genus Holdemanella id.11393) | rs73011279 | 19 | 15020095 | T | -0.0961669 | 0.0199336 | 1.36E-06 | 23.27447 |
| Gut microbiota abundance (genus Holdemanella id.11393) | rs75764681 | 10 | 4494630 | T | -0.283103 | 0.0598999 | 1.94E-06 | 22.33761 |
| Gut microbiota abundance (genus Holdemanella id.11393) | rs8113760 | 19 | 43851935 | G | 0.078999 | 0.01734 | 4.62E-06 | 20.75606 |
| Gut microbiota abundance (genus Holdemania id.2157) | rs111745969 | 15 | 93110222 | A | 0.120677 | 0.0265781 | 3.71E-06 | 20.61585 |
| Gut microbiota abundance (genus Holdemania id.2157) | rs1867876 | 11 | 18768068 | T | 0.0842919 | 0.0162192 | 2.74E-07 | 27.00927 |
| Gut microbiota abundance (genus Holdemania id.2157) | rs6133067 | 20 | 3839754 | T | 0.0910829 | 0.0178561 | 5.17E-07 | 26.01959 |
| Gut microbiota abundance (genus Holdemania id.2157) | rs77293403 | 5 | 77388406 | A | 0.164556 | 0.0341775 | 1.77E-06 | 23.18178 |
| Gut microbiota abundance (genus Holdemania id.2157) | rs9500080 | 6 | 105737322 | C | 0.0926764 | 0.0178886 | 4.09E-07 | 26.84019 |
| Gut microbiota abundance (genus Howardella id.2000) | rs12452946 | 17 | 17253288 | A | -0.105833 | 0.0228941 | 3.80E-06 | 21.36953 |
| Gut microbiota abundance (genus Howardella id.2000) | rs1484873 | 18 | 43206985 | G | 0.227831 | 0.046335 | 2.56E-06 | 24.17727 |
| Gut microbiota abundance (genus Howardella id.2000) | rs17167098 | 7 | 133154356 | G | -0.169366 | 0.0352068 | 1.12E-06 | 23.14192 |
| Gut microbiota abundance (genus Howardella id.2000) | rs36081916 | 7 | 93527414 | T | -0.18123 | 0.0402987 | 4.70E-06 | 20.22451 |
| Gut microbiota abundance (genus Howardella id.2000) | rs609430 | 4 | 169178315 | T | -0.112045 | 0.0239328 | 3.34E-06 | 21.91785 |
| Gut microbiota abundance (genus Howardella id.2000) | rs672217 | 18 | 60125134 | G | 0.164146 | 0.0349993 | 3.52E-06 | 21.99591 |
| Gut microbiota abundance (genus Hungatella id.11306) | rs13128780 | 4 | 166058998 | T | -0.149725 | 0.0312777 | 1.75E-06 | 22.91496 |
| Gut microbiota abundance (genus Hungatella id.11306) | rs72759041 | 15 | 89577376 | G | -0.126025 | 0.0282242 | 3.86E-06 | 19.93747 |
| Gut microbiota abundance (genus Intestinibacter id.11345) | rs10805326 | 4 | 14324623 | A | -0.0775151 | 0.0139665 | 3.55E-08 | 30.80331 |
| Gut microbiota abundance (genus Intestinibacter id.11345) | rs118030283 | 16 | 5993076 | G | -0.151829 | 0.0324471 | 2.67E-06 | 21.89564 |
| Gut microbiota abundance (genus Intestinibacter id.11345) | rs16938435 | 9 | 21502923 | T | -0.112189 | 0.0235439 | 1.80E-06 | 22.70616 |
| Gut microbiota abundance (genus Intestinibacter id.11345) | rs2702387 | 4 | 179361262 | G | -0.0608559 | 0.0132145 | 4.26E-06 | 21.2082 |
| Gut microbiota abundance (genus Intestinibacter id.11345) | rs4327025 | 15 | 92446683 | G | -0.0810346 | 0.0154397 | 1.64E-07 | 27.5463 |
| Gut microbiota abundance (genus Intestinibacter id.11345) | rs478972 | 11 | 125663184 | C | 0.142681 | 0.0297116 | 1.82E-06 | 23.06111 |
| Gut microbiota abundance (genus Intestinibacter id.11345) | rs6875660 | 5 | 159686256 | C | 0.0890455 | 0.0193905 | 3.06E-06 | 21.08851 |
| Gut microbiota abundance (genus Intestinimonas id.2062) | rs10262702 | 7 | 66890843 | T | 0.0918017 | 0.0194885 | 2.06E-06 | 22.18935 |
| Gut microbiota abundance (genus Intestinimonas id.2062) | rs11258178 | 10 | 13124513 | A | 0.0660742 | 0.0134139 | 6.98E-07 | 24.26351 |
| Gut microbiota abundance (genus Intestinimonas id.2062) | rs12226153 | 11 | 94364293 | A | -0.151142 | 0.0306922 | 5.12E-07 | 24.25014 |
| Gut microbiota abundance (genus Intestinimonas id.2062) | rs1859797 | 7 | 22007303 | G | 0.060368 | 0.0131793 | 4.12E-06 | 20.98113 |
| Gut microbiota abundance (genus Intestinimonas id.2062) | rs2731794 | 5 | 17209391 | C | 0.120632 | 0.0257529 | 1.92E-06 | 21.94183 |
| Gut microbiota abundance (genus Intestinimonas id.2062) | rs2930225 | 16 | 85327902 | T | -0.0729524 | 0.0152948 | 1.35E-06 | 22.75053 |
| Gut microbiota abundance (genus Intestinimonas id.2062) | rs62240188 | 3 | 10611142 | G | 0.130074 | 0.0267179 | 2.20E-06 | 23.70153 |
| Gut microbiota abundance (genus Intestinimonas id.2062) | rs716604 | 2 | 6514088 | A | 0.0818068 | 0.0165992 | 8.57E-07 | 24.28871 |
| Gut microbiota abundance (genus Intestinimonas id.2062) | rs7170984 | 15 | 93819735 | T | -0.0658116 | 0.0140767 | 2.98E-06 | 21.85764 |
| Gut microbiota abundance (genus Intestinimonas id.2062) | rs72982915 | 2 | 140898088 | C | 0.183158 | 0.0402743 | 4.91E-06 | 20.68215 |
| Gut microbiota abundance (genus Lachnoclostridium id.11308) | rs4738679 | 8 | 59370320 | A | 0.0520267 | 0.011404 | 4.42E-06 | 20.81317 |
| Gut microbiota abundance (genus Lachnoclostridium id.11308) | rs6112314 | 20 | 19300846 | A | -0.0561715 | 0.0108174 | 2.43E-07 | 26.96412 |
| Gut microbiota abundance (genus Lachnoclostridium id.11308) | rs615997 | 3 | 23037786 | T | 0.0511752 | 0.0106491 | 2.03E-06 | 23.09369 |
| Gut microbiota abundance (genus Lachnoclostridium id.11308) | rs62285313 | 3 | 177470032 | A | 0.0864203 | 0.0181565 | 1.58E-06 | 22.65517 |
| Gut microbiota abundance (genus Lachnoclostridium id.11308) | rs78068103 | 17 | 13816159 | A | 0.0886199 | 0.0194248 | 3.67E-06 | 20.81371 |
| Gut microbiota abundance (genus Lachnoclostridium id.11308) | rs789029 | 18 | 1053252 | C | -0.0641288 | 0.0137974 | 3.75E-06 | 21.60288 |
| Gut microbiota abundance (genus Lachnospira id.2004) | rs56791201 | 2 | 57701660 | C | -0.051823 | 0.0110697 | 2.93E-06 | 21.91661 |
| Gut microbiota abundance (genus Lachnospiraceae FCS020 group id.11314) | rs10093861 | 8 | 121244406 | G | -0.0568869 | 0.0121152 | 3.06E-06 | 22.0477 |
| Gut microbiota abundance (genus Lachnospiraceae FCS020 group id.11314) | rs1363769 | 19 | 17865305 | C | 0.200628 | 0.0449372 | 1.58E-06 | 19.93293 |
| Gut microbiota abundance (genus Lachnospiraceae FCS020 group id.11314) | rs2862811 | 3 | 165996415 | C | -0.0564927 | 0.0121736 | 3.92E-06 | 21.53509 |
| Gut microbiota abundance (genus Lachnospiraceae FCS020 group id.11314) | rs35035870 | 11 | 3248876 | T | -0.190615 | 0.0414403 | 2.62E-06 | 21.1577 |
| Gut microbiota abundance (genus Lachnospiraceae FCS020 group id.11314) | rs7249113 | 19 | 2571232 | G | 0.0679483 | 0.0133496 | 3.72E-07 | 25.90721 |
| Gut microbiota abundance (genus Lachnospiraceae FCS020 group id.11314) | rs72793667 | 2 | 53946431 | A | -0.116881 | 0.0246589 | 1.63E-06 | 22.46676 |
| Gut microbiota abundance (genus Lachnospiraceae FCS020 group id.11314) | rs9788306 | 13 | 40419314 | C | -0.062804 | 0.0130744 | 1.39E-06 | 23.07443 |
| Gut microbiota abundance (genus Lachnospiraceae NC2004 group id.11316) | rs3756315 | 5 | 149544722 | A | -0.0883469 | 0.0188397 | 3.33E-06 | 21.99048 |
| Gut microbiota abundance (genus Lachnospiraceae NC2004 group id.11316) | rs6116753 | 20 | 5330700 | G | 0.0994748 | 0.0209139 | 2.92E-06 | 22.62331 |
| Gut microbiota abundance (genus Lachnospiraceae ND3007 group id.11317) | rs9932954 | 16 | 1100633 | A | -0.0561864 | 0.0115984 | 1.25E-06 | 23.46747 |
| Gut microbiota abundance (genus Lachnospiraceae NK4A136 group id.11319) | rs160061 | 5 | 6116659 | G | -0.0513831 | 0.0108094 | 2.12E-06 | 22.59631 |
| Gut microbiota abundance (genus Lachnospiraceae NK4A136 group id.11319) | rs68104925 | 14 | 100177681 | T | -0.0549104 | 0.0115385 | 2.37E-06 | 22.64699 |
| Gut microbiota abundance (genus Lachnospiraceae NK4A136 group id.11319) | rs73044693 | 19 | 51256120 | A | -0.10758 | 0.0229884 | 3.57E-06 | 21.90007 |
| Gut microbiota abundance (genus Lachnospiraceae NK4A136 group id.11319) | rs7616165 | 3 | 190415145 | G | -0.230542 | 0.0483465 | 2.77E-06 | 22.73893 |
| Gut microbiota abundance (genus Lachnospiraceae NK4A136 group id.11319) | rs76193507 | 3 | 161749981 | A | -0.22973 | 0.0499776 | 2.93E-06 | 21.12928 |
| Gut microbiota abundance (genus Lachnospiraceae NK4A136 group id.11319) | rs7832116 | 8 | 4842929 | A | -0.0714756 | 0.0151703 | 3.57E-06 | 22.19869 |
| Gut microbiota abundance (genus Lachnospiraceae NK4A136 group id.11319) | rs954878 | 1 | 54578401 | A | -0.0520656 | 0.0109082 | 1.78E-06 | 22.7822 |
| Gut microbiota abundance (genus Lachnospiraceae UCG001 id.11321) | rs2050911 | 1 | 82384407 | G | 0.0751466 | 0.0153934 | 1.11E-06 | 23.8314 |
| Gut microbiota abundance (genus Lachnospiraceae UCG001 id.11321) | rs437876 | 3 | 42568440 | T | 0.0784637 | 0.0144768 | 7.17E-08 | 29.37598 |
| Gut microbiota abundance (genus Lachnospiraceae UCG001 id.11321) | rs573933 | 9 | 14477851 | T | -0.107898 | 0.0232346 | 3.11E-06 | 21.56534 |
| Gut microbiota abundance (genus Lachnospiraceae UCG001 id.11321) | rs74034332 | 16 | 79092359 | G | 0.168045 | 0.0382637 | 3.33E-06 | 19.28756 |
| Gut microbiota abundance (genus Lachnospiraceae UCG001 id.11321) | rs78848836 | 1 | 53773248 | A | -0.118868 | 0.0259753 | 3.38E-06 | 20.94155 |
| Gut microbiota abundance (genus Lachnospiraceae UCG001 id.11321) | rs9403580 | 6 | 145006780 | C | 0.107801 | 0.0229777 | 3.47E-06 | 22.01063 |
| Gut microbiota abundance (genus Lachnospiraceae UCG001 id.11321) | rs985416 | 3 | 148269083 | T | -0.0970258 | 0.0181807 | 1.46E-07 | 28.48087 |
| Gut microbiota abundance (genus Lachnospiraceae UCG004 id.11324) | rs11128180 | 3 | 70592215 | A | 0.0648288 | 0.0140126 | 4.52E-06 | 21.40418 |
| Gut microbiota abundance (genus Lachnospiraceae UCG004 id.11324) | rs12673420 | 7 | 71303007 | G | 0.0554351 | 0.0118375 | 2.98E-06 | 21.93056 |
| Gut microbiota abundance (genus Lachnospiraceae UCG004 id.11324) | rs12747809 | 1 | 240359089 | A | 0.062197 | 0.0125685 | 8.65E-07 | 24.48905 |
| Gut microbiota abundance (genus Lachnospiraceae UCG004 id.11324) | rs12894272 | 14 | 40536481 | G | -0.0579792 | 0.0125229 | 4.34E-06 | 21.43555 |
| Gut microbiota abundance (genus Lachnospiraceae UCG004 id.11324) | rs2444793 | 6 | 81318836 | T | 0.0542433 | 0.0118188 | 4.77E-06 | 21.06422 |
| Gut microbiota abundance (genus Lachnospiraceae UCG004 id.11324) | rs2882478 | 2 | 49904589 | G | -0.0577091 | 0.0118338 | 1.21E-06 | 23.78155 |
| Gut microbiota abundance (genus Lachnospiraceae UCG004 id.11324) | rs35182105 | 12 | 24572337 | A | -0.109724 | 0.024221 | 4.87E-06 | 20.52197 |
| Gut microbiota abundance (genus Lachnospiraceae UCG008 id.11328) | rs10741777 | 11 | 19572557 | T | -0.0973842 | 0.0194791 | 7.69E-07 | 24.9942 |
| Gut microbiota abundance (genus Lachnospiraceae UCG008 id.11328) | rs10793103 | 11 | 74391775 | T | -0.0974344 | 0.0181279 | 9.35E-08 | 28.88881 |
| Gut microbiota abundance (genus Lachnospiraceae UCG008 id.11328) | rs10801803 | 1 | 90818872 | G | -0.117029 | 0.0243085 | 1.40E-06 | 23.17772 |
| Gut microbiota abundance (genus Lachnospiraceae UCG008 id.11328) | rs13024781 | 2 | 168628871 | T | -0.0798769 | 0.0168846 | 2.29E-06 | 22.38004 |
| Gut microbiota abundance (genus Lachnospiraceae UCG008 id.11328) | rs57091572 | 6 | 81214803 | A | -0.110443 | 0.0235801 | 2.86E-06 | 21.9374 |
| Gut microbiota abundance (genus Lachnospiraceae UCG008 id.11328) | rs62277846 | 2 | 228100140 | C | 0.102292 | 0.0212319 | 1.59E-06 | 23.21163 |
| Gut microbiota abundance (genus Lachnospiraceae UCG008 id.11328) | rs67078837 | 4 | 114220647 | T | -0.0845848 | 0.0170693 | 7.68E-07 | 24.55575 |
| Gut microbiota abundance (genus Lachnospiraceae UCG008 id.11328) | rs955844 | 16 | 84992314 | A | 0.112064 | 0.0228369 | 1.81E-06 | 24.08008 |
| Gut microbiota abundance (genus Lachnospiraceae UCG010 id.11330) | rs10414815 | 19 | 42586245 | C | -0.10453 | 0.0230253 | 4.24E-06 | 20.60968 |
| Gut microbiota abundance (genus Lachnospiraceae UCG010 id.11330) | rs11192447 | 10 | 83848397 | A | 0.126592 | 0.024345 | 4.69E-07 | 27.03915 |
| Gut microbiota abundance (genus Lachnospiraceae UCG010 id.11330) | rs12346653 | 9 | 92267239 | C | 0.0657686 | 0.0139569 | 2.70E-06 | 22.20543 |
| Gut microbiota abundance (genus Lachnospiraceae UCG010 id.11330) | rs74315802 | 14 | 29872341 | G | 0.0867262 | 0.0183477 | 3.19E-06 | 22.34279 |
| Gut microbiota abundance (genus Lachnospiraceae UCG010 id.11330) | rs9981767 | 21 | 44079875 | A | 0.0655062 | 0.0131994 | 9.96E-07 | 24.62955 |
| Gut microbiota abundance (genus Lactobacillus id.1837) | rs11674854 | 2 | 28903275 | C | -0.0852686 | 0.0176504 | 1.59E-06 | 23.3383 |
| Gut microbiota abundance (genus Lactobacillus id.1837) | rs1530559 | 2 | 135755629 | G | 0.0804001 | 0.0178207 | 4.93E-06 | 20.35465 |
| Gut microbiota abundance (genus Lactobacillus id.1837) | rs16861661 | 1 | 18501459 | G | -0.183147 | 0.038148 | 1.28E-06 | 23.04921 |
| Gut microbiota abundance (genus Lactobacillus id.1837) | rs62314653 | 4 | 109896462 | C | 0.187692 | 0.0394585 | 2.24E-06 | 22.62614 |
| Gut microbiota abundance (genus Lactobacillus id.1837) | rs7399658 | 13 | 23834968 | G | -0.107134 | 0.0221883 | 3.12E-06 | 23.31345 |
| Gut microbiota abundance (genus Lactobacillus id.1837) | rs768253 | 8 | 69010103 | T | -0.079195 | 0.0171791 | 4.25E-06 | 21.25175 |
| Gut microbiota abundance (genus Lactobacillus id.1837) | rs921925 | 19 | 6928017 | A | 0.0985077 | 0.0203229 | 9.72E-07 | 23.49465 |
| Gut microbiota abundance (genus Lactococcus id.1851) | rs10417872 | 19 | 28767353 | G | -0.118306 | 0.024522 | 1.29E-06 | 23.27565 |
| Gut microbiota abundance (genus Lactococcus id.1851) | rs123059 | 17 | 2699935 | C | 0.13671 | 0.0274694 | 1.27E-06 | 24.76864 |
| Gut microbiota abundance (genus Lactococcus id.1851) | rs2293361 | 2 | 54114864 | C | -0.199221 | 0.0430969 | 1.40E-06 | 21.3687 |
| Gut microbiota abundance (genus Lactococcus id.1851) | rs4766997 | 12 | 113161438 | C | 0.114599 | 0.023839 | 2.06E-06 | 23.10924 |
| Gut microbiota abundance (genus Lactococcus id.1851) | rs55910161 | 10 | 71518643 | C | 0.146426 | 0.0307367 | 2.36E-06 | 22.69457 |
| Gut microbiota abundance (genus Marvinbryantia id.2005) | rs1187983 | 1 | 58441860 | C | -0.0935456 | 0.0193174 | 2.02E-06 | 23.45035 |
| Gut microbiota abundance (genus Marvinbryantia id.2005) | rs2724813 | 10 | 12516924 | G | 0.0840769 | 0.0167551 | 6.28E-07 | 25.18021 |
| Gut microbiota abundance (genus Marvinbryantia id.2005) | rs2842896 | 6 | 132881664 | C | -0.0649396 | 0.0131146 | 7.25E-07 | 24.51936 |
| Gut microbiota abundance (genus Marvinbryantia id.2005) | rs61884471 | 11 | 45826561 | G | 0.124426 | 0.0248431 | 1.01E-06 | 25.0848 |
| Gut microbiota abundance (genus Methanobrevibacter id.123) | rs10202904 | 2 | 125440268 | G | 0.112811 | 0.0239106 | 3.09E-06 | 22.25984 |
| Gut microbiota abundance (genus Methanobrevibacter id.123) | rs76029318 | 13 | 41963791 | T | 0.222849 | 0.0454319 | 1.08E-06 | 24.06022 |
| Gut microbiota abundance (genus Methanobrevibacter id.123) | rs894996 | 4 | 104418307 | C | 0.214213 | 0.0456045 | 3.82E-06 | 22.06359 |
| Gut microbiota abundance (genus Odoribacter id.952) | rs10093869 | 8 | 1266824 | A | -0.0577795 | 0.0125389 | 3.67E-06 | 21.23385 |
| Gut microbiota abundance (genus Odoribacter id.952) | rs28417404 | 14 | 70944224 | A | -0.0726896 | 0.0161372 | 3.68E-06 | 20.29029 |
| Gut microbiota abundance (genus Odoribacter id.952) | rs77779484 | 12 | 67655943 | G | -0.133489 | 0.0268526 | 6.56E-07 | 24.71259 |
| Gut microbiota abundance (genus Olsenella id.822) | rs1035588 | 2 | 150077190 | A | -0.108148 | 0.0236848 | 4.86E-06 | 20.84959 |
| Gut microbiota abundance (genus Olsenella id.822) | rs17148768 | 10 | 10777085 | G | 0.140434 | 0.0295602 | 2.20E-06 | 22.56991 |
| Gut microbiota abundance (genus Olsenella id.822) | rs2759329 | 1 | 231960352 | A | 0.111131 | 0.0237218 | 3.43E-06 | 21.947 |
| Gut microbiota abundance (genus Olsenella id.822) | rs35225860 | 1 | 247642270 | A | -0.223604 | 0.0482391 | 3.87E-06 | 21.48626 |
| Gut microbiota abundance (genus Olsenella id.822) | rs62112538 | 19 | 4925018 | C | -0.19943 | 0.0407033 | 1.19E-06 | 24.00611 |
| Gut microbiota abundance (genus Olsenella id.822) | rs72691585 | 9 | 21884425 | C | -0.249079 | 0.0520814 | 2.95E-06 | 22.87225 |
| Gut microbiota abundance (genus Oscillibacter id.2063) | rs11627628 | 14 | 21479605 | T | 0.143961 | 0.0290223 | 1.01E-06 | 24.60515 |
| Gut microbiota abundance (genus Oscillibacter id.2063) | rs12649930 | 4 | 3656291 | T | 0.121589 | 0.0259612 | 4.09E-06 | 21.93507 |
| Gut microbiota abundance (genus Oscillibacter id.2063) | rs133832 | 22 | 44834707 | A | -0.0795527 | 0.0162411 | 1.15E-06 | 23.99269 |
| Gut microbiota abundance (genus Oscillibacter id.2063) | rs16866406 | 2 | 179457147 | A | 0.0988765 | 0.0208795 | 3.08E-06 | 22.4257 |
| Gut microbiota abundance (genus Oscillibacter id.2063) | rs16934185 | 9 | 1798324 | A | -0.129568 | 0.028157 | 4.38E-06 | 21.17497 |
| Gut microbiota abundance (genus Oscillibacter id.2063) | rs234108 | 1 | 184942671 | A | 0.0749553 | 0.0152632 | 9.16E-07 | 24.11646 |
| Gut microbiota abundance (genus Oscillibacter id.2063) | rs36095275 | 14 | 32270129 | C | -0.0752368 | 0.0156861 | 1.40E-06 | 23.00545 |
| Gut microbiota abundance (genus Oscillibacter id.2063) | rs4506202 | 8 | 21598077 | G | 0.0711323 | 0.0152261 | 3.21E-06 | 21.82511 |
| Gut microbiota abundance (genus Oscillibacter id.2063) | rs61883564 | 11 | 79013843 | A | -0.101351 | 0.0221016 | 3.39E-06 | 21.02852 |
| Gut microbiota abundance (genus Oscillibacter id.2063) | rs761240 | 20 | 49507892 | G | 0.17664 | 0.0388812 | 2.04E-06 | 20.63948 |
| Gut microbiota abundance (genus Oscillibacter id.2063) | rs9393920 | 6 | 28580593 | G | 0.0744662 | 0.0151081 | 9.92E-07 | 24.29398 |
| Gut microbiota abundance (genus Oscillospira id.2064) | rs12206468 | 6 | 18093691 | G | -0.133016 | 0.0269733 | 1.04E-06 | 24.31866 |
| Gut microbiota abundance (genus Oscillospira id.2064) | rs1954532 | 14 | 28151415 | C | 0.0826246 | 0.0175252 | 2.27E-06 | 22.22761 |
| Gut microbiota abundance (genus Oscillospira id.2064) | rs28889936 | 4 | 89483300 | A | 0.114051 | 0.0252838 | 3.37E-06 | 20.34761 |
| Gut microbiota abundance (genus Oxalobacter id.2978) | rs10464997 | 8 | 20902693 | G | 0.137691 | 0.0294804 | 3.30E-06 | 21.81445 |
| Gut microbiota abundance (genus Oxalobacter id.2978) | rs11108500 | 12 | 96819204 | A | -0.199099 | 0.0427327 | 3.74E-06 | 21.70788 |
| Gut microbiota abundance (genus Oxalobacter id.2978) | rs12002250 | 9 | 19682558 | A | 0.217122 | 0.0466317 | 1.42E-06 | 21.6793 |
| Gut microbiota abundance (genus Oxalobacter id.2978) | rs1569853 | 6 | 38550301 | T | -0.138078 | 0.0296981 | 3.65E-06 | 21.61681 |
| Gut microbiota abundance (genus Oxalobacter id.2978) | rs36057338 | 4 | 189935314 | G | 0.207847 | 0.0421439 | 8.80E-07 | 24.32305 |
| Gut microbiota abundance (genus Oxalobacter id.2978) | rs4428215 | 3 | 171947435 | G | 0.130293 | 0.0242237 | 7.51E-08 | 28.93085 |
| Gut microbiota abundance (genus Oxalobacter id.2978) | rs6000536 | 22 | 37421469 | C | -0.130992 | 0.0253804 | 2.06E-07 | 26.63745 |
| Gut microbiota abundance (genus Oxalobacter id.2978) | rs736744 | 9 | 87514407 | T | -0.117882 | 0.0211262 | 2.57E-08 | 31.13524 |
| Gut microbiota abundance (genus Parabacteroides id.954) | rs115602804 | 3 | 191572365 | G | 0.10308 | 0.0222736 | 1.93E-06 | 21.41746 |
| Gut microbiota abundance (genus Parabacteroides id.954) | rs4236095 | 6 | 47368687 | A | -0.0761961 | 0.0157042 | 1.93E-06 | 23.54148 |
| Gut microbiota abundance (genus Parabacteroides id.954) | rs60884758 | 9 | 2217340 | C | -0.0702667 | 0.0142249 | 5.71E-07 | 24.40061 |
| Gut microbiota abundance (genus Paraprevotella id.962) | rs140997932 | 3 | 149298792 | T | -0.162376 | 0.0354178 | 2.11E-06 | 21.01844 |
| Gut microbiota abundance (genus Paraprevotella id.962) | rs145020347 | 11 | 114526679 | A | -0.12465 | 0.0262323 | 4.03E-06 | 22.57937 |
| Gut microbiota abundance (genus Paraprevotella id.962) | rs17785622 | 6 | 83459314 | A | 0.248065 | 0.0524304 | 1.93E-06 | 22.38539 |
| Gut microbiota abundance (genus Paraprevotella id.962) | rs2081023 | 5 | 174606261 | A | -0.122561 | 0.0236507 | 2.64E-07 | 26.85447 |
| Gut microbiota abundance (genus Paraprevotella id.962) | rs3008582 | 1 | 195971173 | T | 0.10572 | 0.0227246 | 4.36E-06 | 21.64322 |
| Gut microbiota abundance (genus Paraprevotella id.962) | rs4756632 | 11 | 41079703 | G | -0.138905 | 0.0289897 | 3.82E-06 | 22.95875 |
| Gut microbiota abundance (genus Paraprevotella id.962) | rs4767113 | 12 | 114131978 | C | 0.0882466 | 0.018382 | 2.14E-06 | 23.04679 |
| Gut microbiota abundance (genus Paraprevotella id.962) | rs9900242 | 17 | 69135631 | A | -0.0852967 | 0.0175212 | 1.14E-06 | 23.69937 |
| Gut microbiota abundance (genus Parasutterella id.2892) | rs10899911 | 10 | 44293839 | A | -0.0717102 | 0.0148151 | 1.15E-06 | 23.42894 |
| Gut microbiota abundance (genus Parasutterella id.2892) | rs2090816 | 6 | 137615592 | C | -0.0840967 | 0.0177315 | 2.90E-06 | 22.49401 |
| Gut microbiota abundance (genus Parasutterella id.2892) | rs35055552 | 8 | 114804024 | T | 0.109554 | 0.0235433 | 3.35E-06 | 21.65319 |
| Gut microbiota abundance (genus Parasutterella id.2892) | rs55877868 | 17 | 14692867 | A | -0.104458 | 0.0228089 | 2.87E-06 | 20.97368 |
| Gut microbiota abundance (genus Parasutterella id.2892) | rs7303158 | 12 | 5275540 | C | 0.0646859 | 0.0134256 | 1.33E-06 | 23.2141 |
| Gut microbiota abundance (genus Parasutterella id.2892) | rs7572229 | 2 | 72235444 | A | -0.066273 | 0.0132736 | 6.32E-07 | 24.92848 |
| Gut microbiota abundance (genus Parasutterella id.2892) | rs78383039 | 2 | 178954708 | T | -0.146315 | 0.0297119 | 1.57E-06 | 24.25029 |
| Gut microbiota abundance (genus Parasutterella id.2892) | rs8039785 | 15 | 67316307 | G | -0.0618345 | 0.0133 | 3.62E-06 | 21.61516 |
| Gut microbiota abundance (genus Parasutterella id.2892) | rs823424 | 8 | 16674526 | G | -0.0713448 | 0.0156958 | 4.95E-06 | 20.6613 |
| Gut microbiota abundance (genus Peptococcus id.2037) | rs10031059 | 4 | 35358594 | C | 0.121167 | 0.0225844 | 1.24E-07 | 28.78403 |
| Gut microbiota abundance (genus Peptococcus id.2037) | rs11001941 | 10 | 78716030 | G | -0.195611 | 0.0392217 | 1.33E-06 | 24.87332 |
| Gut microbiota abundance (genus Peptococcus id.2037) | rs2054133 | 2 | 33691809 | A | -0.0895433 | 0.0188332 | 2.14E-06 | 22.6057 |
| Gut microbiota abundance (genus Peptococcus id.2037) | rs413827 | 14 | 57985112 | G | 0.110229 | 0.0237523 | 3.30E-06 | 21.53676 |
| Gut microbiota abundance (genus Peptococcus id.2037) | rs5770862 | 22 | 50973113 | T | 0.162018 | 0.0356813 | 3.22E-06 | 20.61794 |
| Gut microbiota abundance (genus Peptococcus id.2037) | rs6918730 | 6 | 99020289 | A | -0.135311 | 0.0289742 | 1.15E-06 | 21.80938 |
| Gut microbiota abundance (genus Peptococcus id.2037) | rs7033353 | 9 | 104595848 | G | -0.090152 | 0.018995 | 2.22E-06 | 22.52538 |
| Gut microbiota abundance (genus Peptococcus id.2037) | rs77681628 | 13 | 91182556 | C | 0.200307 | 0.0387328 | 2.69E-07 | 26.7445 |
| Gut microbiota abundance (genus Phascolarctobacterium id.2168) | rs12618201 | 2 | 174436533 | A | 0.0641657 | 0.0138202 | 3.38E-06 | 21.55645 |
| Gut microbiota abundance (genus Phascolarctobacterium id.2168) | rs1264476 | 8 | 102434276 | G | -0.0767335 | 0.0166041 | 4.30E-06 | 21.35696 |
| Gut microbiota abundance (genus Phascolarctobacterium id.2168) | rs56069061 | 3 | 168339417 | G | -0.111306 | 0.0230694 | 1.87E-06 | 23.27901 |
| Gut microbiota abundance (genus Phascolarctobacterium id.2168) | rs56157888 | 4 | 183252836 | A | 0.0954864 | 0.0193976 | 1.09E-06 | 24.23188 |
| Gut microbiota abundance (genus Phascolarctobacterium id.2168) | rs74540770 | 3 | 186553389 | G | -0.121014 | 0.0258621 | 3.60E-06 | 21.89493 |
| Gut microbiota abundance (genus Phascolarctobacterium id.2168) | rs75882962 | 12 | 53098742 | T | 0.0968661 | 0.0190575 | 3.19E-07 | 25.8352 |
| Gut microbiota abundance (genus Prevotella7 id.11182) | rs2240542 | 2 | 242066314 | C | 0.12085 | 0.0261776 | 4.84E-06 | 21.31246 |
| Gut microbiota abundance (genus Prevotella7 id.11182) | rs430270 | 3 | 60474379 | A | 0.139155 | 0.0297055 | 2.87E-06 | 21.94441 |
| Gut microbiota abundance (genus Prevotella7 id.11182) | rs57404562 | 2 | 12101762 | C | 0.155486 | 0.0316057 | 6.22E-07 | 24.20203 |
| Gut microbiota abundance (genus Prevotella7 id.11182) | rs9959718 | 18 | 71442664 | G | 0.133012 | 0.0275361 | 1.90E-06 | 23.33333 |
| Gut microbiota abundance (genus Prevotella9 id.11183) | rs111509883 | 19 | 639161 | T | 0.171131 | 0.0347624 | 1.24E-06 | 24.23471 |
| Gut microbiota abundance (genus Prevotella9 id.11183) | rs11685699 | 2 | 11232685 | C | -0.141357 | 0.0295665 | 2.03E-06 | 22.85782 |
| Gut microbiota abundance (genus Prevotella9 id.11183) | rs117271932 | 22 | 44236460 | A | 0.208089 | 0.0440393 | 2.82E-06 | 22.32633 |
| Gut microbiota abundance (genus Prevotella9 id.11183) | rs2683313 | 8 | 19115604 | G | 0.0724576 | 0.0151603 | 1.69E-06 | 22.84296 |
| Gut microbiota abundance (genus Prevotella9 id.11183) | rs9428102 | 1 | 118852817 | A | -0.0778845 | 0.0176068 | 4.62E-06 | 19.56776 |
| Gut microbiota abundance (genus Rikenellaceae RC9 gut group id.11191) | rs17582787 | 4 | 149562638 | A | -0.157739 | 0.0339924 | 3.55E-06 | 21.53349 |
| Gut microbiota abundance (genus Rikenellaceae RC9 gut group id.11191) | rs2900503 | 9 | 112657543 | T | 0.172329 | 0.0326671 | 1.55E-07 | 27.82887 |
| Gut microbiota abundance (genus Rikenellaceae RC9 gut group id.11191) | rs2998141 | 10 | 135010198 | C | 0.136346 | 0.0293073 | 4.42E-06 | 21.64378 |
| Gut microbiota abundance (genus Rikenellaceae RC9 gut group id.11191) | rs4717843 | 7 | 73334987 | G | -0.119376 | 0.0260548 | 4.72E-06 | 20.99223 |
| Gut microbiota abundance (genus Rikenellaceae RC9 gut group id.11191) | rs80309088 | 6 | 165668732 | G | 0.17405 | 0.0383423 | 4.56E-06 | 20.60591 |
| Gut microbiota abundance (genus Rikenellaceae RC9 gut group id.11191) | rs9887954 | 1 | 164800873 | G | -0.114849 | 0.0249013 | 4.81E-06 | 21.2721 |
| Gut microbiota abundance (genus Romboutsia id.11347) | rs10279978 | 7 | 5319387 | A | -0.0622202 | 0.0127675 | 1.17E-06 | 23.74931 |
| Gut microbiota abundance (genus Romboutsia id.11347) | rs61841503 | 10 | 17019559 | G | 0.092888 | 0.0171454 | 4.00E-08 | 29.35107 |
| Gut microbiota abundance (genus Romboutsia id.11347) | rs62504452 | 7 | 145760298 | A | -0.0710048 | 0.0156654 | 4.66E-06 | 20.54435 |
| Gut microbiota abundance (genus Roseburia id.2012) | rs16910295 | 11 | 12009569 | T | -0.0980434 | 0.020957 | 2.91E-06 | 21.88661 |
| Gut microbiota abundance (genus Roseburia id.2012) | rs2160994 | 12 | 50650057 | C | -0.0550687 | 0.0112481 | 9.70E-07 | 23.96908 |
| Gut microbiota abundance (genus Roseburia id.2012) | rs2943022 | 5 | 89598914 | T | 0.0493786 | 0.0106763 | 4.11E-06 | 21.39124 |
| Gut microbiota abundance (genus Roseburia id.2012) | rs6445851 | 3 | 57116228 | A | 0.0497336 | 0.0108158 | 3.53E-06 | 21.14377 |
| Gut microbiota abundance (genus Roseburia id.2012) | rs6930661 | 6 | 12774611 | C | -0.0961587 | 0.0204975 | 2.48E-06 | 22.00774 |
| Gut microbiota abundance (genus Roseburia id.2012) | rs9300744 | 13 | 103117486 | C | -0.0588452 | 0.0126228 | 4.75E-06 | 21.73255 |
| Gut microbiota abundance (genus Ruminiclostridium5 id.11355) | rs10827477 | 10 | 35262588 | A | -0.0547387 | 0.0115163 | 2.19E-06 | 22.59243 |
| Gut microbiota abundance (genus Ruminiclostridium5 id.11355) | rs113753996 | 5 | 32477664 | T | 0.0820689 | 0.0174463 | 3.99E-06 | 22.12843 |
| Gut microbiota abundance (genus Ruminiclostridium5 id.11355) | rs1492620 | 6 | 50405920 | T | -0.0830505 | 0.0180073 | 3.53E-06 | 21.27097 |
| Gut microbiota abundance (genus Ruminiclostridium5 id.11355) | rs2482038 | 10 | 12835085 | C | 0.0518991 | 0.0108777 | 1.70E-06 | 22.76384 |
| Gut microbiota abundance (genus Ruminiclostridium5 id.11355) | rs6121460 | 20 | 60293505 | G | 0.0932955 | 0.0199206 | 2.64E-06 | 21.93394 |
| Gut microbiota abundance (genus Ruminiclostridium5 id.11355) | rs79968837 | 20 | 41177956 | A | -0.0950311 | 0.0193507 | 1.15E-06 | 24.11783 |
| Gut microbiota abundance (genus Ruminiclostridium6 id.11356) | rs10829821 | 10 | 132651293 | T | -0.097606 | 0.0216072 | 3.47E-06 | 20.40591 |
| Gut microbiota abundance (genus Ruminiclostridium6 id.11356) | rs11992182 | 8 | 79766499 | A | 0.0625299 | 0.0137876 | 4.65E-06 | 20.56829 |
| Gut microbiota abundance (genus Ruminiclostridium6 id.11356) | rs61060922 | 16 | 72136154 | T | 0.15913 | 0.0322283 | 1.09E-06 | 24.37975 |
| Gut microbiota abundance (genus Ruminiclostridium6 id.11356) | rs663262 | 11 | 86179076 | C | 0.13498 | 0.0310715 | 3.39E-06 | 18.87185 |
| Gut microbiota abundance (genus Ruminiclostridium6 id.11356) | rs71414120 | 14 | 56938952 | T | 0.200894 | 0.0406434 | 1.08E-06 | 24.43171 |
| Gut microbiota abundance (genus Ruminiclostridium6 id.11356) | rs72991535 | 18 | 76018244 | T | 0.135604 | 0.0295174 | 4.95E-06 | 21.10517 |
| Gut microbiota abundance (genus Ruminiclostridium6 id.11356) | rs77193512 | 11 | 40289063 | A | 0.0736533 | 0.0153163 | 1.30E-06 | 23.12473 |
| Gut microbiota abundance (genus Ruminiclostridium6 id.11356) | rs79968172 | 1 | 240503826 | G | 0.11614 | 0.0243083 | 1.66E-06 | 22.8273 |
| Gut microbiota abundance (genus Ruminiclostridium9 id.11357) | rs115044523 | 7 | 47013498 | G | -0.0980026 | 0.0202616 | 2.37E-06 | 23.39525 |
| Gut microbiota abundance (genus Ruminiclostridium9 id.11357) | rs12040548 | 1 | 247710285 | G | 0.0570422 | 0.0122359 | 3.15E-06 | 21.73305 |
| Gut microbiota abundance (genus Ruminiclostridium9 id.11357) | rs6082461 | 20 | 2210530 | C | -0.0586425 | 0.0131004 | 4.87E-06 | 20.03807 |
| Gut microbiota abundance (genus Ruminiclostridium9 id.11357) | rs9522712 | 13 | 90441578 | T | 0.0699728 | 0.0154939 | 4.66E-06 | 20.39562 |
| Gut microbiota abundance (genus Ruminococcaceae NK4A214 group id.11358) | rs12642039 | 4 | 159932022 | C | 0.0553003 | 0.0119397 | 3.43E-06 | 21.45202 |
| Gut microbiota abundance (genus Ruminococcaceae NK4A214 group id.11358) | rs12731 | 2 | 239087912 | A | -0.0527684 | 0.0115053 | 4.87E-06 | 21.03546 |
| Gut microbiota abundance (genus Ruminococcaceae NK4A214 group id.11358) | rs136761 | 22 | 49796014 | A | 0.0587521 | 0.0119155 | 8.15E-07 | 24.31209 |
| Gut microbiota abundance (genus Ruminococcaceae NK4A214 group id.11358) | rs147475196 | 3 | 26509287 | A | -0.133769 | 0.0295196 | 4.72E-06 | 20.53478 |
| Gut microbiota abundance (genus Ruminococcaceae NK4A214 group id.11358) | rs35559912 | 5 | 35288938 | T | -0.0925071 | 0.0203674 | 4.89E-06 | 20.62904 |
| Gut microbiota abundance (genus Ruminococcaceae NK4A214 group id.11358) | rs4814689 | 20 | 18030357 | C | -0.1083 | 0.0230708 | 4.55E-06 | 22.03594 |
| Gut microbiota abundance (genus Ruminococcaceae NK4A214 group id.11358) | rs5994253 | 22 | 17793012 | A | -0.0811302 | 0.0157621 | 2.35E-07 | 26.49335 |
| Gut microbiota abundance (genus Ruminococcaceae NK4A214 group id.11358) | rs7573569 | 2 | 141897042 | T | 0.107739 | 0.0233638 | 3.23E-06 | 21.26468 |
| Gut microbiota abundance (genus Ruminococcaceae UCG002 id.11360) | rs10916131 | 1 | 227563126 | C | -0.069333 | 0.0146753 | 2.87E-06 | 22.32061 |
| Gut microbiota abundance (genus Ruminococcaceae UCG002 id.11360) | rs10927423 | 1 | 14732458 | C | -0.0713617 | 0.0147712 | 8.50E-07 | 23.33989 |
| Gut microbiota abundance (genus Ruminococcaceae UCG002 id.11360) | rs116974815 | 11 | 111712942 | C | -0.189731 | 0.0396566 | 2.03E-06 | 22.88999 |
| Gut microbiota abundance (genus Ruminococcaceae UCG002 id.11360) | rs11750293 | 5 | 123822114 | G | -0.0578304 | 0.0120512 | 1.76E-06 | 23.02777 |
| Gut microbiota abundance (genus Ruminococcaceae UCG002 id.11360) | rs12463378 | 19 | 54475808 | A | -0.0522061 | 0.0112086 | 2.96E-06 | 21.69401 |
| Gut microbiota abundance (genus Ruminococcaceae UCG002 id.11360) | rs55793120 | 12 | 47384118 | T | 0.137396 | 0.027414 | 4.81E-07 | 25.11906 |
| Gut microbiota abundance (genus Ruminococcaceae UCG002 id.11360) | rs7120052 | 11 | 86335459 | A | 0.0624796 | 0.0135523 | 1.97E-06 | 21.25448 |
| Gut microbiota abundance (genus Ruminococcaceae UCG002 id.11360) | rs7155595 | 14 | 77502546 | C | 0.0569929 | 0.0116986 | 1.15E-06 | 23.73415 |
| Gut microbiota abundance (genus Ruminococcaceae UCG002 id.11360) | rs77564310 | 15 | 80713184 | A | -0.0713093 | 0.0140855 | 3.29E-07 | 25.62995 |
| Gut microbiota abundance (genus Ruminococcaceae UCG002 id.11360) | rs79016051 | 1 | 238938497 | C | -0.0887747 | 0.0189424 | 2.34E-06 | 21.96385 |
| Gut microbiota abundance (genus Ruminococcaceae UCG003 id.11361) | rs10490280 | 2 | 37905976 | C | -0.0672094 | 0.0143309 | 4.16E-06 | 21.99445 |
| Gut microbiota abundance (genus Ruminococcaceae UCG003 id.11361) | rs11613919 | 12 | 75496463 | G | 0.0727604 | 0.0155716 | 1.63E-06 | 21.83352 |
| Gut microbiota abundance (genus Ruminococcaceae UCG003 id.11361) | rs16959793 | 15 | 35071718 | A | -0.062527 | 0.0131259 | 2.22E-06 | 22.69222 |
| Gut microbiota abundance (genus Ruminococcaceae UCG003 id.11361) | rs3013089 | 1 | 13794594 | G | -0.0551473 | 0.0120346 | 4.38E-06 | 20.99835 |
| Gut microbiota abundance (genus Ruminococcaceae UCG003 id.11361) | rs4452755 | 8 | 82026852 | A | -0.0634463 | 0.0134741 | 3.29E-06 | 22.17242 |
| Gut microbiota abundance (genus Ruminococcaceae UCG003 id.11361) | rs4532474 | 6 | 105781538 | G | 0.0769231 | 0.017045 | 4.82E-06 | 20.36665 |
| Gut microbiota abundance (genus Ruminococcaceae UCG003 id.11361) | rs646327 | 19 | 49209851 | G | 0.0586696 | 0.0118369 | 7.83E-07 | 24.5669 |
| Gut microbiota abundance (genus Ruminococcaceae UCG003 id.11361) | rs6759615 | 2 | 205238716 | A | 0.102523 | 0.0200204 | 7.86E-07 | 26.22389 |
| Gut microbiota abundance (genus Ruminococcaceae UCG003 id.11361) | rs73341549 | 7 | 51541468 | T | -0.169857 | 0.0318768 | 1.51E-07 | 28.3934 |
| Gut microbiota abundance (genus Ruminococcaceae UCG004 id.11362) | rs12125734 | 1 | 103732214 | G | 0.133972 | 0.0257416 | 2.09E-07 | 27.08676 |
| Gut microbiota abundance (genus Ruminococcaceae UCG004 id.11362) | rs511258 | 3 | 169966722 | G | -0.0757474 | 0.0162475 | 4.52E-06 | 21.73514 |
| Gut microbiota abundance (genus Ruminococcaceae UCG004 id.11362) | rs6769553 | 3 | 54427795 | A | 0.0849743 | 0.0157451 | 7.91E-08 | 29.12623 |
| Gut microbiota abundance (genus Ruminococcaceae UCG005 id.11363) | rs10873449 | 14 | 94630731 | C | -0.0654843 | 0.0143973 | 4.11E-06 | 20.6877 |
| Gut microbiota abundance (genus Ruminococcaceae UCG005 id.11363) | rs10950694 | 7 | 18026604 | C | -0.0577628 | 0.011412 | 4.30E-07 | 25.61964 |
| Gut microbiota abundance (genus Ruminococcaceae UCG005 id.11363) | rs114279581 | 2 | 198122357 | A | -0.146604 | 0.0316034 | 3.22E-06 | 21.5191 |
| Gut microbiota abundance (genus Ruminococcaceae UCG005 id.11363) | rs12288512 | 11 | 27747671 | A | 0.066635 | 0.0144356 | 3.10E-06 | 21.30763 |
| Gut microbiota abundance (genus Ruminococcaceae UCG005 id.11363) | rs12458218 | 18 | 22575871 | T | 0.0677322 | 0.0144525 | 2.41E-06 | 21.96364 |
| Gut microbiota abundance (genus Ruminococcaceae UCG005 id.11363) | rs2893871 | 10 | 62662781 | G | -0.073644 | 0.015548 | 3.54E-06 | 22.43498 |
| Gut microbiota abundance (genus Ruminococcaceae UCG005 id.11363) | rs34781347 | 20 | 16312851 | G | 0.188685 | 0.0386484 | 6.05E-07 | 23.83481 |
| Gut microbiota abundance (genus Ruminococcaceae UCG005 id.11363) | rs7449320 | 5 | 154003091 | C | 0.0599163 | 0.0130836 | 4.81E-06 | 20.97179 |
| Gut microbiota abundance (genus Ruminococcaceae UCG005 id.11363) | rs7555878 | 1 | 187906034 | G | -0.0586677 | 0.0125277 | 2.81E-06 | 21.93085 |
| Gut microbiota abundance (genus Ruminococcaceae UCG009 id.11366) | rs12508214 | 4 | 7426937 | C | -0.0774649 | 0.0168937 | 4.75E-06 | 21.02618 |
| Gut microbiota abundance (genus Ruminococcaceae UCG009 id.11366) | rs1550196 | 17 | 32786955 | A | -0.130842 | 0.0262478 | 1.13E-06 | 24.84898 |
| Gut microbiota abundance (genus Ruminococcaceae UCG009 id.11366) | rs2058609 | 12 | 12970792 | G | -0.0816464 | 0.017472 | 3.12E-06 | 21.83679 |
| Gut microbiota abundance (genus Ruminococcaceae UCG009 id.11366) | rs2192926 | 2 | 75512668 | A | -0.089048 | 0.0192989 | 4.88E-06 | 21.29037 |
| Gut microbiota abundance (genus Ruminococcaceae UCG009 id.11366) | rs4079028 | 1 | 202299085 | C | 0.0915623 | 0.019936 | 3.28E-06 | 21.09392 |
| Gut microbiota abundance (genus Ruminococcaceae UCG009 id.11366) | rs4708333 | 6 | 78106701 | T | -0.084033 | 0.0174757 | 1.56E-06 | 23.12228 |
| Gut microbiota abundance (genus Ruminococcaceae UCG010 id.11367) | rs12597105 | 16 | 5233941 | A | -0.0670855 | 0.0144415 | 4.87E-06 | 21.57907 |
| Gut microbiota abundance (genus Ruminococcaceae UCG010 id.11367) | rs2820282 | 6 | 104728618 | C | 0.0592304 | 0.0125917 | 2.85E-06 | 22.1269 |
| Gut microbiota abundance (genus Ruminococcaceae UCG010 id.11367) | rs682403 | 9 | 135968557 | A | -0.0588161 | 0.0124671 | 2.37E-06 | 22.25674 |
| Gut microbiota abundance (genus Ruminococcaceae UCG010 id.11367) | rs6958419 | 7 | 16349864 | C | -0.0585718 | 0.0124994 | 2.84E-06 | 21.9583 |
| Gut microbiota abundance (genus Ruminococcaceae UCG011 id.11368) | rs12636310 | 3 | 185469491 | G | 0.132725 | 0.0282037 | 2.81E-06 | 22.1459 |
| Gut microbiota abundance (genus Ruminococcaceae UCG011 id.11368) | rs12724320 | 1 | 179370499 | C | -0.120881 | 0.0249229 | 1.52E-06 | 23.52442 |
| Gut microbiota abundance (genus Ruminococcaceae UCG011 id.11368) | rs1416041 | 6 | 105073676 | A | -0.182339 | 0.0339898 | 7.04E-08 | 28.77809 |
| Gut microbiota abundance (genus Ruminococcaceae UCG011 id.11368) | rs2729556 | 7 | 111763988 | T | 0.109097 | 0.0233699 | 3.19E-06 | 21.79274 |
| Gut microbiota abundance (genus Ruminococcaceae UCG011 id.11368) | rs79113084 | 12 | 29634919 | C | -0.152166 | 0.0317519 | 2.06E-06 | 22.96655 |
| Gut microbiota abundance (genus Ruminococcaceae UCG011 id.11368) | rs9729514 | 1 | 219901055 | G | -0.184934 | 0.0394616 | 2.37E-06 | 21.96262 |
| Gut microbiota abundance (genus Ruminococcaceae UCG013 id.11370) | rs11581881 | 1 | 9361576 | C | 0.0661211 | 0.0144736 | 4.73E-06 | 20.87022 |
| Gut microbiota abundance (genus Ruminococcaceae UCG013 id.11370) | rs12189346 | 5 | 141395140 | G | 0.0684961 | 0.014558 | 1.68E-06 | 22.13748 |
| Gut microbiota abundance (genus Ruminococcaceae UCG013 id.11370) | rs12485353 | 3 | 197049996 | G | -0.0607903 | 0.0130847 | 4.19E-06 | 21.58445 |
| Gut microbiota abundance (genus Ruminococcaceae UCG013 id.11370) | rs12781711 | 10 | 2219930 | C | -0.0656129 | 0.0117477 | 2.55E-08 | 31.19412 |
| Gut microbiota abundance (genus Ruminococcaceae UCG013 id.11370) | rs16918863 | 10 | 19780463 | A | 0.111491 | 0.0240157 | 4.15E-06 | 21.55208 |
| Gut microbiota abundance (genus Ruminococcaceae UCG013 id.11370) | rs75088940 | 12 | 30245557 | T | -0.0942968 | 0.0200712 | 2.55E-06 | 22.07228 |
| Gut microbiota abundance (genus Ruminococcaceae UCG013 id.11370) | rs76973485 | 3 | 9534657 | G | 0.194976 | 0.0418213 | 3.35E-06 | 21.73538 |
| Gut microbiota abundance (genus Ruminococcaceae UCG014 id.11371) | rs10941294 | 5 | 36435597 | C | -0.122057 | 0.0260017 | 2.40E-06 | 22.03545 |
| Gut microbiota abundance (genus Ruminococcaceae UCG014 id.11371) | rs115777838 | 5 | 26110626 | T | -0.18835 | 0.0386643 | 4.62E-07 | 23.73072 |
| Gut microbiota abundance (genus Ruminococcaceae UCG014 id.11371) | rs12638134 | 3 | 101334609 | T | 0.0582548 | 0.0119657 | 1.21E-06 | 23.70212 |
| Gut microbiota abundance (genus Ruminococcaceae UCG014 id.11371) | rs56105232 | 9 | 14363769 | G | 0.139276 | 0.0299132 | 2.91E-06 | 21.67838 |
| Gut microbiota abundance (genus Ruminococcaceae UCG014 id.11371) | rs72809222 | 2 | 57205854 | T | 0.0671775 | 0.0139838 | 2.41E-06 | 23.07795 |
| Gut microbiota abundance (genus Ruminococcaceae UCG014 id.11371) | rs995642 | 2 | 134854659 | C | 0.060048 | 0.0126417 | 1.90E-06 | 22.56244 |
| Gut microbiota abundance (genus Ruminococcus gauvreauii group id.11342) | rs10931481 | 2 | 191954852 | A | -0.0610096 | 0.0130484 | 3.38E-06 | 21.86159 |
| Gut microbiota abundance (genus Ruminococcus gauvreauii group id.11342) | rs12539819 | 7 | 153487396 | C | 0.110654 | 0.0240684 | 4.49E-06 | 21.13683 |
| Gut microbiota abundance (genus Ruminococcus gauvreauii group id.11342) | rs1391597 | 12 | 61119266 | C | 0.0590284 | 0.0124874 | 1.86E-06 | 22.34488 |
| Gut microbiota abundance (genus Ruminococcus gauvreauii group id.11342) | rs2047242 | 10 | 29201860 | A | -0.067603 | 0.0133738 | 2.46E-07 | 25.55185 |
| Gut microbiota abundance (genus Ruminococcus gauvreauii group id.11342) | rs289410 | 15 | 85563483 | A | 0.0654926 | 0.0139101 | 2.27E-06 | 22.16787 |
| Gut microbiota abundance (genus Ruminococcus gauvreauii group id.11342) | rs71386687 | 16 | 2767894 | T | 0.121037 | 0.02386 | 2.91E-07 | 25.7333 |
| Gut microbiota abundance (genus Ruminococcus gauvreauii group id.11342) | rs9870933 | 3 | 112372317 | G | -0.0621643 | 0.0126072 | 8.49E-07 | 24.31335 |
| Gut microbiota abundance (genus Ruminococcus gnavus group id.14376) | rs12136548 | 1 | 115168097 | C | 0.0901946 | 0.0196476 | 3.10E-06 | 21.07376 |
| Gut microbiota abundance (genus Ruminococcus gnavus group id.14376) | rs13163520 | 5 | 18662686 | G | -0.127377 | 0.0233883 | 5.61E-08 | 29.66093 |
| Gut microbiota abundance (genus Ruminococcus gnavus group id.14376) | rs2909242 | 8 | 129224680 | A | 0.0909965 | 0.018351 | 7.41E-07 | 24.58838 |
| Gut microbiota abundance (genus Ruminococcus gnavus group id.14376) | rs3124783 | 9 | 135838591 | G | 0.11599 | 0.0249099 | 2.67E-06 | 21.68189 |
| Gut microbiota abundance (genus Ruminococcus gnavus group id.14376) | rs62167033 | 2 | 144700007 | T | 0.185289 | 0.0396295 | 3.50E-06 | 21.8606 |
| Gut microbiota abundance (genus Ruminococcus gnavus group id.14376) | rs934940 | 2 | 122096018 | A | -0.105045 | 0.0229588 | 2.74E-06 | 20.93401 |
| Gut microbiota abundance (genus Ruminococcus gnavus group id.14376) | rs9872758 | 3 | 66701616 | T | 0.0849233 | 0.0176643 | 1.66E-06 | 23.11324 |
| Gut microbiota abundance (genus Ruminococcus torques group id.14377) | rs10904297 | 10 | 4639762 | A | -0.167804 | 0.0389812 | 2.69E-06 | 18.5308 |
| Gut microbiota abundance (genus Ruminococcus torques group id.14377) | rs35866622 | 19 | 49218060 | T | -0.0612024 | 0.0109421 | 2.21E-08 | 31.28496 |
| Gut microbiota abundance (genus Ruminococcus torques group id.14377) | rs4073731 | 11 | 132663147 | T | 0.0651948 | 0.0142221 | 4.05E-06 | 21.0135 |
| Gut microbiota abundance (genus Ruminococcus torques group id.14377) | rs8080469 | 17 | 14532347 | G | 0.0490727 | 0.0107036 | 3.50E-06 | 21.01939 |
| Gut microbiota abundance (genus Ruminococcus1 id.11373) | rs11783695 | 8 | 144596903 | G | -0.0734114 | 0.0161398 | 4.73E-06 | 20.68858 |
| Gut microbiota abundance (genus Ruminococcus1 id.11373) | rs17781867 | 17 | 71256018 | C | 0.0999271 | 0.0211728 | 1.96E-06 | 22.2746 |
| Gut microbiota abundance (genus Ruminococcus1 id.11373) | rs6493760 | 15 | 55439182 | T | -0.0535262 | 0.0115885 | 3.38E-06 | 21.3343 |
| Gut microbiota abundance (genus Ruminococcus1 id.11373) | rs7117576 | 11 | 114023573 | A | 0.0829505 | 0.0170891 | 6.48E-07 | 23.56132 |
| Gut microbiota abundance (genus Ruminococcus1 id.11373) | rs7583465 | 2 | 10533921 | C | 0.0527654 | 0.0112619 | 2.56E-06 | 21.95205 |
| Gut microbiota abundance (genus Ruminococcus2 id.11374) | rs12986628 | 2 | 238103439 | T | -0.0665937 | 0.0140168 | 2.14E-06 | 22.57192 |
| Gut microbiota abundance (genus Ruminococcus2 id.11374) | rs2368224 | 2 | 182656416 | T | 0.199648 | 0.0438453 | 3.63E-06 | 20.73404 |
| Gut microbiota abundance (genus Ruminococcus2 id.11374) | rs58681734 | 9 | 137557890 | A | 0.0723875 | 0.0160787 | 4.18E-06 | 20.26867 |
| Gut microbiota abundance (genus Ruminococcus2 id.11374) | rs75140805 | 11 | 12148588 | T | 0.0836825 | 0.0176445 | 3.95E-06 | 22.49317 |
| Gut microbiota abundance (genus Ruminococcus2 id.11374) | rs7635831 | 3 | 173040924 | G | 0.0618344 | 0.0129031 | 1.98E-06 | 22.9653 |
| Gut microbiota abundance (genus Ruminococcus2 id.11374) | rs78120384 | 3 | 106664363 | A | -0.192815 | 0.0392045 | 3.31E-07 | 24.18855 |
| Gut microbiota abundance (genus Sellimonas id.14369) | rs13417181 | 2 | 174308394 | T | 0.166509 | 0.0337521 | 7.62E-07 | 24.33738 |
| Gut microbiota abundance (genus Sellimonas id.14369) | rs2016057 | 15 | 52105282 | C | 0.125878 | 0.0256153 | 1.03E-06 | 24.14909 |
| Gut microbiota abundance (genus Sellimonas id.14369) | rs2187447 | 11 | 79332972 | A | 0.243457 | 0.0527695 | 3.98E-06 | 21.28524 |
| Gut microbiota abundance (genus Sellimonas id.14369) | rs2371572 | 2 | 213214174 | A | 0.127349 | 0.0250862 | 4.46E-07 | 25.77041 |
| Gut microbiota abundance (genus Sellimonas id.14369) | rs4600608 | 2 | 180278564 | G | 0.137152 | 0.0301702 | 4.95E-06 | 20.66559 |
| Gut microbiota abundance (genus Sellimonas id.14369) | rs56203279 | 7 | 111501524 | T | -0.124045 | 0.0269115 | 3.72E-06 | 21.24627 |
| Gut microbiota abundance (genus Senegalimassilia id.11160) | rs11787826 | 9 | 34332382 | C | 0.081327 | 0.0171154 | 2.63E-06 | 22.57852 |
| Gut microbiota abundance (genus Senegalimassilia id.11160) | rs7225245 | 17 | 48379615 | A | -0.079173 | 0.0170421 | 4.18E-06 | 21.58281 |
| Gut microbiota abundance (genus Slackia id.825) | rs12440440 | 15 | 34041896 | A | 0.0901934 | 0.0190582 | 2.63E-06 | 22.39679 |
| Gut microbiota abundance (genus Slackia id.825) | rs16894137 | 8 | 96946291 | C | -0.122792 | 0.0263047 | 2.71E-06 | 21.79081 |
| Gut microbiota abundance (genus Slackia id.825) | rs4492265 | 7 | 13523683 | G | 0.0905757 | 0.0191658 | 2.41E-06 | 22.33415 |
| Gut microbiota abundance (genus Slackia id.825) | rs8901 | 17 | 74267010 | C | 0.0934594 | 0.0186813 | 6.07E-07 | 25.02833 |
| Gut microbiota abundance (genus Streptococcus id.1853) | rs10448310 | 9 | 93556174 | A | -0.0517935 | 0.0111324 | 3.31E-06 | 21.64576 |
| Gut microbiota abundance (genus Streptococcus id.1853) | rs11110281 | 12 | 100584014 | T | -0.137519 | 0.0227398 | 2.58E-09 | 36.57229 |
| Gut microbiota abundance (genus Streptococcus id.1853) | rs11720390 | 3 | 94103591 | G | 0.107024 | 0.0228121 | 3.59E-06 | 22.0106 |
| Gut microbiota abundance (genus Streptococcus id.1853) | rs11764382 | 7 | 46774896 | A | -0.0695345 | 0.0143671 | 1.29E-06 | 23.42407 |
| Gut microbiota abundance (genus Streptococcus id.1853) | rs17708276 | 8 | 10199548 | A | -0.0793955 | 0.0170628 | 3.04E-06 | 21.65166 |
| Gut microbiota abundance (genus Streptococcus id.1853) | rs1918540 | 11 | 131649446 | A | -0.059639 | 0.0128148 | 2.44E-06 | 21.65893 |
| Gut microbiota abundance (genus Streptococcus id.1853) | rs4968759 | 17 | 61298020 | A | -0.0515109 | 0.0112068 | 3.78E-06 | 21.12686 |
| Gut microbiota abundance (genus Streptococcus id.1853) | rs6806351 | 3 | 132058723 | T | -0.0633829 | 0.0136647 | 4.94E-06 | 21.51513 |
| Gut microbiota abundance (genus Streptococcus id.1853) | rs7916711 | 10 | 28588269 | A | 0.102891 | 0.0217362 | 2.72E-06 | 22.4072 |
| Gut microbiota abundance (genus Streptococcus id.1853) | rs9903102 | 17 | 80576624 | C | -0.0709483 | 0.0155275 | 4.18E-06 | 20.87762 |
| Gut microbiota abundance (genus Subdoligranulum id.2070) | rs10065321 | 5 | 141857415 | T | -0.0512828 | 0.0108105 | 2.10E-06 | 22.50359 |
| Gut microbiota abundance (genus Subdoligranulum id.2070) | rs2114677 | 10 | 125472937 | C | -0.104176 | 0.0230829 | 2.72E-06 | 20.36829 |
| Gut microbiota abundance (genus Subdoligranulum id.2070) | rs2171249 | 6 | 153694029 | C | 0.106744 | 0.0233213 | 4.51E-06 | 20.94987 |
| Gut microbiota abundance (genus Subdoligranulum id.2070) | rs3761728 | 4 | 48990885 | G | 0.0543458 | 0.0118866 | 3.87E-06 | 20.90339 |
| Gut microbiota abundance (genus Subdoligranulum id.2070) | rs4347804 | 2 | 218215847 | G | -0.166063 | 0.0357483 | 2.18E-06 | 21.57918 |
| Gut microbiota abundance (genus Subdoligranulum id.2070) | rs6555306 | 5 | 4671383 | C | 0.0740777 | 0.0155463 | 2.81E-06 | 22.70497 |
| Gut microbiota abundance (genus Sutterella id.2896) | rs13173038 | 5 | 58499183 | A | -0.0718076 | 0.0151627 | 2.73E-06 | 22.42785 |
| Gut microbiota abundance (genus Sutterella id.2896) | rs143438747 | 1 | 63278549 | T | -0.145793 | 0.0306867 | 3.28E-06 | 22.57215 |
| Gut microbiota abundance (genus Sutterella id.2896) | rs2321387 | 13 | 58689340 | G | -0.0592876 | 0.0124509 | 1.87E-06 | 22.6739 |
| Gut microbiota abundance (genus Sutterella id.2896) | rs7499539 | 16 | 85038065 | A | 0.0617478 | 0.0130998 | 2.36E-06 | 22.21844 |
| Gut microbiota abundance (genus Terrisporobacter id.11348) | rs1883097 | 11 | 8938819 | C | 0.226399 | 0.0454642 | 4.16E-07 | 24.79761 |
| Gut microbiota abundance (genus Terrisporobacter id.11348) | rs2872237 | 19 | 17026940 | A | 0.0814505 | 0.0175943 | 3.97E-06 | 21.43105 |
| Gut microbiota abundance (genus Turicibacter id.2162) | rs11054680 | 12 | 12242455 | T | -0.104751 | 0.0226997 | 2.31E-06 | 21.29493 |
| Gut microbiota abundance (genus Turicibacter id.2162) | rs12603364 | 17 | 43117476 | T | 0.110861 | 0.0225598 | 8.67E-07 | 24.14833 |
| Gut microbiota abundance (genus Turicibacter id.2162) | rs149744580 | 2 | 63366962 | A | 0.169883 | 0.0315478 | 7.01E-08 | 28.99758 |
| Gut microbiota abundance (genus Turicibacter id.2162) | rs2834977 | 21 | 36929643 | T | -0.0959995 | 0.0208261 | 3.96E-06 | 21.2482 |
| Gut microbiota abundance (genus Turicibacter id.2162) | rs4869133 | 5 | 95717619 | G | 0.131186 | 0.027197 | 2.55E-06 | 23.2666 |
| Gut microbiota abundance (genus Turicibacter id.2162) | rs55756211 | 7 | 130972179 | T | -0.115115 | 0.0240708 | 2.81E-06 | 22.87088 |
| Gut microbiota abundance (genus Tyzzerella3 id.11335) | rs4904512 | 14 | 89595945 | T | -0.117151 | 0.0250306 | 3.09E-06 | 21.90531 |
| Gut microbiota abundance (genus Tyzzerella3 id.11335) | rs55799124 | 17 | 3738781 | A | -0.11435 | 0.0238604 | 1.34E-06 | 22.96767 |
| Gut microbiota abundance (genus Tyzzerella3 id.11335) | rs67476743 | 19 | 1030320 | T | 0.132164 | 0.022208 | 3.74E-09 | 35.41665 |
| Gut microbiota abundance (genus Tyzzerella3 id.11335) | rs6920448 | 6 | 5755626 | C | -0.141081 | 0.0305446 | 4.15E-06 | 21.3338 |
| Gut microbiota abundance (genus Tyzzerella3 id.11335) | rs7019909 | 9 | 33113322 | T | 0.144156 | 0.0301626 | 1.76E-06 | 22.84167 |
| Gut microbiota abundance (genus Tyzzerella3 id.11335) | rs7333521 | 13 | 81593016 | T | -0.207191 | 0.0453121 | 4.88E-06 | 20.90804 |
| Gut microbiota abundance (genus Tyzzerella3 id.11335) | rs75091807 | 13 | 35023691 | G | -0.184966 | 0.0383029 | 1.71E-06 | 23.31957 |
| Gut microbiota abundance (genus Tyzzerella3 id.11335) | rs7561370 | 2 | 57810406 | C | -0.131341 | 0.0286292 | 1.52E-06 | 21.04661 |
| Gut microbiota abundance (genus Veillonella id.2198) | rs1882878 | 21 | 30010673 | A | -0.0768966 | 0.0163908 | 2.98E-06 | 22.00969 |
| Gut microbiota abundance (genus Veillonella id.2198) | rs2013594 | 11 | 44302154 | C | 0.0720698 | 0.0155153 | 3.42E-06 | 21.57676 |
| Gut microbiota abundance (genus Veillonella id.2198) | rs62376424 | 5 | 119914285 | C | -0.0762216 | 0.0163499 | 3.65E-06 | 21.73331 |
| Gut microbiota abundance (genus Veillonella id.2198) | rs742016 | 22 | 45604800 | A | -0.0688571 | 0.0149769 | 4.66E-06 | 21.1375 |
| Gut microbiota abundance (genus Victivallis id.2256) | rs11899949 | 2 | 38049069 | G | 0.130568 | 0.0276239 | 2.77E-06 | 22.34105 |
| Gut microbiota abundance (genus Victivallis id.2256) | rs12512543 | 4 | 9608445 | A | -0.178036 | 0.0374344 | 2.54E-06 | 22.61903 |
| Gut microbiota abundance (genus Victivallis id.2256) | rs4764863 | 12 | 102514184 | G | 0.121561 | 0.0246058 | 8.22E-07 | 24.40695 |
| Gut microbiota abundance (genus Victivallis id.2256) | rs4895919 | 6 | 131630319 | C | 0.116918 | 0.0247515 | 2.75E-06 | 22.31309 |
| Gut microbiota abundance (genus Victivallis id.2256) | rs56349194 | 11 | 9312796 | A | -0.158501 | 0.0315231 | 6.26E-07 | 25.28169 |
| Gut microbiota abundance (order Actinomycetales id.420) | rs2889192 | 9 | 76394568 | T | -0.0884052 | 0.0194997 | 3.97E-06 | 20.55416 |
| Gut microbiota abundance (order Actinomycetales id.420) | rs35011108 | 6 | 133007480 | A | 0.241546 | 0.0503803 | 1.88E-06 | 22.98678 |
| Gut microbiota abundance (order Bacillales id.1674) | rs10233278 | 7 | 117496144 | T | -0.116319 | 0.0248527 | 3.51E-06 | 21.90555 |
| Gut microbiota abundance (order Bacillales id.1674) | rs4617108 | 7 | 49423832 | G | -0.248861 | 0.0525929 | 1.98E-06 | 22.39028 |
| Gut microbiota abundance (order Bacillales id.1674) | rs62640857 | 12 | 69332975 | A | 0.148238 | 0.0326455 | 4.49E-06 | 20.61923 |
| Gut microbiota abundance (order Bacillales id.1674) | rs74420793 | 4 | 127766709 | A | -0.164413 | 0.0353763 | 3.07E-06 | 21.59969 |
| Gut microbiota abundance (order Bacteroidales id.913) | rs111845179 | 14 | 59317502 | T | 0.102648 | 0.0213843 | 9.24E-07 | 23.04151 |
| Gut microbiota abundance (order Bacteroidales id.913) | rs2032750 | 2 | 53831026 | C | 0.0508393 | 0.0106807 | 1.92E-06 | 22.65686 |
| Gut microbiota abundance (order Bacteroidales id.913) | rs55773148 | 13 | 70523029 | G | -0.121514 | 0.0236759 | 3.90E-07 | 26.34145 |
| Gut microbiota abundance (order Bacteroidales id.913) | rs73975615 | 17 | 6461200 | G | -0.207018 | 0.0442633 | 1.22E-06 | 21.87402 |
| Gut microbiota abundance (order Bacteroidales id.913) | rs7631304 | 3 | 89339527 | G | -0.0645771 | 0.0132957 | 8.37E-07 | 23.59037 |
| Gut microbiota abundance (order Bacteroidales id.913) | rs929878 | 16 | 74290641 | T | 0.0548532 | 0.012153 | 4.73E-06 | 20.37215 |
| Gut microbiota abundance (order Bifidobacteriales id.432) | rs13020688 | 2 | 192878532 | G | 0.0584045 | 0.0122081 | 1.57E-06 | 22.8874 |
| Gut microbiota abundance (order Bifidobacteriales id.432) | rs182549 | 2 | 136616754 | T | -0.11707 | 0.0126703 | 5.94E-20 | 85.37239 |
| Gut microbiota abundance (order Bifidobacteriales id.432) | rs4957061 | 5 | 521096 | T | 0.0569873 | 0.0116907 | 1.15E-06 | 23.76157 |
| Gut microbiota abundance (order Bifidobacteriales id.432) | rs7322849 | 13 | 112859829 | T | 0.110676 | 0.0200996 | 1.74E-08 | 30.3202 |
| Gut microbiota abundance (order Bifidobacteriales id.432) | rs73797465 | 5 | 142793467 | T | -0.0942612 | 0.0208421 | 4.85E-06 | 20.45422 |
| Gut microbiota abundance (order Bifidobacteriales id.432) | rs857444 | 6 | 14617591 | C | 0.0553965 | 0.0120669 | 3.82E-06 | 21.07527 |
| Gut microbiota abundance (order Burkholderiales id.2874) | rs1928341 | 1 | 153240013 | G | -0.0507782 | 0.0110587 | 4.52E-06 | 21.08368 |
| Gut microbiota abundance (order Burkholderiales id.2874) | rs2321387 | 13 | 58689340 | G | -0.0508514 | 0.0109588 | 3.26E-06 | 21.53177 |
| Gut microbiota abundance (order Burkholderiales id.2874) | rs2613606 | 7 | 111285025 | T | 0.0499561 | 0.0109264 | 4.13E-06 | 20.90369 |
| Gut microbiota abundance (order Burkholderiales id.2874) | rs4033856 | 4 | 45642485 | T | -0.0833445 | 0.0167489 | 5.67E-07 | 24.76175 |
| Gut microbiota abundance (order Burkholderiales id.2874) | rs6087811 | 20 | 30596130 | T | -0.101587 | 0.0198817 | 2.88E-07 | 26.10774 |
| Gut microbiota abundance (order Burkholderiales id.2874) | rs62191117 | 2 | 239900775 | A | 0.0679995 | 0.0132471 | 2.79E-07 | 26.34934 |
| Gut microbiota abundance (order Burkholderiales id.2874) | rs62395635 | 5 | 173497796 | T | 0.109911 | 0.0236375 | 2.90E-06 | 21.62117 |
| Gut microbiota abundance (order Burkholderiales id.2874) | rs7638039 | 3 | 70588939 | T | 0.0580987 | 0.012673 | 4.84E-06 | 21.01716 |
| Gut microbiota abundance (order Clostridiales id.1863) | rs10774377 | 12 | 5942519 | G | -0.0523003 | 0.0113859 | 3.81E-06 | 21.09957 |
| Gut microbiota abundance (order Clostridiales id.1863) | rs112334273 | 21 | 40703251 | G | 0.0639383 | 0.0127404 | 4.07E-07 | 25.18582 |
| Gut microbiota abundance (order Clostridiales id.1863) | rs13179700 | 5 | 149077788 | C | -0.0510953 | 0.0109569 | 3.52E-06 | 21.74636 |
| Gut microbiota abundance (order Clostridiales id.1863) | rs6814436 | 4 | 161507301 | C | -0.0741858 | 0.0150814 | 9.06E-07 | 24.19682 |
| Gut microbiota abundance (order Clostridiales id.1863) | rs6815608 | 4 | 152131744 | C | -0.104089 | 0.0210883 | 3.72E-07 | 24.36276 |
| Gut microbiota abundance (order Clostridiales id.1863) | rs72915163 | 18 | 48792829 | T | -0.0580246 | 0.0120558 | 1.39E-06 | 23.165 |
| Gut microbiota abundance (order Clostridiales id.1863) | rs992074 | 21 | 18567802 | T | -0.255441 | 0.0508788 | 8.95E-07 | 25.20621 |
| Gut microbiota abundance (order Coriobacteriales id.810) | rs11250875 | 10 | 1922731 | T | 0.06075 | 0.0130939 | 4.83E-06 | 21.52556 |
| Gut microbiota abundance (order Coriobacteriales id.810) | rs1816223 | 12 | 11494021 | G | 0.0586265 | 0.0129006 | 4.84E-06 | 20.65229 |
| Gut microbiota abundance (order Coriobacteriales id.810) | rs34739816 | 17 | 37376685 | G | 0.0965017 | 0.0207666 | 3.88E-06 | 21.5943 |
| Gut microbiota abundance (order Coriobacteriales id.810) | rs719099 | 10 | 65799217 | A | 0.0778402 | 0.0155815 | 5.43E-07 | 24.95683 |
| Gut microbiota abundance (order Desulfovibrionales id.3156) | rs112381107 | 9 | 15269705 | C | 0.21009 | 0.0456974 | 3.22E-06 | 21.13624 |
| Gut microbiota abundance (order Desulfovibrionales id.3156) | rs11599763 | 10 | 11855599 | C | 0.0554862 | 0.0117486 | 2.61E-06 | 22.30477 |
| Gut microbiota abundance (order Desulfovibrionales id.3156) | rs17791387 | 9 | 81834426 | A | -0.0727501 | 0.0154369 | 2.25E-06 | 22.20992 |
| Gut microbiota abundance (order Desulfovibrionales id.3156) | rs2692012 | 1 | 203991605 | G | -0.112244 | 0.0253553 | 2.27E-06 | 19.59696 |
| Gut microbiota abundance (order Desulfovibrionales id.3156) | rs2838334 | 21 | 45064961 | G | 0.0569024 | 0.0124223 | 4.17E-06 | 20.9825 |
| Gut microbiota abundance (order Desulfovibrionales id.3156) | rs4506934 | 17 | 2856662 | C | -0.0953383 | 0.0201367 | 2.43E-06 | 22.416 |
| Gut microbiota abundance (order Desulfovibrionales id.3156) | rs6058181 | 20 | 33694801 | C | 0.0836765 | 0.0166098 | 2.53E-07 | 25.37922 |
| Gut microbiota abundance (order Desulfovibrionales id.3156) | rs9928243 | 16 | 71541641 | C | -0.0544713 | 0.0117811 | 3.97E-06 | 21.37784 |
| Gut microbiota abundance (order Enterobacteriales id.3468) | rs2374342 | 2 | 42133542 | C | 0.0582927 | 0.0126195 | 4.52E-06 | 21.33753 |
| Gut microbiota abundance (order Enterobacteriales id.3468) | rs62210023 | 20 | 55340092 | A | 0.0606754 | 0.0130125 | 3.13E-06 | 21.74222 |
| Gut microbiota abundance (order Erysipelotrichales id.2148) | rs10781552 | 10 | 133897233 | C | -0.0552212 | 0.0116074 | 2.33E-06 | 22.63298 |
| Gut microbiota abundance (order Erysipelotrichales id.2148) | rs17530232 | 13 | 40385457 | A | 0.10305 | 0.0224648 | 2.79E-06 | 21.04219 |
| Gut microbiota abundance (order Erysipelotrichales id.2148) | rs2300774 | 3 | 195793712 | A | -0.0524152 | 0.0106784 | 8.95E-07 | 24.09362 |
| Gut microbiota abundance (order Erysipelotrichales id.2148) | rs35161940 | 17 | 70327224 | T | -0.0806041 | 0.0167641 | 1.85E-06 | 23.11819 |
| Gut microbiota abundance (order Erysipelotrichales id.2148) | rs4078432 | 14 | 48997206 | T | 0.0608929 | 0.0133764 | 4.23E-06 | 20.7231 |
| Gut microbiota abundance (order Erysipelotrichales id.2148) | rs62504403 | 8 | 38803551 | C | 0.0681159 | 0.0127883 | 1.12E-07 | 28.37079 |
| Gut microbiota abundance (order Erysipelotrichales id.2148) | rs7234058 | 18 | 5830507 | T | -0.0945759 | 0.0194091 | 9.12E-07 | 23.7438 |
| Gut microbiota abundance (order Erysipelotrichales id.2148) | rs8003149 | 14 | 56156504 | C | 0.0538798 | 0.0116888 | 4.08E-06 | 21.24771 |
| Gut microbiota abundance (order Gastranaerophilales id.1591) | rs11150282 | 16 | 80493705 | T | 0.098235 | 0.0197127 | 7.36E-07 | 24.83363 |
| Gut microbiota abundance (order Gastranaerophilales id.1591) | rs4129395 | 9 | 115975389 | G | 0.0904264 | 0.0185253 | 1.22E-06 | 23.82649 |
| Gut microbiota abundance (order Gastranaerophilales id.1591) | rs79790072 | 15 | 100747683 | T | 0.226103 | 0.0488017 | 3.54E-06 | 21.46559 |
| Gut microbiota abundance (order Gastranaerophilales id.1591) | rs9864379 | 3 | 14306949 | T | -0.160521 | 0.0292752 | 4.66E-08 | 30.06519 |
| Gut microbiota abundance (order Lactobacillales id.1800) | rs11110282 | 12 | 100585559 | A | -0.10239 | 0.0217849 | 3.96E-06 | 22.09042 |
| Gut microbiota abundance (order Lactobacillales id.1800) | rs34989881 | 19 | 51959855 | A | 0.113479 | 0.0246452 | 4.09E-06 | 21.20149 |
| Gut microbiota abundance (order Lactobacillales id.1800) | rs4028634 | 17 | 40835649 | C | -0.0532915 | 0.01101 | 1.35E-06 | 23.42833 |
| Gut microbiota abundance (order Lactobacillales id.1800) | rs57872228 | 1 | 200418805 | C | -0.0688415 | 0.0146994 | 2.58E-06 | 21.93317 |
| Gut microbiota abundance (order Lactobacillales id.1800) | rs77558518 | 5 | 174173171 | A | -0.106466 | 0.0223484 | 1.67E-06 | 22.69494 |
| Gut microbiota abundance (order Lactobacillales id.1800) | rs78938557 | 7 | 36349586 | T | 0.10553 | 0.0233581 | 2.31E-06 | 20.41159 |
| Gut microbiota abundance (order Lactobacillales id.1800) | rs9581006 | 13 | 24973509 | T | -0.22583 | 0.0469228 | 1.77E-06 | 23.16303 |
| Gut microbiota abundance (order Methanobacteriales id.120) | rs10202904 | 2 | 125440268 | G | 0.121754 | 0.0235357 | 3.01E-07 | 26.76161 |
| Gut microbiota abundance (order Methanobacteriales id.120) | rs6776814 | 3 | 15053083 | T | -0.199566 | 0.0411825 | 1.63E-06 | 23.48268 |
| Gut microbiota abundance (order Methanobacteriales id.120) | rs73457410 | 13 | 41956181 | A | 0.21534 | 0.0436695 | 1.41E-06 | 24.31605 |
| Gut microbiota abundance (order Methanobacteriales id.120) | rs894996 | 4 | 104418307 | C | 0.216999 | 0.0449076 | 1.88E-06 | 23.3494 |
| Gut microbiota abundance (order Mollicutes RF9 id.11579) | rs74603314 | 14 | 46519718 | T | 0.230767 | 0.0489701 | 2.28E-06 | 22.20677 |
| Gut microbiota abundance (order Mollicutes RF9 id.11579) | rs7706512 | 5 | 17443654 | A | -0.0657351 | 0.0138979 | 2.27E-06 | 22.37156 |
| Gut microbiota abundance (order NB1n id.3953) | rs11251024 | 10 | 2095726 | G | 0.104201 | 0.0206737 | 6.63E-07 | 25.40431 |
| Gut microbiota abundance (order NB1n id.3953) | rs11606187 | 11 | 91589600 | A | -0.154539 | 0.0326288 | 3.31E-06 | 22.43231 |
| Gut microbiota abundance (order NB1n id.3953) | rs13385922 | 2 | 234081324 | T | 0.0929494 | 0.0201348 | 3.97E-06 | 21.31074 |
| Gut microbiota abundance (order NB1n id.3953) | rs2172426 | 8 | 18229020 | T | 0.10212 | 0.0198947 | 3.17E-07 | 26.34795 |
| Gut microbiota abundance (order NB1n id.3953) | rs267959 | 5 | 10737802 | G | -0.0988612 | 0.0209676 | 2.62E-06 | 22.23076 |
| Gut microbiota abundance (order NB1n id.3953) | rs4383094 | 15 | 81694972 | C | -0.149188 | 0.0320662 | 4.28E-06 | 21.64576 |
| Gut microbiota abundance (order NB1n id.3953) | rs60583455 | 7 | 1321675 | T | 0.108934 | 0.021177 | 2.60E-07 | 26.4605 |
| Gut microbiota abundance (order NB1n id.3953) | rs72671304 | 14 | 39425971 | T | 0.172322 | 0.0370028 | 3.80E-06 | 21.68764 |
| Gut microbiota abundance (order NB1n id.3953) | rs7911787 | 10 | 98619568 | G | -0.223071 | 0.0470232 | 3.39E-06 | 22.50411 |
| Gut microbiota abundance (order Pasteurellales id.3688) | rs10965428 | 9 | 22718481 | C | -0.119902 | 0.0258219 | 4.29E-06 | 21.56138 |
| Gut microbiota abundance (order Pasteurellales id.3688) | rs35510 | 12 | 115491973 | A | 0.122729 | 0.0264958 | 4.02E-06 | 21.45558 |
| Gut microbiota abundance (order Pasteurellales id.3688) | rs4822728 | 22 | 26891808 | T | 0.0685479 | 0.0149032 | 4.72E-06 | 21.15579 |
| Gut microbiota abundance (order Pasteurellales id.3688) | rs72756943 | 5 | 26531908 | G | 0.13988 | 0.0303029 | 3.35E-06 | 21.30801 |
| Gut microbiota abundance (order Pasteurellales id.3688) | rs76022354 | 10 | 94306385 | C | 0.24289 | 0.0500406 | 1.83E-06 | 23.55994 |
| Gut microbiota abundance (order Pasteurellales id.3688) | rs78909003 | 9 | 105650242 | T | -0.241159 | 0.0498372 | 2.05E-06 | 23.4153 |
| Gut microbiota abundance (order Pasteurellales id.3688) | rs9382510 | 6 | 55448491 | C | -0.0881865 | 0.0169965 | 2.48E-07 | 26.92063 |
| Gut microbiota abundance (order Rhodospirillales id.2667) | rs1035406 | 5 | 119372737 | G | -0.114725 | 0.0247994 | 4.07E-06 | 21.40099 |
| Gut microbiota abundance (order Rhodospirillales id.2667) | rs11591293 | 10 | 113419797 | G | 0.0722755 | 0.0158186 | 4.69E-06 | 20.87594 |
| Gut microbiota abundance (order Rhodospirillales id.2667) | rs11630875 | 15 | 61775734 | T | 0.0945198 | 0.0202139 | 3.70E-06 | 21.86479 |
| Gut microbiota abundance (order Rhodospirillales id.2667) | rs1549633 | 5 | 27945645 | A | 0.0996735 | 0.0216667 | 3.88E-06 | 21.16284 |
| Gut microbiota abundance (order Rhodospirillales id.2667) | rs3754624 | 2 | 225633812 | C | 0.094128 | 0.0198389 | 2.68E-06 | 22.5114 |
| Gut microbiota abundance (order Rhodospirillales id.2667) | rs4278423 | 10 | 2670553 | T | 0.105215 | 0.0233778 | 3.98E-06 | 20.25574 |
| Gut microbiota abundance (order Rhodospirillales id.2667) | rs7001029 | 8 | 131958403 | C | 0.120726 | 0.0260989 | 2.83E-06 | 21.39721 |
| Gut microbiota abundance (order Rhodospirillales id.2667) | rs76784716 | 2 | 169033340 | A | 0.136039 | 0.0284399 | 1.31E-06 | 22.88078 |
| Gut microbiota abundance (order Rhodospirillales id.2667) | rs9813022 | 3 | 13726736 | A | -0.0830794 | 0.0162642 | 3.07E-07 | 26.09284 |
| Gut microbiota abundance (order Selenomonadales id.2165) | rs13086907 | 3 | 142135117 | G | 0.0625312 | 0.0131735 | 1.95E-06 | 22.53156 |
| Gut microbiota abundance (order Selenomonadales id.2165) | rs1643968 | 5 | 165266628 | T | -0.0565284 | 0.0112298 | 4.15E-07 | 25.33899 |
| Gut microbiota abundance (order Selenomonadales id.2165) | rs4722181 | 7 | 22817571 | T | 0.0501306 | 0.0105798 | 2.00E-06 | 22.45179 |
| Gut microbiota abundance (order Selenomonadales id.2165) | rs60274479 | 16 | 21249925 | T | -0.0659623 | 0.0134137 | 1.16E-06 | 24.18212 |
| Gut microbiota abundance (order Selenomonadales id.2165) | rs61249479 | 9 | 124912908 | A | 0.0777075 | 0.0168628 | 2.95E-06 | 21.2357 |
| Gut microbiota abundance (order Selenomonadales id.2165) | rs71405394 | 15 | 101245088 | G | -0.114178 | 0.02405 | 2.17E-06 | 22.539 |
| Gut microbiota abundance (order Selenomonadales id.2165) | rs73232831 | 4 | 17413426 | G | -0.151829 | 0.0314932 | 1.87E-06 | 23.24213 |
| Gut microbiota abundance (order Verrucomicrobiales id.4030) | rs111862613 | 12 | 130309670 | T | 0.090699 | 0.0196746 | 3.74E-06 | 21.25168 |
| Gut microbiota abundance (order Verrucomicrobiales id.4030) | rs117107102 | 18 | 49473635 | A | 0.204683 | 0.0431572 | 2.92E-06 | 22.4935 |
| Gut microbiota abundance (order Verrucomicrobiales id.4030) | rs11729256 | 4 | 95027272 | T | 0.0749798 | 0.0150177 | 6.73E-07 | 24.92767 |
| Gut microbiota abundance (order Verrucomicrobiales id.4030) | rs12908520 | 15 | 97570657 | G | 0.0618926 | 0.0130946 | 2.17E-06 | 22.3405 |
| Gut microbiota abundance (order Verrucomicrobiales id.4030) | rs2602429 | 16 | 81063149 | T | -0.0746844 | 0.0156194 | 2.58E-06 | 22.86289 |
| Gut microbiota abundance (order Verrucomicrobiales id.4030) | rs4242783 | 10 | 5064327 | A | -0.0689292 | 0.0147693 | 2.64E-06 | 21.78144 |
| Gut microbiota abundance (order Verrucomicrobiales id.4030) | rs4936098 | 11 | 130280667 | G | -0.0648843 | 0.0135928 | 1.12E-06 | 22.78565 |
| Gut microbiota abundance (order Verrucomicrobiales id.4030) | rs74542928 | 4 | 100544188 | T | 0.112164 | 0.0236422 | 1.63E-06 | 22.5077 |
| Gut microbiota abundance (order Verrucomicrobiales id.4030) | rs9349825 | 6 | 56341481 | A | -0.0704036 | 0.0147128 | 2.54E-06 | 22.89808 |
| Gut microbiota abundance (order Victivallales id.2254) | rs11770843 | 7 | 146795379 | C | 0.109431 | 0.0234879 | 1.91E-06 | 21.70663 |
| Gut microbiota abundance (order Victivallales id.2254) | rs17114848 | 15 | 25162535 | G | 0.152377 | 0.0324332 | 4.06E-06 | 22.07289 |
| Gut microbiota abundance (order Victivallales id.2254) | rs2031282 | 13 | 20687179 | A | 0.122368 | 0.0270329 | 4.38E-06 | 20.4904 |
| Gut microbiota abundance (order Victivallales id.2254) | rs2825714 | 21 | 21023966 | A | -0.13741 | 0.0289246 | 1.72E-06 | 22.56846 |
| Gut microbiota abundance (order Victivallales id.2254) | rs62570196 | 9 | 111086170 | C | -0.21635 | 0.0439866 | 1.08E-06 | 24.19207 |
| Gut microbiota abundance (order Victivallales id.2254) | rs77599476 | 20 | 61394262 | A | 0.230292 | 0.0480168 | 1.86E-06 | 23.0023 |
| Gut microbiota abundance (phylum Actinobacteria id.400) | rs1397793 | 5 | 90471451 | A | 0.0521387 | 0.0111587 | 3.74E-06 | 21.83198 |
| Gut microbiota abundance (phylum Actinobacteria id.400) | rs4429415 | 2 | 213756191 | C | 0.0581908 | 0.0111343 | 2.05E-07 | 27.31384 |
| Gut microbiota abundance (phylum Actinobacteria id.400) | rs55888705 | 4 | 1517826 | A | 0.0534221 | 0.0109975 | 1.31E-06 | 23.59685 |
| Gut microbiota abundance (phylum Actinobacteria id.400) | rs6496870 | 15 | 92467422 | C | -0.0510777 | 0.0113809 | 4.62E-06 | 20.14232 |
| Gut microbiota abundance (phylum Actinobacteria id.400) | rs74037001 | 14 | 23847194 | G | -0.0819391 | 0.0165334 | 6.71E-07 | 24.56168 |
| Gut microbiota abundance (phylum Actinobacteria id.400) | rs7570971 | 2 | 135837906 | A | 0.086653 | 0.0113623 | 1.41E-14 | 58.16138 |
| Gut microbiota abundance (phylum Actinobacteria id.400) | rs8047955 | 16 | 81776768 | G | -0.0521115 | 0.0111192 | 2.66E-06 | 21.96444 |
| Gut microbiota abundance (phylum Actinobacteria id.400) | rs857444 | 6 | 14617591 | C | 0.0507315 | 0.0109885 | 3.80E-06 | 21.31467 |
| Gut microbiota abundance (phylum Bacteroidetes id.905) | rs111845179 | 14 | 59317502 | T | 0.104177 | 0.0213837 | 6.47E-07 | 23.73439 |
| Gut microbiota abundance (phylum Bacteroidetes id.905) | rs2032750 | 2 | 53831026 | C | 0.0510857 | 0.0106804 | 1.71E-06 | 22.8783 |
| Gut microbiota abundance (phylum Bacteroidetes id.905) | rs62575403 | 9 | 136493820 | C | 0.145424 | 0.0311079 | 2.96E-06 | 21.85399 |
| Gut microbiota abundance (phylum Bacteroidetes id.905) | rs73512608 | 13 | 70522683 | G | -0.123117 | 0.0236743 | 2.54E-07 | 27.04467 |
| Gut microbiota abundance (phylum Bacteroidetes id.905) | rs73846128 | 3 | 89340254 | A | -0.0664262 | 0.0133481 | 4.78E-07 | 24.76509 |
| Gut microbiota abundance (phylum Bacteroidetes id.905) | rs73975615 | 17 | 6461200 | G | -0.207163 | 0.0442629 | 1.20E-06 | 21.90507 |
| Gut microbiota abundance (phylum Cyanobacteria id.1500) | rs584122 | 6 | 48288456 | T | 0.151774 | 0.0326827 | 4.23E-06 | 21.56547 |
| Gut microbiota abundance (phylum Cyanobacteria id.1500) | rs76531781 | 7 | 21647735 | T | -0.231868 | 0.0493393 | 2.87E-06 | 22.08491 |
| Gut microbiota abundance (phylum Cyanobacteria id.1500) | rs9864379 | 3 | 14306949 | T | -0.138933 | 0.0268045 | 2.03E-07 | 26.86553 |
| Gut microbiota abundance (phylum Euryarchaeota id.55) | rs10202904 | 2 | 125440268 | G | 0.115986 | 0.0230271 | 6.19E-07 | 25.37071 |
| Gut microbiota abundance (phylum Euryarchaeota id.55) | rs34928225 | 6 | 146840876 | T | 0.19977 | 0.0425421 | 4.33E-06 | 22.05072 |
| Gut microbiota abundance (phylum Euryarchaeota id.55) | rs76029318 | 13 | 41963791 | T | 0.214866 | 0.0438442 | 1.05E-06 | 24.01658 |
| Gut microbiota abundance (phylum Euryarchaeota id.55) | rs7635189 | 3 | 15591607 | A | -0.119983 | 0.0259356 | 4.64E-06 | 21.40163 |
| Gut microbiota abundance (phylum Euryarchaeota id.55) | rs77658038 | 6 | 16284785 | A | -0.160157 | 0.0340856 | 4.75E-06 | 22.0775 |
| Gut microbiota abundance (phylum Firmicutes id.1672) | rs112334273 | 21 | 40703251 | G | 0.0627459 | 0.0127415 | 9.26E-07 | 24.251 |
| Gut microbiota abundance (phylum Firmicutes id.1672) | rs2009919 | 17 | 11780026 | C | -0.0536457 | 0.0106294 | 4.95E-07 | 25.47137 |
| Gut microbiota abundance (phylum Firmicutes id.1672) | rs2332027 | 4 | 171672038 | A | 0.0482656 | 0.0104823 | 4.05E-06 | 21.20129 |
| Gut microbiota abundance (phylum Firmicutes id.1672) | rs3792064 | 2 | 231678413 | G | 0.0897349 | 0.0182532 | 6.75E-07 | 24.16822 |
| Gut microbiota abundance (phylum Firmicutes id.1672) | rs3852931 | 20 | 54087963 | T | 0.0482086 | 0.0105 | 4.53E-06 | 21.07999 |
| Gut microbiota abundance (phylum Firmicutes id.1672) | rs7247191 | 19 | 22899585 | T | -0.0713033 | 0.0157424 | 4.73E-06 | 20.51528 |
| Gut microbiota abundance (phylum Lentisphaerae id.2238) | rs1002941 | 15 | 101242690 | A | -0.107661 | 0.0233382 | 4.31E-06 | 21.28051 |
| Gut microbiota abundance (phylum Lentisphaerae id.2238) | rs11770843 | 7 | 146795379 | C | 0.111896 | 0.0234776 | 1.14E-06 | 22.71547 |
| Gut microbiota abundance (phylum Lentisphaerae id.2238) | rs2825714 | 21 | 21023966 | A | -0.138226 | 0.0289096 | 1.50E-06 | 22.861 |
| Gut microbiota abundance (phylum Lentisphaerae id.2238) | rs62570196 | 9 | 111086170 | C | -0.217203 | 0.0439621 | 9.64E-07 | 24.41039 |
| Gut microbiota abundance (phylum Lentisphaerae id.2238) | rs77599476 | 20 | 61394262 | A | 0.229981 | 0.0479918 | 1.90E-06 | 22.96412 |
| Gut microbiota abundance (phylum Proteobacteria id.2375) | rs12150865 | 19 | 43505320 | C | 0.0511615 | 0.0106407 | 1.54E-06 | 23.11778 |
| Gut microbiota abundance (phylum Proteobacteria id.2375) | rs2347697 | 7 | 134388240 | G | 0.0502281 | 0.0108968 | 4.27E-06 | 21.2469 |
| Gut microbiota abundance (phylum Proteobacteria id.2375) | rs2532663 | 10 | 119247234 | A | 0.125578 | 0.0258034 | 7.47E-07 | 23.68499 |
| Gut microbiota abundance (phylum Proteobacteria id.2375) | rs922773 | 3 | 66318042 | C | -0.0803778 | 0.0157851 | 3.68E-07 | 25.92851 |
| Gut microbiota abundance (phylum Tenericutes id.3919) | rs10108398 | 8 | 59440824 | G | 0.0769142 | 0.0153953 | 1.09E-06 | 24.95955 |
| Gut microbiota abundance (phylum Tenericutes id.3919) | rs11890098 | 2 | 157532549 | A | 0.074438 | 0.0153389 | 9.57E-07 | 23.55054 |
| Gut microbiota abundance (phylum Tenericutes id.3919) | rs12566890 | 1 | 61850864 | T | -0.101147 | 0.0230978 | 3.65E-06 | 19.1763 |
| Gut microbiota abundance (phylum Tenericutes id.3919) | rs3768491 | 1 | 109965986 | G | 0.0681052 | 0.0149061 | 4.23E-06 | 20.87529 |
| Gut microbiota abundance (phylum Tenericutes id.3919) | rs6043847 | 20 | 16259524 | T | -0.114937 | 0.0248606 | 4.55E-06 | 21.37453 |
| Gut microbiota abundance (phylum Tenericutes id.3919) | rs72901605 | 11 | 47103877 | T | -0.0841852 | 0.0178119 | 3.26E-06 | 22.33835 |
| Gut microbiota abundance (phylum Tenericutes id.3919) | rs74603314 | 14 | 46519718 | T | 0.221639 | 0.0462918 | 1.56E-06 | 22.92367 |
| Gut microbiota abundance (phylum Verrucomicrobia id.3982) | rs11252894 | 10 | 5073050 | A | 0.0784055 | 0.0162769 | 1.11E-06 | 23.2033 |
| Gut microbiota abundance (phylum Verrucomicrobia id.3982) | rs117107102 | 18 | 49473635 | A | 0.204283 | 0.0427565 | 2.68E-06 | 22.8276 |
| Gut microbiota abundance (phylum Verrucomicrobia id.3982) | rs11729256 | 4 | 95027272 | T | 0.0696984 | 0.0146953 | 2.23E-06 | 22.49514 |
| Gut microbiota abundance (phylum Verrucomicrobia id.3982) | rs12908520 | 15 | 97570657 | G | 0.0594916 | 0.012823 | 3.40E-06 | 21.52445 |
| Gut microbiota abundance (phylum Verrucomicrobia id.3982) | rs2602429 | 16 | 81063149 | T | -0.0764148 | 0.0153487 | 8.71E-07 | 24.7863 |
| Gut microbiota abundance (phylum Verrucomicrobia id.3982) | rs45598138 | 1 | 55522083 | C | -0.143901 | 0.03054 | 2.19E-06 | 22.20187 |
| Gut microbiota abundance (phylum Verrucomicrobia id.3982) | rs74542928 | 4 | 100544188 | T | 0.115994 | 0.0231324 | 4.08E-07 | 25.14373 |
| Gut microbiota abundance (phylum Verrucomicrobia id.3982) | rs76430504 | 5 | 40438158 | T | -0.11753 | 0.025458 | 3.50E-06 | 21.31321 |
| Gut microbiota abundance (phylum Verrucomicrobia id.3982) | rs117107102 | 18 | 49473635 | A | 0.204283 | 0.0427565 | 2.68E-06 | 22.8276 |
| Gut microbiota abundance (phylum Verrucomicrobia id.3982) | rs11729256 | 4 | 95027272 | T | 0.0696984 | 0.0146953 | 2.23E-06 | 22.49514 |
| Gut microbiota abundance (phylum Verrucomicrobia id.3982) | rs12908520 | 15 | 97570657 | G | 0.0594916 | 0.012823 | 3.40E-06 | 21.52445 |
| Gut microbiota abundance (phylum Verrucomicrobia id.3982) | rs2602429 | 16 | 81063149 | T | -0.0764148 | 0.0153487 | 8.71E-07 | 24.7863 |
| Gut microbiota abundance (phylum Verrucomicrobia id.3982) | rs45598138 | 1 | 55522083 | C | -0.143901 | 0.03054 | 2.19E-06 | 22.20187 |
| Gut microbiota abundance (phylum Verrucomicrobia id.3982) | rs74542928 | 4 | 100544188 | T | 0.115994 | 0.0231324 | 4.08E-07 | 25.14373 |
| Gut microbiota abundance (phylum Verrucomicrobia id.3982) | rs76430504 | 5 | 40438158 | T | -0.11753 | 0.025458 | 3.50E-06 | 21.31321 |
