# Supplementary material for: Causal Mediation Role of Immune Cells in Gut Microbiota–Pneumonia Associations: A Mendelian Randomisation Study
Source: J Cell Mol Med. 2025 Sep 11;29(17):e70839. doi: 10.1111/jcmm.70839 (PMC12425809; doi:10.1111/jcmm.70839)
Supplement: Supplementary file 8 — Table S2: Information about instrumental variables corresponding to immune cell phenotypes. [file JCMM-29-e70839-s012.docx]

Information about instrumental variables corresponding to immune cell phenotypes

| **id.exposure** | **SNPs** | **chr** | **pos** | **EA** | **EAF** | **β** | **SE** | **P** |
| --- | --- | --- | --- | --- | --- | --- | --- | --- |
| ebi-a-GCST90001574 | rs10003087 | 4 | 172937884 | G | 0.3764 | 0.127 | 0.02658 | 1.84E-06 |
| ebi-a-GCST90001572 | rs10003087 | 4 | 172937884 | G | 0.3766 | 0.1291 | 0.0263 | 9.59E-07 |
| ebi-a-GCST90001933 | rs1000411 | 20 | 39634616 | T | 0.2729 | 0.1341 | 0.02931 | 4.96E-06 |
| ebi-a-GCST90001871 | rs10008912 | 4 | 7148069 | G | 0.5754 | 0.1962 | 0.04231 | 3.92E-06 |
| ebi-a-GCST90001525 | rs10011663 | 4 | 190338080 | G | 0.2411 | 0.1837 | 0.03783 | 1.30E-06 |
| ebi-a-GCST90001535 | rs10013469 | 4 | 185924276 | A | 0.0943 | 0.192 | 0.04023 | 1.89E-06 |
| ebi-a-GCST90001525 | rs10025005 | 4 | 181288587 | T | 0.7764 | 0.1934 | 0.03857 | 5.86E-07 |
| ebi-a-GCST90001689 | rs10025910 | 4 | 25610903 | T | 0.0074 | 0.5074 | 0.1043 | 1.21E-06 |
| ebi-a-GCST90001660 | rs1002938 | 10 | 99461284 | T | 0.0776 | -0.275 | 0.04897 | 2.12E-08 |
| ebi-a-GCST90001949 | rs10033650 | 4 | 53954014 | T | 0.1083 | 0.2734 | 0.05678 | 1.60E-06 |
| ebi-a-GCST90001990 | rs10036919 | 5 | 160219464 | A | 0.3392 | -0.129 | 0.02659 | 1.28E-06 |
| ebi-a-GCST90001590 | rs10040274 | 5 | 114822737 | A | 0.2685 | 0.132 | 0.02702 | 1.06E-06 |
| ebi-a-GCST90001959 | rs10047088 | 1 | 228004140 | C | 0.8832 | 0.1915 | 0.04113 | 3.38E-06 |
| ebi-a-GCST90001760 | rs10047347 | 10 | 26741516 | T | 0.4197 | 0.117 | 0.02555 | 4.78E-06 |
| ebi-a-GCST90001795 | rs10047878 | 14 | 22787690 | A | 0.56 | -0.1156 | 0.02506 | 4.12E-06 |
| ebi-a-GCST90001625 | rs10052603 | 5 | 10837029 | G | 0.5772 | -0.1031 | 0.02216 | 3.39E-06 |
| ebi-a-GCST90001666 | rs1005513 | 17 | 49196918 | C | 0.2531 | 0.12 | 0.02541 | 2.45E-06 |
| ebi-a-GCST90001768 | rs10056811 | 5 | 74605220 | A | 0.3022 | -0.1322 | 0.02752 | 1.62E-06 |
| ebi-a-GCST90001481 | rs10059100 | 5 | 120120689 | G | 0.3224 | -0.1194 | 0.02467 | 1.36E-06 |
| ebi-a-GCST90001493 | rs10059100 | 5 | 120120689 | G | 0.3224 | 0.1188 | 0.02585 | 4.48E-06 |
| ebi-a-GCST90001502 | rs10059100 | 5 | 120120689 | G | 0.3224 | 0.1214 | 0.02435 | 6.51E-07 |
| ebi-a-GCST90002084 | rs1006368 | 10 | 126346603 | T | 0.1224 | 0.2024 | 0.04066 | 6.82E-07 |
| ebi-a-GCST90001969 | rs1006368 | 10 | 126346603 | T | 0.1224 | 0.389 | 0.04003 | 5.08E-22 |
| ebi-a-GCST90002076 | rs1006368 | 10 | 126346603 | T | 0.1224 | 0.4144 | 0.04052 | 3.61E-24 |
| ebi-a-GCST90001695 | rs1007033 | 17 | 31695774 | T | 0.0396 | 37.39 | 6.868 | 5.59E-08 |
| ebi-a-GCST90001983 | rs10071982 | 5 | 62703251 | G | 0.512 | -0.1153 | 0.02494 | 3.92E-06 |
| ebi-a-GCST90001752 | rs1007337 | 22 | 39781585 | T | 0.6024 | -0.1389 | 0.02509 | 3.34E-08 |
| ebi-a-GCST90001747 | rs1007337 | 22 | 39781585 | T | 0.6024 | -0.1254 | 0.02513 | 6.28E-07 |
| ebi-a-GCST90001816 | rs10073475 | 5 | 77504427 | C | 0.5953 | -0.1209 | 0.02551 | 2.22E-06 |
| ebi-a-GCST90001820 | rs10076053 | 5 | 150649861 | A | 0.0092 | 0.6192 | 0.1283 | 1.44E-06 |
| ebi-a-GCST90001766 | rs1007713 | 5 | 79150995 | A | 0.1009 | 0.1897 | 0.04144 | 4.85E-06 |
| ebi-a-GCST90001514 | rs10080768 | 6 | 2760245 | T | 0.7374 | 0.1703 | 0.03596 | 2.34E-06 |
| ebi-a-GCST90002004 | rs10081735 | 9 | 105486830 | A | 0.0926 | 0.2007 | 0.04193 | 1.76E-06 |
| ebi-a-GCST90001483 | rs10086961 | 8 | 132897157 | C | 0.2797 | -0.1276 | 0.02767 | 4.10E-06 |
| ebi-a-GCST90001485 | rs10086961 | 8 | 132897157 | C | 0.28 | -0.1275 | 0.02736 | 3.30E-06 |
| ebi-a-GCST90001881 | rs10090960 | 8 | 136809038 | G | 0.4499 | -0.1987 | 0.04027 | 9.11E-07 |
| ebi-a-GCST90001872 | rs10090960 | 8 | 136809038 | G | 0.4499 | -0.1865 | 0.04018 | 3.82E-06 |
| ebi-a-GCST90001582 | rs10094039 | 8 | 130589676 | G | 0.7531 | -0.137 | 0.02784 | 9.09E-07 |
| ebi-a-GCST90001429 | rs10103048 | 8 | 130602281 | C | 0.7321 | 0.126 | 0.02692 | 2.99E-06 |
| ebi-a-GCST90001921 | rs10105402 | 8 | 131518417 | T | 0.2067 | 0.1601 | 0.03244 | 8.45E-07 |
| ebi-a-GCST90001663 | rs10107063 | 8 | 123562235 | C | 0.0091 | -0.6397 | 0.1366 | 2.94E-06 |
| ebi-a-GCST90001520 | rs10112702 | 8 | 127626066 | G | 0.2034 | -0.2209 | 0.04185 | 1.46E-07 |
| ebi-a-GCST90002099 | rs10114587 | 9 | 133795282 | G | 0.0462 | -0.2921 | 0.06387 | 5.00E-06 |
| ebi-a-GCST90002027 | rs10115790 | 9 | 7345304 | A | 0.2312 | -0.1469 | 0.03202 | 4.68E-06 |
| ebi-a-GCST90001647 | rs10118740 | 9 | 122199843 | A | 0.0412 | -0.2745 | 0.05864 | 2.96E-06 |
| ebi-a-GCST90001834 | rs10127711 | 1 | 168483806 | G | 0.3608 | 0.1336 | 0.02899 | 4.27E-06 |
| ebi-a-GCST90001858 | rs10139012 | 14 | 95415204 | C | 0.3088 | -0.1401 | 0.02935 | 1.89E-06 |
| ebi-a-GCST90001624 | rs10148838 | 14 | 20261108 | C | 0.227 | -0.2544 | 0.05136 | 7.63E-07 |
| ebi-a-GCST90001673 | rs1015011 | 22 | 24893021 | G | 0.1608 | 0.162 | 0.03413 | 2.15E-06 |
| ebi-a-GCST90001673 | rs1015011 | 22 | 24893021 | G | 0.1608 | 0.162 | 0.03413 | 2.15E-06 |
| ebi-a-GCST90001698 | rs10155782 | 6 | 37156467 | C | 0.012 | 87.03 | 16.56 | 1.58E-07 |
| ebi-a-GCST90001578 | rs10158474 | 1 | 96015938 | G | 0.602 | 0.1139 | 0.02487 | 4.77E-06 |
| ebi-a-GCST90001917 | rs1015952 | 12 | 77741488 | T | 0.6394 | 0.1213 | 0.02581 | 2.74E-06 |
| ebi-a-GCST90001698 | rs10159645 | 10 | 106333320 | A | 0.0761 | 31.12 | 6.789 | 4.74E-06 |
| ebi-a-GCST90001946 | rs10164761 | 2 | 220066209 | T | 0.6307 | 0.1848 | 0.03745 | 8.84E-07 |
| ebi-a-GCST90001952 | rs10164761 | 2 | 220066209 | T | 0.6348 | 0.1855 | 0.0387 | 1.81E-06 |
| ebi-a-GCST90001971 | rs10169473 | 2 | 224307687 | A | 0.3675 | 0.1191 | 0.02492 | 1.86E-06 |
| ebi-a-GCST90001972 | rs10169473 | 2 | 224307687 | A | 0.3675 | 0.1197 | 0.02532 | 2.37E-06 |
| ebi-a-GCST90001973 | rs10169473 | 2 | 224307687 | A | 0.3675 | 0.1225 | 0.02574 | 2.04E-06 |
| ebi-a-GCST90002081 | rs10169473 | 2 | 224307687 | A | 0.3675 | 0.1231 | 0.02635 | 3.13E-06 |
| ebi-a-GCST90001467 | rs10170363 | 2 | 15272805 | T | 0.2944 | 0.1318 | 0.02687 | 9.80E-07 |
| ebi-a-GCST90002086 | rs1017102 | 19 | 56892632 | C | 0.1376 | 0.1765 | 0.03832 | 4.28E-06 |
| ebi-a-GCST90001682 | rs10171072 | 2 | 113756563 | A | 0.1914 | 0.1532 | 0.03199 | 1.75E-06 |
| ebi-a-GCST90001753 | rs10171238 | 2 | 149568729 | T | 0.7912 | 0.1442 | 0.0302 | 1.86E-06 |
| ebi-a-GCST90001653 | rs10174322 | 2 | 7659940 | G | 0.2719 | -0.1507 | 0.028 | 7.75E-08 |
| ebi-a-GCST90001656 | rs10174322 | 2 | 7659940 | G | 0.2719 | 0.1507 | 0.028 | 7.75E-08 |
| ebi-a-GCST90001530 | rs10176753 | 2 | 133046981 | G | 0.4941 | -0.1561 | 0.03371 | 3.91E-06 |
| ebi-a-GCST90001700 | rs10176830 | 2 | 83631928 | C | 3.00E-04 | 29.23 | 4.452 | 5.97E-11 |
| ebi-a-GCST90001699 | rs10176830 | 2 | 83631928 | C | 3.00E-04 | 49.13 | 10.45 | 2.68E-06 |
| ebi-a-GCST90002118 | rs10181413 | 2 | 80959711 | A | 0.6212 | 0.145 | 0.02796 | 2.28E-07 |
| ebi-a-GCST90001688 | rs10183338 | 2 | 111610816 | T | 0.1891 | 0.1132 | 0.02243 | 4.71E-07 |
| ebi-a-GCST90001553 | rs10183338 | 2 | 111610816 | T | 0.1892 | 0.1206 | 0.02163 | 2.66E-08 |
| ebi-a-GCST90001975 | rs10183967 | 2 | 71797176 | C | 0.5783 | -0.1412 | 0.02636 | 9.18E-08 |
| ebi-a-GCST90001659 | rs10184974 | 2 | 5560128 | T | 0.2904 | -0.1215 | 0.0264 | 4.32E-06 |
| ebi-a-GCST90002072 | rs10193587 | 2 | 136925439 | C | 0.7214 | 0.1493 | 0.03074 | 1.27E-06 |
| ebi-a-GCST90002119 | rs10205992 | 2 | 87279109 | T | 0.4729 | -0.2955 | 0.02653 | 3.00E-28 |
| ebi-a-GCST90001571 | rs10225877 | 7 | 55150822 | T | 0.2946 | -0.1278 | 0.02656 | 1.55E-06 |
| ebi-a-GCST90001391 | rs10226722 | 7 | 68025695 | C | 0.6006 | 0.1184 | 0.0256 | 3.84E-06 |
| ebi-a-GCST90002039 | rs10230431 | 7 | 116586829 | G | 0.1065 | -0.2081 | 0.04416 | 2.56E-06 |
| ebi-a-GCST90001819 | rs10231147 | 7 | 144513549 | T | 0.7066 | 0.1346 | 0.02699 | 6.42E-07 |
| ebi-a-GCST90002040 | rs1023388 | 21 | 19717241 | C | 0.6826 | -0.1484 | 0.02993 | 7.47E-07 |
| ebi-a-GCST90001897 | rs10237725 | 7 | 30558616 | T | 0.6208 | 0.1318 | 0.02811 | 2.87E-06 |
| ebi-a-GCST90001689 | rs10242630 | 7 | 146469943 | A | 0.0541 | 0.1978 | 0.04107 | 1.53E-06 |
| ebi-a-GCST90001620 | rs10244964 | 7 | 46432552 | T | 0.0013 | -1.445 | 0.3106 | 3.43E-06 |
| ebi-a-GCST90001619 | rs10244964 | 7 | 46432552 | T | 0.0013 | -1.414 | 0.3025 | 3.08E-06 |
| ebi-a-GCST90001395 | rs10254361 | 7 | 119564205 | T | 0.2404 | 0.1333 | 0.02899 | 4.40E-06 |
| ebi-a-GCST90001410 | rs10270812 | 7 | 68026886 | C | 0.5103 | -0.118 | 0.0244 | 1.39E-06 |
| ebi-a-GCST90001700 | rs10274897 | 7 | 45344881 | G | 3.00E-04 | 19.66 | 4.1 | 1.70E-06 |
| ebi-a-GCST90001698 | rs10276305 | 7 | 7885705 | A | 0.0104 | 85.37 | 17.66 | 1.40E-06 |
| ebi-a-GCST90001631 | rs10276441 | 7 | 52581385 | A | 0.8246 | 0.1529 | 0.03242 | 2.49E-06 |
| ebi-a-GCST90002002 | rs10276990 | 7 | 16903052 | A | 0.0971 | 0.198 | 0.04119 | 1.59E-06 |
| ebi-a-GCST90001865 | rs10280171 | 7 | 155971101 | C | 0.013 | -0.5408 | 0.1154 | 2.90E-06 |
| ebi-a-GCST90001641 | rs10282551 | 7 | 135898321 | G | 0.0775 | 0.2289 | 0.04732 | 1.37E-06 |
| ebi-a-GCST90001824 | rs1037633 | 3 | 23750521 | G | 0.124 | 0.2035 | 0.03838 | 1.21E-07 |
| ebi-a-GCST90001828 | rs1037633 | 3 | 23750521 | G | 0.124 | 0.234 | 0.03837 | 1.19E-09 |
| ebi-a-GCST90001698 | rs10400782 | 14 | 35130401 | C | 0.0019 | 280.3 | 39.64 | 1.85E-12 |
| ebi-a-GCST90001787 | rs10401190 | 19 | 17987864 | T | 0.0062 | -0.8085 | 0.1646 | 9.45E-07 |
| ebi-a-GCST90001786 | rs10401190 | 19 | 17987864 | T | 0.0062 | -0.7709 | 0.1645 | 2.88E-06 |
| ebi-a-GCST90001883 | rs10401901 | 19 | 54956254 | C | 0.4332 | 0.1276 | 0.02659 | 1.69E-06 |
| ebi-a-GCST90001918 | rs10411493 | 19 | 30617388 | G | 0.0335 | 0.3586 | 0.07583 | 2.35E-06 |
| ebi-a-GCST90001678 | rs10413329 | 19 | 13114434 | G | 0.5032 | 0.1164 | 0.02398 | 1.28E-06 |
| ebi-a-GCST90001986 | rs1041345 | 12 | 41197785 | A | 0.5941 | -0.116 | 0.02484 | 3.14E-06 |
| ebi-a-GCST90001529 | rs10419309 | 19 | 52141162 | G | 0.5 | 0.1792 | 0.03351 | 1.01E-07 |
| ebi-a-GCST90001836 | rs10420273 | 19 | 15177126 | C | 0.9539 | -0.4197 | 0.08946 | 2.97E-06 |
| ebi-a-GCST90001814 | rs10421176 | 19 | 13425338 | A | 0.3279 | -0.1269 | 0.02621 | 1.35E-06 |
| ebi-a-GCST90002029 | rs10422831 | 19 | 2019930 | G | 0.1201 | -0.1983 | 0.04135 | 1.70E-06 |
| ebi-a-GCST90002084 | rs10422871 | 19 | 8789227 | T | 0.0888 | -0.2765 | 0.04672 | 3.60E-09 |
| ebi-a-GCST90001722 | rs10426452 | 19 | 49907690 | A | 0.3815 | -0.1391 | 0.02491 | 2.51E-08 |
| ebi-a-GCST90001744 | rs10426452 | 19 | 49907690 | A | 0.3816 | -0.115 | 0.02506 | 4.64E-06 |
| ebi-a-GCST90001537 | rs1043118 | 20 | 56223607 | A | 0.0376 | 0.3231 | 0.06637 | 1.18E-06 |
| ebi-a-GCST90001535 | rs10432663 | 2 | 41958972 | C | 0.1959 | -0.1402 | 0.03003 | 3.14E-06 |
| ebi-a-GCST90001395 | rs10444793 | 15 | 44517689 | A | 0.018 | -0.4608 | 0.09102 | 4.35E-07 |
| ebi-a-GCST90001431 | rs10444793 | 15 | 44517689 | A | 0.0181 | -0.4323 | 0.09225 | 2.89E-06 |
| ebi-a-GCST90002007 | rs1044722 | 4 | 164457232 | A | 0.3994 | -0.117 | 0.02533 | 3.98E-06 |
| ebi-a-GCST90001667 | rs10456250 | 6 | 22061048 | C | 0.5653 | -0.1123 | 0.02403 | 3.10E-06 |
| ebi-a-GCST90001530 | rs10458377 | 1 | 226154277 | G | 0.8423 | -0.2164 | 0.04679 | 3.99E-06 |
| ebi-a-GCST90002075 | rs10459209 | 12 | 106623009 | A | 0.1721 | 0.1686 | 0.0362 | 3.32E-06 |
| ebi-a-GCST90001553 | rs10474365 | 5 | 90986313 | T | 0.091 | 0.1374 | 0.02986 | 4.35E-06 |
| ebi-a-GCST90001529 | rs1047483 | 5 | 52249612 | T | 0.5579 | -0.1528 | 0.03328 | 4.73E-06 |
| ebi-a-GCST90001698 | rs10484068 | 14 | 99653123 | T | 0.0103 | 118.5 | 17.59 | 1.86E-11 |
| ebi-a-GCST90002009 | rs10485346 | 6 | 33866059 | A | 0.5882 | -0.1208 | 0.02608 | 3.73E-06 |
| ebi-a-GCST90002074 | rs10488833 | 4 | 97953756 | G | 0.1043 | -0.2117 | 0.04376 | 1.39E-06 |
| ebi-a-GCST90001595 | rs10490700 | 2 | 106615183 | G | 0.241 | 0.1298 | 0.02828 | 4.57E-06 |
| ebi-a-GCST90001774 | rs10491456 | 5 | 79152076 | A | 0.1011 | 0.1886 | 0.04063 | 3.58E-06 |
| ebi-a-GCST90001965 | rs10492689 | 13 | 39544533 | A | 7.00E-04 | 2.884 | 0.6047 | 1.94E-06 |
| ebi-a-GCST90001963 | rs10492689 | 13 | 39544533 | A | 7.00E-04 | 3.02 | 0.6138 | 9.09E-07 |
| ebi-a-GCST90001700 | rs10492710 | 13 | 98908125 | A | 0.0036 | 5.636 | 1.205 | 3.02E-06 |
| ebi-a-GCST90001859 | rs10494441 | 1 | 165250607 | T | 0.037 | -0.372 | 0.07117 | 1.85E-07 |
| ebi-a-GCST90001728 | rs10494895 | 1 | 208466901 | G | 0.0314 | 0.4571 | 0.09801 | 3.33E-06 |
| ebi-a-GCST90001761 | rs10501383 | 11 | 60164527 | T | 0.0971 | -0.2991 | 0.04099 | 3.61E-13 |
| ebi-a-GCST90001755 | rs10501383 | 11 | 60164527 | T | 0.0971 | -0.255 | 0.04098 | 5.41E-10 |
| ebi-a-GCST90001700 | rs10505739 | 12 | 10043518 | C | 4.00E-04 | 18.81 | 3.393 | 3.21E-08 |
| ebi-a-GCST90001698 | rs10505739 | 12 | 10043518 | C | 4.00E-04 | 985.5 | 78.14 | 1.12E-35 |
| ebi-a-GCST90001488 | rs10511150 | 3 | 94952474 | A | 0.2376 | 0.1452 | 0.03006 | 1.43E-06 |
| ebi-a-GCST90002110 | rs10511609 | 9 | 15335996 | G | 0.0236 | -0.6195 | 0.1223 | 4.57E-07 |
| ebi-a-GCST90001604 | rs10512469 | 17 | 33770802 | T | 0.1639 | -0.2345 | 0.03229 | 4.61E-13 |
| ebi-a-GCST90002076 | rs10512469 | 17 | 33770802 | T | 0.1646 | -0.1684 | 0.03658 | 4.31E-06 |
| ebi-a-GCST90001647 | rs10512469 | 17 | 33770802 | T | 0.1639 | 0.249 | 0.03122 | 1.98E-15 |
| ebi-a-GCST90001536 | rs1051488 | 6 | 31322980 | T | 0.7693 | 0.1684 | 0.03331 | 4.55E-07 |
| ebi-a-GCST90001692 | rs1051488 | 6 | 31322980 | T | 0.7693 | 0.2395 | 0.03338 | 8.82E-13 |
| ebi-a-GCST90001959 | rs10517576 | 4 | 154561623 | C | 0.0109 | 0.578 | 0.1243 | 3.46E-06 |
| ebi-a-GCST90001642 | rs1057686 | 12 | 120533696 | G | 0.1291 | -0.1735 | 0.0364 | 1.96E-06 |
| ebi-a-GCST90001552 | rs1064086 | 2 | 237034061 | A | 0.328 | -0.1062 | 0.01937 | 4.56E-08 |
| ebi-a-GCST90001689 | rs1064086 | 2 | 237034061 | A | 0.3275 | -0.103 | 0.01985 | 2.23E-07 |
| ebi-a-GCST90001553 | rs1064086 | 2 | 237034061 | A | 0.328 | -0.08993 | 0.01765 | 3.67E-07 |
| ebi-a-GCST90002017 | rs1071849 | 1 | 26646726 | G | 0.7986 | -0.1837 | 0.03406 | 7.50E-08 |
| ebi-a-GCST90002036 | rs10733750 | 9 | 96630555 | C | 0.4342 | 0.1359 | 0.02789 | 1.17E-06 |
| ebi-a-GCST90002046 | rs10735302 | 12 | 93068616 | T | 0.5199 | -0.1815 | 0.03652 | 7.42E-07 |
| ebi-a-GCST90001474 | rs10735429 | 12 | 108728931 | G | 0.7411 | 0.1338 | 0.02924 | 4.92E-06 |
| ebi-a-GCST90001729 | rs10737202 | 1 | 234499381 | C | 0.3389 | -0.1243 | 0.02655 | 2.93E-06 |
| ebi-a-GCST90001813 | rs10737766 | 1 | 116770543 | T | 0.1966 | -0.146 | 0.03105 | 2.68E-06 |
| ebi-a-GCST90001573 | rs10738134 | 8 | 121168145 | C | 0.6129 | -0.1225 | 0.02648 | 3.84E-06 |
| ebi-a-GCST90001976 | rs10739941 | 9 | 95767562 | G | 0.8252 | 0.158 | 0.03392 | 3.31E-06 |
| ebi-a-GCST90001797 | rs10740344 | 10 | 72155840 | T | 0.7394 | -0.1301 | 0.02703 | 1.53E-06 |
| ebi-a-GCST90001417 | rs10743163 | 11 | 11033950 | A | 0.4035 | -0.1205 | 0.02541 | 2.21E-06 |
| ebi-a-GCST90001428 | rs10743163 | 11 | 11033950 | A | 0.4035 | -0.1167 | 0.02529 | 4.11E-06 |
| ebi-a-GCST90001809 | rs10743387 | 12 | 20807481 | A | 0.6382 | -0.1237 | 0.02526 | 1.02E-06 |
| ebi-a-GCST90002042 | rs10744547 | 12 | 1779545 | C | 0.6156 | 0.1866 | 0.03776 | 8.61E-07 |
| ebi-a-GCST90001888 | rs10748649 | 10 | 97601703 | C | 0.4733 | 0.4156 | 0.02562 | 9.56E-57 |
| ebi-a-GCST90002084 | rs10749775 | 1 | 67822087 | A | 0.8657 | 0.1882 | 0.03934 | 1.80E-06 |
| ebi-a-GCST90001547 | rs10750395 | 11 | 99630378 | A | 0.3742 | -0.1274 | 0.02551 | 6.19E-07 |
| ebi-a-GCST90001546 | rs10750395 | 11 | 99630378 | A | 0.3742 | -0.1252 | 0.02568 | 1.14E-06 |
| ebi-a-GCST90001773 | rs10750479 | 11 | 131277609 | C | 0.204 | -0.147 | 0.03065 | 1.70E-06 |
| ebi-a-GCST90001622 | rs10750486 | 11 | 131463916 | A | 0.6732 | 0.1233 | 0.0268 | 4.36E-06 |
| ebi-a-GCST90001621 | rs10750486 | 11 | 131463916 | A | 0.6731 | 0.1234 | 0.02678 | 4.19E-06 |
| ebi-a-GCST90001734 | rs10752607 | 1 | 154716803 | A | 0.7828 | 0.1478 | 0.02969 | 6.76E-07 |
| ebi-a-GCST90001727 | rs10754999 | 2 | 184906937 | C | 0.7503 | -0.181 | 0.03915 | 4.03E-06 |
| ebi-a-GCST90002090 | rs10756625 | 9 | 1501109 | G | 0.4288 | -0.1367 | 0.02892 | 2.36E-06 |
| ebi-a-GCST90001654 | rs10757255 | 9 | 21776631 | A | 0.467 | -0.1252 | 0.02547 | 9.26E-07 |
| ebi-a-GCST90001695 | rs10759064 | 9 | 9405003 | G | 0.5346 | -13.2 | 2.596 | 3.84E-07 |
| ebi-a-GCST90002016 | rs10759524 | 9 | 114834318 | A | 0.4289 | 0.1338 | 0.02821 | 2.20E-06 |
| ebi-a-GCST90002015 | rs10759524 | 9 | 114834318 | A | 0.4289 | 0.1349 | 0.02826 | 1.91E-06 |
| ebi-a-GCST90001950 | rs10759715 | 9 | 117277295 | T | 0.8943 | -0.3443 | 0.0674 | 3.77E-07 |
| ebi-a-GCST90001733 | rs10761194 | 9 | 95899884 | A | 0.4403 | -0.1148 | 0.02474 | 3.56E-06 |
| ebi-a-GCST90001418 | rs10762541 | 10 | 53790461 | T | 0.772 | 0.1488 | 0.02978 | 6.09E-07 |
| ebi-a-GCST90001658 | rs10762791 | 10 | 80309266 | G | 0.5113 | -0.1294 | 0.02464 | 1.60E-07 |
| ebi-a-GCST90001659 | rs10762791 | 10 | 80309266 | G | 0.5113 | -0.1212 | 0.02466 | 9.26E-07 |
| ebi-a-GCST90001783 | rs10765088 | 10 | 133336906 | A | 0.5103 | -0.1198 | 0.02428 | 8.34E-07 |
| ebi-a-GCST90002084 | rs10765790 | 11 | 95852734 | C | 0.5524 | 0.1554 | 0.02741 | 1.55E-08 |
| ebi-a-GCST90001623 | rs10765907 | 11 | 2306683 | C | 0.5677 | -0.1215 | 0.0253 | 1.63E-06 |
| ebi-a-GCST90001622 | rs10765907 | 11 | 2306683 | C | 0.5677 | -0.1174 | 0.02515 | 3.15E-06 |
| ebi-a-GCST90001621 | rs10765907 | 11 | 2306683 | C | 0.5672 | -0.1173 | 0.02511 | 3.07E-06 |
| ebi-a-GCST90001663 | rs10772894 | 12 | 16263287 | G | 0.0753 | -0.2226 | 0.04792 | 3.53E-06 |
| ebi-a-GCST90001807 | rs1077349 | 20 | 57072098 | A | 0.736 | 0.132 | 0.02777 | 2.07E-06 |
| ebi-a-GCST90002074 | rs10774278 | 12 | 629655 | T | 0.6157 | -0.1283 | 0.02705 | 2.22E-06 |
| ebi-a-GCST90001845 | rs1077544 | 3 | 158945280 | T | 0.7898 | -0.1562 | 0.03369 | 3.73E-06 |
| ebi-a-GCST90001769 | rs10777201 | 12 | 77884772 | C | 0.8052 | -0.1487 | 0.03162 | 2.65E-06 |
| ebi-a-GCST90001469 | rs10779896 | 2 | 4620195 | T | 0.0632 | -0.2427 | 0.05258 | 4.04E-06 |
| ebi-a-GCST90002073 | rs10784763 | 12 | 69524025 | G | 0.6168 | -0.1443 | 0.02802 | 2.78E-07 |
| ebi-a-GCST90002074 | rs10784763 | 12 | 69524025 | G | 0.6171 | -0.1296 | 0.02691 | 1.53E-06 |
| ebi-a-GCST90001511 | rs10785396 | 12 | 43312063 | A | 0.6852 | -0.1309 | 0.02734 | 1.75E-06 |
| ebi-a-GCST90001799 | rs10785556 | 12 | 45593682 | A | 0.0394 | 0.4051 | 0.08047 | 5.25E-07 |
| ebi-a-GCST90001495 | rs10786199 | 10 | 97069472 | A | 0.2894 | -0.1932 | 0.02906 | 3.39E-11 |
| ebi-a-GCST90001489 | rs10786199 | 10 | 97069472 | A | 0.2894 | -0.189 | 0.02875 | 5.63E-11 |
| ebi-a-GCST90001491 | rs10786199 | 10 | 97069472 | A | 0.2888 | -0.1864 | 0.02836 | 5.61E-11 |
| ebi-a-GCST90002061 | rs10786281 | 10 | 98119560 | C | 0.6527 | 0.1739 | 0.02896 | 2.18E-09 |
| ebi-a-GCST90001974 | rs10786450 | 10 | 100399366 | A | 0.0149 | 0.4489 | 0.0962 | 3.20E-06 |
| ebi-a-GCST90001619 | rs10788105 | 10 | 122390777 | A | 0.0601 | 0.2157 | 0.04716 | 4.95E-06 |
| ebi-a-GCST90001620 | rs10788105 | 10 | 122390777 | A | 0.0601 | 0.2367 | 0.04839 | 1.04E-06 |
| ebi-a-GCST90001679 | rs10789181 | 1 | 65816567 | G | 0.6221 | 0.127 | 0.02736 | 3.57E-06 |
| ebi-a-GCST90001680 | rs10789181 | 1 | 65816567 | G | 0.6221 | 0.1286 | 0.02678 | 1.66E-06 |
| ebi-a-GCST90001803 | rs10789188 | 1 | 66053791 | G | 0.7554 | 0.1343 | 0.02926 | 4.53E-06 |
| ebi-a-GCST90002057 | rs1079009 | 2 | 121367930 | G | 0.7503 | 0.1556 | 0.03268 | 2.03E-06 |
| ebi-a-GCST90001436 | rs10791220 | 11 | 132153516 | G | 0.1134 | -0.1793 | 0.03894 | 4.28E-06 |
| ebi-a-GCST90001851 | rs10801951 | 1 | 117781742 | T | 0.7143 | -0.1463 | 0.03028 | 1.43E-06 |
| ebi-a-GCST90001867 | rs10801951 | 1 | 117781742 | T | 0.7143 | -0.1455 | 0.03004 | 1.34E-06 |
| ebi-a-GCST90001861 | rs10801951 | 1 | 117781742 | T | 0.7143 | -0.1435 | 0.03024 | 2.19E-06 |
| ebi-a-GCST90001786 | rs10806538 | 6 | 67249077 | G | 0.6476 | -0.1196 | 0.02571 | 3.41E-06 |
| ebi-a-GCST90001514 | rs10809987 | 9 | 13585363 | C | 0.1082 | 0.2534 | 0.05071 | 6.39E-07 |
| ebi-a-GCST90001705 | rs1081003 | 22 | 42583910 | A | 0.0872 | -0.5412 | 0.04473 | 4.59E-33 |
| ebi-a-GCST90001829 | rs1081003 | 22 | 42583910 | A | 0.0874 | -0.5326 | 0.04476 | 4.65E-32 |
| ebi-a-GCST90001706 | rs1081003 | 22 | 42583910 | A | 0.0887 | -0.5202 | 0.05995 | 8.86E-18 |
| ebi-a-GCST90001719 | rs1081003 | 22 | 42583910 | A | 0.0875 | -0.5131 | 0.04473 | 5.92E-30 |
| ebi-a-GCST90001716 | rs1081003 | 22 | 42583910 | A | 0.0874 | -0.5029 | 0.04491 | 1.22E-28 |
| ebi-a-GCST90001709 | rs1081003 | 22 | 42583910 | A | 0.0874 | -0.5028 | 0.04481 | 9.61E-29 |
| ebi-a-GCST90001704 | rs1081003 | 22 | 42583910 | A | 0.0874 | -0.4975 | 0.0448 | 3.32E-28 |
| ebi-a-GCST90001708 | rs1081003 | 22 | 42583910 | A | 0.0874 | -0.4922 | 0.04486 | 1.40E-27 |
| ebi-a-GCST90001720 | rs1081003 | 22 | 42583910 | A | 0.0873 | -0.4717 | 0.0449 | 1.86E-25 |
| ebi-a-GCST90001420 | rs10815908 | 9 | 8537763 | T | 0.0511 | 0.2526 | 0.0538 | 2.76E-06 |
| ebi-a-GCST90001937 | rs10819198 | 9 | 129478793 | C | 0.2713 | -0.1378 | 0.02914 | 2.32E-06 |
| ebi-a-GCST90002069 | rs10819527 | 9 | 132242279 | C | 0.2104 | 0.158 | 0.03323 | 2.08E-06 |
| ebi-a-GCST90001732 | rs10821075 | 9 | 95896099 | A | 0.4393 | -0.1158 | 0.02466 | 2.73E-06 |
| ebi-a-GCST90001823 | rs10823811 | 10 | 73404650 | T | 0.7612 | 0.1865 | 0.04008 | 3.52E-06 |
| ebi-a-GCST90001921 | rs10823981 | 10 | 53792294 | T | 0.502 | -0.1255 | 0.02692 | 3.25E-06 |
| ebi-a-GCST90001700 | rs10824158 | 10 | 53915868 | T | 0.0019 | 9.768 | 1.725 | 1.62E-08 |
| ebi-a-GCST90001698 | rs10824158 | 10 | 53915868 | T | 0.0019 | 407.5 | 40.68 | 2.65E-23 |
| ebi-a-GCST90001429 | rs10832409 | 11 | 15377699 | A | 0.1722 | 0.1495 | 0.03227 | 3.74E-06 |
| ebi-a-GCST90001800 | rs10833056 | 11 | 19105332 | C | 0.3526 | 0.1222 | 0.02627 | 3.44E-06 |
| ebi-a-GCST90001579 | rs10833398 | 11 | 20860026 | C | 0.2123 | -0.14 | 0.02967 | 2.45E-06 |
| ebi-a-GCST90001568 | rs10836189 | 11 | 34366783 | A | 0.1029 | -0.1557 | 0.03377 | 4.17E-06 |
| ebi-a-GCST90001839 | rs10838150 | 11 | 5483246 | T | 0.3101 | 0.1451 | 0.03057 | 2.18E-06 |
| ebi-a-GCST90001482 | rs1084331 | 6 | 125213827 | G | 0.8586 | -0.1546 | 0.03375 | 4.82E-06 |
| ebi-a-GCST90001781 | rs10843928 | 12 | 31502991 | T | 0.5142 | -0.1556 | 0.03376 | 4.34E-06 |
| ebi-a-GCST90001618 | rs1084559 | 16 | 10829280 | A | 0.602 | -0.1472 | 0.02482 | 3.32E-09 |
| ebi-a-GCST90001627 | rs1084560 | 16 | 10824872 | G | 0.5721 | -0.1331 | 0.02498 | 1.06E-07 |
| ebi-a-GCST90001619 | rs1084560 | 16 | 10824872 | G | 0.5726 | -0.1328 | 0.02309 | 9.71E-09 |
| ebi-a-GCST90001897 | rs10847448 | 12 | 128258849 | C | 0.3938 | -0.1275 | 0.02786 | 4.90E-06 |
| ebi-a-GCST90001404 | rs10849436 | 12 | 6409619 | A | 0.1595 | 0.1576 | 0.0337 | 3.02E-06 |
| ebi-a-GCST90001616 | rs10850589 | 12 | 109499490 | T | 0.5139 | -0.1159 | 0.02479 | 3.07E-06 |
| ebi-a-GCST90001615 | rs10850589 | 12 | 109499490 | T | 0.5141 | -0.1129 | 0.02418 | 3.15E-06 |
| ebi-a-GCST90001594 | rs10851760 | 15 | 66871840 | G | 0.8111 | -0.1506 | 0.03184 | 2.34E-06 |
| ebi-a-GCST90001460 | rs10853636 | 18 | 51412543 | G | 0.4413 | 0.1138 | 0.02422 | 2.73E-06 |
| ebi-a-GCST90002039 | rs10857716 | 10 | 135256327 | T | 0.3672 | -0.1284 | 0.02799 | 4.67E-06 |
| ebi-a-GCST90001611 | rs10858526 | 12 | 87314863 | A | 0.5018 | -0.116 | 0.02467 | 2.66E-06 |
| ebi-a-GCST90001865 | rs10861453 | 12 | 105931980 | G | 0.4459 | -0.1298 | 0.0278 | 3.16E-06 |
| ebi-a-GCST90001550 | rs10865142 | 2 | 38892836 | C | 0.3097 | -0.1296 | 0.02672 | 1.28E-06 |
| ebi-a-GCST90001604 | rs10868215 | 9 | 87234111 | C | 0.3684 | -0.1205 | 0.02509 | 1.63E-06 |
| ebi-a-GCST90001948 | rs10869456 | 9 | 77499330 | G | 0.8766 | -0.2576 | 0.05498 | 3.04E-06 |
| ebi-a-GCST90001949 | rs10869456 | 9 | 77499330 | G | 0.8767 | -0.2539 | 0.05483 | 3.94E-06 |
| ebi-a-GCST90001524 | rs10872783 | 6 | 96696878 | C | 0.4278 | 0.1516 | 0.03215 | 2.59E-06 |
| ebi-a-GCST90001666 | rs10875036 | 1 | 97288628 | C | 0.2377 | -0.1218 | 0.02593 | 2.74E-06 |
| ebi-a-GCST90002024 | rs10876154 | 12 | 51607890 | T | 0.6918 | 0.1629 | 0.03018 | 7.30E-08 |
| ebi-a-GCST90001574 | rs10876180 | 12 | 51836616 | T | 0.6315 | -0.1219 | 0.02662 | 4.83E-06 |
| ebi-a-GCST90001950 | rs10876353 | 12 | 53217056 | A | 0.5358 | 0.1969 | 0.04094 | 1.70E-06 |
| ebi-a-GCST90002008 | rs10878258 | 12 | 40641692 | G | 0.4332 | -0.1382 | 0.02572 | 8.13E-08 |
| ebi-a-GCST90002004 | rs10878258 | 12 | 40641692 | G | 0.4332 | -0.1348 | 0.02549 | 1.32E-07 |
| ebi-a-GCST90001611 | rs10880825 | 12 | 45991896 | A | 0.4475 | 0.1141 | 0.02461 | 3.67E-06 |
| ebi-a-GCST90001610 | rs10880825 | 12 | 45991896 | A | 0.4475 | 0.1156 | 0.02452 | 2.52E-06 |
| ebi-a-GCST90001609 | rs10880825 | 12 | 45991896 | A | 0.4482 | 0.1208 | 0.02514 | 1.60E-06 |
| ebi-a-GCST90001911 | rs10882100 | 10 | 94460687 | G | 0.4998 | 0.1317 | 0.02741 | 1.63E-06 |
| ebi-a-GCST90001660 | rs10882324 | 10 | 82674931 | T | 0.3498 | -0.1207 | 0.0264 | 4.96E-06 |
| ebi-a-GCST90002031 | rs10882655 | 10 | 97474371 | A | 0.5276 | -1.008 | 0.02075 | 1.00E-200 |
| ebi-a-GCST90002032 | rs10882655 | 10 | 97474371 | A | 0.5276 | -0.9854 | 0.02111 | 1.00E-200 |
| ebi-a-GCST90001495 | rs10882655 | 10 | 97474371 | A | 0.532 | -0.9594 | 0.02046 | 1.00E-200 |
| ebi-a-GCST90001491 | rs10882655 | 10 | 97474371 | A | 0.5319 | -0.9139 | 0.02033 | 1.00E-200 |
| ebi-a-GCST90002069 | rs10882655 | 10 | 97474371 | A | 0.5276 | -0.2945 | 0.02665 | 7.84E-28 |
| ebi-a-GCST90001854 | rs10882659 | 10 | 97510704 | C | 0.4269 | -0.2226 | 0.02713 | 3.43E-16 |
| ebi-a-GCST90002030 | rs10882701 | 10 | 97741071 | C | 0.5156 | -0.4542 | 0.02621 | 4.05E-64 |
| ebi-a-GCST90001660 | rs10882701 | 10 | 97741071 | C | 0.5139 | -0.4118 | 0.0249 | 3.85E-59 |
| ebi-a-GCST90001495 | rs10882701 | 10 | 97741071 | C | 0.514 | -0.4069 | 0.02528 | 3.19E-56 |
| ebi-a-GCST90001496 | rs10882701 | 10 | 97741071 | C | 0.5141 | -0.4034 | 0.02502 | 2.04E-56 |
| ebi-a-GCST90001659 | rs10882701 | 10 | 97741071 | C | 0.5141 | -0.3997 | 0.02377 | 4.54E-61 |
| ebi-a-GCST90002031 | rs10882701 | 10 | 97741071 | C | 0.5156 | -0.3988 | 0.02725 | 7.28E-47 |
| ebi-a-GCST90001658 | rs10882701 | 10 | 97741071 | C | 0.5141 | -0.3969 | 0.02378 | 3.17E-60 |
| ebi-a-GCST90001497 | rs10882701 | 10 | 97741071 | C | 0.5141 | -0.3962 | 0.02472 | 8.03E-56 |
| ebi-a-GCST90002032 | rs10882701 | 10 | 97741071 | C | 0.5156 | -0.3837 | 0.0273 | 1.78E-43 |
| ebi-a-GCST90001484 | rs10882701 | 10 | 97741071 | C | 0.5141 | -0.3681 | 0.02438 | 6.66E-50 |
| ebi-a-GCST90001489 | rs10882701 | 10 | 97741071 | C | 0.514 | -0.3631 | 0.02528 | 1.80E-45 |
| ebi-a-GCST90001491 | rs10882701 | 10 | 97741071 | C | 0.5141 | -0.3538 | 0.02489 | 1.32E-44 |
| ebi-a-GCST90002029 | rs10882701 | 10 | 97741071 | C | 0.5153 | -0.1936 | 0.02737 | 1.87E-12 |
| ebi-a-GCST90001892 | rs10882701 | 10 | 97741071 | C | 0.5156 | -0.1597 | 0.02758 | 7.66E-09 |
| ebi-a-GCST90001888 | rs10882701 | 10 | 97741071 | C | 0.5156 | -0.136 | 0.02715 | 5.80E-07 |
| ebi-a-GCST90002032 | rs10883000 | 10 | 99565268 | C | 0.5628 | 0.1502 | 0.02818 | 1.07E-07 |
| ebi-a-GCST90001985 | rs10887024 | 10 | 123688152 | T | 0.3512 | -0.1469 | 0.02633 | 2.60E-08 |
| ebi-a-GCST90001980 | rs10887024 | 10 | 123688152 | T | 0.3512 | -0.1354 | 0.02616 | 2.40E-07 |
| ebi-a-GCST90001839 | rs10893076 | 11 | 123569986 | T | 0.4128 | 0.1308 | 0.0279 | 2.88E-06 |
| ebi-a-GCST90001741 | rs10894538 | 11 | 132222058 | A | 0.1059 | -0.1971 | 0.03986 | 8.00E-07 |
| ebi-a-GCST90001782 | rs10905716 | 10 | 6114010 | T | 0.2439 | -0.4537 | 0.03721 | 6.15E-33 |
| ebi-a-GCST90001507 | rs10905719 | 10 | 6115171 | A | 0.2093 | 0.1573 | 0.03239 | 1.26E-06 |
| ebi-a-GCST90001509 | rs10905719 | 10 | 6115171 | A | 0.2082 | 0.1734 | 0.03172 | 4.88E-08 |
| ebi-a-GCST90001508 | rs10905719 | 10 | 6115171 | A | 0.2082 | 0.1841 | 0.03172 | 7.06E-09 |
| ebi-a-GCST90001934 | rs10905719 | 10 | 6115171 | A | 0.2083 | 0.2281 | 0.02982 | 2.56E-14 |
| ebi-a-GCST90001494 | rs10905879 | 10 | 6177083 | T | 0.1286 | 0.1904 | 0.03733 | 3.55E-07 |
| ebi-a-GCST90001731 | rs10906006 | 10 | 11781967 | A | 0.7248 | 0.1265 | 0.0272 | 3.42E-06 |
| ebi-a-GCST90001807 | rs10910352 | 1 | 234267536 | A | 0.463 | -0.1148 | 0.02407 | 1.91E-06 |
| ebi-a-GCST90001474 | rs10910893 | 1 | 181160955 | T | 0.0707 | -0.2262 | 0.04889 | 3.86E-06 |
| ebi-a-GCST90002068 | rs10911945 | 1 | 186852597 | A | 0.0226 | -0.4588 | 0.09334 | 9.34E-07 |
| ebi-a-GCST90002063 | rs10911945 | 1 | 186852597 | A | 0.0226 | -0.4268 | 0.09315 | 4.81E-06 |
| ebi-a-GCST90002013 | rs10914660 | 1 | 33597788 | G | 0.3775 | -0.1399 | 0.02898 | 1.46E-06 |
| ebi-a-GCST90002019 | rs10917759 | 1 | 163435598 | G | 0.1634 | -0.2436 | 0.04863 | 6.06E-07 |
| ebi-a-GCST90001620 | rs1091849 | 16 | 10829280 | G | 0.6022 | -0.1268 | 0.02391 | 1.21E-07 |
| ebi-a-GCST90001628 | rs1091849 | 16 | 10829280 | G | 0.6024 | -0.1196 | 0.02424 | 8.46E-07 |
| ebi-a-GCST90001969 | rs10919543 | 1 | 161508617 | G | 0.5475 | -0.1509 | 0.02717 | 3.03E-08 |
| ebi-a-GCST90001884 | rs10919544 | 1 | 161508763 | C | 0.5475 | -0.2555 | 0.02745 | 2.40E-20 |
| ebi-a-GCST90002045 | rs10919544 | 1 | 161508763 | C | 0.552 | 0.238 | 0.03646 | 8.84E-11 |
| ebi-a-GCST90001527 | rs10921381 | 1 | 193500034 | T | 0.6709 | 0.1587 | 0.03399 | 3.22E-06 |
| ebi-a-GCST90001426 | rs10922490 | 1 | 89286072 | C | 0.0164 | 0.516 | 0.1044 | 8.16E-07 |
| ebi-a-GCST90001453 | rs10924239 | 1 | 245721841 | A | 0.0993 | 0.207 | 0.04455 | 3.52E-06 |
| ebi-a-GCST90001503 | rs10925924 | 1 | 239767145 | G | 0.8829 | -0.1944 | 0.03852 | 4.77E-07 |
| ebi-a-GCST90001494 | rs10925924 | 1 | 239767145 | G | 0.8829 | -0.1919 | 0.03875 | 7.73E-07 |
| ebi-a-GCST90002040 | rs10936226 | 3 | 94179989 | C | 0.164 | 0.173 | 0.03767 | 4.59E-06 |
| ebi-a-GCST90001959 | rs10946649 | 6 | 23957018 | T | 0.0632 | 0.2637 | 0.0551 | 1.79E-06 |
| ebi-a-GCST90001569 | rs10947785 | 6 | 39132818 | A | 0.5449 | -0.1139 | 0.02462 | 3.83E-06 |
| ebi-a-GCST90001647 | rs10948071 | 6 | 43280713 | T | 0.5327 | 0.1124 | 0.0236 | 1.97E-06 |
| ebi-a-GCST90001654 | rs10948242 | 6 | 45553259 | A | 0.3628 | 0.1241 | 0.02667 | 3.41E-06 |
| ebi-a-GCST90001997 | rs10951824 | 7 | 45618083 | C | 0.3248 | -0.1327 | 0.0268 | 7.65E-07 |
| ebi-a-GCST90001995 | rs10951824 | 7 | 45618083 | C | 0.3248 | -0.1268 | 0.02663 | 1.99E-06 |
| ebi-a-GCST90001919 | rs10952012 | 7 | 1335231 | G | 0.8603 | 0.1895 | 0.03861 | 9.65E-07 |
| ebi-a-GCST90001675 | rs10952014 | 7 | 7026245 | G | 0.1823 | -0.1603 | 0.03317 | 1.42E-06 |
| ebi-a-GCST90001698 | rs10965305 | 9 | 22268719 | G | 0.0037 | 142.7 | 28.63 | 6.51E-07 |
| ebi-a-GCST90001751 | rs10965813 | 9 | 23378556 | G | 0.111 | 0.1822 | 0.03837 | 2.12E-06 |
| ebi-a-GCST90001585 | rs10970605 | 9 | 31939695 | C | 0.0107 | -0.5996 | 0.1227 | 1.06E-06 |
| ebi-a-GCST90001580 | rs10970605 | 9 | 31939695 | C | 0.0107 | -0.5822 | 0.1212 | 1.64E-06 |
| ebi-a-GCST90001696 | rs10970967 | 9 | 3250025 | G | 0.3676 | 1.525 | 0.3153 | 1.37E-06 |
| ebi-a-GCST90002121 | rs10974005 | 9 | 38733270 | C | 0.2537 | -0.1518 | 0.03136 | 1.37E-06 |
| ebi-a-GCST90001565 | rs10976338 | 9 | 7510150 | C | 0.0312 | -0.3243 | 0.06286 | 2.62E-07 |
| ebi-a-GCST90001697 | rs10987410 | 9 | 129448120 | C | 0.1001 | 0.9898 | 0.2055 | 1.52E-06 |
| ebi-a-GCST90001423 | rs10988372 | 9 | 132193568 | C | 0.2045 | 0.139 | 0.03012 | 4.09E-06 |
| ebi-a-GCST90001593 | rs10990269 | 9 | 99054924 | T | 0.3525 | 0.1232 | 0.02583 | 1.91E-06 |
| ebi-a-GCST90001817 | rs10991116 | 9 | 106896351 | A | 0.0728 | 0.2334 | 0.04583 | 3.68E-07 |
| ebi-a-GCST90001799 | rs10996192 | 10 | 66846516 | A | 0.0299 | 0.434 | 0.09296 | 3.25E-06 |
| ebi-a-GCST90001851 | rs10996702 | 10 | 67597707 | A | 0.2648 | 0.1468 | 0.03117 | 2.61E-06 |
| ebi-a-GCST90001674 | rs10998956 | 10 | 71525881 | G | 0.0074 | -0.6975 | 0.1481 | 2.58E-06 |
| ebi-a-GCST90001471 | rs11000032 | 10 | 73619601 | A | 0.2843 | 0.1292 | 0.02767 | 3.13E-06 |
| ebi-a-GCST90001489 | rs11002579 | 10 | 80258836 | A | 0.3366 | -0.1316 | 0.02727 | 1.46E-06 |
| ebi-a-GCST90002020 | rs11003240 | 10 | 54641214 | T | 0.0963 | -0.314 | 0.06281 | 6.42E-07 |
| ebi-a-GCST90001806 | rs11007079 | 10 | 28679045 | T | 0.1187 | -0.1822 | 0.03889 | 2.89E-06 |
| ebi-a-GCST90002020 | rs11007733 | 10 | 30059784 | T | 0.0928 | -0.2934 | 0.06355 | 4.20E-06 |
| ebi-a-GCST90002121 | rs11008428 | 10 | 31536806 | C | 0.0379 | -0.3316 | 0.07112 | 3.25E-06 |
| ebi-a-GCST90001879 | rs11014115 | 10 | 24786510 | A | 0.3944 | -0.217 | 0.04205 | 2.85E-07 |
| ebi-a-GCST90001826 | rs11014404 | 10 | 25408128 | G | 0.2874 | 0.1275 | 0.02747 | 3.60E-06 |
| ebi-a-GCST90002097 | rs11015080 | 10 | 26677896 | T | 0.5856 | -0.172 | 0.03681 | 3.23E-06 |
| ebi-a-GCST90001757 | rs11020715 | 11 | 93989208 | G | 0.3418 | -0.1261 | 0.02638 | 1.84E-06 |
| ebi-a-GCST90001594 | rs11025036 | 11 | 19175633 | A | 0.022 | -0.4567 | 0.08253 | 3.35E-08 |
| ebi-a-GCST90001608 | rs11025036 | 11 | 19175633 | A | 0.0221 | -0.4277 | 0.08196 | 1.91E-07 |
| ebi-a-GCST90002026 | rs11026566 | 11 | 22472189 | G | 0.2442 | -0.1442 | 0.03126 | 4.16E-06 |
| ebi-a-GCST90001989 | rs11032433 | 11 | 33903882 | C | 0.2084 | -0.1433 | 0.0307 | 3.15E-06 |
| ebi-a-GCST90002026 | rs11033545 | 11 | 36327582 | G | 0.3779 | 0.1352 | 0.02813 | 1.61E-06 |
| ebi-a-GCST90001787 | rs11036291 | 11 | 41256894 | G | 0.2101 | -0.1424 | 0.03012 | 2.37E-06 |
| ebi-a-GCST90002002 | rs11038817 | 11 | 46226004 | T | 0.0397 | 0.2878 | 0.06275 | 4.66E-06 |
| ebi-a-GCST90001816 | rs11042077 | 11 | 8825966 | A | 0.4943 | 0.1217 | 0.02507 | 1.27E-06 |
| ebi-a-GCST90001811 | rs11042077 | 11 | 8825966 | A | 0.4943 | 0.1233 | 0.02525 | 1.09E-06 |
| ebi-a-GCST90001849 | rs11042750 | 11 | 10372959 | T | 0.6373 | 0.1283 | 0.02769 | 3.78E-06 |
| ebi-a-GCST90001828 | rs11047379 | 12 | 24492761 | G | 0.2949 | 0.1333 | 0.02714 | 9.41E-07 |
| ebi-a-GCST90002059 | rs11050506 | 12 | 30013127 | C | 0.0234 | -0.4095 | 0.08809 | 3.48E-06 |
| ebi-a-GCST90001791 | rs11051398 | 12 | 31508331 | T | 0.5325 | -0.1509 | 0.02607 | 7.67E-09 |
| ebi-a-GCST90001779 | rs11051398 | 12 | 31508331 | T | 0.5325 | -0.1343 | 0.02589 | 2.24E-07 |
| ebi-a-GCST90001784 | rs11051398 | 12 | 31508331 | T | 0.5325 | -0.123 | 0.02607 | 2.49E-06 |
| ebi-a-GCST90001775 | rs11051398 | 12 | 31508331 | T | 0.5325 | -0.1219 | 0.02602 | 2.92E-06 |
| ebi-a-GCST90001794 | rs11051398 | 12 | 31508331 | T | 0.5325 | -0.1194 | 0.02601 | 4.58E-06 |
| ebi-a-GCST90001743 | rs11051833 | 12 | 32341983 | T | 0.0286 | -0.359 | 0.07394 | 1.25E-06 |
| ebi-a-GCST90001754 | rs11051833 | 12 | 32341983 | T | 0.0286 | -0.3556 | 0.07383 | 1.52E-06 |
| ebi-a-GCST90001762 | rs11051833 | 12 | 32341983 | T | 0.0286 | -0.3505 | 0.07384 | 2.15E-06 |
| ebi-a-GCST90001747 | rs11051833 | 12 | 32341983 | T | 0.0286 | -0.3439 | 0.07379 | 3.28E-06 |
| ebi-a-GCST90001759 | rs11051833 | 12 | 32341983 | T | 0.0286 | -0.3438 | 0.07381 | 3.31E-06 |
| ebi-a-GCST90001619 | rs11054779 | 12 | 12458534 | T | 0.0331 | 0.2888 | 0.06232 | 3.70E-06 |
| ebi-a-GCST90001628 | rs11054779 | 12 | 12458534 | T | 0.0331 | 0.2975 | 0.06495 | 4.79E-06 |
| ebi-a-GCST90001629 | rs11054779 | 12 | 12458534 | T | 0.0331 | 0.3118 | 0.06587 | 2.29E-06 |
| ebi-a-GCST90001620 | rs11054779 | 12 | 12458534 | T | 0.0331 | 0.3154 | 0.06396 | 8.55E-07 |
| ebi-a-GCST90001698 | rs11060236 | 12 | 129773605 | G | 0.0031 | 216.9 | 30.85 | 2.49E-12 |
| ebi-a-GCST90001592 | rs11060493 | 12 | 130160924 | C | 0.102 | 0.1796 | 0.03884 | 3.91E-06 |
| ebi-a-GCST90001684 | rs11060493 | 12 | 130160924 | C | 0.1018 | 0.2008 | 0.04204 | 1.85E-06 |
| ebi-a-GCST90001583 | rs11061521 | 12 | 131900390 | A | 0.1962 | 0.1409 | 0.02965 | 2.10E-06 |
| ebi-a-GCST90001537 | rs11062774 | 12 | 3833312 | G | 0.1558 | 0.1592 | 0.03482 | 4.98E-06 |
| ebi-a-GCST90001534 | rs11062774 | 12 | 3833312 | G | 0.1558 | 0.1756 | 0.03579 | 9.68E-07 |
| ebi-a-GCST90001700 | rs1106305 | 11 | 12065087 | T | 0.0172 | 2.681 | 0.5792 | 3.82E-06 |
| ebi-a-GCST90001698 | rs1106305 | 11 | 12065087 | T | 0.0173 | 95.49 | 13.66 | 3.28E-12 |
| ebi-a-GCST90001802 | rs11064198 | 12 | 6558720 | T | 0.1256 | -0.225 | 0.03691 | 1.19E-09 |
| ebi-a-GCST90001990 | rs11065979 | 12 | 112059557 | T | 0.4691 | 0.1181 | 0.0257 | 4.43E-06 |
| ebi-a-GCST90002006 | rs11065979 | 12 | 112059557 | T | 0.4689 | 0.1291 | 0.02624 | 9.05E-07 |
| ebi-a-GCST90002011 | rs11065979 | 12 | 112059557 | T | 0.469 | 0.1497 | 0.02574 | 6.46E-09 |
| ebi-a-GCST90001487 | rs11066188 | 12 | 112610714 | A | 0.4658 | -0.1254 | 0.02574 | 1.15E-06 |
| ebi-a-GCST90001402 | rs11068290 | 12 | 110228055 | T | 0.0231 | -0.407 | 0.08203 | 7.31E-07 |
| ebi-a-GCST90001445 | rs11068290 | 12 | 110228055 | T | 0.0231 | -0.3746 | 0.0813 | 4.21E-06 |
| ebi-a-GCST90001429 | rs11068290 | 12 | 110228055 | T | 0.0231 | 0.3875 | 0.07937 | 1.09E-06 |
| ebi-a-GCST90001391 | rs11068290 | 12 | 110228055 | T | 0.0231 | 0.3904 | 0.08097 | 1.49E-06 |
| ebi-a-GCST90002009 | rs11071444 | 15 | 59740740 | A | 0.036 | -0.3442 | 0.06767 | 3.84E-07 |
| ebi-a-GCST90001947 | rs11073171 | 15 | 36675994 | A | 0.6208 | -0.1734 | 0.03735 | 3.73E-06 |
| ebi-a-GCST90001896 | rs11074594 | 16 | 23973371 | A | 0.3973 | -0.1335 | 0.02879 | 3.70E-06 |
| ebi-a-GCST90001970 | rs11074932 | 16 | 10968336 | C | 0.3061 | -0.1459 | 0.02913 | 5.83E-07 |
| ebi-a-GCST90002111 | rs11074934 | 16 | 10979440 | T | 0.2193 | -0.4126 | 0.04076 | 2.14E-23 |
| ebi-a-GCST90002110 | rs11074934 | 16 | 10979440 | T | 0.2194 | -0.3589 | 0.04157 | 1.36E-17 |
| ebi-a-GCST90001957 | rs11078779 | 17 | 8952193 | G | 0.32 | 0.1935 | 0.03982 | 1.30E-06 |
| ebi-a-GCST90001956 | rs11078779 | 17 | 8952193 | G | 0.3198 | 0.1969 | 0.03979 | 8.29E-07 |
| ebi-a-GCST90001947 | rs11078779 | 17 | 8952193 | G | 0.3196 | 0.1989 | 0.03983 | 6.59E-07 |
| ebi-a-GCST90001922 | rs11078935 | 17 | 38192137 | G | 0.3147 | -0.1365 | 0.02888 | 2.39E-06 |
| ebi-a-GCST90001614 | rs11080350 | 17 | 33774635 | T | 0.4017 | -0.1548 | 0.0252 | 9.08E-10 |
| ebi-a-GCST90001607 | rs11080350 | 17 | 33774635 | T | 0.4017 | -0.1371 | 0.02524 | 5.92E-08 |
| ebi-a-GCST90001592 | rs11080350 | 17 | 33774635 | T | 0.4018 | -0.1209 | 0.02469 | 1.02E-06 |
| ebi-a-GCST90002076 | rs11081793 | 18 | 19722536 | G | 0.0255 | -0.3945 | 0.08374 | 2.57E-06 |
| ebi-a-GCST90001747 | rs11083972 | 19 | 49896752 | G | 0.2234 | -0.155 | 0.02995 | 2.41E-07 |
| ebi-a-GCST90001913 | rs11084094 | 19 | 52119546 | C | 0.4807 | 0.141 | 0.02663 | 1.28E-07 |
| ebi-a-GCST90001399 | rs11086984 | 20 | 44511627 | T | 0.1514 | -0.1811 | 0.03498 | 2.37E-07 |
| ebi-a-GCST90001800 | rs11086984 | 20 | 44511627 | T | 0.1513 | 0.1911 | 0.03505 | 5.27E-08 |
| ebi-a-GCST90001806 | rs11086984 | 20 | 44511627 | T | 0.1513 | 0.1988 | 0.03485 | 1.25E-08 |
| ebi-a-GCST90001805 | rs11086984 | 20 | 44511627 | T | 0.1514 | 0.199 | 0.03488 | 1.25E-08 |
| ebi-a-GCST90001802 | rs11086984 | 20 | 44511627 | T | 0.1514 | 0.2106 | 0.03486 | 1.67E-09 |
| ebi-a-GCST90001798 | rs11086984 | 20 | 44511627 | T | 0.1514 | 0.256 | 0.03444 | 1.30E-13 |
| ebi-a-GCST90001804 | rs11086984 | 20 | 44511627 | T | 0.1514 | 0.2742 | 0.03426 | 1.59E-15 |
| ebi-a-GCST90001808 | rs11086984 | 20 | 44511627 | T | 0.1514 | 0.2761 | 0.03467 | 2.23E-15 |
| ebi-a-GCST90001435 | rs11089305 | 22 | 19729361 | G | 0.2882 | 0.1295 | 0.02684 | 1.45E-06 |
| ebi-a-GCST90001404 | rs11089305 | 22 | 19729361 | G | 0.2881 | 0.1338 | 0.02685 | 6.52E-07 |
| ebi-a-GCST90001626 | rs11090233 | 22 | 23690325 | A | 0.611 | -0.11 | 0.02271 | 1.33E-06 |
| ebi-a-GCST90001707 | rs11090614 | 22 | 44300847 | G | 0.2474 | -0.3049 | 0.03896 | 8.45E-15 |
| ebi-a-GCST90001940 | rs11097003 | 4 | 39796980 | C | 0.5878 | 0.1223 | 0.02581 | 2.26E-06 |
| ebi-a-GCST90001700 | rs111065494 | 7 | 48369369 | T | 0.0079 | 4.133 | 0.8427 | 9.81E-07 |
| ebi-a-GCST90001679 | rs111066413 | 8 | 142715815 | A | 0.004 | 1.038 | 0.2095 | 7.66E-07 |
| ebi-a-GCST90001681 | rs111066413 | 8 | 142715815 | A | 0.0038 | 1.07 | 0.2137 | 5.87E-07 |
| ebi-a-GCST90001999 | rs11107060 | 12 | 93916757 | G | 0.1882 | 0.1532 | 0.03203 | 1.80E-06 |
| ebi-a-GCST90002029 | rs11107237 | 12 | 94282149 | T | 0.5451 | 0.1375 | 0.02722 | 4.60E-07 |
| ebi-a-GCST90001560 | rs11110042 | 12 | 100186724 | T | 0.204 | 0.1248 | 0.02717 | 4.52E-06 |
| ebi-a-GCST90001530 | rs11112649 | 12 | 106064969 | C | 0.2984 | -0.1727 | 0.03637 | 2.20E-06 |
| ebi-a-GCST90001698 | rs11112760 | 12 | 106241673 | T | 0.0173 | 82 | 13.96 | 4.67E-09 |
| ebi-a-GCST90002095 | rs11114574 | 12 | 81094063 | A | 0.0366 | 0.4701 | 0.0999 | 2.76E-06 |
| ebi-a-GCST90002004 | rs111162334 | 7 | 51430245 | T | 0.4928 | 0.1125 | 0.02437 | 4.03E-06 |
| ebi-a-GCST90001741 | rs11120838 | 1 | 7151262 | G | 0.0433 | -0.29 | 0.06156 | 2.56E-06 |
| ebi-a-GCST90002120 | rs11121500 | 1 | 6279616 | G | 0.0906 | -0.22 | 0.04802 | 4.83E-06 |
| ebi-a-GCST90001679 | rs111237279 | 4 | 184874118 | G | 0.2362 | -0.1422 | 0.03075 | 3.89E-06 |
| ebi-a-GCST90001414 | rs111237501 | 1 | 163272701 | C | 0.003 | 1.052 | 0.2261 | 3.35E-06 |
| ebi-a-GCST90001413 | rs111237501 | 1 | 163272701 | C | 0.003 | 1.055 | 0.2248 | 2.81E-06 |
| ebi-a-GCST90001636 | rs11124150 | 2 | 98905012 | G | 0.5968 | -0.1283 | 0.02509 | 3.33E-07 |
| ebi-a-GCST90001598 | rs11124150 | 2 | 98905012 | G | 0.5968 | -0.1256 | 0.02408 | 1.92E-07 |
| ebi-a-GCST90001569 | rs11124150 | 2 | 98905012 | G | 0.5956 | -0.1192 | 0.02478 | 1.59E-06 |
| ebi-a-GCST90001638 | rs11124150 | 2 | 98905012 | G | 0.5966 | -0.1185 | 0.02525 | 2.80E-06 |
| ebi-a-GCST90001637 | rs11124150 | 2 | 98905012 | G | 0.5966 | -0.118 | 0.02536 | 3.39E-06 |
| ebi-a-GCST90001613 | rs11124150 | 2 | 98905012 | G | 0.5966 | -0.115 | 0.02449 | 2.76E-06 |
| ebi-a-GCST90001599 | rs11124150 | 2 | 98905012 | G | 0.5966 | -0.1138 | 0.02481 | 4.66E-06 |
| ebi-a-GCST90002102 | rs11124653 | 2 | 39112166 | A | 0.5925 | -0.2555 | 0.02636 | 6.84E-22 |
| ebi-a-GCST90001508 | rs11124653 | 2 | 39112166 | A | 0.5911 | -0.2138 | 0.02624 | 5.23E-16 |
| ebi-a-GCST90001509 | rs11124653 | 2 | 39112166 | A | 0.5911 | -0.1952 | 0.02627 | 1.36E-13 |
| ebi-a-GCST90001507 | rs11124653 | 2 | 39112166 | A | 0.5907 | -0.1861 | 0.0267 | 3.74E-12 |
| ebi-a-GCST90001541 | rs11124653 | 2 | 39112166 | A | 0.5909 | -0.1137 | 0.0239 | 2.04E-06 |
| ebi-a-GCST90001494 | rs11124653 | 2 | 39112166 | A | 0.5911 | 0.1191 | 0.026 | 4.79E-06 |
| ebi-a-GCST90001535 | rs11124653 | 2 | 39112166 | A | 0.5909 | 0.1345 | 0.02443 | 3.92E-08 |
| ebi-a-GCST90001844 | rs11124860 | 2 | 42337670 | G | 0.4754 | -0.1291 | 0.02737 | 2.49E-06 |
| ebi-a-GCST90002030 | rs111253358 | 10 | 64441033 | T | 0.0192 | -0.5073 | 0.1031 | 8.99E-07 |
| ebi-a-GCST90001745 | rs111259171 | 6 | 32640351 | G | 0.1947 | 0.2545 | 0.04474 | 1.38E-08 |
| ebi-a-GCST90002058 | rs11126482 | 2 | 75977801 | C | 0.2454 | -0.1449 | 0.03072 | 2.49E-06 |
| ebi-a-GCST90001598 | rs111273508 | 6 | 25330739 | A | 0.0116 | 0.5059 | 0.1084 | 3.19E-06 |
| ebi-a-GCST90001663 | rs111273508 | 6 | 25330739 | A | 0.0117 | 0.5414 | 0.118 | 4.62E-06 |
| ebi-a-GCST90001633 | rs111273508 | 6 | 25330739 | A | 0.0116 | 0.5462 | 0.1137 | 1.63E-06 |
| ebi-a-GCST90001596 | rs111273508 | 6 | 25330739 | A | 0.0116 | 0.5877 | 0.1116 | 1.46E-07 |
| ebi-a-GCST90001990 | rs111279951 | 5 | 18068020 | A | 0.0418 | 0.3 | 0.06342 | 2.32E-06 |
| ebi-a-GCST90002116 | rs111281233 | 2 | 230286959 | A | 0.0077 | -0.7625 | 0.1492 | 3.40E-07 |
| ebi-a-GCST90001847 | rs111281575 | 1 | 166737851 | C | 0.0521 | 0.2904 | 0.06132 | 2.28E-06 |
| ebi-a-GCST90001846 | rs111281575 | 1 | 166737851 | C | 0.0529 | 0.3212 | 0.06118 | 1.63E-07 |
| ebi-a-GCST90001654 | rs11128263 | 3 | 72394027 | T | 0.5246 | -0.1189 | 0.02529 | 2.68E-06 |
| ebi-a-GCST90001653 | rs11128263 | 3 | 72394027 | T | 0.524 | -0.117 | 0.02521 | 3.58E-06 |
| ebi-a-GCST90001656 | rs11128263 | 3 | 72394027 | T | 0.524 | 0.117 | 0.02521 | 3.58E-06 |
| ebi-a-GCST90001688 | rs111283043 | 16 | 79205197 | C | 1.00E-04 | 4.463 | 0.7922 | 1.90E-08 |
| ebi-a-GCST90001692 | rs111328168 | 6 | 149103548 | G | 3.00E-04 | -3.338 | 0.7169 | 3.34E-06 |
| ebi-a-GCST90001698 | rs111347604 | 1 | 85641879 | T | 0.0141 | 86.06 | 17.26 | 6.46E-07 |
| ebi-a-GCST90001748 | rs11135082 | 5 | 159137396 | G | 0.0657 | 0.2303 | 0.04967 | 3.66E-06 |
| ebi-a-GCST90002121 | rs111359007 | 6 | 31761201 | A | 0.1015 | -0.2802 | 0.04787 | 5.34E-09 |
| ebi-a-GCST90002073 | rs11136335 | 8 | 145003777 | A | 0.4853 | -0.1653 | 0.02755 | 2.20E-09 |
| ebi-a-GCST90001757 | rs111369841 | 6 | 32921104 | T | 0.0967 | 0.2215 | 0.04688 | 2.39E-06 |
| ebi-a-GCST90001761 | rs111369841 | 6 | 32921104 | T | 0.0967 | 0.2275 | 0.04713 | 1.43E-06 |
| ebi-a-GCST90001698 | rs111373539 | 9 | 107628892 | A | 0.0283 | 52.34 | 10.84 | 1.43E-06 |
| ebi-a-GCST90001602 | rs111373552 | 16 | 25912505 | T | 0.0754 | 0.2168 | 0.04737 | 4.87E-06 |
| ebi-a-GCST90001697 | rs111388649 | 12 | 28246470 | A | 1.00E-04 | 26.26 | 4.684 | 2.22E-08 |
| ebi-a-GCST90001910 | rs111395861 | 2 | 14940150 | A | 0.0164 | 0.4495 | 0.09715 | 3.85E-06 |
| ebi-a-GCST90001698 | rs111399817 | 7 | 146550168 | T | 0.0015 | 303 | 43.46 | 3.75E-12 |
| ebi-a-GCST90002109 | rs111404935 | 6 | 66907682 | C | 0.0608 | -0.4016 | 0.07817 | 3.13E-07 |
| ebi-a-GCST90002009 | rs111407432 | 6 | 152585213 | A | 0.1137 | 0.1916 | 0.03967 | 1.42E-06 |
| ebi-a-GCST90001433 | rs111407982 | 19 | 8814367 | T | 0.1551 | -0.1612 | 0.03377 | 1.87E-06 |
| ebi-a-GCST90001698 | rs111409203 | 2 | 19743796 | G | 0.0035 | 156.4 | 29.26 | 9.60E-08 |
| ebi-a-GCST90001786 | rs111419261 | 9 | 91520493 | A | 0.024 | 0.3724 | 0.07876 | 2.34E-06 |
| ebi-a-GCST90001605 | rs1114226 | 6 | 77834631 | G | 0.5162 | -0.1111 | 0.02422 | 4.64E-06 |
| ebi-a-GCST90001989 | rs111436326 | 20 | 42701487 | T | 0.034 | 0.4119 | 0.06902 | 2.63E-09 |
| ebi-a-GCST90001496 | rs111461378 | 20 | 15575550 | C | 0.0028 | -1.107 | 0.2386 | 3.59E-06 |
| ebi-a-GCST90001657 | rs111474820 | 9 | 4488885 | G | 0.0089 | -0.5354 | 0.1136 | 2.54E-06 |
| ebi-a-GCST90001618 | rs111481219 | 16 | 11544197 | T | 0.0775 | -0.2209 | 0.04518 | 1.05E-06 |
| ebi-a-GCST90002115 | rs111484143 | 13 | 93687655 | G | 0.0466 | 0.2877 | 0.06162 | 3.15E-06 |
| ebi-a-GCST90001785 | rs111500944 | 15 | 80676525 | T | 0.0517 | 0.2685 | 0.05574 | 1.52E-06 |
| ebi-a-GCST90001688 | rs111503027 | 8 | 59820131 | A | 1.00E-04 | 3.141 | 0.639 | 9.27E-07 |
| ebi-a-GCST90001395 | rs11150426 | 16 | 81986583 | C | 0.5386 | -0.1169 | 0.02444 | 1.78E-06 |
| ebi-a-GCST90001628 | rs111509656 | 9 | 101054951 | C | 0.0257 | 0.3641 | 0.07776 | 2.95E-06 |
| ebi-a-GCST90001832 | rs111528892 | 13 | 27228287 | A | 0.028 | 0.4028 | 0.08171 | 8.72E-07 |
| ebi-a-GCST90001554 | rs11153123 | 6 | 109038333 | G | 0.2296 | -0.1435 | 0.03074 | 3.15E-06 |
| ebi-a-GCST90001880 | rs11153673 | 6 | 117863943 | C | 0.0381 | -0.5114 | 0.1019 | 5.96E-07 |
| ebi-a-GCST90001698 | rs111541596 | 21 | 15989102 | C | 0.0028 | 171.6 | 32.8 | 1.78E-07 |
| ebi-a-GCST90001556 | rs111551789 | 6 | 29720277 | A | 0.0204 | 0.4285 | 0.09279 | 4.01E-06 |
| ebi-a-GCST90002048 | rs111573488 | 12 | 82723650 | A | 0.0082 | -0.9592 | 0.2046 | 3.01E-06 |
| ebi-a-GCST90001884 | rs111579151 | 11 | 111848073 | T | 0.0323 | -0.5632 | 0.07427 | 4.45E-14 |
| ebi-a-GCST90002024 | rs11158593 | 14 | 65859984 | A | 0.479 | 0.1981 | 0.02746 | 6.89E-13 |
| ebi-a-GCST90001392 | rs11159480 | 14 | 81466662 | T | 0.8166 | -0.1526 | 0.0323 | 2.40E-06 |
| ebi-a-GCST90001722 | rs11161637 | 1 | 86021169 | G | 0.5694 | -0.1116 | 0.02439 | 4.96E-06 |
| ebi-a-GCST90001517 | rs11161969 | 1 | 87752464 | G | 0.4128 | 0.1593 | 0.03298 | 1.48E-06 |
| ebi-a-GCST90001524 | rs111636975 | 2 | 176606506 | G | 0.0075 | 0.8776 | 0.18 | 1.18E-06 |
| ebi-a-GCST90002072 | rs11164360 | 1 | 102472108 | C | 0.0061 | 0.8331 | 0.1821 | 4.94E-06 |
| ebi-a-GCST90001828 | rs11164796 | 1 | 93144080 | T | 0.5436 | 0.1235 | 0.0259 | 1.94E-06 |
| ebi-a-GCST90001698 | rs111648746 | 13 | 28485345 | A | 0.0015 | 308.6 | 43.15 | 1.04E-12 |
| ebi-a-GCST90002117 | rs111660650 | 6 | 135147576 | T | 0.0024 | -1.344 | 0.2906 | 3.89E-06 |
| ebi-a-GCST90001646 | rs111661128 | 17 | 76403698 | A | 0.0232 | 0.3791 | 0.08035 | 2.47E-06 |
| ebi-a-GCST90001554 | rs11166573 | 1 | 101733861 | A | 0.4127 | -0.1203 | 0.02612 | 4.25E-06 |
| ebi-a-GCST90001840 | rs111666485 | 12 | 15638143 | A | 0.0414 | 0.3232 | 0.06931 | 3.26E-06 |
| ebi-a-GCST90001586 | rs111690368 | 1 | 91084546 | T | 0.0054 | -0.7379 | 0.1589 | 3.54E-06 |
| ebi-a-GCST90001610 | rs111693583 | 16 | 28583610 | T | 0.4027 | 0.1223 | 0.02654 | 4.16E-06 |
| ebi-a-GCST90001477 | rs11169475 | 12 | 50914563 | A | 0.0296 | -0.3396 | 0.07329 | 3.72E-06 |
| ebi-a-GCST90001954 | rs111695695 | 19 | 51897760 | A | 0.0772 | -0.5454 | 0.06799 | 2.04E-15 |
| ebi-a-GCST90001955 | rs111695695 | 19 | 51897760 | A | 0.0772 | -0.5128 | 0.06836 | 1.06E-13 |
| ebi-a-GCST90002052 | rs111695695 | 19 | 51897760 | A | 0.0772 | 0.3963 | 0.06945 | 1.38E-08 |
| ebi-a-GCST90001874 | rs111696805 | 7 | 54565976 | A | 0.0028 | -1.753 | 0.3778 | 3.85E-06 |
| ebi-a-GCST90001769 | rs111701276 | 9 | 28274639 | G | 0.0049 | 0.8833 | 0.1766 | 5.94E-07 |
| ebi-a-GCST90001773 | rs111701276 | 9 | 28274639 | G | 0.0049 | 0.885 | 0.1761 | 5.22E-07 |
| ebi-a-GCST90002049 | rs111704828 | 10 | 2108255 | T | 0.0226 | -0.5854 | 0.1236 | 2.37E-06 |
| ebi-a-GCST90001803 | rs11171232 | 12 | 55446669 | T | 0.047 | -0.2878 | 0.06024 | 1.85E-06 |
| ebi-a-GCST90001698 | rs111714479 | 21 | 35532237 | A | 0.0029 | 199 | 34.88 | 1.26E-08 |
| ebi-a-GCST90001698 | rs111730307 | 14 | 49236170 | T | 0.0022 | 167.3 | 36.46 | 4.64E-06 |
| ebi-a-GCST90001695 | rs111731860 | 5 | 175121768 | T | 0.0296 | 36.01 | 7.839 | 4.51E-06 |
| ebi-a-GCST90001749 | rs111741301 | 13 | 71781323 | C | 0.0592 | 0.3444 | 0.07403 | 3.52E-06 |
| ebi-a-GCST90001784 | rs111741355 | 1 | 48095312 | A | 4.00E-04 | -2.854 | 0.5728 | 6.55E-07 |
| ebi-a-GCST90001747 | rs111741355 | 1 | 48095312 | A | 4.00E-04 | -2.647 | 0.5709 | 3.67E-06 |
| ebi-a-GCST90001794 | rs111741355 | 1 | 48095312 | A | 4.00E-04 | -2.639 | 0.5717 | 4.03E-06 |
| ebi-a-GCST90001779 | rs111741355 | 1 | 48095312 | A | 4.00E-04 | -2.621 | 0.5722 | 4.80E-06 |
| ebi-a-GCST90001752 | rs111741355 | 1 | 48095312 | A | 4.00E-04 | -2.608 | 0.5679 | 4.54E-06 |
| ebi-a-GCST90001698 | rs111745963 | 3 | 67860216 | C | 0.0035 | 196.5 | 29.94 | 6.06E-11 |
| ebi-a-GCST90001700 | rs111747460 | 3 | 74016318 | A | 3.00E-04 | 26.82 | 4.296 | 4.81E-10 |
| ebi-a-GCST90001612 | rs111748563 | 19 | 17901533 | G | 0.0491 | 0.2703 | 0.057 | 2.20E-06 |
| ebi-a-GCST90001663 | rs111748563 | 19 | 17901533 | G | 0.0503 | 0.2783 | 0.05843 | 1.99E-06 |
| ebi-a-GCST90001597 | rs111748563 | 19 | 17901533 | G | 0.0491 | 0.2822 | 0.05658 | 6.43E-07 |
| ebi-a-GCST90001596 | rs111748563 | 19 | 17901533 | G | 0.0493 | 0.287 | 0.0554 | 2.32E-07 |
| ebi-a-GCST90001582 | rs11177104 | 12 | 68605883 | A | 0.0076 | -0.6203 | 0.1351 | 4.58E-06 |
| ebi-a-GCST90001698 | rs111777292 | 3 | 192717255 | T | 0.0051 | 126.7 | 26.42 | 1.71E-06 |
| ebi-a-GCST90001838 | rs111780443 | 11 | 132210362 | T | 0.045 | -0.3659 | 0.06602 | 3.25E-08 |
| ebi-a-GCST90001477 | rs111801150 | 13 | 114508277 | G | 0.0013 | -1.644 | 0.3396 | 1.34E-06 |
| ebi-a-GCST90001489 | rs111806244 | 10 | 47637202 | T | 0.0476 | -0.3653 | 0.07836 | 3.26E-06 |
| ebi-a-GCST90001613 | rs11180778 | 12 | 76371057 | C | 0.2719 | 0.1276 | 0.02655 | 1.59E-06 |
| ebi-a-GCST90001599 | rs11180823 | 12 | 76477941 | C | 0.3615 | -0.1225 | 0.02472 | 7.53E-07 |
| ebi-a-GCST90001986 | rs111820782 | 14 | 28914556 | T | 0.0025 | 1.103 | 0.24 | 4.43E-06 |
| ebi-a-GCST90001441 | rs111830700 | 7 | 121332400 | A | 0.0377 | 0.2933 | 0.0634 | 3.86E-06 |
| ebi-a-GCST90001415 | rs11184230 | 1 | 105159724 | T | 0.1095 | -0.1946 | 0.03983 | 1.08E-06 |
| ebi-a-GCST90002028 | rs111853663 | 16 | 29181468 | A | 8.00E-04 | -1.962 | 0.4185 | 2.89E-06 |
| ebi-a-GCST90001487 | rs11185398 | 1 | 104762898 | G | 0.0615 | -0.2394 | 0.05089 | 2.65E-06 |
| ebi-a-GCST90001428 | rs111854570 | 3 | 8695105 | A | 0.0388 | -0.2872 | 0.06245 | 4.41E-06 |
| ebi-a-GCST90001907 | rs111855096 | 22 | 48693531 | G | 0.0649 | 0.2542 | 0.05482 | 3.69E-06 |
| ebi-a-GCST90001429 | rs11185560 | 7 | 194756 | A | 0.9375 | -0.2347 | 0.05014 | 2.96E-06 |
| ebi-a-GCST90001673 | rs111856210 | 7 | 7026180 | A | 0.1154 | -0.2121 | 0.03973 | 1.00E-07 |
| ebi-a-GCST90001674 | rs111856210 | 7 | 7026180 | A | 0.1154 | -0.2016 | 0.04015 | 5.41E-07 |
| ebi-a-GCST90001889 | rs111860462 | 14 | 82047636 | T | 0.0017 | 1.443 | 0.3087 | 3.08E-06 |
| ebi-a-GCST90001886 | rs111860462 | 14 | 82047636 | T | 0.0017 | 1.539 | 0.3071 | 5.70E-07 |
| ebi-a-GCST90001902 | rs111860462 | 14 | 82047636 | T | 0.0017 | 1.575 | 0.304 | 2.34E-07 |
| ebi-a-GCST90001698 | rs111867376 | 6 | 159962229 | A | 0.0026 | 206.9 | 34.55 | 2.34E-09 |
| ebi-a-GCST90001939 | rs111871083 | 1 | 27823957 | T | 0.1638 | 0.1574 | 0.03433 | 4.69E-06 |
| ebi-a-GCST90001758 | rs11187166 | 10 | 94516008 | T | 0.1993 | 0.1499 | 0.03164 | 2.25E-06 |
| ebi-a-GCST90001745 | rs11187166 | 10 | 94516008 | T | 0.1993 | 0.1756 | 0.03165 | 3.07E-08 |
| ebi-a-GCST90001757 | rs11187166 | 10 | 94516008 | T | 0.1993 | 0.1757 | 0.03163 | 2.97E-08 |
| ebi-a-GCST90001961 | rs11187515 | 10 | 95327429 | T | 0.1491 | -0.2152 | 0.03869 | 2.90E-08 |
| ebi-a-GCST90001495 | rs11188757 | 10 | 98205222 | C | 0.2213 | -0.2549 | 0.03174 | 1.33E-15 |
| ebi-a-GCST90001489 | rs11188757 | 10 | 98205222 | C | 0.2213 | -0.2494 | 0.03146 | 3.06E-15 |
| ebi-a-GCST90002032 | rs11188782 | 10 | 98236565 | G | 0.8461 | -0.2133 | 0.0383 | 2.80E-08 |
| ebi-a-GCST90001496 | rs11188950 | 10 | 98619863 | G | 0.0167 | 0.4852 | 0.103 | 2.59E-06 |
| ebi-a-GCST90002047 | rs111896566 | 16 | 20551336 | A | 0.0064 | -1.153 | 0.2513 | 4.92E-06 |
| ebi-a-GCST90001671 | rs111899271 | 10 | 70317419 | G | 0.161 | 0.1651 | 0.03519 | 2.82E-06 |
| ebi-a-GCST90001670 | rs111899271 | 10 | 70317419 | G | 0.161 | 0.1668 | 0.03518 | 2.22E-06 |
| ebi-a-GCST90001672 | rs111899271 | 10 | 70317419 | G | 0.1611 | 0.1764 | 0.03571 | 8.19E-07 |
| ebi-a-GCST90001698 | rs111902471 | 3 | 14459099 | G | 0.0021 | 237.6 | 38.71 | 9.39E-10 |
| ebi-a-GCST90001573 | rs111902980 | 1 | 157954450 | A | 0.0193 | -0.5021 | 0.09198 | 5.15E-08 |
| ebi-a-GCST90001571 | rs111902980 | 1 | 157954450 | A | 0.0193 | 0.4328 | 0.08768 | 8.35E-07 |
| ebi-a-GCST90001569 | rs111902980 | 1 | 157954450 | A | 0.0193 | 0.4732 | 0.08644 | 4.71E-08 |
| ebi-a-GCST90001570 | rs111902980 | 1 | 157954450 | A | 0.0193 | 0.5134 | 0.09207 | 2.65E-08 |
| ebi-a-GCST90001777 | rs111912558 | 17 | 44356694 | C | 0.203 | 0.1572 | 0.03429 | 4.68E-06 |
| ebi-a-GCST90002019 | rs111912592 | 10 | 10790518 | C | 0.036 | -0.4535 | 0.09652 | 2.85E-06 |
| ebi-a-GCST90002099 | rs111916169 | 5 | 78137524 | T | 0.0107 | 0.6182 | 0.1318 | 2.85E-06 |
| ebi-a-GCST90001947 | rs111918250 | 10 | 21637249 | C | 0.0098 | 0.8635 | 0.187 | 4.20E-06 |
| ebi-a-GCST90002082 | rs11192024 | 10 | 106126407 | T | 0.0267 | -0.34 | 0.07321 | 3.55E-06 |
| ebi-a-GCST90001980 | rs111929038 | 19 | 2660053 | T | 0.0021 | 1.257 | 0.2692 | 3.16E-06 |
| ebi-a-GCST90002026 | rs111931508 | 8 | 9938243 | T | 0.0014 | -1.823 | 0.3702 | 8.97E-07 |
| ebi-a-GCST90001480 | rs111941642 | 22 | 28079761 | T | 0.0144 | -0.476 | 0.09652 | 8.56E-07 |
| ebi-a-GCST90001528 | rs111944709 | 8 | 4886462 | C | 0.0135 | -0.6684 | 0.1454 | 4.58E-06 |
| ebi-a-GCST90001695 | rs111953852 | 14 | 84194540 | G | 0.0025 | 137.6 | 24.38 | 1.82E-08 |
| ebi-a-GCST90001592 | rs11197647 | 10 | 118143363 | T | 0.0754 | 0.2221 | 0.04476 | 7.32E-07 |
| ebi-a-GCST90001627 | rs11197647 | 10 | 118143363 | T | 0.0759 | 0.2373 | 0.04575 | 2.27E-07 |
| ebi-a-GCST90001697 | rs111978771 | 1 | 21025910 | C | 0.0042 | 4.735 | 0.9172 | 2.57E-07 |
| ebi-a-GCST90001695 | rs111978771 | 1 | 21025910 | C | 0.0043 | 123.8 | 19.64 | 3.21E-10 |
| ebi-a-GCST90001697 | rs111981818 | 4 | 2899648 | G | 0.0028 | 5.996 | 1.149 | 1.90E-07 |
| ebi-a-GCST90001695 | rs111981818 | 4 | 2899648 | G | 0.0028 | 133.9 | 24.58 | 5.41E-08 |
| ebi-a-GCST90002116 | rs111983490 | 6 | 32205324 | A | 0.2111 | -0.3007 | 0.03814 | 4.36E-15 |
| ebi-a-GCST90001574 | rs111983490 | 6 | 32205324 | A | 0.2107 | -0.2166 | 0.03665 | 3.74E-09 |
| ebi-a-GCST90001751 | rs111983490 | 6 | 32205324 | A | 0.2097 | 0.1748 | 0.03444 | 4.06E-07 |
| ebi-a-GCST90001799 | rs111986434 | 9 | 132142740 | A | 0.0411 | 0.3983 | 0.08057 | 8.38E-07 |
| ebi-a-GCST90001698 | rs112004249 | 12 | 82550260 | A | 0.0031 | 162.6 | 33.14 | 9.77E-07 |
| ebi-a-GCST90001700 | rs11200733 | 10 | 85685084 | A | 0.0039 | 5.664 | 1.165 | 1.21E-06 |
| ebi-a-GCST90002067 | rs11200863 | 10 | 85875791 | A | 0.1666 | -0.1689 | 0.03679 | 4.62E-06 |
| ebi-a-GCST90001488 | rs112009416 | 1 | 199163362 | C | 0.0089 | -0.6866 | 0.133 | 2.56E-07 |
| ebi-a-GCST90001536 | rs11201613 | 10 | 87172617 | A | 0.0144 | -0.4646 | 0.1007 | 4.12E-06 |
| ebi-a-GCST90001791 | rs112017455 | 3 | 157651153 | T | 0.019 | 0.449 | 0.09078 | 7.93E-07 |
| ebi-a-GCST90001850 | rs112025123 | 18 | 46992743 | C | 0.0185 | -0.4918 | 0.1005 | 1.05E-06 |
| ebi-a-GCST90001700 | rs11202534 | 10 | 89500755 | T | 0.001 | 13.13 | 2.792 | 2.66E-06 |
| ebi-a-GCST90001698 | rs11202534 | 10 | 89500755 | T | 0.001 | 387.4 | 65.92 | 4.58E-09 |
| ebi-a-GCST90001695 | rs112028149 | 4 | 60775910 | T | 0.0035 | 105.2 | 21.94 | 1.70E-06 |
| ebi-a-GCST90001532 | rs11203701 | 8 | 15256015 | A | 0.4631 | 0.1573 | 0.0336 | 3.04E-06 |
| ebi-a-GCST90001531 | rs11203701 | 8 | 15256015 | A | 0.4634 | 0.1574 | 0.03372 | 3.26E-06 |
| ebi-a-GCST90001423 | rs11203884 | 8 | 17516564 | T | 0.1643 | -0.1548 | 0.03269 | 2.27E-06 |
| ebi-a-GCST90001779 | rs112043339 | 3 | 174935606 | T | 0.0138 | -0.5414 | 0.1119 | 1.37E-06 |
| ebi-a-GCST90001794 | rs112043339 | 3 | 174935606 | T | 0.0138 | -0.5221 | 0.1123 | 3.43E-06 |
| ebi-a-GCST90001402 | rs11204752 | 1 | 150958133 | C | 0.5387 | 0.1189 | 0.02561 | 3.59E-06 |
| ebi-a-GCST90001698 | rs112057764 | 3 | 176439376 | A | 0.0343 | 48.23 | 9.695 | 6.86E-07 |
| ebi-a-GCST90001964 | rs112068187 | 2 | 34892862 | A | 0.0209 | -0.4558 | 0.09878 | 4.13E-06 |
| ebi-a-GCST90001612 | rs112072415 | 11 | 33183043 | A | 0.0462 | 0.275 | 0.05986 | 4.50E-06 |
| ebi-a-GCST90002077 | rs112085672 | 3 | 182309260 | C | 0.035 | -0.3599 | 0.07399 | 1.21E-06 |
| ebi-a-GCST90002108 | rs112096735 | 19 | 11733838 | T | 0.1013 | -0.286 | 0.06037 | 2.36E-06 |
| ebi-a-GCST90001893 | rs11210252 | 1 | 73919673 | A | 0.218 | 0.1525 | 0.03317 | 4.47E-06 |
| ebi-a-GCST90001698 | rs112111433 | 13 | 85569939 | A | 0.0119 | 82.56 | 16.61 | 7.03E-07 |
| ebi-a-GCST90001698 | rs112121527 | 12 | 119037578 | C | 0.0019 | 249.4 | 42.57 | 5.14E-09 |
| ebi-a-GCST90001871 | rs11212429 | 11 | 107761682 | T | 0.0064 | -1.345 | 0.256 | 1.73E-07 |
| ebi-a-GCST90001875 | rs11212429 | 11 | 107761682 | T | 0.0064 | -1.284 | 0.2502 | 3.32E-07 |
| ebi-a-GCST90001432 | rs112135308 | 16 | 86794463 | G | 0.0174 | 0.416 | 0.08833 | 2.57E-06 |
| ebi-a-GCST90001883 | rs11214436 | 11 | 112830782 | T | 0.3055 | 0.2713 | 0.02873 | 7.05E-21 |
| ebi-a-GCST90001884 | rs11214436 | 11 | 112830782 | T | 0.3055 | 0.3221 | 0.02878 | 1.59E-28 |
| ebi-a-GCST90001579 | rs112150611 | 1 | 18034211 | T | 0.0113 | 0.5241 | 0.1132 | 3.82E-06 |
| ebi-a-GCST90001966 | rs112157475 | 2 | 195118976 | T | 0.0993 | -0.2428 | 0.04919 | 8.42E-07 |
| ebi-a-GCST90002018 | rs112157475 | 2 | 195118976 | T | 0.0993 | -0.2381 | 0.04846 | 9.46E-07 |
| ebi-a-GCST90001908 | rs112171230 | 7 | 70434581 | T | 0.0536 | 0.2857 | 0.0618 | 3.96E-06 |
| ebi-a-GCST90001842 | rs112171230 | 7 | 70434581 | T | 0.0536 | 0.3132 | 0.06122 | 3.32E-07 |
| ebi-a-GCST90001464 | rs112186537 | 6 | 73397623 | T | 0.0075 | -0.6969 | 0.1444 | 1.45E-06 |
| ebi-a-GCST90001472 | rs112186537 | 6 | 73397623 | T | 0.0075 | -0.691 | 0.1441 | 1.69E-06 |
| ebi-a-GCST90001529 | rs11218704 | 11 | 122458787 | G | 0.0584 | 0.3401 | 0.06951 | 1.08E-06 |
| ebi-a-GCST90001863 | rs11219278 | 11 | 123627668 | A | 0.6244 | -0.1267 | 0.02769 | 4.91E-06 |
| ebi-a-GCST90001801 | rs11220465 | 11 | 126257779 | A | 0.0667 | -0.336 | 0.06884 | 1.14E-06 |
| ebi-a-GCST90001961 | rs112207505 | 16 | 87927291 | A | 0.0106 | 0.6315 | 0.1332 | 2.21E-06 |
| ebi-a-GCST90002017 | rs112216269 | 7 | 119847090 | A | 0.0196 | -0.4899 | 0.1052 | 3.39E-06 |
| ebi-a-GCST90002046 | rs11222974 | 11 | 132073382 | T | 0.0107 | -0.7691 | 0.1667 | 4.25E-06 |
| ebi-a-GCST90001816 | rs112236163 | 18 | 51428440 | G | 0.2246 | 0.1589 | 0.03072 | 2.42E-07 |
| ebi-a-GCST90001507 | rs112238613 | 11 | 110766468 | C | 0.0013 | 1.573 | 0.3324 | 2.32E-06 |
| ebi-a-GCST90001700 | rs112254351 | 5 | 169422728 | A | 4.00E-04 | 19.55 | 3.788 | 2.60E-07 |
| ebi-a-GCST90001689 | rs112258643 | 12 | 55672838 | T | 1.00E-04 | 3.271 | 0.6538 | 5.92E-07 |
| ebi-a-GCST90001688 | rs112258643 | 12 | 55672838 | T | 1.00E-04 | 3.436 | 0.6021 | 1.25E-08 |
| ebi-a-GCST90001514 | rs112258784 | 2 | 191015741 | G | 0.0102 | -0.7371 | 0.1606 | 4.75E-06 |
| ebi-a-GCST90002080 | rs112262410 | 3 | 134400604 | T | 0.0641 | -0.2418 | 0.05178 | 3.16E-06 |
| ebi-a-GCST90002082 | rs112262410 | 3 | 134400604 | T | 0.0641 | -0.2303 | 0.04813 | 1.79E-06 |
| ebi-a-GCST90001698 | rs112278099 | 18 | 5319739 | T | 0.0136 | 82.54 | 15.29 | 7.17E-08 |
| ebi-a-GCST90001688 | rs112280034 | 16 | 78252575 | G | NA | 4.67 | 0.9514 | 9.63E-07 |
| ebi-a-GCST90002074 | rs11228503 | 11 | 68879044 | T | 0.2172 | -0.1555 | 0.03171 | 9.84E-07 |
| ebi-a-GCST90001833 | rs112295585 | 4 | 55258914 | T | 0.0023 | -1.243 | 0.2597 | 1.79E-06 |
| ebi-a-GCST90001750 | rs11230344 | 11 | 60264409 | A | 0.6097 | -0.165 | 0.03378 | 1.13E-06 |
| ebi-a-GCST90001757 | rs11230624 | 11 | 60952231 | C | 0.1263 | -0.1823 | 0.03724 | 1.02E-06 |
| ebi-a-GCST90001752 | rs11230624 | 11 | 60952231 | C | 0.1263 | -0.1726 | 0.03735 | 3.94E-06 |
| ebi-a-GCST90001698 | rs112310272 | 15 | 26944526 | A | 0.0182 | 64.13 | 13.45 | 1.94E-06 |
| ebi-a-GCST90001467 | rs11231693 | 11 | 63862612 | A | 0.0554 | -0.2631 | 0.05451 | 1.45E-06 |
| ebi-a-GCST90001978 | rs11232360 | 11 | 80547943 | A | 0.0015 | 1.521 | 0.332 | 4.83E-06 |
| ebi-a-GCST90001695 | rs11232730 | 11 | 81281823 | A | 0.0283 | 40.36 | 7.773 | 2.20E-07 |
| ebi-a-GCST90001794 | rs112328981 | 13 | 34477079 | T | 3.00E-04 | 3.458 | 0.6771 | 3.43E-07 |
| ebi-a-GCST90001779 | rs112328981 | 13 | 34477079 | T | 3.00E-04 | 3.546 | 0.6803 | 1.96E-07 |
| ebi-a-GCST90001528 | rs112330535 | 8 | 97415887 | G | 0.0439 | -0.4089 | 0.08902 | 4.64E-06 |
| ebi-a-GCST90001527 | rs112332228 | 18 | 36935582 | T | 5.00E-04 | 3.054 | 0.6345 | 1.61E-06 |
| ebi-a-GCST90001697 | rs11233425 | 11 | 82732689 | A | 3.00E-04 | 23.77 | 3.62 | 5.93E-11 |
| ebi-a-GCST90001850 | rs11233873 | 11 | 83711222 | C | 0.0752 | 0.2377 | 0.05106 | 3.39E-06 |
| ebi-a-GCST90001804 | rs112339885 | 5 | 79389016 | C | 0.0755 | 0.2558 | 0.0472 | 6.32E-08 |
| ebi-a-GCST90001920 | rs112341592 | 3 | 87411172 | C | 0.0459 | 0.3453 | 0.06467 | 9.99E-08 |
| ebi-a-GCST90002042 | rs11234542 | 11 | 85803238 | G | 0.1143 | -0.2828 | 0.0577 | 1.05E-06 |
| ebi-a-GCST90001923 | rs112348392 | 11 | 47832118 | T | 0.0262 | -0.4045 | 0.08672 | 3.24E-06 |
| ebi-a-GCST90001698 | rs112357188 | 5 | 16118218 | A | 0.0053 | 119.6 | 24.42 | 1.01E-06 |
| ebi-a-GCST90001888 | rs112358299 | 10 | 95281076 | A | 0.0685 | 0.2671 | 0.05279 | 4.47E-07 |
| ebi-a-GCST90001570 | rs112361235 | 7 | 23599680 | T | 0.0162 | -0.445 | 0.09722 | 4.88E-06 |
| ebi-a-GCST90002045 | rs112370962 | 1 | 15693424 | C | 0.0116 | -0.8187 | 0.1725 | 2.27E-06 |
| ebi-a-GCST90001890 | rs112388922 | 12 | 106627038 | A | 0.3296 | -0.137 | 0.02967 | 4.01E-06 |
| ebi-a-GCST90002017 | rs112391343 | 3 | 165060342 | G | 0.0772 | 0.2617 | 0.05295 | 8.13E-07 |
| ebi-a-GCST90001960 | rs112393619 | 7 | 21800888 | A | 0.0704 | 0.2336 | 0.05083 | 4.50E-06 |
| ebi-a-GCST90001652 | rs112405229 | 1 | 240584083 | A | 0.039 | 0.2941 | 0.06403 | 4.52E-06 |
| ebi-a-GCST90001760 | rs112405902 | 6 | 31809504 | A | 0.1069 | 0.2604 | 0.0461 | 1.75E-08 |
| ebi-a-GCST90001740 | rs112417310 | 1 | 206855483 | A | 0.0201 | 0.4952 | 0.0895 | 3.38E-08 |
| ebi-a-GCST90001736 | rs112417310 | 1 | 206855483 | A | 0.0201 | 0.5116 | 0.08936 | 1.12E-08 |
| ebi-a-GCST90001741 | rs112417310 | 1 | 206855483 | A | 0.0201 | 0.5195 | 0.08901 | 5.79E-09 |
| ebi-a-GCST90001725 | rs112417310 | 1 | 206855483 | A | 0.0201 | 0.5237 | 0.08866 | 3.80E-09 |
| ebi-a-GCST90001735 | rs112417310 | 1 | 206855483 | A | 0.0201 | 0.5373 | 0.08896 | 1.70E-09 |
| ebi-a-GCST90001723 | rs112417310 | 1 | 206855483 | A | 0.0201 | 0.5474 | 0.08918 | 9.28E-10 |
| ebi-a-GCST90001730 | rs112417310 | 1 | 206855483 | A | 0.0201 | 0.5594 | 0.089 | 3.65E-10 |
| ebi-a-GCST90001729 | rs112417310 | 1 | 206855483 | A | 0.0201 | 0.6504 | 0.08983 | 5.43E-13 |
| ebi-a-GCST90001874 | rs112418980 | 2 | 34001527 | T | 0.0405 | 0.479 | 0.1043 | 4.80E-06 |
| ebi-a-GCST90001872 | rs112421400 | 6 | 28205826 | T | 0.1997 | -0.2471 | 0.05362 | 4.47E-06 |
| ebi-a-GCST90001820 | rs112421400 | 6 | 28205826 | T | 0.2101 | -0.1982 | 0.03505 | 1.70E-08 |
| ebi-a-GCST90001401 | rs112432445 | 9 | 4162626 | A | 0.0528 | 0.2796 | 0.05583 | 5.77E-07 |
| ebi-a-GCST90002012 | rs112450242 | 17 | 75274660 | T | 0.0035 | -1.257 | 0.2349 | 9.19E-08 |
| ebi-a-GCST90001996 | rs112450242 | 17 | 75274660 | T | 0.0035 | -1.107 | 0.2399 | 4.09E-06 |
| ebi-a-GCST90001790 | rs11245200 | 10 | 126182752 | T | 0.1303 | 0.1664 | 0.03537 | 2.65E-06 |
| ebi-a-GCST90001785 | rs11245200 | 10 | 126182752 | T | 0.1303 | 0.1667 | 0.03594 | 3.65E-06 |
| ebi-a-GCST90001786 | rs11245200 | 10 | 126182752 | T | 0.1304 | 0.1669 | 0.0356 | 2.86E-06 |
| ebi-a-GCST90001627 | rs112455120 | 16 | 10380785 | T | 0.0302 | 0.3535 | 0.07143 | 7.79E-07 |
| ebi-a-GCST90001628 | rs112455120 | 16 | 10380785 | T | 0.03 | 0.3608 | 0.06884 | 1.69E-07 |
| ebi-a-GCST90001629 | rs112455120 | 16 | 10380785 | T | 0.03 | 0.3662 | 0.06983 | 1.66E-07 |
| ebi-a-GCST90001899 | rs11246934 | 12 | 132036029 | A | 0.043 | -0.3054 | 0.06395 | 1.88E-06 |
| ebi-a-GCST90001689 | rs112471936 | 4 | 83970127 | A | NA | 8.759 | 1.876 | 3.13E-06 |
| ebi-a-GCST90001688 | rs112471936 | 4 | 83970127 | A | NA | 9.954 | 1.725 | 8.55E-09 |
| ebi-a-GCST90001666 | rs112471936 | 4 | 83970127 | A | NA | 10.19 | 2.198 | 3.72E-06 |
| ebi-a-GCST90001664 | rs112471936 | 4 | 83970127 | A | NA | 10.53 | 2.301 | 4.91E-06 |
| ebi-a-GCST90001807 | rs112481165 | 1 | 247244688 | A | 0.1078 | 0.1798 | 0.03883 | 3.75E-06 |
| ebi-a-GCST90001450 | rs11249570 | 5 | 177033794 | A | 0.4581 | -0.1525 | 0.02632 | 7.66E-09 |
| ebi-a-GCST90001451 | rs11249570 | 5 | 177033794 | A | 0.4578 | -0.1315 | 0.02634 | 6.30E-07 |
| ebi-a-GCST90001958 | rs112496462 | 6 | 34155071 | A | 0.0014 | -1.603 | 0.3472 | 4.03E-06 |
| ebi-a-GCST90001467 | rs112496823 | 17 | 33829583 | C | 0.1771 | 0.1585 | 0.03308 | 1.72E-06 |
| ebi-a-GCST90001466 | rs112496823 | 17 | 33829583 | C | 0.1768 | 0.1603 | 0.03305 | 1.29E-06 |
| ebi-a-GCST90001612 | rs112505246 | 6 | 100937868 | C | 0.0785 | 0.2425 | 0.04608 | 1.50E-07 |
| ebi-a-GCST90001597 | rs112505246 | 6 | 100937868 | C | 0.0785 | 0.2492 | 0.04564 | 5.10E-08 |
| ebi-a-GCST90001698 | rs112517700 | 10 | 91013835 | A | 0.0114 | 95.85 | 16.51 | 7.06E-09 |
| ebi-a-GCST90001568 | rs112522622 | 16 | 51946807 | T | 0.0959 | 0.1694 | 0.0351 | 1.45E-06 |
| ebi-a-GCST90001698 | rs112529428 | 12 | 129340399 | T | 0.0015 | 345.5 | 42.75 | 8.74E-16 |
| ebi-a-GCST90001442 | rs112542522 | 2 | 4031340 | A | 0.0082 | 0.6143 | 0.1326 | 3.73E-06 |
| ebi-a-GCST90001502 | rs112553628 | 2 | 38077329 | G | 0.0352 | -0.2867 | 0.0606 | 2.31E-06 |
| ebi-a-GCST90001482 | rs112553628 | 2 | 38077329 | G | 0.0352 | 0.2882 | 0.06287 | 4.73E-06 |
| ebi-a-GCST90001481 | rs112553628 | 2 | 38077329 | G | 0.0352 | 0.2951 | 0.06128 | 1.54E-06 |
| ebi-a-GCST90001697 | rs112568609 | 15 | 27818525 | T | 0.0076 | 3.316 | 0.7127 | 3.40E-06 |
| ebi-a-GCST90001695 | rs112568609 | 15 | 27818525 | T | 0.0076 | 87.63 | 15.24 | 9.60E-09 |
| ebi-a-GCST90001503 | rs11256966 | 10 | 6185310 | G | 0.3848 | -0.1567 | 0.02626 | 2.64E-09 |
| ebi-a-GCST90001411 | rs112570989 | 12 | 2866164 | T | 0.0149 | -0.4808 | 0.1031 | 3.24E-06 |
| ebi-a-GCST90001406 | rs112570989 | 12 | 2866164 | T | 0.0149 | -0.4739 | 0.1022 | 3.63E-06 |
| ebi-a-GCST90001408 | rs112570989 | 12 | 2866164 | T | 0.0149 | 0.4852 | 0.1023 | 2.19E-06 |
| ebi-a-GCST90001688 | rs112571466 | 7 | 111704231 | G | 1.00E-04 | 3.787 | 0.6773 | 2.44E-08 |
| ebi-a-GCST90001896 | rs112581826 | 2 | 204650296 | A | 0.1281 | -0.2422 | 0.04219 | 1.04E-08 |
| ebi-a-GCST90001558 | rs112587592 | 5 | 104220406 | C | 0.0045 | -0.8184 | 0.1784 | 4.64E-06 |
| ebi-a-GCST90002117 | rs11260062 | 19 | 8214970 | C | 0.2986 | 0.1397 | 0.02994 | 3.21E-06 |
| ebi-a-GCST90001698 | rs112604811 | 19 | 46587710 | T | 4.00E-04 | 1015 | 81.57 | 8.89E-35 |
| ebi-a-GCST90001530 | rs112611150 | 7 | 186897 | T | 0.0652 | -0.3141 | 0.06753 | 3.55E-06 |
| ebi-a-GCST90001982 | rs112619771 | 7 | 143427146 | C | 0.044 | 0.2878 | 0.06088 | 2.36E-06 |
| ebi-a-GCST90001688 | rs112637846 | 6 | 149114553 | A | 3.00E-04 | 2.345 | 0.4689 | 5.96E-07 |
| ebi-a-GCST90001666 | rs112637846 | 6 | 149114553 | A | 3.00E-04 | 3.334 | 0.5986 | 2.76E-08 |
| ebi-a-GCST90001664 | rs112637846 | 6 | 149114553 | A | 3.00E-04 | 3.37 | 0.6241 | 7.14E-08 |
| ebi-a-GCST90002091 | rs112645067 | 15 | 99970786 | G | 0.0761 | -0.3328 | 0.06908 | 1.59E-06 |
| ebi-a-GCST90001651 | rs11265198 | 1 | 159420307 | A | 0.9759 | 0.3966 | 0.08035 | 8.35E-07 |
| ebi-a-GCST90001655 | rs112667234 | 5 | 68594062 | C | 0.0846 | 0.1872 | 0.04068 | 4.35E-06 |
| ebi-a-GCST90001749 | rs112695918 | 1 | 1104584 | C | 0.0046 | 1.187 | 0.2474 | 1.75E-06 |
| ebi-a-GCST90002001 | rs112698696 | 17 | 25745048 | A | 0.041 | 0.2831 | 0.06151 | 4.33E-06 |
| ebi-a-GCST90001965 | rs112698900 | 2 | 175649304 | T | 0.0561 | 0.2844 | 0.05928 | 1.69E-06 |
| ebi-a-GCST90001553 | rs112725843 | 16 | 28238440 | T | 0.1326 | 0.1191 | 0.02489 | 1.79E-06 |
| ebi-a-GCST90001977 | rs112728592 | 2 | 179019068 | T | 0.0479 | 0.2828 | 0.06104 | 3.75E-06 |
| ebi-a-GCST90002031 | rs112736587 | 1 | 68140344 | G | 3.00E-04 | 3.142 | 0.6604 | 2.05E-06 |
| ebi-a-GCST90001973 | rs112736938 | 18 | 64359675 | C | 0.0194 | 0.4394 | 0.09464 | 3.58E-06 |
| ebi-a-GCST90001916 | rs112736938 | 18 | 64359675 | C | 0.0194 | 0.4519 | 0.09778 | 3.96E-06 |
| ebi-a-GCST90001987 | rs112755578 | 1 | 149716369 | G | 0.0758 | -0.5111 | 0.04896 | 3.75E-25 |
| ebi-a-GCST90002006 | rs112755578 | 1 | 149716369 | G | 0.0758 | -0.446 | 0.04948 | 3.16E-19 |
| ebi-a-GCST90001697 | rs112774387 | 2 | 100757118 | G | 0.0013 | 7.789 | 1.615 | 1.47E-06 |
| ebi-a-GCST90001696 | rs112774387 | 2 | 100757118 | G | 0.0013 | 20.96 | 3.949 | 1.18E-07 |
| ebi-a-GCST90001688 | rs112779860 | 8 | 127602530 | A | 4.00E-04 | 2.492 | 0.5273 | 2.39E-06 |
| ebi-a-GCST90001775 | rs112782756 | 13 | 34506073 | G | 3.00E-04 | 3.476 | 0.6767 | 2.94E-07 |
| ebi-a-GCST90001695 | rs112785730 | 2 | 127831527 | A | 0.0026 | 115.7 | 24.44 | 2.32E-06 |
| ebi-a-GCST90001698 | rs112785730 | 2 | 127831527 | A | 0.0026 | 206.8 | 32.42 | 2.03E-10 |
| ebi-a-GCST90001520 | rs112790132 | 3 | 115423600 | A | 0.0118 | -0.7381 | 0.1481 | 6.82E-07 |
| ebi-a-GCST90001726 | rs112802874 | 10 | 89640021 | G | 0.0207 | 0.429 | 0.08985 | 1.87E-06 |
| ebi-a-GCST90001949 | rs112807165 | 20 | 38092873 | G | 0.0324 | 0.4861 | 0.1052 | 4.12E-06 |
| ebi-a-GCST90001939 | rs112819427 | 2 | 38588385 | G | 0.1175 | -0.1837 | 0.03952 | 3.47E-06 |
| ebi-a-GCST90001580 | rs112831211 | 4 | 182944203 | G | 0.0415 | 0.2999 | 0.06287 | 1.92E-06 |
| ebi-a-GCST90001398 | rs112839090 | 12 | 119727983 | A | 0.1972 | -0.1468 | 0.03096 | 2.19E-06 |
| ebi-a-GCST90001601 | rs112839163 | 20 | 14708666 | A | 0.0131 | -0.5037 | 0.1017 | 7.70E-07 |
| ebi-a-GCST90001695 | rs112839187 | 2 | 238422350 | A | 3.00E-04 | 444.1 | 76.59 | 7.31E-09 |
| ebi-a-GCST90001723 | rs112846834 | 17 | 7725660 | A | 0.0563 | 0.249 | 0.05331 | 3.10E-06 |
| ebi-a-GCST90001437 | rs112850074 | 14 | 103229069 | G | 0.031 | 0.3445 | 0.07141 | 1.46E-06 |
| ebi-a-GCST90001695 | rs112857714 | 6 | 148462118 | T | 9.00E-04 | 286.9 | 45.35 | 2.84E-10 |
| ebi-a-GCST90001980 | rs112876754 | 12 | 54934521 | G | 0.0375 | 0.3229 | 0.06611 | 1.08E-06 |
| ebi-a-GCST90002109 | rs112878149 | 4 | 184500476 | C | 0.0352 | -0.4619 | 0.09902 | 3.35E-06 |
| ebi-a-GCST90001698 | rs112880431 | 6 | 130091902 | A | 0.0015 | 312.7 | 46.04 | 1.30E-11 |
| ebi-a-GCST90001904 | rs112891649 | 19 | 23466054 | A | 0.0698 | 0.2911 | 0.05373 | 6.54E-08 |
| ebi-a-GCST90001413 | rs112898282 | 10 | 28462184 | C | 0.0152 | 0.4712 | 0.1007 | 2.97E-06 |
| ebi-a-GCST90001757 | rs112898596 | 22 | 42261360 | T | 0.0505 | 0.2709 | 0.0562 | 1.49E-06 |
| ebi-a-GCST90001900 | rs112900587 | 2 | 195082880 | A | 0.0831 | -0.245 | 0.05172 | 2.27E-06 |
| ebi-a-GCST90001428 | rs112904733 | 1 | 214958496 | T | 0.0956 | 0.1953 | 0.04221 | 3.85E-06 |
| ebi-a-GCST90001698 | rs112973846 | 5 | 128365521 | G | 0.0012 | 386.6 | 51.06 | 4.77E-14 |
| ebi-a-GCST90001733 | rs112978597 | 1 | 12881809 | G | 0.06 | 0.2426 | 0.05151 | 2.58E-06 |
| ebi-a-GCST90001929 | rs112984211 | 6 | 32617889 | T | 0.2454 | 0.3196 | 0.05923 | 7.40E-08 |
| ebi-a-GCST90001502 | rs113000944 | 20 | 48836592 | T | 0.063 | -0.2336 | 0.04699 | 6.94E-07 |
| ebi-a-GCST90001722 | rs113004536 | 22 | 18923443 | A | 0.072 | -0.2154 | 0.04611 | 3.10E-06 |
| ebi-a-GCST90001698 | rs113007178 | 13 | 40914557 | A | 0.0018 | 289.8 | 41.55 | 3.67E-12 |
| ebi-a-GCST90002080 | rs113026874 | 22 | 30400641 | T | 0.0389 | -0.3239 | 0.06934 | 3.11E-06 |
| ebi-a-GCST90001540 | rs113039817 | 16 | 7401399 | T | 0.014 | -0.4664 | 0.1018 | 4.76E-06 |
| ebi-a-GCST90001688 | rs113046419 | 2 | 66598636 | C | 3.00E-04 | 2.579 | 0.5321 | 1.31E-06 |
| ebi-a-GCST90001976 | rs113047054 | 9 | 12210926 | C | 0.0567 | -0.2743 | 0.05621 | 1.11E-06 |
| ebi-a-GCST90001981 | rs113063605 | 1 | 161246613 | G | 0.1108 | 0.1912 | 0.03935 | 1.23E-06 |
| ebi-a-GCST90001985 | rs113063605 | 1 | 161246613 | G | 0.1106 | 0.2178 | 0.03952 | 3.83E-08 |
| ebi-a-GCST90001781 | rs113066333 | 2 | 28889318 | A | 0.0572 | -0.3375 | 0.07297 | 4.00E-06 |
| ebi-a-GCST90001782 | rs113066333 | 2 | 28889318 | A | 0.0574 | -0.3274 | 0.07081 | 4.04E-06 |
| ebi-a-GCST90001661 | rs113075410 | 6 | 101435600 | G | 0.0541 | 0.3121 | 0.05748 | 6.06E-08 |
| ebi-a-GCST90001687 | rs113098117 | 7 | 54014110 | G | 0.0072 | -0.6929 | 0.1468 | 2.46E-06 |
| ebi-a-GCST90001666 | rs113098887 | 6 | 84554072 | A | 1.00E-04 | 3.627 | 0.7884 | 4.38E-06 |
| ebi-a-GCST90002008 | rs113111100 | 10 | 129087440 | A | 0.0165 | -0.4412 | 0.09405 | 2.82E-06 |
| ebi-a-GCST90001688 | rs113116201 | 1 | 198830942 | C | 0.0047 | 0.6358 | 0.1227 | 2.30E-07 |
| ebi-a-GCST90001939 | rs113116201 | 1 | 198830942 | C | 0.0044 | 0.9566 | 0.1853 | 2.59E-07 |
| ebi-a-GCST90001943 | rs113116201 | 1 | 198830942 | C | 0.0044 | 1.068 | 0.1846 | 8.02E-09 |
| ebi-a-GCST90001942 | rs113116201 | 1 | 198830942 | C | 0.0044 | 1.189 | 0.186 | 1.82E-10 |
| ebi-a-GCST90001941 | rs113116201 | 1 | 198830942 | C | 0.0044 | 1.214 | 0.1863 | 8.43E-11 |
| ebi-a-GCST90001698 | rs113122796 | 8 | 142876352 | G | 0.001 | 403.5 | 51.12 | 3.95E-15 |
| ebi-a-GCST90002071 | rs113138131 | 2 | 203545127 | T | 0.5045 | 0.2775 | 0.02889 | 1.56E-21 |
| ebi-a-GCST90001637 | rs113145300 | 6 | 92830899 | G | 0.0282 | 0.3542 | 0.07489 | 2.34E-06 |
| ebi-a-GCST90001941 | rs113165806 | 7 | 96426791 | A | 0.1263 | 0.1842 | 0.038 | 1.31E-06 |
| ebi-a-GCST90002087 | rs113167560 | 16 | 51444521 | G | 0.0393 | -0.3213 | 0.06762 | 2.12E-06 |
| ebi-a-GCST90001527 | rs113181189 | 3 | 2089038 | G | 0.021 | 0.5178 | 0.1095 | 2.46E-06 |
| ebi-a-GCST90001605 | rs113185319 | 7 | 100618701 | T | 0.0797 | -0.2011 | 0.04397 | 4.93E-06 |
| ebi-a-GCST90001698 | rs113199364 | 17 | 75157900 | T | 9.00E-04 | 299.9 | 60.1 | 6.32E-07 |
| ebi-a-GCST90001842 | rs113208949 | 20 | 9979604 | T | 0.0112 | 0.6287 | 0.1285 | 1.06E-06 |
| ebi-a-GCST90001985 | rs113231250 | 8 | 107146016 | A | 0.0015 | -1.464 | 0.316 | 3.70E-06 |
| ebi-a-GCST90001541 | rs113243185 | 6 | 32585071 | C | 0.1996 | -0.2104 | 0.03639 | 8.06E-09 |
| ebi-a-GCST90001544 | rs113243185 | 6 | 32585071 | C | 0.1996 | 0.2134 | 0.03693 | 8.19E-09 |
| ebi-a-GCST90002081 | rs113243185 | 6 | 32585071 | C | 0.2014 | 0.2404 | 0.03733 | 1.39E-10 |
| ebi-a-GCST90001546 | rs113243185 | 6 | 32585071 | C | 0.1996 | 0.2773 | 0.03632 | 2.88E-14 |
| ebi-a-GCST90001547 | rs113243185 | 6 | 32585071 | C | 0.1996 | 0.2854 | 0.03605 | 3.28E-15 |
| ebi-a-GCST90001970 | rs113248693 | 1 | 157464822 | G | 0.0066 | -0.7673 | 0.1636 | 2.84E-06 |
| ebi-a-GCST90002029 | rs113261188 | 12 | 107834932 | A | 0.0393 | -0.3478 | 0.07042 | 8.32E-07 |
| ebi-a-GCST90001991 | rs113337372 | 2 | 121677690 | A | 0.0347 | -0.3163 | 0.06905 | 4.80E-06 |
| ebi-a-GCST90001506 | rs113339346 | 2 | 42801648 | T | 0.0634 | 0.2544 | 0.05459 | 3.27E-06 |
| ebi-a-GCST90001867 | rs113348527 | 21 | 24255270 | A | 0.0202 | 0.4337 | 0.09349 | 3.65E-06 |
| ebi-a-GCST90001861 | rs113348527 | 21 | 24255270 | A | 0.0202 | 0.4416 | 0.09394 | 2.71E-06 |
| ebi-a-GCST90001698 | rs113369914 | 20 | 21420694 | T | 0.0021 | 214.8 | 37.5 | 1.10E-08 |
| ebi-a-GCST90001752 | rs113371046 | 1 | 17595034 | A | 0.0639 | 0.2316 | 0.05055 | 4.76E-06 |
| ebi-a-GCST90001759 | rs113371046 | 1 | 17595034 | A | 0.0639 | 0.2324 | 0.05068 | 4.65E-06 |
| ebi-a-GCST90001762 | rs113371046 | 1 | 17595034 | A | 0.0638 | 0.2331 | 0.05062 | 4.28E-06 |
| ebi-a-GCST90001747 | rs113371046 | 1 | 17595034 | A | 0.0639 | 0.2407 | 0.05062 | 2.06E-06 |
| ebi-a-GCST90001500 | rs113376235 | 16 | 75514711 | A | 0.0278 | 0.352 | 0.07659 | 4.45E-06 |
| ebi-a-GCST90001395 | rs113398446 | 6 | 161871755 | C | 0.1687 | 0.1793 | 0.03256 | 3.91E-08 |
| ebi-a-GCST90001672 | rs113398680 | 3 | 145339577 | A | 0.0032 | 1.055 | 0.2209 | 1.86E-06 |
| ebi-a-GCST90002088 | rs113404848 | 16 | 48040615 | G | 0.063 | 0.2651 | 0.0556 | 1.95E-06 |
| ebi-a-GCST90002087 | rs113404848 | 16 | 48040615 | G | 0.063 | 0.2758 | 0.05575 | 7.96E-07 |
| ebi-a-GCST90001698 | rs113411752 | 17 | 35489333 | G | 0.0045 | 146.5 | 27.72 | 1.33E-07 |
| ebi-a-GCST90001926 | rs113425015 | 17 | 58951391 | G | 0.0421 | -0.3296 | 0.06817 | 1.40E-06 |
| ebi-a-GCST90001695 | rs113432575 | 21 | 30128360 | T | 6.00E-04 | 345.8 | 57.03 | 1.48E-09 |
| ebi-a-GCST90001502 | rs113435341 | 1 | 198151688 | G | 0.0032 | -2.621 | 0.1957 | 6.33E-40 |
| ebi-a-GCST90001493 | rs113435341 | 1 | 198151688 | G | 0.0032 | -2.54 | 0.2093 | 3.14E-33 |
| ebi-a-GCST90001494 | rs113435341 | 1 | 198151688 | G | 0.0032 | -2.18 | 0.2206 | 9.94E-23 |
| ebi-a-GCST90001503 | rs113435341 | 1 | 198151688 | G | 0.0032 | -2.11 | 0.2195 | 1.31E-21 |
| ebi-a-GCST90001492 | rs113435341 | 1 | 198151688 | G | 0.0032 | -2.032 | 0.2282 | 8.69E-19 |
| ebi-a-GCST90001501 | rs113435341 | 1 | 198151688 | G | 0.0032 | -2.018 | 0.2282 | 1.47E-18 |
| ebi-a-GCST90001510 | rs113435341 | 1 | 198151688 | G | 0.0032 | -1.681 | 0.2295 | 3.03E-13 |
| ebi-a-GCST90001486 | rs113435341 | 1 | 198151688 | G | 0.0032 | -1.414 | 0.2277 | 5.88E-10 |
| ebi-a-GCST90001548 | rs113435341 | 1 | 198151688 | G | 0.0032 | -1.226 | 0.2117 | 7.49E-09 |
| ebi-a-GCST90001546 | rs113435341 | 1 | 198151688 | G | 0.0032 | 1.234 | 0.2235 | 3.65E-08 |
| ebi-a-GCST90001485 | rs113435341 | 1 | 198151688 | G | 0.0032 | 2.267 | 0.2171 | 3.79E-25 |
| ebi-a-GCST90001499 | rs113435341 | 1 | 198151688 | G | 0.0032 | 2.569 | 0.2103 | 1.28E-33 |
| ebi-a-GCST90001616 | rs113438136 | 3 | 86907637 | C | 0.0115 | 0.544 | 0.1166 | 3.16E-06 |
| ebi-a-GCST90001617 | rs113438136 | 3 | 86907637 | C | 0.0115 | 0.5568 | 0.1161 | 1.68E-06 |
| ebi-a-GCST90002030 | rs113440812 | 10 | 100221844 | A | 0.0228 | -0.4713 | 0.09273 | 3.96E-07 |
| ebi-a-GCST90001433 | rs113442137 | 1 | 160254955 | A | 0.0472 | -0.2734 | 0.0574 | 1.97E-06 |
| ebi-a-GCST90001688 | rs113447229 | 9 | 105346840 | C | 1.00E-04 | 4.765 | 0.8459 | 1.92E-08 |
| ebi-a-GCST90001448 | rs113451095 | 17 | 9975038 | C | 0.0472 | -0.2994 | 0.06307 | 2.17E-06 |
| ebi-a-GCST90001617 | rs113457024 | 18 | 51480039 | C | 0.1148 | 0.1762 | 0.03828 | 4.28E-06 |
| ebi-a-GCST90001616 | rs113457024 | 18 | 51480039 | C | 0.1148 | 0.1769 | 0.03843 | 4.30E-06 |
| ebi-a-GCST90001941 | rs113465498 | 1 | 59362133 | A | 0.2563 | 0.1509 | 0.02892 | 1.92E-07 |
| ebi-a-GCST90001942 | rs113465498 | 1 | 59362133 | A | 0.2563 | 0.1559 | 0.02876 | 6.33E-08 |
| ebi-a-GCST90001985 | rs113466200 | 19 | 54250532 | T | 0.0631 | 0.2378 | 0.05193 | 4.81E-06 |
| ebi-a-GCST90001794 | rs113492859 | 4 | 185217641 | T | 0.0879 | 0.2021 | 0.04381 | 4.10E-06 |
| ebi-a-GCST90001569 | rs113500908 | 6 | 100774499 | T | 0.0449 | 0.2901 | 0.05795 | 5.82E-07 |
| ebi-a-GCST90001571 | rs113500908 | 6 | 100774499 | T | 0.0449 | 0.3476 | 0.05835 | 2.83E-09 |
| ebi-a-GCST90001977 | rs113508249 | 4 | 82455099 | G | 0.0402 | 0.3176 | 0.06604 | 1.58E-06 |
| ebi-a-GCST90001698 | rs113513225 | 2 | 192441892 | A | 0.0078 | 100.5 | 20.04 | 5.55E-07 |
| ebi-a-GCST90001707 | rs113530150 | 22 | 40567196 | T | 0.0806 | -0.3995 | 0.06471 | 8.16E-10 |
| ebi-a-GCST90001711 | rs113530150 | 22 | 40567196 | T | 0.0742 | -0.3778 | 0.04889 | 1.40E-14 |
| ebi-a-GCST90001710 | rs113530150 | 22 | 40567196 | T | 0.0742 | -0.3712 | 0.04904 | 4.73E-14 |
| ebi-a-GCST90001797 | rs113532966 | 2 | 50627626 | C | 0.0846 | 0.1937 | 0.04232 | 4.87E-06 |
| ebi-a-GCST90001519 | rs113548770 | 18 | 70222212 | G | 0.0019 | -1.707 | 0.3645 | 3.02E-06 |
| ebi-a-GCST90001698 | rs113555953 | 4 | 97898706 | A | 0.0139 | 76.89 | 15.6 | 8.71E-07 |
| ebi-a-GCST90001697 | rs113573055 | 16 | 15724919 | A | 0.0239 | 1.889 | 0.395 | 1.80E-06 |
| ebi-a-GCST90001961 | rs113594239 | 10 | 98103215 | T | 0.037 | -0.6028 | 0.07314 | 2.54E-16 |
| ebi-a-GCST90001700 | rs113598754 | 18 | 9944924 | G | 9.00E-04 | 11.3 | 2.457 | 4.39E-06 |
| ebi-a-GCST90001735 | rs113612962 | 22 | 43167446 | C | 0.0402 | -0.3441 | 0.06242 | 3.77E-08 |
| ebi-a-GCST90001740 | rs113612962 | 22 | 43167446 | C | 0.0402 | -0.3426 | 0.06276 | 5.13E-08 |
| ebi-a-GCST90001736 | rs113612962 | 22 | 43167446 | C | 0.0402 | -0.313 | 0.0628 | 6.53E-07 |
| ebi-a-GCST90001733 | rs113612962 | 22 | 43167446 | C | 0.0402 | -0.3129 | 0.06277 | 6.47E-07 |
| ebi-a-GCST90001738 | rs113612962 | 22 | 43167446 | C | 0.0402 | -0.3091 | 0.06276 | 8.80E-07 |
| ebi-a-GCST90001723 | rs113612962 | 22 | 43167446 | C | 0.0402 | -0.3031 | 0.06273 | 1.41E-06 |
| ebi-a-GCST90001724 | rs113612962 | 22 | 43167446 | C | 0.0402 | -0.2991 | 0.06296 | 2.11E-06 |
| ebi-a-GCST90001732 | rs113612962 | 22 | 43167446 | C | 0.0402 | -0.2905 | 0.06279 | 3.85E-06 |
| ebi-a-GCST90001698 | rs113677798 | 7 | 78657809 | G | 0.0114 | 85.98 | 16.13 | 1.05E-07 |
| ebi-a-GCST90001515 | rs113696385 | 2 | 176971968 | T | 0.0293 | -0.5264 | 0.1007 | 1.90E-07 |
| ebi-a-GCST90001698 | rs113698814 | 1 | 207678128 | G | 0.0037 | 189.8 | 29.08 | 7.67E-11 |
| ebi-a-GCST90001698 | rs113699161 | 14 | 58571589 | A | 0.0015 | 337.1 | 43.36 | 9.91E-15 |
| ebi-a-GCST90001558 | rs113702891 | 1 | 9586393 | A | 0.1926 | -0.1459 | 0.03145 | 3.61E-06 |
| ebi-a-GCST90001862 | rs113707507 | 14 | 78276958 | G | 0.1062 | 0.2157 | 0.04517 | 1.88E-06 |
| ebi-a-GCST90001856 | rs113711222 | 19 | 22042048 | A | 0.0624 | 0.2615 | 0.0566 | 4.02E-06 |
| ebi-a-GCST90001804 | rs113711447 | 9 | 96510568 | G | 0.0734 | -0.2355 | 0.04702 | 5.72E-07 |
| ebi-a-GCST90001695 | rs113759128 | 1 | 28458334 | T | 0.0065 | 75.56 | 16.06 | 2.63E-06 |
| ebi-a-GCST90001851 | rs113763504 | 10 | 81110536 | A | 0.0058 | -0.8245 | 0.1754 | 2.72E-06 |
| ebi-a-GCST90001861 | rs113763504 | 10 | 81110536 | A | 0.0058 | -0.8066 | 0.1737 | 3.58E-06 |
| ebi-a-GCST90001520 | rs113771439 | 18 | 5326964 | A | 0.2279 | 0.1922 | 0.03902 | 9.12E-07 |
| ebi-a-GCST90002110 | rs113773853 | 22 | 39607103 | C | 9.00E-04 | 2.611 | 0.5424 | 1.61E-06 |
| ebi-a-GCST90001681 | rs113792557 | 7 | 63496409 | C | 0.1102 | 0.2098 | 0.0421 | 6.62E-07 |
| ebi-a-GCST90001452 | rs113800324 | 12 | 69766606 | T | 0.05 | 0.3099 | 0.05911 | 1.68E-07 |
| ebi-a-GCST90001726 | rs113812519 | 1 | 207276336 | G | 0.0185 | 0.426 | 0.0915 | 3.34E-06 |
| ebi-a-GCST90001740 | rs113812519 | 1 | 207276336 | G | 0.0185 | 0.4305 | 0.09223 | 3.16E-06 |
| ebi-a-GCST90001735 | rs113812519 | 1 | 207276336 | G | 0.0185 | 0.4328 | 0.09174 | 2.47E-06 |
| ebi-a-GCST90001737 | rs113812519 | 1 | 207276336 | G | 0.0185 | 0.4972 | 0.09126 | 5.41E-08 |
| ebi-a-GCST90001723 | rs113812519 | 1 | 207276336 | G | 0.0185 | 0.4979 | 0.09189 | 6.38E-08 |
| ebi-a-GCST90001736 | rs113812519 | 1 | 207276336 | G | 0.0185 | 0.4995 | 0.09201 | 6.04E-08 |
| ebi-a-GCST90001741 | rs113812519 | 1 | 207276336 | G | 0.0185 | 0.5095 | 0.09165 | 2.91E-08 |
| ebi-a-GCST90001725 | rs113812519 | 1 | 207276336 | G | 0.0185 | 0.5314 | 0.09132 | 6.42E-09 |
| ebi-a-GCST90001738 | rs113812519 | 1 | 207276336 | G | 0.0185 | 0.5404 | 0.0918 | 4.29E-09 |
| ebi-a-GCST90001724 | rs113812519 | 1 | 207276336 | G | 0.0185 | 0.5546 | 0.09212 | 1.92E-09 |
| ebi-a-GCST90001730 | rs113812519 | 1 | 207276336 | G | 0.0185 | 0.5583 | 0.09163 | 1.22E-09 |
| ebi-a-GCST90001742 | rs113812519 | 1 | 207276336 | G | 0.0185 | 0.5863 | 0.09251 | 2.61E-10 |
| ebi-a-GCST90001729 | rs113812519 | 1 | 207276336 | G | 0.0185 | 0.6788 | 0.09244 | 2.55E-13 |
| ebi-a-GCST90001728 | rs113812519 | 1 | 207276336 | G | 0.0215 | 0.7181 | 0.116 | 7.39E-10 |
| ebi-a-GCST90001697 | rs113819655 | 13 | 100586942 | T | 4.00E-04 | 14.98 | 3.003 | 6.36E-07 |
| ebi-a-GCST90001695 | rs113819655 | 13 | 100586942 | T | 4.00E-04 | 442.7 | 63.56 | 3.90E-12 |
| ebi-a-GCST90001990 | rs1138358 | 15 | 80263345 | C | 0.2286 | 0.142 | 0.02965 | 1.76E-06 |
| ebi-a-GCST90001909 | rs113853911 | 11 | 5263771 | C | 0.0739 | -0.2456 | 0.05234 | 2.81E-06 |
| ebi-a-GCST90002005 | rs113857680 | 1 | 56634923 | T | 0.0545 | -0.2616 | 0.05555 | 2.57E-06 |
| ebi-a-GCST90002090 | rs113861918 | 19 | 52129097 | T | 0.1677 | -0.215 | 0.03692 | 6.42E-09 |
| ebi-a-GCST90001769 | rs113883551 | 18 | 24190315 | C | 0.237 | 0.1339 | 0.02927 | 4.89E-06 |
| ebi-a-GCST90001698 | rs113883934 | 2 | 104720870 | C | 0.0034 | 157.8 | 30.21 | 1.87E-07 |
| ebi-a-GCST90001698 | rs113884990 | 5 | 96997109 | T | 0.0088 | 93.66 | 19.78 | 2.29E-06 |
| ebi-a-GCST90001690 | rs113911480 | 14 | 29113167 | G | 0.0314 | 0.2261 | 0.04927 | 4.61E-06 |
| ebi-a-GCST90001551 | rs113911480 | 14 | 29113167 | G | 0.0314 | 0.2391 | 0.04742 | 4.83E-07 |
| ebi-a-GCST90001566 | rs113911480 | 14 | 29113167 | G | 0.0314 | 0.2769 | 0.05655 | 1.02E-06 |
| ebi-a-GCST90001722 | rs113932257 | 18 | 3127438 | C | 0.0078 | 0.6748 | 0.1425 | 2.28E-06 |
| ebi-a-GCST90001812 | rs113933327 | 6 | 162656478 | A | 0.0052 | -0.829 | 0.1691 | 9.85E-07 |
| ebi-a-GCST90001966 | rs113949273 | 12 | 115749077 | A | 0.0107 | 0.6333 | 0.1358 | 3.26E-06 |
| ebi-a-GCST90001698 | rs113960013 | 5 | 178662302 | G | 0.0355 | 46.84 | 9.534 | 9.43E-07 |
| ebi-a-GCST90001697 | rs113972785 | 13 | 85399806 | A | 0.0015 | 8.626 | 1.673 | 2.68E-07 |
| ebi-a-GCST90001554 | rs113985770 | 2 | 210377930 | A | 0.0103 | -0.6238 | 0.133 | 2.85E-06 |
| ebi-a-GCST90001465 | rs113995133 | 1 | 163485197 | A | 0.0103 | -0.6667 | 0.1287 | 2.34E-07 |
| ebi-a-GCST90001473 | rs113995133 | 1 | 163485197 | A | 0.0103 | -0.6516 | 0.1277 | 3.54E-07 |
| ebi-a-GCST90001472 | rs113995133 | 1 | 163485197 | A | 0.0103 | -0.6066 | 0.1283 | 2.38E-06 |
| ebi-a-GCST90001464 | rs113995133 | 1 | 163485197 | A | 0.0103 | -0.6058 | 0.1285 | 2.51E-06 |
| ebi-a-GCST90002018 | rs114008100 | 7 | 151516164 | T | 0.0279 | 0.43 | 0.08295 | 2.33E-07 |
| ebi-a-GCST90001700 | rs114021246 | 1 | 46898789 | C | 7.00E-04 | 13.26 | 2.594 | 3.34E-07 |
| ebi-a-GCST90001698 | rs114021246 | 1 | 46898789 | C | 7.00E-04 | 585.1 | 59.78 | 2.53E-22 |
| ebi-a-GCST90001695 | rs114057146 | 3 | 190504703 | T | 0.0013 | 203.6 | 37.91 | 8.35E-08 |
| ebi-a-GCST90002006 | rs114066620 | 2 | 67612820 | A | 6.00E-04 | 2.315 | 0.4984 | 3.53E-06 |
| ebi-a-GCST90002112 | rs114068468 | 6 | 33073038 | T | 0.0963 | 0.3671 | 0.06556 | 2.51E-08 |
| ebi-a-GCST90001957 | rs114090793 | 10 | 132935005 | A | 0.0586 | 0.3722 | 0.08036 | 3.92E-06 |
| ebi-a-GCST90002053 | rs114094080 | 5 | 26499129 | A | 0.0222 | 0.6465 | 0.1357 | 2.06E-06 |
| ebi-a-GCST90002042 | rs114094080 | 5 | 26499129 | A | 0.0222 | 0.6897 | 0.1358 | 4.26E-07 |
| ebi-a-GCST90001928 | rs114103379 | 4 | 114954389 | G | 0.0764 | 0.2367 | 0.05148 | 4.45E-06 |
| ebi-a-GCST90001695 | rs114109475 | 4 | 47216939 | A | 0.0075 | 76.96 | 15.41 | 6.26E-07 |
| ebi-a-GCST90001942 | rs114116419 | 1 | 24521634 | C | 0.0173 | -0.472 | 0.0967 | 1.10E-06 |
| ebi-a-GCST90001941 | rs114116419 | 1 | 24521634 | C | 0.0173 | -0.4548 | 0.09717 | 2.97E-06 |
| ebi-a-GCST90001697 | rs114128029 | 5 | 79070021 | T | 0.0025 | 6.293 | 1.218 | 2.55E-07 |
| ebi-a-GCST90001697 | rs114135956 | 2 | 8930530 | C | 3.00E-04 | 32 | 3.541 | 2.61E-19 |
| ebi-a-GCST90001696 | rs114135956 | 2 | 8930530 | C | 3.00E-04 | 53.93 | 8.721 | 6.98E-10 |
| ebi-a-GCST90001477 | rs114141703 | 3 | 197226213 | A | 0.025 | 0.3833 | 0.08005 | 1.75E-06 |
| ebi-a-GCST90001768 | rs114151424 | 1 | 120674028 | A | 0.0036 | 1.159 | 0.2197 | 1.40E-07 |
| ebi-a-GCST90002045 | rs114152229 | 4 | 92853329 | A | 0.0086 | 0.958 | 0.204 | 2.86E-06 |
| ebi-a-GCST90002059 | rs114165927 | 2 | 163117904 | C | 0.0413 | 0.3178 | 0.0671 | 2.28E-06 |
| ebi-a-GCST90001697 | rs114170505 | 6 | 32666487 | C | 0.1839 | -1.08 | 0.2041 | 1.30E-07 |
| ebi-a-GCST90001653 | rs114170505 | 6 | 32666487 | C | 0.1839 | -0.1978 | 0.04098 | 1.44E-06 |
| ebi-a-GCST90001656 | rs114170505 | 6 | 32666487 | C | 0.1839 | 0.1978 | 0.04098 | 1.44E-06 |
| ebi-a-GCST90001543 | rs114174358 | 4 | 1091560 | T | 0.0018 | -1.26 | 0.2626 | 1.66E-06 |
| ebi-a-GCST90001698 | rs114181559 | 1 | 234239162 | T | 0.0038 | 153.3 | 28.67 | 9.51E-08 |
| ebi-a-GCST90001413 | rs114209430 | 4 | 83018399 | C | 0.0085 | -0.6384 | 0.136 | 2.76E-06 |
| ebi-a-GCST90001763 | rs114212270 | 2 | 174337105 | C | 0.0569 | -0.245 | 0.05299 | 3.89E-06 |
| ebi-a-GCST90001697 | rs114216104 | 5 | 140131486 | T | 1.00E-04 | 30.16 | 4.486 | 2.09E-11 |
| ebi-a-GCST90001696 | rs114216104 | 5 | 140131486 | T | 1.00E-04 | 56.58 | 11.01 | 2.91E-07 |
| ebi-a-GCST90001695 | rs114216104 | 5 | 140131486 | T | 1.00E-04 | 591 | 96.85 | 1.16E-09 |
| ebi-a-GCST90001908 | rs114222516 | 4 | 137459015 | T | 0.0177 | 0.5246 | 0.1044 | 5.37E-07 |
| ebi-a-GCST90001700 | rs114230155 | 1 | 102796337 | A | 0.0023 | 9.265 | 1.585 | 5.47E-09 |
| ebi-a-GCST90001698 | rs114230155 | 1 | 102796337 | A | 0.0023 | 324.4 | 37.63 | 1.01E-17 |
| ebi-a-GCST90001840 | rs114241106 | 2 | 47600591 | A | 0.0053 | -0.9152 | 0.1868 | 1.01E-06 |
| ebi-a-GCST90001698 | rs114245333 | 5 | 112619226 | C | 0.0116 | 84.68 | 17.28 | 1.00E-06 |
| ebi-a-GCST90002039 | rs114253672 | 3 | 11967862 | C | 0.3282 | -0.1546 | 0.02924 | 1.34E-07 |
| ebi-a-GCST90002078 | rs114254337 | 5 | 173631361 | A | 0.013 | -0.5249 | 0.1141 | 4.43E-06 |
| ebi-a-GCST90001658 | rs114309505 | 4 | 4345603 | G | 0.0778 | 0.2137 | 0.04557 | 2.84E-06 |
| ebi-a-GCST90001720 | rs114318843 | 2 | 164755835 | C | 0.0122 | -0.5407 | 0.1126 | 1.64E-06 |
| ebi-a-GCST90001708 | rs114318843 | 2 | 164755835 | C | 0.0122 | -0.5242 | 0.1126 | 3.32E-06 |
| ebi-a-GCST90001543 | rs114348846 | 2 | 241731435 | A | 0.2628 | -0.135 | 0.02714 | 6.93E-07 |
| ebi-a-GCST90001735 | rs114369132 | 6 | 32409141 | A | 0.0082 | 0.6636 | 0.1438 | 4.08E-06 |
| ebi-a-GCST90001733 | rs114369132 | 6 | 32409141 | A | 0.0082 | 0.6915 | 0.1438 | 1.59E-06 |
| ebi-a-GCST90002057 | rs114373132 | 6 | 32106813 | T | 0.0263 | -0.4239 | 0.08997 | 2.57E-06 |
| ebi-a-GCST90001505 | rs114400243 | 9 | 11577096 | A | 0.0198 | -0.4481 | 0.09544 | 2.76E-06 |
| ebi-a-GCST90001564 | rs114405373 | 10 | 88835399 | T | 0.0082 | 0.6456 | 0.1331 | 1.29E-06 |
| ebi-a-GCST90001700 | rs114421150 | 4 | 16986930 | A | 3.00E-04 | 25 | 4.08 | 1.00E-09 |
| ebi-a-GCST90001698 | rs114421150 | 4 | 16986930 | A | 3.00E-04 | 915.9 | 94.17 | 4.51E-22 |
| ebi-a-GCST90001698 | rs114425563 | 5 | 176911575 | T | 0.0038 | 172.3 | 28.61 | 1.93E-09 |
| ebi-a-GCST90001700 | rs114430346 | 4 | 102760648 | A | 3.00E-04 | 20.36 | 4.31 | 2.42E-06 |
| ebi-a-GCST90001492 | rs114437347 | 4 | 86967620 | C | 3.00E-04 | -3.936 | 0.8431 | 3.15E-06 |
| ebi-a-GCST90001501 | rs114437347 | 4 | 86967620 | C | 3.00E-04 | -3.873 | 0.8428 | 4.47E-06 |
| ebi-a-GCST90001494 | rs114437347 | 4 | 86967620 | C | 3.00E-04 | -3.842 | 0.8142 | 2.46E-06 |
| ebi-a-GCST90001503 | rs114437347 | 4 | 86967620 | C | 3.00E-04 | -3.73 | 0.8097 | 4.23E-06 |
| ebi-a-GCST90001666 | rs114437347 | 4 | 86967620 | C | 3.00E-04 | 3.498 | 0.739 | 2.30E-06 |
| ebi-a-GCST90001664 | rs114437347 | 4 | 86967620 | C | 3.00E-04 | 3.727 | 0.7678 | 1.26E-06 |
| ebi-a-GCST90001665 | rs114437347 | 4 | 86967620 | C | 3.00E-04 | 3.787 | 0.8267 | 4.81E-06 |
| ebi-a-GCST90001508 | rs114437347 | 4 | 86967620 | C | 3.00E-04 | 3.89 | 0.8453 | 4.33E-06 |
| ebi-a-GCST90002095 | rs114446732 | 1 | 170916456 | A | 0.033 | -0.5131 | 0.1066 | 1.64E-06 |
| ebi-a-GCST90001963 | rs114452046 | 2 | 109542930 | T | 0.0568 | -0.2781 | 0.05989 | 3.58E-06 |
| ebi-a-GCST90001466 | rs114468547 | 16 | 78055434 | G | 0.0491 | -0.2713 | 0.05921 | 4.77E-06 |
| ebi-a-GCST90001529 | rs114469087 | 4 | 90493819 | A | 0.0285 | 0.4988 | 0.1035 | 1.54E-06 |
| ebi-a-GCST90001695 | rs114507999 | 1 | 21892266 | A | 0.0022 | 176.7 | 29.35 | 1.92E-09 |
| ebi-a-GCST90001698 | rs114507999 | 1 | 21892266 | A | 0.0022 | 234.6 | 39.65 | 3.60E-09 |
| ebi-a-GCST90001888 | rs114508332 | 2 | 163595996 | T | 0.0493 | -0.333 | 0.06307 | 1.39E-07 |
| ebi-a-GCST90001693 | rs114547596 | 4 | 97974344 | G | 7.00E-04 | -3.608 | 0.7117 | 4.19E-07 |
| ebi-a-GCST90001691 | rs114547596 | 4 | 97974344 | G | 7.00E-04 | -3.353 | 0.7077 | 2.25E-06 |
| ebi-a-GCST90001692 | rs114547596 | 4 | 97974344 | G | 7.00E-04 | -3.299 | 0.7035 | 2.83E-06 |
| ebi-a-GCST90001446 | rs114551034 | 2 | 66709035 | A | 0.0509 | -0.2926 | 0.05767 | 4.08E-07 |
| ebi-a-GCST90001401 | rs114551034 | 2 | 66709035 | A | 0.0509 | -0.2787 | 0.05695 | 1.03E-06 |
| ebi-a-GCST90001427 | rs114551034 | 2 | 66709035 | A | 0.0509 | -0.2723 | 0.05703 | 1.87E-06 |
| ebi-a-GCST90001689 | rs114559146 | 6 | 31395062 | C | 0.4567 | 0.107 | 0.02302 | 3.44E-06 |
| ebi-a-GCST90001552 | rs114559146 | 6 | 31395062 | C | 0.4561 | 0.1093 | 0.02325 | 2.68E-06 |
| ebi-a-GCST90001700 | rs114559319 | 4 | 85213802 | A | 4.00E-04 | 18.49 | 3.731 | 7.51E-07 |
| ebi-a-GCST90001698 | rs114559319 | 4 | 85213802 | A | 4.00E-04 | 720.8 | 85.31 | 4.27E-17 |
| ebi-a-GCST90001901 | rs114565907 | 1 | 177284570 | T | 0.0074 | 0.7664 | 0.1598 | 1.71E-06 |
| ebi-a-GCST90001465 | rs114566711 | 2 | 191451636 | T | 9.00E-04 | -1.864 | 0.391 | 1.95E-06 |
| ebi-a-GCST90001621 | rs114574090 | 4 | 46796462 | T | 0.0109 | 0.5615 | 0.1194 | 2.66E-06 |
| ebi-a-GCST90001901 | rs114579738 | 15 | 87662869 | T | 0.0626 | -0.2975 | 0.05552 | 9.11E-08 |
| ebi-a-GCST90001553 | rs114581570 | 6 | 19906546 | G | 0.0012 | -1.192 | 0.2444 | 1.14E-06 |
| ebi-a-GCST90001700 | rs114581990 | 2 | 235962017 | G | 0.0022 | 8.874 | 1.564 | 1.51E-08 |
| ebi-a-GCST90001459 | rs114586806 | 6 | 36323614 | T | 0.0292 | 0.3577 | 0.07639 | 2.93E-06 |
| ebi-a-GCST90001572 | rs114588192 | 5 | 146490228 | T | 0.0249 | 0.3812 | 0.08052 | 2.28E-06 |
| ebi-a-GCST90001532 | rs114590598 | 1 | 185852065 | T | 0.0016 | 2.421 | 0.4204 | 9.84E-09 |
| ebi-a-GCST90001531 | rs114590598 | 1 | 185852065 | T | 0.0016 | 2.431 | 0.4216 | 9.41E-09 |
| ebi-a-GCST90002103 | rs114590935 | 1 | 210056681 | C | 0.0211 | 0.4714 | 0.0968 | 1.18E-06 |
| ebi-a-GCST90001700 | rs114619811 | 4 | 71875684 | G | 6.00E-04 | 15.63 | 2.895 | 7.15E-08 |
| ebi-a-GCST90001695 | rs114619811 | 4 | 71875684 | G | 6.00E-04 | 240.1 | 50.93 | 2.52E-06 |
| ebi-a-GCST90001698 | rs114619811 | 4 | 71875684 | G | 6.00E-04 | 725.8 | 66.51 | 2.81E-27 |
| ebi-a-GCST90002035 | rs1146465 | 1 | 95401613 | G | 0.6075 | -0.1373 | 0.02846 | 1.47E-06 |
| ebi-a-GCST90002036 | rs1146465 | 1 | 95401613 | G | 0.6075 | -0.1371 | 0.02851 | 1.59E-06 |
| ebi-a-GCST90001585 | rs11466597 | 1 | 92185351 | C | 0.0331 | 0.3741 | 0.07173 | 1.94E-07 |
| ebi-a-GCST90001919 | rs114672192 | 6 | 15377905 | C | 0.0071 | -0.7638 | 0.1587 | 1.55E-06 |
| ebi-a-GCST90001683 | rs114672498 | 1 | 4591565 | G | 0.0021 | 1.191 | 0.2564 | 3.56E-06 |
| ebi-a-GCST90001700 | rs114672498 | 1 | 4591565 | G | 0.002 | 7.853 | 1.619 | 1.28E-06 |
| ebi-a-GCST90001698 | rs114672498 | 1 | 4591565 | G | 0.0021 | 267.8 | 37.95 | 2.07E-12 |
| ebi-a-GCST90001855 | rs114672530 | 1 | 168306279 | A | 0.0173 | -0.6777 | 0.105 | 1.29E-10 |
| ebi-a-GCST90001868 | rs114672530 | 1 | 168306279 | A | 0.0173 | -0.6683 | 0.1039 | 1.46E-10 |
| ebi-a-GCST90001857 | rs114672530 | 1 | 168306279 | A | 0.0173 | -0.6462 | 0.1049 | 8.19E-10 |
| ebi-a-GCST90001853 | rs114672530 | 1 | 168306279 | A | 0.0173 | -0.6177 | 0.1046 | 3.99E-09 |
| ebi-a-GCST90001867 | rs114672530 | 1 | 168306279 | A | 0.0173 | -0.5285 | 0.103 | 3.03E-07 |
| ebi-a-GCST90001851 | rs114672530 | 1 | 168306279 | A | 0.0173 | -0.52 | 0.104 | 6.12E-07 |
| ebi-a-GCST90001802 | rs114674204 | 2 | 207963323 | A | 0.0645 | -0.2452 | 0.05104 | 1.62E-06 |
| ebi-a-GCST90001700 | rs114685875 | 4 | 138349095 | A | 4.00E-04 | 17.06 | 3.509 | 1.22E-06 |
| ebi-a-GCST90001698 | rs114685875 | 4 | 138349095 | A | 4.00E-04 | 662.4 | 82.11 | 9.84E-16 |
| ebi-a-GCST90002081 | rs114690517 | 3 | 116324393 | C | 0.0145 | 0.5028 | 0.1087 | 3.86E-06 |
| ebi-a-GCST90001973 | rs114690517 | 3 | 116324393 | C | 0.0145 | 0.5367 | 0.106 | 4.31E-07 |
| ebi-a-GCST90001697 | rs114691245 | 1 | 77137266 | T | 7.00E-04 | 11.07 | 2.327 | 2.04E-06 |
| ebi-a-GCST90001453 | rs114693598 | 1 | 161500712 | T | 0.1418 | -0.195 | 0.03862 | 4.68E-07 |
| ebi-a-GCST90001448 | rs114693598 | 1 | 161500712 | T | 0.1422 | -0.1756 | 0.03803 | 4.06E-06 |
| ebi-a-GCST90002062 | rs114729093 | 6 | 55192621 | C | 0.0139 | -0.5661 | 0.1177 | 1.60E-06 |
| ebi-a-GCST90002064 | rs114729093 | 6 | 55192621 | C | 0.0139 | -0.5372 | 0.114 | 2.57E-06 |
| ebi-a-GCST90001539 | rs114753591 | 5 | 43356042 | A | 0.0603 | 0.2494 | 0.05278 | 2.39E-06 |
| ebi-a-GCST90001697 | rs114763811 | 2 | 191457823 | A | 1.00E-04 | 47.98 | 7.59 | 2.92E-10 |
| ebi-a-GCST90001695 | rs114763811 | 2 | 191457823 | A | 1.00E-04 | 979.6 | 163.9 | 2.52E-09 |
| ebi-a-GCST90001992 | rs114766366 | 2 | 1326700 | T | 0.0286 | -0.3563 | 0.0735 | 1.31E-06 |
| ebi-a-GCST90001545 | rs114780135 | 1 | 197674188 | C | 0.0037 | 1.029 | 0.2071 | 7.17E-07 |
| ebi-a-GCST90001508 | rs114780506 | 2 | 104762054 | T | 6.00E-04 | 3.688 | 0.7859 | 2.81E-06 |
| ebi-a-GCST90001509 | rs114780506 | 2 | 104762054 | T | 6.00E-04 | 3.735 | 0.7854 | 2.05E-06 |
| ebi-a-GCST90001698 | rs114791501 | 1 | 81668305 | T | 0.0059 | 139.3 | 25.24 | 3.67E-08 |
| ebi-a-GCST90001688 | rs114795342 | 3 | 24791112 | T | 0.001 | 1.407 | 0.2995 | 2.73E-06 |
| ebi-a-GCST90001560 | rs114799586 | 5 | 27430294 | T | 0.0396 | 0.2664 | 0.05548 | 1.64E-06 |
| ebi-a-GCST90001695 | rs114801643 | 6 | 66316172 | A | 3.00E-04 | 436 | 75.02 | 6.72E-09 |
| ebi-a-GCST90001674 | rs114824462 | 5 | 166532201 | A | 0.0063 | 0.7596 | 0.1602 | 2.21E-06 |
| ebi-a-GCST90001543 | rs114825362 | 1 | 101526164 | C | 0.0201 | -0.4464 | 0.08643 | 2.55E-07 |
| ebi-a-GCST90001544 | rs114825362 | 1 | 101526164 | C | 0.0201 | -0.4079 | 0.08462 | 1.49E-06 |
| ebi-a-GCST90001535 | rs114825362 | 1 | 101526164 | C | 0.0201 | -0.3913 | 0.08534 | 4.70E-06 |
| ebi-a-GCST90001541 | rs114825362 | 1 | 101526164 | C | 0.0201 | 0.4343 | 0.08333 | 1.98E-07 |
| ebi-a-GCST90001700 | rs114827401 | 3 | 31288457 | T | 0.0012 | 10.71 | 2.132 | 5.39E-07 |
| ebi-a-GCST90001591 | rs114828374 | 2 | 149887929 | G | 0.012 | -0.5469 | 0.1123 | 1.16E-06 |
| ebi-a-GCST90002039 | rs114839459 | 1 | 92412978 | T | 0.0051 | -0.8926 | 0.1889 | 2.40E-06 |
| ebi-a-GCST90002017 | rs114839966 | 3 | 47357047 | T | 0.0139 | 0.6718 | 0.1266 | 1.21E-07 |
| ebi-a-GCST90002030 | rs114844216 | 10 | 98158952 | C | 0.0445 | 0.5168 | 0.06518 | 3.09E-15 |
| ebi-a-GCST90001497 | rs114844216 | 10 | 98158952 | C | 0.0464 | 0.5653 | 0.05954 | 3.98E-21 |
| ebi-a-GCST90001496 | rs114844216 | 10 | 98158952 | C | 0.0464 | 0.6107 | 0.06018 | 7.35E-24 |
| ebi-a-GCST90001658 | rs114844216 | 10 | 98158952 | C | 0.0464 | 0.6181 | 0.05724 | 9.27E-27 |
| ebi-a-GCST90001660 | rs114844216 | 10 | 98158952 | C | 0.0459 | 0.6311 | 0.06024 | 2.66E-25 |
| ebi-a-GCST90001659 | rs114844216 | 10 | 98158952 | C | 0.0464 | 0.6597 | 0.05711 | 2.62E-30 |
| ebi-a-GCST90001638 | rs11485850 | 1 | 248784570 | G | 0.023 | 0.3751 | 0.08192 | 4.82E-06 |
| ebi-a-GCST90001637 | rs11485850 | 1 | 248784570 | G | 0.023 | 0.3772 | 0.08225 | 4.68E-06 |
| ebi-a-GCST90001999 | rs114864027 | 5 | 152958194 | T | 0.005 | 0.8298 | 0.1779 | 3.22E-06 |
| ebi-a-GCST90001574 | rs114892491 | 1 | 156921889 | T | 0.042 | -0.2886 | 0.06257 | 4.13E-06 |
| ebi-a-GCST90001606 | rs114905473 | 4 | 111370027 | C | 0.0589 | -0.2375 | 0.05181 | 4.69E-06 |
| ebi-a-GCST90001802 | rs114911864 | 1 | 16508223 | T | 0.0045 | -0.8961 | 0.1904 | 2.62E-06 |
| ebi-a-GCST90001746 | rs114915558 | 5 | 98564385 | G | 0.0384 | 0.305 | 0.06562 | 3.46E-06 |
| ebi-a-GCST90001402 | rs114917376 | 17 | 51764168 | G | 0.0958 | 0.1985 | 0.04285 | 3.74E-06 |
| ebi-a-GCST90001695 | rs114918537 | 5 | 2669980 | A | NA | 1765 | 368.5 | 1.74E-06 |
| ebi-a-GCST90001759 | rs114931155 | 4 | 130319698 | T | 0.0012 | -1.511 | 0.3296 | 4.74E-06 |
| ebi-a-GCST90001781 | rs114964656 | 2 | 182116922 | T | 0.0025 | 1.613 | 0.3486 | 4.00E-06 |
| ebi-a-GCST90001560 | rs114968045 | 6 | 32666960 | C | 0.2996 | -0.1586 | 0.02793 | 1.48E-08 |
| ebi-a-GCST90001667 | rs114968045 | 6 | 32666960 | C | 0.2999 | 0.1474 | 0.03162 | 3.27E-06 |
| ebi-a-GCST90001805 | rs114976873 | 5 | 36783531 | G | 0.0269 | 0.4195 | 0.07718 | 5.84E-08 |
| ebi-a-GCST90001698 | rs115027982 | 2 | 120693004 | C | 0.0043 | 135.8 | 27.93 | 1.20E-06 |
| ebi-a-GCST90001698 | rs115040341 | 3 | 64543612 | T | 0.0054 | 160.1 | 23.53 | 1.21E-11 |
| ebi-a-GCST90002014 | rs115044799 | 3 | 47053135 | G | 0.0221 | -0.4754 | 0.09743 | 1.12E-06 |
| ebi-a-GCST90001698 | rs115052567 | 3 | 169651872 | A | 0.0028 | 179.7 | 36.1 | 6.78E-07 |
| ebi-a-GCST90001449 | rs115058278 | 3 | 101915678 | A | 0.0168 | 0.4772 | 0.1021 | 3.11E-06 |
| ebi-a-GCST90002047 | rs11506039 | 7 | 50344544 | G | 0.035 | 0.4922 | 0.1062 | 3.97E-06 |
| ebi-a-GCST90001819 | rs115062208 | 4 | 15956227 | G | 0.0115 | -0.912 | 0.1184 | 1.69E-14 |
| ebi-a-GCST90001813 | rs115062208 | 4 | 15956227 | G | 0.0115 | -0.8972 | 0.1213 | 1.71E-13 |
| ebi-a-GCST90001815 | rs115062208 | 4 | 15956227 | G | 0.0115 | -0.8942 | 0.1174 | 3.34E-14 |
| ebi-a-GCST90001816 | rs115062208 | 4 | 15956227 | G | 0.0115 | -0.7874 | 0.1197 | 5.48E-11 |
| ebi-a-GCST90001811 | rs115062208 | 4 | 15956227 | G | 0.0115 | -0.6992 | 0.1209 | 7.97E-09 |
| ebi-a-GCST90001812 | rs115062208 | 4 | 15956227 | G | 0.0115 | -0.5779 | 0.1188 | 1.20E-06 |
| ebi-a-GCST90001688 | rs115077170 | 2 | 53872294 | G | NA | 7.992 | 1.404 | 1.37E-08 |
| ebi-a-GCST90001551 | rs1150829 | 6 | 587532 | T | 0.7244 | -0.08657 | 0.01874 | 4.00E-06 |
| ebi-a-GCST90001888 | rs115086130 | 2 | 217086534 | A | 0.0026 | -1.204 | 0.2583 | 3.25E-06 |
| ebi-a-GCST90002014 | rs115100307 | 4 | 31408232 | C | 0.0132 | 0.5864 | 0.1239 | 2.35E-06 |
| ebi-a-GCST90001461 | rs115102594 | 17 | 35251042 | G | 0.0603 | -0.252 | 0.05419 | 3.43E-06 |
| ebi-a-GCST90001890 | rs115108917 | 2 | 173221757 | G | 0.0822 | 0.2327 | 0.05063 | 4.51E-06 |
| ebi-a-GCST90001662 | rs115125396 | 2 | 147306619 | C | 0.0852 | 0.2113 | 0.0446 | 2.24E-06 |
| ebi-a-GCST90001663 | rs115125396 | 2 | 147306619 | C | 0.0855 | 0.2114 | 0.04624 | 5.00E-06 |
| ebi-a-GCST90001661 | rs115125396 | 2 | 147306619 | C | 0.0852 | 0.2263 | 0.04608 | 9.51E-07 |
| ebi-a-GCST90002060 | rs115127002 | 6 | 159442296 | G | 0.0015 | -1.585 | 0.3464 | 4.96E-06 |
| ebi-a-GCST90001678 | rs115134707 | 3 | 60344346 | C | 0.0088 | -0.6449 | 0.1291 | 6.19E-07 |
| ebi-a-GCST90001698 | rs115135290 | 3 | 105859785 | A | 3.00E-04 | 623.6 | 88.02 | 1.69E-12 |
| ebi-a-GCST90001943 | rs115155204 | 4 | 91991072 | T | 0.0215 | 0.4178 | 0.08904 | 2.80E-06 |
| ebi-a-GCST90001936 | rs115155204 | 4 | 91991072 | T | 0.0215 | 0.419 | 0.08885 | 2.50E-06 |
| ebi-a-GCST90002055 | rs115155396 | 5 | 89015995 | C | 0.0512 | 0.2858 | 0.06206 | 4.28E-06 |
| ebi-a-GCST90001420 | rs115156019 | 1 | 168227395 | T | 0.0081 | -0.6197 | 0.1242 | 6.33E-07 |
| ebi-a-GCST90001698 | rs115175419 | 1 | 100459655 | A | 0.0019 | 342.3 | 48.3 | 1.66E-12 |
| ebi-a-GCST90001437 | rs115204570 | 5 | 135756457 | T | 0.0161 | -0.479 | 0.1028 | 3.27E-06 |
| ebi-a-GCST90001840 | rs115208823 | 3 | 101677297 | T | 0.023 | -0.4444 | 0.09386 | 2.29E-06 |
| ebi-a-GCST90001446 | rs115218350 | 3 | 104146883 | A | 8.00E-04 | -1.898 | 0.408 | 3.41E-06 |
| ebi-a-GCST90001403 | rs115218350 | 3 | 104146883 | A | 8.00E-04 | -1.838 | 0.4006 | 4.61E-06 |
| ebi-a-GCST90001688 | rs115218435 | 4 | 67836131 | G | 1.00E-04 | 4.272 | 0.7543 | 1.60E-08 |
| ebi-a-GCST90001698 | rs115221944 | 7 | 56724250 | G | 0.0041 | 192.2 | 28.52 | 1.88E-11 |
| ebi-a-GCST90001994 | rs115232009 | 2 | 181113420 | G | 0.0017 | 1.535 | 0.325 | 2.43E-06 |
| ebi-a-GCST90001843 | rs115265205 | 4 | 63231400 | A | 0.0015 | 1.579 | 0.3329 | 2.20E-06 |
| ebi-a-GCST90001838 | rs115280990 | 4 | 73709210 | A | 0.0052 | 0.888 | 0.1846 | 1.58E-06 |
| ebi-a-GCST90002073 | rs1152928 | 12 | 69247557 | G | 0.0172 | -0.8234 | 0.1053 | 7.27E-15 |
| ebi-a-GCST90002074 | rs1152928 | 12 | 69247557 | G | 0.0165 | -0.7519 | 0.1023 | 2.48E-13 |
| ebi-a-GCST90001631 | rs115294653 | 1 | 28413298 | A | 0.0115 | -0.5427 | 0.1182 | 4.58E-06 |
| ebi-a-GCST90001698 | rs115307086 | 4 | 131409763 | C | 0.0034 | 158.9 | 30.26 | 1.60E-07 |
| ebi-a-GCST90001484 | rs115328872 | 1 | 197921246 | A | 0.0036 | 1.259 | 0.199 | 2.82E-10 |
| ebi-a-GCST90001749 | rs115329330 | 21 | 27580042 | C | 0.0188 | -0.649 | 0.1347 | 1.58E-06 |
| ebi-a-GCST90001527 | rs115342053 | 2 | 178319837 | C | 0.0089 | -0.7808 | 0.1675 | 3.38E-06 |
| ebi-a-GCST90001697 | rs115353080 | 5 | 82672572 | G | 0.0064 | 3.612 | 0.7679 | 2.66E-06 |
| ebi-a-GCST90001497 | rs115364130 | 7 | 83238053 | A | 0.0223 | -0.4063 | 0.0857 | 2.21E-06 |
| ebi-a-GCST90001598 | rs115364254 | 1 | 222677137 | G | 0.1475 | 0.1552 | 0.03343 | 3.57E-06 |
| ebi-a-GCST90001654 | rs115364254 | 1 | 222677137 | G | 0.1476 | 0.1657 | 0.03605 | 4.44E-06 |
| ebi-a-GCST90001667 | rs115370355 | 4 | 92620279 | T | 0.0016 | 1.355 | 0.2853 | 2.11E-06 |
| ebi-a-GCST90001695 | rs115379408 | 6 | 18617129 | A | 3.00E-04 | 450.6 | 77.86 | 7.79E-09 |
| ebi-a-GCST90001587 | rs115383270 | 1 | 161531340 | A | 0.1714 | -0.5431 | 0.03176 | 4.06E-63 |
| ebi-a-GCST90001579 | rs115383270 | 1 | 161531340 | A | 0.1714 | -0.4624 | 0.03231 | 3.10E-45 |
| ebi-a-GCST90001584 | rs115383270 | 1 | 161531340 | A | 0.1714 | -0.4468 | 0.03195 | 2.52E-43 |
| ebi-a-GCST90001992 | rs115383270 | 1 | 161531340 | A | 0.1689 | 0.2574 | 0.03268 | 4.42E-15 |
| ebi-a-GCST90001990 | rs115383270 | 1 | 161531340 | A | 0.1699 | 0.2927 | 0.0332 | 1.81E-18 |
| ebi-a-GCST90001586 | rs115383270 | 1 | 161531340 | A | 0.1714 | 0.5518 | 0.03171 | 3.21E-65 |
| ebi-a-GCST90001482 | rs115383271 | 2 | 39367533 | A | 0.0541 | 0.2552 | 0.0517 | 8.37E-07 |
| ebi-a-GCST90001808 | rs115383596 | 1 | 99399788 | C | 0.0332 | 0.3216 | 0.0697 | 4.09E-06 |
| ebi-a-GCST90001698 | rs11538695 | 20 | 50004560 | A | 0.0023 | 221.5 | 34.4 | 1.36E-10 |
| ebi-a-GCST90001408 | rs115405874 | 5 | 119742816 | G | 0.0037 | -1.013 | 0.2016 | 5.37E-07 |
| ebi-a-GCST90001417 | rs115405874 | 5 | 119742816 | G | 0.0037 | 0.9325 | 0.2017 | 3.93E-06 |
| ebi-a-GCST90001406 | rs115405874 | 5 | 119742816 | G | 0.0037 | 0.9926 | 0.2014 | 8.61E-07 |
| ebi-a-GCST90001438 | rs115414430 | 2 | 36720835 | G | 0.0286 | 0.3388 | 0.07271 | 3.29E-06 |
| ebi-a-GCST90001403 | rs115414430 | 2 | 36720835 | G | 0.0286 | 0.3537 | 0.07458 | 2.20E-06 |
| ebi-a-GCST90001518 | rs115418145 | 4 | 130012204 | T | 5.00E-04 | 3.845 | 0.8059 | 1.97E-06 |
| ebi-a-GCST90001517 | rs115418145 | 4 | 130012204 | T | 5.00E-04 | 3.847 | 0.8008 | 1.67E-06 |
| ebi-a-GCST90001926 | rs115426429 | 17 | 59341923 | T | 0.0295 | -0.4316 | 0.08247 | 1.78E-07 |
| ebi-a-GCST90001883 | rs115442185 | 4 | 109239280 | G | 0.0392 | 0.3246 | 0.06879 | 2.47E-06 |
| ebi-a-GCST90001695 | rs115445664 | 3 | 126486072 | G | 3.00E-04 | 349.4 | 70.62 | 7.87E-07 |
| ebi-a-GCST90001961 | rs115446744 | 1 | 206716987 | T | 0.0116 | 0.6026 | 0.1264 | 1.97E-06 |
| ebi-a-GCST90001583 | rs115451299 | 1 | 110012633 | C | 0.0018 | -1.305 | 0.2683 | 1.19E-06 |
| ebi-a-GCST90001580 | rs115451299 | 1 | 110012633 | C | 0.0018 | -1.259 | 0.2738 | 4.40E-06 |
| ebi-a-GCST90001677 | rs115466519 | 5 | 96883867 | T | 0.0115 | -0.5294 | 0.1145 | 3.95E-06 |
| ebi-a-GCST90001561 | rs115475145 | 4 | 148303054 | C | 0.0238 | 0.3476 | 0.07361 | 2.43E-06 |
| ebi-a-GCST90001894 | rs115478283 | 4 | 13704839 | G | 0.057 | -0.2738 | 0.05811 | 2.56E-06 |
| ebi-a-GCST90001898 | rs115478283 | 4 | 13704839 | G | 0.057 | -0.2669 | 0.05822 | 4.75E-06 |
| ebi-a-GCST90001526 | rs115483539 | 5 | 50273359 | A | 0.053 | -0.3509 | 0.07502 | 3.12E-06 |
| ebi-a-GCST90001528 | rs115483539 | 5 | 50273359 | A | 0.053 | 0.3458 | 0.07508 | 4.38E-06 |
| ebi-a-GCST90001695 | rs115485721 | 6 | 65783601 | A | 0.0139 | 69.53 | 11.54 | 1.87E-09 |
| ebi-a-GCST90001785 | rs11548656 | 16 | 81916912 | G | 0.1287 | -0.2363 | 0.03676 | 1.47E-10 |
| ebi-a-GCST90001786 | rs11548656 | 16 | 81916912 | G | 0.1289 | -0.1707 | 0.03656 | 3.15E-06 |
| ebi-a-GCST90001428 | rs11548656 | 16 | 81916912 | G | 0.1288 | -0.1701 | 0.0366 | 3.48E-06 |
| ebi-a-GCST90001695 | rs115486816 | 1 | 56588047 | A | 0.0023 | 127.9 | 26.15 | 1.04E-06 |
| ebi-a-GCST90002009 | rs115510074 | 2 | 142724921 | A | NA | -20.77 | 4.02 | 2.50E-07 |
| ebi-a-GCST90001581 | rs115510074 | 2 | 142724921 | A | NA | -20.12 | 4.118 | 1.07E-06 |
| ebi-a-GCST90001698 | rs115517310 | 2 | 170436614 | C | 0.0034 | 177.5 | 30.9 | 1.01E-08 |
| ebi-a-GCST90001621 | rs115536607 | 6 | 101417409 | T | 0.0547 | 0.264 | 0.0552 | 1.79E-06 |
| ebi-a-GCST90001883 | rs115536607 | 6 | 101417409 | T | 0.0544 | 0.2841 | 0.05936 | 1.78E-06 |
| ebi-a-GCST90001623 | rs115536607 | 6 | 101417409 | T | 0.0545 | 0.3109 | 0.05547 | 2.25E-08 |
| ebi-a-GCST90001622 | rs115536607 | 6 | 101417409 | T | 0.0545 | 0.3253 | 0.05512 | 3.91E-09 |
| ebi-a-GCST90001633 | rs115536607 | 6 | 101417409 | T | 0.0548 | 0.3487 | 0.05419 | 1.40E-10 |
| ebi-a-GCST90001638 | rs115536607 | 6 | 101417409 | T | 0.0545 | 0.3576 | 0.05416 | 4.64E-11 |
| ebi-a-GCST90001637 | rs115536607 | 6 | 101417409 | T | 0.0545 | 0.3694 | 0.05436 | 1.25E-11 |
| ebi-a-GCST90001635 | rs115536607 | 6 | 101417409 | T | 0.0545 | 0.3865 | 0.05433 | 1.35E-12 |
| ebi-a-GCST90001634 | rs115536607 | 6 | 101417409 | T | 0.0545 | 0.3978 | 0.05455 | 3.73E-13 |
| ebi-a-GCST90001679 | rs115540740 | 5 | 2863938 | A | 0.0223 | 0.414 | 0.08661 | 1.83E-06 |
| ebi-a-GCST90001681 | rs115540740 | 5 | 2863938 | A | 0.0219 | 0.4219 | 0.08851 | 1.96E-06 |
| ebi-a-GCST90002073 | rs11554159 | 19 | 18285944 | A | 0.2982 | -0.3745 | 0.02938 | 3.14E-36 |
| ebi-a-GCST90002074 | rs11554159 | 19 | 18285944 | A | 0.2995 | -0.2988 | 0.0284 | 1.82E-25 |
| ebi-a-GCST90001661 | rs115545560 | 2 | 7793905 | G | 0.0039 | 0.9405 | 0.2056 | 4.96E-06 |
| ebi-a-GCST90001697 | rs115546994 | 5 | 159062854 | T | 4.00E-04 | 15.77 | 2.724 | 7.76E-09 |
| ebi-a-GCST90001695 | rs115546994 | 5 | 159062854 | T | 4.00E-04 | 383.2 | 58.71 | 7.72E-11 |
| ebi-a-GCST90002038 | rs115568119 | 1 | 118721918 | T | 0.0078 | -0.7767 | 0.1594 | 1.17E-06 |
| ebi-a-GCST90002037 | rs115568119 | 1 | 118721918 | T | 0.0078 | -0.7747 | 0.1594 | 1.24E-06 |
| ebi-a-GCST90001697 | rs115569583 | 5 | 145810889 | T | NA | 68.79 | 13.59 | 4.39E-07 |
| ebi-a-GCST90001695 | rs115569583 | 5 | 145810889 | T | NA | 1448 | 292.2 | 7.53E-07 |
| ebi-a-GCST90001510 | rs115575166 | 2 | 38573789 | A | 0.0113 | -0.5649 | 0.1208 | 3.02E-06 |
| ebi-a-GCST90001477 | rs115589615 | 2 | 106954936 | T | 0.0176 | -0.4684 | 0.09367 | 5.98E-07 |
| ebi-a-GCST90001476 | rs115589615 | 2 | 106954936 | T | 0.0175 | -0.4683 | 0.09513 | 8.93E-07 |
| ebi-a-GCST90001919 | rs115618992 | 4 | 5432284 | T | 0.0019 | 1.419 | 0.3014 | 2.60E-06 |
| ebi-a-GCST90001697 | rs11561957 | 7 | 135470972 | A | 9.00E-04 | 10.19 | 2.094 | 1.19E-06 |
| ebi-a-GCST90002083 | rs115620709 | 4 | 148953372 | T | 0.0059 | -1.106 | 0.2072 | 1.01E-07 |
| ebi-a-GCST90001917 | rs115625722 | 4 | 21411107 | A | 6.00E-04 | -2.286 | 0.4857 | 2.64E-06 |
| ebi-a-GCST90001460 | rs115638174 | 2 | 178694935 | T | 0.0052 | -0.7983 | 0.166 | 1.59E-06 |
| ebi-a-GCST90001698 | rs115640110 | 2 | 181840 | T | 0.0044 | 127.2 | 27.45 | 3.76E-06 |
| ebi-a-GCST90001673 | rs115642223 | 4 | 141162325 | C | 0.0019 | 1.51 | 0.3259 | 3.76E-06 |
| ebi-a-GCST90001674 | rs115642223 | 4 | 141162325 | C | 0.0019 | 1.53 | 0.3289 | 3.43E-06 |
| ebi-a-GCST90001707 | rs115650989 | 8 | 145510474 | A | 0.0299 | -0.5049 | 0.09989 | 4.76E-07 |
| ebi-a-GCST90001706 | rs115650989 | 8 | 145510474 | A | 0.0299 | -0.4764 | 0.09988 | 1.99E-06 |
| ebi-a-GCST90001698 | rs115660429 | 3 | 195531004 | A | 0.0023 | 207.9 | 36.33 | 1.15E-08 |
| ebi-a-GCST90001869 | rs115662161 | 16 | 11511908 | G | 0.1405 | -0.1939 | 0.04161 | 3.32E-06 |
| ebi-a-GCST90001658 | rs115665382 | 3 | 116305711 | A | 0.0087 | -0.625 | 0.1345 | 3.49E-06 |
| ebi-a-GCST90001698 | rs115677012 | 4 | 167555345 | T | 0.0016 | 315.7 | 41.99 | 7.06E-14 |
| ebi-a-GCST90001932 | rs11567754 | 5 | 35872190 | A | 0.105 | -0.2975 | 0.04334 | 8.08E-12 |
| ebi-a-GCST90001773 | rs11568076 | 17 | 4544520 | G | 0.0029 | 1.098 | 0.2337 | 2.70E-06 |
| ebi-a-GCST90001769 | rs11568076 | 17 | 4544520 | G | 0.0029 | 1.2 | 0.2345 | 3.27E-07 |
| ebi-a-GCST90001995 | rs115689956 | 7 | 78978735 | G | 0.1032 | 0.1997 | 0.04096 | 1.13E-06 |
| ebi-a-GCST90001661 | rs115695595 | 6 | 45300097 | T | 0.0626 | -0.246 | 0.053 | 3.60E-06 |
| ebi-a-GCST90001644 | rs115698234 | 5 | 66793608 | G | 0.0105 | 0.5437 | 0.1169 | 3.43E-06 |
| ebi-a-GCST90001700 | rs115715247 | 5 | 68399621 | T | 1.00E-04 | 27.58 | 5.685 | 1.28E-06 |
| ebi-a-GCST90001697 | rs115724263 | 5 | 139389602 | T | 1.00E-04 | 33.78 | 6.59 | 3.13E-07 |
| ebi-a-GCST90001698 | rs11572436 | 1 | 217191870 | C | 0.0028 | 158.3 | 32.6 | 1.25E-06 |
| ebi-a-GCST90001726 | rs115727814 | 4 | 170699036 | A | 0.0021 | -1.275 | 0.2657 | 1.66E-06 |
| ebi-a-GCST90001741 | rs115727814 | 4 | 170699036 | A | 0.0021 | -1.231 | 0.266 | 3.83E-06 |
| ebi-a-GCST90001725 | rs115727814 | 4 | 170699036 | A | 0.0021 | -1.228 | 0.2655 | 3.83E-06 |
| ebi-a-GCST90001461 | rs115751481 | 3 | 41531792 | A | 0.0258 | -0.3782 | 0.08174 | 3.84E-06 |
| ebi-a-GCST90001972 | rs115752428 | 1 | 205560814 | A | 0.0226 | 0.3828 | 0.08303 | 4.17E-06 |
| ebi-a-GCST90002082 | rs115752428 | 1 | 205560814 | A | 0.0226 | 0.3845 | 0.07935 | 1.33E-06 |
| ebi-a-GCST90002079 | rs115752428 | 1 | 205560814 | A | 0.0226 | 0.4358 | 0.08328 | 1.78E-07 |
| ebi-a-GCST90002080 | rs115752428 | 1 | 205560814 | A | 0.0226 | 0.482 | 0.08487 | 1.47E-08 |
| ebi-a-GCST90001698 | rs115755474 | 5 | 168914162 | T | 3.00E-04 | 771.6 | 91.49 | 4.87E-17 |
| ebi-a-GCST90001581 | rs115758894 | 18 | 53138692 | A | 0.0789 | 0.2183 | 0.04551 | 1.68E-06 |
| ebi-a-GCST90002112 | rs11576012 | 6 | 31795619 | T | 0.0523 | 0.4345 | 0.08199 | 1.32E-07 |
| ebi-a-GCST90001700 | rs11576563 | 1 | 6118693 | T | 6.00E-04 | 13.78 | 2.988 | 4.16E-06 |
| ebi-a-GCST90001698 | rs11576563 | 1 | 6118693 | T | 6.00E-04 | 794.2 | 70 | 2.61E-29 |
| ebi-a-GCST90001499 | rs11577174 | 1 | 230492271 | T | 0.0012 | -1.588 | 0.343 | 3.78E-06 |
| ebi-a-GCST90001493 | rs11577174 | 1 | 230492271 | T | 0.0012 | 1.612 | 0.3412 | 2.39E-06 |
| ebi-a-GCST90002071 | rs115780495 | 6 | 32501627 | G | 0.1868 | 0.34 | 0.05429 | 4.35E-10 |
| ebi-a-GCST90002085 | rs115781665 | 6 | 67256822 | A | 0.0041 | 1.05 | 0.2191 | 1.73E-06 |
| ebi-a-GCST90002056 | rs115795061 | 6 | 32731861 | A | 0.0455 | -0.3131 | 0.06839 | 4.90E-06 |
| ebi-a-GCST90001502 | rs11579717 | 1 | 199999121 | A | 0.0044 | -1.821 | 0.1663 | 1.87E-27 |
| ebi-a-GCST90001493 | rs11579717 | 1 | 199999121 | A | 0.0044 | -1.776 | 0.1774 | 2.93E-23 |
| ebi-a-GCST90001494 | rs11579717 | 1 | 199999121 | A | 0.0044 | -1.625 | 0.1865 | 4.49E-18 |
| ebi-a-GCST90001503 | rs11579717 | 1 | 199999121 | A | 0.0044 | -1.573 | 0.1855 | 3.35E-17 |
| ebi-a-GCST90001693 | rs11579717 | 1 | 199999121 | A | 0.0044 | -1.253 | 0.1905 | 5.53E-11 |
| ebi-a-GCST90001692 | rs11579717 | 1 | 199999121 | A | 0.0044 | -1.249 | 0.188 | 3.55E-11 |
| ebi-a-GCST90001668 | rs11579717 | 1 | 199999121 | A | 0.0044 | -1.217 | 0.1857 | 6.36E-11 |
| ebi-a-GCST90001667 | rs11579717 | 1 | 199999121 | A | 0.0044 | -1.201 | 0.1771 | 1.42E-11 |
| ebi-a-GCST90001492 | rs11579717 | 1 | 199999121 | A | 0.0044 | -1.201 | 0.1934 | 5.94E-10 |
| ebi-a-GCST90001501 | rs11579717 | 1 | 199999121 | A | 0.0044 | -1.179 | 0.1933 | 1.18E-09 |
| ebi-a-GCST90001669 | rs11579717 | 1 | 199999121 | A | 0.0044 | -1.125 | 0.169 | 3.18E-11 |
| ebi-a-GCST90001535 | rs11579717 | 1 | 199999121 | A | 0.0044 | -1.081 | 0.1759 | 8.87E-10 |
| ebi-a-GCST90001512 | rs11579717 | 1 | 199999121 | A | 0.0044 | -1.078 | 0.1911 | 1.83E-08 |
| ebi-a-GCST90001510 | rs11579717 | 1 | 199999121 | A | 0.0044 | -1.039 | 0.1939 | 8.77E-08 |
| ebi-a-GCST90001536 | rs11579717 | 1 | 199999121 | A | 0.0044 | -0.9339 | 0.1786 | 1.79E-07 |
| ebi-a-GCST90001543 | rs11579717 | 1 | 199999121 | A | 0.0044 | -0.9188 | 0.1786 | 2.82E-07 |
| ebi-a-GCST90001544 | rs11579717 | 1 | 199999121 | A | 0.0044 | -0.8849 | 0.1748 | 4.33E-07 |
| ebi-a-GCST90001541 | rs11579717 | 1 | 199999121 | A | 0.0044 | 0.8342 | 0.1723 | 1.33E-06 |
| ebi-a-GCST90001558 | rs11579717 | 1 | 199999121 | A | 0.0044 | 0.8882 | 0.1864 | 1.96E-06 |
| ebi-a-GCST90001666 | rs11579717 | 1 | 199999121 | A | 0.0044 | 1.176 | 0.1662 | 1.77E-12 |
| ebi-a-GCST90002099 | rs11579717 | 1 | 199999121 | A | 0.0045 | 1.19 | 0.202 | 4.31E-09 |
| ebi-a-GCST90001664 | rs11579717 | 1 | 199999121 | A | 0.0044 | 1.303 | 0.1744 | 1.02E-13 |
| ebi-a-GCST90001665 | rs11579717 | 1 | 199999121 | A | 0.0044 | 1.307 | 0.1858 | 2.37E-12 |
| ebi-a-GCST90002102 | rs11579717 | 1 | 199999121 | A | 0.0041 | 1.499 | 0.1979 | 4.74E-14 |
| ebi-a-GCST90001485 | rs11579717 | 1 | 199999121 | A | 0.0044 | 1.718 | 0.1835 | 1.35E-20 |
| ebi-a-GCST90001499 | rs11579717 | 1 | 199999121 | A | 0.0044 | 1.815 | 0.1783 | 5.33E-24 |
| ebi-a-GCST90001458 | rs115805162 | 1 | 161410253 | T | 0.2083 | -0.2265 | 0.03229 | 2.75E-12 |
| ebi-a-GCST90001903 | rs115805162 | 1 | 161410253 | T | 0.2088 | -0.1652 | 0.035 | 2.47E-06 |
| ebi-a-GCST90001970 | rs115805162 | 1 | 161410253 | T | 0.2099 | 0.2393 | 0.03389 | 2.03E-12 |
| ebi-a-GCST90002077 | rs115805162 | 1 | 161410253 | T | 0.2099 | 0.2464 | 0.03359 | 2.85E-13 |
| ebi-a-GCST90002005 | rs115805162 | 1 | 161410253 | T | 0.2111 | 0.306 | 0.03117 | 1.81E-22 |
| ebi-a-GCST90001700 | rs11580627 | 1 | 207801545 | C | 7.00E-04 | 15.69 | 3.028 | 2.33E-07 |
| ebi-a-GCST90001636 | rs11580805 | 1 | 56118105 | T | 0.0752 | -0.2119 | 0.04608 | 4.40E-06 |
| ebi-a-GCST90001698 | rs115821332 | 3 | 115341107 | G | 6.00E-04 | 759.4 | 70.06 | 6.21E-27 |
| ebi-a-GCST90001828 | rs115835855 | 4 | 131384142 | C | 0.0479 | 0.268 | 0.0578 | 3.65E-06 |
| ebi-a-GCST90002069 | rs11584259 | 1 | 22732386 | C | 0.1651 | 0.1692 | 0.03656 | 3.87E-06 |
| ebi-a-GCST90001930 | rs115849418 | 1 | 56800633 | A | 0.0017 | 1.501 | 0.3198 | 2.80E-06 |
| ebi-a-GCST90001464 | rs115849513 | 3 | 1827937 | T | 0.0245 | -0.3927 | 0.08325 | 2.50E-06 |
| ebi-a-GCST90002027 | rs11584966 | 1 | 226382922 | G | 0.05 | -0.2921 | 0.06245 | 3.05E-06 |
| ebi-a-GCST90001739 | rs11585051 | 1 | 235762345 | A | 0.5766 | 0.116 | 0.02451 | 2.31E-06 |
| ebi-a-GCST90001780 | rs115861292 | 3 | 126541810 | G | 0.021 | 0.4129 | 0.08871 | 3.36E-06 |
| ebi-a-GCST90001915 | rs115861687 | 1 | 42697317 | C | 6.00E-04 | -2.43 | 0.499 | 1.18E-06 |
| ebi-a-GCST90001487 | rs115862633 | 2 | 229924038 | G | 0.0041 | -0.8837 | 0.192 | 4.31E-06 |
| ebi-a-GCST90002096 | rs115866025 | 2 | 23861433 | T | 0.0167 | -0.6468 | 0.1409 | 4.82E-06 |
| ebi-a-GCST90001801 | rs115870915 | 1 | 114134030 | A | 0.0133 | -0.6879 | 0.1487 | 3.99E-06 |
| ebi-a-GCST90002022 | rs11587381 | 1 | 226377250 | G | 0.0495 | -0.3266 | 0.06286 | 2.18E-07 |
| ebi-a-GCST90001396 | rs1158784 | 6 | 32758761 | T | 0.1554 | 0.174 | 0.03801 | 4.88E-06 |
| ebi-a-GCST90001700 | rs115879183 | 3 | 194347376 | G | 9.00E-04 | 12.99 | 2.578 | 4.96E-07 |
| ebi-a-GCST90001695 | rs115879183 | 3 | 194347376 | G | 9.00E-04 | 281.9 | 45.23 | 5.10E-10 |
| ebi-a-GCST90001698 | rs115879183 | 3 | 194347376 | G | 9.00E-04 | 571.9 | 61.21 | 1.66E-20 |
| ebi-a-GCST90001632 | rs115889733 | 6 | 44207850 | A | 0.0777 | 0.2125 | 0.04611 | 4.19E-06 |
| ebi-a-GCST90002069 | rs115907820 | 7 | 37068759 | C | 0.0166 | 0.5043 | 0.1054 | 1.82E-06 |
| ebi-a-GCST90002034 | rs11592078 | 10 | 30653567 | A | 0.0736 | 0.2483 | 0.05373 | 3.98E-06 |
| ebi-a-GCST90001992 | rs11592564 | 10 | 115126137 | C | 0.0203 | 0.4087 | 0.0855 | 1.82E-06 |
| ebi-a-GCST90001695 | rs115928015 | 4 | 156055689 | C | 4.00E-04 | 318.1 | 62.32 | 3.51E-07 |
| ebi-a-GCST90001808 | rs115933247 | 3 | 107086449 | T | 0.015 | 0.5117 | 0.1095 | 3.07E-06 |
| ebi-a-GCST90001486 | rs11593335 | 10 | 6160702 | T | 0.4164 | -0.1265 | 0.02623 | 1.48E-06 |
| ebi-a-GCST90001514 | rs115937242 | 2 | 136121922 | A | 0.0086 | 1.543 | 0.1747 | 2.30E-18 |
| ebi-a-GCST90001962 | rs115939244 | 1 | 3165628 | G | 0.1485 | -0.1924 | 0.03749 | 3.05E-07 |
| ebi-a-GCST90002114 | rs115943613 | 2 | 40943702 | A | 0.0243 | 0.4269 | 0.08416 | 4.18E-07 |
| ebi-a-GCST90001525 | rs11594395 | 10 | 2817998 | G | 0.024 | 0.514 | 0.1111 | 3.94E-06 |
| ebi-a-GCST90001698 | rs115945729 | 2 | 199934892 | A | 3.00E-04 | 896.2 | 97.49 | 6.49E-20 |
| ebi-a-GCST90001732 | rs115952864 | 4 | 173244216 | A | 0.0208 | 0.414 | 0.08576 | 1.44E-06 |
| ebi-a-GCST90001654 | rs115953192 | 2 | 45208257 | G | 0.0462 | -0.279 | 0.06079 | 4.60E-06 |
| ebi-a-GCST90001697 | rs115954619 | 2 | 174896997 | A | 0.0015 | 9.757 | 1.738 | 2.15E-08 |
| ebi-a-GCST90001695 | rs115954619 | 2 | 174896997 | A | 0.0015 | 213 | 36.73 | 7.23E-09 |
| ebi-a-GCST90001693 | rs11595826 | 10 | 111696639 | C | 0.1174 | 0.1862 | 0.0398 | 2.99E-06 |
| ebi-a-GCST90001650 | rs11595870 | 10 | 12408809 | A | 0.0367 | 0.3054 | 0.06599 | 3.82E-06 |
| ebi-a-GCST90001698 | rs11596188 | 10 | 1734511 | G | 9.00E-04 | 526.2 | 54.32 | 6.51E-22 |
| ebi-a-GCST90001834 | rs11597201 | 10 | 88469837 | G | 0.0212 | -0.4448 | 0.09654 | 4.27E-06 |
| ebi-a-GCST90001485 | rs115977640 | 2 | 160238519 | A | 0.0077 | 0.6189 | 0.1337 | 3.83E-06 |
| ebi-a-GCST90001833 | rs115980202 | 4 | 42489975 | C | 0.0254 | 0.3845 | 0.08218 | 3.03E-06 |
| ebi-a-GCST90001790 | rs11598494 | 10 | 6178941 | C | 0.5587 | -0.1768 | 0.02448 | 6.09E-13 |
| ebi-a-GCST90001777 | rs11598494 | 10 | 6178941 | C | 0.5587 | -0.1762 | 0.02445 | 6.79E-13 |
| ebi-a-GCST90001792 | rs11598494 | 10 | 6178941 | C | 0.5587 | -0.1584 | 0.02453 | 1.20E-10 |
| ebi-a-GCST90001778 | rs11598494 | 10 | 6178941 | C | 0.5587 | -0.1524 | 0.02465 | 6.89E-10 |
| ebi-a-GCST90001495 | rs11598645 | 10 | 68351521 | C | 0.011 | -0.6337 | 0.1381 | 4.64E-06 |
| ebi-a-GCST90001658 | rs11598645 | 10 | 68351521 | C | 0.011 | -0.6106 | 0.1299 | 2.69E-06 |
| ebi-a-GCST90001629 | rs11598687 | 10 | 76609571 | C | 0.0028 | -1.027 | 0.2159 | 2.03E-06 |
| ebi-a-GCST90001628 | rs11598687 | 10 | 76609571 | C | 0.0028 | -1.02 | 0.2128 | 1.70E-06 |
| ebi-a-GCST90001695 | rs11598958 | 10 | 16773515 | G | NA | 1814 | 365.6 | 7.29E-07 |
| ebi-a-GCST90001706 | rs11599977 | 11 | 38419307 | A | 0.7787 | 0.196 | 0.04096 | 1.84E-06 |
| ebi-a-GCST90001707 | rs11599977 | 11 | 38419307 | A | 0.7787 | 0.2152 | 0.04096 | 1.66E-07 |
| ebi-a-GCST90001987 | rs116004327 | 1 | 117459175 | T | 0.0105 | 0.6062 | 0.125 | 1.28E-06 |
| ebi-a-GCST90002006 | rs116004327 | 1 | 117459175 | T | 0.0105 | 0.6192 | 0.1257 | 8.77E-07 |
| ebi-a-GCST90002108 | rs116007826 | 6 | 31569745 | G | 0.0681 | 0.6643 | 0.07716 | 1.75E-17 |
| ebi-a-GCST90002105 | rs116007826 | 6 | 31569745 | G | 0.0686 | 0.751 | 0.06113 | 7.61E-34 |
| ebi-a-GCST90002104 | rs116007826 | 6 | 31569745 | G | 0.0686 | 0.8001 | 0.06015 | 3.14E-39 |
| ebi-a-GCST90002106 | rs116007826 | 6 | 31569745 | G | 0.0686 | 0.8336 | 0.06047 | 6.52E-42 |
| ebi-a-GCST90001678 | rs116011991 | 1 | 181205200 | C | 0.0021 | 1.22 | 0.2569 | 2.13E-06 |
| ebi-a-GCST90001720 | rs116023377 | 4 | 18858102 | C | 0.0066 | -0.7362 | 0.156 | 2.46E-06 |
| ebi-a-GCST90001917 | rs116030815 | 1 | 42691989 | C | 5.00E-04 | -3.125 | 0.5674 | 3.93E-08 |
| ebi-a-GCST90001659 | rs116034038 | 1 | 65028833 | G | 0.016 | -0.4404 | 0.09181 | 1.68E-06 |
| ebi-a-GCST90001698 | rs116034906 | 2 | 118737657 | T | 1.00E-04 | 737.2 | 135.8 | 6.09E-08 |
| ebi-a-GCST90001505 | rs11603619 | 11 | 12094804 | A | 0.0159 | -0.476 | 0.09847 | 1.40E-06 |
| ebi-a-GCST90001698 | rs116039349 | 2 | 132603936 | T | 0.0031 | 173.7 | 33.85 | 3.05E-07 |
| ebi-a-GCST90001749 | rs11604619 | 11 | 60261732 | T | 0.6074 | -0.2292 | 0.03378 | 1.56E-11 |
| ebi-a-GCST90001471 | rs116054627 | 2 | 51168498 | A | 0.0411 | 0.2959 | 0.063 | 2.74E-06 |
| ebi-a-GCST90001470 | rs116054627 | 2 | 51168498 | A | 0.0413 | 0.3356 | 0.06158 | 5.39E-08 |
| ebi-a-GCST90002080 | rs11607999 | 11 | 44623672 | C | 0.2313 | 0.1449 | 0.03088 | 2.80E-06 |
| ebi-a-GCST90001574 | rs116088408 | 12 | 27632799 | T | 0.0083 | -0.6539 | 0.1382 | 2.33E-06 |
| ebi-a-GCST90002089 | rs11608886 | 12 | 105713710 | T | 0.014 | 0.5375 | 0.1161 | 3.86E-06 |
| ebi-a-GCST90001425 | rs11609248 | 12 | 14317207 | G | 0.1916 | -0.1438 | 0.02996 | 1.65E-06 |
| ebi-a-GCST90001453 | rs11610005 | 12 | 27966210 | G | 0.4359 | -0.1298 | 0.02728 | 2.05E-06 |
| ebi-a-GCST90001449 | rs11610005 | 12 | 27966210 | G | 0.4358 | -0.124 | 0.02698 | 4.53E-06 |
| ebi-a-GCST90002068 | rs11610038 | 12 | 4770669 | T | 0.0128 | -0.5841 | 0.1186 | 8.99E-07 |
| ebi-a-GCST90001598 | rs11610256 | 12 | 111734121 | T | 0.27 | -0.1391 | 0.02841 | 1.02E-06 |
| ebi-a-GCST90001904 | rs116105787 | 4 | 167699242 | T | 0.0044 | 1.124 | 0.2001 | 2.16E-08 |
| ebi-a-GCST90002053 | rs11612383 | 12 | 1831355 | A | 0.3367 | -0.1848 | 0.03881 | 2.09E-06 |
| ebi-a-GCST90001851 | rs116128731 | 2 | 215390543 | A | 0.0199 | 0.4577 | 0.09839 | 3.43E-06 |
| ebi-a-GCST90001688 | rs116131582 | 3 | 177291240 | C | 0.0029 | -0.7833 | 0.1645 | 2.01E-06 |
| ebi-a-GCST90002063 | rs116138399 | 6 | 31325478 | C | 0.1094 | 0.2243 | 0.04782 | 2.86E-06 |
| ebi-a-GCST90001754 | rs116144119 | 1 | 165812513 | A | 0.0533 | 0.265 | 0.05664 | 3.00E-06 |
| ebi-a-GCST90001959 | rs11614716 | 12 | 6904146 | G | 0.2485 | -0.1537 | 0.03089 | 6.83E-07 |
| ebi-a-GCST90001814 | rs116152899 | 2 | 1833668 | T | 0.0032 | -1.085 | 0.2285 | 2.12E-06 |
| ebi-a-GCST90002027 | rs11615628 | 12 | 6903631 | A | 0.2465 | -0.3264 | 0.03137 | 6.46E-25 |
| ebi-a-GCST90002025 | rs11615628 | 12 | 6903631 | A | 0.2464 | -0.3219 | 0.03112 | 1.21E-24 |
| ebi-a-GCST90002023 | rs11615628 | 12 | 6903631 | A | 0.2464 | -0.3032 | 0.03145 | 1.12E-21 |
| ebi-a-GCST90002022 | rs11615628 | 12 | 6903631 | A | 0.2464 | -0.2753 | 0.03163 | 5.31E-18 |
| ebi-a-GCST90002066 | rs11615628 | 12 | 6903631 | A | 0.2466 | -0.2479 | 0.03131 | 3.36E-15 |
| ebi-a-GCST90002063 | rs11615628 | 12 | 6903631 | A | 0.2466 | -0.2417 | 0.03133 | 1.66E-14 |
| ebi-a-GCST90002070 | rs11615628 | 12 | 6903631 | A | 0.2466 | -0.2367 | 0.03149 | 7.48E-14 |
| ebi-a-GCST90002067 | rs11615628 | 12 | 6903631 | A | 0.2466 | -0.2062 | 0.03133 | 5.49E-11 |
| ebi-a-GCST90002068 | rs11615628 | 12 | 6903631 | A | 0.2466 | -0.2046 | 0.03153 | 1.00E-10 |
| ebi-a-GCST90002062 | rs11615628 | 12 | 6903631 | A | 0.2466 | -0.2032 | 0.03171 | 1.71E-10 |
| ebi-a-GCST90002064 | rs11615628 | 12 | 6903631 | A | 0.2467 | -0.1847 | 0.03074 | 2.11E-09 |
| ebi-a-GCST90002061 | rs11615628 | 12 | 6903631 | A | 0.2466 | -0.1493 | 0.03191 | 3.01E-06 |
| ebi-a-GCST90001606 | rs116160694 | 6 | 67354817 | C | 0.0067 | 0.6955 | 0.1503 | 3.82E-06 |
| ebi-a-GCST90001783 | rs11616609 | 13 | 35106024 | T | 0.3606 | -0.1207 | 0.02541 | 2.10E-06 |
| ebi-a-GCST90001531 | rs116166244 | 5 | 110463783 | C | 8.00E-04 | 2.675 | 0.5751 | 3.55E-06 |
| ebi-a-GCST90001532 | rs116166244 | 5 | 110463783 | C | 8.00E-04 | 2.755 | 0.5729 | 1.64E-06 |
| ebi-a-GCST90001728 | rs11617402 | 13 | 22970185 | C | 0.0235 | -0.517 | 0.1112 | 3.58E-06 |
| ebi-a-GCST90001775 | rs11617559 | 13 | 24752225 | C | 0.5725 | 0.1143 | 0.02495 | 4.79E-06 |
| ebi-a-GCST90001631 | rs116179687 | 13 | 108490419 | T | 0.0046 | -0.8148 | 0.177 | 4.27E-06 |
| ebi-a-GCST90001742 | rs116180572 | 3 | 61084498 | T | 0.1325 | 0.1959 | 0.03689 | 1.16E-07 |
| ebi-a-GCST90001565 | rs116181048 | 2 | 208596366 | A | 0.0041 | 0.7854 | 0.1713 | 4.69E-06 |
| ebi-a-GCST90001520 | rs11618675 | 13 | 49307675 | A | 0.025 | 0.4989 | 0.1078 | 3.94E-06 |
| ebi-a-GCST90001980 | rs116203109 | 2 | 5943436 | A | 0.0355 | -0.3052 | 0.06544 | 3.21E-06 |
| ebi-a-GCST90002055 | rs116211259 | 4 | 152943585 | A | 0.005 | -0.8861 | 0.191 | 3.65E-06 |
| ebi-a-GCST90001820 | rs11621990 | 14 | 106875768 | T | 0.4199 | -0.2438 | 0.04375 | 2.68E-08 |
| ebi-a-GCST90001978 | rs116220030 | 4 | 68602634 | A | 0.0196 | -0.4245 | 0.09243 | 4.56E-06 |
| ebi-a-GCST90001992 | rs116231488 | 5 | 101620120 | G | 8.00E-04 | 2.043 | 0.4266 | 1.74E-06 |
| ebi-a-GCST90001626 | rs116253486 | 3 | 33925087 | C | 0.0015 | -1.26 | 0.273 | 4.06E-06 |
| ebi-a-GCST90001864 | rs116266532 | 4 | 42485295 | C | 0.0473 | 0.3159 | 0.06429 | 9.45E-07 |
| ebi-a-GCST90001782 | rs116270586 | 3 | 121050031 | G | 0.0131 | 0.6723 | 0.1428 | 2.68E-06 |
| ebi-a-GCST90001644 | rs11627066 | 14 | 66304049 | T | 0.4981 | -0.1185 | 0.02483 | 1.90E-06 |
| ebi-a-GCST90001642 | rs11627066 | 14 | 66304049 | T | 0.4989 | -0.1147 | 0.02444 | 2.81E-06 |
| ebi-a-GCST90001588 | rs11627066 | 14 | 66304049 | T | 0.4989 | 0.1182 | 0.02498 | 2.30E-06 |
| ebi-a-GCST90002006 | rs116275309 | 1 | 150863650 | A | 0.0133 | 0.8229 | 0.1104 | 1.15E-13 |
| ebi-a-GCST90001987 | rs116275309 | 1 | 150863650 | A | 0.0133 | 0.8426 | 0.1098 | 2.16E-14 |
| ebi-a-GCST90001392 | rs116285595 | 1 | 200858159 | T | 0.0068 | 0.6503 | 0.1415 | 4.44E-06 |
| ebi-a-GCST90001577 | rs116285595 | 1 | 200858159 | T | 0.0068 | 0.6676 | 0.1427 | 2.98E-06 |
| ebi-a-GCST90001894 | rs116298583 | 4 | 24489778 | T | 0.1384 | 0.1936 | 0.03974 | 1.17E-06 |
| ebi-a-GCST90001562 | rs11629948 | 15 | 65376824 | A | 0.1358 | 0.1585 | 0.03288 | 1.49E-06 |
| ebi-a-GCST90001937 | rs11631647 | 15 | 63256904 | A | 0.4397 | 0.1264 | 0.02578 | 9.96E-07 |
| ebi-a-GCST90001475 | rs116322630 | 6 | 15221128 | T | 0.0633 | 0.2678 | 0.05377 | 6.64E-07 |
| ebi-a-GCST90001790 | rs11632374 | 15 | 33478617 | G | 0.3404 | 0.1232 | 0.02601 | 2.25E-06 |
| ebi-a-GCST90001804 | rs116327214 | 1 | 32083093 | G | 0.0291 | -0.351 | 0.07197 | 1.12E-06 |
| ebi-a-GCST90001698 | rs116349768 | 3 | 84476620 | G | 0.0031 | 169.7 | 32.81 | 2.46E-07 |
| ebi-a-GCST90001571 | rs11635763 | 15 | 27177182 | G | 0.4234 | 0.1159 | 0.02481 | 3.09E-06 |
| ebi-a-GCST90001741 | rs116364947 | 6 | 36809211 | T | 0.0134 | 0.4837 | 0.1051 | 4.34E-06 |
| ebi-a-GCST90001740 | rs116364947 | 6 | 36809211 | T | 0.0134 | 0.5306 | 0.1052 | 4.80E-07 |
| ebi-a-GCST90001963 | rs1163654 | 13 | 112246767 | C | 0.5113 | 0.1338 | 0.02757 | 1.28E-06 |
| ebi-a-GCST90001958 | rs116366652 | 2 | 160856904 | T | 0.1448 | -0.1783 | 0.03868 | 4.20E-06 |
| ebi-a-GCST90001785 | rs11636705 | 15 | 96605912 | T | 0.1322 | 0.1676 | 0.03665 | 4.99E-06 |
| ebi-a-GCST90002039 | rs116371507 | 3 | 52895436 | T | 0.0139 | -0.5628 | 0.1216 | 3.85E-06 |
| ebi-a-GCST90002018 | rs11637579 | 15 | 52729171 | T | 0.0156 | 0.5237 | 0.1124 | 3.29E-06 |
| ebi-a-GCST90001400 | rs11638959 | 15 | 25171150 | A | 0.1557 | 0.1525 | 0.03281 | 3.47E-06 |
| ebi-a-GCST90001751 | rs116393510 | 2 | 200547937 | T | 0.0328 | -0.3197 | 0.06762 | 2.36E-06 |
| ebi-a-GCST90002077 | rs11639511 | 16 | 10977844 | G | 0.7661 | 0.148 | 0.03108 | 1.99E-06 |
| ebi-a-GCST90001700 | rs116399750 | 4 | 176754667 | A | 6.00E-04 | 17.96 | 3.205 | 2.27E-08 |
| ebi-a-GCST90001698 | rs116399750 | 4 | 176754667 | A | 6.00E-04 | 889.9 | 73.93 | 1.04E-32 |
| ebi-a-GCST90002015 | rs116403890 | 4 | 189033600 | T | 0.0244 | -0.4448 | 0.08991 | 7.97E-07 |
| ebi-a-GCST90002016 | rs116403890 | 4 | 189033600 | T | 0.0244 | -0.4401 | 0.08979 | 1.01E-06 |
| ebi-a-GCST90001818 | rs1164064 | 3 | 109462400 | A | 0.4333 | 0.1198 | 0.02527 | 2.23E-06 |
| ebi-a-GCST90001934 | rs116411520 | 10 | 91549231 | A | 0.0266 | -0.3549 | 0.0748 | 2.18E-06 |
| ebi-a-GCST90001625 | rs11641185 | 16 | 10986596 | T | 0.7744 | -0.2989 | 0.02511 | 4.52E-32 |
| ebi-a-GCST90001732 | rs116412781 | 4 | 2746567 | T | 0.0475 | 0.2691 | 0.05837 | 4.18E-06 |
| ebi-a-GCST90002066 | rs116421623 | 8 | 119192827 | T | 0.0089 | -0.7415 | 0.1461 | 4.13E-07 |
| ebi-a-GCST90002070 | rs116421623 | 8 | 119192827 | T | 0.0089 | -0.6912 | 0.1469 | 2.67E-06 |
| ebi-a-GCST90002067 | rs116421623 | 8 | 119192827 | T | 0.0089 | -0.6839 | 0.1459 | 2.89E-06 |
| ebi-a-GCST90001928 | rs116423144 | 5 | 174310054 | A | 0.0077 | 0.8507 | 0.1494 | 1.37E-08 |
| ebi-a-GCST90002089 | rs11642757 | 16 | 79753467 | A | 0.45 | 0.1384 | 0.02744 | 4.88E-07 |
| ebi-a-GCST90001698 | rs116431337 | 6 | 66416572 | C | 0.0043 | 159.2 | 28.32 | 2.05E-08 |
| ebi-a-GCST90001738 | rs11643297 | 16 | 28988269 | G | 0.6109 | 0.1375 | 0.02549 | 7.32E-08 |
| ebi-a-GCST90001736 | rs11643297 | 16 | 28988269 | G | 0.611 | 0.1475 | 0.02551 | 7.89E-09 |
| ebi-a-GCST90001724 | rs11643297 | 16 | 28988269 | G | 0.6109 | 0.148 | 0.02557 | 7.82E-09 |
| ebi-a-GCST90001723 | rs11643297 | 16 | 28988269 | G | 0.6109 | 0.1545 | 0.02548 | 1.45E-09 |
| ebi-a-GCST90001735 | rs11643297 | 16 | 28988269 | G | 0.611 | 0.167 | 0.02538 | 5.48E-11 |
| ebi-a-GCST90002105 | rs11644019 | 16 | 10863414 | T | 0.1398 | -0.291 | 0.03898 | 1.10E-13 |
| ebi-a-GCST90002106 | rs11644019 | 16 | 10863414 | T | 0.1398 | -0.1978 | 0.04003 | 8.18E-07 |
| ebi-a-GCST90001698 | rs116446467 | 1 | 187795006 | G | 7.00E-04 | 621.6 | 61.6 | 1.28E-23 |
| ebi-a-GCST90001950 | rs1164675 | 11 | 49325972 | T | 0.2484 | 0.2294 | 0.04845 | 2.44E-06 |
| ebi-a-GCST90001704 | rs116470335 | 4 | 24492147 | A | 0.0016 | 1.508 | 0.3285 | 4.54E-06 |
| ebi-a-GCST90001714 | rs116470335 | 4 | 24492147 | A | 0.0016 | 1.508 | 0.3297 | 4.93E-06 |
| ebi-a-GCST90001709 | rs116470335 | 4 | 24492147 | A | 0.0016 | 1.513 | 0.3287 | 4.32E-06 |
| ebi-a-GCST90001712 | rs116470335 | 4 | 24492147 | A | 0.0016 | 1.518 | 0.3289 | 4.06E-06 |
| ebi-a-GCST90001829 | rs116470335 | 4 | 24492147 | A | 0.0016 | 1.525 | 0.329 | 3.66E-06 |
| ebi-a-GCST90001703 | rs116470335 | 4 | 24492147 | A | 0.0016 | 1.543 | 0.329 | 2.84E-06 |
| ebi-a-GCST90001702 | rs116470335 | 4 | 24492147 | A | 0.0016 | 1.559 | 0.3291 | 2.25E-06 |
| ebi-a-GCST90001717 | rs116470335 | 4 | 24492147 | A | 0.0016 | 1.566 | 0.3288 | 1.98E-06 |
| ebi-a-GCST90001705 | rs116470335 | 4 | 24492147 | A | 0.0016 | 1.591 | 0.3278 | 1.26E-06 |
| ebi-a-GCST90001715 | rs116470335 | 4 | 24492147 | A | 0.0016 | 1.618 | 0.3287 | 8.86E-07 |
| ebi-a-GCST90001399 | rs116473015 | 2 | 147859010 | T | 0.029 | 0.3455 | 0.07401 | 3.14E-06 |
| ebi-a-GCST90001665 | rs116478736 | 2 | 18033652 | G | 0.0094 | 0.5926 | 0.1296 | 4.97E-06 |
| ebi-a-GCST90001887 | rs116479405 | 2 | 49884156 | A | 0.0164 | -0.4977 | 0.1035 | 1.60E-06 |
| ebi-a-GCST90001889 | rs116479405 | 2 | 49884156 | A | 0.0164 | -0.4897 | 0.1012 | 1.36E-06 |
| ebi-a-GCST90001698 | rs116498781 | 4 | 183314518 | A | 0.0015 | 268.3 | 44.19 | 1.40E-09 |
| ebi-a-GCST90001904 | rs116499957 | 4 | 23334338 | T | 0.0152 | -0.513 | 0.1113 | 4.21E-06 |
| ebi-a-GCST90001631 | rs11650003 | 17 | 7866604 | G | 0.3867 | -0.116 | 0.0252 | 4.32E-06 |
| ebi-a-GCST90001698 | rs116510159 | 1 | 195636177 | C | 0.0022 | 297 | 38.79 | 2.47E-14 |
| ebi-a-GCST90001549 | rs116515477 | 3 | 68134416 | G | 0.0239 | 0.4004 | 0.0829 | 1.42E-06 |
| ebi-a-GCST90002115 | rs11651658 | 17 | 64215253 | C | 0.0623 | 0.2484 | 0.05207 | 1.92E-06 |
| ebi-a-GCST90002100 | rs116517602 | 1 | 198922068 | T | 0.0041 | 1.147 | 0.201 | 1.29E-08 |
| ebi-a-GCST90001507 | rs116517602 | 1 | 198922068 | T | 0.0041 | 1.722 | 0.1913 | 3.67E-19 |
| ebi-a-GCST90001509 | rs116517602 | 1 | 198922068 | T | 0.0041 | 1.875 | 0.1906 | 1.50E-22 |
| ebi-a-GCST90001508 | rs116517602 | 1 | 198922068 | T | 0.0041 | 1.941 | 0.1905 | 4.76E-24 |
| ebi-a-GCST90002115 | rs11651897 | 17 | 64739971 | G | 0.0373 | 0.3236 | 0.06724 | 1.57E-06 |
| ebi-a-GCST90001698 | rs116521668 | 3 | 43905713 | A | 0.0103 | 80.23 | 17.28 | 3.55E-06 |
| ebi-a-GCST90001798 | rs116522219 | 3 | 36625286 | A | 0.0407 | 0.2952 | 0.06225 | 2.19E-06 |
| ebi-a-GCST90001621 | rs11652532 | 17 | 33769883 | A | 0.1574 | -0.1848 | 0.0342 | 7.00E-08 |
| ebi-a-GCST90001596 | rs11652532 | 17 | 33769883 | A | 0.1574 | -0.1564 | 0.0331 | 2.38E-06 |
| ebi-a-GCST90001784 | rs116525939 | 5 | 97611924 | C | 0.0036 | 0.954 | 0.2076 | 4.47E-06 |
| ebi-a-GCST90001553 | rs11652705 | 17 | 41761741 | G | 0.1707 | -0.1202 | 0.02264 | 1.16E-07 |
| ebi-a-GCST90001688 | rs11652705 | 17 | 41761741 | G | 0.1709 | -0.1174 | 0.02339 | 5.52E-07 |
| ebi-a-GCST90001551 | rs11652705 | 17 | 41761741 | G | 0.1705 | -0.116 | 0.02274 | 3.60E-07 |
| ebi-a-GCST90002030 | rs11653180 | 17 | 3689318 | A | 0.044 | -0.298 | 0.06465 | 4.23E-06 |
| ebi-a-GCST90001519 | rs116534482 | 2 | 142334140 | C | 0.0342 | 0.4192 | 0.09019 | 3.58E-06 |
| ebi-a-GCST90001906 | rs11653491 | 17 | 67953507 | G | 0.3309 | 0.1575 | 0.0299 | 1.48E-07 |
| ebi-a-GCST90001698 | rs116536232 | 4 | 67329531 | G | 0.0062 | 118.7 | 22.96 | 2.48E-07 |
| ebi-a-GCST90001434 | rs11653761 | 17 | 2716535 | T | 0.592 | -0.1491 | 0.02489 | 2.30E-09 |
| ebi-a-GCST90001436 | rs11653761 | 17 | 2716535 | T | 0.592 | -0.1359 | 0.02451 | 3.17E-08 |
| ebi-a-GCST90001403 | rs11653761 | 17 | 2716535 | T | 0.592 | -0.1273 | 0.02469 | 2.65E-07 |
| ebi-a-GCST90001426 | rs11653761 | 17 | 2716535 | T | 0.592 | -0.1257 | 0.02411 | 1.93E-07 |
| ebi-a-GCST90001412 | rs11653761 | 17 | 2716535 | T | 0.592 | -0.125 | 0.02429 | 2.83E-07 |
| ebi-a-GCST90001400 | rs11653761 | 17 | 2716535 | T | 0.5919 | -0.1212 | 0.02449 | 7.77E-07 |
| ebi-a-GCST90001418 | rs11653761 | 17 | 2716535 | T | 0.592 | -0.118 | 0.0247 | 1.84E-06 |
| ebi-a-GCST90001427 | rs11653761 | 17 | 2716535 | T | 0.592 | -0.1169 | 0.0249 | 2.78E-06 |
| ebi-a-GCST90001407 | rs11653761 | 17 | 2716535 | T | 0.592 | -0.1161 | 0.02478 | 2.94E-06 |
| ebi-a-GCST90001438 | rs11653761 | 17 | 2716535 | T | 0.592 | -0.1121 | 0.02405 | 3.29E-06 |
| ebi-a-GCST90001435 | rs116540222 | 3 | 117331256 | T | 0.0011 | 1.756 | 0.3749 | 2.92E-06 |
| ebi-a-GCST90001786 | rs116540618 | 19 | 49487555 | C | 0.0178 | -0.4243 | 0.092 | 4.12E-06 |
| ebi-a-GCST90001800 | rs116551266 | 3 | 36350049 | T | 0.0718 | 0.2378 | 0.04954 | 1.65E-06 |
| ebi-a-GCST90002062 | rs11655205 | 17 | 6630904 | A | 0.1353 | 0.1915 | 0.04082 | 2.84E-06 |
| ebi-a-GCST90001495 | rs11655883 | 17 | 75234757 | G | 0.2902 | 0.1294 | 0.02794 | 3.74E-06 |
| ebi-a-GCST90001553 | rs116562707 | 6 | 31360602 | G | 0.0191 | 0.3952 | 0.06626 | 2.71E-09 |
| ebi-a-GCST90001688 | rs116562707 | 6 | 31360602 | G | 0.0192 | 0.4024 | 0.06819 | 3.97E-09 |
| ebi-a-GCST90001439 | rs11657118 | 17 | 2714118 | C | 0.475 | -0.1428 | 0.02416 | 3.70E-09 |
| ebi-a-GCST90001398 | rs11657118 | 17 | 2714118 | C | 0.475 | -0.1112 | 0.02412 | 4.21E-06 |
| ebi-a-GCST90001424 | rs11657531 | 17 | 2731370 | T | 0.4882 | -0.1468 | 0.0236 | 5.52E-10 |
| ebi-a-GCST90001430 | rs11657531 | 17 | 2731370 | T | 0.4884 | -0.1167 | 0.0237 | 8.84E-07 |
| ebi-a-GCST90001578 | rs11657531 | 17 | 2731370 | T | 0.4884 | -0.1135 | 0.02363 | 1.65E-06 |
| ebi-a-GCST90001442 | rs11657759 | 17 | 2715961 | C | 0.4903 | -0.1352 | 0.02397 | 1.81E-08 |
| ebi-a-GCST90001432 | rs11657759 | 17 | 2715961 | C | 0.4903 | -0.1271 | 0.02394 | 1.16E-07 |
| ebi-a-GCST90001659 | rs11658050 | 17 | 75220287 | T | 0.2094 | 0.1408 | 0.02921 | 1.50E-06 |
| ebi-a-GCST90001658 | rs11658050 | 17 | 75220287 | T | 0.2094 | 0.1439 | 0.0292 | 8.71E-07 |
| ebi-a-GCST90001700 | rs116581718 | 5 | 29413612 | C | 6.00E-04 | 22.4 | 3.301 | 1.35E-11 |
| ebi-a-GCST90001695 | rs116581718 | 5 | 29413612 | C | 6.00E-04 | 384.3 | 58.05 | 4.14E-11 |
| ebi-a-GCST90001698 | rs116581718 | 5 | 29413612 | C | 6.00E-04 | 1006 | 78.94 | 2.29E-36 |
| ebi-a-GCST90001399 | rs11658240 | 17 | 80484139 | G | 0.1362 | -0.1678 | 0.03574 | 2.77E-06 |
| ebi-a-GCST90001630 | rs11658693 | 17 | 33800249 | G | 0.1123 | -0.2038 | 0.03962 | 2.84E-07 |
| ebi-a-GCST90001796 | rs116588562 | 1 | 201089372 | A | 0.0718 | 0.2247 | 0.04697 | 1.79E-06 |
| ebi-a-GCST90001631 | rs11658954 | 17 | 74953620 | G | 0.6265 | -0.1133 | 0.02456 | 4.12E-06 |
| ebi-a-GCST90001698 | rs116589647 | 3 | 122070556 | T | 0.0023 | 212.9 | 34.36 | 6.51E-10 |
| ebi-a-GCST90001553 | rs116590755 | 2 | 173592729 | T | 3.00E-04 | -2.257 | 0.4624 | 1.10E-06 |
| ebi-a-GCST90001551 | rs116590755 | 2 | 173592729 | T | 3.00E-04 | -2.234 | 0.465 | 1.62E-06 |
| ebi-a-GCST90001470 | rs11659751 | 18 | 55736723 | A | 0.0772 | 0.209 | 0.04558 | 4.71E-06 |
| ebi-a-GCST90002104 | rs116607596 | 3 | 125955606 | G | 0.0696 | 0.2553 | 0.05443 | 2.85E-06 |
| ebi-a-GCST90001698 | rs11661882 | 18 | 70504468 | A | 0.9974 | -233.6 | 40.25 | 7.08E-09 |
| ebi-a-GCST90002110 | rs116632210 | 6 | 37856213 | G | 0.0483 | -0.4023 | 0.0854 | 2.68E-06 |
| ebi-a-GCST90001538 | rs116640816 | 1 | 233503332 | T | 0.0219 | 0.4028 | 0.08536 | 2.47E-06 |
| ebi-a-GCST90001825 | rs116643893 | 4 | 137149414 | A | 0.001 | -1.898 | 0.3902 | 1.20E-06 |
| ebi-a-GCST90002029 | rs116646957 | 2 | 115975810 | G | 0.0055 | 0.8837 | 0.1926 | 4.63E-06 |
| ebi-a-GCST90001907 | rs11665125 | 18 | 33295639 | T | 0.0771 | -0.2745 | 0.05196 | 1.37E-07 |
| ebi-a-GCST90001579 | rs116662407 | 1 | 18064955 | C | 0.0065 | 0.6791 | 0.1479 | 4.53E-06 |
| ebi-a-GCST90001475 | rs116670542 | 2 | 217877436 | A | 8.00E-04 | -1.812 | 0.3917 | 3.85E-06 |
| ebi-a-GCST90001984 | rs116671810 | 6 | 31450274 | A | 0.0711 | 0.3239 | 0.05515 | 4.66E-09 |
| ebi-a-GCST90001814 | rs11668091 | 19 | 51583553 | C | 0.139 | -0.1631 | 0.03479 | 2.87E-06 |
| ebi-a-GCST90002103 | rs116682768 | 3 | 22257995 | A | 0.0047 | 0.9547 | 0.1991 | 1.72E-06 |
| ebi-a-GCST90002009 | rs116683794 | 6 | 33034114 | G | 0.096 | 0.3299 | 0.04603 | 9.18E-13 |
| ebi-a-GCST90002040 | rs116690299 | 3 | 16516486 | A | 0.0282 | -0.3941 | 0.08446 | 3.20E-06 |
| ebi-a-GCST90001953 | rs11669394 | 19 | 53262327 | A | 0.3039 | -0.2604 | 0.0392 | 4.22E-11 |
| ebi-a-GCST90002069 | rs116694668 | 5 | 57174385 | A | 0.001 | -2.068 | 0.4319 | 1.77E-06 |
| ebi-a-GCST90001421 | rs116699251 | 5 | 142419461 | A | 0.0446 | -0.2732 | 0.05861 | 3.24E-06 |
| ebi-a-GCST90001928 | rs11670885 | 19 | 50552467 | A | 0.0702 | 0.2456 | 0.05303 | 3.80E-06 |
| ebi-a-GCST90001698 | rs116719160 | 3 | 130201546 | T | 0.0012 | 420 | 62.49 | 2.10E-11 |
| ebi-a-GCST90001588 | rs116735405 | 10 | 119550308 | C | 0.0266 | 0.354 | 0.0754 | 2.77E-06 |
| ebi-a-GCST90001782 | rs11673894 | 2 | 118876072 | T | 0.1892 | -0.1962 | 0.04266 | 4.54E-06 |
| ebi-a-GCST90001922 | rs116757507 | 17 | 5844216 | T | 0.0361 | -0.337 | 0.07069 | 1.96E-06 |
| ebi-a-GCST90001846 | rs116757920 | 1 | 117441813 | A | 0.0225 | 0.4153 | 0.08942 | 3.57E-06 |
| ebi-a-GCST90001859 | rs116757920 | 1 | 117441813 | A | 0.0224 | 0.4338 | 0.09072 | 1.83E-06 |
| ebi-a-GCST90001667 | rs116759767 | 1 | 189981116 | A | 0.0049 | -0.9923 | 0.1747 | 1.45E-08 |
| ebi-a-GCST90001555 | rs116760504 | 3 | 47546791 | A | 0.0696 | -0.2168 | 0.04308 | 5.09E-07 |
| ebi-a-GCST90001665 | rs116760504 | 3 | 47546791 | A | 0.0696 | 0.2365 | 0.04969 | 2.02E-06 |
| ebi-a-GCST90001919 | rs11676095 | 2 | 206656142 | T | 0.0668 | -0.2582 | 0.05405 | 1.86E-06 |
| ebi-a-GCST90001545 | rs116761394 | 3 | 122030442 | T | 0.0627 | 0.2448 | 0.05306 | 4.08E-06 |
| ebi-a-GCST90001596 | rs11676464 | 2 | 86018262 | A | 0.1088 | -0.1862 | 0.03849 | 1.37E-06 |
| ebi-a-GCST90001633 | rs11676464 | 2 | 86018262 | A | 0.1088 | -0.1853 | 0.0392 | 2.37E-06 |
| ebi-a-GCST90001688 | rs116767883 | 6 | 24745313 | T | NA | 9.1 | 1.583 | 9.88E-09 |
| ebi-a-GCST90002006 | rs116770889 | 1 | 116993158 | T | 0.0106 | -0.5648 | 0.1211 | 3.20E-06 |
| ebi-a-GCST90001893 | rs116778614 | 3 | 834951 | G | 0.018 | -0.5313 | 0.103 | 2.66E-07 |
| ebi-a-GCST90001695 | rs116779558 | 3 | 77069014 | G | 7.00E-04 | 248.4 | 47.12 | 1.44E-07 |
| ebi-a-GCST90001493 | rs116794175 | 1 | 194010208 | C | 0.0042 | -1.417 | 0.1841 | 1.78E-14 |
| ebi-a-GCST90001511 | rs116794175 | 1 | 194010208 | C | 0.0042 | -1.101 | 0.1969 | 2.46E-08 |
| ebi-a-GCST90001691 | rs116794175 | 1 | 194010208 | C | 0.0042 | -1.073 | 0.1963 | 4.99E-08 |
| ebi-a-GCST90001512 | rs116794175 | 1 | 194010208 | C | 0.0042 | -0.9442 | 0.197 | 1.72E-06 |
| ebi-a-GCST90001555 | rs116794175 | 1 | 194010208 | C | 0.0042 | -0.7915 | 0.1661 | 1.96E-06 |
| ebi-a-GCST90001561 | rs116794175 | 1 | 194010208 | C | 0.0042 | 0.8233 | 0.1666 | 8.11E-07 |
| ebi-a-GCST90001480 | rs116794175 | 1 | 194010208 | C | 0.0043 | 0.8994 | 0.1806 | 6.68E-07 |
| ebi-a-GCST90001500 | rs116794175 | 1 | 194010208 | C | 0.0042 | 0.9735 | 0.1912 | 3.75E-07 |
| ebi-a-GCST90001666 | rs116794175 | 1 | 194010208 | C | 0.0043 | 1.018 | 0.1715 | 3.23E-09 |
| ebi-a-GCST90001507 | rs116794175 | 1 | 194010208 | C | 0.0043 | 1.029 | 0.1996 | 2.65E-07 |
| ebi-a-GCST90001664 | rs116794175 | 1 | 194010208 | C | 0.0042 | 1.164 | 0.1805 | 1.30E-10 |
| ebi-a-GCST90001665 | rs116794175 | 1 | 194010208 | C | 0.0042 | 1.191 | 0.1918 | 5.92E-10 |
| ebi-a-GCST90001485 | rs116794175 | 1 | 194010208 | C | 0.0042 | 1.311 | 0.1905 | 6.98E-12 |
| ebi-a-GCST90001499 | rs116794175 | 1 | 194010208 | C | 0.0042 | 1.427 | 0.1851 | 1.62E-14 |
| ebi-a-GCST90002013 | rs116800975 | 1 | 162238738 | A | 0.0474 | 0.3008 | 0.0656 | 4.71E-06 |
| ebi-a-GCST90001697 | rs116802453 | 4 | 159714312 | G | 4.00E-04 | 13.13 | 2.827 | 3.56E-06 |
| ebi-a-GCST90001695 | rs116807650 | 4 | 112605741 | T | 7.00E-04 | 276.6 | 51.66 | 9.20E-08 |
| ebi-a-GCST90002033 | rs116817680 | 4 | 20867272 | A | 0.0152 | 0.5343 | 0.1145 | 3.24E-06 |
| ebi-a-GCST90001429 | rs11682311 | 2 | 16464977 | C | 0.3243 | -0.1278 | 0.02599 | 9.11E-07 |
| ebi-a-GCST90001878 | rs116838007 | 2 | 241352278 | A | 0.0209 | 0.6668 | 0.1453 | 4.88E-06 |
| ebi-a-GCST90001415 | rs116844769 | 14 | 65713895 | A | 0.0662 | -0.2773 | 0.0514 | 7.30E-08 |
| ebi-a-GCST90001408 | rs116844769 | 14 | 65713895 | A | 0.0662 | -0.2677 | 0.05076 | 1.42E-07 |
| ebi-a-GCST90001430 | rs116844769 | 14 | 65713895 | A | 0.0662 | -0.2347 | 0.04969 | 2.40E-06 |
| ebi-a-GCST90001437 | rs116844769 | 14 | 65713895 | A | 0.0662 | -0.2342 | 0.05066 | 3.92E-06 |
| ebi-a-GCST90001441 | rs116844769 | 14 | 65713895 | A | 0.0662 | -0.232 | 0.04979 | 3.28E-06 |
| ebi-a-GCST90001417 | rs116844769 | 14 | 65713895 | A | 0.0662 | 0.2482 | 0.05078 | 1.06E-06 |
| ebi-a-GCST90001406 | rs116844769 | 14 | 65713895 | A | 0.0662 | 0.2839 | 0.05066 | 2.26E-08 |
| ebi-a-GCST90001695 | rs116848911 | 14 | 79042294 | A | 0.0029 | 110 | 22.52 | 1.09E-06 |
| ebi-a-GCST90002093 | rs116849735 | 12 | 44943100 | C | 0.072 | -0.3234 | 0.07042 | 4.76E-06 |
| ebi-a-GCST90001502 | rs116850494 | 13 | 60389778 | A | 0.0145 | -0.4419 | 0.09434 | 2.92E-06 |
| ebi-a-GCST90001522 | rs116851051 | 20 | 15544052 | C | 0.014 | -0.648 | 0.1415 | 4.97E-06 |
| ebi-a-GCST90001394 | rs116851238 | 10 | 33792014 | T | 0.0176 | -0.4319 | 0.08996 | 1.64E-06 |
| ebi-a-GCST90001698 | rs116853391 | 14 | 104409277 | C | 0.0023 | 192.4 | 36.48 | 1.42E-07 |
| ebi-a-GCST90001722 | rs116862171 | 6 | 112145789 | G | 0.0218 | -0.3796 | 0.08302 | 4.97E-06 |
| ebi-a-GCST90001836 | rs116864786 | 18 | 39475934 | C | 0.0235 | 0.5904 | 0.1252 | 2.65E-06 |
| ebi-a-GCST90001695 | rs116865617 | 19 | 14535355 | C | 0.0029 | 120.1 | 25.31 | 2.16E-06 |
| ebi-a-GCST90001856 | rs11686866 | 2 | 88740296 | A | 0.211 | -0.1526 | 0.03328 | 4.76E-06 |
| ebi-a-GCST90002021 | rs11687130 | 2 | 56343677 | G | 0.0915 | -0.2867 | 0.06185 | 3.86E-06 |
| ebi-a-GCST90001467 | rs116872766 | 8 | 77883134 | C | 0.0045 | -0.9025 | 0.1818 | 7.20E-07 |
| ebi-a-GCST90002089 | rs116873976 | 17 | 21255388 | A | 9.00E-04 | -2.463 | 0.5227 | 2.57E-06 |
| ebi-a-GCST90001426 | rs11687597 | 2 | 136926146 | G | 0.2069 | 0.1644 | 0.03007 | 4.86E-08 |
| ebi-a-GCST90001697 | rs116876186 | 17 | 16446446 | C | 1.00E-04 | 22.19 | 4.608 | 1.52E-06 |
| ebi-a-GCST90001695 | rs116876186 | 17 | 16446446 | C | 1.00E-04 | 647.9 | 98.9 | 6.57E-11 |
| ebi-a-GCST90001573 | rs116877116 | 8 | 135490341 | A | 0.1008 | -0.2314 | 0.04239 | 5.15E-08 |
| ebi-a-GCST90001571 | rs116877116 | 8 | 135490341 | A | 0.1008 | 0.1973 | 0.04041 | 1.09E-06 |
| ebi-a-GCST90001570 | rs116877116 | 8 | 135490341 | A | 0.1008 | 0.2343 | 0.04232 | 3.32E-08 |
| ebi-a-GCST90001669 | rs116884829 | 11 | 77173517 | G | 0.132 | 0.1788 | 0.03398 | 1.50E-07 |
| ebi-a-GCST90001693 | rs116884829 | 11 | 77173517 | G | 0.132 | 0.1826 | 0.03834 | 1.99E-06 |
| ebi-a-GCST90001401 | rs11688492 | 2 | 64865804 | C | 0.5196 | -0.1216 | 0.02504 | 1.24E-06 |
| ebi-a-GCST90001470 | rs116894787 | 10 | 12240106 | C | 0.0111 | 0.5376 | 0.1152 | 3.19E-06 |
| ebi-a-GCST90001708 | rs116897208 | 10 | 15100608 | C | 0.0022 | -1.261 | 0.2724 | 3.79E-06 |
| ebi-a-GCST90002043 | rs116900648 | 8 | 144503650 | A | 0.0089 | -0.978 | 0.2007 | 1.21E-06 |
| ebi-a-GCST90001424 | rs116906133 | 13 | 100023905 | G | 0.0037 | -1.032 | 0.2178 | 2.22E-06 |
| ebi-a-GCST90001469 | rs116910026 | 11 | 19877912 | T | 0.0512 | 0.273 | 0.05712 | 1.82E-06 |
| ebi-a-GCST90001462 | rs116910026 | 11 | 19877912 | T | 0.0512 | 0.2767 | 0.05667 | 1.09E-06 |
| ebi-a-GCST90001468 | rs116910026 | 11 | 19877912 | T | 0.0512 | 0.2902 | 0.05778 | 5.39E-07 |
| ebi-a-GCST90001700 | rs116915023 | 18 | 62442252 | T | 0.0016 | 10.65 | 1.782 | 2.50E-09 |
| ebi-a-GCST90001700 | rs116916598 | 11 | 121746998 | G | 6.00E-04 | 19.07 | 3.407 | 2.33E-08 |
| ebi-a-GCST90001698 | rs116916598 | 11 | 121746998 | G | 6.00E-04 | 834.4 | 80.31 | 6.48E-25 |
| ebi-a-GCST90001698 | rs116921833 | 7 | 153895185 | T | 0.0065 | 126.2 | 21.64 | 5.99E-09 |
| ebi-a-GCST90001523 | rs116926801 | 17 | 65652250 | T | 0.021 | -0.5549 | 0.1175 | 2.52E-06 |
| ebi-a-GCST90001933 | rs116928928 | 11 | 32030595 | C | 0.0409 | -0.3318 | 0.06563 | 4.52E-07 |
| ebi-a-GCST90001511 | rs116928928 | 11 | 32030595 | C | 0.0407 | -0.2998 | 0.06526 | 4.52E-06 |
| ebi-a-GCST90001505 | rs116928928 | 11 | 32030595 | C | 0.0407 | -0.2994 | 0.06501 | 4.27E-06 |
| ebi-a-GCST90001698 | rs116940971 | 18 | 38279589 | T | 0.001 | 469.1 | 52.88 | 1.14E-18 |
| ebi-a-GCST90001695 | rs116941636 | 16 | 79757046 | A | 0.0019 | 210.2 | 30.1 | 3.44E-12 |
| ebi-a-GCST90001787 | rs11694298 | 2 | 11175178 | A | 0.6849 | 0.1206 | 0.02623 | 4.40E-06 |
| ebi-a-GCST90001556 | rs116944484 | 10 | 83233799 | A | 7.00E-04 | -2.133 | 0.4227 | 4.77E-07 |
| ebi-a-GCST90001553 | rs116954000 | 22 | 40298491 | A | 0.0034 | 0.6951 | 0.141 | 8.59E-07 |
| ebi-a-GCST90001688 | rs116954000 | 22 | 40298491 | A | 0.0033 | 0.7802 | 0.1463 | 1.04E-07 |
| ebi-a-GCST90001562 | rs116954000 | 22 | 40298491 | A | 0.0034 | 0.916 | 0.1866 | 9.60E-07 |
| ebi-a-GCST90001523 | rs11695642 | 2 | 43952695 | T | 0.4325 | -0.1588 | 0.03437 | 4.10E-06 |
| ebi-a-GCST90001695 | rs116959045 | 8 | 53929771 | G | 0.0022 | 172.7 | 28.26 | 1.12E-09 |
| ebi-a-GCST90001695 | rs116960471 | 8 | 1549975 | T | 3.00E-04 | 447.9 | 85.71 | 1.84E-07 |
| ebi-a-GCST90002041 | rs11696662 | 20 | 39790435 | T | 0.0972 | 0.2795 | 0.05921 | 2.56E-06 |
| ebi-a-GCST90001862 | rs116969543 | 8 | 99091321 | C | 0.0084 | 0.7409 | 0.1569 | 2.46E-06 |
| ebi-a-GCST90002089 | rs116977022 | 8 | 131045527 | T | 0.0046 | 0.926 | 0.1986 | 3.25E-06 |
| ebi-a-GCST90001700 | rs116979366 | 7 | 31134950 | A | 0.0041 | 5.888 | 1.203 | 1.04E-06 |
| ebi-a-GCST90001510 | rs11697981 | 20 | 43093014 | C | 0.132 | 0.1755 | 0.03802 | 4.06E-06 |
| ebi-a-GCST90001504 | rs11697981 | 20 | 43093014 | C | 0.132 | 0.1763 | 0.03779 | 3.21E-06 |
| ebi-a-GCST90001583 | rs116981568 | 13 | 28964708 | T | 0.0477 | 0.2687 | 0.05573 | 1.48E-06 |
| ebi-a-GCST90001688 | rs116984539 | 13 | 22348885 | T | NA | 8.74 | 1.55 | 1.83E-08 |
| ebi-a-GCST90001698 | rs116990016 | 18 | 28163696 | A | 0.0021 | 196.9 | 39.12 | 5.08E-07 |
| ebi-a-GCST90001698 | rs117003718 | 10 | 124406240 | A | 0.0035 | 194.3 | 29.94 | 9.89E-11 |
| ebi-a-GCST90001726 | rs117004076 | 14 | 97702226 | G | 0.0147 | 0.4784 | 0.1013 | 2.42E-06 |
| ebi-a-GCST90001741 | rs117004076 | 14 | 97702226 | G | 0.0146 | 0.4855 | 0.1014 | 1.75E-06 |
| ebi-a-GCST90001739 | rs11701275 | 21 | 16962765 | G | 0.0634 | 0.2655 | 0.05094 | 1.96E-07 |
| ebi-a-GCST90001620 | rs117014352 | 10 | 76784567 | T | 0.0028 | -1.013 | 0.2098 | 1.44E-06 |
| ebi-a-GCST90001619 | rs117014352 | 10 | 76784567 | T | 0.0028 | -0.9683 | 0.2044 | 2.25E-06 |
| ebi-a-GCST90002048 | rs117014682 | 16 | 19693627 | T | 0.0375 | -0.4569 | 0.09575 | 2.00E-06 |
| ebi-a-GCST90001518 | rs117019089 | 16 | 69342268 | T | 5.00E-04 | 3.21 | 0.6755 | 2.17E-06 |
| ebi-a-GCST90002076 | rs11703193 | 22 | 18542023 | A | 0.1597 | -0.1793 | 0.03727 | 1.57E-06 |
| ebi-a-GCST90001695 | rs117036387 | 20 | 3521072 | G | 0.0075 | 84.27 | 15.32 | 4.04E-08 |
| ebi-a-GCST90001929 | rs117037628 | 16 | 425605 | G | 0.3072 | -0.1647 | 0.02853 | 8.69E-09 |
| ebi-a-GCST90001460 | rs117039412 | 8 | 34472241 | A | 7.00E-04 | 2.012 | 0.4152 | 1.32E-06 |
| ebi-a-GCST90002085 | rs117045668 | 11 | 21178226 | A | 0.0054 | 0.9152 | 0.1836 | 6.57E-07 |
| ebi-a-GCST90001621 | rs117047316 | 6 | 100317414 | T | 0.048 | 0.29 | 0.05936 | 1.08E-06 |
| ebi-a-GCST90001695 | rs117048727 | 13 | 44264686 | T | 4.00E-04 | 368.8 | 66.09 | 2.59E-08 |
| ebi-a-GCST90001410 | rs11705673 | 22 | 45016637 | G | 0.12 | 0.1701 | 0.0368 | 3.95E-06 |
| ebi-a-GCST90001982 | rs117062237 | 11 | 20572262 | G | 0.0106 | -0.5517 | 0.1205 | 4.88E-06 |
| ebi-a-GCST90001737 | rs117065975 | 6 | 142183647 | G | 0.0081 | 0.639 | 0.1381 | 3.83E-06 |
| ebi-a-GCST90001769 | rs117066281 | 8 | 84921322 | T | 0.0022 | -1.174 | 0.2528 | 3.53E-06 |
| ebi-a-GCST90002072 | rs117067704 | 17 | 62128083 | G | 0.0232 | 0.4562 | 0.08992 | 4.15E-07 |
| ebi-a-GCST90001964 | rs117067704 | 17 | 62128083 | G | 0.0232 | 0.6413 | 0.08914 | 7.97E-13 |
| ebi-a-GCST90002041 | rs11706901 | 3 | 10663917 | G | 0.8459 | 0.2224 | 0.04826 | 4.36E-06 |
| ebi-a-GCST90002100 | rs117074487 | 18 | 64698349 | G | 0.0296 | -0.3733 | 0.07874 | 2.23E-06 |
| ebi-a-GCST90001698 | rs117075995 | 13 | 19869255 | G | 0.0037 | 252.9 | 28.72 | 2.05E-18 |
| ebi-a-GCST90001917 | rs11707721 | 3 | 67272268 | A | 0.2554 | 0.1328 | 0.02816 | 2.49E-06 |
| ebi-a-GCST90001698 | rs117079032 | 7 | 69510130 | C | 0.006 | 163.2 | 23.63 | 6.03E-12 |
| ebi-a-GCST90001864 | rs117079896 | 12 | 101971560 | G | 0.007 | 0.7794 | 0.1633 | 1.92E-06 |
| ebi-a-GCST90002087 | rs11708131 | 3 | 104415905 | A | 0.0695 | -0.2627 | 0.05339 | 9.12E-07 |
| ebi-a-GCST90001771 | rs117084919 | 13 | 112826783 | G | 0.0219 | 0.403 | 0.08668 | 3.45E-06 |
| ebi-a-GCST90001703 | rs117086217 | 22 | 39876838 | G | 0.0343 | -0.5093 | 0.06721 | 4.42E-14 |
| ebi-a-GCST90001715 | rs117086217 | 22 | 39876838 | G | 0.0343 | -0.5069 | 0.06712 | 5.39E-14 |
| ebi-a-GCST90001702 | rs117086217 | 22 | 39876838 | G | 0.0343 | -0.5014 | 0.06723 | 1.08E-13 |
| ebi-a-GCST90001717 | rs117086217 | 22 | 39876838 | G | 0.0343 | -0.5 | 0.06715 | 1.20E-13 |
| ebi-a-GCST90001718 | rs117086217 | 22 | 39876838 | G | 0.0343 | -0.4893 | 0.06729 | 4.34E-13 |
| ebi-a-GCST90001712 | rs117086217 | 22 | 39876838 | G | 0.0343 | -0.4887 | 0.06701 | 3.73E-13 |
| ebi-a-GCST90001940 | rs117086655 | 13 | 114821897 | A | 0.0409 | 0.2982 | 0.06492 | 4.51E-06 |
| ebi-a-GCST90001698 | rs117098395 | 10 | 107595273 | A | 0.0028 | 174.7 | 34.15 | 3.28E-07 |
| ebi-a-GCST90001552 | rs117111088 | 7 | 8319815 | T | 0.0697 | 0.1693 | 0.03644 | 3.52E-06 |
| ebi-a-GCST90001523 | rs117117531 | 17 | 50899429 | A | 0.0032 | -1.57 | 0.3215 | 1.13E-06 |
| ebi-a-GCST90001551 | rs117120215 | 21 | 38377520 | G | 1.00E-04 | -2.447 | 0.507 | 1.45E-06 |
| ebi-a-GCST90001553 | rs117120215 | 21 | 38377520 | G | 1.00E-04 | -2.438 | 0.4999 | 1.12E-06 |
| ebi-a-GCST90001632 | rs117122100 | 13 | 109403845 | T | 0.0142 | -0.4752 | 0.1027 | 3.84E-06 |
| ebi-a-GCST90001631 | rs117122100 | 13 | 109403845 | T | 0.0142 | -0.4711 | 0.1021 | 4.08E-06 |
| ebi-a-GCST90001698 | rs117123362 | 8 | 129915383 | C | 0.0021 | 229.3 | 39.06 | 4.76E-09 |
| ebi-a-GCST90001698 | rs117127715 | 8 | 31315428 | A | 0.0032 | 180.4 | 30.92 | 5.91E-09 |
| ebi-a-GCST90001697 | rs117134167 | 16 | 25778935 | T | 6.00E-04 | 13.59 | 2.627 | 2.42E-07 |
| ebi-a-GCST90001488 | rs117140551 | 19 | 23103264 | T | 0.0285 | 0.3571 | 0.07653 | 3.19E-06 |
| ebi-a-GCST90001487 | rs117140551 | 19 | 23103264 | T | 0.0285 | 0.3619 | 0.0751 | 1.51E-06 |
| ebi-a-GCST90001954 | rs117140964 | 10 | 101688758 | T | 0.0444 | 0.4057 | 0.08837 | 4.77E-06 |
| ebi-a-GCST90001955 | rs117140964 | 10 | 101688758 | T | 0.0444 | 0.418 | 0.08848 | 2.52E-06 |
| ebi-a-GCST90001953 | rs117140964 | 10 | 101688758 | T | 0.0443 | 0.4276 | 0.08828 | 1.40E-06 |
| ebi-a-GCST90001661 | rs117147408 | 6 | 101980039 | G | 0.0273 | 0.4819 | 0.07946 | 1.47E-09 |
| ebi-a-GCST90001623 | rs117147408 | 6 | 101980039 | G | 0.0275 | 0.5685 | 0.07776 | 3.23E-13 |
| ebi-a-GCST90001622 | rs117147408 | 6 | 101980039 | G | 0.0275 | 0.6063 | 0.07722 | 5.37E-15 |
| ebi-a-GCST90001883 | rs117147408 | 6 | 101980039 | G | 0.0276 | 0.6523 | 0.08226 | 3.03E-15 |
| ebi-a-GCST90001633 | rs117147408 | 6 | 101980039 | G | 0.0277 | 0.6679 | 0.0755 | 1.38E-18 |
| ebi-a-GCST90001638 | rs117147408 | 6 | 101980039 | G | 0.0275 | 0.675 | 0.07557 | 6.50E-19 |
| ebi-a-GCST90001637 | rs117147408 | 6 | 101980039 | G | 0.0275 | 0.7037 | 0.07581 | 2.75E-20 |
| ebi-a-GCST90001635 | rs117147408 | 6 | 101980039 | G | 0.0275 | 0.7078 | 0.07572 | 1.51E-20 |
| ebi-a-GCST90001634 | rs117147408 | 6 | 101980039 | G | 0.0275 | 0.7354 | 0.07599 | 6.79E-22 |
| ebi-a-GCST90001459 | rs11715127 | 3 | 384169 | C | 0.1276 | -0.1682 | 0.03678 | 5.00E-06 |
| ebi-a-GCST90001474 | rs11715127 | 3 | 384169 | C | 0.1276 | 0.1657 | 0.03607 | 4.48E-06 |
| ebi-a-GCST90001842 | rs11715223 | 3 | 64581733 | A | 0.2151 | 0.1539 | 0.03327 | 3.90E-06 |
| ebi-a-GCST90001845 | rs11715223 | 3 | 64581733 | A | 0.215 | 0.1595 | 0.03339 | 1.86E-06 |
| ebi-a-GCST90002065 | rs117153869 | 8 | 32077693 | T | 0.0134 | -0.6 | 0.121 | 7.55E-07 |
| ebi-a-GCST90001784 | rs117156856 | 16 | 46798678 | A | 0.0023 | 1.252 | 0.268 | 3.10E-06 |
| ebi-a-GCST90001695 | rs11716645 | 3 | 29402908 | G | 0.0025 | 159.7 | 26.69 | 2.42E-09 |
| ebi-a-GCST90001594 | rs117166897 | 10 | 10964820 | G | 0.0561 | -0.2471 | 0.05363 | 4.23E-06 |
| ebi-a-GCST90001841 | rs117171372 | 15 | 27777649 | C | 0.0014 | 1.721 | 0.3583 | 1.63E-06 |
| ebi-a-GCST90002117 | rs117172940 | 7 | 26706043 | T | 3.00E-04 | 3.291 | 0.7003 | 2.73E-06 |
| ebi-a-GCST90001700 | rs117173207 | 11 | 24544063 | A | 4.00E-04 | 18.77 | 3.342 | 2.10E-08 |
| ebi-a-GCST90001664 | rs117174937 | 8 | 17606838 | G | 3.00E-04 | 3.04 | 0.6254 | 1.22E-06 |
| ebi-a-GCST90001698 | rs117177255 | 13 | 77081029 | T | 0.0028 | 255.1 | 33.32 | 2.52E-14 |
| ebi-a-GCST90001660 | rs11718018 | 3 | 60374028 | C | 0.4134 | -0.1208 | 0.02589 | 3.18E-06 |
| ebi-a-GCST90001484 | rs11718018 | 3 | 60374028 | C | 0.4149 | -0.1175 | 0.02509 | 2.90E-06 |
| ebi-a-GCST90001698 | rs117185099 | 6 | 119514715 | T | 0.0028 | 208.3 | 32.03 | 8.96E-11 |
| ebi-a-GCST90002066 | rs117190770 | 10 | 86457358 | A | 0.0435 | -0.3581 | 0.06734 | 1.13E-07 |
| ebi-a-GCST90002061 | rs117190770 | 10 | 86457358 | A | 0.0435 | -0.3522 | 0.06822 | 2.59E-07 |
| ebi-a-GCST90002070 | rs117190770 | 10 | 86457358 | A | 0.0435 | -0.3413 | 0.06769 | 4.90E-07 |
| ebi-a-GCST90002063 | rs117190770 | 10 | 86457358 | A | 0.0435 | -0.3334 | 0.06738 | 7.90E-07 |
| ebi-a-GCST90002090 | rs11719519 | 3 | 186104558 | T | 0.7893 | 0.167 | 0.03417 | 1.08E-06 |
| ebi-a-GCST90002033 | rs11719519 | 3 | 186104558 | T | 0.789 | 0.1727 | 0.03372 | 3.22E-07 |
| ebi-a-GCST90001695 | rs117196114 | 9 | 83853182 | A | 0.0018 | 201.5 | 32.14 | 4.11E-10 |
| ebi-a-GCST90002056 | rs117197627 | 10 | 18473835 | T | 9.00E-04 | 2.156 | 0.4378 | 8.86E-07 |
| ebi-a-GCST90001693 | rs117198249 | 10 | 28939784 | T | 0.0158 | 0.4924 | 0.1042 | 2.40E-06 |
| ebi-a-GCST90001497 | rs117200502 | 10 | 97070990 | T | 0.1114 | 0.4056 | 0.04033 | 1.80E-23 |
| ebi-a-GCST90001496 | rs117200502 | 10 | 97070990 | T | 0.1114 | 0.4259 | 0.0408 | 3.91E-25 |
| ebi-a-GCST90001796 | rs117208568 | 15 | 54126596 | A | 0.0168 | 0.4344 | 0.09339 | 3.41E-06 |
| ebi-a-GCST90001698 | rs117211386 | 15 | 51649599 | A | 0.001 | 447.6 | 54.04 | 1.71E-16 |
| ebi-a-GCST90001767 | rs117218666 | 6 | 139700367 | T | 0.0366 | 0.4841 | 0.09535 | 4.23E-07 |
| ebi-a-GCST90001698 | rs117219726 | 8 | 33264182 | A | 9.00E-04 | 328.6 | 60.54 | 6.09E-08 |
| ebi-a-GCST90001936 | rs117223635 | 9 | 90443364 | T | 0.0345 | -0.3275 | 0.07027 | 3.26E-06 |
| ebi-a-GCST90002052 | rs117224922 | 12 | 5441979 | C | 0.0032 | -1.632 | 0.3342 | 1.14E-06 |
| ebi-a-GCST90001927 | rs117239506 | 12 | 16351043 | A | 0.001 | 1.832 | 0.3932 | 3.32E-06 |
| ebi-a-GCST90001695 | rs117247210 | 12 | 10416728 | T | NA | 2555 | 497.5 | 2.96E-07 |
| ebi-a-GCST90001576 | rs117249531 | 10 | 81026095 | T | 0.0037 | -0.939 | 0.2005 | 2.94E-06 |
| ebi-a-GCST90001600 | rs11725186 | 4 | 94012466 | A | 0.0229 | 0.3931 | 0.08048 | 1.08E-06 |
| ebi-a-GCST90001696 | rs117252706 | 19 | 3310194 | G | 0.0197 | 4.978 | 1.076 | 3.87E-06 |
| ebi-a-GCST90001697 | rs117262195 | 16 | 5991815 | T | 0.0013 | 8.949 | 1.874 | 1.87E-06 |
| ebi-a-GCST90001695 | rs117262195 | 16 | 5991815 | T | 0.0013 | 270.5 | 39.98 | 1.55E-11 |
| ebi-a-GCST90001698 | rs117270364 | 11 | 60694375 | C | 0.0032 | 186 | 31.2 | 2.73E-09 |
| ebi-a-GCST90001695 | rs117271894 | 8 | 112881674 | A | 0.0022 | 192.8 | 34.06 | 1.63E-08 |
| ebi-a-GCST90001695 | rs117277690 | 11 | 33322159 | G | 4.00E-04 | 356.8 | 65.76 | 6.19E-08 |
| ebi-a-GCST90001686 | rs117279064 | 16 | 14352543 | A | 0.0126 | -0.4812 | 0.1043 | 4.07E-06 |
| ebi-a-GCST90001700 | rs117284243 | 11 | 122918979 | T | 1.00E-04 | 49.65 | 5.795 | 1.57E-17 |
| ebi-a-GCST90001699 | rs117284243 | 11 | 122918979 | T | 1.00E-04 | 64.01 | 13.7 | 3.08E-06 |
| ebi-a-GCST90001698 | rs117284243 | 11 | 122918979 | T | 1.00E-04 | 1783 | 131.7 | 9.60E-41 |
| ebi-a-GCST90001517 | rs117285079 | 18 | 4762780 | G | 0.0401 | -0.3814 | 0.08257 | 4.13E-06 |
| ebi-a-GCST90001840 | rs117286362 | 19 | 31209621 | T | 0.1048 | -0.2026 | 0.04427 | 4.89E-06 |
| ebi-a-GCST90001973 | rs117287835 | 11 | 96273624 | A | 5.00E-04 | -2.444 | 0.5226 | 3.05E-06 |
| ebi-a-GCST90001698 | rs117291729 | 16 | 11975137 | T | 0.004 | 132.9 | 28.79 | 4.05E-06 |
| ebi-a-GCST90001963 | rs117292692 | 10 | 72720949 | A | 0.0324 | 0.3804 | 0.07869 | 1.40E-06 |
| ebi-a-GCST90001928 | rs117293308 | 11 | 43599281 | A | 0.025 | 0.3943 | 0.08577 | 4.47E-06 |
| ebi-a-GCST90001698 | rs117293367 | 12 | 52437438 | T | 6.00E-04 | 437.7 | 69.06 | 2.64E-10 |
| ebi-a-GCST90001698 | rs117294527 | 15 | 100764857 | G | 0.0031 | 155.4 | 31.61 | 9.28E-07 |
| ebi-a-GCST90001985 | rs117295081 | 20 | 45333273 | G | 0.0175 | 0.52 | 0.09717 | 9.29E-08 |
| ebi-a-GCST90001980 | rs117295081 | 20 | 45333273 | G | 0.0175 | 0.5503 | 0.09653 | 1.29E-08 |
| ebi-a-GCST90001700 | rs117296998 | 7 | 33902745 | T | 0.001 | 10.5 | 2.211 | 2.10E-06 |
| ebi-a-GCST90001550 | rs117299036 | 7 | 17645647 | T | 0.0099 | 0.5711 | 0.124 | 4.28E-06 |
| ebi-a-GCST90001549 | rs117299036 | 7 | 17645647 | T | 0.0099 | 0.5813 | 0.1262 | 4.27E-06 |
| ebi-a-GCST90001585 | rs117302238 | 15 | 75293128 | A | 0.0339 | 0.3235 | 0.06785 | 1.93E-06 |
| ebi-a-GCST90001823 | rs117303087 | 14 | 103824600 | G | 0.0714 | 0.303 | 0.06493 | 3.29E-06 |
| ebi-a-GCST90001422 | rs117308259 | 14 | 31993501 | C | 0.0066 | -0.8348 | 0.1494 | 2.48E-08 |
| ebi-a-GCST90001444 | rs117308259 | 14 | 31993501 | C | 0.0066 | -0.7613 | 0.1499 | 4.02E-07 |
| ebi-a-GCST90001423 | rs117308259 | 14 | 31993501 | C | 0.0066 | -0.6917 | 0.1504 | 4.40E-06 |
| ebi-a-GCST90001451 | rs117309559 | 14 | 31030304 | G | 0.013 | 0.5719 | 0.1203 | 2.07E-06 |
| ebi-a-GCST90001828 | rs117309996 | 17 | 59009664 | C | 0.0246 | 0.3995 | 0.08554 | 3.11E-06 |
| ebi-a-GCST90002105 | rs11731221 | 4 | 84168891 | G | 0.3491 | -0.141 | 0.02861 | 8.79E-07 |
| ebi-a-GCST90001836 | rs117312711 | 7 | 112322389 | T | 0.0321 | -0.5516 | 0.1054 | 1.90E-07 |
| ebi-a-GCST90001782 | rs117315714 | 7 | 46669974 | T | 0.0011 | 2.241 | 0.4718 | 2.20E-06 |
| ebi-a-GCST90001726 | rs11733521 | 4 | 68968349 | T | 0.0645 | -0.2298 | 0.05013 | 4.70E-06 |
| ebi-a-GCST90001601 | rs117336432 | 20 | 62523193 | T | 0.0011 | -1.537 | 0.3352 | 4.71E-06 |
| ebi-a-GCST90001649 | rs117338457 | 11 | 69354886 | T | 0.0449 | 0.2936 | 0.06216 | 2.40E-06 |
| ebi-a-GCST90001698 | rs117339150 | 8 | 52563134 | A | 0.0025 | 167.3 | 35.13 | 1.99E-06 |
| ebi-a-GCST90001608 | rs117340321 | 15 | 42829973 | T | 0.0578 | -0.2528 | 0.05364 | 2.54E-06 |
| ebi-a-GCST90001992 | rs117340589 | 8 | 138634227 | G | 0.0012 | 1.695 | 0.3424 | 7.71E-07 |
| ebi-a-GCST90001697 | rs117354956 | 8 | 61304968 | T | 6.00E-04 | 12.6 | 2.429 | 2.25E-07 |
| ebi-a-GCST90001551 | rs11735841 | 4 | 25612506 | A | 0.0074 | 0.435 | 0.09374 | 3.61E-06 |
| ebi-a-GCST90001688 | rs11735841 | 4 | 25612506 | A | 0.0073 | 0.4503 | 0.09716 | 3.72E-06 |
| ebi-a-GCST90001690 | rs11735841 | 4 | 25612506 | A | 0.0073 | 0.4812 | 0.09747 | 8.31E-07 |
| ebi-a-GCST90001994 | rs117379843 | 19 | 56718736 | G | 0.027 | 0.3719 | 0.07579 | 9.70E-07 |
| ebi-a-GCST90002003 | rs117383806 | 8 | 140934719 | T | 0.0138 | 0.483 | 0.1039 | 3.49E-06 |
| ebi-a-GCST90002089 | rs117387675 | 9 | 105955248 | G | 0.066 | -0.2805 | 0.05564 | 4.94E-07 |
| ebi-a-GCST90001698 | rs117389556 | 8 | 61340602 | G | 0.004 | 142.7 | 29.75 | 1.68E-06 |
| ebi-a-GCST90001592 | rs117391590 | 17 | 42759948 | A | 0.0413 | -0.2742 | 0.05955 | 4.29E-06 |
| ebi-a-GCST90001493 | rs11739607 | 5 | 27694229 | C | 0.0214 | -0.3801 | 0.08266 | 4.42E-06 |
| ebi-a-GCST90001707 | rs117397694 | 12 | 5565515 | A | 0.0188 | -0.6104 | 0.1256 | 1.29E-06 |
| ebi-a-GCST90001698 | rs117397768 | 15 | 68230533 | A | 0.0194 | 59.95 | 12.74 | 2.62E-06 |
| ebi-a-GCST90001688 | rs117403703 | 6 | 93203850 | C | 1.00E-04 | 3.353 | 0.6161 | 5.62E-08 |
| ebi-a-GCST90001862 | rs117404466 | 7 | 148293966 | A | 0.0567 | 0.2745 | 0.05883 | 3.20E-06 |
| ebi-a-GCST90002113 | rs117406069 | 10 | 130262493 | A | 0.0281 | 0.3669 | 0.07838 | 2.97E-06 |
| ebi-a-GCST90001695 | rs117413806 | 11 | 16290338 | C | 4.00E-04 | 364.8 | 66.04 | 3.57E-08 |
| ebi-a-GCST90001533 | rs117417480 | 19 | 54429001 | T | 0.0129 | 0.8056 | 0.1475 | 5.32E-08 |
| ebi-a-GCST90001618 | rs117419608 | 8 | 11831788 | A | 0.0053 | -0.8376 | 0.1656 | 4.46E-07 |
| ebi-a-GCST90001698 | rs117420444 | 11 | 31631366 | A | 0.001 | 344.4 | 54.89 | 3.96E-10 |
| ebi-a-GCST90002066 | rs117421233 | 10 | 85630450 | A | 0.0185 | -0.4885 | 0.1022 | 1.83E-06 |
| ebi-a-GCST90002063 | rs117421233 | 10 | 85630450 | A | 0.0185 | -0.4832 | 0.1022 | 2.37E-06 |
| ebi-a-GCST90002070 | rs117421233 | 10 | 85630450 | A | 0.0185 | -0.4791 | 0.1027 | 3.21E-06 |
| ebi-a-GCST90001992 | rs117427319 | 7 | 69208295 | T | 6.00E-04 | 2.862 | 0.6083 | 2.63E-06 |
| ebi-a-GCST90001750 | rs11742841 | 5 | 108992703 | G | 0.0643 | 0.3177 | 0.06851 | 3.79E-06 |
| ebi-a-GCST90001697 | rs117428784 | 19 | 43422957 | A | 4.00E-04 | 24.71 | 3.261 | 4.54E-14 |
| ebi-a-GCST90001696 | rs117428784 | 19 | 43422957 | A | 4.00E-04 | 36.89 | 8.022 | 4.41E-06 |
| ebi-a-GCST90001695 | rs117428784 | 19 | 43422957 | A | 4.00E-04 | 472.2 | 70.2 | 2.03E-11 |
| ebi-a-GCST90001720 | rs117430146 | 22 | 41998660 | C | 0.0561 | 0.2856 | 0.05592 | 3.45E-07 |
| ebi-a-GCST90001716 | rs117430146 | 22 | 41998660 | C | 0.0561 | 0.3056 | 0.05584 | 4.73E-08 |
| ebi-a-GCST90001829 | rs117430146 | 22 | 41998660 | C | 0.056 | 0.3083 | 0.05583 | 3.61E-08 |
| ebi-a-GCST90001719 | rs117430146 | 22 | 41998660 | C | 0.056 | 0.3144 | 0.05574 | 1.83E-08 |
| ebi-a-GCST90001705 | rs117430146 | 22 | 41998660 | C | 0.0562 | 0.3149 | 0.05565 | 1.65E-08 |
| ebi-a-GCST90001704 | rs117430146 | 22 | 41998660 | C | 0.0561 | 0.3164 | 0.0557 | 1.45E-08 |
| ebi-a-GCST90001709 | rs117430146 | 22 | 41998660 | C | 0.0561 | 0.3293 | 0.05572 | 3.72E-09 |
| ebi-a-GCST90001708 | rs117430146 | 22 | 41998660 | C | 0.0561 | 0.3402 | 0.0558 | 1.20E-09 |
| ebi-a-GCST90001695 | rs117431224 | 11 | 44356396 | G | 4.00E-04 | 385.7 | 68.87 | 2.31E-08 |
| ebi-a-GCST90001698 | rs117433099 | 7 | 6934892 | A | 0.0019 | 264.9 | 39.21 | 1.67E-11 |
| ebi-a-GCST90001695 | rs117437702 | 10 | 110752679 | G | 0.0185 | 46.12 | 9.799 | 2.61E-06 |
| ebi-a-GCST90001698 | rs117440863 | 8 | 98513319 | C | 9.00E-04 | 375.1 | 62.7 | 2.43E-09 |
| ebi-a-GCST90001729 | rs117442206 | 12 | 25430787 | T | 0.0481 | -0.2889 | 0.05856 | 8.41E-07 |
| ebi-a-GCST90001938 | rs117447663 | 10 | 68626431 | T | 0.008 | -0.7139 | 0.1399 | 3.55E-07 |
| ebi-a-GCST90001698 | rs117448261 | 8 | 82400020 | C | 0.0022 | 196.1 | 35.66 | 4.08E-08 |
| ebi-a-GCST90002069 | rs117458836 | 10 | 96773382 | A | 0.0187 | 0.5108 | 0.1043 | 1.02E-06 |
| ebi-a-GCST90001888 | rs117458836 | 10 | 96773382 | A | 0.0187 | 0.5498 | 0.1024 | 8.59E-08 |
| ebi-a-GCST90001892 | rs117458836 | 10 | 96773382 | A | 0.0187 | 0.6927 | 0.1041 | 3.45E-11 |
| ebi-a-GCST90002061 | rs117458836 | 10 | 96773382 | A | 0.0187 | 0.8979 | 0.1053 | 2.34E-17 |
| ebi-a-GCST90002019 | rs1174651 | 12 | 58632584 | C | 0.5185 | 0.1846 | 0.03634 | 4.25E-07 |
| ebi-a-GCST90001841 | rs117466579 | 7 | 85315630 | C | 0.0065 | -0.755 | 0.1645 | 4.61E-06 |
| ebi-a-GCST90001930 | rs117467123 | 19 | 16407691 | A | 0.0878 | 0.2296 | 0.04886 | 2.74E-06 |
| ebi-a-GCST90001506 | rs117469911 | 7 | 79791344 | A | 0.0036 | -0.9208 | 0.1957 | 2.63E-06 |
| ebi-a-GCST90001700 | rs117469911 | 7 | 79791344 | A | 0.0036 | 6.228 | 1.155 | 7.39E-08 |
| ebi-a-GCST90001695 | rs117469911 | 7 | 79791344 | A | 0.0035 | 132.8 | 20.58 | 1.24E-10 |
| ebi-a-GCST90001698 | rs117469911 | 7 | 79791344 | A | 0.0035 | 272.5 | 27.65 | 1.31E-22 |
| ebi-a-GCST90001573 | rs117470554 | 9 | 16436647 | C | 0.1062 | -0.1915 | 0.04118 | 3.42E-06 |
| ebi-a-GCST90001795 | rs117473115 | 10 | 54503355 | T | 0.0331 | 0.3516 | 0.06985 | 5.03E-07 |
| ebi-a-GCST90001690 | rs117475733 | 6 | 148775706 | T | 4.00E-04 | 1.373 | 0.3001 | 4.92E-06 |
| ebi-a-GCST90001689 | rs117475733 | 6 | 148775706 | T | 4.00E-04 | 1.491 | 0.3243 | 4.44E-06 |
| ebi-a-GCST90001688 | rs117475733 | 6 | 148775706 | T | 4.00E-04 | 1.818 | 0.2979 | 1.17E-09 |
| ebi-a-GCST90001695 | rs117475878 | 14 | 32709998 | T | 0.0022 | 159.2 | 29.91 | 1.09E-07 |
| ebi-a-GCST90001700 | rs117478414 | 8 | 56081028 | T | 1.00E-04 | 32.87 | 5.796 | 1.53E-08 |
| ebi-a-GCST90002082 | rs117482121 | 18 | 75269360 | A | 0.041 | -0.2912 | 0.0588 | 7.68E-07 |
| ebi-a-GCST90001920 | rs117486308 | 6 | 147964200 | A | 0.0018 | -1.552 | 0.3167 | 1.01E-06 |
| ebi-a-GCST90001937 | rs117486405 | 12 | 32082360 | T | 0.043 | -0.3098 | 0.06384 | 1.27E-06 |
| ebi-a-GCST90001461 | rs11748731 | 5 | 30878060 | T | 0.0362 | 0.3124 | 0.06744 | 3.75E-06 |
| ebi-a-GCST90001721 | rs11748905 | 5 | 80934634 | C | 0.0048 | -1.009 | 0.1845 | 4.78E-08 |
| ebi-a-GCST90001739 | rs11748905 | 5 | 80934634 | C | 0.0048 | -0.8732 | 0.186 | 2.77E-06 |
| ebi-a-GCST90001739 | rs117491079 | 16 | 85272300 | T | 0.0391 | 0.343 | 0.06329 | 6.36E-08 |
| ebi-a-GCST90001911 | rs117491214 | 7 | 26905913 | T | 0.0022 | 1.189 | 0.2565 | 3.73E-06 |
| ebi-a-GCST90001688 | rs117494082 | 14 | 56862154 | A | 1.00E-04 | 3.894 | 0.6914 | 1.94E-08 |
| ebi-a-GCST90001995 | rs117498337 | 11 | 123076792 | G | 0.1411 | -0.1643 | 0.0359 | 4.86E-06 |
| ebi-a-GCST90001708 | rs117499379 | 18 | 4343023 | C | 0.0682 | 0.2411 | 0.05023 | 1.65E-06 |
| ebi-a-GCST90001720 | rs117499379 | 18 | 4343023 | C | 0.0683 | 0.2459 | 0.05026 | 1.05E-06 |
| ebi-a-GCST90001695 | rs117500319 | 16 | 3108127 | A | 7.00E-04 | 230.6 | 48.53 | 2.09E-06 |
| ebi-a-GCST90001894 | rs117505260 | 13 | 113724248 | T | 0.0563 | 0.2676 | 0.05846 | 4.88E-06 |
| ebi-a-GCST90001695 | rs117505371 | 7 | 125564456 | G | NA | 2050 | 399 | 2.93E-07 |
| ebi-a-GCST90001753 | rs11751198 | 6 | 31753526 | A | 0.1839 | 0.1933 | 0.03626 | 1.03E-07 |
| ebi-a-GCST90001755 | rs11751198 | 6 | 31753526 | A | 0.1839 | 0.1995 | 0.03634 | 4.32E-08 |
| ebi-a-GCST90001761 | rs11751198 | 6 | 31753526 | A | 0.1839 | 0.2182 | 0.03712 | 4.52E-09 |
| ebi-a-GCST90001666 | rs117513254 | 12 | 126913667 | G | 0.0018 | 1.37 | 0.2927 | 2.96E-06 |
| ebi-a-GCST90001765 | rs117513979 | 7 | 103968091 | C | 4.00E-04 | -2.508 | 0.5353 | 2.91E-06 |
| ebi-a-GCST90001695 | rs117514047 | 21 | 26316207 | C | 3.00E-04 | 462.3 | 82.97 | 2.71E-08 |
| ebi-a-GCST90001577 | rs117518951 | 16 | 55449193 | C | 0.049 | 0.2696 | 0.05477 | 8.94E-07 |
| ebi-a-GCST90002121 | rs117519014 | 10 | 97403248 | A | 0.0427 | 0.4352 | 0.06661 | 7.57E-11 |
| ebi-a-GCST90001509 | rs117520868 | 19 | 16580644 | A | 0.0343 | 0.332 | 0.0722 | 4.41E-06 |
| ebi-a-GCST90001863 | rs117524375 | 16 | 1790546 | T | 0.018 | 0.4983 | 0.1021 | 1.12E-06 |
| ebi-a-GCST90001914 | rs117527144 | 12 | 3303278 | C | 0.061 | 0.2533 | 0.0541 | 2.97E-06 |
| ebi-a-GCST90001884 | rs117533070 | 11 | 112710664 | T | 0.0816 | 0.2856 | 0.04932 | 7.71E-09 |
| ebi-a-GCST90001958 | rs117535998 | 16 | 71579132 | T | 0.1208 | 0.198 | 0.04213 | 2.72E-06 |
| ebi-a-GCST90001561 | rs117545347 | 15 | 98090362 | T | 0.0025 | 0.9647 | 0.2101 | 4.58E-06 |
| ebi-a-GCST90001456 | rs117545845 | 22 | 25622885 | T | 0.1211 | 0.189 | 0.04018 | 2.68E-06 |
| ebi-a-GCST90001454 | rs117545845 | 22 | 25622885 | T | 0.1209 | 0.1995 | 0.03962 | 5.05E-07 |
| ebi-a-GCST90001999 | rs117552079 | 11 | 73714261 | A | 0.0539 | 0.2553 | 0.05514 | 3.78E-06 |
| ebi-a-GCST90001734 | rs117553718 | 10 | 5865812 | A | 0.0138 | 0.5498 | 0.1088 | 4.57E-07 |
| ebi-a-GCST90001902 | rs11755527 | 6 | 90958231 | G | 0.5224 | 0.1368 | 0.02632 | 2.16E-07 |
| ebi-a-GCST90001889 | rs11755527 | 6 | 90958231 | G | 0.5224 | 0.1488 | 0.02672 | 2.80E-08 |
| ebi-a-GCST90001899 | rs11755527 | 6 | 90958231 | G | 0.5224 | 0.1596 | 0.02601 | 9.68E-10 |
| ebi-a-GCST90001698 | rs117555426 | 16 | 13128176 | C | 0.001 | 414.6 | 53.64 | 1.41E-14 |
| ebi-a-GCST90001742 | rs117562840 | 22 | 42680877 | A | 0.0055 | -0.7994 | 0.166 | 1.53E-06 |
| ebi-a-GCST90001698 | rs117562840 | 22 | 42680877 | A | 0.005 | 122.4 | 24.8 | 8.35E-07 |
| ebi-a-GCST90001542 | rs117563350 | 6 | 151699396 | G | 0.0308 | -0.3637 | 0.07298 | 6.56E-07 |
| ebi-a-GCST90001534 | rs117563350 | 6 | 151699396 | G | 0.0308 | -0.3399 | 0.07398 | 4.49E-06 |
| ebi-a-GCST90001590 | rs117563350 | 6 | 151699396 | G | 0.0312 | -0.324 | 0.06938 | 3.12E-06 |
| ebi-a-GCST90001485 | rs117572834 | 10 | 109896541 | G | 0.0031 | -1.108 | 0.2135 | 2.25E-07 |
| ebi-a-GCST90001483 | rs117572834 | 10 | 109896541 | G | 0.0031 | -1.021 | 0.2137 | 1.83E-06 |
| ebi-a-GCST90001698 | rs117573224 | 11 | 1149087 | A | 0.0029 | 188 | 33.74 | 2.71E-08 |
| ebi-a-GCST90001912 | rs117580642 | 18 | 9097811 | G | 0.0697 | -0.2462 | 0.05326 | 3.95E-06 |
| ebi-a-GCST90001669 | rs117586381 | 19 | 18683695 | A | 7.00E-04 | -2.041 | 0.4452 | 4.71E-06 |
| ebi-a-GCST90001697 | rs117586879 | 20 | 17201328 | G | 1.00E-04 | 32.49 | 6.401 | 4.07E-07 |
| ebi-a-GCST90001878 | rs11758857 | 6 | 117867335 | C | 0.0389 | -0.4723 | 0.1012 | 3.38E-06 |
| ebi-a-GCST90002090 | rs117596617 | 20 | 52794798 | A | 0.1311 | -0.2022 | 0.04167 | 1.29E-06 |
| ebi-a-GCST90001638 | rs11759822 | 6 | 99608776 | T | 0.2803 | 0.1268 | 0.02693 | 2.58E-06 |
| ebi-a-GCST90001681 | rs117603761 | 16 | 77741894 | C | 0.0211 | 0.4369 | 0.08969 | 1.17E-06 |
| ebi-a-GCST90001723 | rs117606004 | 20 | 44812456 | T | 0.0071 | 0.6689 | 0.1441 | 3.59E-06 |
| ebi-a-GCST90001980 | rs117608122 | 7 | 152197572 | C | 0.0171 | -0.4893 | 0.09742 | 5.36E-07 |
| ebi-a-GCST90001985 | rs117608122 | 7 | 152197572 | C | 0.0171 | -0.4601 | 0.09808 | 2.82E-06 |
| ebi-a-GCST90001698 | rs117609491 | 8 | 106840842 | A | 0.0065 | 118.7 | 22.05 | 7.71E-08 |
| ebi-a-GCST90001698 | rs117613430 | 12 | 93540934 | A | 0.0015 | 250.3 | 45.06 | 3.01E-08 |
| ebi-a-GCST90001510 | rs117614870 | 17 | 63857170 | T | 0.0085 | 0.7075 | 0.144 | 9.40E-07 |
| ebi-a-GCST90001877 | rs117618257 | 16 | 425605 | A | 0.3095 | -0.2272 | 0.04419 | 3.16E-07 |
| ebi-a-GCST90001950 | rs11762203 | 7 | 120912956 | T | 0.0233 | 0.6201 | 0.1341 | 4.13E-06 |
| ebi-a-GCST90001668 | rs117626397 | 10 | 131423918 | G | 4.00E-04 | -2.612 | 0.5689 | 4.58E-06 |
| ebi-a-GCST90001664 | rs117626397 | 10 | 131423918 | G | 4.00E-04 | 2.741 | 0.5367 | 3.46E-07 |
| ebi-a-GCST90001509 | rs117626397 | 10 | 131423918 | G | 4.00E-04 | 2.958 | 0.5861 | 4.72E-07 |
| ebi-a-GCST90001508 | rs117626397 | 10 | 131423918 | G | 4.00E-04 | 3.007 | 0.5864 | 3.10E-07 |
| ebi-a-GCST90001698 | rs117626443 | 8 | 69864162 | G | 0.0023 | 196 | 35.52 | 3.68E-08 |
| ebi-a-GCST90001813 | rs11763462 | 7 | 130342978 | G | 0.2196 | 0.1404 | 0.03057 | 4.50E-06 |
| ebi-a-GCST90001818 | rs11763462 | 7 | 130342978 | G | 0.2194 | 0.1429 | 0.03043 | 2.73E-06 |
| ebi-a-GCST90002116 | rs117640098 | 9 | 91723702 | G | 0.009 | 0.6725 | 0.1423 | 2.39E-06 |
| ebi-a-GCST90001565 | rs117646786 | 22 | 33601370 | C | 9.00E-04 | 1.761 | 0.3588 | 9.72E-07 |
| ebi-a-GCST90001670 | rs117648214 | 10 | 98816434 | T | 0.0029 | 1.27 | 0.2262 | 2.14E-08 |
| ebi-a-GCST90001901 | rs117653391 | 10 | 97551952 | A | 0.0449 | 0.4455 | 0.06505 | 9.29E-12 |
| ebi-a-GCST90001884 | rs117655461 | 11 | 114307356 | T | 0.0434 | -0.3041 | 0.06522 | 3.24E-06 |
| ebi-a-GCST90001955 | rs11765781 | 7 | 4764861 | A | 0.6245 | 0.1816 | 0.03963 | 4.97E-06 |
| ebi-a-GCST90002060 | rs117660790 | 14 | 28384801 | C | 0.0894 | -0.2164 | 0.04586 | 2.47E-06 |
| ebi-a-GCST90001449 | rs11766273 | 7 | 22775663 | A | 0.0115 | 0.591 | 0.1283 | 4.23E-06 |
| ebi-a-GCST90002033 | rs11766311 | 7 | 77134132 | A | 0.411 | -0.1526 | 0.02783 | 4.55E-08 |
| ebi-a-GCST90001483 | rs117678654 | 10 | 101548054 | A | 0.0107 | 0.6017 | 0.1206 | 6.32E-07 |
| ebi-a-GCST90001482 | rs117680001 | 17 | 13652401 | A | 0.0105 | 0.5355 | 0.117 | 4.91E-06 |
| ebi-a-GCST90001698 | rs117685161 | 8 | 99344753 | G | 0.0037 | 153.6 | 30.11 | 3.60E-07 |
| ebi-a-GCST90001996 | rs11768517 | 7 | 54919768 | T | 0.3061 | 0.1326 | 0.02691 | 8.75E-07 |
| ebi-a-GCST90001939 | rs117690070 | 18 | 11450416 | T | 0.015 | 0.516 | 0.1036 | 6.57E-07 |
| ebi-a-GCST90001465 | rs117690664 | 8 | 107693714 | A | 7.00E-04 | -2.515 | 0.5157 | 1.12E-06 |
| ebi-a-GCST90001473 | rs117690664 | 8 | 107693714 | A | 7.00E-04 | -2.506 | 0.5056 | 7.49E-07 |
| ebi-a-GCST90001472 | rs117690664 | 8 | 107693714 | A | 7.00E-04 | -2.339 | 0.5107 | 4.81E-06 |
| ebi-a-GCST90002077 | rs117691019 | 11 | 78351106 | G | 0.002 | -1.406 | 0.299 | 2.68E-06 |
| ebi-a-GCST90001806 | rs11769145 | 7 | 70704068 | C | 0.1175 | 0.1836 | 0.03911 | 2.77E-06 |
| ebi-a-GCST90001800 | rs11769145 | 7 | 70704068 | C | 0.1175 | 0.1915 | 0.03929 | 1.14E-06 |
| ebi-a-GCST90001700 | rs117692894 | 7 | 39587008 | T | 1.00E-04 | 33.95 | 5.804 | 5.41E-09 |
| ebi-a-GCST90001642 | rs117693242 | 6 | 104445996 | G | 0.0159 | -0.4542 | 0.09774 | 3.48E-06 |
| ebi-a-GCST90001698 | rs117702997 | 9 | 83435830 | G | 0.0021 | 215.2 | 39.24 | 4.46E-08 |
| ebi-a-GCST90001544 | rs11770479 | 7 | 70906148 | C | 0.007 | -0.6696 | 0.14 | 1.81E-06 |
| ebi-a-GCST90001704 | rs117705111 | 22 | 41957510 | A | 0.0365 | -0.763 | 0.065 | 2.97E-31 |
| ebi-a-GCST90001716 | rs117705111 | 22 | 41957510 | A | 0.0365 | -0.7531 | 0.06516 | 2.27E-30 |
| ebi-a-GCST90001709 | rs117705111 | 22 | 41957510 | A | 0.0365 | -0.7505 | 0.06509 | 3.09E-30 |
| ebi-a-GCST90001719 | rs117705111 | 22 | 41957510 | A | 0.0365 | -0.7498 | 0.06509 | 3.48E-30 |
| ebi-a-GCST90001829 | rs117705111 | 22 | 41957510 | A | 0.0365 | -0.7406 | 0.06518 | 1.97E-29 |
| ebi-a-GCST90001708 | rs117705111 | 22 | 41957510 | A | 0.0365 | -0.7389 | 0.0653 | 3.37E-29 |
| ebi-a-GCST90001705 | rs117705111 | 22 | 41957510 | A | 0.0364 | -0.7379 | 0.06516 | 3.00E-29 |
| ebi-a-GCST90001706 | rs117705111 | 22 | 41957510 | A | 0.0346 | -0.7129 | 0.09169 | 1.25E-14 |
| ebi-a-GCST90001720 | rs117705111 | 22 | 41957510 | A | 0.0365 | -0.6734 | 0.06556 | 2.04E-24 |
| ebi-a-GCST90001695 | rs117708200 | 1 | 121273212 | G | 9.00E-04 | 284.3 | 52.5 | 6.56E-08 |
| ebi-a-GCST90001700 | rs117708929 | 6 | 124879601 | C | 0.0134 | 3.153 | 0.6419 | 9.43E-07 |
| ebi-a-GCST90002056 | rs11771058 | 7 | 53008596 | C | 0.3748 | 0.1285 | 0.02808 | 4.94E-06 |
| ebi-a-GCST90001930 | rs117712600 | 15 | 29470184 | T | 0.0271 | -0.411 | 0.08435 | 1.16E-06 |
| ebi-a-GCST90002040 | rs117718780 | 22 | 28335603 | T | 0.0025 | -1.333 | 0.2906 | 4.70E-06 |
| ebi-a-GCST90001452 | rs11772116 | 7 | 23594922 | C | 0.0766 | 0.225 | 0.04872 | 4.02E-06 |
| ebi-a-GCST90001990 | rs11772145 | 7 | 67584032 | A | 0.1054 | -0.1849 | 0.04036 | 4.78E-06 |
| ebi-a-GCST90001513 | rs11772999 | 7 | 113991516 | G | 0.0078 | 0.6586 | 0.1437 | 4.77E-06 |
| ebi-a-GCST90001521 | rs117734358 | 18 | 13088666 | T | 0.0156 | 0.6437 | 0.1354 | 2.13E-06 |
| ebi-a-GCST90001860 | rs117734680 | 12 | 12952560 | G | 0.0031 | -1.156 | 0.2478 | 3.22E-06 |
| ebi-a-GCST90001832 | rs11773763 | 7 | 50271499 | T | 0.2587 | -0.1856 | 0.03003 | 7.29E-10 |
| ebi-a-GCST90001833 | rs11773763 | 7 | 50271499 | T | 0.2588 | -0.1607 | 0.03014 | 1.04E-07 |
| ebi-a-GCST90001963 | rs117740023 | 10 | 52741522 | C | 0.0042 | 1.015 | 0.2093 | 1.31E-06 |
| ebi-a-GCST90001698 | rs117741105 | 8 | 112234506 | A | 0.0021 | 260.9 | 40.57 | 1.43E-10 |
| ebi-a-GCST90002046 | rs117745284 | 11 | 95746999 | T | 6.00E-04 | -3.259 | 0.6949 | 2.96E-06 |
| ebi-a-GCST90001698 | rs117745825 | 18 | 3305228 | C | 6.00E-04 | 854.9 | 74.11 | 3.13E-30 |
| ebi-a-GCST90002013 | rs11775437 | 8 | 139675584 | G | 0.0026 | 1.197 | 0.2599 | 4.31E-06 |
| ebi-a-GCST90001505 | rs117760133 | 8 | 129187397 | T | 0.07 | 0.2309 | 0.04892 | 2.45E-06 |
| ebi-a-GCST90001878 | rs117761782 | 8 | 22024980 | T | 0.0517 | -0.4469 | 0.09389 | 2.17E-06 |
| ebi-a-GCST90001538 | rs117765614 | 17 | 39786505 | T | 0.02 | 0.4341 | 0.09389 | 3.91E-06 |
| ebi-a-GCST90002086 | rs117767550 | 12 | 98809414 | T | 0.0436 | -0.3058 | 0.06567 | 3.36E-06 |
| ebi-a-GCST90001460 | rs11776881 | 8 | 131805603 | C | 0.4092 | 0.1139 | 0.02439 | 3.14E-06 |
| ebi-a-GCST90002075 | rs11777012 | 8 | 51754521 | C | 0.0654 | 0.2654 | 0.0554 | 1.75E-06 |
| ebi-a-GCST90001429 | rs117772462 | 7 | 48791017 | A | 0.0159 | -0.4644 | 0.09659 | 1.59E-06 |
| ebi-a-GCST90002041 | rs117772551 | 10 | 56075362 | T | 0.0498 | -0.3833 | 0.08282 | 3.99E-06 |
| ebi-a-GCST90001774 | rs117772689 | 20 | 40633066 | G | 0.0222 | -0.3828 | 0.08249 | 3.59E-06 |
| ebi-a-GCST90001772 | rs117772689 | 20 | 40633066 | G | 0.0222 | -0.3796 | 0.08267 | 4.56E-06 |
| ebi-a-GCST90001562 | rs117773023 | 8 | 3996923 | C | 3.00E-04 | 2.855 | 0.5864 | 1.18E-06 |
| ebi-a-GCST90001489 | rs11777493 | 8 | 113921646 | A | 0.2395 | -0.1467 | 0.0305 | 1.57E-06 |
| ebi-a-GCST90001517 | rs11777538 | 8 | 127623130 | G | 0.1889 | -0.1958 | 0.04267 | 4.78E-06 |
| ebi-a-GCST90001427 | rs11777835 | 8 | 144986103 | C | 0.5715 | -0.1246 | 0.02505 | 6.83E-07 |
| ebi-a-GCST90001403 | rs11777835 | 8 | 144986103 | C | 0.5715 | -0.1238 | 0.02489 | 6.83E-07 |
| ebi-a-GCST90001532 | rs117780942 | 11 | 105062174 | A | 0.0269 | -0.5318 | 0.1058 | 5.45E-07 |
| ebi-a-GCST90001531 | rs117780942 | 11 | 105062174 | A | 0.0269 | -0.4877 | 0.1062 | 4.64E-06 |
| ebi-a-GCST90001701 | rs117783732 | 16 | 78797349 | G | 0.0084 | -0.6863 | 0.1343 | 3.41E-07 |
| ebi-a-GCST90001700 | rs117785622 | 12 | 132283582 | T | 0.001 | 13.02 | 2.395 | 5.79E-08 |
| ebi-a-GCST90001695 | rs117785622 | 12 | 132283582 | T | 0.001 | 232.5 | 42.11 | 3.63E-08 |
| ebi-a-GCST90001698 | rs117785622 | 12 | 132283582 | T | 0.001 | 573 | 57.77 | 7.11E-23 |
| ebi-a-GCST90001791 | rs117788286 | 13 | 19370199 | C | 0.0463 | -0.2929 | 0.0623 | 2.69E-06 |
| ebi-a-GCST90001688 | rs117795441 | 17 | 54735696 | A | 3.00E-04 | 2.115 | 0.4548 | 3.42E-06 |
| ebi-a-GCST90001825 | rs117797158 | 17 | 78954840 | A | 0.0351 | 0.3199 | 0.06854 | 3.15E-06 |
| ebi-a-GCST90001821 | rs117797158 | 17 | 78954840 | A | 0.0351 | 0.3316 | 0.06818 | 1.20E-06 |
| ebi-a-GCST90001698 | rs117801457 | 15 | 29421113 | T | 0.0034 | 224.8 | 31.22 | 7.31E-13 |
| ebi-a-GCST90001695 | rs117804407 | 16 | 5944116 | G | NA | 2185 | 391 | 2.46E-08 |
| ebi-a-GCST90001698 | rs117808048 | 8 | 73079210 | A | 0.0026 | 206.4 | 37.47 | 3.85E-08 |
| ebi-a-GCST90001766 | rs11781090 | 8 | 144871633 | T | 0.6622 | 0.1316 | 0.02643 | 6.71E-07 |
| ebi-a-GCST90001839 | rs117820643 | 10 | 480161 | A | 0.0361 | -0.3449 | 0.07401 | 3.30E-06 |
| ebi-a-GCST90001698 | rs117822024 | 6 | 86346609 | C | 0.0079 | 141 | 20.2 | 3.50E-12 |
| ebi-a-GCST90001707 | rs117826659 | 22 | 39920299 | A | 0.0065 | -0.9788 | 0.2051 | 1.97E-06 |
| ebi-a-GCST90001698 | rs117830252 | 17 | 11465523 | A | 0.0032 | 165.2 | 30.55 | 6.77E-08 |
| ebi-a-GCST90001698 | rs117840149 | 22 | 36506460 | A | 0.0021 | 246.8 | 37.81 | 7.63E-11 |
| ebi-a-GCST90001695 | rs117845490 | 7 | 113248370 | C | 0.0022 | 152.1 | 30.1 | 4.58E-07 |
| ebi-a-GCST90001457 | rs11785252 | 8 | 117400280 | A | 0.7429 | -0.1459 | 0.03084 | 2.32E-06 |
| ebi-a-GCST90001680 | rs117858455 | 7 | 70457913 | T | 0.0493 | 0.2962 | 0.05979 | 7.66E-07 |
| ebi-a-GCST90001491 | rs117858605 | 12 | 81434565 | G | 0.0029 | 1.041 | 0.22 | 2.34E-06 |
| ebi-a-GCST90001699 | rs117858699 | 7 | 128071585 | T | 0.0136 | 7.144 | 1.487 | 1.62E-06 |
| ebi-a-GCST90001698 | rs117862145 | 14 | 33586273 | T | 0.0019 | 260.8 | 39.75 | 6.23E-11 |
| ebi-a-GCST90001695 | rs117863142 | 16 | 79245986 | A | 0.0035 | 117.3 | 22.14 | 1.25E-07 |
| ebi-a-GCST90001700 | rs117867588 | 8 | 15699657 | A | 1.00E-04 | 36.56 | 6.272 | 6.06E-09 |
| ebi-a-GCST90001509 | rs117882514 | 18 | 6105334 | C | 0.0319 | 0.3547 | 0.07102 | 6.17E-07 |
| ebi-a-GCST90001698 | rs117885295 | 22 | 27731404 | A | 0.0041 | 139.4 | 26.93 | 2.41E-07 |
| ebi-a-GCST90001955 | rs117893093 | 20 | 36543774 | T | 0.0418 | 0.4385 | 0.09362 | 3.06E-06 |
| ebi-a-GCST90001969 | rs117893932 | 18 | 56570039 | G | 0.0501 | 0.2818 | 0.06153 | 4.84E-06 |
| ebi-a-GCST90001773 | rs117896078 | 9 | 130751060 | T | 0.0285 | -0.3455 | 0.07496 | 4.20E-06 |
| ebi-a-GCST90001698 | rs117898578 | 16 | 50471284 | G | 0.0026 | 221 | 37.13 | 2.91E-09 |
| ebi-a-GCST90001561 | rs117900773 | 11 | 97410635 | T | 0.0179 | 0.3649 | 0.07955 | 4.67E-06 |
| ebi-a-GCST90001449 | rs117909439 | 14 | 29918210 | T | 0.0089 | -0.6761 | 0.1414 | 1.81E-06 |
| ebi-a-GCST90001925 | rs117913408 | 6 | 102173117 | G | 0.0315 | 0.3858 | 0.07822 | 8.57E-07 |
| ebi-a-GCST90001848 | rs117913408 | 6 | 102173117 | G | 0.0321 | 0.4285 | 0.07737 | 3.31E-08 |
| ebi-a-GCST90001698 | rs117922572 | 21 | 27289871 | T | 0.0032 | 172.1 | 31.46 | 4.77E-08 |
| ebi-a-GCST90001698 | rs117922931 | 17 | 30028367 | G | 6.00E-04 | 785.2 | 70.89 | 4.89E-28 |
| ebi-a-GCST90001986 | rs117925981 | 10 | 112247155 | T | 0.07 | -0.2256 | 0.04728 | 1.90E-06 |
| ebi-a-GCST90001688 | rs117927481 | 12 | 49315200 | A | 1.00E-04 | 3.783 | 0.6683 | 1.63E-08 |
| ebi-a-GCST90002111 | rs117936815 | 10 | 92271546 | C | 0.0425 | 0.4059 | 0.08759 | 3.88E-06 |
| ebi-a-GCST90001996 | rs117940302 | 8 | 123834904 | A | 0.0226 | 0.4039 | 0.08531 | 2.28E-06 |
| ebi-a-GCST90001695 | rs117940828 | 17 | 56243643 | C | NA | 763.1 | 152.4 | 5.79E-07 |
| ebi-a-GCST90001576 | rs117943886 | 13 | 95198601 | T | 0.0593 | 0.2444 | 0.05277 | 3.77E-06 |
| ebi-a-GCST90001578 | rs117943886 | 13 | 95198601 | T | 0.0594 | 0.2573 | 0.05195 | 7.63E-07 |
| ebi-a-GCST90001432 | rs11794578 | 9 | 808529 | G | 0.4521 | 0.1189 | 0.02437 | 1.12E-06 |
| ebi-a-GCST90001901 | rs117957417 | 10 | 96597621 | A | 0.0257 | 0.4033 | 0.08514 | 2.28E-06 |
| ebi-a-GCST90002089 | rs117968915 | 16 | 47869527 | G | 0.0144 | 0.542 | 0.1169 | 3.69E-06 |
| ebi-a-GCST90001906 | rs117974128 | 11 | 100371319 | T | 0.0888 | -0.2269 | 0.04952 | 4.78E-06 |
| ebi-a-GCST90001697 | rs117975109 | 12 | 52408142 | T | 6.00E-04 | 15.84 | 2.869 | 3.65E-08 |
| ebi-a-GCST90001695 | rs117975109 | 12 | 52408142 | T | 6.00E-04 | 308.9 | 61.24 | 4.82E-07 |
| ebi-a-GCST90001698 | rs117983267 | 8 | 124174485 | A | 0.0079 | 111 | 19.94 | 2.77E-08 |
| ebi-a-GCST90001552 | rs118003243 | 21 | 43795055 | C | 0.016 | 0.3428 | 0.07388 | 3.63E-06 |
| ebi-a-GCST90001497 | rs118006237 | 1 | 100480230 | A | 7.00E-04 | 1.921 | 0.4076 | 2.53E-06 |
| ebi-a-GCST90001490 | rs118006237 | 1 | 100480230 | A | 7.00E-04 | 1.942 | 0.4013 | 1.36E-06 |
| ebi-a-GCST90001496 | rs118006237 | 1 | 100480230 | A | 7.00E-04 | 1.987 | 0.4127 | 1.54E-06 |
| ebi-a-GCST90001557 | rs118009051 | 21 | 17079538 | G | 0.0311 | 0.3408 | 0.07431 | 4.69E-06 |
| ebi-a-GCST90001698 | rs118014538 | 11 | 3830358 | A | 0.004 | 248.6 | 28.49 | 4.08E-18 |
| ebi-a-GCST90001688 | rs118016388 | 12 | 28662288 | C | NA | 7.82 | 1.424 | 4.27E-08 |
| ebi-a-GCST90001698 | rs118019384 | 17 | 27826991 | C | 0.0254 | 56.72 | 11.4 | 6.89E-07 |
| ebi-a-GCST90002061 | rs118019481 | 10 | 95151086 | A | 0.0183 | 0.497 | 0.1042 | 1.95E-06 |
| ebi-a-GCST90002083 | rs11801958 | 1 | 7176679 | T | 0.4853 | 0.1307 | 0.02678 | 1.11E-06 |
| ebi-a-GCST90002086 | rs11801958 | 1 | 7176679 | T | 0.4853 | 0.1397 | 0.0266 | 1.61E-07 |
| ebi-a-GCST90001695 | rs118024980 | 9 | 9009338 | C | 0.0156 | 50.48 | 10.85 | 3.38E-06 |
| ebi-a-GCST90001698 | rs118044214 | 7 | 147886758 | A | 0.0044 | 137.5 | 26.75 | 2.89E-07 |
| ebi-a-GCST90001470 | rs118054784 | 8 | 130658684 | A | 0.0422 | 0.2985 | 0.0621 | 1.60E-06 |
| ebi-a-GCST90001698 | rs118062750 | 9 | 29889453 | T | 0.0028 | 224.1 | 32 | 3.03E-12 |
| ebi-a-GCST90001614 | rs118063063 | 10 | 83179890 | T | 0.0192 | 0.4447 | 0.08957 | 7.19E-07 |
| ebi-a-GCST90001838 | rs118065562 | 12 | 2419083 | A | 0.0108 | -0.6149 | 0.1327 | 3.77E-06 |
| ebi-a-GCST90001842 | rs118065562 | 12 | 2419083 | A | 0.0108 | -0.6044 | 0.1307 | 3.92E-06 |
| ebi-a-GCST90001698 | rs118065847 | 16 | 54673316 | A | 0.0028 | 180.4 | 34.8 | 2.30E-07 |
| ebi-a-GCST90001882 | rs11807062 | 1 | 3153237 | C | 0.1239 | 0.2812 | 0.06005 | 3.14E-06 |
| ebi-a-GCST90001423 | rs118073973 | 9 | 19948908 | A | 0.0402 | 0.2899 | 0.06184 | 2.87E-06 |
| ebi-a-GCST90001444 | rs118073973 | 9 | 19948908 | A | 0.0402 | 0.2924 | 0.06168 | 2.21E-06 |
| ebi-a-GCST90001698 | rs118076041 | 8 | 144562245 | A | 0.0022 | 248.5 | 37.09 | 2.41E-11 |
| ebi-a-GCST90001434 | rs118076634 | 11 | 94589940 | T | 0.0025 | 1.115 | 0.2283 | 1.08E-06 |
| ebi-a-GCST90001523 | rs118076832 | 15 | 52532599 | G | 0.0143 | 0.678 | 0.1447 | 2.99E-06 |
| ebi-a-GCST90001687 | rs118079184 | 8 | 9002188 | A | 0.0634 | -0.2399 | 0.0518 | 3.77E-06 |
| ebi-a-GCST90001695 | rs118082680 | 8 | 21084293 | C | 0.001 | 217 | 42.86 | 4.34E-07 |
| ebi-a-GCST90001419 | rs118084343 | 11 | 17687837 | G | 0.0547 | -0.2663 | 0.0544 | 1.03E-06 |
| ebi-a-GCST90001698 | rs118084393 | 10 | 6554827 | C | 0.0037 | 144.2 | 29.23 | 8.51E-07 |
| ebi-a-GCST90001426 | rs118086652 | 15 | 76646870 | G | 0.0213 | 0.38 | 0.08281 | 4.61E-06 |
| ebi-a-GCST90001824 | rs118089299 | 14 | 104674020 | T | 0.0261 | -0.3915 | 0.07811 | 5.63E-07 |
| ebi-a-GCST90001700 | rs118089863 | 19 | 24477916 | T | 0.0018 | 12.62 | 2.243 | 1.98E-08 |
| ebi-a-GCST90001698 | rs118089863 | 19 | 24477916 | T | 0.0018 | 516.5 | 52.39 | 1.25E-22 |
| ebi-a-GCST90001398 | rs118092259 | 8 | 89432719 | G | 0.0414 | -0.2912 | 0.06306 | 4.00E-06 |
| ebi-a-GCST90001418 | rs118092259 | 8 | 89432719 | G | 0.0414 | -0.2901 | 0.06345 | 4.98E-06 |
| ebi-a-GCST90001726 | rs118099810 | 16 | 2406183 | T | 0.0572 | 0.2451 | 0.05343 | 4.63E-06 |
| ebi-a-GCST90001533 | rs118101102 | 6 | 79907097 | G | 0.0159 | -0.5982 | 0.1303 | 4.75E-06 |
| ebi-a-GCST90001697 | rs118106010 | 15 | 88790835 | C | 6.00E-04 | 12.27 | 2.63 | 3.19E-06 |
| ebi-a-GCST90001828 | rs118108236 | 6 | 94238065 | T | 0.0223 | 0.3864 | 0.08288 | 3.24E-06 |
| ebi-a-GCST90001960 | rs118110714 | 12 | 16060925 | A | 0.0031 | 1.094 | 0.2367 | 3.94E-06 |
| ebi-a-GCST90001933 | rs118110714 | 12 | 16060925 | A | 0.0031 | 1.117 | 0.2272 | 9.22E-07 |
| ebi-a-GCST90001496 | rs118111902 | 11 | 19624351 | C | 0.0179 | 0.466 | 0.09549 | 1.11E-06 |
| ebi-a-GCST90001484 | rs118112011 | 10 | 97204949 | G | 0.0095 | 0.647 | 0.1268 | 3.50E-07 |
| ebi-a-GCST90001467 | rs11811863 | 1 | 247577856 | T | 0.2186 | -0.1537 | 0.03086 | 6.63E-07 |
| ebi-a-GCST90002025 | rs118120903 | 8 | 30276043 | T | 0.0285 | -0.414 | 0.08345 | 7.41E-07 |
| ebi-a-GCST90002022 | rs118120903 | 8 | 30276043 | T | 0.0285 | -0.3906 | 0.08422 | 3.67E-06 |
| ebi-a-GCST90001901 | rs118122984 | 10 | 122084798 | G | 6.00E-04 | -2.668 | 0.567 | 2.67E-06 |
| ebi-a-GCST90001562 | rs118124576 | 18 | 13390635 | G | 0.0051 | -0.7578 | 0.1525 | 7.00E-07 |
| ebi-a-GCST90001560 | rs118124576 | 18 | 13390635 | G | 0.0052 | -0.6898 | 0.149 | 3.81E-06 |
| ebi-a-GCST90001698 | rs118124593 | 7 | 134350922 | A | 0.0043 | 146.7 | 28.68 | 3.28E-07 |
| ebi-a-GCST90001670 | rs11812465 | 10 | 91738825 | C | 0.3432 | -0.1378 | 0.02735 | 4.91E-07 |
| ebi-a-GCST90001688 | rs118127261 | 8 | 39979373 | A | 1.00E-04 | 3.614 | 0.6762 | 9.65E-08 |
| ebi-a-GCST90001723 | rs118134186 | 13 | 29813364 | C | 0.0011 | -1.89 | 0.4129 | 4.88E-06 |
| ebi-a-GCST90001466 | rs118148569 | 16 | 13019834 | T | 0.0505 | 0.2729 | 0.05772 | 2.36E-06 |
| ebi-a-GCST90001872 | rs118151702 | 8 | 138502532 | T | 0.0421 | 0.4464 | 0.09669 | 4.29E-06 |
| ebi-a-GCST90002042 | rs1181554 | 6 | 106926228 | T | 0.4424 | 0.1762 | 0.03675 | 1.78E-06 |
| ebi-a-GCST90002007 | rs118167703 | 6 | 131880206 | T | 0.0514 | 0.2638 | 0.05749 | 4.60E-06 |
| ebi-a-GCST90001985 | rs118170720 | 11 | 26568364 | T | 0.0493 | 0.2847 | 0.05733 | 7.18E-07 |
| ebi-a-GCST90001828 | rs118171108 | 17 | 3853980 | C | 0.0242 | 0.3961 | 0.08107 | 1.08E-06 |
| ebi-a-GCST90001700 | rs118180479 | 20 | 37027035 | C | 0.001 | 11.65 | 2.356 | 7.99E-07 |
| ebi-a-GCST90001698 | rs118180479 | 20 | 37027035 | C | 0.001 | 534.9 | 55.24 | 6.75E-22 |
| ebi-a-GCST90001670 | rs11818051 | 10 | 97972294 | T | 0.0317 | 0.6769 | 0.07397 | 9.35E-20 |
| ebi-a-GCST90001671 | rs11818051 | 10 | 97972294 | T | 0.0317 | 0.6977 | 0.07403 | 7.75E-21 |
| ebi-a-GCST90001485 | rs11818051 | 10 | 97972294 | T | 0.0317 | 0.6998 | 0.0719 | 4.24E-22 |
| ebi-a-GCST90001672 | rs11818051 | 10 | 97972294 | T | 0.0317 | 0.7156 | 0.07506 | 2.79E-21 |
| ebi-a-GCST90001763 | rs118186161 | 19 | 17549328 | G | 0.0643 | -0.2376 | 0.05084 | 3.06E-06 |
| ebi-a-GCST90001657 | rs11818898 | 10 | 87516854 | G | 0.0467 | 0.2735 | 0.05186 | 1.42E-07 |
| ebi-a-GCST90001826 | rs1181899 | 7 | 76993184 | C | 0.8967 | 0.1892 | 0.04091 | 3.88E-06 |
| ebi-a-GCST90001742 | rs11825488 | 11 | 72165045 | A | 0.0021 | -1.289 | 0.2552 | 4.60E-07 |
| ebi-a-GCST90001737 | rs11825488 | 11 | 72165045 | A | 0.0021 | -1.158 | 0.2525 | 4.70E-06 |
| ebi-a-GCST90001911 | rs1182654 | 9 | 19731896 | T | 0.4088 | 0.1297 | 0.02755 | 2.61E-06 |
| ebi-a-GCST90001910 | rs11830137 | 12 | 10383388 | A | 0.0834 | 0.2168 | 0.04546 | 1.93E-06 |
| ebi-a-GCST90001554 | rs1183901 | 10 | 90231718 | C | 0.5872 | -0.1356 | 0.02714 | 6.17E-07 |
| ebi-a-GCST90002090 | rs11841867 | 13 | 29024953 | A | 0.0725 | 0.253 | 0.05476 | 4.02E-06 |
| ebi-a-GCST90001553 | rs11843149 | 13 | 24258336 | C | 0.2256 | 0.09414 | 0.02024 | 3.42E-06 |
| ebi-a-GCST90001601 | rs11850806 | 14 | 78669137 | G | 0.0083 | -0.6792 | 0.1311 | 2.34E-07 |
| ebi-a-GCST90001603 | rs11850806 | 14 | 78669137 | G | 0.0083 | -0.6286 | 0.1291 | 1.17E-06 |
| ebi-a-GCST90001498 | rs11850806 | 14 | 78669137 | G | 0.0085 | -0.6053 | 0.1301 | 3.41E-06 |
| ebi-a-GCST90001466 | rs11852100 | 14 | 36478046 | C | 0.0587 | 0.2639 | 0.0548 | 1.53E-06 |
| ebi-a-GCST90001580 | rs11856889 | 15 | 24443663 | T | 0.318 | -0.1219 | 0.02604 | 2.93E-06 |
| ebi-a-GCST90001935 | rs11861637 | 16 | 68656715 | T | 0.036 | 0.3202 | 0.06753 | 2.20E-06 |
| ebi-a-GCST90001440 | rs11862268 | 16 | 81239867 | A | 0.0187 | 0.4267 | 0.09042 | 2.46E-06 |
| ebi-a-GCST90001581 | rs11865775 | 16 | 5752110 | T | 0.9212 | -0.2121 | 0.04522 | 2.81E-06 |
| ebi-a-GCST90001591 | rs11869223 | 17 | 33775713 | G | 0.4388 | -0.1197 | 0.02447 | 1.03E-06 |
| ebi-a-GCST90001479 | rs11869223 | 17 | 33775713 | G | 0.4364 | -0.1191 | 0.02574 | 3.89E-06 |
| ebi-a-GCST90001506 | rs11869223 | 17 | 33775713 | G | 0.4364 | -0.1182 | 0.02535 | 3.22E-06 |
| ebi-a-GCST90001698 | rs11879282 | 19 | 48790968 | A | 0.0012 | 227.5 | 49.6 | 4.67E-06 |
| ebi-a-GCST90001946 | rs11882720 | 19 | 52897142 | G | 0.0731 | -0.4764 | 0.06809 | 3.79E-12 |
| ebi-a-GCST90001948 | rs11882720 | 19 | 52897142 | G | 0.0726 | -0.4484 | 0.06841 | 7.43E-11 |
| ebi-a-GCST90001953 | rs11882720 | 19 | 52897142 | G | 0.0696 | -0.3831 | 0.07227 | 1.32E-07 |
| ebi-a-GCST90001851 | rs11885872 | 2 | 104570579 | G | 0.0046 | -0.951 | 0.1993 | 1.92E-06 |
| ebi-a-GCST90001485 | rs11890666 | 2 | 38745822 | A | 0.2866 | -0.1612 | 0.02787 | 7.94E-09 |
| ebi-a-GCST90001483 | rs11890666 | 2 | 38745822 | A | 0.2875 | -0.1566 | 0.02815 | 2.88E-08 |
| ebi-a-GCST90002077 | rs11891318 | 2 | 130608376 | G | 0.105 | 0.2138 | 0.04415 | 1.35E-06 |
| ebi-a-GCST90001970 | rs11891318 | 2 | 130608376 | G | 0.105 | 0.243 | 0.0444 | 4.83E-08 |
| ebi-a-GCST90001555 | rs11896593 | 2 | 38925155 | C | 0.3707 | -0.2549 | 0.02205 | 2.39E-30 |
| ebi-a-GCST90001689 | rs11896593 | 2 | 38925155 | C | 0.3703 | 0.1024 | 0.01981 | 2.50E-07 |
| ebi-a-GCST90001690 | rs11896593 | 2 | 38925155 | C | 0.3706 | 0.1051 | 0.01838 | 1.17E-08 |
| ebi-a-GCST90001560 | rs11896593 | 2 | 38925155 | C | 0.371 | 0.197 | 0.02252 | 3.31E-18 |
| ebi-a-GCST90001561 | rs11896593 | 2 | 38925155 | C | 0.3707 | 0.2701 | 0.02208 | 1.04E-33 |
| ebi-a-GCST90002034 | rs11896747 | 2 | 43764398 | G | 0.477 | 0.1391 | 0.02893 | 1.60E-06 |
| ebi-a-GCST90001695 | rs11899522 | 2 | 54396879 | T | 0.0398 | 35.09 | 6.871 | 3.46E-07 |
| ebi-a-GCST90002117 | rs11901763 | 2 | 235192580 | T | 0.066 | 0.2647 | 0.05523 | 1.72E-06 |
| ebi-a-GCST90001981 | rs11906777 | 20 | 39374731 | A | 0.1142 | 0.2037 | 0.03963 | 2.89E-07 |
| ebi-a-GCST90002089 | rs11913696 | 22 | 19827328 | G | 0.4981 | 0.1286 | 0.0281 | 4.96E-06 |
| ebi-a-GCST90001698 | rs11921864 | 3 | 140104765 | T | 0.0585 | 35.49 | 7.68 | 3.97E-06 |
| ebi-a-GCST90001727 | rs11926501 | 3 | 9722257 | T | 0.059 | -0.3399 | 0.07215 | 2.65E-06 |
| ebi-a-GCST90002032 | rs11929716 | 4 | 28719206 | C | 0.0435 | -0.3283 | 0.06784 | 1.37E-06 |
| ebi-a-GCST90001653 | rs11930576 | 4 | 169595404 | C | 0.2746 | -0.1344 | 0.02812 | 1.83E-06 |
| ebi-a-GCST90001656 | rs11930576 | 4 | 169595404 | C | 0.2746 | 0.1344 | 0.02812 | 1.83E-06 |
| ebi-a-GCST90001987 | rs11934532 | 4 | 182164089 | G | 0.217 | 0.1431 | 0.03052 | 2.86E-06 |
| ebi-a-GCST90002006 | rs11934532 | 4 | 182164089 | G | 0.217 | 0.1539 | 0.03068 | 5.53E-07 |
| ebi-a-GCST90001450 | rs11935230 | 4 | 136447876 | C | 0.1686 | 0.1842 | 0.03607 | 3.50E-07 |
| ebi-a-GCST90001680 | rs11935691 | 4 | 166715039 | T | 0.0419 | -0.2994 | 0.06338 | 2.41E-06 |
| ebi-a-GCST90001916 | rs11944009 | 4 | 1568839 | T | 0.3913 | 0.1236 | 0.02657 | 3.44E-06 |
| ebi-a-GCST90001646 | rs11954223 | 5 | 67413499 | A | 0.001 | -2.093 | 0.3644 | 9.93E-09 |
| ebi-a-GCST90001647 | rs11954223 | 5 | 67413499 | A | 0.001 | -1.682 | 0.3583 | 2.77E-06 |
| ebi-a-GCST90001643 | rs11954223 | 5 | 67413499 | A | 0.001 | 2.135 | 0.3629 | 4.36E-09 |
| ebi-a-GCST90002060 | rs11964370 | 6 | 75445964 | A | 0.0366 | 0.328 | 0.07103 | 4.05E-06 |
| ebi-a-GCST90002058 | rs11964370 | 6 | 75445964 | A | 0.0361 | 0.3338 | 0.07146 | 3.12E-06 |
| ebi-a-GCST90001940 | rs11968423 | 6 | 25422353 | T | 0.1392 | 0.1758 | 0.03715 | 2.31E-06 |
| ebi-a-GCST90001935 | rs11968423 | 6 | 25422353 | T | 0.1392 | 0.1834 | 0.03686 | 6.82E-07 |
| ebi-a-GCST90002104 | rs11979208 | 7 | 80529639 | C | 0.0681 | 0.2542 | 0.05489 | 3.79E-06 |
| ebi-a-GCST90001872 | rs11981290 | 7 | 154686456 | A | 0.2983 | -0.2026 | 0.04387 | 4.28E-06 |
| ebi-a-GCST90001899 | rs11990425 | 8 | 21557285 | T | 0.3147 | -0.1342 | 0.02785 | 1.52E-06 |
| ebi-a-GCST90001602 | rs11998531 | 8 | 120860297 | G | 0.1924 | -0.1499 | 0.03173 | 2.41E-06 |
| ebi-a-GCST90001605 | rs11998531 | 8 | 120860297 | G | 0.1924 | -0.1464 | 0.03082 | 2.13E-06 |
| ebi-a-GCST90001652 | rs11998531 | 8 | 120860297 | G | 0.1924 | 0.1563 | 0.03204 | 1.12E-06 |
| ebi-a-GCST90002024 | rs12000134 | 9 | 3108492 | G | 0.1405 | 0.189 | 0.04017 | 2.67E-06 |
| ebi-a-GCST90001512 | rs12006014 | 9 | 20160505 | A | 0.1158 | 0.183 | 0.03935 | 3.42E-06 |
| ebi-a-GCST90001527 | rs1201943 | 20 | 59282409 | T | 0.5054 | -0.1576 | 0.03144 | 5.88E-07 |
| ebi-a-GCST90001584 | rs1202098 | 6 | 73301721 | A | 0.011 | -0.5784 | 0.1263 | 4.81E-06 |
| ebi-a-GCST90001997 | rs12023218 | 1 | 161269897 | G | 0.4976 | 0.1277 | 0.02531 | 4.69E-07 |
| ebi-a-GCST90001763 | rs12026973 | 1 | 107827638 | T | 0.1162 | -0.1927 | 0.03837 | 5.37E-07 |
| ebi-a-GCST90001858 | rs12027506 | 1 | 167493088 | T | 0.0449 | 0.3141 | 0.06683 | 2.72E-06 |
| ebi-a-GCST90002100 | rs12027761 | 1 | 247974959 | T | 0.8802 | 0.1978 | 0.04085 | 1.35E-06 |
| ebi-a-GCST90002061 | rs12027949 | 1 | 218448425 | C | 0.2265 | 0.1582 | 0.03254 | 1.22E-06 |
| ebi-a-GCST90001627 | rs12029484 | 1 | 230111146 | G | 0.0768 | -0.2266 | 0.0462 | 9.79E-07 |
| ebi-a-GCST90001628 | rs12029484 | 1 | 230111146 | G | 0.0769 | -0.2178 | 0.04445 | 1.00E-06 |
| ebi-a-GCST90001629 | rs12029484 | 1 | 230111146 | G | 0.0769 | -0.2164 | 0.04509 | 1.66E-06 |
| ebi-a-GCST90001761 | rs12030328 | 1 | 24051614 | G | 0.6349 | -0.1301 | 0.02571 | 4.36E-07 |
| ebi-a-GCST90001850 | rs12032130 | 1 | 167156419 | T | 0.3475 | 0.143 | 0.02834 | 4.79E-07 |
| ebi-a-GCST90001816 | rs12032479 | 1 | 194997589 | T | 0.1797 | -0.1719 | 0.03259 | 1.40E-07 |
| ebi-a-GCST90001811 | rs12032479 | 1 | 194997589 | T | 0.1797 | -0.1554 | 0.03281 | 2.27E-06 |
| ebi-a-GCST90001519 | rs12033128 | 1 | 85552939 | C | 0.1389 | -0.262 | 0.04799 | 5.40E-08 |
| ebi-a-GCST90001392 | rs12036556 | 1 | 99149370 | T | 0.0265 | 0.3487 | 0.07421 | 2.71E-06 |
| ebi-a-GCST90002066 | rs12039573 | 1 | 20941515 | T | 0.2421 | -0.16 | 0.03193 | 5.72E-07 |
| ebi-a-GCST90002070 | rs12039573 | 1 | 20941515 | T | 0.2421 | -0.1519 | 0.0321 | 2.32E-06 |
| ebi-a-GCST90002063 | rs12039573 | 1 | 20941515 | T | 0.2421 | -0.1493 | 0.03195 | 3.11E-06 |
| ebi-a-GCST90001833 | rs12040885 | 1 | 167057766 | A | 0.5385 | -0.1219 | 0.02617 | 3.32E-06 |
| ebi-a-GCST90001833 | rs12041883 | 1 | 111320129 | A | 0.1277 | -0.2019 | 0.0393 | 2.97E-07 |
| ebi-a-GCST90001752 | rs12042193 | 1 | 116031657 | G | 0.3687 | -0.1223 | 0.02571 | 2.05E-06 |
| ebi-a-GCST90001759 | rs12042193 | 1 | 116031657 | G | 0.3687 | -0.1193 | 0.02575 | 3.76E-06 |
| ebi-a-GCST90001537 | rs12043060 | 1 | 157061973 | T | 0.4633 | 0.1224 | 0.0255 | 1.65E-06 |
| ebi-a-GCST90001480 | rs12044365 | 1 | 210431761 | G | 0.0248 | -0.3482 | 0.07468 | 3.24E-06 |
| ebi-a-GCST90002118 | rs12046688 | 1 | 47599360 | G | 0.1325 | 0.187 | 0.03957 | 2.40E-06 |
| ebi-a-GCST90001498 | rs12049737 | 10 | 2614502 | A | 0.0548 | 0.2633 | 0.05471 | 1.55E-06 |
| ebi-a-GCST90001522 | rs1205315 | 19 | 54838294 | G | 0.3114 | -0.1664 | 0.03605 | 4.19E-06 |
| ebi-a-GCST90001579 | rs12055035 | 5 | 173542221 | C | 0.7193 | -0.1261 | 0.02734 | 4.13E-06 |
| ebi-a-GCST90001695 | rs12062472 | 1 | 17431300 | T | 0.0012 | 172.5 | 37.71 | 4.91E-06 |
| ebi-a-GCST90001901 | rs12068559 | 1 | 6127983 | C | 0.8096 | -0.1602 | 0.03467 | 3.99E-06 |
| ebi-a-GCST90001700 | rs12068756 | 1 | 233943028 | C | 0.0263 | 2.264 | 0.4741 | 1.86E-06 |
| ebi-a-GCST90002094 | rs12073174 | 1 | 4002766 | G | 0.4904 | 0.1809 | 0.03701 | 1.13E-06 |
| ebi-a-GCST90001860 | rs12077809 | 1 | 167104790 | A | 0.1461 | -0.2013 | 0.03817 | 1.43E-07 |
| ebi-a-GCST90001698 | rs12080172 | 1 | 53470377 | G | 0.0697 | 36.06 | 7.292 | 7.94E-07 |
| ebi-a-GCST90001646 | rs12087279 | 1 | 82243292 | T | 0.131 | -0.1667 | 0.03634 | 4.64E-06 |
| ebi-a-GCST90001643 | rs12087279 | 1 | 82243292 | T | 0.131 | 0.1785 | 0.03618 | 8.39E-07 |
| ebi-a-GCST90001681 | rs12090749 | 1 | 204152575 | T | 0.0498 | -0.2815 | 0.05998 | 2.80E-06 |
| ebi-a-GCST90001516 | rs12094489 | 1 | 247286936 | A | 0.1373 | -0.2383 | 0.04837 | 9.09E-07 |
| ebi-a-GCST90001960 | rs12095517 | 1 | 11797186 | C | 0.6858 | -0.1496 | 0.02874 | 2.07E-07 |
| ebi-a-GCST90001869 | rs12096911 | 1 | 167532812 | C | 0.2947 | -0.1374 | 0.03001 | 4.90E-06 |
| ebi-a-GCST90001836 | rs12102694 | 16 | 76423281 | G | 0.4925 | 0.1786 | 0.03702 | 1.56E-06 |
| ebi-a-GCST90001490 | rs12106894 | 3 | 127066325 | C | 0.1079 | -0.1875 | 0.04072 | 4.30E-06 |
| ebi-a-GCST90001698 | rs12118378 | 1 | 68007080 | G | 0.0031 | 144.8 | 30.72 | 2.54E-06 |
| ebi-a-GCST90001592 | rs12119984 | 1 | 246874501 | G | 0.0084 | -0.6212 | 0.1311 | 2.24E-06 |
| ebi-a-GCST90001913 | rs12120358 | 1 | 161444369 | T | 0.2243 | -0.1561 | 0.03232 | 1.44E-06 |
| ebi-a-GCST90001535 | rs12121018 | 1 | 94176595 | A | 0.1882 | -0.1566 | 0.03132 | 6.00E-07 |
| ebi-a-GCST90001541 | rs12121018 | 1 | 94176595 | A | 0.1882 | 0.141 | 0.03063 | 4.31E-06 |
| ebi-a-GCST90002090 | rs12122013 | 1 | 162860370 | C | 0.6593 | -0.1452 | 0.02974 | 1.11E-06 |
| ebi-a-GCST90001989 | rs12122366 | 1 | 162563661 | T | 0.1034 | 0.2105 | 0.04101 | 3.00E-07 |
| ebi-a-GCST90001600 | rs12124698 | 1 | 246883687 | T | 0.007 | -0.8039 | 0.1584 | 4.09E-07 |
| ebi-a-GCST90001693 | rs12124698 | 1 | 246883687 | T | 0.0069 | -0.7813 | 0.1666 | 2.83E-06 |
| ebi-a-GCST90001825 | rs12126649 | 1 | 4153262 | C | 0.1338 | -0.1785 | 0.03685 | 1.32E-06 |
| ebi-a-GCST90001847 | rs12128267 | 1 | 222089002 | G | 0.198 | -0.1582 | 0.03404 | 3.50E-06 |
| ebi-a-GCST90001427 | rs12128328 | 1 | 59700800 | C | 0.2113 | 0.1428 | 0.0306 | 3.17E-06 |
| ebi-a-GCST90001808 | rs12129123 | 1 | 10083709 | T | 0.222 | 0.1494 | 0.03066 | 1.14E-06 |
| ebi-a-GCST90002073 | rs12129442 | 1 | 15139272 | T | 0.0842 | -0.2601 | 0.04999 | 2.11E-07 |
| ebi-a-GCST90001592 | rs12130486 | 1 | 81455274 | C | 0.0245 | 0.36 | 0.07639 | 2.54E-06 |
| ebi-a-GCST90001698 | rs12130486 | 1 | 81455274 | C | 0.0251 | 76.42 | 11.29 | 1.55E-11 |
| ebi-a-GCST90001859 | rs12132394 | 1 | 235247955 | A | 0.6839 | 0.1355 | 0.02887 | 2.80E-06 |
| ebi-a-GCST90001863 | rs12132394 | 1 | 235247955 | A | 0.684 | 0.1488 | 0.02858 | 2.07E-07 |
| ebi-a-GCST90001570 | rs12133465 | 1 | 195430336 | G | 0.0096 | -0.6622 | 0.1302 | 3.84E-07 |
| ebi-a-GCST90001482 | rs12133465 | 1 | 195430336 | G | 0.0096 | 0.6418 | 0.1191 | 7.60E-08 |
| ebi-a-GCST90001573 | rs12133465 | 1 | 195430336 | G | 0.0096 | 0.6423 | 0.1303 | 8.59E-07 |
| ebi-a-GCST90001481 | rs12133465 | 1 | 195430336 | G | 0.0096 | 0.7179 | 0.1158 | 6.33E-10 |
| ebi-a-GCST90001464 | rs12134798 | 1 | 120115384 | T | 0.1673 | 0.1639 | 0.03471 | 2.45E-06 |
| ebi-a-GCST90001987 | rs12135207 | 1 | 160944691 | T | 0.0271 | 0.4004 | 0.07887 | 4.02E-07 |
| ebi-a-GCST90001857 | rs12138291 | 1 | 166464476 | A | 0.1482 | -0.2836 | 0.0385 | 2.28E-13 |
| ebi-a-GCST90001853 | rs12138291 | 1 | 166464476 | A | 0.1482 | -0.2802 | 0.0384 | 3.82E-13 |
| ebi-a-GCST90001860 | rs12138291 | 1 | 166464476 | A | 0.1482 | -0.2773 | 0.03854 | 7.97E-13 |
| ebi-a-GCST90001864 | rs12138291 | 1 | 166464476 | A | 0.1482 | -0.2709 | 0.03832 | 1.93E-12 |
| ebi-a-GCST90001854 | rs12138291 | 1 | 166464476 | A | 0.1482 | -0.2588 | 0.03815 | 1.41E-11 |
| ebi-a-GCST90001867 | rs12138291 | 1 | 166464476 | A | 0.1482 | -0.2056 | 0.03786 | 6.07E-08 |
| ebi-a-GCST90001866 | rs12138291 | 1 | 166464476 | A | 0.1482 | -0.2 | 0.03811 | 1.66E-07 |
| ebi-a-GCST90001924 | rs12138848 | 1 | 210808177 | C | 0.0122 | 0.6181 | 0.1236 | 6.06E-07 |
| ebi-a-GCST90001790 | rs12139506 | 1 | 61376720 | G | 0.0543 | -0.2766 | 0.05366 | 2.68E-07 |
| ebi-a-GCST90001792 | rs12139506 | 1 | 61376720 | G | 0.0543 | -0.2726 | 0.05371 | 4.07E-07 |
| ebi-a-GCST90001777 | rs12139506 | 1 | 61376720 | G | 0.0543 | -0.2642 | 0.05361 | 8.65E-07 |
| ebi-a-GCST90001778 | rs12139506 | 1 | 61376720 | G | 0.0543 | -0.2488 | 0.05397 | 4.16E-06 |
| ebi-a-GCST90001593 | rs12139740 | 1 | 242332976 | T | 0.1465 | -0.1603 | 0.03454 | 3.59E-06 |
| ebi-a-GCST90002106 | rs12140470 | 1 | 115683097 | A | 0.1637 | -0.1759 | 0.03703 | 2.13E-06 |
| ebi-a-GCST90001930 | rs12141053 | 1 | 28882908 | G | 0.3664 | 0.1488 | 0.0289 | 2.81E-07 |
| ebi-a-GCST90001845 | rs12141563 | 1 | 26589636 | G | 0.1534 | -0.1858 | 0.03779 | 9.26E-07 |
| ebi-a-GCST90001482 | rs12141656 | 1 | 189081342 | G | 0.0092 | 0.6122 | 0.1213 | 4.73E-07 |
| ebi-a-GCST90001594 | rs12142860 | 1 | 215998585 | T | 0.0096 | 0.5894 | 0.1289 | 4.97E-06 |
| ebi-a-GCST90001636 | rs12143791 | 1 | 178104293 | G | 0.3443 | -0.1199 | 0.02601 | 4.19E-06 |
| ebi-a-GCST90001859 | rs12144916 | 1 | 167154534 | G | 0.3494 | 0.1391 | 0.02844 | 1.05E-06 |
| ebi-a-GCST90001919 | rs12145132 | 1 | 198638593 | G | 0.1462 | 0.209 | 0.03794 | 3.88E-08 |
| ebi-a-GCST90001867 | rs1214596 | 1 | 167427170 | A | 0.4464 | 0.4631 | 0.02573 | 9.36E-69 |
| ebi-a-GCST90001869 | rs1214596 | 1 | 167427170 | A | 0.4462 | 0.6523 | 0.02485 | 1.60E-136 |
| ebi-a-GCST90001431 | rs12146653 | 11 | 21411891 | C | 0.0964 | 0.1892 | 0.04126 | 4.66E-06 |
| ebi-a-GCST90001836 | rs12146661 | 11 | 132240457 | A | 0.1519 | 0.2623 | 0.05364 | 1.12E-06 |
| ebi-a-GCST90002091 | rs12147583 | 14 | 87896781 | A | 0.0012 | -2.441 | 0.5152 | 2.35E-06 |
| ebi-a-GCST90001548 | rs12149562 | 16 | 12222651 | C | 0.09 | -0.1945 | 0.04148 | 2.85E-06 |
| ebi-a-GCST90001899 | rs12152264 | 3 | 54889054 | T | 0.1248 | 0.1798 | 0.03861 | 3.34E-06 |
| ebi-a-GCST90001624 | rs12166155 | 22 | 44999166 | G | 0.3564 | 0.1146 | 0.025 | 4.70E-06 |
| ebi-a-GCST90001663 | rs12185529 | 19 | 3059754 | A | 0.0235 | 0.3958 | 0.08382 | 2.43E-06 |
| ebi-a-GCST90001661 | rs12185529 | 19 | 3059754 | A | 0.0234 | 0.3968 | 0.08367 | 2.19E-06 |
| ebi-a-GCST90002068 | rs12185614 | 2 | 26464408 | G | 0.9527 | -0.3031 | 0.06592 | 4.45E-06 |
| ebi-a-GCST90002093 | rs12186328 | 4 | 154428112 | G | 0.2034 | -0.2334 | 0.04621 | 4.97E-07 |
| ebi-a-GCST90001698 | rs12192395 | 6 | 70792408 | G | 0.0116 | 82.52 | 17.15 | 1.56E-06 |
| ebi-a-GCST90001777 | rs12192925 | 6 | 37488754 | T | 0.5633 | 0.1224 | 0.02505 | 1.07E-06 |
| ebi-a-GCST90001982 | rs12193797 | 6 | 41748352 | G | 0.1781 | 0.1624 | 0.03246 | 5.96E-07 |
| ebi-a-GCST90001592 | rs12195711 | 6 | 50386986 | T | 0.1314 | 0.1703 | 0.0353 | 1.46E-06 |
| ebi-a-GCST90001745 | rs12196019 | 6 | 31005483 | A | 0.1117 | 0.2387 | 0.04602 | 2.27E-07 |
| ebi-a-GCST90002007 | rs12196486 | 6 | 3041467 | T | 3.00E-04 | -3.028 | 0.6226 | 1.20E-06 |
| ebi-a-GCST90001400 | rs12197153 | 6 | 100821460 | A | 7.00E-04 | 2.08 | 0.4491 | 3.74E-06 |
| ebi-a-GCST90001416 | rs12197153 | 6 | 100821460 | A | 7.00E-04 | 2.118 | 0.4501 | 2.63E-06 |
| ebi-a-GCST90001394 | rs12197153 | 6 | 100821460 | A | 7.00E-04 | 2.216 | 0.4431 | 5.96E-07 |
| ebi-a-GCST90001431 | rs12197754 | 6 | 130521070 | C | 0.2014 | -0.1427 | 0.03115 | 4.75E-06 |
| ebi-a-GCST90002036 | rs12199079 | 6 | 90935383 | G | 0.4422 | 0.2265 | 0.02793 | 7.54E-16 |
| ebi-a-GCST90001836 | rs12200259 | 6 | 12516242 | G | 0.1638 | 0.2541 | 0.05102 | 7.09E-07 |
| ebi-a-GCST90001765 | rs12200293 | 6 | 101259222 | A | 0.0462 | -0.306 | 0.05889 | 2.14E-07 |
| ebi-a-GCST90001771 | rs12200293 | 6 | 101259222 | A | 0.0463 | -0.2916 | 0.05906 | 8.29E-07 |
| ebi-a-GCST90002061 | rs12200389 | 6 | 38122097 | G | 0.0712 | -0.2507 | 0.05385 | 3.37E-06 |
| ebi-a-GCST90001755 | rs12200643 | 6 | 170846603 | A | 0.0083 | -0.7166 | 0.1417 | 4.48E-07 |
| ebi-a-GCST90001761 | rs12200643 | 6 | 170846603 | A | 0.0083 | -0.6608 | 0.1424 | 3.59E-06 |
| ebi-a-GCST90001663 | rs12204116 | 6 | 91469768 | C | 0.1389 | -0.1732 | 0.03742 | 3.82E-06 |
| ebi-a-GCST90002024 | rs12204644 | 6 | 91794933 | A | 0.0299 | 0.4135 | 0.0818 | 4.57E-07 |
| ebi-a-GCST90002093 | rs12204943 | 6 | 12516245 | A | 0.1638 | 0.2429 | 0.05032 | 1.53E-06 |
| ebi-a-GCST90001774 | rs12206866 | 6 | 107159822 | T | 0.0559 | -0.4459 | 0.05365 | 1.32E-16 |
| ebi-a-GCST90001743 | rs12212931 | 6 | 15913304 | G | 0.8197 | 0.2121 | 0.03182 | 3.02E-11 |
| ebi-a-GCST90001762 | rs12212931 | 6 | 15913304 | G | 0.8197 | 0.2233 | 0.03173 | 2.33E-12 |
| ebi-a-GCST90001396 | rs12216018 | 6 | 130514590 | T | 0.1415 | -0.1686 | 0.03603 | 2.99E-06 |
| ebi-a-GCST90001730 | rs12216684 | 7 | 56291533 | A | 0.0543 | 0.2624 | 0.05367 | 1.06E-06 |
| ebi-a-GCST90001729 | rs12216684 | 7 | 56291533 | A | 0.0543 | 0.2629 | 0.05427 | 1.32E-06 |
| ebi-a-GCST90001742 | rs12216684 | 7 | 56291533 | A | 0.0543 | 0.2634 | 0.05419 | 1.23E-06 |
| ebi-a-GCST90001725 | rs12216684 | 7 | 56291533 | A | 0.0543 | 0.2641 | 0.05347 | 8.15E-07 |
| ebi-a-GCST90001737 | rs12216684 | 7 | 56291533 | A | 0.0543 | 0.2683 | 0.05345 | 5.39E-07 |
| ebi-a-GCST90001741 | rs12216684 | 7 | 56291533 | A | 0.0544 | 0.2695 | 0.05355 | 5.06E-07 |
| ebi-a-GCST90001782 | rs12218872 | 10 | 6177617 | C | 0.5936 | -0.1829 | 0.0338 | 7.04E-08 |
| ebi-a-GCST90002065 | rs12223055 | 11 | 121778965 | C | 0.3249 | 0.1371 | 0.02875 | 1.97E-06 |
| ebi-a-GCST90001632 | rs12224032 | 11 | 112914680 | G | 0.2087 | 0.14 | 0.03055 | 4.76E-06 |
| ebi-a-GCST90001631 | rs12224032 | 11 | 112914680 | G | 0.2087 | 0.142 | 0.03037 | 3.02E-06 |
| ebi-a-GCST90001630 | rs12224032 | 11 | 112914680 | G | 0.2088 | 0.1477 | 0.03079 | 1.69E-06 |
| ebi-a-GCST90001623 | rs12224032 | 11 | 112914680 | G | 0.2086 | 0.1669 | 0.03115 | 8.91E-08 |
| ebi-a-GCST90001622 | rs12224032 | 11 | 112914680 | G | 0.2086 | 0.1713 | 0.03097 | 3.41E-08 |
| ebi-a-GCST90001621 | rs12224032 | 11 | 112914680 | G | 0.2087 | 0.1721 | 0.03092 | 2.78E-08 |
| ebi-a-GCST90002029 | rs12237839 | 9 | 135281367 | C | 0.0803 | -0.2383 | 0.04913 | 1.30E-06 |
| ebi-a-GCST90002037 | rs12239268 | 1 | 202051197 | A | 0.1292 | -0.2001 | 0.04166 | 1.64E-06 |
| ebi-a-GCST90002038 | rs12239268 | 1 | 202051197 | A | 0.1292 | -0.1987 | 0.04166 | 1.95E-06 |
| ebi-a-GCST90001979 | rs12239946 | 1 | 160319055 | C | 0.0827 | 0.2423 | 0.04555 | 1.10E-07 |
| ebi-a-GCST90001936 | rs12244238 | 10 | 6083239 | G | 0.3854 | 0.1324 | 0.02577 | 2.95E-07 |
| ebi-a-GCST90002000 | rs12256905 | 10 | 32700539 | C | 0.1551 | -0.157 | 0.03358 | 3.07E-06 |
| ebi-a-GCST90001491 | rs12268044 | 10 | 98794615 | T | 0.3439 | -0.1886 | 0.02672 | 2.05E-12 |
| ebi-a-GCST90002032 | rs12269710 | 10 | 100218017 | A | 0.0228 | -0.4727 | 0.09468 | 6.32E-07 |
| ebi-a-GCST90001522 | rs12276131 | 11 | 4839164 | A | 0.5081 | -0.1585 | 0.03347 | 2.35E-06 |
| ebi-a-GCST90002074 | rs12276856 | 11 | 122628500 | T | 0.2542 | 0.1441 | 0.03031 | 2.09E-06 |
| ebi-a-GCST90001456 | rs12277397 | 11 | 134569102 | T | 0.1179 | 0.1863 | 0.04033 | 4.00E-06 |
| ebi-a-GCST90001700 | rs12279408 | 11 | 84269972 | T | 7.00E-04 | 16 | 2.92 | 4.60E-08 |
| ebi-a-GCST90001695 | rs12279408 | 11 | 84269972 | T | 7.00E-04 | 259.3 | 51.36 | 4.70E-07 |
| ebi-a-GCST90001698 | rs12279408 | 11 | 84269972 | T | 7.00E-04 | 699.1 | 69.55 | 1.90E-23 |
| ebi-a-GCST90001871 | rs12284339 | 11 | 107699559 | A | 0.0048 | -1.363 | 0.2918 | 3.30E-06 |
| ebi-a-GCST90002078 | rs12286701 | 11 | 31863361 | C | 0.0575 | 0.2694 | 0.05633 | 1.82E-06 |
| ebi-a-GCST90001517 | rs12297491 | 12 | 1731406 | A | 0.1671 | 0.2117 | 0.0447 | 2.35E-06 |
| ebi-a-GCST90001426 | rs12298227 | 12 | 54786414 | C | 0.1013 | -0.2055 | 0.04122 | 6.51E-07 |
| ebi-a-GCST90001467 | rs12311260 | 12 | 18094151 | G | 0.0501 | 0.2649 | 0.0576 | 4.38E-06 |
| ebi-a-GCST90001701 | rs12311737 | 12 | 131958370 | G | 0.1807 | 0.1489 | 0.03198 | 3.34E-06 |
| ebi-a-GCST90001688 | rs12314907 | 12 | 79661771 | G | NA | 6.244 | 1.145 | 5.33E-08 |
| ebi-a-GCST90001666 | rs12314907 | 12 | 79661771 | G | NA | 8.184 | 1.461 | 2.27E-08 |
| ebi-a-GCST90001664 | rs12314907 | 12 | 79661771 | G | NA | 8.253 | 1.525 | 6.72E-08 |
| ebi-a-GCST90001692 | rs12317609 | 12 | 23030395 | G | 0.0215 | -0.4201 | 0.08689 | 1.39E-06 |
| ebi-a-GCST90001506 | rs12325554 | 16 | 84053417 | C | 0.0147 | 0.4872 | 0.1049 | 3.51E-06 |
| ebi-a-GCST90001698 | rs12332976 | 6 | 40619493 | A | 0.0057 | 169.6 | 23.32 | 4.38E-13 |
| ebi-a-GCST90001921 | rs12335987 | 9 | 13235045 | C | 0.0462 | -0.2974 | 0.06197 | 1.66E-06 |
| ebi-a-GCST90001948 | rs12337816 | 9 | 117125236 | C | 0.1589 | 0.236 | 0.04963 | 2.17E-06 |
| ebi-a-GCST90001425 | rs12340211 | 9 | 8531036 | G | 0.0518 | 0.2558 | 0.05361 | 1.91E-06 |
| ebi-a-GCST90001783 | rs12341626 | 9 | 17383792 | C | 0.1539 | -0.1597 | 0.03463 | 4.10E-06 |
| ebi-a-GCST90001787 | rs12342539 | 9 | 4327960 | T | 0.2216 | -0.1657 | 0.02956 | 2.25E-08 |
| ebi-a-GCST90001793 | rs12342539 | 9 | 4327960 | T | 0.2216 | -0.1457 | 0.02959 | 8.91E-07 |
| ebi-a-GCST90002093 | rs12345519 | 9 | 134450295 | A | 0.0113 | 0.8087 | 0.175 | 4.18E-06 |
| ebi-a-GCST90001583 | rs12346499 | 9 | 113902881 | T | 0.5233 | -0.1378 | 0.0244 | 1.77E-08 |
| ebi-a-GCST90001733 | rs12346584 | 9 | 97103437 | C | 0.2743 | 0.136 | 0.02791 | 1.15E-06 |
| ebi-a-GCST90002118 | rs1235080 | 1 | 192838446 | A | 0.4182 | 0.1343 | 0.02796 | 1.64E-06 |
| ebi-a-GCST90001557 | rs12352819 | 9 | 133271663 | T | 0.0277 | -0.3715 | 0.07868 | 2.42E-06 |
| ebi-a-GCST90001578 | rs12360242 | 10 | 132652504 | T | 0.0946 | -0.1966 | 0.04253 | 3.90E-06 |
| ebi-a-GCST90001661 | rs12360943 | 11 | 81821948 | C | 0.3923 | -0.1262 | 0.02644 | 1.89E-06 |
| ebi-a-GCST90001909 | rs12364299 | 11 | 5403108 | T | 0.0782 | -0.2989 | 0.05053 | 3.68E-09 |
| ebi-a-GCST90001967 | rs12364299 | 11 | 5403108 | T | 0.0782 | -0.2594 | 0.05098 | 3.82E-07 |
| ebi-a-GCST90001913 | rs12364299 | 11 | 5403108 | T | 0.0782 | -0.2407 | 0.05061 | 2.06E-06 |
| ebi-a-GCST90001999 | rs12367061 | 12 | 119294464 | A | 0.0163 | -0.4537 | 0.09811 | 3.89E-06 |
| ebi-a-GCST90001922 | rs12370077 | 12 | 55201765 | A | 0.5697 | 0.128 | 0.02728 | 2.82E-06 |
| ebi-a-GCST90002074 | rs12370645 | 12 | 19824999 | G | 0.0079 | -0.6901 | 0.1507 | 4.82E-06 |
| ebi-a-GCST90001734 | rs12374328 | 4 | 61352640 | A | 0.0685 | -0.2445 | 0.05123 | 1.89E-06 |
| ebi-a-GCST90001879 | rs12378405 | 9 | 7820315 | A | 0.7703 | -0.2229 | 0.04832 | 4.37E-06 |
| ebi-a-GCST90001698 | rs12378893 | 9 | 79057643 | A | 0.0025 | 245.4 | 36.99 | 3.79E-11 |
| ebi-a-GCST90001721 | rs1239945 | 13 | 51107757 | A | 0.5341 | 0.1129 | 0.02439 | 3.80E-06 |
| ebi-a-GCST90001853 | rs12401936 | 1 | 163328756 | G | 0.1554 | 0.1738 | 0.03767 | 4.13E-06 |
| ebi-a-GCST90001931 | rs12402082 | 1 | 231966624 | T | 0.0656 | 0.2591 | 0.05623 | 4.23E-06 |
| ebi-a-GCST90001579 | rs12404245 | 1 | 77952299 | C | 0.0303 | 0.3244 | 0.07033 | 4.12E-06 |
| ebi-a-GCST90002067 | rs12404655 | 1 | 20943195 | G | 0.244 | -0.1494 | 0.03167 | 2.50E-06 |
| ebi-a-GCST90002059 | rs12408387 | 1 | 119528329 | A | 0.13 | 0.1953 | 0.03958 | 8.53E-07 |
| ebi-a-GCST90001538 | rs12409155 | 1 | 110998348 | T | 0.1058 | -0.2079 | 0.04135 | 5.22E-07 |
| ebi-a-GCST90001539 | rs12409155 | 1 | 110998348 | T | 0.1058 | -0.1924 | 0.042 | 4.79E-06 |
| ebi-a-GCST90001435 | rs12412378 | 10 | 130483163 | A | 0.1608 | -0.1755 | 0.03276 | 9.04E-08 |
| ebi-a-GCST90001404 | rs12412378 | 10 | 130483163 | A | 0.1607 | -0.1669 | 0.0328 | 3.78E-07 |
| ebi-a-GCST90001405 | rs12412378 | 10 | 130483163 | A | 0.1608 | -0.16 | 0.03222 | 7.19E-07 |
| ebi-a-GCST90002051 | rs12415341 | 10 | 117921247 | A | 0.1793 | -0.2283 | 0.04861 | 2.87E-06 |
| ebi-a-GCST90001601 | rs12417637 | 11 | 128303735 | G | 0.1068 | 0.1866 | 0.04033 | 3.85E-06 |
| ebi-a-GCST90001551 | rs12420840 | 11 | 126523412 | G | 0.0931 | 0.1387 | 0.02921 | 2.13E-06 |
| ebi-a-GCST90002089 | rs12421743 | 11 | 124765894 | G | 0.1626 | 0.1721 | 0.03736 | 4.30E-06 |
| ebi-a-GCST90001643 | rs12422149 | 11 | 74883577 | A | 0.1086 | -0.183 | 0.03848 | 2.05E-06 |
| ebi-a-GCST90001664 | rs12424807 | 12 | 14397702 | C | 0.0875 | 0.1895 | 0.04112 | 4.20E-06 |
| ebi-a-GCST90001969 | rs12425743 | 12 | 86546401 | G | 0.0055 | 0.8813 | 0.1825 | 1.43E-06 |
| ebi-a-GCST90001434 | rs12426629 | 12 | 54797571 | G | 0.0989 | -0.2182 | 0.04316 | 4.52E-07 |
| ebi-a-GCST90001427 | rs12426629 | 12 | 54797571 | G | 0.0989 | -0.1952 | 0.04268 | 4.95E-06 |
| ebi-a-GCST90001816 | rs12428439 | 13 | 59808190 | G | 0.407 | 0.1203 | 0.02527 | 2.01E-06 |
| ebi-a-GCST90001443 | rs12432015 | 14 | 49052782 | C | 0.118 | -0.1712 | 0.0373 | 4.57E-06 |
| ebi-a-GCST90001973 | rs12434214 | 14 | 98114570 | C | 0.0846 | 0.2076 | 0.04523 | 4.60E-06 |
| ebi-a-GCST90001818 | rs12435334 | 14 | 38169407 | A | 0.443 | 0.1246 | 0.02554 | 1.10E-06 |
| ebi-a-GCST90001475 | rs12436392 | 14 | 81293101 | C | 0.9897 | 0.5761 | 0.123 | 2.94E-06 |
| ebi-a-GCST90001824 | rs12436535 | 14 | 106407257 | A | 0.1358 | 0.2501 | 0.04699 | 1.09E-07 |
| ebi-a-GCST90001722 | rs12438058 | 15 | 39819219 | C | 0.1439 | 0.1624 | 0.03416 | 2.06E-06 |
| ebi-a-GCST90001698 | rs12442442 | 15 | 40127028 | A | 0.0041 | 144.4 | 28.08 | 2.86E-07 |
| ebi-a-GCST90001981 | rs12443555 | 16 | 81615369 | A | 0.0023 | 1.31 | 0.2617 | 5.79E-07 |
| ebi-a-GCST90001536 | rs12448994 | 16 | 989381 | T | 0.0925 | 0.1905 | 0.04089 | 3.28E-06 |
| ebi-a-GCST90002113 | rs12449199 | 16 | 10968440 | G | 0.3064 | -0.2433 | 0.02779 | 3.30E-18 |
| ebi-a-GCST90002114 | rs12449199 | 16 | 10968440 | G | 0.3064 | -0.1674 | 0.02775 | 1.83E-09 |
| ebi-a-GCST90001700 | rs12450105 | 17 | 54427514 | C | 0.0031 | 6.576 | 1.424 | 4.02E-06 |
| ebi-a-GCST90001686 | rs12450916 | 17 | 74615933 | T | 0.5433 | 0.108 | 0.02273 | 2.11E-06 |
| ebi-a-GCST90001673 | rs12450916 | 17 | 74615933 | T | 0.5416 | 0.1189 | 0.0253 | 2.71E-06 |
| ebi-a-GCST90001675 | rs12450916 | 17 | 74615933 | T | 0.5425 | 0.1237 | 0.02531 | 1.07E-06 |
| ebi-a-GCST90002096 | rs12451379 | 17 | 70268997 | A | 0.1317 | -0.2732 | 0.05433 | 5.50E-07 |
| ebi-a-GCST90001727 | rs12451409 | 17 | 45544856 | G | 0.5544 | -0.1632 | 0.03336 | 1.09E-06 |
| ebi-a-GCST90001539 | rs12453732 | 17 | 38177839 | C | 0.3439 | -0.1373 | 0.02865 | 1.71E-06 |
| ebi-a-GCST90001654 | rs12453786 | 17 | 33272507 | G | 0.2017 | -0.1497 | 0.03162 | 2.30E-06 |
| ebi-a-GCST90001512 | rs12458491 | 18 | 35307820 | T | 0.0188 | 0.4275 | 0.09269 | 4.13E-06 |
| ebi-a-GCST90001505 | rs12458491 | 18 | 35307820 | T | 0.0188 | 0.4545 | 0.09235 | 9.02E-07 |
| ebi-a-GCST90001511 | rs12458491 | 18 | 35307820 | T | 0.0188 | 0.5009 | 0.09263 | 6.83E-08 |
| ebi-a-GCST90002044 | rs12459419 | 19 | 51728477 | T | 0.211 | -0.2232 | 0.04379 | 3.85E-07 |
| ebi-a-GCST90001950 | rs12459503 | 19 | 52161035 | C | 0.7178 | 0.2252 | 0.04583 | 1.01E-06 |
| ebi-a-GCST90001951 | rs12459503 | 19 | 52161035 | C | 0.7148 | 0.2704 | 0.04205 | 1.74E-10 |
| ebi-a-GCST90002078 | rs12459815 | 19 | 52142313 | A | 0.4452 | 0.125 | 0.02707 | 4.06E-06 |
| ebi-a-GCST90001400 | rs12461001 | 19 | 14885525 | A | 0.6292 | 0.1175 | 0.02527 | 3.43E-06 |
| ebi-a-GCST90001398 | rs12461001 | 19 | 14885525 | A | 0.6291 | 0.1184 | 0.02528 | 2.95E-06 |
| ebi-a-GCST90001695 | rs12464465 | 2 | 176542288 | A | 0.0015 | 189.7 | 34.49 | 4.09E-08 |
| ebi-a-GCST90001749 | rs12470912 | 2 | 168748110 | A | 0.1598 | 0.2166 | 0.04663 | 3.65E-06 |
| ebi-a-GCST90002062 | rs12471945 | 2 | 19654868 | T | 0.0134 | 0.5682 | 0.1213 | 2.94E-06 |
| ebi-a-GCST90001500 | rs1247730 | 11 | 79922768 | T | 0.2232 | 0.1384 | 0.02948 | 2.79E-06 |
| ebi-a-GCST90002056 | rs12478003 | 2 | 47490742 | A | 0.1741 | 0.1702 | 0.03679 | 3.90E-06 |
| ebi-a-GCST90002074 | rs12478601 | 2 | 43721508 | T | 0.4735 | -0.1442 | 0.02805 | 2.90E-07 |
| ebi-a-GCST90001757 | rs12480920 | 20 | 3862580 | A | 0.1792 | -0.1577 | 0.032 | 8.74E-07 |
| ebi-a-GCST90001977 | rs12482528 | 21 | 15710293 | T | 0.1013 | -0.226 | 0.0427 | 1.29E-07 |
| ebi-a-GCST90002085 | rs12482528 | 21 | 15710293 | T | 0.1013 | -0.2044 | 0.0433 | 2.45E-06 |
| ebi-a-GCST90001455 | rs12485444 | 3 | 188135783 | A | 0.4742 | 0.1287 | 0.02682 | 1.67E-06 |
| ebi-a-GCST90002111 | rs12485444 | 3 | 188135783 | A | 0.4725 | 0.1803 | 0.03549 | 4.18E-07 |
| ebi-a-GCST90002110 | rs12485444 | 3 | 188135783 | A | 0.4728 | 0.2278 | 0.03577 | 2.49E-10 |
| ebi-a-GCST90001966 | rs12486053 | 3 | 186182741 | G | 0.0454 | -0.3206 | 0.06773 | 2.32E-06 |
| ebi-a-GCST90001410 | rs12493961 | 3 | 11244825 | G | 0.0219 | 0.3884 | 0.08136 | 1.88E-06 |
| ebi-a-GCST90001808 | rs12495781 | 3 | 127619256 | C | 0.4044 | -0.1176 | 0.02554 | 4.30E-06 |
| ebi-a-GCST90001837 | rs12496184 | 3 | 32299237 | C | 0.6133 | -0.1939 | 0.04095 | 2.45E-06 |
| ebi-a-GCST90001841 | rs12497487 | 3 | 78194307 | T | 0.7615 | -0.1669 | 0.03224 | 2.40E-07 |
| ebi-a-GCST90001536 | rs12498745 | 4 | 99216768 | A | 6.00E-04 | -2.198 | 0.4387 | 5.73E-07 |
| ebi-a-GCST90001700 | rs12499962 | 4 | 99029457 | G | 6.00E-04 | 25.15 | 3.141 | 1.59E-15 |
| ebi-a-GCST90001698 | rs12499962 | 4 | 99029457 | G | 6.00E-04 | 1129 | 72.17 | 2.50E-53 |
| ebi-a-GCST90001583 | rs12501003 | 4 | 92510918 | G | 0.1674 | 0.1594 | 0.03191 | 6.17E-07 |
| ebi-a-GCST90001582 | rs12501003 | 4 | 92510918 | G | 0.1674 | 0.1613 | 0.03189 | 4.45E-07 |
| ebi-a-GCST90001700 | rs12501785 | 4 | 180565400 | A | 0.0013 | 10.8 | 1.881 | 1.00E-08 |
| ebi-a-GCST90001698 | rs12501785 | 4 | 180565400 | A | 0.0013 | 620.5 | 43.09 | 1.11E-45 |
| ebi-a-GCST90001842 | rs12502302 | 4 | 186735780 | T | 0.4043 | 0.1301 | 0.02774 | 2.86E-06 |
| ebi-a-GCST90001856 | rs12502302 | 4 | 186735780 | T | 0.4048 | 0.1306 | 0.02783 | 2.81E-06 |
| ebi-a-GCST90001538 | rs12503846 | 4 | 181498148 | T | 0.1048 | -0.2065 | 0.04115 | 5.52E-07 |
| ebi-a-GCST90001820 | rs12505403 | 4 | 130367811 | A | 0.1156 | 0.212 | 0.03944 | 8.11E-08 |
| ebi-a-GCST90002040 | rs12507081 | 4 | 153889606 | G | 0.8365 | -0.172 | 0.0374 | 4.44E-06 |
| ebi-a-GCST90001924 | rs12513323 | 4 | 185517125 | A | 0.0692 | -0.2663 | 0.05352 | 6.85E-07 |
| ebi-a-GCST90001430 | rs12516105 | 5 | 160198880 | C | 0.1737 | 0.1502 | 0.03243 | 3.75E-06 |
| ebi-a-GCST90001981 | rs12517933 | 5 | 173665451 | C | 0.2168 | -0.1387 | 0.03026 | 4.72E-06 |
| ebi-a-GCST90001485 | rs12518588 | 5 | 85325148 | G | 0.412 | 0.1241 | 0.02523 | 9.15E-07 |
| ebi-a-GCST90001567 | rs1252165 | 1 | 237168044 | T | 0.0035 | 0.8619 | 0.185 | 3.31E-06 |
| ebi-a-GCST90002046 | rs12523072 | 5 | 104197397 | T | 0.1073 | 0.2734 | 0.05833 | 3.01E-06 |
| ebi-a-GCST90001970 | rs12524004 | 6 | 144027987 | C | 0.0236 | -0.4128 | 0.08943 | 4.08E-06 |
| ebi-a-GCST90002077 | rs12524004 | 6 | 144027987 | C | 0.0236 | -0.4118 | 0.08893 | 3.80E-06 |
| ebi-a-GCST90001999 | rs12524781 | 6 | 154891396 | A | 0.106 | -0.1894 | 0.04022 | 2.57E-06 |
| ebi-a-GCST90001785 | rs12529059 | 6 | 64695023 | T | 0.2297 | 0.1341 | 0.02909 | 4.15E-06 |
| ebi-a-GCST90002088 | rs12529747 | 6 | 112955454 | C | 0.2303 | -0.1451 | 0.03167 | 4.80E-06 |
| ebi-a-GCST90001577 | rs12535189 | 7 | 121174063 | T | 0.4795 | 0.1135 | 0.02446 | 3.59E-06 |
| ebi-a-GCST90001567 | rs12537183 | 7 | 153543926 | C | 0.2221 | -0.1284 | 0.02733 | 2.73E-06 |
| ebi-a-GCST90001568 | rs12537183 | 7 | 153543926 | C | 0.2221 | -0.1193 | 0.02479 | 1.55E-06 |
| ebi-a-GCST90002120 | rs12537451 | 7 | 49270809 | T | 0.0966 | -0.2163 | 0.04538 | 1.98E-06 |
| ebi-a-GCST90001397 | rs12539793 | 7 | 25971860 | T | 0.0115 | -0.5404 | 0.1145 | 2.47E-06 |
| ebi-a-GCST90001800 | rs12541523 | 8 | 124577287 | A | 0.9264 | 0.2187 | 0.04697 | 3.34E-06 |
| ebi-a-GCST90001441 | rs12542190 | 8 | 130594761 | C | 0.1943 | -0.1572 | 0.03051 | 2.72E-07 |
| ebi-a-GCST90001969 | rs12542554 | 8 | 5579734 | T | 0.1031 | 0.2057 | 0.04354 | 2.40E-06 |
| ebi-a-GCST90001803 | rs12552851 | 9 | 24488925 | C | 0.1019 | 0.1946 | 0.04123 | 2.43E-06 |
| ebi-a-GCST90002033 | rs12553288 | 9 | 105037632 | T | 0.0539 | 0.2833 | 0.06107 | 3.65E-06 |
| ebi-a-GCST90001748 | rs12554596 | 9 | 37002142 | G | 0.3892 | -0.1558 | 0.02531 | 8.43E-10 |
| ebi-a-GCST90001760 | rs12554596 | 9 | 37002142 | G | 0.3891 | -0.1329 | 0.02547 | 1.92E-07 |
| ebi-a-GCST90001726 | rs12554596 | 9 | 37002142 | G | 0.3892 | -0.1325 | 0.02534 | 1.82E-07 |
| ebi-a-GCST90001822 | rs12554596 | 9 | 37002142 | G | 0.3895 | -0.1292 | 0.02586 | 6.18E-07 |
| ebi-a-GCST90001738 | rs12554596 | 9 | 37002142 | G | 0.3892 | -0.1201 | 0.02547 | 2.50E-06 |
| ebi-a-GCST90001745 | rs12554596 | 9 | 37002142 | G | 0.3891 | -0.1172 | 0.02548 | 4.39E-06 |
| ebi-a-GCST90001758 | rs12554596 | 9 | 37002142 | G | 0.3891 | -0.1168 | 0.02544 | 4.60E-06 |
| ebi-a-GCST90001723 | rs12554596 | 9 | 37002142 | G | 0.3892 | -0.1166 | 0.02549 | 4.96E-06 |
| ebi-a-GCST90002048 | rs12555208 | 9 | 130531142 | T | 0.044 | -0.415 | 0.08894 | 3.35E-06 |
| ebi-a-GCST90001551 | rs12555210 | 9 | 33428882 | G | 0.0887 | 0.1409 | 0.03006 | 2.90E-06 |
| ebi-a-GCST90001688 | rs12555210 | 9 | 33428882 | G | 0.089 | 0.1465 | 0.03099 | 2.38E-06 |
| ebi-a-GCST90001553 | rs12555210 | 9 | 33428882 | G | 0.089 | 0.1558 | 0.0299 | 1.98E-07 |
| ebi-a-GCST90001865 | rs12570957 | 10 | 101981274 | A | 0.2681 | 0.1445 | 0.03082 | 2.88E-06 |
| ebi-a-GCST90001645 | rs1257331 | 14 | 99784255 | T | 0.3785 | 0.1177 | 0.02464 | 1.83E-06 |
| ebi-a-GCST90001690 | rs12575977 | 11 | 126507219 | T | 0.1357 | 0.1204 | 0.0258 | 3.17E-06 |
| ebi-a-GCST90001885 | rs12580221 | 12 | 125621066 | C | 0.3036 | -0.1424 | 0.02904 | 9.92E-07 |
| ebi-a-GCST90002040 | rs12585407 | 13 | 23667413 | C | 0.4444 | 0.1373 | 0.02786 | 8.78E-07 |
| ebi-a-GCST90001658 | rs12587599 | 14 | 104575130 | T | 0.1042 | -0.191 | 0.03906 | 1.05E-06 |
| ebi-a-GCST90001659 | rs12587599 | 14 | 104575130 | T | 0.1042 | -0.1824 | 0.03908 | 3.17E-06 |
| ebi-a-GCST90001836 | rs12587697 | 14 | 20470737 | A | 0.1853 | 0.2354 | 0.04781 | 9.44E-07 |
| ebi-a-GCST90001983 | rs12593041 | 15 | 52443107 | T | 0.1238 | 0.1733 | 0.03657 | 2.23E-06 |
| ebi-a-GCST90001911 | rs12593800 | 15 | 79575521 | C | 0.4162 | -0.1265 | 0.02713 | 3.22E-06 |
| ebi-a-GCST90001700 | rs12596264 | 16 | 1058881 | A | 0.0025 | 7.351 | 1.534 | 1.71E-06 |
| ebi-a-GCST90001849 | rs12596540 | 16 | 10970476 | G | 0.2521 | 0.1435 | 0.03065 | 2.98E-06 |
| ebi-a-GCST90001847 | rs12596540 | 16 | 10970476 | G | 0.2521 | 0.2057 | 0.03039 | 1.56E-11 |
| ebi-a-GCST90002117 | rs12598451 | 16 | 10982607 | A | 0.2279 | -0.378 | 0.03133 | 9.38E-33 |
| ebi-a-GCST90001674 | rs12599120 | 16 | 25477950 | C | 0.6097 | 0.1203 | 0.02627 | 4.88E-06 |
| ebi-a-GCST90002032 | rs12603021 | 17 | 3202903 | T | 0.108 | 0.2077 | 0.04422 | 2.76E-06 |
| ebi-a-GCST90002105 | rs12607423 | 18 | 67084661 | T | 0.1898 | -0.1621 | 0.03448 | 2.71E-06 |
| ebi-a-GCST90001540 | rs12607959 | 18 | 65226341 | G | 0.0337 | -0.3213 | 0.06601 | 1.18E-06 |
| ebi-a-GCST90002035 | rs12609290 | 19 | 17910564 | A | 0.0277 | 0.3923 | 0.08387 | 3.04E-06 |
| ebi-a-GCST90001826 | rs12609856 | 19 | 11807010 | A | 0.481 | 0.1162 | 0.02519 | 4.12E-06 |
| ebi-a-GCST90001626 | rs12609975 | 19 | 39400504 | G | 0.7039 | -0.1153 | 0.02408 | 1.75E-06 |
| ebi-a-GCST90001625 | rs12609975 | 19 | 39400504 | G | 0.7039 | -0.1105 | 0.02341 | 2.43E-06 |
| ebi-a-GCST90002008 | rs12613485 | 2 | 102154764 | A | 0.0845 | -0.2065 | 0.04436 | 3.36E-06 |
| ebi-a-GCST90002045 | rs12614599 | 2 | 232290640 | A | 0.3428 | 0.2608 | 0.03665 | 1.68E-12 |
| ebi-a-GCST90001698 | rs12616335 | 2 | 159969605 | T | 0.0012 | 330.2 | 60.13 | 4.27E-08 |
| ebi-a-GCST90001460 | rs12616455 | 2 | 85696551 | A | 0.7121 | 0.1321 | 0.02633 | 5.57E-07 |
| ebi-a-GCST90001639 | rs12616821 | 2 | 99282659 | C | 0.4655 | 0.1153 | 0.02515 | 4.72E-06 |
| ebi-a-GCST90001651 | rs12618511 | 2 | 1874340 | C | 0.0594 | -0.2653 | 0.05281 | 5.34E-07 |
| ebi-a-GCST90001870 | rs12621242 | 2 | 23022235 | A | 0.0141 | -0.7206 | 0.1516 | 2.19E-06 |
| ebi-a-GCST90001804 | rs12625834 | 20 | 44949039 | A | 0.213 | -0.1592 | 0.03012 | 1.32E-07 |
| ebi-a-GCST90001800 | rs12625834 | 20 | 44949039 | A | 0.2131 | -0.1534 | 0.03074 | 6.37E-07 |
| ebi-a-GCST90001486 | rs12626270 | 21 | 34223997 | T | 0.1232 | 0.1817 | 0.03912 | 3.55E-06 |
| ebi-a-GCST90001618 | rs12627305 | 21 | 21249973 | A | 0.0225 | 0.3836 | 0.07904 | 1.27E-06 |
| ebi-a-GCST90001701 | rs12630507 | 3 | 141650463 | A | 0.0068 | -0.699 | 0.147 | 2.07E-06 |
| ebi-a-GCST90001923 | rs12634099 | 3 | 112869208 | A | 0.2255 | 0.1548 | 0.03293 | 2.70E-06 |
| ebi-a-GCST90001931 | rs12634099 | 3 | 112869208 | A | 0.2255 | 0.1569 | 0.03309 | 2.21E-06 |
| ebi-a-GCST90002080 | rs12635110 | 3 | 129503109 | A | 0.8365 | 0.1743 | 0.03514 | 7.43E-07 |
| ebi-a-GCST90001410 | rs12636371 | 3 | 125894785 | A | 0.4619 | 0.1185 | 0.02414 | 9.61E-07 |
| ebi-a-GCST90001442 | rs12636848 | 3 | 147613080 | G | 0.3031 | 0.1237 | 0.02646 | 3.03E-06 |
| ebi-a-GCST90001436 | rs12636848 | 3 | 147613080 | G | 0.3031 | 0.1242 | 0.02655 | 2.98E-06 |
| ebi-a-GCST90001779 | rs12638268 | 3 | 11933438 | C | 0.3426 | 0.1202 | 0.02592 | 3.66E-06 |
| ebi-a-GCST90001794 | rs12638268 | 3 | 11933438 | C | 0.3425 | 0.1222 | 0.026 | 2.69E-06 |
| ebi-a-GCST90002035 | rs12640679 | 4 | 155764038 | C | 0.3927 | 0.1342 | 0.02856 | 2.74E-06 |
| ebi-a-GCST90001770 | rs12642193 | 4 | 25916457 | G | 0.1576 | -0.168 | 0.0337 | 6.47E-07 |
| ebi-a-GCST90001773 | rs12642193 | 4 | 25916457 | G | 0.1574 | -0.155 | 0.03388 | 4.96E-06 |
| ebi-a-GCST90001862 | rs1264457 | 6 | 30458064 | A | 0.4532 | 0.1413 | 0.02929 | 1.48E-06 |
| ebi-a-GCST90001884 | rs12644590 | 4 | 16657392 | G | 0.0265 | 0.3926 | 0.08376 | 2.90E-06 |
| ebi-a-GCST90002119 | rs1264570 | 6 | 30365210 | T | 0.7204 | 0.1984 | 0.03266 | 1.42E-09 |
| ebi-a-GCST90001965 | rs12646158 | 4 | 14345720 | C | 0.1682 | -0.1703 | 0.03691 | 4.11E-06 |
| ebi-a-GCST90001489 | rs12648210 | 4 | 89266460 | G | 0.0145 | -0.5171 | 0.1056 | 1.02E-06 |
| ebi-a-GCST90001491 | rs12648210 | 4 | 89266460 | G | 0.0145 | -0.5101 | 0.1037 | 9.19E-07 |
| ebi-a-GCST90001806 | rs12651722 | 5 | 161994889 | G | 0.2553 | -0.1321 | 0.02839 | 3.35E-06 |
| ebi-a-GCST90001433 | rs12654363 | 5 | 84064142 | T | 0.9301 | -0.2241 | 0.04812 | 3.31E-06 |
| ebi-a-GCST90001521 | rs12655500 | 5 | 163124522 | A | 0.3251 | 0.173 | 0.03528 | 1.03E-06 |
| ebi-a-GCST90002012 | rs12655590 | 5 | 169085561 | T | 0.2341 | -0.1323 | 0.0286 | 3.87E-06 |
| ebi-a-GCST90001697 | rs12656604 | 5 | 72679056 | A | 0.0047 | 5.388 | 0.8953 | 1.95E-09 |
| ebi-a-GCST90001695 | rs12656604 | 5 | 72679056 | A | 0.0047 | 101.8 | 19.19 | 1.19E-07 |
| ebi-a-GCST90001753 | rs12660382 | 6 | 31443323 | T | 0.3149 | 0.1358 | 0.02903 | 3.00E-06 |
| ebi-a-GCST90001748 | rs12660382 | 6 | 31443323 | T | 0.3145 | 0.1463 | 0.02878 | 3.90E-07 |
| ebi-a-GCST90002119 | rs12660712 | 6 | 29734634 | A | 0.1152 | -0.2799 | 0.04891 | 1.15E-08 |
| ebi-a-GCST90001687 | rs1266079 | 6 | 31504774 | G | 0.3842 | -0.163 | 0.03116 | 1.80E-07 |
| ebi-a-GCST90001692 | rs12663276 | 6 | 29906010 | G | 0.0709 | -0.2958 | 0.05842 | 4.35E-07 |
| ebi-a-GCST90002102 | rs12663871 | 6 | 68875765 | G | 0.0014 | -1.555 | 0.331 | 2.73E-06 |
| ebi-a-GCST90001798 | rs12664485 | 6 | 143890569 | C | 0.012 | 0.5228 | 0.1137 | 4.45E-06 |
| ebi-a-GCST90002040 | rs12675298 | 8 | 6828813 | A | 0.0277 | -0.6716 | 0.08441 | 2.54E-15 |
| ebi-a-GCST90001906 | rs12675298 | 8 | 6828813 | A | 0.0277 | -0.5258 | 0.08507 | 7.25E-10 |
| ebi-a-GCST90001926 | rs12675298 | 8 | 6828813 | A | 0.0272 | -0.446 | 0.08519 | 1.77E-07 |
| ebi-a-GCST90001966 | rs12675298 | 8 | 6828813 | A | 0.0277 | -0.4215 | 0.086 | 1.00E-06 |
| ebi-a-GCST90001963 | rs12680377 | 8 | 5739227 | A | 0.0501 | -0.3019 | 0.06216 | 1.26E-06 |
| ebi-a-GCST90001513 | rs1268153 | 6 | 109035696 | A | 0.0053 | 0.8168 | 0.17 | 1.61E-06 |
| ebi-a-GCST90001501 | rs1268153 | 6 | 109035696 | A | 0.0053 | 0.8482 | 0.1738 | 1.11E-06 |
| ebi-a-GCST90001489 | rs12707040 | 7 | 80480551 | A | 0.1783 | 0.1591 | 0.03418 | 3.37E-06 |
| ebi-a-GCST90001452 | rs12708874 | 16 | 66062093 | T | 0.7957 | -0.1526 | 0.03262 | 3.01E-06 |
| ebi-a-GCST90001404 | rs12709039 | 16 | 59861376 | A | 0.7365 | -0.1407 | 0.02784 | 4.57E-07 |
| ebi-a-GCST90001535 | rs12712610 | 2 | 38897249 | G | 0.2969 | -0.1902 | 0.02599 | 3.13E-13 |
| ebi-a-GCST90001494 | rs12712610 | 2 | 38897249 | G | 0.2975 | -0.1818 | 0.02763 | 5.43E-11 |
| ebi-a-GCST90001536 | rs12712610 | 2 | 38897249 | G | 0.2969 | -0.1632 | 0.02646 | 7.82E-10 |
| ebi-a-GCST90001503 | rs12712610 | 2 | 38897249 | G | 0.2975 | -0.1572 | 0.02752 | 1.21E-08 |
| ebi-a-GCST90001575 | rs12712610 | 2 | 38897249 | G | 0.2969 | 0.1236 | 0.02672 | 3.87E-06 |
| ebi-a-GCST90001541 | rs12712610 | 2 | 38897249 | G | 0.2969 | 0.1457 | 0.02548 | 1.18E-08 |
| ebi-a-GCST90001573 | rs12712610 | 2 | 38897249 | G | 0.2969 | 0.1603 | 0.0281 | 1.26E-08 |
| ebi-a-GCST90001665 | rs12712610 | 2 | 38897249 | G | 0.2977 | 0.1611 | 0.02726 | 3.80E-09 |
| ebi-a-GCST90001666 | rs12712610 | 2 | 38897249 | G | 0.2974 | 0.1631 | 0.02442 | 2.82E-11 |
| ebi-a-GCST90001664 | rs12712610 | 2 | 38897249 | G | 0.2977 | 0.1724 | 0.02569 | 2.27E-11 |
| ebi-a-GCST90001545 | rs12712610 | 2 | 38897249 | G | 0.2966 | 0.183 | 0.02834 | 1.22E-10 |
| ebi-a-GCST90001483 | rs12712610 | 2 | 38897249 | G | 0.2972 | 0.1912 | 0.02749 | 4.16E-12 |
| ebi-a-GCST90001485 | rs12712610 | 2 | 38897249 | G | 0.2975 | 0.201 | 0.02719 | 1.83E-13 |
| ebi-a-GCST90001507 | rs12712610 | 2 | 38897249 | G | 0.2972 | 0.2347 | 0.02843 | 2.15E-16 |
| ebi-a-GCST90002100 | rs12712613 | 2 | 38948860 | T | 0.4847 | -0.1917 | 0.02672 | 9.13E-13 |
| ebi-a-GCST90002084 | rs12716653 | 9 | 89886037 | C | 0.6349 | -0.1377 | 0.02831 | 1.20E-06 |
| ebi-a-GCST90001481 | rs12722496 | 10 | 6096667 | G | 0.0611 | -0.2244 | 0.0482 | 3.35E-06 |
| ebi-a-GCST90001787 | rs12722531 | 10 | 6076076 | A | 0.074 | 0.4479 | 0.04714 | 3.61E-21 |
| ebi-a-GCST90001681 | rs12722558 | 10 | 6070276 | T | 0.0674 | 0.2655 | 0.05335 | 6.80E-07 |
| ebi-a-GCST90001937 | rs12722600 | 10 | 6054765 | T | 0.0705 | 0.3212 | 0.04962 | 1.09E-10 |
| ebi-a-GCST90001804 | rs12727962 | 1 | 31686211 | G | 0.0246 | -0.3676 | 0.07711 | 1.94E-06 |
| ebi-a-GCST90001808 | rs12727962 | 1 | 31686211 | G | 0.0246 | -0.3609 | 0.07818 | 4.05E-06 |
| ebi-a-GCST90001851 | rs12729972 | 1 | 118256346 | T | 0.4056 | -0.1371 | 0.02745 | 6.24E-07 |
| ebi-a-GCST90001539 | rs12734326 | 1 | 240644644 | T | 0.3881 | 0.1218 | 0.02571 | 2.25E-06 |
| ebi-a-GCST90001530 | rs12736179 | 1 | 20887141 | T | 0.3032 | -0.1687 | 0.03599 | 2.96E-06 |
| ebi-a-GCST90001979 | rs12737621 | 1 | 18698947 | G | 0.386 | -0.1212 | 0.02496 | 1.24E-06 |
| ebi-a-GCST90001792 | rs12739411 | 1 | 68752573 | C | 0.018 | 0.4289 | 0.09273 | 3.88E-06 |
| ebi-a-GCST90001856 | rs12742947 | 1 | 165193662 | A | 0.0999 | -0.2345 | 0.04561 | 2.91E-07 |
| ebi-a-GCST90001493 | rs12744266 | 1 | 248047667 | A | 0.2979 | 0.1243 | 0.02583 | 1.55E-06 |
| ebi-a-GCST90001827 | rs12745074 | 1 | 7337805 | G | 0.0805 | 0.2087 | 0.04521 | 4.02E-06 |
| ebi-a-GCST90001698 | rs12750174 | 1 | 184765000 | A | 0.0035 | 166.6 | 29.17 | 1.22E-08 |
| ebi-a-GCST90001869 | rs12752576 | 1 | 168605042 | G | 0.0154 | -0.7279 | 0.1101 | 4.57E-11 |
| ebi-a-GCST90001688 | rs12755474 | 1 | 216352525 | G | 1.00E-04 | 6.643 | 1.199 | 3.22E-08 |
| ebi-a-GCST90001436 | rs12763172 | 10 | 85447519 | C | 0.7213 | 0.132 | 0.02749 | 1.64E-06 |
| ebi-a-GCST90001813 | rs12764658 | 10 | 69890943 | T | 0.1944 | 0.1563 | 0.03179 | 9.20E-07 |
| ebi-a-GCST90001912 | rs12772032 | 10 | 125376027 | T | 0.3482 | -0.1338 | 0.02881 | 3.57E-06 |
| ebi-a-GCST90001961 | rs12778618 | 10 | 95633120 | C | 0.0457 | -0.6486 | 0.06633 | 3.04E-22 |
| ebi-a-GCST90002069 | rs12778618 | 10 | 95633120 | C | 0.0457 | 0.3488 | 0.06589 | 1.29E-07 |
| ebi-a-GCST90001888 | rs12778618 | 10 | 95633120 | C | 0.0457 | 0.4215 | 0.06475 | 8.83E-11 |
| ebi-a-GCST90001892 | rs12778618 | 10 | 95633120 | C | 0.0457 | 0.5196 | 0.06591 | 4.44E-15 |
| ebi-a-GCST90002061 | rs12778618 | 10 | 95633120 | C | 0.0457 | 0.5908 | 0.06675 | 1.47E-18 |
| ebi-a-GCST90001671 | rs12778618 | 10 | 95633120 | C | 0.0462 | 0.8186 | 0.06103 | 5.07E-40 |
| ebi-a-GCST90001670 | rs12778618 | 10 | 95633120 | C | 0.0462 | 0.8332 | 0.06081 | 1.23E-41 |
| ebi-a-GCST90001672 | rs12778618 | 10 | 95633120 | C | 0.0461 | 0.8528 | 0.06178 | 3.23E-42 |
| ebi-a-GCST90001672 | rs12778797 | 10 | 96998688 | C | 0.3841 | 0.2118 | 0.0271 | 7.19E-15 |
| ebi-a-GCST90001659 | rs12778797 | 10 | 96998688 | C | 0.3836 | 0.2506 | 0.02546 | 1.46E-22 |
| ebi-a-GCST90001660 | rs12778797 | 10 | 96998688 | C | 0.3841 | 0.2544 | 0.02672 | 3.12E-21 |
| ebi-a-GCST90001658 | rs12778797 | 10 | 96998688 | C | 0.3836 | 0.2592 | 0.02544 | 4.75E-24 |
| ebi-a-GCST90002031 | rs12778797 | 10 | 96998688 | C | 0.3854 | 0.2849 | 0.02836 | 2.24E-23 |
| ebi-a-GCST90002030 | rs12778797 | 10 | 96998688 | C | 0.3854 | 0.3052 | 0.02778 | 1.54E-27 |
| ebi-a-GCST90001485 | rs12778837 | 10 | 96999787 | C | 0.3595 | 0.1965 | 0.02651 | 1.55E-13 |
| ebi-a-GCST90001484 | rs12778837 | 10 | 96999787 | C | 0.3595 | 0.2009 | 0.02654 | 4.85E-14 |
| ebi-a-GCST90001670 | rs12778837 | 10 | 96999787 | C | 0.3594 | 0.2205 | 0.0273 | 9.08E-16 |
| ebi-a-GCST90002078 | rs12788263 | 11 | 22490590 | A | 0.0031 | 1.117 | 0.2412 | 3.81E-06 |
| ebi-a-GCST90001697 | rs12789925 | 11 | 11865406 | T | 0.0695 | 1.133 | 0.2445 | 3.70E-06 |
| ebi-a-GCST90001696 | rs12791920 | 11 | 910259 | G | 0.4593 | -1.512 | 0.306 | 8.14E-07 |
| ebi-a-GCST90001622 | rs12792298 | 11 | 112189184 | T | 0.0345 | -0.3574 | 0.0667 | 8.94E-08 |
| ebi-a-GCST90001621 | rs12792298 | 11 | 112189184 | T | 0.0345 | -0.3521 | 0.06692 | 1.51E-07 |
| ebi-a-GCST90001623 | rs12792298 | 11 | 112189184 | T | 0.0345 | -0.3438 | 0.06711 | 3.17E-07 |
| ebi-a-GCST90001637 | rs12792298 | 11 | 112189184 | T | 0.0345 | -0.3195 | 0.06599 | 1.34E-06 |
| ebi-a-GCST90001630 | rs12792298 | 11 | 112189184 | T | 0.0345 | -0.3155 | 0.06667 | 2.30E-06 |
| ebi-a-GCST90001631 | rs12792298 | 11 | 112189184 | T | 0.0345 | -0.3117 | 0.0657 | 2.18E-06 |
| ebi-a-GCST90001638 | rs12792298 | 11 | 112189184 | T | 0.0345 | -0.3106 | 0.06573 | 2.38E-06 |
| ebi-a-GCST90001632 | rs12792298 | 11 | 112189184 | T | 0.0345 | -0.3039 | 0.06611 | 4.44E-06 |
| ebi-a-GCST90001394 | rs12799241 | 11 | 119980729 | A | 0.2619 | 0.13 | 0.0273 | 1.99E-06 |
| ebi-a-GCST90001416 | rs12799241 | 11 | 119980729 | A | 0.2619 | 0.132 | 0.02794 | 2.39E-06 |
| ebi-a-GCST90001409 | rs12799241 | 11 | 119980729 | A | 0.2619 | 0.138 | 0.02805 | 9.07E-07 |
| ebi-a-GCST90001921 | rs12820360 | 12 | 82099799 | A | 0.0038 | -1.122 | 0.213 | 1.50E-07 |
| ebi-a-GCST90001795 | rs12822478 | 12 | 31514566 | A | 0.4841 | -0.1156 | 0.02508 | 4.20E-06 |
| ebi-a-GCST90001442 | rs12829274 | 12 | 129349167 | G | 0.0618 | -0.2708 | 0.05111 | 1.24E-07 |
| ebi-a-GCST90001432 | rs12829274 | 12 | 129349167 | G | 0.0618 | -0.2536 | 0.0511 | 7.31E-07 |
| ebi-a-GCST90001431 | rs12829274 | 12 | 129349167 | G | 0.0618 | -0.2515 | 0.05194 | 1.34E-06 |
| ebi-a-GCST90001793 | rs12830810 | 12 | 47930410 | C | 0.4025 | -0.1229 | 0.02556 | 1.59E-06 |
| ebi-a-GCST90001879 | rs12833432 | 12 | 130668546 | T | 0.1526 | -0.2759 | 0.05532 | 6.99E-07 |
| ebi-a-GCST90001832 | rs12864970 | 13 | 26218284 | A | 0.4894 | 0.1188 | 0.02579 | 4.33E-06 |
| ebi-a-GCST90001642 | rs12865434 | 13 | 108970631 | G | 0.4777 | 0.1537 | 0.02404 | 1.83E-10 |
| ebi-a-GCST90001597 | rs12871857 | 13 | 26527159 | T | 0.06 | -0.2478 | 0.0518 | 1.78E-06 |
| ebi-a-GCST90001635 | rs12871857 | 13 | 26527159 | T | 0.06 | -0.241 | 0.05179 | 3.38E-06 |
| ebi-a-GCST90001634 | rs12871857 | 13 | 26527159 | T | 0.06 | -0.2409 | 0.05202 | 3.76E-06 |
| ebi-a-GCST90001612 | rs12871857 | 13 | 26527159 | T | 0.06 | -0.2405 | 0.05208 | 4.01E-06 |
| ebi-a-GCST90001588 | rs12874404 | 13 | 108993494 | G | 0.3195 | -0.2201 | 0.02651 | 1.43E-16 |
| ebi-a-GCST90001646 | rs12874404 | 13 | 108993494 | G | 0.319 | -0.1456 | 0.02572 | 1.60E-08 |
| ebi-a-GCST90001411 | rs12874404 | 13 | 108993494 | G | 0.3257 | 0.1313 | 0.027 | 1.20E-06 |
| ebi-a-GCST90001396 | rs12874404 | 13 | 108993494 | G | 0.3256 | 0.1363 | 0.02669 | 3.42E-07 |
| ebi-a-GCST90001430 | rs12874404 | 13 | 108993494 | G | 0.3256 | 0.1366 | 0.02617 | 1.90E-07 |
| ebi-a-GCST90001427 | rs12874404 | 13 | 108993494 | G | 0.3256 | 0.1408 | 0.02668 | 1.38E-07 |
| ebi-a-GCST90001398 | rs12874404 | 13 | 108993494 | G | 0.3256 | 0.1523 | 0.02639 | 8.62E-09 |
| ebi-a-GCST90001418 | rs12874404 | 13 | 108993494 | G | 0.3256 | 0.1691 | 0.02653 | 2.04E-10 |
| ebi-a-GCST90001431 | rs12874404 | 13 | 108993494 | G | 0.3256 | 0.1714 | 0.02663 | 1.39E-10 |
| ebi-a-GCST90001407 | rs12874404 | 13 | 108993494 | G | 0.3256 | 0.173 | 0.02661 | 9.14E-11 |
| ebi-a-GCST90001432 | rs12874404 | 13 | 108993494 | G | 0.3256 | 0.1827 | 0.02625 | 4.02E-12 |
| ebi-a-GCST90001424 | rs12874404 | 13 | 108993494 | G | 0.3257 | 0.1898 | 0.02599 | 3.43E-13 |
| ebi-a-GCST90001412 | rs12874404 | 13 | 108993494 | G | 0.3256 | 0.1963 | 0.02601 | 5.62E-14 |
| ebi-a-GCST90001442 | rs12874404 | 13 | 108993494 | G | 0.3256 | 0.2114 | 0.02614 | 8.09E-16 |
| ebi-a-GCST90001436 | rs12874404 | 13 | 108993494 | G | 0.3256 | 0.2221 | 0.0262 | 3.31E-17 |
| ebi-a-GCST90001644 | rs12874404 | 13 | 108993494 | G | 0.319 | 0.2272 | 0.02633 | 8.99E-18 |
| ebi-a-GCST90001439 | rs12874404 | 13 | 108993494 | G | 0.3256 | 0.2335 | 0.02632 | 1.09E-18 |
| ebi-a-GCST90001855 | rs12884372 | 14 | 86036273 | G | 0.0766 | 0.2567 | 0.05224 | 9.42E-07 |
| ebi-a-GCST90001918 | rs12884891 | 14 | 90027519 | A | 0.2021 | 0.165 | 0.03315 | 6.79E-07 |
| ebi-a-GCST90001530 | rs12885009 | 14 | 85063049 | A | 0.6873 | -0.1714 | 0.03654 | 2.93E-06 |
| ebi-a-GCST90001793 | rs12886102 | 14 | 100108322 | T | 0.2186 | -0.1394 | 0.029 | 1.61E-06 |
| ebi-a-GCST90001790 | rs12886102 | 14 | 100108322 | T | 0.2186 | -0.1341 | 0.02881 | 3.39E-06 |
| ebi-a-GCST90002057 | rs12894741 | 14 | 53791493 | C | 0.0271 | -0.3959 | 0.08429 | 2.76E-06 |
| ebi-a-GCST90001621 | rs12895031 | 14 | 99846398 | T | 0.1044 | -0.207 | 0.04067 | 3.80E-07 |
| ebi-a-GCST90001750 | rs12898020 | 14 | 90835752 | G | 0.5016 | 0.16 | 0.03318 | 1.55E-06 |
| ebi-a-GCST90001567 | rs1290035 | 14 | 69856859 | C | 0.7776 | -0.126 | 0.02748 | 4.73E-06 |
| ebi-a-GCST90001690 | rs12901644 | 15 | 36456429 | T | 0.0929 | -0.1685 | 0.03056 | 3.80E-08 |
| ebi-a-GCST90001741 | rs1290397 | 17 | 77948063 | A | 0.5101 | 0.1139 | 0.02449 | 3.43E-06 |
| ebi-a-GCST90001730 | rs1290397 | 17 | 77948063 | A | 0.51 | 0.1153 | 0.02452 | 2.67E-06 |
| ebi-a-GCST90001725 | rs1290397 | 17 | 77948063 | A | 0.51 | 0.1188 | 0.0244 | 1.16E-06 |
| ebi-a-GCST90001598 | rs12904219 | 15 | 97967499 | G | 0.4302 | -0.1076 | 0.02338 | 4.34E-06 |
| ebi-a-GCST90001733 | rs12904834 | 15 | 77030792 | C | 0.6325 | -0.1252 | 0.02593 | 1.44E-06 |
| ebi-a-GCST90002078 | rs12906244 | 15 | 72199230 | A | 0.0019 | 1.53 | 0.3106 | 8.80E-07 |
| ebi-a-GCST90001394 | rs12906621 | 15 | 37177413 | A | 0.1091 | 0.1764 | 0.03817 | 3.91E-06 |
| ebi-a-GCST90001793 | rs12907742 | 15 | 101525737 | C | 0.0801 | -0.2324 | 0.04421 | 1.54E-07 |
| ebi-a-GCST90001777 | rs12907742 | 15 | 101525737 | C | 0.0801 | -0.2071 | 0.04383 | 2.37E-06 |
| ebi-a-GCST90001666 | rs12907798 | 15 | 36349479 | T | 0.1533 | -0.1409 | 0.03043 | 3.79E-06 |
| ebi-a-GCST90001566 | rs12907798 | 15 | 36349479 | T | 0.1535 | -0.1339 | 0.02765 | 1.34E-06 |
| ebi-a-GCST90001667 | rs12914989 | 15 | 26221496 | T | 0.4352 | 0.1202 | 0.02355 | 3.52E-07 |
| ebi-a-GCST90001639 | rs12915752 | 15 | 24590168 | C | 0.4387 | -0.1186 | 0.02542 | 3.18E-06 |
| ebi-a-GCST90001858 | rs12921174 | 16 | 430304 | C | 0.2038 | -0.2044 | 0.03371 | 1.49E-09 |
| ebi-a-GCST90001838 | rs12921174 | 16 | 430304 | C | 0.2041 | -0.1937 | 0.03404 | 1.40E-08 |
| ebi-a-GCST90001864 | rs12921174 | 16 | 430304 | C | 0.2038 | -0.1808 | 0.0337 | 8.78E-08 |
| ebi-a-GCST90002083 | rs12924236 | 16 | 10974423 | C | 0.2263 | 0.2199 | 0.03124 | 2.36E-12 |
| ebi-a-GCST90001918 | rs12924236 | 16 | 10974423 | C | 0.2263 | 0.2877 | 0.03115 | 4.55E-20 |
| ebi-a-GCST90002089 | rs12924903 | 16 | 30930983 | G | 0.7304 | 0.1457 | 0.03151 | 3.90E-06 |
| ebi-a-GCST90001911 | rs12928699 | 16 | 5027948 | T | 0.0765 | 0.2398 | 0.05098 | 2.67E-06 |
| ebi-a-GCST90002117 | rs12929277 | 16 | 10700928 | C | 0.2166 | 0.1523 | 0.03321 | 4.70E-06 |
| ebi-a-GCST90002110 | rs12929277 | 16 | 10700928 | C | 0.2166 | 0.2107 | 0.04373 | 1.59E-06 |
| ebi-a-GCST90002111 | rs12929277 | 16 | 10700928 | C | 0.2165 | 0.2132 | 0.04325 | 9.12E-07 |
| ebi-a-GCST90001680 | rs12930684 | 16 | 7317447 | C | 0.1301 | -0.1887 | 0.03842 | 9.45E-07 |
| ebi-a-GCST90001765 | rs12930821 | 16 | 58054671 | C | 0.7592 | -0.1445 | 0.02886 | 5.78E-07 |
| ebi-a-GCST90001771 | rs12930821 | 16 | 58054671 | C | 0.7592 | -0.1445 | 0.02896 | 6.33E-07 |
| ebi-a-GCST90001772 | rs12930821 | 16 | 58054671 | C | 0.7592 | -0.1357 | 0.02902 | 3.03E-06 |
| ebi-a-GCST90001762 | rs1293260 | 1 | 25709104 | G | 0.339 | 0.1227 | 0.02619 | 2.89E-06 |
| ebi-a-GCST90001521 | rs12933012 | 16 | 81880726 | G | 0.2317 | 0.1857 | 0.03951 | 2.79E-06 |
| ebi-a-GCST90001698 | rs12934979 | 16 | 87379050 | T | 0.0013 | 228.7 | 45.62 | 5.59E-07 |
| ebi-a-GCST90001910 | rs12939172 | 17 | 14526450 | C | 0.2906 | 0.1425 | 0.02794 | 3.62E-07 |
| ebi-a-GCST90001577 | rs12939565 | 17 | 38043649 | T | 0.4267 | -0.1444 | 0.0266 | 6.03E-08 |
| ebi-a-GCST90001424 | rs12939565 | 17 | 38043649 | T | 0.4267 | -0.142 | 0.02747 | 2.45E-07 |
| ebi-a-GCST90001392 | rs12939565 | 17 | 38043649 | T | 0.4267 | -0.1364 | 0.02672 | 3.47E-07 |
| ebi-a-GCST90001441 | rs12939565 | 17 | 38043649 | T | 0.4267 | -0.1318 | 0.02761 | 1.87E-06 |
| ebi-a-GCST90001578 | rs12939565 | 17 | 38043649 | T | 0.4267 | -0.1251 | 0.02681 | 3.19E-06 |
| ebi-a-GCST90001394 | rs12939566 | 17 | 38061439 | T | 0.4148 | -0.1486 | 0.02725 | 5.31E-08 |
| ebi-a-GCST90001630 | rs12940345 | 17 | 74612462 | T | 0.5487 | 0.1138 | 0.02473 | 4.33E-06 |
| ebi-a-GCST90001685 | rs12940345 | 17 | 74612462 | T | 0.5494 | 0.1177 | 0.02451 | 1.63E-06 |
| ebi-a-GCST90001687 | rs12940345 | 17 | 74612462 | T | 0.5503 | 0.1212 | 0.02523 | 1.61E-06 |
| ebi-a-GCST90001392 | rs1294253 | 1 | 233503875 | C | 0.7254 | -0.1382 | 0.02658 | 2.10E-07 |
| ebi-a-GCST90001884 | rs12944882 | 17 | 37983492 | C | 0.4639 | -0.1336 | 0.02747 | 1.22E-06 |
| ebi-a-GCST90002002 | rs12953239 | 17 | 35199795 | A | 0.0205 | -0.4006 | 0.08485 | 2.43E-06 |
| ebi-a-GCST90001993 | rs12953239 | 17 | 35199795 | A | 0.0205 | -0.3875 | 0.08446 | 4.62E-06 |
| ebi-a-GCST90001508 | rs12958644 | 18 | 6060693 | C | 0.0599 | 0.2701 | 0.05331 | 4.26E-07 |
| ebi-a-GCST90001732 | rs12968867 | 18 | 60850310 | A | 0.6744 | -0.137 | 0.02626 | 1.92E-07 |
| ebi-a-GCST90001731 | rs12968867 | 18 | 60850310 | A | 0.6743 | -0.1275 | 0.02628 | 1.28E-06 |
| ebi-a-GCST90002053 | rs12970861 | 18 | 66283602 | A | 0.0259 | -0.5575 | 0.1149 | 1.34E-06 |
| ebi-a-GCST90001874 | rs12972416 | 19 | 44220733 | G | 0.2069 | -0.2328 | 0.04967 | 3.09E-06 |
| ebi-a-GCST90001661 | rs12986962 | 2 | 111808558 | G | 0.2827 | -0.1604 | 0.02829 | 1.54E-08 |
| ebi-a-GCST90001663 | rs12986962 | 2 | 111808558 | G | 0.2826 | -0.1568 | 0.0284 | 3.59E-08 |
| ebi-a-GCST90001864 | rs12990220 | 2 | 45451862 | A | 0.1759 | -0.1751 | 0.03614 | 1.34E-06 |
| ebi-a-GCST90001838 | rs12994487 | 2 | 45448525 | G | 0.2589 | -0.1477 | 0.03217 | 4.59E-06 |
| ebi-a-GCST90001502 | rs13000564 | 2 | 41596014 | C | 0.2959 | 0.1141 | 0.02487 | 4.62E-06 |
| ebi-a-GCST90002118 | rs13004867 | 2 | 86982504 | G | 0.1175 | -0.4974 | 0.04176 | 5.63E-32 |
| ebi-a-GCST90001948 | rs13004897 | 2 | 143238505 | A | 0.0472 | 0.4164 | 0.08478 | 9.92E-07 |
| ebi-a-GCST90002119 | rs130065 | 6 | 31122500 | A | 0.1642 | -0.3127 | 0.04043 | 1.42E-14 |
| ebi-a-GCST90001697 | rs130071 | 6 | 31116210 | A | 0.2185 | 1.086 | 0.1686 | 1.37E-10 |
| ebi-a-GCST90001696 | rs130071 | 6 | 31116210 | A | 0.2185 | 2.545 | 0.4129 | 7.93E-10 |
| ebi-a-GCST90001695 | rs130071 | 6 | 31116210 | A | 0.2188 | 22.81 | 3.563 | 1.74E-10 |
| ebi-a-GCST90001848 | rs13009946 | 2 | 219007752 | G | 0.5288 | -0.1342 | 0.02637 | 3.80E-07 |
| ebi-a-GCST90001698 | rs13010105 | 2 | 42107980 | G | 0.0113 | 98.72 | 17.57 | 2.08E-08 |
| ebi-a-GCST90001871 | rs13014690 | 2 | 153797694 | A | 0.2686 | 0.2167 | 0.04612 | 2.91E-06 |
| ebi-a-GCST90002121 | rs13017213 | 2 | 79976286 | C | 0.4882 | -0.1318 | 0.02706 | 1.19E-06 |
| ebi-a-GCST90001899 | rs13017710 | 2 | 131710625 | A | 0.6135 | -0.1325 | 0.02662 | 6.81E-07 |
| ebi-a-GCST90001894 | rs13023055 | 2 | 20856144 | A | 0.0705 | -0.249 | 0.0531 | 2.88E-06 |
| ebi-a-GCST90001559 | rs13023673 | 2 | 38594483 | C | 0.1792 | 0.1566 | 0.03366 | 3.40E-06 |
| ebi-a-GCST90001558 | rs13023673 | 2 | 38594483 | C | 0.1792 | 0.1837 | 0.03302 | 2.87E-08 |
| ebi-a-GCST90002119 | rs13024436 | 2 | 86549088 | A | 0.3759 | 0.1591 | 0.02774 | 1.08E-08 |
| ebi-a-GCST90002018 | rs13024798 | 2 | 3033382 | T | 0.1096 | 0.2128 | 0.04587 | 3.68E-06 |
| ebi-a-GCST90002026 | rs13024840 | 2 | 38897074 | A | 0.2639 | 0.1623 | 0.03094 | 1.66E-07 |
| ebi-a-GCST90001547 | rs13024840 | 2 | 38897074 | A | 0.2578 | 0.2049 | 0.02827 | 5.23E-13 |
| ebi-a-GCST90001546 | rs13024840 | 2 | 38897074 | A | 0.2578 | 0.2065 | 0.02846 | 4.89E-13 |
| ebi-a-GCST90001509 | rs13024840 | 2 | 38897074 | A | 0.2587 | 0.2465 | 0.02917 | 4.22E-17 |
| ebi-a-GCST90001508 | rs13024840 | 2 | 38897074 | A | 0.2587 | 0.2764 | 0.02911 | 4.05E-21 |
| ebi-a-GCST90002102 | rs13024840 | 2 | 38897074 | A | 0.2648 | 0.3454 | 0.02873 | 1.57E-32 |
| ebi-a-GCST90001729 | rs13030427 | 2 | 208879261 | A | 0.1957 | 0.1463 | 0.03178 | 4.29E-06 |
| ebi-a-GCST90001698 | rs13039776 | 20 | 62915126 | G | 0.0029 | 165.5 | 32.04 | 2.55E-07 |
| ebi-a-GCST90001983 | rs13043397 | 20 | 42848689 | T | 0.3021 | 0.1236 | 0.0267 | 3.82E-06 |
| ebi-a-GCST90001545 | rs1304374 | 21 | 32198368 | A | 0.463 | -0.1207 | 0.02593 | 3.36E-06 |
| ebi-a-GCST90001695 | rs13044559 | 20 | 36073660 | A | NA | 1678 | 304.6 | 3.88E-08 |
| ebi-a-GCST90001786 | rs13049974 | 21 | 32810944 | C | 0.0479 | -0.2726 | 0.05958 | 4.92E-06 |
| ebi-a-GCST90001661 | rs13054887 | 22 | 43169818 | C | 0.3499 | -0.1247 | 0.02667 | 3.01E-06 |
| ebi-a-GCST90001717 | rs13058590 | 22 | 39641412 | T | 0.0468 | -0.3847 | 0.05918 | 9.08E-11 |
| ebi-a-GCST90001711 | rs13058590 | 22 | 39641412 | T | 0.0468 | -0.3797 | 0.05894 | 1.33E-10 |
| ebi-a-GCST90001702 | rs13058590 | 22 | 39641412 | T | 0.0468 | -0.3738 | 0.05926 | 3.16E-10 |
| ebi-a-GCST90001718 | rs13058590 | 22 | 39641412 | T | 0.0468 | -0.3673 | 0.05933 | 6.66E-10 |
| ebi-a-GCST90001715 | rs13058590 | 22 | 39641412 | T | 0.0468 | -0.3667 | 0.0592 | 6.49E-10 |
| ebi-a-GCST90001710 | rs13058590 | 22 | 39641412 | T | 0.0468 | -0.355 | 0.05912 | 2.10E-09 |
| ebi-a-GCST90002008 | rs13060505 | 3 | 46968023 | G | 0.0234 | 0.382 | 0.08248 | 3.76E-06 |
| ebi-a-GCST90002004 | rs13060505 | 3 | 46968023 | G | 0.0234 | 0.4217 | 0.08148 | 2.39E-07 |
| ebi-a-GCST90002017 | rs13060505 | 3 | 46968023 | G | 0.026 | 0.4738 | 0.08867 | 9.85E-08 |
| ebi-a-GCST90001749 | rs13061533 | 3 | 188611158 | T | 0.181 | 0.2044 | 0.04395 | 3.54E-06 |
| ebi-a-GCST90001752 | rs1306395 | 2 | 61076272 | C | 0.5145 | 0.1157 | 0.02527 | 4.87E-06 |
| ebi-a-GCST90002106 | rs13064098 | 3 | 73320945 | T | 0.2367 | -0.1564 | 0.03257 | 1.66E-06 |
| ebi-a-GCST90001674 | rs13068352 | 3 | 172724426 | T | 0.0789 | 0.2315 | 0.04763 | 1.23E-06 |
| ebi-a-GCST90001450 | rs13068785 | 3 | 74703663 | T | 0.0581 | 0.269 | 0.05861 | 4.62E-06 |
| ebi-a-GCST90002029 | rs13070466 | 3 | 87382102 | A | 0.0961 | 0.2182 | 0.04676 | 3.20E-06 |
| ebi-a-GCST90001747 | rs13070807 | 3 | 56847945 | G | 0.6374 | -0.1205 | 0.02576 | 2.99E-06 |
| ebi-a-GCST90001849 | rs13072593 | 3 | 150288489 | G | 0.2593 | 0.1445 | 0.03122 | 3.85E-06 |
| ebi-a-GCST90001984 | rs13076004 | 3 | 34698655 | G | 0.1487 | -0.1591 | 0.03462 | 4.47E-06 |
| ebi-a-GCST90001758 | rs13080324 | 3 | 111952407 | A | 0.4322 | 0.1159 | 0.02501 | 3.73E-06 |
| ebi-a-GCST90001466 | rs13088702 | 3 | 121714019 | C | 0.0252 | 0.3946 | 0.07957 | 7.45E-07 |
| ebi-a-GCST90001890 | rs13091429 | 3 | 118956779 | G | 0.1154 | 0.2011 | 0.04337 | 3.71E-06 |
| ebi-a-GCST90001924 | rs130970 | 22 | 34774585 | A | 0.3798 | -0.1281 | 0.028 | 4.93E-06 |
| ebi-a-GCST90001940 | rs13106248 | 4 | 5775106 | G | 0.0017 | -1.534 | 0.3132 | 1.02E-06 |
| ebi-a-GCST90001637 | rs13108209 | 4 | 4355328 | A | 0.7135 | -0.1293 | 0.02706 | 1.83E-06 |
| ebi-a-GCST90001636 | rs13108209 | 4 | 4355328 | A | 0.7133 | -0.1282 | 0.02676 | 1.73E-06 |
| ebi-a-GCST90001803 | rs13111916 | 4 | 13780721 | C | 0.1954 | 0.1445 | 0.0314 | 4.31E-06 |
| ebi-a-GCST90001688 | rs13115676 | 4 | 139253983 | A | 0.0523 | -0.1825 | 0.03943 | 3.84E-06 |
| ebi-a-GCST90001583 | rs13121143 | 4 | 190199873 | C | 0.1536 | 0.1693 | 0.03309 | 3.29E-07 |
| ebi-a-GCST90001582 | rs13121143 | 4 | 190199873 | C | 0.1536 | 0.1703 | 0.03307 | 2.75E-07 |
| ebi-a-GCST90001412 | rs13132141 | 4 | 156739300 | A | 0.6385 | -0.1191 | 0.02557 | 3.30E-06 |
| ebi-a-GCST90001760 | rs13133046 | 4 | 23393356 | G | 0.0245 | -0.3755 | 0.07765 | 1.38E-06 |
| ebi-a-GCST90001699 | rs13133130 | 4 | 67792751 | G | 0.5321 | 1.65 | 0.3487 | 2.33E-06 |
| ebi-a-GCST90001873 | rs13136753 | 4 | 17244840 | G | 0.0609 | -0.4042 | 0.08595 | 2.85E-06 |
| ebi-a-GCST90001468 | rs13148506 | 4 | 43123482 | T | 0.3691 | 0.1304 | 0.02714 | 1.62E-06 |
| ebi-a-GCST90002085 | rs13159652 | 5 | 86130997 | T | 0.2088 | -0.1566 | 0.03208 | 1.11E-06 |
| ebi-a-GCST90001742 | rs13164259 | 5 | 82870211 | A | 0.0083 | 0.6227 | 0.1338 | 3.37E-06 |
| ebi-a-GCST90001927 | rs13165333 | 5 | 82275571 | T | 0.1036 | 0.1984 | 0.04226 | 2.79E-06 |
| ebi-a-GCST90001569 | rs13170001 | 5 | 2685550 | T | 0.2758 | -0.1307 | 0.02667 | 9.92E-07 |
| ebi-a-GCST90001849 | rs13170201 | 5 | 30950823 | C | 0.7173 | -0.1416 | 0.03031 | 3.11E-06 |
| ebi-a-GCST90001765 | rs13171255 | 5 | 159374162 | G | 0.1514 | 0.1606 | 0.03496 | 4.49E-06 |
| ebi-a-GCST90002056 | rs13171873 | 5 | 3080656 | A | 0.1008 | -0.2102 | 0.04581 | 4.66E-06 |
| ebi-a-GCST90002114 | rs13175786 | 5 | 7447525 | C | 0.1028 | -0.1987 | 0.04215 | 2.53E-06 |
| ebi-a-GCST90001845 | rs1317631 | 1 | 165904360 | T | 0.8155 | -0.1828 | 0.03573 | 3.35E-07 |
| ebi-a-GCST90001841 | rs1317631 | 1 | 165904360 | T | 0.8156 | -0.1719 | 0.03607 | 1.97E-06 |
| ebi-a-GCST90001922 | rs13176824 | 5 | 170977932 | C | 0.0377 | -0.326 | 0.07065 | 4.12E-06 |
| ebi-a-GCST90001587 | rs13183256 | 5 | 37116496 | A | 0.121 | -0.1714 | 0.03722 | 4.24E-06 |
| ebi-a-GCST90001586 | rs13183256 | 5 | 37116496 | A | 0.121 | 0.172 | 0.0372 | 3.90E-06 |
| ebi-a-GCST90001744 | rs1318389 | 13 | 35149649 | C | 0.9664 | 0.3135 | 0.06855 | 4.94E-06 |
| ebi-a-GCST90001554 | rs13187183 | 5 | 117976098 | C | 0.0841 | -0.2346 | 0.04655 | 4.90E-07 |
| ebi-a-GCST90001682 | rs13187738 | 5 | 82289904 | G | 0.1099 | -0.1922 | 0.03986 | 1.50E-06 |
| ebi-a-GCST90001837 | rs13189938 | 5 | 60852845 | G | 0.0382 | 0.4694 | 0.1021 | 4.73E-06 |
| ebi-a-GCST90001748 | rs13190846 | 6 | 29905727 | T | 0.328 | 0.1625 | 0.03109 | 1.84E-07 |
| ebi-a-GCST90001758 | rs13190846 | 6 | 29905727 | T | 0.328 | 0.1821 | 0.03141 | 7.35E-09 |
| ebi-a-GCST90001973 | rs13191523 | 6 | 31012256 | G | 0.1118 | 0.2769 | 0.04585 | 1.73E-09 |
| ebi-a-GCST90001447 | rs13196511 | 6 | 44643196 | A | 0.0913 | -0.2113 | 0.04244 | 6.72E-07 |
| ebi-a-GCST90002055 | rs13196568 | 6 | 31098400 | C | 0.273 | -0.178 | 0.03389 | 1.62E-07 |
| ebi-a-GCST90001987 | rs1320440 | 14 | 101333700 | C | 0.0863 | 0.2027 | 0.04342 | 3.13E-06 |
| ebi-a-GCST90001815 | rs13209828 | 6 | 92746910 | C | 0.1406 | -0.1769 | 0.03536 | 5.91E-07 |
| ebi-a-GCST90001606 | rs13210358 | 6 | 102272234 | T | 0.0315 | -0.3968 | 0.07123 | 2.73E-08 |
| ebi-a-GCST90001591 | rs13210358 | 6 | 102272234 | T | 0.0315 | -0.3507 | 0.07187 | 1.11E-06 |
| ebi-a-GCST90001571 | rs13210358 | 6 | 102272234 | T | 0.0312 | 0.4707 | 0.07114 | 4.24E-11 |
| ebi-a-GCST90001624 | rs1322067 | 9 | 117660933 | G | 0.4173 | -0.1511 | 0.02575 | 4.75E-09 |
| ebi-a-GCST90002111 | rs13226058 | 7 | 150536031 | A | 0.0073 | 0.9649 | 0.2049 | 2.70E-06 |
| ebi-a-GCST90001533 | rs13237906 | 7 | 101155469 | T | 0.4297 | 0.157 | 0.03299 | 2.10E-06 |
| ebi-a-GCST90001855 | rs1324073 | 6 | 88822616 | C | 0.6519 | -0.1502 | 0.0289 | 2.18E-07 |
| ebi-a-GCST90001869 | rs1324073 | 6 | 88822616 | C | 0.652 | -0.1407 | 0.0288 | 1.10E-06 |
| ebi-a-GCST90001449 | rs13241407 | 7 | 153684820 | G | 0.0483 | -0.28 | 0.06121 | 4.95E-06 |
| ebi-a-GCST90001688 | rs13243221 | 7 | 80880540 | G | 1.00E-04 | 2.985 | 0.6167 | 1.35E-06 |
| ebi-a-GCST90001666 | rs13243221 | 7 | 80880540 | G | 1.00E-04 | 3.637 | 0.7844 | 3.68E-06 |
| ebi-a-GCST90001435 | rs13244540 | 7 | 151002534 | T | 0.0447 | 0.2681 | 0.05848 | 4.70E-06 |
| ebi-a-GCST90001983 | rs13248932 | 8 | 58311929 | C | 0.1187 | 0.1727 | 0.03761 | 4.55E-06 |
| ebi-a-GCST90001698 | rs13249758 | 8 | 128271231 | G | 0.0106 | 85.91 | 17.34 | 7.63E-07 |
| ebi-a-GCST90001728 | rs13250240 | 8 | 130552677 | C | 0.9945 | 1.066 | 0.228 | 3.17E-06 |
| ebi-a-GCST90002088 | rs13255633 | 8 | 138231016 | T | 0.1784 | 0.1598 | 0.03486 | 4.76E-06 |
| ebi-a-GCST90002087 | rs13255633 | 8 | 138231016 | T | 0.1784 | 0.1629 | 0.03496 | 3.31E-06 |
| ebi-a-GCST90001630 | rs1325907 | 10 | 90237398 | A | 0.5795 | -0.1298 | 0.02573 | 4.73E-07 |
| ebi-a-GCST90001580 | rs13260841 | 8 | 19431500 | T | 0.2451 | -0.1335 | 0.02899 | 4.24E-06 |
| ebi-a-GCST90002046 | rs1326284 | 1 | 198553794 | C | 0.2893 | 0.2087 | 0.03919 | 1.14E-07 |
| ebi-a-GCST90001700 | rs132751 | 22 | 36610967 | T | 0.0012 | 11.84 | 2.178 | 5.73E-08 |
| ebi-a-GCST90001698 | rs132751 | 22 | 36610967 | T | 0.0012 | 417.3 | 51.4 | 6.59E-16 |
| ebi-a-GCST90002016 | rs13276925 | 8 | 62682295 | C | 0.4465 | 0.1321 | 0.02866 | 4.25E-06 |
| ebi-a-GCST90002015 | rs13276925 | 8 | 62682295 | C | 0.4465 | 0.1323 | 0.02871 | 4.24E-06 |
| ebi-a-GCST90001698 | rs13293817 | 9 | 138791163 | A | 0.0053 | 114.6 | 24.58 | 3.27E-06 |
| ebi-a-GCST90001459 | rs1330145 | 9 | 110467582 | G | 0.5802 | 0.1193 | 0.02592 | 4.35E-06 |
| ebi-a-GCST90001959 | rs13316780 | 3 | 108851829 | C | 0.0119 | 0.5642 | 0.1231 | 4.72E-06 |
| ebi-a-GCST90001939 | rs1332159 | 9 | 25552600 | G | 0.4418 | -0.127 | 0.02572 | 8.30E-07 |
| ebi-a-GCST90001587 | rs13330943 | 16 | 8615778 | C | 0.6667 | -0.1248 | 0.026 | 1.64E-06 |
| ebi-a-GCST90001586 | rs13330943 | 16 | 8615778 | C | 0.6667 | 0.122 | 0.02599 | 2.81E-06 |
| ebi-a-GCST90001815 | rs13331697 | 16 | 81910840 | A | 0.1384 | -0.189 | 0.03517 | 8.18E-08 |
| ebi-a-GCST90001518 | rs13334605 | 16 | 8615510 | G | 0.6631 | 0.1663 | 0.03526 | 2.58E-06 |
| ebi-a-GCST90001517 | rs13334605 | 16 | 8615510 | G | 0.6631 | 0.1693 | 0.03482 | 1.26E-06 |
| ebi-a-GCST90001801 | rs13336159 | 16 | 8832952 | T | 0.2508 | 0.1858 | 0.04006 | 3.76E-06 |
| ebi-a-GCST90001835 | rs13336680 | 16 | 5615903 | C | 0.2299 | -0.1476 | 0.03127 | 2.49E-06 |
| ebi-a-GCST90001567 | rs13339407 | 16 | 78159277 | G | 0.2569 | -0.1196 | 0.02611 | 4.79E-06 |
| ebi-a-GCST90001848 | rs13345102 | 19 | 17535188 | T | 0.2067 | 0.1706 | 0.03205 | 1.10E-07 |
| ebi-a-GCST90001552 | rs13359589 | 5 | 73116673 | T | 0.0012 | 1.382 | 0.2769 | 6.34E-07 |
| ebi-a-GCST90001567 | rs13359589 | 5 | 73116673 | T | 0.0012 | 1.751 | 0.3478 | 5.04E-07 |
| ebi-a-GCST90002005 | rs13361130 | 5 | 177378903 | A | 0.0024 | -1.151 | 0.2386 | 1.46E-06 |
| ebi-a-GCST90001754 | rs13381717 | 18 | 352095 | T | 0.0774 | -0.2344 | 0.04602 | 3.69E-07 |
| ebi-a-GCST90001740 | rs13382005 | 18 | 58304032 | G | 0.1065 | 0.1843 | 0.03966 | 3.51E-06 |
| ebi-a-GCST90001553 | rs13386443 | 2 | 111722118 | G | 0.4758 | 0.07817 | 0.01705 | 4.71E-06 |
| ebi-a-GCST90001891 | rs13390252 | 2 | 204596729 | G | 0.199 | -0.4805 | 0.03356 | 5.57E-45 |
| ebi-a-GCST90001721 | rs13390788 | 2 | 235624835 | G | 0.2607 | 0.13 | 0.02761 | 2.60E-06 |
| ebi-a-GCST90001722 | rs13391592 | 2 | 180409011 | A | 0.0708 | 0.2246 | 0.04714 | 1.96E-06 |
| ebi-a-GCST90001997 | rs13396271 | 2 | 17055946 | G | 0.1611 | 0.1581 | 0.03444 | 4.59E-06 |
| ebi-a-GCST90001901 | rs13404978 | 2 | 204653886 | T | 0.1977 | -0.1787 | 0.0342 | 1.87E-07 |
| ebi-a-GCST90001478 | rs13410035 | 2 | 161139489 | T | 0.0346 | -0.3267 | 0.06743 | 1.32E-06 |
| ebi-a-GCST90001666 | rs1341624 | 1 | 191433917 | T | 0.0255 | 0.3324 | 0.07207 | 4.12E-06 |
| ebi-a-GCST90001664 | rs1341624 | 1 | 191433917 | T | 0.0253 | 0.349 | 0.07604 | 4.58E-06 |
| ebi-a-GCST90001589 | rs13418326 | 2 | 113732809 | G | 0.2063 | -0.1442 | 0.03102 | 3.48E-06 |
| ebi-a-GCST90001614 | rs13418326 | 2 | 113732809 | G | 0.2058 | 0.1478 | 0.03049 | 1.31E-06 |
| ebi-a-GCST90001607 | rs13418326 | 2 | 113732809 | G | 0.2058 | 0.1508 | 0.0305 | 7.98E-07 |
| ebi-a-GCST90001593 | rs13418326 | 2 | 113732809 | G | 0.2058 | 0.1508 | 0.03053 | 8.24E-07 |
| ebi-a-GCST90001408 | rs13421263 | 2 | 169182294 | T | 0.0528 | 0.2616 | 0.05638 | 3.62E-06 |
| ebi-a-GCST90001776 | rs13435269 | 4 | 95544162 | A | 0.2682 | 0.1419 | 0.02778 | 3.42E-07 |
| ebi-a-GCST90001564 | rs13439307 | 8 | 123030662 | A | 0.0465 | -0.2629 | 0.05554 | 2.29E-06 |
| ebi-a-GCST90001725 | rs1345010 | 2 | 174113733 | G | 0.0946 | 0.192 | 0.04147 | 3.79E-06 |
| ebi-a-GCST90001730 | rs1345010 | 2 | 174113733 | G | 0.0946 | 0.1933 | 0.04163 | 3.55E-06 |
| ebi-a-GCST90001566 | rs1348953 | 2 | 84213945 | A | 0.0106 | -0.4473 | 0.09644 | 3.65E-06 |
| ebi-a-GCST90002050 | rs1352606 | 5 | 66683389 | G | 0.5949 | -0.1939 | 0.0372 | 2.12E-07 |
| ebi-a-GCST90001472 | rs135299 | 22 | 48479731 | A | 0.2166 | 0.1435 | 0.03123 | 4.46E-06 |
| ebi-a-GCST90001530 | rs1354106 | 19 | 51737991 | G | 0.2237 | -0.4306 | 0.03825 | 1.72E-28 |
| ebi-a-GCST90002044 | rs1354185 | 5 | 13761068 | T | 0.1398 | -0.2367 | 0.05127 | 4.18E-06 |
| ebi-a-GCST90002119 | rs1356625 | 2 | 86022747 | C | 0.463 | 0.192 | 0.02696 | 1.37E-12 |
| ebi-a-GCST90001963 | rs1364526 | 2 | 57315946 | G | 0.789 | 0.1643 | 0.03468 | 2.27E-06 |
| ebi-a-GCST90001958 | rs1365928 | 2 | 19779555 | C | 0.2127 | -0.1661 | 0.03293 | 4.84E-07 |
| ebi-a-GCST90001698 | rs1368539 | 8 | 16526793 | A | 0.995 | -133.6 | 25.24 | 1.28E-07 |
| ebi-a-GCST90002069 | rs136883 | 22 | 45040462 | T | 0.3598 | 0.1498 | 0.02832 | 1.33E-07 |
| ebi-a-GCST90001785 | rs1370277 | 15 | 80445815 | T | 0.2096 | 0.1435 | 0.0305 | 2.65E-06 |
| ebi-a-GCST90001583 | rs1375493 | 2 | 182323766 | A | 0.3781 | 0.127 | 0.02481 | 3.26E-07 |
| ebi-a-GCST90001582 | rs1375493 | 2 | 182323766 | A | 0.3781 | 0.1517 | 0.02476 | 9.92E-10 |
| ebi-a-GCST90001996 | rs1376624 | 2 | 187771135 | C | 0.2161 | 0.1412 | 0.03053 | 3.90E-06 |
| ebi-a-GCST90001927 | rs1377028 | 4 | 75738792 | G | 0.5479 | -0.1222 | 0.02603 | 2.77E-06 |
| ebi-a-GCST90001688 | rs137858581 | 5 | 173002007 | C | 1.00E-04 | 3.974 | 0.7251 | 4.54E-08 |
| ebi-a-GCST90001886 | rs137875117 | 16 | 25627987 | A | 0.0057 | -0.8334 | 0.181 | 4.31E-06 |
| ebi-a-GCST90001921 | rs137878176 | 2 | 51742547 | T | 5.00E-04 | 2.7 | 0.5402 | 6.10E-07 |
| ebi-a-GCST90001504 | rs137903114 | 11 | 130770585 | C | 0.0035 | -0.9764 | 0.2119 | 4.22E-06 |
| ebi-a-GCST90001510 | rs137903114 | 11 | 130770585 | C | 0.0035 | -0.9749 | 0.2132 | 4.96E-06 |
| ebi-a-GCST90001590 | rs137903114 | 11 | 130770585 | C | 0.004 | -0.9366 | 0.1901 | 8.72E-07 |
| ebi-a-GCST90002078 | rs137910312 | 11 | 78834505 | A | 0.0286 | -0.4232 | 0.08297 | 3.58E-07 |
| ebi-a-GCST90001410 | rs137930158 | 8 | 109663198 | C | 0.007 | -0.6636 | 0.1413 | 2.76E-06 |
| ebi-a-GCST90001654 | rs137931178 | 6 | 32329150 | G | 0.1797 | -0.201 | 0.03778 | 1.10E-07 |
| ebi-a-GCST90001662 | rs137931178 | 6 | 32329150 | G | 0.1794 | -0.1841 | 0.03752 | 9.75E-07 |
| ebi-a-GCST90001668 | rs137931178 | 6 | 32329150 | G | 0.1794 | 0.1721 | 0.03698 | 3.37E-06 |
| ebi-a-GCST90001700 | rs137942933 | 20 | 33104122 | T | 6.00E-04 | 23.37 | 3.154 | 1.58E-13 |
| ebi-a-GCST90001695 | rs137942933 | 20 | 33104122 | T | 6.00E-04 | 304 | 55.65 | 5.06E-08 |
| ebi-a-GCST90001698 | rs137942933 | 20 | 33104122 | T | 6.00E-04 | 1000 | 72.99 | 1.18E-41 |
| ebi-a-GCST90001698 | rs137943198 | 12 | 10963475 | A | 9.00E-04 | 515.7 | 55.97 | 5.40E-20 |
| ebi-a-GCST90001735 | rs137949497 | 21 | 32136760 | G | 0.0146 | 0.4884 | 0.1009 | 1.35E-06 |
| ebi-a-GCST90001740 | rs137949497 | 21 | 32136760 | G | 0.0146 | 0.5022 | 0.1014 | 7.71E-07 |
| ebi-a-GCST90001698 | rs137963524 | 19 | 8899157 | C | 0.0029 | 148.2 | 32.28 | 4.56E-06 |
| ebi-a-GCST90001586 | rs137976321 | 20 | 50183940 | A | 0.019 | -0.4021 | 0.08741 | 4.35E-06 |
| ebi-a-GCST90001697 | rs137978465 | 2 | 228411157 | A | 4.00E-04 | 16.19 | 3.137 | 2.58E-07 |
| ebi-a-GCST90001695 | rs137978465 | 2 | 228411157 | A | 4.00E-04 | 641.1 | 67.08 | 2.24E-21 |
| ebi-a-GCST90001698 | rs137997289 | 7 | 115706613 | C | 0.0028 | 219.9 | 33.8 | 8.78E-11 |
| ebi-a-GCST90001695 | rs138010330 | 1 | 161526187 | T | 0.0048 | 111 | 19.7 | 1.89E-08 |
| ebi-a-GCST90002014 | rs138016528 | 16 | 72111834 | A | 0.0249 | 0.4089 | 0.08915 | 4.68E-06 |
| ebi-a-GCST90001698 | rs138020305 | 15 | 95184966 | T | 0.0204 | 64.03 | 12.28 | 1.95E-07 |
| ebi-a-GCST90001776 | rs138027128 | 16 | 7282705 | T | 0.0017 | 1.345 | 0.2886 | 3.27E-06 |
| ebi-a-GCST90002034 | rs138028805 | 1 | 38546640 | G | 0.0144 | -0.6087 | 0.1164 | 1.83E-07 |
| ebi-a-GCST90001913 | rs138030758 | 20 | 7743479 | T | 0.0064 | 0.7724 | 0.1663 | 3.54E-06 |
| ebi-a-GCST90001766 | rs138034828 | 10 | 111599201 | C | 0.0482 | 0.2825 | 0.05859 | 1.49E-06 |
| ebi-a-GCST90002082 | rs138038426 | 17 | 42576434 | C | 0.0039 | -0.8939 | 0.1877 | 2.01E-06 |
| ebi-a-GCST90002087 | rs138069865 | 10 | 115183723 | C | 0.001 | 1.872 | 0.4013 | 3.22E-06 |
| ebi-a-GCST90001536 | rs1380699 | 2 | 57906216 | G | 0.2015 | -0.1468 | 0.03075 | 1.90E-06 |
| ebi-a-GCST90002090 | rs138075266 | 7 | 28584086 | G | 0.003 | -1.161 | 0.2537 | 4.92E-06 |
| ebi-a-GCST90001910 | rs138082872 | 19 | 44344058 | T | 6.00E-04 | 2.331 | 0.5065 | 4.34E-06 |
| ebi-a-GCST90001898 | rs138089143 | 1 | 32615715 | C | 0.0079 | 0.6929 | 0.1489 | 3.39E-06 |
| ebi-a-GCST90001906 | rs138095875 | 3 | 98484408 | T | 0.009 | -0.7518 | 0.1463 | 2.97E-07 |
| ebi-a-GCST90002073 | rs1381043 | 12 | 74806815 | A | 0.0751 | 0.2553 | 0.05363 | 2.02E-06 |
| ebi-a-GCST90002016 | rs138130091 | 3 | 45509296 | T | 0.0098 | -0.6539 | 0.142 | 4.28E-06 |
| ebi-a-GCST90002096 | rs138132338 | 7 | 34480553 | T | 0.0585 | -0.3625 | 0.07652 | 2.37E-06 |
| ebi-a-GCST90001946 | rs138138479 | 10 | 22339808 | T | 0.0104 | 0.8518 | 0.1727 | 8.89E-07 |
| ebi-a-GCST90001698 | rs138152896 | 2 | 40546780 | G | 0.0021 | 244 | 36.89 | 4.30E-11 |
| ebi-a-GCST90001420 | rs138158989 | 2 | 204476635 | A | 0.0018 | -1.339 | 0.2832 | 2.33E-06 |
| ebi-a-GCST90001425 | rs138158989 | 2 | 204476635 | A | 0.0018 | -1.324 | 0.2808 | 2.50E-06 |
| ebi-a-GCST90001698 | rs1381623 | 14 | 84609045 | C | 0.9991 | -289.7 | 55.18 | 1.61E-07 |
| ebi-a-GCST90001901 | rs138199636 | 11 | 37444749 | T | 0.0036 | 1.149 | 0.2378 | 1.45E-06 |
| ebi-a-GCST90001619 | rs138213638 | 18 | 55985546 | C | 0.0014 | 1.483 | 0.3068 | 1.39E-06 |
| ebi-a-GCST90001618 | rs138213638 | 18 | 55985546 | C | 0.0014 | 1.498 | 0.3269 | 4.74E-06 |
| ebi-a-GCST90001628 | rs138213638 | 18 | 55985546 | C | 0.0014 | 1.501 | 0.3194 | 2.68E-06 |
| ebi-a-GCST90001669 | rs138232290 | 4 | 40701822 | A | 3.00E-04 | -2.743 | 0.5787 | 2.23E-06 |
| ebi-a-GCST90001698 | rs138246394 | 20 | 54228961 | A | 0.0016 | 262.1 | 43.79 | 2.38E-09 |
| ebi-a-GCST90001806 | rs138260228 | 14 | 29256374 | T | 0.0034 | -1.069 | 0.2126 | 5.26E-07 |
| ebi-a-GCST90001548 | rs138265071 | 2 | 233371652 | C | 0.0024 | 1.095 | 0.2333 | 2.78E-06 |
| ebi-a-GCST90001518 | rs138272175 | 9 | 945397 | T | 0.0355 | 0.4332 | 0.0905 | 1.83E-06 |
| ebi-a-GCST90002098 | rs138283413 | 2 | 40480936 | T | 0.0375 | 0.3299 | 0.0697 | 2.32E-06 |
| ebi-a-GCST90001926 | rs138284624 | 17 | 56385573 | T | 0.0277 | -0.7524 | 0.08106 | 3.17E-20 |
| ebi-a-GCST90002085 | rs138293681 | 9 | 19994802 | T | 0.0214 | -0.4259 | 0.09115 | 3.10E-06 |
| ebi-a-GCST90001698 | rs138296471 | 1 | 96963270 | A | 0.0183 | 69.27 | 13.49 | 2.97E-07 |
| ebi-a-GCST90001666 | rs138298107 | 1 | 213203523 | T | 0.0034 | 0.9379 | 0.2012 | 3.24E-06 |
| ebi-a-GCST90002037 | rs138318679 | 21 | 46094596 | T | 0.0691 | 0.2612 | 0.05496 | 2.11E-06 |
| ebi-a-GCST90002038 | rs138318679 | 21 | 46094596 | T | 0.0691 | 0.2618 | 0.05496 | 2.00E-06 |
| ebi-a-GCST90001695 | rs138324315 | 5 | 39721163 | A | 0.0028 | 138.5 | 30.03 | 4.11E-06 |
| ebi-a-GCST90001700 | rs138327786 | 6 | 1730982 | A | 3.00E-04 | 21.5 | 3.886 | 3.38E-08 |
| ebi-a-GCST90001698 | rs138327786 | 6 | 1730982 | A | 3.00E-04 | 1285 | 88.96 | 5.98E-46 |
| ebi-a-GCST90001418 | rs138329462 | 12 | 11634360 | A | 0.0174 | -0.4374 | 0.09365 | 3.11E-06 |
| ebi-a-GCST90001695 | rs138358136 | 7 | 194075 | T | 3.00E-04 | 726.8 | 82.02 | 1.26E-18 |
| ebi-a-GCST90002040 | rs138383232 | 9 | 86761155 | C | 0.0523 | -0.296 | 0.06364 | 3.46E-06 |
| ebi-a-GCST90001698 | rs138401052 | 3 | 166256176 | A | 0.0054 | 139.5 | 25.13 | 3.08E-08 |
| ebi-a-GCST90001700 | rs138410422 | 12 | 93329083 | A | 1.00E-04 | 28.99 | 5.954 | 1.17E-06 |
| ebi-a-GCST90001576 | rs1384161 | 1 | 99311831 | G | 0.6035 | 0.1168 | 0.02506 | 3.26E-06 |
| ebi-a-GCST90001577 | rs1384162 | 1 | 99311831 | G | 0.6035 | 0.1161 | 0.02451 | 2.25E-06 |
| ebi-a-GCST90001578 | rs1384162 | 1 | 99311831 | G | 0.6035 | 0.1231 | 0.02468 | 6.40E-07 |
| ebi-a-GCST90001749 | rs138448626 | 1 | 24084235 | T | 0.0823 | -0.2856 | 0.06212 | 4.55E-06 |
| ebi-a-GCST90001698 | rs138449036 | 7 | 29025284 | T | 6.00E-04 | 419.3 | 64.95 | 1.23E-10 |
| ebi-a-GCST90001813 | rs138459438 | 12 | 18290606 | A | 0.0666 | -0.2485 | 0.05 | 7.01E-07 |
| ebi-a-GCST90001818 | rs138459438 | 12 | 18290606 | A | 0.0667 | -0.2365 | 0.04978 | 2.10E-06 |
| ebi-a-GCST90001900 | rs138480662 | 2 | 205918886 | C | 0.0106 | -0.6131 | 0.1328 | 4.06E-06 |
| ebi-a-GCST90001491 | rs138483328 | 6 | 108484291 | T | 0.0141 | 0.4874 | 0.1054 | 3.86E-06 |
| ebi-a-GCST90001801 | rs138487400 | 1 | 65088698 | A | 0.0049 | -1.107 | 0.2335 | 2.29E-06 |
| ebi-a-GCST90001503 | rs138514398 | 11 | 66788802 | G | NA | -9.179 | 1.962 | 3.00E-06 |
| ebi-a-GCST90001494 | rs138514398 | 11 | 66788802 | G | NA | -9.098 | 1.973 | 4.17E-06 |
| ebi-a-GCST90001739 | rs138520806 | 5 | 3781931 | A | 0.047 | 0.2684 | 0.05834 | 4.35E-06 |
| ebi-a-GCST90002041 | rs138535375 | 18 | 22152471 | T | 0.0076 | -0.9674 | 0.204 | 2.31E-06 |
| ebi-a-GCST90001981 | rs138540869 | 5 | 62602530 | T | 0.0253 | 0.3866 | 0.07943 | 1.18E-06 |
| ebi-a-GCST90001932 | rs138540869 | 5 | 62602530 | T | 0.0276 | 0.3883 | 0.0843 | 4.28E-06 |
| ebi-a-GCST90001410 | rs138542577 | 8 | 16074805 | C | 0.0376 | 0.3227 | 0.06331 | 3.64E-07 |
| ebi-a-GCST90001404 | rs138542577 | 8 | 16074805 | C | 0.0376 | 0.3534 | 0.06406 | 3.71E-08 |
| ebi-a-GCST90001905 | rs138552617 | 19 | 47321626 | T | 0.1037 | -0.2153 | 0.04558 | 2.43E-06 |
| ebi-a-GCST90001698 | rs138562176 | 16 | 4207228 | A | 0.0401 | 47.62 | 9.034 | 1.44E-07 |
| ebi-a-GCST90001948 | rs138564050 | 18 | 2873692 | G | 0.1209 | -0.2668 | 0.05482 | 1.25E-06 |
| ebi-a-GCST90001453 | rs138567833 | 4 | 182783833 | A | 0.0895 | -0.2161 | 0.0466 | 3.69E-06 |
| ebi-a-GCST90002045 | rs138593127 | 2 | 126466187 | C | 0.0318 | 0.4898 | 0.1042 | 2.79E-06 |
| ebi-a-GCST90001903 | rs138596085 | 4 | 22730743 | T | 0.0019 | 1.517 | 0.3207 | 2.36E-06 |
| ebi-a-GCST90001819 | rs138612228 | 5 | 27458351 | T | 0.0027 | -1.129 | 0.2416 | 3.09E-06 |
| ebi-a-GCST90001688 | rs138649004 | 7 | 2205884 | A | NA | 8.056 | 1.552 | 2.20E-07 |
| ebi-a-GCST90001688 | rs138653244 | 11 | 47745696 | C | 3.00E-04 | 2.288 | 0.4624 | 7.85E-07 |
| ebi-a-GCST90001835 | rs138669346 | 13 | 24958892 | A | 0.0576 | 0.2867 | 0.05881 | 1.15E-06 |
| ebi-a-GCST90001627 | rs138678763 | 8 | 11817727 | C | 0.005 | -0.8815 | 0.1709 | 2.65E-07 |
| ebi-a-GCST90001628 | rs138678763 | 8 | 11817727 | C | 0.005 | -0.8053 | 0.1649 | 1.08E-06 |
| ebi-a-GCST90001629 | rs138678763 | 8 | 11817727 | C | 0.005 | -0.8001 | 0.1673 | 1.79E-06 |
| ebi-a-GCST90001669 | rs138678985 | 14 | 38052339 | A | 0.0349 | 0.2897 | 0.06184 | 2.92E-06 |
| ebi-a-GCST90001953 | rs138712525 | 19 | 51544665 | A | 0.199 | 0.3426 | 0.04579 | 1.22E-13 |
| ebi-a-GCST90001688 | rs138736730 | 15 | 87138558 | G | 3.00E-04 | 2.587 | 0.526 | 9.13E-07 |
| ebi-a-GCST90001587 | rs138743668 | 5 | 26070685 | A | 0.0076 | -0.6208 | 0.1356 | 4.83E-06 |
| ebi-a-GCST90001487 | rs138751156 | 1 | 77300547 | C | 0.0546 | 0.2723 | 0.05492 | 7.50E-07 |
| ebi-a-GCST90001697 | rs138754438 | 1 | 6329089 | T | 0.0023 | 6.421 | 1.291 | 6.91E-07 |
| ebi-a-GCST90001697 | rs138758802 | 1 | 91894308 | T | 0.0016 | 7.798 | 1.543 | 4.56E-07 |
| ebi-a-GCST90001700 | rs138765259 | 17 | 47337653 | G | 6.00E-04 | 15.44 | 2.921 | 1.33E-07 |
| ebi-a-GCST90001700 | rs138766897 | 18 | 7853372 | T | 0.0058 | 4.991 | 0.9808 | 3.81E-07 |
| ebi-a-GCST90001695 | rs138799468 | 2 | 9441279 | T | 1.00E-04 | 791.9 | 98.43 | 1.17E-15 |
| ebi-a-GCST90001698 | rs138800755 | 5 | 77962505 | C | 0.0078 | 99.32 | 19.64 | 4.51E-07 |
| ebi-a-GCST90001660 | rs138807420 | 5 | 6231929 | T | 0.0034 | 1.022 | 0.204 | 5.82E-07 |
| ebi-a-GCST90001483 | rs138807420 | 5 | 6231929 | T | 0.0034 | 1.024 | 0.2041 | 5.49E-07 |
| ebi-a-GCST90002054 | rs138809534 | 2 | 11541120 | T | 0.0108 | -0.6447 | 0.1321 | 1.11E-06 |
| ebi-a-GCST90001698 | rs138822361 | 1 | 5236853 | A | 0.0034 | 200.7 | 32.7 | 9.35E-10 |
| ebi-a-GCST90001695 | rs138829583 | 1 | 79094990 | A | 4.00E-04 | 463.8 | 58.31 | 2.46E-15 |
| ebi-a-GCST90001521 | rs138834110 | 8 | 5338183 | A | 0.0032 | 1.402 | 0.294 | 1.98E-06 |
| ebi-a-GCST90001812 | rs138847726 | 4 | 13020910 | T | 0.0252 | -0.3653 | 0.07825 | 3.14E-06 |
| ebi-a-GCST90001698 | rs138871895 | 3 | 162179186 | C | 6.00E-04 | 570.9 | 70.25 | 6.12E-16 |
| ebi-a-GCST90001700 | rs138901088 | 9 | 2524656 | A | 9.00E-04 | 12.28 | 2.651 | 3.74E-06 |
| ebi-a-GCST90001698 | rs138905087 | 1 | 87136899 | G | 0.0092 | 90.64 | 18.38 | 8.54E-07 |
| ebi-a-GCST90001925 | rs138908372 | 4 | 173906273 | C | 0.036 | 0.3399 | 0.07075 | 1.63E-06 |
| ebi-a-GCST90001502 | rs138915779 | 1 | 190079170 | A | 0.0035 | -1.784 | 0.2042 | 3.70E-18 |
| ebi-a-GCST90001503 | rs138915779 | 1 | 190079170 | A | 0.0035 | -1.565 | 0.2272 | 6.75E-12 |
| ebi-a-GCST90001501 | rs138915779 | 1 | 190079170 | A | 0.0034 | -1.518 | 0.2369 | 1.67E-10 |
| ebi-a-GCST90001494 | rs138915779 | 1 | 190079170 | A | 0.0035 | -1.514 | 0.2286 | 4.11E-11 |
| ebi-a-GCST90001492 | rs138915779 | 1 | 190079170 | A | 0.0034 | -1.455 | 0.2371 | 9.40E-10 |
| ebi-a-GCST90001487 | rs138915779 | 1 | 190079170 | A | 0.0035 | -1.347 | 0.2241 | 2.06E-09 |
| ebi-a-GCST90001486 | rs138915779 | 1 | 190079170 | A | 0.0034 | -1.288 | 0.2345 | 4.23E-08 |
| ebi-a-GCST90001669 | rs138915779 | 1 | 190079170 | A | 0.0034 | -1.228 | 0.2078 | 3.74E-09 |
| ebi-a-GCST90001693 | rs138915779 | 1 | 190079170 | A | 0.0034 | -1.182 | 0.2347 | 5.02E-07 |
| ebi-a-GCST90001510 | rs138915779 | 1 | 190079170 | A | 0.0034 | -1.153 | 0.2379 | 1.31E-06 |
| ebi-a-GCST90001488 | rs138915779 | 1 | 190079170 | A | 0.0035 | -1.152 | 0.2291 | 5.25E-07 |
| ebi-a-GCST90001668 | rs138915779 | 1 | 190079170 | A | 0.0035 | -1.146 | 0.227 | 4.68E-07 |
| ebi-a-GCST90001692 | rs138915779 | 1 | 190079170 | A | 0.0035 | -1.138 | 0.2303 | 8.12E-07 |
| ebi-a-GCST90001483 | rs138915779 | 1 | 190079170 | A | 0.0034 | 1.188 | 0.2275 | 1.89E-07 |
| ebi-a-GCST90002098 | rs138915779 | 1 | 190079170 | A | 0.0036 | 1.33 | 0.2281 | 6.19E-09 |
| ebi-a-GCST90001482 | rs138915779 | 1 | 190079170 | A | 0.0035 | 1.439 | 0.2131 | 1.72E-11 |
| ebi-a-GCST90002102 | rs138915779 | 1 | 190079170 | A | 0.0033 | 1.586 | 0.2396 | 4.28E-11 |
| ebi-a-GCST90001481 | rs138915779 | 1 | 190079170 | A | 0.0035 | 1.793 | 0.2061 | 4.93E-18 |
| ebi-a-GCST90001391 | rs138919897 | 3 | 106834795 | G | 0.0019 | 1.402 | 0.2997 | 2.99E-06 |
| ebi-a-GCST90001735 | rs138920008 | 16 | 32422808 | C | 0.0041 | -1.514 | 0.2157 | 2.63E-12 |
| ebi-a-GCST90001723 | rs138920008 | 16 | 32422808 | C | 0.0041 | -1.443 | 0.2168 | 3.15E-11 |
| ebi-a-GCST90001729 | rs138920008 | 16 | 32422808 | C | 0.0041 | -1.438 | 0.2195 | 6.50E-11 |
| ebi-a-GCST90001724 | rs138920008 | 16 | 32422808 | C | 0.0041 | -1.326 | 0.2178 | 1.26E-09 |
| ebi-a-GCST90001742 | rs138920008 | 16 | 32422808 | C | 0.0041 | -1.3 | 0.2194 | 3.41E-09 |
| ebi-a-GCST90001738 | rs138920008 | 16 | 32422808 | C | 0.0041 | -1.283 | 0.2175 | 3.96E-09 |
| ebi-a-GCST90001736 | rs138920008 | 16 | 32422808 | C | 0.0041 | -1.277 | 0.2176 | 4.85E-09 |
| ebi-a-GCST90001730 | rs138920008 | 16 | 32422808 | C | 0.0041 | -1.234 | 0.2174 | 1.50E-08 |
| ebi-a-GCST90001725 | rs138920008 | 16 | 32422808 | C | 0.0041 | -1.188 | 0.2168 | 4.50E-08 |
| ebi-a-GCST90001732 | rs138920008 | 16 | 32422808 | C | 0.0041 | -1.105 | 0.2187 | 4.54E-07 |
| ebi-a-GCST90001741 | rs138920008 | 16 | 32422808 | C | 0.0041 | -1.092 | 0.2174 | 5.41E-07 |
| ebi-a-GCST90001737 | rs138920008 | 16 | 32422808 | C | 0.0041 | -1.027 | 0.2171 | 2.30E-06 |
| ebi-a-GCST90001789 | rs138920863 | 13 | 85642896 | C | 0.0458 | -0.2799 | 0.05936 | 2.51E-06 |
| ebi-a-GCST90001700 | rs138937927 | 16 | 51095561 | T | 3.00E-04 | 23.94 | 3.906 | 9.81E-10 |
| ebi-a-GCST90001698 | rs138937927 | 16 | 51095561 | T | 3.00E-04 | 842.4 | 89.95 | 1.34E-20 |
| ebi-a-GCST90001698 | rs138943622 | 4 | 114293931 | A | 0.0015 | 257.5 | 44.78 | 9.76E-09 |
| ebi-a-GCST90001698 | rs138948609 | 10 | 132596864 | A | 0.0318 | 59.98 | 10.31 | 6.47E-09 |
| ebi-a-GCST90001698 | rs138954494 | 4 | 32184694 | C | 4.00E-04 | 836.1 | 73.46 | 1.75E-29 |
| ebi-a-GCST90001477 | rs138955284 | 3 | 154844527 | T | 0.0047 | -0.9382 | 0.1848 | 4.03E-07 |
| ebi-a-GCST90001494 | rs138960714 | 4 | 123422719 | C | 6.00E-04 | -2.942 | 0.5018 | 4.97E-09 |
| ebi-a-GCST90001503 | rs138960714 | 4 | 123422719 | C | 6.00E-04 | -2.923 | 0.4989 | 5.13E-09 |
| ebi-a-GCST90001492 | rs138960714 | 4 | 123422719 | C | 6.00E-04 | -2.391 | 0.5194 | 4.30E-06 |
| ebi-a-GCST90001501 | rs138960714 | 4 | 123422719 | C | 6.00E-04 | -2.389 | 0.5191 | 4.36E-06 |
| ebi-a-GCST90001502 | rs138960714 | 4 | 123422719 | C | 6.00E-04 | -2.124 | 0.451 | 2.59E-06 |
| ebi-a-GCST90001666 | rs138960714 | 4 | 123422719 | C | 6.00E-04 | 2.077 | 0.4504 | 4.13E-06 |
| ebi-a-GCST90001695 | rs138962348 | 2 | 146534594 | C | 7.00E-04 | 259.1 | 45.06 | 9.70E-09 |
| ebi-a-GCST90001692 | rs138969403 | 4 | 88757559 | T | 0.0564 | -0.2553 | 0.05455 | 2.97E-06 |
| ebi-a-GCST90001887 | rs139007177 | 4 | 83560688 | A | 0.0012 | 2.034 | 0.4344 | 2.97E-06 |
| ebi-a-GCST90001998 | rs139016775 | 5 | 172936151 | C | 8.00E-04 | 2.04 | 0.4279 | 1.94E-06 |
| ebi-a-GCST90002000 | rs139016879 | 4 | 77737913 | C | 0.0096 | 0.5921 | 0.1254 | 2.42E-06 |
| ebi-a-GCST90001697 | rs139018186 | 11 | 10046307 | T | 0.0098 | 2.932 | 0.6132 | 1.81E-06 |
| ebi-a-GCST90001579 | rs139025334 | 20 | 48944963 | A | 0.0663 | 0.2343 | 0.04966 | 2.46E-06 |
| ebi-a-GCST90001822 | rs139047 | 22 | 44323074 | A | 0.3587 | -0.1187 | 0.02594 | 4.93E-06 |
| ebi-a-GCST90001512 | rs139048853 | 4 | 69570859 | C | 0.0646 | -0.2433 | 0.05164 | 2.56E-06 |
| ebi-a-GCST90001890 | rs139057975 | 18 | 10278252 | T | 0.0291 | 0.3785 | 0.08199 | 4.08E-06 |
| ebi-a-GCST90001844 | rs139058742 | 9 | 2784214 | G | 0.0024 | 1.352 | 0.2774 | 1.14E-06 |
| ebi-a-GCST90001698 | rs139075636 | 12 | 102082782 | G | 0.001 | 516.8 | 57.96 | 7.67E-19 |
| ebi-a-GCST90001409 | rs139077655 | 2 | 132741527 | T | 0.382 | -0.1159 | 0.02519 | 4.37E-06 |
| ebi-a-GCST90001820 | rs139084170 | 6 | 43351686 | A | 0.052 | 0.3593 | 0.05776 | 5.51E-10 |
| ebi-a-GCST90001826 | rs139084170 | 6 | 43351686 | A | 0.0523 | 0.3794 | 0.05758 | 5.09E-11 |
| ebi-a-GCST90001633 | rs139090356 | 2 | 7451357 | G | 0.0038 | 0.9023 | 0.1958 | 4.18E-06 |
| ebi-a-GCST90001663 | rs139090356 | 2 | 7451357 | G | 0.004 | 0.9354 | 0.2026 | 4.03E-06 |
| ebi-a-GCST90001751 | rs139106 | 22 | 44569390 | A | 0.1043 | -0.194 | 0.0397 | 1.07E-06 |
| ebi-a-GCST90001698 | rs139120487 | 16 | 755030 | A | 0.0045 | 143 | 29.16 | 9.77E-07 |
| ebi-a-GCST90001926 | rs139132726 | 17 | 55643849 | T | 0.014 | -0.659 | 0.1144 | 9.40E-09 |
| ebi-a-GCST90001486 | rs139138505 | 1 | 225667295 | G | 0.0085 | -0.6234 | 0.1355 | 4.38E-06 |
| ebi-a-GCST90001992 | rs139144024 | 2 | 88635490 | A | 0.0181 | 0.4309 | 0.08993 | 1.72E-06 |
| ebi-a-GCST90001698 | rs139145617 | 9 | 138652160 | T | 0.0025 | 177.5 | 35.46 | 5.80E-07 |
| ebi-a-GCST90001768 | rs139160176 | 5 | 13902245 | C | 0.0034 | -0.9719 | 0.2023 | 1.61E-06 |
| ebi-a-GCST90002117 | rs139174173 | 6 | 32592834 | A | 0.0459 | 0.4977 | 0.07912 | 3.62E-10 |
| ebi-a-GCST90002061 | rs139181081 | 8 | 96559364 | G | 0.1067 | -0.2127 | 0.04543 | 2.97E-06 |
| ebi-a-GCST90001477 | rs139181645 | 17 | 76469857 | G | 0.0451 | -0.2801 | 0.05887 | 2.03E-06 |
| ebi-a-GCST90001479 | rs139191636 | 12 | 23591661 | A | 0.0634 | -0.2479 | 0.05372 | 4.06E-06 |
| ebi-a-GCST90001470 | rs1391986 | 5 | 52071311 | T | 0.0061 | 0.7317 | 0.1561 | 2.87E-06 |
| ebi-a-GCST90001681 | rs139199585 | 3 | 189800760 | G | 0.0613 | -0.2529 | 0.05444 | 3.53E-06 |
| ebi-a-GCST90001679 | rs139199585 | 3 | 189800760 | G | 0.061 | -0.2504 | 0.05435 | 4.24E-06 |
| ebi-a-GCST90001695 | rs139243374 | 12 | 21581956 | A | 9.00E-04 | 221.6 | 42.71 | 2.26E-07 |
| ebi-a-GCST90001791 | rs139257812 | 1 | 8194962 | C | 0.0228 | 0.3821 | 0.08268 | 3.94E-06 |
| ebi-a-GCST90002014 | rs139258218 | 1 | 118620702 | C | 0.0275 | -0.4071 | 0.08609 | 2.37E-06 |
| ebi-a-GCST90001527 | rs139262012 | 5 | 99798403 | C | 0.0124 | 0.702 | 0.1468 | 1.87E-06 |
| ebi-a-GCST90001992 | rs139288206 | 17 | 14094524 | T | 0.0182 | 0.4423 | 0.09125 | 1.31E-06 |
| ebi-a-GCST90001707 | rs139290596 | 22 | 44349795 | G | 0.0482 | -0.4589 | 0.07926 | 8.25E-09 |
| ebi-a-GCST90002024 | rs139306421 | 6 | 109298437 | G | 0.0234 | -0.5447 | 0.09175 | 3.24E-09 |
| ebi-a-GCST90001616 | rs139311612 | 6 | 79117697 | C | 0.0329 | -0.3592 | 0.07241 | 7.37E-07 |
| ebi-a-GCST90001617 | rs139311612 | 6 | 79117697 | C | 0.0329 | -0.3562 | 0.07213 | 8.23E-07 |
| ebi-a-GCST90001615 | rs139311612 | 6 | 79117697 | C | 0.0329 | -0.3512 | 0.07076 | 7.23E-07 |
| ebi-a-GCST90002096 | rs139324834 | 1 | 161588466 | T | 0.0408 | 0.4813 | 0.09806 | 1.02E-06 |
| ebi-a-GCST90001700 | rs139329941 | 3 | 166969119 | T | 0.0047 | 5.185 | 1.061 | 1.07E-06 |
| ebi-a-GCST90001699 | rs139329941 | 3 | 166969119 | T | 0.0047 | 11.48 | 2.466 | 3.37E-06 |
| ebi-a-GCST90002053 | rs139335095 | 11 | 62711027 | T | 0.0506 | -0.3912 | 0.08404 | 3.50E-06 |
| ebi-a-GCST90001523 | rs139348198 | 11 | 14699945 | T | 0.0113 | -0.7561 | 0.16 | 2.46E-06 |
| ebi-a-GCST90001836 | rs139348851 | 3 | 50280843 | G | 0.0413 | 0.4539 | 0.09449 | 1.72E-06 |
| ebi-a-GCST90001821 | rs139357193 | 3 | 51971998 | A | 0.0059 | -0.8453 | 0.1669 | 4.26E-07 |
| ebi-a-GCST90001587 | rs139379948 | 7 | 103732660 | A | 0.0116 | -0.5341 | 0.115 | 3.54E-06 |
| ebi-a-GCST90001586 | rs139379948 | 7 | 103732660 | A | 0.0116 | 0.5387 | 0.115 | 2.89E-06 |
| ebi-a-GCST90001904 | rs139387290 | 7 | 145251741 | A | 0.0145 | -0.5368 | 0.1156 | 3.56E-06 |
| ebi-a-GCST90001526 | rs139393711 | 3 | 113287778 | G | 0.0355 | -0.4467 | 0.09624 | 3.70E-06 |
| ebi-a-GCST90001698 | rs139404430 | 2 | 7430709 | T | 0.0047 | 141.4 | 26.91 | 1.58E-07 |
| ebi-a-GCST90002018 | rs139407311 | 1 | 182527138 | T | 9.00E-04 | -2.122 | 0.4452 | 1.97E-06 |
| ebi-a-GCST90001986 | rs139408196 | 12 | 40575173 | T | 0.0864 | 0.2354 | 0.04457 | 1.36E-07 |
| ebi-a-GCST90001697 | rs139422413 | 9 | 9765228 | T | 0.0042 | 4.342 | 0.9345 | 3.49E-06 |
| ebi-a-GCST90001698 | rs139445618 | 10 | 24055451 | T | 0.0169 | 62.93 | 13.44 | 2.95E-06 |
| ebi-a-GCST90001697 | rs139457426 | 12 | 47679643 | T | 0.0147 | 2.408 | 0.5131 | 2.81E-06 |
| ebi-a-GCST90001695 | rs139457426 | 12 | 47679643 | T | 0.0147 | 71.36 | 10.98 | 9.24E-11 |
| ebi-a-GCST90001870 | rs139489968 | 1 | 208017826 | A | 0.1016 | 0.5509 | 0.05917 | 3.99E-20 |
| ebi-a-GCST90001775 | rs139491278 | 17 | 34986043 | T | 0.0153 | -0.4571 | 0.09874 | 3.80E-06 |
| ebi-a-GCST90001487 | rs139502175 | 8 | 131952722 | C | 0.0335 | -0.3153 | 0.06883 | 4.78E-06 |
| ebi-a-GCST90001697 | rs139513469 | 10 | 66332109 | A | 6.00E-04 | 15.24 | 2.947 | 2.47E-07 |
| ebi-a-GCST90001695 | rs139513469 | 10 | 66332109 | A | 6.00E-04 | 295.5 | 63.49 | 3.38E-06 |
| ebi-a-GCST90002071 | rs139527316 | 10 | 104987693 | T | 0.0106 | 0.617 | 0.1311 | 2.63E-06 |
| ebi-a-GCST90002000 | rs139529340 | 1 | 161563554 | G | 0.2371 | 0.1503 | 0.02834 | 1.20E-07 |
| ebi-a-GCST90002071 | rs139529340 | 1 | 161563554 | G | 0.2308 | 0.2016 | 0.03334 | 1.68E-09 |
| ebi-a-GCST90001454 | rs139554006 | 14 | 53831558 | A | 0.0255 | 0.3934 | 0.08287 | 2.17E-06 |
| ebi-a-GCST90001466 | rs139557155 | 9 | 77583476 | G | 0.0578 | 0.2575 | 0.05462 | 2.52E-06 |
| ebi-a-GCST90001467 | rs139557155 | 9 | 77583476 | G | 0.0578 | 0.277 | 0.05451 | 3.93E-07 |
| ebi-a-GCST90001660 | rs139577399 | 7 | 23203415 | C | 0.0414 | -0.3243 | 0.06424 | 4.69E-07 |
| ebi-a-GCST90001489 | rs139577399 | 7 | 23203415 | C | 0.0414 | -0.3155 | 0.06457 | 1.08E-06 |
| ebi-a-GCST90002045 | rs139602776 | 4 | 103564713 | C | 0.0119 | 0.7852 | 0.166 | 2.43E-06 |
| ebi-a-GCST90001695 | rs139613266 | 6 | 101930392 | G | 6.00E-04 | 242.6 | 49.52 | 1.01E-06 |
| ebi-a-GCST90001870 | rs139622960 | 11 | 66287586 | A | 0.1371 | -0.2453 | 0.05171 | 2.28E-06 |
| ebi-a-GCST90001873 | rs139631207 | 3 | 136490521 | A | 0.0225 | 0.6468 | 0.1371 | 2.64E-06 |
| ebi-a-GCST90002049 | rs139648605 | 2 | 59488592 | G | 0.0682 | 0.3404 | 0.07249 | 2.88E-06 |
| ebi-a-GCST90001481 | rs139681117 | 2 | 23924920 | T | 0.0528 | 0.2445 | 0.05221 | 2.94E-06 |
| ebi-a-GCST90001782 | rs139703496 | 10 | 1676361 | T | 0.0016 | 1.904 | 0.4114 | 3.96E-06 |
| ebi-a-GCST90001979 | rs139731096 | 9 | 140753656 | C | 0.414 | 0.122 | 0.02514 | 1.25E-06 |
| ebi-a-GCST90001698 | rs139736375 | 17 | 67625638 | T | 0.001 | 443.1 | 51.97 | 2.25E-17 |
| ebi-a-GCST90001697 | rs139772031 | 3 | 10035985 | G | 1.00E-04 | 21.84 | 4.516 | 1.39E-06 |
| ebi-a-GCST90001695 | rs139772031 | 3 | 10035985 | G | 1.00E-04 | 550.9 | 98.17 | 2.17E-08 |
| ebi-a-GCST90001734 | rs139775632 | 7 | 6917201 | G | 0.009 | -0.651 | 0.1299 | 5.63E-07 |
| ebi-a-GCST90001698 | rs139781702 | 10 | 38359540 | G | 0.0079 | 103.8 | 22.24 | 3.14E-06 |
| ebi-a-GCST90001713 | rs139784925 | 22 | 41079533 | T | 0.0571 | -0.2552 | 0.05474 | 3.23E-06 |
| ebi-a-GCST90001789 | rs1397934 | 4 | 40187596 | G | 0.7003 | -0.1378 | 0.02604 | 1.27E-07 |
| ebi-a-GCST90002039 | rs139795227 | 1 | 92842367 | C | 0.0481 | -1.147 | 0.06195 | 2.34E-72 |
| ebi-a-GCST90002036 | rs139795227 | 1 | 92842367 | C | 0.0479 | -0.5867 | 0.06432 | 1.36E-19 |
| ebi-a-GCST90002035 | rs139795227 | 1 | 92842367 | C | 0.0479 | -0.5725 | 0.0642 | 8.24E-19 |
| ebi-a-GCST90001580 | rs139795227 | 1 | 92842367 | C | 0.0514 | 0.3162 | 0.05876 | 7.85E-08 |
| ebi-a-GCST90001884 | rs139795227 | 1 | 92842367 | C | 0.0496 | 0.3166 | 0.06357 | 6.71E-07 |
| ebi-a-GCST90001698 | rs139800400 | 5 | 134343662 | A | 0.0016 | 200.6 | 42.25 | 2.14E-06 |
| ebi-a-GCST90001698 | rs139819048 | 4 | 67760555 | C | 0.0045 | 143.3 | 26.23 | 5.05E-08 |
| ebi-a-GCST90001873 | rs139827714 | 4 | 47461569 | G | 0.0164 | 0.7533 | 0.1585 | 2.24E-06 |
| ebi-a-GCST90001925 | rs139832224 | 9 | 72035000 | T | 0.0252 | -0.3901 | 0.08384 | 3.43E-06 |
| ebi-a-GCST90001812 | rs139833260 | 7 | 119003006 | A | 0.0256 | 0.3626 | 0.07883 | 4.38E-06 |
| ebi-a-GCST90001981 | rs139839882 | 15 | 30291728 | T | 0.046 | 0.3046 | 0.06158 | 7.89E-07 |
| ebi-a-GCST90001410 | rs139843122 | 6 | 107939307 | T | 0.0123 | -0.5032 | 0.1082 | 3.40E-06 |
| ebi-a-GCST90001951 | rs139845236 | 3 | 187673519 | A | 0.0058 | 1.224 | 0.247 | 8.02E-07 |
| ebi-a-GCST90002033 | rs139849385 | 16 | 4693648 | T | 0.0271 | -0.4654 | 0.0869 | 9.19E-08 |
| ebi-a-GCST90001697 | rs139859886 | 1 | 98055569 | G | 0.0012 | 8.253 | 1.737 | 2.11E-06 |
| ebi-a-GCST90001947 | rs139876813 | 17 | 2689271 | T | 0.1078 | -0.2854 | 0.05815 | 1.01E-06 |
| ebi-a-GCST90001949 | rs139876813 | 17 | 2689271 | T | 0.1092 | -0.2801 | 0.05585 | 5.84E-07 |
| ebi-a-GCST90001956 | rs139876813 | 17 | 2689271 | T | 0.108 | -0.2794 | 0.05794 | 1.56E-06 |
| ebi-a-GCST90001951 | rs139876813 | 17 | 2689271 | T | 0.1096 | -0.2788 | 0.06027 | 4.07E-06 |
| ebi-a-GCST90001957 | rs139876813 | 17 | 2689271 | T | 0.1074 | -0.2749 | 0.0582 | 2.53E-06 |
| ebi-a-GCST90001946 | rs139876813 | 17 | 2689271 | T | 0.1092 | -0.2732 | 0.05633 | 1.35E-06 |
| ebi-a-GCST90001948 | rs139876813 | 17 | 2689271 | T | 0.1093 | -0.2682 | 0.05603 | 1.85E-06 |
| ebi-a-GCST90001695 | rs139889394 | 1 | 41963906 | C | 3.00E-04 | 378 | 77.39 | 1.09E-06 |
| ebi-a-GCST90001698 | rs139898536 | 12 | 109036912 | T | 0.0043 | 161.3 | 28.25 | 1.23E-08 |
| ebi-a-GCST90001489 | rs139917391 | 16 | 18152860 | T | 4.00E-04 | 2.666 | 0.5711 | 3.16E-06 |
| ebi-a-GCST90001465 | rs139939515 | 1 | 163958750 | T | 0.0123 | -0.6222 | 0.1176 | 1.30E-07 |
| ebi-a-GCST90001464 | rs139939515 | 1 | 163958750 | T | 0.0124 | -0.5561 | 0.1174 | 2.28E-06 |
| ebi-a-GCST90002071 | rs139947059 | 12 | 25434182 | A | 0.0266 | 0.4676 | 0.08405 | 2.89E-08 |
| ebi-a-GCST90001472 | rs139956891 | 10 | 127629826 | G | 0.0616 | 0.2686 | 0.05451 | 8.75E-07 |
| ebi-a-GCST90001412 | rs139981802 | 13 | 108488351 | C | 0.0418 | 0.3005 | 0.05978 | 5.23E-07 |
| ebi-a-GCST90001447 | rs139996875 | 5 | 78010341 | T | 0.0063 | 0.6715 | 0.1465 | 4.69E-06 |
| ebi-a-GCST90001863 | rs139997835 | 2 | 220485977 | T | 0.0288 | -0.3728 | 0.08122 | 4.63E-06 |
| ebi-a-GCST90001992 | rs140019375 | 19 | 54863920 | T | 0.0014 | 1.63 | 0.3309 | 8.75E-07 |
| ebi-a-GCST90001864 | rs140019589 | 13 | 62183054 | T | 0.0075 | 0.7678 | 0.1581 | 1.27E-06 |
| ebi-a-GCST90001698 | rs140053174 | 12 | 71043237 | A | 0.0059 | 122 | 24.22 | 5.00E-07 |
| ebi-a-GCST90001688 | rs140064908 | 2 | 141593935 | T | 1.00E-04 | 5.454 | 1.049 | 2.12E-07 |
| ebi-a-GCST90001670 | rs140079267 | 10 | 93272233 | G | 0.023 | -0.4394 | 0.08731 | 5.08E-07 |
| ebi-a-GCST90001672 | rs140079267 | 10 | 93272233 | G | 0.0226 | -0.4226 | 0.08976 | 2.59E-06 |
| ebi-a-GCST90002062 | rs140084476 | 8 | 24184495 | A | 0.0072 | 0.7885 | 0.1681 | 2.85E-06 |
| ebi-a-GCST90001578 | rs140088532 | 2 | 105581937 | C | 0.0368 | -0.3216 | 0.06383 | 4.95E-07 |
| ebi-a-GCST90001577 | rs140088532 | 2 | 105581937 | C | 0.0368 | -0.3106 | 0.0634 | 1.00E-06 |
| ebi-a-GCST90001467 | rs140090913 | 8 | 18584959 | A | 0.0028 | -1.154 | 0.2432 | 2.17E-06 |
| ebi-a-GCST90001738 | rs140108418 | 2 | 13573112 | G | 8.00E-04 | 1.97 | 0.4166 | 2.34E-06 |
| ebi-a-GCST90001724 | rs140108418 | 2 | 13573112 | G | 8.00E-04 | 2.034 | 0.4175 | 1.15E-06 |
| ebi-a-GCST90001728 | rs140108418 | 2 | 13573112 | G | 0.0014 | 2.132 | 0.4562 | 3.16E-06 |
| ebi-a-GCST90001697 | rs140112233 | 7 | 115835641 | C | 1.00E-04 | 43.28 | 6.761 | 1.74E-10 |
| ebi-a-GCST90001695 | rs140112233 | 7 | 115835641 | C | 1.00E-04 | 887.7 | 146 | 1.33E-09 |
| ebi-a-GCST90001541 | rs140125890 | 4 | 40884597 | C | 0.0071 | 0.6384 | 0.132 | 1.39E-06 |
| ebi-a-GCST90001575 | rs140125890 | 4 | 40884597 | C | 0.0071 | 0.6573 | 0.1386 | 2.20E-06 |
| ebi-a-GCST90002097 | rs140129370 | 13 | 74097996 | C | 0.0114 | -0.8413 | 0.1689 | 7.04E-07 |
| ebi-a-GCST90001695 | rs140134394 | 16 | 17341866 | C | 0.0028 | 151.8 | 24.76 | 9.76E-10 |
| ebi-a-GCST90001419 | rs140134875 | 15 | 80306120 | A | 0.0011 | -1.694 | 0.3528 | 1.64E-06 |
| ebi-a-GCST90002060 | rs1401480 | 8 | 4117811 | C | 0.0449 | -0.3063 | 0.06492 | 2.49E-06 |
| ebi-a-GCST90001854 | rs140156664 | 7 | 70488258 | T | 0.0454 | -0.3233 | 0.06515 | 7.37E-07 |
| ebi-a-GCST90001698 | rs140173523 | 2 | 195788562 | A | 0.0026 | 199.4 | 34.68 | 9.74E-09 |
| ebi-a-GCST90001546 | rs140178705 | 18 | 5785815 | A | 0.0048 | -0.8285 | 0.1802 | 4.44E-06 |
| ebi-a-GCST90001402 | rs140191306 | 9 | 2570753 | T | 0.0179 | -0.4624 | 0.09477 | 1.11E-06 |
| ebi-a-GCST90001445 | rs140191306 | 9 | 2570753 | T | 0.0179 | -0.4466 | 0.09399 | 2.10E-06 |
| ebi-a-GCST90001415 | rs140191306 | 9 | 2570753 | T | 0.0179 | 0.4404 | 0.09513 | 3.80E-06 |
| ebi-a-GCST90001391 | rs140191306 | 9 | 2570753 | T | 0.0179 | 0.4421 | 0.09364 | 2.43E-06 |
| ebi-a-GCST90001437 | rs140197720 | 7 | 108191234 | T | 0.0153 | 0.4648 | 0.1016 | 4.94E-06 |
| ebi-a-GCST90001464 | rs140205436 | 15 | 25969797 | T | 6.00E-04 | -2.409 | 0.5106 | 2.48E-06 |
| ebi-a-GCST90001998 | rs1402451 | 3 | 132795714 | A | 0.0746 | 0.2326 | 0.0466 | 6.29E-07 |
| ebi-a-GCST90002003 | rs140264318 | 19 | 29692512 | C | 0.0048 | -0.91 | 0.1835 | 7.42E-07 |
| ebi-a-GCST90001695 | rs140280232 | 21 | 46387655 | T | 0.0013 | 174.4 | 34.67 | 5.20E-07 |
| ebi-a-GCST90001874 | rs140287246 | 15 | 96917459 | T | 0.0148 | -0.7782 | 0.1674 | 3.71E-06 |
| ebi-a-GCST90001695 | rs140311178 | 4 | 8174723 | T | 0.0037 | 142.3 | 22.5 | 2.87E-10 |
| ebi-a-GCST90001774 | rs140315354 | 1 | 161617673 | A | 0.1355 | 0.1704 | 0.03602 | 2.32E-06 |
| ebi-a-GCST90001698 | rs140322068 | 10 | 1765793 | A | 0.0032 | 204.4 | 36.08 | 1.58E-08 |
| ebi-a-GCST90001987 | rs140329252 | 4 | 131026425 | G | 0.0377 | -0.3077 | 0.06533 | 2.57E-06 |
| ebi-a-GCST90001698 | rs140367496 | 4 | 131786765 | C | 0.0012 | 460.1 | 50.43 | 1.21E-19 |
| ebi-a-GCST90001681 | rs1403755 | 18 | 30263492 | T | 0.0864 | 0.2696 | 0.04674 | 8.82E-09 |
| ebi-a-GCST90001985 | rs140379607 | 3 | 4758271 | T | 0.0112 | -0.5209 | 0.1137 | 4.78E-06 |
| ebi-a-GCST90001429 | rs140380950 | 2 | 105587803 | C | 0.0368 | -0.3597 | 0.06402 | 2.07E-08 |
| ebi-a-GCST90001392 | rs140380950 | 2 | 105587803 | C | 0.0368 | -0.3568 | 0.06296 | 1.57E-08 |
| ebi-a-GCST90001447 | rs140380950 | 2 | 105587803 | C | 0.0368 | -0.3401 | 0.06356 | 9.31E-08 |
| ebi-a-GCST90001696 | rs140385926 | 15 | 69903187 | G | 9.00E-04 | 28.75 | 5.206 | 3.59E-08 |
| ebi-a-GCST90001852 | rs140395477 | 13 | 79717709 | G | 0.003 | -1.212 | 0.2512 | 1.47E-06 |
| ebi-a-GCST90001620 | rs140410174 | 19 | 1671156 | T | 0.0598 | -0.2422 | 0.04942 | 9.96E-07 |
| ebi-a-GCST90001619 | rs140410174 | 19 | 1671156 | T | 0.0598 | -0.2381 | 0.04814 | 7.90E-07 |
| ebi-a-GCST90001626 | rs140410174 | 19 | 1671156 | T | 0.0598 | -0.2205 | 0.04729 | 3.24E-06 |
| ebi-a-GCST90001697 | rs140434581 | 1 | 18070672 | A | 0.0042 | 4.653 | 0.9728 | 1.80E-06 |
| ebi-a-GCST90001638 | rs140444563 | 12 | 31103286 | G | 0.1091 | 0.1844 | 0.03996 | 4.09E-06 |
| ebi-a-GCST90001654 | rs140444563 | 12 | 31103286 | G | 0.1084 | 0.1893 | 0.04112 | 4.31E-06 |
| ebi-a-GCST90001819 | rs140447118 | 4 | 24413705 | T | 0.018 | -0.4288 | 0.09163 | 2.98E-06 |
| ebi-a-GCST90001564 | rs140471174 | 1 | 55929294 | T | 0.008 | 0.5755 | 0.1252 | 4.45E-06 |
| ebi-a-GCST90001660 | rs140503524 | 11 | 44646709 | T | 0.0021 | -1.428 | 0.3074 | 3.49E-06 |
| ebi-a-GCST90001695 | rs140517857 | 3 | 70804684 | A | 0.0016 | 179.1 | 36.43 | 9.26E-07 |
| ebi-a-GCST90001441 | rs140523784 | 6 | 16887609 | T | 0.1864 | 0.1447 | 0.03153 | 4.58E-06 |
| ebi-a-GCST90001698 | rs140524161 | 13 | 37301576 | G | 1.00E-04 | 757.5 | 140.6 | 7.55E-08 |
| ebi-a-GCST90001819 | rs140554292 | 5 | 87562189 | A | 0.0145 | 0.4666 | 0.1014 | 4.38E-06 |
| ebi-a-GCST90001523 | rs140563508 | 9 | 140249209 | T | 0.0153 | 0.6759 | 0.1402 | 1.55E-06 |
| ebi-a-GCST90002081 | rs140567398 | 13 | 99878398 | A | 0.0866 | 0.2254 | 0.0474 | 2.08E-06 |
| ebi-a-GCST90002103 | rs1405788 | 3 | 39789191 | C | 0.2945 | 0.1449 | 0.03041 | 2.00E-06 |
| ebi-a-GCST90002077 | rs140589274 | 9 | 138894488 | G | 0.0153 | -0.5433 | 0.1077 | 4.82E-07 |
| ebi-a-GCST90001697 | rs140590797 | 14 | 62568446 | G | 6.00E-04 | 11.92 | 2.463 | 1.37E-06 |
| ebi-a-GCST90001547 | rs140596675 | 4 | 119558596 | A | 0.1094 | -0.1813 | 0.03918 | 3.84E-06 |
| ebi-a-GCST90001833 | rs140597204 | 4 | 43926627 | T | 0.0206 | -0.4709 | 0.09393 | 5.68E-07 |
| ebi-a-GCST90001835 | rs140597204 | 4 | 43926627 | T | 0.0205 | -0.457 | 0.09629 | 2.18E-06 |
| ebi-a-GCST90001919 | rs140611829 | 6 | 14271982 | A | 0.0132 | -0.548 | 0.1173 | 3.14E-06 |
| ebi-a-GCST90001404 | rs140642786 | 5 | 148927495 | A | 0.1223 | 0.1711 | 0.03733 | 4.75E-06 |
| ebi-a-GCST90001505 | rs140655813 | 1 | 163746986 | T | 0.0207 | -0.4015 | 0.08763 | 4.77E-06 |
| ebi-a-GCST90002106 | rs140657974 | 16 | 8010586 | A | 0.0084 | 0.7849 | 0.1496 | 1.67E-07 |
| ebi-a-GCST90001555 | rs14067 | 13 | 114110660 | G | 0.234 | -0.1156 | 0.02505 | 4.10E-06 |
| ebi-a-GCST90001561 | rs14067 | 13 | 114110660 | G | 0.234 | 0.119 | 0.02513 | 2.28E-06 |
| ebi-a-GCST90001695 | rs140677128 | 1 | 229822729 | T | 0.0012 | 180.6 | 39.33 | 4.54E-06 |
| ebi-a-GCST90001780 | rs140678325 | 13 | 42323712 | T | 0.0084 | 0.616 | 0.1308 | 2.56E-06 |
| ebi-a-GCST90001890 | rs140680300 | 14 | 40290794 | A | 0.0805 | 0.233 | 0.05024 | 3.67E-06 |
| ebi-a-GCST90001795 | rs140685335 | 18 | 1584719 | G | 0.0276 | 0.3598 | 0.07544 | 1.92E-06 |
| ebi-a-GCST90001550 | rs140690408 | 16 | 53339004 | T | 0.3637 | -0.1238 | 0.02581 | 1.69E-06 |
| ebi-a-GCST90001698 | rs140702253 | 2 | 194544900 | G | 0.0032 | 165.9 | 34.19 | 1.27E-06 |
| ebi-a-GCST90001926 | rs140712754 | 18 | 45108661 | G | 0.0185 | -0.4958 | 0.1035 | 1.75E-06 |
| ebi-a-GCST90001776 | rs140714148 | 12 | 2859329 | T | 0.0431 | -0.2807 | 0.06109 | 4.46E-06 |
| ebi-a-GCST90001629 | rs1407309 | 9 | 117651780 | A | 0.4179 | -0.1372 | 0.02544 | 7.38E-08 |
| ebi-a-GCST90001628 | rs1407309 | 9 | 117651780 | A | 0.4179 | -0.1339 | 0.02508 | 9.86E-08 |
| ebi-a-GCST90001728 | rs140761841 | 4 | 747378 | T | 0.0065 | 0.9699 | 0.2005 | 1.43E-06 |
| ebi-a-GCST90001750 | rs140761841 | 4 | 747378 | T | 0.0065 | 0.9911 | 0.2025 | 1.07E-06 |
| ebi-a-GCST90001561 | rs140764369 | 5 | 120985438 | A | 0.0463 | 0.2369 | 0.05118 | 3.82E-06 |
| ebi-a-GCST90001698 | rs140778797 | 2 | 130811172 | T | 4.00E-04 | 461.6 | 100.6 | 4.60E-06 |
| ebi-a-GCST90001830 | rs140785480 | 13 | 61781933 | C | 0.0193 | -0.4347 | 0.09328 | 3.28E-06 |
| ebi-a-GCST90001698 | rs140800343 | 19 | 50001170 | T | 0.0211 | 69.9 | 12.06 | 7.48E-09 |
| ebi-a-GCST90002102 | rs140807368 | 1 | 194379933 | T | 0.0053 | 0.827 | 0.1693 | 1.09E-06 |
| ebi-a-GCST90001562 | rs140811183 | 4 | 135317580 | A | 0.0136 | -0.4375 | 0.09313 | 2.73E-06 |
| ebi-a-GCST90001664 | rs140816141 | 22 | 28323098 | A | 4.00E-04 | 2.584 | 0.521 | 7.39E-07 |
| ebi-a-GCST90001926 | rs140819873 | 9 | 116014845 | G | 0.0459 | -0.3274 | 0.06662 | 9.42E-07 |
| ebi-a-GCST90002060 | rs140845172 | 19 | 520519 | C | 0.0026 | 1.301 | 0.26 | 5.88E-07 |
| ebi-a-GCST90001695 | rs140871773 | 17 | 13877125 | G | 0.0012 | 201.6 | 42.28 | 1.95E-06 |
| ebi-a-GCST90002092 | rs140875293 | 2 | 190360243 | A | 0.0092 | 0.9991 | 0.2115 | 2.57E-06 |
| ebi-a-GCST90001832 | rs140893422 | 9 | 131575934 | A | 0.0284 | -0.3623 | 0.0776 | 3.16E-06 |
| ebi-a-GCST90001698 | rs140899856 | 9 | 111201283 | G | 0.0301 | 50.94 | 10.79 | 2.45E-06 |
| ebi-a-GCST90001567 | rs140929446 | 7 | 57131203 | A | 0.0598 | -0.2442 | 0.04996 | 1.07E-06 |
| ebi-a-GCST90001698 | rs140929582 | 2 | 169297185 | T | 0.0026 | 210.6 | 33.76 | 4.98E-10 |
| ebi-a-GCST90001813 | rs1409571 | 13 | 72672465 | A | 0.7778 | -0.1383 | 0.02988 | 3.78E-06 |
| ebi-a-GCST90001513 | rs140960913 | 7 | 158180337 | T | 0.0276 | 0.3563 | 0.07657 | 3.39E-06 |
| ebi-a-GCST90001504 | rs140960913 | 7 | 158180337 | T | 0.0276 | 0.3573 | 0.07789 | 4.66E-06 |
| ebi-a-GCST90001700 | rs140963286 | 1 | 24640467 | T | 1.00E-04 | 33.4 | 5.736 | 6.30E-09 |
| ebi-a-GCST90001695 | rs140963286 | 1 | 24640467 | T | 1.00E-04 | 760.9 | 100.5 | 4.68E-14 |
| ebi-a-GCST90001698 | rs140963286 | 1 | 24640467 | T | 1.00E-04 | 2073 | 128.3 | 1.15E-56 |
| ebi-a-GCST90001979 | rs140966292 | 1 | 175408164 | C | 0.0148 | -0.5466 | 0.1035 | 1.37E-07 |
| ebi-a-GCST90001700 | rs140976870 | 5 | 120420356 | T | 0.0112 | 3.271 | 0.7129 | 4.64E-06 |
| ebi-a-GCST90001923 | rs140978083 | 4 | 114960229 | C | 0.0354 | 0.352 | 0.07528 | 3.06E-06 |
| ebi-a-GCST90002031 | rs141018090 | 10 | 98169221 | T | 0.0517 | 0.3492 | 0.06169 | 1.66E-08 |
| ebi-a-GCST90001491 | rs141018090 | 10 | 98169221 | T | 0.0528 | 0.4456 | 0.05532 | 1.08E-15 |
| ebi-a-GCST90001695 | rs141023610 | 3 | 15790899 | A | 4.00E-04 | 556.4 | 84.21 | 4.51E-11 |
| ebi-a-GCST90001697 | rs141057204 | 11 | 130013797 | G | 9.00E-04 | 13.88 | 2.845 | 1.12E-06 |
| ebi-a-GCST90001695 | rs141057204 | 11 | 130013797 | G | 9.00E-04 | 284.3 | 60.84 | 3.08E-06 |
| ebi-a-GCST90002030 | rs1410600 | 10 | 97070694 | T | 0.4053 | -0.2642 | 0.02755 | 1.82E-21 |
| ebi-a-GCST90001659 | rs1410600 | 10 | 97070694 | T | 0.4068 | -0.2459 | 0.02509 | 2.18E-22 |
| ebi-a-GCST90002031 | rs1410600 | 10 | 97070694 | T | 0.4053 | -0.2442 | 0.02817 | 6.97E-18 |
| ebi-a-GCST90001658 | rs1410600 | 10 | 97070694 | T | 0.4068 | -0.2419 | 0.0251 | 1.06E-21 |
| ebi-a-GCST90001660 | rs1410600 | 10 | 97070694 | T | 0.4073 | -0.2402 | 0.02632 | 1.20E-19 |
| ebi-a-GCST90002032 | rs1410600 | 10 | 97070694 | T | 0.4053 | -0.2329 | 0.02819 | 2.19E-16 |
| ebi-a-GCST90001485 | rs1410600 | 10 | 97070694 | T | 0.4069 | -0.2216 | 0.02536 | 3.59E-18 |
| ebi-a-GCST90001970 | rs141070453 | 15 | 29516794 | G | 0.0284 | -0.3895 | 0.08268 | 2.58E-06 |
| ebi-a-GCST90001525 | rs141080550 | 13 | 111287945 | A | 0.0307 | 0.4746 | 0.09273 | 3.41E-07 |
| ebi-a-GCST90001698 | rs141089930 | 2 | 178028543 | C | 0.0043 | 139.9 | 28.75 | 1.19E-06 |
| ebi-a-GCST90002024 | rs141120003 | 10 | 36139903 | C | 0.0163 | 0.5219 | 0.1121 | 3.34E-06 |
| ebi-a-GCST90001745 | rs141121779 | 19 | 49998328 | A | 0.0647 | 0.2393 | 0.05016 | 1.91E-06 |
| ebi-a-GCST90001981 | rs141130258 | 7 | 101436472 | T | 0.0166 | 0.4455 | 0.09706 | 4.58E-06 |
| ebi-a-GCST90002102 | rs141148183 | 16 | 54755529 | T | 0.0043 | 0.8913 | 0.1947 | 4.89E-06 |
| ebi-a-GCST90001585 | rs141154517 | 4 | 71103396 | A | 0.0331 | -0.3263 | 0.07052 | 3.83E-06 |
| ebi-a-GCST90002119 | rs141200631 | 13 | 94482351 | G | 0.0041 | 0.9591 | 0.2088 | 4.56E-06 |
| ebi-a-GCST90001901 | rs141247369 | 1 | 3272937 | A | 0.0038 | 1.078 | 0.2189 | 9.01E-07 |
| ebi-a-GCST90002039 | rs141264358 | 1 | 90530314 | C | 0.016 | -0.5868 | 0.1067 | 4.18E-08 |
| ebi-a-GCST90001805 | rs141269963 | 1 | 99804475 | G | 0.0105 | 0.549 | 0.1178 | 3.30E-06 |
| ebi-a-GCST90001695 | rs141289216 | 1 | 14539144 | T | 0.0051 | 88.02 | 18.79 | 2.91E-06 |
| ebi-a-GCST90001698 | rs141309221 | 14 | 95018285 | A | 0.0129 | 73.87 | 15.3 | 1.43E-06 |
| ebi-a-GCST90001458 | rs141320646 | 1 | 73622958 | A | 0.005 | 0.8749 | 0.1843 | 2.15E-06 |
| ebi-a-GCST90001688 | rs141341926 | 11 | 119643891 | T | 1.00E-04 | 4.416 | 0.8469 | 1.95E-07 |
| ebi-a-GCST90001535 | rs141350785 | 11 | 5240669 | C | 0.0461 | -0.2543 | 0.0556 | 4.95E-06 |
| ebi-a-GCST90001438 | rs141352577 | 3 | 8492010 | T | 0.0235 | 0.3656 | 0.07671 | 1.96E-06 |
| ebi-a-GCST90001688 | rs141357353 | 1 | 58442574 | A | 4.00E-04 | 1.943 | 0.4052 | 1.71E-06 |
| ebi-a-GCST90001773 | rs141357554 | 10 | 74099495 | A | 0.0094 | -0.6039 | 0.1313 | 4.35E-06 |
| ebi-a-GCST90001435 | rs141365416 | 1 | 102236814 | T | 5.00E-04 | 2.328 | 0.4961 | 2.79E-06 |
| ebi-a-GCST90001814 | rs141365416 | 1 | 102236814 | T | 5.00E-04 | 2.383 | 0.5056 | 2.53E-06 |
| ebi-a-GCST90001698 | rs141389929 | 6 | 142716026 | T | 0.0063 | 111.9 | 22.59 | 7.74E-07 |
| ebi-a-GCST90002004 | rs141411805 | 3 | 53544015 | G | 0.0146 | -0.5993 | 0.1027 | 5.80E-09 |
| ebi-a-GCST90002008 | rs141411805 | 3 | 53544015 | G | 0.0146 | -0.5601 | 0.1038 | 7.20E-08 |
| ebi-a-GCST90001917 | rs141418004 | 18 | 49028375 | A | 0.0106 | -0.5347 | 0.1167 | 4.80E-06 |
| ebi-a-GCST90001851 | rs141421236 | 16 | 4780201 | C | 0.0087 | -0.7094 | 0.1374 | 2.59E-07 |
| ebi-a-GCST90001597 | rs141447985 | 6 | 104062697 | T | 0.0581 | 0.2484 | 0.05362 | 3.73E-06 |
| ebi-a-GCST90001834 | rs141452540 | 20 | 47237403 | C | 0.0037 | -1.064 | 0.2319 | 4.72E-06 |
| ebi-a-GCST90001698 | rs141481912 | 14 | 97924775 | C | 0.0056 | 117.2 | 23.63 | 7.43E-07 |
| ebi-a-GCST90001825 | rs141507756 | 3 | 106999706 | A | 0.0245 | 0.372 | 0.08033 | 3.76E-06 |
| ebi-a-GCST90001695 | rs141515499 | 8 | 57100983 | T | 0.001 | 197.4 | 39.3 | 5.37E-07 |
| ebi-a-GCST90001936 | rs141518333 | 1 | 31011676 | T | 0.0012 | 1.769 | 0.3647 | 1.28E-06 |
| ebi-a-GCST90001943 | rs141518333 | 1 | 31011676 | T | 0.0012 | 1.813 | 0.3651 | 7.17E-07 |
| ebi-a-GCST90001508 | rs141541045 | 1 | 182131075 | T | 0.0065 | 0.7445 | 0.1572 | 2.28E-06 |
| ebi-a-GCST90001507 | rs141541045 | 1 | 182131075 | T | 0.0066 | 0.7554 | 0.1582 | 1.89E-06 |
| ebi-a-GCST90002099 | rs141541045 | 1 | 182131075 | T | 0.0059 | 0.9638 | 0.1767 | 5.33E-08 |
| ebi-a-GCST90001553 | rs141596789 | 7 | 28687227 | C | 9.00E-04 | 1.487 | 0.2796 | 1.12E-07 |
| ebi-a-GCST90001688 | rs141596789 | 7 | 28687227 | C | 9.00E-04 | 1.57 | 0.2927 | 8.71E-08 |
| ebi-a-GCST90002101 | rs141602247 | 13 | 53730995 | C | 0.0194 | 0.4724 | 0.1016 | 3.44E-06 |
| ebi-a-GCST90001776 | rs141602962 | 9 | 7815826 | G | 0.003 | -1.113 | 0.2299 | 1.34E-06 |
| ebi-a-GCST90001698 | rs141644068 | 1 | 220781210 | A | 9.00E-04 | 636.7 | 65.61 | 5.51E-22 |
| ebi-a-GCST90001700 | rs141656769 | 1 | 58381604 | T | 6.00E-04 | 14.34 | 2.896 | 7.73E-07 |
| ebi-a-GCST90001484 | rs141700904 | 6 | 57764436 | G | 0.0124 | 0.5307 | 0.1133 | 2.91E-06 |
| ebi-a-GCST90001869 | rs141784254 | 11 | 120364753 | G | 0.0038 | -1.123 | 0.2176 | 2.64E-07 |
| ebi-a-GCST90001842 | rs141784254 | 11 | 120364753 | G | 0.0038 | -1.116 | 0.2145 | 2.12E-07 |
| ebi-a-GCST90001858 | rs141784254 | 11 | 120364753 | G | 0.0038 | -1.015 | 0.2156 | 2.62E-06 |
| ebi-a-GCST90001515 | rs141792521 | 16 | 58686745 | G | 0.0137 | -0.7864 | 0.1448 | 6.37E-08 |
| ebi-a-GCST90001698 | rs141812086 | 2 | 204682687 | G | 0.0037 | 138.4 | 29.65 | 3.14E-06 |
| ebi-a-GCST90001997 | rs141845371 | 1 | 194738786 | A | 0.0138 | 0.5297 | 0.1072 | 8.03E-07 |
| ebi-a-GCST90001772 | rs141846315 | 1 | 173414084 | T | 8.00E-04 | -1.906 | 0.414 | 4.29E-06 |
| ebi-a-GCST90001700 | rs141850210 | 14 | 54222912 | A | 0.0018 | 9.155 | 1.747 | 1.71E-07 |
| ebi-a-GCST90001846 | rs141851092 | 4 | 178824850 | A | 0.0115 | 0.635 | 0.1277 | 6.92E-07 |
| ebi-a-GCST90001880 | rs141874683 | 2 | 5836918 | T | 0.0569 | -0.4355 | 0.08845 | 9.63E-07 |
| ebi-a-GCST90001875 | rs141874683 | 2 | 5836918 | T | 0.0569 | -0.4072 | 0.08767 | 3.76E-06 |
| ebi-a-GCST90001808 | rs141876897 | 9 | 94241794 | A | 0.0053 | 0.7649 | 0.1658 | 4.08E-06 |
| ebi-a-GCST90001394 | rs141880653 | 5 | 166970737 | T | 0.001 | 1.849 | 0.3979 | 3.49E-06 |
| ebi-a-GCST90001815 | rs141885962 | 10 | 66791343 | C | 0.0112 | -0.5286 | 0.1143 | 3.90E-06 |
| ebi-a-GCST90001965 | rs141889079 | 8 | 68736534 | T | 0.0368 | 0.3884 | 0.07414 | 1.73E-07 |
| ebi-a-GCST90001695 | rs141926147 | 6 | 146863903 | C | 0.0147 | 50.22 | 10.87 | 3.96E-06 |
| ebi-a-GCST90001567 | rs141935759 | 6 | 145631468 | A | 0.0108 | 0.494 | 0.1072 | 4.24E-06 |
| ebi-a-GCST90001570 | rs141942432 | 7 | 2243789 | T | NA | -8.344 | 1.804 | 3.90E-06 |
| ebi-a-GCST90001572 | rs141942432 | 7 | 2243789 | T | NA | 8.277 | 1.773 | 3.17E-06 |
| ebi-a-GCST90001574 | rs141942432 | 7 | 2243789 | T | NA | 8.507 | 1.797 | 2.29E-06 |
| ebi-a-GCST90002005 | rs141952082 | 6 | 3541155 | T | 0.1279 | 0.1784 | 0.03753 | 2.07E-06 |
| ebi-a-GCST90001921 | rs141959698 | 15 | 81676142 | A | 0.0011 | -1.818 | 0.3797 | 1.77E-06 |
| ebi-a-GCST90001918 | rs141959698 | 15 | 81676142 | A | 0.0011 | -1.77 | 0.3841 | 4.22E-06 |
| ebi-a-GCST90001698 | rs141976683 | 12 | 71790032 | C | 0.0031 | 154.9 | 31.41 | 8.51E-07 |
| ebi-a-GCST90001998 | rs1419935 | 7 | 151327301 | A | 0.0911 | -0.1939 | 0.04192 | 3.87E-06 |
| ebi-a-GCST90001964 | rs142005014 | 13 | 64051675 | T | 0.0068 | 0.7974 | 0.1638 | 1.19E-06 |
| ebi-a-GCST90002110 | rs142006783 | 16 | 74651733 | C | 0.0064 | -1.2 | 0.2618 | 4.97E-06 |
| ebi-a-GCST90001789 | rs142063706 | 8 | 141253242 | A | 0.0104 | -0.5336 | 0.1166 | 4.89E-06 |
| ebi-a-GCST90001615 | rs142067182 | 18 | 51354134 | T | 0.0508 | 0.2644 | 0.05643 | 2.89E-06 |
| ebi-a-GCST90001698 | rs142076427 | 16 | 5628078 | A | 0.0029 | 181.9 | 32.71 | 2.90E-08 |
| ebi-a-GCST90001642 | rs142089808 | 11 | 116171120 | G | 0.0037 | -1.048 | 0.1941 | 7.04E-08 |
| ebi-a-GCST90001644 | rs142089808 | 11 | 116171120 | G | 0.0038 | -1.044 | 0.1936 | 7.51E-08 |
| ebi-a-GCST90001588 | rs142089808 | 11 | 116171120 | G | 0.0037 | 1.056 | 0.1984 | 1.09E-07 |
| ebi-a-GCST90002016 | rs142099221 | 10 | 85391963 | T | 0.0787 | -0.2562 | 0.05261 | 1.18E-06 |
| ebi-a-GCST90002015 | rs142099221 | 10 | 85391963 | T | 0.0787 | -0.2544 | 0.0527 | 1.46E-06 |
| ebi-a-GCST90001727 | rs142109788 | 1 | 149815561 | G | 0.0014 | -2.259 | 0.4629 | 1.15E-06 |
| ebi-a-GCST90001725 | rs142109788 | 1 | 149815561 | G | 0.001 | -2.026 | 0.3889 | 1.99E-07 |
| ebi-a-GCST90001741 | rs142109788 | 1 | 149815561 | G | 0.001 | -1.964 | 0.3894 | 4.77E-07 |
| ebi-a-GCST90001730 | rs142109788 | 1 | 149815561 | G | 0.001 | -1.903 | 0.39 | 1.11E-06 |
| ebi-a-GCST90001737 | rs142109788 | 1 | 149815561 | G | 0.001 | -1.789 | 0.3894 | 4.49E-06 |
| ebi-a-GCST90001514 | rs142114110 | 7 | 82240518 | G | 0.0334 | -0.4209 | 0.08967 | 2.87E-06 |
| ebi-a-GCST90001998 | rs142118225 | 2 | 173198472 | G | 0.0025 | -1.041 | 0.2275 | 4.92E-06 |
| ebi-a-GCST90002065 | rs142122078 | 3 | 36456475 | A | 0.0066 | -0.856 | 0.1722 | 7.12E-07 |
| ebi-a-GCST90001855 | rs142130759 | 1 | 166075360 | A | 0.0113 | 0.6893 | 0.1357 | 4.00E-07 |
| ebi-a-GCST90001678 | rs142133440 | 9 | 128841396 | T | 0.0053 | -0.7914 | 0.1676 | 2.45E-06 |
| ebi-a-GCST90001698 | rs142151887 | 16 | 1071217 | A | 0.0019 | 274.8 | 38.79 | 1.69E-12 |
| ebi-a-GCST90001515 | rs1421603 | 7 | 131327890 | T | 0.9047 | 0.2687 | 0.05618 | 1.86E-06 |
| ebi-a-GCST90002049 | rs142164490 | 6 | 102083682 | A | 0.0099 | -1.008 | 0.1949 | 2.60E-07 |
| ebi-a-GCST90002094 | rs142164490 | 6 | 102083682 | A | 0.0099 | -0.9332 | 0.1964 | 2.21E-06 |
| ebi-a-GCST90002036 | rs142175829 | 3 | 128313034 | C | 0.0054 | -0.9346 | 0.1895 | 8.54E-07 |
| ebi-a-GCST90002109 | rs142186496 | 6 | 31473707 | G | 0.0688 | 0.4579 | 0.07698 | 3.34E-09 |
| ebi-a-GCST90001690 | rs142208889 | 19 | 51829114 | T | 1.00E-04 | 3.043 | 0.6653 | 4.96E-06 |
| ebi-a-GCST90001688 | rs142208889 | 19 | 51829114 | T | 1.00E-04 | 3.814 | 0.6612 | 8.68E-09 |
| ebi-a-GCST90001686 | rs142216336 | 12 | 59178989 | T | 0.0273 | 0.3436 | 0.06925 | 7.31E-07 |
| ebi-a-GCST90002109 | rs142241717 | 8 | 82111249 | A | 0.0304 | 0.5061 | 0.107 | 2.47E-06 |
| ebi-a-GCST90001466 | rs142245023 | 14 | 69634234 | A | 0.0067 | 0.7025 | 0.1505 | 3.17E-06 |
| ebi-a-GCST90001910 | rs142266193 | 13 | 30673693 | A | 0.0138 | -0.531 | 0.1096 | 1.33E-06 |
| ebi-a-GCST90001758 | rs1422711 | 5 | 143621838 | C | 0.6626 | -0.119 | 0.02595 | 4.68E-06 |
| ebi-a-GCST90001504 | rs142289362 | 16 | 77778611 | A | 0.016 | 0.4896 | 0.1007 | 1.23E-06 |
| ebi-a-GCST90001510 | rs142289362 | 16 | 77778611 | A | 0.016 | 0.4965 | 0.1013 | 1.01E-06 |
| ebi-a-GCST90001698 | rs142302016 | 7 | 54517004 | A | 0.0038 | 139.5 | 30.32 | 4.31E-06 |
| ebi-a-GCST90001586 | rs142340414 | 1 | 212967660 | T | 1.00E-04 | -3.05 | 0.6547 | 3.29E-06 |
| ebi-a-GCST90002115 | rs142342151 | 14 | 104847658 | C | 0.0083 | -0.6523 | 0.1423 | 4.77E-06 |
| ebi-a-GCST90001673 | rs142394211 | 1 | 20454583 | T | 0.0751 | -0.2225 | 0.04743 | 2.82E-06 |
| ebi-a-GCST90002046 | rs142398114 | 9 | 106075712 | T | 0.0018 | -2.042 | 0.4267 | 1.85E-06 |
| ebi-a-GCST90001536 | rs142403852 | 12 | 86979143 | C | 0.0077 | -0.6934 | 0.1433 | 1.37E-06 |
| ebi-a-GCST90002006 | rs142408635 | 11 | 92180250 | C | 0.0242 | -0.3944 | 0.08159 | 1.39E-06 |
| ebi-a-GCST90001532 | rs142419042 | 11 | 104445613 | T | 0.025 | -0.5889 | 0.1119 | 1.59E-07 |
| ebi-a-GCST90001531 | rs142419042 | 11 | 104445613 | T | 0.0251 | -0.5472 | 0.1123 | 1.19E-06 |
| ebi-a-GCST90001695 | rs142419740 | 21 | 47472186 | T | 3.00E-04 | 557.7 | 103 | 6.61E-08 |
| ebi-a-GCST90001699 | rs142420464 | 10 | 12757694 | T | 0.0016 | 21.72 | 4.38 | 7.47E-07 |
| ebi-a-GCST90001489 | rs142422333 | 5 | 155375550 | T | 0.0515 | -0.2705 | 0.05905 | 4.78E-06 |
| ebi-a-GCST90002097 | rs142436127 | 3 | 118883955 | A | 0.0067 | -1.066 | 0.2325 | 4.95E-06 |
| ebi-a-GCST90002091 | rs142456232 | 11 | 4960640 | T | 0.0627 | -0.3457 | 0.07534 | 4.79E-06 |
| ebi-a-GCST90002072 | rs142456232 | 11 | 4960640 | T | 0.0573 | -0.3341 | 0.05919 | 1.81E-08 |
| ebi-a-GCST90001967 | rs142456232 | 11 | 4960640 | T | 0.0572 | -0.3287 | 0.05796 | 1.55E-08 |
| ebi-a-GCST90001695 | rs142470414 | 13 | 73498369 | T | 9.00E-04 | 259.6 | 43.23 | 2.11E-09 |
| ebi-a-GCST90001458 | rs142472859 | 2 | 40026310 | C | 0.0061 | 0.7935 | 0.1634 | 1.25E-06 |
| ebi-a-GCST90001971 | rs142476157 | 11 | 101729595 | T | 0.0514 | 0.2789 | 0.06042 | 4.08E-06 |
| ebi-a-GCST90002108 | rs1424773 | 2 | 108017810 | A | 0.7612 | -0.1924 | 0.04154 | 3.95E-06 |
| ebi-a-GCST90001918 | rs142492586 | 6 | 33202756 | C | 0.198 | -0.1762 | 0.0355 | 7.32E-07 |
| ebi-a-GCST90001915 | rs142492586 | 6 | 33202756 | C | 0.1971 | -0.1691 | 0.03504 | 1.46E-06 |
| ebi-a-GCST90001698 | rs142505312 | 22 | 50155532 | C | 0.0035 | 173.5 | 32.21 | 7.70E-08 |
| ebi-a-GCST90001697 | rs142507918 | 4 | 188764989 | A | 0.0035 | 4.697 | 1.007 | 3.21E-06 |
| ebi-a-GCST90001696 | rs142507918 | 4 | 188764989 | A | 0.0035 | 11.97 | 2.464 | 1.23E-06 |
| ebi-a-GCST90001697 | rs142522853 | 7 | 1084031 | T | 3.00E-04 | 17.94 | 3.35 | 9.19E-08 |
| ebi-a-GCST90001695 | rs142522853 | 7 | 1084031 | T | 3.00E-04 | 595.1 | 71.5 | 1.22E-16 |
| ebi-a-GCST90001646 | rs142527102 | 8 | 17769401 | C | 0.0061 | -0.7471 | 0.1632 | 4.85E-06 |
| ebi-a-GCST90001643 | rs142527102 | 8 | 17769401 | C | 0.0061 | 0.7585 | 0.1625 | 3.16E-06 |
| ebi-a-GCST90001668 | rs142531546 | 7 | 37829371 | T | 0.0317 | -0.3326 | 0.07262 | 4.80E-06 |
| ebi-a-GCST90001662 | rs142531546 | 7 | 37829371 | T | 0.0317 | 0.3425 | 0.07276 | 2.62E-06 |
| ebi-a-GCST90001779 | rs142537458 | 10 | 5982082 | C | 0.0215 | 0.4001 | 0.08706 | 4.47E-06 |
| ebi-a-GCST90002010 | rs142573049 | 20 | 60176623 | T | 0.0043 | 0.8337 | 0.1784 | 3.08E-06 |
| ebi-a-GCST90001583 | rs142584082 | 14 | 98059388 | G | 0.0015 | 1.359 | 0.2946 | 4.09E-06 |
| ebi-a-GCST90001926 | rs142587417 | 17 | 58777014 | A | 0.0144 | -0.8103 | 0.1169 | 5.08E-12 |
| ebi-a-GCST90001698 | rs142604048 | 2 | 87503702 | C | 0.0026 | 216.5 | 35.34 | 1.00E-09 |
| ebi-a-GCST90001698 | rs142609156 | 4 | 11158267 | T | 0.0531 | 41.07 | 8.012 | 3.12E-07 |
| ebi-a-GCST90001659 | rs142629161 | 21 | 29333825 | C | 3.00E-04 | -2.79 | 0.586 | 2.01E-06 |
| ebi-a-GCST90002104 | rs142640535 | 8 | 122566151 | G | 0.0012 | -2.015 | 0.4281 | 2.64E-06 |
| ebi-a-GCST90001844 | rs142679567 | 2 | 173083726 | T | 0.0305 | 0.3911 | 0.08237 | 2.16E-06 |
| ebi-a-GCST90001698 | rs142686680 | 12 | 29095512 | C | 7.00E-04 | 440.2 | 64.38 | 9.46E-12 |
| ebi-a-GCST90001835 | rs142693862 | 4 | 154572780 | C | 0.0047 | -0.9466 | 0.1941 | 1.14E-06 |
| ebi-a-GCST90001697 | rs142702764 | 1 | 158276239 | T | 1.00E-04 | 30.28 | 5.61 | 7.23E-08 |
| ebi-a-GCST90001634 | rs142704274 | 17 | 39899038 | A | 0.02 | -0.4672 | 0.08984 | 2.10E-07 |
| ebi-a-GCST90001697 | rs142707561 | 1 | 42381260 | A | 0.0058 | 4.375 | 0.8431 | 2.23E-07 |
| ebi-a-GCST90001695 | rs142707561 | 1 | 42381260 | A | 0.0059 | 84.4 | 18.01 | 2.88E-06 |
| ebi-a-GCST90001982 | rs142752828 | 4 | 14542063 | T | 0.0041 | 0.952 | 0.1916 | 7.04E-07 |
| ebi-a-GCST90001909 | rs142767469 | 5 | 26288128 | A | 0.0093 | -0.7064 | 0.1376 | 3.01E-07 |
| ebi-a-GCST90001552 | rs142772175 | 2 | 120240225 | T | 1.00E-04 | 3.437 | 0.7107 | 1.38E-06 |
| ebi-a-GCST90001712 | rs142780018 | 22 | 44395926 | A | 0.0463 | -0.3333 | 0.05866 | 1.44E-08 |
| ebi-a-GCST90001711 | rs142780018 | 22 | 44395926 | A | 0.0463 | -0.3313 | 0.05854 | 1.63E-08 |
| ebi-a-GCST90001710 | rs142780018 | 22 | 44395926 | A | 0.0463 | -0.3245 | 0.05867 | 3.41E-08 |
| ebi-a-GCST90001520 | rs142786778 | 6 | 23419953 | A | 0.0019 | -1.725 | 0.3728 | 3.96E-06 |
| ebi-a-GCST90002086 | rs142804909 | 1 | 64510605 | A | 0.0118 | 0.6154 | 0.1271 | 1.34E-06 |
| ebi-a-GCST90002061 | rs142814565 | 17 | 75698827 | T | 0.0017 | 1.533 | 0.3197 | 1.70E-06 |
| ebi-a-GCST90001828 | rs142817955 | 7 | 33903759 | A | 0.0023 | -1.195 | 0.2497 | 1.78E-06 |
| ebi-a-GCST90001430 | rs142838362 | 21 | 47606966 | T | 0.0577 | 0.2463 | 0.05243 | 2.72E-06 |
| ebi-a-GCST90001695 | rs142857708 | 4 | 133864790 | C | 9.00E-04 | 269.9 | 57.94 | 3.33E-06 |
| ebi-a-GCST90001888 | rs142863502 | 3 | 78828398 | G | 0.0166 | 0.5215 | 0.1066 | 1.05E-06 |
| ebi-a-GCST90001698 | rs142869493 | 11 | 87291175 | T | 0.0013 | 394.5 | 51.12 | 1.55E-14 |
| ebi-a-GCST90001977 | rs142882340 | 3 | 98779043 | G | 0.006 | -0.829 | 0.166 | 6.20E-07 |
| ebi-a-GCST90001781 | rs142906903 | 8 | 82141410 | C | 0.0112 | 0.747 | 0.1576 | 2.31E-06 |
| ebi-a-GCST90001977 | rs142909976 | 9 | 31120417 | A | 0.0101 | -0.6483 | 0.1312 | 8.23E-07 |
| ebi-a-GCST90001695 | rs142911819 | 12 | 114081812 | A | 6.00E-04 | 320.2 | 56.32 | 1.42E-08 |
| ebi-a-GCST90001461 | rs142917473 | 12 | 133840072 | C | 0.3072 | -0.1534 | 0.03214 | 1.89E-06 |
| ebi-a-GCST90001698 | rs142922198 | 5 | 145713781 | C | 0.0072 | 127.1 | 21.37 | 3.00E-09 |
| ebi-a-GCST90001855 | rs142935756 | 6 | 68280346 | G | 0.0046 | -0.9409 | 0.1983 | 2.18E-06 |
| ebi-a-GCST90001912 | rs142947331 | 6 | 13780175 | C | 0.0416 | -0.319 | 0.06852 | 3.37E-06 |
| ebi-a-GCST90001811 | rs142962704 | 3 | 128075585 | T | 0.0034 | 0.9983 | 0.2077 | 1.60E-06 |
| ebi-a-GCST90001688 | rs142967914 | 9 | 12800264 | C | 1.00E-04 | 3.794 | 0.6676 | 1.44E-08 |
| ebi-a-GCST90001698 | rs142978760 | 6 | 6047112 | A | 0.0142 | 80.7 | 15.86 | 3.78E-07 |
| ebi-a-GCST90001697 | rs142980650 | 10 | 109010961 | T | 0.0035 | 5.278 | 1.026 | 2.82E-07 |
| ebi-a-GCST90001508 | rs1430018 | 2 | 38132316 | C | 0.9022 | -0.2323 | 0.04369 | 1.13E-07 |
| ebi-a-GCST90001996 | rs143002526 | 3 | 184649632 | A | 0.0133 | -0.55 | 0.1127 | 1.10E-06 |
| ebi-a-GCST90001436 | rs143003356 | 4 | 142016471 | A | 0.0115 | -0.5299 | 0.1157 | 4.79E-06 |
| ebi-a-GCST90001474 | rs143032729 | 1 | 160353789 | G | 0.0326 | -0.3966 | 0.07264 | 5.11E-08 |
| ebi-a-GCST90001463 | rs143032729 | 1 | 160353789 | G | 0.0326 | 0.4142 | 0.07525 | 3.97E-08 |
| ebi-a-GCST90001695 | rs143044126 | 4 | 31116547 | A | 0.0037 | 98.05 | 21.43 | 4.93E-06 |
| ebi-a-GCST90001766 | rs143053604 | 11 | 12834785 | G | 0.0056 | -0.8464 | 0.1643 | 2.70E-07 |
| ebi-a-GCST90001653 | rs143066848 | 5 | 83073780 | G | 0.0448 | -0.293 | 0.06073 | 1.47E-06 |
| ebi-a-GCST90001656 | rs143066848 | 5 | 83073780 | G | 0.0448 | 0.293 | 0.06073 | 1.47E-06 |
| ebi-a-GCST90001711 | rs143106266 | 13 | 29908421 | A | 0.0025 | -1.252 | 0.246 | 3.76E-07 |
| ebi-a-GCST90001710 | rs143106266 | 13 | 29908421 | A | 0.0025 | -1.247 | 0.2463 | 4.36E-07 |
| ebi-a-GCST90001712 | rs143106266 | 13 | 29908421 | A | 0.0025 | -1.237 | 0.246 | 5.18E-07 |
| ebi-a-GCST90001709 | rs143106266 | 13 | 29908421 | A | 0.0025 | -1.229 | 0.2472 | 6.96E-07 |
| ebi-a-GCST90001718 | rs143106266 | 13 | 29908421 | A | 0.0025 | -1.223 | 0.2472 | 7.95E-07 |
| ebi-a-GCST90001715 | rs143106266 | 13 | 29908421 | A | 0.0025 | -1.22 | 0.2468 | 8.07E-07 |
| ebi-a-GCST90001702 | rs143106266 | 13 | 29908421 | A | 0.0025 | -1.2 | 0.2472 | 1.27E-06 |
| ebi-a-GCST90001716 | rs143106266 | 13 | 29908421 | A | 0.0025 | -1.196 | 0.2474 | 1.39E-06 |
| ebi-a-GCST90001704 | rs143106266 | 13 | 29908421 | A | 0.0025 | -1.183 | 0.247 | 1.75E-06 |
| ebi-a-GCST90001829 | rs143106266 | 13 | 29908421 | A | 0.0025 | -1.177 | 0.2473 | 2.01E-06 |
| ebi-a-GCST90001719 | rs143106266 | 13 | 29908421 | A | 0.0025 | -1.176 | 0.2473 | 2.03E-06 |
| ebi-a-GCST90001720 | rs143106266 | 13 | 29908421 | A | 0.0025 | -1.171 | 0.2485 | 2.53E-06 |
| ebi-a-GCST90001705 | rs143106266 | 13 | 29908421 | A | 0.0025 | -1.163 | 0.2462 | 2.39E-06 |
| ebi-a-GCST90001703 | rs143106266 | 13 | 29908421 | A | 0.0025 | -1.155 | 0.2473 | 3.11E-06 |
| ebi-a-GCST90001717 | rs143106266 | 13 | 29908421 | A | 0.0025 | -1.148 | 0.247 | 3.48E-06 |
| ebi-a-GCST90001708 | rs143106266 | 13 | 29908421 | A | 0.0025 | -1.135 | 0.2481 | 4.94E-06 |
| ebi-a-GCST90001424 | rs143109147 | 1 | 40387371 | C | 4.00E-04 | 2.746 | 0.5892 | 3.27E-06 |
| ebi-a-GCST90001430 | rs143109147 | 1 | 40387371 | C | 4.00E-04 | 2.959 | 0.5843 | 4.28E-07 |
| ebi-a-GCST90001761 | rs143129208 | 11 | 99391820 | G | 0.0025 | 1.068 | 0.2304 | 3.71E-06 |
| ebi-a-GCST90001759 | rs143129208 | 11 | 99391820 | G | 0.0025 | 1.096 | 0.2323 | 2.46E-06 |
| ebi-a-GCST90001483 | rs143132810 | 10 | 96890004 | C | 0.0263 | 0.6358 | 0.0789 | 1.07E-15 |
| ebi-a-GCST90001782 | rs143132954 | 1 | 61322867 | C | 0.0161 | -0.6306 | 0.1363 | 3.97E-06 |
| ebi-a-GCST90002005 | rs143140559 | 1 | 160972255 | T | 0.0097 | -0.6462 | 0.1283 | 4.95E-07 |
| ebi-a-GCST90001459 | rs143140559 | 1 | 160972255 | T | 0.0094 | 0.6438 | 0.1286 | 5.78E-07 |
| ebi-a-GCST90001463 | rs143140559 | 1 | 160972255 | T | 0.0094 | 0.6557 | 0.1304 | 5.15E-07 |
| ebi-a-GCST90001465 | rs143140559 | 1 | 160972255 | T | 0.0094 | 0.6732 | 0.1308 | 2.80E-07 |
| ebi-a-GCST90001473 | rs143140559 | 1 | 160972255 | T | 0.0094 | 0.71 | 0.1289 | 3.88E-08 |
| ebi-a-GCST90001464 | rs143140559 | 1 | 160972255 | T | 0.0094 | 0.7391 | 0.1304 | 1.55E-08 |
| ebi-a-GCST90001462 | rs143140559 | 1 | 160972255 | T | 0.0094 | 0.7418 | 0.1284 | 8.28E-09 |
| ebi-a-GCST90001472 | rs143140559 | 1 | 160972255 | T | 0.0094 | 0.7645 | 0.1298 | 4.22E-09 |
| ebi-a-GCST90001469 | rs143140559 | 1 | 160972255 | T | 0.0094 | 0.7929 | 0.1294 | 1.01E-09 |
| ebi-a-GCST90001468 | rs143140559 | 1 | 160972255 | T | 0.0094 | 0.8211 | 0.1303 | 3.34E-10 |
| ebi-a-GCST90001629 | rs1431485 | 10 | 118193216 | C | 0.9184 | -0.2005 | 0.04329 | 3.74E-06 |
| ebi-a-GCST90002046 | rs143150964 | 1 | 238709665 | A | 0.0174 | -0.6499 | 0.1405 | 4.04E-06 |
| ebi-a-GCST90001450 | rs143153359 | 9 | 76145588 | T | 0.0013 | 2.05 | 0.4039 | 4.12E-07 |
| ebi-a-GCST90001808 | rs143164248 | 10 | 28945318 | A | 0.0632 | 0.2346 | 0.05074 | 3.91E-06 |
| ebi-a-GCST90001806 | rs143164248 | 10 | 28945318 | A | 0.0632 | 0.2363 | 0.05087 | 3.53E-06 |
| ebi-a-GCST90001394 | rs143176410 | 16 | 84305239 | C | 0.001 | 1.912 | 0.3929 | 1.19E-06 |
| ebi-a-GCST90001514 | rs1432243 | 2 | 137773206 | C | 0.9771 | -0.5029 | 0.1032 | 1.20E-06 |
| ebi-a-GCST90001695 | rs143230107 | 12 | 26115369 | G | 0.0032 | 115.3 | 24.76 | 3.34E-06 |
| ebi-a-GCST90001554 | rs143237141 | 22 | 45176439 | A | 0.0365 | 0.3181 | 0.06864 | 3.71E-06 |
| ebi-a-GCST90001903 | rs143271161 | 15 | 35493491 | G | 0.0259 | -0.4124 | 0.08661 | 2.02E-06 |
| ebi-a-GCST90001897 | rs143273680 | 12 | 14071670 | G | 0.0185 | 0.4873 | 0.09956 | 1.04E-06 |
| ebi-a-GCST90001511 | rs143283244 | 15 | 94643151 | A | 6.00E-04 | -2.405 | 0.4952 | 1.24E-06 |
| ebi-a-GCST90001510 | rs143283244 | 15 | 94643151 | A | 6.00E-04 | -2.405 | 0.5001 | 1.59E-06 |
| ebi-a-GCST90001512 | rs143283244 | 15 | 94643151 | A | 6.00E-04 | -2.27 | 0.4951 | 4.70E-06 |
| ebi-a-GCST90001972 | rs143298995 | 7 | 48232877 | G | 0.7695 | -0.1477 | 0.0291 | 4.08E-07 |
| ebi-a-GCST90001971 | rs143298995 | 7 | 48232877 | G | 0.7695 | -0.1443 | 0.02866 | 5.01E-07 |
| ebi-a-GCST90001973 | rs143298995 | 7 | 48232877 | G | 0.7695 | -0.1416 | 0.02961 | 1.81E-06 |
| ebi-a-GCST90001974 | rs143298995 | 7 | 48232877 | G | 0.7695 | -0.136 | 0.02744 | 7.65E-07 |
| ebi-a-GCST90002116 | rs143305120 | 6 | 13991643 | C | 0.0359 | -0.3957 | 0.07188 | 4.01E-08 |
| ebi-a-GCST90002028 | rs143306403 | 2 | 232218321 | G | 0.0186 | -0.4915 | 0.1073 | 4.82E-06 |
| ebi-a-GCST90001733 | rs143330436 | 9 | 26801194 | T | 0.0021 | -1.278 | 0.2768 | 4.03E-06 |
| ebi-a-GCST90002050 | rs143331027 | 1 | 28663941 | A | 0.0215 | -0.6068 | 0.1297 | 3.11E-06 |
| ebi-a-GCST90001547 | rs143346058 | 3 | 141674361 | G | 3.00E-04 | -2.989 | 0.6329 | 2.41E-06 |
| ebi-a-GCST90001587 | rs143356597 | 11 | 588659 | A | 0.0404 | 0.2916 | 0.06193 | 2.59E-06 |
| ebi-a-GCST90001584 | rs143356597 | 11 | 588659 | A | 0.0404 | 0.2965 | 0.06165 | 1.58E-06 |
| ebi-a-GCST90002013 | rs143366996 | 10 | 83561663 | C | 0.0061 | -0.8552 | 0.1806 | 2.30E-06 |
| ebi-a-GCST90001525 | rs143383466 | 3 | 196068066 | T | 0.0043 | 1.142 | 0.2466 | 3.90E-06 |
| ebi-a-GCST90001771 | rs143386390 | 1 | 6146588 | T | 0.0026 | 1.197 | 0.2535 | 2.41E-06 |
| ebi-a-GCST90001765 | rs143386390 | 1 | 6146588 | T | 0.0026 | 1.222 | 0.2527 | 1.39E-06 |
| ebi-a-GCST90001698 | rs143393137 | 17 | 30967385 | T | 0.0028 | 189.1 | 33.53 | 1.84E-08 |
| ebi-a-GCST90001700 | rs143395500 | 10 | 69536834 | T | 1.00E-04 | 51.17 | 8.845 | 7.92E-09 |
| ebi-a-GCST90001698 | rs143395500 | 10 | 69536834 | T | 1.00E-04 | 1232 | 203.7 | 1.63E-09 |
| ebi-a-GCST90001577 | rs143397127 | 7 | 12847391 | C | 0.0157 | -0.4629 | 0.09637 | 1.62E-06 |
| ebi-a-GCST90001392 | rs143397127 | 7 | 12847391 | C | 0.0157 | -0.4626 | 0.09576 | 1.42E-06 |
| ebi-a-GCST90001409 | rs143397127 | 7 | 12847391 | C | 0.0157 | -0.4624 | 0.09926 | 3.30E-06 |
| ebi-a-GCST90001394 | rs143397127 | 7 | 12847391 | C | 0.0157 | -0.4549 | 0.09626 | 2.38E-06 |
| ebi-a-GCST90001730 | rs143403164 | 3 | 111246616 | A | 0.01 | -0.5736 | 0.1253 | 4.86E-06 |
| ebi-a-GCST90002016 | rs143407337 | 7 | 1095701 | T | 0.2142 | 0.2427 | 0.03394 | 1.10E-12 |
| ebi-a-GCST90002015 | rs143407337 | 7 | 1095701 | T | 0.2142 | 0.2439 | 0.03399 | 9.17E-13 |
| ebi-a-GCST90001517 | rs143438920 | 3 | 15416436 | A | 0.01 | -0.7744 | 0.1677 | 4.15E-06 |
| ebi-a-GCST90002120 | rs143440491 | 7 | 46541742 | T | 0.0384 | -0.3393 | 0.07232 | 2.82E-06 |
| ebi-a-GCST90001905 | rs143443965 | 8 | 142893697 | T | 0.0312 | 0.3746 | 0.08064 | 3.54E-06 |
| ebi-a-GCST90001756 | rs143489681 | 15 | 94271829 | A | 0.002 | 1.224 | 0.2628 | 3.33E-06 |
| ebi-a-GCST90001688 | rs143498795 | 8 | 140342260 | A | 1.00E-04 | 3.717 | 0.67 | 3.11E-08 |
| ebi-a-GCST90001465 | rs143512471 | 5 | 63081773 | A | 0.026 | -0.3741 | 0.08154 | 4.63E-06 |
| ebi-a-GCST90001698 | rs143517669 | 19 | 868083 | T | 0.0041 | 131.3 | 26.91 | 1.12E-06 |
| ebi-a-GCST90001884 | rs143549694 | 4 | 161858005 | G | 0.0161 | 0.535 | 0.1102 | 1.25E-06 |
| ebi-a-GCST90001432 | rs143553941 | 12 | 109368119 | C | 0.013 | 0.501 | 0.107 | 2.96E-06 |
| ebi-a-GCST90001800 | rs143553941 | 12 | 109368119 | C | 0.013 | 0.5135 | 0.1095 | 2.83E-06 |
| ebi-a-GCST90001397 | rs143553941 | 12 | 109368119 | C | 0.013 | 0.5187 | 0.1092 | 2.09E-06 |
| ebi-a-GCST90002042 | rs143599997 | 12 | 130798660 | G | 0.0446 | 0.4268 | 0.0895 | 2.02E-06 |
| ebi-a-GCST90001989 | rs143602490 | 20 | 44010127 | T | 0.0093 | 0.6066 | 0.1294 | 2.85E-06 |
| ebi-a-GCST90001698 | rs143604762 | 12 | 131000132 | C | 0.0117 | 76.34 | 16.34 | 3.12E-06 |
| ebi-a-GCST90001518 | rs143612223 | 10 | 112429560 | T | 5.00E-04 | 3.174 | 0.6763 | 2.89E-06 |
| ebi-a-GCST90001520 | rs143612223 | 10 | 112429560 | T | 5.00E-04 | 3.383 | 0.6763 | 6.23E-07 |
| ebi-a-GCST90001517 | rs143612223 | 10 | 112429560 | T | 5.00E-04 | 3.416 | 0.6702 | 3.80E-07 |
| ebi-a-GCST90001538 | rs143624012 | 13 | 74308892 | C | 0.0098 | 0.5892 | 0.1288 | 4.95E-06 |
| ebi-a-GCST90001923 | rs143650607 | 2 | 192303701 | A | 0.0279 | 0.3859 | 0.08324 | 3.70E-06 |
| ebi-a-GCST90001398 | rs143666202 | 14 | 77579227 | A | 0.0502 | 0.268 | 0.05703 | 2.71E-06 |
| ebi-a-GCST90001397 | rs143666202 | 14 | 77579227 | A | 0.0503 | 0.2759 | 0.0579 | 1.96E-06 |
| ebi-a-GCST90001698 | rs143772909 | 10 | 101494118 | T | 0.001 | 328.1 | 52.32 | 4.02E-10 |
| ebi-a-GCST90001426 | rs143785715 | 1 | 19500582 | T | 0.0027 | 1.034 | 0.2251 | 4.55E-06 |
| ebi-a-GCST90001552 | rs143788880 | 17 | 57361218 | A | 0.0074 | 0.4915 | 0.106 | 3.66E-06 |
| ebi-a-GCST90001552 | rs143809235 | 2 | 167484735 | G | NA | -12.2 | 2.267 | 7.88E-08 |
| ebi-a-GCST90001553 | rs143809235 | 2 | 167484735 | G | NA | -12.04 | 2.063 | 5.88E-09 |
| ebi-a-GCST90001551 | rs143809235 | 2 | 167484735 | G | NA | -11.47 | 2.101 | 5.17E-08 |
| ebi-a-GCST90001997 | rs143813477 | 3 | 44507750 | A | 0.2104 | -0.1451 | 0.03138 | 3.90E-06 |
| ebi-a-GCST90001698 | rs143824026 | 19 | 33797284 | T | 0.0067 | 110.1 | 20.78 | 1.23E-07 |
| ebi-a-GCST90001697 | rs143825022 | 8 | 28561116 | T | 0.001 | 9.628 | 1.921 | 5.66E-07 |
| ebi-a-GCST90001695 | rs143825022 | 8 | 28561116 | T | 0.001 | 251.2 | 41.16 | 1.15E-09 |
| ebi-a-GCST90001700 | rs143845801 | 7 | 84434549 | C | 7.00E-04 | 18.54 | 2.753 | 1.90E-11 |
| ebi-a-GCST90001695 | rs143845801 | 7 | 84434549 | C | 7.00E-04 | 271.2 | 48.48 | 2.39E-08 |
| ebi-a-GCST90001698 | rs143845801 | 7 | 84434549 | C | 7.00E-04 | 767.7 | 64.23 | 2.77E-32 |
| ebi-a-GCST90001908 | rs143859887 | 20 | 58399063 | G | 0.0015 | 1.585 | 0.3407 | 3.43E-06 |
| ebi-a-GCST90001698 | rs143871291 | 19 | 1191667 | G | 0.0092 | 111.8 | 18.01 | 6.12E-10 |
| ebi-a-GCST90001738 | rs1439005 | 3 | 61094800 | C | 0.4587 | -0.125 | 0.0246 | 3.97E-07 |
| ebi-a-GCST90001723 | rs1439005 | 3 | 61094800 | C | 0.4587 | -0.1201 | 0.0246 | 1.10E-06 |
| ebi-a-GCST90001740 | rs1439005 | 3 | 61094800 | C | 0.4588 | -0.1184 | 0.02464 | 1.60E-06 |
| ebi-a-GCST90001736 | rs1439005 | 3 | 61094800 | C | 0.4588 | -0.1134 | 0.02463 | 4.29E-06 |
| ebi-a-GCST90002000 | rs143917558 | 9 | 106448354 | G | 0.0131 | 0.5222 | 0.1101 | 2.20E-06 |
| ebi-a-GCST90001697 | rs143930177 | 10 | 70543706 | A | 0.0072 | 4.44 | 0.7441 | 2.66E-09 |
| ebi-a-GCST90001695 | rs143930177 | 10 | 70543706 | A | 0.0072 | 80 | 15.92 | 5.26E-07 |
| ebi-a-GCST90001993 | rs143930325 | 4 | 146544530 | G | 0.0134 | 0.4918 | 0.1063 | 3.86E-06 |
| ebi-a-GCST90001698 | rs143940797 | 1 | 52644883 | A | 0.0565 | 43.94 | 7.987 | 4.03E-08 |
| ebi-a-GCST90001702 | rs143944768 | 22 | 44378670 | T | 0.0463 | -0.3468 | 0.0588 | 4.03E-09 |
| ebi-a-GCST90001718 | rs143944768 | 22 | 44378670 | T | 0.0463 | -0.3347 | 0.05887 | 1.41E-08 |
| ebi-a-GCST90001698 | rs143946135 | 16 | 69023689 | G | 0.0053 | 161.6 | 24.83 | 8.64E-11 |
| ebi-a-GCST90001876 | rs143967899 | 3 | 33728588 | C | 0.0028 | 1.68 | 0.3578 | 2.94E-06 |
| ebi-a-GCST90001586 | rs143984211 | 11 | 395746 | T | 0.032 | -0.3205 | 0.06928 | 3.85E-06 |
| ebi-a-GCST90001579 | rs143984211 | 11 | 395746 | T | 0.032 | 0.3255 | 0.06954 | 2.96E-06 |
| ebi-a-GCST90001971 | rs144027086 | 7 | 15148913 | A | 5.00E-04 | 2.42 | 0.5066 | 1.86E-06 |
| ebi-a-GCST90001973 | rs144027086 | 7 | 15148913 | A | 5.00E-04 | 2.441 | 0.5191 | 2.68E-06 |
| ebi-a-GCST90001972 | rs144027086 | 7 | 15148913 | A | 5.00E-04 | 2.452 | 0.515 | 2.01E-06 |
| ebi-a-GCST90001974 | rs144027086 | 7 | 15148913 | A | 5.00E-04 | 2.485 | 0.4906 | 4.33E-07 |
| ebi-a-GCST90002082 | rs144027086 | 7 | 15148913 | A | 5.00E-04 | 2.503 | 0.4929 | 4.02E-07 |
| ebi-a-GCST90001975 | rs144027086 | 7 | 15148913 | A | 5.00E-04 | 2.546 | 0.5562 | 4.89E-06 |
| ebi-a-GCST90001985 | rs144062689 | 22 | 25308118 | G | 0.0014 | 1.531 | 0.3212 | 1.96E-06 |
| ebi-a-GCST90001537 | rs144079997 | 17 | 72909337 | T | 0.1081 | 0.1872 | 0.04058 | 4.09E-06 |
| ebi-a-GCST90002116 | rs144091827 | 2 | 69187142 | A | 0.0175 | -0.4602 | 0.1001 | 4.49E-06 |
| ebi-a-GCST90001700 | rs144104650 | 17 | 5023557 | A | 6.00E-04 | 15.28 | 3.08 | 7.31E-07 |
| ebi-a-GCST90001698 | rs144104650 | 17 | 5023557 | A | 6.00E-04 | 591.4 | 73.04 | 7.80E-16 |
| ebi-a-GCST90001509 | rs144105723 | 10 | 119138812 | G | 0.0649 | 0.2382 | 0.05143 | 3.74E-06 |
| ebi-a-GCST90002007 | rs144126567 | 1 | 161510516 | G | 0.1712 | -0.3054 | 0.03316 | 5.39E-20 |
| ebi-a-GCST90001982 | rs144126567 | 1 | 161510516 | G | 0.1721 | 0.2691 | 0.03257 | 2.01E-16 |
| ebi-a-GCST90001581 | rs144126567 | 1 | 161510516 | G | 0.1737 | 0.3274 | 0.03235 | 9.29E-24 |
| ebi-a-GCST90001994 | rs144126567 | 1 | 161510516 | G | 0.1735 | 0.5108 | 0.03154 | 5.51E-57 |
| ebi-a-GCST90001814 | rs144135856 | 9 | 129618074 | T | 4.00E-04 | -4.574 | 0.9957 | 4.50E-06 |
| ebi-a-GCST90001867 | rs144137185 | 3 | 19582653 | T | 0.0863 | 0.2413 | 0.04833 | 6.35E-07 |
| ebi-a-GCST90001861 | rs144137185 | 3 | 19582653 | T | 0.0863 | 0.2524 | 0.04859 | 2.20E-07 |
| ebi-a-GCST90001460 | rs144152085 | 6 | 67895544 | G | 9.00E-04 | -1.824 | 0.3956 | 4.17E-06 |
| ebi-a-GCST90001630 | rs144156455 | 11 | 70988762 | C | 0.0356 | 0.3176 | 0.06734 | 2.49E-06 |
| ebi-a-GCST90001632 | rs144156455 | 11 | 70988762 | C | 0.0361 | 0.324 | 0.06651 | 1.15E-06 |
| ebi-a-GCST90001631 | rs144156455 | 11 | 70988762 | C | 0.0361 | 0.3243 | 0.06611 | 9.69E-07 |
| ebi-a-GCST90001697 | rs144159369 | 11 | 126353889 | C | 4.00E-04 | 14.72 | 2.717 | 6.52E-08 |
| ebi-a-GCST90001787 | rs144182927 | 21 | 23113704 | T | 0.0191 | -0.4156 | 0.08772 | 2.25E-06 |
| ebi-a-GCST90001793 | rs144182927 | 21 | 23113704 | T | 0.0191 | -0.413 | 0.08755 | 2.48E-06 |
| ebi-a-GCST90001536 | rs144196931 | 7 | 139232896 | A | 0.0229 | -0.3683 | 0.08013 | 4.45E-06 |
| ebi-a-GCST90001698 | rs144206851 | 9 | 77966779 | T | 0.0012 | 494.7 | 55.7 | 1.04E-18 |
| ebi-a-GCST90001756 | rs144229045 | 3 | 132962425 | T | 0.067 | -0.2348 | 0.04995 | 2.70E-06 |
| ebi-a-GCST90001698 | rs144258173 | 8 | 2859400 | G | 7.00E-04 | 369.2 | 61.96 | 2.79E-09 |
| ebi-a-GCST90002075 | rs144261817 | 5 | 40009649 | C | 0.021 | -0.4662 | 0.09343 | 6.38E-07 |
| ebi-a-GCST90001700 | rs144275943 | 14 | 34065832 | G | 3.00E-04 | 19.21 | 4.183 | 4.53E-06 |
| ebi-a-GCST90001698 | rs144275943 | 14 | 34065832 | G | 3.00E-04 | 730 | 96.39 | 4.65E-14 |
| ebi-a-GCST90001685 | rs144285452 | 8 | 8977165 | G | 0.0624 | -0.2393 | 0.05081 | 2.58E-06 |
| ebi-a-GCST90001418 | rs144295598 | 22 | 45164171 | A | 0.0103 | 0.5664 | 0.1203 | 2.60E-06 |
| ebi-a-GCST90001446 | rs144295598 | 22 | 45164171 | A | 0.0103 | 0.5677 | 0.1226 | 3.78E-06 |
| ebi-a-GCST90001407 | rs144295598 | 22 | 45164171 | A | 0.0103 | 0.6102 | 0.1207 | 4.46E-07 |
| ebi-a-GCST90001872 | rs144299393 | 13 | 109533726 | A | 0.0012 | -2.807 | 0.6024 | 3.51E-06 |
| ebi-a-GCST90001401 | rs144306835 | 8 | 96356983 | A | 0.0234 | 0.3826 | 0.07964 | 1.62E-06 |
| ebi-a-GCST90002057 | rs144350282 | 6 | 1689657 | G | 0.0318 | 0.3651 | 0.07697 | 2.21E-06 |
| ebi-a-GCST90001509 | rs144350975 | 1 | 188000439 | A | 0.0253 | 0.4042 | 0.08214 | 9.02E-07 |
| ebi-a-GCST90001507 | rs144350975 | 1 | 188000439 | A | 0.0251 | 0.4366 | 0.08386 | 2.04E-07 |
| ebi-a-GCST90002073 | rs144373670 | 14 | 96537074 | A | 0.0189 | -0.477 | 0.09981 | 1.85E-06 |
| ebi-a-GCST90001699 | rs144378515 | 10 | 60542770 | G | 0.0069 | 9.884 | 2.114 | 3.04E-06 |
| ebi-a-GCST90001766 | rs144378737 | 4 | 144991868 | G | 7.00E-04 | -2.105 | 0.4528 | 3.47E-06 |
| ebi-a-GCST90001498 | rs144404462 | 1 | 198005348 | A | 0.0211 | 0.4385 | 0.08542 | 3.01E-07 |
| ebi-a-GCST90001892 | rs144419206 | 11 | 24126713 | T | 7.00E-04 | -2.461 | 0.4985 | 8.36E-07 |
| ebi-a-GCST90001644 | rs144426024 | 18 | 56897917 | T | 0.0121 | 0.5227 | 0.114 | 4.71E-06 |
| ebi-a-GCST90002078 | rs1444301 | 15 | 86683981 | C | 0.1536 | 0.1773 | 0.03745 | 2.30E-06 |
| ebi-a-GCST90001697 | rs144434471 | 2 | 153006087 | T | 0.001 | 18.09 | 3.474 | 2.02E-07 |
| ebi-a-GCST90001887 | rs144535716 | 18 | 64170750 | C | 0.0019 | -1.382 | 0.3004 | 4.43E-06 |
| ebi-a-GCST90001697 | rs144571871 | 1 | 65133062 | G | 1.00E-04 | 22.03 | 4.696 | 2.82E-06 |
| ebi-a-GCST90001695 | rs144571871 | 1 | 65133062 | G | 1.00E-04 | 725.9 | 100.6 | 6.76E-13 |
| ebi-a-GCST90001732 | rs144574165 | 4 | 163336202 | T | 0.0581 | 0.2567 | 0.05347 | 1.64E-06 |
| ebi-a-GCST90001698 | rs144582155 | 8 | 41997183 | A | 0.0029 | 169.6 | 32.72 | 2.31E-07 |
| ebi-a-GCST90001698 | rs144585837 | 5 | 120363630 | G | 0.0106 | 81.64 | 17.53 | 3.34E-06 |
| ebi-a-GCST90001688 | rs144591335 | 16 | 59010741 | G | 1.00E-04 | 3.946 | 0.6967 | 1.60E-08 |
| ebi-a-GCST90001695 | rs144599349 | 22 | 45887357 | T | 0.0051 | 87.78 | 18.78 | 3.06E-06 |
| ebi-a-GCST90001700 | rs144613377 | 5 | 81311763 | C | 0.0101 | 3.834 | 0.7616 | 5.04E-07 |
| ebi-a-GCST90001698 | rs144613377 | 5 | 81311763 | C | 0.01 | 159.5 | 18.29 | 4.20E-18 |
| ebi-a-GCST90001442 | rs144625305 | 7 | 7871676 | T | 0.0086 | 0.6284 | 0.1342 | 2.95E-06 |
| ebi-a-GCST90001802 | rs144642376 | 3 | 36349691 | G | 0.0718 | 0.2366 | 0.04932 | 1.67E-06 |
| ebi-a-GCST90001798 | rs144642376 | 3 | 36349691 | G | 0.0718 | 0.2632 | 0.04883 | 7.51E-08 |
| ebi-a-GCST90001806 | rs144642376 | 3 | 36349691 | G | 0.0718 | 0.2712 | 0.04922 | 3.82E-08 |
| ebi-a-GCST90001808 | rs144642376 | 3 | 36349691 | G | 0.0718 | 0.2781 | 0.0492 | 1.69E-08 |
| ebi-a-GCST90001620 | rs144674286 | 9 | 101028704 | A | 0.0628 | 0.2474 | 0.05016 | 8.50E-07 |
| ebi-a-GCST90001629 | rs144674286 | 9 | 101028704 | A | 0.0629 | 0.2485 | 0.05156 | 1.50E-06 |
| ebi-a-GCST90002008 | rs144682094 | 7 | 6359771 | A | 6.00E-04 | 2.499 | 0.5457 | 4.84E-06 |
| ebi-a-GCST90001698 | rs144682449 | 12 | 53402638 | A | 0.0062 | 144.1 | 25.27 | 1.30E-08 |
| ebi-a-GCST90001579 | rs144707945 | 11 | 115147742 | C | 0.0025 | -1.202 | 0.244 | 8.75E-07 |
| ebi-a-GCST90001584 | rs144707945 | 11 | 115147742 | C | 0.0025 | -1.147 | 0.2422 | 2.25E-06 |
| ebi-a-GCST90001757 | rs1447085 | 12 | 127798516 | G | 0.6416 | 0.1294 | 0.02572 | 5.12E-07 |
| ebi-a-GCST90001757 | rs1447085 | 12 | 127798516 | G | 0.6416 | 0.1294 | 0.02572 | 5.12E-07 |
| ebi-a-GCST90001695 | rs144711723 | 2 | 145836600 | T | 3.00E-04 | 429 | 74.81 | 1.06E-08 |
| ebi-a-GCST90001692 | rs144716339 | 3 | 131340841 | T | 0.0624 | -0.2404 | 0.05231 | 4.46E-06 |
| ebi-a-GCST90001866 | rs144727554 | 8 | 80078162 | C | 0.0038 | -1.092 | 0.2291 | 1.98E-06 |
| ebi-a-GCST90001723 | rs144743804 | 1 | 207018206 | A | 0.0197 | 0.4457 | 0.08939 | 6.46E-07 |
| ebi-a-GCST90001724 | rs144743804 | 1 | 207018206 | A | 0.0197 | 0.4882 | 0.08965 | 5.50E-08 |
| ebi-a-GCST90001738 | rs144743804 | 1 | 207018206 | A | 0.0197 | 0.4919 | 0.08935 | 3.95E-08 |
| ebi-a-GCST90001730 | rs144743804 | 1 | 207018206 | A | 0.0197 | 0.5079 | 0.0892 | 1.34E-08 |
| ebi-a-GCST90001742 | rs144743804 | 1 | 207018206 | A | 0.0197 | 0.539 | 0.09004 | 2.36E-09 |
| ebi-a-GCST90001729 | rs144743804 | 1 | 207018206 | A | 0.0197 | 0.6138 | 0.09002 | 1.07E-11 |
| ebi-a-GCST90001728 | rs144743804 | 1 | 207018206 | A | 0.0224 | 0.6567 | 0.1158 | 1.65E-08 |
| ebi-a-GCST90001658 | rs144764562 | 19 | 43539305 | A | 4.00E-04 | -2.568 | 0.5373 | 1.82E-06 |
| ebi-a-GCST90001658 | rs144769320 | 10 | 99527224 | T | 0.0417 | -0.291 | 0.06271 | 3.61E-06 |
| ebi-a-GCST90001659 | rs144769320 | 10 | 99527224 | T | 0.0417 | -0.2881 | 0.06272 | 4.52E-06 |
| ebi-a-GCST90002116 | rs144782687 | 4 | 167696884 | G | 0.0041 | 0.9344 | 0.1917 | 1.15E-06 |
| ebi-a-GCST90001688 | rs144788631 | 6 | 145827461 | T | 1.00E-04 | 3.533 | 0.6507 | 6.06E-08 |
| ebi-a-GCST90002017 | rs144804416 | 3 | 46861400 | T | 0.0289 | -0.4442 | 0.08419 | 1.42E-07 |
| ebi-a-GCST90002105 | rs144816332 | 14 | 67537526 | A | 0.0057 | -0.8192 | 0.1791 | 4.97E-06 |
| ebi-a-GCST90002006 | rs144823175 | 1 | 149924994 | G | 0.0133 | 0.8668 | 0.1115 | 1.01E-14 |
| ebi-a-GCST90001987 | rs144823175 | 1 | 149924994 | G | 0.0133 | 0.8884 | 0.1109 | 1.51E-15 |
| ebi-a-GCST90002072 | rs144843975 | 17 | 63066415 | T | 0.0519 | 0.2991 | 0.06248 | 1.78E-06 |
| ebi-a-GCST90001964 | rs144843975 | 17 | 63066415 | T | 0.0519 | 0.3683 | 0.06208 | 3.33E-09 |
| ebi-a-GCST90001776 | rs144856909 | 22 | 29988779 | A | 0.0496 | 0.2806 | 0.0571 | 9.34E-07 |
| ebi-a-GCST90001975 | rs144869562 | 7 | 33734811 | G | 5.00E-04 | 3.046 | 0.617 | 8.35E-07 |
| ebi-a-GCST90001525 | rs144894656 | 9 | 118222268 | C | 0.0094 | -0.7728 | 0.1667 | 3.78E-06 |
| ebi-a-GCST90001695 | rs144920942 | 6 | 104569120 | T | 0.0016 | 151.3 | 32.96 | 4.58E-06 |
| ebi-a-GCST90001741 | rs1449311 | 3 | 100703124 | A | 0.3074 | 0.1247 | 0.02709 | 4.33E-06 |
| ebi-a-GCST90001737 | rs1449311 | 3 | 100703124 | A | 0.3073 | 0.1261 | 0.027 | 3.10E-06 |
| ebi-a-GCST90001725 | rs1449311 | 3 | 100703124 | A | 0.3074 | 0.1265 | 0.02699 | 2.90E-06 |
| ebi-a-GCST90001993 | rs144941477 | 19 | 47853865 | T | 0.0012 | 1.564 | 0.3409 | 4.63E-06 |
| ebi-a-GCST90001784 | rs144941792 | 10 | 5777776 | T | 0.1001 | 0.2117 | 0.04191 | 4.62E-07 |
| ebi-a-GCST90002045 | rs144954155 | 3 | 177691462 | T | 0.0431 | -0.4271 | 0.09032 | 2.45E-06 |
| ebi-a-GCST90001695 | rs144967884 | 5 | 142943186 | T | NA | 1142 | 209.6 | 5.48E-08 |
| ebi-a-GCST90001695 | rs144993401 | 12 | 38374287 | A | NA | 1279 | 227.4 | 2.03E-08 |
| ebi-a-GCST90001649 | rs145013190 | 8 | 98791630 | A | 0.0209 | -0.4439 | 0.08931 | 7.00E-07 |
| ebi-a-GCST90001650 | rs145013190 | 8 | 98791630 | A | 0.0209 | -0.4035 | 0.08761 | 4.25E-06 |
| ebi-a-GCST90001691 | rs1450147 | 4 | 183111829 | T | 0.7064 | -0.1281 | 0.02787 | 4.42E-06 |
| ebi-a-GCST90001652 | rs145016200 | 14 | 101601125 | T | 0.0496 | -0.2627 | 0.057 | 4.18E-06 |
| ebi-a-GCST90001698 | rs145021762 | 10 | 22411454 | A | 0.0207 | 77.03 | 13.23 | 6.37E-09 |
| ebi-a-GCST90001695 | rs145022527 | 13 | 37118716 | T | 0.0013 | 162.1 | 34.1 | 2.09E-06 |
| ebi-a-GCST90001698 | rs145057921 | 7 | 6231210 | T | 0.0026 | 155 | 33.08 | 2.88E-06 |
| ebi-a-GCST90001406 | rs145123647 | 10 | 26959594 | T | 0.003 | -1.082 | 0.2244 | 1.49E-06 |
| ebi-a-GCST90001408 | rs145123647 | 10 | 26959594 | T | 0.003 | 1.077 | 0.2247 | 1.72E-06 |
| ebi-a-GCST90002012 | rs145135505 | 3 | 38928831 | C | 0.0667 | -0.2254 | 0.04869 | 3.79E-06 |
| ebi-a-GCST90001413 | rs145137909 | 2 | 215618238 | A | 4.00E-04 | -2.77 | 0.5672 | 1.09E-06 |
| ebi-a-GCST90001687 | rs1451433 | 4 | 179935752 | C | 0.3885 | -0.1221 | 0.02604 | 2.83E-06 |
| ebi-a-GCST90001500 | rs145165211 | 8 | 21371903 | A | 0.0167 | -0.5131 | 0.1022 | 5.39E-07 |
| ebi-a-GCST90001695 | rs145174666 | 6 | 6745192 | A | 0.0129 | 52.78 | 11.43 | 4.04E-06 |
| ebi-a-GCST90001525 | rs145231100 | 18 | 33141890 | A | 0.0137 | -0.6579 | 0.1354 | 1.28E-06 |
| ebi-a-GCST90001698 | rs145250558 | 20 | 15245578 | C | 0.0012 | 378.7 | 48.86 | 1.18E-14 |
| ebi-a-GCST90001698 | rs145263449 | 20 | 12493170 | A | 0.0028 | 169.2 | 33.84 | 6.05E-07 |
| ebi-a-GCST90001695 | rs145266404 | 5 | 172300229 | C | 7.00E-04 | 240 | 48.24 | 6.85E-07 |
| ebi-a-GCST90001899 | rs145288523 | 9 | 21854434 | T | 7.00E-04 | -2.144 | 0.4613 | 3.53E-06 |
| ebi-a-GCST90002049 | rs145290654 | 3 | 46613040 | A | 0.0204 | 0.6077 | 0.1319 | 4.39E-06 |
| ebi-a-GCST90001522 | rs145308006 | 6 | 103221111 | G | 0.0102 | -0.8101 | 0.1738 | 3.37E-06 |
| ebi-a-GCST90001830 | rs145320860 | 13 | 62765406 | A | 0.0077 | -0.6925 | 0.1429 | 1.32E-06 |
| ebi-a-GCST90001459 | rs145330472 | 9 | 31374800 | A | 0.0265 | -0.3648 | 0.07932 | 4.40E-06 |
| ebi-a-GCST90001633 | rs145331774 | 6 | 402147 | T | 0.0227 | -0.3842 | 0.08337 | 4.20E-06 |
| ebi-a-GCST90001695 | rs145336406 | 6 | 78745175 | T | 0.0026 | 148.2 | 25.16 | 4.22E-09 |
| ebi-a-GCST90001698 | rs145336406 | 6 | 78745175 | T | 0.0026 | 186.1 | 33.82 | 4.06E-08 |
| ebi-a-GCST90001731 | rs1453509 | 16 | 17016084 | A | 0.1181 | -0.1879 | 0.03799 | 7.90E-07 |
| ebi-a-GCST90001398 | rs145371905 | 13 | 70325772 | T | 0.0194 | 0.4126 | 0.08871 | 3.43E-06 |
| ebi-a-GCST90001412 | rs145371905 | 13 | 70325772 | T | 0.0194 | 0.4292 | 0.08767 | 1.02E-06 |
| ebi-a-GCST90001814 | rs145388152 | 6 | 4347705 | A | 0.001 | 1.935 | 0.3853 | 5.34E-07 |
| ebi-a-GCST90001725 | rs145392098 | 2 | 95682916 | A | 0.0272 | 0.3549 | 0.07695 | 4.11E-06 |
| ebi-a-GCST90001502 | rs145406940 | 12 | 23093493 | A | 0.001 | 1.967 | 0.4233 | 3.51E-06 |
| ebi-a-GCST90001700 | rs145410896 | 19 | 34111992 | G | 0.005 | 5.395 | 1.046 | 2.66E-07 |
| ebi-a-GCST90001698 | rs145410896 | 19 | 34111992 | G | 0.005 | 202.5 | 24.61 | 2.68E-16 |
| ebi-a-GCST90001969 | rs145419574 | 6 | 17386586 | T | 0.0029 | 1.098 | 0.2386 | 4.30E-06 |
| ebi-a-GCST90001711 | rs145423893 | 15 | 28254280 | C | 0.0055 | -0.82 | 0.1702 | 1.50E-06 |
| ebi-a-GCST90001856 | rs145438716 | 3 | 127159039 | G | 0.0303 | 0.3752 | 0.08068 | 3.46E-06 |
| ebi-a-GCST90001417 | rs145442262 | 3 | 186702869 | A | 0.0906 | -0.2083 | 0.04276 | 1.15E-06 |
| ebi-a-GCST90001503 | rs145444215 | 14 | 103258906 | A | 9.00E-04 | -1.986 | 0.4154 | 1.83E-06 |
| ebi-a-GCST90001583 | rs145530955 | 7 | 65604757 | T | 0.0285 | -0.332 | 0.07238 | 4.65E-06 |
| ebi-a-GCST90001522 | rs145540967 | 19 | 31193989 | G | 0.0038 | -1.312 | 0.2783 | 2.61E-06 |
| ebi-a-GCST90002030 | rs145557266 | 10 | 95850055 | G | 0.0211 | 0.569 | 0.09629 | 3.84E-09 |
| ebi-a-GCST90001658 | rs145557266 | 10 | 95850055 | G | 0.0211 | 0.6746 | 0.08639 | 7.61E-15 |
| ebi-a-GCST90001497 | rs145557266 | 10 | 95850055 | G | 0.0211 | 0.7299 | 0.08928 | 4.09E-16 |
| ebi-a-GCST90001496 | rs145557266 | 10 | 95850055 | G | 0.0211 | 0.7359 | 0.09042 | 5.55E-16 |
| ebi-a-GCST90001659 | rs145557266 | 10 | 95850055 | G | 0.0211 | 0.7452 | 0.08624 | 8.40E-18 |
| ebi-a-GCST90001660 | rs145557266 | 10 | 95850055 | G | 0.021 | 0.7936 | 0.09034 | 2.46E-18 |
| ebi-a-GCST90001698 | rs145563511 | 9 | 106801353 | G | 0.0031 | 185.9 | 33.18 | 2.28E-08 |
| ebi-a-GCST90001501 | rs145600407 | 13 | 19280601 | T | 0.0206 | -0.5371 | 0.1044 | 2.85E-07 |
| ebi-a-GCST90001492 | rs145600407 | 13 | 19280601 | T | 0.0206 | -0.5134 | 0.1045 | 9.43E-07 |
| ebi-a-GCST90001513 | rs145600407 | 13 | 19280601 | T | 0.0206 | -0.4993 | 0.1023 | 1.10E-06 |
| ebi-a-GCST90001504 | rs145600407 | 13 | 19280601 | T | 0.0206 | -0.4767 | 0.1041 | 4.82E-06 |
| ebi-a-GCST90001700 | rs145604620 | 6 | 11491250 | G | 3.00E-04 | 34.45 | 4.832 | 1.22E-12 |
| ebi-a-GCST90001698 | rs145604620 | 6 | 11491250 | G | 3.00E-04 | 1994 | 108.9 | 1.48E-71 |
| ebi-a-GCST90001697 | rs145613913 | 6 | 70855865 | A | 9.00E-04 | 11.66 | 2.24 | 2.03E-07 |
| ebi-a-GCST90001696 | rs145613913 | 6 | 70855865 | A | 9.00E-04 | 26.75 | 5.484 | 1.12E-06 |
| ebi-a-GCST90001695 | rs145613913 | 6 | 70855865 | A | 9.00E-04 | 281.9 | 47.31 | 2.80E-09 |
| ebi-a-GCST90002081 | rs145630857 | 2 | 1273732 | A | 0.1237 | -0.185 | 0.0391 | 2.34E-06 |
| ebi-a-GCST90002046 | rs145631483 | 8 | 4517297 | A | 6.00E-04 | 3.13 | 0.6817 | 4.74E-06 |
| ebi-a-GCST90001414 | rs145638783 | 6 | 1238442 | A | 0.0033 | -0.9851 | 0.2144 | 4.47E-06 |
| ebi-a-GCST90001800 | rs145656540 | 20 | 57966338 | T | 0.0152 | 0.486 | 0.1009 | 1.51E-06 |
| ebi-a-GCST90001665 | rs145682498 | 10 | 54508949 | A | 0.0593 | -0.2425 | 0.05206 | 3.30E-06 |
| ebi-a-GCST90001596 | rs145682498 | 10 | 54508949 | A | 0.059 | 0.2353 | 0.05119 | 4.45E-06 |
| ebi-a-GCST90001698 | rs145720280 | 13 | 43404037 | C | 0.0031 | 202.4 | 32.76 | 7.31E-10 |
| ebi-a-GCST90001697 | rs145744161 | 10 | 108259029 | T | 0.0438 | 1.416 | 0.3046 | 3.44E-06 |
| ebi-a-GCST90001695 | rs145744161 | 10 | 108259029 | T | 0.0439 | 32.56 | 6.516 | 6.13E-07 |
| ebi-a-GCST90002064 | rs145783964 | 6 | 169727141 | A | 0.4829 | -0.1347 | 0.02731 | 8.60E-07 |
| ebi-a-GCST90001849 | rs145799392 | 1 | 30362673 | T | 0.0364 | -0.3383 | 0.07098 | 1.96E-06 |
| ebi-a-GCST90001688 | rs145827119 | 20 | 57132122 | T | 1.00E-04 | 4.893 | 0.8386 | 5.91E-09 |
| ebi-a-GCST90001666 | rs145827119 | 20 | 57132122 | T | 1.00E-04 | 5.042 | 1.068 | 2.46E-06 |
| ebi-a-GCST90001987 | rs145828126 | 1 | 120233380 | A | 0.0478 | -0.3359 | 0.05863 | 1.09E-08 |
| ebi-a-GCST90002006 | rs145828126 | 1 | 120233380 | A | 0.0478 | -0.3227 | 0.05896 | 4.73E-08 |
| ebi-a-GCST90001482 | rs145833948 | 12 | 41804479 | A | 0.0425 | 0.2778 | 0.05849 | 2.12E-06 |
| ebi-a-GCST90001698 | rs145835734 | 10 | 66659421 | G | 4.00E-04 | 479.2 | 79.88 | 2.19E-09 |
| ebi-a-GCST90001836 | rs145836694 | 6 | 106222810 | C | 0.0242 | -0.5523 | 0.1189 | 3.72E-06 |
| ebi-a-GCST90001688 | rs145865638 | 17 | 2098010 | T | NA | 12.72 | 2.366 | 8.24E-08 |
| ebi-a-GCST90001677 | rs145868945 | 16 | 54688102 | T | 0.0043 | -0.8804 | 0.1898 | 3.66E-06 |
| ebi-a-GCST90001740 | rs145924813 | 1 | 28617041 | T | 0.0327 | 0.3277 | 0.07109 | 4.15E-06 |
| ebi-a-GCST90001735 | rs145924813 | 1 | 28617041 | T | 0.0327 | 0.3435 | 0.07069 | 1.23E-06 |
| ebi-a-GCST90001941 | rs145947216 | 2 | 38596674 | T | 0.036 | 0.3369 | 0.0715 | 2.56E-06 |
| ebi-a-GCST90001939 | rs145947216 | 2 | 38596674 | T | 0.036 | 0.342 | 0.07099 | 1.52E-06 |
| ebi-a-GCST90002103 | rs145947216 | 2 | 38596674 | T | 0.0375 | 0.3763 | 0.07577 | 7.27E-07 |
| ebi-a-GCST90002098 | rs145947216 | 2 | 38596674 | T | 0.0375 | 0.7147 | 0.07055 | 9.98E-24 |
| ebi-a-GCST90001698 | rs145978106 | 18 | 7839215 | A | 0.006 | 207 | 22.8 | 1.80E-19 |
| ebi-a-GCST90001718 | rs146069723 | 22 | 43339331 | T | 0.0756 | -0.5915 | 0.04776 | 1.54E-34 |
| ebi-a-GCST90001710 | rs146069723 | 22 | 43339331 | T | 0.0756 | -0.5779 | 0.0476 | 2.82E-33 |
| ebi-a-GCST90001702 | rs146069723 | 22 | 43339331 | T | 0.0756 | -0.574 | 0.04773 | 1.06E-32 |
| ebi-a-GCST90001715 | rs146069723 | 22 | 43339331 | T | 0.0756 | -0.5694 | 0.04769 | 2.95E-32 |
| ebi-a-GCST90001711 | rs146069723 | 22 | 43339331 | T | 0.0756 | -0.5638 | 0.04748 | 6.25E-32 |
| ebi-a-GCST90001717 | rs146069723 | 22 | 43339331 | T | 0.0756 | -0.5628 | 0.04773 | 1.58E-31 |
| ebi-a-GCST90001707 | rs146069723 | 22 | 43339331 | T | 0.0762 | -0.5625 | 0.06459 | 6.78E-18 |
| ebi-a-GCST90001713 | rs146069723 | 22 | 43339331 | T | 0.0756 | -0.2315 | 0.04704 | 8.99E-07 |
| ebi-a-GCST90001479 | rs146090095 | 14 | 25035095 | T | 0.0708 | -0.2506 | 0.04997 | 5.54E-07 |
| ebi-a-GCST90001698 | rs146101423 | 19 | 6753000 | A | 7.00E-04 | 417.2 | 62.38 | 2.64E-11 |
| ebi-a-GCST90002110 | rs1461277 | 3 | 174918975 | G | 0.4988 | 0.1774 | 0.03589 | 8.56E-07 |
| ebi-a-GCST90001695 | rs146127940 | 17 | 29929827 | A | 0.0022 | 145.9 | 28.52 | 3.34E-07 |
| ebi-a-GCST90001698 | rs146135726 | 5 | 154015729 | T | 0.0016 | 235.3 | 42.7 | 3.85E-08 |
| ebi-a-GCST90002097 | rs146147816 | 14 | 73295385 | A | 0.0067 | 1.104 | 0.2236 | 8.81E-07 |
| ebi-a-GCST90001695 | rs146164275 | 17 | 76005958 | G | 9.00E-04 | 262.3 | 44.92 | 5.71E-09 |
| ebi-a-GCST90001700 | rs146171997 | 5 | 52147101 | G | 1.00E-04 | 35.54 | 7.417 | 1.73E-06 |
| ebi-a-GCST90001698 | rs146204142 | 7 | 112317649 | A | 0.0044 | 131.3 | 26.82 | 1.02E-06 |
| ebi-a-GCST90001698 | rs146217030 | 2 | 41343706 | C | 0.005 | 143.9 | 26.11 | 3.78E-08 |
| ebi-a-GCST90001698 | rs146252263 | 11 | 2477438 | T | 0.0082 | 100 | 19.92 | 5.37E-07 |
| ebi-a-GCST90001785 | rs146277285 | 10 | 107243367 | T | 0.0221 | 0.3946 | 0.08332 | 2.26E-06 |
| ebi-a-GCST90001698 | rs146314225 | 5 | 141469416 | C | 0.0018 | 225.4 | 40.79 | 3.49E-08 |
| ebi-a-GCST90001697 | rs146319541 | 11 | 16659699 | A | 9.00E-04 | 10.25 | 2.165 | 2.29E-06 |
| ebi-a-GCST90001697 | rs146323263 | 2 | 87376401 | T | 0.0016 | 6.561 | 1.381 | 2.10E-06 |
| ebi-a-GCST90001541 | rs146329634 | 14 | 89433599 | T | 0.0328 | -0.3196 | 0.06393 | 6.03E-07 |
| ebi-a-GCST90001575 | rs146329634 | 14 | 89433599 | T | 0.0328 | -0.3103 | 0.0671 | 3.89E-06 |
| ebi-a-GCST90001535 | rs146329634 | 14 | 89433599 | T | 0.0328 | 0.3128 | 0.06543 | 1.82E-06 |
| ebi-a-GCST90001842 | rs146330992 | 1 | 165888466 | T | 0.0844 | 0.2517 | 0.04997 | 5.05E-07 |
| ebi-a-GCST90001578 | rs146347064 | 16 | 8263526 | A | 0.0187 | -0.4087 | 0.08671 | 2.52E-06 |
| ebi-a-GCST90001768 | rs146379662 | 2 | 240297559 | T | 0.0111 | 0.5365 | 0.1154 | 3.47E-06 |
| ebi-a-GCST90001816 | rs146407097 | 9 | 34935516 | A | 0.0613 | -0.2522 | 0.05285 | 1.89E-06 |
| ebi-a-GCST90001496 | rs146426185 | 10 | 99452494 | C | 0.0634 | -0.3085 | 0.05356 | 9.17E-09 |
| ebi-a-GCST90001497 | rs146426185 | 10 | 99452494 | C | 0.0634 | -0.3048 | 0.05289 | 8.96E-09 |
| ebi-a-GCST90001671 | rs146426185 | 10 | 99452494 | C | 0.0635 | -0.3008 | 0.05314 | 1.64E-08 |
| ebi-a-GCST90001672 | rs146426185 | 10 | 99452494 | C | 0.064 | -0.2939 | 0.05387 | 5.24E-08 |
| ebi-a-GCST90001397 | rs146430312 | 1 | 236060214 | T | 0.0027 | 1.017 | 0.2222 | 4.87E-06 |
| ebi-a-GCST90001698 | rs146461857 | 22 | 23709958 | A | 0.0013 | 355.7 | 52.34 | 1.26E-11 |
| ebi-a-GCST90002035 | rs146503240 | 17 | 6496912 | T | 0.0453 | 0.3095 | 0.06674 | 3.70E-06 |
| ebi-a-GCST90002036 | rs146503240 | 17 | 6496912 | T | 0.0453 | 0.3138 | 0.06679 | 2.76E-06 |
| ebi-a-GCST90001911 | rs146515193 | 15 | 69715791 | T | 0.0064 | 0.8128 | 0.1769 | 4.53E-06 |
| ebi-a-GCST90001869 | rs146597227 | 2 | 57424952 | T | 0.091 | -0.2189 | 0.04747 | 4.15E-06 |
| ebi-a-GCST90001692 | rs146620586 | 1 | 198524264 | T | 0.0032 | -2.596 | 0.2832 | 8.26E-20 |
| ebi-a-GCST90001535 | rs146620586 | 1 | 198524264 | T | 0.0032 | -2.021 | 0.2655 | 3.46E-14 |
| ebi-a-GCST90001536 | rs146620586 | 1 | 198524264 | T | 0.0032 | -1.991 | 0.2693 | 1.78E-13 |
| ebi-a-GCST90001544 | rs146620586 | 1 | 198524264 | T | 0.0032 | -1.711 | 0.2639 | 1.02E-10 |
| ebi-a-GCST90001543 | rs146620586 | 1 | 198524264 | T | 0.0032 | -1.617 | 0.2699 | 2.30E-09 |
| ebi-a-GCST90001562 | rs146620586 | 1 | 198524264 | T | 0.0032 | 1.455 | 0.2533 | 1.00E-08 |
| ebi-a-GCST90001541 | rs146620586 | 1 | 198524264 | T | 0.0032 | 1.495 | 0.2604 | 1.01E-08 |
| ebi-a-GCST90001559 | rs146620586 | 1 | 198524264 | T | 0.0032 | 1.574 | 0.2873 | 4.57E-08 |
| ebi-a-GCST90001699 | rs146620586 | 1 | 198524264 | T | 0.0032 | 18.78 | 4.013 | 2.97E-06 |
| ebi-a-GCST90001700 | rs146627868 | 2 | 81365670 | A | 0.0147 | 2.823 | 0.6163 | 4.79E-06 |
| ebi-a-GCST90001700 | rs146650864 | 19 | 22454852 | A | 0.002 | 9.123 | 1.757 | 2.19E-07 |
| ebi-a-GCST90001698 | rs146650864 | 19 | 22454852 | A | 0.0021 | 338.4 | 41.59 | 5.64E-16 |
| ebi-a-GCST90001698 | rs146661200 | 12 | 100494123 | T | 0.0056 | 152.8 | 23.81 | 1.58E-10 |
| ebi-a-GCST90001697 | rs146663516 | 20 | 35238641 | T | 0.0018 | 7.394 | 1.479 | 6.08E-07 |
| ebi-a-GCST90002089 | rs146665828 | 8 | 15677874 | A | 0.0484 | -0.3202 | 0.0651 | 9.19E-07 |
| ebi-a-GCST90001396 | rs146687492 | 11 | 125619694 | T | 0.0291 | -0.3372 | 0.07361 | 4.77E-06 |
| ebi-a-GCST90001784 | rs146720368 | 22 | 32003463 | T | 0.0042 | 0.9178 | 0.1934 | 2.15E-06 |
| ebi-a-GCST90001794 | rs146720368 | 22 | 32003463 | T | 0.0042 | 0.9262 | 0.1929 | 1.64E-06 |
| ebi-a-GCST90002094 | rs146739860 | 5 | 174996664 | G | 0.0159 | -0.7107 | 0.1485 | 1.87E-06 |
| ebi-a-GCST90001689 | rs146741472 | 2 | 120215812 | A | 1.00E-04 | 3.535 | 0.7519 | 2.70E-06 |
| ebi-a-GCST90001498 | rs146746694 | 6 | 155566192 | A | 0.0101 | 0.5612 | 0.121 | 3.65E-06 |
| ebi-a-GCST90001698 | rs146756366 | 3 | 76660068 | T | 0.0018 | 313.5 | 41.76 | 7.66E-14 |
| ebi-a-GCST90001976 | rs146765347 | 11 | 11413255 | T | 0.0495 | -0.3331 | 0.06077 | 4.55E-08 |
| ebi-a-GCST90001841 | rs146780525 | 1 | 26780027 | T | 0.0469 | -0.3063 | 0.06557 | 3.12E-06 |
| ebi-a-GCST90002108 | rs146788953 | 3 | 19007547 | A | 0.0345 | -0.481 | 0.1005 | 1.86E-06 |
| ebi-a-GCST90001751 | rs146797770 | 17 | 6772746 | C | 0.0179 | 0.4285 | 0.0922 | 3.48E-06 |
| ebi-a-GCST90001700 | rs146822590 | 12 | 130060949 | T | 9.00E-04 | 14.19 | 2.379 | 2.68E-09 |
| ebi-a-GCST90001698 | rs146822590 | 12 | 130060949 | T | 9.00E-04 | 642.9 | 54.5 | 1.66E-31 |
| ebi-a-GCST90002033 | rs146832619 | 1 | 182145692 | T | 9.00E-04 | -2.209 | 0.4706 | 2.82E-06 |
| ebi-a-GCST90001471 | rs146839224 | 10 | 131367364 | A | 0.0025 | 1.182 | 0.2447 | 1.42E-06 |
| ebi-a-GCST90001641 | rs146849959 | 4 | 90761054 | A | 0.0194 | 0.4251 | 0.0928 | 4.79E-06 |
| ebi-a-GCST90002026 | rs146861616 | 8 | 8216308 | G | 0.0515 | 0.321 | 0.06123 | 1.69E-07 |
| ebi-a-GCST90002105 | rs146863538 | 19 | 19396439 | C | 0.001 | 1.974 | 0.4037 | 1.07E-06 |
| ebi-a-GCST90001991 | rs146897285 | 1 | 213984208 | A | 0.0034 | 0.9514 | 0.2078 | 4.85E-06 |
| ebi-a-GCST90001988 | rs146897285 | 1 | 213984208 | A | 0.0034 | 0.9798 | 0.2073 | 2.36E-06 |
| ebi-a-GCST90001697 | rs146914701 | 10 | 109409332 | A | 0.0136 | 2.78 | 0.5423 | 3.13E-07 |
| ebi-a-GCST90001695 | rs146914701 | 10 | 109409332 | A | 0.0135 | 61.08 | 11.67 | 1.77E-07 |
| ebi-a-GCST90001426 | rs146936090 | 6 | 41401285 | T | 8.00E-04 | 1.843 | 0.3841 | 1.66E-06 |
| ebi-a-GCST90001672 | rs146940094 | 15 | 94946993 | T | 7.00E-04 | 2.067 | 0.4485 | 4.21E-06 |
| ebi-a-GCST90001695 | rs146940094 | 15 | 94946993 | T | 7.00E-04 | 285.3 | 47.21 | 1.66E-09 |
| ebi-a-GCST90001695 | rs146954403 | 17 | 58220407 | C | NA | 1169 | 246.4 | 2.16E-06 |
| ebi-a-GCST90001392 | rs146959444 | 1 | 167027268 | C | 0.0023 | 1.249 | 0.2574 | 1.29E-06 |
| ebi-a-GCST90002102 | rs146962738 | 8 | 122930949 | G | 0.0012 | 1.893 | 0.3655 | 2.38E-07 |
| ebi-a-GCST90002011 | rs146963227 | 4 | 17530633 | A | 0.015 | -0.5728 | 0.1006 | 1.35E-08 |
| ebi-a-GCST90001989 | rs146963227 | 4 | 17530633 | A | 0.0151 | -0.5007 | 0.1014 | 8.23E-07 |
| ebi-a-GCST90001990 | rs146963227 | 4 | 17530633 | A | 0.0149 | -0.4704 | 0.1015 | 3.74E-06 |
| ebi-a-GCST90001561 | rs146969910 | 5 | 171664213 | C | 0.0267 | -0.3103 | 0.06712 | 3.94E-06 |
| ebi-a-GCST90001555 | rs146969910 | 5 | 171664213 | C | 0.0267 | 0.3142 | 0.06689 | 2.75E-06 |
| ebi-a-GCST90001673 | rs146970469 | 2 | 30292698 | A | 0.0501 | -0.2637 | 0.05741 | 4.54E-06 |
| ebi-a-GCST90001698 | rs146982369 | 15 | 25983333 | G | 0.0213 | 57.45 | 12.21 | 2.62E-06 |
| ebi-a-GCST90001690 | rs146985291 | 3 | 170169608 | G | 1.00E-04 | 5.307 | 1.132 | 2.88E-06 |
| ebi-a-GCST90001688 | rs146985291 | 3 | 170169608 | G | 1.00E-04 | 6.493 | 1.126 | 8.69E-09 |
| ebi-a-GCST90001695 | rs146986354 | 15 | 29116366 | T | 0.0013 | 216.5 | 40.13 | 7.27E-08 |
| ebi-a-GCST90001698 | rs146989801 | 19 | 45630360 | G | 0.0026 | 187.4 | 33.91 | 3.51E-08 |
| ebi-a-GCST90001695 | rs147014864 | 5 | 22132055 | T | 0.0018 | 164.9 | 31.9 | 2.47E-07 |
| ebi-a-GCST90001698 | rs147015694 | 18 | 2896235 | A | 0.0069 | 99.23 | 21.38 | 3.60E-06 |
| ebi-a-GCST90001698 | rs147026949 | 4 | 100588891 | T | 0.0032 | 181.2 | 33.13 | 4.82E-08 |
| ebi-a-GCST90001698 | rs147069965 | 17 | 36231492 | T | 0.0029 | 184 | 34.62 | 1.14E-07 |
| ebi-a-GCST90001487 | rs147071297 | 1 | 191648600 | A | 0.0039 | -0.964 | 0.1968 | 1.02E-06 |
| ebi-a-GCST90001482 | rs147071297 | 1 | 191648600 | A | 0.0039 | 1.123 | 0.1849 | 1.40E-09 |
| ebi-a-GCST90001481 | rs147071297 | 1 | 191648600 | A | 0.0039 | 1.292 | 0.1788 | 6.14E-13 |
| ebi-a-GCST90002098 | rs147071297 | 1 | 191648600 | A | 0.0036 | 1.525 | 0.2126 | 9.25E-13 |
| ebi-a-GCST90001700 | rs147093663 | 15 | 81422902 | T | 1.00E-04 | 42.63 | 7.413 | 9.65E-09 |
| ebi-a-GCST90002007 | rs147095653 | 3 | 42422119 | T | 0.0138 | -0.5013 | 0.107 | 2.87E-06 |
| ebi-a-GCST90001700 | rs147107195 | 12 | 48190275 | T | 3.00E-04 | 23.9 | 4.049 | 3.92E-09 |
| ebi-a-GCST90001698 | rs147107195 | 12 | 48190275 | T | 3.00E-04 | 851.2 | 92.57 | 6.37E-20 |
| ebi-a-GCST90002030 | rs147121076 | 10 | 95924521 | G | 0.0286 | 0.3983 | 0.08447 | 2.52E-06 |
| ebi-a-GCST90001496 | rs147121076 | 10 | 95924521 | G | 0.0262 | 0.4594 | 0.08235 | 2.63E-08 |
| ebi-a-GCST90001497 | rs147121076 | 10 | 95924521 | G | 0.0262 | 0.4681 | 0.0813 | 9.29E-09 |
| ebi-a-GCST90001698 | rs147136626 | 18 | 73901698 | T | 0.0041 | 160.7 | 28.09 | 1.16E-08 |
| ebi-a-GCST90001764 | rs1471412 | 4 | 131810654 | TRUE | 0.8537 | -0.1618 | 0.03443 | 2.71E-06 |
| ebi-a-GCST90001797 | rs147153707 | 9 | 31165407 | A | 0.0129 | 0.5283 | 0.11 | 1.64E-06 |
| ebi-a-GCST90001480 | rs147199078 | 7 | 80542211 | C | 0.0295 | 0.3441 | 0.06816 | 4.69E-07 |
| ebi-a-GCST90001498 | rs147199078 | 7 | 80542211 | C | 0.0295 | 0.354 | 0.07196 | 9.09E-07 |
| ebi-a-GCST90002099 | rs147201746 | 2 | 15706963 | A | 0.0126 | -0.6008 | 0.1219 | 8.72E-07 |
| ebi-a-GCST90001394 | rs147207149 | 5 | 128786753 | G | 0.0075 | 0.6732 | 0.1407 | 1.78E-06 |
| ebi-a-GCST90001662 | rs147207959 | 4 | 182642975 | T | 0.0023 | -1.192 | 0.2451 | 1.22E-06 |
| ebi-a-GCST90002116 | rs147210938 | 6 | 13892767 | A | 0.0332 | -0.4296 | 0.07489 | 1.06E-08 |
| ebi-a-GCST90001585 | rs147218533 | 3 | 167152276 | C | 0.0164 | 0.4909 | 0.09721 | 4.64E-07 |
| ebi-a-GCST90001697 | rs147264423 | 15 | 78644995 | G | 3.00E-04 | 19.07 | 3.318 | 9.78E-09 |
| ebi-a-GCST90002013 | rs147275564 | 4 | 135382945 | C | 3.00E-04 | -3.383 | 0.6452 | 1.69E-07 |
| ebi-a-GCST90001470 | rs1472757 | 5 | 6455872 | C | 0.6057 | -0.1252 | 0.02495 | 5.45E-07 |
| ebi-a-GCST90002120 | rs147279479 | 7 | 145302741 | A | 0.036 | -0.3424 | 0.07343 | 3.26E-06 |
| ebi-a-GCST90001692 | rs147285635 | 3 | 84498712 | A | 3.00E-04 | -3.123 | 0.6744 | 3.77E-06 |
| ebi-a-GCST90001516 | rs147302158 | 9 | 32093300 | G | 0.0369 | -0.4335 | 0.09126 | 2.19E-06 |
| ebi-a-GCST90001420 | rs147319637 | 11 | 111211623 | A | 0.0094 | -0.5657 | 0.1224 | 3.92E-06 |
| ebi-a-GCST90001886 | rs147329602 | 19 | 6350406 | C | 0.0241 | -0.4147 | 0.0846 | 1.00E-06 |
| ebi-a-GCST90001576 | rs1473575 | 1 | 242619029 | T | 0.4013 | 0.1162 | 0.02482 | 2.95E-06 |
| ebi-a-GCST90001698 | rs147370318 | 19 | 31892272 | G | 0.0089 | 97.34 | 19.04 | 3.37E-07 |
| ebi-a-GCST90001880 | rs147371997 | 19 | 36235084 | G | 0.0096 | 0.9881 | 0.2118 | 3.43E-06 |
| ebi-a-GCST90001878 | rs147371997 | 19 | 36235084 | G | 0.0096 | 0.9888 | 0.2139 | 4.19E-06 |
| ebi-a-GCST90001698 | rs147381303 | 13 | 28258062 | A | NA | 1449 | 295.6 | 9.98E-07 |
| ebi-a-GCST90001695 | rs147385696 | 6 | 143880059 | G | 6.00E-04 | 326.5 | 55.73 | 5.13E-09 |
| ebi-a-GCST90001804 | rs147388542 | 9 | 94318432 | C | 0.0055 | 0.746 | 0.1629 | 4.83E-06 |
| ebi-a-GCST90001802 | rs147388542 | 9 | 94318432 | C | 0.0055 | 0.8406 | 0.1653 | 3.84E-07 |
| ebi-a-GCST90001773 | rs147397792 | 10 | 18427081 | G | 0.091 | 0.2079 | 0.04446 | 3.04E-06 |
| ebi-a-GCST90001695 | rs147398419 | 13 | 23525796 | T | 0.0157 | 51.71 | 10.96 | 2.49E-06 |
| ebi-a-GCST90001681 | rs147417158 | 2 | 28165568 | G | 0.0157 | -0.5137 | 0.108 | 2.06E-06 |
| ebi-a-GCST90001977 | rs147422381 | 15 | 38054883 | A | 0.0154 | -0.496 | 0.1058 | 2.90E-06 |
| ebi-a-GCST90001695 | rs147427289 | 2 | 40489896 | A | 7.00E-04 | 270.7 | 56.22 | 1.54E-06 |
| ebi-a-GCST90002112 | rs1474618 | 6 | 18655570 | A | 0.6101 | -0.1723 | 0.03685 | 3.19E-06 |
| ebi-a-GCST90001937 | rs147487472 | 10 | 6191376 | A | 0.0396 | 0.2997 | 0.06421 | 3.17E-06 |
| ebi-a-GCST90001785 | rs147487472 | 10 | 6191376 | A | 0.0412 | 0.3278 | 0.06162 | 1.10E-07 |
| ebi-a-GCST90001794 | rs147487472 | 10 | 6191376 | A | 0.0416 | 0.3795 | 0.0615 | 7.55E-10 |
| ebi-a-GCST90001784 | rs147487472 | 10 | 6191376 | A | 0.0414 | 0.4191 | 0.06162 | 1.20E-11 |
| ebi-a-GCST90001775 | rs147487472 | 10 | 6191376 | A | 0.0415 | 0.4239 | 0.06144 | 6.12E-12 |
| ebi-a-GCST90001779 | rs147487472 | 10 | 6191376 | A | 0.0414 | 0.4264 | 0.06126 | 4.00E-12 |
| ebi-a-GCST90001791 | rs147487472 | 10 | 6191376 | A | 0.0414 | 0.4465 | 0.0617 | 5.60E-13 |
| ebi-a-GCST90001412 | rs147556975 | 5 | 148456360 | T | 0.039 | -0.3012 | 0.06251 | 1.51E-06 |
| ebi-a-GCST90001815 | rs147563555 | 22 | 30044036 | G | 0.001 | 2.396 | 0.4982 | 1.58E-06 |
| ebi-a-GCST90001698 | rs147563624 | 10 | 5377215 | T | 9.00E-04 | 549.6 | 57.08 | 1.16E-21 |
| ebi-a-GCST90001994 | rs147571616 | 19 | 36362737 | A | 0.0017 | -1.407 | 0.3002 | 2.87E-06 |
| ebi-a-GCST90001700 | rs147572731 | 19 | 49416171 | T | 7.00E-04 | 14.48 | 3.001 | 1.44E-06 |
| ebi-a-GCST90001698 | rs147572731 | 19 | 49416171 | T | 7.00E-04 | 826.8 | 69.68 | 7.54E-32 |
| ebi-a-GCST90001698 | rs147590366 | 15 | 63216446 | T | 0.0041 | 151.8 | 28.99 | 1.74E-07 |
| ebi-a-GCST90001698 | rs147591823 | 1 | 47595103 | G | 0.0016 | 301 | 43.5 | 5.38E-12 |
| ebi-a-GCST90002053 | rs147597282 | 19 | 49965537 | A | 0.0016 | -2.088 | 0.4531 | 4.39E-06 |
| ebi-a-GCST90001667 | rs147612560 | 1 | 201600103 | A | 1.00E-04 | 4.119 | 0.8553 | 1.53E-06 |
| ebi-a-GCST90001669 | rs147612560 | 1 | 201600103 | A | 1.00E-04 | 4.289 | 0.8146 | 1.49E-07 |
| ebi-a-GCST90001501 | rs147638541 | 17 | 66206097 | G | 0.0018 | -1.375 | 0.2938 | 2.95E-06 |
| ebi-a-GCST90001885 | rs147640330 | 1 | 161536758 | C | 0.2437 | 0.2402 | 0.03117 | 1.79E-14 |
| ebi-a-GCST90001530 | rs147643522 | 3 | 185678332 | C | 0.0035 | 1.28 | 0.2755 | 3.63E-06 |
| ebi-a-GCST90002005 | rs147678401 | 1 | 161035780 | A | 0.0405 | -0.3505 | 0.06354 | 3.72E-08 |
| ebi-a-GCST90001469 | rs147678401 | 1 | 161035780 | A | 0.0437 | 0.3059 | 0.06344 | 1.48E-06 |
| ebi-a-GCST90001464 | rs147678401 | 1 | 161035780 | A | 0.0437 | 0.3116 | 0.06427 | 1.30E-06 |
| ebi-a-GCST90001458 | rs147678401 | 1 | 161035780 | A | 0.0437 | 0.319 | 0.06262 | 3.69E-07 |
| ebi-a-GCST90001473 | rs147678401 | 1 | 161035780 | A | 0.0437 | 0.3215 | 0.06383 | 4.97E-07 |
| ebi-a-GCST90001462 | rs147678401 | 1 | 161035780 | A | 0.0437 | 0.3283 | 0.06281 | 1.83E-07 |
| ebi-a-GCST90001468 | rs147678401 | 1 | 161035780 | A | 0.0437 | 0.3306 | 0.06429 | 2.86E-07 |
| ebi-a-GCST90001472 | rs147678401 | 1 | 161035780 | A | 0.0437 | 0.3417 | 0.06421 | 1.10E-07 |
| ebi-a-GCST90001797 | rs147680281 | 5 | 127113029 | T | 8.00E-04 | 2.007 | 0.4386 | 4.92E-06 |
| ebi-a-GCST90001698 | rs147681963 | 7 | 37590076 | T | 0.0094 | 114.2 | 18.22 | 4.11E-10 |
| ebi-a-GCST90001608 | rs147688743 | 10 | 64810319 | A | 0.0153 | -0.4693 | 0.09847 | 1.96E-06 |
| ebi-a-GCST90001594 | rs147688743 | 10 | 64810319 | A | 0.0153 | -0.4601 | 0.09898 | 3.45E-06 |
| ebi-a-GCST90002098 | rs147692482 | 2 | 39864242 | T | 0.0246 | 0.6089 | 0.08426 | 6.32E-13 |
| ebi-a-GCST90001800 | rs147714617 | 12 | 6615199 | T | 0.1224 | 0.186 | 0.0379 | 9.66E-07 |
| ebi-a-GCST90001806 | rs147714617 | 12 | 6615199 | T | 0.1224 | 0.2484 | 0.03759 | 4.48E-11 |
| ebi-a-GCST90001805 | rs147714617 | 12 | 6615199 | T | 0.1224 | 0.2563 | 0.03761 | 1.11E-11 |
| ebi-a-GCST90001798 | rs147714617 | 12 | 6615199 | T | 0.1224 | 0.2579 | 0.03725 | 5.23E-12 |
| ebi-a-GCST90001804 | rs147714617 | 12 | 6615199 | T | 0.1224 | 0.264 | 0.03709 | 1.33E-12 |
| ebi-a-GCST90001808 | rs147714617 | 12 | 6615199 | T | 0.1224 | 0.299 | 0.03747 | 1.93E-15 |
| ebi-a-GCST90001760 | rs147717587 | 6 | 32623828 | T | 0.0544 | -0.438 | 0.09174 | 1.88E-06 |
| ebi-a-GCST90001706 | rs147730115 | 21 | 26858465 | A | 0.0046 | 1.122 | 0.2447 | 4.84E-06 |
| ebi-a-GCST90002115 | rs147748599 | 17 | 63501173 | A | 0.0577 | 0.2621 | 0.05518 | 2.13E-06 |
| ebi-a-GCST90002065 | rs147763768 | 10 | 97548303 | A | 0.0434 | 0.3722 | 0.06802 | 4.86E-08 |
| ebi-a-GCST90001734 | rs147763919 | 8 | 62358083 | C | 0.0854 | 0.2034 | 0.04414 | 4.19E-06 |
| ebi-a-GCST90001698 | rs147774047 | 3 | 23633832 | C | 0.0037 | 160 | 29.14 | 4.31E-08 |
| ebi-a-GCST90001917 | rs147779398 | 7 | 42652757 | C | 0.0122 | -0.5313 | 0.1151 | 4.09E-06 |
| ebi-a-GCST90001697 | rs147794754 | 8 | 130397472 | T | 9.00E-04 | 10.43 | 2.108 | 7.76E-07 |
| ebi-a-GCST90002002 | rs147810622 | 7 | 151910695 | G | 0.0163 | -0.5881 | 0.0998 | 4.15E-09 |
| ebi-a-GCST90001993 | rs147810622 | 7 | 151910695 | G | 0.0163 | -0.507 | 0.09946 | 3.61E-07 |
| ebi-a-GCST90001503 | rs147838148 | 22 | 47814108 | G | 6.00E-04 | -2.662 | 0.4776 | 2.68E-08 |
| ebi-a-GCST90001683 | rs147839296 | 1 | 97114345 | T | 0.0948 | -0.1959 | 0.04115 | 2.00E-06 |
| ebi-a-GCST90001700 | rs147841321 | 4 | 130934266 | T | 0.0019 | 8.69 | 1.67 | 2.08E-07 |
| ebi-a-GCST90001698 | rs147848662 | 13 | 112802184 | T | 0.015 | 67.87 | 14.62 | 3.59E-06 |
| ebi-a-GCST90001820 | rs147854460 | 6 | 143010177 | A | 0.0067 | 0.7291 | 0.1487 | 9.91E-07 |
| ebi-a-GCST90001659 | rs147888698 | 10 | 96576314 | T | 0.0174 | -0.4543 | 0.09394 | 1.38E-06 |
| ebi-a-GCST90001496 | rs147911368 | 1 | 97319841 | C | 0.0063 | -0.772 | 0.1665 | 3.69E-06 |
| ebi-a-GCST90001840 | rs147934227 | 4 | 144501508 | T | 0.0024 | -1.264 | 0.2719 | 3.51E-06 |
| ebi-a-GCST90002108 | rs147951036 | 7 | 78355906 | A | 0.0149 | -0.7396 | 0.1516 | 1.17E-06 |
| ebi-a-GCST90001482 | rs147958143 | 1 | 238096255 | T | 0.0111 | -0.5364 | 0.1072 | 5.94E-07 |
| ebi-a-GCST90001698 | rs147959386 | 8 | 124197307 | A | 0.0079 | 123.5 | 20 | 7.32E-10 |
| ebi-a-GCST90001700 | rs147968748 | 10 | 54704473 | G | 0.0057 | 4.517 | 0.9486 | 2.00E-06 |
| ebi-a-GCST90001698 | rs147968748 | 10 | 54704473 | G | 0.0057 | 183.9 | 22.31 | 2.32E-16 |
| ebi-a-GCST90001919 | rs147981444 | 1 | 155359033 | A | 0.0137 | 0.5449 | 0.1169 | 3.31E-06 |
| ebi-a-GCST90001655 | rs147994116 | 7 | 110080212 | C | 9.00E-04 | 1.736 | 0.3581 | 1.31E-06 |
| ebi-a-GCST90001695 | rs148026776 | 2 | 155487782 | A | 0.0012 | 174.2 | 36.66 | 2.11E-06 |
| ebi-a-GCST90001541 | rs148031710 | 6 | 31148873 | C | 0.1083 | -0.2454 | 0.05086 | 1.45E-06 |
| ebi-a-GCST90002081 | rs148031710 | 6 | 31148873 | C | 0.1078 | 0.2974 | 0.04937 | 1.91E-09 |
| ebi-a-GCST90001996 | rs148057702 | 7 | 97552526 | T | 0.0305 | -0.3442 | 0.07454 | 4.01E-06 |
| ebi-a-GCST90001929 | rs148058371 | 9 | 5035205 | C | 0.0248 | -0.4286 | 0.08697 | 8.76E-07 |
| ebi-a-GCST90001688 | rs148064908 | 3 | 162634226 | A | NA | 11.05 | 2.095 | 1.42E-07 |
| ebi-a-GCST90001982 | rs148096757 | 8 | 11715710 | T | 0.0015 | 1.46 | 0.3091 | 2.39E-06 |
| ebi-a-GCST90001801 | rs148107267 | 15 | 33896396 | G | 0.003 | 1.39 | 0.2992 | 3.64E-06 |
| ebi-a-GCST90002073 | rs148146302 | 12 | 70360198 | C | 0.0239 | -0.8766 | 0.08769 | 3.80E-23 |
| ebi-a-GCST90002074 | rs148146302 | 12 | 70360198 | C | 0.023 | -0.8012 | 0.08571 | 1.65E-20 |
| ebi-a-GCST90001463 | rs148188224 | 16 | 65149970 | G | 0.0019 | -1.426 | 0.3084 | 3.95E-06 |
| ebi-a-GCST90001695 | rs148225159 | 18 | 56129994 | T | 0.0032 | 113 | 23.6 | 1.75E-06 |
| ebi-a-GCST90001695 | rs148242949 | 3 | 59179373 | T | 0.004 | 116.5 | 21.81 | 9.78E-08 |
| ebi-a-GCST90001893 | rs1482537 | 18 | 65833632 | A | 0.0233 | -0.4158 | 0.09066 | 4.69E-06 |
| ebi-a-GCST90001696 | rs148265436 | 17 | 71407818 | T | 0.0218 | 5.121 | 1.023 | 5.91E-07 |
| ebi-a-GCST90001700 | rs148291161 | 5 | 168556910 | G | 0.0012 | 14.11 | 2.744 | 2.86E-07 |
| ebi-a-GCST90001698 | rs148291161 | 5 | 168556910 | G | 0.0012 | 299.7 | 64.16 | 3.10E-06 |
| ebi-a-GCST90001857 | rs148303369 | 9 | 28514040 | C | 0.0408 | 0.3254 | 0.06869 | 2.27E-06 |
| ebi-a-GCST90001853 | rs148303369 | 9 | 28514040 | C | 0.0408 | 0.3289 | 0.06852 | 1.67E-06 |
| ebi-a-GCST90002060 | rs148307858 | 10 | 114431196 | T | 0.0013 | -1.762 | 0.3493 | 4.84E-07 |
| ebi-a-GCST90001619 | rs148323639 | 4 | 27183687 | A | 0.0317 | 0.3143 | 0.06409 | 9.80E-07 |
| ebi-a-GCST90002087 | rs148343003 | 10 | 101863324 | T | 0.0014 | 1.711 | 0.3584 | 1.91E-06 |
| ebi-a-GCST90001695 | rs148362510 | 18 | 3267975 | C | 4.00E-04 | 363.2 | 60.02 | 1.59E-09 |
| ebi-a-GCST90001539 | rs148365679 | 3 | 194035416 | A | 0.098 | 0.2134 | 0.04271 | 6.09E-07 |
| ebi-a-GCST90001410 | rs148377259 | 7 | 106306631 | C | 0.034 | -0.3437 | 0.06603 | 2.04E-07 |
| ebi-a-GCST90001425 | rs148377259 | 7 | 106306631 | C | 0.0341 | -0.311 | 0.06449 | 1.47E-06 |
| ebi-a-GCST90001961 | rs148377476 | 10 | 86411525 | C | 0.0276 | -0.3924 | 0.08273 | 2.21E-06 |
| ebi-a-GCST90001698 | rs148380088 | 9 | 28759554 | T | 0.0044 | 149.3 | 26.38 | 1.64E-08 |
| ebi-a-GCST90001741 | rs148381451 | 17 | 63085557 | T | 0.0496 | -0.2879 | 0.05601 | 2.90E-07 |
| ebi-a-GCST90001742 | rs148381451 | 17 | 63085557 | T | 0.0496 | -0.2814 | 0.05662 | 7.03E-07 |
| ebi-a-GCST90001725 | rs148381451 | 17 | 63085557 | T | 0.0496 | -0.2703 | 0.05589 | 1.38E-06 |
| ebi-a-GCST90001724 | rs148381451 | 17 | 63085557 | T | 0.0496 | -0.27 | 0.0563 | 1.68E-06 |
| ebi-a-GCST90001737 | rs148381451 | 17 | 63085557 | T | 0.0497 | -0.2588 | 0.05589 | 3.77E-06 |
| ebi-a-GCST90001730 | rs148381451 | 17 | 63085557 | T | 0.0496 | -0.257 | 0.05611 | 4.80E-06 |
| ebi-a-GCST90001697 | rs148384064 | 10 | 71016160 | T | 0.0063 | 3.801 | 0.8065 | 2.54E-06 |
| ebi-a-GCST90001507 | rs148388592 | 19 | 16483486 | A | 0.0377 | 0.3347 | 0.06838 | 1.03E-06 |
| ebi-a-GCST90001615 | rs148395664 | 20 | 6008247 | A | 0.0215 | 0.3857 | 0.08384 | 4.35E-06 |
| ebi-a-GCST90001454 | rs148420500 | 13 | 76034172 | A | 0.0209 | -0.4212 | 0.08981 | 2.86E-06 |
| ebi-a-GCST90002094 | rs148428675 | 6 | 112788900 | T | 0.0038 | -1.271 | 0.2748 | 4.08E-06 |
| ebi-a-GCST90001491 | rs148430006 | 10 | 95923091 | T | 0.0227 | 0.4454 | 0.08835 | 4.87E-07 |
| ebi-a-GCST90001686 | rs148460149 | 20 | 50115542 | A | 0.0339 | -0.3239 | 0.06304 | 2.92E-07 |
| ebi-a-GCST90001698 | rs148484526 | 1 | 204459946 | A | 0.0021 | 262.1 | 38.78 | 1.63E-11 |
| ebi-a-GCST90001391 | rs148496020 | 17 | 51092001 | A | 0.0193 | -0.4088 | 0.08921 | 4.75E-06 |
| ebi-a-GCST90001782 | rs148503715 | 20 | 61958726 | A | 5.00E-04 | 3.855 | 0.8138 | 2.34E-06 |
| ebi-a-GCST90001698 | rs148513824 | 20 | 61268143 | T | 0.0018 | 282.1 | 43.4 | 9.20E-11 |
| ebi-a-GCST90001699 | rs148524572 | 3 | 185618728 | C | 0.0054 | 11.65 | 2.417 | 1.48E-06 |
| ebi-a-GCST90001716 | rs148563640 | 17 | 48145906 | T | 0.0219 | 0.3942 | 0.08577 | 4.45E-06 |
| ebi-a-GCST90001709 | rs148563640 | 17 | 48145906 | T | 0.0219 | 0.3965 | 0.08567 | 3.81E-06 |
| ebi-a-GCST90001704 | rs148563640 | 17 | 48145906 | T | 0.0219 | 0.4012 | 0.0856 | 2.88E-06 |
| ebi-a-GCST90001720 | rs148563640 | 17 | 48145906 | T | 0.0219 | 0.4066 | 0.08597 | 2.33E-06 |
| ebi-a-GCST90001708 | rs148563640 | 17 | 48145906 | T | 0.0219 | 0.4447 | 0.08584 | 2.33E-07 |
| ebi-a-GCST90001698 | rs148590919 | 4 | 187560601 | A | 0.0015 | 262.7 | 44.6 | 4.22E-09 |
| ebi-a-GCST90001731 | rs148597489 | 6 | 72550483 | A | 0.0045 | -0.8449 | 0.18 | 2.78E-06 |
| ebi-a-GCST90001698 | rs148602493 | 9 | 23576735 | C | 0.0028 | 161.3 | 34.68 | 3.43E-06 |
| ebi-a-GCST90001695 | rs148620542 | 1 | 178035728 | T | 6.00E-04 | 358.6 | 51.64 | 4.54E-12 |
| ebi-a-GCST90001698 | rs148629497 | 9 | 10593877 | G | 0.0059 | 112.9 | 22.17 | 3.76E-07 |
| ebi-a-GCST90001700 | rs148658406 | 3 | 153311399 | T | 1.00E-04 | 32.81 | 5.716 | 1.02E-08 |
| ebi-a-GCST90001695 | rs148658406 | 3 | 153311399 | T | 1.00E-04 | 751.4 | 100.1 | 7.82E-14 |
| ebi-a-GCST90001698 | rs148658406 | 3 | 153311399 | T | 1.00E-04 | 2047 | 127.9 | 1.21E-55 |
| ebi-a-GCST90001944 | rs148659293 | 17 | 79284358 | T | 0.0315 | 0.3969 | 0.07954 | 6.38E-07 |
| ebi-a-GCST90001945 | rs148659293 | 17 | 79284358 | T | 0.0315 | 0.3981 | 0.07953 | 5.89E-07 |
| ebi-a-GCST90001922 | rs148709019 | 19 | 23371494 | T | 0.0387 | -0.3347 | 0.06975 | 1.67E-06 |
| ebi-a-GCST90001542 | rs148709760 | 9 | 136923252 | A | 0.0411 | -0.3035 | 0.0642 | 2.36E-06 |
| ebi-a-GCST90001534 | rs148716007 | 5 | 120094917 | T | 0.0442 | 0.2941 | 0.06366 | 3.97E-06 |
| ebi-a-GCST90001697 | rs148760558 | 17 | 72284938 | G | 0.0026 | 6.053 | 1.152 | 1.59E-07 |
| ebi-a-GCST90001989 | rs148765873 | 1 | 161221713 | C | 0.0453 | -0.3497 | 0.06275 | 2.69E-08 |
| ebi-a-GCST90001567 | rs148768795 | 6 | 150715047 | A | 0.0198 | 0.3892 | 0.08116 | 1.69E-06 |
| ebi-a-GCST90001728 | rs148774606 | 5 | 149841015 | C | 0.0019 | -1.674 | 0.365 | 4.81E-06 |
| ebi-a-GCST90001695 | rs148799115 | 4 | 45244488 | C | 0.0175 | 55.39 | 9.959 | 2.87E-08 |
| ebi-a-GCST90002032 | rs148810393 | 12 | 39023475 | T | 0.0469 | 0.3194 | 0.06785 | 2.62E-06 |
| ebi-a-GCST90001698 | rs148816938 | 5 | 71107850 | G | 7.00E-04 | 438.9 | 64.07 | 8.69E-12 |
| ebi-a-GCST90001698 | rs148835086 | 9 | 10278984 | T | 4.00E-04 | 751.9 | 78.49 | 1.83E-21 |
| ebi-a-GCST90001884 | rs148849191 | 11 | 113153223 | T | 0.0124 | -0.7822 | 0.1208 | 1.11E-10 |
| ebi-a-GCST90002077 | rs148866760 | 11 | 4509339 | G | 0.0061 | -0.8982 | 0.1871 | 1.65E-06 |
| ebi-a-GCST90001512 | rs148871284 | 20 | 15546558 | T | 0.0343 | 0.3269 | 0.07002 | 3.15E-06 |
| ebi-a-GCST90001511 | rs148871284 | 20 | 15546558 | T | 0.0343 | 0.3478 | 0.07003 | 7.13E-07 |
| ebi-a-GCST90002107 | rs148887724 | 3 | 101266753 | G | 0.0031 | 1.423 | 0.3097 | 4.68E-06 |
| ebi-a-GCST90001695 | rs148889514 | 10 | 111548575 | A | 0.0023 | 118.5 | 25.62 | 3.88E-06 |
| ebi-a-GCST90001491 | rs148903835 | 10 | 108283818 | A | 0.0106 | 0.6005 | 0.1288 | 3.23E-06 |
| ebi-a-GCST90001981 | rs148922794 | 20 | 44930372 | T | 0.0712 | 0.2587 | 0.0482 | 8.55E-08 |
| ebi-a-GCST90001845 | rs148932595 | 16 | 4725694 | A | 0.0101 | -0.6105 | 0.1289 | 2.27E-06 |
| ebi-a-GCST90001867 | rs148932595 | 16 | 4725694 | A | 0.0103 | -0.5793 | 0.1257 | 4.25E-06 |
| ebi-a-GCST90001638 | rs148936961 | 12 | 61264786 | T | 0.0228 | 0.367 | 0.0801 | 4.77E-06 |
| ebi-a-GCST90001637 | rs148936961 | 12 | 61264786 | T | 0.0228 | 0.3766 | 0.08041 | 2.92E-06 |
| ebi-a-GCST90001698 | rs148939501 | 5 | 12480500 | G | 0.0013 | 266.7 | 46.83 | 1.34E-08 |
| ebi-a-GCST90001700 | rs148947489 | 2 | 10326418 | T | 0.0022 | 8.302 | 1.533 | 6.58E-08 |
| ebi-a-GCST90001698 | rs148947489 | 2 | 10326418 | T | 0.0022 | 257.4 | 36.09 | 1.20E-12 |
| ebi-a-GCST90001993 | rs148958034 | 6 | 140202191 | G | 0.0105 | -0.5666 | 0.1195 | 2.22E-06 |
| ebi-a-GCST90001838 | rs148965436 | 4 | 53322015 | C | 0.0854 | -0.2297 | 0.04947 | 3.59E-06 |
| ebi-a-GCST90002010 | rs148974843 | 1 | 237971715 | G | 0.0473 | 0.2711 | 0.05867 | 3.95E-06 |
| ebi-a-GCST90001991 | rs148974843 | 1 | 237971715 | G | 0.0473 | 0.2717 | 0.05882 | 3.97E-06 |
| ebi-a-GCST90001988 | rs148974843 | 1 | 237971715 | G | 0.0473 | 0.2723 | 0.05871 | 3.64E-06 |
| ebi-a-GCST90002040 | rs1489849 | 3 | 79404844 | C | 0.3874 | 0.1469 | 0.02874 | 3.43E-07 |
| ebi-a-GCST90002121 | rs148989317 | 3 | 59931183 | T | 0.0117 | -0.6071 | 0.1264 | 1.63E-06 |
| ebi-a-GCST90001486 | rs148991751 | 12 | 53114409 | C | 4.00E-04 | -2.9 | 0.633 | 4.78E-06 |
| ebi-a-GCST90001616 | rs149000203 | 4 | 111341950 | A | 0.0267 | -0.3645 | 0.07702 | 2.29E-06 |
| ebi-a-GCST90001617 | rs149000203 | 4 | 111341950 | A | 0.0267 | -0.3616 | 0.07672 | 2.52E-06 |
| ebi-a-GCST90001626 | rs149004280 | 22 | 48226137 | T | 0.0079 | -0.5613 | 0.1224 | 4.67E-06 |
| ebi-a-GCST90001625 | rs149004280 | 22 | 48226137 | T | 0.0079 | -0.5593 | 0.119 | 2.68E-06 |
| ebi-a-GCST90001438 | rs149006488 | 11 | 67523715 | G | 0.1496 | -0.1579 | 0.03413 | 3.84E-06 |
| ebi-a-GCST90001893 | rs149008245 | 5 | 110580004 | G | 0.0072 | -0.7358 | 0.156 | 2.51E-06 |
| ebi-a-GCST90001697 | rs149015425 | 22 | 50545634 | T | 0.0047 | 4.189 | 0.8876 | 2.45E-06 |
| ebi-a-GCST90001700 | rs149021870 | 4 | 135635954 | G | 0.0096 | 3.698 | 0.7669 | 1.49E-06 |
| ebi-a-GCST90001688 | rs149023182 | 1 | 232756653 | G | 1.00E-04 | 4.52 | 0.7897 | 1.13E-08 |
| ebi-a-GCST90001523 | rs149034028 | 6 | 138310176 | T | 0.0258 | -0.4903 | 0.104 | 2.59E-06 |
| ebi-a-GCST90001698 | rs149037944 | 12 | 103661467 | T | 0.0029 | 150.1 | 32.81 | 4.95E-06 |
| ebi-a-GCST90001738 | rs149041258 | 18 | 74408048 | G | 0.0759 | -0.2229 | 0.04815 | 3.81E-06 |
| ebi-a-GCST90001695 | rs149043871 | 13 | 102446635 | A | 7.00E-04 | 230.8 | 48.41 | 1.93E-06 |
| ebi-a-GCST90001548 | rs149060712 | 8 | 60906565 | G | 0.0259 | 0.361 | 0.07686 | 2.75E-06 |
| ebi-a-GCST90001836 | rs149073662 | 1 | 9801707 | A | 0.0819 | 0.3103 | 0.06739 | 4.49E-06 |
| ebi-a-GCST90002069 | rs149080865 | 8 | 80655797 | A | 0.0164 | 0.491 | 0.1065 | 4.21E-06 |
| ebi-a-GCST90002095 | rs149090801 | 7 | 48224317 | C | 0.0234 | -0.647 | 0.1228 | 1.58E-07 |
| ebi-a-GCST90002036 | rs149094296 | 3 | 128701091 | G | 0.0044 | -1.077 | 0.208 | 2.42E-07 |
| ebi-a-GCST90001996 | rs149100 | 1 | 162441037 | G | 0.4225 | -0.1347 | 0.0251 | 8.49E-08 |
| ebi-a-GCST90002012 | rs149100 | 1 | 162441037 | G | 0.4223 | -0.121 | 0.02458 | 8.87E-07 |
| ebi-a-GCST90001788 | rs149114716 | 8 | 3816956 | C | 0.0283 | -0.3504 | 0.07552 | 3.62E-06 |
| ebi-a-GCST90001697 | rs149124296 | 1 | 213124992 | C | 6.00E-04 | 13.39 | 2.614 | 3.18E-07 |
| ebi-a-GCST90001695 | rs149124296 | 1 | 213124992 | C | 6.00E-04 | 307.6 | 55.58 | 3.35E-08 |
| ebi-a-GCST90001698 | rs149131245 | 2 | 234725994 | C | 0.001 | 332.2 | 53.59 | 6.35E-10 |
| ebi-a-GCST90001698 | rs149134258 | 14 | 93148478 | T | 0.0076 | 97.05 | 20.8 | 3.20E-06 |
| ebi-a-GCST90001698 | rs149136965 | 6 | 30958409 | C | 0.0022 | 203.1 | 36.33 | 2.44E-08 |
| ebi-a-GCST90001488 | rs149143064 | 2 | 39755475 | A | 0.0276 | -0.354 | 0.07702 | 4.46E-06 |
| ebi-a-GCST90001893 | rs149149608 | 19 | 14599430 | A | 0.026 | 0.4267 | 0.08617 | 7.75E-07 |
| ebi-a-GCST90001738 | rs149168550 | 12 | 111354000 | G | 0.0673 | 0.2262 | 0.04939 | 4.81E-06 |
| ebi-a-GCST90001723 | rs149168550 | 12 | 111354000 | G | 0.0673 | 0.234 | 0.04939 | 2.25E-06 |
| ebi-a-GCST90001698 | rs149168806 | 7 | 78834873 | T | 0.0107 | 76.79 | 16.56 | 3.66E-06 |
| ebi-a-GCST90001713 | rs149192849 | 22 | 42270864 | C | 0.0536 | -0.3114 | 0.0547 | 1.35E-08 |
| ebi-a-GCST90001830 | rs149192849 | 22 | 42270864 | C | 0.0537 | -0.2588 | 0.05351 | 1.37E-06 |
| ebi-a-GCST90001700 | rs149208831 | 6 | 10017676 | C | 3.00E-04 | 25.69 | 4.095 | 3.97E-10 |
| ebi-a-GCST90001698 | rs149208831 | 6 | 10017676 | C | 3.00E-04 | 1445 | 93.44 | 3.23E-52 |
| ebi-a-GCST90001745 | rs149214933 | 13 | 64174465 | G | 0.0062 | -0.782 | 0.1638 | 1.87E-06 |
| ebi-a-GCST90001717 | rs149228586 | 22 | 41608058 | T | 0.0286 | 0.3769 | 0.07881 | 1.80E-06 |
| ebi-a-GCST90001703 | rs149228586 | 22 | 41608058 | T | 0.0286 | 0.3809 | 0.07887 | 1.42E-06 |
| ebi-a-GCST90001702 | rs149228586 | 22 | 41608058 | T | 0.0286 | 0.3864 | 0.07888 | 1.00E-06 |
| ebi-a-GCST90001715 | rs149228586 | 22 | 41608058 | T | 0.0286 | 0.3904 | 0.07877 | 7.53E-07 |
| ebi-a-GCST90001718 | rs149228586 | 22 | 41608058 | T | 0.0286 | 0.3907 | 0.07895 | 7.80E-07 |
| ebi-a-GCST90001581 | rs149241803 | 7 | 39649629 | A | 0.0218 | -0.4023 | 0.08341 | 1.47E-06 |
| ebi-a-GCST90001908 | rs1492564 | 16 | 73774321 | T | 0.4414 | -0.1266 | 0.02735 | 3.85E-06 |
| ebi-a-GCST90002058 | rs149303959 | 2 | 97046283 | C | 0.0235 | 0.4055 | 0.08862 | 4.93E-06 |
| ebi-a-GCST90001698 | rs149355173 | 7 | 91861162 | G | 0.0051 | 119.8 | 24.82 | 1.45E-06 |
| ebi-a-GCST90001685 | rs149359490 | 6 | 42968121 | A | 0.0448 | -0.2814 | 0.05962 | 2.47E-06 |
| ebi-a-GCST90001747 | rs149360160 | 2 | 229499798 | T | 5.00E-04 | -2.452 | 0.5179 | 2.28E-06 |
| ebi-a-GCST90001759 | rs149360160 | 2 | 229499798 | T | 5.00E-04 | -2.418 | 0.5188 | 3.26E-06 |
| ebi-a-GCST90001762 | rs149360160 | 2 | 229499798 | T | 5.00E-04 | -2.41 | 0.5174 | 3.30E-06 |
| ebi-a-GCST90001674 | rs149389411 | 15 | 36411730 | C | 0.1904 | 0.1532 | 0.03295 | 3.45E-06 |
| ebi-a-GCST90001700 | rs149394991 | 7 | 30944767 | A | 6.00E-04 | 15.07 | 2.977 | 4.40E-07 |
| ebi-a-GCST90002121 | rs1493960 | 11 | 12344362 | C | 0.8242 | 0.1723 | 0.03536 | 1.16E-06 |
| ebi-a-GCST90001710 | rs149398740 | 22 | 23924845 | T | 0.0027 | -1.126 | 0.2389 | 2.53E-06 |
| ebi-a-GCST90001704 | rs149398740 | 22 | 23924845 | T | 0.0027 | -1.117 | 0.2393 | 3.14E-06 |
| ebi-a-GCST90001716 | rs149398740 | 22 | 23924845 | T | 0.0027 | -1.111 | 0.2398 | 3.74E-06 |
| ebi-a-GCST90001511 | rs149406003 | 13 | 101206935 | A | 0.0499 | -0.2876 | 0.05719 | 5.18E-07 |
| ebi-a-GCST90002000 | rs149427550 | 2 | 240353586 | T | 0.001 | 1.88 | 0.3759 | 5.99E-07 |
| ebi-a-GCST90001509 | rs149429639 | 18 | 42913522 | C | 0.001 | 1.778 | 0.3739 | 2.06E-06 |
| ebi-a-GCST90001508 | rs149429639 | 18 | 42913522 | C | 0.001 | 1.783 | 0.3741 | 1.96E-06 |
| ebi-a-GCST90001698 | rs149438077 | 15 | 30234373 | G | 0.004 | 159.7 | 28.3 | 1.82E-08 |
| ebi-a-GCST90001698 | rs149483829 | 20 | 49499194 | G | 0.0045 | 128.9 | 25.73 | 5.77E-07 |
| ebi-a-GCST90001697 | rs149487182 | 8 | 131518018 | T | 4.00E-04 | 20.95 | 2.979 | 2.46E-12 |
| ebi-a-GCST90001696 | rs149487182 | 8 | 131518018 | T | 4.00E-04 | 36.99 | 7.317 | 4.54E-07 |
| ebi-a-GCST90001924 | rs149514573 | 13 | 84155288 | A | 0.0466 | 0.3029 | 0.06608 | 4.75E-06 |
| ebi-a-GCST90001963 | rs149524008 | 12 | 104048446 | T | 0.001 | 1.948 | 0.4208 | 3.84E-06 |
| ebi-a-GCST90001604 | rs149528313 | 13 | 110658783 | A | 0.0307 | 0.3387 | 0.0677 | 5.94E-07 |
| ebi-a-GCST90001697 | rs149532201 | 18 | 26538114 | A | 0.0019 | 7.225 | 1.576 | 4.74E-06 |
| ebi-a-GCST90001695 | rs149532201 | 18 | 26538114 | A | 0.0019 | 180 | 33.61 | 9.07E-08 |
| ebi-a-GCST90001508 | rs149541387 | 12 | 15408104 | A | 0.0212 | 0.4045 | 0.08806 | 4.52E-06 |
| ebi-a-GCST90001836 | rs149541654 | 14 | 64186810 | G | 0.0464 | 0.4225 | 0.09013 | 3.02E-06 |
| ebi-a-GCST90001450 | rs149543954 | 20 | 15302488 | T | 0.0517 | 0.295 | 0.06108 | 1.43E-06 |
| ebi-a-GCST90001672 | rs149574697 | 18 | 67477449 | T | 0.018 | 0.4576 | 0.09369 | 1.08E-06 |
| ebi-a-GCST90001749 | rs1495924 | 2 | 241183284 | G | 0.3978 | -0.1614 | 0.03478 | 3.72E-06 |
| ebi-a-GCST90001478 | rs149595513 | 14 | 56535269 | T | 0.0031 | 1.04 | 0.227 | 4.79E-06 |
| ebi-a-GCST90001696 | rs149600977 | 6 | 9242018 | G | 0.0012 | 19.53 | 4.148 | 2.59E-06 |
| ebi-a-GCST90001697 | rs149604038 | 3 | 185463545 | G | 0.0016 | 8.464 | 1.614 | 1.65E-07 |
| ebi-a-GCST90002041 | rs149609625 | 5 | 53714969 | A | 0.0073 | 0.9673 | 0.2045 | 2.45E-06 |
| ebi-a-GCST90002058 | rs149618549 | 3 | 180210710 | T | 0.0185 | -0.4692 | 0.09891 | 2.19E-06 |
| ebi-a-GCST90001698 | rs149627909 | 8 | 28381797 | T | 1.00E-04 | 991 | 183.2 | 6.80E-08 |
| ebi-a-GCST90001981 | rs149632680 | 8 | 24034923 | G | 0.005 | 0.8538 | 0.1863 | 4.76E-06 |
| ebi-a-GCST90001698 | rs149649979 | 9 | 10889621 | T | 7.00E-04 | 708.1 | 63.75 | 3.47E-28 |
| ebi-a-GCST90001689 | rs149655274 | 4 | 154029285 | T | 0.0012 | 1.504 | 0.3069 | 1.00E-06 |
| ebi-a-GCST90001784 | rs149662107 | 7 | 47871972 | A | 0.0062 | -0.9269 | 0.1592 | 6.27E-09 |
| ebi-a-GCST90001779 | rs149662107 | 7 | 47871972 | A | 0.0062 | -0.8467 | 0.1584 | 9.62E-08 |
| ebi-a-GCST90001794 | rs149662107 | 7 | 47871972 | A | 0.0062 | -0.8253 | 0.1589 | 2.17E-07 |
| ebi-a-GCST90001775 | rs149662107 | 7 | 47871972 | A | 0.0061 | -0.8039 | 0.159 | 4.50E-07 |
| ebi-a-GCST90001780 | rs149662107 | 7 | 47871972 | A | 0.0062 | -0.8029 | 0.1574 | 3.57E-07 |
| ebi-a-GCST90001791 | rs149662107 | 7 | 47871972 | A | 0.0062 | -0.8004 | 0.1597 | 5.68E-07 |
| ebi-a-GCST90001758 | rs149695230 | 11 | 60288804 | G | 0.1079 | 0.1855 | 0.04005 | 3.73E-06 |
| ebi-a-GCST90001760 | rs149695230 | 11 | 60288804 | G | 0.1079 | 0.1869 | 0.04012 | 3.31E-06 |
| ebi-a-GCST90001745 | rs149695230 | 11 | 60288804 | G | 0.1079 | 0.1899 | 0.04009 | 2.27E-06 |
| ebi-a-GCST90002051 | rs149715992 | 6 | 133440965 | A | 0.0386 | 0.4742 | 0.09792 | 1.41E-06 |
| ebi-a-GCST90001697 | rs149716976 | 16 | 75062625 | G | 0.0057 | 4.577 | 0.79 | 7.53E-09 |
| ebi-a-GCST90001695 | rs149716976 | 16 | 75062625 | G | 0.0057 | 118.8 | 16.87 | 2.27E-12 |
| ebi-a-GCST90001695 | rs149737122 | 1 | 96560455 | T | 3.00E-04 | 342.5 | 71.9 | 1.98E-06 |
| ebi-a-GCST90001563 | rs149764572 | 14 | 95378018 | G | 0.0027 | 1.119 | 0.2235 | 5.77E-07 |
| ebi-a-GCST90001695 | rs149782409 | 6 | 14931262 | T | 4.00E-04 | 314.9 | 66.75 | 2.48E-06 |
| ebi-a-GCST90001993 | rs149793797 | 19 | 1781867 | C | 0.0376 | -0.308 | 0.06444 | 1.83E-06 |
| ebi-a-GCST90001698 | rs149801155 | 6 | 162457335 | G | 0.0028 | 203.9 | 36.54 | 2.60E-08 |
| ebi-a-GCST90001704 | rs149806587 | 22 | 40774205 | G | 0.0526 | -0.5384 | 0.05731 | 9.77E-21 |
| ebi-a-GCST90001719 | rs149806587 | 22 | 40774205 | G | 0.0526 | -0.5364 | 0.05734 | 1.41E-20 |
| ebi-a-GCST90001705 | rs149806587 | 22 | 40774205 | G | 0.0527 | -0.5355 | 0.05733 | 1.61E-20 |
| ebi-a-GCST90001829 | rs149806587 | 22 | 40774205 | G | 0.0526 | -0.5338 | 0.05744 | 2.50E-20 |
| ebi-a-GCST90001716 | rs149806587 | 22 | 40774205 | G | 0.0526 | -0.5248 | 0.05747 | 1.09E-19 |
| ebi-a-GCST90001709 | rs149806587 | 22 | 40774205 | G | 0.0526 | -0.5223 | 0.05739 | 1.44E-19 |
| ebi-a-GCST90001706 | rs149806587 | 22 | 40774205 | G | 0.0517 | -0.5047 | 0.07979 | 3.18E-10 |
| ebi-a-GCST90001708 | rs149806587 | 22 | 40774205 | G | 0.0527 | -0.4826 | 0.05757 | 7.25E-17 |
| ebi-a-GCST90001720 | rs149806587 | 22 | 40774205 | G | 0.0525 | -0.4771 | 0.05763 | 1.73E-16 |
| ebi-a-GCST90001462 | rs149812305 | 11 | 5480718 | C | 0.0272 | -0.36 | 0.0755 | 1.94E-06 |
| ebi-a-GCST90001463 | rs149812305 | 11 | 5480718 | C | 0.0271 | -0.3592 | 0.07663 | 2.88E-06 |
| ebi-a-GCST90001698 | rs149828559 | 6 | 84898123 | T | 0.0022 | 339.6 | 33.81 | 2.09E-23 |
| ebi-a-GCST90001876 | rs149830152 | 2 | 59392272 | C | 0.0048 | 1.423 | 0.3016 | 2.64E-06 |
| ebi-a-GCST90001698 | rs149844272 | 4 | 87144762 | A | 0.0019 | 240.6 | 38.16 | 3.26E-10 |
| ebi-a-GCST90001819 | rs149852991 | 4 | 15044338 | T | 0.0321 | -0.5334 | 0.06859 | 9.69E-15 |
| ebi-a-GCST90001698 | rs149860203 | 10 | 31026572 | T | 0.0013 | 453.6 | 47.7 | 3.55E-21 |
| ebi-a-GCST90001567 | rs149868216 | 8 | 89666365 | A | 0.0066 | 0.6688 | 0.1404 | 1.99E-06 |
| ebi-a-GCST90001698 | rs149876155 | 2 | 24167677 | C | 7.00E-04 | 562.4 | 67.24 | 8.73E-17 |
| ebi-a-GCST90002039 | rs149889574 | 2 | 195749242 | C | 0.0325 | -0.3642 | 0.07829 | 3.43E-06 |
| ebi-a-GCST90001885 | rs149897374 | 7 | 108731721 | T | 0.0608 | -0.2767 | 0.05561 | 6.90E-07 |
| ebi-a-GCST90001698 | rs149898247 | 5 | 129034231 | G | 0.0022 | 193.9 | 36.98 | 1.67E-07 |
| ebi-a-GCST90001757 | rs149908209 | 11 | 127473611 | C | 4.00E-04 | -2.552 | 0.5321 | 1.68E-06 |
| ebi-a-GCST90001698 | rs149908314 | 15 | 54344919 | T | 0.0016 | 416.3 | 45.67 | 1.31E-19 |
| ebi-a-GCST90002088 | rs149922594 | 2 | 24117291 | T | 0.0017 | -1.442 | 0.3111 | 3.71E-06 |
| ebi-a-GCST90001688 | rs149926070 | 21 | 41143748 | A | 1.00E-04 | 3.533 | 0.6368 | 3.12E-08 |
| ebi-a-GCST90002119 | rs149943125 | 2 | 102826146 | C | 0.0055 | -0.9617 | 0.1808 | 1.13E-07 |
| ebi-a-GCST90001688 | rs149948027 | 16 | 89718638 | T | 3.00E-04 | 2.886 | 0.5527 | 1.88E-07 |
| ebi-a-GCST90001660 | rs149951966 | 17 | 41137136 | C | 0.1034 | 0.1983 | 0.04337 | 4.99E-06 |
| ebi-a-GCST90001495 | rs149951966 | 17 | 41137136 | C | 0.1035 | 0.2069 | 0.04388 | 2.51E-06 |
| ebi-a-GCST90001501 | rs149951966 | 17 | 41137136 | C | 0.1035 | 0.2078 | 0.04392 | 2.33E-06 |
| ebi-a-GCST90001513 | rs149951966 | 17 | 41137136 | C | 0.1035 | 0.2109 | 0.04275 | 8.46E-07 |
| ebi-a-GCST90001492 | rs149951966 | 17 | 41137136 | C | 0.1035 | 0.2109 | 0.04394 | 1.66E-06 |
| ebi-a-GCST90001504 | rs149951966 | 17 | 41137136 | C | 0.1035 | 0.2152 | 0.04361 | 8.48E-07 |
| ebi-a-GCST90001695 | rs149957527 | 6 | 52375282 | A | 1.00E-04 | 913.1 | 98.95 | 4.71E-20 |
| ebi-a-GCST90001871 | rs1499672 | 1 | 56037629 | G | 0.5978 | -0.194 | 0.04209 | 4.48E-06 |
| ebi-a-GCST90001698 | rs149983528 | 2 | 61772377 | C | 1.00E-04 | 1006 | 184.8 | 5.60E-08 |
| ebi-a-GCST90001697 | rs149994650 | 3 | 18550864 | A | 3.00E-04 | 22.84 | 3.766 | 1.47E-09 |
| ebi-a-GCST90001695 | rs149994650 | 3 | 18550864 | A | 3.00E-04 | 473.4 | 81.22 | 6.10E-09 |
| ebi-a-GCST90001698 | rs150007303 | 16 | 23350716 | C | 0.0026 | 192.1 | 35.2 | 5.15E-08 |
| ebi-a-GCST90001698 | rs150027262 | 3 | 72800530 | G | 0.0034 | 169.8 | 31.47 | 7.29E-08 |
| ebi-a-GCST90001833 | rs150038452 | 21 | 27810403 | C | 0.0253 | 0.4093 | 0.0837 | 1.06E-06 |
| ebi-a-GCST90001832 | rs150038452 | 21 | 27810403 | C | 0.0252 | 0.4551 | 0.08329 | 5.05E-08 |
| ebi-a-GCST90001610 | rs150052968 | 3 | 42526600 | C | 0.2531 | 0.1444 | 0.02982 | 1.33E-06 |
| ebi-a-GCST90001609 | rs150052968 | 3 | 42526600 | C | 0.2534 | 0.1472 | 0.03061 | 1.59E-06 |
| ebi-a-GCST90001611 | rs150052968 | 3 | 42526600 | C | 0.2531 | 0.1477 | 0.02993 | 8.41E-07 |
| ebi-a-GCST90001698 | rs150053624 | 17 | 80299376 | T | 9.00E-04 | 496.1 | 55.44 | 5.81E-19 |
| ebi-a-GCST90001698 | rs150055480 | 14 | 55295865 | A | 0.0025 | 262.4 | 37.13 | 1.91E-12 |
| ebi-a-GCST90001967 | rs150082330 | 15 | 24565307 | T | 0.0114 | -0.6313 | 0.1286 | 9.54E-07 |
| ebi-a-GCST90001765 | rs150087880 | 6 | 66021291 | T | 0.0011 | -2.09 | 0.4017 | 2.08E-07 |
| ebi-a-GCST90001688 | rs150096082 | 3 | 126743223 | A | NA | 10.96 | 2.094 | 1.77E-07 |
| ebi-a-GCST90001485 | rs150096082 | 3 | 126743223 | A | NA | 14.69 | 2.928 | 5.53E-07 |
| ebi-a-GCST90001650 | rs150102419 | 17 | 61353821 | A | 0.0074 | 0.6357 | 0.135 | 2.60E-06 |
| ebi-a-GCST90001990 | rs150145799 | 1 | 149738590 | C | 0.1328 | 0.2869 | 0.03746 | 2.36E-14 |
| ebi-a-GCST90002001 | rs150145799 | 1 | 149738590 | C | 0.1327 | 0.2881 | 0.0365 | 3.92E-15 |
| ebi-a-GCST90002006 | rs150145799 | 1 | 149738590 | C | 0.1327 | 0.7181 | 0.03619 | 2.76E-83 |
| ebi-a-GCST90001987 | rs150145799 | 1 | 149738590 | C | 0.1327 | 0.7751 | 0.03562 | 8.90E-99 |
| ebi-a-GCST90001697 | rs150146567 | 2 | 12784208 | A | 0.0019 | 8.79 | 1.394 | 3.28E-10 |
| ebi-a-GCST90001695 | rs150146567 | 2 | 12784208 | A | 0.0019 | 148.4 | 29.95 | 7.54E-07 |
| ebi-a-GCST90001605 | rs150146725 | 20 | 49694567 | C | 0.0248 | -0.3712 | 0.07717 | 1.57E-06 |
| ebi-a-GCST90002079 | rs150148686 | 1 | 214244868 | C | 0.0053 | 0.8542 | 0.1756 | 1.20E-06 |
| ebi-a-GCST90001605 | rs1501750 | 5 | 4747892 | G | 0.9406 | 0.2324 | 0.05021 | 3.79E-06 |
| ebi-a-GCST90001522 | rs150206406 | 18 | 35502118 | T | 0.0135 | 0.6723 | 0.1447 | 3.61E-06 |
| ebi-a-GCST90001552 | rs150213926 | 1 | 200202822 | G | 0.0067 | -0.5394 | 0.1137 | 2.17E-06 |
| ebi-a-GCST90001452 | rs150248455 | 21 | 46373171 | A | 0.0673 | 0.2439 | 0.05149 | 2.27E-06 |
| ebi-a-GCST90001448 | rs150248455 | 21 | 46373171 | A | 0.0674 | 0.25 | 0.05201 | 1.60E-06 |
| ebi-a-GCST90001851 | rs1502523 | 17 | 51481273 | A | 0.5466 | 0.1262 | 0.02696 | 2.97E-06 |
| ebi-a-GCST90001697 | rs150285115 | 15 | 67108460 | A | 6.00E-04 | 14.23 | 2.741 | 2.22E-07 |
| ebi-a-GCST90001700 | rs150287087 | 6 | 125051261 | C | 7.00E-04 | 11.37 | 2.328 | 1.07E-06 |
| ebi-a-GCST90001698 | rs150287087 | 6 | 125051261 | C | 7.00E-04 | 487.3 | 54.77 | 9.16E-19 |
| ebi-a-GCST90001805 | rs150306315 | 19 | 16824730 | A | 0.0088 | 0.652 | 0.138 | 2.41E-06 |
| ebi-a-GCST90001869 | rs150306980 | 15 | 100531503 | C | 0.0375 | -0.3481 | 0.07239 | 1.60E-06 |
| ebi-a-GCST90001495 | rs150315125 | 8 | 10090715 | T | 0.0295 | -0.4106 | 0.08147 | 4.89E-07 |
| ebi-a-GCST90001658 | rs150315125 | 8 | 10090715 | T | 0.0297 | -0.3512 | 0.07659 | 4.68E-06 |
| ebi-a-GCST90001555 | rs150329295 | 1 | 241856210 | A | 4.00E-04 | -2.298 | 0.4826 | 2.00E-06 |
| ebi-a-GCST90001698 | rs150336475 | 9 | 111627658 | A | 0.0351 | 45.29 | 9.88 | 4.72E-06 |
| ebi-a-GCST90001609 | rs150339178 | 2 | 85647906 | T | 0.0498 | -0.3114 | 0.05886 | 1.29E-07 |
| ebi-a-GCST90001611 | rs150339178 | 2 | 85647906 | T | 0.0496 | -0.3086 | 0.05764 | 9.12E-08 |
| ebi-a-GCST90001610 | rs150339178 | 2 | 85647906 | T | 0.0496 | -0.299 | 0.05744 | 2.05E-07 |
| ebi-a-GCST90001990 | rs150375532 | 1 | 38146353 | A | 0.0026 | 1.124 | 0.2456 | 4.86E-06 |
| ebi-a-GCST90001695 | rs150383571 | 14 | 43151367 | C | 0.0141 | 61.29 | 11.2 | 4.77E-08 |
| ebi-a-GCST90001700 | rs150400236 | 7 | 136855654 | T | 0.0018 | 8.214 | 1.748 | 2.70E-06 |
| ebi-a-GCST90001698 | rs150400236 | 7 | 136855654 | T | 0.0018 | 299.6 | 41.15 | 4.15E-13 |
| ebi-a-GCST90001695 | rs150416566 | 1 | 108257502 | T | 0.0283 | 38.01 | 8.027 | 2.27E-06 |
| ebi-a-GCST90001521 | rs1504215 | 6 | 91006227 | A | 0.4249 | 0.1629 | 0.03418 | 2.02E-06 |
| ebi-a-GCST90001536 | rs150422994 | 1 | 21018820 | G | 0.0306 | -0.3206 | 0.06789 | 2.42E-06 |
| ebi-a-GCST90001695 | rs150427323 | 11 | 118189351 | A | 7.00E-04 | 352.4 | 49.32 | 1.09E-12 |
| ebi-a-GCST90002099 | rs1504496 | 4 | 45951996 | A | 0.0264 | 0.3839 | 0.08369 | 4.68E-06 |
| ebi-a-GCST90001698 | rs150476409 | 13 | 113289664 | A | 0.0012 | 432.3 | 52.29 | 1.92E-16 |
| ebi-a-GCST90001881 | rs150482210 | 3 | 135398834 | T | 0.02 | 0.632 | 0.1378 | 5.00E-06 |
| ebi-a-GCST90001827 | rs150521764 | 18 | 67714307 | C | 0.0135 | -0.4925 | 0.107 | 4.31E-06 |
| ebi-a-GCST90001825 | rs150521764 | 18 | 67714307 | C | 0.0135 | -0.4914 | 0.1068 | 4.32E-06 |
| ebi-a-GCST90001843 | rs150528455 | 1 | 26740555 | A | 0.0469 | -0.3078 | 0.06611 | 3.38E-06 |
| ebi-a-GCST90001958 | rs150579420 | 4 | 154119021 | A | 0.0048 | -0.8765 | 0.1831 | 1.78E-06 |
| ebi-a-GCST90001541 | rs150607181 | 1 | 239680551 | C | 0.0168 | 0.4446 | 0.09195 | 1.39E-06 |
| ebi-a-GCST90001575 | rs150607181 | 1 | 239680551 | C | 0.0168 | 0.4463 | 0.09653 | 3.93E-06 |
| ebi-a-GCST90001710 | rs150612106 | 22 | 40175781 | A | 0.0261 | -0.7252 | 0.07811 | 2.74E-20 |
| ebi-a-GCST90001711 | rs150612106 | 22 | 40175781 | A | 0.0261 | -0.725 | 0.07793 | 2.29E-20 |
| ebi-a-GCST90001707 | rs150612106 | 22 | 40175781 | A | 0.025 | -0.6961 | 0.1109 | 4.23E-10 |
| ebi-a-GCST90001698 | rs150635355 | 18 | 38662297 | A | 0.0135 | 93.79 | 15.05 | 5.14E-10 |
| ebi-a-GCST90002070 | rs150643215 | 7 | 20291018 | C | 0.0228 | -0.4284 | 0.09159 | 3.03E-06 |
| ebi-a-GCST90001905 | rs150649461 | 1 | 92925654 | C | 0.0428 | -0.4543 | 0.07237 | 3.96E-10 |
| ebi-a-GCST90001965 | rs150649461 | 1 | 92925654 | C | 0.0428 | -0.3725 | 0.07298 | 3.56E-07 |
| ebi-a-GCST90001582 | rs150649461 | 1 | 92925654 | C | 0.0459 | 0.2921 | 0.06379 | 4.83E-06 |
| ebi-a-GCST90001583 | rs150649461 | 1 | 92925654 | C | 0.0459 | 0.3032 | 0.06381 | 2.09E-06 |
| ebi-a-GCST90001697 | rs150650566 | 1 | 83046200 | C | 4.00E-04 | 13.74 | 2.735 | 5.33E-07 |
| ebi-a-GCST90001695 | rs150650566 | 1 | 83046200 | C | 4.00E-04 | 530.8 | 58.46 | 1.81E-19 |
| ebi-a-GCST90001400 | rs150663332 | 8 | 2044843 | T | 0.0839 | -0.2314 | 0.04374 | 1.28E-07 |
| ebi-a-GCST90001993 | rs150687633 | 20 | 49270830 | G | 0.0449 | 0.2755 | 0.05902 | 3.15E-06 |
| ebi-a-GCST90001924 | rs150735276 | 7 | 21186016 | C | 0.0034 | 1.223 | 0.2644 | 3.88E-06 |
| ebi-a-GCST90002096 | rs150751001 | 6 | 94714285 | T | 0.0392 | -0.4566 | 0.09488 | 1.63E-06 |
| ebi-a-GCST90001644 | rs150774187 | 7 | 100536678 | A | 0.1423 | 0.1705 | 0.0357 | 1.86E-06 |
[truncated: 653,727 more chars]
