# Supplementary material for: Causal Mediation Role of Immune Cells in Gut Microbiota–Pneumonia Associations: A Mendelian Randomisation Study
Source: J Cell Mol Med. 2025 Sep 11;29(17):e70839. doi: 10.1111/jcmm.70839 (PMC12425809; doi:10.1111/jcmm.70839)
Supplement: Supplementary file 9 — Table S3: Information about instrumental variables corresponding to gut microbiota (Mendelian randomization of gut microbiota on immune cells). [file JCMM-29-e70839-s001.docx]

Information about instrumental variables corresponding to gut microbiota(Mendelian randomization of gut microbiota on immune cells)

| **id.exposure** | **exposure** | **outcome** | **SNPs** | **chr** | **pos** | **EA** | **β** | **SE** | **P** |
| --- | --- | --- | --- | --- | --- | --- | --- | --- | --- |
| ebi-a-GCST90016921 | Gut microbiota abundance (class Mollicutes id.3920) || id:ebi-a-GCST90016921 | Myeloid Dendritic Cell Absolute Count || id:ebi-a-GCST90001458 | rs10108398 | 8 | 59440824 | G | 0.0769142 | 0.0153953 | 1.09E-06 |
| ebi-a-GCST90016921 | Gut microbiota abundance (class Mollicutes id.3920) || id:ebi-a-GCST90016921 | CD86+ myeloid Dendritic Cell %Dendritic Cell || id:ebi-a-GCST90001465 | rs10108398 | 8 | 59440824 | G | 0.0769142 | 0.0153953 | 1.09E-06 |
| ebi-a-GCST90016921 | Gut microbiota abundance (class Mollicutes id.3920) || id:ebi-a-GCST90016921 | CD62L- myeloid Dendritic Cell Absolute Count || id:ebi-a-GCST90001468 | rs10108398 | 8 | 59440824 | G | 0.0769142 | 0.0153953 | 1.09E-06 |
| ebi-a-GCST90016921 | Gut microbiota abundance (class Mollicutes id.3920) || id:ebi-a-GCST90016921 | CD62L- myeloid Dendritic Cell %Dendritic Cell || id:ebi-a-GCST90001469 | rs10108398 | 8 | 59440824 | G | 0.0769142 | 0.0153953 | 1.09E-06 |
| ebi-a-GCST90016921 | Gut microbiota abundance (class Mollicutes id.3920) || id:ebi-a-GCST90016921 | CD62L- CD86+ myeloid Dendritic Cell Absolute Count || id:ebi-a-GCST90001472 | rs10108398 | 8 | 59440824 | G | 0.0769142 | 0.0153953 | 1.09E-06 |
| ebi-a-GCST90016921 | Gut microbiota abundance (class Mollicutes id.3920) || id:ebi-a-GCST90016921 | CD62L- CD86+ myeloid Dendritic Cell %Dendritic Cell || id:ebi-a-GCST90001473 | rs10108398 | 8 | 59440824 | G | 0.0769142 | 0.0153953 | 1.09E-06 |
| ebi-a-GCST90016921 | Gut microbiota abundance (class Mollicutes id.3920) || id:ebi-a-GCST90016921 | HLA DR++ monocyte %monocyte || id:ebi-a-GCST90001475 | rs10108398 | 8 | 59440824 | G | 0.0769142 | 0.0153953 | 1.09E-06 |
| ebi-a-GCST90016921 | Gut microbiota abundance (class Mollicutes id.3920) || id:ebi-a-GCST90016921 | HLA DR++ monocyte Absolute Count || id:ebi-a-GCST90001477 | rs10108398 | 8 | 59440824 | G | 0.0769142 | 0.0153953 | 1.09E-06 |
| ebi-a-GCST90016921 | Gut microbiota abundance (class Mollicutes id.3920) || id:ebi-a-GCST90016921 | Basophil %CD33dim HLA DR- CD66b- || id:ebi-a-GCST90001533 | rs10108398 | 8 | 59440824 | G | 0.0769142 | 0.0153953 | 1.09E-06 |
| ebi-a-GCST90016921 | Gut microbiota abundance (class Mollicutes id.3920) || id:ebi-a-GCST90016921 | CD16+ monocyte %monocyte || id:ebi-a-GCST90001587 | rs10108398 | 8 | 59440824 | G | 0.0769142 | 0.0153953 | 1.09E-06 |
| ebi-a-GCST90016921 | Gut microbiota abundance (class Mollicutes id.3920) || id:ebi-a-GCST90016921 | T/B cell || id:ebi-a-GCST90001588 | rs10108398 | 8 | 59440824 | G | 0.0769142 | 0.0153953 | 1.09E-06 |
| ebi-a-GCST90016921 | Gut microbiota abundance (class Mollicutes id.3920) || id:ebi-a-GCST90016921 | CD8+ Natural Killer T %T cell || id:ebi-a-GCST90001631 | rs10108398 | 8 | 59440824 | G | 0.0769142 | 0.0153953 | 1.09E-06 |
| ebi-a-GCST90016921 | Gut microbiota abundance (class Mollicutes id.3920) || id:ebi-a-GCST90016921 | CD4-CD8- Natural Killer T %lymphocyte || id:ebi-a-GCST90001638 | rs10108398 | 8 | 59440824 | G | 0.0769142 | 0.0153953 | 1.09E-06 |
| ebi-a-GCST90016921 | Gut microbiota abundance (class Mollicutes id.3920) || id:ebi-a-GCST90016921 | HLA DR+ Natural Killer Absolute Count || id:ebi-a-GCST90001648 | rs10108398 | 8 | 59440824 | G | 0.0769142 | 0.0153953 | 1.09E-06 |
| ebi-a-GCST90016921 | Gut microbiota abundance (class Mollicutes id.3920) || id:ebi-a-GCST90016921 | HLA DR+ Natural Killer %Natural Killer || id:ebi-a-GCST90001649 | rs10108398 | 8 | 59440824 | G | 0.0769142 | 0.0153953 | 1.09E-06 |
| ebi-a-GCST90016921 | Gut microbiota abundance (class Mollicutes id.3920) || id:ebi-a-GCST90016921 | HLA DR+ Natural Killer %CD3- lymphocyte || id:ebi-a-GCST90001650 | rs10108398 | 8 | 59440824 | G | 0.0769142 | 0.0153953 | 1.09E-06 |
| ebi-a-GCST90016921 | Gut microbiota abundance (class Mollicutes id.3920) || id:ebi-a-GCST90016921 | CD28- CD25++ CD8+ T cell Absolute Count || id:ebi-a-GCST90001678 | rs10108398 | 8 | 59440824 | G | 0.0769142 | 0.0153953 | 1.09E-06 |
| ebi-a-GCST90016921 | Gut microbiota abundance (class Mollicutes id.3920) || id:ebi-a-GCST90016921 | CD25++ CD8+ T cell %T cell || id:ebi-a-GCST90001679 | rs10108398 | 8 | 59440824 | G | 0.0769142 | 0.0153953 | 1.09E-06 |
| ebi-a-GCST90016921 | Gut microbiota abundance (class Mollicutes id.3920) || id:ebi-a-GCST90016921 | CD19 on CD20- CD38- B cell || id:ebi-a-GCST90001722 | rs10108398 | 8 | 59440824 | G | 0.0769142 | 0.0153953 | 1.09E-06 |
| ebi-a-GCST90016921 | Gut microbiota abundance (class Mollicutes id.3920) || id:ebi-a-GCST90016921 | CD25 on B cell || id:ebi-a-GCST90001775 | rs10108398 | 8 | 59440824 | G | 0.0769142 | 0.0153953 | 1.09E-06 |
| ebi-a-GCST90016921 | Gut microbiota abundance (class Mollicutes id.3920) || id:ebi-a-GCST90016921 | CD25 on IgD+ CD24- B cell || id:ebi-a-GCST90001779 | rs10108398 | 8 | 59440824 | G | 0.0769142 | 0.0153953 | 1.09E-06 |
| ebi-a-GCST90016921 | Gut microbiota abundance (class Mollicutes id.3920) || id:ebi-a-GCST90016921 | CD25 on IgD+ CD38- naive B cell || id:ebi-a-GCST90001781 | rs10108398 | 8 | 59440824 | G | 0.0769142 | 0.0153953 | 1.09E-06 |
| ebi-a-GCST90016921 | Gut microbiota abundance (class Mollicutes id.3920) || id:ebi-a-GCST90016921 | CD25 on IgD+ CD38+ B cell || id:ebi-a-GCST90001783 | rs10108398 | 8 | 59440824 | G | 0.0769142 | 0.0153953 | 1.09E-06 |
| ebi-a-GCST90016921 | Gut microbiota abundance (class Mollicutes id.3920) || id:ebi-a-GCST90016921 | CD86 on myeloid Dendritic Cell || id:ebi-a-GCST90001903 | rs10108398 | 8 | 59440824 | G | 0.0769142 | 0.0153953 | 1.09E-06 |
| ebi-a-GCST90016921 | Gut microbiota abundance (class Mollicutes id.3920) || id:ebi-a-GCST90016921 | CD33 on CD14+ monocyte || id:ebi-a-GCST90001946 | rs10108398 | 8 | 59440824 | G | 0.0769142 | 0.0153953 | 1.09E-06 |
| ebi-a-GCST90016921 | Gut microbiota abundance (class Mollicutes id.3920) || id:ebi-a-GCST90016921 | CD33 on CD33+ HLA DR+ CD14dim || id:ebi-a-GCST90001947 | rs10108398 | 8 | 59440824 | G | 0.0769142 | 0.0153953 | 1.09E-06 |
| ebi-a-GCST90016921 | Gut microbiota abundance (class Mollicutes id.3920) || id:ebi-a-GCST90016921 | CD33 on CD33dim HLA DR+ CD11b+ || id:ebi-a-GCST90001948 | rs10108398 | 8 | 59440824 | G | 0.0769142 | 0.0153953 | 1.09E-06 |
| ebi-a-GCST90016921 | Gut microbiota abundance (class Mollicutes id.3920) || id:ebi-a-GCST90016921 | CD33 on Granulocytic Myeloid-Derived Suppressor Cells || id:ebi-a-GCST90001950 | rs10108398 | 8 | 59440824 | G | 0.0769142 | 0.0153953 | 1.09E-06 |
| ebi-a-GCST90016921 | Gut microbiota abundance (class Mollicutes id.3920) || id:ebi-a-GCST90016921 | CD33 on CD66b++ myeloid cell || id:ebi-a-GCST90001951 | rs10108398 | 8 | 59440824 | G | 0.0769142 | 0.0153953 | 1.09E-06 |
| ebi-a-GCST90016921 | Gut microbiota abundance (class Mollicutes id.3920) || id:ebi-a-GCST90016921 | CD33 on CD33dim HLA DR- || id:ebi-a-GCST90001953 | rs10108398 | 8 | 59440824 | G | 0.0769142 | 0.0153953 | 1.09E-06 |
| ebi-a-GCST90016921 | Gut microbiota abundance (class Mollicutes id.3920) || id:ebi-a-GCST90016921 | CD33 on basophil || id:ebi-a-GCST90001954 | rs10108398 | 8 | 59440824 | G | 0.0769142 | 0.0153953 | 1.09E-06 |
| ebi-a-GCST90016921 | Gut microbiota abundance (class Mollicutes id.3920) || id:ebi-a-GCST90016921 | CD33 on Immature Myeloid-Derived Suppressor Cells || id:ebi-a-GCST90001955 | rs10108398 | 8 | 59440824 | G | 0.0769142 | 0.0153953 | 1.09E-06 |
| ebi-a-GCST90016921 | Gut microbiota abundance (class Mollicutes id.3920) || id:ebi-a-GCST90016921 | FSC-A on HLA DR+ CD8+ T cell || id:ebi-a-GCST90001978 | rs10108398 | 8 | 59440824 | G | 0.0769142 | 0.0153953 | 1.09E-06 |
| ebi-a-GCST90016921 | Gut microbiota abundance (class Mollicutes id.3920) || id:ebi-a-GCST90016921 | HLA DR on CD14+ CD16- monocyte || id:ebi-a-GCST90001988 | rs10108398 | 8 | 59440824 | G | 0.0769142 | 0.0153953 | 1.09E-06 |
| ebi-a-GCST90016921 | Gut microbiota abundance (class Mollicutes id.3920) || id:ebi-a-GCST90016921 | HLA DR on CD14+ monocyte || id:ebi-a-GCST90001991 | rs10108398 | 8 | 59440824 | G | 0.0769142 | 0.0153953 | 1.09E-06 |
| ebi-a-GCST90016921 | Gut microbiota abundance (class Mollicutes id.3920) || id:ebi-a-GCST90016921 | CD16 on CD14+ CD16+ monocyte || id:ebi-a-GCST90002005 | rs10108398 | 8 | 59440824 | G | 0.0769142 | 0.0153953 | 1.09E-06 |
| ebi-a-GCST90016921 | Gut microbiota abundance (class Mollicutes id.3920) || id:ebi-a-GCST90016921 | CD45 on CD33+ HLA DR+ CD14- || id:ebi-a-GCST90002042 | rs10108398 | 8 | 59440824 | G | 0.0769142 | 0.0153953 | 1.09E-06 |
| ebi-a-GCST90016921 | Gut microbiota abundance (class Mollicutes id.3920) || id:ebi-a-GCST90016921 | CD8 on Natural Killer T || id:ebi-a-GCST90002059 | rs10108398 | 8 | 59440824 | G | 0.0769142 | 0.0153953 | 1.09E-06 |
| ebi-a-GCST90017117 | Gut microbiota abundance (phylum Tenericutes id.3919) || id:ebi-a-GCST90017117 | Myeloid Dendritic Cell Absolute Count || id:ebi-a-GCST90001458 | rs10108398 | 8 | 59440824 | G | 0.0769142 | 0.0153953 | 1.09E-06 |
| ebi-a-GCST90017117 | Gut microbiota abundance (phylum Tenericutes id.3919) || id:ebi-a-GCST90017117 | CD86+ myeloid Dendritic Cell %Dendritic Cell || id:ebi-a-GCST90001465 | rs10108398 | 8 | 59440824 | G | 0.0769142 | 0.0153953 | 1.09E-06 |
| ebi-a-GCST90017117 | Gut microbiota abundance (phylum Tenericutes id.3919) || id:ebi-a-GCST90017117 | CD62L- myeloid Dendritic Cell Absolute Count || id:ebi-a-GCST90001468 | rs10108398 | 8 | 59440824 | G | 0.0769142 | 0.0153953 | 1.09E-06 |
| ebi-a-GCST90017117 | Gut microbiota abundance (phylum Tenericutes id.3919) || id:ebi-a-GCST90017117 | CD62L- myeloid Dendritic Cell %Dendritic Cell || id:ebi-a-GCST90001469 | rs10108398 | 8 | 59440824 | G | 0.0769142 | 0.0153953 | 1.09E-06 |
| ebi-a-GCST90017117 | Gut microbiota abundance (phylum Tenericutes id.3919) || id:ebi-a-GCST90017117 | CD62L- CD86+ myeloid Dendritic Cell Absolute Count || id:ebi-a-GCST90001472 | rs10108398 | 8 | 59440824 | G | 0.0769142 | 0.0153953 | 1.09E-06 |
| ebi-a-GCST90017117 | Gut microbiota abundance (phylum Tenericutes id.3919) || id:ebi-a-GCST90017117 | CD62L- CD86+ myeloid Dendritic Cell %Dendritic Cell || id:ebi-a-GCST90001473 | rs10108398 | 8 | 59440824 | G | 0.0769142 | 0.0153953 | 1.09E-06 |
| ebi-a-GCST90017117 | Gut microbiota abundance (phylum Tenericutes id.3919) || id:ebi-a-GCST90017117 | HLA DR++ monocyte %monocyte || id:ebi-a-GCST90001475 | rs10108398 | 8 | 59440824 | G | 0.0769142 | 0.0153953 | 1.09E-06 |
| ebi-a-GCST90017117 | Gut microbiota abundance (phylum Tenericutes id.3919) || id:ebi-a-GCST90017117 | HLA DR++ monocyte Absolute Count || id:ebi-a-GCST90001477 | rs10108398 | 8 | 59440824 | G | 0.0769142 | 0.0153953 | 1.09E-06 |
| ebi-a-GCST90017117 | Gut microbiota abundance (phylum Tenericutes id.3919) || id:ebi-a-GCST90017117 | Basophil %CD33dim HLA DR- CD66b- || id:ebi-a-GCST90001533 | rs10108398 | 8 | 59440824 | G | 0.0769142 | 0.0153953 | 1.09E-06 |
| ebi-a-GCST90017117 | Gut microbiota abundance (phylum Tenericutes id.3919) || id:ebi-a-GCST90017117 | CD16+ monocyte %monocyte || id:ebi-a-GCST90001587 | rs10108398 | 8 | 59440824 | G | 0.0769142 | 0.0153953 | 1.09E-06 |
| ebi-a-GCST90017117 | Gut microbiota abundance (phylum Tenericutes id.3919) || id:ebi-a-GCST90017117 | T/B cell || id:ebi-a-GCST90001588 | rs10108398 | 8 | 59440824 | G | 0.0769142 | 0.0153953 | 1.09E-06 |
| ebi-a-GCST90017117 | Gut microbiota abundance (phylum Tenericutes id.3919) || id:ebi-a-GCST90017117 | CD8+ Natural Killer T %T cell || id:ebi-a-GCST90001631 | rs10108398 | 8 | 59440824 | G | 0.0769142 | 0.0153953 | 1.09E-06 |
| ebi-a-GCST90017117 | Gut microbiota abundance (phylum Tenericutes id.3919) || id:ebi-a-GCST90017117 | CD4-CD8- Natural Killer T %lymphocyte || id:ebi-a-GCST90001638 | rs10108398 | 8 | 59440824 | G | 0.0769142 | 0.0153953 | 1.09E-06 |
| ebi-a-GCST90017117 | Gut microbiota abundance (phylum Tenericutes id.3919) || id:ebi-a-GCST90017117 | HLA DR+ Natural Killer Absolute Count || id:ebi-a-GCST90001648 | rs10108398 | 8 | 59440824 | G | 0.0769142 | 0.0153953 | 1.09E-06 |
| ebi-a-GCST90017117 | Gut microbiota abundance (phylum Tenericutes id.3919) || id:ebi-a-GCST90017117 | HLA DR+ Natural Killer %Natural Killer || id:ebi-a-GCST90001649 | rs10108398 | 8 | 59440824 | G | 0.0769142 | 0.0153953 | 1.09E-06 |
| ebi-a-GCST90017117 | Gut microbiota abundance (phylum Tenericutes id.3919) || id:ebi-a-GCST90017117 | HLA DR+ Natural Killer %CD3- lymphocyte || id:ebi-a-GCST90001650 | rs10108398 | 8 | 59440824 | G | 0.0769142 | 0.0153953 | 1.09E-06 |
| ebi-a-GCST90017117 | Gut microbiota abundance (phylum Tenericutes id.3919) || id:ebi-a-GCST90017117 | CD28- CD25++ CD8+ T cell Absolute Count || id:ebi-a-GCST90001678 | rs10108398 | 8 | 59440824 | G | 0.0769142 | 0.0153953 | 1.09E-06 |
| ebi-a-GCST90017117 | Gut microbiota abundance (phylum Tenericutes id.3919) || id:ebi-a-GCST90017117 | CD25++ CD8+ T cell %T cell || id:ebi-a-GCST90001679 | rs10108398 | 8 | 59440824 | G | 0.0769142 | 0.0153953 | 1.09E-06 |
| ebi-a-GCST90017117 | Gut microbiota abundance (phylum Tenericutes id.3919) || id:ebi-a-GCST90017117 | CD19 on CD20- CD38- B cell || id:ebi-a-GCST90001722 | rs10108398 | 8 | 59440824 | G | 0.0769142 | 0.0153953 | 1.09E-06 |
| ebi-a-GCST90017117 | Gut microbiota abundance (phylum Tenericutes id.3919) || id:ebi-a-GCST90017117 | CD25 on B cell || id:ebi-a-GCST90001775 | rs10108398 | 8 | 59440824 | G | 0.0769142 | 0.0153953 | 1.09E-06 |
| ebi-a-GCST90017117 | Gut microbiota abundance (phylum Tenericutes id.3919) || id:ebi-a-GCST90017117 | CD25 on IgD+ CD24- B cell || id:ebi-a-GCST90001779 | rs10108398 | 8 | 59440824 | G | 0.0769142 | 0.0153953 | 1.09E-06 |
| ebi-a-GCST90017117 | Gut microbiota abundance (phylum Tenericutes id.3919) || id:ebi-a-GCST90017117 | CD25 on IgD+ CD38- naive B cell || id:ebi-a-GCST90001781 | rs10108398 | 8 | 59440824 | G | 0.0769142 | 0.0153953 | 1.09E-06 |
| ebi-a-GCST90017117 | Gut microbiota abundance (phylum Tenericutes id.3919) || id:ebi-a-GCST90017117 | CD25 on IgD+ CD38+ B cell || id:ebi-a-GCST90001783 | rs10108398 | 8 | 59440824 | G | 0.0769142 | 0.0153953 | 1.09E-06 |
| ebi-a-GCST90017117 | Gut microbiota abundance (phylum Tenericutes id.3919) || id:ebi-a-GCST90017117 | CD86 on myeloid Dendritic Cell || id:ebi-a-GCST90001903 | rs10108398 | 8 | 59440824 | G | 0.0769142 | 0.0153953 | 1.09E-06 |
| ebi-a-GCST90017117 | Gut microbiota abundance (phylum Tenericutes id.3919) || id:ebi-a-GCST90017117 | CD33 on CD14+ monocyte || id:ebi-a-GCST90001946 | rs10108398 | 8 | 59440824 | G | 0.0769142 | 0.0153953 | 1.09E-06 |
| ebi-a-GCST90017117 | Gut microbiota abundance (phylum Tenericutes id.3919) || id:ebi-a-GCST90017117 | CD33 on CD33+ HLA DR+ CD14dim || id:ebi-a-GCST90001947 | rs10108398 | 8 | 59440824 | G | 0.0769142 | 0.0153953 | 1.09E-06 |
| ebi-a-GCST90017117 | Gut microbiota abundance (phylum Tenericutes id.3919) || id:ebi-a-GCST90017117 | CD33 on CD33dim HLA DR+ CD11b+ || id:ebi-a-GCST90001948 | rs10108398 | 8 | 59440824 | G | 0.0769142 | 0.0153953 | 1.09E-06 |
| ebi-a-GCST90017117 | Gut microbiota abundance (phylum Tenericutes id.3919) || id:ebi-a-GCST90017117 | CD33 on Granulocytic Myeloid-Derived Suppressor Cells || id:ebi-a-GCST90001950 | rs10108398 | 8 | 59440824 | G | 0.0769142 | 0.0153953 | 1.09E-06 |
| ebi-a-GCST90017117 | Gut microbiota abundance (phylum Tenericutes id.3919) || id:ebi-a-GCST90017117 | CD33 on CD66b++ myeloid cell || id:ebi-a-GCST90001951 | rs10108398 | 8 | 59440824 | G | 0.0769142 | 0.0153953 | 1.09E-06 |
| ebi-a-GCST90017117 | Gut microbiota abundance (phylum Tenericutes id.3919) || id:ebi-a-GCST90017117 | CD33 on CD33dim HLA DR- || id:ebi-a-GCST90001953 | rs10108398 | 8 | 59440824 | G | 0.0769142 | 0.0153953 | 1.09E-06 |
| ebi-a-GCST90017117 | Gut microbiota abundance (phylum Tenericutes id.3919) || id:ebi-a-GCST90017117 | CD33 on basophil || id:ebi-a-GCST90001954 | rs10108398 | 8 | 59440824 | G | 0.0769142 | 0.0153953 | 1.09E-06 |
| ebi-a-GCST90017117 | Gut microbiota abundance (phylum Tenericutes id.3919) || id:ebi-a-GCST90017117 | CD33 on Immature Myeloid-Derived Suppressor Cells || id:ebi-a-GCST90001955 | rs10108398 | 8 | 59440824 | G | 0.0769142 | 0.0153953 | 1.09E-06 |
| ebi-a-GCST90017117 | Gut microbiota abundance (phylum Tenericutes id.3919) || id:ebi-a-GCST90017117 | FSC-A on HLA DR+ CD8+ T cell || id:ebi-a-GCST90001978 | rs10108398 | 8 | 59440824 | G | 0.0769142 | 0.0153953 | 1.09E-06 |
| ebi-a-GCST90017117 | Gut microbiota abundance (phylum Tenericutes id.3919) || id:ebi-a-GCST90017117 | HLA DR on CD14+ CD16- monocyte || id:ebi-a-GCST90001988 | rs10108398 | 8 | 59440824 | G | 0.0769142 | 0.0153953 | 1.09E-06 |
| ebi-a-GCST90017117 | Gut microbiota abundance (phylum Tenericutes id.3919) || id:ebi-a-GCST90017117 | HLA DR on CD14+ monocyte || id:ebi-a-GCST90001991 | rs10108398 | 8 | 59440824 | G | 0.0769142 | 0.0153953 | 1.09E-06 |
| ebi-a-GCST90017117 | Gut microbiota abundance (phylum Tenericutes id.3919) || id:ebi-a-GCST90017117 | CD16 on CD14+ CD16+ monocyte || id:ebi-a-GCST90002005 | rs10108398 | 8 | 59440824 | G | 0.0769142 | 0.0153953 | 1.09E-06 |
| ebi-a-GCST90017117 | Gut microbiota abundance (phylum Tenericutes id.3919) || id:ebi-a-GCST90017117 | CD45 on CD33+ HLA DR+ CD14- || id:ebi-a-GCST90002042 | rs10108398 | 8 | 59440824 | G | 0.0769142 | 0.0153953 | 1.09E-06 |
| ebi-a-GCST90017117 | Gut microbiota abundance (phylum Tenericutes id.3919) || id:ebi-a-GCST90017117 | CD8 on Natural Killer T || id:ebi-a-GCST90002059 | rs10108398 | 8 | 59440824 | G | 0.0769142 | 0.0153953 | 1.09E-06 |
| ebi-a-GCST90016957 | Gut microbiota abundance (family Verrucomicrobiaceae id.4036) || id:ebi-a-GCST90016957 | Myeloid Dendritic Cell Absolute Count || id:ebi-a-GCST90001458 | rs11184341 | 1 | 105422565 | C | -0.0655301 | 0.014223 | 4.14E-06 |
| ebi-a-GCST90016957 | Gut microbiota abundance (family Verrucomicrobiaceae id.4036) || id:ebi-a-GCST90016957 | CD86+ myeloid Dendritic Cell %Dendritic Cell || id:ebi-a-GCST90001465 | rs11184341 | 1 | 105422565 | C | -0.0655301 | 0.014223 | 4.14E-06 |
| ebi-a-GCST90016957 | Gut microbiota abundance (family Verrucomicrobiaceae id.4036) || id:ebi-a-GCST90016957 | CD62L- myeloid Dendritic Cell Absolute Count || id:ebi-a-GCST90001468 | rs11184341 | 1 | 105422565 | C | -0.0655301 | 0.014223 | 4.14E-06 |
| ebi-a-GCST90016957 | Gut microbiota abundance (family Verrucomicrobiaceae id.4036) || id:ebi-a-GCST90016957 | CD62L- myeloid Dendritic Cell %Dendritic Cell || id:ebi-a-GCST90001469 | rs11184341 | 1 | 105422565 | C | -0.0655301 | 0.014223 | 4.14E-06 |
| ebi-a-GCST90016957 | Gut microbiota abundance (family Verrucomicrobiaceae id.4036) || id:ebi-a-GCST90016957 | CD62L- CD86+ myeloid Dendritic Cell Absolute Count || id:ebi-a-GCST90001472 | rs11184341 | 1 | 105422565 | C | -0.0655301 | 0.014223 | 4.14E-06 |
| ebi-a-GCST90016957 | Gut microbiota abundance (family Verrucomicrobiaceae id.4036) || id:ebi-a-GCST90016957 | CD62L- CD86+ myeloid Dendritic Cell %Dendritic Cell || id:ebi-a-GCST90001473 | rs11184341 | 1 | 105422565 | C | -0.0655301 | 0.014223 | 4.14E-06 |
| ebi-a-GCST90016957 | Gut microbiota abundance (family Verrucomicrobiaceae id.4036) || id:ebi-a-GCST90016957 | HLA DR++ monocyte %monocyte || id:ebi-a-GCST90001475 | rs11184341 | 1 | 105422565 | C | -0.0655301 | 0.014223 | 4.14E-06 |
| ebi-a-GCST90016957 | Gut microbiota abundance (family Verrucomicrobiaceae id.4036) || id:ebi-a-GCST90016957 | HLA DR++ monocyte Absolute Count || id:ebi-a-GCST90001477 | rs11184341 | 1 | 105422565 | C | -0.0655301 | 0.014223 | 4.14E-06 |
| ebi-a-GCST90016957 | Gut microbiota abundance (family Verrucomicrobiaceae id.4036) || id:ebi-a-GCST90016957 | Basophil %CD33dim HLA DR- CD66b- || id:ebi-a-GCST90001533 | rs11184341 | 1 | 105422565 | C | -0.0655301 | 0.014223 | 4.14E-06 |
| ebi-a-GCST90016957 | Gut microbiota abundance (family Verrucomicrobiaceae id.4036) || id:ebi-a-GCST90016957 | CD16+ monocyte %monocyte || id:ebi-a-GCST90001587 | rs11184341 | 1 | 105422565 | C | -0.0655301 | 0.014223 | 4.14E-06 |
| ebi-a-GCST90016957 | Gut microbiota abundance (family Verrucomicrobiaceae id.4036) || id:ebi-a-GCST90016957 | T/B cell || id:ebi-a-GCST90001588 | rs11184341 | 1 | 105422565 | C | -0.0655301 | 0.014223 | 4.14E-06 |
| ebi-a-GCST90016957 | Gut microbiota abundance (family Verrucomicrobiaceae id.4036) || id:ebi-a-GCST90016957 | CD4+CD8+ T cell %T cell || id:ebi-a-GCST90001595 | rs11184341 | 1 | 105422565 | C | -0.0655301 | 0.014223 | 4.14E-06 |
| ebi-a-GCST90016957 | Gut microbiota abundance (family Verrucomicrobiaceae id.4036) || id:ebi-a-GCST90016957 | CD8+ Natural Killer T %T cell || id:ebi-a-GCST90001631 | rs11184341 | 1 | 105422565 | C | -0.0655301 | 0.014223 | 4.14E-06 |
| ebi-a-GCST90016957 | Gut microbiota abundance (family Verrucomicrobiaceae id.4036) || id:ebi-a-GCST90016957 | CD4-CD8- Natural Killer T %lymphocyte || id:ebi-a-GCST90001638 | rs11184341 | 1 | 105422565 | C | -0.0655301 | 0.014223 | 4.14E-06 |
| ebi-a-GCST90016957 | Gut microbiota abundance (family Verrucomicrobiaceae id.4036) || id:ebi-a-GCST90016957 | HLA DR+ Natural Killer Absolute Count || id:ebi-a-GCST90001648 | rs11184341 | 1 | 105422565 | C | -0.0655301 | 0.014223 | 4.14E-06 |
| ebi-a-GCST90016957 | Gut microbiota abundance (family Verrucomicrobiaceae id.4036) || id:ebi-a-GCST90016957 | HLA DR+ Natural Killer %Natural Killer || id:ebi-a-GCST90001649 | rs11184341 | 1 | 105422565 | C | -0.0655301 | 0.014223 | 4.14E-06 |
| ebi-a-GCST90016957 | Gut microbiota abundance (family Verrucomicrobiaceae id.4036) || id:ebi-a-GCST90016957 | HLA DR+ Natural Killer %CD3- lymphocyte || id:ebi-a-GCST90001650 | rs11184341 | 1 | 105422565 | C | -0.0655301 | 0.014223 | 4.14E-06 |
| ebi-a-GCST90016957 | Gut microbiota abundance (family Verrucomicrobiaceae id.4036) || id:ebi-a-GCST90016957 | CD28- CD25++ CD8+ T cell Absolute Count || id:ebi-a-GCST90001678 | rs11184341 | 1 | 105422565 | C | -0.0655301 | 0.014223 | 4.14E-06 |
| ebi-a-GCST90016957 | Gut microbiota abundance (family Verrucomicrobiaceae id.4036) || id:ebi-a-GCST90016957 | CD25++ CD8+ T cell %T cell || id:ebi-a-GCST90001679 | rs11184341 | 1 | 105422565 | C | -0.0655301 | 0.014223 | 4.14E-06 |
| ebi-a-GCST90016957 | Gut microbiota abundance (family Verrucomicrobiaceae id.4036) || id:ebi-a-GCST90016957 | CD19 on CD20- CD38- B cell || id:ebi-a-GCST90001722 | rs11184341 | 1 | 105422565 | C | -0.0655301 | 0.014223 | 4.14E-06 |
| ebi-a-GCST90016957 | Gut microbiota abundance (family Verrucomicrobiaceae id.4036) || id:ebi-a-GCST90016957 | CD25 on B cell || id:ebi-a-GCST90001775 | rs11184341 | 1 | 105422565 | C | -0.0655301 | 0.014223 | 4.14E-06 |
| ebi-a-GCST90016957 | Gut microbiota abundance (family Verrucomicrobiaceae id.4036) || id:ebi-a-GCST90016957 | CD25 on IgD+ CD24- B cell || id:ebi-a-GCST90001779 | rs11184341 | 1 | 105422565 | C | -0.0655301 | 0.014223 | 4.14E-06 |
| ebi-a-GCST90016957 | Gut microbiota abundance (family Verrucomicrobiaceae id.4036) || id:ebi-a-GCST90016957 | CD25 on IgD+ CD38- naive B cell || id:ebi-a-GCST90001781 | rs11184341 | 1 | 105422565 | C | -0.0655301 | 0.014223 | 4.14E-06 |
| ebi-a-GCST90016957 | Gut microbiota abundance (family Verrucomicrobiaceae id.4036) || id:ebi-a-GCST90016957 | CD25 on IgD+ CD38+ B cell || id:ebi-a-GCST90001783 | rs11184341 | 1 | 105422565 | C | -0.0655301 | 0.014223 | 4.14E-06 |
| ebi-a-GCST90016957 | Gut microbiota abundance (family Verrucomicrobiaceae id.4036) || id:ebi-a-GCST90016957 | CD86 on myeloid Dendritic Cell || id:ebi-a-GCST90001903 | rs11184341 | 1 | 105422565 | C | -0.0655301 | 0.014223 | 4.14E-06 |
| ebi-a-GCST90016957 | Gut microbiota abundance (family Verrucomicrobiaceae id.4036) || id:ebi-a-GCST90016957 | CD33 on CD14+ monocyte || id:ebi-a-GCST90001946 | rs11184341 | 1 | 105422565 | C | -0.0655301 | 0.014223 | 4.14E-06 |
| ebi-a-GCST90016957 | Gut microbiota abundance (family Verrucomicrobiaceae id.4036) || id:ebi-a-GCST90016957 | CD33 on CD33+ HLA DR+ CD14dim || id:ebi-a-GCST90001947 | rs11184341 | 1 | 105422565 | C | -0.0655301 | 0.014223 | 4.14E-06 |
| ebi-a-GCST90016957 | Gut microbiota abundance (family Verrucomicrobiaceae id.4036) || id:ebi-a-GCST90016957 | CD33 on CD33dim HLA DR+ CD11b+ || id:ebi-a-GCST90001948 | rs11184341 | 1 | 105422565 | C | -0.0655301 | 0.014223 | 4.14E-06 |
| ebi-a-GCST90016957 | Gut microbiota abundance (family Verrucomicrobiaceae id.4036) || id:ebi-a-GCST90016957 | CD33 on Granulocytic Myeloid-Derived Suppressor Cells || id:ebi-a-GCST90001950 | rs11184341 | 1 | 105422565 | C | -0.0655301 | 0.014223 | 4.14E-06 |
| ebi-a-GCST90016957 | Gut microbiota abundance (family Verrucomicrobiaceae id.4036) || id:ebi-a-GCST90016957 | CD33 on CD66b++ myeloid cell || id:ebi-a-GCST90001951 | rs11184341 | 1 | 105422565 | C | -0.0655301 | 0.014223 | 4.14E-06 |
| ebi-a-GCST90016957 | Gut microbiota abundance (family Verrucomicrobiaceae id.4036) || id:ebi-a-GCST90016957 | CD33 on CD33dim HLA DR- || id:ebi-a-GCST90001953 | rs11184341 | 1 | 105422565 | C | -0.0655301 | 0.014223 | 4.14E-06 |
| ebi-a-GCST90016957 | Gut microbiota abundance (family Verrucomicrobiaceae id.4036) || id:ebi-a-GCST90016957 | CD33 on basophil || id:ebi-a-GCST90001954 | rs11184341 | 1 | 105422565 | C | -0.0655301 | 0.014223 | 4.14E-06 |
| ebi-a-GCST90016957 | Gut microbiota abundance (family Verrucomicrobiaceae id.4036) || id:ebi-a-GCST90016957 | CD33 on Immature Myeloid-Derived Suppressor Cells || id:ebi-a-GCST90001955 | rs11184341 | 1 | 105422565 | C | -0.0655301 | 0.014223 | 4.14E-06 |
| ebi-a-GCST90016957 | Gut microbiota abundance (family Verrucomicrobiaceae id.4036) || id:ebi-a-GCST90016957 | FSC-A on HLA DR+ CD8+ T cell || id:ebi-a-GCST90001978 | rs11184341 | 1 | 105422565 | C | -0.0655301 | 0.014223 | 4.14E-06 |
| ebi-a-GCST90016957 | Gut microbiota abundance (family Verrucomicrobiaceae id.4036) || id:ebi-a-GCST90016957 | HLA DR on CD14+ CD16- monocyte || id:ebi-a-GCST90001988 | rs11184341 | 1 | 105422565 | C | -0.0655301 | 0.014223 | 4.14E-06 |
| ebi-a-GCST90016957 | Gut microbiota abundance (family Verrucomicrobiaceae id.4036) || id:ebi-a-GCST90016957 | HLA DR on CD14+ monocyte || id:ebi-a-GCST90001991 | rs11184341 | 1 | 105422565 | C | -0.0655301 | 0.014223 | 4.14E-06 |
| ebi-a-GCST90016957 | Gut microbiota abundance (family Verrucomicrobiaceae id.4036) || id:ebi-a-GCST90016957 | CD16 on CD14+ CD16+ monocyte || id:ebi-a-GCST90002005 | rs11184341 | 1 | 105422565 | C | -0.0655301 | 0.014223 | 4.14E-06 |
| ebi-a-GCST90016957 | Gut microbiota abundance (family Verrucomicrobiaceae id.4036) || id:ebi-a-GCST90016957 | CD45 on CD33+ HLA DR+ CD14- || id:ebi-a-GCST90002042 | rs11184341 | 1 | 105422565 | C | -0.0655301 | 0.014223 | 4.14E-06 |
| ebi-a-GCST90016957 | Gut microbiota abundance (family Verrucomicrobiaceae id.4036) || id:ebi-a-GCST90016957 | CD8 on Natural Killer T || id:ebi-a-GCST90002059 | rs11184341 | 1 | 105422565 | C | -0.0655301 | 0.014223 | 4.14E-06 |
| ebi-a-GCST90016923 | Gut microbiota abundance (class Verrucomicrobiae id.4029) || id:ebi-a-GCST90016923 | Myeloid Dendritic Cell Absolute Count || id:ebi-a-GCST90001458 | rs11184341 | 1 | 105422565 | C | -0.0655342 | 0.0142231 | 4.13E-06 |
| ebi-a-GCST90016923 | Gut microbiota abundance (class Verrucomicrobiae id.4029) || id:ebi-a-GCST90016923 | CD86+ myeloid Dendritic Cell %Dendritic Cell || id:ebi-a-GCST90001465 | rs11184341 | 1 | 105422565 | C | -0.0655342 | 0.0142231 | 4.13E-06 |
| ebi-a-GCST90016923 | Gut microbiota abundance (class Verrucomicrobiae id.4029) || id:ebi-a-GCST90016923 | CD62L- myeloid Dendritic Cell Absolute Count || id:ebi-a-GCST90001468 | rs11184341 | 1 | 105422565 | C | -0.0655342 | 0.0142231 | 4.13E-06 |
| ebi-a-GCST90016923 | Gut microbiota abundance (class Verrucomicrobiae id.4029) || id:ebi-a-GCST90016923 | CD62L- myeloid Dendritic Cell %Dendritic Cell || id:ebi-a-GCST90001469 | rs11184341 | 1 | 105422565 | C | -0.0655342 | 0.0142231 | 4.13E-06 |
| ebi-a-GCST90016923 | Gut microbiota abundance (class Verrucomicrobiae id.4029) || id:ebi-a-GCST90016923 | CD62L- CD86+ myeloid Dendritic Cell Absolute Count || id:ebi-a-GCST90001472 | rs11184341 | 1 | 105422565 | C | -0.0655342 | 0.0142231 | 4.13E-06 |
| ebi-a-GCST90016923 | Gut microbiota abundance (class Verrucomicrobiae id.4029) || id:ebi-a-GCST90016923 | CD62L- CD86+ myeloid Dendritic Cell %Dendritic Cell || id:ebi-a-GCST90001473 | rs11184341 | 1 | 105422565 | C | -0.0655342 | 0.0142231 | 4.13E-06 |
| ebi-a-GCST90016923 | Gut microbiota abundance (class Verrucomicrobiae id.4029) || id:ebi-a-GCST90016923 | HLA DR++ monocyte %monocyte || id:ebi-a-GCST90001475 | rs11184341 | 1 | 105422565 | C | -0.0655342 | 0.0142231 | 4.13E-06 |
| ebi-a-GCST90016923 | Gut microbiota abundance (class Verrucomicrobiae id.4029) || id:ebi-a-GCST90016923 | HLA DR++ monocyte Absolute Count || id:ebi-a-GCST90001477 | rs11184341 | 1 | 105422565 | C | -0.0655342 | 0.0142231 | 4.13E-06 |
| ebi-a-GCST90016923 | Gut microbiota abundance (class Verrucomicrobiae id.4029) || id:ebi-a-GCST90016923 | Basophil %CD33dim HLA DR- CD66b- || id:ebi-a-GCST90001533 | rs11184341 | 1 | 105422565 | C | -0.0655342 | 0.0142231 | 4.13E-06 |
| ebi-a-GCST90016923 | Gut microbiota abundance (class Verrucomicrobiae id.4029) || id:ebi-a-GCST90016923 | CD16+ monocyte %monocyte || id:ebi-a-GCST90001587 | rs11184341 | 1 | 105422565 | C | -0.0655342 | 0.0142231 | 4.13E-06 |
| ebi-a-GCST90016923 | Gut microbiota abundance (class Verrucomicrobiae id.4029) || id:ebi-a-GCST90016923 | T/B cell || id:ebi-a-GCST90001588 | rs11184341 | 1 | 105422565 | C | -0.0655342 | 0.0142231 | 4.13E-06 |
| ebi-a-GCST90016923 | Gut microbiota abundance (class Verrucomicrobiae id.4029) || id:ebi-a-GCST90016923 | CD4+CD8+ T cell %T cell || id:ebi-a-GCST90001595 | rs11184341 | 1 | 105422565 | C | -0.0655342 | 0.0142231 | 4.13E-06 |
| ebi-a-GCST90016923 | Gut microbiota abundance (class Verrucomicrobiae id.4029) || id:ebi-a-GCST90016923 | CD8+ Natural Killer T %T cell || id:ebi-a-GCST90001631 | rs11184341 | 1 | 105422565 | C | -0.0655342 | 0.0142231 | 4.13E-06 |
| ebi-a-GCST90016923 | Gut microbiota abundance (class Verrucomicrobiae id.4029) || id:ebi-a-GCST90016923 | CD4-CD8- Natural Killer T %lymphocyte || id:ebi-a-GCST90001638 | rs11184341 | 1 | 105422565 | C | -0.0655342 | 0.0142231 | 4.13E-06 |
| ebi-a-GCST90016923 | Gut microbiota abundance (class Verrucomicrobiae id.4029) || id:ebi-a-GCST90016923 | HLA DR+ Natural Killer Absolute Count || id:ebi-a-GCST90001648 | rs11184341 | 1 | 105422565 | C | -0.0655342 | 0.0142231 | 4.13E-06 |
| ebi-a-GCST90016923 | Gut microbiota abundance (class Verrucomicrobiae id.4029) || id:ebi-a-GCST90016923 | HLA DR+ Natural Killer %Natural Killer || id:ebi-a-GCST90001649 | rs11184341 | 1 | 105422565 | C | -0.0655342 | 0.0142231 | 4.13E-06 |
| ebi-a-GCST90016923 | Gut microbiota abundance (class Verrucomicrobiae id.4029) || id:ebi-a-GCST90016923 | HLA DR+ Natural Killer %CD3- lymphocyte || id:ebi-a-GCST90001650 | rs11184341 | 1 | 105422565 | C | -0.0655342 | 0.0142231 | 4.13E-06 |
| ebi-a-GCST90016923 | Gut microbiota abundance (class Verrucomicrobiae id.4029) || id:ebi-a-GCST90016923 | CD28- CD25++ CD8+ T cell Absolute Count || id:ebi-a-GCST90001678 | rs11184341 | 1 | 105422565 | C | -0.0655342 | 0.0142231 | 4.13E-06 |
| ebi-a-GCST90016923 | Gut microbiota abundance (class Verrucomicrobiae id.4029) || id:ebi-a-GCST90016923 | CD25++ CD8+ T cell %T cell || id:ebi-a-GCST90001679 | rs11184341 | 1 | 105422565 | C | -0.0655342 | 0.0142231 | 4.13E-06 |
| ebi-a-GCST90016923 | Gut microbiota abundance (class Verrucomicrobiae id.4029) || id:ebi-a-GCST90016923 | CD19 on CD20- CD38- B cell || id:ebi-a-GCST90001722 | rs11184341 | 1 | 105422565 | C | -0.0655342 | 0.0142231 | 4.13E-06 |
| ebi-a-GCST90016923 | Gut microbiota abundance (class Verrucomicrobiae id.4029) || id:ebi-a-GCST90016923 | CD25 on B cell || id:ebi-a-GCST90001775 | rs11184341 | 1 | 105422565 | C | -0.0655342 | 0.0142231 | 4.13E-06 |
| ebi-a-GCST90016923 | Gut microbiota abundance (class Verrucomicrobiae id.4029) || id:ebi-a-GCST90016923 | CD25 on IgD+ CD24- B cell || id:ebi-a-GCST90001779 | rs11184341 | 1 | 105422565 | C | -0.0655342 | 0.0142231 | 4.13E-06 |
| ebi-a-GCST90016923 | Gut microbiota abundance (class Verrucomicrobiae id.4029) || id:ebi-a-GCST90016923 | CD25 on IgD+ CD38- naive B cell || id:ebi-a-GCST90001781 | rs11184341 | 1 | 105422565 | C | -0.0655342 | 0.0142231 | 4.13E-06 |
| ebi-a-GCST90016923 | Gut microbiota abundance (class Verrucomicrobiae id.4029) || id:ebi-a-GCST90016923 | CD25 on IgD+ CD38+ B cell || id:ebi-a-GCST90001783 | rs11184341 | 1 | 105422565 | C | -0.0655342 | 0.0142231 | 4.13E-06 |
| ebi-a-GCST90016923 | Gut microbiota abundance (class Verrucomicrobiae id.4029) || id:ebi-a-GCST90016923 | CD86 on myeloid Dendritic Cell || id:ebi-a-GCST90001903 | rs11184341 | 1 | 105422565 | C | -0.0655342 | 0.0142231 | 4.13E-06 |
| ebi-a-GCST90016923 | Gut microbiota abundance (class Verrucomicrobiae id.4029) || id:ebi-a-GCST90016923 | CD33 on CD14+ monocyte || id:ebi-a-GCST90001946 | rs11184341 | 1 | 105422565 | C | -0.0655342 | 0.0142231 | 4.13E-06 |
| ebi-a-GCST90016923 | Gut microbiota abundance (class Verrucomicrobiae id.4029) || id:ebi-a-GCST90016923 | CD33 on CD33+ HLA DR+ CD14dim || id:ebi-a-GCST90001947 | rs11184341 | 1 | 105422565 | C | -0.0655342 | 0.0142231 | 4.13E-06 |
| ebi-a-GCST90016923 | Gut microbiota abundance (class Verrucomicrobiae id.4029) || id:ebi-a-GCST90016923 | CD33 on CD33dim HLA DR+ CD11b+ || id:ebi-a-GCST90001948 | rs11184341 | 1 | 105422565 | C | -0.0655342 | 0.0142231 | 4.13E-06 |
| ebi-a-GCST90016923 | Gut microbiota abundance (class Verrucomicrobiae id.4029) || id:ebi-a-GCST90016923 | CD33 on Granulocytic Myeloid-Derived Suppressor Cells || id:ebi-a-GCST90001950 | rs11184341 | 1 | 105422565 | C | -0.0655342 | 0.0142231 | 4.13E-06 |
| ebi-a-GCST90016923 | Gut microbiota abundance (class Verrucomicrobiae id.4029) || id:ebi-a-GCST90016923 | CD33 on CD66b++ myeloid cell || id:ebi-a-GCST90001951 | rs11184341 | 1 | 105422565 | C | -0.0655342 | 0.0142231 | 4.13E-06 |
| ebi-a-GCST90016923 | Gut microbiota abundance (class Verrucomicrobiae id.4029) || id:ebi-a-GCST90016923 | CD33 on CD33dim HLA DR- || id:ebi-a-GCST90001953 | rs11184341 | 1 | 105422565 | C | -0.0655342 | 0.0142231 | 4.13E-06 |
| ebi-a-GCST90016923 | Gut microbiota abundance (class Verrucomicrobiae id.4029) || id:ebi-a-GCST90016923 | CD33 on basophil || id:ebi-a-GCST90001954 | rs11184341 | 1 | 105422565 | C | -0.0655342 | 0.0142231 | 4.13E-06 |
| ebi-a-GCST90016923 | Gut microbiota abundance (class Verrucomicrobiae id.4029) || id:ebi-a-GCST90016923 | CD33 on Immature Myeloid-Derived Suppressor Cells || id:ebi-a-GCST90001955 | rs11184341 | 1 | 105422565 | C | -0.0655342 | 0.0142231 | 4.13E-06 |
| ebi-a-GCST90016923 | Gut microbiota abundance (class Verrucomicrobiae id.4029) || id:ebi-a-GCST90016923 | FSC-A on HLA DR+ CD8+ T cell || id:ebi-a-GCST90001978 | rs11184341 | 1 | 105422565 | C | -0.0655342 | 0.0142231 | 4.13E-06 |
| ebi-a-GCST90016923 | Gut microbiota abundance (class Verrucomicrobiae id.4029) || id:ebi-a-GCST90016923 | HLA DR on CD14+ CD16- monocyte || id:ebi-a-GCST90001988 | rs11184341 | 1 | 105422565 | C | -0.0655342 | 0.0142231 | 4.13E-06 |
| ebi-a-GCST90016923 | Gut microbiota abundance (class Verrucomicrobiae id.4029) || id:ebi-a-GCST90016923 | HLA DR on CD14+ monocyte || id:ebi-a-GCST90001991 | rs11184341 | 1 | 105422565 | C | -0.0655342 | 0.0142231 | 4.13E-06 |
| ebi-a-GCST90016923 | Gut microbiota abundance (class Verrucomicrobiae id.4029) || id:ebi-a-GCST90016923 | CD16 on CD14+ CD16+ monocyte || id:ebi-a-GCST90002005 | rs11184341 | 1 | 105422565 | C | -0.0655342 | 0.0142231 | 4.13E-06 |
| ebi-a-GCST90016923 | Gut microbiota abundance (class Verrucomicrobiae id.4029) || id:ebi-a-GCST90016923 | CD45 on CD33+ HLA DR+ CD14- || id:ebi-a-GCST90002042 | rs11184341 | 1 | 105422565 | C | -0.0655342 | 0.0142231 | 4.13E-06 |
| ebi-a-GCST90016923 | Gut microbiota abundance (class Verrucomicrobiae id.4029) || id:ebi-a-GCST90016923 | CD8 on Natural Killer T || id:ebi-a-GCST90002059 | rs11184341 | 1 | 105422565 | C | -0.0655342 | 0.0142231 | 4.13E-06 |
| ebi-a-GCST90017108 | Gut microbiota abundance (order Verrucomicrobiales id.4030) || id:ebi-a-GCST90017108 | Myeloid Dendritic Cell Absolute Count || id:ebi-a-GCST90001458 | rs11184341 | 1 | 105422565 | C | -0.0655342 | 0.0142231 | 4.13E-06 |
| ebi-a-GCST90017108 | Gut microbiota abundance (order Verrucomicrobiales id.4030) || id:ebi-a-GCST90017108 | CD86+ myeloid Dendritic Cell %Dendritic Cell || id:ebi-a-GCST90001465 | rs11184341 | 1 | 105422565 | C | -0.0655342 | 0.0142231 | 4.13E-06 |
| ebi-a-GCST90017108 | Gut microbiota abundance (order Verrucomicrobiales id.4030) || id:ebi-a-GCST90017108 | CD62L- myeloid Dendritic Cell Absolute Count || id:ebi-a-GCST90001468 | rs11184341 | 1 | 105422565 | C | -0.0655342 | 0.0142231 | 4.13E-06 |
| ebi-a-GCST90017108 | Gut microbiota abundance (order Verrucomicrobiales id.4030) || id:ebi-a-GCST90017108 | CD62L- myeloid Dendritic Cell %Dendritic Cell || id:ebi-a-GCST90001469 | rs11184341 | 1 | 105422565 | C | -0.0655342 | 0.0142231 | 4.13E-06 |
| ebi-a-GCST90017108 | Gut microbiota abundance (order Verrucomicrobiales id.4030) || id:ebi-a-GCST90017108 | CD62L- CD86+ myeloid Dendritic Cell Absolute Count || id:ebi-a-GCST90001472 | rs11184341 | 1 | 105422565 | C | -0.0655342 | 0.0142231 | 4.13E-06 |
| ebi-a-GCST90017108 | Gut microbiota abundance (order Verrucomicrobiales id.4030) || id:ebi-a-GCST90017108 | CD62L- CD86+ myeloid Dendritic Cell %Dendritic Cell || id:ebi-a-GCST90001473 | rs11184341 | 1 | 105422565 | C | -0.0655342 | 0.0142231 | 4.13E-06 |
| ebi-a-GCST90017108 | Gut microbiota abundance (order Verrucomicrobiales id.4030) || id:ebi-a-GCST90017108 | HLA DR++ monocyte %monocyte || id:ebi-a-GCST90001475 | rs11184341 | 1 | 105422565 | C | -0.0655342 | 0.0142231 | 4.13E-06 |
| ebi-a-GCST90017108 | Gut microbiota abundance (order Verrucomicrobiales id.4030) || id:ebi-a-GCST90017108 | HLA DR++ monocyte Absolute Count || id:ebi-a-GCST90001477 | rs11184341 | 1 | 105422565 | C | -0.0655342 | 0.0142231 | 4.13E-06 |
| ebi-a-GCST90017108 | Gut microbiota abundance (order Verrucomicrobiales id.4030) || id:ebi-a-GCST90017108 | Basophil %CD33dim HLA DR- CD66b- || id:ebi-a-GCST90001533 | rs11184341 | 1 | 105422565 | C | -0.0655342 | 0.0142231 | 4.13E-06 |
| ebi-a-GCST90017108 | Gut microbiota abundance (order Verrucomicrobiales id.4030) || id:ebi-a-GCST90017108 | CD16+ monocyte %monocyte || id:ebi-a-GCST90001587 | rs11184341 | 1 | 105422565 | C | -0.0655342 | 0.0142231 | 4.13E-06 |
| ebi-a-GCST90017108 | Gut microbiota abundance (order Verrucomicrobiales id.4030) || id:ebi-a-GCST90017108 | T/B cell || id:ebi-a-GCST90001588 | rs11184341 | 1 | 105422565 | C | -0.0655342 | 0.0142231 | 4.13E-06 |
| ebi-a-GCST90017108 | Gut microbiota abundance (order Verrucomicrobiales id.4030) || id:ebi-a-GCST90017108 | CD4+CD8+ T cell %T cell || id:ebi-a-GCST90001595 | rs11184341 | 1 | 105422565 | C | -0.0655342 | 0.0142231 | 4.13E-06 |
| ebi-a-GCST90017108 | Gut microbiota abundance (order Verrucomicrobiales id.4030) || id:ebi-a-GCST90017108 | CD8+ Natural Killer T %T cell || id:ebi-a-GCST90001631 | rs11184341 | 1 | 105422565 | C | -0.0655342 | 0.0142231 | 4.13E-06 |
| ebi-a-GCST90017108 | Gut microbiota abundance (order Verrucomicrobiales id.4030) || id:ebi-a-GCST90017108 | CD4-CD8- Natural Killer T %lymphocyte || id:ebi-a-GCST90001638 | rs11184341 | 1 | 105422565 | C | -0.0655342 | 0.0142231 | 4.13E-06 |
| ebi-a-GCST90017108 | Gut microbiota abundance (order Verrucomicrobiales id.4030) || id:ebi-a-GCST90017108 | HLA DR+ Natural Killer Absolute Count || id:ebi-a-GCST90001648 | rs11184341 | 1 | 105422565 | C | -0.0655342 | 0.0142231 | 4.13E-06 |
| ebi-a-GCST90017108 | Gut microbiota abundance (order Verrucomicrobiales id.4030) || id:ebi-a-GCST90017108 | HLA DR+ Natural Killer %Natural Killer || id:ebi-a-GCST90001649 | rs11184341 | 1 | 105422565 | C | -0.0655342 | 0.0142231 | 4.13E-06 |
| ebi-a-GCST90017108 | Gut microbiota abundance (order Verrucomicrobiales id.4030) || id:ebi-a-GCST90017108 | HLA DR+ Natural Killer %CD3- lymphocyte || id:ebi-a-GCST90001650 | rs11184341 | 1 | 105422565 | C | -0.0655342 | 0.0142231 | 4.13E-06 |
| ebi-a-GCST90017108 | Gut microbiota abundance (order Verrucomicrobiales id.4030) || id:ebi-a-GCST90017108 | CD28- CD25++ CD8+ T cell Absolute Count || id:ebi-a-GCST90001678 | rs11184341 | 1 | 105422565 | C | -0.0655342 | 0.0142231 | 4.13E-06 |
| ebi-a-GCST90017108 | Gut microbiota abundance (order Verrucomicrobiales id.4030) || id:ebi-a-GCST90017108 | CD25++ CD8+ T cell %T cell || id:ebi-a-GCST90001679 | rs11184341 | 1 | 105422565 | C | -0.0655342 | 0.0142231 | 4.13E-06 |
| ebi-a-GCST90017108 | Gut microbiota abundance (order Verrucomicrobiales id.4030) || id:ebi-a-GCST90017108 | CD19 on CD20- CD38- B cell || id:ebi-a-GCST90001722 | rs11184341 | 1 | 105422565 | C | -0.0655342 | 0.0142231 | 4.13E-06 |
| ebi-a-GCST90017108 | Gut microbiota abundance (order Verrucomicrobiales id.4030) || id:ebi-a-GCST90017108 | CD25 on B cell || id:ebi-a-GCST90001775 | rs11184341 | 1 | 105422565 | C | -0.0655342 | 0.0142231 | 4.13E-06 |
| ebi-a-GCST90017108 | Gut microbiota abundance (order Verrucomicrobiales id.4030) || id:ebi-a-GCST90017108 | CD25 on IgD+ CD24- B cell || id:ebi-a-GCST90001779 | rs11184341 | 1 | 105422565 | C | -0.0655342 | 0.0142231 | 4.13E-06 |
| ebi-a-GCST90017108 | Gut microbiota abundance (order Verrucomicrobiales id.4030) || id:ebi-a-GCST90017108 | CD25 on IgD+ CD38- naive B cell || id:ebi-a-GCST90001781 | rs11184341 | 1 | 105422565 | C | -0.0655342 | 0.0142231 | 4.13E-06 |
| ebi-a-GCST90017108 | Gut microbiota abundance (order Verrucomicrobiales id.4030) || id:ebi-a-GCST90017108 | CD25 on IgD+ CD38+ B cell || id:ebi-a-GCST90001783 | rs11184341 | 1 | 105422565 | C | -0.0655342 | 0.0142231 | 4.13E-06 |
| ebi-a-GCST90017108 | Gut microbiota abundance (order Verrucomicrobiales id.4030) || id:ebi-a-GCST90017108 | CD86 on myeloid Dendritic Cell || id:ebi-a-GCST90001903 | rs11184341 | 1 | 105422565 | C | -0.0655342 | 0.0142231 | 4.13E-06 |
| ebi-a-GCST90017108 | Gut microbiota abundance (order Verrucomicrobiales id.4030) || id:ebi-a-GCST90017108 | CD33 on CD14+ monocyte || id:ebi-a-GCST90001946 | rs11184341 | 1 | 105422565 | C | -0.0655342 | 0.0142231 | 4.13E-06 |
| ebi-a-GCST90017108 | Gut microbiota abundance (order Verrucomicrobiales id.4030) || id:ebi-a-GCST90017108 | CD33 on CD33+ HLA DR+ CD14dim || id:ebi-a-GCST90001947 | rs11184341 | 1 | 105422565 | C | -0.0655342 | 0.0142231 | 4.13E-06 |
| ebi-a-GCST90017108 | Gut microbiota abundance (order Verrucomicrobiales id.4030) || id:ebi-a-GCST90017108 | CD33 on CD33dim HLA DR+ CD11b+ || id:ebi-a-GCST90001948 | rs11184341 | 1 | 105422565 | C | -0.0655342 | 0.0142231 | 4.13E-06 |
| ebi-a-GCST90017108 | Gut microbiota abundance (order Verrucomicrobiales id.4030) || id:ebi-a-GCST90017108 | CD33 on Granulocytic Myeloid-Derived Suppressor Cells || id:ebi-a-GCST90001950 | rs11184341 | 1 | 105422565 | C | -0.0655342 | 0.0142231 | 4.13E-06 |
| ebi-a-GCST90017108 | Gut microbiota abundance (order Verrucomicrobiales id.4030) || id:ebi-a-GCST90017108 | CD33 on CD66b++ myeloid cell || id:ebi-a-GCST90001951 | rs11184341 | 1 | 105422565 | C | -0.0655342 | 0.0142231 | 4.13E-06 |
| ebi-a-GCST90017108 | Gut microbiota abundance (order Verrucomicrobiales id.4030) || id:ebi-a-GCST90017108 | CD33 on CD33dim HLA DR- || id:ebi-a-GCST90001953 | rs11184341 | 1 | 105422565 | C | -0.0655342 | 0.0142231 | 4.13E-06 |
| ebi-a-GCST90017108 | Gut microbiota abundance (order Verrucomicrobiales id.4030) || id:ebi-a-GCST90017108 | CD33 on basophil || id:ebi-a-GCST90001954 | rs11184341 | 1 | 105422565 | C | -0.0655342 | 0.0142231 | 4.13E-06 |
| ebi-a-GCST90017108 | Gut microbiota abundance (order Verrucomicrobiales id.4030) || id:ebi-a-GCST90017108 | CD33 on Immature Myeloid-Derived Suppressor Cells || id:ebi-a-GCST90001955 | rs11184341 | 1 | 105422565 | C | -0.0655342 | 0.0142231 | 4.13E-06 |
| ebi-a-GCST90017108 | Gut microbiota abundance (order Verrucomicrobiales id.4030) || id:ebi-a-GCST90017108 | FSC-A on HLA DR+ CD8+ T cell || id:ebi-a-GCST90001978 | rs11184341 | 1 | 105422565 | C | -0.0655342 | 0.0142231 | 4.13E-06 |
| ebi-a-GCST90017108 | Gut microbiota abundance (order Verrucomicrobiales id.4030) || id:ebi-a-GCST90017108 | HLA DR on CD14+ CD16- monocyte || id:ebi-a-GCST90001988 | rs11184341 | 1 | 105422565 | C | -0.0655342 | 0.0142231 | 4.13E-06 |
| ebi-a-GCST90017108 | Gut microbiota abundance (order Verrucomicrobiales id.4030) || id:ebi-a-GCST90017108 | HLA DR on CD14+ monocyte || id:ebi-a-GCST90001991 | rs11184341 | 1 | 105422565 | C | -0.0655342 | 0.0142231 | 4.13E-06 |
| ebi-a-GCST90017108 | Gut microbiota abundance (order Verrucomicrobiales id.4030) || id:ebi-a-GCST90017108 | CD16 on CD14+ CD16+ monocyte || id:ebi-a-GCST90002005 | rs11184341 | 1 | 105422565 | C | -0.0655342 | 0.0142231 | 4.13E-06 |
| ebi-a-GCST90017108 | Gut microbiota abundance (order Verrucomicrobiales id.4030) || id:ebi-a-GCST90017108 | CD45 on CD33+ HLA DR+ CD14- || id:ebi-a-GCST90002042 | rs11184341 | 1 | 105422565 | C | -0.0655342 | 0.0142231 | 4.13E-06 |
| ebi-a-GCST90017108 | Gut microbiota abundance (order Verrucomicrobiales id.4030) || id:ebi-a-GCST90017108 | CD8 on Natural Killer T || id:ebi-a-GCST90002059 | rs11184341 | 1 | 105422565 | C | -0.0655342 | 0.0142231 | 4.13E-06 |
| ebi-a-GCST90016961 | Gut microbiota abundance (genus Akkermansia id.4037) || id:ebi-a-GCST90016961 | Myeloid Dendritic Cell Absolute Count || id:ebi-a-GCST90001458 | rs11184341 | 1 | 105422565 | C | -0.0655855 | 0.0142236 | 4.06E-06 |
| ebi-a-GCST90016961 | Gut microbiota abundance (genus Akkermansia id.4037) || id:ebi-a-GCST90016961 | CD86+ myeloid Dendritic Cell %Dendritic Cell || id:ebi-a-GCST90001465 | rs11184341 | 1 | 105422565 | C | -0.0655855 | 0.0142236 | 4.06E-06 |
| ebi-a-GCST90016961 | Gut microbiota abundance (genus Akkermansia id.4037) || id:ebi-a-GCST90016961 | CD62L- myeloid Dendritic Cell Absolute Count || id:ebi-a-GCST90001468 | rs11184341 | 1 | 105422565 | C | -0.0655855 | 0.0142236 | 4.06E-06 |
| ebi-a-GCST90016961 | Gut microbiota abundance (genus Akkermansia id.4037) || id:ebi-a-GCST90016961 | CD62L- myeloid Dendritic Cell %Dendritic Cell || id:ebi-a-GCST90001469 | rs11184341 | 1 | 105422565 | C | -0.0655855 | 0.0142236 | 4.06E-06 |
| ebi-a-GCST90016961 | Gut microbiota abundance (genus Akkermansia id.4037) || id:ebi-a-GCST90016961 | CD62L- CD86+ myeloid Dendritic Cell Absolute Count || id:ebi-a-GCST90001472 | rs11184341 | 1 | 105422565 | C | -0.0655855 | 0.0142236 | 4.06E-06 |
| ebi-a-GCST90016961 | Gut microbiota abundance (genus Akkermansia id.4037) || id:ebi-a-GCST90016961 | CD62L- CD86+ myeloid Dendritic Cell %Dendritic Cell || id:ebi-a-GCST90001473 | rs11184341 | 1 | 105422565 | C | -0.0655855 | 0.0142236 | 4.06E-06 |
| ebi-a-GCST90016961 | Gut microbiota abundance (genus Akkermansia id.4037) || id:ebi-a-GCST90016961 | HLA DR++ monocyte %monocyte || id:ebi-a-GCST90001475 | rs11184341 | 1 | 105422565 | C | -0.0655855 | 0.0142236 | 4.06E-06 |
| ebi-a-GCST90016961 | Gut microbiota abundance (genus Akkermansia id.4037) || id:ebi-a-GCST90016961 | HLA DR++ monocyte Absolute Count || id:ebi-a-GCST90001477 | rs11184341 | 1 | 105422565 | C | -0.0655855 | 0.0142236 | 4.06E-06 |
| ebi-a-GCST90016961 | Gut microbiota abundance (genus Akkermansia id.4037) || id:ebi-a-GCST90016961 | Basophil %CD33dim HLA DR- CD66b- || id:ebi-a-GCST90001533 | rs11184341 | 1 | 105422565 | C | -0.0655855 | 0.0142236 | 4.06E-06 |
| ebi-a-GCST90016961 | Gut microbiota abundance (genus Akkermansia id.4037) || id:ebi-a-GCST90016961 | CD16+ monocyte %monocyte || id:ebi-a-GCST90001587 | rs11184341 | 1 | 105422565 | C | -0.0655855 | 0.0142236 | 4.06E-06 |
| ebi-a-GCST90016961 | Gut microbiota abundance (genus Akkermansia id.4037) || id:ebi-a-GCST90016961 | T/B cell || id:ebi-a-GCST90001588 | rs11184341 | 1 | 105422565 | C | -0.0655855 | 0.0142236 | 4.06E-06 |
| ebi-a-GCST90016961 | Gut microbiota abundance (genus Akkermansia id.4037) || id:ebi-a-GCST90016961 | CD4+CD8+ T cell %T cell || id:ebi-a-GCST90001595 | rs11184341 | 1 | 105422565 | C | -0.0655855 | 0.0142236 | 4.06E-06 |
| ebi-a-GCST90016961 | Gut microbiota abundance (genus Akkermansia id.4037) || id:ebi-a-GCST90016961 | CD8+ Natural Killer T %T cell || id:ebi-a-GCST90001631 | rs11184341 | 1 | 105422565 | C | -0.0655855 | 0.0142236 | 4.06E-06 |
| ebi-a-GCST90016961 | Gut microbiota abundance (genus Akkermansia id.4037) || id:ebi-a-GCST90016961 | CD4-CD8- Natural Killer T %lymphocyte || id:ebi-a-GCST90001638 | rs11184341 | 1 | 105422565 | C | -0.0655855 | 0.0142236 | 4.06E-06 |
| ebi-a-GCST90016961 | Gut microbiota abundance (genus Akkermansia id.4037) || id:ebi-a-GCST90016961 | HLA DR+ Natural Killer Absolute Count || id:ebi-a-GCST90001648 | rs11184341 | 1 | 105422565 | C | -0.0655855 | 0.0142236 | 4.06E-06 |
| ebi-a-GCST90016961 | Gut microbiota abundance (genus Akkermansia id.4037) || id:ebi-a-GCST90016961 | HLA DR+ Natural Killer %Natural Killer || id:ebi-a-GCST90001649 | rs11184341 | 1 | 105422565 | C | -0.0655855 | 0.0142236 | 4.06E-06 |
| ebi-a-GCST90016961 | Gut microbiota abundance (genus Akkermansia id.4037) || id:ebi-a-GCST90016961 | HLA DR+ Natural Killer %CD3- lymphocyte || id:ebi-a-GCST90001650 | rs11184341 | 1 | 105422565 | C | -0.0655855 | 0.0142236 | 4.06E-06 |
| ebi-a-GCST90016961 | Gut microbiota abundance (genus Akkermansia id.4037) || id:ebi-a-GCST90016961 | CD28- CD25++ CD8+ T cell Absolute Count || id:ebi-a-GCST90001678 | rs11184341 | 1 | 105422565 | C | -0.0655855 | 0.0142236 | 4.06E-06 |
| ebi-a-GCST90016961 | Gut microbiota abundance (genus Akkermansia id.4037) || id:ebi-a-GCST90016961 | CD25++ CD8+ T cell %T cell || id:ebi-a-GCST90001679 | rs11184341 | 1 | 105422565 | C | -0.0655855 | 0.0142236 | 4.06E-06 |
| ebi-a-GCST90016961 | Gut microbiota abundance (genus Akkermansia id.4037) || id:ebi-a-GCST90016961 | CD19 on CD20- CD38- B cell || id:ebi-a-GCST90001722 | rs11184341 | 1 | 105422565 | C | -0.0655855 | 0.0142236 | 4.06E-06 |
| ebi-a-GCST90016961 | Gut microbiota abundance (genus Akkermansia id.4037) || id:ebi-a-GCST90016961 | CD25 on B cell || id:ebi-a-GCST90001775 | rs11184341 | 1 | 105422565 | C | -0.0655855 | 0.0142236 | 4.06E-06 |
| ebi-a-GCST90016961 | Gut microbiota abundance (genus Akkermansia id.4037) || id:ebi-a-GCST90016961 | CD25 on IgD+ CD24- B cell || id:ebi-a-GCST90001779 | rs11184341 | 1 | 105422565 | C | -0.0655855 | 0.0142236 | 4.06E-06 |
| ebi-a-GCST90016961 | Gut microbiota abundance (genus Akkermansia id.4037) || id:ebi-a-GCST90016961 | CD25 on IgD+ CD38- naive B cell || id:ebi-a-GCST90001781 | rs11184341 | 1 | 105422565 | C | -0.0655855 | 0.0142236 | 4.06E-06 |
| ebi-a-GCST90016961 | Gut microbiota abundance (genus Akkermansia id.4037) || id:ebi-a-GCST90016961 | CD25 on IgD+ CD38+ B cell || id:ebi-a-GCST90001783 | rs11184341 | 1 | 105422565 | C | -0.0655855 | 0.0142236 | 4.06E-06 |
| ebi-a-GCST90016961 | Gut microbiota abundance (genus Akkermansia id.4037) || id:ebi-a-GCST90016961 | CD86 on myeloid Dendritic Cell || id:ebi-a-GCST90001903 | rs11184341 | 1 | 105422565 | C | -0.0655855 | 0.0142236 | 4.06E-06 |
| ebi-a-GCST90016961 | Gut microbiota abundance (genus Akkermansia id.4037) || id:ebi-a-GCST90016961 | CD33 on CD14+ monocyte || id:ebi-a-GCST90001946 | rs11184341 | 1 | 105422565 | C | -0.0655855 | 0.0142236 | 4.06E-06 |
| ebi-a-GCST90016961 | Gut microbiota abundance (genus Akkermansia id.4037) || id:ebi-a-GCST90016961 | CD33 on CD33+ HLA DR+ CD14dim || id:ebi-a-GCST90001947 | rs11184341 | 1 | 105422565 | C | -0.0655855 | 0.0142236 | 4.06E-06 |
| ebi-a-GCST90016961 | Gut microbiota abundance (genus Akkermansia id.4037) || id:ebi-a-GCST90016961 | CD33 on CD33dim HLA DR+ CD11b+ || id:ebi-a-GCST90001948 | rs11184341 | 1 | 105422565 | C | -0.0655855 | 0.0142236 | 4.06E-06 |
| ebi-a-GCST90016961 | Gut microbiota abundance (genus Akkermansia id.4037) || id:ebi-a-GCST90016961 | CD33 on Granulocytic Myeloid-Derived Suppressor Cells || id:ebi-a-GCST90001950 | rs11184341 | 1 | 105422565 | C | -0.0655855 | 0.0142236 | 4.06E-06 |
| ebi-a-GCST90016961 | Gut microbiota abundance (genus Akkermansia id.4037) || id:ebi-a-GCST90016961 | CD33 on CD66b++ myeloid cell || id:ebi-a-GCST90001951 | rs11184341 | 1 | 105422565 | C | -0.0655855 | 0.0142236 | 4.06E-06 |
| ebi-a-GCST90016961 | Gut microbiota abundance (genus Akkermansia id.4037) || id:ebi-a-GCST90016961 | CD33 on CD33dim HLA DR- || id:ebi-a-GCST90001953 | rs11184341 | 1 | 105422565 | C | -0.0655855 | 0.0142236 | 4.06E-06 |
| ebi-a-GCST90016961 | Gut microbiota abundance (genus Akkermansia id.4037) || id:ebi-a-GCST90016961 | CD33 on basophil || id:ebi-a-GCST90001954 | rs11184341 | 1 | 105422565 | C | -0.0655855 | 0.0142236 | 4.06E-06 |
| ebi-a-GCST90016961 | Gut microbiota abundance (genus Akkermansia id.4037) || id:ebi-a-GCST90016961 | CD33 on Immature Myeloid-Derived Suppressor Cells || id:ebi-a-GCST90001955 | rs11184341 | 1 | 105422565 | C | -0.0655855 | 0.0142236 | 4.06E-06 |
| ebi-a-GCST90016961 | Gut microbiota abundance (genus Akkermansia id.4037) || id:ebi-a-GCST90016961 | FSC-A on HLA DR+ CD8+ T cell || id:ebi-a-GCST90001978 | rs11184341 | 1 | 105422565 | C | -0.0655855 | 0.0142236 | 4.06E-06 |
| ebi-a-GCST90016961 | Gut microbiota abundance (genus Akkermansia id.4037) || id:ebi-a-GCST90016961 | HLA DR on CD14+ CD16- monocyte || id:ebi-a-GCST90001988 | rs11184341 | 1 | 105422565 | C | -0.0655855 | 0.0142236 | 4.06E-06 |
| ebi-a-GCST90016961 | Gut microbiota abundance (genus Akkermansia id.4037) || id:ebi-a-GCST90016961 | HLA DR on CD14+ monocyte || id:ebi-a-GCST90001991 | rs11184341 | 1 | 105422565 | C | -0.0655855 | 0.0142236 | 4.06E-06 |
| ebi-a-GCST90016961 | Gut microbiota abundance (genus Akkermansia id.4037) || id:ebi-a-GCST90016961 | CD16 on CD14+ CD16+ monocyte || id:ebi-a-GCST90002005 | rs11184341 | 1 | 105422565 | C | -0.0655855 | 0.0142236 | 4.06E-06 |
| ebi-a-GCST90016961 | Gut microbiota abundance (genus Akkermansia id.4037) || id:ebi-a-GCST90016961 | CD45 on CD33+ HLA DR+ CD14- || id:ebi-a-GCST90002042 | rs11184341 | 1 | 105422565 | C | -0.0655855 | 0.0142236 | 4.06E-06 |
| ebi-a-GCST90016961 | Gut microbiota abundance (genus Akkermansia id.4037) || id:ebi-a-GCST90016961 | CD8 on Natural Killer T || id:ebi-a-GCST90002059 | rs11184341 | 1 | 105422565 | C | -0.0655855 | 0.0142236 | 4.06E-06 |
| ebi-a-GCST90016957 | Gut microbiota abundance (family Verrucomicrobiaceae id.4036) || id:ebi-a-GCST90016957 | Myeloid Dendritic Cell Absolute Count || id:ebi-a-GCST90001458 | rs111862613 | 12 | 130309670 | T | 0.0907065 | 0.0196746 | 3.73E-06 |
| ebi-a-GCST90016957 | Gut microbiota abundance (family Verrucomicrobiaceae id.4036) || id:ebi-a-GCST90016957 | CD86+ myeloid Dendritic Cell %Dendritic Cell || id:ebi-a-GCST90001465 | rs111862613 | 12 | 130309670 | T | 0.0907065 | 0.0196746 | 3.73E-06 |
| ebi-a-GCST90016957 | Gut microbiota abundance (family Verrucomicrobiaceae id.4036) || id:ebi-a-GCST90016957 | CD62L- myeloid Dendritic Cell Absolute Count || id:ebi-a-GCST90001468 | rs111862613 | 12 | 130309670 | T | 0.0907065 | 0.0196746 | 3.73E-06 |
| ebi-a-GCST90016957 | Gut microbiota abundance (family Verrucomicrobiaceae id.4036) || id:ebi-a-GCST90016957 | CD62L- myeloid Dendritic Cell %Dendritic Cell || id:ebi-a-GCST90001469 | rs111862613 | 12 | 130309670 | T | 0.0907065 | 0.0196746 | 3.73E-06 |
| ebi-a-GCST90016957 | Gut microbiota abundance (family Verrucomicrobiaceae id.4036) || id:ebi-a-GCST90016957 | CD62L- CD86+ myeloid Dendritic Cell Absolute Count || id:ebi-a-GCST90001472 | rs111862613 | 12 | 130309670 | T | 0.0907065 | 0.0196746 | 3.73E-06 |
| ebi-a-GCST90016957 | Gut microbiota abundance (family Verrucomicrobiaceae id.4036) || id:ebi-a-GCST90016957 | CD62L- CD86+ myeloid Dendritic Cell %Dendritic Cell || id:ebi-a-GCST90001473 | rs111862613 | 12 | 130309670 | T | 0.0907065 | 0.0196746 | 3.73E-06 |
| ebi-a-GCST90016957 | Gut microbiota abundance (family Verrucomicrobiaceae id.4036) || id:ebi-a-GCST90016957 | HLA DR++ monocyte %monocyte || id:ebi-a-GCST90001475 | rs111862613 | 12 | 130309670 | T | 0.0907065 | 0.0196746 | 3.73E-06 |
| ebi-a-GCST90016957 | Gut microbiota abundance (family Verrucomicrobiaceae id.4036) || id:ebi-a-GCST90016957 | HLA DR++ monocyte Absolute Count || id:ebi-a-GCST90001477 | rs111862613 | 12 | 130309670 | T | 0.0907065 | 0.0196746 | 3.73E-06 |
| ebi-a-GCST90016957 | Gut microbiota abundance (family Verrucomicrobiaceae id.4036) || id:ebi-a-GCST90016957 | Basophil %CD33dim HLA DR- CD66b- || id:ebi-a-GCST90001533 | rs111862613 | 12 | 130309670 | T | 0.0907065 | 0.0196746 | 3.73E-06 |
| ebi-a-GCST90016957 | Gut microbiota abundance (family Verrucomicrobiaceae id.4036) || id:ebi-a-GCST90016957 | CD16+ monocyte %monocyte || id:ebi-a-GCST90001587 | rs111862613 | 12 | 130309670 | T | 0.0907065 | 0.0196746 | 3.73E-06 |
| ebi-a-GCST90016957 | Gut microbiota abundance (family Verrucomicrobiaceae id.4036) || id:ebi-a-GCST90016957 | T/B cell || id:ebi-a-GCST90001588 | rs111862613 | 12 | 130309670 | T | 0.0907065 | 0.0196746 | 3.73E-06 |
| ebi-a-GCST90016957 | Gut microbiota abundance (family Verrucomicrobiaceae id.4036) || id:ebi-a-GCST90016957 | CD8+ Natural Killer T %T cell || id:ebi-a-GCST90001631 | rs111862613 | 12 | 130309670 | T | 0.0907065 | 0.0196746 | 3.73E-06 |
| ebi-a-GCST90016957 | Gut microbiota abundance (family Verrucomicrobiaceae id.4036) || id:ebi-a-GCST90016957 | CD4-CD8- Natural Killer T %lymphocyte || id:ebi-a-GCST90001638 | rs111862613 | 12 | 130309670 | T | 0.0907065 | 0.0196746 | 3.73E-06 |
| ebi-a-GCST90016957 | Gut microbiota abundance (family Verrucomicrobiaceae id.4036) || id:ebi-a-GCST90016957 | HLA DR+ Natural Killer Absolute Count || id:ebi-a-GCST90001648 | rs111862613 | 12 | 130309670 | T | 0.0907065 | 0.0196746 | 3.73E-06 |
| ebi-a-GCST90016957 | Gut microbiota abundance (family Verrucomicrobiaceae id.4036) || id:ebi-a-GCST90016957 | HLA DR+ Natural Killer %Natural Killer || id:ebi-a-GCST90001649 | rs111862613 | 12 | 130309670 | T | 0.0907065 | 0.0196746 | 3.73E-06 |
| ebi-a-GCST90016957 | Gut microbiota abundance (family Verrucomicrobiaceae id.4036) || id:ebi-a-GCST90016957 | HLA DR+ Natural Killer %CD3- lymphocyte || id:ebi-a-GCST90001650 | rs111862613 | 12 | 130309670 | T | 0.0907065 | 0.0196746 | 3.73E-06 |
| ebi-a-GCST90016957 | Gut microbiota abundance (family Verrucomicrobiaceae id.4036) || id:ebi-a-GCST90016957 | CD28- CD25++ CD8+ T cell Absolute Count || id:ebi-a-GCST90001678 | rs111862613 | 12 | 130309670 | T | 0.0907065 | 0.0196746 | 3.73E-06 |
| ebi-a-GCST90016957 | Gut microbiota abundance (family Verrucomicrobiaceae id.4036) || id:ebi-a-GCST90016957 | CD25++ CD8+ T cell %T cell || id:ebi-a-GCST90001679 | rs111862613 | 12 | 130309670 | T | 0.0907065 | 0.0196746 | 3.73E-06 |
| ebi-a-GCST90016957 | Gut microbiota abundance (family Verrucomicrobiaceae id.4036) || id:ebi-a-GCST90016957 | CD19 on CD20- CD38- B cell || id:ebi-a-GCST90001722 | rs111862613 | 12 | 130309670 | T | 0.0907065 | 0.0196746 | 3.73E-06 |
| ebi-a-GCST90016957 | Gut microbiota abundance (family Verrucomicrobiaceae id.4036) || id:ebi-a-GCST90016957 | CD25 on B cell || id:ebi-a-GCST90001775 | rs111862613 | 12 | 130309670 | T | 0.0907065 | 0.0196746 | 3.73E-06 |
| ebi-a-GCST90016957 | Gut microbiota abundance (family Verrucomicrobiaceae id.4036) || id:ebi-a-GCST90016957 | CD25 on IgD+ CD24- B cell || id:ebi-a-GCST90001779 | rs111862613 | 12 | 130309670 | T | 0.0907065 | 0.0196746 | 3.73E-06 |
| ebi-a-GCST90016957 | Gut microbiota abundance (family Verrucomicrobiaceae id.4036) || id:ebi-a-GCST90016957 | CD25 on IgD+ CD38- naive B cell || id:ebi-a-GCST90001781 | rs111862613 | 12 | 130309670 | T | 0.0907065 | 0.0196746 | 3.73E-06 |
| ebi-a-GCST90016957 | Gut microbiota abundance (family Verrucomicrobiaceae id.4036) || id:ebi-a-GCST90016957 | CD25 on IgD+ CD38+ B cell || id:ebi-a-GCST90001783 | rs111862613 | 12 | 130309670 | T | 0.0907065 | 0.0196746 | 3.73E-06 |
| ebi-a-GCST90016957 | Gut microbiota abundance (family Verrucomicrobiaceae id.4036) || id:ebi-a-GCST90016957 | CD86 on myeloid Dendritic Cell || id:ebi-a-GCST90001903 | rs111862613 | 12 | 130309670 | T | 0.0907065 | 0.0196746 | 3.73E-06 |
| ebi-a-GCST90016957 | Gut microbiota abundance (family Verrucomicrobiaceae id.4036) || id:ebi-a-GCST90016957 | CD33 on CD14+ monocyte || id:ebi-a-GCST90001946 | rs111862613 | 12 | 130309670 | T | 0.0907065 | 0.0196746 | 3.73E-06 |
| ebi-a-GCST90016957 | Gut microbiota abundance (family Verrucomicrobiaceae id.4036) || id:ebi-a-GCST90016957 | CD33 on CD33+ HLA DR+ CD14dim || id:ebi-a-GCST90001947 | rs111862613 | 12 | 130309670 | T | 0.0907065 | 0.0196746 | 3.73E-06 |
| ebi-a-GCST90016957 | Gut microbiota abundance (family Verrucomicrobiaceae id.4036) || id:ebi-a-GCST90016957 | CD33 on CD33dim HLA DR+ CD11b+ || id:ebi-a-GCST90001948 | rs111862613 | 12 | 130309670 | T | 0.0907065 | 0.0196746 | 3.73E-06 |
| ebi-a-GCST90016957 | Gut microbiota abundance (family Verrucomicrobiaceae id.4036) || id:ebi-a-GCST90016957 | CD33 on Granulocytic Myeloid-Derived Suppressor Cells || id:ebi-a-GCST90001950 | rs111862613 | 12 | 130309670 | T | 0.0907065 | 0.0196746 | 3.73E-06 |
| ebi-a-GCST90016957 | Gut microbiota abundance (family Verrucomicrobiaceae id.4036) || id:ebi-a-GCST90016957 | CD33 on CD66b++ myeloid cell || id:ebi-a-GCST90001951 | rs111862613 | 12 | 130309670 | T | 0.0907065 | 0.0196746 | 3.73E-06 |
| ebi-a-GCST90016957 | Gut microbiota abundance (family Verrucomicrobiaceae id.4036) || id:ebi-a-GCST90016957 | CD33 on CD33dim HLA DR- || id:ebi-a-GCST90001953 | rs111862613 | 12 | 130309670 | T | 0.0907065 | 0.0196746 | 3.73E-06 |
| ebi-a-GCST90016957 | Gut microbiota abundance (family Verrucomicrobiaceae id.4036) || id:ebi-a-GCST90016957 | CD33 on basophil || id:ebi-a-GCST90001954 | rs111862613 | 12 | 130309670 | T | 0.0907065 | 0.0196746 | 3.73E-06 |
| ebi-a-GCST90016957 | Gut microbiota abundance (family Verrucomicrobiaceae id.4036) || id:ebi-a-GCST90016957 | CD33 on Immature Myeloid-Derived Suppressor Cells || id:ebi-a-GCST90001955 | rs111862613 | 12 | 130309670 | T | 0.0907065 | 0.0196746 | 3.73E-06 |
| ebi-a-GCST90016957 | Gut microbiota abundance (family Verrucomicrobiaceae id.4036) || id:ebi-a-GCST90016957 | FSC-A on HLA DR+ CD8+ T cell || id:ebi-a-GCST90001978 | rs111862613 | 12 | 130309670 | T | 0.0907065 | 0.0196746 | 3.73E-06 |
| ebi-a-GCST90016957 | Gut microbiota abundance (family Verrucomicrobiaceae id.4036) || id:ebi-a-GCST90016957 | HLA DR on CD14+ CD16- monocyte || id:ebi-a-GCST90001988 | rs111862613 | 12 | 130309670 | T | 0.0907065 | 0.0196746 | 3.73E-06 |
| ebi-a-GCST90016957 | Gut microbiota abundance (family Verrucomicrobiaceae id.4036) || id:ebi-a-GCST90016957 | HLA DR on CD14+ monocyte || id:ebi-a-GCST90001991 | rs111862613 | 12 | 130309670 | T | 0.0907065 | 0.0196746 | 3.73E-06 |
| ebi-a-GCST90016957 | Gut microbiota abundance (family Verrucomicrobiaceae id.4036) || id:ebi-a-GCST90016957 | CD16 on CD14+ CD16+ monocyte || id:ebi-a-GCST90002005 | rs111862613 | 12 | 130309670 | T | 0.0907065 | 0.0196746 | 3.73E-06 |
| ebi-a-GCST90016957 | Gut microbiota abundance (family Verrucomicrobiaceae id.4036) || id:ebi-a-GCST90016957 | CD45 on CD33+ HLA DR+ CD14- || id:ebi-a-GCST90002042 | rs111862613 | 12 | 130309670 | T | 0.0907065 | 0.0196746 | 3.73E-06 |
| ebi-a-GCST90016957 | Gut microbiota abundance (family Verrucomicrobiaceae id.4036) || id:ebi-a-GCST90016957 | CD8 on Natural Killer T || id:ebi-a-GCST90002059 | rs111862613 | 12 | 130309670 | T | 0.0907065 | 0.0196746 | 3.73E-06 |
| ebi-a-GCST90016923 | Gut microbiota abundance (class Verrucomicrobiae id.4029) || id:ebi-a-GCST90016923 | Myeloid Dendritic Cell Absolute Count || id:ebi-a-GCST90001458 | rs111862613 | 12 | 130309670 | T | 0.090699 | 0.0196746 | 3.74E-06 |
| ebi-a-GCST90016923 | Gut microbiota abundance (class Verrucomicrobiae id.4029) || id:ebi-a-GCST90016923 | CD86+ myeloid Dendritic Cell %Dendritic Cell || id:ebi-a-GCST90001465 | rs111862613 | 12 | 130309670 | T | 0.090699 | 0.0196746 | 3.74E-06 |
| ebi-a-GCST90016923 | Gut microbiota abundance (class Verrucomicrobiae id.4029) || id:ebi-a-GCST90016923 | CD62L- myeloid Dendritic Cell Absolute Count || id:ebi-a-GCST90001468 | rs111862613 | 12 | 130309670 | T | 0.090699 | 0.0196746 | 3.74E-06 |
| ebi-a-GCST90016923 | Gut microbiota abundance (class Verrucomicrobiae id.4029) || id:ebi-a-GCST90016923 | CD62L- myeloid Dendritic Cell %Dendritic Cell || id:ebi-a-GCST90001469 | rs111862613 | 12 | 130309670 | T | 0.090699 | 0.0196746 | 3.74E-06 |
| ebi-a-GCST90016923 | Gut microbiota abundance (class Verrucomicrobiae id.4029) || id:ebi-a-GCST90016923 | CD62L- CD86+ myeloid Dendritic Cell Absolute Count || id:ebi-a-GCST90001472 | rs111862613 | 12 | 130309670 | T | 0.090699 | 0.0196746 | 3.74E-06 |
| ebi-a-GCST90016923 | Gut microbiota abundance (class Verrucomicrobiae id.4029) || id:ebi-a-GCST90016923 | CD62L- CD86+ myeloid Dendritic Cell %Dendritic Cell || id:ebi-a-GCST90001473 | rs111862613 | 12 | 130309670 | T | 0.090699 | 0.0196746 | 3.74E-06 |
| ebi-a-GCST90016923 | Gut microbiota abundance (class Verrucomicrobiae id.4029) || id:ebi-a-GCST90016923 | HLA DR++ monocyte %monocyte || id:ebi-a-GCST90001475 | rs111862613 | 12 | 130309670 | T | 0.090699 | 0.0196746 | 3.74E-06 |
| ebi-a-GCST90016923 | Gut microbiota abundance (class Verrucomicrobiae id.4029) || id:ebi-a-GCST90016923 | HLA DR++ monocyte Absolute Count || id:ebi-a-GCST90001477 | rs111862613 | 12 | 130309670 | T | 0.090699 | 0.0196746 | 3.74E-06 |
| ebi-a-GCST90016923 | Gut microbiota abundance (class Verrucomicrobiae id.4029) || id:ebi-a-GCST90016923 | Basophil %CD33dim HLA DR- CD66b- || id:ebi-a-GCST90001533 | rs111862613 | 12 | 130309670 | T | 0.090699 | 0.0196746 | 3.74E-06 |
| ebi-a-GCST90016923 | Gut microbiota abundance (class Verrucomicrobiae id.4029) || id:ebi-a-GCST90016923 | CD16+ monocyte %monocyte || id:ebi-a-GCST90001587 | rs111862613 | 12 | 130309670 | T | 0.090699 | 0.0196746 | 3.74E-06 |
| ebi-a-GCST90016923 | Gut microbiota abundance (class Verrucomicrobiae id.4029) || id:ebi-a-GCST90016923 | T/B cell || id:ebi-a-GCST90001588 | rs111862613 | 12 | 130309670 | T | 0.090699 | 0.0196746 | 3.74E-06 |
| ebi-a-GCST90016923 | Gut microbiota abundance (class Verrucomicrobiae id.4029) || id:ebi-a-GCST90016923 | CD8+ Natural Killer T %T cell || id:ebi-a-GCST90001631 | rs111862613 | 12 | 130309670 | T | 0.090699 | 0.0196746 | 3.74E-06 |
| ebi-a-GCST90016923 | Gut microbiota abundance (class Verrucomicrobiae id.4029) || id:ebi-a-GCST90016923 | CD4-CD8- Natural Killer T %lymphocyte || id:ebi-a-GCST90001638 | rs111862613 | 12 | 130309670 | T | 0.090699 | 0.0196746 | 3.74E-06 |
| ebi-a-GCST90016923 | Gut microbiota abundance (class Verrucomicrobiae id.4029) || id:ebi-a-GCST90016923 | HLA DR+ Natural Killer Absolute Count || id:ebi-a-GCST90001648 | rs111862613 | 12 | 130309670 | T | 0.090699 | 0.0196746 | 3.74E-06 |
| ebi-a-GCST90016923 | Gut microbiota abundance (class Verrucomicrobiae id.4029) || id:ebi-a-GCST90016923 | HLA DR+ Natural Killer %Natural Killer || id:ebi-a-GCST90001649 | rs111862613 | 12 | 130309670 | T | 0.090699 | 0.0196746 | 3.74E-06 |
| ebi-a-GCST90016923 | Gut microbiota abundance (class Verrucomicrobiae id.4029) || id:ebi-a-GCST90016923 | HLA DR+ Natural Killer %CD3- lymphocyte || id:ebi-a-GCST90001650 | rs111862613 | 12 | 130309670 | T | 0.090699 | 0.0196746 | 3.74E-06 |
| ebi-a-GCST90016923 | Gut microbiota abundance (class Verrucomicrobiae id.4029) || id:ebi-a-GCST90016923 | CD28- CD25++ CD8+ T cell Absolute Count || id:ebi-a-GCST90001678 | rs111862613 | 12 | 130309670 | T | 0.090699 | 0.0196746 | 3.74E-06 |
| ebi-a-GCST90016923 | Gut microbiota abundance (class Verrucomicrobiae id.4029) || id:ebi-a-GCST90016923 | CD25++ CD8+ T cell %T cell || id:ebi-a-GCST90001679 | rs111862613 | 12 | 130309670 | T | 0.090699 | 0.0196746 | 3.74E-06 |
| ebi-a-GCST90016923 | Gut microbiota abundance (class Verrucomicrobiae id.4029) || id:ebi-a-GCST90016923 | CD19 on CD20- CD38- B cell || id:ebi-a-GCST90001722 | rs111862613 | 12 | 130309670 | T | 0.090699 | 0.0196746 | 3.74E-06 |
| ebi-a-GCST90016923 | Gut microbiota abundance (class Verrucomicrobiae id.4029) || id:ebi-a-GCST90016923 | CD25 on B cell || id:ebi-a-GCST90001775 | rs111862613 | 12 | 130309670 | T | 0.090699 | 0.0196746 | 3.74E-06 |
| ebi-a-GCST90016923 | Gut microbiota abundance (class Verrucomicrobiae id.4029) || id:ebi-a-GCST90016923 | CD25 on IgD+ CD24- B cell || id:ebi-a-GCST90001779 | rs111862613 | 12 | 130309670 | T | 0.090699 | 0.0196746 | 3.74E-06 |
| ebi-a-GCST90016923 | Gut microbiota abundance (class Verrucomicrobiae id.4029) || id:ebi-a-GCST90016923 | CD25 on IgD+ CD38- naive B cell || id:ebi-a-GCST90001781 | rs111862613 | 12 | 130309670 | T | 0.090699 | 0.0196746 | 3.74E-06 |
| ebi-a-GCST90016923 | Gut microbiota abundance (class Verrucomicrobiae id.4029) || id:ebi-a-GCST90016923 | CD25 on IgD+ CD38+ B cell || id:ebi-a-GCST90001783 | rs111862613 | 12 | 130309670 | T | 0.090699 | 0.0196746 | 3.74E-06 |
| ebi-a-GCST90016923 | Gut microbiota abundance (class Verrucomicrobiae id.4029) || id:ebi-a-GCST90016923 | CD86 on myeloid Dendritic Cell || id:ebi-a-GCST90001903 | rs111862613 | 12 | 130309670 | T | 0.090699 | 0.0196746 | 3.74E-06 |
| ebi-a-GCST90016923 | Gut microbiota abundance (class Verrucomicrobiae id.4029) || id:ebi-a-GCST90016923 | CD33 on CD14+ monocyte || id:ebi-a-GCST90001946 | rs111862613 | 12 | 130309670 | T | 0.090699 | 0.0196746 | 3.74E-06 |
| ebi-a-GCST90016923 | Gut microbiota abundance (class Verrucomicrobiae id.4029) || id:ebi-a-GCST90016923 | CD33 on CD33+ HLA DR+ CD14dim || id:ebi-a-GCST90001947 | rs111862613 | 12 | 130309670 | T | 0.090699 | 0.0196746 | 3.74E-06 |
| ebi-a-GCST90016923 | Gut microbiota abundance (class Verrucomicrobiae id.4029) || id:ebi-a-GCST90016923 | CD33 on CD33dim HLA DR+ CD11b+ || id:ebi-a-GCST90001948 | rs111862613 | 12 | 130309670 | T | 0.090699 | 0.0196746 | 3.74E-06 |
| ebi-a-GCST90016923 | Gut microbiota abundance (class Verrucomicrobiae id.4029) || id:ebi-a-GCST90016923 | CD33 on Granulocytic Myeloid-Derived Suppressor Cells || id:ebi-a-GCST90001950 | rs111862613 | 12 | 130309670 | T | 0.090699 | 0.0196746 | 3.74E-06 |
| ebi-a-GCST90016923 | Gut microbiota abundance (class Verrucomicrobiae id.4029) || id:ebi-a-GCST90016923 | CD33 on CD66b++ myeloid cell || id:ebi-a-GCST90001951 | rs111862613 | 12 | 130309670 | T | 0.090699 | 0.0196746 | 3.74E-06 |
| ebi-a-GCST90016923 | Gut microbiota abundance (class Verrucomicrobiae id.4029) || id:ebi-a-GCST90016923 | CD33 on CD33dim HLA DR- || id:ebi-a-GCST90001953 | rs111862613 | 12 | 130309670 | T | 0.090699 | 0.0196746 | 3.74E-06 |
| ebi-a-GCST90016923 | Gut microbiota abundance (class Verrucomicrobiae id.4029) || id:ebi-a-GCST90016923 | CD33 on basophil || id:ebi-a-GCST90001954 | rs111862613 | 12 | 130309670 | T | 0.090699 | 0.0196746 | 3.74E-06 |
| ebi-a-GCST90016923 | Gut microbiota abundance (class Verrucomicrobiae id.4029) || id:ebi-a-GCST90016923 | CD33 on Immature Myeloid-Derived Suppressor Cells || id:ebi-a-GCST90001955 | rs111862613 | 12 | 130309670 | T | 0.090699 | 0.0196746 | 3.74E-06 |
| ebi-a-GCST90016923 | Gut microbiota abundance (class Verrucomicrobiae id.4029) || id:ebi-a-GCST90016923 | FSC-A on HLA DR+ CD8+ T cell || id:ebi-a-GCST90001978 | rs111862613 | 12 | 130309670 | T | 0.090699 | 0.0196746 | 3.74E-06 |
| ebi-a-GCST90016923 | Gut microbiota abundance (class Verrucomicrobiae id.4029) || id:ebi-a-GCST90016923 | HLA DR on CD14+ CD16- monocyte || id:ebi-a-GCST90001988 | rs111862613 | 12 | 130309670 | T | 0.090699 | 0.0196746 | 3.74E-06 |
| ebi-a-GCST90016923 | Gut microbiota abundance (class Verrucomicrobiae id.4029) || id:ebi-a-GCST90016923 | HLA DR on CD14+ monocyte || id:ebi-a-GCST90001991 | rs111862613 | 12 | 130309670 | T | 0.090699 | 0.0196746 | 3.74E-06 |
| ebi-a-GCST90016923 | Gut microbiota abundance (class Verrucomicrobiae id.4029) || id:ebi-a-GCST90016923 | CD16 on CD14+ CD16+ monocyte || id:ebi-a-GCST90002005 | rs111862613 | 12 | 130309670 | T | 0.090699 | 0.0196746 | 3.74E-06 |
| ebi-a-GCST90016923 | Gut microbiota abundance (class Verrucomicrobiae id.4029) || id:ebi-a-GCST90016923 | CD45 on CD33+ HLA DR+ CD14- || id:ebi-a-GCST90002042 | rs111862613 | 12 | 130309670 | T | 0.090699 | 0.0196746 | 3.74E-06 |
| ebi-a-GCST90016923 | Gut microbiota abundance (class Verrucomicrobiae id.4029) || id:ebi-a-GCST90016923 | CD8 on Natural Killer T || id:ebi-a-GCST90002059 | rs111862613 | 12 | 130309670 | T | 0.090699 | 0.0196746 | 3.74E-06 |
| ebi-a-GCST90017108 | Gut microbiota abundance (order Verrucomicrobiales id.4030) || id:ebi-a-GCST90017108 | Myeloid Dendritic Cell Absolute Count || id:ebi-a-GCST90001458 | rs111862613 | 12 | 130309670 | T | 0.090699 | 0.0196746 | 3.74E-06 |
| ebi-a-GCST90017108 | Gut microbiota abundance (order Verrucomicrobiales id.4030) || id:ebi-a-GCST90017108 | CD86+ myeloid Dendritic Cell %Dendritic Cell || id:ebi-a-GCST90001465 | rs111862613 | 12 | 130309670 | T | 0.090699 | 0.0196746 | 3.74E-06 |
| ebi-a-GCST90017108 | Gut microbiota abundance (order Verrucomicrobiales id.4030) || id:ebi-a-GCST90017108 | CD62L- myeloid Dendritic Cell Absolute Count || id:ebi-a-GCST90001468 | rs111862613 | 12 | 130309670 | T | 0.090699 | 0.0196746 | 3.74E-06 |
| ebi-a-GCST90017108 | Gut microbiota abundance (order Verrucomicrobiales id.4030) || id:ebi-a-GCST90017108 | CD62L- myeloid Dendritic Cell %Dendritic Cell || id:ebi-a-GCST90001469 | rs111862613 | 12 | 130309670 | T | 0.090699 | 0.0196746 | 3.74E-06 |
| ebi-a-GCST90017108 | Gut microbiota abundance (order Verrucomicrobiales id.4030) || id:ebi-a-GCST90017108 | CD62L- CD86+ myeloid Dendritic Cell Absolute Count || id:ebi-a-GCST90001472 | rs111862613 | 12 | 130309670 | T | 0.090699 | 0.0196746 | 3.74E-06 |
| ebi-a-GCST90017108 | Gut microbiota abundance (order Verrucomicrobiales id.4030) || id:ebi-a-GCST90017108 | CD62L- CD86+ myeloid Dendritic Cell %Dendritic Cell || id:ebi-a-GCST90001473 | rs111862613 | 12 | 130309670 | T | 0.090699 | 0.0196746 | 3.74E-06 |
| ebi-a-GCST90017108 | Gut microbiota abundance (order Verrucomicrobiales id.4030) || id:ebi-a-GCST90017108 | HLA DR++ monocyte %monocyte || id:ebi-a-GCST90001475 | rs111862613 | 12 | 130309670 | T | 0.090699 | 0.0196746 | 3.74E-06 |
| ebi-a-GCST90017108 | Gut microbiota abundance (order Verrucomicrobiales id.4030) || id:ebi-a-GCST90017108 | HLA DR++ monocyte Absolute Count || id:ebi-a-GCST90001477 | rs111862613 | 12 | 130309670 | T | 0.090699 | 0.0196746 | 3.74E-06 |
| ebi-a-GCST90017108 | Gut microbiota abundance (order Verrucomicrobiales id.4030) || id:ebi-a-GCST90017108 | Basophil %CD33dim HLA DR- CD66b- || id:ebi-a-GCST90001533 | rs111862613 | 12 | 130309670 | T | 0.090699 | 0.0196746 | 3.74E-06 |
| ebi-a-GCST90017108 | Gut microbiota abundance (order Verrucomicrobiales id.4030) || id:ebi-a-GCST90017108 | CD16+ monocyte %monocyte || id:ebi-a-GCST90001587 | rs111862613 | 12 | 130309670 | T | 0.090699 | 0.0196746 | 3.74E-06 |
| ebi-a-GCST90017108 | Gut microbiota abundance (order Verrucomicrobiales id.4030) || id:ebi-a-GCST90017108 | T/B cell || id:ebi-a-GCST90001588 | rs111862613 | 12 | 130309670 | T | 0.090699 | 0.0196746 | 3.74E-06 |
| ebi-a-GCST90017108 | Gut microbiota abundance (order Verrucomicrobiales id.4030) || id:ebi-a-GCST90017108 | CD8+ Natural Killer T %T cell || id:ebi-a-GCST90001631 | rs111862613 | 12 | 130309670 | T | 0.090699 | 0.0196746 | 3.74E-06 |
| ebi-a-GCST90017108 | Gut microbiota abundance (order Verrucomicrobiales id.4030) || id:ebi-a-GCST90017108 | CD4-CD8- Natural Killer T %lymphocyte || id:ebi-a-GCST90001638 | rs111862613 | 12 | 130309670 | T | 0.090699 | 0.0196746 | 3.74E-06 |
| ebi-a-GCST90017108 | Gut microbiota abundance (order Verrucomicrobiales id.4030) || id:ebi-a-GCST90017108 | HLA DR+ Natural Killer Absolute Count || id:ebi-a-GCST90001648 | rs111862613 | 12 | 130309670 | T | 0.090699 | 0.0196746 | 3.74E-06 |
| ebi-a-GCST90017108 | Gut microbiota abundance (order Verrucomicrobiales id.4030) || id:ebi-a-GCST90017108 | HLA DR+ Natural Killer %Natural Killer || id:ebi-a-GCST90001649 | rs111862613 | 12 | 130309670 | T | 0.090699 | 0.0196746 | 3.74E-06 |
| ebi-a-GCST90017108 | Gut microbiota abundance (order Verrucomicrobiales id.4030) || id:ebi-a-GCST90017108 | HLA DR+ Natural Killer %CD3- lymphocyte || id:ebi-a-GCST90001650 | rs111862613 | 12 | 130309670 | T | 0.090699 | 0.0196746 | 3.74E-06 |
| ebi-a-GCST90017108 | Gut microbiota abundance (order Verrucomicrobiales id.4030) || id:ebi-a-GCST90017108 | CD28- CD25++ CD8+ T cell Absolute Count || id:ebi-a-GCST90001678 | rs111862613 | 12 | 130309670 | T | 0.090699 | 0.0196746 | 3.74E-06 |
| ebi-a-GCST90017108 | Gut microbiota abundance (order Verrucomicrobiales id.4030) || id:ebi-a-GCST90017108 | CD25++ CD8+ T cell %T cell || id:ebi-a-GCST90001679 | rs111862613 | 12 | 130309670 | T | 0.090699 | 0.0196746 | 3.74E-06 |
| ebi-a-GCST90017108 | Gut microbiota abundance (order Verrucomicrobiales id.4030) || id:ebi-a-GCST90017108 | CD19 on CD20- CD38- B cell || id:ebi-a-GCST90001722 | rs111862613 | 12 | 130309670 | T | 0.090699 | 0.0196746 | 3.74E-06 |
| ebi-a-GCST90017108 | Gut microbiota abundance (order Verrucomicrobiales id.4030) || id:ebi-a-GCST90017108 | CD25 on B cell || id:ebi-a-GCST90001775 | rs111862613 | 12 | 130309670 | T | 0.090699 | 0.0196746 | 3.74E-06 |
| ebi-a-GCST90017108 | Gut microbiota abundance (order Verrucomicrobiales id.4030) || id:ebi-a-GCST90017108 | CD25 on IgD+ CD24- B cell || id:ebi-a-GCST90001779 | rs111862613 | 12 | 130309670 | T | 0.090699 | 0.0196746 | 3.74E-06 |
| ebi-a-GCST90017108 | Gut microbiota abundance (order Verrucomicrobiales id.4030) || id:ebi-a-GCST90017108 | CD25 on IgD+ CD38- naive B cell || id:ebi-a-GCST90001781 | rs111862613 | 12 | 130309670 | T | 0.090699 | 0.0196746 | 3.74E-06 |
| ebi-a-GCST90017108 | Gut microbiota abundance (order Verrucomicrobiales id.4030) || id:ebi-a-GCST90017108 | CD25 on IgD+ CD38+ B cell || id:ebi-a-GCST90001783 | rs111862613 | 12 | 130309670 | T | 0.090699 | 0.0196746 | 3.74E-06 |
| ebi-a-GCST90017108 | Gut microbiota abundance (order Verrucomicrobiales id.4030) || id:ebi-a-GCST90017108 | CD86 on myeloid Dendritic Cell || id:ebi-a-GCST90001903 | rs111862613 | 12 | 130309670 | T | 0.090699 | 0.0196746 | 3.74E-06 |
| ebi-a-GCST90017108 | Gut microbiota abundance (order Verrucomicrobiales id.4030) || id:ebi-a-GCST90017108 | CD33 on CD14+ monocyte || id:ebi-a-GCST90001946 | rs111862613 | 12 | 130309670 | T | 0.090699 | 0.0196746 | 3.74E-06 |
| ebi-a-GCST90017108 | Gut microbiota abundance (order Verrucomicrobiales id.4030) || id:ebi-a-GCST90017108 | CD33 on CD33+ HLA DR+ CD14dim || id:ebi-a-GCST90001947 | rs111862613 | 12 | 130309670 | T | 0.090699 | 0.0196746 | 3.74E-06 |
| ebi-a-GCST90017108 | Gut microbiota abundance (order Verrucomicrobiales id.4030) || id:ebi-a-GCST90017108 | CD33 on CD33dim HLA DR+ CD11b+ || id:ebi-a-GCST90001948 | rs111862613 | 12 | 130309670 | T | 0.090699 | 0.0196746 | 3.74E-06 |
| ebi-a-GCST90017108 | Gut microbiota abundance (order Verrucomicrobiales id.4030) || id:ebi-a-GCST90017108 | CD33 on Granulocytic Myeloid-Derived Suppressor Cells || id:ebi-a-GCST90001950 | rs111862613 | 12 | 130309670 | T | 0.090699 | 0.0196746 | 3.74E-06 |
| ebi-a-GCST90017108 | Gut microbiota abundance (order Verrucomicrobiales id.4030) || id:ebi-a-GCST90017108 | CD33 on CD66b++ myeloid cell || id:ebi-a-GCST90001951 | rs111862613 | 12 | 130309670 | T | 0.090699 | 0.0196746 | 3.74E-06 |
| ebi-a-GCST90017108 | Gut microbiota abundance (order Verrucomicrobiales id.4030) || id:ebi-a-GCST90017108 | CD33 on CD33dim HLA DR- || id:ebi-a-GCST90001953 | rs111862613 | 12 | 130309670 | T | 0.090699 | 0.0196746 | 3.74E-06 |
| ebi-a-GCST90017108 | Gut microbiota abundance (order Verrucomicrobiales id.4030) || id:ebi-a-GCST90017108 | CD33 on basophil || id:ebi-a-GCST90001954 | rs111862613 | 12 | 130309670 | T | 0.090699 | 0.0196746 | 3.74E-06 |
| ebi-a-GCST90017108 | Gut microbiota abundance (order Verrucomicrobiales id.4030) || id:ebi-a-GCST90017108 | CD33 on Immature Myeloid-Derived Suppressor Cells || id:ebi-a-GCST90001955 | rs111862613 | 12 | 130309670 | T | 0.090699 | 0.0196746 | 3.74E-06 |
| ebi-a-GCST90017108 | Gut microbiota abundance (order Verrucomicrobiales id.4030) || id:ebi-a-GCST90017108 | FSC-A on HLA DR+ CD8+ T cell || id:ebi-a-GCST90001978 | rs111862613 | 12 | 130309670 | T | 0.090699 | 0.0196746 | 3.74E-06 |
| ebi-a-GCST90017108 | Gut microbiota abundance (order Verrucomicrobiales id.4030) || id:ebi-a-GCST90017108 | HLA DR on CD14+ CD16- monocyte || id:ebi-a-GCST90001988 | rs111862613 | 12 | 130309670 | T | 0.090699 | 0.0196746 | 3.74E-06 |
| ebi-a-GCST90017108 | Gut microbiota abundance (order Verrucomicrobiales id.4030) || id:ebi-a-GCST90017108 | HLA DR on CD14+ monocyte || id:ebi-a-GCST90001991 | rs111862613 | 12 | 130309670 | T | 0.090699 | 0.0196746 | 3.74E-06 |
| ebi-a-GCST90017108 | Gut microbiota abundance (order Verrucomicrobiales id.4030) || id:ebi-a-GCST90017108 | CD16 on CD14+ CD16+ monocyte || id:ebi-a-GCST90002005 | rs111862613 | 12 | 130309670 | T | 0.090699 | 0.0196746 | 3.74E-06 |
| ebi-a-GCST90017108 | Gut microbiota abundance (order Verrucomicrobiales id.4030) || id:ebi-a-GCST90017108 | CD45 on CD33+ HLA DR+ CD14- || id:ebi-a-GCST90002042 | rs111862613 | 12 | 130309670 | T | 0.090699 | 0.0196746 | 3.74E-06 |
| ebi-a-GCST90017108 | Gut microbiota abundance (order Verrucomicrobiales id.4030) || id:ebi-a-GCST90017108 | CD8 on Natural Killer T || id:ebi-a-GCST90002059 | rs111862613 | 12 | 130309670 | T | 0.090699 | 0.0196746 | 3.74E-06 |
| ebi-a-GCST90016961 | Gut microbiota abundance (genus Akkermansia id.4037) || id:ebi-a-GCST90016961 | Myeloid Dendritic Cell Absolute Count || id:ebi-a-GCST90001458 | rs111862613 | 12 | 130309670 | T | 0.0911199 | 0.0196748 | 3.39E-06 |
| ebi-a-GCST90016961 | Gut microbiota abundance (genus Akkermansia id.4037) || id:ebi-a-GCST90016961 | CD86+ myeloid Dendritic Cell %Dendritic Cell || id:ebi-a-GCST90001465 | rs111862613 | 12 | 130309670 | T | 0.0911199 | 0.0196748 | 3.39E-06 |
| ebi-a-GCST90016961 | Gut microbiota abundance (genus Akkermansia id.4037) || id:ebi-a-GCST90016961 | CD62L- myeloid Dendritic Cell Absolute Count || id:ebi-a-GCST90001468 | rs111862613 | 12 | 130309670 | T | 0.0911199 | 0.0196748 | 3.39E-06 |
| ebi-a-GCST90016961 | Gut microbiota abundance (genus Akkermansia id.4037) || id:ebi-a-GCST90016961 | CD62L- myeloid Dendritic Cell %Dendritic Cell || id:ebi-a-GCST90001469 | rs111862613 | 12 | 130309670 | T | 0.0911199 | 0.0196748 | 3.39E-06 |
| ebi-a-GCST90016961 | Gut microbiota abundance (genus Akkermansia id.4037) || id:ebi-a-GCST90016961 | CD62L- CD86+ myeloid Dendritic Cell Absolute Count || id:ebi-a-GCST90001472 | rs111862613 | 12 | 130309670 | T | 0.0911199 | 0.0196748 | 3.39E-06 |
| ebi-a-GCST90016961 | Gut microbiota abundance (genus Akkermansia id.4037) || id:ebi-a-GCST90016961 | CD62L- CD86+ myeloid Dendritic Cell %Dendritic Cell || id:ebi-a-GCST90001473 | rs111862613 | 12 | 130309670 | T | 0.0911199 | 0.0196748 | 3.39E-06 |
| ebi-a-GCST90016961 | Gut microbiota abundance (genus Akkermansia id.4037) || id:ebi-a-GCST90016961 | HLA DR++ monocyte %monocyte || id:ebi-a-GCST90001475 | rs111862613 | 12 | 130309670 | T | 0.0911199 | 0.0196748 | 3.39E-06 |
| ebi-a-GCST90016961 | Gut microbiota abundance (genus Akkermansia id.4037) || id:ebi-a-GCST90016961 | HLA DR++ monocyte Absolute Count || id:ebi-a-GCST90001477 | rs111862613 | 12 | 130309670 | T | 0.0911199 | 0.0196748 | 3.39E-06 |
| ebi-a-GCST90016961 | Gut microbiota abundance (genus Akkermansia id.4037) || id:ebi-a-GCST90016961 | Basophil %CD33dim HLA DR- CD66b- || id:ebi-a-GCST90001533 | rs111862613 | 12 | 130309670 | T | 0.0911199 | 0.0196748 | 3.39E-06 |
| ebi-a-GCST90016961 | Gut microbiota abundance (genus Akkermansia id.4037) || id:ebi-a-GCST90016961 | CD16+ monocyte %monocyte || id:ebi-a-GCST90001587 | rs111862613 | 12 | 130309670 | T | 0.0911199 | 0.0196748 | 3.39E-06 |
| ebi-a-GCST90016961 | Gut microbiota abundance (genus Akkermansia id.4037) || id:ebi-a-GCST90016961 | T/B cell || id:ebi-a-GCST90001588 | rs111862613 | 12 | 130309670 | T | 0.0911199 | 0.0196748 | 3.39E-06 |
| ebi-a-GCST90016961 | Gut microbiota abundance (genus Akkermansia id.4037) || id:ebi-a-GCST90016961 | CD8+ Natural Killer T %T cell || id:ebi-a-GCST90001631 | rs111862613 | 12 | 130309670 | T | 0.0911199 | 0.0196748 | 3.39E-06 |
| ebi-a-GCST90016961 | Gut microbiota abundance (genus Akkermansia id.4037) || id:ebi-a-GCST90016961 | CD4-CD8- Natural Killer T %lymphocyte || id:ebi-a-GCST90001638 | rs111862613 | 12 | 130309670 | T | 0.0911199 | 0.0196748 | 3.39E-06 |
| ebi-a-GCST90016961 | Gut microbiota abundance (genus Akkermansia id.4037) || id:ebi-a-GCST90016961 | HLA DR+ Natural Killer Absolute Count || id:ebi-a-GCST90001648 | rs111862613 | 12 | 130309670 | T | 0.0911199 | 0.0196748 | 3.39E-06 |
| ebi-a-GCST90016961 | Gut microbiota abundance (genus Akkermansia id.4037) || id:ebi-a-GCST90016961 | HLA DR+ Natural Killer %Natural Killer || id:ebi-a-GCST90001649 | rs111862613 | 12 | 130309670 | T | 0.0911199 | 0.0196748 | 3.39E-06 |
| ebi-a-GCST90016961 | Gut microbiota abundance (genus Akkermansia id.4037) || id:ebi-a-GCST90016961 | HLA DR+ Natural Killer %CD3- lymphocyte || id:ebi-a-GCST90001650 | rs111862613 | 12 | 130309670 | T | 0.0911199 | 0.0196748 | 3.39E-06 |
| ebi-a-GCST90016961 | Gut microbiota abundance (genus Akkermansia id.4037) || id:ebi-a-GCST90016961 | CD28- CD25++ CD8+ T cell Absolute Count || id:ebi-a-GCST90001678 | rs111862613 | 12 | 130309670 | T | 0.0911199 | 0.0196748 | 3.39E-06 |
| ebi-a-GCST90016961 | Gut microbiota abundance (genus Akkermansia id.4037) || id:ebi-a-GCST90016961 | CD25++ CD8+ T cell %T cell || id:ebi-a-GCST90001679 | rs111862613 | 12 | 130309670 | T | 0.0911199 | 0.0196748 | 3.39E-06 |
| ebi-a-GCST90016961 | Gut microbiota abundance (genus Akkermansia id.4037) || id:ebi-a-GCST90016961 | CD19 on CD20- CD38- B cell || id:ebi-a-GCST90001722 | rs111862613 | 12 | 130309670 | T | 0.0911199 | 0.0196748 | 3.39E-06 |
| ebi-a-GCST90016961 | Gut microbiota abundance (genus Akkermansia id.4037) || id:ebi-a-GCST90016961 | CD25 on B cell || id:ebi-a-GCST90001775 | rs111862613 | 12 | 130309670 | T | 0.0911199 | 0.0196748 | 3.39E-06 |
| ebi-a-GCST90016961 | Gut microbiota abundance (genus Akkermansia id.4037) || id:ebi-a-GCST90016961 | CD25 on IgD+ CD24- B cell || id:ebi-a-GCST90001779 | rs111862613 | 12 | 130309670 | T | 0.0911199 | 0.0196748 | 3.39E-06 |
| ebi-a-GCST90016961 | Gut microbiota abundance (genus Akkermansia id.4037) || id:ebi-a-GCST90016961 | CD25 on IgD+ CD38- naive B cell || id:ebi-a-GCST90001781 | rs111862613 | 12 | 130309670 | T | 0.0911199 | 0.0196748 | 3.39E-06 |
| ebi-a-GCST90016961 | Gut microbiota abundance (genus Akkermansia id.4037) || id:ebi-a-GCST90016961 | CD25 on IgD+ CD38+ B cell || id:ebi-a-GCST90001783 | rs111862613 | 12 | 130309670 | T | 0.0911199 | 0.0196748 | 3.39E-06 |
| ebi-a-GCST90016961 | Gut microbiota abundance (genus Akkermansia id.4037) || id:ebi-a-GCST90016961 | CD86 on myeloid Dendritic Cell || id:ebi-a-GCST90001903 | rs111862613 | 12 | 130309670 | T | 0.0911199 | 0.0196748 | 3.39E-06 |
| ebi-a-GCST90016961 | Gut microbiota abundance (genus Akkermansia id.4037) || id:ebi-a-GCST90016961 | CD33 on CD14+ monocyte || id:ebi-a-GCST90001946 | rs111862613 | 12 | 130309670 | T | 0.0911199 | 0.0196748 | 3.39E-06 |
| ebi-a-GCST90016961 | Gut microbiota abundance (genus Akkermansia id.4037) || id:ebi-a-GCST90016961 | CD33 on CD33+ HLA DR+ CD14dim || id:ebi-a-GCST90001947 | rs111862613 | 12 | 130309670 | T | 0.0911199 | 0.0196748 | 3.39E-06 |
| ebi-a-GCST90016961 | Gut microbiota abundance (genus Akkermansia id.4037) || id:ebi-a-GCST90016961 | CD33 on CD33dim HLA DR+ CD11b+ || id:ebi-a-GCST90001948 | rs111862613 | 12 | 130309670 | T | 0.0911199 | 0.0196748 | 3.39E-06 |
| ebi-a-GCST90016961 | Gut microbiota abundance (genus Akkermansia id.4037) || id:ebi-a-GCST90016961 | CD33 on Granulocytic Myeloid-Derived Suppressor Cells || id:ebi-a-GCST90001950 | rs111862613 | 12 | 130309670 | T | 0.0911199 | 0.0196748 | 3.39E-06 |
| ebi-a-GCST90016961 | Gut microbiota abundance (genus Akkermansia id.4037) || id:ebi-a-GCST90016961 | CD33 on CD66b++ myeloid cell || id:ebi-a-GCST90001951 | rs111862613 | 12 | 130309670 | T | 0.0911199 | 0.0196748 | 3.39E-06 |
| ebi-a-GCST90016961 | Gut microbiota abundance (genus Akkermansia id.4037) || id:ebi-a-GCST90016961 | CD33 on CD33dim HLA DR- || id:ebi-a-GCST90001953 | rs111862613 | 12 | 130309670 | T | 0.0911199 | 0.0196748 | 3.39E-06 |
| ebi-a-GCST90016961 | Gut microbiota abundance (genus Akkermansia id.4037) || id:ebi-a-GCST90016961 | CD33 on basophil || id:ebi-a-GCST90001954 | rs111862613 | 12 | 130309670 | T | 0.0911199 | 0.0196748 | 3.39E-06 |
| ebi-a-GCST90016961 | Gut microbiota abundance (genus Akkermansia id.4037) || id:ebi-a-GCST90016961 | CD33 on Immature Myeloid-Derived Suppressor Cells || id:ebi-a-GCST90001955 | rs111862613 | 12 | 130309670 | T | 0.0911199 | 0.0196748 | 3.39E-06 |
| ebi-a-GCST90016961 | Gut microbiota abundance (genus Akkermansia id.4037) || id:ebi-a-GCST90016961 | FSC-A on HLA DR+ CD8+ T cell || id:ebi-a-GCST90001978 | rs111862613 | 12 | 130309670 | T | 0.0911199 | 0.0196748 | 3.39E-06 |
| ebi-a-GCST90016961 | Gut microbiota abundance (genus Akkermansia id.4037) || id:ebi-a-GCST90016961 | HLA DR on CD14+ CD16- monocyte || id:ebi-a-GCST90001988 | rs111862613 | 12 | 130309670 | T | 0.0911199 | 0.0196748 | 3.39E-06 |
| ebi-a-GCST90016961 | Gut microbiota abundance (genus Akkermansia id.4037) || id:ebi-a-GCST90016961 | HLA DR on CD14+ monocyte || id:ebi-a-GCST90001991 | rs111862613 | 12 | 130309670 | T | 0.0911199 | 0.0196748 | 3.39E-06 |
| ebi-a-GCST90016961 | Gut microbiota abundance (genus Akkermansia id.4037) || id:ebi-a-GCST90016961 | CD16 on CD14+ CD16+ monocyte || id:ebi-a-GCST90002005 | rs111862613 | 12 | 130309670 | T | 0.0911199 | 0.0196748 | 3.39E-06 |
| ebi-a-GCST90016961 | Gut microbiota abundance (genus Akkermansia id.4037) || id:ebi-a-GCST90016961 | CD45 on CD33+ HLA DR+ CD14- || id:ebi-a-GCST90002042 | rs111862613 | 12 | 130309670 | T | 0.0911199 | 0.0196748 | 3.39E-06 |
| ebi-a-GCST90016961 | Gut microbiota abundance (genus Akkermansia id.4037) || id:ebi-a-GCST90016961 | CD8 on Natural Killer T || id:ebi-a-GCST90002059 | rs111862613 | 12 | 130309670 | T | 0.0911199 | 0.0196748 | 3.39E-06 |
| ebi-a-GCST90016943 | Gut microbiota abundance (family Oxalobacteraceae id.2966) || id:ebi-a-GCST90016943 | Myeloid Dendritic Cell Absolute Count || id:ebi-a-GCST90001458 | rs111966731 | 15 | 93941937 | T | 0.204031 | 0.0445746 | 4.56E-06 |
| ebi-a-GCST90016943 | Gut microbiota abundance (family Oxalobacteraceae id.2966) || id:ebi-a-GCST90016943 | CD86+ myeloid Dendritic Cell %Dendritic Cell || id:ebi-a-GCST90001465 | rs111966731 | 15 | 93941937 | T | 0.204031 | 0.0445746 | 4.56E-06 |
| ebi-a-GCST90016943 | Gut microbiota abundance (family Oxalobacteraceae id.2966) || id:ebi-a-GCST90016943 | CD62L- myeloid Dendritic Cell Absolute Count || id:ebi-a-GCST90001468 | rs111966731 | 15 | 93941937 | T | 0.204031 | 0.0445746 | 4.56E-06 |
| ebi-a-GCST90016943 | Gut microbiota abundance (family Oxalobacteraceae id.2966) || id:ebi-a-GCST90016943 | CD62L- myeloid Dendritic Cell %Dendritic Cell || id:ebi-a-GCST90001469 | rs111966731 | 15 | 93941937 | T | 0.204031 | 0.0445746 | 4.56E-06 |
| ebi-a-GCST90016943 | Gut microbiota abundance (family Oxalobacteraceae id.2966) || id:ebi-a-GCST90016943 | CD62L- CD86+ myeloid Dendritic Cell Absolute Count || id:ebi-a-GCST90001472 | rs111966731 | 15 | 93941937 | T | 0.204031 | 0.0445746 | 4.56E-06 |
| ebi-a-GCST90016943 | Gut microbiota abundance (family Oxalobacteraceae id.2966) || id:ebi-a-GCST90016943 | CD62L- CD86+ myeloid Dendritic Cell %Dendritic Cell || id:ebi-a-GCST90001473 | rs111966731 | 15 | 93941937 | T | 0.204031 | 0.0445746 | 4.56E-06 |
| ebi-a-GCST90016943 | Gut microbiota abundance (family Oxalobacteraceae id.2966) || id:ebi-a-GCST90016943 | HLA DR++ monocyte %monocyte || id:ebi-a-GCST90001475 | rs111966731 | 15 | 93941937 | T | 0.204031 | 0.0445746 | 4.56E-06 |
| ebi-a-GCST90016943 | Gut microbiota abundance (family Oxalobacteraceae id.2966) || id:ebi-a-GCST90016943 | HLA DR++ monocyte Absolute Count || id:ebi-a-GCST90001477 | rs111966731 | 15 | 93941937 | T | 0.204031 | 0.0445746 | 4.56E-06 |
| ebi-a-GCST90016943 | Gut microbiota abundance (family Oxalobacteraceae id.2966) || id:ebi-a-GCST90016943 | Basophil %CD33dim HLA DR- CD66b- || id:ebi-a-GCST90001533 | rs111966731 | 15 | 93941937 | T | 0.204031 | 0.0445746 | 4.56E-06 |
| ebi-a-GCST90016943 | Gut microbiota abundance (family Oxalobacteraceae id.2966) || id:ebi-a-GCST90016943 | CD16+ monocyte %monocyte || id:ebi-a-GCST90001587 | rs111966731 | 15 | 93941937 | T | 0.204031 | 0.0445746 | 4.56E-06 |
| ebi-a-GCST90016943 | Gut microbiota abundance (family Oxalobacteraceae id.2966) || id:ebi-a-GCST90016943 | T/B cell || id:ebi-a-GCST90001588 | rs111966731 | 15 | 93941937 | T | 0.204031 | 0.0445746 | 4.56E-06 |
| ebi-a-GCST90016943 | Gut microbiota abundance (family Oxalobacteraceae id.2966) || id:ebi-a-GCST90016943 | CD8+ Natural Killer T %T cell || id:ebi-a-GCST90001631 | rs111966731 | 15 | 93941937 | T | 0.204031 | 0.0445746 | 4.56E-06 |
| ebi-a-GCST90016943 | Gut microbiota abundance (family Oxalobacteraceae id.2966) || id:ebi-a-GCST90016943 | CD4-CD8- Natural Killer T %lymphocyte || id:ebi-a-GCST90001638 | rs111966731 | 15 | 93941937 | T | 0.204031 | 0.0445746 | 4.56E-06 |
| ebi-a-GCST90016943 | Gut microbiota abundance (family Oxalobacteraceae id.2966) || id:ebi-a-GCST90016943 | HLA DR+ Natural Killer Absolute Count || id:ebi-a-GCST90001648 | rs111966731 | 15 | 93941937 | T | 0.204031 | 0.0445746 | 4.56E-06 |
| ebi-a-GCST90016943 | Gut microbiota abundance (family Oxalobacteraceae id.2966) || id:ebi-a-GCST90016943 | HLA DR+ Natural Killer %Natural Killer || id:ebi-a-GCST90001649 | rs111966731 | 15 | 93941937 | T | 0.204031 | 0.0445746 | 4.56E-06 |
| ebi-a-GCST90016943 | Gut microbiota abundance (family Oxalobacteraceae id.2966) || id:ebi-a-GCST90016943 | HLA DR+ Natural Killer %CD3- lymphocyte || id:ebi-a-GCST90001650 | rs111966731 | 15 | 93941937 | T | 0.204031 | 0.0445746 | 4.56E-06 |
| ebi-a-GCST90016943 | Gut microbiota abundance (family Oxalobacteraceae id.2966) || id:ebi-a-GCST90016943 | CD28- CD25++ CD8+ T cell Absolute Count || id:ebi-a-GCST90001678 | rs111966731 | 15 | 93941937 | T | 0.204031 | 0.0445746 | 4.56E-06 |
| ebi-a-GCST90016943 | Gut microbiota abundance (family Oxalobacteraceae id.2966) || id:ebi-a-GCST90016943 | CD25++ CD8+ T cell %T cell || id:ebi-a-GCST90001679 | rs111966731 | 15 | 93941937 | T | 0.204031 | 0.0445746 | 4.56E-06 |
| ebi-a-GCST90016943 | Gut microbiota abundance (family Oxalobacteraceae id.2966) || id:ebi-a-GCST90016943 | CD19 on CD20- CD38- B cell || id:ebi-a-GCST90001722 | rs111966731 | 15 | 93941937 | T | 0.204031 | 0.0445746 | 4.56E-06 |
| ebi-a-GCST90016943 | Gut microbiota abundance (family Oxalobacteraceae id.2966) || id:ebi-a-GCST90016943 | CD25 on B cell || id:ebi-a-GCST90001775 | rs111966731 | 15 | 93941937 | T | 0.204031 | 0.0445746 | 4.56E-06 |
| ebi-a-GCST90016943 | Gut microbiota abundance (family Oxalobacteraceae id.2966) || id:ebi-a-GCST90016943 | CD25 on IgD+ CD24- B cell || id:ebi-a-GCST90001779 | rs111966731 | 15 | 93941937 | T | 0.204031 | 0.0445746 | 4.56E-06 |
| ebi-a-GCST90016943 | Gut microbiota abundance (family Oxalobacteraceae id.2966) || id:ebi-a-GCST90016943 | CD25 on IgD+ CD38- naive B cell || id:ebi-a-GCST90001781 | rs111966731 | 15 | 93941937 | T | 0.204031 | 0.0445746 | 4.56E-06 |
| ebi-a-GCST90016943 | Gut microbiota abundance (family Oxalobacteraceae id.2966) || id:ebi-a-GCST90016943 | CD25 on IgD+ CD38+ B cell || id:ebi-a-GCST90001783 | rs111966731 | 15 | 93941937 | T | 0.204031 | 0.0445746 | 4.56E-06 |
| ebi-a-GCST90016943 | Gut microbiota abundance (family Oxalobacteraceae id.2966) || id:ebi-a-GCST90016943 | CD86 on myeloid Dendritic Cell || id:ebi-a-GCST90001903 | rs111966731 | 15 | 93941937 | T | 0.204031 | 0.0445746 | 4.56E-06 |
| ebi-a-GCST90016943 | Gut microbiota abundance (family Oxalobacteraceae id.2966) || id:ebi-a-GCST90016943 | CD33 on CD14+ monocyte || id:ebi-a-GCST90001946 | rs111966731 | 15 | 93941937 | T | 0.204031 | 0.0445746 | 4.56E-06 |
| ebi-a-GCST90016943 | Gut microbiota abundance (family Oxalobacteraceae id.2966) || id:ebi-a-GCST90016943 | CD33 on CD33+ HLA DR+ CD14dim || id:ebi-a-GCST90001947 | rs111966731 | 15 | 93941937 | T | 0.204031 | 0.0445746 | 4.56E-06 |
| ebi-a-GCST90016943 | Gut microbiota abundance (family Oxalobacteraceae id.2966) || id:ebi-a-GCST90016943 | CD33 on CD33dim HLA DR+ CD11b+ || id:ebi-a-GCST90001948 | rs111966731 | 15 | 93941937 | T | 0.204031 | 0.0445746 | 4.56E-06 |
| ebi-a-GCST90016943 | Gut microbiota abundance (family Oxalobacteraceae id.2966) || id:ebi-a-GCST90016943 | CD33 on Granulocytic Myeloid-Derived Suppressor Cells || id:ebi-a-GCST90001950 | rs111966731 | 15 | 93941937 | T | 0.204031 | 0.0445746 | 4.56E-06 |
| ebi-a-GCST90016943 | Gut microbiota abundance (family Oxalobacteraceae id.2966) || id:ebi-a-GCST90016943 | CD33 on CD66b++ myeloid cell || id:ebi-a-GCST90001951 | rs111966731 | 15 | 93941937 | T | 0.204031 | 0.0445746 | 4.56E-06 |
| ebi-a-GCST90016943 | Gut microbiota abundance (family Oxalobacteraceae id.2966) || id:ebi-a-GCST90016943 | CD33 on CD33dim HLA DR- || id:ebi-a-GCST90001953 | rs111966731 | 15 | 93941937 | T | 0.204031 | 0.0445746 | 4.56E-06 |
| ebi-a-GCST90016943 | Gut microbiota abundance (family Oxalobacteraceae id.2966) || id:ebi-a-GCST90016943 | CD33 on basophil || id:ebi-a-GCST90001954 | rs111966731 | 15 | 93941937 | T | 0.204031 | 0.0445746 | 4.56E-06 |
| ebi-a-GCST90016943 | Gut microbiota abundance (family Oxalobacteraceae id.2966) || id:ebi-a-GCST90016943 | CD33 on Immature Myeloid-Derived Suppressor Cells || id:ebi-a-GCST90001955 | rs111966731 | 15 | 93941937 | T | 0.204031 | 0.0445746 | 4.56E-06 |
| ebi-a-GCST90016943 | Gut microbiota abundance (family Oxalobacteraceae id.2966) || id:ebi-a-GCST90016943 | FSC-A on HLA DR+ CD8+ T cell || id:ebi-a-GCST90001978 | rs111966731 | 15 | 93941937 | T | 0.204031 | 0.0445746 | 4.56E-06 |
| ebi-a-GCST90016943 | Gut microbiota abundance (family Oxalobacteraceae id.2966) || id:ebi-a-GCST90016943 | HLA DR on CD14+ CD16- monocyte || id:ebi-a-GCST90001988 | rs111966731 | 15 | 93941937 | T | 0.204031 | 0.0445746 | 4.56E-06 |
| ebi-a-GCST90016943 | Gut microbiota abundance (family Oxalobacteraceae id.2966) || id:ebi-a-GCST90016943 | HLA DR on CD14+ monocyte || id:ebi-a-GCST90001991 | rs111966731 | 15 | 93941937 | T | 0.204031 | 0.0445746 | 4.56E-06 |
| ebi-a-GCST90016943 | Gut microbiota abundance (family Oxalobacteraceae id.2966) || id:ebi-a-GCST90016943 | CD16 on CD14+ CD16+ monocyte || id:ebi-a-GCST90002005 | rs111966731 | 15 | 93941937 | T | 0.204031 | 0.0445746 | 4.56E-06 |
| ebi-a-GCST90016943 | Gut microbiota abundance (family Oxalobacteraceae id.2966) || id:ebi-a-GCST90016943 | CD45 on CD33+ HLA DR+ CD14- || id:ebi-a-GCST90002042 | rs111966731 | 15 | 93941937 | T | 0.204031 | 0.0445746 | 4.56E-06 |
| ebi-a-GCST90016943 | Gut microbiota abundance (family Oxalobacteraceae id.2966) || id:ebi-a-GCST90016943 | CD8 on Natural Killer T || id:ebi-a-GCST90002059 | rs111966731 | 15 | 93941937 | T | 0.204031 | 0.0445746 | 4.56E-06 |
| ebi-a-GCST90016943 | Gut microbiota abundance (family Oxalobacteraceae id.2966) || id:ebi-a-GCST90016943 | Myeloid Dendritic Cell Absolute Count || id:ebi-a-GCST90001458 | rs11246212 | 11 | 610277 | C | -0.13607 | 0.0291797 | 4.51E-06 |
| ebi-a-GCST90016943 | Gut microbiota abundance (family Oxalobacteraceae id.2966) || id:ebi-a-GCST90016943 | CD86+ myeloid Dendritic Cell %Dendritic Cell || id:ebi-a-GCST90001465 | rs11246212 | 11 | 610277 | C | -0.13607 | 0.0291797 | 4.51E-06 |
| ebi-a-GCST90016943 | Gut microbiota abundance (family Oxalobacteraceae id.2966) || id:ebi-a-GCST90016943 | CD62L- myeloid Dendritic Cell Absolute Count || id:ebi-a-GCST90001468 | rs11246212 | 11 | 610277 | C | -0.13607 | 0.0291797 | 4.51E-06 |
| ebi-a-GCST90016943 | Gut microbiota abundance (family Oxalobacteraceae id.2966) || id:ebi-a-GCST90016943 | CD62L- myeloid Dendritic Cell %Dendritic Cell || id:ebi-a-GCST90001469 | rs11246212 | 11 | 610277 | C | -0.13607 | 0.0291797 | 4.51E-06 |
| ebi-a-GCST90016943 | Gut microbiota abundance (family Oxalobacteraceae id.2966) || id:ebi-a-GCST90016943 | CD62L- CD86+ myeloid Dendritic Cell Absolute Count || id:ebi-a-GCST90001472 | rs11246212 | 11 | 610277 | C | -0.13607 | 0.0291797 | 4.51E-06 |
| ebi-a-GCST90016943 | Gut microbiota abundance (family Oxalobacteraceae id.2966) || id:ebi-a-GCST90016943 | CD62L- CD86+ myeloid Dendritic Cell %Dendritic Cell || id:ebi-a-GCST90001473 | rs11246212 | 11 | 610277 | C | -0.13607 | 0.0291797 | 4.51E-06 |
| ebi-a-GCST90016943 | Gut microbiota abundance (family Oxalobacteraceae id.2966) || id:ebi-a-GCST90016943 | HLA DR++ monocyte %monocyte || id:ebi-a-GCST90001475 | rs11246212 | 11 | 610277 | C | -0.13607 | 0.0291797 | 4.51E-06 |
| ebi-a-GCST90016943 | Gut microbiota abundance (family Oxalobacteraceae id.2966) || id:ebi-a-GCST90016943 | HLA DR++ monocyte Absolute Count || id:ebi-a-GCST90001477 | rs11246212 | 11 | 610277 | C | -0.13607 | 0.0291797 | 4.51E-06 |
| ebi-a-GCST90016943 | Gut microbiota abundance (family Oxalobacteraceae id.2966) || id:ebi-a-GCST90016943 | Basophil %CD33dim HLA DR- CD66b- || id:ebi-a-GCST90001533 | rs11246212 | 11 | 610277 | C | -0.13607 | 0.0291797 | 4.51E-06 |
| ebi-a-GCST90016943 | Gut microbiota abundance (family Oxalobacteraceae id.2966) || id:ebi-a-GCST90016943 | CD16+ monocyte %monocyte || id:ebi-a-GCST90001587 | rs11246212 | 11 | 610277 | C | -0.13607 | 0.0291797 | 4.51E-06 |
| ebi-a-GCST90016943 | Gut microbiota abundance (family Oxalobacteraceae id.2966) || id:ebi-a-GCST90016943 | T/B cell || id:ebi-a-GCST90001588 | rs11246212 | 11 | 610277 | C | -0.13607 | 0.0291797 | 4.51E-06 |
| ebi-a-GCST90016943 | Gut microbiota abundance (family Oxalobacteraceae id.2966) || id:ebi-a-GCST90016943 | CD8+ Natural Killer T %T cell || id:ebi-a-GCST90001631 | rs11246212 | 11 | 610277 | C | -0.13607 | 0.0291797 | 4.51E-06 |
| ebi-a-GCST90016943 | Gut microbiota abundance (family Oxalobacteraceae id.2966) || id:ebi-a-GCST90016943 | CD4-CD8- Natural Killer T %lymphocyte || id:ebi-a-GCST90001638 | rs11246212 | 11 | 610277 | C | -0.13607 | 0.0291797 | 4.51E-06 |
| ebi-a-GCST90016943 | Gut microbiota abundance (family Oxalobacteraceae id.2966) || id:ebi-a-GCST90016943 | HLA DR+ Natural Killer Absolute Count || id:ebi-a-GCST90001648 | rs11246212 | 11 | 610277 | C | -0.13607 | 0.0291797 | 4.51E-06 |
| ebi-a-GCST90016943 | Gut microbiota abundance (family Oxalobacteraceae id.2966) || id:ebi-a-GCST90016943 | HLA DR+ Natural Killer %Natural Killer || id:ebi-a-GCST90001649 | rs11246212 | 11 | 610277 | C | -0.13607 | 0.0291797 | 4.51E-06 |
| ebi-a-GCST90016943 | Gut microbiota abundance (family Oxalobacteraceae id.2966) || id:ebi-a-GCST90016943 | HLA DR+ Natural Killer %CD3- lymphocyte || id:ebi-a-GCST90001650 | rs11246212 | 11 | 610277 | C | -0.13607 | 0.0291797 | 4.51E-06 |
| ebi-a-GCST90016943 | Gut microbiota abundance (family Oxalobacteraceae id.2966) || id:ebi-a-GCST90016943 | CD28- CD25++ CD8+ T cell Absolute Count || id:ebi-a-GCST90001678 | rs11246212 | 11 | 610277 | C | -0.13607 | 0.0291797 | 4.51E-06 |
| ebi-a-GCST90016943 | Gut microbiota abundance (family Oxalobacteraceae id.2966) || id:ebi-a-GCST90016943 | CD25++ CD8+ T cell %T cell || id:ebi-a-GCST90001679 | rs11246212 | 11 | 610277 | C | -0.13607 | 0.0291797 | 4.51E-06 |
| ebi-a-GCST90016943 | Gut microbiota abundance (family Oxalobacteraceae id.2966) || id:ebi-a-GCST90016943 | CD19 on CD20- CD38- B cell || id:ebi-a-GCST90001722 | rs11246212 | 11 | 610277 | C | -0.13607 | 0.0291797 | 4.51E-06 |
| ebi-a-GCST90016943 | Gut microbiota abundance (family Oxalobacteraceae id.2966) || id:ebi-a-GCST90016943 | CD25 on B cell || id:ebi-a-GCST90001775 | rs11246212 | 11 | 610277 | C | -0.13607 | 0.0291797 | 4.51E-06 |
| ebi-a-GCST90016943 | Gut microbiota abundance (family Oxalobacteraceae id.2966) || id:ebi-a-GCST90016943 | CD25 on IgD+ CD24- B cell || id:ebi-a-GCST90001779 | rs11246212 | 11 | 610277 | C | -0.13607 | 0.0291797 | 4.51E-06 |
| ebi-a-GCST90016943 | Gut microbiota abundance (family Oxalobacteraceae id.2966) || id:ebi-a-GCST90016943 | CD25 on IgD+ CD38- naive B cell || id:ebi-a-GCST90001781 | rs11246212 | 11 | 610277 | C | -0.13607 | 0.0291797 | 4.51E-06 |
| ebi-a-GCST90016943 | Gut microbiota abundance (family Oxalobacteraceae id.2966) || id:ebi-a-GCST90016943 | CD25 on IgD+ CD38+ B cell || id:ebi-a-GCST90001783 | rs11246212 | 11 | 610277 | C | -0.13607 | 0.0291797 | 4.51E-06 |
| ebi-a-GCST90016943 | Gut microbiota abundance (family Oxalobacteraceae id.2966) || id:ebi-a-GCST90016943 | CD86 on myeloid Dendritic Cell || id:ebi-a-GCST90001903 | rs11246212 | 11 | 610277 | C | -0.13607 | 0.0291797 | 4.51E-06 |
| ebi-a-GCST90016943 | Gut microbiota abundance (family Oxalobacteraceae id.2966) || id:ebi-a-GCST90016943 | CD33 on CD14+ monocyte || id:ebi-a-GCST90001946 | rs11246212 | 11 | 610277 | C | -0.13607 | 0.0291797 | 4.51E-06 |
| ebi-a-GCST90016943 | Gut microbiota abundance (family Oxalobacteraceae id.2966) || id:ebi-a-GCST90016943 | CD33 on CD33+ HLA DR+ CD14dim || id:ebi-a-GCST90001947 | rs11246212 | 11 | 610277 | C | -0.13607 | 0.0291797 | 4.51E-06 |
| ebi-a-GCST90016943 | Gut microbiota abundance (family Oxalobacteraceae id.2966) || id:ebi-a-GCST90016943 | CD33 on CD33dim HLA DR+ CD11b+ || id:ebi-a-GCST90001948 | rs11246212 | 11 | 610277 | C | -0.13607 | 0.0291797 | 4.51E-06 |
| ebi-a-GCST90016943 | Gut microbiota abundance (family Oxalobacteraceae id.2966) || id:ebi-a-GCST90016943 | CD33 on Granulocytic Myeloid-Derived Suppressor Cells || id:ebi-a-GCST90001950 | rs11246212 | 11 | 610277 | C | -0.13607 | 0.0291797 | 4.51E-06 |
| ebi-a-GCST90016943 | Gut microbiota abundance (family Oxalobacteraceae id.2966) || id:ebi-a-GCST90016943 | CD33 on CD66b++ myeloid cell || id:ebi-a-GCST90001951 | rs11246212 | 11 | 610277 | C | -0.13607 | 0.0291797 | 4.51E-06 |
| ebi-a-GCST90016943 | Gut microbiota abundance (family Oxalobacteraceae id.2966) || id:ebi-a-GCST90016943 | CD33 on CD33dim HLA DR- || id:ebi-a-GCST90001953 | rs11246212 | 11 | 610277 | C | -0.13607 | 0.0291797 | 4.51E-06 |
| ebi-a-GCST90016943 | Gut microbiota abundance (family Oxalobacteraceae id.2966) || id:ebi-a-GCST90016943 | CD33 on basophil || id:ebi-a-GCST90001954 | rs11246212 | 11 | 610277 | C | -0.13607 | 0.0291797 | 4.51E-06 |
| ebi-a-GCST90016943 | Gut microbiota abundance (family Oxalobacteraceae id.2966) || id:ebi-a-GCST90016943 | CD33 on Immature Myeloid-Derived Suppressor Cells || id:ebi-a-GCST90001955 | rs11246212 | 11 | 610277 | C | -0.13607 | 0.0291797 | 4.51E-06 |
| ebi-a-GCST90016943 | Gut microbiota abundance (family Oxalobacteraceae id.2966) || id:ebi-a-GCST90016943 | FSC-A on HLA DR+ CD8+ T cell || id:ebi-a-GCST90001978 | rs11246212 | 11 | 610277 | C | -0.13607 | 0.0291797 | 4.51E-06 |
| ebi-a-GCST90016943 | Gut microbiota abundance (family Oxalobacteraceae id.2966) || id:ebi-a-GCST90016943 | HLA DR on CD14+ CD16- monocyte || id:ebi-a-GCST90001988 | rs11246212 | 11 | 610277 | C | -0.13607 | 0.0291797 | 4.51E-06 |
| ebi-a-GCST90016943 | Gut microbiota abundance (family Oxalobacteraceae id.2966) || id:ebi-a-GCST90016943 | HLA DR on CD14+ monocyte || id:ebi-a-GCST90001991 | rs11246212 | 11 | 610277 | C | -0.13607 | 0.0291797 | 4.51E-06 |
| ebi-a-GCST90016943 | Gut microbiota abundance (family Oxalobacteraceae id.2966) || id:ebi-a-GCST90016943 | CD16 on CD14+ CD16+ monocyte || id:ebi-a-GCST90002005 | rs11246212 | 11 | 610277 | C | -0.13607 | 0.0291797 | 4.51E-06 |
| ebi-a-GCST90016943 | Gut microbiota abundance (family Oxalobacteraceae id.2966) || id:ebi-a-GCST90016943 | CD45 on CD33+ HLA DR+ CD14- || id:ebi-a-GCST90002042 | rs11246212 | 11 | 610277 | C | -0.13607 | 0.0291797 | 4.51E-06 |
| ebi-a-GCST90016943 | Gut microbiota abundance (family Oxalobacteraceae id.2966) || id:ebi-a-GCST90016943 | CD8 on Natural Killer T || id:ebi-a-GCST90002059 | rs11246212 | 11 | 610277 | C | -0.13607 | 0.0291797 | 4.51E-06 |
| ebi-a-GCST90016957 | Gut microbiota abundance (family Verrucomicrobiaceae id.4036) || id:ebi-a-GCST90016957 | Myeloid Dendritic Cell Absolute Count || id:ebi-a-GCST90001458 | rs117107102 | 18 | 49473635 | A | 0.204683 | 0.0431572 | 2.92E-06 |
| ebi-a-GCST90016957 | Gut microbiota abundance (family Verrucomicrobiaceae id.4036) || id:ebi-a-GCST90016957 | CD86+ myeloid Dendritic Cell %Dendritic Cell || id:ebi-a-GCST90001465 | rs117107102 | 18 | 49473635 | A | 0.204683 | 0.0431572 | 2.92E-06 |
| ebi-a-GCST90016957 | Gut microbiota abundance (family Verrucomicrobiaceae id.4036) || id:ebi-a-GCST90016957 | CD62L- myeloid Dendritic Cell Absolute Count || id:ebi-a-GCST90001468 | rs117107102 | 18 | 49473635 | A | 0.204683 | 0.0431572 | 2.92E-06 |
| ebi-a-GCST90016957 | Gut microbiota abundance (family Verrucomicrobiaceae id.4036) || id:ebi-a-GCST90016957 | CD62L- myeloid Dendritic Cell %Dendritic Cell || id:ebi-a-GCST90001469 | rs117107102 | 18 | 49473635 | A | 0.204683 | 0.0431572 | 2.92E-06 |
| ebi-a-GCST90016957 | Gut microbiota abundance (family Verrucomicrobiaceae id.4036) || id:ebi-a-GCST90016957 | CD62L- CD86+ myeloid Dendritic Cell Absolute Count || id:ebi-a-GCST90001472 | rs117107102 | 18 | 49473635 | A | 0.204683 | 0.0431572 | 2.92E-06 |
| ebi-a-GCST90016957 | Gut microbiota abundance (family Verrucomicrobiaceae id.4036) || id:ebi-a-GCST90016957 | CD62L- CD86+ myeloid Dendritic Cell %Dendritic Cell || id:ebi-a-GCST90001473 | rs117107102 | 18 | 49473635 | A | 0.204683 | 0.0431572 | 2.92E-06 |
| ebi-a-GCST90016957 | Gut microbiota abundance (family Verrucomicrobiaceae id.4036) || id:ebi-a-GCST90016957 | HLA DR++ monocyte %monocyte || id:ebi-a-GCST90001475 | rs117107102 | 18 | 49473635 | A | 0.204683 | 0.0431572 | 2.92E-06 |
| ebi-a-GCST90016957 | Gut microbiota abundance (family Verrucomicrobiaceae id.4036) || id:ebi-a-GCST90016957 | HLA DR++ monocyte Absolute Count || id:ebi-a-GCST90001477 | rs117107102 | 18 | 49473635 | A | 0.204683 | 0.0431572 | 2.92E-06 |
| ebi-a-GCST90016957 | Gut microbiota abundance (family Verrucomicrobiaceae id.4036) || id:ebi-a-GCST90016957 | Basophil %CD33dim HLA DR- CD66b- || id:ebi-a-GCST90001533 | rs117107102 | 18 | 49473635 | A | 0.204683 | 0.0431572 | 2.92E-06 |
| ebi-a-GCST90016957 | Gut microbiota abundance (family Verrucomicrobiaceae id.4036) || id:ebi-a-GCST90016957 | CD16+ monocyte %monocyte || id:ebi-a-GCST90001587 | rs117107102 | 18 | 49473635 | A | 0.204683 | 0.0431572 | 2.92E-06 |
| ebi-a-GCST90016957 | Gut microbiota abundance (family Verrucomicrobiaceae id.4036) || id:ebi-a-GCST90016957 | T/B cell || id:ebi-a-GCST90001588 | rs117107102 | 18 | 49473635 | A | 0.204683 | 0.0431572 | 2.92E-06 |
| ebi-a-GCST90016957 | Gut microbiota abundance (family Verrucomicrobiaceae id.4036) || id:ebi-a-GCST90016957 | CD8+ Natural Killer T %T cell || id:ebi-a-GCST90001631 | rs117107102 | 18 | 49473635 | A | 0.204683 | 0.0431572 | 2.92E-06 |
| ebi-a-GCST90016957 | Gut microbiota abundance (family Verrucomicrobiaceae id.4036) || id:ebi-a-GCST90016957 | CD4-CD8- Natural Killer T %lymphocyte || id:ebi-a-GCST90001638 | rs117107102 | 18 | 49473635 | A | 0.204683 | 0.0431572 | 2.92E-06 |
| ebi-a-GCST90016957 | Gut microbiota abundance (family Verrucomicrobiaceae id.4036) || id:ebi-a-GCST90016957 | HLA DR+ Natural Killer Absolute Count || id:ebi-a-GCST90001648 | rs117107102 | 18 | 49473635 | A | 0.204683 | 0.0431572 | 2.92E-06 |
| ebi-a-GCST90016957 | Gut microbiota abundance (family Verrucomicrobiaceae id.4036) || id:ebi-a-GCST90016957 | HLA DR+ Natural Killer %Natural Killer || id:ebi-a-GCST90001649 | rs117107102 | 18 | 49473635 | A | 0.204683 | 0.0431572 | 2.92E-06 |
| ebi-a-GCST90016957 | Gut microbiota abundance (family Verrucomicrobiaceae id.4036) || id:ebi-a-GCST90016957 | HLA DR+ Natural Killer %CD3- lymphocyte || id:ebi-a-GCST90001650 | rs117107102 | 18 | 49473635 | A | 0.204683 | 0.0431572 | 2.92E-06 |
| ebi-a-GCST90016957 | Gut microbiota abundance (family Verrucomicrobiaceae id.4036) || id:ebi-a-GCST90016957 | CD28- CD25++ CD8+ T cell Absolute Count || id:ebi-a-GCST90001678 | rs117107102 | 18 | 49473635 | A | 0.204683 | 0.0431572 | 2.92E-06 |
| ebi-a-GCST90016957 | Gut microbiota abundance (family Verrucomicrobiaceae id.4036) || id:ebi-a-GCST90016957 | CD25++ CD8+ T cell %T cell || id:ebi-a-GCST90001679 | rs117107102 | 18 | 49473635 | A | 0.204683 | 0.0431572 | 2.92E-06 |
| ebi-a-GCST90016957 | Gut microbiota abundance (family Verrucomicrobiaceae id.4036) || id:ebi-a-GCST90016957 | CD19 on CD20- CD38- B cell || id:ebi-a-GCST90001722 | rs117107102 | 18 | 49473635 | A | 0.204683 | 0.0431572 | 2.92E-06 |
| ebi-a-GCST90016957 | Gut microbiota abundance (family Verrucomicrobiaceae id.4036) || id:ebi-a-GCST90016957 | CD25 on B cell || id:ebi-a-GCST90001775 | rs117107102 | 18 | 49473635 | A | 0.204683 | 0.0431572 | 2.92E-06 |
| ebi-a-GCST90016957 | Gut microbiota abundance (family Verrucomicrobiaceae id.4036) || id:ebi-a-GCST90016957 | CD25 on IgD+ CD24- B cell || id:ebi-a-GCST90001779 | rs117107102 | 18 | 49473635 | A | 0.204683 | 0.0431572 | 2.92E-06 |
| ebi-a-GCST90016957 | Gut microbiota abundance (family Verrucomicrobiaceae id.4036) || id:ebi-a-GCST90016957 | CD25 on IgD+ CD38- naive B cell || id:ebi-a-GCST90001781 | rs117107102 | 18 | 49473635 | A | 0.204683 | 0.0431572 | 2.92E-06 |
| ebi-a-GCST90016957 | Gut microbiota abundance (family Verrucomicrobiaceae id.4036) || id:ebi-a-GCST90016957 | CD25 on IgD+ CD38+ B cell || id:ebi-a-GCST90001783 | rs117107102 | 18 | 49473635 | A | 0.204683 | 0.0431572 | 2.92E-06 |
| ebi-a-GCST90016957 | Gut microbiota abundance (family Verrucomicrobiaceae id.4036) || id:ebi-a-GCST90016957 | CD86 on myeloid Dendritic Cell || id:ebi-a-GCST90001903 | rs117107102 | 18 | 49473635 | A | 0.204683 | 0.0431572 | 2.92E-06 |
| ebi-a-GCST90016957 | Gut microbiota abundance (family Verrucomicrobiaceae id.4036) || id:ebi-a-GCST90016957 | CD33 on CD14+ monocyte || id:ebi-a-GCST90001946 | rs117107102 | 18 | 49473635 | A | 0.204683 | 0.0431572 | 2.92E-06 |
| ebi-a-GCST90016957 | Gut microbiota abundance (family Verrucomicrobiaceae id.4036) || id:ebi-a-GCST90016957 | CD33 on CD33+ HLA DR+ CD14dim || id:ebi-a-GCST90001947 | rs117107102 | 18 | 49473635 | A | 0.204683 | 0.0431572 | 2.92E-06 |
| ebi-a-GCST90016957 | Gut microbiota abundance (family Verrucomicrobiaceae id.4036) || id:ebi-a-GCST90016957 | CD33 on CD33dim HLA DR+ CD11b+ || id:ebi-a-GCST90001948 | rs117107102 | 18 | 49473635 | A | 0.204683 | 0.0431572 | 2.92E-06 |
| ebi-a-GCST90016957 | Gut microbiota abundance (family Verrucomicrobiaceae id.4036) || id:ebi-a-GCST90016957 | CD33 on Granulocytic Myeloid-Derived Suppressor Cells || id:ebi-a-GCST90001950 | rs117107102 | 18 | 49473635 | A | 0.204683 | 0.0431572 | 2.92E-06 |
| ebi-a-GCST90016957 | Gut microbiota abundance (family Verrucomicrobiaceae id.4036) || id:ebi-a-GCST90016957 | CD33 on CD66b++ myeloid cell || id:ebi-a-GCST90001951 | rs117107102 | 18 | 49473635 | A | 0.204683 | 0.0431572 | 2.92E-06 |
| ebi-a-GCST90016957 | Gut microbiota abundance (family Verrucomicrobiaceae id.4036) || id:ebi-a-GCST90016957 | CD33 on CD33dim HLA DR- || id:ebi-a-GCST90001953 | rs117107102 | 18 | 49473635 | A | 0.204683 | 0.0431572 | 2.92E-06 |
| ebi-a-GCST90016957 | Gut microbiota abundance (family Verrucomicrobiaceae id.4036) || id:ebi-a-GCST90016957 | CD33 on basophil || id:ebi-a-GCST90001954 | rs117107102 | 18 | 49473635 | A | 0.204683 | 0.0431572 | 2.92E-06 |
| ebi-a-GCST90016957 | Gut microbiota abundance (family Verrucomicrobiaceae id.4036) || id:ebi-a-GCST90016957 | CD33 on Immature Myeloid-Derived Suppressor Cells || id:ebi-a-GCST90001955 | rs117107102 | 18 | 49473635 | A | 0.204683 | 0.0431572 | 2.92E-06 |
| ebi-a-GCST90016957 | Gut microbiota abundance (family Verrucomicrobiaceae id.4036) || id:ebi-a-GCST90016957 | FSC-A on HLA DR+ CD8+ T cell || id:ebi-a-GCST90001978 | rs117107102 | 18 | 49473635 | A | 0.204683 | 0.0431572 | 2.92E-06 |
| ebi-a-GCST90016957 | Gut microbiota abundance (family Verrucomicrobiaceae id.4036) || id:ebi-a-GCST90016957 | HLA DR on CD14+ CD16- monocyte || id:ebi-a-GCST90001988 | rs117107102 | 18 | 49473635 | A | 0.204683 | 0.0431572 | 2.92E-06 |
| ebi-a-GCST90016957 | Gut microbiota abundance (family Verrucomicrobiaceae id.4036) || id:ebi-a-GCST90016957 | HLA DR on CD14+ monocyte || id:ebi-a-GCST90001991 | rs117107102 | 18 | 49473635 | A | 0.204683 | 0.0431572 | 2.92E-06 |
| ebi-a-GCST90016957 | Gut microbiota abundance (family Verrucomicrobiaceae id.4036) || id:ebi-a-GCST90016957 | CD16 on CD14+ CD16+ monocyte || id:ebi-a-GCST90002005 | rs117107102 | 18 | 49473635 | A | 0.204683 | 0.0431572 | 2.92E-06 |
| ebi-a-GCST90016957 | Gut microbiota abundance (family Verrucomicrobiaceae id.4036) || id:ebi-a-GCST90016957 | CD45 on CD33+ HLA DR+ CD14- || id:ebi-a-GCST90002042 | rs117107102 | 18 | 49473635 | A | 0.204683 | 0.0431572 | 2.92E-06 |
| ebi-a-GCST90016957 | Gut microbiota abundance (family Verrucomicrobiaceae id.4036) || id:ebi-a-GCST90016957 | CD8 on Natural Killer T || id:ebi-a-GCST90002059 | rs117107102 | 18 | 49473635 | A | 0.204683 | 0.0431572 | 2.92E-06 |
| ebi-a-GCST90016923 | Gut microbiota abundance (class Verrucomicrobiae id.4029) || id:ebi-a-GCST90016923 | Myeloid Dendritic Cell Absolute Count || id:ebi-a-GCST90001458 | rs117107102 | 18 | 49473635 | A | 0.204683 | 0.0431572 | 2.92E-06 |
| ebi-a-GCST90016923 | Gut microbiota abundance (class Verrucomicrobiae id.4029) || id:ebi-a-GCST90016923 | CD86+ myeloid Dendritic Cell %Dendritic Cell || id:ebi-a-GCST90001465 | rs117107102 | 18 | 49473635 | A | 0.204683 | 0.0431572 | 2.92E-06 |
| ebi-a-GCST90016923 | Gut microbiota abundance (class Verrucomicrobiae id.4029) || id:ebi-a-GCST90016923 | CD62L- myeloid Dendritic Cell Absolute Count || id:ebi-a-GCST90001468 | rs117107102 | 18 | 49473635 | A | 0.204683 | 0.0431572 | 2.92E-06 |
| ebi-a-GCST90016923 | Gut microbiota abundance (class Verrucomicrobiae id.4029) || id:ebi-a-GCST90016923 | CD62L- myeloid Dendritic Cell %Dendritic Cell || id:ebi-a-GCST90001469 | rs117107102 | 18 | 49473635 | A | 0.204683 | 0.0431572 | 2.92E-06 |
| ebi-a-GCST90016923 | Gut microbiota abundance (class Verrucomicrobiae id.4029) || id:ebi-a-GCST90016923 | CD62L- CD86+ myeloid Dendritic Cell Absolute Count || id:ebi-a-GCST90001472 | rs117107102 | 18 | 49473635 | A | 0.204683 | 0.0431572 | 2.92E-06 |
| ebi-a-GCST90016923 | Gut microbiota abundance (class Verrucomicrobiae id.4029) || id:ebi-a-GCST90016923 | CD62L- CD86+ myeloid Dendritic Cell %Dendritic Cell || id:ebi-a-GCST90001473 | rs117107102 | 18 | 49473635 | A | 0.204683 | 0.0431572 | 2.92E-06 |
| ebi-a-GCST90016923 | Gut microbiota abundance (class Verrucomicrobiae id.4029) || id:ebi-a-GCST90016923 | HLA DR++ monocyte %monocyte || id:ebi-a-GCST90001475 | rs117107102 | 18 | 49473635 | A | 0.204683 | 0.0431572 | 2.92E-06 |
| ebi-a-GCST90016923 | Gut microbiota abundance (class Verrucomicrobiae id.4029) || id:ebi-a-GCST90016923 | HLA DR++ monocyte Absolute Count || id:ebi-a-GCST90001477 | rs117107102 | 18 | 49473635 | A | 0.204683 | 0.0431572 | 2.92E-06 |
| ebi-a-GCST90016923 | Gut microbiota abundance (class Verrucomicrobiae id.4029) || id:ebi-a-GCST90016923 | Basophil %CD33dim HLA DR- CD66b- || id:ebi-a-GCST90001533 | rs117107102 | 18 | 49473635 | A | 0.204683 | 0.0431572 | 2.92E-06 |
| ebi-a-GCST90016923 | Gut microbiota abundance (class Verrucomicrobiae id.4029) || id:ebi-a-GCST90016923 | CD16+ monocyte %monocyte || id:ebi-a-GCST90001587 | rs117107102 | 18 | 49473635 | A | 0.204683 | 0.0431572 | 2.92E-06 |
| ebi-a-GCST90016923 | Gut microbiota abundance (class Verrucomicrobiae id.4029) || id:ebi-a-GCST90016923 | T/B cell || id:ebi-a-GCST90001588 | rs117107102 | 18 | 49473635 | A | 0.204683 | 0.0431572 | 2.92E-06 |
| ebi-a-GCST90016923 | Gut microbiota abundance (class Verrucomicrobiae id.4029) || id:ebi-a-GCST90016923 | CD8+ Natural Killer T %T cell || id:ebi-a-GCST90001631 | rs117107102 | 18 | 49473635 | A | 0.204683 | 0.0431572 | 2.92E-06 |
| ebi-a-GCST90016923 | Gut microbiota abundance (class Verrucomicrobiae id.4029) || id:ebi-a-GCST90016923 | CD4-CD8- Natural Killer T %lymphocyte || id:ebi-a-GCST90001638 | rs117107102 | 18 | 49473635 | A | 0.204683 | 0.0431572 | 2.92E-06 |
| ebi-a-GCST90016923 | Gut microbiota abundance (class Verrucomicrobiae id.4029) || id:ebi-a-GCST90016923 | HLA DR+ Natural Killer Absolute Count || id:ebi-a-GCST90001648 | rs117107102 | 18 | 49473635 | A | 0.204683 | 0.0431572 | 2.92E-06 |
| ebi-a-GCST90016923 | Gut microbiota abundance (class Verrucomicrobiae id.4029) || id:ebi-a-GCST90016923 | HLA DR+ Natural Killer %Natural Killer || id:ebi-a-GCST90001649 | rs117107102 | 18 | 49473635 | A | 0.204683 | 0.0431572 | 2.92E-06 |
| ebi-a-GCST90016923 | Gut microbiota abundance (class Verrucomicrobiae id.4029) || id:ebi-a-GCST90016923 | HLA DR+ Natural Killer %CD3- lymphocyte || id:ebi-a-GCST90001650 | rs117107102 | 18 | 49473635 | A | 0.204683 | 0.0431572 | 2.92E-06 |
| ebi-a-GCST90016923 | Gut microbiota abundance (class Verrucomicrobiae id.4029) || id:ebi-a-GCST90016923 | CD28- CD25++ CD8+ T cell Absolute Count || id:ebi-a-GCST90001678 | rs117107102 | 18 | 49473635 | A | 0.204683 | 0.0431572 | 2.92E-06 |
| ebi-a-GCST90016923 | Gut microbiota abundance (class Verrucomicrobiae id.4029) || id:ebi-a-GCST90016923 | CD25++ CD8+ T cell %T cell || id:ebi-a-GCST90001679 | rs117107102 | 18 | 49473635 | A | 0.204683 | 0.0431572 | 2.92E-06 |
| ebi-a-GCST90016923 | Gut microbiota abundance (class Verrucomicrobiae id.4029) || id:ebi-a-GCST90016923 | CD19 on CD20- CD38- B cell || id:ebi-a-GCST90001722 | rs117107102 | 18 | 49473635 | A | 0.204683 | 0.0431572 | 2.92E-06 |
| ebi-a-GCST90016923 | Gut microbiota abundance (class Verrucomicrobiae id.4029) || id:ebi-a-GCST90016923 | CD25 on B cell || id:ebi-a-GCST90001775 | rs117107102 | 18 | 49473635 | A | 0.204683 | 0.0431572 | 2.92E-06 |
| ebi-a-GCST90016923 | Gut microbiota abundance (class Verrucomicrobiae id.4029) || id:ebi-a-GCST90016923 | CD25 on IgD+ CD24- B cell || id:ebi-a-GCST90001779 | rs117107102 | 18 | 49473635 | A | 0.204683 | 0.0431572 | 2.92E-06 |
| ebi-a-GCST90016923 | Gut microbiota abundance (class Verrucomicrobiae id.4029) || id:ebi-a-GCST90016923 | CD25 on IgD+ CD38- naive B cell || id:ebi-a-GCST90001781 | rs117107102 | 18 | 49473635 | A | 0.204683 | 0.0431572 | 2.92E-06 |
| ebi-a-GCST90016923 | Gut microbiota abundance (class Verrucomicrobiae id.4029) || id:ebi-a-GCST90016923 | CD25 on IgD+ CD38+ B cell || id:ebi-a-GCST90001783 | rs117107102 | 18 | 49473635 | A | 0.204683 | 0.0431572 | 2.92E-06 |
| ebi-a-GCST90016923 | Gut microbiota abundance (class Verrucomicrobiae id.4029) || id:ebi-a-GCST90016923 | CD86 on myeloid Dendritic Cell || id:ebi-a-GCST90001903 | rs117107102 | 18 | 49473635 | A | 0.204683 | 0.0431572 | 2.92E-06 |
| ebi-a-GCST90016923 | Gut microbiota abundance (class Verrucomicrobiae id.4029) || id:ebi-a-GCST90016923 | CD33 on CD14+ monocyte || id:ebi-a-GCST90001946 | rs117107102 | 18 | 49473635 | A | 0.204683 | 0.0431572 | 2.92E-06 |
| ebi-a-GCST90016923 | Gut microbiota abundance (class Verrucomicrobiae id.4029) || id:ebi-a-GCST90016923 | CD33 on CD33+ HLA DR+ CD14dim || id:ebi-a-GCST90001947 | rs117107102 | 18 | 49473635 | A | 0.204683 | 0.0431572 | 2.92E-06 |
| ebi-a-GCST90016923 | Gut microbiota abundance (class Verrucomicrobiae id.4029) || id:ebi-a-GCST90016923 | CD33 on CD33dim HLA DR+ CD11b+ || id:ebi-a-GCST90001948 | rs117107102 | 18 | 49473635 | A | 0.204683 | 0.0431572 | 2.92E-06 |
| ebi-a-GCST90016923 | Gut microbiota abundance (class Verrucomicrobiae id.4029) || id:ebi-a-GCST90016923 | CD33 on Granulocytic Myeloid-Derived Suppressor Cells || id:ebi-a-GCST90001950 | rs117107102 | 18 | 49473635 | A | 0.204683 | 0.0431572 | 2.92E-06 |
| ebi-a-GCST90016923 | Gut microbiota abundance (class Verrucomicrobiae id.4029) || id:ebi-a-GCST90016923 | CD33 on CD66b++ myeloid cell || id:ebi-a-GCST90001951 | rs117107102 | 18 | 49473635 | A | 0.204683 | 0.0431572 | 2.92E-06 |
| ebi-a-GCST90016923 | Gut microbiota abundance (class Verrucomicrobiae id.4029) || id:ebi-a-GCST90016923 | CD33 on CD33dim HLA DR- || id:ebi-a-GCST90001953 | rs117107102 | 18 | 49473635 | A | 0.204683 | 0.0431572 | 2.92E-06 |
| ebi-a-GCST90016923 | Gut microbiota abundance (class Verrucomicrobiae id.4029) || id:ebi-a-GCST90016923 | CD33 on basophil || id:ebi-a-GCST90001954 | rs117107102 | 18 | 49473635 | A | 0.204683 | 0.0431572 | 2.92E-06 |
| ebi-a-GCST90016923 | Gut microbiota abundance (class Verrucomicrobiae id.4029) || id:ebi-a-GCST90016923 | CD33 on Immature Myeloid-Derived Suppressor Cells || id:ebi-a-GCST90001955 | rs117107102 | 18 | 49473635 | A | 0.204683 | 0.0431572 | 2.92E-06 |
| ebi-a-GCST90016923 | Gut microbiota abundance (class Verrucomicrobiae id.4029) || id:ebi-a-GCST90016923 | FSC-A on HLA DR+ CD8+ T cell || id:ebi-a-GCST90001978 | rs117107102 | 18 | 49473635 | A | 0.204683 | 0.0431572 | 2.92E-06 |
| ebi-a-GCST90016923 | Gut microbiota abundance (class Verrucomicrobiae id.4029) || id:ebi-a-GCST90016923 | HLA DR on CD14+ CD16- monocyte || id:ebi-a-GCST90001988 | rs117107102 | 18 | 49473635 | A | 0.204683 | 0.0431572 | 2.92E-06 |
| ebi-a-GCST90016923 | Gut microbiota abundance (class Verrucomicrobiae id.4029) || id:ebi-a-GCST90016923 | HLA DR on CD14+ monocyte || id:ebi-a-GCST90001991 | rs117107102 | 18 | 49473635 | A | 0.204683 | 0.0431572 | 2.92E-06 |
| ebi-a-GCST90016923 | Gut microbiota abundance (class Verrucomicrobiae id.4029) || id:ebi-a-GCST90016923 | CD16 on CD14+ CD16+ monocyte || id:ebi-a-GCST90002005 | rs117107102 | 18 | 49473635 | A | 0.204683 | 0.0431572 | 2.92E-06 |
| ebi-a-GCST90016923 | Gut microbiota abundance (class Verrucomicrobiae id.4029) || id:ebi-a-GCST90016923 | CD45 on CD33+ HLA DR+ CD14- || id:ebi-a-GCST90002042 | rs117107102 | 18 | 49473635 | A | 0.204683 | 0.0431572 | 2.92E-06 |
| ebi-a-GCST90016923 | Gut microbiota abundance (class Verrucomicrobiae id.4029) || id:ebi-a-GCST90016923 | CD8 on Natural Killer T || id:ebi-a-GCST90002059 | rs117107102 | 18 | 49473635 | A | 0.204683 | 0.0431572 | 2.92E-06 |
| ebi-a-GCST90017108 | Gut microbiota abundance (order Verrucomicrobiales id.4030) || id:ebi-a-GCST90017108 | Myeloid Dendritic Cell Absolute Count || id:ebi-a-GCST90001458 | rs117107102 | 18 | 49473635 | A | 0.204683 | 0.0431572 | 2.92E-06 |
| ebi-a-GCST90017108 | Gut microbiota abundance (order Verrucomicrobiales id.4030) || id:ebi-a-GCST90017108 | CD86+ myeloid Dendritic Cell %Dendritic Cell || id:ebi-a-GCST90001465 | rs117107102 | 18 | 49473635 | A | 0.204683 | 0.0431572 | 2.92E-06 |
| ebi-a-GCST90017108 | Gut microbiota abundance (order Verrucomicrobiales id.4030) || id:ebi-a-GCST90017108 | CD62L- myeloid Dendritic Cell Absolute Count || id:ebi-a-GCST90001468 | rs117107102 | 18 | 49473635 | A | 0.204683 | 0.0431572 | 2.92E-06 |
| ebi-a-GCST90017108 | Gut microbiota abundance (order Verrucomicrobiales id.4030) || id:ebi-a-GCST90017108 | CD62L- myeloid Dendritic Cell %Dendritic Cell || id:ebi-a-GCST90001469 | rs117107102 | 18 | 49473635 | A | 0.204683 | 0.0431572 | 2.92E-06 |
| ebi-a-GCST90017108 | Gut microbiota abundance (order Verrucomicrobiales id.4030) || id:ebi-a-GCST90017108 | CD62L- CD86+ myeloid Dendritic Cell Absolute Count || id:ebi-a-GCST90001472 | rs117107102 | 18 | 49473635 | A | 0.204683 | 0.0431572 | 2.92E-06 |
| ebi-a-GCST90017108 | Gut microbiota abundance (order Verrucomicrobiales id.4030) || id:ebi-a-GCST90017108 | CD62L- CD86+ myeloid Dendritic Cell %Dendritic Cell || id:ebi-a-GCST90001473 | rs117107102 | 18 | 49473635 | A | 0.204683 | 0.0431572 | 2.92E-06 |
| ebi-a-GCST90017108 | Gut microbiota abundance (order Verrucomicrobiales id.4030) || id:ebi-a-GCST90017108 | HLA DR++ monocyte %monocyte || id:ebi-a-GCST90001475 | rs117107102 | 18 | 49473635 | A | 0.204683 | 0.0431572 | 2.92E-06 |
| ebi-a-GCST90017108 | Gut microbiota abundance (order Verrucomicrobiales id.4030) || id:ebi-a-GCST90017108 | HLA DR++ monocyte Absolute Count || id:ebi-a-GCST90001477 | rs117107102 | 18 | 49473635 | A | 0.204683 | 0.0431572 | 2.92E-06 |
| ebi-a-GCST90017108 | Gut microbiota abundance (order Verrucomicrobiales id.4030) || id:ebi-a-GCST90017108 | Basophil %CD33dim HLA DR- CD66b- || id:ebi-a-GCST90001533 | rs117107102 | 18 | 49473635 | A | 0.204683 | 0.0431572 | 2.92E-06 |
| ebi-a-GCST90017108 | Gut microbiota abundance (order Verrucomicrobiales id.4030) || id:ebi-a-GCST90017108 | CD16+ monocyte %monocyte || id:ebi-a-GCST90001587 | rs117107102 | 18 | 49473635 | A | 0.204683 | 0.0431572 | 2.92E-06 |
| ebi-a-GCST90017108 | Gut microbiota abundance (order Verrucomicrobiales id.4030) || id:ebi-a-GCST90017108 | T/B cell || id:ebi-a-GCST90001588 | rs117107102 | 18 | 49473635 | A | 0.204683 | 0.0431572 | 2.92E-06 |
| ebi-a-GCST90017108 | Gut microbiota abundance (order Verrucomicrobiales id.4030) || id:ebi-a-GCST90017108 | CD8+ Natural Killer T %T cell || id:ebi-a-GCST90001631 | rs117107102 | 18 | 49473635 | A | 0.204683 | 0.0431572 | 2.92E-06 |
| ebi-a-GCST90017108 | Gut microbiota abundance (order Verrucomicrobiales id.4030) || id:ebi-a-GCST90017108 | CD4-CD8- Natural Killer T %lymphocyte || id:ebi-a-GCST90001638 | rs117107102 | 18 | 49473635 | A | 0.204683 | 0.0431572 | 2.92E-06 |
| ebi-a-GCST90017108 | Gut microbiota abundance (order Verrucomicrobiales id.4030) || id:ebi-a-GCST90017108 | HLA DR+ Natural Killer Absolute Count || id:ebi-a-GCST90001648 | rs117107102 | 18 | 49473635 | A | 0.204683 | 0.0431572 | 2.92E-06 |
| ebi-a-GCST90017108 | Gut microbiota abundance (order Verrucomicrobiales id.4030) || id:ebi-a-GCST90017108 | HLA DR+ Natural Killer %Natural Killer || id:ebi-a-GCST90001649 | rs117107102 | 18 | 49473635 | A | 0.204683 | 0.0431572 | 2.92E-06 |
| ebi-a-GCST90017108 | Gut microbiota abundance (order Verrucomicrobiales id.4030) || id:ebi-a-GCST90017108 | HLA DR+ Natural Killer %CD3- lymphocyte || id:ebi-a-GCST90001650 | rs117107102 | 18 | 49473635 | A | 0.204683 | 0.0431572 | 2.92E-06 |
| ebi-a-GCST90017108 | Gut microbiota abundance (order Verrucomicrobiales id.4030) || id:ebi-a-GCST90017108 | CD28- CD25++ CD8+ T cell Absolute Count || id:ebi-a-GCST90001678 | rs117107102 | 18 | 49473635 | A | 0.204683 | 0.0431572 | 2.92E-06 |
| ebi-a-GCST90017108 | Gut microbiota abundance (order Verrucomicrobiales id.4030) || id:ebi-a-GCST90017108 | CD25++ CD8+ T cell %T cell || id:ebi-a-GCST90001679 | rs117107102 | 18 | 49473635 | A | 0.204683 | 0.0431572 | 2.92E-06 |
| ebi-a-GCST90017108 | Gut microbiota abundance (order Verrucomicrobiales id.4030) || id:ebi-a-GCST90017108 | CD19 on CD20- CD38- B cell || id:ebi-a-GCST90001722 | rs117107102 | 18 | 49473635 | A | 0.204683 | 0.0431572 | 2.92E-06 |
| ebi-a-GCST90017108 | Gut microbiota abundance (order Verrucomicrobiales id.4030) || id:ebi-a-GCST90017108 | CD25 on B cell || id:ebi-a-GCST90001775 | rs117107102 | 18 | 49473635 | A | 0.204683 | 0.0431572 | 2.92E-06 |
| ebi-a-GCST90017108 | Gut microbiota abundance (order Verrucomicrobiales id.4030) || id:ebi-a-GCST90017108 | CD25 on IgD+ CD24- B cell || id:ebi-a-GCST90001779 | rs117107102 | 18 | 49473635 | A | 0.204683 | 0.0431572 | 2.92E-06 |
| ebi-a-GCST90017108 | Gut microbiota abundance (order Verrucomicrobiales id.4030) || id:ebi-a-GCST90017108 | CD25 on IgD+ CD38- naive B cell || id:ebi-a-GCST90001781 | rs117107102 | 18 | 49473635 | A | 0.204683 | 0.0431572 | 2.92E-06 |
| ebi-a-GCST90017108 | Gut microbiota abundance (order Verrucomicrobiales id.4030) || id:ebi-a-GCST90017108 | CD25 on IgD+ CD38+ B cell || id:ebi-a-GCST90001783 | rs117107102 | 18 | 49473635 | A | 0.204683 | 0.0431572 | 2.92E-06 |
| ebi-a-GCST90017108 | Gut microbiota abundance (order Verrucomicrobiales id.4030) || id:ebi-a-GCST90017108 | CD86 on myeloid Dendritic Cell || id:ebi-a-GCST90001903 | rs117107102 | 18 | 49473635 | A | 0.204683 | 0.0431572 | 2.92E-06 |
| ebi-a-GCST90017108 | Gut microbiota abundance (order Verrucomicrobiales id.4030) || id:ebi-a-GCST90017108 | CD33 on CD14+ monocyte || id:ebi-a-GCST90001946 | rs117107102 | 18 | 49473635 | A | 0.204683 | 0.0431572 | 2.92E-06 |
| ebi-a-GCST90017108 | Gut microbiota abundance (order Verrucomicrobiales id.4030) || id:ebi-a-GCST90017108 | CD33 on CD33+ HLA DR+ CD14dim || id:ebi-a-GCST90001947 | rs117107102 | 18 | 49473635 | A | 0.204683 | 0.0431572 | 2.92E-06 |
| ebi-a-GCST90017108 | Gut microbiota abundance (order Verrucomicrobiales id.4030) || id:ebi-a-GCST90017108 | CD33 on CD33dim HLA DR+ CD11b+ || id:ebi-a-GCST90001948 | rs117107102 | 18 | 49473635 | A | 0.204683 | 0.0431572 | 2.92E-06 |
| ebi-a-GCST90017108 | Gut microbiota abundance (order Verrucomicrobiales id.4030) || id:ebi-a-GCST90017108 | CD33 on Granulocytic Myeloid-Derived Suppressor Cells || id:ebi-a-GCST90001950 | rs117107102 | 18 | 49473635 | A | 0.204683 | 0.0431572 | 2.92E-06 |
| ebi-a-GCST90017108 | Gut microbiota abundance (order Verrucomicrobiales id.4030) || id:ebi-a-GCST90017108 | CD33 on CD66b++ myeloid cell || id:ebi-a-GCST90001951 | rs117107102 | 18 | 49473635 | A | 0.204683 | 0.0431572 | 2.92E-06 |
| ebi-a-GCST90017108 | Gut microbiota abundance (order Verrucomicrobiales id.4030) || id:ebi-a-GCST90017108 | CD33 on CD33dim HLA DR- || id:ebi-a-GCST90001953 | rs117107102 | 18 | 49473635 | A | 0.204683 | 0.0431572 | 2.92E-06 |
| ebi-a-GCST90017108 | Gut microbiota abundance (order Verrucomicrobiales id.4030) || id:ebi-a-GCST90017108 | CD33 on basophil || id:ebi-a-GCST90001954 | rs117107102 | 18 | 49473635 | A | 0.204683 | 0.0431572 | 2.92E-06 |
| ebi-a-GCST90017108 | Gut microbiota abundance (order Verrucomicrobiales id.4030) || id:ebi-a-GCST90017108 | CD33 on Immature Myeloid-Derived Suppressor Cells || id:ebi-a-GCST90001955 | rs117107102 | 18 | 49473635 | A | 0.204683 | 0.0431572 | 2.92E-06 |
| ebi-a-GCST90017108 | Gut microbiota abundance (order Verrucomicrobiales id.4030) || id:ebi-a-GCST90017108 | FSC-A on HLA DR+ CD8+ T cell || id:ebi-a-GCST90001978 | rs117107102 | 18 | 49473635 | A | 0.204683 | 0.0431572 | 2.92E-06 |
| ebi-a-GCST90017108 | Gut microbiota abundance (order Verrucomicrobiales id.4030) || id:ebi-a-GCST90017108 | HLA DR on CD14+ CD16- monocyte || id:ebi-a-GCST90001988 | rs117107102 | 18 | 49473635 | A | 0.204683 | 0.0431572 | 2.92E-06 |
| ebi-a-GCST90017108 | Gut microbiota abundance (order Verrucomicrobiales id.4030) || id:ebi-a-GCST90017108 | HLA DR on CD14+ monocyte || id:ebi-a-GCST90001991 | rs117107102 | 18 | 49473635 | A | 0.204683 | 0.0431572 | 2.92E-06 |
| ebi-a-GCST90017108 | Gut microbiota abundance (order Verrucomicrobiales id.4030) || id:ebi-a-GCST90017108 | CD16 on CD14+ CD16+ monocyte || id:ebi-a-GCST90002005 | rs117107102 | 18 | 49473635 | A | 0.204683 | 0.0431572 | 2.92E-06 |
| ebi-a-GCST90017108 | Gut microbiota abundance (order Verrucomicrobiales id.4030) || id:ebi-a-GCST90017108 | CD45 on CD33+ HLA DR+ CD14- || id:ebi-a-GCST90002042 | rs117107102 | 18 | 49473635 | A | 0.204683 | 0.0431572 | 2.92E-06 |
| ebi-a-GCST90017108 | Gut microbiota abundance (order Verrucomicrobiales id.4030) || id:ebi-a-GCST90017108 | CD8 on Natural Killer T || id:ebi-a-GCST90002059 | rs117107102 | 18 | 49473635 | A | 0.204683 | 0.0431572 | 2.92E-06 |
| ebi-a-GCST90016961 | Gut microbiota abundance (genus Akkermansia id.4037) || id:ebi-a-GCST90016961 | Myeloid Dendritic Cell Absolute Count || id:ebi-a-GCST90001458 | rs117107102 | 18 | 49473635 | A | 0.204406 | 0.0431629 | 3.01E-06 |
| ebi-a-GCST90016961 | Gut microbiota abundance (genus Akkermansia id.4037) || id:ebi-a-GCST90016961 | CD86+ myeloid Dendritic Cell %Dendritic Cell || id:ebi-a-GCST90001465 | rs117107102 | 18 | 49473635 | A | 0.204406 | 0.0431629 | 3.01E-06 |
| ebi-a-GCST90016961 | Gut microbiota abundance (genus Akkermansia id.4037) || id:ebi-a-GCST90016961 | CD62L- myeloid Dendritic Cell Absolute Count || id:ebi-a-GCST90001468 | rs117107102 | 18 | 49473635 | A | 0.204406 | 0.0431629 | 3.01E-06 |
| ebi-a-GCST90016961 | Gut microbiota abundance (genus Akkermansia id.4037) || id:ebi-a-GCST90016961 | CD62L- myeloid Dendritic Cell %Dendritic Cell || id:ebi-a-GCST90001469 | rs117107102 | 18 | 49473635 | A | 0.204406 | 0.0431629 | 3.01E-06 |
| ebi-a-GCST90016961 | Gut microbiota abundance (genus Akkermansia id.4037) || id:ebi-a-GCST90016961 | CD62L- CD86+ myeloid Dendritic Cell Absolute Count || id:ebi-a-GCST90001472 | rs117107102 | 18 | 49473635 | A | 0.204406 | 0.0431629 | 3.01E-06 |
| ebi-a-GCST90016961 | Gut microbiota abundance (genus Akkermansia id.4037) || id:ebi-a-GCST90016961 | CD62L- CD86+ myeloid Dendritic Cell %Dendritic Cell || id:ebi-a-GCST90001473 | rs117107102 | 18 | 49473635 | A | 0.204406 | 0.0431629 | 3.01E-06 |
| ebi-a-GCST90016961 | Gut microbiota abundance (genus Akkermansia id.4037) || id:ebi-a-GCST90016961 | HLA DR++ monocyte %monocyte || id:ebi-a-GCST90001475 | rs117107102 | 18 | 49473635 | A | 0.204406 | 0.0431629 | 3.01E-06 |
| ebi-a-GCST90016961 | Gut microbiota abundance (genus Akkermansia id.4037) || id:ebi-a-GCST90016961 | HLA DR++ monocyte Absolute Count || id:ebi-a-GCST90001477 | rs117107102 | 18 | 49473635 | A | 0.204406 | 0.0431629 | 3.01E-06 |
| ebi-a-GCST90016961 | Gut microbiota abundance (genus Akkermansia id.4037) || id:ebi-a-GCST90016961 | Basophil %CD33dim HLA DR- CD66b- || id:ebi-a-GCST90001533 | rs117107102 | 18 | 49473635 | A | 0.204406 | 0.0431629 | 3.01E-06 |
| ebi-a-GCST90016961 | Gut microbiota abundance (genus Akkermansia id.4037) || id:ebi-a-GCST90016961 | CD16+ monocyte %monocyte || id:ebi-a-GCST90001587 | rs117107102 | 18 | 49473635 | A | 0.204406 | 0.0431629 | 3.01E-06 |
| ebi-a-GCST90016961 | Gut microbiota abundance (genus Akkermansia id.4037) || id:ebi-a-GCST90016961 | T/B cell || id:ebi-a-GCST90001588 | rs117107102 | 18 | 49473635 | A | 0.204406 | 0.0431629 | 3.01E-06 |
| ebi-a-GCST90016961 | Gut microbiota abundance (genus Akkermansia id.4037) || id:ebi-a-GCST90016961 | CD8+ Natural Killer T %T cell || id:ebi-a-GCST90001631 | rs117107102 | 18 | 49473635 | A | 0.204406 | 0.0431629 | 3.01E-06 |
| ebi-a-GCST90016961 | Gut microbiota abundance (genus Akkermansia id.4037) || id:ebi-a-GCST90016961 | CD4-CD8- Natural Killer T %lymphocyte || id:ebi-a-GCST90001638 | rs117107102 | 18 | 49473635 | A | 0.204406 | 0.0431629 | 3.01E-06 |
| ebi-a-GCST90016961 | Gut microbiota abundance (genus Akkermansia id.4037) || id:ebi-a-GCST90016961 | HLA DR+ Natural Killer Absolute Count || id:ebi-a-GCST90001648 | rs117107102 | 18 | 49473635 | A | 0.204406 | 0.0431629 | 3.01E-06 |
| ebi-a-GCST90016961 | Gut microbiota abundance (genus Akkermansia id.4037) || id:ebi-a-GCST90016961 | HLA DR+ Natural Killer %Natural Killer || id:ebi-a-GCST90001649 | rs117107102 | 18 | 49473635 | A | 0.204406 | 0.0431629 | 3.01E-06 |
| ebi-a-GCST90016961 | Gut microbiota abundance (genus Akkermansia id.4037) || id:ebi-a-GCST90016961 | HLA DR+ Natural Killer %CD3- lymphocyte || id:ebi-a-GCST90001650 | rs117107102 | 18 | 49473635 | A | 0.204406 | 0.0431629 | 3.01E-06 |
| ebi-a-GCST90016961 | Gut microbiota abundance (genus Akkermansia id.4037) || id:ebi-a-GCST90016961 | CD28- CD25++ CD8+ T cell Absolute Count || id:ebi-a-GCST90001678 | rs117107102 | 18 | 49473635 | A | 0.204406 | 0.0431629 | 3.01E-06 |
| ebi-a-GCST90016961 | Gut microbiota abundance (genus Akkermansia id.4037) || id:ebi-a-GCST90016961 | CD25++ CD8+ T cell %T cell || id:ebi-a-GCST90001679 | rs117107102 | 18 | 49473635 | A | 0.204406 | 0.0431629 | 3.01E-06 |
| ebi-a-GCST90016961 | Gut microbiota abundance (genus Akkermansia id.4037) || id:ebi-a-GCST90016961 | CD19 on CD20- CD38- B cell || id:ebi-a-GCST90001722 | rs117107102 | 18 | 49473635 | A | 0.204406 | 0.0431629 | 3.01E-06 |
| ebi-a-GCST90016961 | Gut microbiota abundance (genus Akkermansia id.4037) || id:ebi-a-GCST90016961 | CD25 on B cell || id:ebi-a-GCST90001775 | rs117107102 | 18 | 49473635 | A | 0.204406 | 0.0431629 | 3.01E-06 |
| ebi-a-GCST90016961 | Gut microbiota abundance (genus Akkermansia id.4037) || id:ebi-a-GCST90016961 | CD25 on IgD+ CD24- B cell || id:ebi-a-GCST90001779 | rs117107102 | 18 | 49473635 | A | 0.204406 | 0.0431629 | 3.01E-06 |
| ebi-a-GCST90016961 | Gut microbiota abundance (genus Akkermansia id.4037) || id:ebi-a-GCST90016961 | CD25 on IgD+ CD38- naive B cell || id:ebi-a-GCST90001781 | rs117107102 | 18 | 49473635 | A | 0.204406 | 0.0431629 | 3.01E-06 |
| ebi-a-GCST90016961 | Gut microbiota abundance (genus Akkermansia id.4037) || id:ebi-a-GCST90016961 | CD25 on IgD+ CD38+ B cell || id:ebi-a-GCST90001783 | rs117107102 | 18 | 49473635 | A | 0.204406 | 0.0431629 | 3.01E-06 |
| ebi-a-GCST90016961 | Gut microbiota abundance (genus Akkermansia id.4037) || id:ebi-a-GCST90016961 | CD86 on myeloid Dendritic Cell || id:ebi-a-GCST90001903 | rs117107102 | 18 | 49473635 | A | 0.204406 | 0.0431629 | 3.01E-06 |
| ebi-a-GCST90016961 | Gut microbiota abundance (genus Akkermansia id.4037) || id:ebi-a-GCST90016961 | CD33 on CD14+ monocyte || id:ebi-a-GCST90001946 | rs117107102 | 18 | 49473635 | A | 0.204406 | 0.0431629 | 3.01E-06 |
| ebi-a-GCST90016961 | Gut microbiota abundance (genus Akkermansia id.4037) || id:ebi-a-GCST90016961 | CD33 on CD33+ HLA DR+ CD14dim || id:ebi-a-GCST90001947 | rs117107102 | 18 | 49473635 | A | 0.204406 | 0.0431629 | 3.01E-06 |
| ebi-a-GCST90016961 | Gut microbiota abundance (genus Akkermansia id.4037) || id:ebi-a-GCST90016961 | CD33 on CD33dim HLA DR+ CD11b+ || id:ebi-a-GCST90001948 | rs117107102 | 18 | 49473635 | A | 0.204406 | 0.0431629 | 3.01E-06 |
| ebi-a-GCST90016961 | Gut microbiota abundance (genus Akkermansia id.4037) || id:ebi-a-GCST90016961 | CD33 on Granulocytic Myeloid-Derived Suppressor Cells || id:ebi-a-GCST90001950 | rs117107102 | 18 | 49473635 | A | 0.204406 | 0.0431629 | 3.01E-06 |
| ebi-a-GCST90016961 | Gut microbiota abundance (genus Akkermansia id.4037) || id:ebi-a-GCST90016961 | CD33 on CD66b++ myeloid cell || id:ebi-a-GCST90001951 | rs117107102 | 18 | 49473635 | A | 0.204406 | 0.0431629 | 3.01E-06 |
| ebi-a-GCST90016961 | Gut microbiota abundance (genus Akkermansia id.4037) || id:ebi-a-GCST90016961 | CD33 on CD33dim HLA DR- || id:ebi-a-GCST90001953 | rs117107102 | 18 | 49473635 | A | 0.204406 | 0.0431629 | 3.01E-06 |
| ebi-a-GCST90016961 | Gut microbiota abundance (genus Akkermansia id.4037) || id:ebi-a-GCST90016961 | CD33 on basophil || id:ebi-a-GCST90001954 | rs117107102 | 18 | 49473635 | A | 0.204406 | 0.0431629 | 3.01E-06 |
| ebi-a-GCST90016961 | Gut microbiota abundance (genus Akkermansia id.4037) || id:ebi-a-GCST90016961 | CD33 on Immature Myeloid-Derived Suppressor Cells || id:ebi-a-GCST90001955 | rs117107102 | 18 | 49473635 | A | 0.204406 | 0.0431629 | 3.01E-06 |
| ebi-a-GCST90016961 | Gut microbiota abundance (genus Akkermansia id.4037) || id:ebi-a-GCST90016961 | FSC-A on HLA DR+ CD8+ T cell || id:ebi-a-GCST90001978 | rs117107102 | 18 | 49473635 | A | 0.204406 | 0.0431629 | 3.01E-06 |
| ebi-a-GCST90016961 | Gut microbiota abundance (genus Akkermansia id.4037) || id:ebi-a-GCST90016961 | HLA DR on CD14+ CD16- monocyte || id:ebi-a-GCST90001988 | rs117107102 | 18 | 49473635 | A | 0.204406 | 0.0431629 | 3.01E-06 |
| ebi-a-GCST90016961 | Gut microbiota abundance (genus Akkermansia id.4037) || id:ebi-a-GCST90016961 | HLA DR on CD14+ monocyte || id:ebi-a-GCST90001991 | rs117107102 | 18 | 49473635 | A | 0.204406 | 0.0431629 | 3.01E-06 |
| ebi-a-GCST90016961 | Gut microbiota abundance (genus Akkermansia id.4037) || id:ebi-a-GCST90016961 | CD16 on CD14+ CD16+ monocyte || id:ebi-a-GCST90002005 | rs117107102 | 18 | 49473635 | A | 0.204406 | 0.0431629 | 3.01E-06 |
| ebi-a-GCST90016961 | Gut microbiota abundance (genus Akkermansia id.4037) || id:ebi-a-GCST90016961 | CD45 on CD33+ HLA DR+ CD14- || id:ebi-a-GCST90002042 | rs117107102 | 18 | 49473635 | A | 0.204406 | 0.0431629 | 3.01E-06 |
| ebi-a-GCST90016961 | Gut microbiota abundance (genus Akkermansia id.4037) || id:ebi-a-GCST90016961 | CD8 on Natural Killer T || id:ebi-a-GCST90002059 | rs117107102 | 18 | 49473635 | A | 0.204406 | 0.0431629 | 3.01E-06 |
| ebi-a-GCST90016957 | Gut microbiota abundance (family Verrucomicrobiaceae id.4036) || id:ebi-a-GCST90016957 | Myeloid Dendritic Cell Absolute Count || id:ebi-a-GCST90001458 | rs11729256 | 4 | 95027272 | T | 0.0749798 | 0.0150177 | 6.73E-07 |
| ebi-a-GCST90016957 | Gut microbiota abundance (family Verrucomicrobiaceae id.4036) || id:ebi-a-GCST90016957 | CD86+ myeloid Dendritic Cell %Dendritic Cell || id:ebi-a-GCST90001465 | rs11729256 | 4 | 95027272 | T | 0.0749798 | 0.0150177 | 6.73E-07 |
| ebi-a-GCST90016957 | Gut microbiota abundance (family Verrucomicrobiaceae id.4036) || id:ebi-a-GCST90016957 | CD62L- myeloid Dendritic Cell Absolute Count || id:ebi-a-GCST90001468 | rs11729256 | 4 | 95027272 | T | 0.0749798 | 0.0150177 | 6.73E-07 |
| ebi-a-GCST90016957 | Gut microbiota abundance (family Verrucomicrobiaceae id.4036) || id:ebi-a-GCST90016957 | CD62L- myeloid Dendritic Cell %Dendritic Cell || id:ebi-a-GCST90001469 | rs11729256 | 4 | 95027272 | T | 0.0749798 | 0.0150177 | 6.73E-07 |
| ebi-a-GCST90016957 | Gut microbiota abundance (family Verrucomicrobiaceae id.4036) || id:ebi-a-GCST90016957 | CD62L- CD86+ myeloid Dendritic Cell Absolute Count || id:ebi-a-GCST90001472 | rs11729256 | 4 | 95027272 | T | 0.0749798 | 0.0150177 | 6.73E-07 |
| ebi-a-GCST90016957 | Gut microbiota abundance (family Verrucomicrobiaceae id.4036) || id:ebi-a-GCST90016957 | CD62L- CD86+ myeloid Dendritic Cell %Dendritic Cell || id:ebi-a-GCST90001473 | rs11729256 | 4 | 95027272 | T | 0.0749798 | 0.0150177 | 6.73E-07 |
| ebi-a-GCST90016957 | Gut microbiota abundance (family Verrucomicrobiaceae id.4036) || id:ebi-a-GCST90016957 | HLA DR++ monocyte %monocyte || id:ebi-a-GCST90001475 | rs11729256 | 4 | 95027272 | T | 0.0749798 | 0.0150177 | 6.73E-07 |
| ebi-a-GCST90016957 | Gut microbiota abundance (family Verrucomicrobiaceae id.4036) || id:ebi-a-GCST90016957 | HLA DR++ monocyte Absolute Count || id:ebi-a-GCST90001477 | rs11729256 | 4 | 95027272 | T | 0.0749798 | 0.0150177 | 6.73E-07 |
| ebi-a-GCST90016957 | Gut microbiota abundance (family Verrucomicrobiaceae id.4036) || id:ebi-a-GCST90016957 | Basophil %CD33dim HLA DR- CD66b- || id:ebi-a-GCST90001533 | rs11729256 | 4 | 95027272 | T | 0.0749798 | 0.0150177 | 6.73E-07 |
| ebi-a-GCST90016957 | Gut microbiota abundance (family Verrucomicrobiaceae id.4036) || id:ebi-a-GCST90016957 | CD16+ monocyte %monocyte || id:ebi-a-GCST90001587 | rs11729256 | 4 | 95027272 | T | 0.0749798 | 0.0150177 | 6.73E-07 |
| ebi-a-GCST90016957 | Gut microbiota abundance (family Verrucomicrobiaceae id.4036) || id:ebi-a-GCST90016957 | T/B cell || id:ebi-a-GCST90001588 | rs11729256 | 4 | 95027272 | T | 0.0749798 | 0.0150177 | 6.73E-07 |
| ebi-a-GCST90016957 | Gut microbiota abundance (family Verrucomicrobiaceae id.4036) || id:ebi-a-GCST90016957 | CD4+CD8+ T cell %T cell || id:ebi-a-GCST90001595 | rs11729256 | 4 | 95027272 | T | 0.0749798 | 0.0150177 | 6.73E-07 |
| ebi-a-GCST90016957 | Gut microbiota abundance (family Verrucomicrobiaceae id.4036) || id:ebi-a-GCST90016957 | CD8+ Natural Killer T %T cell || id:ebi-a-GCST90001631 | rs11729256 | 4 | 95027272 | T | 0.0749798 | 0.0150177 | 6.73E-07 |
| ebi-a-GCST90016957 | Gut microbiota abundance (family Verrucomicrobiaceae id.4036) || id:ebi-a-GCST90016957 | CD4-CD8- Natural Killer T %lymphocyte || id:ebi-a-GCST90001638 | rs11729256 | 4 | 95027272 | T | 0.0749798 | 0.0150177 | 6.73E-07 |
| ebi-a-GCST90016957 | Gut microbiota abundance (family Verrucomicrobiaceae id.4036) || id:ebi-a-GCST90016957 | HLA DR+ Natural Killer Absolute Count || id:ebi-a-GCST90001648 | rs11729256 | 4 | 95027272 | T | 0.0749798 | 0.0150177 | 6.73E-07 |
| ebi-a-GCST90016957 | Gut microbiota abundance (family Verrucomicrobiaceae id.4036) || id:ebi-a-GCST90016957 | HLA DR+ Natural Killer %Natural Killer || id:ebi-a-GCST90001649 | rs11729256 | 4 | 95027272 | T | 0.0749798 | 0.0150177 | 6.73E-07 |
| ebi-a-GCST90016957 | Gut microbiota abundance (family Verrucomicrobiaceae id.4036) || id:ebi-a-GCST90016957 | HLA DR+ Natural Killer %CD3- lymphocyte || id:ebi-a-GCST90001650 | rs11729256 | 4 | 95027272 | T | 0.0749798 | 0.0150177 | 6.73E-07 |
| ebi-a-GCST90016957 | Gut microbiota abundance (family Verrucomicrobiaceae id.4036) || id:ebi-a-GCST90016957 | CD28- CD25++ CD8+ T cell Absolute Count || id:ebi-a-GCST90001678 | rs11729256 | 4 | 95027272 | T | 0.0749798 | 0.0150177 | 6.73E-07 |
| ebi-a-GCST90016957 | Gut microbiota abundance (family Verrucomicrobiaceae id.4036) || id:ebi-a-GCST90016957 | CD25++ CD8+ T cell %T cell || id:ebi-a-GCST90001679 | rs11729256 | 4 | 95027272 | T | 0.0749798 | 0.0150177 | 6.73E-07 |
| ebi-a-GCST90016957 | Gut microbiota abundance (family Verrucomicrobiaceae id.4036) || id:ebi-a-GCST90016957 | CD19 on CD20- CD38- B cell || id:ebi-a-GCST90001722 | rs11729256 | 4 | 95027272 | T | 0.0749798 | 0.0150177 | 6.73E-07 |
| ebi-a-GCST90016957 | Gut microbiota abundance (family Verrucomicrobiaceae id.4036) || id:ebi-a-GCST90016957 | CD25 on B cell || id:ebi-a-GCST90001775 | rs11729256 | 4 | 95027272 | T | 0.0749798 | 0.0150177 | 6.73E-07 |
| ebi-a-GCST90016957 | Gut microbiota abundance (family Verrucomicrobiaceae id.4036) || id:ebi-a-GCST90016957 | CD25 on IgD+ CD24- B cell || id:ebi-a-GCST90001779 | rs11729256 | 4 | 95027272 | T | 0.0749798 | 0.0150177 | 6.73E-07 |
| ebi-a-GCST90016957 | Gut microbiota abundance (family Verrucomicrobiaceae id.4036) || id:ebi-a-GCST90016957 | CD25 on IgD+ CD38- naive B cell || id:ebi-a-GCST90001781 | rs11729256 | 4 | 95027272 | T | 0.0749798 | 0.0150177 | 6.73E-07 |
| ebi-a-GCST90016957 | Gut microbiota abundance (family Verrucomicrobiaceae id.4036) || id:ebi-a-GCST90016957 | CD25 on IgD+ CD38+ B cell || id:ebi-a-GCST90001783 | rs11729256 | 4 | 95027272 | T | 0.0749798 | 0.0150177 | 6.73E-07 |
| ebi-a-GCST90016957 | Gut microbiota abundance (family Verrucomicrobiaceae id.4036) || id:ebi-a-GCST90016957 | CD86 on myeloid Dendritic Cell || id:ebi-a-GCST90001903 | rs11729256 | 4 | 95027272 | T | 0.0749798 | 0.0150177 | 6.73E-07 |
| ebi-a-GCST90016957 | Gut microbiota abundance (family Verrucomicrobiaceae id.4036) || id:ebi-a-GCST90016957 | CD33 on CD14+ monocyte || id:ebi-a-GCST90001946 | rs11729256 | 4 | 95027272 | T | 0.0749798 | 0.0150177 | 6.73E-07 |
| ebi-a-GCST90016957 | Gut microbiota abundance (family Verrucomicrobiaceae id.4036) || id:ebi-a-GCST90016957 | CD33 on CD33+ HLA DR+ CD14dim || id:ebi-a-GCST90001947 | rs11729256 | 4 | 95027272 | T | 0.0749798 | 0.0150177 | 6.73E-07 |
| ebi-a-GCST90016957 | Gut microbiota abundance (family Verrucomicrobiaceae id.4036) || id:ebi-a-GCST90016957 | CD33 on CD33dim HLA DR+ CD11b+ || id:ebi-a-GCST90001948 | rs11729256 | 4 | 95027272 | T | 0.0749798 | 0.0150177 | 6.73E-07 |
| ebi-a-GCST90016957 | Gut microbiota abundance (family Verrucomicrobiaceae id.4036) || id:ebi-a-GCST90016957 | CD33 on Granulocytic Myeloid-Derived Suppressor Cells || id:ebi-a-GCST90001950 | rs11729256 | 4 | 95027272 | T | 0.0749798 | 0.0150177 | 6.73E-07 |
| ebi-a-GCST90016957 | Gut microbiota abundance (family Verrucomicrobiaceae id.4036) || id:ebi-a-GCST90016957 | CD33 on CD66b++ myeloid cell || id:ebi-a-GCST90001951 | rs11729256 | 4 | 95027272 | T | 0.0749798 | 0.0150177 | 6.73E-07 |
| ebi-a-GCST90016957 | Gut microbiota abundance (family Verrucomicrobiaceae id.4036) || id:ebi-a-GCST90016957 | CD33 on CD33dim HLA DR- || id:ebi-a-GCST90001953 | rs11729256 | 4 | 95027272 | T | 0.0749798 | 0.0150177 | 6.73E-07 |
| ebi-a-GCST90016957 | Gut microbiota abundance (family Verrucomicrobiaceae id.4036) || id:ebi-a-GCST90016957 | CD33 on basophil || id:ebi-a-GCST90001954 | rs11729256 | 4 | 95027272 | T | 0.0749798 | 0.0150177 | 6.73E-07 |
| ebi-a-GCST90016957 | Gut microbiota abundance (family Verrucomicrobiaceae id.4036) || id:ebi-a-GCST90016957 | CD33 on Immature Myeloid-Derived Suppressor Cells || id:ebi-a-GCST90001955 | rs11729256 | 4 | 95027272 | T | 0.0749798 | 0.0150177 | 6.73E-07 |
| ebi-a-GCST90016957 | Gut microbiota abundance (family Verrucomicrobiaceae id.4036) || id:ebi-a-GCST90016957 | FSC-A on HLA DR+ CD8+ T cell || id:ebi-a-GCST90001978 | rs11729256 | 4 | 95027272 | T | 0.0749798 | 0.0150177 | 6.73E-07 |
| ebi-a-GCST90016957 | Gut microbiota abundance (family Verrucomicrobiaceae id.4036) || id:ebi-a-GCST90016957 | HLA DR on CD14+ CD16- monocyte || id:ebi-a-GCST90001988 | rs11729256 | 4 | 95027272 | T | 0.0749798 | 0.0150177 | 6.73E-07 |
| ebi-a-GCST90016957 | Gut microbiota abundance (family Verrucomicrobiaceae id.4036) || id:ebi-a-GCST90016957 | HLA DR on CD14+ monocyte || id:ebi-a-GCST90001991 | rs11729256 | 4 | 95027272 | T | 0.0749798 | 0.0150177 | 6.73E-07 |
| ebi-a-GCST90016957 | Gut microbiota abundance (family Verrucomicrobiaceae id.4036) || id:ebi-a-GCST90016957 | CD16 on CD14+ CD16+ monocyte || id:ebi-a-GCST90002005 | rs11729256 | 4 | 95027272 | T | 0.0749798 | 0.0150177 | 6.73E-07 |
| ebi-a-GCST90016957 | Gut microbiota abundance (family Verrucomicrobiaceae id.4036) || id:ebi-a-GCST90016957 | CD45 on CD33+ HLA DR+ CD14- || id:ebi-a-GCST90002042 | rs11729256 | 4 | 95027272 | T | 0.0749798 | 0.0150177 | 6.73E-07 |
| ebi-a-GCST90016957 | Gut microbiota abundance (family Verrucomicrobiaceae id.4036) || id:ebi-a-GCST90016957 | CD8 on Natural Killer T || id:ebi-a-GCST90002059 | rs11729256 | 4 | 95027272 | T | 0.0749798 | 0.0150177 | 6.73E-07 |
| ebi-a-GCST90016923 | Gut microbiota abundance (class Verrucomicrobiae id.4029) || id:ebi-a-GCST90016923 | Myeloid Dendritic Cell Absolute Count || id:ebi-a-GCST90001458 | rs11729256 | 4 | 95027272 | T | 0.0749798 | 0.0150177 | 6.73E-07 |
| ebi-a-GCST90016923 | Gut microbiota abundance (class Verrucomicrobiae id.4029) || id:ebi-a-GCST90016923 | CD86+ myeloid Dendritic Cell %Dendritic Cell || id:ebi-a-GCST90001465 | rs11729256 | 4 | 95027272 | T | 0.0749798 | 0.0150177 | 6.73E-07 |
| ebi-a-GCST90016923 | Gut microbiota abundance (class Verrucomicrobiae id.4029) || id:ebi-a-GCST90016923 | CD62L- myeloid Dendritic Cell Absolute Count || id:ebi-a-GCST90001468 | rs11729256 | 4 | 95027272 | T | 0.0749798 | 0.0150177 | 6.73E-07 |
| ebi-a-GCST90016923 | Gut microbiota abundance (class Verrucomicrobiae id.4029) || id:ebi-a-GCST90016923 | CD62L- myeloid Dendritic Cell %Dendritic Cell || id:ebi-a-GCST90001469 | rs11729256 | 4 | 95027272 | T | 0.0749798 | 0.0150177 | 6.73E-07 |
| ebi-a-GCST90016923 | Gut microbiota abundance (class Verrucomicrobiae id.4029) || id:ebi-a-GCST90016923 | CD62L- CD86+ myeloid Dendritic Cell Absolute Count || id:ebi-a-GCST90001472 | rs11729256 | 4 | 95027272 | T | 0.0749798 | 0.0150177 | 6.73E-07 |
| ebi-a-GCST90016923 | Gut microbiota abundance (class Verrucomicrobiae id.4029) || id:ebi-a-GCST90016923 | CD62L- CD86+ myeloid Dendritic Cell %Dendritic Cell || id:ebi-a-GCST90001473 | rs11729256 | 4 | 95027272 | T | 0.0749798 | 0.0150177 | 6.73E-07 |
| ebi-a-GCST90016923 | Gut microbiota abundance (class Verrucomicrobiae id.4029) || id:ebi-a-GCST90016923 | HLA DR++ monocyte %monocyte || id:ebi-a-GCST90001475 | rs11729256 | 4 | 95027272 | T | 0.0749798 | 0.0150177 | 6.73E-07 |
| ebi-a-GCST90016923 | Gut microbiota abundance (class Verrucomicrobiae id.4029) || id:ebi-a-GCST90016923 | HLA DR++ monocyte Absolute Count || id:ebi-a-GCST90001477 | rs11729256 | 4 | 95027272 | T | 0.0749798 | 0.0150177 | 6.73E-07 |
| ebi-a-GCST90016923 | Gut microbiota abundance (class Verrucomicrobiae id.4029) || id:ebi-a-GCST90016923 | Basophil %CD33dim HLA DR- CD66b- || id:ebi-a-GCST90001533 | rs11729256 | 4 | 95027272 | T | 0.0749798 | 0.0150177 | 6.73E-07 |
| ebi-a-GCST90016923 | Gut microbiota abundance (class Verrucomicrobiae id.4029) || id:ebi-a-GCST90016923 | CD16+ monocyte %monocyte || id:ebi-a-GCST90001587 | rs11729256 | 4 | 95027272 | T | 0.0749798 | 0.0150177 | 6.73E-07 |
| ebi-a-GCST90016923 | Gut microbiota abundance (class Verrucomicrobiae id.4029) || id:ebi-a-GCST90016923 | T/B cell || id:ebi-a-GCST90001588 | rs11729256 | 4 | 95027272 | T | 0.0749798 | 0.0150177 | 6.73E-07 |
| ebi-a-GCST90016923 | Gut microbiota abundance (class Verrucomicrobiae id.4029) || id:ebi-a-GCST90016923 | CD4+CD8+ T cell %T cell || id:ebi-a-GCST90001595 | rs11729256 | 4 | 95027272 | T | 0.0749798 | 0.0150177 | 6.73E-07 |
| ebi-a-GCST90016923 | Gut microbiota abundance (class Verrucomicrobiae id.4029) || id:ebi-a-GCST90016923 | CD8+ Natural Killer T %T cell || id:ebi-a-GCST90001631 | rs11729256 | 4 | 95027272 | T | 0.0749798 | 0.0150177 | 6.73E-07 |
| ebi-a-GCST90016923 | Gut microbiota abundance (class Verrucomicrobiae id.4029) || id:ebi-a-GCST90016923 | CD4-CD8- Natural Killer T %lymphocyte || id:ebi-a-GCST90001638 | rs11729256 | 4 | 95027272 | T | 0.0749798 | 0.0150177 | 6.73E-07 |
| ebi-a-GCST90016923 | Gut microbiota abundance (class Verrucomicrobiae id.4029) || id:ebi-a-GCST90016923 | HLA DR+ Natural Killer Absolute Count || id:ebi-a-GCST90001648 | rs11729256 | 4 | 95027272 | T | 0.0749798 | 0.0150177 | 6.73E-07 |
| ebi-a-GCST90016923 | Gut microbiota abundance (class Verrucomicrobiae id.4029) || id:ebi-a-GCST90016923 | HLA DR+ Natural Killer %Natural Killer || id:ebi-a-GCST90001649 | rs11729256 | 4 | 95027272 | T | 0.0749798 | 0.0150177 | 6.73E-07 |
| ebi-a-GCST90016923 | Gut microbiota abundance (class Verrucomicrobiae id.4029) || id:ebi-a-GCST90016923 | HLA DR+ Natural Killer %CD3- lymphocyte || id:ebi-a-GCST90001650 | rs11729256 | 4 | 95027272 | T | 0.0749798 | 0.0150177 | 6.73E-07 |
| ebi-a-GCST90016923 | Gut microbiota abundance (class Verrucomicrobiae id.4029) || id:ebi-a-GCST90016923 | CD28- CD25++ CD8+ T cell Absolute Count || id:ebi-a-GCST90001678 | rs11729256 | 4 | 95027272 | T | 0.0749798 | 0.0150177 | 6.73E-07 |
| ebi-a-GCST90016923 | Gut microbiota abundance (class Verrucomicrobiae id.4029) || id:ebi-a-GCST90016923 | CD25++ CD8+ T cell %T cell || id:ebi-a-GCST90001679 | rs11729256 | 4 | 95027272 | T | 0.0749798 | 0.0150177 | 6.73E-07 |
| ebi-a-GCST90016923 | Gut microbiota abundance (class Verrucomicrobiae id.4029) || id:ebi-a-GCST90016923 | CD19 on CD20- CD38- B cell || id:ebi-a-GCST90001722 | rs11729256 | 4 | 95027272 | T | 0.0749798 | 0.0150177 | 6.73E-07 |
| ebi-a-GCST90016923 | Gut microbiota abundance (class Verrucomicrobiae id.4029) || id:ebi-a-GCST90016923 | CD25 on B cell || id:ebi-a-GCST90001775 | rs11729256 | 4 | 95027272 | T | 0.0749798 | 0.0150177 | 6.73E-07 |
| ebi-a-GCST90016923 | Gut microbiota abundance (class Verrucomicrobiae id.4029) || id:ebi-a-GCST90016923 | CD25 on IgD+ CD24- B cell || id:ebi-a-GCST90001779 | rs11729256 | 4 | 95027272 | T | 0.0749798 | 0.0150177 | 6.73E-07 |
| ebi-a-GCST90016923 | Gut microbiota abundance (class Verrucomicrobiae id.4029) || id:ebi-a-GCST90016923 | CD25 on IgD+ CD38- naive B cell || id:ebi-a-GCST90001781 | rs11729256 | 4 | 95027272 | T | 0.0749798 | 0.0150177 | 6.73E-07 |
| ebi-a-GCST90016923 | Gut microbiota abundance (class Verrucomicrobiae id.4029) || id:ebi-a-GCST90016923 | CD25 on IgD+ CD38+ B cell || id:ebi-a-GCST90001783 | rs11729256 | 4 | 95027272 | T | 0.0749798 | 0.0150177 | 6.73E-07 |
| ebi-a-GCST90016923 | Gut microbiota abundance (class Verrucomicrobiae id.4029) || id:ebi-a-GCST90016923 | CD86 on myeloid Dendritic Cell || id:ebi-a-GCST90001903 | rs11729256 | 4 | 95027272 | T | 0.0749798 | 0.0150177 | 6.73E-07 |
| ebi-a-GCST90016923 | Gut microbiota abundance (class Verrucomicrobiae id.4029) || id:ebi-a-GCST90016923 | CD33 on CD14+ monocyte || id:ebi-a-GCST90001946 | rs11729256 | 4 | 95027272 | T | 0.0749798 | 0.0150177 | 6.73E-07 |
| ebi-a-GCST90016923 | Gut microbiota abundance (class Verrucomicrobiae id.4029) || id:ebi-a-GCST90016923 | CD33 on CD33+ HLA DR+ CD14dim || id:ebi-a-GCST90001947 | rs11729256 | 4 | 95027272 | T | 0.0749798 | 0.0150177 | 6.73E-07 |
| ebi-a-GCST90016923 | Gut microbiota abundance (class Verrucomicrobiae id.4029) || id:ebi-a-GCST90016923 | CD33 on CD33dim HLA DR+ CD11b+ || id:ebi-a-GCST90001948 | rs11729256 | 4 | 95027272 | T | 0.0749798 | 0.0150177 | 6.73E-07 |
| ebi-a-GCST90016923 | Gut microbiota abundance (class Verrucomicrobiae id.4029) || id:ebi-a-GCST90016923 | CD33 on Granulocytic Myeloid-Derived Suppressor Cells || id:ebi-a-GCST90001950 | rs11729256 | 4 | 95027272 | T | 0.0749798 | 0.0150177 | 6.73E-07 |
| ebi-a-GCST90016923 | Gut microbiota abundance (class Verrucomicrobiae id.4029) || id:ebi-a-GCST90016923 | CD33 on CD66b++ myeloid cell || id:ebi-a-GCST90001951 | rs11729256 | 4 | 95027272 | T | 0.0749798 | 0.0150177 | 6.73E-07 |
| ebi-a-GCST90016923 | Gut microbiota abundance (class Verrucomicrobiae id.4029) || id:ebi-a-GCST90016923 | CD33 on CD33dim HLA DR- || id:ebi-a-GCST90001953 | rs11729256 | 4 | 95027272 | T | 0.0749798 | 0.0150177 | 6.73E-07 |
| ebi-a-GCST90016923 | Gut microbiota abundance (class Verrucomicrobiae id.4029) || id:ebi-a-GCST90016923 | CD33 on basophil || id:ebi-a-GCST90001954 | rs11729256 | 4 | 95027272 | T | 0.0749798 | 0.0150177 | 6.73E-07 |
| ebi-a-GCST90016923 | Gut microbiota abundance (class Verrucomicrobiae id.4029) || id:ebi-a-GCST90016923 | CD33 on Immature Myeloid-Derived Suppressor Cells || id:ebi-a-GCST90001955 | rs11729256 | 4 | 95027272 | T | 0.0749798 | 0.0150177 | 6.73E-07 |
| ebi-a-GCST90016923 | Gut microbiota abundance (class Verrucomicrobiae id.4029) || id:ebi-a-GCST90016923 | FSC-A on HLA DR+ CD8+ T cell || id:ebi-a-GCST90001978 | rs11729256 | 4 | 95027272 | T | 0.0749798 | 0.0150177 | 6.73E-07 |
| ebi-a-GCST90016923 | Gut microbiota abundance (class Verrucomicrobiae id.4029) || id:ebi-a-GCST90016923 | HLA DR on CD14+ CD16- monocyte || id:ebi-a-GCST90001988 | rs11729256 | 4 | 95027272 | T | 0.0749798 | 0.0150177 | 6.73E-07 |
| ebi-a-GCST90016923 | Gut microbiota abundance (class Verrucomicrobiae id.4029) || id:ebi-a-GCST90016923 | HLA DR on CD14+ monocyte || id:ebi-a-GCST90001991 | rs11729256 | 4 | 95027272 | T | 0.0749798 | 0.0150177 | 6.73E-07 |
| ebi-a-GCST90016923 | Gut microbiota abundance (class Verrucomicrobiae id.4029) || id:ebi-a-GCST90016923 | CD16 on CD14+ CD16+ monocyte || id:ebi-a-GCST90002005 | rs11729256 | 4 | 95027272 | T | 0.0749798 | 0.0150177 | 6.73E-07 |
| ebi-a-GCST90016923 | Gut microbiota abundance (class Verrucomicrobiae id.4029) || id:ebi-a-GCST90016923 | CD45 on CD33+ HLA DR+ CD14- || id:ebi-a-GCST90002042 | rs11729256 | 4 | 95027272 | T | 0.0749798 | 0.0150177 | 6.73E-07 |
| ebi-a-GCST90016923 | Gut microbiota abundance (class Verrucomicrobiae id.4029) || id:ebi-a-GCST90016923 | CD8 on Natural Killer T || id:ebi-a-GCST90002059 | rs11729256 | 4 | 95027272 | T | 0.0749798 | 0.0150177 | 6.73E-07 |
| ebi-a-GCST90017108 | Gut microbiota abundance (order Verrucomicrobiales id.4030) || id:ebi-a-GCST90017108 | Myeloid Dendritic Cell Absolute Count || id:ebi-a-GCST90001458 | rs11729256 | 4 | 95027272 | T | 0.0749798 | 0.0150177 | 6.73E-07 |
| ebi-a-GCST90017108 | Gut microbiota abundance (order Verrucomicrobiales id.4030) || id:ebi-a-GCST90017108 | CD86+ myeloid Dendritic Cell %Dendritic Cell || id:ebi-a-GCST90001465 | rs11729256 | 4 | 95027272 | T | 0.0749798 | 0.0150177 | 6.73E-07 |
| ebi-a-GCST90017108 | Gut microbiota abundance (order Verrucomicrobiales id.4030) || id:ebi-a-GCST90017108 | CD62L- myeloid Dendritic Cell Absolute Count || id:ebi-a-GCST90001468 | rs11729256 | 4 | 95027272 | T | 0.0749798 | 0.0150177 | 6.73E-07 |
| ebi-a-GCST90017108 | Gut microbiota abundance (order Verrucomicrobiales id.4030) || id:ebi-a-GCST90017108 | CD62L- myeloid Dendritic Cell %Dendritic Cell || id:ebi-a-GCST90001469 | rs11729256 | 4 | 95027272 | T | 0.0749798 | 0.0150177 | 6.73E-07 |
| ebi-a-GCST90017108 | Gut microbiota abundance (order Verrucomicrobiales id.4030) || id:ebi-a-GCST90017108 | CD62L- CD86+ myeloid Dendritic Cell Absolute Count || id:ebi-a-GCST90001472 | rs11729256 | 4 | 95027272 | T | 0.0749798 | 0.0150177 | 6.73E-07 |
| ebi-a-GCST90017108 | Gut microbiota abundance (order Verrucomicrobiales id.4030) || id:ebi-a-GCST90017108 | CD62L- CD86+ myeloid Dendritic Cell %Dendritic Cell || id:ebi-a-GCST90001473 | rs11729256 | 4 | 95027272 | T | 0.0749798 | 0.0150177 | 6.73E-07 |
| ebi-a-GCST90017108 | Gut microbiota abundance (order Verrucomicrobiales id.4030) || id:ebi-a-GCST90017108 | HLA DR++ monocyte %monocyte || id:ebi-a-GCST90001475 | rs11729256 | 4 | 95027272 | T | 0.0749798 | 0.0150177 | 6.73E-07 |
| ebi-a-GCST90017108 | Gut microbiota abundance (order Verrucomicrobiales id.4030) || id:ebi-a-GCST90017108 | HLA DR++ monocyte Absolute Count || id:ebi-a-GCST90001477 | rs11729256 | 4 | 95027272 | T | 0.0749798 | 0.0150177 | 6.73E-07 |
| ebi-a-GCST90017108 | Gut microbiota abundance (order Verrucomicrobiales id.4030) || id:ebi-a-GCST90017108 | Basophil %CD33dim HLA DR- CD66b- || id:ebi-a-GCST90001533 | rs11729256 | 4 | 95027272 | T | 0.0749798 | 0.0150177 | 6.73E-07 |
| ebi-a-GCST90017108 | Gut microbiota abundance (order Verrucomicrobiales id.4030) || id:ebi-a-GCST90017108 | CD16+ monocyte %monocyte || id:ebi-a-GCST90001587 | rs11729256 | 4 | 95027272 | T | 0.0749798 | 0.0150177 | 6.73E-07 |
| ebi-a-GCST90017108 | Gut microbiota abundance (order Verrucomicrobiales id.4030) || id:ebi-a-GCST90017108 | T/B cell || id:ebi-a-GCST90001588 | rs11729256 | 4 | 95027272 | T | 0.0749798 | 0.0150177 | 6.73E-07 |
| ebi-a-GCST90017108 | Gut microbiota abundance (order Verrucomicrobiales id.4030) || id:ebi-a-GCST90017108 | CD4+CD8+ T cell %T cell || id:ebi-a-GCST90001595 | rs11729256 | 4 | 95027272 | T | 0.0749798 | 0.0150177 | 6.73E-07 |
| ebi-a-GCST90017108 | Gut microbiota abundance (order Verrucomicrobiales id.4030) || id:ebi-a-GCST90017108 | CD8+ Natural Killer T %T cell || id:ebi-a-GCST90001631 | rs11729256 | 4 | 95027272 | T | 0.0749798 | 0.0150177 | 6.73E-07 |
| ebi-a-GCST90017108 | Gut microbiota abundance (order Verrucomicrobiales id.4030) || id:ebi-a-GCST90017108 | CD4-CD8- Natural Killer T %lymphocyte || id:ebi-a-GCST90001638 | rs11729256 | 4 | 95027272 | T | 0.0749798 | 0.0150177 | 6.73E-07 |
| ebi-a-GCST90017108 | Gut microbiota abundance (order Verrucomicrobiales id.4030) || id:ebi-a-GCST90017108 | HLA DR+ Natural Killer Absolute Count || id:ebi-a-GCST90001648 | rs11729256 | 4 | 95027272 | T | 0.0749798 | 0.0150177 | 6.73E-07 |
| ebi-a-GCST90017108 | Gut microbiota abundance (order Verrucomicrobiales id.4030) || id:ebi-a-GCST90017108 | HLA DR+ Natural Killer %Natural Killer || id:ebi-a-GCST90001649 | rs11729256 | 4 | 95027272 | T | 0.0749798 | 0.0150177 | 6.73E-07 |
| ebi-a-GCST90017108 | Gut microbiota abundance (order Verrucomicrobiales id.4030) || id:ebi-a-GCST90017108 | HLA DR+ Natural Killer %CD3- lymphocyte || id:ebi-a-GCST90001650 | rs11729256 | 4 | 95027272 | T | 0.0749798 | 0.0150177 | 6.73E-07 |
| ebi-a-GCST90017108 | Gut microbiota abundance (order Verrucomicrobiales id.4030) || id:ebi-a-GCST90017108 | CD28- CD25++ CD8+ T cell Absolute Count || id:ebi-a-GCST90001678 | rs11729256 | 4 | 95027272 | T | 0.0749798 | 0.0150177 | 6.73E-07 |
| ebi-a-GCST90017108 | Gut microbiota abundance (order Verrucomicrobiales id.4030) || id:ebi-a-GCST90017108 | CD25++ CD8+ T cell %T cell || id:ebi-a-GCST90001679 | rs11729256 | 4 | 95027272 | T | 0.0749798 | 0.0150177 | 6.73E-07 |
| ebi-a-GCST90017108 | Gut microbiota abundance (order Verrucomicrobiales id.4030) || id:ebi-a-GCST90017108 | CD19 on CD20- CD38- B cell || id:ebi-a-GCST90001722 | rs11729256 | 4 | 95027272 | T | 0.0749798 | 0.0150177 | 6.73E-07 |
| ebi-a-GCST90017108 | Gut microbiota abundance (order Verrucomicrobiales id.4030) || id:ebi-a-GCST90017108 | CD25 on B cell || id:ebi-a-GCST90001775 | rs11729256 | 4 | 95027272 | T | 0.0749798 | 0.0150177 | 6.73E-07 |
| ebi-a-GCST90017108 | Gut microbiota abundance (order Verrucomicrobiales id.4030) || id:ebi-a-GCST90017108 | CD25 on IgD+ CD24- B cell || id:ebi-a-GCST90001779 | rs11729256 | 4 | 95027272 | T | 0.0749798 | 0.0150177 | 6.73E-07 |
| ebi-a-GCST90017108 | Gut microbiota abundance (order Verrucomicrobiales id.4030) || id:ebi-a-GCST90017108 | CD25 on IgD+ CD38- naive B cell || id:ebi-a-GCST90001781 | rs11729256 | 4 | 95027272 | T | 0.0749798 | 0.0150177 | 6.73E-07 |
| ebi-a-GCST90017108 | Gut microbiota abundance (order Verrucomicrobiales id.4030) || id:ebi-a-GCST90017108 | CD25 on IgD+ CD38+ B cell || id:ebi-a-GCST90001783 | rs11729256 | 4 | 95027272 | T | 0.0749798 | 0.0150177 | 6.73E-07 |
| ebi-a-GCST90017108 | Gut microbiota abundance (order Verrucomicrobiales id.4030) || id:ebi-a-GCST90017108 | CD86 on myeloid Dendritic Cell || id:ebi-a-GCST90001903 | rs11729256 | 4 | 95027272 | T | 0.0749798 | 0.0150177 | 6.73E-07 |
| ebi-a-GCST90017108 | Gut microbiota abundance (order Verrucomicrobiales id.4030) || id:ebi-a-GCST90017108 | CD33 on CD14+ monocyte || id:ebi-a-GCST90001946 | rs11729256 | 4 | 95027272 | T | 0.0749798 | 0.0150177 | 6.73E-07 |
| ebi-a-GCST90017108 | Gut microbiota abundance (order Verrucomicrobiales id.4030) || id:ebi-a-GCST90017108 | CD33 on CD33+ HLA DR+ CD14dim || id:ebi-a-GCST90001947 | rs11729256 | 4 | 95027272 | T | 0.0749798 | 0.0150177 | 6.73E-07 |
| ebi-a-GCST90017108 | Gut microbiota abundance (order Verrucomicrobiales id.4030) || id:ebi-a-GCST90017108 | CD33 on CD33dim HLA DR+ CD11b+ || id:ebi-a-GCST90001948 | rs11729256 | 4 | 95027272 | T | 0.0749798 | 0.0150177 | 6.73E-07 |
| ebi-a-GCST90017108 | Gut microbiota abundance (order Verrucomicrobiales id.4030) || id:ebi-a-GCST90017108 | CD33 on Granulocytic Myeloid-Derived Suppressor Cells || id:ebi-a-GCST90001950 | rs11729256 | 4 | 95027272 | T | 0.0749798 | 0.0150177 | 6.73E-07 |
| ebi-a-GCST90017108 | Gut microbiota abundance (order Verrucomicrobiales id.4030) || id:ebi-a-GCST90017108 | CD33 on CD66b++ myeloid cell || id:ebi-a-GCST90001951 | rs11729256 | 4 | 95027272 | T | 0.0749798 | 0.0150177 | 6.73E-07 |
| ebi-a-GCST90017108 | Gut microbiota abundance (order Verrucomicrobiales id.4030) || id:ebi-a-GCST90017108 | CD33 on CD33dim HLA DR- || id:ebi-a-GCST90001953 | rs11729256 | 4 | 95027272 | T | 0.0749798 | 0.0150177 | 6.73E-07 |
| ebi-a-GCST90017108 | Gut microbiota abundance (order Verrucomicrobiales id.4030) || id:ebi-a-GCST90017108 | CD33 on basophil || id:ebi-a-GCST90001954 | rs11729256 | 4 | 95027272 | T | 0.0749798 | 0.0150177 | 6.73E-07 |
| ebi-a-GCST90017108 | Gut microbiota abundance (order Verrucomicrobiales id.4030) || id:ebi-a-GCST90017108 | CD33 on Immature Myeloid-Derived Suppressor Cells || id:ebi-a-GCST90001955 | rs11729256 | 4 | 95027272 | T | 0.0749798 | 0.0150177 | 6.73E-07 |
| ebi-a-GCST90017108 | Gut microbiota abundance (order Verrucomicrobiales id.4030) || id:ebi-a-GCST90017108 | FSC-A on HLA DR+ CD8+ T cell || id:ebi-a-GCST90001978 | rs11729256 | 4 | 95027272 | T | 0.0749798 | 0.0150177 | 6.73E-07 |
| ebi-a-GCST90017108 | Gut microbiota abundance (order Verrucomicrobiales id.4030) || id:ebi-a-GCST90017108 | HLA DR on CD14+ CD16- monocyte || id:ebi-a-GCST90001988 | rs11729256 | 4 | 95027272 | T | 0.0749798 | 0.0150177 | 6.73E-07 |
| ebi-a-GCST90017108 | Gut microbiota abundance (order Verrucomicrobiales id.4030) || id:ebi-a-GCST90017108 | HLA DR on CD14+ monocyte || id:ebi-a-GCST90001991 | rs11729256 | 4 | 95027272 | T | 0.0749798 | 0.0150177 | 6.73E-07 |
| ebi-a-GCST90017108 | Gut microbiota abundance (order Verrucomicrobiales id.4030) || id:ebi-a-GCST90017108 | CD16 on CD14+ CD16+ monocyte || id:ebi-a-GCST90002005 | rs11729256 | 4 | 95027272 | T | 0.0749798 | 0.0150177 | 6.73E-07 |
| ebi-a-GCST90017108 | Gut microbiota abundance (order Verrucomicrobiales id.4030) || id:ebi-a-GCST90017108 | CD45 on CD33+ HLA DR+ CD14- || id:ebi-a-GCST90002042 | rs11729256 | 4 | 95027272 | T | 0.0749798 | 0.0150177 | 6.73E-07 |
| ebi-a-GCST90017108 | Gut microbiota abundance (order Verrucomicrobiales id.4030) || id:ebi-a-GCST90017108 | CD8 on Natural Killer T || id:ebi-a-GCST90002059 | rs11729256 | 4 | 95027272 | T | 0.0749798 | 0.0150177 | 6.73E-07 |
| ebi-a-GCST90016961 | Gut microbiota abundance (genus Akkermansia id.4037) || id:ebi-a-GCST90016961 | Myeloid Dendritic Cell Absolute Count || id:ebi-a-GCST90001458 | rs11729256 | 4 | 95027272 | T | 0.0750473 | 0.0150184 | 6.58E-07 |
| ebi-a-GCST90016961 | Gut microbiota abundance (genus Akkermansia id.4037) || id:ebi-a-GCST90016961 | CD86+ myeloid Dendritic Cell %Dendritic Cell || id:ebi-a-GCST90001465 | rs11729256 | 4 | 95027272 | T | 0.0750473 | 0.0150184 | 6.58E-07 |
| ebi-a-GCST90016961 | Gut microbiota abundance (genus Akkermansia id.4037) || id:ebi-a-GCST90016961 | CD62L- myeloid Dendritic Cell Absolute Count || id:ebi-a-GCST90001468 | rs11729256 | 4 | 95027272 | T | 0.0750473 | 0.0150184 | 6.58E-07 |
| ebi-a-GCST90016961 | Gut microbiota abundance (genus Akkermansia id.4037) || id:ebi-a-GCST90016961 | CD62L- myeloid Dendritic Cell %Dendritic Cell || id:ebi-a-GCST90001469 | rs11729256 | 4 | 95027272 | T | 0.0750473 | 0.0150184 | 6.58E-07 |
| ebi-a-GCST90016961 | Gut microbiota abundance (genus Akkermansia id.4037) || id:ebi-a-GCST90016961 | CD62L- CD86+ myeloid Dendritic Cell Absolute Count || id:ebi-a-GCST90001472 | rs11729256 | 4 | 95027272 | T | 0.0750473 | 0.0150184 | 6.58E-07 |
| ebi-a-GCST90016961 | Gut microbiota abundance (genus Akkermansia id.4037) || id:ebi-a-GCST90016961 | CD62L- CD86+ myeloid Dendritic Cell %Dendritic Cell || id:ebi-a-GCST90001473 | rs11729256 | 4 | 95027272 | T | 0.0750473 | 0.0150184 | 6.58E-07 |
| ebi-a-GCST90016961 | Gut microbiota abundance (genus Akkermansia id.4037) || id:ebi-a-GCST90016961 | HLA DR++ monocyte %monocyte || id:ebi-a-GCST90001475 | rs11729256 | 4 | 95027272 | T | 0.0750473 | 0.0150184 | 6.58E-07 |
| ebi-a-GCST90016961 | Gut microbiota abundance (genus Akkermansia id.4037) || id:ebi-a-GCST90016961 | HLA DR++ monocyte Absolute Count || id:ebi-a-GCST90001477 | rs11729256 | 4 | 95027272 | T | 0.0750473 | 0.0150184 | 6.58E-07 |
| ebi-a-GCST90016961 | Gut microbiota abundance (genus Akkermansia id.4037) || id:ebi-a-GCST90016961 | Basophil %CD33dim HLA DR- CD66b- || id:ebi-a-GCST90001533 | rs11729256 | 4 | 95027272 | T | 0.0750473 | 0.0150184 | 6.58E-07 |
| ebi-a-GCST90016961 | Gut microbiota abundance (genus Akkermansia id.4037) || id:ebi-a-GCST90016961 | CD16+ monocyte %monocyte || id:ebi-a-GCST90001587 | rs11729256 | 4 | 95027272 | T | 0.0750473 | 0.0150184 | 6.58E-07 |
| ebi-a-GCST90016961 | Gut microbiota abundance (genus Akkermansia id.4037) || id:ebi-a-GCST90016961 | T/B cell || id:ebi-a-GCST90001588 | rs11729256 | 4 | 95027272 | T | 0.0750473 | 0.0150184 | 6.58E-07 |
| ebi-a-GCST90016961 | Gut microbiota abundance (genus Akkermansia id.4037) || id:ebi-a-GCST90016961 | CD4+CD8+ T cell %T cell || id:ebi-a-GCST90001595 | rs11729256 | 4 | 95027272 | T | 0.0750473 | 0.0150184 | 6.58E-07 |
| ebi-a-GCST90016961 | Gut microbiota abundance (genus Akkermansia id.4037) || id:ebi-a-GCST90016961 | CD8+ Natural Killer T %T cell || id:ebi-a-GCST90001631 | rs11729256 | 4 | 95027272 | T | 0.0750473 | 0.0150184 | 6.58E-07 |
| ebi-a-GCST90016961 | Gut microbiota abundance (genus Akkermansia id.4037) || id:ebi-a-GCST90016961 | CD4-CD8- Natural Killer T %lymphocyte || id:ebi-a-GCST90001638 | rs11729256 | 4 | 95027272 | T | 0.0750473 | 0.0150184 | 6.58E-07 |
| ebi-a-GCST90016961 | Gut microbiota abundance (genus Akkermansia id.4037) || id:ebi-a-GCST90016961 | HLA DR+ Natural Killer Absolute Count || id:ebi-a-GCST90001648 | rs11729256 | 4 | 95027272 | T | 0.0750473 | 0.0150184 | 6.58E-07 |
| ebi-a-GCST90016961 | Gut microbiota abundance (genus Akkermansia id.4037) || id:ebi-a-GCST90016961 | HLA DR+ Natural Killer %Natural Killer || id:ebi-a-GCST90001649 | rs11729256 | 4 | 95027272 | T | 0.0750473 | 0.0150184 | 6.58E-07 |
| ebi-a-GCST90016961 | Gut microbiota abundance (genus Akkermansia id.4037) || id:ebi-a-GCST90016961 | HLA DR+ Natural Killer %CD3- lymphocyte || id:ebi-a-GCST90001650 | rs11729256 | 4 | 95027272 | T | 0.0750473 | 0.0150184 | 6.58E-07 |
| ebi-a-GCST90016961 | Gut microbiota abundance (genus Akkermansia id.4037) || id:ebi-a-GCST90016961 | CD28- CD25++ CD8+ T cell Absolute Count || id:ebi-a-GCST90001678 | rs11729256 | 4 | 95027272 | T | 0.0750473 | 0.0150184 | 6.58E-07 |
| ebi-a-GCST90016961 | Gut microbiota abundance (genus Akkermansia id.4037) || id:ebi-a-GCST90016961 | CD25++ CD8+ T cell %T cell || id:ebi-a-GCST90001679 | rs11729256 | 4 | 95027272 | T | 0.0750473 | 0.0150184 | 6.58E-07 |
| ebi-a-GCST90016961 | Gut microbiota abundance (genus Akkermansia id.4037) || id:ebi-a-GCST90016961 | CD19 on CD20- CD38- B cell || id:ebi-a-GCST90001722 | rs11729256 | 4 | 95027272 | T | 0.0750473 | 0.0150184 | 6.58E-07 |
| ebi-a-GCST90016961 | Gut microbiota abundance (genus Akkermansia id.4037) || id:ebi-a-GCST90016961 | CD25 on B cell || id:ebi-a-GCST90001775 | rs11729256 | 4 | 95027272 | T | 0.0750473 | 0.0150184 | 6.58E-07 |
| ebi-a-GCST90016961 | Gut microbiota abundance (genus Akkermansia id.4037) || id:ebi-a-GCST90016961 | CD25 on IgD+ CD24- B cell || id:ebi-a-GCST90001779 | rs11729256 | 4 | 95027272 | T | 0.0750473 | 0.0150184 | 6.58E-07 |
| ebi-a-GCST90016961 | Gut microbiota abundance (genus Akkermansia id.4037) || id:ebi-a-GCST90016961 | CD25 on IgD+ CD38- naive B cell || id:ebi-a-GCST90001781 | rs11729256 | 4 | 95027272 | T | 0.0750473 | 0.0150184 | 6.58E-07 |
| ebi-a-GCST90016961 | Gut microbiota abundance (genus Akkermansia id.4037) || id:ebi-a-GCST90016961 | CD25 on IgD+ CD38+ B cell || id:ebi-a-GCST90001783 | rs11729256 | 4 | 95027272 | T | 0.0750473 | 0.0150184 | 6.58E-07 |
| ebi-a-GCST90016961 | Gut microbiota abundance (genus Akkermansia id.4037) || id:ebi-a-GCST90016961 | CD86 on myeloid Dendritic Cell || id:ebi-a-GCST90001903 | rs11729256 | 4 | 95027272 | T | 0.0750473 | 0.0150184 | 6.58E-07 |
| ebi-a-GCST90016961 | Gut microbiota abundance (genus Akkermansia id.4037) || id:ebi-a-GCST90016961 | CD33 on CD14+ monocyte || id:ebi-a-GCST90001946 | rs11729256 | 4 | 95027272 | T | 0.0750473 | 0.0150184 | 6.58E-07 |
| ebi-a-GCST90016961 | Gut microbiota abundance (genus Akkermansia id.4037) || id:ebi-a-GCST90016961 | CD33 on CD33+ HLA DR+ CD14dim || id:ebi-a-GCST90001947 | rs11729256 | 4 | 95027272 | T | 0.0750473 | 0.0150184 | 6.58E-07 |
| ebi-a-GCST90016961 | Gut microbiota abundance (genus Akkermansia id.4037) || id:ebi-a-GCST90016961 | CD33 on CD33dim HLA DR+ CD11b+ || id:ebi-a-GCST90001948 | rs11729256 | 4 | 95027272 | T | 0.0750473 | 0.0150184 | 6.58E-07 |
| ebi-a-GCST90016961 | Gut microbiota abundance (genus Akkermansia id.4037) || id:ebi-a-GCST90016961 | CD33 on Granulocytic Myeloid-Derived Suppressor Cells || id:ebi-a-GCST90001950 | rs11729256 | 4 | 95027272 | T | 0.0750473 | 0.0150184 | 6.58E-07 |
| ebi-a-GCST90016961 | Gut microbiota abundance (genus Akkermansia id.4037) || id:ebi-a-GCST90016961 | CD33 on CD66b++ myeloid cell || id:ebi-a-GCST90001951 | rs11729256 | 4 | 95027272 | T | 0.0750473 | 0.0150184 | 6.58E-07 |
| ebi-a-GCST90016961 | Gut microbiota abundance (genus Akkermansia id.4037) || id:ebi-a-GCST90016961 | CD33 on CD33dim HLA DR- || id:ebi-a-GCST90001953 | rs11729256 | 4 | 95027272 | T | 0.0750473 | 0.0150184 | 6.58E-07 |
| ebi-a-GCST90016961 | Gut microbiota abundance (genus Akkermansia id.4037) || id:ebi-a-GCST90016961 | CD33 on basophil || id:ebi-a-GCST90001954 | rs11729256 | 4 | 95027272 | T | 0.0750473 | 0.0150184 | 6.58E-07 |
| ebi-a-GCST90016961 | Gut microbiota abundance (genus Akkermansia id.4037) || id:ebi-a-GCST90016961 | CD33 on Immature Myeloid-Derived Suppressor Cells || id:ebi-a-GCST90001955 | rs11729256 | 4 | 95027272 | T | 0.0750473 | 0.0150184 | 6.58E-07 |
| ebi-a-GCST90016961 | Gut microbiota abundance (genus Akkermansia id.4037) || id:ebi-a-GCST90016961 | FSC-A on HLA DR+ CD8+ T cell || id:ebi-a-GCST90001978 | rs11729256 | 4 | 95027272 | T | 0.0750473 | 0.0150184 | 6.58E-07 |
| ebi-a-GCST90016961 | Gut microbiota abundance (genus Akkermansia id.4037) || id:ebi-a-GCST90016961 | HLA DR on CD14+ CD16- monocyte || id:ebi-a-GCST90001988 | rs11729256 | 4 | 95027272 | T | 0.0750473 | 0.0150184 | 6.58E-07 |
| ebi-a-GCST90016961 | Gut microbiota abundance (genus Akkermansia id.4037) || id:ebi-a-GCST90016961 | HLA DR on CD14+ monocyte || id:ebi-a-GCST90001991 | rs11729256 | 4 | 95027272 | T | 0.0750473 | 0.0150184 | 6.58E-07 |
| ebi-a-GCST90016961 | Gut microbiota abundance (genus Akkermansia id.4037) || id:ebi-a-GCST90016961 | CD16 on CD14+ CD16+ monocyte || id:ebi-a-GCST90002005 | rs11729256 | 4 | 95027272 | T | 0.0750473 | 0.0150184 | 6.58E-07 |
| ebi-a-GCST90016961 | Gut microbiota abundance (genus Akkermansia id.4037) || id:ebi-a-GCST90016961 | CD45 on CD33+ HLA DR+ CD14- || id:ebi-a-GCST90002042 | rs11729256 | 4 | 95027272 | T | 0.0750473 | 0.0150184 | 6.58E-07 |
| ebi-a-GCST90016961 | Gut microbiota abundance (genus Akkermansia id.4037) || id:ebi-a-GCST90016961 | CD8 on Natural Killer T || id:ebi-a-GCST90002059 | rs11729256 | 4 | 95027272 | T | 0.0750473 | 0.0150184 | 6.58E-07 |
| ebi-a-GCST90016921 | Gut microbiota abundance (class Mollicutes id.3920) || id:ebi-a-GCST90016921 | Myeloid Dendritic Cell Absolute Count || id:ebi-a-GCST90001458 | rs11890098 | 2 | 157532549 | A | 0.074438 | 0.0153389 | 9.57E-07 |
| ebi-a-GCST90016921 | Gut microbiota abundance (class Mollicutes id.3920) || id:ebi-a-GCST90016921 | CD86+ myeloid Dendritic Cell %Dendritic Cell || id:ebi-a-GCST90001465 | rs11890098 | 2 | 157532549 | A | 0.074438 | 0.0153389 | 9.57E-07 |
| ebi-a-GCST90016921 | Gut microbiota abundance (class Mollicutes id.3920) || id:ebi-a-GCST90016921 | CD62L- myeloid Dendritic Cell Absolute Count || id:ebi-a-GCST90001468 | rs11890098 | 2 | 157532549 | A | 0.074438 | 0.0153389 | 9.57E-07 |
| ebi-a-GCST90016921 | Gut microbiota abundance (class Mollicutes id.3920) || id:ebi-a-GCST90016921 | CD62L- myeloid Dendritic Cell %Dendritic Cell || id:ebi-a-GCST90001469 | rs11890098 | 2 | 157532549 | A | 0.074438 | 0.0153389 | 9.57E-07 |
| ebi-a-GCST90016921 | Gut microbiota abundance (class Mollicutes id.3920) || id:ebi-a-GCST90016921 | CD62L- CD86+ myeloid Dendritic Cell Absolute Count || id:ebi-a-GCST90001472 | rs11890098 | 2 | 157532549 | A | 0.074438 | 0.0153389 | 9.57E-07 |
| ebi-a-GCST90016921 | Gut microbiota abundance (class Mollicutes id.3920) || id:ebi-a-GCST90016921 | CD62L- CD86+ myeloid Dendritic Cell %Dendritic Cell || id:ebi-a-GCST90001473 | rs11890098 | 2 | 157532549 | A | 0.074438 | 0.0153389 | 9.57E-07 |
| ebi-a-GCST90016921 | Gut microbiota abundance (class Mollicutes id.3920) || id:ebi-a-GCST90016921 | HLA DR++ monocyte %monocyte || id:ebi-a-GCST90001475 | rs11890098 | 2 | 157532549 | A | 0.074438 | 0.0153389 | 9.57E-07 |
| ebi-a-GCST90016921 | Gut microbiota abundance (class Mollicutes id.3920) || id:ebi-a-GCST90016921 | HLA DR++ monocyte Absolute Count || id:ebi-a-GCST90001477 | rs11890098 | 2 | 157532549 | A | 0.074438 | 0.0153389 | 9.57E-07 |
| ebi-a-GCST90016921 | Gut microbiota abundance (class Mollicutes id.3920) || id:ebi-a-GCST90016921 | Basophil %CD33dim HLA DR- CD66b- || id:ebi-a-GCST90001533 | rs11890098 | 2 | 157532549 | A | 0.074438 | 0.0153389 | 9.57E-07 |
| ebi-a-GCST90016921 | Gut microbiota abundance (class Mollicutes id.3920) || id:ebi-a-GCST90016921 | CD16+ monocyte %monocyte || id:ebi-a-GCST90001587 | rs11890098 | 2 | 157532549 | A | 0.074438 | 0.0153389 | 9.57E-07 |
| ebi-a-GCST90016921 | Gut microbiota abundance (class Mollicutes id.3920) || id:ebi-a-GCST90016921 | T/B cell || id:ebi-a-GCST90001588 | rs11890098 | 2 | 157532549 | A | 0.074438 | 0.0153389 | 9.57E-07 |
| ebi-a-GCST90016921 | Gut microbiota abundance (class Mollicutes id.3920) || id:ebi-a-GCST90016921 | CD4+CD8+ T cell %T cell || id:ebi-a-GCST90001595 | rs11890098 | 2 | 157532549 | A | 0.074438 | 0.0153389 | 9.57E-07 |
| ebi-a-GCST90016921 | Gut microbiota abundance (class Mollicutes id.3920) || id:ebi-a-GCST90016921 | CD8+ Natural Killer T %T cell || id:ebi-a-GCST90001631 | rs11890098 | 2 | 157532549 | A | 0.074438 | 0.0153389 | 9.57E-07 |
| ebi-a-GCST90016921 | Gut microbiota abundance (class Mollicutes id.3920) || id:ebi-a-GCST90016921 | CD4-CD8- Natural Killer T %lymphocyte || id:ebi-a-GCST90001638 | rs11890098 | 2 | 157532549 | A | 0.074438 | 0.0153389 | 9.57E-07 |
| ebi-a-GCST90016921 | Gut microbiota abundance (class Mollicutes id.3920) || id:ebi-a-GCST90016921 | HLA DR+ Natural Killer Absolute Count || id:ebi-a-GCST90001648 | rs11890098 | 2 | 157532549 | A | 0.074438 | 0.0153389 | 9.57E-07 |
| ebi-a-GCST90016921 | Gut microbiota abundance (class Mollicutes id.3920) || id:ebi-a-GCST90016921 | HLA DR+ Natural Killer %Natural Killer || id:ebi-a-GCST90001649 | rs11890098 | 2 | 157532549 | A | 0.074438 | 0.0153389 | 9.57E-07 |
| ebi-a-GCST90016921 | Gut microbiota abundance (class Mollicutes id.3920) || id:ebi-a-GCST90016921 | HLA DR+ Natural Killer %CD3- lymphocyte || id:ebi-a-GCST90001650 | rs11890098 | 2 | 157532549 | A | 0.074438 | 0.0153389 | 9.57E-07 |
| ebi-a-GCST90016921 | Gut microbiota abundance (class Mollicutes id.3920) || id:ebi-a-GCST90016921 | CD28- CD25++ CD8+ T cell Absolute Count || id:ebi-a-GCST90001678 | rs11890098 | 2 | 157532549 | A | 0.074438 | 0.0153389 | 9.57E-07 |
| ebi-a-GCST90016921 | Gut microbiota abundance (class Mollicutes id.3920) || id:ebi-a-GCST90016921 | CD25++ CD8+ T cell %T cell || id:ebi-a-GCST90001679 | rs11890098 | 2 | 157532549 | A | 0.074438 | 0.0153389 | 9.57E-07 |
| ebi-a-GCST90016921 | Gut microbiota abundance (class Mollicutes id.3920) || id:ebi-a-GCST90016921 | CD19 on CD20- CD38- B cell || id:ebi-a-GCST90001722 | rs11890098 | 2 | 157532549 | A | 0.074438 | 0.0153389 | 9.57E-07 |
| ebi-a-GCST90016921 | Gut microbiota abundance (class Mollicutes id.3920) || id:ebi-a-GCST90016921 | CD25 on B cell || id:ebi-a-GCST90001775 | rs11890098 | 2 | 157532549 | A | 0.074438 | 0.0153389 | 9.57E-07 |
| ebi-a-GCST90016921 | Gut microbiota abundance (class Mollicutes id.3920) || id:ebi-a-GCST90016921 | CD25 on IgD+ CD24- B cell || id:ebi-a-GCST90001779 | rs11890098 | 2 | 157532549 | A | 0.074438 | 0.0153389 | 9.57E-07 |
| ebi-a-GCST90016921 | Gut microbiota abundance (class Mollicutes id.3920) || id:ebi-a-GCST90016921 | CD25 on IgD+ CD38- naive B cell || id:ebi-a-GCST90001781 | rs11890098 | 2 | 157532549 | A | 0.074438 | 0.0153389 | 9.57E-07 |
| ebi-a-GCST90016921 | Gut microbiota abundance (class Mollicutes id.3920) || id:ebi-a-GCST90016921 | CD25 on IgD+ CD38+ B cell || id:ebi-a-GCST90001783 | rs11890098 | 2 | 157532549 | A | 0.074438 | 0.0153389 | 9.57E-07 |
| ebi-a-GCST90016921 | Gut microbiota abundance (class Mollicutes id.3920) || id:ebi-a-GCST90016921 | CD86 on myeloid Dendritic Cell || id:ebi-a-GCST90001903 | rs11890098 | 2 | 157532549 | A | 0.074438 | 0.0153389 | 9.57E-07 |
| ebi-a-GCST90016921 | Gut microbiota abundance (class Mollicutes id.3920) || id:ebi-a-GCST90016921 | CD33 on CD14+ monocyte || id:ebi-a-GCST90001946 | rs11890098 | 2 | 157532549 | A | 0.074438 | 0.0153389 | 9.57E-07 |
| ebi-a-GCST90016921 | Gut microbiota abundance (class Mollicutes id.3920) || id:ebi-a-GCST90016921 | CD33 on CD33+ HLA DR+ CD14dim || id:ebi-a-GCST90001947 | rs11890098 | 2 | 157532549 | A | 0.074438 | 0.0153389 | 9.57E-07 |
| ebi-a-GCST90016921 | Gut microbiota abundance (class Mollicutes id.3920) || id:ebi-a-GCST90016921 | CD33 on CD33dim HLA DR+ CD11b+ || id:ebi-a-GCST90001948 | rs11890098 | 2 | 157532549 | A | 0.074438 | 0.0153389 | 9.57E-07 |
| ebi-a-GCST90016921 | Gut microbiota abundance (class Mollicutes id.3920) || id:ebi-a-GCST90016921 | CD33 on Granulocytic Myeloid-Derived Suppressor Cells || id:ebi-a-GCST90001950 | rs11890098 | 2 | 157532549 | A | 0.074438 | 0.0153389 | 9.57E-07 |
| ebi-a-GCST90016921 | Gut microbiota abundance (class Mollicutes id.3920) || id:ebi-a-GCST90016921 | CD33 on CD66b++ myeloid cell || id:ebi-a-GCST90001951 | rs11890098 | 2 | 157532549 | A | 0.074438 | 0.0153389 | 9.57E-07 |
| ebi-a-GCST90016921 | Gut microbiota abundance (class Mollicutes id.3920) || id:ebi-a-GCST90016921 | CD33 on CD33dim HLA DR- || id:ebi-a-GCST90001953 | rs11890098 | 2 | 157532549 | A | 0.074438 | 0.0153389 | 9.57E-07 |
| ebi-a-GCST90016921 | Gut microbiota abundance (class Mollicutes id.3920) || id:ebi-a-GCST90016921 | CD33 on basophil || id:ebi-a-GCST90001954 | rs11890098 | 2 | 157532549 | A | 0.074438 | 0.0153389 | 9.57E-07 |
| ebi-a-GCST90016921 | Gut microbiota abundance (class Mollicutes id.3920) || id:ebi-a-GCST90016921 | CD33 on Immature Myeloid-Derived Suppressor Cells || id:ebi-a-GCST90001955 | rs11890098 | 2 | 157532549 | A | 0.074438 | 0.0153389 | 9.57E-07 |
| ebi-a-GCST90016921 | Gut microbiota abundance (class Mollicutes id.3920) || id:ebi-a-GCST90016921 | FSC-A on HLA DR+ CD8+ T cell || id:ebi-a-GCST90001978 | rs11890098 | 2 | 157532549 | A | 0.074438 | 0.0153389 | 9.57E-07 |
| ebi-a-GCST90016921 | Gut microbiota abundance (class Mollicutes id.3920) || id:ebi-a-GCST90016921 | HLA DR on CD14+ CD16- monocyte || id:ebi-a-GCST90001988 | rs11890098 | 2 | 157532549 | A | 0.074438 | 0.0153389 | 9.57E-07 |
| ebi-a-GCST90016921 | Gut microbiota abundance (class Mollicutes id.3920) || id:ebi-a-GCST90016921 | HLA DR on CD14+ monocyte || id:ebi-a-GCST90001991 | rs11890098 | 2 | 157532549 | A | 0.074438 | 0.0153389 | 9.57E-07 |
| ebi-a-GCST90016921 | Gut microbiota abundance (class Mollicutes id.3920) || id:ebi-a-GCST90016921 | CD16 on CD14+ CD16+ monocyte || id:ebi-a-GCST90002005 | rs11890098 | 2 | 157532549 | A | 0.074438 | 0.0153389 | 9.57E-07 |
| ebi-a-GCST90016921 | Gut microbiota abundance (class Mollicutes id.3920) || id:ebi-a-GCST90016921 | CD45 on CD33+ HLA DR+ CD14- || id:ebi-a-GCST90002042 | rs11890098 | 2 | 157532549 | A | 0.074438 | 0.0153389 | 9.57E-07 |
| ebi-a-GCST90016921 | Gut microbiota abundance (class Mollicutes id.3920) || id:ebi-a-GCST90016921 | CD8 on Natural Killer T || id:ebi-a-GCST90002059 | rs11890098 | 2 | 157532549 | A | 0.074438 | 0.0153389 | 9.57E-07 |
| ebi-a-GCST90017117 | Gut microbiota abundance (phylum Tenericutes id.3919) || id:ebi-a-GCST90017117 | Myeloid Dendritic Cell Absolute Count || id:ebi-a-GCST90001458 | rs11890098 | 2 | 157532549 | A | 0.074438 | 0.0153389 | 9.57E-07 |
| ebi-a-GCST90017117 | Gut microbiota abundance (phylum Tenericutes id.3919) || id:ebi-a-GCST90017117 | CD86+ myeloid Dendritic Cell %Dendritic Cell || id:ebi-a-GCST90001465 | rs11890098 | 2 | 157532549 | A | 0.074438 | 0.0153389 | 9.57E-07 |
| ebi-a-GCST90017117 | Gut microbiota abundance (phylum Tenericutes id.3919) || id:ebi-a-GCST90017117 | CD62L- myeloid Dendritic Cell Absolute Count || id:ebi-a-GCST90001468 | rs11890098 | 2 | 157532549 | A | 0.074438 | 0.0153389 | 9.57E-07 |
| ebi-a-GCST90017117 | Gut microbiota abundance (phylum Tenericutes id.3919) || id:ebi-a-GCST90017117 | CD62L- myeloid Dendritic Cell %Dendritic Cell || id:ebi-a-GCST90001469 | rs11890098 | 2 | 157532549 | A | 0.074438 | 0.0153389 | 9.57E-07 |
| ebi-a-GCST90017117 | Gut microbiota abundance (phylum Tenericutes id.3919) || id:ebi-a-GCST90017117 | CD62L- CD86+ myeloid Dendritic Cell Absolute Count || id:ebi-a-GCST90001472 | rs11890098 | 2 | 157532549 | A | 0.074438 | 0.0153389 | 9.57E-07 |
| ebi-a-GCST90017117 | Gut microbiota abundance (phylum Tenericutes id.3919) || id:ebi-a-GCST90017117 | CD62L- CD86+ myeloid Dendritic Cell %Dendritic Cell || id:ebi-a-GCST90001473 | rs11890098 | 2 | 157532549 | A | 0.074438 | 0.0153389 | 9.57E-07 |
| ebi-a-GCST90017117 | Gut microbiota abundance (phylum Tenericutes id.3919) || id:ebi-a-GCST90017117 | HLA DR++ monocyte %monocyte || id:ebi-a-GCST90001475 | rs11890098 | 2 | 157532549 | A | 0.074438 | 0.0153389 | 9.57E-07 |
| ebi-a-GCST90017117 | Gut microbiota abundance (phylum Tenericutes id.3919) || id:ebi-a-GCST90017117 | HLA DR++ monocyte Absolute Count || id:ebi-a-GCST90001477 | rs11890098 | 2 | 157532549 | A | 0.074438 | 0.0153389 | 9.57E-07 |
| ebi-a-GCST90017117 | Gut microbiota abundance (phylum Tenericutes id.3919) || id:ebi-a-GCST90017117 | Basophil %CD33dim HLA DR- CD66b- || id:ebi-a-GCST90001533 | rs11890098 | 2 | 157532549 | A | 0.074438 | 0.0153389 | 9.57E-07 |
| ebi-a-GCST90017117 | Gut microbiota abundance (phylum Tenericutes id.3919) || id:ebi-a-GCST90017117 | CD16+ monocyte %monocyte || id:ebi-a-GCST90001587 | rs11890098 | 2 | 157532549 | A | 0.074438 | 0.0153389 | 9.57E-07 |
| ebi-a-GCST90017117 | Gut microbiota abundance (phylum Tenericutes id.3919) || id:ebi-a-GCST90017117 | T/B cell || id:ebi-a-GCST90001588 | rs11890098 | 2 | 157532549 | A | 0.074438 | 0.0153389 | 9.57E-07 |
| ebi-a-GCST90017117 | Gut microbiota abundance (phylum Tenericutes id.3919) || id:ebi-a-GCST90017117 | CD4+CD8+ T cell %T cell || id:ebi-a-GCST90001595 | rs11890098 | 2 | 157532549 | A | 0.074438 | 0.0153389 | 9.57E-07 |
| ebi-a-GCST90017117 | Gut microbiota abundance (phylum Tenericutes id.3919) || id:ebi-a-GCST90017117 | CD8+ Natural Killer T %T cell || id:ebi-a-GCST90001631 | rs11890098 | 2 | 157532549 | A | 0.074438 | 0.0153389 | 9.57E-07 |
| ebi-a-GCST90017117 | Gut microbiota abundance (phylum Tenericutes id.3919) || id:ebi-a-GCST90017117 | CD4-CD8- Natural Killer T %lymphocyte || id:ebi-a-GCST90001638 | rs11890098 | 2 | 157532549 | A | 0.074438 | 0.0153389 | 9.57E-07 |
| ebi-a-GCST90017117 | Gut microbiota abundance (phylum Tenericutes id.3919) || id:ebi-a-GCST90017117 | HLA DR+ Natural Killer Absolute Count || id:ebi-a-GCST90001648 | rs11890098 | 2 | 157532549 | A | 0.074438 | 0.0153389 | 9.57E-07 |
| ebi-a-GCST90017117 | Gut microbiota abundance (phylum Tenericutes id.3919) || id:ebi-a-GCST90017117 | HLA DR+ Natural Killer %Natural Killer || id:ebi-a-GCST90001649 | rs11890098 | 2 | 157532549 | A | 0.074438 | 0.0153389 | 9.57E-07 |
| ebi-a-GCST90017117 | Gut microbiota abundance (phylum Tenericutes id.3919) || id:ebi-a-GCST90017117 | HLA DR+ Natural Killer %CD3- lymphocyte || id:ebi-a-GCST90001650 | rs11890098 | 2 | 157532549 | A | 0.074438 | 0.0153389 | 9.57E-07 |
| ebi-a-GCST90017117 | Gut microbiota abundance (phylum Tenericutes id.3919) || id:ebi-a-GCST90017117 | CD28- CD25++ CD8+ T cell Absolute Count || id:ebi-a-GCST90001678 | rs11890098 | 2 | 157532549 | A | 0.074438 | 0.0153389 | 9.57E-07 |
| ebi-a-GCST90017117 | Gut microbiota abundance (phylum Tenericutes id.3919) || id:ebi-a-GCST90017117 | CD25++ CD8+ T cell %T cell || id:ebi-a-GCST90001679 | rs11890098 | 2 | 157532549 | A | 0.074438 | 0.0153389 | 9.57E-07 |
| ebi-a-GCST90017117 | Gut microbiota abundance (phylum Tenericutes id.3919) || id:ebi-a-GCST90017117 | CD19 on CD20- CD38- B cell || id:ebi-a-GCST90001722 | rs11890098 | 2 | 157532549 | A | 0.074438 | 0.0153389 | 9.57E-07 |
| ebi-a-GCST90017117 | Gut microbiota abundance (phylum Tenericutes id.3919) || id:ebi-a-GCST90017117 | CD25 on B cell || id:ebi-a-GCST90001775 | rs11890098 | 2 | 157532549 | A | 0.074438 | 0.0153389 | 9.57E-07 |
| ebi-a-GCST90017117 | Gut microbiota abundance (phylum Tenericutes id.3919) || id:ebi-a-GCST90017117 | CD25 on IgD+ CD24- B cell || id:ebi-a-GCST90001779 | rs11890098 | 2 | 157532549 | A | 0.074438 | 0.0153389 | 9.57E-07 |
| ebi-a-GCST90017117 | Gut microbiota abundance (phylum Tenericutes id.3919) || id:ebi-a-GCST90017117 | CD25 on IgD+ CD38- naive B cell || id:ebi-a-GCST90001781 | rs11890098 | 2 | 157532549 | A | 0.074438 | 0.0153389 | 9.57E-07 |
| ebi-a-GCST90017117 | Gut microbiota abundance (phylum Tenericutes id.3919) || id:ebi-a-GCST90017117 | CD25 on IgD+ CD38+ B cell || id:ebi-a-GCST90001783 | rs11890098 | 2 | 157532549 | A | 0.074438 | 0.0153389 | 9.57E-07 |
| ebi-a-GCST90017117 | Gut microbiota abundance (phylum Tenericutes id.3919) || id:ebi-a-GCST90017117 | CD86 on myeloid Dendritic Cell || id:ebi-a-GCST90001903 | rs11890098 | 2 | 157532549 | A | 0.074438 | 0.0153389 | 9.57E-07 |
| ebi-a-GCST90017117 | Gut microbiota abundance (phylum Tenericutes id.3919) || id:ebi-a-GCST90017117 | CD33 on CD14+ monocyte || id:ebi-a-GCST90001946 | rs11890098 | 2 | 157532549 | A | 0.074438 | 0.0153389 | 9.57E-07 |
| ebi-a-GCST90017117 | Gut microbiota abundance (phylum Tenericutes id.3919) || id:ebi-a-GCST90017117 | CD33 on CD33+ HLA DR+ CD14dim || id:ebi-a-GCST90001947 | rs11890098 | 2 | 157532549 | A | 0.074438 | 0.0153389 | 9.57E-07 |
| ebi-a-GCST90017117 | Gut microbiota abundance (phylum Tenericutes id.3919) || id:ebi-a-GCST90017117 | CD33 on CD33dim HLA DR+ CD11b+ || id:ebi-a-GCST90001948 | rs11890098 | 2 | 157532549 | A | 0.074438 | 0.0153389 | 9.57E-07 |
| ebi-a-GCST90017117 | Gut microbiota abundance (phylum Tenericutes id.3919) || id:ebi-a-GCST90017117 | CD33 on Granulocytic Myeloid-Derived Suppressor Cells || id:ebi-a-GCST90001950 | rs11890098 | 2 | 157532549 | A | 0.074438 | 0.0153389 | 9.57E-07 |
| ebi-a-GCST90017117 | Gut microbiota abundance (phylum Tenericutes id.3919) || id:ebi-a-GCST90017117 | CD33 on CD66b++ myeloid cell || id:ebi-a-GCST90001951 | rs11890098 | 2 | 157532549 | A | 0.074438 | 0.0153389 | 9.57E-07 |
| ebi-a-GCST90017117 | Gut microbiota abundance (phylum Tenericutes id.3919) || id:ebi-a-GCST90017117 | CD33 on CD33dim HLA DR- || id:ebi-a-GCST90001953 | rs11890098 | 2 | 157532549 | A | 0.074438 | 0.0153389 | 9.57E-07 |
| ebi-a-GCST90017117 | Gut microbiota abundance (phylum Tenericutes id.3919) || id:ebi-a-GCST90017117 | CD33 on basophil || id:ebi-a-GCST90001954 | rs11890098 | 2 | 157532549 | A | 0.074438 | 0.0153389 | 9.57E-07 |
| ebi-a-GCST90017117 | Gut microbiota abundance (phylum Tenericutes id.3919) || id:ebi-a-GCST90017117 | CD33 on Immature Myeloid-Derived Suppressor Cells || id:ebi-a-GCST90001955 | rs11890098 | 2 | 157532549 | A | 0.074438 | 0.0153389 | 9.57E-07 |
| ebi-a-GCST90017117 | Gut microbiota abundance (phylum Tenericutes id.3919) || id:ebi-a-GCST90017117 | FSC-A on HLA DR+ CD8+ T cell || id:ebi-a-GCST90001978 | rs11890098 | 2 | 157532549 | A | 0.074438 | 0.0153389 | 9.57E-07 |
| ebi-a-GCST90017117 | Gut microbiota abundance (phylum Tenericutes id.3919) || id:ebi-a-GCST90017117 | HLA DR on CD14+ CD16- monocyte || id:ebi-a-GCST90001988 | rs11890098 | 2 | 157532549 | A | 0.074438 | 0.0153389 | 9.57E-07 |
| ebi-a-GCST90017117 | Gut microbiota abundance (phylum Tenericutes id.3919) || id:ebi-a-GCST90017117 | HLA DR on CD14+ monocyte || id:ebi-a-GCST90001991 | rs11890098 | 2 | 157532549 | A | 0.074438 | 0.0153389 | 9.57E-07 |
| ebi-a-GCST90017117 | Gut microbiota abundance (phylum Tenericutes id.3919) || id:ebi-a-GCST90017117 | CD16 on CD14+ CD16+ monocyte || id:ebi-a-GCST90002005 | rs11890098 | 2 | 157532549 | A | 0.074438 | 0.0153389 | 9.57E-07 |
| ebi-a-GCST90017117 | Gut microbiota abundance (phylum Tenericutes id.3919) || id:ebi-a-GCST90017117 | CD45 on CD33+ HLA DR+ CD14- || id:ebi-a-GCST90002042 | rs11890098 | 2 | 157532549 | A | 0.074438 | 0.0153389 | 9.57E-07 |
| ebi-a-GCST90017117 | Gut microbiota abundance (phylum Tenericutes id.3919) || id:ebi-a-GCST90017117 | CD8 on Natural Killer T || id:ebi-a-GCST90002059 | rs11890098 | 2 | 157532549 | A | 0.074438 | 0.0153389 | 9.57E-07 |
| ebi-a-GCST90017037 | Gut microbiota abundance (genus Oscillospira id.2064) || id:ebi-a-GCST90017037 | Myeloid Dendritic Cell Absolute Count || id:ebi-a-GCST90001458 | rs12206468 | 6 | 18093691 | G | -0.133016 | 0.0269733 | 1.04E-06 |
| ebi-a-GCST90017037 | Gut microbiota abundance (genus Oscillospira id.2064) || id:ebi-a-GCST90017037 | CD86+ myeloid Dendritic Cell %Dendritic Cell || id:ebi-a-GCST90001465 | rs12206468 | 6 | 18093691 | G | -0.133016 | 0.0269733 | 1.04E-06 |
| ebi-a-GCST90017037 | Gut microbiota abundance (genus Oscillospira id.2064) || id:ebi-a-GCST90017037 | CD62L- myeloid Dendritic Cell Absolute Count || id:ebi-a-GCST90001468 | rs12206468 | 6 | 18093691 | G | -0.133016 | 0.0269733 | 1.04E-06 |
| ebi-a-GCST90017037 | Gut microbiota abundance (genus Oscillospira id.2064) || id:ebi-a-GCST90017037 | CD62L- myeloid Dendritic Cell %Dendritic Cell || id:ebi-a-GCST90001469 | rs12206468 | 6 | 18093691 | G | -0.133016 | 0.0269733 | 1.04E-06 |
| ebi-a-GCST90017037 | Gut microbiota abundance (genus Oscillospira id.2064) || id:ebi-a-GCST90017037 | CD62L- CD86+ myeloid Dendritic Cell Absolute Count || id:ebi-a-GCST90001472 | rs12206468 | 6 | 18093691 | G | -0.133016 | 0.0269733 | 1.04E-06 |
| ebi-a-GCST90017037 | Gut microbiota abundance (genus Oscillospira id.2064) || id:ebi-a-GCST90017037 | CD62L- CD86+ myeloid Dendritic Cell %Dendritic Cell || id:ebi-a-GCST90001473 | rs12206468 | 6 | 18093691 | G | -0.133016 | 0.0269733 | 1.04E-06 |
| ebi-a-GCST90017037 | Gut microbiota abundance (genus Oscillospira id.2064) || id:ebi-a-GCST90017037 | HLA DR++ monocyte %monocyte || id:ebi-a-GCST90001475 | rs12206468 | 6 | 18093691 | G | -0.133016 | 0.0269733 | 1.04E-06 |
| ebi-a-GCST90017037 | Gut microbiota abundance (genus Oscillospira id.2064) || id:ebi-a-GCST90017037 | HLA DR++ monocyte Absolute Count || id:ebi-a-GCST90001477 | rs12206468 | 6 | 18093691 | G | -0.133016 | 0.0269733 | 1.04E-06 |
| ebi-a-GCST90017037 | Gut microbiota abundance (genus Oscillospira id.2064) || id:ebi-a-GCST90017037 | Basophil %CD33dim HLA DR- CD66b- || id:ebi-a-GCST90001533 | rs12206468 | 6 | 18093691 | G | -0.133016 | 0.0269733 | 1.04E-06 |
| ebi-a-GCST90017037 | Gut microbiota abundance (genus Oscillospira id.2064) || id:ebi-a-GCST90017037 | CD16+ monocyte %monocyte || id:ebi-a-GCST90001587 | rs12206468 | 6 | 18093691 | G | -0.133016 | 0.0269733 | 1.04E-06 |
| ebi-a-GCST90017037 | Gut microbiota abundance (genus Oscillospira id.2064) || id:ebi-a-GCST90017037 | T/B cell || id:ebi-a-GCST90001588 | rs12206468 | 6 | 18093691 | G | -0.133016 | 0.0269733 | 1.04E-06 |
| ebi-a-GCST90017037 | Gut microbiota abundance (genus Oscillospira id.2064) || id:ebi-a-GCST90017037 | CD4+CD8+ T cell %T cell || id:ebi-a-GCST90001595 | rs12206468 | 6 | 18093691 | G | -0.133016 | 0.0269733 | 1.04E-06 |
| ebi-a-GCST90017037 | Gut microbiota abundance (genus Oscillospira id.2064) || id:ebi-a-GCST90017037 | CD8+ Natural Killer T %T cell || id:ebi-a-GCST90001631 | rs12206468 | 6 | 18093691 | G | -0.133016 | 0.0269733 | 1.04E-06 |
| ebi-a-GCST90017037 | Gut microbiota abundance (genus Oscillospira id.2064) || id:ebi-a-GCST90017037 | CD4-CD8- Natural Killer T %lymphocyte || id:ebi-a-GCST90001638 | rs12206468 | 6 | 18093691 | G | -0.133016 | 0.0269733 | 1.04E-06 |
| ebi-a-GCST90017037 | Gut microbiota abundance (genus Oscillospira id.2064) || id:ebi-a-GCST90017037 | HLA DR+ Natural Killer Absolute Count || id:ebi-a-GCST90001648 | rs12206468 | 6 | 18093691 | G | -0.133016 | 0.0269733 | 1.04E-06 |
| ebi-a-GCST90017037 | Gut microbiota abundance (genus Oscillospira id.2064) || id:ebi-a-GCST90017037 | HLA DR+ Natural Killer %Natural Killer || id:ebi-a-GCST90001649 | rs12206468 | 6 | 18093691 | G | -0.133016 | 0.0269733 | 1.04E-06 |
| ebi-a-GCST90017037 | Gut microbiota abundance (genus Oscillospira id.2064) || id:ebi-a-GCST90017037 | HLA DR+ Natural Killer %CD3- lymphocyte || id:ebi-a-GCST90001650 | rs12206468 | 6 | 18093691 | G | -0.133016 | 0.0269733 | 1.04E-06 |
| ebi-a-GCST90017037 | Gut microbiota abundance (genus Oscillospira id.2064) || id:ebi-a-GCST90017037 | CD28- CD25++ CD8+ T cell Absolute Count || id:ebi-a-GCST90001678 | rs12206468 | 6 | 18093691 | G | -0.133016 | 0.0269733 | 1.04E-06 |
| ebi-a-GCST90017037 | Gut microbiota abundance (genus Oscillospira id.2064) || id:ebi-a-GCST90017037 | CD25++ CD8+ T cell %T cell || id:ebi-a-GCST90001679 | rs12206468 | 6 | 18093691 | G | -0.133016 | 0.0269733 | 1.04E-06 |
| ebi-a-GCST90017037 | Gut microbiota abundance (genus Oscillospira id.2064) || id:ebi-a-GCST90017037 | CD19 on CD20- CD38- B cell || id:ebi-a-GCST90001722 | rs12206468 | 6 | 18093691 | G | -0.133016 | 0.0269733 | 1.04E-06 |
| ebi-a-GCST90017037 | Gut microbiota abundance (genus Oscillospira id.2064) || id:ebi-a-GCST90017037 | CD25 on B cell || id:ebi-a-GCST90001775 | rs12206468 | 6 | 18093691 | G | -0.133016 | 0.0269733 | 1.04E-06 |
| ebi-a-GCST90017037 | Gut microbiota abundance (genus Oscillospira id.2064) || id:ebi-a-GCST90017037 | CD25 on IgD+ CD24- B cell || id:ebi-a-GCST90001779 | rs12206468 | 6 | 18093691 | G | -0.133016 | 0.0269733 | 1.04E-06 |
| ebi-a-GCST90017037 | Gut microbiota abundance (genus Oscillospira id.2064) || id:ebi-a-GCST90017037 | CD25 on IgD+ CD38- naive B cell || id:ebi-a-GCST90001781 | rs12206468 | 6 | 18093691 | G | -0.133016 | 0.0269733 | 1.04E-06 |
| ebi-a-GCST90017037 | Gut microbiota abundance (genus Oscillospira id.2064) || id:ebi-a-GCST90017037 | CD25 on IgD+ CD38+ B cell || id:ebi-a-GCST90001783 | rs12206468 | 6 | 18093691 | G | -0.133016 | 0.0269733 | 1.04E-06 |
| ebi-a-GCST90017037 | Gut microbiota abundance (genus Oscillospira id.2064) || id:ebi-a-GCST90017037 | CD86 on myeloid Dendritic Cell || id:ebi-a-GCST90001903 | rs12206468 | 6 | 18093691 | G | -0.133016 | 0.0269733 | 1.04E-06 |
| ebi-a-GCST90017037 | Gut microbiota abundance (genus Oscillospira id.2064) || id:ebi-a-GCST90017037 | CD33 on CD14+ monocyte || id:ebi-a-GCST90001946 | rs12206468 | 6 | 18093691 | G | -0.133016 | 0.0269733 | 1.04E-06 |
| ebi-a-GCST90017037 | Gut microbiota abundance (genus Oscillospira id.2064) || id:ebi-a-GCST90017037 | CD33 on CD33+ HLA DR+ CD14dim || id:ebi-a-GCST90001947 | rs12206468 | 6 | 18093691 | G | -0.133016 | 0.0269733 | 1.04E-06 |
| ebi-a-GCST90017037 | Gut microbiota abundance (genus Oscillospira id.2064) || id:ebi-a-GCST90017037 | CD33 on CD33dim HLA DR+ CD11b+ || id:ebi-a-GCST90001948 | rs12206468 | 6 | 18093691 | G | -0.133016 | 0.0269733 | 1.04E-06 |
| ebi-a-GCST90017037 | Gut microbiota abundance (genus Oscillospira id.2064) || id:ebi-a-GCST90017037 | CD33 on Granulocytic Myeloid-Derived Suppressor Cells || id:ebi-a-GCST90001950 | rs12206468 | 6 | 18093691 | G | -0.133016 | 0.0269733 | 1.04E-06 |
| ebi-a-GCST90017037 | Gut microbiota abundance (genus Oscillospira id.2064) || id:ebi-a-GCST90017037 | CD33 on CD66b++ myeloid cell || id:ebi-a-GCST90001951 | rs12206468 | 6 | 18093691 | G | -0.133016 | 0.0269733 | 1.04E-06 |
| ebi-a-GCST90017037 | Gut microbiota abundance (genus Oscillospira id.2064) || id:ebi-a-GCST90017037 | CD33 on CD33dim HLA DR- || id:ebi-a-GCST90001953 | rs12206468 | 6 | 18093691 | G | -0.133016 | 0.0269733 | 1.04E-06 |
| ebi-a-GCST90017037 | Gut microbiota abundance (genus Oscillospira id.2064) || id:ebi-a-GCST90017037 | CD33 on basophil || id:ebi-a-GCST90001954 | rs12206468 | 6 | 18093691 | G | -0.133016 | 0.0269733 | 1.04E-06 |
| ebi-a-GCST90017037 | Gut microbiota abundance (genus Oscillospira id.2064) || id:ebi-a-GCST90017037 | CD33 on Immature Myeloid-Derived Suppressor Cells || id:ebi-a-GCST90001955 | rs12206468 | 6 | 18093691 | G | -0.133016 | 0.0269733 | 1.04E-06 |
| ebi-a-GCST90017037 | Gut microbiota abundance (genus Oscillospira id.2064) || id:ebi-a-GCST90017037 | FSC-A on HLA DR+ CD8+ T cell || id:ebi-a-GCST90001978 | rs12206468 | 6 | 18093691 | G | -0.133016 | 0.0269733 | 1.04E-06 |
| ebi-a-GCST90017037 | Gut microbiota abundance (genus Oscillospira id.2064) || id:ebi-a-GCST90017037 | HLA DR on CD14+ CD16- monocyte || id:ebi-a-GCST90001988 | rs12206468 | 6 | 18093691 | G | -0.133016 | 0.0269733 | 1.04E-06 |
| ebi-a-GCST90017037 | Gut microbiota abundance (genus Oscillospira id.2064) || id:ebi-a-GCST90017037 | HLA DR on CD14+ monocyte || id:ebi-a-GCST90001991 | rs12206468 | 6 | 18093691 | G | -0.133016 | 0.0269733 | 1.04E-06 |
| ebi-a-GCST90017037 | Gut microbiota abundance (genus Oscillospira id.2064) || id:ebi-a-GCST90017037 | CD16 on CD14+ CD16+ monocyte || id:ebi-a-GCST90002005 | rs12206468 | 6 | 18093691 | G | -0.133016 | 0.0269733 | 1.04E-06 |
| ebi-a-GCST90017037 | Gut microbiota abundance (genus Oscillospira id.2064) || id:ebi-a-GCST90017037 | CD45 on CD33+ HLA DR+ CD14- || id:ebi-a-GCST90002042 | rs12206468 | 6 | 18093691 | G | -0.133016 | 0.0269733 | 1.04E-06 |
| ebi-a-GCST90017037 | Gut microbiota abundance (genus Oscillospira id.2064) || id:ebi-a-GCST90017037 | CD8 on Natural Killer T || id:ebi-a-GCST90002059 | rs12206468 | 6 | 18093691 | G | -0.133016 | 0.0269733 | 1.04E-06 |
| ebi-a-GCST90016921 | Gut microbiota abundance (class Mollicutes id.3920) || id:ebi-a-GCST90016921 | Myeloid Dendritic Cell Absolute Count || id:ebi-a-GCST90001458 | rs12566890 | 1 | 61850864 | T | -0.101147 | 0.0230978 | 3.65E-06 |
| ebi-a-GCST90016921 | Gut microbiota abundance (class Mollicutes id.3920) || id:ebi-a-GCST90016921 | CD86+ myeloid Dendritic Cell %Dendritic Cell || id:ebi-a-GCST90001465 | rs12566890 | 1 | 61850864 | T | -0.101147 | 0.0230978 | 3.65E-06 |
| ebi-a-GCST90016921 | Gut microbiota abundance (class Mollicutes id.3920) || id:ebi-a-GCST90016921 | CD62L- myeloid Dendritic Cell Absolute Count || id:ebi-a-GCST90001468 | rs12566890 | 1 | 61850864 | T | -0.101147 | 0.0230978 | 3.65E-06 |
| ebi-a-GCST90016921 | Gut microbiota abundance (class Mollicutes id.3920) || id:ebi-a-GCST90016921 | CD62L- myeloid Dendritic Cell %Dendritic Cell || id:ebi-a-GCST90001469 | rs12566890 | 1 | 61850864 | T | -0.101147 | 0.0230978 | 3.65E-06 |
| ebi-a-GCST90016921 | Gut microbiota abundance (class Mollicutes id.3920) || id:ebi-a-GCST90016921 | CD62L- CD86+ myeloid Dendritic Cell Absolute Count || id:ebi-a-GCST90001472 | rs12566890 | 1 | 61850864 | T | -0.101147 | 0.0230978 | 3.65E-06 |
| ebi-a-GCST90016921 | Gut microbiota abundance (class Mollicutes id.3920) || id:ebi-a-GCST90016921 | CD62L- CD86+ myeloid Dendritic Cell %Dendritic Cell || id:ebi-a-GCST90001473 | rs12566890 | 1 | 61850864 | T | -0.101147 | 0.0230978 | 3.65E-06 |
| ebi-a-GCST90016921 | Gut microbiota abundance (class Mollicutes id.3920) || id:ebi-a-GCST90016921 | HLA DR++ monocyte %monocyte || id:ebi-a-GCST90001475 | rs12566890 | 1 | 61850864 | T | -0.101147 | 0.0230978 | 3.65E-06 |
| ebi-a-GCST90016921 | Gut microbiota abundance (class Mollicutes id.3920) || id:ebi-a-GCST90016921 | HLA DR++ monocyte Absolute Count || id:ebi-a-GCST90001477 | rs12566890 | 1 | 61850864 | T | -0.101147 | 0.0230978 | 3.65E-06 |
| ebi-a-GCST90016921 | Gut microbiota abundance (class Mollicutes id.3920) || id:ebi-a-GCST90016921 | Basophil %CD33dim HLA DR- CD66b- || id:ebi-a-GCST90001533 | rs12566890 | 1 | 61850864 | T | -0.101147 | 0.0230978 | 3.65E-06 |
| ebi-a-GCST90016921 | Gut microbiota abundance (class Mollicutes id.3920) || id:ebi-a-GCST90016921 | CD16+ monocyte %monocyte || id:ebi-a-GCST90001587 | rs12566890 | 1 | 61850864 | T | -0.101147 | 0.0230978 | 3.65E-06 |
| ebi-a-GCST90016921 | Gut microbiota abundance (class Mollicutes id.3920) || id:ebi-a-GCST90016921 | T/B cell || id:ebi-a-GCST90001588 | rs12566890 | 1 | 61850864 | T | -0.101147 | 0.0230978 | 3.65E-06 |
| ebi-a-GCST90016921 | Gut microbiota abundance (class Mollicutes id.3920) || id:ebi-a-GCST90016921 | CD4+CD8+ T cell %T cell || id:ebi-a-GCST90001595 | rs12566890 | 1 | 61850864 | T | -0.101147 | 0.0230978 | 3.65E-06 |
| ebi-a-GCST90016921 | Gut microbiota abundance (class Mollicutes id.3920) || id:ebi-a-GCST90016921 | CD8+ Natural Killer T %T cell || id:ebi-a-GCST90001631 | rs12566890 | 1 | 61850864 | T | -0.101147 | 0.0230978 | 3.65E-06 |
| ebi-a-GCST90016921 | Gut microbiota abundance (class Mollicutes id.3920) || id:ebi-a-GCST90016921 | CD4-CD8- Natural Killer T %lymphocyte || id:ebi-a-GCST90001638 | rs12566890 | 1 | 61850864 | T | -0.101147 | 0.0230978 | 3.65E-06 |
| ebi-a-GCST90016921 | Gut microbiota abundance (class Mollicutes id.3920) || id:ebi-a-GCST90016921 | HLA DR+ Natural Killer Absolute Count || id:ebi-a-GCST90001648 | rs12566890 | 1 | 61850864 | T | -0.101147 | 0.0230978 | 3.65E-06 |
| ebi-a-GCST90016921 | Gut microbiota abundance (class Mollicutes id.3920) || id:ebi-a-GCST90016921 | HLA DR+ Natural Killer %Natural Killer || id:ebi-a-GCST90001649 | rs12566890 | 1 | 61850864 | T | -0.101147 | 0.0230978 | 3.65E-06 |
| ebi-a-GCST90016921 | Gut microbiota abundance (class Mollicutes id.3920) || id:ebi-a-GCST90016921 | HLA DR+ Natural Killer %CD3- lymphocyte || id:ebi-a-GCST90001650 | rs12566890 | 1 | 61850864 | T | -0.101147 | 0.0230978 | 3.65E-06 |
| ebi-a-GCST90016921 | Gut microbiota abundance (class Mollicutes id.3920) || id:ebi-a-GCST90016921 | CD28- CD25++ CD8+ T cell Absolute Count || id:ebi-a-GCST90001678 | rs12566890 | 1 | 61850864 | T | -0.101147 | 0.0230978 | 3.65E-06 |
| ebi-a-GCST90016921 | Gut microbiota abundance (class Mollicutes id.3920) || id:ebi-a-GCST90016921 | CD25++ CD8+ T cell %T cell || id:ebi-a-GCST90001679 | rs12566890 | 1 | 61850864 | T | -0.101147 | 0.0230978 | 3.65E-06 |
| ebi-a-GCST90016921 | Gut microbiota abundance (class Mollicutes id.3920) || id:ebi-a-GCST90016921 | CD19 on CD20- CD38- B cell || id:ebi-a-GCST90001722 | rs12566890 | 1 | 61850864 | T | -0.101147 | 0.0230978 | 3.65E-06 |
| ebi-a-GCST90016921 | Gut microbiota abundance (class Mollicutes id.3920) || id:ebi-a-GCST90016921 | CD25 on B cell || id:ebi-a-GCST90001775 | rs12566890 | 1 | 61850864 | T | -0.101147 | 0.0230978 | 3.65E-06 |
| ebi-a-GCST90016921 | Gut microbiota abundance (class Mollicutes id.3920) || id:ebi-a-GCST90016921 | CD25 on IgD+ CD24- B cell || id:ebi-a-GCST90001779 | rs12566890 | 1 | 61850864 | T | -0.101147 | 0.0230978 | 3.65E-06 |
| ebi-a-GCST90016921 | Gut microbiota abundance (class Mollicutes id.3920) || id:ebi-a-GCST90016921 | CD25 on IgD+ CD38- naive B cell || id:ebi-a-GCST90001781 | rs12566890 | 1 | 61850864 | T | -0.101147 | 0.0230978 | 3.65E-06 |
| ebi-a-GCST90016921 | Gut microbiota abundance (class Mollicutes id.3920) || id:ebi-a-GCST90016921 | CD25 on IgD+ CD38+ B cell || id:ebi-a-GCST90001783 | rs12566890 | 1 | 61850864 | T | -0.101147 | 0.0230978 | 3.65E-06 |
| ebi-a-GCST90016921 | Gut microbiota abundance (class Mollicutes id.3920) || id:ebi-a-GCST90016921 | CD86 on myeloid Dendritic Cell || id:ebi-a-GCST90001903 | rs12566890 | 1 | 61850864 | T | -0.101147 | 0.0230978 | 3.65E-06 |
| ebi-a-GCST90016921 | Gut microbiota abundance (class Mollicutes id.3920) || id:ebi-a-GCST90016921 | CD33 on CD14+ monocyte || id:ebi-a-GCST90001946 | rs12566890 | 1 | 61850864 | T | -0.101147 | 0.0230978 | 3.65E-06 |
| ebi-a-GCST90016921 | Gut microbiota abundance (class Mollicutes id.3920) || id:ebi-a-GCST90016921 | CD33 on CD33+ HLA DR+ CD14dim || id:ebi-a-GCST90001947 | rs12566890 | 1 | 61850864 | T | -0.101147 | 0.0230978 | 3.65E-06 |
| ebi-a-GCST90016921 | Gut microbiota abundance (class Mollicutes id.3920) || id:ebi-a-GCST90016921 | CD33 on CD33dim HLA DR+ CD11b+ || id:ebi-a-GCST90001948 | rs12566890 | 1 | 61850864 | T | -0.101147 | 0.0230978 | 3.65E-06 |
| ebi-a-GCST90016921 | Gut microbiota abundance (class Mollicutes id.3920) || id:ebi-a-GCST90016921 | CD33 on Granulocytic Myeloid-Derived Suppressor Cells || id:ebi-a-GCST90001950 | rs12566890 | 1 | 61850864 | T | -0.101147 | 0.0230978 | 3.65E-06 |
| ebi-a-GCST90016921 | Gut microbiota abundance (class Mollicutes id.3920) || id:ebi-a-GCST90016921 | CD33 on CD66b++ myeloid cell || id:ebi-a-GCST90001951 | rs12566890 | 1 | 61850864 | T | -0.101147 | 0.0230978 | 3.65E-06 |
| ebi-a-GCST90016921 | Gut microbiota abundance (class Mollicutes id.3920) || id:ebi-a-GCST90016921 | CD33 on CD33dim HLA DR- || id:ebi-a-GCST90001953 | rs12566890 | 1 | 61850864 | T | -0.101147 | 0.0230978 | 3.65E-06 |
| ebi-a-GCST90016921 | Gut microbiota abundance (class Mollicutes id.3920) || id:ebi-a-GCST90016921 | CD33 on basophil || id:ebi-a-GCST90001954 | rs12566890 | 1 | 61850864 | T | -0.101147 | 0.0230978 | 3.65E-06 |
| ebi-a-GCST90016921 | Gut microbiota abundance (class Mollicutes id.3920) || id:ebi-a-GCST90016921 | CD33 on Immature Myeloid-Derived Suppressor Cells || id:ebi-a-GCST90001955 | rs12566890 | 1 | 61850864 | T | -0.101147 | 0.0230978 | 3.65E-06 |
| ebi-a-GCST90016921 | Gut microbiota abundance (class Mollicutes id.3920) || id:ebi-a-GCST90016921 | FSC-A on HLA DR+ CD8+ T cell || id:ebi-a-GCST90001978 | rs12566890 | 1 | 61850864 | T | -0.101147 | 0.0230978 | 3.65E-06 |
| ebi-a-GCST90016921 | Gut microbiota abundance (class Mollicutes id.3920) || id:ebi-a-GCST90016921 | HLA DR on CD14+ CD16- monocyte || id:ebi-a-GCST90001988 | rs12566890 | 1 | 61850864 | T | -0.101147 | 0.0230978 | 3.65E-06 |
| ebi-a-GCST90016921 | Gut microbiota abundance (class Mollicutes id.3920) || id:ebi-a-GCST90016921 | HLA DR on CD14+ monocyte || id:ebi-a-GCST90001991 | rs12566890 | 1 | 61850864 | T | -0.101147 | 0.0230978 | 3.65E-06 |
| ebi-a-GCST90016921 | Gut microbiota abundance (class Mollicutes id.3920) || id:ebi-a-GCST90016921 | CD16 on CD14+ CD16+ monocyte || id:ebi-a-GCST90002005 | rs12566890 | 1 | 61850864 | T | -0.101147 | 0.0230978 | 3.65E-06 |
| ebi-a-GCST90016921 | Gut microbiota abundance (class Mollicutes id.3920) || id:ebi-a-GCST90016921 | CD45 on CD33+ HLA DR+ CD14- || id:ebi-a-GCST90002042 | rs12566890 | 1 | 61850864 | T | -0.101147 | 0.0230978 | 3.65E-06 |
| ebi-a-GCST90016921 | Gut microbiota abundance (class Mollicutes id.3920) || id:ebi-a-GCST90016921 | CD8 on Natural Killer T || id:ebi-a-GCST90002059 | rs12566890 | 1 | 61850864 | T | -0.101147 | 0.0230978 | 3.65E-06 |
| ebi-a-GCST90017117 | Gut microbiota abundance (phylum Tenericutes id.3919) || id:ebi-a-GCST90017117 | Myeloid Dendritic Cell Absolute Count || id:ebi-a-GCST90001458 | rs12566890 | 1 | 61850864 | T | -0.101147 | 0.0230978 | 3.65E-06 |
| ebi-a-GCST90017117 | Gut microbiota abundance (phylum Tenericutes id.3919) || id:ebi-a-GCST90017117 | CD86+ myeloid Dendritic Cell %Dendritic Cell || id:ebi-a-GCST90001465 | rs12566890 | 1 | 61850864 | T | -0.101147 | 0.0230978 | 3.65E-06 |
| ebi-a-GCST90017117 | Gut microbiota abundance (phylum Tenericutes id.3919) || id:ebi-a-GCST90017117 | CD62L- myeloid Dendritic Cell Absolute Count || id:ebi-a-GCST90001468 | rs12566890 | 1 | 61850864 | T | -0.101147 | 0.0230978 | 3.65E-06 |
| ebi-a-GCST90017117 | Gut microbiota abundance (phylum Tenericutes id.3919) || id:ebi-a-GCST90017117 | CD62L- myeloid Dendritic Cell %Dendritic Cell || id:ebi-a-GCST90001469 | rs12566890 | 1 | 61850864 | T | -0.101147 | 0.0230978 | 3.65E-06 |
| ebi-a-GCST90017117 | Gut microbiota abundance (phylum Tenericutes id.3919) || id:ebi-a-GCST90017117 | CD62L- CD86+ myeloid Dendritic Cell Absolute Count || id:ebi-a-GCST90001472 | rs12566890 | 1 | 61850864 | T | -0.101147 | 0.0230978 | 3.65E-06 |
| ebi-a-GCST90017117 | Gut microbiota abundance (phylum Tenericutes id.3919) || id:ebi-a-GCST90017117 | CD62L- CD86+ myeloid Dendritic Cell %Dendritic Cell || id:ebi-a-GCST90001473 | rs12566890 | 1 | 61850864 | T | -0.101147 | 0.0230978 | 3.65E-06 |
| ebi-a-GCST90017117 | Gut microbiota abundance (phylum Tenericutes id.3919) || id:ebi-a-GCST90017117 | HLA DR++ monocyte %monocyte || id:ebi-a-GCST90001475 | rs12566890 | 1 | 61850864 | T | -0.101147 | 0.0230978 | 3.65E-06 |
| ebi-a-GCST90017117 | Gut microbiota abundance (phylum Tenericutes id.3919) || id:ebi-a-GCST90017117 | HLA DR++ monocyte Absolute Count || id:ebi-a-GCST90001477 | rs12566890 | 1 | 61850864 | T | -0.101147 | 0.0230978 | 3.65E-06 |
| ebi-a-GCST90017117 | Gut microbiota abundance (phylum Tenericutes id.3919) || id:ebi-a-GCST90017117 | Basophil %CD33dim HLA DR- CD66b- || id:ebi-a-GCST90001533 | rs12566890 | 1 | 61850864 | T | -0.101147 | 0.0230978 | 3.65E-06 |
| ebi-a-GCST90017117 | Gut microbiota abundance (phylum Tenericutes id.3919) || id:ebi-a-GCST90017117 | CD16+ monocyte %monocyte || id:ebi-a-GCST90001587 | rs12566890 | 1 | 61850864 | T | -0.101147 | 0.0230978 | 3.65E-06 |
| ebi-a-GCST90017117 | Gut microbiota abundance (phylum Tenericutes id.3919) || id:ebi-a-GCST90017117 | T/B cell || id:ebi-a-GCST90001588 | rs12566890 | 1 | 61850864 | T | -0.101147 | 0.0230978 | 3.65E-06 |
| ebi-a-GCST90017117 | Gut microbiota abundance (phylum Tenericutes id.3919) || id:ebi-a-GCST90017117 | CD4+CD8+ T cell %T cell || id:ebi-a-GCST90001595 | rs12566890 | 1 | 61850864 | T | -0.101147 | 0.0230978 | 3.65E-06 |
| ebi-a-GCST90017117 | Gut microbiota abundance (phylum Tenericutes id.3919) || id:ebi-a-GCST90017117 | CD8+ Natural Killer T %T cell || id:ebi-a-GCST90001631 | rs12566890 | 1 | 61850864 | T | -0.101147 | 0.0230978 | 3.65E-06 |
| ebi-a-GCST90017117 | Gut microbiota abundance (phylum Tenericutes id.3919) || id:ebi-a-GCST90017117 | CD4-CD8- Natural Killer T %lymphocyte || id:ebi-a-GCST90001638 | rs12566890 | 1 | 61850864 | T | -0.101147 | 0.0230978 | 3.65E-06 |
| ebi-a-GCST90017117 | Gut microbiota abundance (phylum Tenericutes id.3919) || id:ebi-a-GCST90017117 | HLA DR+ Natural Killer Absolute Count || id:ebi-a-GCST90001648 | rs12566890 | 1 | 61850864 | T | -0.101147 | 0.0230978 | 3.65E-06 |
| ebi-a-GCST90017117 | Gut microbiota abundance (phylum Tenericutes id.3919) || id:ebi-a-GCST90017117 | HLA DR+ Natural Killer %Natural Killer || id:ebi-a-GCST90001649 | rs12566890 | 1 | 61850864 | T | -0.101147 | 0.0230978 | 3.65E-06 |
| ebi-a-GCST90017117 | Gut microbiota abundance (phylum Tenericutes id.3919) || id:ebi-a-GCST90017117 | HLA DR+ Natural Killer %CD3- lymphocyte || id:ebi-a-GCST90001650 | rs12566890 | 1 | 61850864 | T | -0.101147 | 0.0230978 | 3.65E-06 |
| ebi-a-GCST90017117 | Gut microbiota abundance (phylum Tenericutes id.3919) || id:ebi-a-GCST90017117 | CD28- CD25++ CD8+ T cell Absolute Count || id:ebi-a-GCST90001678 | rs12566890 | 1 | 61850864 | T | -0.101147 | 0.0230978 | 3.65E-06 |
| ebi-a-GCST90017117 | Gut microbiota abundance (phylum Tenericutes id.3919) || id:ebi-a-GCST90017117 | CD25++ CD8+ T cell %T cell || id:ebi-a-GCST90001679 | rs12566890 | 1 | 61850864 | T | -0.101147 | 0.0230978 | 3.65E-06 |
| ebi-a-GCST90017117 | Gut microbiota abundance (phylum Tenericutes id.3919) || id:ebi-a-GCST90017117 | CD19 on CD20- CD38- B cell || id:ebi-a-GCST90001722 | rs12566890 | 1 | 61850864 | T | -0.101147 | 0.0230978 | 3.65E-06 |
| ebi-a-GCST90017117 | Gut microbiota abundance (phylum Tenericutes id.3919) || id:ebi-a-GCST90017117 | CD25 on B cell || id:ebi-a-GCST90001775 | rs12566890 | 1 | 61850864 | T | -0.101147 | 0.0230978 | 3.65E-06 |
| ebi-a-GCST90017117 | Gut microbiota abundance (phylum Tenericutes id.3919) || id:ebi-a-GCST90017117 | CD25 on IgD+ CD24- B cell || id:ebi-a-GCST90001779 | rs12566890 | 1 | 61850864 | T | -0.101147 | 0.0230978 | 3.65E-06 |
| ebi-a-GCST90017117 | Gut microbiota abundance (phylum Tenericutes id.3919) || id:ebi-a-GCST90017117 | CD25 on IgD+ CD38- naive B cell || id:ebi-a-GCST90001781 | rs12566890 | 1 | 61850864 | T | -0.101147 | 0.0230978 | 3.65E-06 |
| ebi-a-GCST90017117 | Gut microbiota abundance (phylum Tenericutes id.3919) || id:ebi-a-GCST90017117 | CD25 on IgD+ CD38+ B cell || id:ebi-a-GCST90001783 | rs12566890 | 1 | 61850864 | T | -0.101147 | 0.0230978 | 3.65E-06 |
| ebi-a-GCST90017117 | Gut microbiota abundance (phylum Tenericutes id.3919) || id:ebi-a-GCST90017117 | CD86 on myeloid Dendritic Cell || id:ebi-a-GCST90001903 | rs12566890 | 1 | 61850864 | T | -0.101147 | 0.0230978 | 3.65E-06 |
| ebi-a-GCST90017117 | Gut microbiota abundance (phylum Tenericutes id.3919) || id:ebi-a-GCST90017117 | CD33 on CD14+ monocyte || id:ebi-a-GCST90001946 | rs12566890 | 1 | 61850864 | T | -0.101147 | 0.0230978 | 3.65E-06 |
| ebi-a-GCST90017117 | Gut microbiota abundance (phylum Tenericutes id.3919) || id:ebi-a-GCST90017117 | CD33 on CD33+ HLA DR+ CD14dim || id:ebi-a-GCST90001947 | rs12566890 | 1 | 61850864 | T | -0.101147 | 0.0230978 | 3.65E-06 |
| ebi-a-GCST90017117 | Gut microbiota abundance (phylum Tenericutes id.3919) || id:ebi-a-GCST90017117 | CD33 on CD33dim HLA DR+ CD11b+ || id:ebi-a-GCST90001948 | rs12566890 | 1 | 61850864 | T | -0.101147 | 0.0230978 | 3.65E-06 |
| ebi-a-GCST90017117 | Gut microbiota abundance (phylum Tenericutes id.3919) || id:ebi-a-GCST90017117 | CD33 on Granulocytic Myeloid-Derived Suppressor Cells || id:ebi-a-GCST90001950 | rs12566890 | 1 | 61850864 | T | -0.101147 | 0.0230978 | 3.65E-06 |
| ebi-a-GCST90017117 | Gut microbiota abundance (phylum Tenericutes id.3919) || id:ebi-a-GCST90017117 | CD33 on CD66b++ myeloid cell || id:ebi-a-GCST90001951 | rs12566890 | 1 | 61850864 | T | -0.101147 | 0.0230978 | 3.65E-06 |
| ebi-a-GCST90017117 | Gut microbiota abundance (phylum Tenericutes id.3919) || id:ebi-a-GCST90017117 | CD33 on CD33dim HLA DR- || id:ebi-a-GCST90001953 | rs12566890 | 1 | 61850864 | T | -0.101147 | 0.0230978 | 3.65E-06 |
| ebi-a-GCST90017117 | Gut microbiota abundance (phylum Tenericutes id.3919) || id:ebi-a-GCST90017117 | CD33 on basophil || id:ebi-a-GCST90001954 | rs12566890 | 1 | 61850864 | T | -0.101147 | 0.0230978 | 3.65E-06 |
| ebi-a-GCST90017117 | Gut microbiota abundance (phylum Tenericutes id.3919) || id:ebi-a-GCST90017117 | CD33 on Immature Myeloid-Derived Suppressor Cells || id:ebi-a-GCST90001955 | rs12566890 | 1 | 61850864 | T | -0.101147 | 0.0230978 | 3.65E-06 |
| ebi-a-GCST90017117 | Gut microbiota abundance (phylum Tenericutes id.3919) || id:ebi-a-GCST90017117 | FSC-A on HLA DR+ CD8+ T cell || id:ebi-a-GCST90001978 | rs12566890 | 1 | 61850864 | T | -0.101147 | 0.0230978 | 3.65E-06 |
| ebi-a-GCST90017117 | Gut microbiota abundance (phylum Tenericutes id.3919) || id:ebi-a-GCST90017117 | HLA DR on CD14+ CD16- monocyte || id:ebi-a-GCST90001988 | rs12566890 | 1 | 61850864 | T | -0.101147 | 0.0230978 | 3.65E-06 |
| ebi-a-GCST90017117 | Gut microbiota abundance (phylum Tenericutes id.3919) || id:ebi-a-GCST90017117 | HLA DR on CD14+ monocyte || id:ebi-a-GCST90001991 | rs12566890 | 1 | 61850864 | T | -0.101147 | 0.0230978 | 3.65E-06 |
| ebi-a-GCST90017117 | Gut microbiota abundance (phylum Tenericutes id.3919) || id:ebi-a-GCST90017117 | CD16 on CD14+ CD16+ monocyte || id:ebi-a-GCST90002005 | rs12566890 | 1 | 61850864 | T | -0.101147 | 0.0230978 | 3.65E-06 |
| ebi-a-GCST90017117 | Gut microbiota abundance (phylum Tenericutes id.3919) || id:ebi-a-GCST90017117 | CD45 on CD33+ HLA DR+ CD14- || id:ebi-a-GCST90002042 | rs12566890 | 1 | 61850864 | T | -0.101147 | 0.0230978 | 3.65E-06 |
| ebi-a-GCST90017117 | Gut microbiota abundance (phylum Tenericutes id.3919) || id:ebi-a-GCST90017117 | CD8 on Natural Killer T || id:ebi-a-GCST90002059 | rs12566890 | 1 | 61850864 | T | -0.101147 | 0.0230978 | 3.65E-06 |
| ebi-a-GCST90016957 | Gut microbiota abundance (family Verrucomicrobiaceae id.4036) || id:ebi-a-GCST90016957 | Myeloid Dendritic Cell Absolute Count || id:ebi-a-GCST90001458 | rs12908520 | 15 | 97570657 | G | 0.0619104 | 0.0130946 | 2.15E-06 |
| ebi-a-GCST90016957 | Gut microbiota abundance (family Verrucomicrobiaceae id.4036) || id:ebi-a-GCST90016957 | CD86+ myeloid Dendritic Cell %Dendritic Cell || id:ebi-a-GCST90001465 | rs12908520 | 15 | 97570657 | G | 0.0619104 | 0.0130946 | 2.15E-06 |
| ebi-a-GCST90016957 | Gut microbiota abundance (family Verrucomicrobiaceae id.4036) || id:ebi-a-GCST90016957 | CD62L- myeloid Dendritic Cell Absolute Count || id:ebi-a-GCST90001468 | rs12908520 | 15 | 97570657 | G | 0.0619104 | 0.0130946 | 2.15E-06 |
| ebi-a-GCST90016957 | Gut microbiota abundance (family Verrucomicrobiaceae id.4036) || id:ebi-a-GCST90016957 | CD62L- myeloid Dendritic Cell %Dendritic Cell || id:ebi-a-GCST90001469 | rs12908520 | 15 | 97570657 | G | 0.0619104 | 0.0130946 | 2.15E-06 |
| ebi-a-GCST90016957 | Gut microbiota abundance (family Verrucomicrobiaceae id.4036) || id:ebi-a-GCST90016957 | CD62L- CD86+ myeloid Dendritic Cell Absolute Count || id:ebi-a-GCST90001472 | rs12908520 | 15 | 97570657 | G | 0.0619104 | 0.0130946 | 2.15E-06 |
| ebi-a-GCST90016957 | Gut microbiota abundance (family Verrucomicrobiaceae id.4036) || id:ebi-a-GCST90016957 | CD62L- CD86+ myeloid Dendritic Cell %Dendritic Cell || id:ebi-a-GCST90001473 | rs12908520 | 15 | 97570657 | G | 0.0619104 | 0.0130946 | 2.15E-06 |
| ebi-a-GCST90016957 | Gut microbiota abundance (family Verrucomicrobiaceae id.4036) || id:ebi-a-GCST90016957 | HLA DR++ monocyte %monocyte || id:ebi-a-GCST90001475 | rs12908520 | 15 | 97570657 | G | 0.0619104 | 0.0130946 | 2.15E-06 |
| ebi-a-GCST90016957 | Gut microbiota abundance (family Verrucomicrobiaceae id.4036) || id:ebi-a-GCST90016957 | HLA DR++ monocyte Absolute Count || id:ebi-a-GCST90001477 | rs12908520 | 15 | 97570657 | G | 0.0619104 | 0.0130946 | 2.15E-06 |
| ebi-a-GCST90016957 | Gut microbiota abundance (family Verrucomicrobiaceae id.4036) || id:ebi-a-GCST90016957 | Basophil %CD33dim HLA DR- CD66b- || id:ebi-a-GCST90001533 | rs12908520 | 15 | 97570657 | G | 0.0619104 | 0.0130946 | 2.15E-06 |
| ebi-a-GCST90016957 | Gut microbiota abundance (family Verrucomicrobiaceae id.4036) || id:ebi-a-GCST90016957 | CD16+ monocyte %monocyte || id:ebi-a-GCST90001587 | rs12908520 | 15 | 97570657 | G | 0.0619104 | 0.0130946 | 2.15E-06 |
| ebi-a-GCST90016957 | Gut microbiota abundance (family Verrucomicrobiaceae id.4036) || id:ebi-a-GCST90016957 | T/B cell || id:ebi-a-GCST90001588 | rs12908520 | 15 | 97570657 | G | 0.0619104 | 0.0130946 | 2.15E-06 |
| ebi-a-GCST90016957 | Gut microbiota abundance (family Verrucomicrobiaceae id.4036) || id:ebi-a-GCST90016957 | CD8+ Natural Killer T %T cell || id:ebi-a-GCST90001631 | rs12908520 | 15 | 97570657 | G | 0.0619104 | 0.0130946 | 2.15E-06 |
| ebi-a-GCST90016957 | Gut microbiota abundance (family Verrucomicrobiaceae id.4036) || id:ebi-a-GCST90016957 | CD4-CD8- Natural Killer T %lymphocyte || id:ebi-a-GCST90001638 | rs12908520 | 15 | 97570657 | G | 0.0619104 | 0.0130946 | 2.15E-06 |
| ebi-a-GCST90016957 | Gut microbiota abundance (family Verrucomicrobiaceae id.4036) || id:ebi-a-GCST90016957 | HLA DR+ Natural Killer Absolute Count || id:ebi-a-GCST90001648 | rs12908520 | 15 | 97570657 | G | 0.0619104 | 0.0130946 | 2.15E-06 |
| ebi-a-GCST90016957 | Gut microbiota abundance (family Verrucomicrobiaceae id.4036) || id:ebi-a-GCST90016957 | HLA DR+ Natural Killer %Natural Killer || id:ebi-a-GCST90001649 | rs12908520 | 15 | 97570657 | G | 0.0619104 | 0.0130946 | 2.15E-06 |
| ebi-a-GCST90016957 | Gut microbiota abundance (family Verrucomicrobiaceae id.4036) || id:ebi-a-GCST90016957 | HLA DR+ Natural Killer %CD3- lymphocyte || id:ebi-a-GCST90001650 | rs12908520 | 15 | 97570657 | G | 0.0619104 | 0.0130946 | 2.15E-06 |
| ebi-a-GCST90016957 | Gut microbiota abundance (family Verrucomicrobiaceae id.4036) || id:ebi-a-GCST90016957 | CD28- CD25++ CD8+ T cell Absolute Count || id:ebi-a-GCST90001678 | rs12908520 | 15 | 97570657 | G | 0.0619104 | 0.0130946 | 2.15E-06 |
| ebi-a-GCST90016957 | Gut microbiota abundance (family Verrucomicrobiaceae id.4036) || id:ebi-a-GCST90016957 | CD25++ CD8+ T cell %T cell || id:ebi-a-GCST90001679 | rs12908520 | 15 | 97570657 | G | 0.0619104 | 0.0130946 | 2.15E-06 |
| ebi-a-GCST90016957 | Gut microbiota abundance (family Verrucomicrobiaceae id.4036) || id:ebi-a-GCST90016957 | CD19 on CD20- CD38- B cell || id:ebi-a-GCST90001722 | rs12908520 | 15 | 97570657 | G | 0.0619104 | 0.0130946 | 2.15E-06 |
| ebi-a-GCST90016957 | Gut microbiota abundance (family Verrucomicrobiaceae id.4036) || id:ebi-a-GCST90016957 | CD25 on B cell || id:ebi-a-GCST90001775 | rs12908520 | 15 | 97570657 | G | 0.0619104 | 0.0130946 | 2.15E-06 |
| ebi-a-GCST90016957 | Gut microbiota abundance (family Verrucomicrobiaceae id.4036) || id:ebi-a-GCST90016957 | CD25 on IgD+ CD24- B cell || id:ebi-a-GCST90001779 | rs12908520 | 15 | 97570657 | G | 0.0619104 | 0.0130946 | 2.15E-06 |
| ebi-a-GCST90016957 | Gut microbiota abundance (family Verrucomicrobiaceae id.4036) || id:ebi-a-GCST90016957 | CD25 on IgD+ CD38- naive B cell || id:ebi-a-GCST90001781 | rs12908520 | 15 | 97570657 | G | 0.0619104 | 0.0130946 | 2.15E-06 |
| ebi-a-GCST90016957 | Gut microbiota abundance (family Verrucomicrobiaceae id.4036) || id:ebi-a-GCST90016957 | CD25 on IgD+ CD38+ B cell || id:ebi-a-GCST90001783 | rs12908520 | 15 | 97570657 | G | 0.0619104 | 0.0130946 | 2.15E-06 |
| ebi-a-GCST90016957 | Gut microbiota abundance (family Verrucomicrobiaceae id.4036) || id:ebi-a-GCST90016957 | CD86 on myeloid Dendritic Cell || id:ebi-a-GCST90001903 | rs12908520 | 15 | 97570657 | G | 0.0619104 | 0.0130946 | 2.15E-06 |
| ebi-a-GCST90016957 | Gut microbiota abundance (family Verrucomicrobiaceae id.4036) || id:ebi-a-GCST90016957 | CD33 on CD14+ monocyte || id:ebi-a-GCST90001946 | rs12908520 | 15 | 97570657 | G | 0.0619104 | 0.0130946 | 2.15E-06 |
| ebi-a-GCST90016957 | Gut microbiota abundance (family Verrucomicrobiaceae id.4036) || id:ebi-a-GCST90016957 | CD33 on CD33+ HLA DR+ CD14dim || id:ebi-a-GCST90001947 | rs12908520 | 15 | 97570657 | G | 0.0619104 | 0.0130946 | 2.15E-06 |
| ebi-a-GCST90016957 | Gut microbiota abundance (family Verrucomicrobiaceae id.4036) || id:ebi-a-GCST90016957 | CD33 on CD33dim HLA DR+ CD11b+ || id:ebi-a-GCST90001948 | rs12908520 | 15 | 97570657 | G | 0.0619104 | 0.0130946 | 2.15E-06 |
| ebi-a-GCST90016957 | Gut microbiota abundance (family Verrucomicrobiaceae id.4036) || id:ebi-a-GCST90016957 | CD33 on Granulocytic Myeloid-Derived Suppressor Cells || id:ebi-a-GCST90001950 | rs12908520 | 15 | 97570657 | G | 0.0619104 | 0.0130946 | 2.15E-06 |
| ebi-a-GCST90016957 | Gut microbiota abundance (family Verrucomicrobiaceae id.4036) || id:ebi-a-GCST90016957 | CD33 on CD66b++ myeloid cell || id:ebi-a-GCST90001951 | rs12908520 | 15 | 97570657 | G | 0.0619104 | 0.0130946 | 2.15E-06 |
| ebi-a-GCST90016957 | Gut microbiota abundance (family Verrucomicrobiaceae id.4036) || id:ebi-a-GCST90016957 | CD33 on CD33dim HLA DR- || id:ebi-a-GCST90001953 | rs12908520 | 15 | 97570657 | G | 0.0619104 | 0.0130946 | 2.15E-06 |
| ebi-a-GCST90016957 | Gut microbiota abundance (family Verrucomicrobiaceae id.4036) || id:ebi-a-GCST90016957 | CD33 on basophil || id:ebi-a-GCST90001954 | rs12908520 | 15 | 97570657 | G | 0.0619104 | 0.0130946 | 2.15E-06 |
| ebi-a-GCST90016957 | Gut microbiota abundance (family Verrucomicrobiaceae id.4036) || id:ebi-a-GCST90016957 | CD33 on Immature Myeloid-Derived Suppressor Cells || id:ebi-a-GCST90001955 | rs12908520 | 15 | 97570657 | G | 0.0619104 | 0.0130946 | 2.15E-06 |
| ebi-a-GCST90016957 | Gut microbiota abundance (family Verrucomicrobiaceae id.4036) || id:ebi-a-GCST90016957 | FSC-A on HLA DR+ CD8+ T cell || id:ebi-a-GCST90001978 | rs12908520 | 15 | 97570657 | G | 0.0619104 | 0.0130946 | 2.15E-06 |
| ebi-a-GCST90016957 | Gut microbiota abundance (family Verrucomicrobiaceae id.4036) || id:ebi-a-GCST90016957 | HLA DR on CD14+ CD16- monocyte || id:ebi-a-GCST90001988 | rs12908520 | 15 | 97570657 | G | 0.0619104 | 0.0130946 | 2.15E-06 |
| ebi-a-GCST90016957 | Gut microbiota abundance (family Verrucomicrobiaceae id.4036) || id:ebi-a-GCST90016957 | HLA DR on CD14+ monocyte || id:ebi-a-GCST90001991 | rs12908520 | 15 | 97570657 | G | 0.0619104 | 0.0130946 | 2.15E-06 |
| ebi-a-GCST90016957 | Gut microbiota abundance (family Verrucomicrobiaceae id.4036) || id:ebi-a-GCST90016957 | CD16 on CD14+ CD16+ monocyte || id:ebi-a-GCST90002005 | rs12908520 | 15 | 97570657 | G | 0.0619104 | 0.0130946 | 2.15E-06 |
| ebi-a-GCST90016957 | Gut microbiota abundance (family Verrucomicrobiaceae id.4036) || id:ebi-a-GCST90016957 | CD45 on CD33+ HLA DR+ CD14- || id:ebi-a-GCST90002042 | rs12908520 | 15 | 97570657 | G | 0.0619104 | 0.0130946 | 2.15E-06 |
| ebi-a-GCST90016957 | Gut microbiota abundance (family Verrucomicrobiaceae id.4036) || id:ebi-a-GCST90016957 | CD8 on Natural Killer T || id:ebi-a-GCST90002059 | rs12908520 | 15 | 97570657 | G | 0.0619104 | 0.0130946 | 2.15E-06 |
| ebi-a-GCST90016923 | Gut microbiota abundance (class Verrucomicrobiae id.4029) || id:ebi-a-GCST90016923 | Myeloid Dendritic Cell Absolute Count || id:ebi-a-GCST90001458 | rs12908520 | 15 | 97570657 | G | 0.0618926 | 0.0130946 | 2.17E-06 |
| ebi-a-GCST90016923 | Gut microbiota abundance (class Verrucomicrobiae id.4029) || id:ebi-a-GCST90016923 | CD86+ myeloid Dendritic Cell %Dendritic Cell || id:ebi-a-GCST90001465 | rs12908520 | 15 | 97570657 | G | 0.0618926 | 0.0130946 | 2.17E-06 |
| ebi-a-GCST90016923 | Gut microbiota abundance (class Verrucomicrobiae id.4029) || id:ebi-a-GCST90016923 | CD62L- myeloid Dendritic Cell Absolute Count || id:ebi-a-GCST90001468 | rs12908520 | 15 | 97570657 | G | 0.0618926 | 0.0130946 | 2.17E-06 |
| ebi-a-GCST90016923 | Gut microbiota abundance (class Verrucomicrobiae id.4029) || id:ebi-a-GCST90016923 | CD62L- myeloid Dendritic Cell %Dendritic Cell || id:ebi-a-GCST90001469 | rs12908520 | 15 | 97570657 | G | 0.0618926 | 0.0130946 | 2.17E-06 |
| ebi-a-GCST90016923 | Gut microbiota abundance (class Verrucomicrobiae id.4029) || id:ebi-a-GCST90016923 | CD62L- CD86+ myeloid Dendritic Cell Absolute Count || id:ebi-a-GCST90001472 | rs12908520 | 15 | 97570657 | G | 0.0618926 | 0.0130946 | 2.17E-06 |
| ebi-a-GCST90016923 | Gut microbiota abundance (class Verrucomicrobiae id.4029) || id:ebi-a-GCST90016923 | CD62L- CD86+ myeloid Dendritic Cell %Dendritic Cell || id:ebi-a-GCST90001473 | rs12908520 | 15 | 97570657 | G | 0.0618926 | 0.0130946 | 2.17E-06 |
| ebi-a-GCST90016923 | Gut microbiota abundance (class Verrucomicrobiae id.4029) || id:ebi-a-GCST90016923 | HLA DR++ monocyte %monocyte || id:ebi-a-GCST90001475 | rs12908520 | 15 | 97570657 | G | 0.0618926 | 0.0130946 | 2.17E-06 |
| ebi-a-GCST90016923 | Gut microbiota abundance (class Verrucomicrobiae id.4029) || id:ebi-a-GCST90016923 | HLA DR++ monocyte Absolute Count || id:ebi-a-GCST90001477 | rs12908520 | 15 | 97570657 | G | 0.0618926 | 0.0130946 | 2.17E-06 |
| ebi-a-GCST90016923 | Gut microbiota abundance (class Verrucomicrobiae id.4029) || id:ebi-a-GCST90016923 | Basophil %CD33dim HLA DR- CD66b- || id:ebi-a-GCST90001533 | rs12908520 | 15 | 97570657 | G | 0.0618926 | 0.0130946 | 2.17E-06 |
| ebi-a-GCST90016923 | Gut microbiota abundance (class Verrucomicrobiae id.4029) || id:ebi-a-GCST90016923 | CD16+ monocyte %monocyte || id:ebi-a-GCST90001587 | rs12908520 | 15 | 97570657 | G | 0.0618926 | 0.0130946 | 2.17E-06 |
| ebi-a-GCST90016923 | Gut microbiota abundance (class Verrucomicrobiae id.4029) || id:ebi-a-GCST90016923 | T/B cell || id:ebi-a-GCST90001588 | rs12908520 | 15 | 97570657 | G | 0.0618926 | 0.0130946 | 2.17E-06 |
| ebi-a-GCST90016923 | Gut microbiota abundance (class Verrucomicrobiae id.4029) || id:ebi-a-GCST90016923 | CD8+ Natural Killer T %T cell || id:ebi-a-GCST90001631 | rs12908520 | 15 | 97570657 | G | 0.0618926 | 0.0130946 | 2.17E-06 |
| ebi-a-GCST90016923 | Gut microbiota abundance (class Verrucomicrobiae id.4029) || id:ebi-a-GCST90016923 | CD4-CD8- Natural Killer T %lymphocyte || id:ebi-a-GCST90001638 | rs12908520 | 15 | 97570657 | G | 0.0618926 | 0.0130946 | 2.17E-06 |
| ebi-a-GCST90016923 | Gut microbiota abundance (class Verrucomicrobiae id.4029) || id:ebi-a-GCST90016923 | HLA DR+ Natural Killer Absolute Count || id:ebi-a-GCST90001648 | rs12908520 | 15 | 97570657 | G | 0.0618926 | 0.0130946 | 2.17E-06 |
| ebi-a-GCST90016923 | Gut microbiota abundance (class Verrucomicrobiae id.4029) || id:ebi-a-GCST90016923 | HLA DR+ Natural Killer %Natural Killer || id:ebi-a-GCST90001649 | rs12908520 | 15 | 97570657 | G | 0.0618926 | 0.0130946 | 2.17E-06 |
| ebi-a-GCST90016923 | Gut microbiota abundance (class Verrucomicrobiae id.4029) || id:ebi-a-GCST90016923 | HLA DR+ Natural Killer %CD3- lymphocyte || id:ebi-a-GCST90001650 | rs12908520 | 15 | 97570657 | G | 0.0618926 | 0.0130946 | 2.17E-06 |
| ebi-a-GCST90016923 | Gut microbiota abundance (class Verrucomicrobiae id.4029) || id:ebi-a-GCST90016923 | CD28- CD25++ CD8+ T cell Absolute Count || id:ebi-a-GCST90001678 | rs12908520 | 15 | 97570657 | G | 0.0618926 | 0.0130946 | 2.17E-06 |
| ebi-a-GCST90016923 | Gut microbiota abundance (class Verrucomicrobiae id.4029) || id:ebi-a-GCST90016923 | CD25++ CD8+ T cell %T cell || id:ebi-a-GCST90001679 | rs12908520 | 15 | 97570657 | G | 0.0618926 | 0.0130946 | 2.17E-06 |
| ebi-a-GCST90016923 | Gut microbiota abundance (class Verrucomicrobiae id.4029) || id:ebi-a-GCST90016923 | CD19 on CD20- CD38- B cell || id:ebi-a-GCST90001722 | rs12908520 | 15 | 97570657 | G | 0.0618926 | 0.0130946 | 2.17E-06 |
| ebi-a-GCST90016923 | Gut microbiota abundance (class Verrucomicrobiae id.4029) || id:ebi-a-GCST90016923 | CD25 on B cell || id:ebi-a-GCST90001775 | rs12908520 | 15 | 97570657 | G | 0.0618926 | 0.0130946 | 2.17E-06 |
| ebi-a-GCST90016923 | Gut microbiota abundance (class Verrucomicrobiae id.4029) || id:ebi-a-GCST90016923 | CD25 on IgD+ CD24- B cell || id:ebi-a-GCST90001779 | rs12908520 | 15 | 97570657 | G | 0.0618926 | 0.0130946 | 2.17E-06 |
| ebi-a-GCST90016923 | Gut microbiota abundance (class Verrucomicrobiae id.4029) || id:ebi-a-GCST90016923 | CD25 on IgD+ CD38- naive B cell || id:ebi-a-GCST90001781 | rs12908520 | 15 | 97570657 | G | 0.0618926 | 0.0130946 | 2.17E-06 |
| ebi-a-GCST90016923 | Gut microbiota abundance (class Verrucomicrobiae id.4029) || id:ebi-a-GCST90016923 | CD25 on IgD+ CD38+ B cell || id:ebi-a-GCST90001783 | rs12908520 | 15 | 97570657 | G | 0.0618926 | 0.0130946 | 2.17E-06 |
| ebi-a-GCST90016923 | Gut microbiota abundance (class Verrucomicrobiae id.4029) || id:ebi-a-GCST90016923 | CD86 on myeloid Dendritic Cell || id:ebi-a-GCST90001903 | rs12908520 | 15 | 97570657 | G | 0.0618926 | 0.0130946 | 2.17E-06 |
| ebi-a-GCST90016923 | Gut microbiota abundance (class Verrucomicrobiae id.4029) || id:ebi-a-GCST90016923 | CD33 on CD14+ monocyte || id:ebi-a-GCST90001946 | rs12908520 | 15 | 97570657 | G | 0.0618926 | 0.0130946 | 2.17E-06 |
| ebi-a-GCST90016923 | Gut microbiota abundance (class Verrucomicrobiae id.4029) || id:ebi-a-GCST90016923 | CD33 on CD33+ HLA DR+ CD14dim || id:ebi-a-GCST90001947 | rs12908520 | 15 | 97570657 | G | 0.0618926 | 0.0130946 | 2.17E-06 |
| ebi-a-GCST90016923 | Gut microbiota abundance (class Verrucomicrobiae id.4029) || id:ebi-a-GCST90016923 | CD33 on CD33dim HLA DR+ CD11b+ || id:ebi-a-GCST90001948 | rs12908520 | 15 | 97570657 | G | 0.0618926 | 0.0130946 | 2.17E-06 |
| ebi-a-GCST90016923 | Gut microbiota abundance (class Verrucomicrobiae id.4029) || id:ebi-a-GCST90016923 | CD33 on Granulocytic Myeloid-Derived Suppressor Cells || id:ebi-a-GCST90001950 | rs12908520 | 15 | 97570657 | G | 0.0618926 | 0.0130946 | 2.17E-06 |
| ebi-a-GCST90016923 | Gut microbiota abundance (class Verrucomicrobiae id.4029) || id:ebi-a-GCST90016923 | CD33 on CD66b++ myeloid cell || id:ebi-a-GCST90001951 | rs12908520 | 15 | 97570657 | G | 0.0618926 | 0.0130946 | 2.17E-06 |
| ebi-a-GCST90016923 | Gut microbiota abundance (class Verrucomicrobiae id.4029) || id:ebi-a-GCST90016923 | CD33 on CD33dim HLA DR- || id:ebi-a-GCST90001953 | rs12908520 | 15 | 97570657 | G | 0.0618926 | 0.0130946 | 2.17E-06 |
| ebi-a-GCST90016923 | Gut microbiota abundance (class Verrucomicrobiae id.4029) || id:ebi-a-GCST90016923 | CD33 on basophil || id:ebi-a-GCST90001954 | rs12908520 | 15 | 97570657 | G | 0.0618926 | 0.0130946 | 2.17E-06 |
| ebi-a-GCST90016923 | Gut microbiota abundance (class Verrucomicrobiae id.4029) || id:ebi-a-GCST90016923 | CD33 on Immature Myeloid-Derived Suppressor Cells || id:ebi-a-GCST90001955 | rs12908520 | 15 | 97570657 | G | 0.0618926 | 0.0130946 | 2.17E-06 |
| ebi-a-GCST90016923 | Gut microbiota abundance (class Verrucomicrobiae id.4029) || id:ebi-a-GCST90016923 | FSC-A on HLA DR+ CD8+ T cell || id:ebi-a-GCST90001978 | rs12908520 | 15 | 97570657 | G | 0.0618926 | 0.0130946 | 2.17E-06 |
| ebi-a-GCST90016923 | Gut microbiota abundance (class Verrucomicrobiae id.4029) || id:ebi-a-GCST90016923 | HLA DR on CD14+ CD16- monocyte || id:ebi-a-GCST90001988 | rs12908520 | 15 | 97570657 | G | 0.0618926 | 0.0130946 | 2.17E-06 |
| ebi-a-GCST90016923 | Gut microbiota abundance (class Verrucomicrobiae id.4029) || id:ebi-a-GCST90016923 | HLA DR on CD14+ monocyte || id:ebi-a-GCST90001991 | rs12908520 | 15 | 97570657 | G | 0.0618926 | 0.0130946 | 2.17E-06 |
| ebi-a-GCST90016923 | Gut microbiota abundance (class Verrucomicrobiae id.4029) || id:ebi-a-GCST90016923 | CD16 on CD14+ CD16+ monocyte || id:ebi-a-GCST90002005 | rs12908520 | 15 | 97570657 | G | 0.0618926 | 0.0130946 | 2.17E-06 |
| ebi-a-GCST90016923 | Gut microbiota abundance (class Verrucomicrobiae id.4029) || id:ebi-a-GCST90016923 | CD45 on CD33+ HLA DR+ CD14- || id:ebi-a-GCST90002042 | rs12908520 | 15 | 97570657 | G | 0.0618926 | 0.0130946 | 2.17E-06 |
| ebi-a-GCST90016923 | Gut microbiota abundance (class Verrucomicrobiae id.4029) || id:ebi-a-GCST90016923 | CD8 on Natural Killer T || id:ebi-a-GCST90002059 | rs12908520 | 15 | 97570657 | G | 0.0618926 | 0.0130946 | 2.17E-06 |
| ebi-a-GCST90017108 | Gut microbiota abundance (order Verrucomicrobiales id.4030) || id:ebi-a-GCST90017108 | Myeloid Dendritic Cell Absolute Count || id:ebi-a-GCST90001458 | rs12908520 | 15 | 97570657 | G | 0.0618926 | 0.0130946 | 2.17E-06 |
| ebi-a-GCST90017108 | Gut microbiota abundance (order Verrucomicrobiales id.4030) || id:ebi-a-GCST90017108 | CD86+ myeloid Dendritic Cell %Dendritic Cell || id:ebi-a-GCST90001465 | rs12908520 | 15 | 97570657 | G | 0.0618926 | 0.0130946 | 2.17E-06 |
| ebi-a-GCST90017108 | Gut microbiota abundance (order Verrucomicrobiales id.4030) || id:ebi-a-GCST90017108 | CD62L- myeloid Dendritic Cell Absolute Count || id:ebi-a-GCST90001468 | rs12908520 | 15 | 97570657 | G | 0.0618926 | 0.0130946 | 2.17E-06 |
| ebi-a-GCST90017108 | Gut microbiota abundance (order Verrucomicrobiales id.4030) || id:ebi-a-GCST90017108 | CD62L- myeloid Dendritic Cell %Dendritic Cell || id:ebi-a-GCST90001469 | rs12908520 | 15 | 97570657 | G | 0.0618926 | 0.0130946 | 2.17E-06 |
| ebi-a-GCST90017108 | Gut microbiota abundance (order Verrucomicrobiales id.4030) || id:ebi-a-GCST90017108 | CD62L- CD86+ myeloid Dendritic Cell Absolute Count || id:ebi-a-GCST90001472 | rs12908520 | 15 | 97570657 | G | 0.0618926 | 0.0130946 | 2.17E-06 |
| ebi-a-GCST90017108 | Gut microbiota abundance (order Verrucomicrobiales id.4030) || id:ebi-a-GCST90017108 | CD62L- CD86+ myeloid Dendritic Cell %Dendritic Cell || id:ebi-a-GCST90001473 | rs12908520 | 15 | 97570657 | G | 0.0618926 | 0.0130946 | 2.17E-06 |
| ebi-a-GCST90017108 | Gut microbiota abundance (order Verrucomicrobiales id.4030) || id:ebi-a-GCST90017108 | HLA DR++ monocyte %monocyte || id:ebi-a-GCST90001475 | rs12908520 | 15 | 97570657 | G | 0.0618926 | 0.0130946 | 2.17E-06 |
| ebi-a-GCST90017108 | Gut microbiota abundance (order Verrucomicrobiales id.4030) || id:ebi-a-GCST90017108 | HLA DR++ monocyte Absolute Count || id:ebi-a-GCST90001477 | rs12908520 | 15 | 97570657 | G | 0.0618926 | 0.0130946 | 2.17E-06 |
| ebi-a-GCST90017108 | Gut microbiota abundance (order Verrucomicrobiales id.4030) || id:ebi-a-GCST90017108 | Basophil %CD33dim HLA DR- CD66b- || id:ebi-a-GCST90001533 | rs12908520 | 15 | 97570657 | G | 0.0618926 | 0.0130946 | 2.17E-06 |
| ebi-a-GCST90017108 | Gut microbiota abundance (order Verrucomicrobiales id.4030) || id:ebi-a-GCST90017108 | CD16+ monocyte %monocyte || id:ebi-a-GCST90001587 | rs12908520 | 15 | 97570657 | G | 0.0618926 | 0.0130946 | 2.17E-06 |
| ebi-a-GCST90017108 | Gut microbiota abundance (order Verrucomicrobiales id.4030) || id:ebi-a-GCST90017108 | T/B cell || id:ebi-a-GCST90001588 | rs12908520 | 15 | 97570657 | G | 0.0618926 | 0.0130946 | 2.17E-06 |
| ebi-a-GCST90017108 | Gut microbiota abundance (order Verrucomicrobiales id.4030) || id:ebi-a-GCST90017108 | CD8+ Natural Killer T %T cell || id:ebi-a-GCST90001631 | rs12908520 | 15 | 97570657 | G | 0.0618926 | 0.0130946 | 2.17E-06 |
| ebi-a-GCST90017108 | Gut microbiota abundance (order Verrucomicrobiales id.4030) || id:ebi-a-GCST90017108 | CD4-CD8- Natural Killer T %lymphocyte || id:ebi-a-GCST90001638 | rs12908520 | 15 | 97570657 | G | 0.0618926 | 0.0130946 | 2.17E-06 |
| ebi-a-GCST90017108 | Gut microbiota abundance (order Verrucomicrobiales id.4030) || id:ebi-a-GCST90017108 | HLA DR+ Natural Killer Absolute Count || id:ebi-a-GCST90001648 | rs12908520 | 15 | 97570657 | G | 0.0618926 | 0.0130946 | 2.17E-06 |
| ebi-a-GCST90017108 | Gut microbiota abundance (order Verrucomicrobiales id.4030) || id:ebi-a-GCST90017108 | HLA DR+ Natural Killer %Natural Killer || id:ebi-a-GCST90001649 | rs12908520 | 15 | 97570657 | G | 0.0618926 | 0.0130946 | 2.17E-06 |
| ebi-a-GCST90017108 | Gut microbiota abundance (order Verrucomicrobiales id.4030) || id:ebi-a-GCST90017108 | HLA DR+ Natural Killer %CD3- lymphocyte || id:ebi-a-GCST90001650 | rs12908520 | 15 | 97570657 | G | 0.0618926 | 0.0130946 | 2.17E-06 |
| ebi-a-GCST90017108 | Gut microbiota abundance (order Verrucomicrobiales id.4030) || id:ebi-a-GCST90017108 | CD28- CD25++ CD8+ T cell Absolute Count || id:ebi-a-GCST90001678 | rs12908520 | 15 | 97570657 | G | 0.0618926 | 0.0130946 | 2.17E-06 |
| ebi-a-GCST90017108 | Gut microbiota abundance (order Verrucomicrobiales id.4030) || id:ebi-a-GCST90017108 | CD25++ CD8+ T cell %T cell || id:ebi-a-GCST90001679 | rs12908520 | 15 | 97570657 | G | 0.0618926 | 0.0130946 | 2.17E-06 |
| ebi-a-GCST90017108 | Gut microbiota abundance (order Verrucomicrobiales id.4030) || id:ebi-a-GCST90017108 | CD19 on CD20- CD38- B cell || id:ebi-a-GCST90001722 | rs12908520 | 15 | 97570657 | G | 0.0618926 | 0.0130946 | 2.17E-06 |
| ebi-a-GCST90017108 | Gut microbiota abundance (order Verrucomicrobiales id.4030) || id:ebi-a-GCST90017108 | CD25 on B cell || id:ebi-a-GCST90001775 | rs12908520 | 15 | 97570657 | G | 0.0618926 | 0.0130946 | 2.17E-06 |
| ebi-a-GCST90017108 | Gut microbiota abundance (order Verrucomicrobiales id.4030) || id:ebi-a-GCST90017108 | CD25 on IgD+ CD24- B cell || id:ebi-a-GCST90001779 | rs12908520 | 15 | 97570657 | G | 0.0618926 | 0.0130946 | 2.17E-06 |
| ebi-a-GCST90017108 | Gut microbiota abundance (order Verrucomicrobiales id.4030) || id:ebi-a-GCST90017108 | CD25 on IgD+ CD38- naive B cell || id:ebi-a-GCST90001781 | rs12908520 | 15 | 97570657 | G | 0.0618926 | 0.0130946 | 2.17E-06 |
| ebi-a-GCST90017108 | Gut microbiota abundance (order Verrucomicrobiales id.4030) || id:ebi-a-GCST90017108 | CD25 on IgD+ CD38+ B cell || id:ebi-a-GCST90001783 | rs12908520 | 15 | 97570657 | G | 0.0618926 | 0.0130946 | 2.17E-06 |
| ebi-a-GCST90017108 | Gut microbiota abundance (order Verrucomicrobiales id.4030) || id:ebi-a-GCST90017108 | CD86 on myeloid Dendritic Cell || id:ebi-a-GCST90001903 | rs12908520 | 15 | 97570657 | G | 0.0618926 | 0.0130946 | 2.17E-06 |
| ebi-a-GCST90017108 | Gut microbiota abundance (order Verrucomicrobiales id.4030) || id:ebi-a-GCST90017108 | CD33 on CD14+ monocyte || id:ebi-a-GCST90001946 | rs12908520 | 15 | 97570657 | G | 0.0618926 | 0.0130946 | 2.17E-06 |
| ebi-a-GCST90017108 | Gut microbiota abundance (order Verrucomicrobiales id.4030) || id:ebi-a-GCST90017108 | CD33 on CD33+ HLA DR+ CD14dim || id:ebi-a-GCST90001947 | rs12908520 | 15 | 97570657 | G | 0.0618926 | 0.0130946 | 2.17E-06 |
| ebi-a-GCST90017108 | Gut microbiota abundance (order Verrucomicrobiales id.4030) || id:ebi-a-GCST90017108 | CD33 on CD33dim HLA DR+ CD11b+ || id:ebi-a-GCST90001948 | rs12908520 | 15 | 97570657 | G | 0.0618926 | 0.0130946 | 2.17E-06 |
| ebi-a-GCST90017108 | Gut microbiota abundance (order Verrucomicrobiales id.4030) || id:ebi-a-GCST90017108 | CD33 on Granulocytic Myeloid-Derived Suppressor Cells || id:ebi-a-GCST90001950 | rs12908520 | 15 | 97570657 | G | 0.0618926 | 0.0130946 | 2.17E-06 |
| ebi-a-GCST90017108 | Gut microbiota abundance (order Verrucomicrobiales id.4030) || id:ebi-a-GCST90017108 | CD33 on CD66b++ myeloid cell || id:ebi-a-GCST90001951 | rs12908520 | 15 | 97570657 | G | 0.0618926 | 0.0130946 | 2.17E-06 |
| ebi-a-GCST90017108 | Gut microbiota abundance (order Verrucomicrobiales id.4030) || id:ebi-a-GCST90017108 | CD33 on CD33dim HLA DR- || id:ebi-a-GCST90001953 | rs12908520 | 15 | 97570657 | G | 0.0618926 | 0.0130946 | 2.17E-06 |
| ebi-a-GCST90017108 | Gut microbiota abundance (order Verrucomicrobiales id.4030) || id:ebi-a-GCST90017108 | CD33 on basophil || id:ebi-a-GCST90001954 | rs12908520 | 15 | 97570657 | G | 0.0618926 | 0.0130946 | 2.17E-06 |
| ebi-a-GCST90017108 | Gut microbiota abundance (order Verrucomicrobiales id.4030) || id:ebi-a-GCST90017108 | CD33 on Immature Myeloid-Derived Suppressor Cells || id:ebi-a-GCST90001955 | rs12908520 | 15 | 97570657 | G | 0.0618926 | 0.0130946 | 2.17E-06 |
| ebi-a-GCST90017108 | Gut microbiota abundance (order Verrucomicrobiales id.4030) || id:ebi-a-GCST90017108 | FSC-A on HLA DR+ CD8+ T cell || id:ebi-a-GCST90001978 | rs12908520 | 15 | 97570657 | G | 0.0618926 | 0.0130946 | 2.17E-06 |
| ebi-a-GCST90017108 | Gut microbiota abundance (order Verrucomicrobiales id.4030) || id:ebi-a-GCST90017108 | HLA DR on CD14+ CD16- monocyte || id:ebi-a-GCST90001988 | rs12908520 | 15 | 97570657 | G | 0.0618926 | 0.0130946 | 2.17E-06 |
| ebi-a-GCST90017108 | Gut microbiota abundance (order Verrucomicrobiales id.4030) || id:ebi-a-GCST90017108 | HLA DR on CD14+ monocyte || id:ebi-a-GCST90001991 | rs12908520 | 15 | 97570657 | G | 0.0618926 | 0.0130946 | 2.17E-06 |
| ebi-a-GCST90017108 | Gut microbiota abundance (order Verrucomicrobiales id.4030) || id:ebi-a-GCST90017108 | CD16 on CD14+ CD16+ monocyte || id:ebi-a-GCST90002005 | rs12908520 | 15 | 97570657 | G | 0.0618926 | 0.0130946 | 2.17E-06 |
| ebi-a-GCST90017108 | Gut microbiota abundance (order Verrucomicrobiales id.4030) || id:ebi-a-GCST90017108 | CD45 on CD33+ HLA DR+ CD14- || id:ebi-a-GCST90002042 | rs12908520 | 15 | 97570657 | G | 0.0618926 | 0.0130946 | 2.17E-06 |
| ebi-a-GCST90017108 | Gut microbiota abundance (order Verrucomicrobiales id.4030) || id:ebi-a-GCST90017108 | CD8 on Natural Killer T || id:ebi-a-GCST90002059 | rs12908520 | 15 | 97570657 | G | 0.0618926 | 0.0130946 | 2.17E-06 |
| ebi-a-GCST90016961 | Gut microbiota abundance (genus Akkermansia id.4037) || id:ebi-a-GCST90016961 | Myeloid Dendritic Cell Absolute Count || id:ebi-a-GCST90001458 | rs12908520 | 15 | 97570657 | G | 0.061772 | 0.0130954 | 2.26E-06 |
| ebi-a-GCST90016961 | Gut microbiota abundance (genus Akkermansia id.4037) || id:ebi-a-GCST90016961 | CD86+ myeloid Dendritic Cell %Dendritic Cell || id:ebi-a-GCST90001465 | rs12908520 | 15 | 97570657 | G | 0.061772 | 0.0130954 | 2.26E-06 |
| ebi-a-GCST90016961 | Gut microbiota abundance (genus Akkermansia id.4037) || id:ebi-a-GCST90016961 | CD62L- myeloid Dendritic Cell Absolute Count || id:ebi-a-GCST90001468 | rs12908520 | 15 | 97570657 | G | 0.061772 | 0.0130954 | 2.26E-06 |
| ebi-a-GCST90016961 | Gut microbiota abundance (genus Akkermansia id.4037) || id:ebi-a-GCST90016961 | CD62L- myeloid Dendritic Cell %Dendritic Cell || id:ebi-a-GCST90001469 | rs12908520 | 15 | 97570657 | G | 0.061772 | 0.0130954 | 2.26E-06 |
| ebi-a-GCST90016961 | Gut microbiota abundance (genus Akkermansia id.4037) || id:ebi-a-GCST90016961 | CD62L- CD86+ myeloid Dendritic Cell Absolute Count || id:ebi-a-GCST90001472 | rs12908520 | 15 | 97570657 | G | 0.061772 | 0.0130954 | 2.26E-06 |
| ebi-a-GCST90016961 | Gut microbiota abundance (genus Akkermansia id.4037) || id:ebi-a-GCST90016961 | CD62L- CD86+ myeloid Dendritic Cell %Dendritic Cell || id:ebi-a-GCST90001473 | rs12908520 | 15 | 97570657 | G | 0.061772 | 0.0130954 | 2.26E-06 |
| ebi-a-GCST90016961 | Gut microbiota abundance (genus Akkermansia id.4037) || id:ebi-a-GCST90016961 | HLA DR++ monocyte %monocyte || id:ebi-a-GCST90001475 | rs12908520 | 15 | 97570657 | G | 0.061772 | 0.0130954 | 2.26E-06 |
| ebi-a-GCST90016961 | Gut microbiota abundance (genus Akkermansia id.4037) || id:ebi-a-GCST90016961 | HLA DR++ monocyte Absolute Count || id:ebi-a-GCST90001477 | rs12908520 | 15 | 97570657 | G | 0.061772 | 0.0130954 | 2.26E-06 |
| ebi-a-GCST90016961 | Gut microbiota abundance (genus Akkermansia id.4037) || id:ebi-a-GCST90016961 | Basophil %CD33dim HLA DR- CD66b- || id:ebi-a-GCST90001533 | rs12908520 | 15 | 97570657 | G | 0.061772 | 0.0130954 | 2.26E-06 |
| ebi-a-GCST90016961 | Gut microbiota abundance (genus Akkermansia id.4037) || id:ebi-a-GCST90016961 | CD16+ monocyte %monocyte || id:ebi-a-GCST90001587 | rs12908520 | 15 | 97570657 | G | 0.061772 | 0.0130954 | 2.26E-06 |
| ebi-a-GCST90016961 | Gut microbiota abundance (genus Akkermansia id.4037) || id:ebi-a-GCST90016961 | T/B cell || id:ebi-a-GCST90001588 | rs12908520 | 15 | 97570657 | G | 0.061772 | 0.0130954 | 2.26E-06 |
| ebi-a-GCST90016961 | Gut microbiota abundance (genus Akkermansia id.4037) || id:ebi-a-GCST90016961 | CD8+ Natural Killer T %T cell || id:ebi-a-GCST90001631 | rs12908520 | 15 | 97570657 | G | 0.061772 | 0.0130954 | 2.26E-06 |
| ebi-a-GCST90016961 | Gut microbiota abundance (genus Akkermansia id.4037) || id:ebi-a-GCST90016961 | CD4-CD8- Natural Killer T %lymphocyte || id:ebi-a-GCST90001638 | rs12908520 | 15 | 97570657 | G | 0.061772 | 0.0130954 | 2.26E-06 |
| ebi-a-GCST90016961 | Gut microbiota abundance (genus Akkermansia id.4037) || id:ebi-a-GCST90016961 | HLA DR+ Natural Killer Absolute Count || id:ebi-a-GCST90001648 | rs12908520 | 15 | 97570657 | G | 0.061772 | 0.0130954 | 2.26E-06 |
| ebi-a-GCST90016961 | Gut microbiota abundance (genus Akkermansia id.4037) || id:ebi-a-GCST90016961 | HLA DR+ Natural Killer %Natural Killer || id:ebi-a-GCST90001649 | rs12908520 | 15 | 97570657 | G | 0.061772 | 0.0130954 | 2.26E-06 |
| ebi-a-GCST90016961 | Gut microbiota abundance (genus Akkermansia id.4037) || id:ebi-a-GCST90016961 | HLA DR+ Natural Killer %CD3- lymphocyte || id:ebi-a-GCST90001650 | rs12908520 | 15 | 97570657 | G | 0.061772 | 0.0130954 | 2.26E-06 |
| ebi-a-GCST90016961 | Gut microbiota abundance (genus Akkermansia id.4037) || id:ebi-a-GCST90016961 | CD28- CD25++ CD8+ T cell Absolute Count || id:ebi-a-GCST90001678 | rs12908520 | 15 | 97570657 | G | 0.061772 | 0.0130954 | 2.26E-06 |
| ebi-a-GCST90016961 | Gut microbiota abundance (genus Akkermansia id.4037) || id:ebi-a-GCST90016961 | CD25++ CD8+ T cell %T cell || id:ebi-a-GCST90001679 | rs12908520 | 15 | 97570657 | G | 0.061772 | 0.0130954 | 2.26E-06 |
| ebi-a-GCST90016961 | Gut microbiota abundance (genus Akkermansia id.4037) || id:ebi-a-GCST90016961 | CD19 on CD20- CD38- B cell || id:ebi-a-GCST90001722 | rs12908520 | 15 | 97570657 | G | 0.061772 | 0.0130954 | 2.26E-06 |
| ebi-a-GCST90016961 | Gut microbiota abundance (genus Akkermansia id.4037) || id:ebi-a-GCST90016961 | CD25 on B cell || id:ebi-a-GCST90001775 | rs12908520 | 15 | 97570657 | G | 0.061772 | 0.0130954 | 2.26E-06 |
| ebi-a-GCST90016961 | Gut microbiota abundance (genus Akkermansia id.4037) || id:ebi-a-GCST90016961 | CD25 on IgD+ CD24- B cell || id:ebi-a-GCST90001779 | rs12908520 | 15 | 97570657 | G | 0.061772 | 0.0130954 | 2.26E-06 |
| ebi-a-GCST90016961 | Gut microbiota abundance (genus Akkermansia id.4037) || id:ebi-a-GCST90016961 | CD25 on IgD+ CD38- naive B cell || id:ebi-a-GCST90001781 | rs12908520 | 15 | 97570657 | G | 0.061772 | 0.0130954 | 2.26E-06 |
| ebi-a-GCST90016961 | Gut microbiota abundance (genus Akkermansia id.4037) || id:ebi-a-GCST90016961 | CD25 on IgD+ CD38+ B cell || id:ebi-a-GCST90001783 | rs12908520 | 15 | 97570657 | G | 0.061772 | 0.0130954 | 2.26E-06 |
| ebi-a-GCST90016961 | Gut microbiota abundance (genus Akkermansia id.4037) || id:ebi-a-GCST90016961 | CD86 on myeloid Dendritic Cell || id:ebi-a-GCST90001903 | rs12908520 | 15 | 97570657 | G | 0.061772 | 0.0130954 | 2.26E-06 |
| ebi-a-GCST90016961 | Gut microbiota abundance (genus Akkermansia id.4037) || id:ebi-a-GCST90016961 | CD33 on CD14+ monocyte || id:ebi-a-GCST90001946 | rs12908520 | 15 | 97570657 | G | 0.061772 | 0.0130954 | 2.26E-06 |
| ebi-a-GCST90016961 | Gut microbiota abundance (genus Akkermansia id.4037) || id:ebi-a-GCST90016961 | CD33 on CD33+ HLA DR+ CD14dim || id:ebi-a-GCST90001947 | rs12908520 | 15 | 97570657 | G | 0.061772 | 0.0130954 | 2.26E-06 |
| ebi-a-GCST90016961 | Gut microbiota abundance (genus Akkermansia id.4037) || id:ebi-a-GCST90016961 | CD33 on CD33dim HLA DR+ CD11b+ || id:ebi-a-GCST90001948 | rs12908520 | 15 | 97570657 | G | 0.061772 | 0.0130954 | 2.26E-06 |
| ebi-a-GCST90016961 | Gut microbiota abundance (genus Akkermansia id.4037) || id:ebi-a-GCST90016961 | CD33 on Granulocytic Myeloid-Derived Suppressor Cells || id:ebi-a-GCST90001950 | rs12908520 | 15 | 97570657 | G | 0.061772 | 0.0130954 | 2.26E-06 |
| ebi-a-GCST90016961 | Gut microbiota abundance (genus Akkermansia id.4037) || id:ebi-a-GCST90016961 | CD33 on CD66b++ myeloid cell || id:ebi-a-GCST90001951 | rs12908520 | 15 | 97570657 | G | 0.061772 | 0.0130954 | 2.26E-06 |
| ebi-a-GCST90016961 | Gut microbiota abundance (genus Akkermansia id.4037) || id:ebi-a-GCST90016961 | CD33 on CD33dim HLA DR- || id:ebi-a-GCST90001953 | rs12908520 | 15 | 97570657 | G | 0.061772 | 0.0130954 | 2.26E-06 |
| ebi-a-GCST90016961 | Gut microbiota abundance (genus Akkermansia id.4037) || id:ebi-a-GCST90016961 | CD33 on basophil || id:ebi-a-GCST90001954 | rs12908520 | 15 | 97570657 | G | 0.061772 | 0.0130954 | 2.26E-06 |
| ebi-a-GCST90016961 | Gut microbiota abundance (genus Akkermansia id.4037) || id:ebi-a-GCST90016961 | CD33 on Immature Myeloid-Derived Suppressor Cells || id:ebi-a-GCST90001955 | rs12908520 | 15 | 97570657 | G | 0.061772 | 0.0130954 | 2.26E-06 |
| ebi-a-GCST90016961 | Gut microbiota abundance (genus Akkermansia id.4037) || id:ebi-a-GCST90016961 | FSC-A on HLA DR+ CD8+ T cell || id:ebi-a-GCST90001978 | rs12908520 | 15 | 97570657 | G | 0.061772 | 0.0130954 | 2.26E-06 |
| ebi-a-GCST90016961 | Gut microbiota abundance (genus Akkermansia id.4037) || id:ebi-a-GCST90016961 | HLA DR on CD14+ CD16- monocyte || id:ebi-a-GCST90001988 | rs12908520 | 15 | 97570657 | G | 0.061772 | 0.0130954 | 2.26E-06 |
| ebi-a-GCST90016961 | Gut microbiota abundance (genus Akkermansia id.4037) || id:ebi-a-GCST90016961 | HLA DR on CD14+ monocyte || id:ebi-a-GCST90001991 | rs12908520 | 15 | 97570657 | G | 0.061772 | 0.0130954 | 2.26E-06 |
| ebi-a-GCST90016961 | Gut microbiota abundance (genus Akkermansia id.4037) || id:ebi-a-GCST90016961 | CD16 on CD14+ CD16+ monocyte || id:ebi-a-GCST90002005 | rs12908520 | 15 | 97570657 | G | 0.061772 | 0.0130954 | 2.26E-06 |
| ebi-a-GCST90016961 | Gut microbiota abundance (genus Akkermansia id.4037) || id:ebi-a-GCST90016961 | CD45 on CD33+ HLA DR+ CD14- || id:ebi-a-GCST90002042 | rs12908520 | 15 | 97570657 | G | 0.061772 | 0.0130954 | 2.26E-06 |
| ebi-a-GCST90016961 | Gut microbiota abundance (genus Akkermansia id.4037) || id:ebi-a-GCST90016961 | CD8 on Natural Killer T || id:ebi-a-GCST90002059 | rs12908520 | 15 | 97570657 | G | 0.061772 | 0.0130954 | 2.26E-06 |
| ebi-a-GCST90016943 | Gut microbiota abundance (family Oxalobacteraceae id.2966) || id:ebi-a-GCST90016943 | Myeloid Dendritic Cell Absolute Count || id:ebi-a-GCST90001458 | rs1569853 | 6 | 38550301 | T | -0.140043 | 0.0281704 | 7.45E-07 |
| ebi-a-GCST90016943 | Gut microbiota abundance (family Oxalobacteraceae id.2966) || id:ebi-a-GCST90016943 | CD86+ myeloid Dendritic Cell %Dendritic Cell || id:ebi-a-GCST90001465 | rs1569853 | 6 | 38550301 | T | -0.140043 | 0.0281704 | 7.45E-07 |
| ebi-a-GCST90016943 | Gut microbiota abundance (family Oxalobacteraceae id.2966) || id:ebi-a-GCST90016943 | CD62L- myeloid Dendritic Cell Absolute Count || id:ebi-a-GCST90001468 | rs1569853 | 6 | 38550301 | T | -0.140043 | 0.0281704 | 7.45E-07 |
| ebi-a-GCST90016943 | Gut microbiota abundance (family Oxalobacteraceae id.2966) || id:ebi-a-GCST90016943 | CD62L- myeloid Dendritic Cell %Dendritic Cell || id:ebi-a-GCST90001469 | rs1569853 | 6 | 38550301 | T | -0.140043 | 0.0281704 | 7.45E-07 |
| ebi-a-GCST90016943 | Gut microbiota abundance (family Oxalobacteraceae id.2966) || id:ebi-a-GCST90016943 | CD62L- CD86+ myeloid Dendritic Cell Absolute Count || id:ebi-a-GCST90001472 | rs1569853 | 6 | 38550301 | T | -0.140043 | 0.0281704 | 7.45E-07 |
| ebi-a-GCST90016943 | Gut microbiota abundance (family Oxalobacteraceae id.2966) || id:ebi-a-GCST90016943 | CD62L- CD86+ myeloid Dendritic Cell %Dendritic Cell || id:ebi-a-GCST90001473 | rs1569853 | 6 | 38550301 | T | -0.140043 | 0.0281704 | 7.45E-07 |
| ebi-a-GCST90016943 | Gut microbiota abundance (family Oxalobacteraceae id.2966) || id:ebi-a-GCST90016943 | HLA DR++ monocyte %monocyte || id:ebi-a-GCST90001475 | rs1569853 | 6 | 38550301 | T | -0.140043 | 0.0281704 | 7.45E-07 |
| ebi-a-GCST90016943 | Gut microbiota abundance (family Oxalobacteraceae id.2966) || id:ebi-a-GCST90016943 | HLA DR++ monocyte Absolute Count || id:ebi-a-GCST90001477 | rs1569853 | 6 | 38550301 | T | -0.140043 | 0.0281704 | 7.45E-07 |
| ebi-a-GCST90016943 | Gut microbiota abundance (family Oxalobacteraceae id.2966) || id:ebi-a-GCST90016943 | Basophil %CD33dim HLA DR- CD66b- || id:ebi-a-GCST90001533 | rs1569853 | 6 | 38550301 | T | -0.140043 | 0.0281704 | 7.45E-07 |
| ebi-a-GCST90016943 | Gut microbiota abundance (family Oxalobacteraceae id.2966) || id:ebi-a-GCST90016943 | CD16+ monocyte %monocyte || id:ebi-a-GCST90001587 | rs1569853 | 6 | 38550301 | T | -0.140043 | 0.0281704 | 7.45E-07 |
| ebi-a-GCST90016943 | Gut microbiota abundance (family Oxalobacteraceae id.2966) || id:ebi-a-GCST90016943 | T/B cell || id:ebi-a-GCST90001588 | rs1569853 | 6 | 38550301 | T | -0.140043 | 0.0281704 | 7.45E-07 |
| ebi-a-GCST90016943 | Gut microbiota abundance (family Oxalobacteraceae id.2966) || id:ebi-a-GCST90016943 | CD4+CD8+ T cell %T cell || id:ebi-a-GCST90001595 | rs1569853 | 6 | 38550301 | T | -0.140043 | 0.0281704 | 7.45E-07 |
| ebi-a-GCST90016943 | Gut microbiota abundance (family Oxalobacteraceae id.2966) || id:ebi-a-GCST90016943 | CD8+ Natural Killer T %T cell || id:ebi-a-GCST90001631 | rs1569853 | 6 | 38550301 | T | -0.140043 | 0.0281704 | 7.45E-07 |
| ebi-a-GCST90016943 | Gut microbiota abundance (family Oxalobacteraceae id.2966) || id:ebi-a-GCST90016943 | CD4-CD8- Natural Killer T %lymphocyte || id:ebi-a-GCST90001638 | rs1569853 | 6 | 38550301 | T | -0.140043 | 0.0281704 | 7.45E-07 |
| ebi-a-GCST90016943 | Gut microbiota abundance (family Oxalobacteraceae id.2966) || id:ebi-a-GCST90016943 | HLA DR+ Natural Killer Absolute Count || id:ebi-a-GCST90001648 | rs1569853 | 6 | 38550301 | T | -0.140043 | 0.0281704 | 7.45E-07 |
| ebi-a-GCST90016943 | Gut microbiota abundance (family Oxalobacteraceae id.2966) || id:ebi-a-GCST90016943 | HLA DR+ Natural Killer %Natural Killer || id:ebi-a-GCST90001649 | rs1569853 | 6 | 38550301 | T | -0.140043 | 0.0281704 | 7.45E-07 |
| ebi-a-GCST90016943 | Gut microbiota abundance (family Oxalobacteraceae id.2966) || id:ebi-a-GCST90016943 | HLA DR+ Natural Killer %CD3- lymphocyte || id:ebi-a-GCST90001650 | rs1569853 | 6 | 38550301 | T | -0.140043 | 0.0281704 | 7.45E-07 |
| ebi-a-GCST90016943 | Gut microbiota abundance (family Oxalobacteraceae id.2966) || id:ebi-a-GCST90016943 | CD28- CD25++ CD8+ T cell Absolute Count || id:ebi-a-GCST90001678 | rs1569853 | 6 | 38550301 | T | -0.140043 | 0.0281704 | 7.45E-07 |
| ebi-a-GCST90016943 | Gut microbiota abundance (family Oxalobacteraceae id.2966) || id:ebi-a-GCST90016943 | CD25++ CD8+ T cell %T cell || id:ebi-a-GCST90001679 | rs1569853 | 6 | 38550301 | T | -0.140043 | 0.0281704 | 7.45E-07 |
| ebi-a-GCST90016943 | Gut microbiota abundance (family Oxalobacteraceae id.2966) || id:ebi-a-GCST90016943 | CD19 on CD20- CD38- B cell || id:ebi-a-GCST90001722 | rs1569853 | 6 | 38550301 | T | -0.140043 | 0.0281704 | 7.45E-07 |
| ebi-a-GCST90016943 | Gut microbiota abundance (family Oxalobacteraceae id.2966) || id:ebi-a-GCST90016943 | CD25 on B cell || id:ebi-a-GCST90001775 | rs1569853 | 6 | 38550301 | T | -0.140043 | 0.0281704 | 7.45E-07 |
| ebi-a-GCST90016943 | Gut microbiota abundance (family Oxalobacteraceae id.2966) || id:ebi-a-GCST90016943 | CD25 on IgD+ CD24- B cell || id:ebi-a-GCST90001779 | rs1569853 | 6 | 38550301 | T | -0.140043 | 0.0281704 | 7.45E-07 |
| ebi-a-GCST90016943 | Gut microbiota abundance (family Oxalobacteraceae id.2966) || id:ebi-a-GCST90016943 | CD25 on IgD+ CD38- naive B cell || id:ebi-a-GCST90001781 | rs1569853 | 6 | 38550301 | T | -0.140043 | 0.0281704 | 7.45E-07 |
| ebi-a-GCST90016943 | Gut microbiota abundance (family Oxalobacteraceae id.2966) || id:ebi-a-GCST90016943 | CD25 on IgD+ CD38+ B cell || id:ebi-a-GCST90001783 | rs1569853 | 6 | 38550301 | T | -0.140043 | 0.0281704 | 7.45E-07 |
| ebi-a-GCST90016943 | Gut microbiota abundance (family Oxalobacteraceae id.2966) || id:ebi-a-GCST90016943 | CD86 on myeloid Dendritic Cell || id:ebi-a-GCST90001903 | rs1569853 | 6 | 38550301 | T | -0.140043 | 0.0281704 | 7.45E-07 |
| ebi-a-GCST90016943 | Gut microbiota abundance (family Oxalobacteraceae id.2966) || id:ebi-a-GCST90016943 | CD33 on CD14+ monocyte || id:ebi-a-GCST90001946 | rs1569853 | 6 | 38550301 | T | -0.140043 | 0.0281704 | 7.45E-07 |
| ebi-a-GCST90016943 | Gut microbiota abundance (family Oxalobacteraceae id.2966) || id:ebi-a-GCST90016943 | CD33 on CD33+ HLA DR+ CD14dim || id:ebi-a-GCST90001947 | rs1569853 | 6 | 38550301 | T | -0.140043 | 0.0281704 | 7.45E-07 |
| ebi-a-GCST90016943 | Gut microbiota abundance (family Oxalobacteraceae id.2966) || id:ebi-a-GCST90016943 | CD33 on CD33dim HLA DR+ CD11b+ || id:ebi-a-GCST90001948 | rs1569853 | 6 | 38550301 | T | -0.140043 | 0.0281704 | 7.45E-07 |
| ebi-a-GCST90016943 | Gut microbiota abundance (family Oxalobacteraceae id.2966) || id:ebi-a-GCST90016943 | CD33 on Granulocytic Myeloid-Derived Suppressor Cells || id:ebi-a-GCST90001950 | rs1569853 | 6 | 38550301 | T | -0.140043 | 0.0281704 | 7.45E-07 |
| ebi-a-GCST90016943 | Gut microbiota abundance (family Oxalobacteraceae id.2966) || id:ebi-a-GCST90016943 | CD33 on CD66b++ myeloid cell || id:ebi-a-GCST90001951 | rs1569853 | 6 | 38550301 | T | -0.140043 | 0.0281704 | 7.45E-07 |
| ebi-a-GCST90016943 | Gut microbiota abundance (family Oxalobacteraceae id.2966) || id:ebi-a-GCST90016943 | CD33 on CD33dim HLA DR- || id:ebi-a-GCST90001953 | rs1569853 | 6 | 38550301 | T | -0.140043 | 0.0281704 | 7.45E-07 |
| ebi-a-GCST90016943 | Gut microbiota abundance (family Oxalobacteraceae id.2966) || id:ebi-a-GCST90016943 | CD33 on basophil || id:ebi-a-GCST90001954 | rs1569853 | 6 | 38550301 | T | -0.140043 | 0.0281704 | 7.45E-07 |
| ebi-a-GCST90016943 | Gut microbiota abundance (family Oxalobacteraceae id.2966) || id:ebi-a-GCST90016943 | CD33 on Immature Myeloid-Derived Suppressor Cells || id:ebi-a-GCST90001955 | rs1569853 | 6 | 38550301 | T | -0.140043 | 0.0281704 | 7.45E-07 |
| ebi-a-GCST90016943 | Gut microbiota abundance (family Oxalobacteraceae id.2966) || id:ebi-a-GCST90016943 | FSC-A on HLA DR+ CD8+ T cell || id:ebi-a-GCST90001978 | rs1569853 | 6 | 38550301 | T | -0.140043 | 0.0281704 | 7.45E-07 |
| ebi-a-GCST90016943 | Gut microbiota abundance (family Oxalobacteraceae id.2966) || id:ebi-a-GCST90016943 | HLA DR on CD14+ CD16- monocyte || id:ebi-a-GCST90001988 | rs1569853 | 6 | 38550301 | T | -0.140043 | 0.0281704 | 7.45E-07 |
| ebi-a-GCST90016943 | Gut microbiota abundance (family Oxalobacteraceae id.2966) || id:ebi-a-GCST90016943 | HLA DR on CD14+ monocyte || id:ebi-a-GCST90001991 | rs1569853 | 6 | 38550301 | T | -0.140043 | 0.0281704 | 7.45E-07 |
| ebi-a-GCST90016943 | Gut microbiota abundance (family Oxalobacteraceae id.2966) || id:ebi-a-GCST90016943 | CD16 on CD14+ CD16+ monocyte || id:ebi-a-GCST90002005 | rs1569853 | 6 | 38550301 | T | -0.140043 | 0.0281704 | 7.45E-07 |
| ebi-a-GCST90016943 | Gut microbiota abundance (family Oxalobacteraceae id.2966) || id:ebi-a-GCST90016943 | CD45 on CD33+ HLA DR+ CD14- || id:ebi-a-GCST90002042 | rs1569853 | 6 | 38550301 | T | -0.140043 | 0.0281704 | 7.45E-07 |
| ebi-a-GCST90016943 | Gut microbiota abundance (family Oxalobacteraceae id.2966) || id:ebi-a-GCST90016943 | CD8 on Natural Killer T || id:ebi-a-GCST90002059 | rs1569853 | 6 | 38550301 | T | -0.140043 | 0.0281704 | 7.45E-07 |
| ebi-a-GCST90017037 | Gut microbiota abundance (genus Oscillospira id.2064) || id:ebi-a-GCST90017037 | Myeloid Dendritic Cell Absolute Count || id:ebi-a-GCST90001458 | rs1954532 | 14 | 28151415 | C | 0.0826246 | 0.0175252 | 2.27E-06 |
| ebi-a-GCST90017037 | Gut microbiota abundance (genus Oscillospira id.2064) || id:ebi-a-GCST90017037 | CD86+ myeloid Dendritic Cell %Dendritic Cell || id:ebi-a-GCST90001465 | rs1954532 | 14 | 28151415 | C | 0.0826246 | 0.0175252 | 2.27E-06 |
| ebi-a-GCST90017037 | Gut microbiota abundance (genus Oscillospira id.2064) || id:ebi-a-GCST90017037 | CD62L- myeloid Dendritic Cell Absolute Count || id:ebi-a-GCST90001468 | rs1954532 | 14 | 28151415 | C | 0.0826246 | 0.0175252 | 2.27E-06 |
| ebi-a-GCST90017037 | Gut microbiota abundance (genus Oscillospira id.2064) || id:ebi-a-GCST90017037 | CD62L- myeloid Dendritic Cell %Dendritic Cell || id:ebi-a-GCST90001469 | rs1954532 | 14 | 28151415 | C | 0.0826246 | 0.0175252 | 2.27E-06 |
| ebi-a-GCST90017037 | Gut microbiota abundance (genus Oscillospira id.2064) || id:ebi-a-GCST90017037 | CD62L- CD86+ myeloid Dendritic Cell Absolute Count || id:ebi-a-GCST90001472 | rs1954532 | 14 | 28151415 | C | 0.0826246 | 0.0175252 | 2.27E-06 |
| ebi-a-GCST90017037 | Gut microbiota abundance (genus Oscillospira id.2064) || id:ebi-a-GCST90017037 | CD62L- CD86+ myeloid Dendritic Cell %Dendritic Cell || id:ebi-a-GCST90001473 | rs1954532 | 14 | 28151415 | C | 0.0826246 | 0.0175252 | 2.27E-06 |
| ebi-a-GCST90017037 | Gut microbiota abundance (genus Oscillospira id.2064) || id:ebi-a-GCST90017037 | HLA DR++ monocyte %monocyte || id:ebi-a-GCST90001475 | rs1954532 | 14 | 28151415 | C | 0.0826246 | 0.0175252 | 2.27E-06 |
| ebi-a-GCST90017037 | Gut microbiota abundance (genus Oscillospira id.2064) || id:ebi-a-GCST90017037 | HLA DR++ monocyte Absolute Count || id:ebi-a-GCST90001477 | rs1954532 | 14 | 28151415 | C | 0.0826246 | 0.0175252 | 2.27E-06 |
| ebi-a-GCST90017037 | Gut microbiota abundance (genus Oscillospira id.2064) || id:ebi-a-GCST90017037 | Basophil %CD33dim HLA DR- CD66b- || id:ebi-a-GCST90001533 | rs1954532 | 14 | 28151415 | C | 0.0826246 | 0.0175252 | 2.27E-06 |
| ebi-a-GCST90017037 | Gut microbiota abundance (genus Oscillospira id.2064) || id:ebi-a-GCST90017037 | CD16+ monocyte %monocyte || id:ebi-a-GCST90001587 | rs1954532 | 14 | 28151415 | C | 0.0826246 | 0.0175252 | 2.27E-06 |
| ebi-a-GCST90017037 | Gut microbiota abundance (genus Oscillospira id.2064) || id:ebi-a-GCST90017037 | T/B cell || id:ebi-a-GCST90001588 | rs1954532 | 14 | 28151415 | C | 0.0826246 | 0.0175252 | 2.27E-06 |
| ebi-a-GCST90017037 | Gut microbiota abundance (genus Oscillospira id.2064) || id:ebi-a-GCST90017037 | CD8+ Natural Killer T %T cell || id:ebi-a-GCST90001631 | rs1954532 | 14 | 28151415 | C | 0.0826246 | 0.0175252 | 2.27E-06 |
| ebi-a-GCST90017037 | Gut microbiota abundance (genus Oscillospira id.2064) || id:ebi-a-GCST90017037 | CD4-CD8- Natural Killer T %lymphocyte || id:ebi-a-GCST90001638 | rs1954532 | 14 | 28151415 | C | 0.0826246 | 0.0175252 | 2.27E-06 |
| ebi-a-GCST90017037 | Gut microbiota abundance (genus Oscillospira id.2064) || id:ebi-a-GCST90017037 | HLA DR+ Natural Killer Absolute Count || id:ebi-a-GCST90001648 | rs1954532 | 14 | 28151415 | C | 0.0826246 | 0.0175252 | 2.27E-06 |
| ebi-a-GCST90017037 | Gut microbiota abundance (genus Oscillospira id.2064) || id:ebi-a-GCST90017037 | HLA DR+ Natural Killer %Natural Killer || id:ebi-a-GCST90001649 | rs1954532 | 14 | 28151415 | C | 0.0826246 | 0.0175252 | 2.27E-06 |
| ebi-a-GCST90017037 | Gut microbiota abundance (genus Oscillospira id.2064) || id:ebi-a-GCST90017037 | HLA DR+ Natural Killer %CD3- lymphocyte || id:ebi-a-GCST90001650 | rs1954532 | 14 | 28151415 | C | 0.0826246 | 0.0175252 | 2.27E-06 |
| ebi-a-GCST90017037 | Gut microbiota abundance (genus Oscillospira id.2064) || id:ebi-a-GCST90017037 | CD28- CD25++ CD8+ T cell Absolute Count || id:ebi-a-GCST90001678 | rs1954532 | 14 | 28151415 | C | 0.0826246 | 0.0175252 | 2.27E-06 |
| ebi-a-GCST90017037 | Gut microbiota abundance (genus Oscillospira id.2064) || id:ebi-a-GCST90017037 | CD25++ CD8+ T cell %T cell || id:ebi-a-GCST90001679 | rs1954532 | 14 | 28151415 | C | 0.0826246 | 0.0175252 | 2.27E-06 |
| ebi-a-GCST90017037 | Gut microbiota abundance (genus Oscillospira id.2064) || id:ebi-a-GCST90017037 | CD19 on CD20- CD38- B cell || id:ebi-a-GCST90001722 | rs1954532 | 14 | 28151415 | C | 0.0826246 | 0.0175252 | 2.27E-06 |
| ebi-a-GCST90017037 | Gut microbiota abundance (genus Oscillospira id.2064) || id:ebi-a-GCST90017037 | CD25 on B cell || id:ebi-a-GCST90001775 | rs1954532 | 14 | 28151415 | C | 0.0826246 | 0.0175252 | 2.27E-06 |
| ebi-a-GCST90017037 | Gut microbiota abundance (genus Oscillospira id.2064) || id:ebi-a-GCST90017037 | CD25 on IgD+ CD24- B cell || id:ebi-a-GCST90001779 | rs1954532 | 14 | 28151415 | C | 0.0826246 | 0.0175252 | 2.27E-06 |
| ebi-a-GCST90017037 | Gut microbiota abundance (genus Oscillospira id.2064) || id:ebi-a-GCST90017037 | CD25 on IgD+ CD38- naive B cell || id:ebi-a-GCST90001781 | rs1954532 | 14 | 28151415 | C | 0.0826246 | 0.0175252 | 2.27E-06 |
| ebi-a-GCST90017037 | Gut microbiota abundance (genus Oscillospira id.2064) || id:ebi-a-GCST90017037 | CD25 on IgD+ CD38+ B cell || id:ebi-a-GCST90001783 | rs1954532 | 14 | 28151415 | C | 0.0826246 | 0.0175252 | 2.27E-06 |
| ebi-a-GCST90017037 | Gut microbiota abundance (genus Oscillospira id.2064) || id:ebi-a-GCST90017037 | CD86 on myeloid Dendritic Cell || id:ebi-a-GCST90001903 | rs1954532 | 14 | 28151415 | C | 0.0826246 | 0.0175252 | 2.27E-06 |
| ebi-a-GCST90017037 | Gut microbiota abundance (genus Oscillospira id.2064) || id:ebi-a-GCST90017037 | CD33 on CD14+ monocyte || id:ebi-a-GCST90001946 | rs1954532 | 14 | 28151415 | C | 0.0826246 | 0.0175252 | 2.27E-06 |
| ebi-a-GCST90017037 | Gut microbiota abundance (genus Oscillospira id.2064) || id:ebi-a-GCST90017037 | CD33 on CD33+ HLA DR+ CD14dim || id:ebi-a-GCST90001947 | rs1954532 | 14 | 28151415 | C | 0.0826246 | 0.0175252 | 2.27E-06 |
| ebi-a-GCST90017037 | Gut microbiota abundance (genus Oscillospira id.2064) || id:ebi-a-GCST90017037 | CD33 on CD33dim HLA DR+ CD11b+ || id:ebi-a-GCST90001948 | rs1954532 | 14 | 28151415 | C | 0.0826246 | 0.0175252 | 2.27E-06 |
| ebi-a-GCST90017037 | Gut microbiota abundance (genus Oscillospira id.2064) || id:ebi-a-GCST90017037 | CD33 on Granulocytic Myeloid-Derived Suppressor Cells || id:ebi-a-GCST90001950 | rs1954532 | 14 | 28151415 | C | 0.0826246 | 0.0175252 | 2.27E-06 |
| ebi-a-GCST90017037 | Gut microbiota abundance (genus Oscillospira id.2064) || id:ebi-a-GCST90017037 | CD33 on CD66b++ myeloid cell || id:ebi-a-GCST90001951 | rs1954532 | 14 | 28151415 | C | 0.0826246 | 0.0175252 | 2.27E-06 |
| ebi-a-GCST90017037 | Gut microbiota abundance (genus Oscillospira id.2064) || id:ebi-a-GCST90017037 | CD33 on CD33dim HLA DR- || id:ebi-a-GCST90001953 | rs1954532 | 14 | 28151415 | C | 0.0826246 | 0.0175252 | 2.27E-06 |
| ebi-a-GCST90017037 | Gut microbiota abundance (genus Oscillospira id.2064) || id:ebi-a-GCST90017037 | CD33 on basophil || id:ebi-a-GCST90001954 | rs1954532 | 14 | 28151415 | C | 0.0826246 | 0.0175252 | 2.27E-06 |
| ebi-a-GCST90017037 | Gut microbiota abundance (genus Oscillospira id.2064) || id:ebi-a-GCST90017037 | CD33 on Immature Myeloid-Derived Suppressor Cells || id:ebi-a-GCST90001955 | rs1954532 | 14 | 28151415 | C | 0.0826246 | 0.0175252 | 2.27E-06 |
| ebi-a-GCST90017037 | Gut microbiota abundance (genus Oscillospira id.2064) || id:ebi-a-GCST90017037 | FSC-A on HLA DR+ CD8+ T cell || id:ebi-a-GCST90001978 | rs1954532 | 14 | 28151415 | C | 0.0826246 | 0.0175252 | 2.27E-06 |
| ebi-a-GCST90017037 | Gut microbiota abundance (genus Oscillospira id.2064) || id:ebi-a-GCST90017037 | HLA DR on CD14+ CD16- monocyte || id:ebi-a-GCST90001988 | rs1954532 | 14 | 28151415 | C | 0.0826246 | 0.0175252 | 2.27E-06 |
| ebi-a-GCST90017037 | Gut microbiota abundance (genus Oscillospira id.2064) || id:ebi-a-GCST90017037 | HLA DR on CD14+ monocyte || id:ebi-a-GCST90001991 | rs1954532 | 14 | 28151415 | C | 0.0826246 | 0.0175252 | 2.27E-06 |
| ebi-a-GCST90017037 | Gut microbiota abundance (genus Oscillospira id.2064) || id:ebi-a-GCST90017037 | CD16 on CD14+ CD16+ monocyte || id:ebi-a-GCST90002005 | rs1954532 | 14 | 28151415 | C | 0.0826246 | 0.0175252 | 2.27E-06 |
| ebi-a-GCST90017037 | Gut microbiota abundance (genus Oscillospira id.2064) || id:ebi-a-GCST90017037 | CD45 on CD33+ HLA DR+ CD14- || id:ebi-a-GCST90002042 | rs1954532 | 14 | 28151415 | C | 0.0826246 | 0.0175252 | 2.27E-06 |
| ebi-a-GCST90017037 | Gut microbiota abundance (genus Oscillospira id.2064) || id:ebi-a-GCST90017037 | CD8 on Natural Killer T || id:ebi-a-GCST90002059 | rs1954532 | 14 | 28151415 | C | 0.0826246 | 0.0175252 | 2.27E-06 |
| ebi-a-GCST90016957 | Gut microbiota abundance (family Verrucomicrobiaceae id.4036) || id:ebi-a-GCST90016957 | Myeloid Dendritic Cell Absolute Count || id:ebi-a-GCST90001458 | rs2602429 | 16 | 81063149 | T | -0.0745516 | 0.0156195 | 2.70E-06 |
| ebi-a-GCST90016957 | Gut microbiota abundance (family Verrucomicrobiaceae id.4036) || id:ebi-a-GCST90016957 | CD86+ myeloid Dendritic Cell %Dendritic Cell || id:ebi-a-GCST90001465 | rs2602429 | 16 | 81063149 | T | -0.0745516 | 0.0156195 | 2.70E-06 |
| ebi-a-GCST90016957 | Gut microbiota abundance (family Verrucomicrobiaceae id.4036) || id:ebi-a-GCST90016957 | CD62L- myeloid Dendritic Cell Absolute Count || id:ebi-a-GCST90001468 | rs2602429 | 16 | 81063149 | T | -0.0745516 | 0.0156195 | 2.70E-06 |
| ebi-a-GCST90016957 | Gut microbiota abundance (family Verrucomicrobiaceae id.4036) || id:ebi-a-GCST90016957 | CD62L- myeloid Dendritic Cell %Dendritic Cell || id:ebi-a-GCST90001469 | rs2602429 | 16 | 81063149 | T | -0.0745516 | 0.0156195 | 2.70E-06 |
| ebi-a-GCST90016957 | Gut microbiota abundance (family Verrucomicrobiaceae id.4036) || id:ebi-a-GCST90016957 | CD62L- CD86+ myeloid Dendritic Cell Absolute Count || id:ebi-a-GCST90001472 | rs2602429 | 16 | 81063149 | T | -0.0745516 | 0.0156195 | 2.70E-06 |
| ebi-a-GCST90016957 | Gut microbiota abundance (family Verrucomicrobiaceae id.4036) || id:ebi-a-GCST90016957 | CD62L- CD86+ myeloid Dendritic Cell %Dendritic Cell || id:ebi-a-GCST90001473 | rs2602429 | 16 | 81063149 | T | -0.0745516 | 0.0156195 | 2.70E-06 |
| ebi-a-GCST90016957 | Gut microbiota abundance (family Verrucomicrobiaceae id.4036) || id:ebi-a-GCST90016957 | HLA DR++ monocyte %monocyte || id:ebi-a-GCST90001475 | rs2602429 | 16 | 81063149 | T | -0.0745516 | 0.0156195 | 2.70E-06 |
| ebi-a-GCST90016957 | Gut microbiota abundance (family Verrucomicrobiaceae id.4036) || id:ebi-a-GCST90016957 | HLA DR++ monocyte Absolute Count || id:ebi-a-GCST90001477 | rs2602429 | 16 | 81063149 | T | -0.0745516 | 0.0156195 | 2.70E-06 |
| ebi-a-GCST90016957 | Gut microbiota abundance (family Verrucomicrobiaceae id.4036) || id:ebi-a-GCST90016957 | Basophil %CD33dim HLA DR- CD66b- || id:ebi-a-GCST90001533 | rs2602429 | 16 | 81063149 | T | -0.0745516 | 0.0156195 | 2.70E-06 |
| ebi-a-GCST90016957 | Gut microbiota abundance (family Verrucomicrobiaceae id.4036) || id:ebi-a-GCST90016957 | CD16+ monocyte %monocyte || id:ebi-a-GCST90001587 | rs2602429 | 16 | 81063149 | T | -0.0745516 | 0.0156195 | 2.70E-06 |
| ebi-a-GCST90016957 | Gut microbiota abundance (family Verrucomicrobiaceae id.4036) || id:ebi-a-GCST90016957 | T/B cell || id:ebi-a-GCST90001588 | rs2602429 | 16 | 81063149 | T | -0.0745516 | 0.0156195 | 2.70E-06 |
| ebi-a-GCST90016957 | Gut microbiota abundance (family Verrucomicrobiaceae id.4036) || id:ebi-a-GCST90016957 | CD8+ Natural Killer T %T cell || id:ebi-a-GCST90001631 | rs2602429 | 16 | 81063149 | T | -0.0745516 | 0.0156195 | 2.70E-06 |
| ebi-a-GCST90016957 | Gut microbiota abundance (family Verrucomicrobiaceae id.4036) || id:ebi-a-GCST90016957 | CD4-CD8- Natural Killer T %lymphocyte || id:ebi-a-GCST90001638 | rs2602429 | 16 | 81063149 | T | -0.0745516 | 0.0156195 | 2.70E-06 |
| ebi-a-GCST90016957 | Gut microbiota abundance (family Verrucomicrobiaceae id.4036) || id:ebi-a-GCST90016957 | HLA DR+ Natural Killer Absolute Count || id:ebi-a-GCST90001648 | rs2602429 | 16 | 81063149 | T | -0.0745516 | 0.0156195 | 2.70E-06 |
| ebi-a-GCST90016957 | Gut microbiota abundance (family Verrucomicrobiaceae id.4036) || id:ebi-a-GCST90016957 | HLA DR+ Natural Killer %Natural Killer || id:ebi-a-GCST90001649 | rs2602429 | 16 | 81063149 | T | -0.0745516 | 0.0156195 | 2.70E-06 |
| ebi-a-GCST90016957 | Gut microbiota abundance (family Verrucomicrobiaceae id.4036) || id:ebi-a-GCST90016957 | HLA DR+ Natural Killer %CD3- lymphocyte || id:ebi-a-GCST90001650 | rs2602429 | 16 | 81063149 | T | -0.0745516 | 0.0156195 | 2.70E-06 |
| ebi-a-GCST90016957 | Gut microbiota abundance (family Verrucomicrobiaceae id.4036) || id:ebi-a-GCST90016957 | CD28- CD25++ CD8+ T cell Absolute Count || id:ebi-a-GCST90001678 | rs2602429 | 16 | 81063149 | T | -0.0745516 | 0.0156195 | 2.70E-06 |
| ebi-a-GCST90016957 | Gut microbiota abundance (family Verrucomicrobiaceae id.4036) || id:ebi-a-GCST90016957 | CD25++ CD8+ T cell %T cell || id:ebi-a-GCST90001679 | rs2602429 | 16 | 81063149 | T | -0.0745516 | 0.0156195 | 2.70E-06 |
| ebi-a-GCST90016957 | Gut microbiota abundance (family Verrucomicrobiaceae id.4036) || id:ebi-a-GCST90016957 | CD19 on CD20- CD38- B cell || id:ebi-a-GCST90001722 | rs2602429 | 16 | 81063149 | T | -0.0745516 | 0.0156195 | 2.70E-06 |
| ebi-a-GCST90016957 | Gut microbiota abundance (family Verrucomicrobiaceae id.4036) || id:ebi-a-GCST90016957 | CD25 on B cell || id:ebi-a-GCST90001775 | rs2602429 | 16 | 81063149 | T | -0.0745516 | 0.0156195 | 2.70E-06 |
| ebi-a-GCST90016957 | Gut microbiota abundance (family Verrucomicrobiaceae id.4036) || id:ebi-a-GCST90016957 | CD25 on IgD+ CD24- B cell || id:ebi-a-GCST90001779 | rs2602429 | 16 | 81063149 | T | -0.0745516 | 0.0156195 | 2.70E-06 |
| ebi-a-GCST90016957 | Gut microbiota abundance (family Verrucomicrobiaceae id.4036) || id:ebi-a-GCST90016957 | CD25 on IgD+ CD38- naive B cell || id:ebi-a-GCST90001781 | rs2602429 | 16 | 81063149 | T | -0.0745516 | 0.0156195 | 2.70E-06 |
| ebi-a-GCST90016957 | Gut microbiota abundance (family Verrucomicrobiaceae id.4036) || id:ebi-a-GCST90016957 | CD25 on IgD+ CD38+ B cell || id:ebi-a-GCST90001783 | rs2602429 | 16 | 81063149 | T | -0.0745516 | 0.0156195 | 2.70E-06 |
| ebi-a-GCST90016957 | Gut microbiota abundance (family Verrucomicrobiaceae id.4036) || id:ebi-a-GCST90016957 | CD86 on myeloid Dendritic Cell || id:ebi-a-GCST90001903 | rs2602429 | 16 | 81063149 | T | -0.0745516 | 0.0156195 | 2.70E-06 |
| ebi-a-GCST90016957 | Gut microbiota abundance (family Verrucomicrobiaceae id.4036) || id:ebi-a-GCST90016957 | CD33 on CD14+ monocyte || id:ebi-a-GCST90001946 | rs2602429 | 16 | 81063149 | T | -0.0745516 | 0.0156195 | 2.70E-06 |
| ebi-a-GCST90016957 | Gut microbiota abundance (family Verrucomicrobiaceae id.4036) || id:ebi-a-GCST90016957 | CD33 on CD33+ HLA DR+ CD14dim || id:ebi-a-GCST90001947 | rs2602429 | 16 | 81063149 | T | -0.0745516 | 0.0156195 | 2.70E-06 |
| ebi-a-GCST90016957 | Gut microbiota abundance (family Verrucomicrobiaceae id.4036) || id:ebi-a-GCST90016957 | CD33 on CD33dim HLA DR+ CD11b+ || id:ebi-a-GCST90001948 | rs2602429 | 16 | 81063149 | T | -0.0745516 | 0.0156195 | 2.70E-06 |
| ebi-a-GCST90016957 | Gut microbiota abundance (family Verrucomicrobiaceae id.4036) || id:ebi-a-GCST90016957 | CD33 on Granulocytic Myeloid-Derived Suppressor Cells || id:ebi-a-GCST90001950 | rs2602429 | 16 | 81063149 | T | -0.0745516 | 0.0156195 | 2.70E-06 |
| ebi-a-GCST90016957 | Gut microbiota abundance (family Verrucomicrobiaceae id.4036) || id:ebi-a-GCST90016957 | CD33 on CD66b++ myeloid cell || id:ebi-a-GCST90001951 | rs2602429 | 16 | 81063149 | T | -0.0745516 | 0.0156195 | 2.70E-06 |
| ebi-a-GCST90016957 | Gut microbiota abundance (family Verrucomicrobiaceae id.4036) || id:ebi-a-GCST90016957 | CD33 on CD33dim HLA DR- || id:ebi-a-GCST90001953 | rs2602429 | 16 | 81063149 | T | -0.0745516 | 0.0156195 | 2.70E-06 |
| ebi-a-GCST90016957 | Gut microbiota abundance (family Verrucomicrobiaceae id.4036) || id:ebi-a-GCST90016957 | CD33 on basophil || id:ebi-a-GCST90001954 | rs2602429 | 16 | 81063149 | T | -0.0745516 | 0.0156195 | 2.70E-06 |
| ebi-a-GCST90016957 | Gut microbiota abundance (family Verrucomicrobiaceae id.4036) || id:ebi-a-GCST90016957 | CD33 on Immature Myeloid-Derived Suppressor Cells || id:ebi-a-GCST90001955 | rs2602429 | 16 | 81063149 | T | -0.0745516 | 0.0156195 | 2.70E-06 |
| ebi-a-GCST90016957 | Gut microbiota abundance (family Verrucomicrobiaceae id.4036) || id:ebi-a-GCST90016957 | FSC-A on HLA DR+ CD8+ T cell || id:ebi-a-GCST90001978 | rs2602429 | 16 | 81063149 | T | -0.0745516 | 0.0156195 | 2.70E-06 |
| ebi-a-GCST90016957 | Gut microbiota abundance (family Verrucomicrobiaceae id.4036) || id:ebi-a-GCST90016957 | HLA DR on CD14+ CD16- monocyte || id:ebi-a-GCST90001988 | rs2602429 | 16 | 81063149 | T | -0.0745516 | 0.0156195 | 2.70E-06 |
| ebi-a-GCST90016957 | Gut microbiota abundance (family Verrucomicrobiaceae id.4036) || id:ebi-a-GCST90016957 | HLA DR on CD14+ monocyte || id:ebi-a-GCST90001991 | rs2602429 | 16 | 81063149 | T | -0.0745516 | 0.0156195 | 2.70E-06 |
| ebi-a-GCST90016957 | Gut microbiota abundance (family Verrucomicrobiaceae id.4036) || id:ebi-a-GCST90016957 | CD16 on CD14+ CD16+ monocyte || id:ebi-a-GCST90002005 | rs2602429 | 16 | 81063149 | T | -0.0745516 | 0.0156195 | 2.70E-06 |
| ebi-a-GCST90016957 | Gut microbiota abundance (family Verrucomicrobiaceae id.4036) || id:ebi-a-GCST90016957 | CD45 on CD33+ HLA DR+ CD14- || id:ebi-a-GCST90002042 | rs2602429 | 16 | 81063149 | T | -0.0745516 | 0.0156195 | 2.70E-06 |
| ebi-a-GCST90016957 | Gut microbiota abundance (family Verrucomicrobiaceae id.4036) || id:ebi-a-GCST90016957 | CD8 on Natural Killer T || id:ebi-a-GCST90002059 | rs2602429 | 16 | 81063149 | T | -0.0745516 | 0.0156195 | 2.70E-06 |
| ebi-a-GCST90016923 | Gut microbiota abundance (class Verrucomicrobiae id.4029) || id:ebi-a-GCST90016923 | Myeloid Dendritic Cell Absolute Count || id:ebi-a-GCST90001458 | rs2602429 | 16 | 81063149 | T | -0.0746844 | 0.0156194 | 2.58E-06 |
| ebi-a-GCST90016923 | Gut microbiota abundance (class Verrucomicrobiae id.4029) || id:ebi-a-GCST90016923 | CD86+ myeloid Dendritic Cell %Dendritic Cell || id:ebi-a-GCST90001465 | rs2602429 | 16 | 81063149 | T | -0.0746844 | 0.0156194 | 2.58E-06 |
| ebi-a-GCST90016923 | Gut microbiota abundance (class Verrucomicrobiae id.4029) || id:ebi-a-GCST90016923 | CD62L- myeloid Dendritic Cell Absolute Count || id:ebi-a-GCST90001468 | rs2602429 | 16 | 81063149 | T | -0.0746844 | 0.0156194 | 2.58E-06 |
| ebi-a-GCST90016923 | Gut microbiota abundance (class Verrucomicrobiae id.4029) || id:ebi-a-GCST90016923 | CD62L- myeloid Dendritic Cell %Dendritic Cell || id:ebi-a-GCST90001469 | rs2602429 | 16 | 81063149 | T | -0.0746844 | 0.0156194 | 2.58E-06 |
| ebi-a-GCST90016923 | Gut microbiota abundance (class Verrucomicrobiae id.4029) || id:ebi-a-GCST90016923 | CD62L- CD86+ myeloid Dendritic Cell Absolute Count || id:ebi-a-GCST90001472 | rs2602429 | 16 | 81063149 | T | -0.0746844 | 0.0156194 | 2.58E-06 |
| ebi-a-GCST90016923 | Gut microbiota abundance (class Verrucomicrobiae id.4029) || id:ebi-a-GCST90016923 | CD62L- CD86+ myeloid Dendritic Cell %Dendritic Cell || id:ebi-a-GCST90001473 | rs2602429 | 16 | 81063149 | T | -0.0746844 | 0.0156194 | 2.58E-06 |
| ebi-a-GCST90016923 | Gut microbiota abundance (class Verrucomicrobiae id.4029) || id:ebi-a-GCST90016923 | HLA DR++ monocyte %monocyte || id:ebi-a-GCST90001475 | rs2602429 | 16 | 81063149 | T | -0.0746844 | 0.0156194 | 2.58E-06 |
| ebi-a-GCST90016923 | Gut microbiota abundance (class Verrucomicrobiae id.4029) || id:ebi-a-GCST90016923 | HLA DR++ monocyte Absolute Count || id:ebi-a-GCST90001477 | rs2602429 | 16 | 81063149 | T | -0.0746844 | 0.0156194 | 2.58E-06 |
| ebi-a-GCST90016923 | Gut microbiota abundance (class Verrucomicrobiae id.4029) || id:ebi-a-GCST90016923 | Basophil %CD33dim HLA DR- CD66b- || id:ebi-a-GCST90001533 | rs2602429 | 16 | 81063149 | T | -0.0746844 | 0.0156194 | 2.58E-06 |
| ebi-a-GCST90016923 | Gut microbiota abundance (class Verrucomicrobiae id.4029) || id:ebi-a-GCST90016923 | CD16+ monocyte %monocyte || id:ebi-a-GCST90001587 | rs2602429 | 16 | 81063149 | T | -0.0746844 | 0.0156194 | 2.58E-06 |
| ebi-a-GCST90016923 | Gut microbiota abundance (class Verrucomicrobiae id.4029) || id:ebi-a-GCST90016923 | T/B cell || id:ebi-a-GCST90001588 | rs2602429 | 16 | 81063149 | T | -0.0746844 | 0.0156194 | 2.58E-06 |
| ebi-a-GCST90016923 | Gut microbiota abundance (class Verrucomicrobiae id.4029) || id:ebi-a-GCST90016923 | CD8+ Natural Killer T %T cell || id:ebi-a-GCST90001631 | rs2602429 | 16 | 81063149 | T | -0.0746844 | 0.0156194 | 2.58E-06 |
| ebi-a-GCST90016923 | Gut microbiota abundance (class Verrucomicrobiae id.4029) || id:ebi-a-GCST90016923 | CD4-CD8- Natural Killer T %lymphocyte || id:ebi-a-GCST90001638 | rs2602429 | 16 | 81063149 | T | -0.0746844 | 0.0156194 | 2.58E-06 |
| ebi-a-GCST90016923 | Gut microbiota abundance (class Verrucomicrobiae id.4029) || id:ebi-a-GCST90016923 | HLA DR+ Natural Killer Absolute Count || id:ebi-a-GCST90001648 | rs2602429 | 16 | 81063149 | T | -0.0746844 | 0.0156194 | 2.58E-06 |
| ebi-a-GCST90016923 | Gut microbiota abundance (class Verrucomicrobiae id.4029) || id:ebi-a-GCST90016923 | HLA DR+ Natural Killer %Natural Killer || id:ebi-a-GCST90001649 | rs2602429 | 16 | 81063149 | T | -0.0746844 | 0.0156194 | 2.58E-06 |
| ebi-a-GCST90016923 | Gut microbiota abundance (class Verrucomicrobiae id.4029) || id:ebi-a-GCST90016923 | HLA DR+ Natural Killer %CD3- lymphocyte || id:ebi-a-GCST90001650 | rs2602429 | 16 | 81063149 | T | -0.0746844 | 0.0156194 | 2.58E-06 |
| ebi-a-GCST90016923 | Gut microbiota abundance (class Verrucomicrobiae id.4029) || id:ebi-a-GCST90016923 | CD28- CD25++ CD8+ T cell Absolute Count || id:ebi-a-GCST90001678 | rs2602429 | 16 | 81063149 | T | -0.0746844 | 0.0156194 | 2.58E-06 |
| ebi-a-GCST90016923 | Gut microbiota abundance (class Verrucomicrobiae id.4029) || id:ebi-a-GCST90016923 | CD25++ CD8+ T cell %T cell || id:ebi-a-GCST90001679 | rs2602429 | 16 | 81063149 | T | -0.0746844 | 0.0156194 | 2.58E-06 |
| ebi-a-GCST90016923 | Gut microbiota abundance (class Verrucomicrobiae id.4029) || id:ebi-a-GCST90016923 | CD19 on CD20- CD38- B cell || id:ebi-a-GCST90001722 | rs2602429 | 16 | 81063149 | T | -0.0746844 | 0.0156194 | 2.58E-06 |
| ebi-a-GCST90016923 | Gut microbiota abundance (class Verrucomicrobiae id.4029) || id:ebi-a-GCST90016923 | CD25 on B cell || id:ebi-a-GCST90001775 | rs2602429 | 16 | 81063149 | T | -0.0746844 | 0.0156194 | 2.58E-06 |
| ebi-a-GCST90016923 | Gut microbiota abundance (class Verrucomicrobiae id.4029) || id:ebi-a-GCST90016923 | CD25 on IgD+ CD24- B cell || id:ebi-a-GCST90001779 | rs2602429 | 16 | 81063149 | T | -0.0746844 | 0.0156194 | 2.58E-06 |
| ebi-a-GCST90016923 | Gut microbiota abundance (class Verrucomicrobiae id.4029) || id:ebi-a-GCST90016923 | CD25 on IgD+ CD38- naive B cell || id:ebi-a-GCST90001781 | rs2602429 | 16 | 81063149 | T | -0.0746844 | 0.0156194 | 2.58E-06 |
| ebi-a-GCST90016923 | Gut microbiota abundance (class Verrucomicrobiae id.4029) || id:ebi-a-GCST90016923 | CD25 on IgD+ CD38+ B cell || id:ebi-a-GCST90001783 | rs2602429 | 16 | 81063149 | T | -0.0746844 | 0.0156194 | 2.58E-06 |
| ebi-a-GCST90016923 | Gut microbiota abundance (class Verrucomicrobiae id.4029) || id:ebi-a-GCST90016923 | CD86 on myeloid Dendritic Cell || id:ebi-a-GCST90001903 | rs2602429 | 16 | 81063149 | T | -0.0746844 | 0.0156194 | 2.58E-06 |
| ebi-a-GCST90016923 | Gut microbiota abundance (class Verrucomicrobiae id.4029) || id:ebi-a-GCST90016923 | CD33 on CD14+ monocyte || id:ebi-a-GCST90001946 | rs2602429 | 16 | 81063149 | T | -0.0746844 | 0.0156194 | 2.58E-06 |
| ebi-a-GCST90016923 | Gut microbiota abundance (class Verrucomicrobiae id.4029) || id:ebi-a-GCST90016923 | CD33 on CD33+ HLA DR+ CD14dim || id:ebi-a-GCST90001947 | rs2602429 | 16 | 81063149 | T | -0.0746844 | 0.0156194 | 2.58E-06 |
| ebi-a-GCST90016923 | Gut microbiota abundance (class Verrucomicrobiae id.4029) || id:ebi-a-GCST90016923 | CD33 on CD33dim HLA DR+ CD11b+ || id:ebi-a-GCST90001948 | rs2602429 | 16 | 81063149 | T | -0.0746844 | 0.0156194 | 2.58E-06 |
| ebi-a-GCST90016923 | Gut microbiota abundance (class Verrucomicrobiae id.4029) || id:ebi-a-GCST90016923 | CD33 on Granulocytic Myeloid-Derived Suppressor Cells || id:ebi-a-GCST90001950 | rs2602429 | 16 | 81063149 | T | -0.0746844 | 0.0156194 | 2.58E-06 |
| ebi-a-GCST90016923 | Gut microbiota abundance (class Verrucomicrobiae id.4029) || id:ebi-a-GCST90016923 | CD33 on CD66b++ myeloid cell || id:ebi-a-GCST90001951 | rs2602429 | 16 | 81063149 | T | -0.0746844 | 0.0156194 | 2.58E-06 |
| ebi-a-GCST90016923 | Gut microbiota abundance (class Verrucomicrobiae id.4029) || id:ebi-a-GCST90016923 | CD33 on CD33dim HLA DR- || id:ebi-a-GCST90001953 | rs2602429 | 16 | 81063149 | T | -0.0746844 | 0.0156194 | 2.58E-06 |
| ebi-a-GCST90016923 | Gut microbiota abundance (class Verrucomicrobiae id.4029) || id:ebi-a-GCST90016923 | CD33 on basophil || id:ebi-a-GCST90001954 | rs2602429 | 16 | 81063149 | T | -0.0746844 | 0.0156194 | 2.58E-06 |
| ebi-a-GCST90016923 | Gut microbiota abundance (class Verrucomicrobiae id.4029) || id:ebi-a-GCST90016923 | CD33 on Immature Myeloid-Derived Suppressor Cells || id:ebi-a-GCST90001955 | rs2602429 | 16 | 81063149 | T | -0.0746844 | 0.0156194 | 2.58E-06 |
| ebi-a-GCST90016923 | Gut microbiota abundance (class Verrucomicrobiae id.4029) || id:ebi-a-GCST90016923 | FSC-A on HLA DR+ CD8+ T cell || id:ebi-a-GCST90001978 | rs2602429 | 16 | 81063149 | T | -0.0746844 | 0.0156194 | 2.58E-06 |
| ebi-a-GCST90016923 | Gut microbiota abundance (class Verrucomicrobiae id.4029) || id:ebi-a-GCST90016923 | HLA DR on CD14+ CD16- monocyte || id:ebi-a-GCST90001988 | rs2602429 | 16 | 81063149 | T | -0.0746844 | 0.0156194 | 2.58E-06 |
| ebi-a-GCST90016923 | Gut microbiota abundance (class Verrucomicrobiae id.4029) || id:ebi-a-GCST90016923 | HLA DR on CD14+ monocyte || id:ebi-a-GCST90001991 | rs2602429 | 16 | 81063149 | T | -0.0746844 | 0.0156194 | 2.58E-06 |
| ebi-a-GCST90016923 | Gut microbiota abundance (class Verrucomicrobiae id.4029) || id:ebi-a-GCST90016923 | CD16 on CD14+ CD16+ monocyte || id:ebi-a-GCST90002005 | rs2602429 | 16 | 81063149 | T | -0.0746844 | 0.0156194 | 2.58E-06 |
| ebi-a-GCST90016923 | Gut microbiota abundance (class Verrucomicrobiae id.4029) || id:ebi-a-GCST90016923 | CD45 on CD33+ HLA DR+ CD14- || id:ebi-a-GCST90002042 | rs2602429 | 16 | 81063149 | T | -0.0746844 | 0.0156194 | 2.58E-06 |
| ebi-a-GCST90016923 | Gut microbiota abundance (class Verrucomicrobiae id.4029) || id:ebi-a-GCST90016923 | CD8 on Natural Killer T || id:ebi-a-GCST90002059 | rs2602429 | 16 | 81063149 | T | -0.0746844 | 0.0156194 | 2.58E-06 |
| ebi-a-GCST90017108 | Gut microbiota abundance (order Verrucomicrobiales id.4030) || id:ebi-a-GCST90017108 | Myeloid Dendritic Cell Absolute Count || id:ebi-a-GCST90001458 | rs2602429 | 16 | 81063149 | T | -0.0746844 | 0.0156194 | 2.58E-06 |
| ebi-a-GCST90017108 | Gut microbiota abundance (order Verrucomicrobiales id.4030) || id:ebi-a-GCST90017108 | CD86+ myeloid Dendritic Cell %Dendritic Cell || id:ebi-a-GCST90001465 | rs2602429 | 16 | 81063149 | T | -0.0746844 | 0.0156194 | 2.58E-06 |
| ebi-a-GCST90017108 | Gut microbiota abundance (order Verrucomicrobiales id.4030) || id:ebi-a-GCST90017108 | CD62L- myeloid Dendritic Cell Absolute Count || id:ebi-a-GCST90001468 | rs2602429 | 16 | 81063149 | T | -0.0746844 | 0.0156194 | 2.58E-06 |
| ebi-a-GCST90017108 | Gut microbiota abundance (order Verrucomicrobiales id.4030) || id:ebi-a-GCST90017108 | CD62L- myeloid Dendritic Cell %Dendritic Cell || id:ebi-a-GCST90001469 | rs2602429 | 16 | 81063149 | T | -0.0746844 | 0.0156194 | 2.58E-06 |
| ebi-a-GCST90017108 | Gut microbiota abundance (order Verrucomicrobiales id.4030) || id:ebi-a-GCST90017108 | CD62L- CD86+ myeloid Dendritic Cell Absolute Count || id:ebi-a-GCST90001472 | rs2602429 | 16 | 81063149 | T | -0.0746844 | 0.0156194 | 2.58E-06 |
| ebi-a-GCST90017108 | Gut microbiota abundance (order Verrucomicrobiales id.4030) || id:ebi-a-GCST90017108 | CD62L- CD86+ myeloid Dendritic Cell %Dendritic Cell || id:ebi-a-GCST90001473 | rs2602429 | 16 | 81063149 | T | -0.0746844 | 0.0156194 | 2.58E-06 |
| ebi-a-GCST90017108 | Gut microbiota abundance (order Verrucomicrobiales id.4030) || id:ebi-a-GCST90017108 | HLA DR++ monocyte %monocyte || id:ebi-a-GCST90001475 | rs2602429 | 16 | 81063149 | T | -0.0746844 | 0.0156194 | 2.58E-06 |
| ebi-a-GCST90017108 | Gut microbiota abundance (order Verrucomicrobiales id.4030) || id:ebi-a-GCST90017108 | HLA DR++ monocyte Absolute Count || id:ebi-a-GCST90001477 | rs2602429 | 16 | 81063149 | T | -0.0746844 | 0.0156194 | 2.58E-06 |
| ebi-a-GCST90017108 | Gut microbiota abundance (order Verrucomicrobiales id.4030) || id:ebi-a-GCST90017108 | Basophil %CD33dim HLA DR- CD66b- || id:ebi-a-GCST90001533 | rs2602429 | 16 | 81063149 | T | -0.0746844 | 0.0156194 | 2.58E-06 |
| ebi-a-GCST90017108 | Gut microbiota abundance (order Verrucomicrobiales id.4030) || id:ebi-a-GCST90017108 | CD16+ monocyte %monocyte || id:ebi-a-GCST90001587 | rs2602429 | 16 | 81063149 | T | -0.0746844 | 0.0156194 | 2.58E-06 |
| ebi-a-GCST90017108 | Gut microbiota abundance (order Verrucomicrobiales id.4030) || id:ebi-a-GCST90017108 | T/B cell || id:ebi-a-GCST90001588 | rs2602429 | 16 | 81063149 | T | -0.0746844 | 0.0156194 | 2.58E-06 |
| ebi-a-GCST90017108 | Gut microbiota abundance (order Verrucomicrobiales id.4030) || id:ebi-a-GCST90017108 | CD8+ Natural Killer T %T cell || id:ebi-a-GCST90001631 | rs2602429 | 16 | 81063149 | T | -0.0746844 | 0.0156194 | 2.58E-06 |
| ebi-a-GCST90017108 | Gut microbiota abundance (order Verrucomicrobiales id.4030) || id:ebi-a-GCST90017108 | CD4-CD8- Natural Killer T %lymphocyte || id:ebi-a-GCST90001638 | rs2602429 | 16 | 81063149 | T | -0.0746844 | 0.0156194 | 2.58E-06 |
| ebi-a-GCST90017108 | Gut microbiota abundance (order Verrucomicrobiales id.4030) || id:ebi-a-GCST90017108 | HLA DR+ Natural Killer Absolute Count || id:ebi-a-GCST90001648 | rs2602429 | 16 | 81063149 | T | -0.0746844 | 0.0156194 | 2.58E-06 |
| ebi-a-GCST90017108 | Gut microbiota abundance (order Verrucomicrobiales id.4030) || id:ebi-a-GCST90017108 | HLA DR+ Natural Killer %Natural Killer || id:ebi-a-GCST90001649 | rs2602429 | 16 | 81063149 | T | -0.0746844 | 0.0156194 | 2.58E-06 |
| ebi-a-GCST90017108 | Gut microbiota abundance (order Verrucomicrobiales id.4030) || id:ebi-a-GCST90017108 | HLA DR+ Natural Killer %CD3- lymphocyte || id:ebi-a-GCST90001650 | rs2602429 | 16 | 81063149 | T | -0.0746844 | 0.0156194 | 2.58E-06 |
| ebi-a-GCST90017108 | Gut microbiota abundance (order Verrucomicrobiales id.4030) || id:ebi-a-GCST90017108 | CD28- CD25++ CD8+ T cell Absolute Count || id:ebi-a-GCST90001678 | rs2602429 | 16 | 81063149 | T | -0.0746844 | 0.0156194 | 2.58E-06 |
| ebi-a-GCST90017108 | Gut microbiota abundance (order Verrucomicrobiales id.4030) || id:ebi-a-GCST90017108 | CD25++ CD8+ T cell %T cell || id:ebi-a-GCST90001679 | rs2602429 | 16 | 81063149 | T | -0.0746844 | 0.0156194 | 2.58E-06 |
| ebi-a-GCST90017108 | Gut microbiota abundance (order Verrucomicrobiales id.4030) || id:ebi-a-GCST90017108 | CD19 on CD20- CD38- B cell || id:ebi-a-GCST90001722 | rs2602429 | 16 | 81063149 | T | -0.0746844 | 0.0156194 | 2.58E-06 |
| ebi-a-GCST90017108 | Gut microbiota abundance (order Verrucomicrobiales id.4030) || id:ebi-a-GCST90017108 | CD25 on B cell || id:ebi-a-GCST90001775 | rs2602429 | 16 | 81063149 | T | -0.0746844 | 0.0156194 | 2.58E-06 |
| ebi-a-GCST90017108 | Gut microbiota abundance (order Verrucomicrobiales id.4030) || id:ebi-a-GCST90017108 | CD25 on IgD+ CD24- B cell || id:ebi-a-GCST90001779 | rs2602429 | 16 | 81063149 | T | -0.0746844 | 0.0156194 | 2.58E-06 |
| ebi-a-GCST90017108 | Gut microbiota abundance (order Verrucomicrobiales id.4030) || id:ebi-a-GCST90017108 | CD25 on IgD+ CD38- naive B cell || id:ebi-a-GCST90001781 | rs2602429 | 16 | 81063149 | T | -0.0746844 | 0.0156194 | 2.58E-06 |
| ebi-a-GCST90017108 | Gut microbiota abundance (order Verrucomicrobiales id.4030) || id:ebi-a-GCST90017108 | CD25 on IgD+ CD38+ B cell || id:ebi-a-GCST90001783 | rs2602429 | 16 | 81063149 | T | -0.0746844 | 0.0156194 | 2.58E-06 |
| ebi-a-GCST90017108 | Gut microbiota abundance (order Verrucomicrobiales id.4030) || id:ebi-a-GCST90017108 | CD86 on myeloid Dendritic Cell || id:ebi-a-GCST90001903 | rs2602429 | 16 | 81063149 | T | -0.0746844 | 0.0156194 | 2.58E-06 |
| ebi-a-GCST90017108 | Gut microbiota abundance (order Verrucomicrobiales id.4030) || id:ebi-a-GCST90017108 | CD33 on CD14+ monocyte || id:ebi-a-GCST90001946 | rs2602429 | 16 | 81063149 | T | -0.0746844 | 0.0156194 | 2.58E-06 |
| ebi-a-GCST90017108 | Gut microbiota abundance (order Verrucomicrobiales id.4030) || id:ebi-a-GCST90017108 | CD33 on CD33+ HLA DR+ CD14dim || id:ebi-a-GCST90001947 | rs2602429 | 16 | 81063149 | T | -0.0746844 | 0.0156194 | 2.58E-06 |
| ebi-a-GCST90017108 | Gut microbiota abundance (order Verrucomicrobiales id.4030) || id:ebi-a-GCST90017108 | CD33 on CD33dim HLA DR+ CD11b+ || id:ebi-a-GCST90001948 | rs2602429 | 16 | 81063149 | T | -0.0746844 | 0.0156194 | 2.58E-06 |
| ebi-a-GCST90017108 | Gut microbiota abundance (order Verrucomicrobiales id.4030) || id:ebi-a-GCST90017108 | CD33 on Granulocytic Myeloid-Derived Suppressor Cells || id:ebi-a-GCST90001950 | rs2602429 | 16 | 81063149 | T | -0.0746844 | 0.0156194 | 2.58E-06 |
| ebi-a-GCST90017108 | Gut microbiota abundance (order Verrucomicrobiales id.4030) || id:ebi-a-GCST90017108 | CD33 on CD66b++ myeloid cell || id:ebi-a-GCST90001951 | rs2602429 | 16 | 81063149 | T | -0.0746844 | 0.0156194 | 2.58E-06 |
| ebi-a-GCST90017108 | Gut microbiota abundance (order Verrucomicrobiales id.4030) || id:ebi-a-GCST90017108 | CD33 on CD33dim HLA DR- || id:ebi-a-GCST90001953 | rs2602429 | 16 | 81063149 | T | -0.0746844 | 0.0156194 | 2.58E-06 |
| ebi-a-GCST90017108 | Gut microbiota abundance (order Verrucomicrobiales id.4030) || id:ebi-a-GCST90017108 | CD33 on basophil || id:ebi-a-GCST90001954 | rs2602429 | 16 | 81063149 | T | -0.0746844 | 0.0156194 | 2.58E-06 |
| ebi-a-GCST90017108 | Gut microbiota abundance (order Verrucomicrobiales id.4030) || id:ebi-a-GCST90017108 | CD33 on Immature Myeloid-Derived Suppressor Cells || id:ebi-a-GCST90001955 | rs2602429 | 16 | 81063149 | T | -0.0746844 | 0.0156194 | 2.58E-06 |
| ebi-a-GCST90017108 | Gut microbiota abundance (order Verrucomicrobiales id.4030) || id:ebi-a-GCST90017108 | FSC-A on HLA DR+ CD8+ T cell || id:ebi-a-GCST90001978 | rs2602429 | 16 | 81063149 | T | -0.0746844 | 0.0156194 | 2.58E-06 |
| ebi-a-GCST90017108 | Gut microbiota abundance (order Verrucomicrobiales id.4030) || id:ebi-a-GCST90017108 | HLA DR on CD14+ CD16- monocyte || id:ebi-a-GCST90001988 | rs2602429 | 16 | 81063149 | T | -0.0746844 | 0.0156194 | 2.58E-06 |
| ebi-a-GCST90017108 | Gut microbiota abundance (order Verrucomicrobiales id.4030) || id:ebi-a-GCST90017108 | HLA DR on CD14+ monocyte || id:ebi-a-GCST90001991 | rs2602429 | 16 | 81063149 | T | -0.0746844 | 0.0156194 | 2.58E-06 |
| ebi-a-GCST90017108 | Gut microbiota abundance (order Verrucomicrobiales id.4030) || id:ebi-a-GCST90017108 | CD16 on CD14+ CD16+ monocyte || id:ebi-a-GCST90002005 | rs2602429 | 16 | 81063149 | T | -0.0746844 | 0.0156194 | 2.58E-06 |
| ebi-a-GCST90017108 | Gut microbiota abundance (order Verrucomicrobiales id.4030) || id:ebi-a-GCST90017108 | CD45 on CD33+ HLA DR+ CD14- || id:ebi-a-GCST90002042 | rs2602429 | 16 | 81063149 | T | -0.0746844 | 0.0156194 | 2.58E-06 |
| ebi-a-GCST90017108 | Gut microbiota abundance (order Verrucomicrobiales id.4030) || id:ebi-a-GCST90017108 | CD8 on Natural Killer T || id:ebi-a-GCST90002059 | rs2602429 | 16 | 81063149 | T | -0.0746844 | 0.0156194 | 2.58E-06 |
| ebi-a-GCST90016961 | Gut microbiota abundance (genus Akkermansia id.4037) || id:ebi-a-GCST90016961 | Myeloid Dendritic Cell Absolute Count || id:ebi-a-GCST90001458 | rs2602429 | 16 | 81063149 | T | -0.0745352 | 0.0156201 | 2.72E-06 |
| ebi-a-GCST90016961 | Gut microbiota abundance (genus Akkermansia id.4037) || id:ebi-a-GCST90016961 | CD86+ myeloid Dendritic Cell %Dendritic Cell || id:ebi-a-GCST90001465 | rs2602429 | 16 | 81063149 | T | -0.0745352 | 0.0156201 | 2.72E-06 |
| ebi-a-GCST90016961 | Gut microbiota abundance (genus Akkermansia id.4037) || id:ebi-a-GCST90016961 | CD62L- myeloid Dendritic Cell Absolute Count || id:ebi-a-GCST90001468 | rs2602429 | 16 | 81063149 | T | -0.0745352 | 0.0156201 | 2.72E-06 |
| ebi-a-GCST90016961 | Gut microbiota abundance (genus Akkermansia id.4037) || id:ebi-a-GCST90016961 | CD62L- myeloid Dendritic Cell %Dendritic Cell || id:ebi-a-GCST90001469 | rs2602429 | 16 | 81063149 | T | -0.0745352 | 0.0156201 | 2.72E-06 |
| ebi-a-GCST90016961 | Gut microbiota abundance (genus Akkermansia id.4037) || id:ebi-a-GCST90016961 | CD62L- CD86+ myeloid Dendritic Cell Absolute Count || id:ebi-a-GCST90001472 | rs2602429 | 16 | 81063149 | T | -0.0745352 | 0.0156201 | 2.72E-06 |
| ebi-a-GCST90016961 | Gut microbiota abundance (genus Akkermansia id.4037) || id:ebi-a-GCST90016961 | CD62L- CD86+ myeloid Dendritic Cell %Dendritic Cell || id:ebi-a-GCST90001473 | rs2602429 | 16 | 81063149 | T | -0.0745352 | 0.0156201 | 2.72E-06 |
| ebi-a-GCST90016961 | Gut microbiota abundance (genus Akkermansia id.4037) || id:ebi-a-GCST90016961 | HLA DR++ monocyte %monocyte || id:ebi-a-GCST90001475 | rs2602429 | 16 | 81063149 | T | -0.0745352 | 0.0156201 | 2.72E-06 |
| ebi-a-GCST90016961 | Gut microbiota abundance (genus Akkermansia id.4037) || id:ebi-a-GCST90016961 | HLA DR++ monocyte Absolute Count || id:ebi-a-GCST90001477 | rs2602429 | 16 | 81063149 | T | -0.0745352 | 0.0156201 | 2.72E-06 |
| ebi-a-GCST90016961 | Gut microbiota abundance (genus Akkermansia id.4037) || id:ebi-a-GCST90016961 | Basophil %CD33dim HLA DR- CD66b- || id:ebi-a-GCST90001533 | rs2602429 | 16 | 81063149 | T | -0.0745352 | 0.0156201 | 2.72E-06 |
| ebi-a-GCST90016961 | Gut microbiota abundance (genus Akkermansia id.4037) || id:ebi-a-GCST90016961 | CD16+ monocyte %monocyte || id:ebi-a-GCST90001587 | rs2602429 | 16 | 81063149 | T | -0.0745352 | 0.0156201 | 2.72E-06 |
| ebi-a-GCST90016961 | Gut microbiota abundance (genus Akkermansia id.4037) || id:ebi-a-GCST90016961 | T/B cell || id:ebi-a-GCST90001588 | rs2602429 | 16 | 81063149 | T | -0.0745352 | 0.0156201 | 2.72E-06 |
| ebi-a-GCST90016961 | Gut microbiota abundance (genus Akkermansia id.4037) || id:ebi-a-GCST90016961 | CD8+ Natural Killer T %T cell || id:ebi-a-GCST90001631 | rs2602429 | 16 | 81063149 | T | -0.0745352 | 0.0156201 | 2.72E-06 |
| ebi-a-GCST90016961 | Gut microbiota abundance (genus Akkermansia id.4037) || id:ebi-a-GCST90016961 | CD4-CD8- Natural Killer T %lymphocyte || id:ebi-a-GCST90001638 | rs2602429 | 16 | 81063149 | T | -0.0745352 | 0.0156201 | 2.72E-06 |
| ebi-a-GCST90016961 | Gut microbiota abundance (genus Akkermansia id.4037) || id:ebi-a-GCST90016961 | HLA DR+ Natural Killer Absolute Count || id:ebi-a-GCST90001648 | rs2602429 | 16 | 81063149 | T | -0.0745352 | 0.0156201 | 2.72E-06 |
| ebi-a-GCST90016961 | Gut microbiota abundance (genus Akkermansia id.4037) || id:ebi-a-GCST90016961 | HLA DR+ Natural Killer %Natural Killer || id:ebi-a-GCST90001649 | rs2602429 | 16 | 81063149 | T | -0.0745352 | 0.0156201 | 2.72E-06 |
| ebi-a-GCST90016961 | Gut microbiota abundance (genus Akkermansia id.4037) || id:ebi-a-GCST90016961 | HLA DR+ Natural Killer %CD3- lymphocyte || id:ebi-a-GCST90001650 | rs2602429 | 16 | 81063149 | T | -0.0745352 | 0.0156201 | 2.72E-06 |
| ebi-a-GCST90016961 | Gut microbiota abundance (genus Akkermansia id.4037) || id:ebi-a-GCST90016961 | CD28- CD25++ CD8+ T cell Absolute Count || id:ebi-a-GCST90001678 | rs2602429 | 16 | 81063149 | T | -0.0745352 | 0.0156201 | 2.72E-06 |
| ebi-a-GCST90016961 | Gut microbiota abundance (genus Akkermansia id.4037) || id:ebi-a-GCST90016961 | CD25++ CD8+ T cell %T cell || id:ebi-a-GCST90001679 | rs2602429 | 16 | 81063149 | T | -0.0745352 | 0.0156201 | 2.72E-06 |
| ebi-a-GCST90016961 | Gut microbiota abundance (genus Akkermansia id.4037) || id:ebi-a-GCST90016961 | CD19 on CD20- CD38- B cell || id:ebi-a-GCST90001722 | rs2602429 | 16 | 81063149 | T | -0.0745352 | 0.0156201 | 2.72E-06 |
| ebi-a-GCST90016961 | Gut microbiota abundance (genus Akkermansia id.4037) || id:ebi-a-GCST90016961 | CD25 on B cell || id:ebi-a-GCST90001775 | rs2602429 | 16 | 81063149 | T | -0.0745352 | 0.0156201 | 2.72E-06 |
| ebi-a-GCST90016961 | Gut microbiota abundance (genus Akkermansia id.4037) || id:ebi-a-GCST90016961 | CD25 on IgD+ CD24- B cell || id:ebi-a-GCST90001779 | rs2602429 | 16 | 81063149 | T | -0.0745352 | 0.0156201 | 2.72E-06 |
| ebi-a-GCST90016961 | Gut microbiota abundance (genus Akkermansia id.4037) || id:ebi-a-GCST90016961 | CD25 on IgD+ CD38- naive B cell || id:ebi-a-GCST90001781 | rs2602429 | 16 | 81063149 | T | -0.0745352 | 0.0156201 | 2.72E-06 |
| ebi-a-GCST90016961 | Gut microbiota abundance (genus Akkermansia id.4037) || id:ebi-a-GCST90016961 | CD25 on IgD+ CD38+ B cell || id:ebi-a-GCST90001783 | rs2602429 | 16 | 81063149 | T | -0.0745352 | 0.0156201 | 2.72E-06 |
| ebi-a-GCST90016961 | Gut microbiota abundance (genus Akkermansia id.4037) || id:ebi-a-GCST90016961 | CD86 on myeloid Dendritic Cell || id:ebi-a-GCST90001903 | rs2602429 | 16 | 81063149 | T | -0.0745352 | 0.0156201 | 2.72E-06 |
| ebi-a-GCST90016961 | Gut microbiota abundance (genus Akkermansia id.4037) || id:ebi-a-GCST90016961 | CD33 on CD14+ monocyte || id:ebi-a-GCST90001946 | rs2602429 | 16 | 81063149 | T | -0.0745352 | 0.0156201 | 2.72E-06 |
| ebi-a-GCST90016961 | Gut microbiota abundance (genus Akkermansia id.4037) || id:ebi-a-GCST90016961 | CD33 on CD33+ HLA DR+ CD14dim || id:ebi-a-GCST90001947 | rs2602429 | 16 | 81063149 | T | -0.0745352 | 0.0156201 | 2.72E-06 |
| ebi-a-GCST90016961 | Gut microbiota abundance (genus Akkermansia id.4037) || id:ebi-a-GCST90016961 | CD33 on CD33dim HLA DR+ CD11b+ || id:ebi-a-GCST90001948 | rs2602429 | 16 | 81063149 | T | -0.0745352 | 0.0156201 | 2.72E-06 |
| ebi-a-GCST90016961 | Gut microbiota abundance (genus Akkermansia id.4037) || id:ebi-a-GCST90016961 | CD33 on Granulocytic Myeloid-Derived Suppressor Cells || id:ebi-a-GCST90001950 | rs2602429 | 16 | 81063149 | T | -0.0745352 | 0.0156201 | 2.72E-06 |
| ebi-a-GCST90016961 | Gut microbiota abundance (genus Akkermansia id.4037) || id:ebi-a-GCST90016961 | CD33 on CD66b++ myeloid cell || id:ebi-a-GCST90001951 | rs2602429 | 16 | 81063149 | T | -0.0745352 | 0.0156201 | 2.72E-06 |
| ebi-a-GCST90016961 | Gut microbiota abundance (genus Akkermansia id.4037) || id:ebi-a-GCST90016961 | CD33 on CD33dim HLA DR- || id:ebi-a-GCST90001953 | rs2602429 | 16 | 81063149 | T | -0.0745352 | 0.0156201 | 2.72E-06 |
| ebi-a-GCST90016961 | Gut microbiota abundance (genus Akkermansia id.4037) || id:ebi-a-GCST90016961 | CD33 on basophil || id:ebi-a-GCST90001954 | rs2602429 | 16 | 81063149 | T | -0.0745352 | 0.0156201 | 2.72E-06 |
| ebi-a-GCST90016961 | Gut microbiota abundance (genus Akkermansia id.4037) || id:ebi-a-GCST90016961 | CD33 on Immature Myeloid-Derived Suppressor Cells || id:ebi-a-GCST90001955 | rs2602429 | 16 | 81063149 | T | -0.0745352 | 0.0156201 | 2.72E-06 |
| ebi-a-GCST90016961 | Gut microbiota abundance (genus Akkermansia id.4037) || id:ebi-a-GCST90016961 | FSC-A on HLA DR+ CD8+ T cell || id:ebi-a-GCST90001978 | rs2602429 | 16 | 81063149 | T | -0.0745352 | 0.0156201 | 2.72E-06 |
| ebi-a-GCST90016961 | Gut microbiota abundance (genus Akkermansia id.4037) || id:ebi-a-GCST90016961 | HLA DR on CD14+ CD16- monocyte || id:ebi-a-GCST90001988 | rs2602429 | 16 | 81063149 | T | -0.0745352 | 0.0156201 | 2.72E-06 |
| ebi-a-GCST90016961 | Gut microbiota abundance (genus Akkermansia id.4037) || id:ebi-a-GCST90016961 | HLA DR on CD14+ monocyte || id:ebi-a-GCST90001991 | rs2602429 | 16 | 81063149 | T | -0.0745352 | 0.0156201 | 2.72E-06 |
| ebi-a-GCST90016961 | Gut microbiota abundance (genus Akkermansia id.4037) || id:ebi-a-GCST90016961 | CD16 on CD14+ CD16+ monocyte || id:ebi-a-GCST90002005 | rs2602429 | 16 | 81063149 | T | -0.0745352 | 0.0156201 | 2.72E-06 |
| ebi-a-GCST90016961 | Gut microbiota abundance (genus Akkermansia id.4037) || id:ebi-a-GCST90016961 | CD45 on CD33+ HLA DR+ CD14- || id:ebi-a-GCST90002042 | rs2602429 | 16 | 81063149 | T | -0.0745352 | 0.0156201 | 2.72E-06 |
| ebi-a-GCST90016961 | Gut microbiota abundance (genus Akkermansia id.4037) || id:ebi-a-GCST90016961 | CD8 on Natural Killer T || id:ebi-a-GCST90002059 | rs2602429 | 16 | 81063149 | T | -0.0745352 | 0.0156201 | 2.72E-06 |
| ebi-a-GCST90017037 | Gut microbiota abundance (genus Oscillospira id.2064) || id:ebi-a-GCST90017037 | Myeloid Dendritic Cell Absolute Count || id:ebi-a-GCST90001458 | rs28889936 | 4 | 89483300 | A | 0.114051 | 0.0252838 | 3.37E-06 |
| ebi-a-GCST90017037 | Gut microbiota abundance (genus Oscillospira id.2064) || id:ebi-a-GCST90017037 | CD86+ myeloid Dendritic Cell %Dendritic Cell || id:ebi-a-GCST90001465 | rs28889936 | 4 | 89483300 | A | 0.114051 | 0.0252838 | 3.37E-06 |
| ebi-a-GCST90017037 | Gut microbiota abundance (genus Oscillospira id.2064) || id:ebi-a-GCST90017037 | CD62L- myeloid Dendritic Cell Absolute Count || id:ebi-a-GCST90001468 | rs28889936 | 4 | 89483300 | A | 0.114051 | 0.0252838 | 3.37E-06 |
| ebi-a-GCST90017037 | Gut microbiota abundance (genus Oscillospira id.2064) || id:ebi-a-GCST90017037 | CD62L- myeloid Dendritic Cell %Dendritic Cell || id:ebi-a-GCST90001469 | rs28889936 | 4 | 89483300 | A | 0.114051 | 0.0252838 | 3.37E-06 |
| ebi-a-GCST90017037 | Gut microbiota abundance (genus Oscillospira id.2064) || id:ebi-a-GCST90017037 | CD62L- CD86+ myeloid Dendritic Cell Absolute Count || id:ebi-a-GCST90001472 | rs28889936 | 4 | 89483300 | A | 0.114051 | 0.0252838 | 3.37E-06 |
| ebi-a-GCST90017037 | Gut microbiota abundance (genus Oscillospira id.2064) || id:ebi-a-GCST90017037 | CD62L- CD86+ myeloid Dendritic Cell %Dendritic Cell || id:ebi-a-GCST90001473 | rs28889936 | 4 | 89483300 | A | 0.114051 | 0.0252838 | 3.37E-06 |
| ebi-a-GCST90017037 | Gut microbiota abundance (genus Oscillospira id.2064) || id:ebi-a-GCST90017037 | HLA DR++ monocyte %monocyte || id:ebi-a-GCST90001475 | rs28889936 | 4 | 89483300 | A | 0.114051 | 0.0252838 | 3.37E-06 |
| ebi-a-GCST90017037 | Gut microbiota abundance (genus Oscillospira id.2064) || id:ebi-a-GCST90017037 | HLA DR++ monocyte Absolute Count || id:ebi-a-GCST90001477 | rs28889936 | 4 | 89483300 | A | 0.114051 | 0.0252838 | 3.37E-06 |
| ebi-a-GCST90017037 | Gut microbiota abundance (genus Oscillospira id.2064) || id:ebi-a-GCST90017037 | Basophil %CD33dim HLA DR- CD66b- || id:ebi-a-GCST90001533 | rs28889936 | 4 | 89483300 | A | 0.114051 | 0.0252838 | 3.37E-06 |
| ebi-a-GCST90017037 | Gut microbiota abundance (genus Oscillospira id.2064) || id:ebi-a-GCST90017037 | CD16+ monocyte %monocyte || id:ebi-a-GCST90001587 | rs28889936 | 4 | 89483300 | A | 0.114051 | 0.0252838 | 3.37E-06 |
| ebi-a-GCST90017037 | Gut microbiota abundance (genus Oscillospira id.2064) || id:ebi-a-GCST90017037 | T/B cell || id:ebi-a-GCST90001588 | rs28889936 | 4 | 89483300 | A | 0.114051 | 0.0252838 | 3.37E-06 |
| ebi-a-GCST90017037 | Gut microbiota abundance (genus Oscillospira id.2064) || id:ebi-a-GCST90017037 | CD4+CD8+ T cell %T cell || id:ebi-a-GCST90001595 | rs28889936 | 4 | 89483300 | A | 0.114051 | 0.0252838 | 3.37E-06 |
| ebi-a-GCST90017037 | Gut microbiota abundance (genus Oscillospira id.2064) || id:ebi-a-GCST90017037 | CD8+ Natural Killer T %T cell || id:ebi-a-GCST90001631 | rs28889936 | 4 | 89483300 | A | 0.114051 | 0.0252838 | 3.37E-06 |
| ebi-a-GCST90017037 | Gut microbiota abundance (genus Oscillospira id.2064) || id:ebi-a-GCST90017037 | CD4-CD8- Natural Killer T %lymphocyte || id:ebi-a-GCST90001638 | rs28889936 | 4 | 89483300 | A | 0.114051 | 0.0252838 | 3.37E-06 |
| ebi-a-GCST90017037 | Gut microbiota abundance (genus Oscillospira id.2064) || id:ebi-a-GCST90017037 | HLA DR+ Natural Killer Absolute Count || id:ebi-a-GCST90001648 | rs28889936 | 4 | 89483300 | A | 0.114051 | 0.0252838 | 3.37E-06 |
| ebi-a-GCST90017037 | Gut microbiota abundance (genus Oscillospira id.2064) || id:ebi-a-GCST90017037 | HLA DR+ Natural Killer %Natural Killer || id:ebi-a-GCST90001649 | rs28889936 | 4 | 89483300 | A | 0.114051 | 0.0252838 | 3.37E-06 |
| ebi-a-GCST90017037 | Gut microbiota abundance (genus Oscillospira id.2064) || id:ebi-a-GCST90017037 | HLA DR+ Natural Killer %CD3- lymphocyte || id:ebi-a-GCST90001650 | rs28889936 | 4 | 89483300 | A | 0.114051 | 0.0252838 | 3.37E-06 |
| ebi-a-GCST90017037 | Gut microbiota abundance (genus Oscillospira id.2064) || id:ebi-a-GCST90017037 | CD28- CD25++ CD8+ T cell Absolute Count || id:ebi-a-GCST90001678 | rs28889936 | 4 | 89483300 | A | 0.114051 | 0.0252838 | 3.37E-06 |
| ebi-a-GCST90017037 | Gut microbiota abundance (genus Oscillospira id.2064) || id:ebi-a-GCST90017037 | CD25++ CD8+ T cell %T cell || id:ebi-a-GCST90001679 | rs28889936 | 4 | 89483300 | A | 0.114051 | 0.0252838 | 3.37E-06 |
| ebi-a-GCST90017037 | Gut microbiota abundance (genus Oscillospira id.2064) || id:ebi-a-GCST90017037 | CD19 on CD20- CD38- B cell || id:ebi-a-GCST90001722 | rs28889936 | 4 | 89483300 | A | 0.114051 | 0.0252838 | 3.37E-06 |
| ebi-a-GCST90017037 | Gut microbiota abundance (genus Oscillospira id.2064) || id:ebi-a-GCST90017037 | CD25 on B cell || id:ebi-a-GCST90001775 | rs28889936 | 4 | 89483300 | A | 0.114051 | 0.0252838 | 3.37E-06 |
| ebi-a-GCST90017037 | Gut microbiota abundance (genus Oscillospira id.2064) || id:ebi-a-GCST90017037 | CD25 on IgD+ CD24- B cell || id:ebi-a-GCST90001779 | rs28889936 | 4 | 89483300 | A | 0.114051 | 0.0252838 | 3.37E-06 |
| ebi-a-GCST90017037 | Gut microbiota abundance (genus Oscillospira id.2064) || id:ebi-a-GCST90017037 | CD25 on IgD+ CD38- naive B cell || id:ebi-a-GCST90001781 | rs28889936 | 4 | 89483300 | A | 0.114051 | 0.0252838 | 3.37E-06 |
| ebi-a-GCST90017037 | Gut microbiota abundance (genus Oscillospira id.2064) || id:ebi-a-GCST90017037 | CD25 on IgD+ CD38+ B cell || id:ebi-a-GCST90001783 | rs28889936 | 4 | 89483300 | A | 0.114051 | 0.0252838 | 3.37E-06 |
| ebi-a-GCST90017037 | Gut microbiota abundance (genus Oscillospira id.2064) || id:ebi-a-GCST90017037 | CD86 on myeloid Dendritic Cell || id:ebi-a-GCST90001903 | rs28889936 | 4 | 89483300 | A | 0.114051 | 0.0252838 | 3.37E-06 |
| ebi-a-GCST90017037 | Gut microbiota abundance (genus Oscillospira id.2064) || id:ebi-a-GCST90017037 | CD33 on CD14+ monocyte || id:ebi-a-GCST90001946 | rs28889936 | 4 | 89483300 | A | 0.114051 | 0.0252838 | 3.37E-06 |
| ebi-a-GCST90017037 | Gut microbiota abundance (genus Oscillospira id.2064) || id:ebi-a-GCST90017037 | CD33 on CD33+ HLA DR+ CD14dim || id:ebi-a-GCST90001947 | rs28889936 | 4 | 89483300 | A | 0.114051 | 0.0252838 | 3.37E-06 |
| ebi-a-GCST90017037 | Gut microbiota abundance (genus Oscillospira id.2064) || id:ebi-a-GCST90017037 | CD33 on CD33dim HLA DR+ CD11b+ || id:ebi-a-GCST90001948 | rs28889936 | 4 | 89483300 | A | 0.114051 | 0.0252838 | 3.37E-06 |
| ebi-a-GCST90017037 | Gut microbiota abundance (genus Oscillospira id.2064) || id:ebi-a-GCST90017037 | CD33 on Granulocytic Myeloid-Derived Suppressor Cells || id:ebi-a-GCST90001950 | rs28889936 | 4 | 89483300 | A | 0.114051 | 0.0252838 | 3.37E-06 |
| ebi-a-GCST90017037 | Gut microbiota abundance (genus Oscillospira id.2064) || id:ebi-a-GCST90017037 | CD33 on CD66b++ myeloid cell || id:ebi-a-GCST90001951 | rs28889936 | 4 | 89483300 | A | 0.114051 | 0.0252838 | 3.37E-06 |
| ebi-a-GCST90017037 | Gut microbiota abundance (genus Oscillospira id.2064) || id:ebi-a-GCST90017037 | CD33 on CD33dim HLA DR- || id:ebi-a-GCST90001953 | rs28889936 | 4 | 89483300 | A | 0.114051 | 0.0252838 | 3.37E-06 |
| ebi-a-GCST90017037 | Gut microbiota abundance (genus Oscillospira id.2064) || id:ebi-a-GCST90017037 | CD33 on basophil || id:ebi-a-GCST90001954 | rs28889936 | 4 | 89483300 | A | 0.114051 | 0.0252838 | 3.37E-06 |
| ebi-a-GCST90017037 | Gut microbiota abundance (genus Oscillospira id.2064) || id:ebi-a-GCST90017037 | CD33 on Immature Myeloid-Derived Suppressor Cells || id:ebi-a-GCST90001955 | rs28889936 | 4 | 89483300 | A | 0.114051 | 0.0252838 | 3.37E-06 |
| ebi-a-GCST90017037 | Gut microbiota abundance (genus Oscillospira id.2064) || id:ebi-a-GCST90017037 | FSC-A on HLA DR+ CD8+ T cell || id:ebi-a-GCST90001978 | rs28889936 | 4 | 89483300 | A | 0.114051 | 0.0252838 | 3.37E-06 |
| ebi-a-GCST90017037 | Gut microbiota abundance (genus Oscillospira id.2064) || id:ebi-a-GCST90017037 | HLA DR on CD14+ CD16- monocyte || id:ebi-a-GCST90001988 | rs28889936 | 4 | 89483300 | A | 0.114051 | 0.0252838 | 3.37E-06 |
| ebi-a-GCST90017037 | Gut microbiota abundance (genus Oscillospira id.2064) || id:ebi-a-GCST90017037 | HLA DR on CD14+ monocyte || id:ebi-a-GCST90001991 | rs28889936 | 4 | 89483300 | A | 0.114051 | 0.0252838 | 3.37E-06 |
| ebi-a-GCST90017037 | Gut microbiota abundance (genus Oscillospira id.2064) || id:ebi-a-GCST90017037 | CD16 on CD14+ CD16+ monocyte || id:ebi-a-GCST90002005 | rs28889936 | 4 | 89483300 | A | 0.114051 | 0.0252838 | 3.37E-06 |
| ebi-a-GCST90017037 | Gut microbiota abundance (genus Oscillospira id.2064) || id:ebi-a-GCST90017037 | CD45 on CD33+ HLA DR+ CD14- || id:ebi-a-GCST90002042 | rs28889936 | 4 | 89483300 | A | 0.114051 | 0.0252838 | 3.37E-06 |
| ebi-a-GCST90017037 | Gut microbiota abundance (genus Oscillospira id.2064) || id:ebi-a-GCST90017037 | CD8 on Natural Killer T || id:ebi-a-GCST90002059 | rs28889936 | 4 | 89483300 | A | 0.114051 | 0.0252838 | 3.37E-06 |
| ebi-a-GCST90016921 | Gut microbiota abundance (class Mollicutes id.3920) || id:ebi-a-GCST90016921 | Myeloid Dendritic Cell Absolute Count || id:ebi-a-GCST90001458 | rs3768491 | 1 | 109965986 | G | 0.0681052 | 0.0149061 | 4.23E-06 |
| ebi-a-GCST90016921 | Gut microbiota abundance (class Mollicutes id.3920) || id:ebi-a-GCST90016921 | CD86+ myeloid Dendritic Cell %Dendritic Cell || id:ebi-a-GCST90001465 | rs3768491 | 1 | 109965986 | G | 0.0681052 | 0.0149061 | 4.23E-06 |
| ebi-a-GCST90016921 | Gut microbiota abundance (class Mollicutes id.3920) || id:ebi-a-GCST90016921 | CD62L- myeloid Dendritic Cell Absolute Count || id:ebi-a-GCST90001468 | rs3768491 | 1 | 109965986 | G | 0.0681052 | 0.0149061 | 4.23E-06 |
| ebi-a-GCST90016921 | Gut microbiota abundance (class Mollicutes id.3920) || id:ebi-a-GCST90016921 | CD62L- myeloid Dendritic Cell %Dendritic Cell || id:ebi-a-GCST90001469 | rs3768491 | 1 | 109965986 | G | 0.0681052 | 0.0149061 | 4.23E-06 |
| ebi-a-GCST90016921 | Gut microbiota abundance (class Mollicutes id.3920) || id:ebi-a-GCST90016921 | CD62L- CD86+ myeloid Dendritic Cell Absolute Count || id:ebi-a-GCST90001472 | rs3768491 | 1 | 109965986 | G | 0.0681052 | 0.0149061 | 4.23E-06 |
| ebi-a-GCST90016921 | Gut microbiota abundance (class Mollicutes id.3920) || id:ebi-a-GCST90016921 | CD62L- CD86+ myeloid Dendritic Cell %Dendritic Cell || id:ebi-a-GCST90001473 | rs3768491 | 1 | 109965986 | G | 0.0681052 | 0.0149061 | 4.23E-06 |
| ebi-a-GCST90016921 | Gut microbiota abundance (class Mollicutes id.3920) || id:ebi-a-GCST90016921 | HLA DR++ monocyte %monocyte || id:ebi-a-GCST90001475 | rs3768491 | 1 | 109965986 | G | 0.0681052 | 0.0149061 | 4.23E-06 |
| ebi-a-GCST90016921 | Gut microbiota abundance (class Mollicutes id.3920) || id:ebi-a-GCST90016921 | HLA DR++ monocyte Absolute Count || id:ebi-a-GCST90001477 | rs3768491 | 1 | 109965986 | G | 0.0681052 | 0.0149061 | 4.23E-06 |
| ebi-a-GCST90016921 | Gut microbiota abundance (class Mollicutes id.3920) || id:ebi-a-GCST90016921 | Basophil %CD33dim HLA DR- CD66b- || id:ebi-a-GCST90001533 | rs3768491 | 1 | 109965986 | G | 0.0681052 | 0.0149061 | 4.23E-06 |
| ebi-a-GCST90016921 | Gut microbiota abundance (class Mollicutes id.3920) || id:ebi-a-GCST90016921 | CD16+ monocyte %monocyte || id:ebi-a-GCST90001587 | rs3768491 | 1 | 109965986 | G | 0.0681052 | 0.0149061 | 4.23E-06 |
| ebi-a-GCST90016921 | Gut microbiota abundance (class Mollicutes id.3920) || id:ebi-a-GCST90016921 | T/B cell || id:ebi-a-GCST90001588 | rs3768491 | 1 | 109965986 | G | 0.0681052 | 0.0149061 | 4.23E-06 |
| ebi-a-GCST90016921 | Gut microbiota abundance (class Mollicutes id.3920) || id:ebi-a-GCST90016921 | CD4+CD8+ T cell %T cell || id:ebi-a-GCST90001595 | rs3768491 | 1 | 109965986 | G | 0.0681052 | 0.0149061 | 4.23E-06 |
| ebi-a-GCST90016921 | Gut microbiota abundance (class Mollicutes id.3920) || id:ebi-a-GCST90016921 | CD8+ Natural Killer T %T cell || id:ebi-a-GCST90001631 | rs3768491 | 1 | 109965986 | G | 0.0681052 | 0.0149061 | 4.23E-06 |
| ebi-a-GCST90016921 | Gut microbiota abundance (class Mollicutes id.3920) || id:ebi-a-GCST90016921 | CD4-CD8- Natural Killer T %lymphocyte || id:ebi-a-GCST90001638 | rs3768491 | 1 | 109965986 | G | 0.0681052 | 0.0149061 | 4.23E-06 |
| ebi-a-GCST90016921 | Gut microbiota abundance (class Mollicutes id.3920) || id:ebi-a-GCST90016921 | HLA DR+ Natural Killer Absolute Count || id:ebi-a-GCST90001648 | rs3768491 | 1 | 109965986 | G | 0.0681052 | 0.0149061 | 4.23E-06 |
| ebi-a-GCST90016921 | Gut microbiota abundance (class Mollicutes id.3920) || id:ebi-a-GCST90016921 | HLA DR+ Natural Killer %Natural Killer || id:ebi-a-GCST90001649 | rs3768491 | 1 | 109965986 | G | 0.0681052 | 0.0149061 | 4.23E-06 |
| ebi-a-GCST90016921 | Gut microbiota abundance (class Mollicutes id.3920) || id:ebi-a-GCST90016921 | HLA DR+ Natural Killer %CD3- lymphocyte || id:ebi-a-GCST90001650 | rs3768491 | 1 | 109965986 | G | 0.0681052 | 0.0149061 | 4.23E-06 |
| ebi-a-GCST90016921 | Gut microbiota abundance (class Mollicutes id.3920) || id:ebi-a-GCST90016921 | CD28- CD25++ CD8+ T cell Absolute Count || id:ebi-a-GCST90001678 | rs3768491 | 1 | 109965986 | G | 0.0681052 | 0.0149061 | 4.23E-06 |
| ebi-a-GCST90016921 | Gut microbiota abundance (class Mollicutes id.3920) || id:ebi-a-GCST90016921 | CD25++ CD8+ T cell %T cell || id:ebi-a-GCST90001679 | rs3768491 | 1 | 109965986 | G | 0.0681052 | 0.0149061 | 4.23E-06 |
| ebi-a-GCST90016921 | Gut microbiota abundance (class Mollicutes id.3920) || id:ebi-a-GCST90016921 | CD19 on CD20- CD38- B cell || id:ebi-a-GCST90001722 | rs3768491 | 1 | 109965986 | G | 0.0681052 | 0.0149061 | 4.23E-06 |
| ebi-a-GCST90016921 | Gut microbiota abundance (class Mollicutes id.3920) || id:ebi-a-GCST90016921 | CD25 on B cell || id:ebi-a-GCST90001775 | rs3768491 | 1 | 109965986 | G | 0.0681052 | 0.0149061 | 4.23E-06 |
| ebi-a-GCST90016921 | Gut microbiota abundance (class Mollicutes id.3920) || id:ebi-a-GCST90016921 | CD25 on IgD+ CD24- B cell || id:ebi-a-GCST90001779 | rs3768491 | 1 | 109965986 | G | 0.0681052 | 0.0149061 | 4.23E-06 |
| ebi-a-GCST90016921 | Gut microbiota abundance (class Mollicutes id.3920) || id:ebi-a-GCST90016921 | CD25 on IgD+ CD38- naive B cell || id:ebi-a-GCST90001781 | rs3768491 | 1 | 109965986 | G | 0.0681052 | 0.0149061 | 4.23E-06 |
| ebi-a-GCST90016921 | Gut microbiota abundance (class Mollicutes id.3920) || id:ebi-a-GCST90016921 | CD25 on IgD+ CD38+ B cell || id:ebi-a-GCST90001783 | rs3768491 | 1 | 109965986 | G | 0.0681052 | 0.0149061 | 4.23E-06 |
| ebi-a-GCST90016921 | Gut microbiota abundance (class Mollicutes id.3920) || id:ebi-a-GCST90016921 | CD86 on myeloid Dendritic Cell || id:ebi-a-GCST90001903 | rs3768491 | 1 | 109965986 | G | 0.0681052 | 0.0149061 | 4.23E-06 |
| ebi-a-GCST90016921 | Gut microbiota abundance (class Mollicutes id.3920) || id:ebi-a-GCST90016921 | CD33 on CD14+ monocyte || id:ebi-a-GCST90001946 | rs3768491 | 1 | 109965986 | G | 0.0681052 | 0.0149061 | 4.23E-06 |
| ebi-a-GCST90016921 | Gut microbiota abundance (class Mollicutes id.3920) || id:ebi-a-GCST90016921 | CD33 on CD33+ HLA DR+ CD14dim || id:ebi-a-GCST90001947 | rs3768491 | 1 | 109965986 | G | 0.0681052 | 0.0149061 | 4.23E-06 |
| ebi-a-GCST90016921 | Gut microbiota abundance (class Mollicutes id.3920) || id:ebi-a-GCST90016921 | CD33 on CD33dim HLA DR+ CD11b+ || id:ebi-a-GCST90001948 | rs3768491 | 1 | 109965986 | G | 0.0681052 | 0.0149061 | 4.23E-06 |
| ebi-a-GCST90016921 | Gut microbiota abundance (class Mollicutes id.3920) || id:ebi-a-GCST90016921 | CD33 on Granulocytic Myeloid-Derived Suppressor Cells || id:ebi-a-GCST90001950 | rs3768491 | 1 | 109965986 | G | 0.0681052 | 0.0149061 | 4.23E-06 |
| ebi-a-GCST90016921 | Gut microbiota abundance (class Mollicutes id.3920) || id:ebi-a-GCST90016921 | CD33 on CD66b++ myeloid cell || id:ebi-a-GCST90001951 | rs3768491 | 1 | 109965986 | G | 0.0681052 | 0.0149061 | 4.23E-06 |
| ebi-a-GCST90016921 | Gut microbiota abundance (class Mollicutes id.3920) || id:ebi-a-GCST90016921 | CD33 on CD33dim HLA DR- || id:ebi-a-GCST90001953 | rs3768491 | 1 | 109965986 | G | 0.0681052 | 0.0149061 | 4.23E-06 |
| ebi-a-GCST90016921 | Gut microbiota abundance (class Mollicutes id.3920) || id:ebi-a-GCST90016921 | CD33 on basophil || id:ebi-a-GCST90001954 | rs3768491 | 1 | 109965986 | G | 0.0681052 | 0.0149061 | 4.23E-06 |
| ebi-a-GCST90016921 | Gut microbiota abundance (class Mollicutes id.3920) || id:ebi-a-GCST90016921 | CD33 on Immature Myeloid-Derived Suppressor Cells || id:ebi-a-GCST90001955 | rs3768491 | 1 | 109965986 | G | 0.0681052 | 0.0149061 | 4.23E-06 |
| ebi-a-GCST90016921 | Gut microbiota abundance (class Mollicutes id.3920) || id:ebi-a-GCST90016921 | FSC-A on HLA DR+ CD8+ T cell || id:ebi-a-GCST90001978 | rs3768491 | 1 | 109965986 | G | 0.0681052 | 0.0149061 | 4.23E-06 |
| ebi-a-GCST90016921 | Gut microbiota abundance (class Mollicutes id.3920) || id:ebi-a-GCST90016921 | HLA DR on CD14+ CD16- monocyte || id:ebi-a-GCST90001988 | rs3768491 | 1 | 109965986 | G | 0.0681052 | 0.0149061 | 4.23E-06 |
| ebi-a-GCST90016921 | Gut microbiota abundance (class Mollicutes id.3920) || id:ebi-a-GCST90016921 | HLA DR on CD14+ monocyte || id:ebi-a-GCST90001991 | rs3768491 | 1 | 109965986 | G | 0.0681052 | 0.0149061 | 4.23E-06 |
| ebi-a-GCST90016921 | Gut microbiota abundance (class Mollicutes id.3920) || id:ebi-a-GCST90016921 | CD16 on CD14+ CD16+ monocyte || id:ebi-a-GCST90002005 | rs3768491 | 1 | 109965986 | G | 0.0681052 | 0.0149061 | 4.23E-06 |
| ebi-a-GCST90016921 | Gut microbiota abundance (class Mollicutes id.3920) || id:ebi-a-GCST90016921 | CD45 on CD33+ HLA DR+ CD14- || id:ebi-a-GCST90002042 | rs3768491 | 1 | 109965986 | G | 0.0681052 | 0.0149061 | 4.23E-06 |
| ebi-a-GCST90016921 | Gut microbiota abundance (class Mollicutes id.3920) || id:ebi-a-GCST90016921 | CD8 on Natural Killer T || id:ebi-a-GCST90002059 | rs3768491 | 1 | 109965986 | G | 0.0681052 | 0.0149061 | 4.23E-06 |
| ebi-a-GCST90017117 | Gut microbiota abundance (phylum Tenericutes id.3919) || id:ebi-a-GCST90017117 | Myeloid Dendritic Cell Absolute Count || id:ebi-a-GCST90001458 | rs3768491 | 1 | 109965986 | G | 0.0681052 | 0.0149061 | 4.23E-06 |
| ebi-a-GCST90017117 | Gut microbiota abundance (phylum Tenericutes id.3919) || id:ebi-a-GCST90017117 | CD86+ myeloid Dendritic Cell %Dendritic Cell || id:ebi-a-GCST90001465 | rs3768491 | 1 | 109965986 | G | 0.0681052 | 0.0149061 | 4.23E-06 |
| ebi-a-GCST90017117 | Gut microbiota abundance (phylum Tenericutes id.3919) || id:ebi-a-GCST90017117 | CD62L- myeloid Dendritic Cell Absolute Count || id:ebi-a-GCST90001468 | rs3768491 | 1 | 109965986 | G | 0.0681052 | 0.0149061 | 4.23E-06 |
| ebi-a-GCST90017117 | Gut microbiota abundance (phylum Tenericutes id.3919) || id:ebi-a-GCST90017117 | CD62L- myeloid Dendritic Cell %Dendritic Cell || id:ebi-a-GCST90001469 | rs3768491 | 1 | 109965986 | G | 0.0681052 | 0.0149061 | 4.23E-06 |
| ebi-a-GCST90017117 | Gut microbiota abundance (phylum Tenericutes id.3919) || id:ebi-a-GCST90017117 | CD62L- CD86+ myeloid Dendritic Cell Absolute Count || id:ebi-a-GCST90001472 | rs3768491 | 1 | 109965986 | G | 0.0681052 | 0.0149061 | 4.23E-06 |
| ebi-a-GCST90017117 | Gut microbiota abundance (phylum Tenericutes id.3919) || id:ebi-a-GCST90017117 | CD62L- CD86+ myeloid Dendritic Cell %Dendritic Cell || id:ebi-a-GCST90001473 | rs3768491 | 1 | 109965986 | G | 0.0681052 | 0.0149061 | 4.23E-06 |
| ebi-a-GCST90017117 | Gut microbiota abundance (phylum Tenericutes id.3919) || id:ebi-a-GCST90017117 | HLA DR++ monocyte %monocyte || id:ebi-a-GCST90001475 | rs3768491 | 1 | 109965986 | G | 0.0681052 | 0.0149061 | 4.23E-06 |
| ebi-a-GCST90017117 | Gut microbiota abundance (phylum Tenericutes id.3919) || id:ebi-a-GCST90017117 | HLA DR++ monocyte Absolute Count || id:ebi-a-GCST90001477 | rs3768491 | 1 | 109965986 | G | 0.0681052 | 0.0149061 | 4.23E-06 |
| ebi-a-GCST90017117 | Gut microbiota abundance (phylum Tenericutes id.3919) || id:ebi-a-GCST90017117 | Basophil %CD33dim HLA DR- CD66b- || id:ebi-a-GCST90001533 | rs3768491 | 1 | 109965986 | G | 0.0681052 | 0.0149061 | 4.23E-06 |
| ebi-a-GCST90017117 | Gut microbiota abundance (phylum Tenericutes id.3919) || id:ebi-a-GCST90017117 | CD16+ monocyte %monocyte || id:ebi-a-GCST90001587 | rs3768491 | 1 | 109965986 | G | 0.0681052 | 0.0149061 | 4.23E-06 |
| ebi-a-GCST90017117 | Gut microbiota abundance (phylum Tenericutes id.3919) || id:ebi-a-GCST90017117 | T/B cell || id:ebi-a-GCST90001588 | rs3768491 | 1 | 109965986 | G | 0.0681052 | 0.0149061 | 4.23E-06 |
| ebi-a-GCST90017117 | Gut microbiota abundance (phylum Tenericutes id.3919) || id:ebi-a-GCST90017117 | CD4+CD8+ T cell %T cell || id:ebi-a-GCST90001595 | rs3768491 | 1 | 109965986 | G | 0.0681052 | 0.0149061 | 4.23E-06 |
| ebi-a-GCST90017117 | Gut microbiota abundance (phylum Tenericutes id.3919) || id:ebi-a-GCST90017117 | CD8+ Natural Killer T %T cell || id:ebi-a-GCST90001631 | rs3768491 | 1 | 109965986 | G | 0.0681052 | 0.0149061 | 4.23E-06 |
| ebi-a-GCST90017117 | Gut microbiota abundance (phylum Tenericutes id.3919) || id:ebi-a-GCST90017117 | CD4-CD8- Natural Killer T %lymphocyte || id:ebi-a-GCST90001638 | rs3768491 | 1 | 109965986 | G | 0.0681052 | 0.0149061 | 4.23E-06 |
| ebi-a-GCST90017117 | Gut microbiota abundance (phylum Tenericutes id.3919) || id:ebi-a-GCST90017117 | HLA DR+ Natural Killer Absolute Count || id:ebi-a-GCST90001648 | rs3768491 | 1 | 109965986 | G | 0.0681052 | 0.0149061 | 4.23E-06 |
| ebi-a-GCST90017117 | Gut microbiota abundance (phylum Tenericutes id.3919) || id:ebi-a-GCST90017117 | HLA DR+ Natural Killer %Natural Killer || id:ebi-a-GCST90001649 | rs3768491 | 1 | 109965986 | G | 0.0681052 | 0.0149061 | 4.23E-06 |
| ebi-a-GCST90017117 | Gut microbiota abundance (phylum Tenericutes id.3919) || id:ebi-a-GCST90017117 | HLA DR+ Natural Killer %CD3- lymphocyte || id:ebi-a-GCST90001650 | rs3768491 | 1 | 109965986 | G | 0.0681052 | 0.0149061 | 4.23E-06 |
| ebi-a-GCST90017117 | Gut microbiota abundance (phylum Tenericutes id.3919) || id:ebi-a-GCST90017117 | CD28- CD25++ CD8+ T cell Absolute Count || id:ebi-a-GCST90001678 | rs3768491 | 1 | 109965986 | G | 0.0681052 | 0.0149061 | 4.23E-06 |
| ebi-a-GCST90017117 | Gut microbiota abundance (phylum Tenericutes id.3919) || id:ebi-a-GCST90017117 | CD25++ CD8+ T cell %T cell || id:ebi-a-GCST90001679 | rs3768491 | 1 | 109965986 | G | 0.0681052 | 0.0149061 | 4.23E-06 |
| ebi-a-GCST90017117 | Gut microbiota abundance (phylum Tenericutes id.3919) || id:ebi-a-GCST90017117 | CD19 on CD20- CD38- B cell || id:ebi-a-GCST90001722 | rs3768491 | 1 | 109965986 | G | 0.0681052 | 0.0149061 | 4.23E-06 |
| ebi-a-GCST90017117 | Gut microbiota abundance (phylum Tenericutes id.3919) || id:ebi-a-GCST90017117 | CD25 on B cell || id:ebi-a-GCST90001775 | rs3768491 | 1 | 109965986 | G | 0.0681052 | 0.0149061 | 4.23E-06 |
| ebi-a-GCST90017117 | Gut microbiota abundance (phylum Tenericutes id.3919) || id:ebi-a-GCST90017117 | CD25 on IgD+ CD24- B cell || id:ebi-a-GCST90001779 | rs3768491 | 1 | 109965986 | G | 0.0681052 | 0.0149061 | 4.23E-06 |
| ebi-a-GCST90017117 | Gut microbiota abundance (phylum Tenericutes id.3919) || id:ebi-a-GCST90017117 | CD25 on IgD+ CD38- naive B cell || id:ebi-a-GCST90001781 | rs3768491 | 1 | 109965986 | G | 0.0681052 | 0.0149061 | 4.23E-06 |
| ebi-a-GCST90017117 | Gut microbiota abundance (phylum Tenericutes id.3919) || id:ebi-a-GCST90017117 | CD25 on IgD+ CD38+ B cell || id:ebi-a-GCST90001783 | rs3768491 | 1 | 109965986 | G | 0.0681052 | 0.0149061 | 4.23E-06 |
| ebi-a-GCST90017117 | Gut microbiota abundance (phylum Tenericutes id.3919) || id:ebi-a-GCST90017117 | CD86 on myeloid Dendritic Cell || id:ebi-a-GCST90001903 | rs3768491 | 1 | 109965986 | G | 0.0681052 | 0.0149061 | 4.23E-06 |
| ebi-a-GCST90017117 | Gut microbiota abundance (phylum Tenericutes id.3919) || id:ebi-a-GCST90017117 | CD33 on CD14+ monocyte || id:ebi-a-GCST90001946 | rs3768491 | 1 | 109965986 | G | 0.0681052 | 0.0149061 | 4.23E-06 |
| ebi-a-GCST90017117 | Gut microbiota abundance (phylum Tenericutes id.3919) || id:ebi-a-GCST90017117 | CD33 on CD33+ HLA DR+ CD14dim || id:ebi-a-GCST90001947 | rs3768491 | 1 | 109965986 | G | 0.0681052 | 0.0149061 | 4.23E-06 |
| ebi-a-GCST90017117 | Gut microbiota abundance (phylum Tenericutes id.3919) || id:ebi-a-GCST90017117 | CD33 on CD33dim HLA DR+ CD11b+ || id:ebi-a-GCST90001948 | rs3768491 | 1 | 109965986 | G | 0.0681052 | 0.0149061 | 4.23E-06 |
| ebi-a-GCST90017117 | Gut microbiota abundance (phylum Tenericutes id.3919) || id:ebi-a-GCST90017117 | CD33 on Granulocytic Myeloid-Derived Suppressor Cells || id:ebi-a-GCST90001950 | rs3768491 | 1 | 109965986 | G | 0.0681052 | 0.0149061 | 4.23E-06 |
| ebi-a-GCST90017117 | Gut microbiota abundance (phylum Tenericutes id.3919) || id:ebi-a-GCST90017117 | CD33 on CD66b++ myeloid cell || id:ebi-a-GCST90001951 | rs3768491 | 1 | 109965986 | G | 0.0681052 | 0.0149061 | 4.23E-06 |
| ebi-a-GCST90017117 | Gut microbiota abundance (phylum Tenericutes id.3919) || id:ebi-a-GCST90017117 | CD33 on CD33dim HLA DR- || id:ebi-a-GCST90001953 | rs3768491 | 1 | 109965986 | G | 0.0681052 | 0.0149061 | 4.23E-06 |
| ebi-a-GCST90017117 | Gut microbiota abundance (phylum Tenericutes id.3919) || id:ebi-a-GCST90017117 | CD33 on basophil || id:ebi-a-GCST90001954 | rs3768491 | 1 | 109965986 | G | 0.0681052 | 0.0149061 | 4.23E-06 |
| ebi-a-GCST90017117 | Gut microbiota abundance (phylum Tenericutes id.3919) || id:ebi-a-GCST90017117 | CD33 on Immature Myeloid-Derived Suppressor Cells || id:ebi-a-GCST90001955 | rs3768491 | 1 | 109965986 | G | 0.0681052 | 0.0149061 | 4.23E-06 |
| ebi-a-GCST90017117 | Gut microbiota abundance (phylum Tenericutes id.3919) || id:ebi-a-GCST90017117 | FSC-A on HLA DR+ CD8+ T cell || id:ebi-a-GCST90001978 | rs3768491 | 1 | 109965986 | G | 0.0681052 | 0.0149061 | 4.23E-06 |
| ebi-a-GCST90017117 | Gut microbiota abundance (phylum Tenericutes id.3919) || id:ebi-a-GCST90017117 | HLA DR on CD14+ CD16- monocyte || id:ebi-a-GCST90001988 | rs3768491 | 1 | 109965986 | G | 0.0681052 | 0.0149061 | 4.23E-06 |
| ebi-a-GCST90017117 | Gut microbiota abundance (phylum Tenericutes id.3919) || id:ebi-a-GCST90017117 | HLA DR on CD14+ monocyte || id:ebi-a-GCST90001991 | rs3768491 | 1 | 109965986 | G | 0.0681052 | 0.0149061 | 4.23E-06 |
| ebi-a-GCST90017117 | Gut microbiota abundance (phylum Tenericutes id.3919) || id:ebi-a-GCST90017117 | CD16 on CD14+ CD16+ monocyte || id:ebi-a-GCST90002005 | rs3768491 | 1 | 109965986 | G | 0.0681052 | 0.0149061 | 4.23E-06 |
| ebi-a-GCST90017117 | Gut microbiota abundance (phylum Tenericutes id.3919) || id:ebi-a-GCST90017117 | CD45 on CD33+ HLA DR+ CD14- || id:ebi-a-GCST90002042 | rs3768491 | 1 | 109965986 | G | 0.0681052 | 0.0149061 | 4.23E-06 |
| ebi-a-GCST90017117 | Gut microbiota abundance (phylum Tenericutes id.3919) || id:ebi-a-GCST90017117 | CD8 on Natural Killer T || id:ebi-a-GCST90002059 | rs3768491 | 1 | 109965986 | G | 0.0681052 | 0.0149061 | 4.23E-06 |
| ebi-a-GCST90016957 | Gut microbiota abundance (family Verrucomicrobiaceae id.4036) || id:ebi-a-GCST90016957 | Myeloid Dendritic Cell Absolute Count || id:ebi-a-GCST90001458 | rs4242783 | 10 | 5064327 | A | -0.0687992 | 0.0147694 | 2.75E-06 |
| ebi-a-GCST90016957 | Gut microbiota abundance (family Verrucomicrobiaceae id.4036) || id:ebi-a-GCST90016957 | CD86+ myeloid Dendritic Cell %Dendritic Cell || id:ebi-a-GCST90001465 | rs4242783 | 10 | 5064327 | A | -0.0687992 | 0.0147694 | 2.75E-06 |
| ebi-a-GCST90016957 | Gut microbiota abundance (family Verrucomicrobiaceae id.4036) || id:ebi-a-GCST90016957 | CD62L- myeloid Dendritic Cell Absolute Count || id:ebi-a-GCST90001468 | rs4242783 | 10 | 5064327 | A | -0.0687992 | 0.0147694 | 2.75E-06 |
| ebi-a-GCST90016957 | Gut microbiota abundance (family Verrucomicrobiaceae id.4036) || id:ebi-a-GCST90016957 | CD62L- myeloid Dendritic Cell %Dendritic Cell || id:ebi-a-GCST90001469 | rs4242783 | 10 | 5064327 | A | -0.0687992 | 0.0147694 | 2.75E-06 |
| ebi-a-GCST90016957 | Gut microbiota abundance (family Verrucomicrobiaceae id.4036) || id:ebi-a-GCST90016957 | CD62L- CD86+ myeloid Dendritic Cell Absolute Count || id:ebi-a-GCST90001472 | rs4242783 | 10 | 5064327 | A | -0.0687992 | 0.0147694 | 2.75E-06 |
| ebi-a-GCST90016957 | Gut microbiota abundance (family Verrucomicrobiaceae id.4036) || id:ebi-a-GCST90016957 | CD62L- CD86+ myeloid Dendritic Cell %Dendritic Cell || id:ebi-a-GCST90001473 | rs4242783 | 10 | 5064327 | A | -0.0687992 | 0.0147694 | 2.75E-06 |
| ebi-a-GCST90016957 | Gut microbiota abundance (family Verrucomicrobiaceae id.4036) || id:ebi-a-GCST90016957 | HLA DR++ monocyte %monocyte || id:ebi-a-GCST90001475 | rs4242783 | 10 | 5064327 | A | -0.0687992 | 0.0147694 | 2.75E-06 |
| ebi-a-GCST90016957 | Gut microbiota abundance (family Verrucomicrobiaceae id.4036) || id:ebi-a-GCST90016957 | HLA DR++ monocyte Absolute Count || id:ebi-a-GCST90001477 | rs4242783 | 10 | 5064327 | A | -0.0687992 | 0.0147694 | 2.75E-06 |
| ebi-a-GCST90016957 | Gut microbiota abundance (family Verrucomicrobiaceae id.4036) || id:ebi-a-GCST90016957 | Basophil %CD33dim HLA DR- CD66b- || id:ebi-a-GCST90001533 | rs4242783 | 10 | 5064327 | A | -0.0687992 | 0.0147694 | 2.75E-06 |
| ebi-a-GCST90016957 | Gut microbiota abundance (family Verrucomicrobiaceae id.4036) || id:ebi-a-GCST90016957 | CD16+ monocyte %monocyte || id:ebi-a-GCST90001587 | rs4242783 | 10 | 5064327 | A | -0.0687992 | 0.0147694 | 2.75E-06 |
| ebi-a-GCST90016957 | Gut microbiota abundance (family Verrucomicrobiaceae id.4036) || id:ebi-a-GCST90016957 | T/B cell || id:ebi-a-GCST90001588 | rs4242783 | 10 | 5064327 | A | -0.0687992 | 0.0147694 | 2.75E-06 |
| ebi-a-GCST90016957 | Gut microbiota abundance (family Verrucomicrobiaceae id.4036) || id:ebi-a-GCST90016957 | CD8+ Natural Killer T %T cell || id:ebi-a-GCST90001631 | rs4242783 | 10 | 5064327 | A | -0.0687992 | 0.0147694 | 2.75E-06 |
| ebi-a-GCST90016957 | Gut microbiota abundance (family Verrucomicrobiaceae id.4036) || id:ebi-a-GCST90016957 | CD4-CD8- Natural Killer T %lymphocyte || id:ebi-a-GCST90001638 | rs4242783 | 10 | 5064327 | A | -0.0687992 | 0.0147694 | 2.75E-06 |
| ebi-a-GCST90016957 | Gut microbiota abundance (family Verrucomicrobiaceae id.4036) || id:ebi-a-GCST90016957 | HLA DR+ Natural Killer Absolute Count || id:ebi-a-GCST90001648 | rs4242783 | 10 | 5064327 | A | -0.0687992 | 0.0147694 | 2.75E-06 |
| ebi-a-GCST90016957 | Gut microbiota abundance (family Verrucomicrobiaceae id.4036) || id:ebi-a-GCST90016957 | HLA DR+ Natural Killer %Natural Killer || id:ebi-a-GCST90001649 | rs4242783 | 10 | 5064327 | A | -0.0687992 | 0.0147694 | 2.75E-06 |
| ebi-a-GCST90016957 | Gut microbiota abundance (family Verrucomicrobiaceae id.4036) || id:ebi-a-GCST90016957 | HLA DR+ Natural Killer %CD3- lymphocyte || id:ebi-a-GCST90001650 | rs4242783 | 10 | 5064327 | A | -0.0687992 | 0.0147694 | 2.75E-06 |
| ebi-a-GCST90016957 | Gut microbiota abundance (family Verrucomicrobiaceae id.4036) || id:ebi-a-GCST90016957 | CD28- CD25++ CD8+ T cell Absolute Count || id:ebi-a-GCST90001678 | rs4242783 | 10 | 5064327 | A | -0.0687992 | 0.0147694 | 2.75E-06 |
| ebi-a-GCST90016957 | Gut microbiota abundance (family Verrucomicrobiaceae id.4036) || id:ebi-a-GCST90016957 | CD25++ CD8+ T cell %T cell || id:ebi-a-GCST90001679 | rs4242783 | 10 | 5064327 | A | -0.0687992 | 0.0147694 | 2.75E-06 |
| ebi-a-GCST90016957 | Gut microbiota abundance (family Verrucomicrobiaceae id.4036) || id:ebi-a-GCST90016957 | CD19 on CD20- CD38- B cell || id:ebi-a-GCST90001722 | rs4242783 | 10 | 5064327 | A | -0.0687992 | 0.0147694 | 2.75E-06 |
| ebi-a-GCST90016957 | Gut microbiota abundance (family Verrucomicrobiaceae id.4036) || id:ebi-a-GCST90016957 | CD25 on B cell || id:ebi-a-GCST90001775 | rs4242783 | 10 | 5064327 | A | -0.0687992 | 0.0147694 | 2.75E-06 |
| ebi-a-GCST90016957 | Gut microbiota abundance (family Verrucomicrobiaceae id.4036) || id:ebi-a-GCST90016957 | CD25 on IgD+ CD24- B cell || id:ebi-a-GCST90001779 | rs4242783 | 10 | 5064327 | A | -0.0687992 | 0.0147694 | 2.75E-06 |
| ebi-a-GCST90016957 | Gut microbiota abundance (family Verrucomicrobiaceae id.4036) || id:ebi-a-GCST90016957 | CD25 on IgD+ CD38- naive B cell || id:ebi-a-GCST90001781 | rs4242783 | 10 | 5064327 | A | -0.0687992 | 0.0147694 | 2.75E-06 |
| ebi-a-GCST90016957 | Gut microbiota abundance (family Verrucomicrobiaceae id.4036) || id:ebi-a-GCST90016957 | CD25 on IgD+ CD38+ B cell || id:ebi-a-GCST90001783 | rs4242783 | 10 | 5064327 | A | -0.0687992 | 0.0147694 | 2.75E-06 |
| ebi-a-GCST90016957 | Gut microbiota abundance (family Verrucomicrobiaceae id.4036) || id:ebi-a-GCST90016957 | CD86 on myeloid Dendritic Cell || id:ebi-a-GCST90001903 | rs4242783 | 10 | 5064327 | A | -0.0687992 | 0.0147694 | 2.75E-06 |
| ebi-a-GCST90016957 | Gut microbiota abundance (family Verrucomicrobiaceae id.4036) || id:ebi-a-GCST90016957 | CD33 on CD14+ monocyte || id:ebi-a-GCST90001946 | rs4242783 | 10 | 5064327 | A | -0.0687992 | 0.0147694 | 2.75E-06 |
| ebi-a-GCST90016957 | Gut microbiota abundance (family Verrucomicrobiaceae id.4036) || id:ebi-a-GCST90016957 | CD33 on CD33+ HLA DR+ CD14dim || id:ebi-a-GCST90001947 | rs4242783 | 10 | 5064327 | A | -0.0687992 | 0.0147694 | 2.75E-06 |
| ebi-a-GCST90016957 | Gut microbiota abundance (family Verrucomicrobiaceae id.4036) || id:ebi-a-GCST90016957 | CD33 on CD33dim HLA DR+ CD11b+ || id:ebi-a-GCST90001948 | rs4242783 | 10 | 5064327 | A | -0.0687992 | 0.0147694 | 2.75E-06 |
| ebi-a-GCST90016957 | Gut microbiota abundance (family Verrucomicrobiaceae id.4036) || id:ebi-a-GCST90016957 | CD33 on Granulocytic Myeloid-Derived Suppressor Cells || id:ebi-a-GCST90001950 | rs4242783 | 10 | 5064327 | A | -0.0687992 | 0.0147694 | 2.75E-06 |
| ebi-a-GCST90016957 | Gut microbiota abundance (family Verrucomicrobiaceae id.4036) || id:ebi-a-GCST90016957 | CD33 on CD66b++ myeloid cell || id:ebi-a-GCST90001951 | rs4242783 | 10 | 5064327 | A | -0.0687992 | 0.0147694 | 2.75E-06 |
| ebi-a-GCST90016957 | Gut microbiota abundance (family Verrucomicrobiaceae id.4036) || id:ebi-a-GCST90016957 | CD33 on CD33dim HLA DR- || id:ebi-a-GCST90001953 | rs4242783 | 10 | 5064327 | A | -0.0687992 | 0.0147694 | 2.75E-06 |
| ebi-a-GCST90016957 | Gut microbiota abundance (family Verrucomicrobiaceae id.4036) || id:ebi-a-GCST90016957 | CD33 on basophil || id:ebi-a-GCST90001954 | rs4242783 | 10 | 5064327 | A | -0.0687992 | 0.0147694 | 2.75E-06 |
| ebi-a-GCST90016957 | Gut microbiota abundance (family Verrucomicrobiaceae id.4036) || id:ebi-a-GCST90016957 | CD33 on Immature Myeloid-Derived Suppressor Cells || id:ebi-a-GCST90001955 | rs4242783 | 10 | 5064327 | A | -0.0687992 | 0.0147694 | 2.75E-06 |
| ebi-a-GCST90016957 | Gut microbiota abundance (family Verrucomicrobiaceae id.4036) || id:ebi-a-GCST90016957 | FSC-A on HLA DR+ CD8+ T cell || id:ebi-a-GCST90001978 | rs4242783 | 10 | 5064327 | A | -0.0687992 | 0.0147694 | 2.75E-06 |
| ebi-a-GCST90016957 | Gut microbiota abundance (family Verrucomicrobiaceae id.4036) || id:ebi-a-GCST90016957 | HLA DR on CD14+ CD16- monocyte || id:ebi-a-GCST90001988 | rs4242783 | 10 | 5064327 | A | -0.0687992 | 0.0147694 | 2.75E-06 |
| ebi-a-GCST90016957 | Gut microbiota abundance (family Verrucomicrobiaceae id.4036) || id:ebi-a-GCST90016957 | HLA DR on CD14+ monocyte || id:ebi-a-GCST90001991 | rs4242783 | 10 | 5064327 | A | -0.0687992 | 0.0147694 | 2.75E-06 |
| ebi-a-GCST90016957 | Gut microbiota abundance (family Verrucomicrobiaceae id.4036) || id:ebi-a-GCST90016957 | CD16 on CD14+ CD16+ monocyte || id:ebi-a-GCST90002005 | rs4242783 | 10 | 5064327 | A | -0.0687992 | 0.0147694 | 2.75E-06 |
| ebi-a-GCST90016957 | Gut microbiota abundance (family Verrucomicrobiaceae id.4036) || id:ebi-a-GCST90016957 | CD45 on CD33+ HLA DR+ CD14- || id:ebi-a-GCST90002042 | rs4242783 | 10 | 5064327 | A | -0.0687992 | 0.0147694 | 2.75E-06 |
| ebi-a-GCST90016957 | Gut microbiota abundance (family Verrucomicrobiaceae id.4036) || id:ebi-a-GCST90016957 | CD8 on Natural Killer T || id:ebi-a-GCST90002059 | rs4242783 | 10 | 5064327 | A | -0.0687992 | 0.0147694 | 2.75E-06 |
| ebi-a-GCST90016923 | Gut microbiota abundance (class Verrucomicrobiae id.4029) || id:ebi-a-GCST90016923 | Myeloid Dendritic Cell Absolute Count || id:ebi-a-GCST90001458 | rs4242783 | 10 | 5064327 | A | -0.0689292 | 0.0147693 | 2.64E-06 |
| ebi-a-GCST90016923 | Gut microbiota abundance (class Verrucomicrobiae id.4029) || id:ebi-a-GCST90016923 | CD86+ myeloid Dendritic Cell %Dendritic Cell || id:ebi-a-GCST90001465 | rs4242783 | 10 | 5064327 | A | -0.0689292 | 0.0147693 | 2.64E-06 |
| ebi-a-GCST90016923 | Gut microbiota abundance (class Verrucomicrobiae id.4029) || id:ebi-a-GCST90016923 | CD62L- myeloid Dendritic Cell Absolute Count || id:ebi-a-GCST90001468 | rs4242783 | 10 | 5064327 | A | -0.0689292 | 0.0147693 | 2.64E-06 |
| ebi-a-GCST90016923 | Gut microbiota abundance (class Verrucomicrobiae id.4029) || id:ebi-a-GCST90016923 | CD62L- myeloid Dendritic Cell %Dendritic Cell || id:ebi-a-GCST90001469 | rs4242783 | 10 | 5064327 | A | -0.0689292 | 0.0147693 | 2.64E-06 |
| ebi-a-GCST90016923 | Gut microbiota abundance (class Verrucomicrobiae id.4029) || id:ebi-a-GCST90016923 | CD62L- CD86+ myeloid Dendritic Cell Absolute Count || id:ebi-a-GCST90001472 | rs4242783 | 10 | 5064327 | A | -0.0689292 | 0.0147693 | 2.64E-06 |
| ebi-a-GCST90016923 | Gut microbiota abundance (class Verrucomicrobiae id.4029) || id:ebi-a-GCST90016923 | CD62L- CD86+ myeloid Dendritic Cell %Dendritic Cell || id:ebi-a-GCST90001473 | rs4242783 | 10 | 5064327 | A | -0.0689292 | 0.0147693 | 2.64E-06 |
| ebi-a-GCST90016923 | Gut microbiota abundance (class Verrucomicrobiae id.4029) || id:ebi-a-GCST90016923 | HLA DR++ monocyte %monocyte || id:ebi-a-GCST90001475 | rs4242783 | 10 | 5064327 | A | -0.0689292 | 0.0147693 | 2.64E-06 |
| ebi-a-GCST90016923 | Gut microbiota abundance (class Verrucomicrobiae id.4029) || id:ebi-a-GCST90016923 | HLA DR++ monocyte Absolute Count || id:ebi-a-GCST90001477 | rs4242783 | 10 | 5064327 | A | -0.0689292 | 0.0147693 | 2.64E-06 |
| ebi-a-GCST90016923 | Gut microbiota abundance (class Verrucomicrobiae id.4029) || id:ebi-a-GCST90016923 | Basophil %CD33dim HLA DR- CD66b- || id:ebi-a-GCST90001533 | rs4242783 | 10 | 5064327 | A | -0.0689292 | 0.0147693 | 2.64E-06 |
| ebi-a-GCST90016923 | Gut microbiota abundance (class Verrucomicrobiae id.4029) || id:ebi-a-GCST90016923 | CD16+ monocyte %monocyte || id:ebi-a-GCST90001587 | rs4242783 | 10 | 5064327 | A | -0.0689292 | 0.0147693 | 2.64E-06 |
| ebi-a-GCST90016923 | Gut microbiota abundance (class Verrucomicrobiae id.4029) || id:ebi-a-GCST90016923 | T/B cell || id:ebi-a-GCST90001588 | rs4242783 | 10 | 5064327 | A | -0.0689292 | 0.0147693 | 2.64E-06 |
| ebi-a-GCST90016923 | Gut microbiota abundance (class Verrucomicrobiae id.4029) || id:ebi-a-GCST90016923 | CD8+ Natural Killer T %T cell || id:ebi-a-GCST90001631 | rs4242783 | 10 | 5064327 | A | -0.0689292 | 0.0147693 | 2.64E-06 |
| ebi-a-GCST90016923 | Gut microbiota abundance (class Verrucomicrobiae id.4029) || id:ebi-a-GCST90016923 | CD4-CD8- Natural Killer T %lymphocyte || id:ebi-a-GCST90001638 | rs4242783 | 10 | 5064327 | A | -0.0689292 | 0.0147693 | 2.64E-06 |
| ebi-a-GCST90016923 | Gut microbiota abundance (class Verrucomicrobiae id.4029) || id:ebi-a-GCST90016923 | HLA DR+ Natural Killer Absolute Count || id:ebi-a-GCST90001648 | rs4242783 | 10 | 5064327 | A | -0.0689292 | 0.0147693 | 2.64E-06 |
| ebi-a-GCST90016923 | Gut microbiota abundance (class Verrucomicrobiae id.4029) || id:ebi-a-GCST90016923 | HLA DR+ Natural Killer %Natural Killer || id:ebi-a-GCST90001649 | rs4242783 | 10 | 5064327 | A | -0.0689292 | 0.0147693 | 2.64E-06 |
| ebi-a-GCST90016923 | Gut microbiota abundance (class Verrucomicrobiae id.4029) || id:ebi-a-GCST90016923 | HLA DR+ Natural Killer %CD3- lymphocyte || id:ebi-a-GCST90001650 | rs4242783 | 10 | 5064327 | A | -0.0689292 | 0.0147693 | 2.64E-06 |
| ebi-a-GCST90016923 | Gut microbiota abundance (class Verrucomicrobiae id.4029) || id:ebi-a-GCST90016923 | CD28- CD25++ CD8+ T cell Absolute Count || id:ebi-a-GCST90001678 | rs4242783 | 10 | 5064327 | A | -0.0689292 | 0.0147693 | 2.64E-06 |
| ebi-a-GCST90016923 | Gut microbiota abundance (class Verrucomicrobiae id.4029) || id:ebi-a-GCST90016923 | CD25++ CD8+ T cell %T cell || id:ebi-a-GCST90001679 | rs4242783 | 10 | 5064327 | A | -0.0689292 | 0.0147693 | 2.64E-06 |
| ebi-a-GCST90016923 | Gut microbiota abundance (class Verrucomicrobiae id.4029) || id:ebi-a-GCST90016923 | CD19 on CD20- CD38- B cell || id:ebi-a-GCST90001722 | rs4242783 | 10 | 5064327 | A | -0.0689292 | 0.0147693 | 2.64E-06 |
| ebi-a-GCST90016923 | Gut microbiota abundance (class Verrucomicrobiae id.4029) || id:ebi-a-GCST90016923 | CD25 on B cell || id:ebi-a-GCST90001775 | rs4242783 | 10 | 5064327 | A | -0.0689292 | 0.0147693 | 2.64E-06 |
| ebi-a-GCST90016923 | Gut microbiota abundance (class Verrucomicrobiae id.4029) || id:ebi-a-GCST90016923 | CD25 on IgD+ CD24- B cell || id:ebi-a-GCST90001779 | rs4242783 | 10 | 5064327 | A | -0.0689292 | 0.0147693 | 2.64E-06 |
| ebi-a-GCST90016923 | Gut microbiota abundance (class Verrucomicrobiae id.4029) || id:ebi-a-GCST90016923 | CD25 on IgD+ CD38- naive B cell || id:ebi-a-GCST90001781 | rs4242783 | 10 | 5064327 | A | -0.0689292 | 0.0147693 | 2.64E-06 |
| ebi-a-GCST90016923 | Gut microbiota abundance (class Verrucomicrobiae id.4029) || id:ebi-a-GCST90016923 | CD25 on IgD+ CD38+ B cell || id:ebi-a-GCST90001783 | rs4242783 | 10 | 5064327 | A | -0.0689292 | 0.0147693 | 2.64E-06 |
| ebi-a-GCST90016923 | Gut microbiota abundance (class Verrucomicrobiae id.4029) || id:ebi-a-GCST90016923 | CD86 on myeloid Dendritic Cell || id:ebi-a-GCST90001903 | rs4242783 | 10 | 5064327 | A | -0.0689292 | 0.0147693 | 2.64E-06 |
| ebi-a-GCST90016923 | Gut microbiota abundance (class Verrucomicrobiae id.4029) || id:ebi-a-GCST90016923 | CD33 on CD14+ monocyte || id:ebi-a-GCST90001946 | rs4242783 | 10 | 5064327 | A | -0.0689292 | 0.0147693 | 2.64E-06 |
| ebi-a-GCST90016923 | Gut microbiota abundance (class Verrucomicrobiae id.4029) || id:ebi-a-GCST90016923 | CD33 on CD33+ HLA DR+ CD14dim || id:ebi-a-GCST90001947 | rs4242783 | 10 | 5064327 | A | -0.0689292 | 0.0147693 | 2.64E-06 |
| ebi-a-GCST90016923 | Gut microbiota abundance (class Verrucomicrobiae id.4029) || id:ebi-a-GCST90016923 | CD33 on CD33dim HLA DR+ CD11b+ || id:ebi-a-GCST90001948 | rs4242783 | 10 | 5064327 | A | -0.0689292 | 0.0147693 | 2.64E-06 |
| ebi-a-GCST90016923 | Gut microbiota abundance (class Verrucomicrobiae id.4029) || id:ebi-a-GCST90016923 | CD33 on Granulocytic Myeloid-Derived Suppressor Cells || id:ebi-a-GCST90001950 | rs4242783 | 10 | 5064327 | A | -0.0689292 | 0.0147693 | 2.64E-06 |
| ebi-a-GCST90016923 | Gut microbiota abundance (class Verrucomicrobiae id.4029) || id:ebi-a-GCST90016923 | CD33 on CD66b++ myeloid cell || id:ebi-a-GCST90001951 | rs4242783 | 10 | 5064327 | A | -0.0689292 | 0.0147693 | 2.64E-06 |
| ebi-a-GCST90016923 | Gut microbiota abundance (class Verrucomicrobiae id.4029) || id:ebi-a-GCST90016923 | CD33 on CD33dim HLA DR- || id:ebi-a-GCST90001953 | rs4242783 | 10 | 5064327 | A | -0.0689292 | 0.0147693 | 2.64E-06 |
| ebi-a-GCST90016923 | Gut microbiota abundance (class Verrucomicrobiae id.4029) || id:ebi-a-GCST90016923 | CD33 on basophil || id:ebi-a-GCST90001954 | rs4242783 | 10 | 5064327 | A | -0.0689292 | 0.0147693 | 2.64E-06 |
| ebi-a-GCST90016923 | Gut microbiota abundance (class Verrucomicrobiae id.4029) || id:ebi-a-GCST90016923 | CD33 on Immature Myeloid-Derived Suppressor Cells || id:ebi-a-GCST90001955 | rs4242783 | 10 | 5064327 | A | -0.0689292 | 0.0147693 | 2.64E-06 |
| ebi-a-GCST90016923 | Gut microbiota abundance (class Verrucomicrobiae id.4029) || id:ebi-a-GCST90016923 | FSC-A on HLA DR+ CD8+ T cell || id:ebi-a-GCST90001978 | rs4242783 | 10 | 5064327 | A | -0.0689292 | 0.0147693 | 2.64E-06 |
| ebi-a-GCST90016923 | Gut microbiota abundance (class Verrucomicrobiae id.4029) || id:ebi-a-GCST90016923 | HLA DR on CD14+ CD16- monocyte || id:ebi-a-GCST90001988 | rs4242783 | 10 | 5064327 | A | -0.0689292 | 0.0147693 | 2.64E-06 |
| ebi-a-GCST90016923 | Gut microbiota abundance (class Verrucomicrobiae id.4029) || id:ebi-a-GCST90016923 | HLA DR on CD14+ monocyte || id:ebi-a-GCST90001991 | rs4242783 | 10 | 5064327 | A | -0.0689292 | 0.0147693 | 2.64E-06 |
| ebi-a-GCST90016923 | Gut microbiota abundance (class Verrucomicrobiae id.4029) || id:ebi-a-GCST90016923 | CD16 on CD14+ CD16+ monocyte || id:ebi-a-GCST90002005 | rs4242783 | 10 | 5064327 | A | -0.0689292 | 0.0147693 | 2.64E-06 |
| ebi-a-GCST90016923 | Gut microbiota abundance (class Verrucomicrobiae id.4029) || id:ebi-a-GCST90016923 | CD45 on CD33+ HLA DR+ CD14- || id:ebi-a-GCST90002042 | rs4242783 | 10 | 5064327 | A | -0.0689292 | 0.0147693 | 2.64E-06 |
| ebi-a-GCST90016923 | Gut microbiota abundance (class Verrucomicrobiae id.4029) || id:ebi-a-GCST90016923 | CD8 on Natural Killer T || id:ebi-a-GCST90002059 | rs4242783 | 10 | 5064327 | A | -0.0689292 | 0.0147693 | 2.64E-06 |
| ebi-a-GCST90017108 | Gut microbiota abundance (order Verrucomicrobiales id.4030) || id:ebi-a-GCST90017108 | Myeloid Dendritic Cell Absolute Count || id:ebi-a-GCST90001458 | rs4242783 | 10 | 5064327 | A | -0.0689292 | 0.0147693 | 2.64E-06 |
| ebi-a-GCST90017108 | Gut microbiota abundance (order Verrucomicrobiales id.4030) || id:ebi-a-GCST90017108 | CD86+ myeloid Dendritic Cell %Dendritic Cell || id:ebi-a-GCST90001465 | rs4242783 | 10 | 5064327 | A | -0.0689292 | 0.0147693 | 2.64E-06 |
| ebi-a-GCST90017108 | Gut microbiota abundance (order Verrucomicrobiales id.4030) || id:ebi-a-GCST90017108 | CD62L- myeloid Dendritic Cell Absolute Count || id:ebi-a-GCST90001468 | rs4242783 | 10 | 5064327 | A | -0.0689292 | 0.0147693 | 2.64E-06 |
| ebi-a-GCST90017108 | Gut microbiota abundance (order Verrucomicrobiales id.4030) || id:ebi-a-GCST90017108 | CD62L- myeloid Dendritic Cell %Dendritic Cell || id:ebi-a-GCST90001469 | rs4242783 | 10 | 5064327 | A | -0.0689292 | 0.0147693 | 2.64E-06 |
| ebi-a-GCST90017108 | Gut microbiota abundance (order Verrucomicrobiales id.4030) || id:ebi-a-GCST90017108 | CD62L- CD86+ myeloid Dendritic Cell Absolute Count || id:ebi-a-GCST90001472 | rs4242783 | 10 | 5064327 | A | -0.0689292 | 0.0147693 | 2.64E-06 |
| ebi-a-GCST90017108 | Gut microbiota abundance (order Verrucomicrobiales id.4030) || id:ebi-a-GCST90017108 | CD62L- CD86+ myeloid Dendritic Cell %Dendritic Cell || id:ebi-a-GCST90001473 | rs4242783 | 10 | 5064327 | A | -0.0689292 | 0.0147693 | 2.64E-06 |
| ebi-a-GCST90017108 | Gut microbiota abundance (order Verrucomicrobiales id.4030) || id:ebi-a-GCST90017108 | HLA DR++ monocyte %monocyte || id:ebi-a-GCST90001475 | rs4242783 | 10 | 5064327 | A | -0.0689292 | 0.0147693 | 2.64E-06 |
| ebi-a-GCST90017108 | Gut microbiota abundance (order Verrucomicrobiales id.4030) || id:ebi-a-GCST90017108 | HLA DR++ monocyte Absolute Count || id:ebi-a-GCST90001477 | rs4242783 | 10 | 5064327 | A | -0.0689292 | 0.0147693 | 2.64E-06 |
| ebi-a-GCST90017108 | Gut microbiota abundance (order Verrucomicrobiales id.4030) || id:ebi-a-GCST90017108 | Basophil %CD33dim HLA DR- CD66b- || id:ebi-a-GCST90001533 | rs4242783 | 10 | 5064327 | A | -0.0689292 | 0.0147693 | 2.64E-06 |
| ebi-a-GCST90017108 | Gut microbiota abundance (order Verrucomicrobiales id.4030) || id:ebi-a-GCST90017108 | CD16+ monocyte %monocyte || id:ebi-a-GCST90001587 | rs4242783 | 10 | 5064327 | A | -0.0689292 | 0.0147693 | 2.64E-06 |
| ebi-a-GCST90017108 | Gut microbiota abundance (order Verrucomicrobiales id.4030) || id:ebi-a-GCST90017108 | T/B cell || id:ebi-a-GCST90001588 | rs4242783 | 10 | 5064327 | A | -0.0689292 | 0.0147693 | 2.64E-06 |
| ebi-a-GCST90017108 | Gut microbiota abundance (order Verrucomicrobiales id.4030) || id:ebi-a-GCST90017108 | CD8+ Natural Killer T %T cell || id:ebi-a-GCST90001631 | rs4242783 | 10 | 5064327 | A | -0.0689292 | 0.0147693 | 2.64E-06 |
| ebi-a-GCST90017108 | Gut microbiota abundance (order Verrucomicrobiales id.4030) || id:ebi-a-GCST90017108 | CD4-CD8- Natural Killer T %lymphocyte || id:ebi-a-GCST90001638 | rs4242783 | 10 | 5064327 | A | -0.0689292 | 0.0147693 | 2.64E-06 |
| ebi-a-GCST90017108 | Gut microbiota abundance (order Verrucomicrobiales id.4030) || id:ebi-a-GCST90017108 | HLA DR+ Natural Killer Absolute Count || id:ebi-a-GCST90001648 | rs4242783 | 10 | 5064327 | A | -0.0689292 | 0.0147693 | 2.64E-06 |
| ebi-a-GCST90017108 | Gut microbiota abundance (order Verrucomicrobiales id.4030) || id:ebi-a-GCST90017108 | HLA DR+ Natural Killer %Natural Killer || id:ebi-a-GCST90001649 | rs4242783 | 10 | 5064327 | A | -0.0689292 | 0.0147693 | 2.64E-06 |
| ebi-a-GCST90017108 | Gut microbiota abundance (order Verrucomicrobiales id.4030) || id:ebi-a-GCST90017108 | HLA DR+ Natural Killer %CD3- lymphocyte || id:ebi-a-GCST90001650 | rs4242783 | 10 | 5064327 | A | -0.0689292 | 0.0147693 | 2.64E-06 |
| ebi-a-GCST90017108 | Gut microbiota abundance (order Verrucomicrobiales id.4030) || id:ebi-a-GCST90017108 | CD28- CD25++ CD8+ T cell Absolute Count || id:ebi-a-GCST90001678 | rs4242783 | 10 | 5064327 | A | -0.0689292 | 0.0147693 | 2.64E-06 |
| ebi-a-GCST90017108 | Gut microbiota abundance (order Verrucomicrobiales id.4030) || id:ebi-a-GCST90017108 | CD25++ CD8+ T cell %T cell || id:ebi-a-GCST90001679 | rs4242783 | 10 | 5064327 | A | -0.0689292 | 0.0147693 | 2.64E-06 |
| ebi-a-GCST90017108 | Gut microbiota abundance (order Verrucomicrobiales id.4030) || id:ebi-a-GCST90017108 | CD19 on CD20- CD38- B cell || id:ebi-a-GCST90001722 | rs4242783 | 10 | 5064327 | A | -0.0689292 | 0.0147693 | 2.64E-06 |
| ebi-a-GCST90017108 | Gut microbiota abundance (order Verrucomicrobiales id.4030) || id:ebi-a-GCST90017108 | CD25 on B cell || id:ebi-a-GCST90001775 | rs4242783 | 10 | 5064327 | A | -0.0689292 | 0.0147693 | 2.64E-06 |
| ebi-a-GCST90017108 | Gut microbiota abundance (order Verrucomicrobiales id.4030) || id:ebi-a-GCST90017108 | CD25 on IgD+ CD24- B cell || id:ebi-a-GCST90001779 | rs4242783 | 10 | 5064327 | A | -0.0689292 | 0.0147693 | 2.64E-06 |
| ebi-a-GCST90017108 | Gut microbiota abundance (order Verrucomicrobiales id.4030) || id:ebi-a-GCST90017108 | CD25 on IgD+ CD38- naive B cell || id:ebi-a-GCST90001781 | rs4242783 | 10 | 5064327 | A | -0.0689292 | 0.0147693 | 2.64E-06 |
| ebi-a-GCST90017108 | Gut microbiota abundance (order Verrucomicrobiales id.4030) || id:ebi-a-GCST90017108 | CD25 on IgD+ CD38+ B cell || id:ebi-a-GCST90001783 | rs4242783 | 10 | 5064327 | A | -0.0689292 | 0.0147693 | 2.64E-06 |
| ebi-a-GCST90017108 | Gut microbiota abundance (order Verrucomicrobiales id.4030) || id:ebi-a-GCST90017108 | CD86 on myeloid Dendritic Cell || id:ebi-a-GCST90001903 | rs4242783 | 10 | 5064327 | A | -0.0689292 | 0.0147693 | 2.64E-06 |
| ebi-a-GCST90017108 | Gut microbiota abundance (order Verrucomicrobiales id.4030) || id:ebi-a-GCST90017108 | CD33 on CD14+ monocyte || id:ebi-a-GCST90001946 | rs4242783 | 10 | 5064327 | A | -0.0689292 | 0.0147693 | 2.64E-06 |
| ebi-a-GCST90017108 | Gut microbiota abundance (order Verrucomicrobiales id.4030) || id:ebi-a-GCST90017108 | CD33 on CD33+ HLA DR+ CD14dim || id:ebi-a-GCST90001947 | rs4242783 | 10 | 5064327 | A | -0.0689292 | 0.0147693 | 2.64E-06 |
| ebi-a-GCST90017108 | Gut microbiota abundance (order Verrucomicrobiales id.4030) || id:ebi-a-GCST90017108 | CD33 on CD33dim HLA DR+ CD11b+ || id:ebi-a-GCST90001948 | rs4242783 | 10 | 5064327 | A | -0.0689292 | 0.0147693 | 2.64E-06 |
| ebi-a-GCST90017108 | Gut microbiota abundance (order Verrucomicrobiales id.4030) || id:ebi-a-GCST90017108 | CD33 on Granulocytic Myeloid-Derived Suppressor Cells || id:ebi-a-GCST90001950 | rs4242783 | 10 | 5064327 | A | -0.0689292 | 0.0147693 | 2.64E-06 |
| ebi-a-GCST90017108 | Gut microbiota abundance (order Verrucomicrobiales id.4030) || id:ebi-a-GCST90017108 | CD33 on CD66b++ myeloid cell || id:ebi-a-GCST90001951 | rs4242783 | 10 | 5064327 | A | -0.0689292 | 0.0147693 | 2.64E-06 |
| ebi-a-GCST90017108 | Gut microbiota abundance (order Verrucomicrobiales id.4030) || id:ebi-a-GCST90017108 | CD33 on CD33dim HLA DR- || id:ebi-a-GCST90001953 | rs4242783 | 10 | 5064327 | A | -0.0689292 | 0.0147693 | 2.64E-06 |
| ebi-a-GCST90017108 | Gut microbiota abundance (order Verrucomicrobiales id.4030) || id:ebi-a-GCST90017108 | CD33 on basophil || id:ebi-a-GCST90001954 | rs4242783 | 10 | 5064327 | A | -0.0689292 | 0.0147693 | 2.64E-06 |
| ebi-a-GCST90017108 | Gut microbiota abundance (order Verrucomicrobiales id.4030) || id:ebi-a-GCST90017108 | CD33 on Immature Myeloid-Derived Suppressor Cells || id:ebi-a-GCST90001955 | rs4242783 | 10 | 5064327 | A | -0.0689292 | 0.0147693 | 2.64E-06 |
| ebi-a-GCST90017108 | Gut microbiota abundance (order Verrucomicrobiales id.4030) || id:ebi-a-GCST90017108 | FSC-A on HLA DR+ CD8+ T cell || id:ebi-a-GCST90001978 | rs4242783 | 10 | 5064327 | A | -0.0689292 | 0.0147693 | 2.64E-06 |
| ebi-a-GCST90017108 | Gut microbiota abundance (order Verrucomicrobiales id.4030) || id:ebi-a-GCST90017108 | HLA DR on CD14+ CD16- monocyte || id:ebi-a-GCST90001988 | rs4242783 | 10 | 5064327 | A | -0.0689292 | 0.0147693 | 2.64E-06 |
| ebi-a-GCST90017108 | Gut microbiota abundance (order Verrucomicrobiales id.4030) || id:ebi-a-GCST90017108 | HLA DR on CD14+ monocyte || id:ebi-a-GCST90001991 | rs4242783 | 10 | 5064327 | A | -0.0689292 | 0.0147693 | 2.64E-06 |
| ebi-a-GCST90017108 | Gut microbiota abundance (order Verrucomicrobiales id.4030) || id:ebi-a-GCST90017108 | CD16 on CD14+ CD16+ monocyte || id:ebi-a-GCST90002005 | rs4242783 | 10 | 5064327 | A | -0.0689292 | 0.0147693 | 2.64E-06 |
| ebi-a-GCST90017108 | Gut microbiota abundance (order Verrucomicrobiales id.4030) || id:ebi-a-GCST90017108 | CD45 on CD33+ HLA DR+ CD14- || id:ebi-a-GCST90002042 | rs4242783 | 10 | 5064327 | A | -0.0689292 | 0.0147693 | 2.64E-06 |
| ebi-a-GCST90017108 | Gut microbiota abundance (order Verrucomicrobiales id.4030) || id:ebi-a-GCST90017108 | CD8 on Natural Killer T || id:ebi-a-GCST90002059 | rs4242783 | 10 | 5064327 | A | -0.0689292 | 0.0147693 | 2.64E-06 |
| ebi-a-GCST90016961 | Gut microbiota abundance (genus Akkermansia id.4037) || id:ebi-a-GCST90016961 | Myeloid Dendritic Cell Absolute Count || id:ebi-a-GCST90001458 | rs4242783 | 10 | 5064327 | A | -0.0685454 | 0.0147701 | 3.00E-06 |
| ebi-a-GCST90016961 | Gut microbiota abundance (genus Akkermansia id.4037) || id:ebi-a-GCST90016961 | CD86+ myeloid Dendritic Cell %Dendritic Cell || id:ebi-a-GCST90001465 | rs4242783 | 10 | 5064327 | A | -0.0685454 | 0.0147701 | 3.00E-06 |
| ebi-a-GCST90016961 | Gut microbiota abundance (genus Akkermansia id.4037) || id:ebi-a-GCST90016961 | CD62L- myeloid Dendritic Cell Absolute Count || id:ebi-a-GCST90001468 | rs4242783 | 10 | 5064327 | A | -0.0685454 | 0.0147701 | 3.00E-06 |
| ebi-a-GCST90016961 | Gut microbiota abundance (genus Akkermansia id.4037) || id:ebi-a-GCST90016961 | CD62L- myeloid Dendritic Cell %Dendritic Cell || id:ebi-a-GCST90001469 | rs4242783 | 10 | 5064327 | A | -0.0685454 | 0.0147701 | 3.00E-06 |
| ebi-a-GCST90016961 | Gut microbiota abundance (genus Akkermansia id.4037) || id:ebi-a-GCST90016961 | CD62L- CD86+ myeloid Dendritic Cell Absolute Count || id:ebi-a-GCST90001472 | rs4242783 | 10 | 5064327 | A | -0.0685454 | 0.0147701 | 3.00E-06 |
| ebi-a-GCST90016961 | Gut microbiota abundance (genus Akkermansia id.4037) || id:ebi-a-GCST90016961 | CD62L- CD86+ myeloid Dendritic Cell %Dendritic Cell || id:ebi-a-GCST90001473 | rs4242783 | 10 | 5064327 | A | -0.0685454 | 0.0147701 | 3.00E-06 |
| ebi-a-GCST90016961 | Gut microbiota abundance (genus Akkermansia id.4037) || id:ebi-a-GCST90016961 | HLA DR++ monocyte %monocyte || id:ebi-a-GCST90001475 | rs4242783 | 10 | 5064327 | A | -0.0685454 | 0.0147701 | 3.00E-06 |
| ebi-a-GCST90016961 | Gut microbiota abundance (genus Akkermansia id.4037) || id:ebi-a-GCST90016961 | HLA DR++ monocyte Absolute Count || id:ebi-a-GCST90001477 | rs4242783 | 10 | 5064327 | A | -0.0685454 | 0.0147701 | 3.00E-06 |
| ebi-a-GCST90016961 | Gut microbiota abundance (genus Akkermansia id.4037) || id:ebi-a-GCST90016961 | Basophil %CD33dim HLA DR- CD66b- || id:ebi-a-GCST90001533 | rs4242783 | 10 | 5064327 | A | -0.0685454 | 0.0147701 | 3.00E-06 |
| ebi-a-GCST90016961 | Gut microbiota abundance (genus Akkermansia id.4037) || id:ebi-a-GCST90016961 | CD16+ monocyte %monocyte || id:ebi-a-GCST90001587 | rs4242783 | 10 | 5064327 | A | -0.0685454 | 0.0147701 | 3.00E-06 |
| ebi-a-GCST90016961 | Gut microbiota abundance (genus Akkermansia id.4037) || id:ebi-a-GCST90016961 | T/B cell || id:ebi-a-GCST90001588 | rs4242783 | 10 | 5064327 | A | -0.0685454 | 0.0147701 | 3.00E-06 |
| ebi-a-GCST90016961 | Gut microbiota abundance (genus Akkermansia id.4037) || id:ebi-a-GCST90016961 | CD8+ Natural Killer T %T cell || id:ebi-a-GCST90001631 | rs4242783 | 10 | 5064327 | A | -0.0685454 | 0.0147701 | 3.00E-06 |
| ebi-a-GCST90016961 | Gut microbiota abundance (genus Akkermansia id.4037) || id:ebi-a-GCST90016961 | CD4-CD8- Natural Killer T %lymphocyte || id:ebi-a-GCST90001638 | rs4242783 | 10 | 5064327 | A | -0.0685454 | 0.0147701 | 3.00E-06 |
| ebi-a-GCST90016961 | Gut microbiota abundance (genus Akkermansia id.4037) || id:ebi-a-GCST90016961 | HLA DR+ Natural Killer Absolute Count || id:ebi-a-GCST90001648 | rs4242783 | 10 | 5064327 | A | -0.0685454 | 0.0147701 | 3.00E-06 |
| ebi-a-GCST90016961 | Gut microbiota abundance (genus Akkermansia id.4037) || id:ebi-a-GCST90016961 | HLA DR+ Natural Killer %Natural Killer || id:ebi-a-GCST90001649 | rs4242783 | 10 | 5064327 | A | -0.0685454 | 0.0147701 | 3.00E-06 |
| ebi-a-GCST90016961 | Gut microbiota abundance (genus Akkermansia id.4037) || id:ebi-a-GCST90016961 | HLA DR+ Natural Killer %CD3- lymphocyte || id:ebi-a-GCST90001650 | rs4242783 | 10 | 5064327 | A | -0.0685454 | 0.0147701 | 3.00E-06 |
| ebi-a-GCST90016961 | Gut microbiota abundance (genus Akkermansia id.4037) || id:ebi-a-GCST90016961 | CD28- CD25++ CD8+ T cell Absolute Count || id:ebi-a-GCST90001678 | rs4242783 | 10 | 5064327 | A | -0.0685454 | 0.0147701 | 3.00E-06 |
| ebi-a-GCST90016961 | Gut microbiota abundance (genus Akkermansia id.4037) || id:ebi-a-GCST90016961 | CD25++ CD8+ T cell %T cell || id:ebi-a-GCST90001679 | rs4242783 | 10 | 5064327 | A | -0.0685454 | 0.0147701 | 3.00E-06 |
| ebi-a-GCST90016961 | Gut microbiota abundance (genus Akkermansia id.4037) || id:ebi-a-GCST90016961 | CD19 on CD20- CD38- B cell || id:ebi-a-GCST90001722 | rs4242783 | 10 | 5064327 | A | -0.0685454 | 0.0147701 | 3.00E-06 |
| ebi-a-GCST90016961 | Gut microbiota abundance (genus Akkermansia id.4037) || id:ebi-a-GCST90016961 | CD25 on B cell || id:ebi-a-GCST90001775 | rs4242783 | 10 | 5064327 | A | -0.0685454 | 0.0147701 | 3.00E-06 |
| ebi-a-GCST90016961 | Gut microbiota abundance (genus Akkermansia id.4037) || id:ebi-a-GCST90016961 | CD25 on IgD+ CD24- B cell || id:ebi-a-GCST90001779 | rs4242783 | 10 | 5064327 | A | -0.0685454 | 0.0147701 | 3.00E-06 |
| ebi-a-GCST90016961 | Gut microbiota abundance (genus Akkermansia id.4037) || id:ebi-a-GCST90016961 | CD25 on IgD+ CD38- naive B cell || id:ebi-a-GCST90001781 | rs4242783 | 10 | 5064327 | A | -0.0685454 | 0.0147701 | 3.00E-06 |
| ebi-a-GCST90016961 | Gut microbiota abundance (genus Akkermansia id.4037) || id:ebi-a-GCST90016961 | CD25 on IgD+ CD38+ B cell || id:ebi-a-GCST90001783 | rs4242783 | 10 | 5064327 | A | -0.0685454 | 0.0147701 | 3.00E-06 |
| ebi-a-GCST90016961 | Gut microbiota abundance (genus Akkermansia id.4037) || id:ebi-a-GCST90016961 | CD86 on myeloid Dendritic Cell || id:ebi-a-GCST90001903 | rs4242783 | 10 | 5064327 | A | -0.0685454 | 0.0147701 | 3.00E-06 |
| ebi-a-GCST90016961 | Gut microbiota abundance (genus Akkermansia id.4037) || id:ebi-a-GCST90016961 | CD33 on CD14+ monocyte || id:ebi-a-GCST90001946 | rs4242783 | 10 | 5064327 | A | -0.0685454 | 0.0147701 | 3.00E-06 |
| ebi-a-GCST90016961 | Gut microbiota abundance (genus Akkermansia id.4037) || id:ebi-a-GCST90016961 | CD33 on CD33+ HLA DR+ CD14dim || id:ebi-a-GCST90001947 | rs4242783 | 10 | 5064327 | A | -0.0685454 | 0.0147701 | 3.00E-06 |
| ebi-a-GCST90016961 | Gut microbiota abundance (genus Akkermansia id.4037) || id:ebi-a-GCST90016961 | CD33 on CD33dim HLA DR+ CD11b+ || id:ebi-a-GCST90001948 | rs4242783 | 10 | 5064327 | A | -0.0685454 | 0.0147701 | 3.00E-06 |
| ebi-a-GCST90016961 | Gut microbiota abundance (genus Akkermansia id.4037) || id:ebi-a-GCST90016961 | CD33 on Granulocytic Myeloid-Derived Suppressor Cells || id:ebi-a-GCST90001950 | rs4242783 | 10 | 5064327 | A | -0.0685454 | 0.0147701 | 3.00E-06 |
| ebi-a-GCST90016961 | Gut microbiota abundance (genus Akkermansia id.4037) || id:ebi-a-GCST90016961 | CD33 on CD66b++ myeloid cell || id:ebi-a-GCST90001951 | rs4242783 | 10 | 5064327 | A | -0.0685454 | 0.0147701 | 3.00E-06 |
| ebi-a-GCST90016961 | Gut microbiota abundance (genus Akkermansia id.4037) || id:ebi-a-GCST90016961 | CD33 on CD33dim HLA DR- || id:ebi-a-GCST90001953 | rs4242783 | 10 | 5064327 | A | -0.0685454 | 0.0147701 | 3.00E-06 |
| ebi-a-GCST90016961 | Gut microbiota abundance (genus Akkermansia id.4037) || id:ebi-a-GCST90016961 | CD33 on basophil || id:ebi-a-GCST90001954 | rs4242783 | 10 | 5064327 | A | -0.0685454 | 0.0147701 | 3.00E-06 |
| ebi-a-GCST90016961 | Gut microbiota abundance (genus Akkermansia id.4037) || id:ebi-a-GCST90016961 | CD33 on Immature Myeloid-Derived Suppressor Cells || id:ebi-a-GCST90001955 | rs4242783 | 10 | 5064327 | A | -0.0685454 | 0.0147701 | 3.00E-06 |
| ebi-a-GCST90016961 | Gut microbiota abundance (genus Akkermansia id.4037) || id:ebi-a-GCST90016961 | FSC-A on HLA DR+ CD8+ T cell || id:ebi-a-GCST90001978 | rs4242783 | 10 | 5064327 | A | -0.0685454 | 0.0147701 | 3.00E-06 |
| ebi-a-GCST90016961 | Gut microbiota abundance (genus Akkermansia id.4037) || id:ebi-a-GCST90016961 | HLA DR on CD14+ CD16- monocyte || id:ebi-a-GCST90001988 | rs4242783 | 10 | 5064327 | A | -0.0685454 | 0.0147701 | 3.00E-06 |
| ebi-a-GCST90016961 | Gut microbiota abundance (genus Akkermansia id.4037) || id:ebi-a-GCST90016961 | HLA DR on CD14+ monocyte || id:ebi-a-GCST90001991 | rs4242783 | 10 | 5064327 | A | -0.0685454 | 0.0147701 | 3.00E-06 |
| ebi-a-GCST90016961 | Gut microbiota abundance (genus Akkermansia id.4037) || id:ebi-a-GCST90016961 | CD16 on CD14+ CD16+ monocyte || id:ebi-a-GCST90002005 | rs4242783 | 10 | 5064327 | A | -0.0685454 | 0.0147701 | 3.00E-06 |
| ebi-a-GCST90016961 | Gut microbiota abundance (genus Akkermansia id.4037) || id:ebi-a-GCST90016961 | CD45 on CD33+ HLA DR+ CD14- || id:ebi-a-GCST90002042 | rs4242783 | 10 | 5064327 | A | -0.0685454 | 0.0147701 | 3.00E-06 |
| ebi-a-GCST90016961 | Gut microbiota abundance (genus Akkermansia id.4037) || id:ebi-a-GCST90016961 | CD8 on Natural Killer T || id:ebi-a-GCST90002059 | rs4242783 | 10 | 5064327 | A | -0.0685454 | 0.0147701 | 3.00E-06 |
| ebi-a-GCST90016943 | Gut microbiota abundance (family Oxalobacteraceae id.2966) || id:ebi-a-GCST90016943 | Myeloid Dendritic Cell Absolute Count || id:ebi-a-GCST90001458 | rs4428215 | 3 | 171947435 | G | 0.12561 | 0.0230053 | 4.88E-08 |
| ebi-a-GCST90016943 | Gut microbiota abundance (family Oxalobacteraceae id.2966) || id:ebi-a-GCST90016943 | CD86+ myeloid Dendritic Cell %Dendritic Cell || id:ebi-a-GCST90001465 | rs4428215 | 3 | 171947435 | G | 0.12561 | 0.0230053 | 4.88E-08 |
| ebi-a-GCST90016943 | Gut microbiota abundance (family Oxalobacteraceae id.2966) || id:ebi-a-GCST90016943 | CD62L- myeloid Dendritic Cell Absolute Count || id:ebi-a-GCST90001468 | rs4428215 | 3 | 171947435 | G | 0.12561 | 0.0230053 | 4.88E-08 |
| ebi-a-GCST90016943 | Gut microbiota abundance (family Oxalobacteraceae id.2966) || id:ebi-a-GCST90016943 | CD62L- myeloid Dendritic Cell %Dendritic Cell || id:ebi-a-GCST90001469 | rs4428215 | 3 | 171947435 | G | 0.12561 | 0.0230053 | 4.88E-08 |
| ebi-a-GCST90016943 | Gut microbiota abundance (family Oxalobacteraceae id.2966) || id:ebi-a-GCST90016943 | CD62L- CD86+ myeloid Dendritic Cell Absolute Count || id:ebi-a-GCST90001472 | rs4428215 | 3 | 171947435 | G | 0.12561 | 0.0230053 | 4.88E-08 |
| ebi-a-GCST90016943 | Gut microbiota abundance (family Oxalobacteraceae id.2966) || id:ebi-a-GCST90016943 | CD62L- CD86+ myeloid Dendritic Cell %Dendritic Cell || id:ebi-a-GCST90001473 | rs4428215 | 3 | 171947435 | G | 0.12561 | 0.0230053 | 4.88E-08 |
| ebi-a-GCST90016943 | Gut microbiota abundance (family Oxalobacteraceae id.2966) || id:ebi-a-GCST90016943 | HLA DR++ monocyte %monocyte || id:ebi-a-GCST90001475 | rs4428215 | 3 | 171947435 | G | 0.12561 | 0.0230053 | 4.88E-08 |
| ebi-a-GCST90016943 | Gut microbiota abundance (family Oxalobacteraceae id.2966) || id:ebi-a-GCST90016943 | HLA DR++ monocyte Absolute Count || id:ebi-a-GCST90001477 | rs4428215 | 3 | 171947435 | G | 0.12561 | 0.0230053 | 4.88E-08 |
| ebi-a-GCST90016943 | Gut microbiota abundance (family Oxalobacteraceae id.2966) || id:ebi-a-GCST90016943 | Basophil %CD33dim HLA DR- CD66b- || id:ebi-a-GCST90001533 | rs4428215 | 3 | 171947435 | G | 0.12561 | 0.0230053 | 4.88E-08 |
| ebi-a-GCST90016943 | Gut microbiota abundance (family Oxalobacteraceae id.2966) || id:ebi-a-GCST90016943 | CD16+ monocyte %monocyte || id:ebi-a-GCST90001587 | rs4428215 | 3 | 171947435 | G | 0.12561 | 0.0230053 | 4.88E-08 |
| ebi-a-GCST90016943 | Gut microbiota abundance (family Oxalobacteraceae id.2966) || id:ebi-a-GCST90016943 | T/B cell || id:ebi-a-GCST90001588 | rs4428215 | 3 | 171947435 | G | 0.12561 | 0.0230053 | 4.88E-08 |
| ebi-a-GCST90016943 | Gut microbiota abundance (family Oxalobacteraceae id.2966) || id:ebi-a-GCST90016943 | CD4+CD8+ T cell %T cell || id:ebi-a-GCST90001595 | rs4428215 | 3 | 171947435 | G | 0.12561 | 0.0230053 | 4.88E-08 |
| ebi-a-GCST90016943 | Gut microbiota abundance (family Oxalobacteraceae id.2966) || id:ebi-a-GCST90016943 | CD8+ Natural Killer T %T cell || id:ebi-a-GCST90001631 | rs4428215 | 3 | 171947435 | G | 0.12561 | 0.0230053 | 4.88E-08 |
| ebi-a-GCST90016943 | Gut microbiota abundance (family Oxalobacteraceae id.2966) || id:ebi-a-GCST90016943 | CD4-CD8- Natural Killer T %lymphocyte || id:ebi-a-GCST90001638 | rs4428215 | 3 | 171947435 | G | 0.12561 | 0.0230053 | 4.88E-08 |
| ebi-a-GCST90016943 | Gut microbiota abundance (family Oxalobacteraceae id.2966) || id:ebi-a-GCST90016943 | HLA DR+ Natural Killer Absolute Count || id:ebi-a-GCST90001648 | rs4428215 | 3 | 171947435 | G | 0.12561 | 0.0230053 | 4.88E-08 |
| ebi-a-GCST90016943 | Gut microbiota abundance (family Oxalobacteraceae id.2966) || id:ebi-a-GCST90016943 | HLA DR+ Natural Killer %Natural Killer || id:ebi-a-GCST90001649 | rs4428215 | 3 | 171947435 | G | 0.12561 | 0.0230053 | 4.88E-08 |
| ebi-a-GCST90016943 | Gut microbiota abundance (family Oxalobacteraceae id.2966) || id:ebi-a-GCST90016943 | HLA DR+ Natural Killer %CD3- lymphocyte || id:ebi-a-GCST90001650 | rs4428215 | 3 | 171947435 | G | 0.12561 | 0.0230053 | 4.88E-08 |
| ebi-a-GCST90016943 | Gut microbiota abundance (family Oxalobacteraceae id.2966) || id:ebi-a-GCST90016943 | CD28- CD25++ CD8+ T cell Absolute Count || id:ebi-a-GCST90001678 | rs4428215 | 3 | 171947435 | G | 0.12561 | 0.0230053 | 4.88E-08 |
| ebi-a-GCST90016943 | Gut microbiota abundance (family Oxalobacteraceae id.2966) || id:ebi-a-GCST90016943 | CD25++ CD8+ T cell %T cell || id:ebi-a-GCST90001679 | rs4428215 | 3 | 171947435 | G | 0.12561 | 0.0230053 | 4.88E-08 |
| ebi-a-GCST90016943 | Gut microbiota abundance (family Oxalobacteraceae id.2966) || id:ebi-a-GCST90016943 | CD19 on CD20- CD38- B cell || id:ebi-a-GCST90001722 | rs4428215 | 3 | 171947435 | G | 0.12561 | 0.0230053 | 4.88E-08 |
| ebi-a-GCST90016943 | Gut microbiota abundance (family Oxalobacteraceae id.2966) || id:ebi-a-GCST90016943 | CD25 on B cell || id:ebi-a-GCST90001775 | rs4428215 | 3 | 171947435 | G | 0.12561 | 0.0230053 | 4.88E-08 |
| ebi-a-GCST90016943 | Gut microbiota abundance (family Oxalobacteraceae id.2966) || id:ebi-a-GCST90016943 | CD25 on IgD+ CD24- B cell || id:ebi-a-GCST90001779 | rs4428215 | 3 | 171947435 | G | 0.12561 | 0.0230053 | 4.88E-08 |
| ebi-a-GCST90016943 | Gut microbiota abundance (family Oxalobacteraceae id.2966) || id:ebi-a-GCST90016943 | CD25 on IgD+ CD38- naive B cell || id:ebi-a-GCST90001781 | rs4428215 | 3 | 171947435 | G | 0.12561 | 0.0230053 | 4.88E-08 |
| ebi-a-GCST90016943 | Gut microbiota abundance (family Oxalobacteraceae id.2966) || id:ebi-a-GCST90016943 | CD25 on IgD+ CD38+ B cell || id:ebi-a-GCST90001783 | rs4428215 | 3 | 171947435 | G | 0.12561 | 0.0230053 | 4.88E-08 |
| ebi-a-GCST90016943 | Gut microbiota abundance (family Oxalobacteraceae id.2966) || id:ebi-a-GCST90016943 | CD86 on myeloid Dendritic Cell || id:ebi-a-GCST90001903 | rs4428215 | 3 | 171947435 | G | 0.12561 | 0.0230053 | 4.88E-08 |
| ebi-a-GCST90016943 | Gut microbiota abundance (family Oxalobacteraceae id.2966) || id:ebi-a-GCST90016943 | CD33 on CD14+ monocyte || id:ebi-a-GCST90001946 | rs4428215 | 3 | 171947435 | G | 0.12561 | 0.0230053 | 4.88E-08 |
| ebi-a-GCST90016943 | Gut microbiota abundance (family Oxalobacteraceae id.2966) || id:ebi-a-GCST90016943 | CD33 on CD33+ HLA DR+ CD14dim || id:ebi-a-GCST90001947 | rs4428215 | 3 | 171947435 | G | 0.12561 | 0.0230053 | 4.88E-08 |
| ebi-a-GCST90016943 | Gut microbiota abundance (family Oxalobacteraceae id.2966) || id:ebi-a-GCST90016943 | CD33 on CD33dim HLA DR+ CD11b+ || id:ebi-a-GCST90001948 | rs4428215 | 3 | 171947435 | G | 0.12561 | 0.0230053 | 4.88E-08 |
| ebi-a-GCST90016943 | Gut microbiota abundance (family Oxalobacteraceae id.2966) || id:ebi-a-GCST90016943 | CD33 on Granulocytic Myeloid-Derived Suppressor Cells || id:ebi-a-GCST90001950 | rs4428215 | 3 | 171947435 | G | 0.12561 | 0.0230053 | 4.88E-08 |
| ebi-a-GCST90016943 | Gut microbiota abundance (family Oxalobacteraceae id.2966) || id:ebi-a-GCST90016943 | CD33 on CD66b++ myeloid cell || id:ebi-a-GCST90001951 | rs4428215 | 3 | 171947435 | G | 0.12561 | 0.0230053 | 4.88E-08 |
| ebi-a-GCST90016943 | Gut microbiota abundance (family Oxalobacteraceae id.2966) || id:ebi-a-GCST90016943 | CD33 on CD33dim HLA DR- || id:ebi-a-GCST90001953 | rs4428215 | 3 | 171947435 | G | 0.12561 | 0.0230053 | 4.88E-08 |
| ebi-a-GCST90016943 | Gut microbiota abundance (family Oxalobacteraceae id.2966) || id:ebi-a-GCST90016943 | CD33 on basophil || id:ebi-a-GCST90001954 | rs4428215 | 3 | 171947435 | G | 0.12561 | 0.0230053 | 4.88E-08 |
| ebi-a-GCST90016943 | Gut microbiota abundance (family Oxalobacteraceae id.2966) || id:ebi-a-GCST90016943 | CD33 on Immature Myeloid-Derived Suppressor Cells || id:ebi-a-GCST90001955 | rs4428215 | 3 | 171947435 | G | 0.12561 | 0.0230053 | 4.88E-08 |
| ebi-a-GCST90016943 | Gut microbiota abundance (family Oxalobacteraceae id.2966) || id:ebi-a-GCST90016943 | FSC-A on HLA DR+ CD8+ T cell || id:ebi-a-GCST90001978 | rs4428215 | 3 | 171947435 | G | 0.12561 | 0.0230053 | 4.88E-08 |
| ebi-a-GCST90016943 | Gut microbiota abundance (family Oxalobacteraceae id.2966) || id:ebi-a-GCST90016943 | HLA DR on CD14+ CD16- monocyte || id:ebi-a-GCST90001988 | rs4428215 | 3 | 171947435 | G | 0.12561 | 0.0230053 | 4.88E-08 |
| ebi-a-GCST90016943 | Gut microbiota abundance (family Oxalobacteraceae id.2966) || id:ebi-a-GCST90016943 | HLA DR on CD14+ monocyte || id:ebi-a-GCST90001991 | rs4428215 | 3 | 171947435 | G | 0.12561 | 0.0230053 | 4.88E-08 |
| ebi-a-GCST90016943 | Gut microbiota abundance (family Oxalobacteraceae id.2966) || id:ebi-a-GCST90016943 | CD16 on CD14+ CD16+ monocyte || id:ebi-a-GCST90002005 | rs4428215 | 3 | 171947435 | G | 0.12561 | 0.0230053 | 4.88E-08 |
| ebi-a-GCST90016943 | Gut microbiota abundance (family Oxalobacteraceae id.2966) || id:ebi-a-GCST90016943 | CD45 on CD33+ HLA DR+ CD14- || id:ebi-a-GCST90002042 | rs4428215 | 3 | 171947435 | G | 0.12561 | 0.0230053 | 4.88E-08 |
| ebi-a-GCST90016943 | Gut microbiota abundance (family Oxalobacteraceae id.2966) || id:ebi-a-GCST90016943 | CD8 on Natural Killer T || id:ebi-a-GCST90002059 | rs4428215 | 3 | 171947435 | G | 0.12561 | 0.0230053 | 4.88E-08 |
| ebi-a-GCST90016957 | Gut microbiota abundance (family Verrucomicrobiaceae id.4036) || id:ebi-a-GCST90016957 | Myeloid Dendritic Cell Absolute Count || id:ebi-a-GCST90001458 | rs4936098 | 11 | 130280667 | G | -0.0648691 | 0.0135928 | 1.13E-06 |
| ebi-a-GCST90016957 | Gut microbiota abundance (family Verrucomicrobiaceae id.4036) || id:ebi-a-GCST90016957 | CD86+ myeloid Dendritic Cell %Dendritic Cell || id:ebi-a-GCST90001465 | rs4936098 | 11 | 130280667 | G | -0.0648691 | 0.0135928 | 1.13E-06 |
| ebi-a-GCST90016957 | Gut microbiota abundance (family Verrucomicrobiaceae id.4036) || id:ebi-a-GCST90016957 | CD62L- myeloid Dendritic Cell Absolute Count || id:ebi-a-GCST90001468 | rs4936098 | 11 | 130280667 | G | -0.0648691 | 0.0135928 | 1.13E-06 |
| ebi-a-GCST90016957 | Gut microbiota abundance (family Verrucomicrobiaceae id.4036) || id:ebi-a-GCST90016957 | CD62L- myeloid Dendritic Cell %Dendritic Cell || id:ebi-a-GCST90001469 | rs4936098 | 11 | 130280667 | G | -0.0648691 | 0.0135928 | 1.13E-06 |
| ebi-a-GCST90016957 | Gut microbiota abundance (family Verrucomicrobiaceae id.4036) || id:ebi-a-GCST90016957 | CD62L- CD86+ myeloid Dendritic Cell Absolute Count || id:ebi-a-GCST90001472 | rs4936098 | 11 | 130280667 | G | -0.0648691 | 0.0135928 | 1.13E-06 |
| ebi-a-GCST90016957 | Gut microbiota abundance (family Verrucomicrobiaceae id.4036) || id:ebi-a-GCST90016957 | CD62L- CD86+ myeloid Dendritic Cell %Dendritic Cell || id:ebi-a-GCST90001473 | rs4936098 | 11 | 130280667 | G | -0.0648691 | 0.0135928 | 1.13E-06 |
| ebi-a-GCST90016957 | Gut microbiota abundance (family Verrucomicrobiaceae id.4036) || id:ebi-a-GCST90016957 | HLA DR++ monocyte %monocyte || id:ebi-a-GCST90001475 | rs4936098 | 11 | 130280667 | G | -0.0648691 | 0.0135928 | 1.13E-06 |
| ebi-a-GCST90016957 | Gut microbiota abundance (family Verrucomicrobiaceae id.4036) || id:ebi-a-GCST90016957 | HLA DR++ monocyte Absolute Count || id:ebi-a-GCST90001477 | rs4936098 | 11 | 130280667 | G | -0.0648691 | 0.0135928 | 1.13E-06 |
| ebi-a-GCST90016957 | Gut microbiota abundance (family Verrucomicrobiaceae id.4036) || id:ebi-a-GCST90016957 | Basophil %CD33dim HLA DR- CD66b- || id:ebi-a-GCST90001533 | rs4936098 | 11 | 130280667 | G | -0.0648691 | 0.0135928 | 1.13E-06 |
| ebi-a-GCST90016957 | Gut microbiota abundance (family Verrucomicrobiaceae id.4036) || id:ebi-a-GCST90016957 | CD16+ monocyte %monocyte || id:ebi-a-GCST90001587 | rs4936098 | 11 | 130280667 | G | -0.0648691 | 0.0135928 | 1.13E-06 |
| ebi-a-GCST90016957 | Gut microbiota abundance (family Verrucomicrobiaceae id.4036) || id:ebi-a-GCST90016957 | T/B cell || id:ebi-a-GCST90001588 | rs4936098 | 11 | 130280667 | G | -0.0648691 | 0.0135928 | 1.13E-06 |
| ebi-a-GCST90016957 | Gut microbiota abundance (family Verrucomicrobiaceae id.4036) || id:ebi-a-GCST90016957 | CD8+ Natural Killer T %T cell || id:ebi-a-GCST90001631 | rs4936098 | 11 | 130280667 | G | -0.0648691 | 0.0135928 | 1.13E-06 |
| ebi-a-GCST90016957 | Gut microbiota abundance (family Verrucomicrobiaceae id.4036) || id:ebi-a-GCST90016957 | CD4-CD8- Natural Killer T %lymphocyte || id:ebi-a-GCST90001638 | rs4936098 | 11 | 130280667 | G | -0.0648691 | 0.0135928 | 1.13E-06 |
| ebi-a-GCST90016957 | Gut microbiota abundance (family Verrucomicrobiaceae id.4036) || id:ebi-a-GCST90016957 | HLA DR+ Natural Killer Absolute Count || id:ebi-a-GCST90001648 | rs4936098 | 11 | 130280667 | G | -0.0648691 | 0.0135928 | 1.13E-06 |
| ebi-a-GCST90016957 | Gut microbiota abundance (family Verrucomicrobiaceae id.4036) || id:ebi-a-GCST90016957 | HLA DR+ Natural Killer %Natural Killer || id:ebi-a-GCST90001649 | rs4936098 | 11 | 130280667 | G | -0.0648691 | 0.0135928 | 1.13E-06 |
| ebi-a-GCST90016957 | Gut microbiota abundance (family Verrucomicrobiaceae id.4036) || id:ebi-a-GCST90016957 | HLA DR+ Natural Killer %CD3- lymphocyte || id:ebi-a-GCST90001650 | rs4936098 | 11 | 130280667 | G | -0.0648691 | 0.0135928 | 1.13E-06 |
| ebi-a-GCST90016957 | Gut microbiota abundance (family Verrucomicrobiaceae id.4036) || id:ebi-a-GCST90016957 | CD28- CD25++ CD8+ T cell Absolute Count || id:ebi-a-GCST90001678 | rs4936098 | 11 | 130280667 | G | -0.0648691 | 0.0135928 | 1.13E-06 |
| ebi-a-GCST90016957 | Gut microbiota abundance (family Verrucomicrobiaceae id.4036) || id:ebi-a-GCST90016957 | CD25++ CD8+ T cell %T cell || id:ebi-a-GCST90001679 | rs4936098 | 11 | 130280667 | G | -0.0648691 | 0.0135928 | 1.13E-06 |
| ebi-a-GCST90016957 | Gut microbiota abundance (family Verrucomicrobiaceae id.4036) || id:ebi-a-GCST90016957 | CD19 on CD20- CD38- B cell || id:ebi-a-GCST90001722 | rs4936098 | 11 | 130280667 | G | -0.0648691 | 0.0135928 | 1.13E-06 |
| ebi-a-GCST90016957 | Gut microbiota abundance (family Verrucomicrobiaceae id.4036) || id:ebi-a-GCST90016957 | CD25 on B cell || id:ebi-a-GCST90001775 | rs4936098 | 11 | 130280667 | G | -0.0648691 | 0.0135928 | 1.13E-06 |
| ebi-a-GCST90016957 | Gut microbiota abundance (family Verrucomicrobiaceae id.4036) || id:ebi-a-GCST90016957 | CD25 on IgD+ CD24- B cell || id:ebi-a-GCST90001779 | rs4936098 | 11 | 130280667 | G | -0.0648691 | 0.0135928 | 1.13E-06 |
| ebi-a-GCST90016957 | Gut microbiota abundance (family Verrucomicrobiaceae id.4036) || id:ebi-a-GCST90016957 | CD25 on IgD+ CD38- naive B cell || id:ebi-a-GCST90001781 | rs4936098 | 11 | 130280667 | G | -0.0648691 | 0.0135928 | 1.13E-06 |
| ebi-a-GCST90016957 | Gut microbiota abundance (family Verrucomicrobiaceae id.4036) || id:ebi-a-GCST90016957 | CD25 on IgD+ CD38+ B cell || id:ebi-a-GCST90001783 | rs4936098 | 11 | 130280667 | G | -0.0648691 | 0.0135928 | 1.13E-06 |
| ebi-a-GCST90016957 | Gut microbiota abundance (family Verrucomicrobiaceae id.4036) || id:ebi-a-GCST90016957 | CD86 on myeloid Dendritic Cell || id:ebi-a-GCST90001903 | rs4936098 | 11 | 130280667 | G | -0.0648691 | 0.0135928 | 1.13E-06 |
| ebi-a-GCST90016957 | Gut microbiota abundance (family Verrucomicrobiaceae id.4036) || id:ebi-a-GCST90016957 | CD33 on CD14+ monocyte || id:ebi-a-GCST90001946 | rs4936098 | 11 | 130280667 | G | -0.0648691 | 0.0135928 | 1.13E-06 |
| ebi-a-GCST90016957 | Gut microbiota abundance (family Verrucomicrobiaceae id.4036) || id:ebi-a-GCST90016957 | CD33 on CD33+ HLA DR+ CD14dim || id:ebi-a-GCST90001947 | rs4936098 | 11 | 130280667 | G | -0.0648691 | 0.0135928 | 1.13E-06 |
| ebi-a-GCST90016957 | Gut microbiota abundance (family Verrucomicrobiaceae id.4036) || id:ebi-a-GCST90016957 | CD33 on CD33dim HLA DR+ CD11b+ || id:ebi-a-GCST90001948 | rs4936098 | 11 | 130280667 | G | -0.0648691 | 0.0135928 | 1.13E-06 |
| ebi-a-GCST90016957 | Gut microbiota abundance (family Verrucomicrobiaceae id.4036) || id:ebi-a-GCST90016957 | CD33 on Granulocytic Myeloid-Derived Suppressor Cells || id:ebi-a-GCST90001950 | rs4936098 | 11 | 130280667 | G | -0.0648691 | 0.0135928 | 1.13E-06 |
| ebi-a-GCST90016957 | Gut microbiota abundance (family Verrucomicrobiaceae id.4036) || id:ebi-a-GCST90016957 | CD33 on CD66b++ myeloid cell || id:ebi-a-GCST90001951 | rs4936098 | 11 | 130280667 | G | -0.0648691 | 0.0135928 | 1.13E-06 |
| ebi-a-GCST90016957 | Gut microbiota abundance (family Verrucomicrobiaceae id.4036) || id:ebi-a-GCST90016957 | CD33 on CD33dim HLA DR- || id:ebi-a-GCST90001953 | rs4936098 | 11 | 130280667 | G | -0.0648691 | 0.0135928 | 1.13E-06 |
| ebi-a-GCST90016957 | Gut microbiota abundance (family Verrucomicrobiaceae id.4036) || id:ebi-a-GCST90016957 | CD33 on basophil || id:ebi-a-GCST90001954 | rs4936098 | 11 | 130280667 | G | -0.0648691 | 0.0135928 | 1.13E-06 |
| ebi-a-GCST90016957 | Gut microbiota abundance (family Verrucomicrobiaceae id.4036) || id:ebi-a-GCST90016957 | CD33 on Immature Myeloid-Derived Suppressor Cells || id:ebi-a-GCST90001955 | rs4936098 | 11 | 130280667 | G | -0.0648691 | 0.0135928 | 1.13E-06 |
| ebi-a-GCST90016957 | Gut microbiota abundance (family Verrucomicrobiaceae id.4036) || id:ebi-a-GCST90016957 | FSC-A on HLA DR+ CD8+ T cell || id:ebi-a-GCST90001978 | rs4936098 | 11 | 130280667 | G | -0.0648691 | 0.0135928 | 1.13E-06 |
| ebi-a-GCST90016957 | Gut microbiota abundance (family Verrucomicrobiaceae id.4036) || id:ebi-a-GCST90016957 | HLA DR on CD14+ CD16- monocyte || id:ebi-a-GCST90001988 | rs4936098 | 11 | 130280667 | G | -0.0648691 | 0.0135928 | 1.13E-06 |
| ebi-a-GCST90016957 | Gut microbiota abundance (family Verrucomicrobiaceae id.4036) || id:ebi-a-GCST90016957 | HLA DR on CD14+ monocyte || id:ebi-a-GCST90001991 | rs4936098 | 11 | 130280667 | G | -0.0648691 | 0.0135928 | 1.13E-06 |
| ebi-a-GCST90016957 | Gut microbiota abundance (family Verrucomicrobiaceae id.4036) || id:ebi-a-GCST90016957 | CD16 on CD14+ CD16+ monocyte || id:ebi-a-GCST90002005 | rs4936098 | 11 | 130280667 | G | -0.0648691 | 0.0135928 | 1.13E-06 |
| ebi-a-GCST90016957 | Gut microbiota abundance (family Verrucomicrobiaceae id.4036) || id:ebi-a-GCST90016957 | CD45 on CD33+ HLA DR+ CD14- || id:ebi-a-GCST90002042 | rs4936098 | 11 | 130280667 | G | -0.0648691 | 0.0135928 | 1.13E-06 |
| ebi-a-GCST90016957 | Gut microbiota abundance (family Verrucomicrobiaceae id.4036) || id:ebi-a-GCST90016957 | CD8 on Natural Killer T || id:ebi-a-GCST90002059 | rs4936098 | 11 | 130280667 | G | -0.0648691 | 0.0135928 | 1.13E-06 |
| ebi-a-GCST90016923 | Gut microbiota abundance (class Verrucomicrobiae id.4029) || id:ebi-a-GCST90016923 | Myeloid Dendritic Cell Absolute Count || id:ebi-a-GCST90001458 | rs4936098 | 11 | 130280667 | G | -0.0648843 | 0.0135928 | 1.12E-06 |
| ebi-a-GCST90016923 | Gut microbiota abundance (class Verrucomicrobiae id.4029) || id:ebi-a-GCST90016923 | CD86+ myeloid Dendritic Cell %Dendritic Cell || id:ebi-a-GCST90001465 | rs4936098 | 11 | 130280667 | G | -0.0648843 | 0.0135928 | 1.12E-06 |
| ebi-a-GCST90016923 | Gut microbiota abundance (class Verrucomicrobiae id.4029) || id:ebi-a-GCST90016923 | CD62L- myeloid Dendritic Cell Absolute Count || id:ebi-a-GCST90001468 | rs4936098 | 11 | 130280667 | G | -0.0648843 | 0.0135928 | 1.12E-06 |
| ebi-a-GCST90016923 | Gut microbiota abundance (class Verrucomicrobiae id.4029) || id:ebi-a-GCST90016923 | CD62L- myeloid Dendritic Cell %Dendritic Cell || id:ebi-a-GCST90001469 | rs4936098 | 11 | 130280667 | G | -0.0648843 | 0.0135928 | 1.12E-06 |
| ebi-a-GCST90016923 | Gut microbiota abundance (class Verrucomicrobiae id.4029) || id:ebi-a-GCST90016923 | CD62L- CD86+ myeloid Dendritic Cell Absolute Count || id:ebi-a-GCST90001472 | rs4936098 | 11 | 130280667 | G | -0.0648843 | 0.0135928 | 1.12E-06 |
| ebi-a-GCST90016923 | Gut microbiota abundance (class Verrucomicrobiae id.4029) || id:ebi-a-GCST90016923 | CD62L- CD86+ myeloid Dendritic Cell %Dendritic Cell || id:ebi-a-GCST90001473 | rs4936098 | 11 | 130280667 | G | -0.0648843 | 0.0135928 | 1.12E-06 |
| ebi-a-GCST90016923 | Gut microbiota abundance (class Verrucomicrobiae id.4029) || id:ebi-a-GCST90016923 | HLA DR++ monocyte %monocyte || id:ebi-a-GCST90001475 | rs4936098 | 11 | 130280667 | G | -0.0648843 | 0.0135928 | 1.12E-06 |
| ebi-a-GCST90016923 | Gut microbiota abundance (class Verrucomicrobiae id.4029) || id:ebi-a-GCST90016923 | HLA DR++ monocyte Absolute Count || id:ebi-a-GCST90001477 | rs4936098 | 11 | 130280667 | G | -0.0648843 | 0.0135928 | 1.12E-06 |
| ebi-a-GCST90016923 | Gut microbiota abundance (class Verrucomicrobiae id.4029) || id:ebi-a-GCST90016923 | Basophil %CD33dim HLA DR- CD66b- || id:ebi-a-GCST90001533 | rs4936098 | 11 | 130280667 | G | -0.0648843 | 0.0135928 | 1.12E-06 |
| ebi-a-GCST90016923 | Gut microbiota abundance (class Verrucomicrobiae id.4029) || id:ebi-a-GCST90016923 | CD16+ monocyte %monocyte || id:ebi-a-GCST90001587 | rs4936098 | 11 | 130280667 | G | -0.0648843 | 0.0135928 | 1.12E-06 |
| ebi-a-GCST90016923 | Gut microbiota abundance (class Verrucomicrobiae id.4029) || id:ebi-a-GCST90016923 | T/B cell || id:ebi-a-GCST90001588 | rs4936098 | 11 | 130280667 | G | -0.0648843 | 0.0135928 | 1.12E-06 |
| ebi-a-GCST90016923 | Gut microbiota abundance (class Verrucomicrobiae id.4029) || id:ebi-a-GCST90016923 | CD8+ Natural Killer T %T cell || id:ebi-a-GCST90001631 | rs4936098 | 11 | 130280667 | G | -0.0648843 | 0.0135928 | 1.12E-06 |
| ebi-a-GCST90016923 | Gut microbiota abundance (class Verrucomicrobiae id.4029) || id:ebi-a-GCST90016923 | CD4-CD8- Natural Killer T %lymphocyte || id:ebi-a-GCST90001638 | rs4936098 | 11 | 130280667 | G | -0.0648843 | 0.0135928 | 1.12E-06 |
| ebi-a-GCST90016923 | Gut microbiota abundance (class Verrucomicrobiae id.4029) || id:ebi-a-GCST90016923 | HLA DR+ Natural Killer Absolute Count || id:ebi-a-GCST90001648 | rs4936098 | 11 | 130280667 | G | -0.0648843 | 0.0135928 | 1.12E-06 |
| ebi-a-GCST90016923 | Gut microbiota abundance (class Verrucomicrobiae id.4029) || id:ebi-a-GCST90016923 | HLA DR+ Natural Killer %Natural Killer || id:ebi-a-GCST90001649 | rs4936098 | 11 | 130280667 | G | -0.0648843 | 0.0135928 | 1.12E-06 |
| ebi-a-GCST90016923 | Gut microbiota abundance (class Verrucomicrobiae id.4029) || id:ebi-a-GCST90016923 | HLA DR+ Natural Killer %CD3- lymphocyte || id:ebi-a-GCST90001650 | rs4936098 | 11 | 130280667 | G | -0.0648843 | 0.0135928 | 1.12E-06 |
[truncated: 183,665 more chars]
