# Supplementary material for: Causal Mediation Role of Immune Cells in Gut Microbiota–Pneumonia Associations: A Mendelian Randomisation Study
Source: J Cell Mol Med. 2025 Sep 11;29(17):e70839. doi: 10.1111/jcmm.70839 (PMC12425809; doi:10.1111/jcmm.70839)
Supplement: Supplementary file 10 — Table S4: Causal effects of gut microbiota on immune cells. [file JCMM-29-e70839-s011.docx]

Causal effects of gut microbiota on immune cells

| **Exposure** | **outcome** | **Methods** | **P** | **Heterogeneity and**  **pleiotropy test** | |
| --- | --- | --- | --- | --- | --- |
|  |  |  |  | **Heterogeneity Q-pvalue** | **pleiotropy test** |
| Gut microbiota abundance (genus Oscillospira id.2064) \|\| id:ebi-a-GCST90017037 | Basophil %CD33dim HLA DR- CD66b- \|\| id:ebi-a-GCST90001533 | MR Egger | 0.5643539 |  | 0.817324289 |
| Gut microbiota abundance (genus Oscillospira id.2064) \|\| id:ebi-a-GCST90017037 | Basophil %CD33dim HLA DR- CD66b- \|\| id:ebi-a-GCST90001533 | Weighted median | 0.0411022 |  | |
| Gut microbiota abundance (genus Oscillospira id.2064) \|\| id:ebi-a-GCST90017037 | Basophil %CD33dim HLA DR- CD66b- \|\| id:ebi-a-GCST90001533 | Inverse variance weighted | 0.0025323 | 0.842931802 |  |
| Gut microbiota abundance (family Verrucomicrobiaceae id.4036) \|\| id:ebi-a-GCST90016957 | T/B cell \|\| id:ebi-a-GCST90001588 | MR Egger | 0.5719946 |  | 0.557828692 |
| Gut microbiota abundance (family Verrucomicrobiaceae id.4036) \|\| id:ebi-a-GCST90016957 | T/B cell \|\| id:ebi-a-GCST90001588 | Weighted median | 0.0964343 |  | |
| Gut microbiota abundance (family Verrucomicrobiaceae id.4036) \|\| id:ebi-a-GCST90016957 | T/B cell \|\| id:ebi-a-GCST90001588 | Inverse variance weighted | 0.008273 | 0.555152127 |  |
| Gut microbiota abundance (family Verrucomicrobiaceae id.4036) \|\| id:ebi-a-GCST90016957 | CD28- CD25++ CD8+ T cell Absolute Count \|\| id:ebi-a-GCST90001678 | MR Egger | 0.5757896 |  | 0.980449901 |
| Gut microbiota abundance (family Verrucomicrobiaceae id.4036) \|\| id:ebi-a-GCST90016957 | CD28- CD25++ CD8+ T cell Absolute Count \|\| id:ebi-a-GCST90001678 | Weighted median | 0.0853015 |  | |
| Gut microbiota abundance (family Verrucomicrobiaceae id.4036) \|\| id:ebi-a-GCST90016957 | CD28- CD25++ CD8+ T cell Absolute Count \|\| id:ebi-a-GCST90001678 | Inverse variance weighted | 0.0083064 | 0.897352556 |  |
| Gut microbiota abundance (class Verrucomicrobiae id.4029) \|\| id:ebi-a-GCST90016923 | T/B cell \|\| id:ebi-a-GCST90001588 | MR Egger | 0.57765 |  | 0.557586544 |
| Gut microbiota abundance (class Verrucomicrobiae id.4029) \|\| id:ebi-a-GCST90016923 | T/B cell \|\| id:ebi-a-GCST90001588 | Weighted median | 0.1127071 |  | |
| Gut microbiota abundance (class Verrucomicrobiae id.4029) \|\| id:ebi-a-GCST90016923 | T/B cell \|\| id:ebi-a-GCST90001588 | Inverse variance weighted | 0.0083229 | 0.555134313 |  |
| Gut microbiota abundance (class Verrucomicrobiae id.4029) \|\| id:ebi-a-GCST90016923 | CD28- CD25++ CD8+ T cell Absolute Count \|\| id:ebi-a-GCST90001678 | MR Egger | 0.57765 |  | 0.978479352 |
| Gut microbiota abundance (class Verrucomicrobiae id.4029) \|\| id:ebi-a-GCST90016923 | CD28- CD25++ CD8+ T cell Absolute Count \|\| id:ebi-a-GCST90001678 | Weighted median | 0.1156391 |  | |
| Gut microbiota abundance (class Verrucomicrobiae id.4029) \|\| id:ebi-a-GCST90016923 | CD28- CD25++ CD8+ T cell Absolute Count \|\| id:ebi-a-GCST90001678 | Inverse variance weighted | 0.0083229 | 0.897073715 |  |
| Gut microbiota abundance (order Verrucomicrobiales id.4030) \|\| id:ebi-a-GCST90017108 | T/B cell \|\| id:ebi-a-GCST90001588 | MR Egger | 0.0552623 |  | 0.557586544 |
| Gut microbiota abundance (order Verrucomicrobiales id.4030) \|\| id:ebi-a-GCST90017108 | T/B cell \|\| id:ebi-a-GCST90001588 | Weighted median | 0.0140921 |  | |
| Gut microbiota abundance (order Verrucomicrobiales id.4030) \|\| id:ebi-a-GCST90017108 | T/B cell \|\| id:ebi-a-GCST90001588 | Inverse variance weighted | 0.0098959 | 0.555134313 |  |
| Gut microbiota abundance (order Verrucomicrobiales id.4030) \|\| id:ebi-a-GCST90017108 | CD28- CD25++ CD8+ T cell Absolute Count \|\| id:ebi-a-GCST90001678 | MR Egger | 0.4761123 |  | 0.978479352 |
| Gut microbiota abundance (order Verrucomicrobiales id.4030) \|\| id:ebi-a-GCST90017108 | CD28- CD25++ CD8+ T cell Absolute Count \|\| id:ebi-a-GCST90001678 | Weighted median | 0.0359396 |  | |
| Gut microbiota abundance (order Verrucomicrobiales id.4030) \|\| id:ebi-a-GCST90017108 | CD28- CD25++ CD8+ T cell Absolute Count \|\| id:ebi-a-GCST90001678 | Inverse variance weighted | 0.0121198 | 0.897073715 |  |
| Gut microbiota abundance (genus Akkermansia id.4037) \|\| id:ebi-a-GCST90016961 | T/B cell \|\| id:ebi-a-GCST90001588 | MR Egger | 0.3344589 |  | 0.558535849 |
| Gut microbiota abundance (genus Akkermansia id.4037) \|\| id:ebi-a-GCST90016961 | T/B cell \|\| id:ebi-a-GCST90001588 | Weighted median | 0.05736 |  | |
| Gut microbiota abundance (genus Akkermansia id.4037) \|\| id:ebi-a-GCST90016961 | T/B cell \|\| id:ebi-a-GCST90001588 | Inverse variance weighted | 0.0148326 | 0.555655452 |  |
| Gut microbiota abundance (genus Akkermansia id.4037) \|\| id:ebi-a-GCST90016961 | CD28- CD25++ CD8+ T cell Absolute Count \|\| id:ebi-a-GCST90001678 | MR Egger | 0.6401521 |  | 0.982642359 |
| Gut microbiota abundance (genus Akkermansia id.4037) \|\| id:ebi-a-GCST90016961 | CD28- CD25++ CD8+ T cell Absolute Count \|\| id:ebi-a-GCST90001678 | Weighted median | 0.1508071 |  | |
| Gut microbiota abundance (genus Akkermansia id.4037) \|\| id:ebi-a-GCST90016961 | CD28- CD25++ CD8+ T cell Absolute Count \|\| id:ebi-a-GCST90001678 | Inverse variance weighted | 0.0167039 | 0.89791487 |  |
| Gut microbiota abundance (family Oxalobacteraceae id.2966) \|\| id:ebi-a-GCST90016943 | CD16+ monocyte %monocyte \|\| id:ebi-a-GCST90001587 | MR Egger | 0.2926137 |  | 0.87200038 |
| Gut microbiota abundance (family Oxalobacteraceae id.2966) \|\| id:ebi-a-GCST90016943 | CD16+ monocyte %monocyte \|\| id:ebi-a-GCST90001587 | Weighted median | 0.0263982 |  | |
| Gut microbiota abundance (family Oxalobacteraceae id.2966) \|\| id:ebi-a-GCST90016943 | CD16+ monocyte %monocyte \|\| id:ebi-a-GCST90001587 | Inverse variance weighted | 0.0219099 | 0.629517829 |  |
| Gut microbiota abundance (family Oxalobacteraceae id.2966) \|\| id:ebi-a-GCST90016943 | CD25 on B cell \|\| id:ebi-a-GCST90001775 | MR Egger | 0.2931668 |  | 0.492612559 |
| Gut microbiota abundance (family Oxalobacteraceae id.2966) \|\| id:ebi-a-GCST90016943 | CD25 on B cell \|\| id:ebi-a-GCST90001775 | Weighted median | 0.0229135 |  | |
| Gut microbiota abundance (family Oxalobacteraceae id.2966) \|\| id:ebi-a-GCST90016943 | CD25 on B cell \|\| id:ebi-a-GCST90001775 | Inverse variance weighted | 0.021968 | 0.585604258 |  |
| Gut microbiota abundance (family Oxalobacteraceae id.2966) \|\| id:ebi-a-GCST90016943 | CD25 on IgD+ CD38- naive B cell \|\| id:ebi-a-GCST90001781 | MR Egger | 0.2931127 |  | 0.690268541 |
| Gut microbiota abundance (family Oxalobacteraceae id.2966) \|\| id:ebi-a-GCST90016943 | CD25 on IgD+ CD38- naive B cell \|\| id:ebi-a-GCST90001781 | Weighted median | 0.0220021 |  | |
| Gut microbiota abundance (family Oxalobacteraceae id.2966) \|\| id:ebi-a-GCST90016943 | CD25 on IgD+ CD38- naive B cell \|\| id:ebi-a-GCST90001781 | Inverse variance weighted | 0.0219701 | 0.91712152 |  |
| Gut microbiota abundance (family Oxalobacteraceae id.2966) \|\| id:ebi-a-GCST90016943 | FSC-A on HLA DR+ CD8+ T cell \|\| id:ebi-a-GCST90001978 | MR Egger | 0.2931127 |  | 0.08588983 |
| Gut microbiota abundance (family Oxalobacteraceae id.2966) \|\| id:ebi-a-GCST90016943 | FSC-A on HLA DR+ CD8+ T cell \|\| id:ebi-a-GCST90001978 | Weighted median | 0.0339915 |  | |
| Gut microbiota abundance (family Oxalobacteraceae id.2966) \|\| id:ebi-a-GCST90016943 | FSC-A on HLA DR+ CD8+ T cell \|\| id:ebi-a-GCST90001978 | Inverse variance weighted | 0.0219701 | 0.362695573 |  |
| Gut microbiota abundance (class Mollicutes id.3920) \|\| id:ebi-a-GCST90016921 | CD8 on Natural Killer T \|\| id:ebi-a-GCST90002059 | MR Egger | 0.5852591 |  | 0.250429112 |
| Gut microbiota abundance (class Mollicutes id.3920) \|\| id:ebi-a-GCST90016921 | CD8 on Natural Killer T \|\| id:ebi-a-GCST90002059 | Weighted median | 0.1018401 |  | |
| Gut microbiota abundance (class Mollicutes id.3920) \|\| id:ebi-a-GCST90016921 | CD8 on Natural Killer T \|\| id:ebi-a-GCST90002059 | Inverse variance weighted | 0.0416222 | 0.603617411 |  |
| Gut microbiota abundance (phylum Tenericutes id.3919) \|\| id:ebi-a-GCST90017117 | CD8 on Natural Killer T \|\| id:ebi-a-GCST90002059 | MR Egger | 0.5852591 |  | 0.250429112 |
| Gut microbiota abundance (phylum Tenericutes id.3919) \|\| id:ebi-a-GCST90017117 | CD8 on Natural Killer T \|\| id:ebi-a-GCST90002059 | Weighted median | 0.1023017 |  | |
| Gut microbiota abundance (phylum Tenericutes id.3919) \|\| id:ebi-a-GCST90017117 | CD8 on Natural Killer T \|\| id:ebi-a-GCST90002059 | Inverse variance weighted | 0.0416222 | 0.603617411 |  |
