# Supplementary material for: Causal Mediation Role of Immune Cells in Gut Microbiota–Pneumonia Associations: A Mendelian Randomisation Study
Source: J Cell Mol Med. 2025 Sep 11;29(17):e70839. doi: 10.1111/jcmm.70839 (PMC12425809; doi:10.1111/jcmm.70839)
Supplement: Supplementary file 11 — Table S5: Causal effects of immune cells on Pneumonia. [file JCMM-29-e70839-s003.docx]

Causal effects of immune cells on Pneumonia

| **id.exposure** | **Methods** | **OR** | **Low** | **Up** | **P** | **Heterogeneity and**  **pleiotropy test** | |
| --- | --- | --- | --- | --- | --- | --- | --- |
|  |  |  |  |  |  | **Heterogeneity Q-pvalue** | **pleiotropy test** |
| ebi-a-GCST90001458 | Inverse variance weighted | 1.049526525 | 1.011135 | 1.089376 | 0.011009 | 0.182766461 |  |
| ebi-a-GCST90001458 | MR Egger | 1.029440392 | 0.969623 | 1.092948 | 0.3669229 |  | 0.434825525 |
| ebi-a-GCST90001458 | Weighted median | 1.066617795 | 1.02337 | 1.111694 | 0.0022592 |  | |
| ebi-a-GCST90001465 | Inverse variance weighted | 1.029614466 | 1.000252 | 1.059839 | 0.0480323 | 0.000606526 |  |
| ebi-a-GCST90001465 | MR Egger | 1.027408283 | 0.989754 | 1.066495 | 0.1776718 |  | 0.853495207 |
| ebi-a-GCST90001465 | Weighted median | 1.038933711 | 1.008006 | 1.07081 | 0.0132416 |  | |
| ebi-a-GCST90001468 | Inverse variance weighted | 1.045095439 | 1.013296 | 1.077893 | 0.0051447 | 0.327972933 |  |
| ebi-a-GCST90001468 | MR Egger | 1.040462451 | 0.987546 | 1.096215 | 0.1747077 |  | 0.835936229 |
| ebi-a-GCST90001468 | Weighted median | 1.051231369 | 1.014983 | 1.088774 | 0.0052598 |  | |
| ebi-a-GCST90001469 | Inverse variance weighted | 1.026835653 | 1.006527 | 1.047554 | 0.0093673 | 0.346146383 |  |
| ebi-a-GCST90001469 | MR Egger | 1.011669657 | 0.983696 | 1.040439 | 0.4331708 |  | 0.178650212 |
| ebi-a-GCST90001469 | Weighted median | 1.01617303 | 0.987807 | 1.045354 | 0.2667072 |  | |
| ebi-a-GCST90001472 | Inverse variance weighted | 1.049867317 | 1.018629 | 1.082064 | 0.0015905 | 0.03142338 |  |
| ebi-a-GCST90001472 | MR Egger | 1.049617998 | 1.003706 | 1.09763 | 0.0573621 |  | 0.60478066 |
| ebi-a-GCST90001472 | Weighted median | 1.050137629 | 1.019153 | 1.082064 | 0.0013667 |  | |
| ebi-a-GCST90001473 | Inverse variance weighted | 1.047999972 | 1.016812 | 1.080144 | 0.0023528 | 0.024925995 |  |
| ebi-a-GCST90001473 | MR Egger | 1.051362917 | 1.007176 | 1.097488 | 0.0430476 |  | 0.497130097 |
| ebi-a-GCST90001473 | Weighted median | 1.050137572 | 1.019675 | 1.08151 | 0.0011249 |  | |
| ebi-a-GCST90001475 | Inverse variance weighted | 1.04019244 | 1.011305 | 1.069905 | 0.0061007 | 0.891267582 |  |
| ebi-a-GCST90001475 | MR Egger | 1.031858889 | 0.98843 | 1.077196 | 0.1866294 |  | 0.639110715 |
| ebi-a-GCST90001475 | Weighted median | 1.032493881 | 0.99191 | 1.074738 | 0.1180566 |  | |
| ebi-a-GCST90001477 | Inverse variance weighted | 1.028572508 | 1.005659 | 1.052008 | 0.0142475 | 0.628458001 |  |
| ebi-a-GCST90001477 | MR Egger | 1.008621744 | 0.977586 | 1.040643 | 0.5987812 |  | 0.098116827 |
| ebi-a-GCST90001477 | Weighted median | 1.015848868 | 0.982737 | 1.050076 | 0.3523477 |  | |
| ebi-a-GCST90001533 | Inverse variance weighted | 1.031080636 | 1.005578 | 1.05723 | 0.0166068 | 0.750484799 |  |
| ebi-a-GCST90001533 | MR Egger | 1.048191327 | 0.996639 | 1.10241 | 0.0973101 |  | 0.478094965 |
| ebi-a-GCST90001533 | Weighted median | 1.044597222 | 1.008337 | 1.082161 | 0.0154933 |  | |
| ebi-a-GCST90001587 | Inverse variance weighted | 0.936288253 | 0.885913 | 0.989528 | 0.0196432 | 0.024160901 |  |
| ebi-a-GCST90001587 | MR Egger | 0.938119358 | 0.837111 | 1.051316 | 0.2952325 |  | 0.96958979 |
| ebi-a-GCST90001587 | Weighted median | 0.920735357 | 0.872863 | 0.971233 | 0.0024338 |  | |
| ebi-a-GCST90001588 | Inverse variance weighted | 1.037554067 | 1.003021 | 1.073276 | 0.0327864 | 0.935804793 |  |
| ebi-a-GCST90001588 | MR Egger | 1.052675264 | 0.992998 | 1.115939 | 0.1083871 |  | 0.561093035 |
| ebi-a-GCST90001588 | Weighted median | 1.046809445 | 0.997377 | 1.098692 | 0.063798 |  | |
| ebi-a-GCST90001595 | Inverse variance weighted | 1.084173153 | 1.004067 | 1.170671 | 0.0390528 | 0.452134209 |  |
| ebi-a-GCST90001595 | MR Egger | 1.100407412 | 0.88947 | 1.361369 | 0.4711487 |  | 0.894398021 |
| ebi-a-GCST90001595 | Weighted median | 1.103456868 | 1.002905 | 1.21409 | 0.0434344 |  | |
| ebi-a-GCST90001631 | Inverse variance weighted | 1.040717489 | 1.000117 | 1.082966 | 0.0493235 | 0.142776336 |  |
| ebi-a-GCST90001631 | MR Egger | 1.027917975 | 0.965743 | 1.094096 | 0.4006667 |  | 0.615229929 |
| ebi-a-GCST90001631 | Weighted median | 1.031507763 | 0.97923 | 1.086577 | 0.2423893 |  | |
| ebi-a-GCST90001638 | Inverse variance weighted | 0.968983426 | 0.939489 | 0.999404 | 0.0457368 | 0.623539197 |  |
| ebi-a-GCST90001638 | MR Egger | 0.90675462 | 0.850119 | 0.967163 | 0.007786 |  | 0.03306554 |
| ebi-a-GCST90001638 | Weighted median | 0.968506106 | 0.924931 | 1.014135 | 0.1730624 |  | |
| ebi-a-GCST90001648 | Inverse variance weighted | 0.953707164 | 0.921678 | 0.986849 | 0.0065366 | 0.371964208 |  |
| ebi-a-GCST90001648 | MR Egger | 0.93935534 | 0.885385 | 0.996616 | 0.0769569 |  | 0.54987413 |
| ebi-a-GCST90001648 | Weighted median | 0.949238251 | 0.912494 | 0.987462 | 0.0096973 |  | |
| ebi-a-GCST90001649 | Inverse variance weighted | 0.955757627 | 0.924676 | 0.987884 | 0.0073035 | 0.048664009 |  |
| ebi-a-GCST90001649 | MR Egger | 0.970452491 | 0.91588 | 1.028277 | 0.3270059 |  | 0.535083729 |
| ebi-a-GCST90001649 | Weighted median | 0.943792098 | 0.908307 | 0.980663 | 0.0030901 |  | |
| ebi-a-GCST90001650 | Inverse variance weighted | 0.958256357 | 0.924001 | 0.993782 | 0.0216847 | 0.047181984 |  |
| ebi-a-GCST90001650 | MR Egger | 0.964925512 | 0.90421 | 1.029718 | 0.3011474 |  | 0.801409363 |
| ebi-a-GCST90001650 | Weighted median | 0.943402719 | 0.907839 | 0.980359 | 0.0029606 |  | |
| ebi-a-GCST90001678 | Inverse variance weighted | 0.955243719 | 0.926324 | 0.985067 | 0.0035088 | 0.674611092 |  |
| ebi-a-GCST90001678 | MR Egger | 0.969197063 | 0.925782 | 1.014648 | 0.2056714 |  | 0.41934478 |
| ebi-a-GCST90001678 | Weighted median | 0.988449202 | 0.943379 | 1.035672 | 0.6255966 |  | |
| ebi-a-GCST90001679 | Inverse variance weighted | 0.956061962 | 0.924685 | 0.988503 | 0.0083107 | 0.690803683 |  |
| ebi-a-GCST90001679 | MR Egger | 0.970229645 | 0.918535 | 1.024834 | 0.307443 |  | 0.523226735 |
| ebi-a-GCST90001679 | Weighted median | 0.961058633 | 0.916808 | 1.007445 | 0.0986184 |  | |
| ebi-a-GCST90001722 | Inverse variance weighted | 1.036013142 | 1.001278 | 1.071953 | 0.0420123 | 0.941904771 |  |
| ebi-a-GCST90001722 | MR Egger | 1.056707652 | 1.000833 | 1.115702 | 0.0720236 |  | 0.37896289 |
| ebi-a-GCST90001722 | Weighted median | 1.031901461 | 0.984718 | 1.081346 | 0.1884785 |  | |
| ebi-a-GCST90001775 | Inverse variance weighted | 1.020641143 | 1.00047 | 1.041219 | 0.0448451 | 0.439405485 |  |
| ebi-a-GCST90001775 | MR Egger | 1.029226357 | 1.005449 | 1.053566 | 0.0325703 |  | 0.206528543 |
| ebi-a-GCST90001775 | Weighted median | 1.031210627 | 1.000441 | 1.062927 | 0.0467555 |  | |
| ebi-a-GCST90001779 | Inverse variance weighted | 1.017049878 | 1.002631 | 1.031676 | 0.0203039 | 0.323580592 |  |
| ebi-a-GCST90001779 | MR Egger | 1.008960539 | 0.993444 | 1.02472 | 0.2769882 |  | 0.059696242 |
| ebi-a-GCST90001779 | Weighted median | 1.000949622 | 0.981335 | 1.020956 | 0.9251067 |  | |
| ebi-a-GCST90001781 | Inverse variance weighted | 1.031412367 | 1.007474 | 1.055919 | 0.0098365 | 0.804562055 |  |
| ebi-a-GCST90001781 | MR Egger | 1.022438213 | 0.986465 | 1.059723 | 0.2500649 |  | 0.539475671 |
| ebi-a-GCST90001781 | Weighted median | 1.032256417 | 0.998102 | 1.067579 | 0.0644076 |  | |
| ebi-a-GCST90001783 | Inverse variance weighted | 1.055441812 | 1.003962 | 1.109562 | 0.0344327 | 0.272949118 |  |
| ebi-a-GCST90001783 | MR Egger | 1.072866445 | 0.896643 | 1.283724 | 0.4643892 |  | 0.856169268 |
| ebi-a-GCST90001783 | Weighted median | 1.062434704 | 0.999908 | 1.128871 | 0.0503447 |  | |
| ebi-a-GCST90001903 | Inverse variance weighted | 1.044218261 | 1.007445 | 1.082333 | 0.0180035 | 0.328198561 |  |
| ebi-a-GCST90001903 | MR Egger | 1.043473991 | 0.977184 | 1.114261 | 0.2325715 |  | 0.979787432 |
| ebi-a-GCST90001903 | Weighted median | 1.063853359 | 1.012935 | 1.117331 | 0.0133756 |  | |
| ebi-a-GCST90001946 | Inverse variance weighted | 1.01620518 | 1.000752 | 1.031897 | 0.0397732 | 0.42956469 |  |
| ebi-a-GCST90001946 | MR Egger | 1.024781233 | 0.999703 | 1.050489 | 0.0789111 |  | 0.412259576 |
| ebi-a-GCST90001946 | Weighted median | 1.019117982 | 1.001935 | 1.036596 | 0.0290506 |  | |
| ebi-a-GCST90001947 | Inverse variance weighted | 1.014233036 | 1.000361 | 1.028297 | 0.0442811 | 0.941650282 |  |
| ebi-a-GCST90001947 | MR Egger | 1.014697217 | 0.993185 | 1.036675 | 0.2049296 |  | 0.957265566 |
| ebi-a-GCST90001947 | Weighted median | 1.017866177 | 1.001006 | 1.03501 | 0.0377108 |  | |
| ebi-a-GCST90001948 | Inverse variance weighted | 1.017454351 | 1.002799 | 1.032324 | 0.0194071 | 0.697848922 |  |
| ebi-a-GCST90001948 | MR Egger | 1.023798755 | 0.999468 | 1.048722 | 0.0733274 |  | 0.534342735 |
| ebi-a-GCST90001948 | Weighted median | 1.020402876 | 1.003046 | 1.038061 | 0.0210322 |  | |
| ebi-a-GCST90001950 | Inverse variance weighted | 1.021806773 | 1.001363 | 1.042668 | 0.0364307 | 0.389512818 |  |
| ebi-a-GCST90001950 | MR Egger | 1.040105947 | 1.003646 | 1.07789 | 0.0676155 |  | 0.279968654 |
| ebi-a-GCST90001950 | Weighted median | 1.030497965 | 1.007332 | 1.054197 | 0.0096057 |  | |
| ebi-a-GCST90001951 | Inverse variance weighted | 1.017636379 | 1.000624 | 1.034938 | 0.0421045 | 0.71540187 |  |
| ebi-a-GCST90001951 | MR Egger | 1.042148622 | 1.01078 | 1.074491 | 0.0201024 |  | 0.090255636 |
| ebi-a-GCST90001951 | Weighted median | 1.026467882 | 1.005276 | 1.048106 | 0.0141101 |  | |
| ebi-a-GCST90001953 | Inverse variance weighted | 1.016524967 | 1.001329 | 1.031952 | 0.0329428 | 0.549361677 |  |
| ebi-a-GCST90001953 | MR Egger | 1.024614497 | 0.998679 | 1.051223 | 0.0926734 |  | 0.471209749 |
| ebi-a-GCST90001953 | Weighted median | 1.018194902 | 1.000957 | 1.03573 | 0.0384734 |  | |
| ebi-a-GCST90001954 | Inverse variance weighted | 1.023287727 | 1.008189 | 1.038613 | 0.0024034 | 0.519939533 |  |
| ebi-a-GCST90001954 | MR Egger | 1.02817894 | 1.003813 | 1.053136 | 0.046491 |  | 0.630212948 |
| ebi-a-GCST90001954 | Weighted median | 1.026639872 | 1.008803 | 1.044793 | 0.0032816 |  | |
| ebi-a-GCST90001955 | Inverse variance weighted | 1.026624683 | 1.007206 | 1.046418 | 0.0069969 | 0.078263688 |  |
| ebi-a-GCST90001955 | MR Egger | 1.027315693 | 0.994897 | 1.06079 | 0.1305169 |  | 0.959038463 |
| ebi-a-GCST90001955 | Weighted median | 1.027049262 | 1.009414 | 1.044993 | 0.0025255 |  | |
| ebi-a-GCST90001978 | Inverse variance weighted | 1.027426243 | 1.003195 | 1.052243 | 0.0262871 | 0.494973139 |  |
| ebi-a-GCST90001978 | MR Egger | 1.045638529 | 1.015508 | 1.076663 | 0.0242712 |  | 0.087567391 |
| ebi-a-GCST90001978 | Weighted median | 1.032113203 | 1.000152 | 1.065096 | 0.0488982 |  | |
| ebi-a-GCST90001988 | Inverse variance weighted | 1.028212352 | 1.003531 | 1.053501 | 0.0248117 | 0.510045875 |  |
| ebi-a-GCST90001988 | MR Egger | 1.060142787 | 1.012607 | 1.109911 | 0.0372172 |  | 0.162038243 |
| ebi-a-GCST90001988 | Weighted median | 1.034635116 | 1.00515 | 1.064985 | 0.020986 |  | |
| ebi-a-GCST90001991 | Inverse variance weighted | 1.031150759 | 1.004038 | 1.058996 | 0.0240449 | 0.342096177 |  |
| ebi-a-GCST90001991 | MR Egger | 1.062439509 | 1.013165 | 1.11411 | 0.0369514 |  | 0.183910112 |
| ebi-a-GCST90001991 | Weighted median | 1.028931718 | 0.996634 | 1.062276 | 0.0796357 |  | |
| ebi-a-GCST90002005 | Inverse variance weighted | 0.96948756 | 0.94045 | 0.999421 | 0.0457918 | 0.075564896 |  |
| ebi-a-GCST90002005 | MR Egger | 0.942431523 | 0.897537 | 0.989572 | 0.0332475 |  | 0.243710077 |
| ebi-a-GCST90002005 | Weighted median | 0.953529848 | 0.923716 | 0.984306 | 0.0033247 |  | |
| ebi-a-GCST90002042 | Inverse variance weighted | 1.040511098 | 1.004541 | 1.077769 | 0.026938 | 0.608431 |  |
| ebi-a-GCST90002042 | MR Egger | 1.05847498 | 0.963409 | 1.162922 | 0.2705424 |  | 0.710713803 |
| ebi-a-GCST90002042 | Weighted median | 1.037747687 | 0.990675 | 1.087057 | 0.117716 |  | |
| ebi-a-GCST90002059 | Inverse variance weighted | 0.956055406 | 0.918748 | 0.994878 | 0.0269082 | 0.425342752 |  |
| ebi-a-GCST90002059 | MR Egger | 0.993876612 | 0.873188 | 1.131246 | 0.9285165 |  | 0.55490903 |
| ebi-a-GCST90002059 | Weighted median | 0.949493948 | 0.901452 | 1.000096 | 0.0504229 |  | |
