# Supplementary material for: Causal Mediation Role of Immune Cells in Gut Microbiota–Pneumonia Associations: A Mendelian Randomisation Study
Source: J Cell Mol Med. 2025 Sep 11;29(17):e70839. doi: 10.1111/jcmm.70839 (PMC12425809; doi:10.1111/jcmm.70839)
Supplement: Supplementary file 12 — Table S6: Causal effects of gut microbiota on Pneumonia. [file JCMM-29-e70839-s008.docx]

Causal effects of gut microbiota on Pneumonia

| Traits | Methods | OR | Low | Up | P | Heterogeneity and  pleiotropy test | |
| --- | --- | --- | --- | --- | --- | --- | --- |
|  |  |  |  |  |  | Heterogeneity Q-pvalue | pleiotropy test |
| Gut microbiota abundance (class Mollicutes id.3920) | Inverse variance weighted | 1.122956525 | 1.006245728 | 1.253204184 | 0.0383396 | 0.472489301 |  |
| Gut microbiota abundance (class Mollicutes id.3920) | MR Egger | 1.019580462 | 0.743395761 | 1.398372673 | 0.9089256 |  | 0.550174241 |
| Gut microbiota abundance (class Mollicutes id.3920) | Weighted median | 1.057249097 | 0.916807603 | 1.21920417 | 0.4439375 |  | |
| Gut microbiota abundance (class Verrucomicrobiae id.4029) | Inverse variance weighted | 0.858463466 | 0.773763428 | 0.952435195 | 0.0039829 | 0.352561222 |  |
| Gut microbiota abundance (class Verrucomicrobiae id.4029) | MR Egger | 0.902246784 | 0.627101564 | 1.298113904 | 0.5966707 |  | 0.786623211 |
| Gut microbiota abundance (class Verrucomicrobiae id.4029) | Weighted median | 0.867460827 | 0.757462532 | 0.993433014 | 0.0398561 |  | |
| Gut microbiota abundance (family Oxalobacteraceae id.2966) | Inverse variance weighted | 1.089522898 | 1.010097686 | 1.175193412 | 0.0264079 | 0.41196978 |  |
| Gut microbiota abundance (family Oxalobacteraceae id.2966) | MR Egger | 1.174835007 | 0.742502815 | 1.8588984 | 0.5219151 |  | 0.756705303 |
| Gut microbiota abundance (family Oxalobacteraceae id.2966) | Weighted median | 1.095846646 | 0.990142884 | 1.212834926 | 0.0769631 |  | |
| Gut microbiota abundance (family Verrucomicrobiaceae id.4036) | Inverse variance weighted | 0.85841743 | 0.773696845 | 0.952415003 | 0.0039815 | 0.352591729 |  |
| Gut microbiota abundance (family Verrucomicrobiaceae id.4036) | MR Egger | 0.902205478 | 0.627098636 | 1.298001109 | 0.5964701 |  | 0.786558279 |
| Gut microbiota abundance (family Verrucomicrobiaceae id.4036) | Weighted median | 0.86749714 | 0.755975389 | 0.995470619 | 0.0429021 |  | |
| Gut microbiota abundance (genus Akkermansia id.4037) | Inverse variance weighted | 0.858463421 | 0.773755354 | 0.952445036 | 0.0039865 | 0.352483751 |  |
| Gut microbiota abundance (genus Akkermansia id.4037) | MR Egger | 0.902409816 | 0.627707114 | 1.297330327 | 0.5965266 |  | 0.785375674 |
| Gut microbiota abundance (genus Akkermansia id.4037) | Weighted median | 0.86729304 | 0.754755896 | 0.996609926 | 0.044655 |  | |
| Gut microbiota abundance (genus Oscillospira id.2064) | Inverse variance weighted | 1.320753451 | 1.112942514 | 1.567367277 | 0.0014469 | 0.622898631 |  |
| Gut microbiota abundance (genus Oscillospira id.2064) | MR Egger | 1.027746441 | 0.448056505 | 2.357432008 | 0.958923 |  | 0.653533684 |
| Gut microbiota abundance (genus Oscillospira id.2064) | Weighted median | 1.325188037 | 1.067399457 | 1.645235363 | 0.0107427 |  | |
| Gut microbiota abundance (order Verrucomicrobiales id.4030) | Inverse variance weighted | 0.858463466 | 0.773763428 | 0.952435195 | 0.0039829 | 0.352561222 |  |
| Gut microbiota abundance (order Verrucomicrobiales id.4030) | MR Egger | 0.902246784 | 0.627101564 | 1.298113904 | 0.5966707 |  | 0.786623211 |
| Gut microbiota abundance (order Verrucomicrobiales id.4030) | Weighted median | 0.867460827 | 0.753242211 | 0.998999094 | 0.0483929 |  | |
| Gut microbiota abundance (phylum Tenericutes id.3919) | Inverse variance weighted | 1.122956525 | 1.006245728 | 1.253204184 | 0.0383396 | 0.472489301 |  |
| Gut microbiota abundance (phylum Tenericutes id.3919) | MR Egger | 1.019580462 | 0.743395761 | 1.398372673 | 0.9089256 |  | 0.550174241 |
| Gut microbiota abundance (phylum Tenericutes id.3919) | Weighted median | 1.057249097 | 0.910028652 | 1.22828622 | 0.4668152 |  | |
